# *Lactiplantibacillus plantarum* 299v supplementation modulates β-cell ER stress and antioxidative defense pathways and prevents type 1 diabetes in gluten-free BioBreeding rats

**DOI:** 10.1080/19490976.2022.2136467

**Published:** 2022-10-19

**Authors:** Pinar Sargin, Mark F. Roethle, Shuang Jia, Tarun Pant, Ashley E. Ciecko, Samantha N. Atkinson, Nita H. Salzman, Ru-Jeng Teng, Yi-Guang Chen, Susanne M. Cabrera, Martin J. Hessner

**Affiliations:** a The Max McGee Research Center for Juvenile Diabetes, Children’s Research Institute of Children’s Hospital of Wisconsin, Milwaukee, WI, USA; b Department of Pediatrics, Division of Endocrinology, the Medical College of Wisconsin, Milwaukee, WI, USA; c Center for Microbiome Research, Medical College of Wisconsin, Milwaukee, WI, USA; d Department of Microbiology & Immunology, Medical College of Wisconsin, Milwaukee, WI, USA; e Department of Pediatrics, Division of Gastroenterology, the Medical College of Wisconsin, Milwaukee, WI, USA; f Department of Pediatrics, Division of Neonatology, the Medical College of Wisconsin, Milwaukee, WI, USA

**Keywords:** Type 1 diabetes, probiotic supplement, endoplasmic reticulum stress, *Lactiplantibacillus plantarum*, beta cell, Nrf2, antioxidative defense, unfolded protein response

## Abstract

The increasing incidence of Type 1 diabetes has coincided with the emergence of the low-fiber, high-gluten Western diet and other environmental factors linked to dysbiosis. Since *Lactiplantibacillus plantarum* 299 v (Lp299v) supplementation improves gut barrier function and reduces systemic inflammation, we studied its effects in spontaneously diabetic DR*lyp/lyp* rats provided a normal cereal diet (ND) or a gluten-free hydrolyzed casein diet (HCD). All rats provided ND developed diabetes (62.5±7.7 days); combining ND with Lp299v did not improve survival. Diabetes was delayed by HCD (72.2±9.4 days, p = .01) and further delayed by HCD+Lp299v (84.9±14.3 days, p < .001). HCD+Lp299v pups exhibited increased plasma propionate and butyrate levels, which correlated with enriched fecal *Bifidobacteriaceae* and *Clostridiales* taxa. Islet transcriptomic and histologic analyses at 40-days of age revealed that rats fed HCD expressed an autophagy profile, while those provided HCD+Lp299v expressed ER-associated protein degradation (ERAD) and antioxidative defense pathways, including Nrf2. Exposing insulinoma cells to propionate and butyrate promoted the antioxidative defense response but did not recapitulate the HCD+Lp299v islet ERAD transcriptomic profile. Here, both diet and microbiota influenced diabetes susceptibility. Moreover, Lp299v supplement modulated antioxidative defense and ER stress responses in β-cells, potentially offering a new therapeutic direction to thwart diabetes progression and preserve insulin secretion.

## Graphical Abstract

**Figure:**
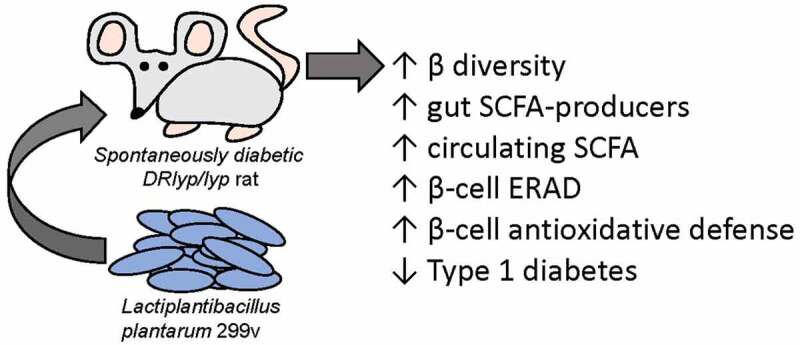

## Introduction

Type 1 diabetes (T1D) arises through autoimmunity toward pancreatic β-cells. In recent decades T1D incidence has increased, while the age of onset and prevalence of high-risk HLA haplotypes among newly diagnosed patients has declined.^1^ These shifts are consistent with new environmental pressures that promote β-cell autoimmunity^2^ and coincide with the increasing use of antibiotics and popularity of the low-fiber, high-gluten Western diet.^2–4^ These altered environmental factors likely underlie the distinct gut microbiota of modern humans, which possess reduced abundances of fiber-fermenting, short-chain fatty acid (SCFA)-producing taxa.^5^ Together these changes are thought to foster dysbiosis and gut hyperpermeability, thereby increasing microbial antigen exposure and systemic inflammation.^2,3^ An inflammatory state consistent with microbial antigen exposure exists in T1D families^6^ and the BioBreeding (BB) rat diabetes model.^7,8^ In BB rats, this inflammatory state extends to the pancreatic islets, which express a transcriptome consistent with microbial antigen exposure, and where β-cells begin expressing immunocyte-recruiting chemokines by 40-days of age, prior to insulitis.^8–10^

Exposure of β-cells to cytokines and chronic inflammation disrupts endoplasmic reticulum (ER) homeostasis, promotes the accumulation of unfolded/misfolded protein, and triggers the unfolded protein response (UPR).^11^ The UPR is activated through three sensor proteins: protein kinase RNA-like ER kinase (Perk), activating transcription factor 6 (Atf6), and inositol requiring kinase-1α (Ire1α). The UPR promotes recovery and survival by arresting translation to reduce ER input, increasing chaperone expression to enhance protein folding capacity, enhancing antioxidative stress responses, and upregulating transcription of the ER-associated protein degradation (ERAD) and autophagy pathways to respectively foster removal of misfolded proteins and protein aggregates from the ER. Chronic hyperactivation of the UPR induces apoptosis.^11^

ER homeostasis is closely linked to the ER redox state, as disulfide bond formation involves the transfer of electrons from protein disulfide isomerase to endoplasmic reticulum oxidase 1 to molecular oxygen, thereby generating hydrogen peroxide. While normal cellular functions, including mitochondrial respiration, produce reactive oxygen species (ROS), inflammation and ER stress can promote ROS production that outpace antioxidative defense mechanisms. In many model systems, ER stress and oxidative stress promote one another, ultimately impairing cell function and activating pro-apoptotic signaling.^12^

In non-obese diabetic (NOD) mice, diabetes onset is preceded by elevated ER-stress markers within the islet,^13^ and chemical chaperone treatment mitigates diabetes by attenuating ER stress.^14^ ER stress in β-cells precedes virally induced diabetes in BB DR+/+ rats,^15^ and UPR activation has been described in β-cells of T1D patients.^16^ Importantly, ER stress in β-cells may foster post-translational protein modifications that generate neoantigens recognized by diabetogenic T-cells.^17,18^ In this way, environmental changes that foster dysbiosis, systemic inflammation, and islet stress may promote breaks in tolerance and T1D progression.

Gluten intolerance is associated with T1D. In rodent models, gluten-free diets favorably alter the gut microbiome, lower systemic inflammation, and prevent diabetes,^8,19^ whereas high gluten consumption early in life has been associated with T1D development in children.^20^ Probiotic and dietary supplements that increase SCFA levels prevent diabetes in rodent models and favorably alter immune profiles in T1D family members and patients.^21,22^ How such supplements impact pancreatic islets *in vivo* remains incomplete. Therefore, we studied *Lactiplantibacillus plantarum* 299 v(Lp299v) supplementation in spontaneously diabetic BB DR*lyp/lyp* rats provided gluten-containing and gluten-free diets. In the absence of gluten, Lp299v favorably alters the gut microbiota, increases circulating SCFA, modulates β-cell UPR and antioxidative defenses, and prevents diabetes.

## Methods

### Animals

This study utilized DR*lyp/lyp* rats. DR*lyp/lyp* rats possess the RT1^u/u^ class II MHC (*Iddm1*) and are lymphopenic due to *Gimap5* deficiency (*Iddm2*).^23^ When provided a normal cereal diet (ND), 100% of DR*lyp/lyp* rats develop diabetes independent of gender.^8,9^ Nondiabetic F*lyp/lyp* rats were generated through introgression of *Iddm1* and *Iddm2* from BB rats onto the Fischer (F344) background.^24^ Rats, sourced from colonies maintained at the Medical College of Wisconsin, were provided chow and water *ad libitum*, and housed at 20.9°C with 12-hour light/dark cycles. All protocols followed The National Institutes of Health Guide for the Care and Use of Laboratory and were approved by the Medical College of Wisconsin Institutional Animal Care and Use Committee (Assurance Number A3102-01).

At 21-days of age, DR*lyp/lyp* littermates were randomly weaned onto ND, containing both plant and animal protein sources (LabDiet 5L0D, Purina, St. Louis, MO, USA), or a gluten-free hydrolyzed casein diet (HCD, Modified AIN-93 G diet, Dyets Inc., Bethlehem, PA, USA). These compositionally similar diets differ in protein source and gluten content.^8^ Subgroups of DR*lyp/lyp* rats receiving these diets were supplemented with 50 × 10^6^ CFU/gram body weight/day *Lactiplantibacillus plantarum* 299v (Lp299v, NextFoods, Boulder, CO, USA) by oral gavage from weaning. The composition of the probiotic was confirmed to be >99.999% Lp299v through metagenomic sequencing using MetaPhlAn v.3.0 (20x10^6^ reads per sample; Diversigen, Waco, TX, USA). T1D onset was defined as the first of two consecutive days with fasting blood glucose ≥ 250 mg/dl. F*lyp/lyp* rats were weaned at 21 days onto ND without Lp299v. Blood was drawn by cardiac puncture into EDTA vacutainer tubes while rats were under isoflurane anesthesia. Plasma was separated by centrifugation and stored at −80°C.

### Analysis of fecal microbiota

Stool, collected from 40-day old pups, was immediately homogenized in phosphate buffered saline (PBS). DNA was extracted using the DNeasy PowerLyzer PowerSoil Kit (QIAGEN, Germantown, MD, USA). The V4 region of the 16S rDNA gene was amplified by polymerase chain reaction (PCR) and sequenced (Diversigen) on the MiSeq platform (Illumina, San Diego, CA, USA) using the 2x250-bp protocol.^25^ Paired-end 16S rRNA gene sequencing reads were analyzed with QIIME2 (v.2020.8).^26^ Representative sequences were selected, and chimeric sequences were removed using DADA2.^27^ The representative sequences were aligned,^28^ masked for hypervariable regions, and phylogenetic trees were produced.^29^ A classifier was generated to assign taxonomy to the reads using the 99% similarity files of the SILVA 132 release and the 515–806 region (V4) of the 16S gene.^30^ Taxonomy was assigned to the feature table to generate relative abundance tables. Alpha and beta diversity were analyzed using QIIME2. Principal Coordinate Analysis plots were examined using Emperor.^31^ LEfSe, Linear discriminant analysis (LDA) effect size was used to identify enriched taxa in each treatment group.^32^ Sequencing data has been deposited at The National Center for Biotechnology Information Sequence Read Archive (Accession Number: PRJNA854152).

### Measurement of plasma analytes

Plasma cytokine/chemokine levels (eotaxin, CCL5, CXCL10, G-CSF, GM-CSF, Fractalkine, IL-1a, IL-1b, IL-2, IL-4, IL-5, IL-6, IL-10, IL-12(p70), IL-13, IL-17A, IL-18, MCP-1, MIP-1a, RANTES, TGFB1, TGFB2, TGFB3, TNFa, VEGF) were measured by 30-plex ELISA (Eve Technologies, Calgary, AB. Canada). Circulating SCFA levels (acetate, propionate, isobutyrate, butyrate, isovalerate, valerate, isocaproate, hexanoate) were measured by mass spectrometry at the Mayo Clinic Metabolomics Research Core as described.^33^ Commercial ELISAs were used to quantify non-fasting proinsulin (Mercodia, Uppsala, Sweden) and C-peptide (Crystal Chem, Elk Grove, IL, USA) in plasma.

### Islet transcriptomic analyses

Pancreatic islets were isolated from 40-day-old rats and transcriptomic analysis utilized Affymetrix RG230 2.0 arrays (Affymetrix, Santa Clara, CA, USA) as described.^8^ Array images were quantified with Affymetrix Expression Console Software, then normalized and analyzed with Partek Genomic Suite (Partek Inc, St. Louis, MO, USA). Data files have been deposited at The National Center for Biotechnology Information Gene Expression Omnibus (accession number: GSE198617). Expression differences were assessed by non-parametric rank product tests and false discovery rates (FDR) to investigate the rate of type I errors in multiple testing.^34^ Ontological analyses were conducted with the Database for Annotation, Visualization, and Integrated Discovery version 6.7.^35^

Spliced *Xbp1* (*sXbp1*) transcript was measured by quantitative real-time PCR using *sXbp1*-specific primers,^36^ QuantumRNA 18S internal standards kit (ThermoFisher, Waltham, MA, USA), and QuantiTect SYBR Green PCR Master Mix (Qiagen, Hilden, Germany) per the manufacturer’s instructions.

### Immunofluorescent staining

Immunofluorescent staining of pancreata was conducted as described.^9^ Primary staining utilized the following antibodies: Eif4g (Cell Signaling Technology, Beverly, MA, USA; #8701), Os9 (Abcam, Cambridge, UK; ab109510), Tollip (Proteintech, Chicago, IL, USA; 11315-1-AP), Gsta (Thermofisher Scientific; PA5-79335), Nrf2 (Proteintech; 16396-1-AP), and Keap1 (Proteintech; 10503-2-AP), in combination with mouse monoclonal anti-insulin Abs (Sigma-Aldrich, St. Louis, MO, USA, I2018). Secondary staining utilized Alexa594-conjugated donkey anti-goat IgG, or Alexa594-conjugated donkey anti-rabbit IgG, and FITC-conjugated donkey anti-mouse IgG (all from Jackson ImmunoResearch Laboratories, West Grove PA, USA). Nuclear counterstaining utilized DAPI (Invitrogen, Waltham, MA). Images were collected using a fluorescence microscope (Keyence, Itasca, IL, USA) and analyzed with the Fiji software package for ImageJ (https://imagej.nih.gov.ij/).

### Rat insulinoma cell culture

RINm5f cells (ATCC, Rockville, MD, USA) were propagated as described^37^ and subcultured for 24 hours in medium possessing propionate and butyrate at each of the following concentrations: 0 µM/0 µM, 30 µM/6 µM, 60 µM/12 µM, 120 µM/24 µM, 240 µM/48 µM, 1500 µM/300 µM, and 500 µM/500 µM. For each experimental condition, RNA was prepared from 3 replicate pools; each pool was prepared from 3 individual cultures. Transcriptomic analysis was conducted as described above.

## Results

### Lp299v supplement delays T1D onset

**Figure 1.**
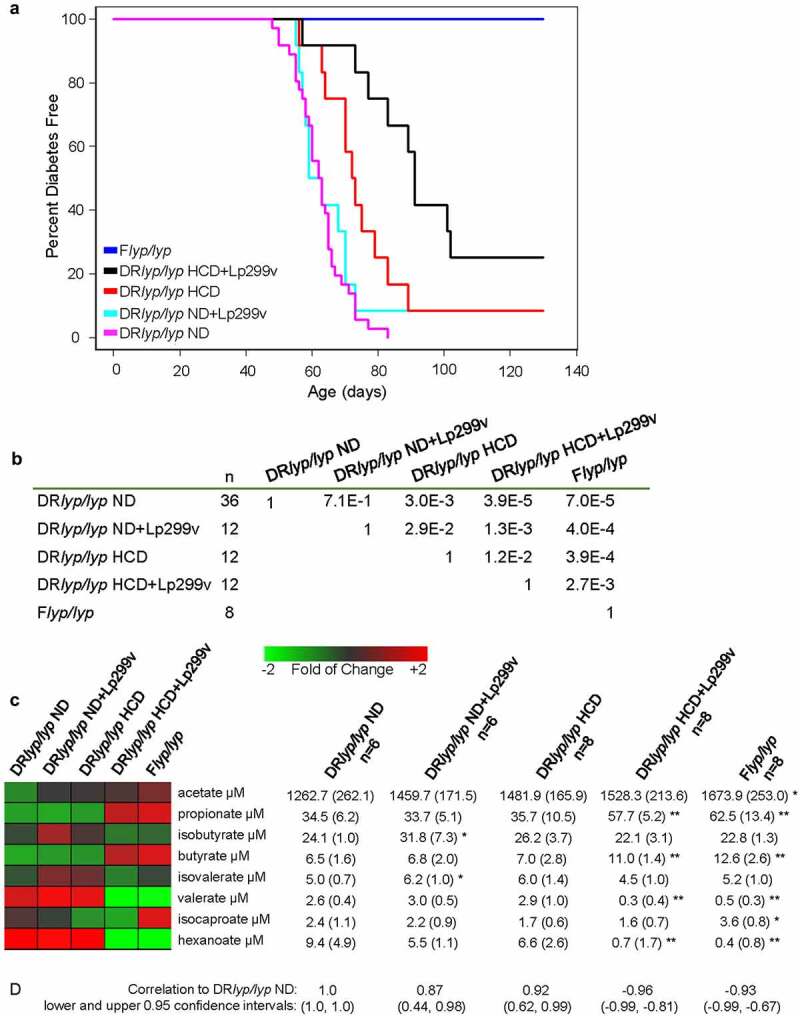
Lp299v supplement is associated with increased survival and elevated plasma propionate and butyrate levels. The probiotic was suspended in PBS and administered by oral gavage. Blood glucose was monitored from 40-days of age. T1D onset was defined as the first of two consecutive days with fasting blood glucose ≥ 250 mg/dl. A. Progressive improvements in diabetes-free survival were observed when providing DR*lyp/lyp* rats ND, ND+Lp299v, HCD, and HCD+Lp299v from weaning. The median age of survival of the ND group was 70 days (range 48–83 days), for the ND+Lp299v group it was 61 days (range 55–130 days), for the HCD group it was 72.5 days (range 56–130 days), and for the HCD+Lp299v group it was 91 days (range 57–130 days). As expected, F*lyp/lyp* control rats did not develop diabetes. B. Statistical significance of survival differences (Gehan-Wilcoxon test) are tabulated for each pairwise comparison. C. Plasma samples of 40-day-old rats were assayed in duplicate for circulating SCFA. Levels are shown as a heatmap with tabulated means and standard deviations for each study group. All groups were compared to DR*lyp/lyp* ND: * indicates p < .05 and ** represents p < .001 (t-test, two-tailed). D. Pearsons Correlation Coefficients between the SCFA profile of the DR*lyp/lyp* ND group versus the other groups are shown. Indicated in parentheses are, respectively, the lower and upper 0.95 confidence intervals.

As previously observed,^8,9^ all DR*lyp/lyp* ND rats developed T1D by day 83. The mean time to onset for DR*lyp/lyp* ND rats was 62.5±7.7 days (mean ± standard deviation), 62.6±6.5 days for DR*lyp/lyp* ND+Lp299v rats, 72.2±9.4 days for DR*lyp/lyp* HCD rats, and 84.9±14.3 days for DR*lyp/lyp* HCD+Lp299v rats (Figure 1a, b). While T1D was not delayed in DR*lyp/lyp* ND+Lp299v rats relative to DR*lyp/lyp* ND rats, one DR*lyp/lyp* ND+Lp299v rat did not develop diabetes. Relative to DR*lyp/lyp* ND rats, onset was delayed in DR*lyp/lyp* HCD rats and 8.3% did not develop diabetes. DR*lyp/lyp* HCD+Lp299v rats exhibited the most robust delay of T1D onset relative to DR*lyp/lyp* ND rats and were also delayed relative to DR*lyp/lyp* HCD rats; further, 25% of the DR*lyp/lyp* HCD+Lp299v rats were protected from developing diabetes during the 130-day study period.

### Plasma cytokine and metabolite analyses

Plasma cytokine/chemokine levels were measured at 40-days of age, which is prior to the development of insulitis in DR*lyp/lyp* rats provided ND.^10^ Elevated levels of IL-1B, CCL5, GM-CSF, IL-10, MCP-1, eotaxin, IL-4, IL-12(p70), IL-6, TNFa and G-CSF were measured in DR*lyp/lyp* ND rats compared to F*lyp/lyp* ND rats. Lp299v supplement of DR*lyp/lyp* rats under either diet did not alter plasma cytokine/chemokine levels (Figure S1).

SCFA exert anti-inflammatory effects on immune cells^38–41^ as well as beneficial effects on insulin secretion and β-cell survival/proliferation.^42^ Acetate, propionate and butyrate are the most abundant SCFA in the gut compared to isobutyrate, valerate, and isovalerate.^43^ This pattern was generally observed for plasma SCFA of 40-day-old rats across the groups (Figure 1c). Significantly higher propionate and butyrate levels were measured in DR*lyp/lyp* HCD+Lp299v rats relative to all other DR*lyp/lyp* groups. Overall, the SCFA profile of DR*lyp/lyp* HCD+Lp299v rats was most correlated with nondiabetic F*lyp/lyp* rats (Figure 1d).

### Analysis of the microbiota

**Figure 2.**
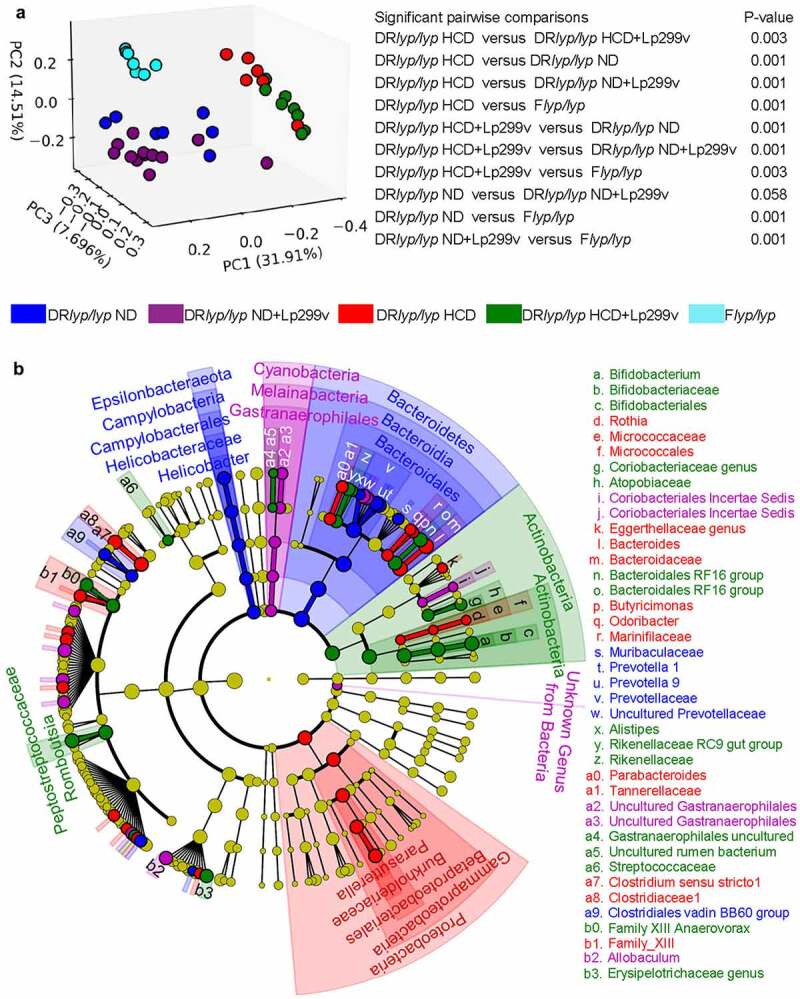
Analysis of the fecal microbiota in the DR*lyp/lyp* ND (n = 7), DR*lyp/lyp* ND+Lp299v (n = 13), DR*lyp/lyp* HCD (n = 7), DR*lyp/lyp* HCD+Lp299v (n = 8) and F*lyp/lyp* (n = 7) groups. A total of 857,129 quality reads were obtained from the 40 samples. The sequences were collapsed into 1,465 unique ASVs, and represented a total of 14 phyla, 22 classes, 31 orders, 62 families, 172 genera, and 292 species. A. Beta diversity was assessed among experimental conditions using the Bray-Curtis dissimilarity index and displayed as a Principal Coordinate Analysis plot. B. Cladogram of the fecal communities of the DR*lyp/lyp* ND, DR*lyp/lyp* ND+Lp299v, DR*lyp/lyp* HCD, and DR*lyp/lyp* HCD+Lp299v groups. The nodes indicate the abundance of the microorganism. The segments with different colors show the most abundant phyla and the corresponding dominant branches. Different shading denotes different taxonomic levels.

The stool microbiome of 40-day old rats from each group was analyzed by 16S rRNA gene sequencing. Among the groups, α-diversity (richness and evenness of amplicon sequence variants (ASVs) within a population) did not significantly differ. Among the groups, β-diversity (differences in ASV abundances between populations) was different in 9 of the 10 pair-wise comparisons (Figure 2a), and lower in DR*lyp/lyp* HCD rats compared to DR*lyp/lyp* HCD+Lp299v rats. Principal coordinate analysis shows that strain and diet had a larger influence on β-diversity than did Lp299v supplement.

Among DR*lyp/lyp* rats provided ND and HCD, with and without Lp299v supplementation, we identified differentiating features within the stool communities using the Linear Discriminant Analysis (LDA)-based LEfSe approach.^32^ The structures of enriched bacteria in the communities were distinct (Figure 2b). The phylum Bacteroidetes was a differentiating feature of DR*lyp/lyp* ND rats (LDA score = 4.84, p = .02), consistent with the associations of this phylum with human T1D progression.^44^ *Helicobacteraceae* family members, constituents of the normal gut flora and recognized pathogenic agents in colitic diseases,^45^ were also associated with the DR*lyp/lyp* ND group (LDA score = 4.1, p = 2.3E-03). The phylum Actinobacteria (LDA score = 4.75, p = 1.2E-03) which includes the family *Bifidobacteriaceae* (LDA score = 4.72, p = 1.1E-03) were distinguishing features of DR*lyp/lyp* HCD+Lp299v rats. Actinobacteria generally comprise a small percentage of the microbiota but are important in maintaining gut homeostasis,^46^ some *Bifidobacteriaceae* family members directly produce butyrate, propionate, and/or acetate, while others promote the growth of butyrogenic taxa through bacterial cross-feeding.^47^ The plasma propionate and butyrate levels positively correlated with the abundance of *Bifidobacteriaceae* in the stool community of DR*lyp/lyp* HCD+Lp299v rats (Spearman Correlation: propionate = 0.47, butyrate = 0.45, p = .001). *Erysipelotrichaceae* and *Clostridiales FamilyXIII Anaerovorax* were also differentiating features of the DR*lyplyp* HCD+Lp299v microbiota. The *Clostridiales Family XIII Anaerovorax* in DR*lyp/lyp* HCD+Lp299v stool also exhibited high correlations with plasma levels of propionate and butyrate (Spearman correlation: propionate = 0.58, p = .007, butyrate = 0.47, p = .03). Taken with the survival data and SCFA measurements, this analysis suggests that DR*lyp/lyp* HCD+Lp299v rats experienced the most beneficial alterations in the microbiota.

### Analysis of islet transcriptomes

The islet transcriptome of each group was examined at 40-days of age. We compared all groups to DR*lyp/lyp* ND islets and also directly compared DR*lyp/lyp* HCD and DR*lyp/lyp* HCD+Lp299v islets. A total of 5,325 differentially expressed probe sets were identified (│log_2_ ratio│ ≥0.263 and FDR<20%, Figure S2A). Hierarchical clustering and Pearson’s correlations found DR*lyp/lyp* ND and F*lyp/lyp* ND islets most dissimilar (Figure S2B and S2C). Pathway analyses identified Gene Ontology (GO) annotations related to bacterial antigen exposure, inflammation, ER function, ER stress, UPR, and glutathione metabolism (Figure S2D) differently enriched across the groups.

**Figure 3.**
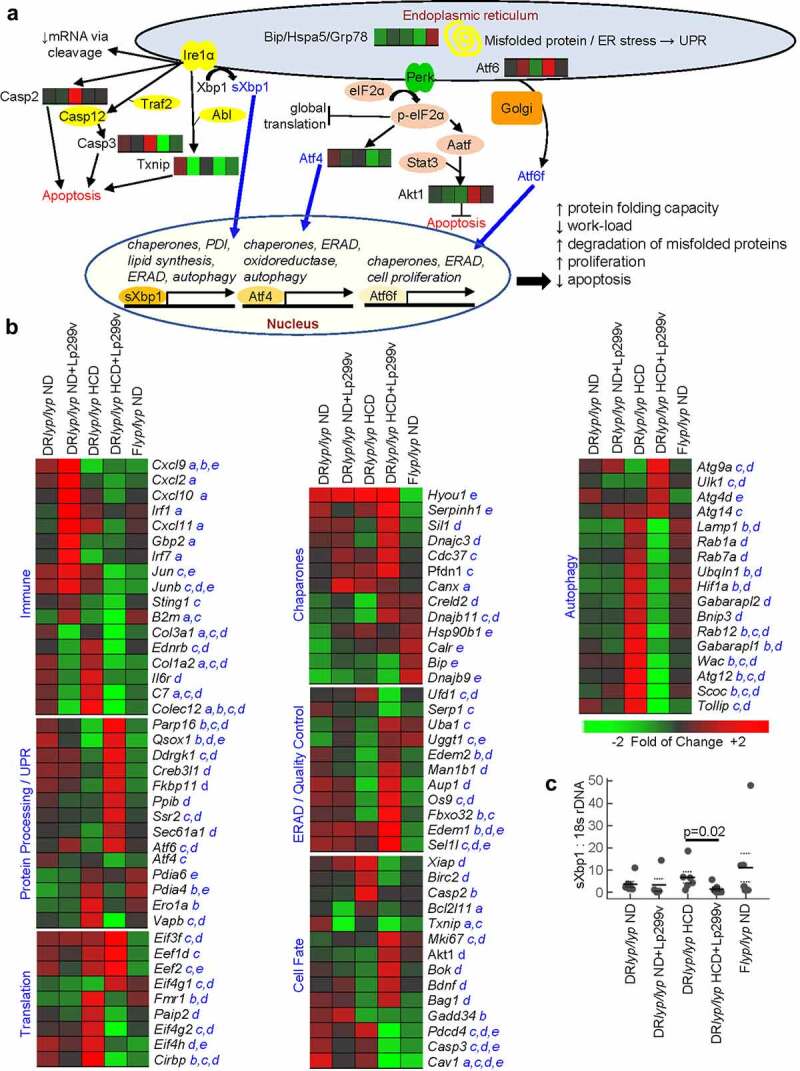
Components of the UPR are differentially expressed by DR*lyp/lyp* ND and F*lyp/lyp* ND islets and are modulated by HCD and Lp299v supplement. A. UPR activation pathways. Under normal conditions, the three major arms of the UPR are inactive due to the binding of Bip (binding immunoglobulin protein), which serves both as a chaperone and sensor of unfolded protein. With the accumulation of unfolded protein, Bip disassociates from Ire1α, Perk, and Atf6. Ire1α and Perk undergo dimerization and autophosphorylation. Activated IRE1α cleaves *Xbp1* mRNA, and when translated, spliced Xbp1 (sXbp1) mediates transcription of chaperones and genes necessary for lipid synthesis, ERAD, and autophagy. Activated Perk phosphorylates eIF2α, which inihibits translation and activates Atf4. Atf4 fosters the transcription of genes encoding chaperones, oxidoreductases, ERAD, and autophagy. Atf6 translocates to the Golgi complex after it is released from Bip, where it is cleaved to generate Atf6f. Atf6f fosters transcription of genes encoding protein chaperones and ERAD, and triggers β-cell proliferation. The induction of Akt1 and reduction of Txnip inhibit cell death. Prolonged ER stress and UPR activation increase caspase and Txnip expression, and activate apoptotic pathways. Heatmaps for regulated transcripts belonging to these pathways are shown, group order and thresholds match heatmaps shown in panel B. B. Expression levels of well annotated transcripts related to immune function and UPR activation. The comparisons that reached a threshold of │log_2_ ratio│ ≥ 0.263 and FDR<20% are indicated: a. DR*lyp/lyp* ND versus DR*lyp/lyp* ND+Lp299v; b. DR*lyp/lyp* ND versus DR*lyp/lyp* HCD; c. DR*lyp/lyp* ND versus DR*lyp/lyp* HCD+Lp299v; d. DR*lyp/lyp* HCD versus DR*lyp/lyp* HCD+Lp299v; e. DR*lyp/lyp* ND versus F*lyp/lyp* ND. C. Measurement of islet sXBP1 expression by quantitative RT-PCR in 5–6 individual rats per group at 40-days of age. Solid line indicates mean, dotted line indicates standard deviation. The statistical significance of differences was assessed using a t-test (two-tailed).

Independent of diet, Lp299v supplement reduced islet expression of certain transcripts associated with immune regulation (*Il6r*), fibrosis (*Col1a2, Col1a3*), and exposure to lipopolysaccharide (*C7, Ednrb, Colec12*). However, many immune transcripts exhibited discordant changes after Lp299v supplement in a diet dependent manner (Figure 3). Increased expression of *B2m, Cxcl2, Cxcl9, Cxcl10, Cxcl11, Irf1*, and *Irf7* was observed in DR*lyp/lyp* ND+Lp299v islets, while decreased expression was observed in DR*lyp/lyp* HCD+Lp299v islets, suggesting a greater anti-inflammatory effect under gluten-free conditions.

Across the groups, numerous UPR-related transcripts exhibited differential expression (Figure 3a, Table S1). Relative to F*lyp/lyp* rats, DR*lyp/lyp* islets expressed lower levels of the chaperone *Bip* and its insulin-binding co-chaperone *Dnajb9*, which were not modulated by diet or Lp299v. Differences in transcripts encoding IRE1α (*Ern1*), Perk (*Eif2ak3*), or *Eif2a* expression were not observed. However, *Parp16*, which encodes a protein required for Perk, Ire1α and UPR activation,^48^ and *Ddrgk1* which encodes a protein that controls IRE1α protein stability,^49^ were significantly upregulated in DR*lyp/lyp* HCD+Lp299v islets. *Atf4* expression was highest in DR*lyp/lyp* ND islets and significantly reduced in DR*lyp/lyp* HCD+Lp299v islets. *Atf6* transcript was not different between DR*lyp/lyp* ND and F*lyp/lyp* ND islets, however, Lp299v increased *Atf6* expression, most significantly in DR*lyp/lyp* rats provided HCD. DR*lyp/lyp* HCD+Lp299v islets expressed the lowest levels of *Vapb*, which encodes a protein that activates the Ire1/Xbp pathway and inhibits the Atf6 pathway.^50^ Differences in total *Xbp1* expression were not detected, however the highest spliced Xbp1 (*sXbp1*) expression was observed in DR*lyp/lyp* HCD islets (Figure 3c).

All three arms of the UPR can activate ERAD, which targets misfolded proteins within the ER for ubiquitination and proteasomal degradation (Figure 3a). Independent of diet, Lp299v supplement increased UDP-glucose-glycoprotein glucosyltransferase (*Uggt1*) expression, which reglucosylates incompletely folded glycoproteins and promotes their reassociation with the chaperones calreticulin and calnexin. Transcripts encoding these chaperones (*Calr, Canx*) were also increased by HCD and Lp299v in DR*lyp/lyp* islets. Misfolded proteins are removed from calnexin and calreticulin by ER degradation-enhancing α-mannosidase-like protein family members (EDEM) and ER mannosidase I, fostering their retrotranslocation from the ER to the cytosol. Numerous ERAD-related transcripts (*Aup1, Edem1, Edem2, Fbxo32, Man1b1, Os9, Sel1l*, and *Uba1*) exhibited the highest expression in DR*lyp/lyp* HCD+Lp299v islets. Further, *Serp1*, which is induced by ER stress and protects unfolded proteins from ERAD, exhibited the lowest expression in DR*lyp/lyp* HCD+Lp299v islets. *Ufd1* transcript was lowest in DR*lyp/lyp* HCD+Lp299v islets, repression of this ubiquitin-recognition protein triggers cell cycle delay to foster efficient ERAD-mediated clearance of misfolded protein.^51^ Overall, the DR*lyp/lyp* HCD+Lp299v islet transcriptome was consistent with enhanced ERAD activity (Figure 3b).

The DR*lyp/lyp* HCD+Lp299v islet transcriptome exhibited higher abundance of transcripts related to translation initiation, positive regulation of insulin secretion, chaperone binding and protein processing (Figure 3b). This included eukaryotic initiation factors, including *Eif4g1* which regulates glucose homeostasis and β-cell function,^52^ and *Eif3f* a translational enhancer that improves protein synthesis efficiency.^53^ *Eef2 and Eef1d*, which encode translation elongation factors, were also most abundant in DR*lyp/lyp* HCD+Lp299v islets. DR*lyp/lyp* HCD+Lp299v islets exhibited reduced abundance of translational repressors (*Paip2, Eif4g2, Fmr, Cirbp*), while showing high abundance of transcripts encoding products required for protein processing within the ER. These included *Ssr2 and Sec61a1*, necessary for translocating nascent peptides into the ER, and chaperones/co-chaperones (*Cdc37, Creld2, Dnajb11, Hsp90b1, Pfdn1, Serpinh1, Sil1*) including *Dnajc3* which maintains insulin-folding homeostasis.^54^ Transcripts encoding isomerases (*Ppib, Fkbp11, Pdia6*) and oxidases necessary for protein maturation within the ER exhibited higher abundance in DR*lyp/lyp* HCD+Lp299v islets, this included quiescin sulfhydryl oxidase 1 (*Qsox1*), which also acts to inhibit autophagy.

ER stress that exceeds the capacity of the UPR and ERAD can induce autophagy, a process that sequesters compromised portions of the ER within autophagic vesicles and delivers them to lysosomes for degradation. While DR*lyp/lyp* HCD+Lp299v islets exhibited high relative expression of some autophagy-related transcripts (*Atg4d, Atg9a, Atg14, Ulk1*), the biological process “positive regulation of macroautophagy” was most enriched in DR*lyp/lyp* HCD islets (Figure S2D) and numerous transcripts annotated under this GO Term (*Atg12, Bnip3, Hif1a, Gabarapl1, Gabarapl2, Rab1a, Rab7a, Rab12, Scoc, Tollip, Ubqln1, Wac*) were most abundant in this group (Figure 3b).

Unresolved ER stress promotes apoptosis. Across the groups, transcript levels for many pro-apoptotic Bcl2 family members (*Bad, Bak, Bax, Bik, Bid)* were not different. However, DR*lyp/lyp* HCD+Lp299v islets exhibited greater abundance of the apoptosis inducer *Bok*, while DR*lyp/lyp* HCD islets exhibited greater abundance of *Bcl2l11*, which encodes the apoptotic initiator Bim. *Casp2* and *Casp3*, which are involved in the execution-phase of apoptosis, were elevated in DR*lyp/lyp* HCD islets and reduced by Lp299v supplement. Transcripts for other apoptosis mediators also showed reduced abundance in DR*lyp/lyp* HCD+Lp299v islets (*Cav1, Pdcd4, Gadd34, Txnip*). DR*lyp/lyp* HCD islets expressed the highest expression of the caspase inhibitors *Xiap* and *Birc2*, while DR*lyp/lyp* HCD+Lp299v islets exhibited higher abundance of transcripts related to survival/proliferation (*Akt1, Mki67, Hyou1, Bdnf, Bag1*).

Overall, the analyses suggest that DR*lyp/lyp* HCD+Lp299v islets expressed a transcriptional program favoring ERAD, chaperone expression/protein processing, and cell survival/proliferation, whereas DR*lyp/lyp* HCD islets exhibited a signature consistent with autophagy.

### UPR protein expression and localization

Pancreatic islets consist of α, β, γ, and δ cells which respectively produce glucagon, insulin, pancreatic polypeptide, and somatostatin and possess respective distributions of ~21%, ~68%, ~5%, and ~6%. To colocalize and confirm activities detected in the transcriptomic analyses to β-cells, pancreata of 40-day old rats were subjected to immunofluorescence staining for insulin and UPR-related proteins.

**Figure 4.**
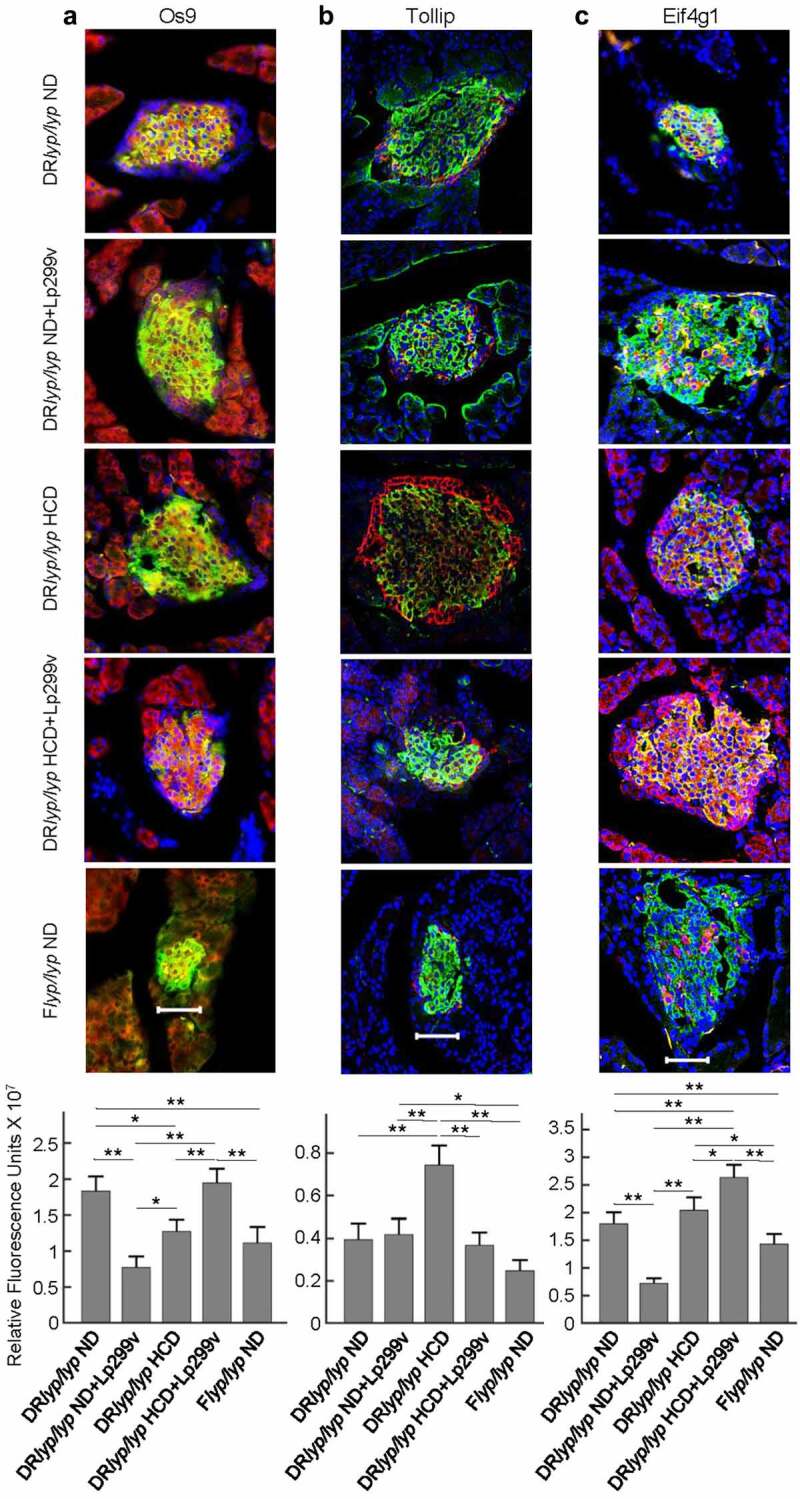
Immunofluorescence staining was performed on frozen pancreatic sections of 40-day-old rats for localization and quantification of relevant markers to β-cells. For each staining protocol, 3–5 animals were evaluated for each experimental condition, and 12–30 islets per animal were examined. For each protocol, negative controls lacking primary antibodies failed to show staining (not shown). As indicated, panels A-C show representative staining for DR*lyp/lyp* ND, DR*lyp/lyp* ND+Lp299v, DR*lyp/lyp* HCD, DR*lyp/lyp* HCD+Lp299v, and F+/+ ND islets under each staining protocol. The bottom panel of each analysis shows quantification of fluorescence intensity in islets of the five experimental conditions for each staining protocol. * indicates p < .05; ** indicates p < .01 (t-test, 1-tailed). Magnification ×40; scale bar 50 μm. A. Os9 (ERAD marker, Alexa594/red), insulin (FITC/green), and nuclei (DAPI/blue). B. Tollip (autophagy marker, Alexa594/red), insulin (FITC/green), and nuclei (DAPI/blue). C. Eif4g1 (eukaryotic initiation factor, Alexa594), insulin (FITC/green), and nuclei (DAPI/blue).

The DR*lyp/lyp* HCD+Lp299v islet transcriptome was consistent with enhanced ERAD and included *Os9*, which encodes an ER-resident lectin that delivers misfolded glycoproteins to the Sel1L-Hrd1 ERAD complex. Staining for Os9 colocalized with insulin. In line with the transcriptomic data, the quantified Os9 signal in β-cells was higher in the DR*lyp/lyp* HCD+Lp299v group relative to the other groups (Figure 4a).

The DR*lyp/lyp* HCD islets transcriptome was consistent with enhanced autophagy and included *Tollip*, which encodes a protein that mediates the recruitment of autophagy receptors to protein aggregates.^55^ While immunofluorescent staining for Tollip was detected in cells located at the islet periphery (α-cells) in all groups, the quantified Tollip signal of insulin-positive β-cells was significantly higher in DR*lyp/lyp* HCD+Lp299v islets relative to the other groups, paralleling the transcriptomic data (Figure 4b).

Translation initiation complex proteins, including Eif4g1,^52^ are key regulators of insulin biosynthesis. Immunofluorescent staining localized islet Eif4g1 expression to β-cells. Consistent with the transcriptomic analysis, higher Eif4g1 expression was observed in DR*lyp/lyp* HCD+Lp299v β-cells relative to the other groups (Figure 4c).

The transcriptomic studies revealed differential expression related to cell fate/proliferation, particularly between DR*lyp/lyp* HCD and DR*lyp/lyp* HCD+Lp299v islets. However, TUNEL and Ki67 immunofluorescent staining did not detect differences in the number of apoptotic and proliferating islet cells in 40-day-old rats of the groups (data not shown).

### Response of rat insulinoma cells to SCFA

**Figure 5.**
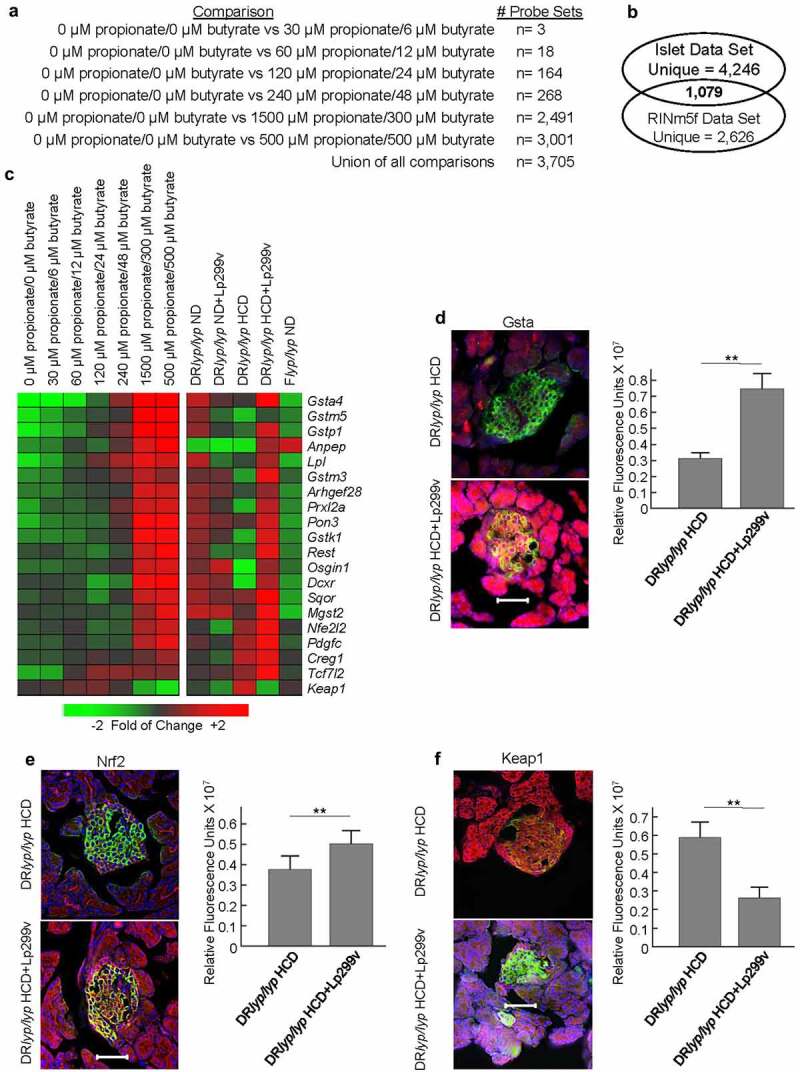
Induction of antioxidative defense responses by SCFA. A. RINm5f cells were cultured in the presence of propionate and butyrate for 24 hours. Gene expression profiling was conducted on three independent cultures for each experimental condition. The data structure defining the number of total probe sets regulated to thresholds (│log_2_ ratio│ ≥ 0.585 (1.5-fold) and FDR<20%) in each comparison when compared to cultures lacking SCFA is shown. B. Venn diagram illustrating relationship between the RINm5f data set to the islet data set (Figure 4). C. Expression levels of well-annotated transcripts related to antioxidative defense in RINm5f cells after exposure to SCFA (left) and in day 40 DR*lyp/lyp* ND, DR*lyp/lyp* ND+Lp299v, DR*lyp/lyp* HCD, DR*lyp/lyp* HCD+Lp299v, and F+/+ ND islets (*Nfe2l2* did not meet both fold of change threshold in RINm5f cells). D., E., F., Immunofluorescence staining was performed on frozen pancreatic sections of 40-day-old rats for localization and quantification of antioxidative defense markers to β-cells. For each staining protocol, 3–5 animals were evaluated for each experimental condition, and 12–30 islets per animal were examined. For each protocol, negative controls lacking primary antibodies failed to show staining (not shown). As indicated, panels D-F show representive staining for DR*lyp/lyp* HCD and DR*lyp/lyp* HCD+Lp299v under each staining protocol. The right panel of each analysis shows quantification of fluorescence intensity in islets of the two experimental conditions for each staining protocol. * indicates p < .05; ** indicates p < .01 (t-test, 1-tailed). Magnification, ×40; scale bar, 50 μm. D. Gsta (Alexa594/red), insulin (FITC/green), and nuclei (DAPI/blue). E. Nrf2 (Alexa594/red), insulin (FITC/green), and nuclei (DAPI/blue). F. Keap1 (Alexa594/red), insulin (FITC/green), and nuclei (DAPI/blue).

To gain insight on how SCFA potentially influenced the islet phenotypes, we conducted transcriptomic analyses on RINm5f cells cultured with propionate and butyrate levels that spanned the plasma concentrations measured across the groups. The number of differentially expressed transcripts increased in a concentration-dependent manner across the culture conditions (Figure 5a, b, Table S1). In total, 3,705 probe sets were detected and a significant proportion of these overlapped with those regulated among islets of the groups (1,079/5,325 probe sets, 20.3%, *X^2^*, p < 10E-10). However, this intersection did not include the well-annotated UPR-related transcripts illustrated in Figure 3. Further, ontological analysis failed to identify GO annotations related to UPR, ERAD, or autophagy, suggesting that butyrate and propionate did not directly mediate the enhanced ERAD activity observed in DR*lyp/lyp* HCD+Lp299v islets.

Consistent with the islet data set, ontological analysis of the 1,079 probe set intersection detected GO annotations related to glutathione metabolism and antioxidant defense, including glutathione transferase activity (p = 1.6E-2), cellular oxidant detoxification (p = 2.7E-2), and glutathione binding (p = 4.1E-2). Transcripts associated with these terms exhibited SCFA concentration-dependent increases in RINm5f cells and also exhibited the highest expression in DR*lyp/lyp* islets (Figure 5c). These included many glutathione S-transferase (Gst) isoforms (*Gsta4, Gstk1, Gstp1, Gstm3, Gstm5*, and *Mgst2*), enzymes that detoxify reactive electrophilic compounds by catalyzing their conjugation to glutathione. Transcripts for other antioxidative defense (*Anpep, Creg1, Pon3, Prxl2a, Rest, Sqor*), cytoprotective (*Arhgef28, Dcxr, Osgin1, Pdgfc*) and antiapoptotic proteins (*Tcf7l2*) were similarly upregulated. *Keap1* was down-regulated by SCFA and exhibited the lowest expression DR*lyp/lyp* HCD+Lp299v islets. *Keap1* encodes a negative regulator for nuclear factor E2 p45-related factor 2 (Nrf2), a transcription factor that activates many antioxidant defense genes. While exhibiting an expression pattern paralleling the aforementioned genes, Nrf2 transcript (*Nfe2l2)* was not regulated to thresholds in RINm5f cells. However, *Nfe2l2* was regulated to thresholds in the islet data set and exhibited the highest expression in DR*lyp/lyp* HCD+Lp299v islets. Consistent with this observation, numerous transcripts downstream of Nrf2 were more abundant in DR*lyp/lyp* HCD+Lp299v islets (*Gsta4, Gstm3, Gstp1, Lpl, Mgst2*). Immunofluorescent staining localized islet Gsta and Nrf2 expression to β-cells and confirmed their higher expression in DR*lyp/lyp* HCD+Lp299v compared to DR*lyp/lyp* HCD islets (Figure 5d,e). Immunofluorescent staining confirmed lower Keap1 expression by DR*lyp/lyp* HCD+Lp299v islets compared to DR*lyp/lyp* HCD islets (figure 5f). These results support that butyrate and propionate contributed to the increased antioxidative defense response observed in DR*lyp/lyp* HCD+Lp299v islets.

### Assessment of β-cell function

ER dysfunction in β-cells is characterized by the accumulation and secretion of proinsulin,^56^ which can be detected by measurement of the ratio of circulating proinsulin to C-peptide. Higher proinsulin to C-peptide ratios denote ER dysfunction and elevated ratios have been found to precede diabetes onset. DR*lyp/lyp* ND rats possessed significantly higher proinsulin:C-peptide ratios compared to DR*lyp/lyp* HCD and DR*lyp/lyp* HCD+Lp299v rats (Figure S3). The proinsulin:C-peptide ratio was impacted more by the diet than by Lp299v supplement, and when comparing within diet, Lp299v did not significantly improve or reduce the proinsulin:C-peptide ratio in 40-day-old rats.

## Discussion:

Since its isolation from healthy human intestinal mucosa, Lp299v supplementation has been reported to benefit gastrointestinal health, improve gut barrier function, and reduce inflammation.^57–59^ While the beneficial effects of other lactic acid bacteria taxa have been examined in diabetes,^60,61^ to our knowledge, Lp299v has not been studied in T1D. Given our experience with this probiotic to lower systemic inflammation in men with coronary artery disease,^62^ we hypothesized that Lp299v supplementation would lower the endogenous inflammatory state in BB rats and slow diabetes progression. Because BB rats are gluten intolerant and gluten-free HCDs slow diabetes progression in this model,^8,19^ we investigated Lp299v supplementation in the presence of gluten-containing and gluten-free diets. Diabetes-free survival of DR*lyp/lyp* rats provided HCD+Lp299v was greater than animals provided ND, ND+Lp299v, or HCD alone.

Previously, we determined that weaning diabetes-inducible BB DR+/+ pups onto HCD increased the abundance of lactobacilli and butyrate-producing taxa compared to animals provided ND.^8^ Given the improved diabetes-free survival of DR*lyp/lyp* HCD+Lp299v rats over the DR*lyp/lyp* HCD group, we postulated that Lp299v supplement promoted additional beneficial shifts in the microbiota. In humans, Lp299v supplement improved microbial diversity.^63^ Here, higher β-diversity was measured in DR*lyp/lyp* HCD+Lp299v rats compared to the other groups. Circulating propionate and butyrate levels were increased in DR*lyp/lyp* HCD+Lp299v rats, and these correlated with the abundances of *Bifidobacteriaceae* and *Clostridiales*, families known to include members that directly produce these SCFA.

SCFA are recognized by the G-protein coupled receptors free fatty acid receptor 2 (FFAR2) and FFAR3. Activation of these receptors by acetate, propionate, and butyrate promote anti-inflammatory responses in innate and adaptive immune cells,^38–40^ while fostering Treg differentiation and activation.^41^ Probiotic supplementation of unaffected T1D siblings^22^ and metabolite-based dietary supplementation of T1D patients^21^ have been shown to increase circulating SCFA levels and promote greater regulatory bias in T and B cell profiles. Such analyses were not conducted here because the lymphopenia of DR*lyp/lyp* rats^23^ confounds peripheral immunophenotyping studies and comparisons to human interventions.

In contrast, islets of DR*lyp/lyp* rats and human T1D patients exhibit important similarities. The DR*lyp/lyp* insulitic lesion begins 2–3 weeks before the onset of hyperglycemia and like humans, insulitis in DR*lyp/lyp* rats is not preceded by peri-insulitis and consists mainly of Th1-lymphocytes. Prior studies have shown that islets of DR*lyp/lyp* rats and human T1D patients both exhibit elevated innate immune activity that includes NF-κB activation and β-cell chemokine expression.^8,64,65^ β-cells are susceptible to overload and ER stress due to high fluctuating secretory demands, the propensity of proinsulin to misfolding,^66^ and relatively weak anti-oxidative defenses and repair systems for oxidative DNA damage.^67,68^ Notably, DR*lyp/lyp* islets exhibit reduced anti-oxidative defenses compared to F*lyp/lyp* rats.^37^ This islet-specific deficiency may contribute to DR*lyp/lyp* T1D susceptibility because treatment of pups with the antioxidant N-acetyl cysteine reduces the severity of insulitis and delays diabetes onset.^37^ Importantly, β-cells express FFAR2 and FFAR3, and prior *in vitro* studies demonstrate that SCFA enhance viability, stimulate insulin secretion, support mitochondrial function, and promote antioxidant defense.^69–71^

DR*lyp/lyp* ND rats and DR*lyp/lyp* ND+Lp299v rats were indifferent in terms of diabetes-free survival and SCFA levels. The DR*lyp/lyp* ND+Lp299v islet transcriptome exhibited elevated inflammatory activity in the ontological analysis as well as the highest transcript abundance for several proinflammatory mediators. This potentially detrimental change was observed only when Lp299v was provided with ND, but not when Lp299v was provided with HCD. Similarly, in our study of coronary artery disease, Lp299v failed to lower inflammation among daily alcohol drinkers.^62^ Like alcohol consumption, gluten intolerance promotes dysbiosis, as well as increased gut permeability, circulating endotoxin, and systemic inflammation.^8,72^ In DR*lyp/lyp* ND+Lp299v rats, such physiologic changes may account for the blunted anti-inflammatory effects observed in the islet transcriptome and the failure of the probiotic to improve diabetes-free survival. This may be relevant to probiotic interventions aimed at benefiting children with or at risk of developing T1D, as ~6% of T1D patients develop celiac disease, compared to ~1% of the general population, suggesting a shared role of dietary gluten exposure in T1D and celiac disease pathogenesis.

The DR*lyp/lyp* HCD and DR*lyp/lyp* HCD+Lp299v groups both exhibited delayed T1D onset compared to DR*lyp/lyp* rats provided ND. Further, compared to DR*lyp*/*lyp* ND rats, the islet transcriptomes of rats provided HCD exhibited reduced inflammatory activity, and this was more evident in DR*lyp/lyp* HCD+Lp299v islets. Inflammation promotes oxidative stress and disrupts ER proteostasis, which fosters β-cell dysfunction and T1D pathogenesis. Consistent with this paradigm, in the presence of the gluten-mediated inflammatory state, islets of the DR*lyp/lyp* ND and DR*lyp/lyp* ND+Lp299v groups did not exhibit elevated ERAD, autophagy or cell survival/proliferation activity, and exhibited poorer function as reflected by higher proinsulin:C-peptide ratios in DR*lyp/lyp* ND rats. DR*lyp/lyp* HCD rats exhibited elevated autophagy-related activity which was confirmed by immunostaining for Tollip. In contrast to DR*lyp/lyp* HCD rats, DR*lyp/lyp* HCD+Lp299v rats exhibited the greatest diabetes-free survival, the highest *Atf6* expression, the lowest *sXbp1* and *Atf4* islet gene expression, and elevated ERAD-related activity. This observation is potentially important, as ERAD has recently been determined to play a key role in targeting misfolded proinsulin for proteosomal degradation in support of glucose-stimulated insulin secretion.^73^

To address whether the enhanced ERAD-related activity in DR*lyp/lyp* HCD+Lp299v islets was a consequence of SCFA directly acting on β-cells, RINm5f cells were cultured with propionate and butyrate. While reports describing the SCFA levels normally present in islets are lacking, we tested concentrations spanning 1) the plasma levels measured in the study groups; and 2) the SCFA gradient that exists between human hepatic portal blood and peripheral circulation.^74^ In RINm5f cells, transcripts related to ERAD and autophagy were not directly modulated in response to increasing SCFA concentrations. This does not exclude the possibility that these SCFA indirectly impact islet function through other mechanisms, including enteroendocrine, neural, and/or immune pathways (reviewed in^42^). It is also possible that other microbial metabolite/s associated with Lp299v supplement modulate the UPR. However, consistent with butyrate and propionate as established activators of the Keap1-Nrf2 pathway,^74^ these SCFA inhibited *Keap1* expression, promoted *Nfe2l2* expression, and fostered expression of antioxidant target genes in RINm5f cells. This paralleled the transcriptomic profile and β-cell immunostaining for Nrf2, Keap1, and Gsta in DR*lyp/lyp* HCD+Lp299v islets. Importantly, Nrf2 has emerged as a promising therapeutic target for diabetes. Increasing Nrf2 activity, either through *Keap1* deletion or through treatment with the Nrf2-activator bardoxolone methyl, promotes rodent and human β-cell proliferation in both *in vitro* and *in vivo* settings.^75–77^ While the altered UPR responses in DR*lyp/lyp* HCD and DR*lyp/lyp* HCD+Lp299v islets cannot be directly attributed to SCFA levels, these effects may indirectly stem from the SCFA-mediated anti-inflammatory effects of probiotic supplementation and/or increased expression of Nrf2 and its downstream targets.

Our future studies are aimed at understanding how environmental changes and dysbiosis may have contributed the higher T1D incidences observed worldwide. This preclinical study links Lp299v supplement to improved diabetes-free survival as well as enhanced expression of β-cell ERAD and antioxidative defense responses. It also provides an initial framework for understanding how β-cells in newly diagnosed T1D patients may be influenced by Lp299v supplement in an ongoing intervention (https://www.clinicaltrials.gov:NCT04335656). Limitations of the present study include focus on a single pre-insulitis time-point, analysis of the microbiota by only amplicon sequencing, and lack of a heat-killed Lp299v group to enable differentiation of contact-mediated (e.g., lipoteichoic acid, exopolysaccharides, cell surface appendages) versus secreted (e.g., SCFAs, bacteriocins) molecules. The ability to modulate the β-cell UPR and boost antioxidative defense responses is significant as there is a need for safe, broadly applicable therapies to reduce diabetes risk in susceptible individuals and slow progression before and after clinical onset.

## Supplementary Material

Supplemental Material

## Data Availability

The data that support the findings of this study are available at The National Center for Biotechnology Information Sequence Read Archive (https://www.ncbi.nlm.nih.gov/sra; accession number PRJNA854152), The National Center for Biotechnology Information Gene Expression Omnibus (https://www.ncbi.nlm.nih.gov/geo/; accession number GSE198617), and within the article and its supplementary materials.

## References

[1] Patterson CC, Gyurus E, Rosenbauer J, Cinek O, Neu A, Schober E, Parslow RC, Joner G, Svensson J, Castell C, et al. Trends in childhood type 1 diabetes incidence in Europe during 1989-2008: evidence of non-uniformity over time in rates of increase. Diabetologia. 2012;55:2142–19. doi:10.1007/s00125-012-2571-8. 22638547

[2] Vaarala O, Atkinson MA, Neu J. The “perfect storm” for type 1 diabetes: the complex interplay between intestinal microbiota, gut permeability, and mucosal immunity. Diabetes. 2008;57:2555–2562. doi:10.2337/db08-0331. 18820210 PMC2551660

[3] Vehik K, Dabelea D. The changing epidemiology of type 1 diabetes: why is it going through the roof? Diabetes Metab Res Rev. 2011;27:3–13. doi:10.1002/dmrr.1141. 21218503

[4] Niland B, Cash BD. Health benefits and adverse effects of a Gluten-Free diet in non-Celiac disease patients. Gastroenterol Hepatol (N Y). 2018;14:82–91. 29606920 PMC5866307

[5] Cushing K, Alvarado DM, Ciorba MA. Butyrate and mucosal inflammation: new scientific evidence supports clinical observation. Clin Transl Gastroenterol. 2015;6:e108. doi:10.1038/ctg.2015.34. 26312412 PMC4816278

[6] Chen YG, Cabrera SM, Jia S, Kaldunski ML, Kramer J, Cheong S, Geoffrey R, Roethle MF, Woodliff JE, Greenbaum CJ, et al. Molecular signatures differentiate immune States in type 1 diabetic families. Diabetes. 2014;63:3960–3973. doi:10.2337/db14-0214. 24760139 PMC4207392

[7] Chen YG, Mordes JP, Blankenhorn EP, Kashmiri H, Kaldunski ML, Jia S. Temporal induction of immunoregulatory processes coincides with age-dependent resistance to viral-induced type 1 diabetes. Genes Immun. 2013;14:387–400. doi:10.1038/gene.2013.31. 23739610 PMC4027975

[8] Henschel AM, Cabrera SM, Kaldunski ML, Jia S, Geoffrey R, Roethle MF. Modulation of the diet and gastrointestinal microbiota normalizes systemic inflammation and beta-cell chemokine expression associated with autoimmune diabetes susceptibility. PloS one. 2018;13:e0190351. doi:10.1371/journal.pone.0190351. 29293587 PMC5749787

[9] Geoffrey R, Jia S, Kwitek AE, Woodliff J, Ghosh S, Lernmark A. Evidence of a functional role for mast cells in the development of type 1 diabetes mellitus in the BioBreeding rat. J Immunol. 2006;177:7275–7286. doi:10.4049/jimmunol.177.10.7275. 17082646

[10] Hessner MJ, Wang X, Meyer L, Geoffrey R, Jia S, Fuller J, Lernmark A, Ghosh S. Involvement of eotaxin, eosinophils, and pancreatic predisposition in development of type 1 diabetes mellitus in the BioBreeding rat. J Immunol. 2004;173:6993–7002. doi:10.4049/jimmunol.173.11.6993. 15557196

[11] Zhang IX, Raghavan M, Satin LS, Casanueva FF, Trimboli P. The endoplasmic reticulum and calcium homeostasis in pancreatic beta cells. Endocrinology. 2020;46(2):161. doi:10.23736/S2724-6507.20.03356-8. 31796960 PMC7028010

[12] Cao SS, Kaufman RJ. Endoplasmic reticulum stress and oxidative stress in cell fate decision and human disease. Antioxid Redox Signal. 2014;21(3):396–413. doi:10.1089/ars.2014.5851. 24702237 PMC4076992

[13] Tersey SA, Nishiki Y, Templin AT, Cabrera SM, Stull ND, Colvin SC, Evans-Molina C, Rickus JL, Maier B, Mirmira RG, et al. Islet beta-cell endoplasmic reticulum stress precedes the onset of type 1 diabetes in the nonobese diabetic mouse model. Diabetes. 2012;61:818–827. doi:10.2337/db11-1293. 22442300 PMC3314371

[14] Engin F, Yermalovich A, Nguyen T, Hummasti S, Fu W, Eizirik DL, Mathis D, Hotamisligil GS. Restoration of the unfolded protein response in pancreatic beta cells protects mice against type 1 diabetes. Sci Transl Med. 2013;5:211ra156. doi:10.1126/scitranslmed.3006534. PMC4169117 24225943

[15] Yang C, Diiorio P, Jurczyk A, O’Sullivan-Murphy B, Urano F, Bortell R. Pathological endoplasmic reticulum stress mediated by the IRE1 pathway contributes to pre-insulitic beta cell apoptosis in a virus-induced rat model of type 1 diabetes. Diabetologia. 2013;56(12):2638–2646. doi:10.1007/s00125-013-3044-4. 24121653 PMC4845659

[16] Marhfour I, Lopez XM, Lefkaditis D, Salmon I, Allagnat F, Richardson SJ, Morgan NG, Eizirik DL. Expression of endoplasmic reticulum stress markers in the islets of patients with type 1 diabetes. Diabetologia. 2012;55(9):2417–2420. doi:10.1007/s00125-012-2604-3. 22699564

[17] Marre ML, Piganelli JD. Environmental factors contribute to beta cell endoplasmic reticulum stress and neo-Antigen formation in type 1 diabetes. Front Endocrinol (Lausanne). 2017;8:262. doi:10.3389/fendo.2017.00262. 29033899 PMC5626851

[18] Delong T, Wiles TA, Baker RL, Bradley B, Barbour G, Reisdorph R, Armstrong M, Powell RL, Reisdorph N, Kumar N, et al. Pathogenic CD4 T cells in type 1 diabetes recognize epitopes formed by peptide fusion. Science. 2016;351(6274):711–714. doi:10.1126/science.aad2791. 26912858 PMC4884646

[19] Funda DP, Kaas A, Bock T, Tlaskalova-Hogenova H, Buschard K. Gluten-free diet prevents diabetes in NOD mice. Diabetes Metab Res Rev. 1999;15(5):323–327. doi:10.1002/(SICI)1520-7560(199909/10)15:5<323::AID-DMRR53>3.0.CO;2-P. 10585617

[20] Lund-Blix NA, Tapia G, Mårild K, Brantsaeter AL, Njølstad PR, Joner G, Skrivarhaug T, Størdal K, Stene LC. Amount of gluten in early life and risk of type 1. In: Sally MM, editor. diabetes 55th EASD annual meeting of the European Association for the study of diabetes. Barcelona Spain: Diabetologia; 2019. p. 1–600.

[21] Bell KJ, Saad S, Tillett BJ, McGuire HM, Bordbar S, Yap YA, Nguyen LT, Wilkins MR, Corley S, Brodie S, et al. Metabolite-based dietary supplementation in human type 1 diabetes is associated with microbiota and immune modulation. Microbiome. 2022;10:9. doi:10.1186/s40168-021-01193-9. 35045871 PMC8772108

[22] Cabrera SM, Coren AT, Pant T, Ciecko AE, Jia S, Roethle MF, Simpson PM, Atkinson SN, Salzman NH, Chen Y-G, et al. Probiotic normalization of systemic inflammation in siblings of type 1 diabetes patients: an open-label pilot study. Sci Rep. 2022;12:3306. doi:10.1038/s41598-022-07203-6. 35228584 PMC8885673

[23] MacMurray AJ, Moralejo DH, Kwitek AE, Rutledge EA, Van Yserloo B, Gohlke P, Speros SJ, Snyder B, Schaefer J, Bieg S, et al. Lymphopenia in the BB rat model of type 1 diabetes is due to a mutation in a novel immune-Associated nucleotide (Ian)-Related gene. Genome Res. 2002;12(7):1029–1039. doi:10.1101/gr.412702. 12097339 PMC186618

[24] Moralejo DH, Park HA, Speros SJ, MacMurray AJ, Kwitek AE, Jacob HJ, Lander ES, Lernmark Å. Genetic dissection of lymphopenia from autoimmunity by introgression of mutated Ian5 gene onto the F344 rat. J Autoimmun. 2003;21:315–324. doi:10.1016/S0896-8411(03)00138-0. 14624755 PMC7126882

[25] Caporaso JG, Lauber CL, Walters WA, Berg-Lyons D, Huntley J, Fierer N, Owens SM, Betley J, Fraser L, Bauer M, et al. Ultra-high-throughput microbial community analysis on the illumina HiSeq and MiSeq platforms. ISME J. 2012;6:1621–1624. doi:10.1038/ismej.2012.8. 22402401 PMC3400413

[26] Bolyen E, Rideout JR, Dillon MR, Bokulich NA, Abnet CC, Al-Ghalith GA, Alexander H, Alm EJ, Arumugam M, Asnicar F, et al. Reproducible, interactive, scalable and extensible microbiome data science using QIIME 2. Nat Biotechnol. 2019;37:852–857. doi:10.1038/s41587-019-0209-9. 31341288 PMC7015180

[27] Callahan BJ, McMurdie PJ, Rosen MJ, Han AW, Johnson AJ, Holmes SP. DADA2: high-resolution sample inference from Illumina amplicon data. Nat Methods. 2016;13:581–583. doi:10.1038/nmeth.3869. 27214047 PMC4927377

[28] Katoh K, Standley DM. MAFFT multiple sequence alignment software version 7: improvements in performance and usability. Mol Biol Evol. 2013;30:772–780. doi:10.1093/molbev/mst010. 23329690 PMC3603318

[29] Price MN, Dehal PS, Arkin AP. FastTree 2–approximately maximum-likelihood trees for large alignments. PLoS One. 2010;5:e9490. doi:10.1371/journal.pone.0009490. 20224823 PMC2835736

[30] Yilmaz P, Parfrey LW, Yarza P, Gerken J, Pruesse E, Quast C. The SILVA and “All-species Living Tree Project (LTP)” taxonomic frameworks. Nucleic Acids Res. 2014;42:D643–8. 24293649 10.1093/nar/gkt1209 PMC3965112

[31] Vazquez-Baeza Y, Pirrung M, Gonzalez A, Knight R. EMPeror: a tool for visualizing high-throughput microbial community data. Gigascience. 2013;2:16. doi:10.1186/2047-217X-2-16. 24280061 PMC4076506

[32] Segata N, Izard J, Waldron L, Gevers D, Miropolsky L, Garrett WS, Huttenhower C. Metagenomic biomarker discovery and explanation. Genome Biol. 2011;12:R60. doi:10.1186/gb-2011-12-6-r60. 21702898 PMC3218848

[33] Moreau NM, Goupry SM, Antignac JP, Monteau FJ, Le Bizec BJ, Champ MM, MARTIN L, Dumon H. Simultaneous measurement of plasma concentrations and 13C-enrichment of short-chain fatty acids, lactic acid and ketone bodies by gas chromatography coupled to mass spectrometry. J Chromatogr B Analyt Technol Biomed Life Sci. 2003;784:395–403. doi:10.1016/S1570-0232(02)00827-9. 12505787

[34] Gentleman RC, Carey VJ, Bates DM, Bolstad B, Dettling M, Dudoit S. Bioconductor: open software development for computational biology and bioinformatics. Genome Biol. 2004;5:R80. doi:10.1186/gb-2004-5-10-r80. 15461798 PMC545600

[35] Huang da W, Sherman BT, Lempicki RA. Systematic and integrative analysis of large gene lists using DAVID bioinformatics resources. Nat Protoc. 2009;4:44–57. doi:10.1038/nprot.2008.211. 19131956

[36] Yoon SB, Park YH, Choi SA, Yang HJ, Jeong PS, Cha JJ. Real-time PCR quantification of spliced X-box binding protein 1 (XBP1) using a universal primer method. PLoS One. 2019;14:e0219978. doi:10.1371/journal.pone.0219978. 31329612 PMC6645673

[37] Bogdani M, Henschel AM, Kansra S, Fuller JM, Geoffrey R, Jia S. Biobreeding rat islets exhibit reduced antioxidative defense and N-acetyl cysteine treatment delays type 1 diabetes. J Endocrinol. 2013;216:111–123. doi:10.1530/JOE-12-0385. 23111281 PMC4077722

[38] Usami M, Kishimoto K, Ohata A, Miyoshi M, Aoyama M, Fueda Y, Kotani J. Butyrate and trichostatin A attenuate nuclear factor kappaB activation and tumor necrosis factor alpha secretion and increase prostaglandin E2 secretion in human peripheral blood mononuclear cells. Nutr Res. 2008;28:321–328. doi:10.1016/j.nutres.2008.02.012. 19083427

[39] Vinolo MA, Rodrigues HG, Hatanaka E, Sato FT, Sampaio SC, Curi R. Suppressive effect of short-chain fatty acids on production of proinflammatory mediators by neutrophils. J Nutr Biochem. 2011;22:849–855. doi:10.1016/j.jnutbio.2010.07.009. 21167700

[40] Haase S, Haghikia A, Wilck N, Muller DN, Linker RA. Impacts of microbiome metabolites on immune regulation and autoimmunity. Immunology. 2018;154:230–238. doi:10.1111/imm.12933. 29637999 PMC5980218

[41] Smith PM, Howitt MR, Panikov N, Michaud M, Gallini CA, Bohlooly YM, Glickman JN, Garrett WS. The microbial metabolites, short-chain fatty acids, regulate colonic T reg cell homeostasis. Science. 2013;341:569–573. doi:10.1126/science.1241165. 23828891 PMC3807819

[42] Priyadarshini M, Navarro G, Layden BT. Gut Microbiota: FFAR Reaching Effects on Islets. Endocrinology. 2018;159:2495–2505. doi:10.1210/en.2018-00296. 29846565 PMC6692871

[43] Kinross JM, Darzi AW, Nicholson JK. Gut microbiome-host interactions in health and disease. Genome Med. 2011;3:14. doi:10.1186/gm228. 21392406 PMC3092099

[44] Zhou H, Sun L, Zhang S, Zhao X, Gang X, Wang G. Evaluating the causal role of gut microbiota in type 1 diabetes and its possible pathogenic mechanisms. Front Endocrinol (Lausanne). 2020;11:125. doi:10.3389/fendo.2020.00125. 32265832 PMC7105744

[45] Hansen R, Thomson JM, Fox JG, El-Omar EM, Hold GL. Could Helicobacter organisms cause inflammatory bowel disease? FEMS Immunol Med Microbiol. 2011;61:1–14. doi:10.1111/j.1574-695X.2010.00744.x. 20955468

[46] Binda C, Lopetuso LR, Rizzatti G, Gibiino G, Cennamo V, Gasbarrini A. Actinobacteria: a relevant minority for the maintenance of gut homeostasis. Dig Liver Dis. 2018;50:421–428. doi:10.1016/j.dld.2018.02.012. 29567414

[47] LeBlanc JG, Chain F, Martin R, Bermudez-Humaran LG, Courau S, Langella P. Beneficial effects on host energy metabolism of short-chain fatty acids and vitamins produced by commensal and probiotic bacteria. Microb Cell Fact. 2017;16:79. doi:10.1186/s12934-017-0691-z. 28482838 PMC5423028

[48] Jwa M, Chang P. PARP16 is a tail-anchored endoplasmic reticulum protein required for the PERK- and IRE1alpha-mediated unfolded protein response. Nat Cell Biol. 2012;14:1223–1230. doi:10.1038/ncb2593. 23103912 PMC3494284

[49] Liu J, Wang Y, Song L, Zeng L, Yi W, Liu T, Chen H, Wang M, Ju Z, Cong Y-S, et al. A critical role of DDRGK1 in endoplasmic reticulum homoeostasis via regulation of IRE1alpha stability. Nat Commun. 2017;8:14186. doi:10.1038/ncomms14186. 28128204 PMC5290148

[50] Gkogkas C, Middleton S, Kremer AM, Wardrope C, Hannah M, Gillingwater TH. VAPB interacts with and modulates the activity of ATF6. Hum Mol Genet. 2008;17:1517–1526. doi:10.1093/hmg/ddn040. 18263603

[51] Chen M, Gutierrez GJ, Ronai ZA. Ubiquitin-recognition protein Ufd1 couples the endoplasmic reticulum (ER) stress response to cell cycle control. Proc Natl Acad Sci U S A. 2011;108:9119–9124. doi:10.1073/pnas.1100028108. 21571647 PMC3107271

[52] Jo S, Lockridge A, Mohan R, Esch N, Schlichting R, Panigrahy N, Essawy A, Gustafson E, Alejandro EU. Translational factor eIF4G1 regulates glucose homeostasis and pancreatic beta-cell function. Diabetes. 2021;70:155–170. doi:10.2337/db20-0057. 33115825 PMC7881850

[53] Csibi A, Tintignac LA, Leibovitch MP, Leibovitch SA. eIF3-f function in skeletal muscles: to stand at the crossroads of atrophy and hypertrophy. Cell Cycle. 2008;7:1698–1701. doi:10.4161/cc.7.12.6090. 18583931

[54] Tao J, Sha B. Structural insight into the protective role of P58(IPK) during unfolded protein response. Methods Enzymol. 2011;490:259–270. 21266255 10.1016/B978-0-12-385114-7.00015-5

[55] Lu K, Psakhye I, Jentsch S. Autophagic clearance of polyQ proteins mediated by ubiquitin-Atg8 adaptors of the conserved CUET protein family. Cell. 2014;158:549–563. doi:10.1016/j.cell.2014.05.048. 25042851

[56] Eizirik DL, Miani M, Cardozo AK. Signalling danger: endoplasmic reticulum stress and the unfolded protein response in pancreatic islet inflammation. Diabetologia. 2013;56:234–241. doi:10.1007/s00125-012-2762-3. 23132339

[57] van Baarlen P, Troost FJ, van Hemert S, van der Meer C, de Vos WM, de Groot PJ. Differential NF-kappaB pathways induction by Lactobacillus plantarum in the duodenum of healthy humans correlating with immune tolerance. Proceedings of the National Academy of Sciences of the United States of America. 2009;106:2371–2376. 10.1073/pnas.0809919106 PMC2650163 19190178

[58] Naruszewicz M, Johansson ML, Zapolska-Downar D, Bukowska H. Effect of lactobacillus plantarum 299v on cardiovascular disease risk factors in smokers. Am J Clin Nutr. 2002;76:1249–1255. doi:10.1093/ajcn/76.6.1249. 12450890

[59] Karczewski J, Troost FJ, Konings I, Dekker J, Kleerebezem M, Brummer RJ, Wells JM. Regulation of human epithelial tight junction proteins by Lactobacillus plantarum in vivo and protective effects on the epithelial barrier. Am J Physiol Gastrointest Liver Physiol. 2010;298:G851–9. doi:10.1152/ajpgi.00327.2009. 20224007

[60] Wang G, Si Q, Yang S, Jiao T, Zhu H, Tian P. Lactic acid bacteria reduce diabetes symptoms in mice by alleviating gut microbiota dysbiosis and inflammation in different manners. Food Funct. 2020;11:5898–5914. doi:10.1039/C9FO02761K. 32572400

[61] Calcinaro F, Dionisi S, Marinaro M, Candeloro P, Bonato V, Marzotti S, Corneli RB, Ferretti E, Gulino A, Grasso F, et al. Oral probiotic administration induces interleukin-10 production and prevents spontaneous autoimmune diabetes in the non-obese diabetic mouse. Diabetologia. 2005;48:1565–1575. doi:10.1007/s00125-005-1831-2. 15986236

[62] Hofeld BC, Puppala VK, Tyagi S, Ahn KW, Anger A, Jia S. Lactobacillus plantarum 299v probiotic supplementation in men with stable coronary artery disease suppresses systemic inflammation. Sci Rep. 2021;11:3972. doi:10.1038/s41598-021-83252-7. 33597583 PMC7889883

[63] Karlsson C, Ahrne S, Molin G, Berggren A, Palmquist I, Fredrikson GN. Probiotic therapy to men with incipient arteriosclerosis initiates increased bacterial diversity in colon: a randomized controlled trial. Atherosclerosis. 2010;208:228–233. 19608185 10.1016/j.atherosclerosis.2009.06.019

[64] Roep BO, Kleijwegt FS, van Halteren AG, Bonato V, Boggi U, Vendrame F, Marchetti P, Dotta F. Islet inflammation and CXCL10 in recent-onset type 1 diabetes. Clin Exp Immunol. 2010;159:338–343. doi:10.1111/j.1365-2249.2009.04087.x. 20059481 PMC2819499

[65] Planas R, Carrillo J, Sanchez A, de Villa MC, Nunez F, Verdaguer J, James RFL, Pujol-Borrell R, Vives-Pi M. Gene expression profiles for the human pancreas and purified islets in type 1 diabetes: new findings at clinical onset and in long-standing diabetes. Clin Exp Immunol. 2010;159:23–44. doi:10.1111/j.1365-2249.2009.04053.x. 19912253 PMC2802692

[66] Guo H, Xiong Y, Witkowski P, Cui J, Wang LJ, Sun J, Lara-Lemus R, Haataja L, Hutchison K, Shan S-O, et al. Inefficient translocation of preproinsulin contributes to pancreatic beta cell failure and late-onset diabetes. J Biol Chem. 2014;289:16290–16302. doi:10.1074/jbc.M114.562355. 24770419 PMC4047398

[67] Danobeitia JS, Chlebeck PJ, Shokolenko I, Ma X, Wilson G, Fernandez LA. Novel fusion protein targeting mitochondrial DNA improves pancreatic islet functional potency and islet transplantation outcomes. Cell Transplant. 2017;26:1742–1754. doi:10.1177/0963689717727542. 29338388 PMC5784523

[68] Modak MA, Datar SP, Bhonde RR, Ghaskadbi SS. Differential susceptibility of chick and mouse islets to streptozotocin and its co-relation with islet antioxidant status. J Comp Physiol B. 2007;177:247–257. doi:10.1007/s00360-006-0126-3. 17205303

[69] Hu S, Kuwabara R, de Haan BJ, Smink AM, de Vos P. Acetate and butyrate improve β-cell metabolism and mitochondrial respiration under oxidative stress. Int J Mol Sci. 2020;21:1542. doi:10.3390/ijms21041542. PMC7073211 32102422

[70] Pingitore A, Chambers ES, Hill T, Maldonado IR, Liu B, Bewick G, Morrison DJ, Preston T, Wallis GA, Tedford C, et al. The diet-derived short chain fatty acid propionate improves beta-cell function in humans and stimulates insulin secretion from human islets in vitro. Diabetes Obes Metab. 2017;19:257–265. doi:10.1111/dom.12811. 27761989

[71] Villa SR, Priyadarshini M, Fuller MH, Bhardwaj T, Brodsky MR, Angueira AR. Loss of free fatty acid receptor 2 leads to impaired islet mass and beta cell survival. Sci Rep. 2016;6:28159. doi:10.1038/srep28159. 27324831 PMC4914960

[72] Daulatzai MA. Non-celiac gluten sensitivity triggers gut dysbiosis, neuroinflammation, gut-brain axis dysfunction, and vulnerability for dementia. CNS Neurol Disord Drug Targets. 2015;14:110–131. doi:10.2174/1871527314666150202152436. 25642988

[73] Hu Y, Gao Y, Zhang M, Deng KY, Singh R, Tian Q, Gong Y, Pan Z, Liu Q, Boisclair YR, et al. Endoplasmic Reticulum-Associated Degradation (ERAD) Has a Critical Role in Supporting Glucose-Stimulated Insulin Secretion in Pancreatic beta-Cells. Diabetes. 2019;68:733–746. doi:10.2337/db18-0624. 30626610

[74] Gonzalez-Bosch C, Boorman E, Zunszain PA, Mann GE. Short-chain fatty acids as modulators of redox signaling in health and disease. Redox Biol. 2021;47:102165. doi:10.1016/j.redox.2021.102165. 34662811 PMC8577496

[75] Kumar A, Katz LS, Schulz AM, Kim M, Honig LB, Li L, Davenport B, Homann D, Garcia-Ocaña A, Herman MA, et al. Activation of Nrf2 is required for normal and ChREBPalpha-Augmented glucose-Stimulated beta-Cell proliferation. Diabetes. 2018;67:1561–1575. doi:10.2337/db17-0943. 29764859 PMC6054434

[76] Uruno A, Furusawa Y, Yagishita Y, Fukutomi T, Muramatsu H, Negishi T, Sugawara A, Kensler TW, Yamamoto M. The Keap1-Nrf2 system prevents onset of diabetes mellitus. Mol Cell Biol. 2013;33(15):2996–3010. doi:10.1128/MCB.00225-13. 23716596 PMC3719683

[77] Baumel-Alterzon S, Katz LS, Brill G, Jean-Pierre C, Li Y, Tse I, Biswal S, Garcia-Ocaña A, Scott DK. Nrf2 regulates beta-Cell mass by suppressing beta-Cell death and promoting beta-Cell proliferation. Diabetes. 2022;71:989–1011. doi:10.2337/db21-0581. 35192689 PMC9044139

