# Supplementary material for: Lactiplantibacillus plantarum 299v supplementation modulates β-cell ER stress and antioxidative defense pathways and prevents type 1 diabetes in gluten-free BioBreeding rats
Source: Gut Microbes. 2022 Oct 19;14(1):2136467. doi: 10.1080/19490976.2022.2136467 (PMC9586621; doi:10.1080/19490976.2022.2136467)
Supplement: Supplemental Material [file KGMI_A_2136467_SM6651.zip › Supplemental_Figures_Revised_10_7_22.pptx]

## Slide 1
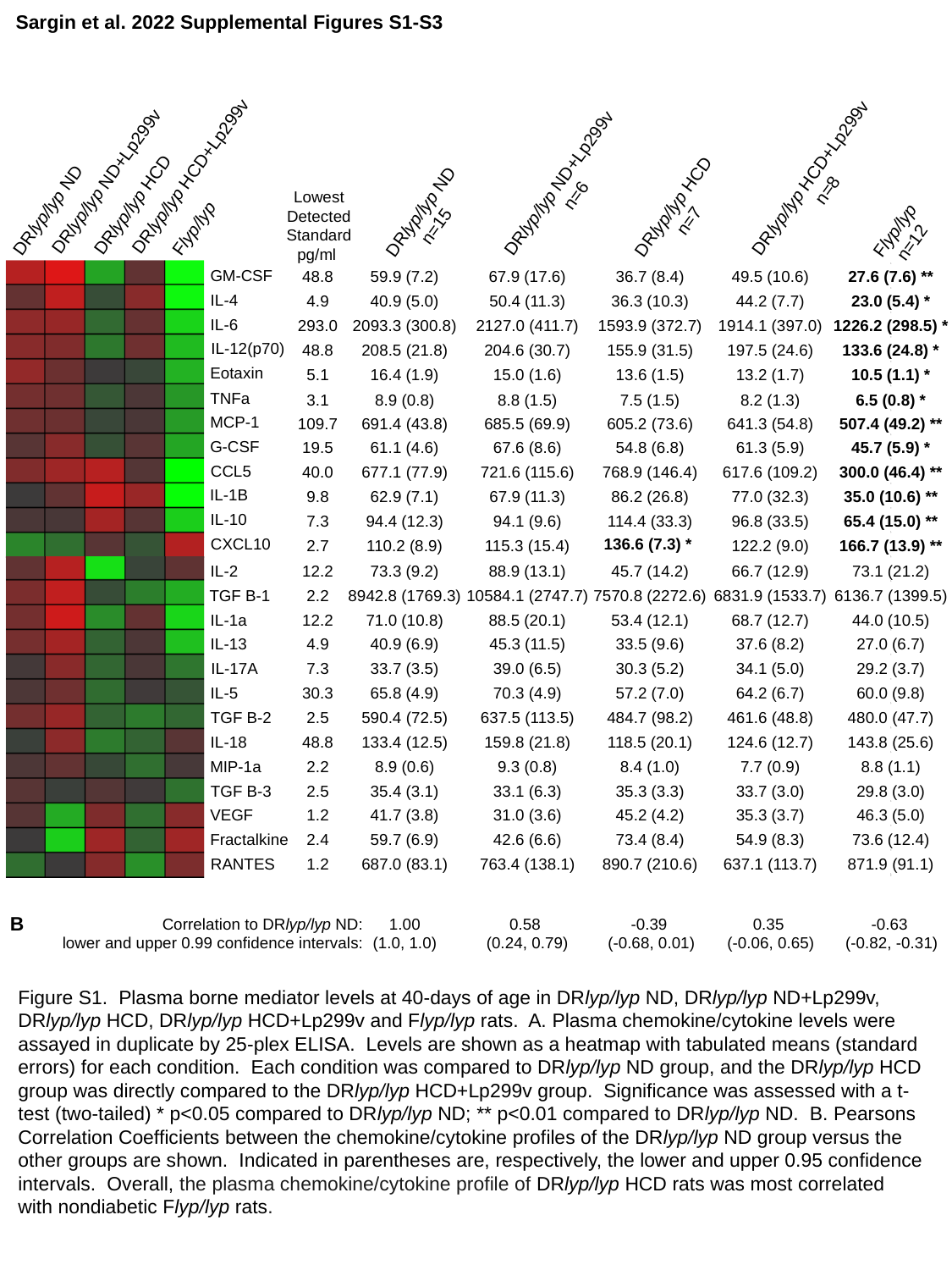

Sargin et al. 2022 Supplemental Figures S1-S3
DRlyp/lyp HCD+Lp299v
n=8
DRlyp/lyp HCD+Lp299v
DRlyp/lyp ND+Lp299v
n=6
DRlyp/lyp ND+Lp299v
Lowest
Detected
Standard
pg/ml
DRlyp/lyp HCD
n=7
DRlyp/lyp HCD
DRlyp/lyp ND
n=15
DRlyp/lyp ND
Flyp/lyp
n=12
Flyp/lyp
27.6 (7.6) **
23.0 (5.4) *
1226.2 (298.5) *
133.6 (24.8) *
10.5 (1.1) *
6.5 (0.8) *
507.4 (49.2) **
45.7 (5.9) *
300.0 (46.4) **
35.0 (10.6) **
65.4 (15.0) **
166.7 (13.9) **
73.1 (21.2)
6136.7 (1399.5)
44.0 (10.5)
27.0 (6.7)
29.2 (3.7)
60.0 (9.8)
480.0 (47.7)
143.8 (25.6)
8.8 (1.1)
29.8 (3.0)
46.3 (5.0)
73.6 (12.4)
871.9 (91.1)
GM-CSF
IL-4
IL-6
IL-12(p70)
Eotaxin
TNFa
MCP-1
G-CSF
CCL5
IL-1B
IL-10
CXCL10
IL-2
TGF B-1
IL-1a
IL-13
IL-17A
IL-5
TGF B-2
IL-18
MIP-1a
TGF B-3
VEGF
Fractalkine
RANTES
48.8
59.9 (7.2)
40.9 (5.0)
2093.3 (300.8)
208.5 (21.8)
16.4 (1.9)
8.9 (0.8)
691.4 (43.8)
61.1 (4.6)
677.1 (77.9)
62.9 (7.1)
94.4 (12.3)
110.2 (8.9)
73.3 (9.2)
8942.8 (1769.3)
71.0 (10.8)
40.9 (6.9)
33.7 (3.5)
65.8 (4.9)
590.4 (72.5)
133.4 (12.5)
8.9 (0.6)
35.4 (3.1)
41.7 (3.8)
59.7 (6.9)
687.0 (83.1)
67.9 (17.6)
50.4 (11.3)
2127.0 (411.7)
204.6 (30.7)
15.0 (1.6)
8.8 (1.5)
685.5 (69.9)
67.6 (8.6)
721.6 (115.6)
67.9 (11.3)
94.1 (9.6)
115.3 (15.4)
88.9 (13.1)
10584.1 (2747.7)
88.5 (20.1)
45.3 (11.5)
39.0 (6.5)
70.3 (4.9)
637.5 (113.5)
159.8 (21.8)
9.3 (0.8)
33.1 (6.3)
31.0 (3.6)
42.6 (6.6)
763.4 (138.1)
36.7 (8.4)
36.3 (10.3)
1593.9 (372.7)
155.9 (31.5)
13.6 (1.5)
7.5 (1.5)
605.2 (73.6)
54.8 (6.8)
768.9 (146.4)
86.2 (26.8)
114.4 (33.3)
136.6 (7.3) *
45.7 (14.2)
7570.8 (2272.6)
53.4 (12.1)
33.5 (9.6)
30.3 (5.2)
57.2 (7.0)
484.7 (98.2)
118.5 (20.1)
8.4 (1.0)
35.3 (3.3)
45.2 (4.2)
73.4 (8.4)
890.7 (210.6)
49.5 (10.6)
44.2 (7.7)
1914.1 (397.0)
197.5 (24.6)
13.2 (1.7)
8.2 (1.3)
641.3 (54.8)
61.3 (5.9)
617.6 (109.2)
77.0 (32.3)
96.8 (33.5)
122.2 (9.0)
66.7 (12.9)
6831.9 (1533.7)
68.7 (12.7)
37.6 (8.2)
34.1 (5.0)
64.2 (6.7)
461.6 (48.8)
124.6 (12.7)
7.7 (0.9)
33.7 (3.0)
35.3 (3.7)
54.9 (8.3)
637.1 (113.7)
4.9
293.0
48.8
5.1
3.1
109.7
19.5
40.0
9.8
7.3
2.7
12.2
2.2
12.2
4.9
7.3
30.3
2.5
48.8
2.2
2.5
1.2
2.4
1.2
B
Correlation to DRlyp/lyp ND:
lower and upper 0.99 confidence intervals:
1.00
(1.0, 1.0)
0.58
(0.24, 0.79)
-0.39
(-0.68, 0.01)
0.35
(-0.06, 0.65)
-0.63
(-0.82, -0.31)
Figure S1. Plasma borne mediator levels at 40-days of age in DRlyp/lyp ND, DRlyp/lyp ND+Lp299v, DRlyp/lyp HCD, DRlyp/lyp HCD+Lp299v and Flyp/lyp rats. A. Plasma chemokine/cytokine levels were assayed in duplicate by 25-plex ELISA. Levels are shown as a heatmap with tabulated means (standard errors) for each condition. Each condition was compared to DRlyp/lyp ND group, and the DRlyp/lyp HCD group was directly compared to the DRlyp/lyp HCD+Lp299v group. Significance was assessed with a t-test (two-tailed) * p<0.05 compared to DRlyp/lyp ND; ** p<0.01 compared to DRlyp/lyp ND. B. Pearsons Correlation Coefficients between the chemokine/cytokine profiles of the DRlyp/lyp ND group versus the other groups are shown. Indicated in parentheses are, respectively, the lower and upper 0.95 confidence intervals. Overall, the plasma chemokine/cytokine profile of DRlyp/lyp HCD rats was most correlated with nondiabetic Flyp/lyp rats.

## Slide 2
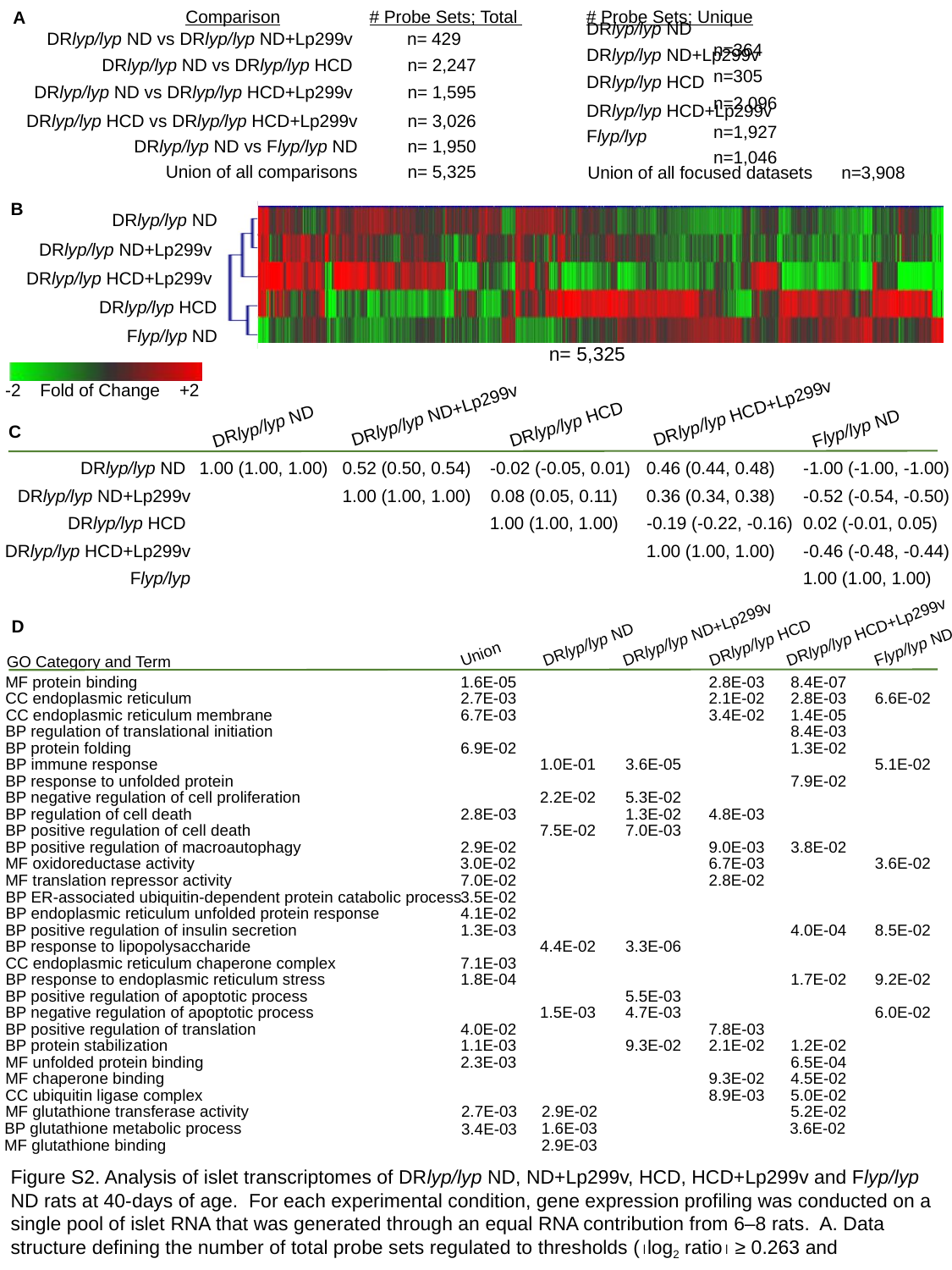

Comparison
# Probe Sets; Total
# Probe Sets; Unique
A
DRlyp/lyp ND vs DRlyp/lyp ND+Lp299v
n= 429
DRlyp/lyp ND			n=364
DRlyp/lyp ND vs DRlyp/lyp HCD
n= 2,247
DRlyp/lyp ND+Lp299v		n=305
DRlyp/lyp ND vs DRlyp/lyp HCD+Lp299v
n= 1,595
DRlyp/lyp HCD			n=2,096
DRlyp/lyp HCD vs DRlyp/lyp HCD+Lp299v
n= 3,026
DRlyp/lyp HCD+Lp299v		n=1,927
DRlyp/lyp ND vs Flyp/lyp ND
n= 1,950
Flyp/lyp 			n=1,046
Union of all comparisons
n= 5,325
Union of all focused datasets	n=3,908
B
DRlyp/lyp ND
DRlyp/lyp ND+Lp299v
DRlyp/lyp HCD+Lp299v
DRlyp/lyp HCD
Flyp/lyp ND
n= 5,325
-2 Fold of Change +2
DRlyp/lyp HCD+Lp299v
DRlyp/lyp ND+Lp299v
DRlyp/lyp HCD
DRlyp/lyp ND
Flyp/lyp ND
C
DRlyp/lyp ND
1.00 (1.00, 1.00)
0.52 (0.50, 0.54)
1.00 (1.00, 1.00)
-0.02 (-0.05, 0.01)
0.08 (0.05, 0.11)
1.00 (1.00, 1.00)
0.46 (0.44, 0.48)
0.36 (0.34, 0.38)
-0.19 (-0.22, -0.16)
1.00 (1.00, 1.00)
-1.00 (-1.00, -1.00)
-0.52 (-0.54, -0.50)
0.02 (-0.01, 0.05)
-0.46 (-0.48, -0.44)
1.00 (1.00, 1.00)
DRlyp/lyp ND+Lp299v
DRlyp/lyp HCD
DRlyp/lyp HCD+Lp299v
Flyp/lyp
D
DRlyp/lyp HCD+Lp299v
DRlyp/lyp ND+Lp299v
DRlyp/lyp HCD
Union
DRlyp/lyp ND
Flyp/lyp ND
GO Category and Term
MF protein binding
1.6E-05
2.8E-03
8.4E-07
CC endoplasmic reticulum
2.7E-03
2.1E-02
2.8E-03
6.6E-02
CC endoplasmic reticulum membrane
6.7E-03
3.4E-02
1.4E-05
BP regulation of translational initiation
8.4E-03
BP protein folding
6.9E-02
1.3E-02
BP immune response
1.0E-01
3.6E-05
5.1E-02
BP response to unfolded protein
7.9E-02
BP negative regulation of cell proliferation
2.2E-02
5.3E-02
BP regulation of cell death
2.8E-03
1.3E-02
4.8E-03
BP positive regulation of cell death
7.5E-02
7.0E-03
BP positive regulation of macroautophagy
2.9E-02
9.0E-03
3.8E-02
3.0E-02
MF oxidoreductase activity
6.7E-03
3.6E-02
MF translation repressor activity
7.0E-02
2.8E-02
BP ER-associated ubiquitin-dependent protein catabolic process
3.5E-02
BP endoplasmic reticulum unfolded protein response
4.1E-02
BP positive regulation of insulin secretion
1.3E-03
4.0E-04
8.5E-02
BP response to lipopolysaccharide
4.4E-02
3.3E-06
CC endoplasmic reticulum chaperone complex
7.1E-03
BP response to endoplasmic reticulum stress
1.8E-04
1.7E-02
9.2E-02
BP positive regulation of apoptotic process
5.5E-03
BP negative regulation of apoptotic process
1.5E-03
4.7E-03
6.0E-02
BP positive regulation of translation
4.0E-02
7.8E-03
BP protein stabilization
1.1E-03
9.3E-02
2.1E-02
1.2E-02
MF unfolded protein binding
2.3E-03
6.5E-04
MF chaperone binding
9.3E-02
4.5E-02
CC ubiquitin ligase complex
8.9E-03
5.0E-02
MF glutathione transferase activity
2.7E-03
2.9E-02
5.2E-02
BP glutathione metabolic process
1.6E-03
3.6E-02
3.4E-03
MF glutathione binding
2.9E-03
Figure S2. Analysis of islet transcriptomes of DRlyp/lyp ND, ND+Lp299v, HCD, HCD+Lp299v and Flyp/lyp ND rats at 40-days of age. For each experimental condition, gene expression profiling was conducted on a single pool of islet RNA that was generated through an equal RNA contribution from 6–8 rats. A. Data structure defining the number of total probe sets regulated to thresholds (log2 ratio ≥ 0.263 and

## Slide 3
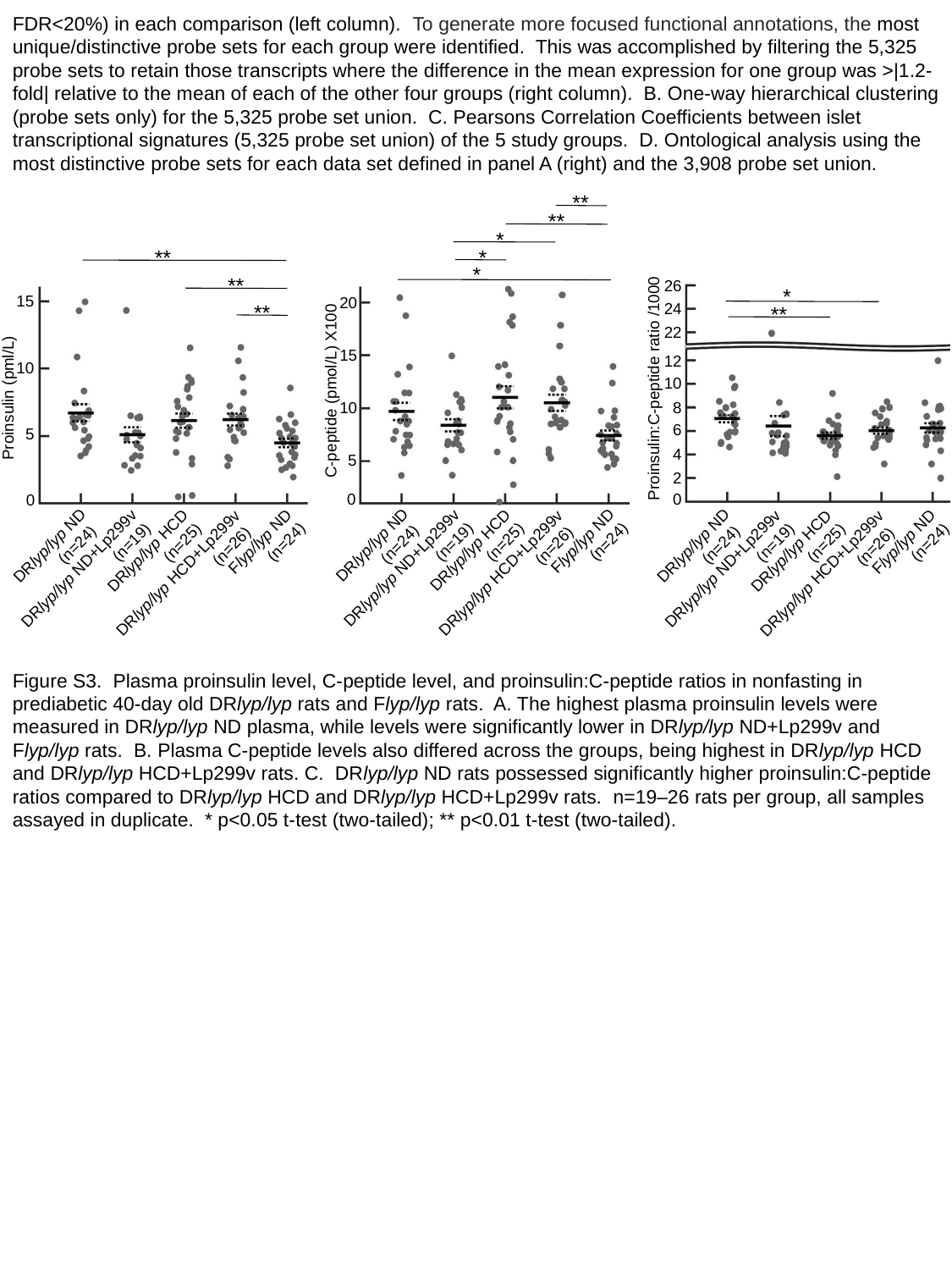

FDR<20%) in each comparison (left column). To generate more focused functional annotations, the most unique/distinctive probe sets for each group were identified. This was accomplished by filtering the 5,325 probe sets to retain those transcripts where the difference in the mean expression for one group was >|1.2-fold| relative to the mean of each of the other four groups (right column). B. One-way hierarchical clustering (probe sets only) for the 5,325 probe set union. C. Pearsons Correlation Coefficients between islet transcriptional signatures (5,325 probe set union) of the 5 study groups. D. Ontological analysis using the most distinctive probe sets for each data set defined in panel A (right) and the 3,908 probe set union.
**
**
*
*
**
*
**
26
*
20
15
C-peptide (pmol/L) X100
10
5
0
15
10
5
0
24
**
**
22
12
10
Proinsulin:C-peptide ratio /1000
Proinsulin (pml/L)
8
6
4
2
Flyp/lyp ND
(n=24)
DRlyp/lyp ND
 (n=24)
DRlyp/lyp HCD
 (n=25)
DRlyp/lyp ND+Lp299v
 (n=19)
DRlyp/lyp HCD+Lp299v
 (n=26)
Flyp/lyp ND
(n=24)
DRlyp/lyp ND
 (n=24)
DRlyp/lyp HCD
 (n=25)
DRlyp/lyp ND+Lp299v
 (n=19)
DRlyp/lyp HCD+Lp299v
 (n=26)
0
Flyp/lyp ND
(n=24)
DRlyp/lyp ND
 (n=24)
DRlyp/lyp HCD
 (n=25)
DRlyp/lyp ND+Lp299v
 (n=19)
DRlyp/lyp HCD+Lp299v
 (n=26)
Figure S3. Plasma proinsulin level, C-peptide level, and proinsulin:C-peptide ratios in nonfasting in prediabetic 40-day old DRlyp/lyp rats and Flyp/lyp rats. A. The highest plasma proinsulin levels were measured in DRlyp/lyp ND plasma, while levels were significantly lower in DRlyp/lyp ND+Lp299v and Flyp/lyp rats. B. Plasma C-peptide levels also differed across the groups, being highest in DRlyp/lyp HCD and DRlyp/lyp HCD+Lp299v rats. C. DRlyp/lyp ND rats possessed significantly higher proinsulin:C-peptide ratios compared to DRlyp/lyp HCD and DRlyp/lyp HCD+Lp299v rats. n=19–26 rats per group, all samples assayed in duplicate. * p<0.05 t-test (two-tailed); ** p<0.01 t-test (two-tailed).
