# Supplementary material for: Lactiplantibacillus plantarum 299v supplementation modulates β-cell ER stress and antioxidative defense pathways and prevents type 1 diabetes in gluten-free BioBreeding rats
Source: Gut Microbes. 2022 Oct 19;14(1):2136467. doi: 10.1080/19490976.2022.2136467 (PMC9586621; doi:10.1080/19490976.2022.2136467)
Supplement: Supplemental Material [file KGMI_A_2136467_SM6651.zip › Table S1.pdf]

Probeset ID	Gene Symbol	Islet Data Set (log2 ratio ≥0.263; FDR <20%) union n=5325 1=YES 0=NO	Islet DRlyp/lyp ND vs ND+Lp299v (log2 ratio ≥0.263; FDR<20%) n=429 1=YES 0=NO	Islet DRlyp/lyp ND vs HCD (log2 ratio ≥0.263; FDR<20%) n=2247 1=YES 0=NO	Islet DRlyp/lyp ND vs HCD+Lp299v (log2 ratio ≥0.263; FDR<20%) n=1595 1=YES 0=NO	Islet DRlyp/lyp HCD vs HCD+Lp299v (log2 ratio ≥0.263; FDR<20%) n=3026 1=YES 0=NO	Islet DRlyp/lyp ND vs Flyp/lyp ND (log2 ratio ≥ 0.263; FDR<20%) n=1950 1=YES 0=NO	Islet Mean Log2 Intensity DRlyp/lyp ND	Islet Mean Log2 Intensity DRlyp/lyp ND+Lp299v	Islet Mean Log2 Intensity DRlyp/lyp HCD	Islet Mean Log2 Intensity DRlyp/lyp HCD+Lp299v	Islet Mean Log2 Intensity Flyp40 ND	Rinm5f data union of all conditions n=3705 1=YES 0=NO	Rinm5f 30 µM propionate/ 6 µM butyrate vs Media (FDR<20%; 1.5 fold) n=3 1=YES 0=NO	Rinm5f 60 µM propionate/ 12 µM butyrate vs Media (FDR<20%; 1.5 fold) n=18 1=YES 0=NO	Rinm5f 120 µM propionate/ 24 µM butyrate vs Media (FDR<20%; 1.5 fold) n=164 1=YES 0=NO	Rinm5f 240 µM propionate /48 µM butyrate vs Media (FDR<20%; 1.5 fold) n=268 1=YES 0=NO	Rinm5f 1500 µM propionate/ 300 µM butyrate vs Media (FDR<20%; 1.5 fold) n=2491 1=YES 0=NO	Rinm5f 500 µM propionate/ 500 µM butyrate vs Media (FDR<20%; 1.5 fold) n=3001 1=YES 0=NO	Rinm5f Mean Log2 Intensity Media only	Rinm5f Mean Log2 Intensity 30 µM propionate/ 6 µM butyrate	Rinm5f Mean Log2 Intensity 60 µM propionate/ 12 µM butyrate	Rinm5f Mean Log2 Intensity 120 µM propionate/ 24 µM butyrate	Rinm5f Mean Log2 Intensity 240 µM propionate/ 48 µM butyrate	Rinm5f Mean Log2 Intensity 1500 µM propionate /300 µM butyrate	Rinm5f Mean Log2 Intensity 500 µM propionate/ 500 µM butyrate
1367577_at	Hspb1	1	0	1	1	0	1	8.24	8.66	7.51	7.72	7.42	1	0	0	0	0	1	1	5.82	5.89	6.12	6.11	6.24	7.30	8.07
1367595_s_at	B2m	1	0	0	1	1	1	10.42	10.85	10.70	9.81	11.14	1	0	0	0	0	1	0	11.50	11.52	11.50	11.60	11.72	12.23	11.95
1367601_at	Cited2	1	0	0	0	0	1	8.97	9.01	8.57	9.06	8.33	1	0	0	0	0	1	1	11.26	11.17	11.09	10.68	10.71	9.89	9.91
1367624_at	Atf4	1	0	0	1	0	0	11.12	10.91	11.00	10.61	10.86	1	0	0	0	0	0	1	12.62	12.64	12.68	12.73	12.75	12.57	11.67
1367628_at	Lgals1	1	0	1	0	1	0	7.14	7.17	6.20	6.91	6.82	1	0	0	0	0	1	1	5.75	5.77	5.90	6.12	6.13	6.90	6.80
1367632_at	Glul	1	1	0	1	0	0	9.09	8.49	9.23	8.54	9.22	1	0	0	0	0	1	1	8.69	8.75	8.97	9.14	9.09	9.45	9.46
1367633_at	Glul	1	1	0	1	1	0	8.57	7.97	8.85	7.22	8.18	1	0	0	0	0	1	0	8.17	8.21	8.44	8.54	8.42	8.79	8.45
1367652_at	Igfbp3	1	0	0	1	1	0	7.98	7.66	8.16	7.38	8.29	1	0	0	0	0	0	1	9.03	8.98	8.94	8.92	8.90	9.52	9.52
1367661_at	S100a6	1	0	1	1	0	0	7.15	6.93	6.62	6.42	6.92	1	0	0	0	0	0	1	3.94	3.88	3.86	3.85	3.91	4.23	5.13
1367673_at	LOC103689947	1	0	1	0	1	1	8.86	8.56	8.04	8.85	7.89	1	0	0	0	0	0	1	4.16	4.23	4.23	4.10	4.27	4.42	5.78
1367676_at	Hmgb2	1	0	1	0	0	1	7.67	7.62	8.25	7.92	8.18	1	0	0	0	0	0	1	12.63	12.65	12.63	12.64	12.59	12.12	11.70
1367679_at	Cd74	1	1	0	0	0	1	7.75	8.46	7.47	7.49	8.70	1	0	0	0	0	0	1	4.24	4.15	4.25	4.00	4.14	4.59	5.04
1367689_a_at	Cd36 /// LOC100	1	1	0	1	1	1	6.97	6.36	6.87	5.20	5.79	1	0	0	0	0	0	1	3.46	3.43	3.49	3.37	3.36	3.87	3.82
1367698_a_at	Septin9	1	0	0	0	1	0	6.77	6.66	6.45	7.10	7.03	1	0	0	0	0	1	1	10.46	10.44	10.43	10.16	10.25	9.67	9.32
1367701_at	Ramp2	1	0	0	1	0	0	7.88	7.84	8.02	7.38	7.93	1	0	0	0	0	0	1	9.72	9.84	9.91	10.05	10.06	9.50	8.70
1367705_at	Glrx	1	0	1	0	1	1	6.90	6.87	7.50	6.55	7.85	1	0	0	1	1	1	1	8.41	8.48	8.72	9.00	9.12	10.04	9.59
1367707_at	Fasn	1	0	0	0	1	0	6.35	6.38	6.09	6.69	6.49	1	0	0	0	0	1	1	7.97	7.88	8.05	8.08	7.96	7.37	7.38
1367743_at	Pfkl	1	0	0	0	0	1	4.17	4.30	4.22	4.49	4.72	1	0	0	0	0	1	1	8.55	8.64	8.52	8.25	8.23	7.70	7.52
1367759_at	H1f0	1	0	0	1	1	0	9.52	9.36	9.25	10.15	9.52	1	0	0	0	0	1	1	8.89	9.01	9.05	9.17	9.30	9.94	9.97
1367765_at	Tcn2	1	0	0	0	1	0	11.16	11.03	10.77	11.40	10.72	1	0	0	0	0	1	0	10.49	10.61	10.72	10.84	11.02	11.21	10.82
1367776_at	Cdk1	1	0	0	0	0	1	6.53	6.32	7.06	6.85	7.04	1	0	0	0	0	1	1	11.31	11.25	11.30	11.27	11.20	10.65	10.10
1367777_at	Decr1	1	1	0	0	0	1	8.79	8.08	9.34	8.81	7.97	1	0	0	0	0	1	0	9.61	9.68	9.73	9.84	9.89	10.27	10.14
1367786_at	Psmb8	1	1	0	0	0	0	5.82	6.77	5.55	5.36	6.02	1	0	0	0	0	1	0	7.77	7.90	7.90	8.06	8.00	8.43	8.17
1367789_at	Slc27a1	1	0	0	0	0	1	7.43	7.05	7.06	7.18	6.78	1	0	0	0	0	1	1	7.87	7.83	7.90	7.86	7.99	8.52	8.21
1367800_at	Plat	1	0	0	1	1	0	5.54	5.33	5.82	4.97	5.37	1	0	0	1	1	1	1	9.17	9.43	9.52	9.84	10.18	10.78	10.12
1367802_at	Sgk1	1	1	0	1	0	0	8.01	7.53	8.07	7.46	7.71	1	0	0	0	0	0	1	7.95	7.81	8.06	8.34	8.15	8.52	8.93
1367816_at	Hopx	1	0	0	1	0	0	6.26	6.04	6.31	5.70	5.99	1	0	0	0	0	1	1	8.78	8.90	8.85	8.97	9.11	9.88	9.53
1367836_at	Cpt1a	1	1	0	1	1	1	6.34	5.74	6.36	5.58	5.58	1	0	0	1	1	1	1	7.73	7.64	7.96	8.39	8.32	8.94	8.21
1367838_at	Cth	1	1	1	1	0	1	10.91	10.07	9.34	9.86	9.62	1	0	0	0	0	0	1	4.02	3.94	4.03	4.00	4.11	4.49	4.38
1367847_at	Nupr1	1	1	1	1	1	0	11.40	10.69	9.60	10.77	11.18	1	0	0	0	0	0	1	11.51	11.57	11.56	11.55	11.67	11.03	9.92
1367905_at	Enpp3	1	0	0	1	1	1	8.36	8.14	8.36	9.06	7.87	1	0	0	0	0	1	1	5.70	5.88	6.06	5.84	6.03	6.54	6.87
1367909_at	Dcxr	1	0	1	0	1	1	8.05	7.97	7.21	8.13	7.49	1	0	0	0	0	1	1	6.48	6.48	6.44	6.18	6.32	7.17	7.21
1367919_at	Nup210	1	0	0	1	0	0	7.44	7.45	7.48	7.92	7.20	1	0	0	0	0	1	0	9.81	9.74	9.76	9.64	9.56	9.20	9.38
1367929_at	Cd59	1	0	0	0	0	1	9.68	9.52	9.27	9.39	9.07	1	0	0	0	0	1	1	8.95	9.06	9.19	9.24	9.39	10.02	9.89
1367930_at	Gap43	1	0	0	0	0	1	4.24	4.65	4.50	4.21	4.91	1	0	0	0	0	1	0	9.68	9.55	9.52	9.48	9.28	8.78	8.68
1367942_at	Acp5 /// LOC100	1	0	0	1	1	0	5.97	5.84	5.57	6.53	5.80	1	0	0	0	1	1	1	5.15	5.27	5.59	5.61	5.76	6.15	6.03
1367977_at	Snca	1	0	1	1	1	1	6.00	5.93	7.07	5.49	6.52	1	0	0	0	0	1	1	7.61	7.61	7.72	7.82	7.69	8.68	9.05
1367998_at	Slpi	1	0	1	1	1	1	6.24	6.37	5.03	5.67	8.03	1	0	0	0	0	0	1	4.67	4.73	4.59	4.38	4.48	4.76	5.04
1367999_at	Aldh2	1	0	1	0	1	1	8.48	8.19	7.75	8.53	7.91	1	0	0	0	0	1	1	4.94	5.07	5.13	4.90	5.04	5.93	7.09
1368016_at	Pecr	1	1	1	0	1	1	9.49	8.98	8.80	9.62	9.00	1	0	0	0	0	1	1	6.47	6.73	6.67	6.56	6.70	7.38	7.51
1368046_at	SLC31A1	1	0	0	1	1	0	4.22	3.97	4.42	3.43	4.02	1	0	0	0	0	1	1	5.40	5.45	5.49	5.71	5.64	6.18	5.91
1368073_at	Irf1	1	1	0	0	0	0	7.24	8.38	7.48	7.30	7.40	1	0	0	0	0	1	1	7.06	7.04	7.16	7.15	7.28	7.72	7.77
1368075_at	Lipa	1	0	0	1	0	0	5.24	5.43	5.49	5.91	5.57	1	0	0	0	0	1	1	7.68	7.72	7.85	7.92	7.91	8.51	8.94
1368079_at	Pdk1	1	1	1	0	0	1	7.56	6.95	6.90	7.41	6.57	1	0	0	0	1	1	0	8.79	8.85	8.62	8.22	8.19	7.68	7.99
1368085_at	Gchfr	1	0	1	0	0	0	6.58	6.41	6.04	6.35	6.13	1	0	0	0	0	1	1	6.84	6.91	6.97	7.08	7.29	8.93	9.21
1368089_at	Pde2a	1	0	0	0	0	1	7.73	7.73	7.42	7.59	7.19	1	0	0	0	0	1	1	8.65	8.64	8.68	8.70	8.84	9.37	9.35
1368096_at	Rab29	1	0	0	0	1	1	9.60	9.50	9.23	10.01	9.11	1	0	0	0	0	1	1	8.48	8.52	8.56	8.63	8.75	9.26	9.07
1368097_a_at	Rtn1	1	0	0	0	1	0	8.09	8.25	8.52	7.72	8.28	1	0	0	0	0	0	1	10.37	10.38	10.41	10.37	10.46	10.71	10.74
1368114_at	Fgf13	1	1	0	0	0	0	5.50	5.01	5.66	5.53	5.79	1	0	0	0	1	1	1	8.53	8.49	8.19	8.01	7.90	7.47	7.36
1368120_at	Nell1	1	0	1	0	1	0	7.69	7.84	8.43	7.37	8.11	1	0	0	0	1	1	1	8.31	8.42	8.63	8.87	9.03	9.39	9.09
1368124_at	Dusp5	1	0	0	0	0	1	4.79	5.19	4.96	4.80	5.43	1	0	0	0	0	1	0	7.64	7.63	7.69	7.79	7.83	8.26	8.67
1368144_at	Rgs2	1	1	0	0	0	1	7.38	8.06	7.70	7.16	8.06	1	0	0	0	0	0	1	5.35	5.32	5.32	5.26	5.10	5.80	6.53
1368145_at	Pcp4	1	1	1	0	0	0	8.25	8.83	8.89	8.45	7.83	1	0	0	0	0	1	0	10.25	10.22	10.31	10.52	10.58	11.22	10.92
1368213_at	Por	1	0	1	0	1	0	6.61	6.70	7.30	6.21	6.81	1	0	0	0	0	1	0	8.57	8.72	8.84	8.91	8.87	9.18	8.26
1368238_at	Reg3a	1	1	1	1	1	1	10.58	9.15	8.38	9.29	11.96	1	0	0	0	0	1	1	7.46	7.83	7.46	7.09	7.43	5.95	4.38
1368247_at	Hspa1a /// Hspa	1	1	0	1	0	0	7.68	9.02	7.67	7.04	7.77	1	0	0	0	0	1	1	5.24	5.24	5.30	5.43	5.69	6.07	6.25
1368253_at	Gamt	1	0	1	0	1	1	10.28	9.95	9.06	10.13	9.51	1	0	0	0	0	1	1	7.64	7.67	7.72	7.85	7.90	8.57	8.37
1368256_at	Serpini1	1	0	1	0	0	1	7.00	7.01	7.72	7.41	7.69	1	0	0	0	0	1	1	9.23	9.32	9.38	9.60	9.76	10.47	10.32
1368259_at	Ptgs1	1	0	0	0	0	1	5.48	5.61	5.18	5.41	6.44	1	0	0	0	0	1	1	9.30	9.41	9.53	9.67	9.73	10.09	9.91
1368260_at	Aurkb	1	0	0	0	0	1	5.04	5.11	5.48	5.40	5.52	1	0	0	0	0	1	1	8.57	8.53	8.57	8.48	8.43	7.50	7.15
1368283_at	Ehhadh	1	1	0	0	0	1	5.58	4.82	5.68	5.54	4.98	1	0	0	1	1	1	0	5.09	5.31	5.47	5.76	5.83	6.00	5.78
1368288_at	Gc	1	0	1	0	1	0	10.74	10.69	11.60	10.38	11.21	1	0	0	0	0	1	0	10.07	10.03	9.97	9.76	9.67	9.44	10.20
1368289_at	Gc	1	0	0	0	0	1	5.48	5.15	5.68	5.27	6.53	1	0	0	0	0	0	1	4.55	4.48	4.52	4.26	4.24	4.16	4.01
1368309_at	Txnrd2	1	0	1	1	0	1	7.39	6.98	6.47	6.42	6.24	1	0	0	0	0	0	1	7.68	7.77	7.85	7.81	7.65	7.23	6.92
1368321_at	Egr1	1	0	0	0	0	1	11.40	11.68	11.28	11.12	10.64	1	0	0	0	0	0	1	10.74	10.68	10.70	10.60	10.64	10.99	11.09
1368350_at	Ptprz1	1	0	0	0	1	0	6.21	6.64	6.74	5.81	6.64	1	0	0	0	0	1	1	10.68	10.72	10.55	10.40	10.60	9.78	9.13
1368355_at	Myo5b	1	0	0	1	0	0	5.34	5.26	5.46	5.82	5.70	1	0	0	0	0	0	1	6.33	6.17	6.48	6.44	6.48	6.71	6.87
1368358_a_at	Ptprr	1	0	1	0	1	0	6.29	6.27	7.06	5.84	6.27	1	0	0	0	0	1	1	6.26	6.12	6.36	6.49	6.45	7.31	7.56
1368371_at	Kcnq1	1	0	1	1	1	1	7.80	7.57	7.01	8.50	7.19	1	0	0	0	0	1	0	7.63	7.64	7.84	7.94	8.05	8.60	8.38
1368401_at	Gria2	1	0	1	0	1	0	8.85	8.93	9.97	8.51	9.26	1	0	0	0	0	1	1	7.89	8.03	8.09	8.24	8.20	9.01	9.22
1368411_a_at	Map2	1	0	1	0	1	1	5.24	5.61	6.10	5.15	5.99	1	0	0	0	0	0	1	5.00	4.80	4.88	5.10	5.01	5.42	6.34
1368426_at	Crot	1	1	0	0	0	0	8.01	7.55	8.04	8.28	8.09	1	0	0	0	0	1	1	6.27	6.37	6.48	6.58	6.76	7.28	7.12
1368429_at	Taf9b	1	0	1	0	1	0	5.72	5.75	6.31	5.61	6.12	1	0	0	0	0	0	1	6.89	6.91	6.93	7.06	6.95	6.55	5.77
1368430_at	Lgmn	1	0	0	1	0	0	9.82	9.60	10.24	10.31	9.72	1	0	0	0	1	1	1	10.07	10.24	10.28	10.46	10.66	11.44	11.33
1368462_at	Itpka	1	0	1	0	0	0	6.83	6.70	6.26	6.65	6.50	1	0	0	0	0	1	1	5.55	5.56	5.55	5.48	5.75	6.20	6.42
1368466_a_at	Odf2	1	0	0	0	0	1	5.53	5.32	5.05	5.16	5.04	1	0	0	0	0	0	1	8.12	8.07	8.16	8.11	7.98	7.72	7.30
1368481_at	Gipr	1	0	1	0	1	1	8.30	8.26	8.97	8.00	8.95	1	0	0	0	0	1	1	11.15	11.19	11.15	10.89	10.87	10.13	9.48
1368488_at	Nfil3	1	1	1	1	1	0	6.57	7.56	6.02	7.16	6.99	1	0	0	0	0	1	0	7.39	7.29	7.15	6.97	6.85	6.67	6.69
1368502_at	Gast	1	0	0	0	0	1	5.49	5.69	5.33	5.26	5.02	1	0	0	0	0	1	0	8.89	8.90	9.05	9.28	9.36	9.56	10.01
1368512_a_at	Enpep	1	0	0	1	1	0	6.70	6.65	7.14	5.93	7.02	1	0	0	0	0	1	1	5.39	5.56	5.70	5.58	5.95	7.45	8.07
1368514_at	Maob	1	0	1	0	1	1	10.11	10.35	10.92	9.67	10.84	1	0	0	0	0	1	1	8.85	8.69	8.94	9.07	8.98	9.59	9.36
1368523_at	Cadps /// LOC10	1	0	1	0	1	0	8.84	9.02	9.53	8.59	9.19	1	0	0	0	0	1	1	8.08	8.09	8.13	8.14	8.04	6.99	6.73
1368536_at	Enpp2	1	0	0	1	1	1	6.21	6.26	6.32	5.55	6.73	1	0	0	0	0	1	1	7.58	7.61	7.64	7.82	8.01	9.52	9.23
1368539_at	Scn9a	1	0	1	1	1	0	5.59	5.69	6.76	4.85	5.19	1	0	0	0	0	0	1	3.65	3.59	3.58	3.50	3.63	3.72	4.56
1368593_at	Cd1d	1	0	0	0	1	1	7.09	6.92	6.85	7.41	6.60	1	0	0	0	0	0	1	4.92	4.88	4.87	4.79	4.99	5.08	5.44
1368612_at	Itgb4	1	0	0	1	1	0	6.79	6.60	6.35	7.49	6.43	1	0	0	0	0	1	1	4.31	4.69	4.64	4.46	4.68	5.11	5.24
1368622_at	Fbp2	1	1	1	1	0	1	8.32	7.58	7.40	7.83	7.21	1	0	0	0	0	1	1	4.86	4.85	4.80	4.77	4.93	5.58	6.20
1368709_at	Fut1	1	1	1	0	1	1	8.47	7.97	7.41	8.72	7.85	1	0	0	0	1	0	0	6.83	6.98	7.15	7.39	7.68	7.40	6.43
1368711_at	Foxa2	1	0	1	0	0	0	8.90	9.08	9.50	8.86	9.22	1	0	0	0	0	1	1	11.27	11.19	11.19	11.11	11.11	10.66	10.34
1368725_at	Jag1	1	0	0	0	1	1	6.47	6.35	6.29	6.91	5.91	1	0	0	0	0	0	1	4.24	4.33	4.24	4.13	4.13	4.72	4.56
1368801_at	Cxxc4	1	1	1	0	0	1	6.80	7.30	7.67	6.99	7.53	1	0	0	0	0	1	1	7.46	7.42	7.40	7.48	7.64	8.24	8.41
1368826_at	Comt	1	0	1	0	1	1	8.80	9.01	9.53	8.64	7.83	1	1	0	1	1	1	1	9.64	8.98	9.56	8.84	8.76	7.49	7.48
1368834_at	Camk2d	1	0	0	0	0	1	8.70	8.52	8.68	8.93	8.09	1	0	0	0	0	1	1	10.47	10.38	10.33	10.12	9.91	9.53	9.79
1368838_at	Tpm4	1	0	1	1	1	0	7.57	7.61	8.22	6.86	7.66	1	0	0	0	0	1	1	11.37	11.38	11.37	11.28	11.23	10.78	9.98
1368839_at	Wfs1	1	0	0	0	0	1	7.30	7.45	7.17	7.39	8.00	1	0	0	0	0	1	1	7.89	7.87	7.96	8.00	8.06	8.79	9.20
1368853_at	Vsnl1	1	1	1	1	1	1	6.91	7.45	7.97	5.73	7.47	1	0	0	0	0	1	1	4.48	4.38	4.62	4.61	4.63	5.64	6.04
1368854_at	Vsnl1	1	0	1	1	1	0	8.84	9.01	9.53	8.33	9.28	1	0	0	0	0	1	1	5.48	5.28	5.77	5.67	5.64	7.10	7.25
1368870_at	Id2	1	0	0	1	1	0	6.40	6.58	6.56	7.25	6.42	1	0	0	1	1	0	1	6.95	6.96	6.92	6.35	6.31	6.78	9.36
1368874_a_at	Mafg	1	0	1	0	1	0	5.17	5.14	5.76	4.74	5.29	1	0	0	0	0	0	1	8.23	8.19	8.30	8.23	8.16	7.68	6.99
1368885_at	Entpd1	1	0	1	1	1	1	7.41	7.28	6.88	8.03	6.76	1	0	0	1	1	1	1	5.70	5.80	6.24	6.46	6.49	7.01	7.08
1368902_at	Pak3	1	0	0	0	0	1	6.40	6.61	6.39	6.42	6.93	1	0	0	0	0	0	1	7.94	7.96	8.13	8.22	8.28	8.48	8.53
1368944_at	Dlg1	1	0	0	0	1	0	5.98	6.11	6.42	5.62	5.94	1	0	0	0	0	1	0	6.97	7.00	7.22	7.36	7.45	7.57	7.27
1368947_at	Gadd45a	1	0	1	1	1	1	5.85	6.25	6.44	5.23	6.65	1	0	0	0	0	1	1	9.43	9.47	9.51	9.54	9.51	10.17	10.22
1368960_at	Lgals8	1	0	0	0	1	0	6.56	6.56	7.09	6.14	6.40	1	0	0	0	0	0	1	8.00	7.96	7.99	7.92	7.87	7.47	6.90
1369000_at	Ntrk1	1	0	1	0	1	1	6.63	6.72	7.37	6.60	6.00	1	0	0	0	0	0	1	8.96	8.95	8.96	8.69	9.03	9.54	9.81
1369018_at	Foxm1	1	0	1	0	1	0	6.02	5.93	5.49	6.09	5.87	1	0	0	0	0	1	1	7.00	6.99	7.06	6.98	6.73	6.08	5.64
1369077_at	Asah1	1	0	1	1	1	0	7.64	7.81	8.41	6.85	7.71	1	0	0	0	0	1	0	10.14	10.17	10.21	10.28	10.35	10.79	9.70
1369097_s_at	Gucy1b1	1	0	0	0	1	0	4.14	4.16	4.69	3.86	4.25	1	0	0	0	0	1	1	7.83	7.88	7.98	8.22	8.20	8.55	8.50
1369153_at	Nphs1	1	0	0	0	0	1	4.06	3.93	3.61	3.86	3.22	1	0	0	0	0	0	1	3.48	3.65	3.80	3.82	3.61	3.97	4.38
1369157_at	Pde3b	1	0	1	1	1	0	7.05	7.30	8.07	6.11	7.48	1	0	0	0	0	0	1	9.43	9.34	9.52	9.64	9.52	9.20	7.82
1369182_at	F3	1	0	1	1	1	1	7.28	7.40	7.98	6.71	5.66	1	0	0	0	0	1	0	6.62	6.77	6.82	6.94	7.17	7.23	6.71
1369185_a_at	Syt7	1	0	1	0	0	0	4.73	5.13	5.32	4.72	5.18	1	0	0	0	0	1	0	8.83	8.51	8.81	8.60	8.59	8.05	7.13
1369187_a_at	Ptger3	1	0	1	0	0	1	9.08	8.65	8.47	8.77	8.48	1	0	0	0	0	0	1	8.64	8.74	8.59	8.47	8.56	8.62	7.73
1369203_at	Wif1	1	0	1	0	1	0	9.97	10.16	10.98	9.65	10.41	1	0	0	0	1	1	1	7.42	7.55	7.72	7.92	8.16	9.49	9.58
1369457_a_at	Sytl4	1	1	1	1	1	0	7.58	8.23	8.58	6.90	7.70	1	0	0	0	0	1	1	10.29	10.24	10.14	10.14	10.00	9.53	8.03
1369499_at	Tyms	1	0	0	1	0	0	5.78	5.94	6.21	6.26	6.14	1	0	0	0	0	1	1	7.57	7.34	7.47	7.58	7.33	6.76	6.24
1369554_at	Syngr2	1	0	0	0	0	1	8.51	8.14	8.30	8.38	7.97	1	0	0	0	0	1	1	8.85	8.95	9.00	9.00	9.04	9.69	9.19
1369562_at	Hpcal1	1	1	0	1	0	1	7.93	7.46	7.79	7.45	7.30	1	0	0	0	0	1	0	8.47	8.53	8.63	8.69	8.76	9.35	8.81
1369568_at	Stx6	1	0	0	0	1	0	5.98	6.03	6.45	5.55	6.02	1	0	0	0	0	1	0	6.45	6.54	6.68	6.70	6.67	7.10	6.56
1369579_at	Stc2	1	0	0	1	1	0	5.53	5.67	5.59	6.20	5.29	1	0	0	0	0	1	1	7.67	7.71	7.68	7.52	7.61	6.27	5.32
1369638_at	Eef2k	1	0	0	1	0	0	6.53	6.34	6.47	7.01	6.48	1	0	0	0	0	1	1	6.55	6.67	6.84	6.76	6.87	7.41	7.36
1369670_at	Cd200	1	0	1	1	1	0	8.49	8.40	9.35	7.79	8.51	1	0	0	0	0	0	1	10.49	10.60	10.69	10.71	10.79	10.24	9.03
1369679_a_at	Nfia	1	0	0	1	1	0	8.35	8.13	8.44	9.63	7.99	1	0	0	0	0	1	1	3.95	3.88	4.03	3.85	3.98	5.06	5.99
1369681_at	Isl1	1	0	1	1	1	1	7.81	7.85	8.54	7.16	8.29	1	0	0	0	0	1	0	10.93	10.98	11.12	11.27	11.34	11.67	11.35
1369777_a_at	Shank2	1	0	0	1	1	0	5.38	5.27	5.30	6.10	5.29	1	0	0	0	0	0	1	10.48	10.65	10.50	10.28	10.52	10.00	9.38
1369782_a_at	Kcnj11	1	0	0	1	1	0	6.44	6.28	6.95	5.66	6.23	1	0	0	0	0	1	1	9.75	9.72	9.65	9.56	9.35	8.33	6.85
1369792_at	Gpr6	1	0	0	0	0	1	5.02	5.25	5.42	5.03	5.66	1	0	0	0	0	0	1	11.22	11.23	11.15	11.02	11.01	10.74	9.91
1369816_at	Rab3a	1	0	0	1	1	0	5.37	5.29	5.80	4.72	5.35	1	0	0	0	0	1	0	8.88	8.94	9.11	9.27	9.25	9.58	8.30
1369888_at	Gcg	1	0	0	1	1	0	13.89	13.76	14.07	13.23	13.91	1	0	0	1	1	1	0	4.96	4.86	5.33	5.78	6.02	5.93	4.17
1369943_at	Tgm2	1	0	1	0	0	0	7.16	7.17	6.64	7.03	7.03	1	0	0	1	1	1	1	6.86	7.06	7.14	7.55	7.71	8.09	8.06
1369955_at	Col5a1	1	1	0	0	0	0	5.78	5.24	5.54	5.56	5.46	1	0	0	0	0	0	1	7.23	7.24	7.39	7.22	7.35	7.59	7.59
1369958_at	Rhob	1	0	0	1	0	0	8.35	8.63	8.30	7.85	8.08	1	0	0	0	0	0	1	10.02	9.96	10.10	10.13	10.06	10.60	10.51
1369964_at	Coro1a	1	0	0	0	0	1	4.58	4.59	4.42	4.31	5.51	1	0	0	1	1	1	0	6.92	7.17	7.40	7.64	7.58	7.72	7.28
1370034_at	Cdc25b	1	0	0	0	0	1	4.63	4.62	4.73	4.88	5.22	1	0	0	0	0	1	1	7.89	7.76	7.95	7.91	7.81	7.21	6.85
1370047_at	Enpp1	1	0	0	0	0	1	5.78	5.68	5.68	6.21	4.72	1	0	0	0	1	1	1	9.33	9.35	9.57	9.79	9.93	10.36	10.03

Probeset ID	Gene Symbol	Islet Data Set (log2 ratio ≥0.263; FDR <20%) union n=5325 1=YES 0=NO	Islet DRlyp/lyp ND vs ND+Lp299v (log2 ratio ≥0.263; FDR<20%) n=429 1=YES 0=NO	Islet DRlyp/lyp ND vs HCD (log2 ratio ≥0.263; FDR<20%) n=2247 1=YES 0=NO	Islet DRlyp/lyp ND vs HCD+Lp299v (log2 ratio ≥0.263; FDR<20%) n=1595 1=YES 0=NO	Islet DRlyp/lyp HCD vs HCD+Lp299v (log2 ratio ≥0.263; FDR<20%) n=3026 1=YES 0=NO	Islet DRlyp/lyp ND vs Flyp/lyp ND (log2 ratio ≥ 0.263; FDR<20%) n=1950 1=YES 0=NO	Islet Mean Log2 Intensity DRlyp/lyp ND	Islet Mean Log2 Intensity DRlyp/lyp ND+Lp299v	Islet Mean Log2 Intensity DRlyp/lyp HCD	Islet Mean Log2 Intensity DRlyp/lyp HCD+Lp299v	Islet Mean Log2 Intensity Flyp40 ND	Rinm5f data union of all conditions n=3705 1=YES 0=NO	Rinm5f 30 µM propionate/ 6 µM butyrate vs Media (FDR<20%; 1.5 fold) n=3 1=YES 0=NO	Rinm5f 60 µM propionate/ 12 µM butyrate vs Media (FDR<20%; 1.5 fold) n=18 1=YES 0=NO	Rinm5f 120 µM propionate/ 24 µM butyrate vs Media (FDR<20%; 1.5 fold) n=164 1=YES 0=NO	Rinm5f 240 µM propionate /48 µM butyrate vs Media (FDR<20%; 1.5 fold) n=268 1=YES 0=NO	Rinm5f 1500 µM propionate/ 300 µM butyrate vs Media (FDR<20%; 1.5 fold) n=2491 1=YES 0=NO	Rinm5f 500 µM propionate/ 500 µM butyrate vs Media (FDR<20%; 1.5 fold) n=3001 1=YES 0=NO	Rinm5f Mean Log2 Intensity Media only	Rinm5f Mean Log2 Intensity 30 µM propionate/ 6 µM butyrate	Rinm5f Mean Log2 Intensity 60 µM propionate/ 12 µM butyrate	Rinm5f Mean Log2 Intensity 120 µM propionate/ 24 µM butyrate	Rinm5f Mean Log2 Intensity 240 µM propionate/ 48 µM butyrate	Rinm5f Mean Log2 Intensity 1500 µM propionate /300 µM butyrate	Rinm5f Mean Log2 Intensity 500 µM propionate/ 500 µM butyrate
1370057_at	Csrp1	1	0	1	0	0	0	7.51	7.34	6.95	7.48	7.14	1	0	0	0	0	1	1	9.26	9.34	9.40	9.37	9.43	10.24	10.28
1370061_at	Rab3b	1	0	1	0	1	0	7.46	7.65	8.07	7.15	7.84	1	0	0	0	0	1	0	7.95	8.07	8.26	8.30	8.44	9.00	8.91
1370097_a_at	Cxcr4	1	0	0	0	1	0	4.43	4.26	4.80	4.02	4.76	1	0	0	0	0	1	1	4.99	5.33	5.31	5.46	5.35	6.18	6.34
1370122_at	Rab27b	1	0	1	0	1	1	5.11	5.04	4.52	5.49	4.61	1	0	0	0	0	0	1	5.10	5.05	5.05	5.05	5.11	5.30	5.57
1370186_at	Psmb9	1	1	0	0	0	0	5.86	7.03	6.02	5.41	6.22	1	0	0	0	0	1	1	8.29	8.28	8.27	8.41	8.41	9.21	9.29
1370201_at	Calb1	1	0	1	0	1	1	5.65	5.49	6.54	5.24	5.14	1	0	0	0	0	1	1	7.30	7.23	7.17	7.21	7.31	8.92	9.47
1370202_at	Plaat3	1	0	1	1	1	1	7.55	7.39	6.46	7.07	6.94	1	0	0	0	0	1	1	5.10	5.21	5.14	5.14	5.22	6.11	6.32
1370229_at	Ndrg4	1	0	1	0	1	1	8.84	9.03	9.80	8.48	9.48	1	0	0	0	0	1	1	12.28	12.21	12.15	11.90	11.89	11.15	10.21
1370239_at	Hba-a1 /// Hba-	1	1	0	1	1	1	11.49	11.00	11.38	10.54	10.42	1	0	0	0	0	0	1	5.33	5.52	5.36	5.19	5.33	5.72	5.82
1370240_x_at	Hba-a1 /// Hba-	1	0	0	1	1	1	11.48	11.04	11.33	10.59	10.47	1	0	0	0	0	0	1	5.99	6.08	6.04	5.85	5.94	6.42	6.61
1370244_at	Ctsv	1	0	0	0	0	1	10.84	10.54	10.68	10.82	10.32	1	0	0	0	0	1	1	10.83	10.95	11.03	11.21	11.28	11.89	11.53
1370248_at	Fxyd6	1	0	1	1	1	0	8.10	8.13	8.89	7.45	8.46	1	0	0	0	0	1	0	9.32	9.47	9.56	9.63	9.72	9.91	9.59
1370260_at	Add3	1	0	0	1	0	0	6.66	7.05	6.80	7.29	6.79	1	0	0	0	0	0	1	7.75	7.60	7.72	7.76	7.71	8.25	8.23
1370282_at	Csrp2	1	0	1	0	1	0	6.88	7.15	7.47	6.73	7.07	1	0	0	0	0	1	1	9.04	9.00	9.09	9.18	9.13	9.99	10.36
1370310_at	Hmgcs2	1	1	1	1	1	1	6.95	5.78	7.62	6.25	5.79	1	0	0	0	0	1	1	5.35	5.22	5.26	5.06	5.03	6.01	7.11
1370312_at	Spon1	1	0	0	1	1	1	5.71	5.58	6.16	5.14	4.92	1	0	0	0	0	1	1	4.17	4.06	4.15	4.30	4.38	5.59	6.00
1370323_at	Thop1	1	0	0	0	1	0	5.64	5.42	5.30	6.01	5.26	1	0	0	0	0	1	0	7.94	8.05	8.16	8.33	8.16	8.62	8.32
1370334_at	Plekhb1	1	0	0	0	0	1	7.18	7.14	7.10	7.11	6.66	1	0	0	0	0	1	1	6.43	6.53	6.73	6.82	6.89	7.24	7.52
1370336_at	Osgin1	1	0	1	0	1	0	6.80	7.08	5.94	6.91	6.50	1	0	0	0	0	1	1	6.05	6.05	6.11	6.14	6.21	6.66	6.81
1370341_at	Eno2	1	0	0	0	0	1	8.34	8.58	8.46	8.44	8.98	1	0	0	0	0	1	0	10.57	10.58	10.45	10.03	10.24	9.74	9.34
1370342_at	Kcnk2	1	0	1	0	0	0	4.62	4.59	5.28	4.65	4.53	1	0	0	0	1	1	0	6.42	6.24	6.23	6.08	5.81	5.52	5.26
1370348_at	Ninj1	1	0	0	0	1	0	8.07	8.15	7.67	8.38	7.72	1	0	0	0	0	0	1	11.73	11.77	11.61	11.23	11.39	11.22	10.66
1370374_at	Steap3	1	0	1	0	1	0	6.12	5.89	5.46	6.28	5.69	1	0	0	0	0	0	1	4.96	4.97	4.85	4.63	4.85	5.35	5.79
1370375_at	Gls2	1	1	0	0	0	1	10.30	9.84	10.19	10.49	9.65	1	0	0	0	0	0	1	7.99	8.04	7.91	7.79	7.96	8.31	8.13
1370384_a_at	Prlr	1	1	0	0	0	1	9.23	9.72	9.74	9.31	9.76	1	0	0	0	0	0	1	10.47	10.49	10.47	10.52	10.42	9.92	9.28
1370392_at	Trpm4	1	0	1	1	1	1	6.47	6.43	5.68	6.97	5.76	1	0	0	0	0	1	0	5.93	6.18	6.08	6.12	6.34	6.75	6.27
1370439_a_at	Kcnc2	1	0	1	0	0	0	3.59	3.66	4.19	3.61	3.59	1	0	0	1	1	1	0	5.49	5.73	5.88	6.25	6.36	6.39	5.84
1370442_at	Tmsb15b2	1	0	0	1	0	0	10.64	10.67	10.65	10.15	10.95	1	0	0	0	0	1	0	9.20	9.33	9.41	9.66	9.57	10.12	11.39
1370443_at	Dnase2	1	0	0	0	1	0	6.98	6.74	6.53	7.11	6.64	1	0	0	0	0	1	1	6.46	6.53	6.53	6.61	6.73	7.87	7.66
1370522_at	Gcgr	1	0	0	0	0	1	5.83	5.95	6.03	5.78	6.80	1	0	0	0	0	0	1	7.77	7.86	7.86	7.71	7.79	7.41	7.12
1370534_at	Acvr1c	1	0	0	0	1	0	6.04	5.99	6.53	5.70	6.09	1	0	0	0	0	1	1	5.08	5.09	5.27	5.20	5.27	6.55	7.30
1370548_at	Slc16a10	1	0	1	0	1	0	6.18	6.45	6.82	6.06	6.56	1	0	0	0	0	0	1	6.07	6.01	6.00	6.15	6.12	6.50	6.76
1370609_a_at	Slc16a7	1	0	0	0	0	1	7.59	7.31	7.41	7.81	7.12	1	0	0	0	0	1	1	7.93	7.96	7.85	7.72	7.84	6.62	6.46
1370687_a_at	Ntrk2	1	0	1	0	1	0	7.83	7.82	8.67	7.65	7.46	1	0	0	0	0	1	1	9.70	9.69	9.61	9.48	9.50	9.08	8.46
1370691_a_at	Thra	1	0	0	0	1	0	6.75	6.69	6.25	6.92	6.37	1	0	0	0	0	1	1	6.75	6.82	7.15	7.02	7.11	7.34	7.58
1370694_at	Trib3	1	0	0	1	1	0	5.39	5.26	5.40	6.50	5.25	1	0	0	0	0	0	1	7.34	7.41	7.38	7.62	7.60	6.97	6.31
1370714_a_at	St6gal1	1	0	1	1	1	0	5.81	6.05	6.72	5.10	5.65	1	0	0	0	0	0	1	5.64	5.77	5.64	5.34	5.58	5.32	4.14
1370746_at	Prkacb	1	0	1	1	1	0	6.37	6.77	7.13	5.51	6.05	1	0	0	0	0	0	1	7.76	7.68	7.78	7.92	7.71	7.33	5.67
1370750_a_at	Il1r1	1	1	1	0	1	0	5.63	6.37	6.33	5.64	5.36	1	0	0	0	0	0	1	8.20	8.10	8.33	8.41	8.34	8.29	6.20
1370789_a_at	Prlr	1	0	1	1	1	0	7.62	8.04	8.35	7.10	7.97	1	0	0	0	0	1	1	9.01	9.00	8.97	9.08	8.91	8.32	7.03
1370813_at	Gstm3	1	0	0	0	0	1	6.37	6.23	6.14	6.55	5.77	1	0	0	0	0	1	1	9.01	9.19	9.24	9.25	9.47	10.21	10.58
1370823_at	Bambi	1	0	1	0	1	0	7.22	7.04	7.94	6.90	7.17	1	0	0	0	0	1	1	7.31	7.37	7.48	7.67	7.67	8.32	9.13
1370880_at	Rnh1	1	0	1	0	1	1	10.86	10.67	10.16	10.76	10.23	1	0	0	0	0	0	1	10.21	10.27	10.28	10.27	10.23	9.88	9.50
1370881_at	Tst	1	0	0	1	1	1	8.75	8.44	8.36	9.28	7.87	1	0	0	0	0	1	1	6.14	6.37	6.33	6.44	6.44	7.64	7.70
1370913_at	Rsad2	1	1	0	0	0	0	4.52	5.58	4.58	4.45	4.54	1	0	0	1	1	1	1	5.82	5.69	6.17	7.03	6.53	6.78	6.77
1370932_at	Lrp4	1	1	0	0	0	1	5.33	4.85	5.00	5.33	4.71	1	0	0	0	0	0	1	3.66	3.67	3.67	3.52	3.46	3.92	4.27
1370948_a_at	Marcks	1	0	1	0	0	1	8.13	8.14	8.84	8.49	9.26	1	0	0	0	0	0	1	10.20	10.17	10.32	10.42	10.34	9.93	9.17
1370949_at	Marcks	1	0	0	0	0	1	8.72	8.81	8.99	9.10	9.53	1	0	0	0	0	0	1	9.54	9.53	9.57	9.65	9.61	9.38	8.77
1370952_at	Gstm1	1	1	1	1	0	1	7.82	7.32	6.80	7.31	7.24	1	0	0	0	0	0	1	4.48	4.42	4.44	4.30	4.26	4.83	5.63
1370973_at	Scn7a	1	0	1	0	1	0	8.57	8.55	9.39	8.21	8.27	1	0	0	0	0	1	1	6.82	6.80	7.04	7.14	7.21	7.78	8.83
1370989_at	Ret	1	0	0	0	1	0	7.77	7.73	8.32	7.42	8.14	1	0	0	0	0	0	1	7.11	7.14	7.22	7.17	7.31	7.60	7.01
1371003_at	Map1b	1	0	1	1	0	0	4.87	5.14	5.84	5.45	5.09	1	1	0	0	0	0	0	7.46	6.85	7.32	7.24	7.09	6.94	7.51
1371020_at	Rimbp2	1	0	0	0	1	0	7.27	7.35	7.66	6.92	7.37	1	0	0	0	0	0	1	8.73	8.65	8.86	8.85	8.78	8.16	7.58
1371031_at	Mat1a	1	0	1	0	1	1	9.14	8.78	8.02	9.16	8.18	1	0	0	0	0	1	1	7.61	7.64	7.82	7.91	7.95	8.35	8.56
1371034_at	Onecut1	1	0	0	1	1	0	6.76	6.73	6.43	7.57	6.73	1	0	0	0	0	1	1	5.24	5.17	5.36	5.32	5.42	6.06	6.72
1371099_at	Pigr	1	0	1	0	1	1	9.42	9.32	8.48	9.42	8.46	1	0	1	1	1	1	1	6.14	6.32	6.78	7.20	7.64	8.23	7.56
1371133_a_at	Prkar2b	1	0	1	0	1	0	6.83	7.18	7.65	6.79	7.27	1	0	0	0	0	1	1	10.04	10.06	10.12	10.16	10.14	10.79	11.17
1371139_at	Pls3	1	0	1	1	1	0	6.33	6.26	6.99	5.00	6.37	1	0	0	0	0	1	1	7.66	7.67	7.81	7.94	7.91	8.37	7.96
1371209_at	Hla-a	1	1	0	1	1	0	4.26	3.65	4.17	5.22	4.14	1	0	1	1	1	1	1	4.24	4.48	4.88	5.04	5.22	6.56	6.52
1371237_a_at	Mt1	1	1	1	1	0	1	12.87	12.33	11.71	11.66	12.09	1	0	0	1	0	0	1	11.20	11.29	11.08	10.58	10.76	11.23	12.14
1371263_a_at	Camk2d	1	0	0	0	0	1	4.75	4.44	4.84	4.29	4.22	1	0	0	0	0	1	1	7.63	7.41	7.54	7.33	7.11	6.68	6.08
1371352_at	Hmgn2	1	0	1	0	1	0	10.22	10.06	10.87	10.06	10.37	1	0	0	0	0	1	1	13.05	12.99	13.04	12.99	12.87	12.25	11.98
1371357_at	Igfbp7	1	0	0	0	1	0	11.02	11.12	11.58	10.60	11.40	1	0	0	0	0	1	1	9.19	9.13	9.16	9.24	9.22	9.89	10.03
1371382_at	Flna	1	0	1	0	1	0	6.87	6.86	6.27	6.95	6.67	1	0	0	0	0	1	0	6.63	6.67	6.73	6.63	6.75	7.31	7.50
1371390_at	Tubb4b	1	0	0	0	0	1	8.48	8.64	8.76	8.68	8.98	1	0	0	0	0	0	1	12.25	12.21	12.23	12.18	12.14	11.76	11.56
1371406_at	Cavin1	1	0	1	0	0	0	8.39	8.24	7.78	8.32	7.96	1	0	0	0	0	1	0	6.19	6.30	6.32	6.19	6.31	6.87	6.92
1371414_at	Gsn	1	1	1	1	0	1	8.60	8.00	8.08	7.48	7.96	1	0	0	0	0	1	1	7.13	7.29	7.41	7.43	7.58	9.26	9.89
1371421_at	Oxct1	1	0	0	1	1	0	8.84	8.85	9.28	8.34	9.07	1	0	0	0	0	0	1	9.62	9.53	9.56	9.55	9.51	9.85	10.28
1371440_at	B2m	1	1	0	1	0	0	8.49	9.00	8.40	7.88	8.89	1	0	0	0	0	1	1	9.21	9.26	9.29	9.43	9.58	10.40	9.95
1371447_at	Plac8	1	0	1	0	1	1	7.10	7.40	6.34	6.93	7.89	1	0	0	0	1	1	1	9.41	9.55	9.64	9.93	10.16	10.66	10.17
1371471_at	Nop53	1	0	0	0	0	1	9.68	9.39	9.79	10.05	8.92	1	0	0	0	0	0	1	10.15	10.19	10.12	10.06	9.97	9.60	9.29
1371499_at	Cd9	1	0	0	1	0	0	5.98	5.83	6.49	6.51	6.11	1	0	0	0	0	1	1	7.59	7.75	7.72	7.80	8.00	8.84	8.87
1371539_at	Nhp2	1	0	1	0	1	0	8.41	8.18	7.88	8.64	8.19	1	0	0	0	0	1	1	8.94	8.86	8.90	8.85	8.65	8.24	8.05
1371545_at	PECAM1	1	0	1	1	0	1	6.98	6.57	6.23	6.39	6.38	1	0	0	0	0	1	1	4.45	4.48	4.58	4.59	4.54	5.53	6.65
1371549_at	Dcaf11	1	0	0	0	1	0	9.53	9.52	9.25	9.90	9.15	1	0	0	0	0	1	1	9.33	9.31	9.34	9.25	9.14	8.66	8.42
1371568_at	Sncb	1	0	0	0	0	1	7.17	7.49	7.60	7.23	7.66	1	0	0	0	0	1	1	11.37	11.35	11.27	10.97	10.98	10.73	10.51
1371583_at	Rbm3	1	1	0	0	0	0	9.51	9.02	9.82	9.43	9.24	1	0	0	0	0	0	1	11.79	11.81	11.81	11.87	11.85	11.29	10.77
1371606_at	Wdtc1	1	0	0	0	1	0	7.87	7.93	7.41	8.22	7.64	1	0	0	0	0	0	1	7.14	7.14	7.17	7.00	6.97	7.38	7.74
1371618_s_at	Tubb3	1	0	0	0	0	1	5.89	5.83	6.10	5.77	6.51	1	0	0	0	0	1	1	9.82	9.84	9.88	9.85	9.70	9.10	8.48
1371692_at	Mllt11	1	0	0	0	0	1	7.40	7.63	7.54	7.43	8.00	1	0	0	0	0	1	0	9.12	9.11	9.22	9.23	9.20	8.49	8.07
1371703_at	Ahnak	1	0	1	1	0	0	7.71	7.33	7.01	7.20	7.33	1	0	0	0	0	1	1	5.18	5.17	5.25	5.18	5.35	6.24	7.59
1371735_at	---	1	0	1	1	0	0	7.33	7.30	8.28	8.53	7.08	1	0	0	0	0	0	1	10.09	10.13	10.15	10.26	10.17	9.66	8.91
1371762_at	Rbp4	1	0	0	0	0	1	6.69	6.47	6.79	6.25	7.41	1	0	0	0	1	1	1	6.33	6.45	6.57	6.78	7.02	7.22	7.02
1371763_at	C11orf54	1	0	1	0	1	0	8.07	8.06	8.83	7.71	8.23	1	0	0	0	0	0	1	10.00	9.98	9.97	9.94	9.84	9.46	9.09
1371774_at	Sat1	1	1	0	1	1	0	9.01	9.62	9.52	8.49	9.13	1	0	0	0	0	1	1	8.07	8.10	8.07	8.08	8.17	9.10	8.70
1371775_at	Acadsb /// LOC1	1	0	0	0	0	1	9.97	9.76	9.89	9.88	9.27	1	0	0	0	0	0	1	7.30	7.39	7.41	7.28	7.36	7.58	7.64
1371849_at	Nt5dc2	1	0	1	0	1	0	7.98	7.91	7.41	8.21	7.62	1	0	0	0	0	1	1	6.24	6.38	6.50	6.53	6.73	7.42	7.98
1371854_at	---	1	0	1	1	0	1	8.55	8.41	9.17	9.34	8.07	1	0	0	0	0	1	1	10.22	10.21	10.15	10.05	9.95	9.53	8.92
1371875_at	Manba	1	0	1	0	1	0	8.24	8.20	8.92	8.00	8.04	1	0	0	0	0	1	1	8.88	8.97	8.97	9.08	9.28	10.23	10.17
1371912_at	Ndufs7	1	0	0	0	1	0	8.86	9.05	8.60	9.26	8.56	1	0	0	0	0	0	1	10.72	10.71	10.63	10.55	10.43	10.15	9.70
1371988_at	Man1a1	1	0	0	0	0	1	7.97	7.77	7.89	7.95	7.50	1	0	0	0	0	1	1	7.78	7.66	7.68	7.66	7.64	8.72	9.15
1372004_at	Hebp1	1	0	0	1	1	1	6.89	6.77	6.69	7.38	6.36	1	0	0	0	0	1	1	5.82	6.06	5.93	5.75	6.04	6.48	7.36
1372011_at	---	1	0	1	0	1	1	7.30	6.98	6.56	7.28	6.44	1	0	0	0	0	1	1	4.37	4.42	4.34	4.44	4.41	5.92	6.59
1372013_at	Ifitm1	1	0	0	0	0	1	6.41	6.53	6.26	6.27	6.98	1	0	0	0	0	0	1	9.78	9.93	9.81	9.77	9.97	9.66	8.54
1372016_at	Gadd45b	1	0	1	1	0	1	5.99	6.20	5.00	5.06	5.51	1	0	0	0	0	1	0	7.58	7.57	7.51	7.42	7.42	8.25	8.13
1372043_at	Mrto4	1	0	0	0	1	0	7.49	7.42	7.24	7.90	7.13	1	0	0	0	0	1	1	8.46	8.46	8.49	8.48	8.25	7.77	7.62
1372072_at	Hdhd2	1	0	0	0	0	1	6.86	6.82	6.90	6.69	6.26	1	0	0	0	0	1	0	8.34	8.44	8.52	8.52	8.57	9.01	8.77
1372095_at	Myorg	1	0	0	1	1	0	6.22	6.09	5.75	7.00	5.86	1	0	0	0	0	1	1	6.43	6.48	6.63	6.50	6.77	7.41	7.52
1372101_at	Plpp3	1	0	0	0	1	0	9.49	9.04	9.85	9.10	9.04	1	0	0	0	0	0	1	3.57	3.59	3.55	3.64	3.65	4.14	4.83
1372111_at	Cav1	1	0	0	1	0	1	8.24	7.83	7.80	7.61	7.50	1	0	0	0	0	0	1	3.81	3.93	3.94	3.93	3.89	4.32	4.78
1372119_at	Uba5	1	0	0	0	1	0	10.03	9.99	9.65	10.31	9.66	1	0	0	0	0	0	1	9.23	9.20	9.32	9.31	9.26	8.73	8.26
1372133_at	Rras2	1	0	0	0	0	1	7.45	7.19	7.07	7.60	6.87	1	0	0	0	0	1	1	9.06	9.16	9.17	9.27	9.36	9.96	10.14
1372137_at	Bloc1s1	1	0	0	0	1	0	7.56	7.81	7.34	8.02	7.36	1	0	0	0	0	1	1	9.69	9.68	9.64	9.66	9.53	9.06	8.89
1372163_at	---	1	0	1	1	1	0	6.89	7.02	7.51	6.33	7.31	1	0	0	1	1	1	1	9.23	9.34	9.57	9.84	9.87	10.05	9.55
1372170_at	Acy1	1	0	0	0	0	1	7.83	7.64	7.49	8.03	7.30	1	0	0	0	0	1	1	6.84	6.84	6.95	7.14	7.01	7.66	8.11
1372219_at	Tpm2	1	1	0	0	1	1	5.83	6.45	5.45	6.22	5.25	1	0	0	1	1	1	1	6.47	6.72	6.94	7.29	7.73	8.63	9.01
1372226_at	---	1	0	0	1	0	0	4.70	4.92	4.80	5.34	4.89	1	0	0	0	0	1	1	5.33	5.38	5.29	5.28	5.27	5.92	5.91
1372296_at	Sh3bgr	1	0	0	0	0	1	5.39	5.70	5.91	5.43	5.90	1	0	0	0	0	1	0	7.00	7.07	7.14	7.25	7.37	7.59	7.74
1372297_at	Gsta4	1	0	0	1	1	1	7.98	7.66	7.60	8.47	7.17	1	0	0	1	1	1	1	5.12	5.19	5.45	5.93	6.31	8.44	8.86
1372307_at	Arhgap23	1	0	0	1	1	0	7.44	7.32	6.97	7.96	7.20	1	0	0	0	0	0	1	6.81	6.92	6.98	6.86	7.01	7.19	7.32
1372413_at	Mcrs1	1	0	0	0	1	0	7.81	7.73	7.45	8.04	7.67	1	0	0	0	0	0	1	10.02	9.98	10.01	9.91	9.85	9.58	9.21
1372440_at	Serpine2	1	1	1	1	1	0	7.39	6.87	8.00	6.78	6.95	1	0	0	0	0	1	1	10.60	10.69	10.55	10.45	10.50	9.55	8.79
1372447_at	Fgfr1	1	0	1	0	1	0	7.66	7.39	7.12	7.86	7.42	1	0	0	0	0	1	0	8.83	8.87	8.73	8.32	8.52	8.20	7.90
1372485_at	Pcbd1	1	0	1	0	0	0	8.78	8.82	9.41	8.94	9.01	1	0	0	0	0	1	1	11.87	11.89	11.82	11.83	11.72	11.13	10.63
1372511_at	Dazap2	1	0	0	1	1	0	8.32	8.25	8.88	7.57	8.32	1	0	0	0	0	0	1	11.28	11.25	11.20	11.14	11.06	10.82	10.22
1372542_at	Qki	1	0	0	1	1	1	5.71	5.39	5.78	4.98	5.23	1	0	0	0	0	1	1	4.79	4.69	4.95	5.16	5.13	5.87	5.72
1372563_at	Rgd1308143	1	0	1	0	1	1	8.03	8.12	8.80	7.89	8.53	1	0	0	0	0	1	0	8.13	8.09	8.27	8.43	8.42	8.79	8.43
1372578_at	Vars1	1	0	0	1	1	0	6.71	6.85	6.41	7.32	6.64	1	0	0	0	0	1	0	8.33	8.21	8.41	8.21	8.26	7.69	7.92
1372584_at	Cnrip1	1	0	0	0	1	1	7.06	7.22	7.55	6.79	7.60	1	0	0	0	0	1	1	10.08	10.12	10.11	10.09	10.04	9.46	8.95
1372599_at	Mgst2	1	0	0	1	0	0	7.55	7.26	7.81	8.22	7.55	1	0	0	0	0	0	1	6.98	7.02	7.08	6.92	6.97	7.39	7.79
1372601_at	Atf5	1	0	0	1	1	0	9.59	9.23	9.39	10.39	9.26	1	0	0	0	0	1	1	10.34	10.25	10.46	10.72	10.47	9.44	8.56
1372603_at	Pacsin3	1	0	1	0	1	1	7.56	7.27	6.96	7.52	6.87	1	0	0	0	0	1	1	7.08	7.19	7.24	7.20	7.34	7.84	7.43
1372604_at	LOC100910885 /	1	1	1	0	1	1	7.45	6.94	6.85	7.44	6.85	1	0	0	0	0	1	1	7.13	7.18	7.32	7.37	7.44	7.96	8.00
1372613_at	Bdh2	1	0	0	0	0	1	7.50	7.10	7.46	7.92	6.18	1	0	0	0	0	0	1	4.48	4.39	4.29	4.34	4.32	4.93	5.56
1372626_at	Tpd52l1	1	0	0	0	1	1	6.24	6.34	6.78	5.92	7.11	1	0	0	0	0	1	1	7.49	7.53	7.69	7.91	7.91	8.31	8.40
1372653_at	Arf3 /// Fkbp11	1	0	0	0	1	0	10.59	10.45	10.17	10.94	10.17	1	0	0	0	0	0	1	10.38	10.52	10.51	10.56	10.61	10.14	9.70
1372669_at	---	1	0	0	1	1	0	6.53	6.72	6.98	5.96	6.79	1	0	0	0	0	0	1	7.47	7.47	7.61	7.77	7.69	7.35	6.90
1372681_at	Stxbp6	1	0	0	1	1	1	7.70	7.48	7.39	8.19	7.22	1	0	0	0	0	0	1	4.21	4.42	4.17	4.37	4.18	4.74	4.95
1372697_at	Mrps15	1	0	0	1	1	0	8.17	8.49	8.07	8.77	7.88	1	0	0	0	0	0	1	9.96	9.94	9.92	9.95	9.84	9.40	9.00
1372774_at	Entpd5	1	0	0	0	0	1	6.08	6.16	6.62	6.36	6.56	1	0	0	0	0	0	1	7.80	7.70	7.76	7.69	7.70	8.06	8.60
1372790_at	Mdh1	1	0	1	0	1	0	8.63	9.00	9.29	8.36	8.86	1	0	0	0	0	1	1	10.07	10.11	10.21	10.39	10.49	10.89	10.81
1372844_at	Efna1	1	0	0	1	0	0	5.65	5.90	5.72	6.20	5.49	1	0	0	0	0	0	1	8.96	9.04	8.96	8.87	8.77	8.58	8.20
1372845_at	Rpp21	1	0	0	1	1	1	7.45	7.50	7.26	8.04	6.88	1	0	0	0	0	0	1	10.00	9.98	9.99	10.01	9.89	9.48	9.22
1372870_at	Kdelr3	1	0	1	0	1	1	7.34	7.12	6.48	7.30	6.64	1	0	0	0	0	0	1	9.16	9.27	9.17	9.00	9.15	9.69	10.01
1372895_at	Prxl2a	1	0	0	0	0	1	8.59	8.48	8.29	8.59	8.10	1	0	0	0	0	1	1	10.59	10.58	10.70	10.74	10.76	11.28	11.35

Probeset ID	Gene Symbol	Islet Data Set (log2 ratio ≥0.263; FDR <20%) union n=5325 1=YES 0=NO	Islet DRlyp/lyp ND vs ND+Lp299v (log2 ratio ≥0.263; FDR<20%) n=429 1=YES 0=NO	Islet DRlyp/lyp ND vs HCD (log2 ratio ≥0.263; FDR<20%) n=2247 1=YES 0=NO	Islet DRlyp/lyp ND vs HCD+Lp299v (log2 ratio ≥0.263; FDR<20%) n=1595 1=YES 0=NO	Islet DRlyp/lyp HCD vs HCD+Lp299v (log2 ratio ≥0.263; FDR<20%) n=3026 1=YES 0=NO	Islet DRlyp/lyp ND vs Flyp/lyp ND (log2 ratio ≥ 0.263; FDR<20%) n=1950 1=YES 0=NO	Islet Mean Log2 Intensity DRlyp/lyp ND	Islet Mean Log2 Intensity DRlyp/lyp ND+Lp299v	Islet Mean Log2 Intensity DRlyp/lyp HCD	Islet Mean Log2 Intensity DRlyp/lyp HCD+Lp299v	Islet Mean Log2 Intensity Flyp40 ND	Rinm5f data union of all conditions n=3705 1=YES 0=NO	Rinm5f 30 µM propionate/ 6 µM butyrate vs Media (FDR<20%; 1.5 fold) n=3 1=YES 0=NO	Rinm5f 60 µM propionate/ 12 µM butyrate vs Media (FDR<20%; 1.5 fold) n=18 1=YES 0=NO	Rinm5f 120 µM propionate/ 24 µM butyrate vs Media (FDR<20%; 1.5 fold) n=164 1=YES 0=NO	Rinm5f 240 µM propionate /48 µM butyrate vs Media (FDR<20%; 1.5 fold) n=268 1=YES 0=NO	Rinm5f 1500 µM propionate/ 300 µM butyrate vs Media (FDR<20%; 1.5 fold) n=2491 1=YES 0=NO	Rinm5f 500 µM propionate/ 500 µM butyrate vs Media (FDR<20%; 1.5 fold) n=3001 1=YES 0=NO	Rinm5f Mean Log2 Intensity Media only	Rinm5f Mean Log2 Intensity 30 µM propionate/ 6 µM butyrate	Rinm5f Mean Log2 Intensity 60 µM propionate/ 12 µM butyrate	Rinm5f Mean Log2 Intensity 120 µM propionate/ 24 µM butyrate	Rinm5f Mean Log2 Intensity 240 µM propionate/ 48 µM butyrate	Rinm5f Mean Log2 Intensity 1500 µM propionate /300 µM butyrate	Rinm5f Mean Log2 Intensity 500 µM propionate/ 500 µM butyrate
1372921_at	Slc38a1	1	1	1	0	0	0	5.29	4.83	5.93	5.29	5.57	1	0	0	0	0	1	1	9.74	9.69	9.84	9.85	9.82	9.13	9.08
1372928_at	Thumpd3-as1	1	0	1	0	0	1	6.05	6.03	6.66	6.45	6.67	1	0	0	0	0	0	1	8.26	8.30	8.26	8.36	8.30	7.79	7.23
1372929_at	Kcnma1	1	0	1	0	1	0	9.24	9.49	10.10	9.27	9.66	1	0	0	0	0	1	1	10.49	10.59	10.48	10.39	10.47	9.62	8.93
1372947_at	Pls3	1	0	0	0	1	0	8.76	8.76	9.24	8.30	9.17	1	0	0	0	0	1	1	8.99	9.14	9.13	9.31	9.35	9.90	9.97
1372953_at	Ncald	1	0	0	0	1	0	7.45	7.75	7.97	7.20	7.83	1	0	0	0	0	1	1	9.79	9.88	9.82	9.66	9.88	10.52	11.01
1372961_at	Tmub1	1	0	0	0	1	0	6.59	6.65	6.29	6.88	6.50	1	0	0	0	0	0	1	8.14	8.16	8.24	8.21	8.10	7.84	7.30
1373032_at	Mustn1	1	0	1	0	0	1	5.96	6.23	5.36	5.91	5.31	1	0	0	0	0	0	1	7.55	7.48	7.50	7.40	7.29	7.09	6.72
1373036_at	Iqgap2	1	1	0	0	0	1	7.42	6.80	6.94	7.23	6.74	1	0	0	0	0	1	0	5.65	5.80	6.05	6.04	5.97	6.36	6.41
1373049_at	Macir	1	0	1	0	0	0	6.30	6.11	6.90	6.21	6.39	1	0	0	0	0	0	1	7.27	7.29	7.37	7.47	7.36	7.71	7.94
1373093_at	Errfi1	1	1	0	1	1	1	10.54	9.06	10.13	9.21	10.06	1	0	0	1	1	1	0	11.19	11.17	10.98	10.40	10.57	10.02	10.23
1373151_at	Lhfpl6	1	0	0	0	1	0	5.25	5.22	5.64	4.86	5.14	1	0	0	0	0	0	1	5.78	5.97	6.02	6.04	6.03	6.34	6.36
1373152_at	Prss23	1	0	0	0	1	0	9.93	9.82	10.33	9.62	10.19	1	0	0	0	0	1	1	6.58	6.61	6.54	6.64	6.79	7.52	8.34
1373158_at	Gpr146	1	0	0	0	0	1	4.97	4.67	4.63	4.61	4.43	1	0	0	0	0	1	1	5.36	5.36	5.40	5.22	5.31	6.03	6.28
1373172_at	Lrrc75a	1	0	0	0	1	0	6.10	6.14	5.83	6.45	5.82	1	0	0	0	0	1	1	6.30	6.26	6.41	6.49	6.54	7.29	7.72
1373178_at	LOC103691479	1	1	0	0	0	0	9.38	8.73	9.66	9.35	8.97	1	0	0	0	0	0	1	7.26	7.23	7.35	7.34	7.37	7.81	8.11
1373200_at	Eef1e1	1	0	1	0	0	1	7.87	7.84	8.48	7.79	8.55	1	0	0	0	0	0	1	10.78	10.74	10.81	10.91	10.81	10.39	9.72
1373210_at	Lamb1	1	0	0	1	1	1	8.19	7.88	8.20	8.80	7.69	1	0	0	0	1	1	1	7.35	7.69	7.71	7.84	8.09	8.53	8.99
1373240_at	Dhrs3	1	0	0	1	1	0	7.21	7.14	6.86	7.75	6.84	1	0	0	0	0	0	1	4.67	4.60	4.67	4.53	4.68	5.18	5.02
1373288_at	Dennd2b	1	0	0	0	1	1	7.70	7.59	7.37	8.05	7.24	1	0	0	0	0	0	1	5.02	5.02	5.14	4.94	5.23	5.47	5.65
1373326_at	Slc4a10	1	0	1	0	1	0	3.76	3.78	4.37	3.66	3.97	1	0	0	1	1	1	1	5.05	5.24	5.49	5.86	5.92	6.19	5.28
1373329_at	Tmprss2	1	0	0	0	1	0	7.22	7.10	6.87	7.52	7.01	1	0	0	0	0	1	1	5.05	5.16	5.28	5.21	5.22	5.80	5.74
1373333_at	Arxes2 /// MGC	1	0	1	0	1	1	6.17	6.39	7.18	6.33	6.90	1	0	0	0	0	1	1	9.12	9.28	9.40	9.65	9.67	10.09	10.13
1373336_at	Gprc5b	1	0	1	1	1	1	6.22	6.38	6.94	5.69	6.91	1	0	0	0	0	1	0	8.90	8.90	8.94	9.02	9.16	9.52	9.39
1373345_at	Amigo2	1	0	1	0	1	1	8.39	8.34	9.02	8.12	7.86	1	0	0	0	0	0	1	9.28	9.47	9.48	9.58	9.78	9.86	9.75
1373490_at	Gmfg	1	0	0	0	0	1	5.15	5.09	5.15	5.23	5.75	1	0	0	0	0	1	1	6.88	6.95	6.94	7.08	7.25	7.57	7.58
1373499_at	Gas5	1	0	0	1	0	0	8.38	8.23	8.68	8.94	8.16	1	0	0	0	0	1	1	10.34	10.46	10.44	10.49	10.43	9.58	8.82
1373546_at	Atp11a	1	0	0	0	0	1	7.91	7.76	8.23	7.82	9.32	1	0	0	0	0	1	1	10.35	10.42	10.53	10.58	10.73	11.25	10.71
1373574_at	Cnpy4	1	0	0	0	0	1	5.70	5.66	5.96	5.63	6.23	1	0	0	0	0	1	0	8.51	8.63	8.74	8.86	8.97	9.19	8.63
1373601_at	---	1	0	0	0	1	0	5.11	5.07	4.96	5.55	5.29	1	0	0	0	0	1	1	7.39	7.36	7.62	7.64	7.44	6.70	6.33
1373610_at	Sec24d	1	0	0	0	1	1	9.45	9.43	9.90	9.10	10.03	1	0	0	0	0	0	1	10.84	10.85	10.86	10.82	10.83	10.42	9.98
1373624_at	Rassf8	1	0	0	0	1	0	7.33	7.34	7.87	7.09	7.73	1	0	0	0	0	0	1	8.89	8.90	8.92	8.78	8.93	9.30	9.10
1373657_at	Slc31a2	1	0	1	0	1	1	7.06	7.16	7.77	6.98	7.76	1	0	0	0	0	1	1	7.62	7.60	7.78	7.90	7.92	8.42	8.22
1373658_at	Racgap1	1	0	0	0	0	1	7.22	7.28	7.65	7.51	7.87	1	0	0	0	0	1	1	11.66	11.58	11.64	11.62	11.51	10.94	10.92
1373662_at	Tor2a	1	0	0	0	1	1	7.67	7.31	7.35	7.99	6.90	1	0	0	0	0	0	1	8.98	9.04	9.00	8.88	8.95	8.56	8.17
1373666_at	Rapgef5	1	0	0	0	0	1	5.61	5.42	6.12	5.95	5.06	1	0	0	0	0	1	1	6.75	6.78	6.89	6.89	7.02	7.44	7.66
1373685_at	Ankrd37	1	1	0	0	0	0	5.98	6.62	6.47	5.85	6.38	1	0	0	0	0	1	1	9.28	9.26	9.14	8.89	8.74	8.14	8.14
1373732_at	Acp6	1	0	0	0	0	1	6.09	5.89	6.05	6.25	5.62	1	0	0	0	1	1	1	6.95	7.12	7.23	7.46	7.54	8.07	8.04
1373748_at	Pdzrn3	1	0	0	1	1	0	8.36	8.29	8.25	8.87	7.96	1	0	0	0	0	1	1	6.87	7.01	7.09	7.24	7.37	8.38	8.56
1373754_at	Golm1	1	0	0	0	0	1	7.57	7.69	7.78	7.28	8.10	1	0	0	0	0	1	1	8.46	8.51	8.67	8.61	8.79	9.21	9.10
1373774_at	Rit2	1	0	0	0	0	1	5.86	6.24	5.89	5.61	6.72	1	0	0	0	0	1	0	9.53	9.60	9.50	9.50	9.42	8.92	8.15
1373777_at	Rgs16	1	0	0	0	1	1	7.29	7.53	7.70	6.87	7.81	1	0	0	0	0	1	0	9.45	9.49	9.57	9.61	9.78	10.30	9.96
1373788_at	Slc41a1	1	0	1	0	1	1	8.52	8.52	7.77	8.96	8.00	1	0	0	0	0	1	0	7.94	7.88	7.73	7.45	7.42	7.09	7.46
1373818_at	Kctd12	1	0	0	0	0	1	7.78	8.00	8.17	7.72	8.26	1	0	0	0	0	0	1	7.53	7.45	7.41	7.47	7.19	7.57	8.25
1373823_at	Cks2	1	0	1	0	0	1	5.79	6.00	6.37	6.15	6.70	1	0	0	0	0	1	1	10.42	10.36	10.31	10.30	10.11	9.15	9.10
1373838_at	Fut4	1	0	0	0	0	1	6.50	6.33	6.54	6.53	6.02	1	0	0	0	0	1	1	10.29	10.31	10.25	10.20	10.21	9.65	9.08
1373839_at	Igdcc4	1	0	0	0	0	1	5.36	5.58	5.40	5.28	5.91	1	0	0	0	0	0	1	6.27	6.43	6.54	6.55	6.63	6.73	6.62
1373850_at	Smpdl3b	1	0	0	1	1	0	7.59	7.52	7.14	8.30	7.29	1	0	0	0	0	1	1	6.28	6.44	6.40	6.56	6.62	7.39	7.78
1373860_at	Sox4	1	0	0	1	1	0	7.17	7.25	7.24	7.83	7.07	1	0	0	0	0	1	1	10.11	10.05	10.22	10.18	10.24	10.82	11.02
1373897_at	LMNB1	1	0	0	1	0	0	6.39	6.77	6.75	7.17	6.62	1	0	0	0	0	1	1	10.77	10.65	10.74	10.39	10.58	10.11	10.04
1373904_at	Lysmd2	1	0	1	0	1	1	6.89	6.99	7.82	6.48	7.49	1	0	0	0	0	1	1	8.55	8.57	8.65	8.64	8.50	7.95	7.22
1373922_at	Tanc1	1	0	0	0	1	0	6.02	5.77	5.84	6.42	6.03	1	0	0	0	0	1	1	5.33	5.42	5.62	5.49	5.59	6.01	6.38
1373944_at	---	1	0	0	1	1	0	6.51	6.54	7.02	6.02	6.34	1	0	0	0	0	1	1	7.34	7.37	7.64	7.71	7.90	8.18	7.51
1373967_at	Sub1	1	0	1	0	1	1	8.19	8.41	8.83	7.85	8.72	1	0	0	0	0	1	0	9.73	9.91	9.95	10.11	10.28	10.55	9.91
1373970_at	Il33	1	0	1	1	0	0	4.78	4.60	3.93	4.25	4.67	1	0	0	0	0	0	1	3.95	3.75	3.95	3.83	3.93	4.28	4.85
1374006_at	Kyat3	1	0	1	0	1	1	7.16	6.93	6.62	7.23	6.54	1	0	0	0	0	0	1	5.63	5.60	5.53	5.42	5.58	5.98	6.32
1374033_at	Psmb10	1	0	1	1	1	0	9.44	9.66	8.45	9.93	9.14	1	0	0	0	0	1	1	7.76	7.64	7.67	7.77	7.53	6.93	6.34
1374049_at	---	1	0	0	1	0	0	5.59	5.55	5.65	6.17	5.89	1	0	0	0	0	0	1	7.98	8.14	8.28	8.29	8.28	7.85	7.17
1374126_at	Gpr137b	1	0	1	1	1	1	5.53	5.37	6.17	4.94	5.04	1	0	0	0	0	1	1	7.48	7.59	7.67	7.83	7.79	8.44	8.68
1374151_at	---	1	0	1	0	0	0	7.50	7.57	8.10	7.69	7.73	1	0	0	0	0	0	1	9.52	9.56	9.63	9.72	9.62	9.04	8.49
1374227_at	Ttc27	1	0	0	0	0	1	7.07	6.96	6.76	7.00	6.49	1	0	0	0	0	0	1	7.95	8.02	8.16	8.30	8.10	7.76	7.22
1374283_at	Bptf	1	1	1	0	1	1	6.22	6.50	7.44	6.62	6.97	1	0	0	0	0	1	0	8.36	8.18	8.27	8.28	8.11	7.76	7.39
1374329_at	Mcf2l	1	0	0	0	0	1	8.51	8.54	8.90	8.35	9.26	1	0	0	0	0	1	1	10.15	10.17	10.19	10.12	10.10	9.28	8.86
1374368_at	Tmem164	1	0	1	0	1	0	7.27	7.43	8.05	7.28	7.60	1	0	0	0	0	0	1	9.62	9.62	9.65	9.68	9.77	10.10	10.40
1374376_at	Arhgef28	1	0	0	1	1	0	7.28	7.30	6.87	7.82	7.06	1	0	0	0	0	1	0	6.37	6.43	6.59	6.63	6.74	7.01	6.97
1374389_at	Gucy1b1	1	0	1	0	1	0	5.24	5.35	5.89	5.03	5.41	1	0	0	0	0	1	1	8.24	8.31	8.24	8.39	8.56	8.86	8.79
1374448_at	Reck	1	0	1	0	1	0	7.52	7.48	8.22	7.06	7.76	1	0	0	0	0	1	1	8.21	8.26	8.39	8.45	8.66	9.50	9.25
1374454_at	Pcmtd2	1	0	1	0	1	1	8.00	7.93	8.67	7.93	8.53	1	0	0	0	0	1	0	9.19	9.29	9.40	9.45	9.63	9.82	9.12
1374455_at	Rnaseh2c	1	0	0	0	1	0	6.18	6.22	5.67	6.39	5.80	1	0	0	0	0	1	1	8.88	8.84	8.84	8.78	8.58	8.08	8.33
1374493_at	Bmf	1	0	0	1	1	0	6.04	5.91	5.58	6.67	5.72	1	0	0	0	0	1	1	6.56	6.69	6.57	6.45	6.73	7.97	8.33
1374523_at	Pgls	1	0	0	0	1	1	7.00	6.90	6.54	7.37	6.51	1	0	0	0	0	0	1	9.77	9.73	9.73	9.80	9.67	9.35	9.00
1374524_at	Scly	1	0	0	1	1	0	7.44	7.52	7.11	8.15	7.34	1	0	0	0	0	1	0	6.03	6.25	6.20	6.58	6.34	6.63	6.48
1374529_at	---	1	0	0	1	1	0	7.83	7.78	8.36	7.16	7.82	1	0	1	1	1	1	1	5.80	5.99	6.44	6.77	6.77	7.66	8.28
1374550_at	Mknk1	1	0	0	0	0	1	8.16	7.86	7.82	8.32	7.58	1	0	0	0	0	0	1	7.42	7.41	7.43	7.48	7.45	7.85	7.96
1374560_at	Prrc1	1	0	0	0	1	0	7.10	7.04	6.76	7.32	7.24	1	0	0	0	0	1	1	8.42	8.38	8.38	8.25	8.14	7.78	7.45
1374586_at	Arl15	1	0	0	0	0	1	6.46	6.57	6.71	6.45	7.27	1	0	0	0	0	1	1	7.52	7.54	7.67	7.75	7.74	8.15	8.49
1374616_at	Pdgfrl	1	0	1	0	1	1	7.61	7.57	8.42	7.29	5.75	1	0	0	0	0	0	1	9.97	10.10	10.09	10.10	10.25	9.65	8.55
1374628_at	Cryz	1	0	0	0	0	1	6.08	5.82	6.04	6.17	5.59	1	0	0	0	0	1	1	6.35	6.32	6.59	6.51	6.83	7.62	7.43
1374657_at	Anks4b	1	0	0	0	0	1	6.59	6.84	7.14	6.60	7.18	1	0	0	0	0	1	1	8.03	8.00	8.05	8.02	7.71	7.32	7.26
1374760_at	---	1	1	1	0	1	1	4.81	4.28	5.51	4.79	5.31	1	0	0	0	0	0	1	3.63	3.55	3.55	3.52	3.71	3.98	4.30
1374775_at	Mki67	1	0	0	1	1	0	6.71	6.83	6.78	7.37	6.93	1	0	0	0	0	0	1	10.96	10.99	11.03	11.06	10.96	10.41	10.41
1374794_at	Kif15	1	0	1	0	0	0	4.25	4.26	4.89	4.67	4.67	1	0	0	0	0	1	1	7.93	7.99	8.01	8.08	7.93	7.19	7.13
1374816_at	Myzap	1	0	1	0	0	0	6.21	5.97	5.58	5.79	5.87	1	0	0	0	0	0	1	6.01	6.12	6.13	6.10	6.02	6.37	6.33
1374857_at	Gar1	1	0	0	0	0	1	6.77	6.51	6.64	6.96	6.12	1	0	0	0	0	0	1	9.10	9.03	9.16	9.22	9.13	8.59	8.00
1374874_at	Cc2d2a	1	0	1	0	1	0	6.64	6.60	7.44	6.29	6.95	1	0	0	0	0	0	1	6.74	6.70	6.86	6.89	6.77	6.19	5.77
1374899_at	Tmem255b	1	0	0	0	0	1	6.11	6.15	5.79	6.21	7.21	1	0	0	0	0	1	1	7.99	8.04	7.98	7.73	7.96	7.33	6.51
1374903_at	Gcnt2	1	0	0	0	1	0	7.73	7.68	8.07	7.31	7.43	1	0	0	0	0	0	1	8.52	8.40	8.48	8.36	8.31	8.00	8.27
1374932_at	Angptl8	1	0	1	0	0	1	6.19	6.29	5.62	6.09	5.11	1	0	0	0	0	1	1	10.66	10.68	10.55	10.44	10.33	8.94	8.20
1374933_at	Mcam	1	0	0	1	0	0	7.15	6.70	6.98	6.46	6.96	1	0	0	0	0	0	1	4.50	4.61	4.59	4.47	4.79	5.07	5.22
1374943_at	Fam210b	1	0	0	1	1	0	7.31	7.37	7.44	9.14	7.33	1	0	0	0	0	1	1	7.67	7.66	7.68	7.69	7.73	8.51	8.24
1374953_at	Tstd3	1	0	0	0	0	1	6.13	6.23	6.40	6.00	6.63	1	0	0	0	1	1	1	7.55	7.75	7.88	8.13	8.22	8.55	8.05
1374963_s_at	LOC102556459	1	0	0	0	0	1	3.55	3.73	3.95	3.87	4.76	1	0	1	1	1	1	1	6.11	6.66	6.82	6.87	7.30	7.71	6.57
1374969_at	---	1	0	1	0	1	0	9.62	9.65	10.40	9.20	9.50	1	0	0	1	1	1	1	7.89	8.12	8.25	8.55	8.67	9.58	9.30
1375052_at	C2cd4b	1	1	0	1	1	1	8.82	9.60	9.10	9.87	8.34	1	0	0	0	0	0	1	11.40	11.47	11.45	11.16	11.37	10.94	9.73
1375088_at	---	1	0	0	0	0	1	5.27	5.51	5.27	5.35	5.75	1	0	0	0	0	1	0	6.37	6.47	6.77	6.85	6.93	7.02	6.16
1375213_at	Pck2	1	0	0	1	1	0	9.61	9.38	9.34	10.12	9.16	1	0	0	0	0	1	1	8.92	8.85	8.99	9.06	8.97	8.25	7.75
1375223_at	---	1	0	0	0	0	1	5.80	6.04	6.01	5.80	6.79	1	0	0	0	0	0	1	7.30	7.20	7.43	7.30	7.22	6.74	6.85
1375247_at	Mgll	1	0	0	0	1	0	5.91	5.67	5.50	6.16	5.46	1	0	0	0	0	1	1	4.72	4.74	4.83	4.57	4.66	5.77	7.01
1375278_at	Trim2	1	0	1	0	1	0	6.77	6.69	6.24	6.84	6.35	1	0	0	0	0	1	1	7.99	8.02	8.20	8.33	8.29	8.64	8.49
1375305_at	Snhg11	1	0	1	0	1	0	9.83	10.04	10.47	9.45	9.97	1	0	0	0	1	1	1	5.70	6.09	6.09	6.23	6.35	6.94	7.15
1375343_at	---	1	0	1	1	1	0	7.43	7.34	8.08	6.44	7.39	1	0	0	1	0	1	0	7.83	7.91	8.21	8.42	8.27	8.44	7.87
1375377_at	Igsf3	1	0	0	1	1	0	5.70	5.51	5.79	6.37	5.88	1	0	0	0	0	1	1	8.04	8.06	8.10	8.14	8.23	8.82	8.97
1375430_at	Mmgt2	1	0	0	1	1	0	7.43	7.57	7.32	7.91	7.44	1	0	0	0	0	0	1	7.71	7.73	7.80	7.93	7.91	8.22	8.29
1375475_at	---	1	1	0	0	0	0	3.74	4.29	3.83	3.58	3.72	1	0	0	0	0	1	0	5.55	5.45	5.76	5.50	5.82	6.67	5.28
1375495_at	Cdkl4	1	1	1	1	0	0	4.74	5.31	5.47	5.29	5.12	1	0	0	0	0	1	1	4.33	4.26	4.23	4.37	4.53	5.61	6.24
1375531_at	Lrig3	1	0	0	0	1	0	4.18	4.18	4.00	4.63	3.93	1	0	0	0	0	1	0	4.92	5.07	4.93	4.99	4.98	5.59	5.34
1375532_at	Id2	1	0	0	1	0	0	7.66	7.82	7.87	8.15	7.71	1	0	0	1	1	0	1	8.01	7.74	7.71	7.08	7.08	7.69	10.00
1375643_at	Ckap4	1	0	0	0	1	0	6.38	6.62	6.15	6.76	6.61	1	0	0	0	0	1	1	8.33	8.33	8.31	8.24	8.15	7.71	7.62
1375654_at	Ckap4	1	0	0	1	0	1	5.99	6.46	6.27	6.68	6.67	1	0	0	0	0	1	1	10.28	10.13	10.21	10.08	9.97	9.57	9.41
1375662_at	Smim10l1	1	0	1	0	0	0	9.08	8.98	9.67	9.01	9.30	1	0	0	0	0	0	1	10.36	10.31	10.34	10.38	10.30	9.79	9.32
1375726_at	Lmo7	1	0	0	1	0	0	6.93	6.75	7.09	7.48	6.90	1	0	0	0	0	1	0	8.20	8.05	7.99	7.80	7.67	7.56	7.77
1375846_at	Xpr1	1	0	1	0	1	1	5.83	6.18	6.72	5.89	6.34	1	0	0	0	0	1	1	6.92	6.88	6.96	7.15	7.03	7.57	7.70
1375855_at	Epdr1	1	0	0	1	1	0	7.62	7.67	8.10	7.08	7.71	1	0	0	0	0	1	1	8.79	8.83	8.81	8.84	8.75	7.88	7.46
1375856_at	---	1	0	1	0	0	1	7.56	7.77	8.13	7.54	8.24	1	0	0	0	0	1	1	5.12	5.03	5.48	5.47	5.51	5.83	6.58
1375877_at	Syt4	1	0	0	0	0	1	7.62	8.07	8.16	7.71	8.70	1	0	0	0	0	0	1	11.03	11.01	10.95	10.83	10.79	10.51	9.66
1375885_at	Mtmr1	1	0	0	0	0	1	4.45	4.20	4.60	4.14	3.93	1	0	0	0	0	0	1	6.57	6.48	6.76	6.74	6.56	6.27	4.75
1375886_at	Ipp	1	0	1	0	1	1	6.45	6.54	7.22	6.35	6.96	1	0	0	0	0	0	1	9.20	9.25	9.33	9.39	9.42	9.76	9.71
1375944_at	Acss2	1	0	0	0	0	1	6.39	6.80	6.90	6.50	7.10	1	0	0	0	0	1	1	9.76	9.87	9.86	9.91	9.98	10.49	10.70
1376042_at	Tsr2	1	0	0	0	1	0	5.61	5.56	6.12	5.42	5.96	1	0	0	0	0	1	0	7.07	7.08	7.32	7.48	7.59	7.85	7.81
1376052_at	Tmem160	1	0	1	0	1	1	8.79	8.68	8.15	9.00	8.00	1	0	0	0	0	1	0	9.39	9.46	9.43	9.42	9.50	9.97	9.92
1376098_a_at	LOC102547700	1	0	0	1	1	0	8.37	8.27	8.10	8.99	8.38	1	0	0	0	0	1	1	9.00	8.98	9.02	9.17	9.06	8.22	7.73
1376129_at	---	1	0	1	0	1	0	6.62	6.72	7.36	6.16	6.76	1	0	0	0	0	1	1	7.66	7.59	7.80	7.68	7.76	8.29	7.51
1376174_at	Serpina11	1	0	0	0	1	0	5.27	4.95	4.78	5.57	5.01	1	0	0	0	1	1	1	5.59	5.59	5.93	6.08	6.44	7.24	7.12
1376178_at	---	1	1	1	1	0	1	6.27	7.08	5.69	5.09	5.05	1	0	0	0	0	0	1	7.65	7.61	7.84	7.50	7.58	7.20	5.94
1376187_at	Slc35d1	1	0	0	0	0	1	5.52	5.60	5.90	5.57	6.01	1	0	0	0	0	1	1	9.35	9.34	9.28	9.17	9.16	8.29	7.54
1376191_at	Hpgd	1	0	1	0	1	0	6.93	7.03	8.25	6.96	7.36	1	0	0	0	0	1	1	5.38	5.32	5.46	5.63	5.79	6.74	6.40
1376197_at	Tcf7	1	0	0	1	1	0	5.07	5.32	4.84	5.93	5.41	1	0	0	0	0	1	1	3.60	3.64	3.70	3.75	3.74	4.27	5.06
1376200_at	Etfbkmt	1	0	0	0	0	1	6.03	5.75	6.37	6.43	4.58	1	0	0	0	0	0	1	3.60	3.57	3.76	3.73	3.63	3.85	3.74
1376240_at	Kat6b	1	0	0	0	1	0	6.47	6.48	6.08	6.75	6.35	1	0	0	0	0	1	0	6.71	6.64	6.69	6.55	6.37	5.99	6.22
1376247_at	Pck2	1	0	0	0	0	1	7.80	7.73	7.56	8.10	7.32	1	0	0	0	0	1	1	8.49	8.50	8.56	8.62	8.51	7.80	7.17
1376267_at	Slc16a6	1	0	1	0	0	0	5.05	4.96	4.42	4.93	5.03	1	0	0	0	0	0	1	4.86	4.98	5.13	4.95	5.08	5.43	6.52
1376283_at	Gad2	1	0	0	0	1	0	9.06	9.22	9.55	8.75	9.14	1	0	0	0	0	0	1	6.95	6.98	7.16	7.35	7.21	7.36	8.07
1376292_at	---	1	1	0	0	1	0	6.44	5.93	5.96	6.77	6.16	1	0	0	0	0	1	1	6.65	6.64	6.65	6.55	6.46	5.48	5.67
1376486_at	Sh3glb2	1	0	0	0	0	1	5.69	5.84	5.44	5.97	4.73	1	0	0	0	0	1	0	7.95	7.94	8.08	8.09	8.06	8.53	8.34
1376533_at	Baz2b	1	0	1	0	0	0	6.01	6.05	6.66	6.34	6.37	1	0	0	0	0	1	1	7.55	7.42	7.57	7.42	7.40	6.75	6.77
1376577_at	---	1	0	1	0	0	1	7.43	7.82	8.19	7.53	8.81	1	0	0	0	0	1	1	8.61	8.66	8.67	8.78	8.91	9.21	8.74

Probeset ID	Gene Symbol	Islet Data Set (log2 ratio ≥0.263; FDR <20%) union n=5325 1=YES 0=NO	Islet DRlyp/lyp ND vs ND+Lp299v (log2 ratio ≥0.263; FDR<20%) n=429 1=YES 0=NO	Islet DRlyp/lyp ND vs HCD (log2 ratio ≥0.263; FDR<20%) n=2247 1=YES 0=NO	Islet DRlyp/lyp ND vs HCD+Lp299v (log2 ratio ≥0.263; FDR<20%) n=1595 1=YES 0=NO	Islet DRlyp/lyp HCD vs HCD+Lp299v (log2 ratio ≥0.263; FDR<20%) n=3026 1=YES 0=NO	Islet DRlyp/lyp ND vs Flyp/lyp ND (log2 ratio ≥ 0.263; FDR<20%) n=1950 1=YES 0=NO	Islet Mean Log2 Intensity DRlyp/lyp ND	Islet Mean Log2 Intensity DRlyp/lyp ND+Lp299v	Islet Mean Log2 Intensity DRlyp/lyp HCD	Islet Mean Log2 Intensity DRlyp/lyp HCD+Lp299v	Islet Mean Log2 Intensity Flyp40 ND	Rinm5f data union of all conditions n=3705 1=YES 0=NO	Rinm5f 30 µM propionate/ 6 µM butyrate vs Media (FDR<20%; 1.5 fold) n=3 1=YES 0=NO	Rinm5f 60 µM propionate/ 12 µM butyrate vs Media (FDR<20%; 1.5 fold) n=18 1=YES 0=NO	Rinm5f 120 µM propionate/ 24 µM butyrate vs Media (FDR<20%; 1.5 fold) n=164 1=YES 0=NO	Rinm5f 240 µM propionate /48 µM butyrate vs Media (FDR<20%; 1.5 fold) n=268 1=YES 0=NO	Rinm5f 1500 µM propionate/ 300 µM butyrate vs Media (FDR<20%; 1.5 fold) n=2491 1=YES 0=NO	Rinm5f 500 µM propionate/ 500 µM butyrate vs Media (FDR<20%; 1.5 fold) n=3001 1=YES 0=NO	Rinm5f Mean Log2 Intensity Media only	Rinm5f Mean Log2 Intensity 30 µM propionate/ 6 µM butyrate	Rinm5f Mean Log2 Intensity 60 µM propionate/ 12 µM butyrate	Rinm5f Mean Log2 Intensity 120 µM propionate/ 24 µM butyrate	Rinm5f Mean Log2 Intensity 240 µM propionate/ 48 µM butyrate	Rinm5f Mean Log2 Intensity 1500 µM propionate /300 µM butyrate	Rinm5f Mean Log2 Intensity 500 µM propionate/ 500 µM butyrate
1376581_at	Dtd2	1	0	0	1	0	0	5.34	5.47	5.77	5.82	5.42	1	0	0	0	0	0	1	7.03	6.97	7.28	7.30	7.34	7.60	7.71
1376584_at	Ppm1l	1	0	0	0	0	1	7.23	7.09	7.22	6.96	8.07	1	0	0	0	0	0	1	9.02	9.06	9.14	9.17	9.22	9.56	9.62
1376612_at	Dtwd1	1	1	0	0	0	0	5.10	4.62	5.41	5.25	5.16	1	0	0	0	0	0	1	7.34	7.37	7.46	7.44	7.34	6.86	6.24
1376654_at	Plcxd3	1	0	0	0	0	1	5.75	6.07	6.26	5.70	6.45	1	0	0	0	0	0	1	11.31	11.43	11.30	11.11	11.22	10.73	10.09
1376657_at	Cadm1	1	0	0	0	0	1	6.67	6.85	6.80	6.93	7.21	1	0	0	0	0	1	0	8.42	8.56	8.64	8.82	8.90	9.07	8.80
1376673_at	Zeb1	1	0	1	0	1	0	6.16	6.18	6.75	5.85	6.31	1	0	0	0	0	1	0	6.42	6.47	6.72	6.72	6.88	7.04	7.37
1376708_at	Fam115c /// LOC	1	0	0	0	0	1	4.33	4.33	4.46	4.52	4.93	1	0	0	0	1	1	1	6.98	6.87	7.32	7.37	7.62	9.21	9.11
1376723_a_at	LOC103691688	1	0	1	1	0	0	8.63	8.48	9.28	9.80	8.18	1	0	0	0	0	0	1	11.32	11.30	11.26	11.32	11.26	10.94	10.27
1376726_at	Car10	1	0	1	0	0	0	5.99	6.14	6.59	6.15	6.09	1	0	0	0	0	0	1	9.46	9.53	9.47	9.22	9.41	9.06	8.52
1376733_at	Igsf11	1	0	1	0	0	0	8.48	8.52	9.15	8.62	8.94	1	0	0	0	0	1	1	4.28	4.34	4.45	4.39	4.40	5.45	5.77
1376734_at	Ccn3	1	0	0	1	0	0	5.24	5.02	4.98	4.51	5.07	1	0	0	0	0	0	1	9.63	9.75	9.70	9.85	10.03	9.65	8.75
1376747_at	---	1	0	0	1	1	0	3.84	3.94	3.88	4.72	3.96	1	0	0	0	0	1	1	4.50	4.60	4.68	4.63	4.82	5.46	6.04
1376754_at	Cars	1	0	0	0	0	1	7.83	7.68	7.36	7.86	7.27	1	0	0	0	0	0	1	8.53	8.53	8.61	8.61	8.56	8.09	7.34
1376782_at	Cdc14a	1	0	1	0	1	0	5.98	5.91	6.82	5.54	6.10	1	0	0	0	0	1	0	6.38	6.40	6.53	6.55	6.59	7.00	7.31
1376829_at	Unc5d	1	0	1	1	1	0	5.02	4.92	4.39	5.60	4.78	1	0	0	0	0	1	1	3.91	3.80	4.02	3.95	3.85	5.25	5.83
1376842_at	Dgkh	1	0	1	0	1	1	4.42	4.71	5.36	4.41	5.09	1	0	0	0	0	1	0	7.10	7.03	6.98	6.95	6.84	6.43	5.91
1376872_at	Plxnc1	1	0	1	0	1	1	4.94	5.19	5.59	4.89	6.06	1	0	0	0	0	1	1	6.03	6.26	6.41	6.56	6.48	6.95	6.57
1376891_at	---	1	0	0	0	0	1	6.18	6.26	6.58	6.36	6.72	1	0	0	1	1	1	1	9.23	9.33	9.67	9.98	10.08	10.37	10.47
1376893_at	Nrsn1	1	0	0	0	0	1	5.75	5.99	6.24	5.67	6.56	1	0	0	0	0	1	0	9.87	10.00	10.06	10.12	10.24	10.60	10.75
1376944_at	---	1	1	1	0	1	1	8.22	8.81	9.37	8.22	9.21	1	0	0	0	0	0	1	9.85	9.90	9.86	9.98	9.98	9.39	8.35
1376966_at	---	1	0	1	0	0	0	6.97	6.99	7.61	7.16	7.14	1	0	0	0	0	0	1	11.45	11.49	11.50	11.41	11.56	11.06	9.93
1376977_at	Ptger3	1	1	1	0	1	1	10.45	9.84	9.71	10.64	9.80	1	0	0	0	0	0	1	9.74	9.90	9.70	9.54	9.72	9.76	8.74
1377032_at	---	1	0	0	1	1	0	4.92	5.16	4.65	5.45	4.56	1	0	0	0	0	0	1	5.15	5.32	5.29	4.93	5.08	5.44	5.74
1377034_at	Serpinb1	1	0	0	0	0	1	11.69	11.35	11.51	11.85	10.85	1	0	0	0	0	1	1	4.47	4.53	4.33	4.40	4.39	6.35	6.75
1377046_at	Ankrd6	1	0	1	0	1	0	4.05	4.21	4.77	4.06	4.12	1	0	0	0	0	1	1	4.87	4.92	4.89	5.20	5.23	5.69	6.52
1377154_at	Znf613	1	0	1	0	0	0	5.60	6.01	6.20	5.76	5.82	1	0	0	0	1	0	0	6.90	7.10	7.26	7.44	7.50	7.39	7.20
1377155_at	LOC102549803 /	1	0	0	0	1	0	7.53	7.39	7.30	7.88	7.28	1	0	0	0	0	1	1	7.33	7.26	7.45	7.55	7.42	6.72	6.64
1377156_at	Tcf7l2	1	0	0	1	1	1	6.21	6.08	6.12	6.88	5.62	1	0	0	0	0	0	1	8.30	8.37	8.33	8.16	8.39	8.84	8.40
1377351_at	Susd3	1	1	1	1	1	1	7.81	6.90	6.59	7.19	6.67	1	0	0	0	0	1	1	5.51	5.52	5.43	5.46	5.45	6.46	6.39
1377356_at	Gdpd5	1	0	1	1	1	1	5.97	5.82	5.22	6.76	5.46	1	0	0	0	0	1	1	6.69	6.85	6.95	6.89	7.15	7.79	7.76
1377422_at	---	1	0	0	0	0	1	6.28	5.95	5.89	6.27	5.11	1	0	0	0	0	1	1	6.56	6.36	6.49	6.60	6.37	5.72	4.89
1377518_at	Camk1g	1	0	0	0	1	0	4.82	5.07	5.37	4.38	4.90	1	0	0	0	0	1	1	5.11	5.23	5.48	5.49	5.56	6.15	5.29
1377573_at	Car5b	1	0	0	1	0	0	7.26	7.18	7.31	6.74	7.14	1	0	0	0	1	1	1	5.39	5.86	5.58	5.95	6.06	7.83	8.15
1377636_at	---	1	0	0	1	1	0	5.38	5.55	5.41	5.99	5.65	1	0	0	0	0	0	1	6.64	6.39	6.81	6.83	6.81	7.20	8.06
1377644_at	C16orf87	1	0	1	0	1	0	7.03	6.84	7.65	6.82	7.14	1	0	0	0	0	1	1	7.03	7.10	7.03	7.23	7.26	8.37	8.42
1377650_at	Ogfrl1	1	0	0	0	0	1	3.07	2.93	3.40	3.02	3.57	1	0	0	0	0	1	0	6.25	6.31	6.41	6.62	6.55	7.00	6.68
1377655_at	Fgf12	1	0	0	0	1	1	3.35	3.04	2.90	3.50	2.87	1	0	0	0	0	1	1	5.79	5.79	5.93	6.00	6.00	6.64	6.99
1377686_at	---	1	0	0	1	1	1	7.80	7.39	7.29	6.59	4.58	1	0	0	0	0	1	0	3.91	4.03	4.15	4.07	4.04	4.82	4.31
1377739_at	Gng12	1	0	0	0	1	0	6.61	6.62	6.92	6.20	6.74	1	0	0	0	0	0	1	7.76	7.72	7.80	7.97	7.86	8.31	8.61
1377750_at	Arhgef3	1	0	1	0	1	0	6.67	6.58	7.26	6.41	6.47	1	0	0	0	0	0	1	8.38	8.43	8.38	8.19	8.33	8.77	9.25
1377773_at	Gabrb2	1	0	0	0	1	1	5.88	5.79	5.49	6.35	4.82	1	0	0	0	0	0	1	4.13	3.93	4.04	3.91	3.92	4.57	4.53
1377774_at	---	1	0	0	0	1	0	10.09	10.05	9.98	10.55	9.83	1	0	0	0	0	1	1	8.34	8.40	8.48	8.59	8.71	9.19	8.59
1377785_at	Dhx40	1	0	0	1	1	0	7.21	7.17	7.49	6.66	7.20	1	0	0	0	0	1	1	8.50	8.59	8.70	8.83	8.85	9.26	9.19
1377818_at	---	1	0	0	1	1	0	7.72	7.58	7.44	8.43	7.48	1	0	0	0	0	1	0	9.37	9.44	9.56	9.72	9.77	10.07	10.00
1377831_at	---	1	0	1	0	0	0	7.32	7.48	7.95	7.42	7.79	1	0	0	0	0	1	1	8.71	8.71	8.82	8.95	8.94	9.41	9.86
1377835_at	Dock8	1	0	0	0	1	1	8.43	8.62	8.05	8.82	7.97	1	0	0	0	0	0	1	6.77	6.70	6.69	6.72	6.77	7.33	8.15
1377879_at	---	1	1	0	0	1	0	5.75	5.28	5.50	6.14	5.52	1	0	0	0	0	0	1	4.86	4.90	5.01	4.88	4.85	4.98	5.80
1377880_at	LOC683508	1	0	1	0	1	0	5.15	5.02	5.84	5.10	5.49	1	0	0	0	0	0	1	7.12	7.08	7.25	7.19	7.10	6.63	6.25
1377916_at	Slfn2	1	0	1	0	0	0	5.37	5.52	4.78	4.94	5.31	1	0	0	0	0	1	1	8.28	8.34	8.35	8.22	8.14	7.66	7.18
1377940_at	Rflnb	1	0	0	0	1	0	7.16	6.90	6.69	7.45	6.78	1	0	0	0	0	1	1	7.58	7.66	7.69	7.73	7.76	8.34	8.32
1377970_at	Parp8	1	0	1	0	1	1	6.38	6.73	7.29	6.15	7.26	1	0	0	0	0	0	1	8.01	8.09	8.07	8.05	8.11	7.62	6.80
1378027_at	Nectin3	1	0	1	0	1	0	8.06	8.18	8.92	7.58	8.43	1	0	0	0	1	1	1	10.03	9.92	9.79	9.60	9.42	9.11	8.92
1378030_at	---	1	0	1	0	0	0	4.49	4.85	5.14	4.54	4.76	1	0	0	0	0	1	1	6.99	7.12	7.44	7.39	7.54	7.90	7.44
1378032_at	Nfkbiz	1	1	0	1	0	1	7.59	8.95	7.41	6.79	8.29	1	0	0	0	0	1	0	8.91	9.00	8.91	8.81	8.90	9.57	8.88
1378074_at	Pdk4	1	1	1	1	1	0	8.51	7.94	9.08	7.65	8.34	1	0	0	0	0	1	1	4.03	4.06	4.21	4.09	4.49	5.19	5.94
1378107_at	Rab27b	1	0	0	1	1	1	7.11	7.10	6.96	7.82	7.61	1	0	0	0	0	0	1	6.15	5.94	6.10	6.08	6.11	6.53	7.21
1378129_a_at	LOC102553824	1	0	0	1	0	0	5.32	5.58	5.53	6.08	5.33	1	0	0	0	0	0	1	7.38	7.30	7.50	7.55	7.40	6.91	6.94
1378133_at	Slc7a11	1	1	0	1	0	0	6.69	5.73	6.26	5.92	6.37	1	0	0	0	0	0	1	6.81	6.78	6.95	7.05	7.01	6.29	5.89
1378161_at	---	1	0	1	0	1	0	4.87	5.04	5.57	4.51	5.03	1	0	0	0	0	1	1	6.75	6.96	7.12	7.08	7.20	7.39	7.27
1378241_at	Lrrc75b	1	0	0	1	1	1	5.87	5.73	5.65	6.92	5.37	1	0	0	0	0	1	1	7.42	7.36	7.52	7.42	7.37	6.81	6.75
1378283_at	RGD1562465	1	0	0	1	0	0	4.15	4.11	4.49	4.74	4.13	1	0	0	0	0	1	1	7.79	7.85	7.95	7.92	7.86	6.75	6.35
1378292_at	---	1	0	1	0	1	0	6.53	6.52	7.12	6.24	6.77	1	0	0	0	0	0	1	9.30	9.39	9.39	9.48	9.60	9.66	9.24
1378315_at	---	1	0	1	0	1	1	5.41	5.11	6.28	5.53	6.03	1	0	0	0	0	0	1	3.84	3.81	3.86	3.75	3.81	4.34	4.79
1378323_at	Nol4	1	0	1	0	1	1	6.35	6.51	6.95	6.10	7.18	1	0	0	0	0	1	0	8.06	8.00	8.19	8.30	8.30	8.67	8.65
1378325_at	---	1	0	1	0	1	0	6.40	6.38	7.09	6.02	6.64	1	0	0	0	0	1	0	8.29	8.00	8.00	7.79	7.91	7.56	7.06
1378408_at	Pcdh15	1	0	0	0	0	1	6.04	6.37	6.35	6.06	6.91	1	0	0	0	0	1	1	8.12	8.01	8.08	7.80	7.84	7.15	6.92
1378413_at	---	1	1	0	1	1	0	6.66	6.13	6.48	7.62	6.21	1	0	0	0	0	0	1	4.19	4.22	4.27	4.28	4.41	4.39	5.48
1378430_at	Moxd1	1	0	1	0	0	0	6.00	6.25	6.59	6.00	6.27	1	0	0	0	0	1	1	5.99	6.03	6.17	6.15	6.25	7.03	7.82
1378472_at	Unc5d	1	0	1	1	1	0	6.04	6.01	5.44	6.67	5.93	1	0	0	0	0	1	1	4.45	4.43	4.42	4.40	4.34	5.14	6.66
1378535_at	Phf7	1	0	1	0	1	0	6.37	6.16	5.82	6.62	6.32	1	0	0	0	0	0	1	6.83	6.79	6.81	6.82	6.77	7.26	7.40
1378555_at	Lrrc28	1	0	0	0	0	1	5.53	5.44	5.46	5.90	6.55	1	0	0	0	0	1	1	5.97	5.97	6.22	6.19	6.38	6.56	6.58
1378572_at	Tollip	1	0	1	1	1	0	5.08	5.08	5.73	4.35	4.95	1	0	0	0	0	0	1	7.18	7.23	7.27	7.31	7.17	6.96	5.70
1378580_at	---	1	0	0	0	1	1	9.94	10.04	10.47	9.61	6.99	1	0	0	0	1	0	0	6.03	6.10	6.34	6.57	6.72	6.44	5.65
1378603_at	---	1	0	0	1	0	0	5.98	5.66	5.58	5.05	6.29	1	0	0	0	1	1	0	8.01	8.14	8.36	8.57	8.79	8.76	8.31
1378611_at	---	1	0	0	0	0	1	4.91	4.67	5.21	4.94	5.56	1	0	0	1	1	1	1	7.27	7.46	7.73	8.22	8.15	8.16	7.98
1378665_at	---	1	0	1	0	0	1	6.21	6.53	7.00	6.35	7.52	1	0	0	1	0	1	1	7.45	7.67	7.85	8.10	7.92	8.53	8.62
1378777_at	---	1	0	0	0	1	0	3.94	3.86	4.41	3.49	3.89	1	0	0	0	0	1	1	5.72	5.50	5.80	5.74	5.87	6.42	5.67
1378861_at	LOC103692056	1	0	0	0	0	1	6.42	6.09	6.21	6.00	5.87	1	0	0	0	1	1	0	8.62	8.71	8.90	9.13	9.38	9.41	8.75
1378942_at	---	1	0	0	1	0	0	3.63	3.79	3.69	4.10	4.04	1	0	0	0	0	0	1	5.12	5.00	4.92	5.12	4.94	5.50	5.78
1379010_at	Srsf3	1	0	0	0	1	0	4.40	4.56	4.97	4.09	4.47	1	0	0	0	0	1	0	7.15	7.06	7.32	7.59	7.45	7.91	6.22
1379046_at	Mlec	1	0	0	0	1	0	6.55	6.55	6.23	6.81	6.12	1	0	0	0	0	0	1	7.55	7.46	7.58	7.45	7.34	7.13	6.67
1379055_x_at	---	1	0	1	0	1	0	3.73	3.77	2.99	3.66	3.33	1	0	0	0	0	1	0	4.98	4.91	5.15	5.09	5.21	5.95	4.89
1379093_at	---	1	0	1	0	1	1	6.33	6.70	6.99	6.21	6.86	1	0	0	0	0	1	1	7.90	7.78	7.88	7.75	7.71	6.76	6.18
1379274_at	---	1	0	1	0	1	0	8.20	8.43	9.00	7.81	8.57	1	0	0	0	0	0	1	10.32	10.38	10.46	10.57	10.57	10.14	9.15
1379277_at	Utrn	1	0	0	0	0	1	8.17	8.44	8.68	8.12	8.92	1	0	0	0	0	1	0	8.23	8.33	8.43	8.54	8.62	8.98	8.82
1379285_at	Rtp4	1	1	0	0	1	0	5.75	6.71	5.52	6.18	5.63	1	0	0	0	1	1	1	6.43	6.68	6.77	6.87	7.02	7.37	7.19
1379311_at	Arid5a	1	0	1	0	1	0	4.83	4.79	4.25	4.85	4.54	1	0	0	0	1	1	1	6.57	6.51	6.37	6.03	5.96	5.64	5.57
1379352_at	Pip5k1b	1	0	0	0	0	1	6.74	6.82	7.19	6.96	7.31	1	0	0	0	0	0	1	8.61	8.63	8.70	8.77	8.80	9.17	9.22
1379368_at	Bcl6	1	1	0	0	0	0	4.55	3.95	4.30	4.43	4.33	1	0	0	0	0	1	1	6.41	6.25	6.28	6.01	5.95	5.57	5.60
1379440_at	Fstl3	1	0	1	0	1	0	5.93	5.87	5.24	5.96	5.54	1	0	0	0	0	1	1	9.46	9.58	9.50	9.45	9.71	10.57	10.25
1379445_at	---	1	0	1	0	1	1	5.62	5.97	6.30	5.31	6.49	1	0	0	1	1	0	0	7.54	7.83	7.92	8.14	8.12	7.90	7.96
1379464_at	---	1	0	1	0	1	0	3.74	3.54	4.36	3.55	3.44	1	0	0	0	0	1	1	4.38	4.22	4.55	4.55	4.62	6.61	7.55
1379482_at	Tm6sf1	1	0	1	0	1	0	5.63	5.74	6.19	5.15	5.42	1	0	0	0	0	1	1	6.71	6.85	7.00	7.04	7.12	7.35	6.93
1379484_at	Aven	1	0	0	0	1	0	6.98	6.84	6.55	7.22	6.61	1	0	0	0	0	1	1	7.79	7.60	7.77	7.73	7.43	6.48	6.24
1379499_at	Ltb	1	0	0	0	0	1	3.69	3.84	3.64	3.88	4.46	1	0	0	0	0	1	0	4.45	4.45	4.46	4.57	4.44	5.08	5.15
1379538_at	Cenpj	1	0	0	0	1	0	5.66	5.65	6.07	5.36	5.78	1	0	0	0	0	0	1	7.12	7.08	7.27	7.25	7.21	6.59	6.38
1379562_at	---	1	0	0	1	1	0	4.93	4.68	4.93	4.09	4.53	1	0	0	0	0	1	0	5.73	5.61	6.12	6.08	6.15	6.81	6.14
1379582_a_at	Ccna2	1	0	0	0	0	1	4.70	4.81	5.19	5.05	5.23	1	0	0	0	0	1	1	10.13	10.04	10.02	10.06	9.90	8.80	8.13
1379660_at	Mpzl3	1	0	1	0	0	1	7.19	6.88	6.55	6.94	6.46	1	0	0	0	0	0	1	5.99	5.95	6.11	6.17	6.12	5.41	5.42
1379661_at	---	1	0	1	0	0	0	4.59	4.92	5.35	4.81	4.99	1	0	0	0	1	1	0	5.71	6.04	6.14	6.24	6.31	6.59	5.99
1379663_at	---	1	0	0	1	0	1	5.71	5.69	5.96	6.20	6.19	1	0	0	0	0	1	1	4.86	4.67	4.79	4.76	4.71	5.80	7.09
1379717_at	Abcg3l2 /// Abcg	1	0	1	0	1	0	6.88	7.08	7.58	6.61	7.23	1	0	0	0	0	1	1	6.98	7.16	7.34	7.45	7.57	8.01	7.54
1379719_at	Syde2	1	0	1	0	0	0	6.25	6.10	6.86	6.25	6.30	1	0	0	0	0	1	1	8.15	8.14	8.28	8.40	8.36	8.80	8.76
1379740_at	LOC361346	1	0	1	0	1	0	2.88	2.92	3.50	2.76	2.96	1	0	0	0	0	0	1	8.57	8.61	8.63	8.84	8.86	8.36	7.14
1379770_at	Chpt1	1	0	1	0	0	0	4.26	4.46	4.88	4.61	4.59	1	0	0	0	0	1	0	6.34	6.25	6.20	6.08	5.96	5.65	5.49
1379799_at	---	1	0	1	1	0	1	5.12	5.09	5.78	6.29	4.44	1	0	0	0	0	1	1	8.24	8.40	8.42	8.31	8.52	7.30	5.65
1379815_at	---	1	0	0	1	0	0	3.93	4.01	4.07	4.43	4.02	1	0	0	0	0	1	0	5.85	5.99	6.12	5.83	6.24	6.65	5.62
1379824_at	Tox	1	1	0	0	0	0	7.63	7.16	7.78	7.30	7.42	1	0	0	1	0	0	0	8.92	8.95	9.21	9.56	9.37	8.98	8.66
1379833_at	Lingo4	1	0	0	1	1	1	4.58	5.05	4.84	5.91	5.06	1	0	0	0	0	1	1	7.71	7.76	7.59	7.15	7.15	6.65	7.00
1379879_at	LOC102548199 /	1	0	1	0	0	0	4.69	4.72	5.36	4.68	5.10	1	0	0	0	0	1	1	4.40	4.44	4.54	4.54	4.61	5.16	4.50
1379906_at	Atp1b2	1	0	0	1	0	0	7.34	7.36	7.43	7.81	7.31	1	0	0	0	0	1	1	5.86	5.86	5.99	5.76	5.94	7.12	7.55
1379932_at	Clcn4	1	0	0	0	1	1	4.73	4.63	4.60	5.19	5.41	1	0	0	0	0	1	1	6.76	6.75	7.01	7.15	7.12	7.68	7.53
1379950_at	---	1	1	0	0	0	0	5.22	4.59	5.06	4.89	4.94	1	0	0	0	0	0	1	5.92	5.87	5.89	5.75	5.68	5.38	4.71
1379971_at	Zc3h6	1	0	1	0	1	0	5.30	5.31	5.98	5.12	5.34	1	0	0	0	0	0	1	6.94	6.95	6.96	6.55	6.60	6.88	7.07
1380044_x_at	---	1	0	1	0	1	1	7.87	7.52	7.28	8.01	6.81	1	0	0	0	1	1	1	4.10	4.33	4.50	4.66	4.88	5.10	4.66
1380121_at	Nek7	1	0	0	1	1	0	5.54	5.58	6.11	4.96	5.43	1	0	0	0	1	1	1	5.20	5.20	5.50	5.71	5.88	6.59	5.81
1380185_at	Cxorf56	1	0	1	0	0	1	5.66	5.69	6.41	5.99	6.24	1	0	0	0	0	1	0	7.29	7.38	7.55	7.70	7.78	7.91	7.72
1380248_at	Sema4g	1	0	1	0	1	0	5.82	5.73	5.14	5.87	5.55	1	0	0	0	0	1	1	5.65	5.65	5.67	5.52	5.73	6.27	6.02
1380321_at	Mtus1	1	0	0	0	0	1	6.55	6.69	6.89	6.42	5.92	1	0	0	0	0	1	0	8.81	8.87	8.99	9.10	9.21	9.43	8.18
1380425_at	Rnasel	1	0	1	0	1	0	4.69	4.58	5.50	4.75	4.83	1	0	0	0	0	1	1	5.34	5.43	5.67	5.72	5.68	6.09	6.37
1380570_at	---	1	0	1	0	1	1	6.58	6.70	7.22	6.50	7.20	1	0	0	0	0	1	1	7.16	7.19	7.30	7.46	7.38	7.78	7.94
1380682_at	Mex3b	1	0	1	0	0	0	3.46	3.52	4.11	3.46	3.54	1	0	0	0	0	1	1	7.35	7.37	7.34	7.43	7.28	6.68	6.48
1380723_at	---	1	0	1	0	1	0	6.19	6.14	5.58	6.51	5.81	1	0	0	0	0	0	1	5.06	5.12	5.15	4.97	5.01	4.75	4.44
1380808_at	Tollip	1	0	0	1	1	0	6.41	6.15	6.88	5.31	6.03	1	0	0	0	0	0	1	7.47	7.67	7.69	7.87	7.63	7.53	6.43
1380872_at	Faxc	1	0	1	0	1	0	4.31	4.15	4.98	4.28	4.71	1	0	0	0	0	0	1	7.66	7.69	7.84	7.91	7.87	7.35	7.13
1380964_at	Dtna	1	0	0	1	1	0	5.62	5.45	5.55	6.19	5.60	1	0	0	0	0	1	1	9.22	9.28	9.30	9.14	9.05	8.48	8.18
1380981_at	Ston2	1	0	1	0	0	0	4.98	5.12	5.60	5.03	5.08	1	0	0	0	0	1	1	4.15	4.05	4.22	4.33	4.43	6.12	6.75
1381024_at	Ppp2r1a	1	0	0	1	1	0	7.55	7.33	8.04	6.78	7.43	1	0	0	0	0	0	1	9.43	9.47	9.61	9.58	9.47	9.30	8.26
1381082_at	Igsf23	1	0	1	0	1	1	5.42	5.43	4.76	5.40	4.88	1	0	0	0	0	1	1	4.35	4.31	4.33	4.33	4.46	5.40	5.76
1381190_at	Lmo7	1	0	1	1	0	0	7.07	6.92	7.69	7.60	7.08	1	0	0	0	0	1	0	9.66	9.55	9.46	9.27	9.13	9.05	8.70
1381206_at	Plcxd2	1	1	0	0	0	1	4.03	4.56	4.49	4.19	4.74	1	0	0	0	0	1	1	5.93	5.98	6.10	6.15	6.20	6.62	6.84
1381246_at	Phf21b	1	0	1	0	1	0	5.23	5.18	5.83	5.10	5.52	1	0	0	0	0	0	1	9.19	9.16	9.40	9.42	9.41	9.73	9.65
1381291_at	---	1	0	0	1	0	0	3.82	3.82	3.92	4.32	3.84	1	0	0	0	0	1	1	6.02	6.05	6.15	5.93	5.77	5.32	4.22
1381447_at	C1orf159	1	0	1	0	0	0	4.77	4.61	4.20	4.46	4.41	1	0	0	0	0	1	1	6.45	6.40	6.22	6.17	6.03	5.61	5.57
1381465_at	Tmem229a	1	0	1	0	0	0	6.13	6.16	5.60	5.94	6.05	1	0	0	0	0	1	1	7.76	7.72	7.74	7.77	7.66	6.92	5.92

Probeset ID	Gene Symbol	Islet Data Set (log2 ratio ≥0.263; FDR <20%) union n=5325 1=YES 0=NO	Islet DRlyp/lyp ND vs ND+Lp299v (log2 ratio ≥0.263; FDR<20%) n=429 1=YES 0=NO	Islet DRlyp/lyp ND vs HCD (log2 ratio ≥0.263; FDR<20%) n=2247 1=YES 0=NO	Islet DRlyp/lyp ND vs HCD+Lp299v (log2 ratio ≥0.263; FDR<20%) n=1595 1=YES 0=NO	Islet DRlyp/lyp HCD vs HCD+Lp299v (log2 ratio ≥0.263; FDR<20%) n=3026 1=YES 0=NO	Islet DRlyp/lyp ND vs Flyp/lyp ND (log2 ratio ≥ 0.263; FDR<20%) n=1950 1=YES 0=NO	Islet Mean Log2 Intensity DRlyp/lyp ND	Islet Mean Log2 Intensity DRlyp/lyp ND+Lp299v	Islet Mean Log2 Intensity DRlyp/lyp HCD	Islet Mean Log2 Intensity DRlyp/lyp HCD+Lp299v	Islet Mean Log2 Intensity Flyp40 ND	Rinm5f data union of all conditions n=3705 1=YES 0=NO	Rinm5f 30 µM propionate/ 6 µM butyrate vs Media (FDR<20%; 1.5 fold) n=3 1=YES 0=NO	Rinm5f 60 µM propionate/ 12 µM butyrate vs Media (FDR<20%; 1.5 fold) n=18 1=YES 0=NO	Rinm5f 120 µM propionate/ 24 µM butyrate vs Media (FDR<20%; 1.5 fold) n=164 1=YES 0=NO	Rinm5f 240 µM propionate /48 µM butyrate vs Media (FDR<20%; 1.5 fold) n=268 1=YES 0=NO	Rinm5f 1500 µM propionate/ 300 µM butyrate vs Media (FDR<20%; 1.5 fold) n=2491 1=YES 0=NO	Rinm5f 500 µM propionate/ 500 µM butyrate vs Media (FDR<20%; 1.5 fold) n=3001 1=YES 0=NO	Rinm5f Mean Log2 Intensity Media only	Rinm5f Mean Log2 Intensity 30 µM propionate/ 6 µM butyrate	Rinm5f Mean Log2 Intensity 60 µM propionate/ 12 µM butyrate	Rinm5f Mean Log2 Intensity 120 µM propionate/ 24 µM butyrate	Rinm5f Mean Log2 Intensity 240 µM propionate/ 48 µM butyrate	Rinm5f Mean Log2 Intensity 1500 µM propionate /300 µM butyrate	Rinm5f Mean Log2 Intensity 500 µM propionate/ 500 µM butyrate
1381498_at	---	1	0	0	1	1	1	6.96	7.24	6.95	6.16	6.34	1	0	0	0	0	1	1	6.57	6.38	6.58	6.31	6.22	5.61	4.39
1381605_at	Usp13	1	1	1	0	1	1	6.34	6.82	7.09	6.17	7.36	1	0	0	0	0	0	1	7.91	7.91	7.98	7.96	7.86	7.47	6.98
1381638_at	---	1	0	0	1	1	0	4.14	4.36	4.25	5.18	4.34	1	0	0	0	0	0	1	3.70	3.76	3.90	3.73	3.83	3.95	4.48
1381713_at	---	1	0	0	1	0	0	5.09	5.12	5.63	5.56	5.31	1	0	0	0	0	1	0	8.26	8.59	8.41	8.59	8.77	8.93	8.21
1381775_at	LOC103691564	1	0	0	0	0	1	6.50	6.62	6.44	6.20	5.65	1	0	0	0	0	1	0	5.93	6.12	6.06	5.94	6.11	6.55	6.63
1381798_at	Lmo7	1	1	0	0	0	1	5.15	4.50	5.07	5.17	4.35	1	0	0	0	0	1	0	6.65	6.70	6.64	6.36	6.35	5.96	6.08
1381871_at	Magi1	1	0	0	1	1	0	7.21	7.19	6.97	8.05	7.00	1	0	0	0	0	1	0	6.97	7.10	7.04	6.99	7.10	7.94	8.44
1381940_at	---	1	1	1	1	0	0	4.32	4.80	4.95	5.33	4.72	1	0	0	0	0	0	1	8.18	8.11	8.18	8.08	7.91	7.65	7.62
1381968_at	Creg1	1	0	0	0	1	1	7.59	7.54	7.25	7.86	6.77	1	0	0	0	0	0	1	7.64	7.61	7.63	7.62	7.57	7.92	8.01
1381996_at	Gpm6b	1	0	1	0	1	1	8.00	8.02	8.63	7.58	8.39	1	0	0	0	1	1	0	7.99	7.99	8.25	8.52	8.69	9.03	8.51
1382032_at	---	1	0	0	0	1	0	6.96	7.04	7.38	6.66	7.30	1	0	0	0	0	0	1	8.10	8.10	8.11	8.08	8.01	8.27	8.49
1382058_at	Rras2	1	0	0	0	0	1	7.61	7.39	7.12	7.38	7.10	1	0	0	0	0	1	1	8.79	8.77	8.90	9.03	9.07	9.56	9.43
1382098_at	Cntn1	1	0	1	0	1	1	7.84	8.08	8.91	7.45	8.86	1	0	0	0	0	1	1	10.20	10.38	10.32	10.34	10.62	11.36	10.89
1382130_at	Pcdh19	1	0	0	1	0	0	5.89	5.54	5.51	5.30	5.55	1	0	0	0	0	1	1	7.24	7.14	7.29	7.14	7.07	8.29	8.69
1382137_at	Abhd3	1	0	1	0	1	1	3.93	4.13	4.75	3.86	5.09	1	0	0	1	1	1	1	8.25	8.37	8.50	8.91	9.05	10.05	9.50
1382183_at	---	1	0	0	0	0	1	7.19	7.34	7.45	7.01	8.20	1	0	0	0	0	1	1	7.39	7.48	7.48	7.53	7.65	8.27	8.28
1382230_at	Klhdc8a	1	0	0	0	0	1	6.64	6.88	6.58	6.83	6.18	1	0	0	0	0	1	1	10.76	10.70	10.63	10.39	10.33	9.49	8.98
1382262_at	UBE2E3	1	0	0	0	0	1	6.17	6.19	6.64	6.12	6.74	1	0	0	0	0	1	0	6.59	6.80	6.93	7.07	7.16	7.58	7.29
1382263_at	Odf2l	1	0	1	0	0	1	4.53	4.77	5.25	4.86	5.15	1	0	0	0	0	1	0	7.86	8.06	8.11	8.22	8.26	8.48	8.25
1382271_at	Rps6ka5	1	0	1	0	1	0	4.80	5.06	5.68	4.93	4.99	1	0	0	0	0	0	1	6.93	6.96	6.99	7.05	7.05	7.47	7.41
1382272_at	Agtrap	1	0	0	1	0	0	6.02	6.34	6.09	6.53	6.03	1	0	0	0	0	1	0	6.14	6.17	6.49	6.42	6.47	6.87	7.11
1382294_at	---	1	0	0	1	0	0	7.39	7.58	7.53	7.93	7.59	1	0	0	0	0	1	1	7.85	7.76	7.85	7.83	7.69	8.90	9.06
1382303_at	Phactr1	1	0	0	0	0	1	6.21	6.60	6.62	6.13	7.28	1	0	0	0	0	1	1	7.92	7.74	7.79	7.91	7.69	7.11	7.12
1382325_at	Gcat	1	0	1	0	1	1	10.71	10.77	9.69	10.77	9.89	1	0	0	0	0	1	1	7.05	7.07	7.17	7.16	7.37	8.42	8.82
1382387_at	Ano1	1	0	0	0	1	0	7.48	7.21	7.15	7.90	7.06	1	0	0	0	0	1	1	7.51	7.56	7.70	7.69	7.95	8.21	7.93
1382404_at	Rnf125	1	0	0	1	1	0	3.98	3.83	3.87	4.48	4.10	1	0	0	0	0	0	1	3.80	3.86	3.68	3.61	3.69	4.06	4.34
1382436_at	Chmp1b /// RGD	1	0	1	0	0	0	4.64	4.54	5.20	4.92	5.01	1	0	0	0	0	1	1	7.77	7.71	7.70	7.61	7.43	6.44	5.95
1382443_at	Pabpc4	1	1	1	0	0	1	10.00	9.47	9.45	9.59	9.12	1	0	0	0	0	0	1	8.81	8.75	8.91	9.06	8.93	8.47	7.19
1382472_at	E2f2	1	0	1	1	1	0	5.21	5.39	4.59	6.03	5.55	1	0	0	0	0	0	1	7.54	7.49	7.60	7.59	7.62	8.05	8.61
1382521_at	Gls	1	0	0	1	1	0	9.04	9.02	9.42	8.32	9.50	1	0	0	0	0	1	0	11.14	11.29	11.28	11.39	11.41	11.79	11.33
1382537_at	Rragc	1	0	0	1	1	0	7.37	7.07	7.85	6.83	7.41	1	0	0	0	0	0	1	9.60	9.59	9.62	9.62	9.46	9.14	8.88
1382542_at	Nexmif	1	0	0	1	1	0	3.37	3.51	3.40	3.97	3.42	1	0	0	0	0	0	1	5.33	5.33	5.51	5.51	5.42	4.92	4.25
1382567_at	Nmt2	1	0	1	0	1	0	4.43	4.55	5.37	4.14	4.63	1	0	0	0	0	1	0	7.65	7.60	7.74	7.83	7.81	8.26	7.32
1382587_at	---	1	0	0	0	0	1	4.45	4.49	4.80	4.51	4.99	1	0	0	0	0	1	1	7.52	7.45	7.61	7.40	7.33	6.65	5.85
1382589_at	Ankrd55	1	1	0	0	0	0	4.89	5.39	5.22	4.69	4.96	1	0	0	0	0	1	1	5.53	5.72	5.84	5.89	5.91	6.54	6.44
1382596_a_at	Tmem214	1	0	0	0	1	0	8.25	8.49	7.91	8.68	8.04	1	0	0	0	0	0	1	8.79	8.75	8.83	8.73	8.68	8.23	7.96
1382603_at	Cd274	1	0	1	1	1	1	5.49	5.72	6.07	4.92	6.08	1	0	0	0	1	1	1	8.09	8.25	8.36	8.58	8.76	9.02	8.57
1382604_at	Polr3g	1	0	0	0	1	0	7.12	7.09	7.60	6.75	7.47	1	0	0	0	0	1	1	7.41	7.46	7.58	7.69	7.83	8.92	9.12
1382616_at	Gls	1	0	1	1	1	0	4.64	4.77	5.22	3.49	4.90	1	0	0	0	0	1	1	8.66	8.59	8.81	9.02	8.96	9.59	8.28
1382628_at	---	1	0	0	0	1	0	5.63	5.62	6.02	5.29	5.74	1	0	0	0	0	1	1	6.32	6.31	6.50	6.61	6.75	7.22	7.39
1382651_at	---	1	0	1	0	1	1	6.86	7.12	7.80	6.71	7.70	1	0	0	0	0	1	1	7.97	8.15	8.24	8.28	8.42	8.74	8.93
1382680_at	Plin2	1	0	1	0	1	1	4.50	4.81	5.37	4.43	5.30	1	0	0	0	0	1	1	4.83	4.76	5.10	5.14	5.10	6.06	6.53
1382847_at	Ash1l	1	0	1	0	0	0	6.73	6.67	7.17	6.61	6.88	1	0	0	0	0	1	0	7.54	7.37	7.39	7.44	7.26	6.90	6.58
1382882_x_at	---	1	1	1	0	1	1	10.96	11.50	11.78	11.05	11.53	1	0	0	0	0	1	1	3.65	3.72	3.69	3.80	3.77	4.57	5.22
1382989_at	---	1	0	0	0	1	1	7.57	7.69	7.89	7.13	8.24	1	0	0	0	1	0	0	8.82	9.16	9.37	9.27	9.42	9.06	7.10
1383040_a_at	LOC102552818	1	0	1	0	1	0	10.29	10.43	9.73	10.55	10.08	1	0	0	0	0	0	1	10.96	11.00	10.92	10.96	10.94	10.53	9.99
1383041_x_at	LOC102552818	1	0	1	0	1	0	10.00	10.07	9.45	10.28	9.80	1	0	0	0	0	0	1	10.32	10.33	10.26	10.35	10.29	9.93	9.27
1383056_a_at	Tmem238	1	0	1	1	1	1	9.72	9.92	9.19	10.23	9.22	1	0	0	0	0	1	1	9.95	9.89	9.92	9.93	9.81	9.10	8.72
1383058_at	---	1	1	0	0	0	0	5.37	5.85	5.37	5.56	5.31	1	0	0	0	0	1	1	10.06	10.10	10.07	9.91	9.97	10.81	11.17
1383097_at	Borcs7	1	0	1	0	1	0	6.19	6.10	6.84	5.83	6.13	1	0	0	0	0	1	1	8.70	8.54	8.56	8.46	8.24	7.70	7.42
1383104_at	Aamdc	1	0	1	0	1	1	7.65	7.66	6.99	7.88	7.12	1	0	0	0	0	0	1	6.83	6.79	6.80	6.67	6.66	6.94	7.75
1383129_at	---	1	0	1	0	1	0	7.24	7.50	7.95	7.17	7.56	1	0	0	0	1	1	1	7.69	7.80	7.93	8.20	8.32	8.95	9.03
1383146_at	Nbea	1	0	1	1	1	0	8.69	8.73	9.45	8.11	9.06	1	0	0	0	0	1	1	10.77	10.81	10.93	11.01	11.08	11.43	11.42
1383149_at	Asap3	1	0	0	0	1	0	4.84	4.84	4.57	5.20	4.59	1	0	0	0	0	1	1	5.60	5.85	5.82	5.81	6.01	6.75	7.23
1383164_at	Arhgef26	1	1	0	1	0	0	4.66	4.09	5.08	5.39	4.39	1	0	0	0	0	1	1	4.79	4.74	4.75	4.78	4.71	5.47	4.99
1383169_at	LOC103691479	1	1	0	0	0	0	9.68	9.13	9.66	10.01	9.30	1	0	0	0	0	1	1	6.53	6.64	6.72	6.83	6.80	7.39	8.25
1383188_at	Ppara	1	0	0	0	1	1	5.30	5.27	4.91	5.54	4.69	1	0	0	0	0	1	1	5.03	5.10	5.25	5.33	5.40	5.82	6.47
1383191_at	Ppp1r1a	1	1	1	0	0	1	7.63	8.21	8.44	7.85	8.33	1	0	0	0	0	1	1	8.14	7.97	8.12	8.26	8.00	7.42	6.91
1383211_at	---	1	0	0	0	0	1	3.60	3.59	3.38	3.63	5.14	1	0	0	0	0	1	1	4.69	4.68	4.85	4.97	4.98	5.95	6.60
1383212_at	Psd3	1	0	0	0	0	1	6.02	5.73	6.25	5.86	5.37	1	0	0	0	0	1	1	7.65	7.78	7.95	8.04	8.02	8.52	8.82
1383229_at	Abca7	1	0	1	0	1	0	7.78	7.91	7.21	8.04	7.42	1	0	0	0	1	1	0	7.89	8.05	8.29	8.30	8.50	8.76	8.57
1383238_at	Qtrt1	1	0	0	0	0	1	5.95	5.83	5.87	6.24	5.48	1	0	0	0	0	0	1	7.85	7.81	7.96	8.09	7.96	7.40	6.82
1383240_at	Itga6	1	0	0	1	1	0	7.47	7.39	7.36	8.13	7.53	1	0	0	0	0	0	1	6.13	6.13	6.14	6.01	6.04	6.22	6.97
1383241_at	C1r	1	0	0	1	0	0	6.45	6.24	6.09	5.92	6.21	1	0	1	1	1	1	1	6.32	6.47	6.92	7.07	7.32	7.51	7.28
1383276_at	Lancl2	1	0	1	1	1	0	4.45	4.46	5.24	3.95	4.37	1	0	0	0	0	1	1	8.35	8.26	8.37	8.39	8.21	7.60	6.79
1383299_at	Stk38l	1	0	1	0	0	1	6.70	6.62	7.27	6.85	7.17	1	0	0	0	0	1	1	6.54	6.73	6.82	6.94	7.02	7.62	7.81
1383366_at	---	1	0	0	1	0	0	5.37	5.17	5.79	5.98	5.33	1	0	0	0	0	1	0	6.68	6.40	6.77	6.72	6.56	5.89	5.88
1383401_at	Tes	1	0	0	0	0	1	9.34	9.45	9.76	9.33	9.86	1	0	0	0	0	0	1	10.39	10.38	10.34	10.27	10.31	10.85	11.47
1383427_at	Man1b1	1	0	1	0	1	0	6.83	6.61	6.04	6.95	6.49	1	0	0	0	0	1	1	7.71	7.64	7.64	7.64	7.53	7.04	6.56
1383447_at	Etv5	1	1	1	1	0	1	5.80	5.28	7.34	7.53	5.06	1	0	0	0	0	0	1	5.37	5.38	5.43	5.43	5.49	5.67	6.30
1383467_at	Epc2	1	0	0	0	1	0	6.45	6.20	6.89	6.10	6.19	1	0	0	0	0	1	1	7.97	7.83	8.05	8.08	7.89	7.18	6.75
1383511_at	---	1	0	1	0	1	1	7.60	7.94	8.45	7.53	8.36	1	0	0	0	0	1	1	8.04	8.06	8.24	8.51	8.43	8.78	8.77
1383516_at	Fgl2	1	0	0	1	1	0	4.25	4.25	4.35	3.63	4.17	1	0	0	0	0	0	1	11.19	11.26	11.12	10.98	11.24	10.99	9.87
1383517_at	---	1	0	1	0	1	0	6.89	7.11	5.86	7.08	7.05	1	0	0	0	0	1	1	6.29	6.34	6.53	6.55	6.64	7.22	7.13
1383520_at	Srek1	1	0	1	0	1	0	7.77	7.56	8.44	7.62	7.90	1	0	0	0	0	1	1	10.05	10.06	10.14	10.16	10.08	9.14	8.50
1383527_at	NFATC2	1	0	0	1	0	0	6.41	6.38	6.35	6.89	6.16	1	0	0	0	0	1	1	5.41	5.58	5.85	5.81	5.99	6.63	7.32
1383554_at	Rnf128	1	1	0	0	1	0	6.17	5.59	5.74	6.31	5.77	1	0	0	0	0	1	1	6.45	6.40	6.36	6.35	6.36	7.36	7.45
1383555_at	Nupr2	1	0	0	0	1	1	7.23	7.09	6.86	7.44	6.64	1	0	0	0	0	1	1	7.76	7.88	7.96	8.04	8.12	9.13	9.25
1383561_at	Lig4	1	0	1	0	1	1	5.92	5.91	6.73	5.68	4.82	1	0	0	0	0	0	1	7.96	7.97	8.06	8.08	8.13	8.49	8.30
1383565_at	---	1	0	1	0	1	0	3.72	3.87	4.50	3.70	3.94	1	0	0	0	0	0	1	5.48	5.37	5.37	5.46	5.47	5.91	6.33
1383606_at	Tc2n	1	1	0	0	0	1	10.66	10.13	10.61	10.68	9.99	1	0	0	0	0	1	1	4.00	3.97	4.22	4.38	4.49	4.81	4.75
1383638_at	---	1	0	1	1	1	0	6.20	6.15	6.81	5.69	6.52	1	0	0	0	0	0	1	8.10	8.21	8.25	8.27	8.33	8.56	8.40
1383649_a_at	Ints6l	1	0	1	1	1	1	6.99	6.77	7.59	6.42	7.54	1	0	0	0	0	1	0	10.22	10.32	10.35	10.53	10.65	11.26	9.64
1383721_at	Fzd8	1	0	0	0	0	1	5.18	5.36	5.46	5.33	5.71	1	0	0	0	0	1	1	6.68	6.65	6.61	6.25	6.33	6.03	5.84
1383744_at	---	1	0	1	1	1	1	5.62	5.93	6.38	4.91	6.45	1	0	0	0	0	0	1	7.99	7.99	8.09	7.95	8.13	7.85	7.06
1383747_at	Ect2	1	0	1	1	0	1	5.04	5.09	5.98	5.64	5.99	1	0	0	0	0	1	1	10.49	10.47	10.41	10.46	10.33	9.35	9.22
1383783_at	Pcdh9	1	1	1	0	1	1	5.97	6.49	7.06	5.87	7.05	1	0	0	0	0	1	1	6.09	6.14	6.24	6.27	6.06	6.80	7.15
1383798_at	Fam151a	1	1	1	0	0	0	9.41	9.94	10.12	9.52	9.69	1	0	0	1	1	1	1	6.90	7.16	7.42	7.72	7.99	8.94	8.68
1383800_at	Fam155a	1	0	1	0	1	1	5.78	5.94	6.57	5.71	6.76	1	0	0	0	0	1	0	9.84	9.77	9.82	9.71	9.71	9.24	9.27
1383826_at	Rab40b	1	0	1	0	1	0	7.73	7.89	8.54	7.60	8.11	1	0	0	0	0	1	1	8.67	8.78	8.68	8.67	8.93	9.79	9.83
1383879_at	Prr15l	1	1	1	0	0	0	7.45	6.68	6.90	7.33	7.37	1	0	0	0	0	1	0	7.44	7.51	7.61	7.72	7.85	8.11	7.88
1383948_at	Ttc28	1	0	0	0	0	1	5.26	5.43	5.78	5.47	5.87	1	0	0	0	0	0	1	5.82	5.72	5.77	5.81	5.93	6.13	6.30
1384000_at	Sox4	1	0	1	1	0	0	5.78	5.75	6.65	6.26	6.20	1	0	0	0	0	0	1	9.81	9.69	9.87	9.92	9.98	10.34	10.33
1384059_at	---	1	0	1	0	1	1	5.79	6.10	6.72	5.37	6.38	1	0	0	0	0	1	0	6.80	6.74	6.91	7.13	7.20	7.44	6.98
1384087_at	LOC102552369	1	0	0	0	0	1	5.30	5.23	5.48	5.59	4.09	1	0	0	0	0	0	1	8.87	8.94	9.08	9.29	9.22	8.46	7.56
1384098_at	Rnf125	1	0	0	0	1	0	4.23	4.19	4.12	4.69	4.50	1	0	0	0	0	1	1	4.02	4.12	4.01	3.93	4.09	4.69	5.43
1384124_at	---	1	1	0	0	0	0	6.16	5.69	6.51	6.24	6.11	1	0	0	0	0	1	0	10.88	10.98	11.11	11.34	11.43	11.48	10.68
1384127_at	Cebpa	1	0	1	1	1	1	6.25	6.35	5.55	6.93	5.75	1	0	0	0	0	0	1	4.35	4.41	4.37	4.42	4.36	4.93	5.08
1384143_at	Cgn	1	0	0	0	1	0	5.68	5.65	5.43	6.08	5.70	1	0	0	0	0	1	1	6.16	6.01	6.41	6.45	6.62	7.35	7.62
1384163_at	---	1	1	0	1	1	0	7.89	7.42	8.04	8.88	7.74	1	0	0	0	0	1	1	8.29	8.28	8.34	8.31	8.40	9.41	10.05
1384166_a_at	Lysmd4	1	0	0	0	0	1	6.01	6.17	6.24	6.20	7.28	1	0	0	0	0	1	0	7.44	7.34	7.30	7.32	7.21	6.82	6.94
1384174_at	Shcbp1	1	0	1	0	1	0	4.51	4.56	3.82	4.56	4.44	1	0	0	0	0	1	1	8.63	8.54	8.69	8.76	8.64	7.78	8.03
1384179_at	LOC102554200	1	0	0	0	1	0	4.27	4.00	4.81	4.07	4.43	1	0	0	0	1	0	0	6.01	6.32	6.16	6.25	6.63	6.49	5.60
1384202_at	Tesc	1	0	1	0	1	1	9.27	9.19	8.04	9.57	8.46	1	0	0	0	0	1	0	9.96	10.07	10.14	10.17	10.33	10.71	10.53
1384215_at	---	1	0	0	0	1	0	6.77	6.46	6.31	6.96	6.34	1	0	0	0	0	0	1	6.63	6.68	6.78	6.86	6.91	7.18	7.11
1384231_at	Shcbp1	1	0	1	0	1	0	5.28	5.67	4.06	5.46	5.40	1	0	0	0	0	1	1	9.41	9.33	9.37	9.45	9.27	8.32	8.04
1384247_at	Acsl6	1	0	1	0	0	1	4.78	5.05	5.48	4.81	5.69	1	0	0	0	1	0	0	6.79	6.88	7.17	7.35	7.51	7.26	6.10
1384250_a_at	Pde5a	1	0	1	0	0	0	3.77	3.72	4.37	3.77	3.98	1	0	0	0	0	1	1	6.26	6.25	6.43	6.56	6.68	7.21	6.85
1384253_at	Palldl1	1	0	1	0	1	0	8.97	9.22	9.94	8.83	9.01	1	0	0	0	0	1	1	7.30	7.12	7.12	7.16	6.84	5.56	5.08
1384280_at	Nusap1	1	0	0	1	0	1	6.02	6.19	6.51	6.59	6.52	1	0	0	0	0	1	1	10.49	10.40	10.45	10.39	10.26	9.08	8.97
1384415_at	Sox7	1	0	0	1	0	0	5.46	5.39	5.40	4.90	5.44	1	0	0	0	0	1	0	7.86	7.84	7.74	7.56	7.60	7.26	6.75
1384437_at	Smarca1	1	0	1	0	1	0	5.76	5.69	6.79	5.49	6.02	1	0	0	0	0	0	1	8.47	8.60	8.58	8.77	8.72	8.19	7.10
1384468_at	---	1	0	0	1	1	0	8.16	8.28	7.80	8.88	8.09	1	0	0	0	0	1	1	7.20	7.17	7.21	7.33	7.41	7.91	7.95
1384515_at	Hey2	1	0	0	0	0	1	3.93	4.20	4.18	4.10	4.49	1	0	0	0	0	0	1	3.91	3.95	3.94	4.08	3.97	4.45	4.50
1384526_at	Tex15	1	0	1	1	1	0	7.27	7.56	5.39	6.67	7.03	1	0	0	0	0	0	1	4.70	4.88	4.96	5.14	5.08	5.25	6.03
1384544_at	Pon3	1	0	1	0	1	0	7.56	7.40	6.96	7.87	7.11	1	0	0	0	0	1	1	7.65	7.68	7.71	7.81	7.91	8.58	8.48
1384587_at	Pak3	1	0	0	0	0	1	5.07	5.24	5.17	5.23	6.00	1	0	0	0	0	1	1	7.52	7.54	7.74	7.91	7.89	8.24	8.18
1384602_at	Gpr107	1	1	0	0	0	1	5.51	5.05	5.06	5.34	4.96	1	0	0	0	0	1	1	7.65	7.67	7.66	7.42	7.36	6.99	6.20
1384605_at	---	1	0	0	1	0	1	6.24	6.65	6.71	6.71	6.78	1	0	0	0	0	1	1	9.18	9.00	9.20	9.11	9.07	8.38	8.26
1384634_at	Fam222a	1	0	0	0	0	1	4.62	4.54	5.03	4.83	5.58	1	0	0	0	0	1	0	8.58	8.61	8.58	8.33	8.40	7.97	7.86
1384743_at	---	1	0	1	0	1	0	4.12	4.04	4.84	3.91	4.39	1	0	0	0	0	1	1	7.56	7.64	7.78	7.96	8.13	8.76	8.82
1384799_at	Nexmif	1	0	1	1	1	1	4.60	4.51	3.82	5.29	4.01	1	0	0	0	0	1	1	5.69	5.67	5.85	5.73	5.43	4.88	4.26
1384812_at	---	1	0	0	0	0	1	5.25	5.62	5.64	5.45	5.79	1	0	0	0	0	1	1	7.34	7.39	7.38	7.43	7.51	8.05	8.12
1384839_at	---	1	0	0	0	1	1	5.31	5.43	5.84	5.12	5.98	1	0	0	0	0	0	1	10.10	10.30	10.31	10.40	10.50	10.27	9.05
1384971_at	Depdc7	1	0	1	1	1	0	5.99	5.65	5.16	6.93	5.66	1	0	0	0	0	1	1	5.27	5.17	5.15	5.46	5.36	6.17	6.97
1384975_at	Ermp1	1	0	0	0	0	1	6.32	5.99	6.28	6.09	5.00	1	0	0	0	0	1	1	8.39	8.28	8.39	8.49	8.51	8.99	8.56
1385016_at	RGD1560784	1	0	0	0	0	1	6.30	6.31	6.75	6.09	6.78	1	0	0	0	0	1	1	9.57	9.52	9.47	9.21	9.27	8.46	8.21
1385061_at	Tmem229a	1	0	0	0	0	1	2.91	3.13	3.15	3.02	3.48	1	0	0	0	0	1	1	10.47	10.67	10.64	10.64	10.83	9.52	8.27
1385077_at	Gbf1	1	0	0	0	1	0	6.33	6.55	5.91	6.65	6.49	1	0	0	0	0	1	0	6.27	6.00	6.16	6.08	5.84	5.42	5.19
1385086_at	Bub1	1	0	1	0	0	1	4.24	4.44	4.89	4.56	4.84	1	0	0	0	0	1	1	9.25	9.17	9.17	9.30	9.15	8.03	8.23
1385098_at	---	1	0	0	0	1	1	4.46	4.35	4.98	4.11	3.89	1	0	0	0	0	0	1	3.57	3.53	3.53	3.49	3.57	3.86	4.62
1385128_at	Atp8a1	1	0	1	0	0	0	4.03	4.07	4.62	4.34	4.14	1	0	0	0	0	0	1	7.31	7.28	7.36	7.39	7.40	7.84	8.73
1385170_at	Fstl5	1	0	1	0	1	0	3.80	4.01	4.51	3.59	3.38	1	0	0	0	0	1	1	5.68	5.51	5.57	5.80	5.52	7.23	7.68
1385220_at	Zwilch	1	0	0	1	0	0	4.00	3.94	4.02	4.47	3.89	1	0	0	0	0	1	1	7.34	7.18	7.41	7.44	7.23	6.42	6.87
1385252_at	Trim6-trim34	1	0	1	0	0	0	5.83	6.31	6.47	6.06	6.05	1	0	0	0	0	1	1	7.29	7.40	7.51	7.55	7.63	8.06	7.38

Probeset ID	Gene Symbol	Islet Data Set (log2 ratio ≥0.263; FDR <20%) union n=5325 1=YES 0=NO	Islet DRlyp/lyp ND vs ND+Lp299v (log2 ratio ≥0.263; FDR<20%) n=429 1=YES 0=NO	Islet DRlyp/lyp ND vs HCD (log2 ratio ≥0.263; FDR<20%) n=2247 1=YES 0=NO	Islet DRlyp/lyp ND vs HCD+Lp299v (log2 ratio ≥0.263; FDR<20%) n=1595 1=YES 0=NO	Islet DRlyp/lyp HCD vs HCD+Lp299v (log2 ratio ≥0.263; FDR<20%) n=3026 1=YES 0=NO	Islet DRlyp/lyp ND vs Flyp/lyp ND (log2 ratio ≥ 0.263; FDR<20%) n=1950 1=YES 0=NO	Islet Mean Log2 Intensity DRlyp/lyp ND	Islet Mean Log2 Intensity DRlyp/lyp ND+Lp299v	Islet Mean Log2 Intensity DRlyp/lyp HCD	Islet Mean Log2 Intensity DRlyp/lyp HCD+Lp299v	Islet Mean Log2 Intensity Flyp40 ND	Rinm5f data union of all conditions n=3705 1=YES 0=NO	Rinm5f 30 µM propionate/ 6 µM butyrate vs Media (FDR<20%; 1.5 fold) n=3 1=YES 0=NO	Rinm5f 60 µM propionate/ 12 µM butyrate vs Media (FDR<20%; 1.5 fold) n=18 1=YES 0=NO	Rinm5f 120 µM propionate/ 24 µM butyrate vs Media (FDR<20%; 1.5 fold) n=164 1=YES 0=NO	Rinm5f 240 µM propionate /48 µM butyrate vs Media (FDR<20%; 1.5 fold) n=268 1=YES 0=NO	Rinm5f 1500 µM propionate/ 300 µM butyrate vs Media (FDR<20%; 1.5 fold) n=2491 1=YES 0=NO	Rinm5f 500 µM propionate/ 500 µM butyrate vs Media (FDR<20%; 1.5 fold) n=3001 1=YES 0=NO	Rinm5f Mean Log2 Intensity Media only	Rinm5f Mean Log2 Intensity 30 µM propionate/ 6 µM butyrate	Rinm5f Mean Log2 Intensity 60 µM propionate/ 12 µM butyrate	Rinm5f Mean Log2 Intensity 120 µM propionate/ 24 µM butyrate	Rinm5f Mean Log2 Intensity 240 µM propionate/ 48 µM butyrate	Rinm5f Mean Log2 Intensity 1500 µM propionate /300 µM butyrate	Rinm5f Mean Log2 Intensity 500 µM propionate/ 500 µM butyrate
1385334_at	Fam149a	1	0	1	0	0	1	7.57	7.34	8.23	7.98	8.38	1	0	0	0	1	0	0	8.27	8.47	8.40	8.57	8.90	8.57	7.39
1385387_at	NKX2-2	1	0	0	0	1	0	8.33	8.38	8.75	8.03	8.73	1	0	0	0	0	0	1	9.13	9.00	9.20	9.31	9.17	8.81	8.41
1385427_at	Msi2	1	1	1	1	0	1	5.63	6.33	6.22	6.18	6.12	1	0	0	0	0	1	0	6.70	6.53	6.56	6.40	6.36	6.06	5.45
1385503_at	Casd1	1	0	1	0	1	0	6.65	6.37	7.24	6.52	6.72	1	0	0	0	0	0	1	7.92	7.92	8.06	8.20	8.16	7.83	7.19
1385505_at	Cpeb3	1	0	1	0	0	0	5.65	5.66	6.37	5.88	5.71	1	0	0	0	0	1	0	6.69	6.75	6.89	7.03	7.12	7.34	7.39
1385560_at	---	1	0	1	0	1	1	6.70	6.86	7.28	6.48	7.22	1	0	0	0	0	1	1	9.53	9.67	9.65	9.60	9.67	8.84	7.80
1385581_at	Far1	1	0	0	0	0	1	6.66	6.69	6.98	6.32	7.37	1	0	0	0	0	1	1	8.87	8.96	9.08	9.18	9.23	9.64	9.34
1385639_at	Casp8ap2	1	0	1	0	0	1	3.60	3.59	4.33	3.77	4.19	1	0	0	0	0	1	0	6.99	7.25	7.27	7.45	7.55	7.79	6.91
1385671_at	---	1	0	0	0	1	0	8.58	8.59	9.03	8.31	8.68	1	0	0	0	0	1	1	8.62	8.59	8.65	8.56	8.45	7.64	7.17
1385706_at	---	1	0	0	0	0	1	6.70	6.88	7.08	6.73	7.51	1	0	0	0	0	1	1	7.73	7.64	7.75	7.76	7.73	8.36	8.55
1385716_at	Tbc1d16	1	0	1	0	1	1	7.65	7.40	7.11	8.04	6.86	1	0	0	0	0	1	1	8.13	8.20	8.24	8.11	8.30	8.84	9.40
1385765_at	Lin9	1	0	1	0	1	0	4.77	4.76	5.51	4.80	4.95	1	0	0	0	0	0	1	7.55	7.34	7.51	7.44	7.35	7.03	6.94
1385788_at	Ephb3	1	0	1	1	1	1	6.88	7.05	6.35	7.43	6.00	1	0	0	0	0	0	1	6.31	6.30	6.29	6.23	6.35	6.84	7.22
1385798_at	Inpp4b	1	0	1	0	1	0	6.49	6.34	7.66	6.40	6.57	1	0	0	0	0	0	1	4.47	4.47	4.62	4.53	4.34	4.66	5.10
1385826_at	---	1	0	1	0	1	0	6.05	6.20	6.78	5.70	6.49	1	0	0	0	0	0	1	6.28	6.33	6.39	6.28	6.19	5.81	5.02
1385852_at	Crebbp	1	0	0	1	1	0	3.76	3.87	4.07	4.51	4.13	1	0	0	0	0	1	1	5.93	5.51	5.93	5.92	5.63	4.97	5.95
1385859_at	---	1	0	1	1	1	0	4.31	4.30	5.68	3.41	4.60	1	0	0	0	0	1	0	7.45	7.59	7.66	7.87	7.94	8.11	7.09
1385868_at	---	1	0	1	0	1	0	8.35	8.50	9.07	7.99	8.64	1	0	0	0	0	0	1	10.80	10.87	10.96	11.03	11.03	10.57	9.75
1385909_at	Rims2	1	0	1	0	1	0	5.98	6.15	6.75	5.84	6.32	1	0	0	0	0	0	1	9.35	9.35	9.42	9.39	9.39	8.88	8.40
1385944_at	---	1	0	1	0	1	1	4.30	4.52	5.06	4.32	4.96	1	0	0	0	0	1	1	6.82	6.81	6.98	6.84	6.74	5.96	5.08
1386029_at	LOC103691124	1	0	1	1	1	0	5.27	5.20	6.29	4.78	5.73	1	0	0	0	0	1	1	7.67	7.93	7.80	7.97	7.99	7.73	6.43
1386114_at	Ints6l	1	0	0	0	1	0	6.52	6.30	6.88	6.10	6.79	1	0	0	0	0	1	0	9.65	9.70	9.78	9.94	10.03	10.62	8.85
1386119_at	Necab1	1	0	1	0	1	0	4.30	4.22	5.07	4.07	4.06	1	0	0	0	0	1	0	4.91	4.85	5.10	5.00	4.99	5.56	5.40
1386321_s_at	Trib3	1	0	0	1	1	0	7.12	7.06	6.96	8.14	6.90	1	0	0	0	0	0	1	8.04	8.03	8.08	8.24	8.18	7.69	7.39
1386392_at	Anapc10	1	0	1	0	1	0	3.43	3.58	4.05	3.33	3.60	1	0	0	0	0	0	1	6.09	6.14	6.22	6.34	6.20	5.92	4.98
1386535_at	Ddx3x	1	0	1	1	1	0	6.90	7.21	7.69	5.92	6.75	1	0	0	0	0	0	1	8.88	8.88	8.96	9.05	9.03	8.56	7.59
1386602_at	Rab40b	1	0	0	0	1	0	5.24	5.06	5.71	4.86	5.01	1	0	0	0	0	1	1	6.35	6.25	6.36	6.13	6.18	7.13	7.38
1386632_at	Pip4p2	1	0	1	0	1	1	5.88	5.51	6.82	5.51	6.46	1	0	0	0	0	1	1	8.28	8.39	8.38	8.48	8.36	7.69	7.17
1386870_at	Glul	1	0	0	1	1	1	8.48	8.05	8.56	7.61	7.97	1	0	0	0	0	1	0	8.73	8.76	8.96	9.03	9.00	9.37	9.33
1386881_at	Igfbp3	1	1	0	1	0	0	6.05	5.29	6.12	5.47	6.22	1	0	0	0	0	0	1	7.69	7.70	7.69	7.70	7.69	8.24	8.45
1386889_at	Scd2	1	0	0	1	1	0	6.38	6.51	6.90	5.67	6.44	1	0	0	0	0	0	1	10.83	10.80	10.83	10.69	10.69	10.59	9.41
1386901_at	Cd36 /// LOC10(	1	1	0	1	1	1	7.99	7.33	7.74	6.76	7.26	1	0	0	0	0	0	1	4.38	4.46	4.52	4.28	4.29	4.83	5.90
1386908_at	Glrx	1	0	0	0	0	1	8.64	8.49	8.90	8.71	9.50	1	0	0	0	1	1	1	9.11	9.05	9.29	9.58	9.79	10.69	10.59
1386914_at	Gmpr	1	0	0	0	0	1	5.27	5.38	5.35	5.09	4.65	1	0	0	0	0	1	1	7.30	7.26	7.44	7.39	7.38	8.23	8.64
1386927_at	Cpt2	1	0	0	1	0	0	7.90	7.68	7.90	7.35	7.83	1	0	0	0	0	0	1	8.10	8.08	8.13	8.19	8.17	8.65	8.87
1386938_at	Anpep	1	0	0	1	1	1	6.36	6.20	6.23	7.10	7.26	1	0	0	0	0	1	1	6.35	6.42	6.58	6.47	6.70	7.15	7.73
1386946_at	Cpt1a	1	1	0	0	0	1	8.29	7.49	8.57	8.31	7.57	1	0	0	1	0	1	1	9.12	9.12	9.40	9.73	9.71	10.27	10.35
1386960_at	Slc37a4	1	0	0	0	1	0	9.09	8.96	9.58	8.80	9.35	1	0	0	0	0	0	1	8.93	8.86	8.93	8.87	8.86	8.50	8.08
1386965_at	Lpl	1	1	0	0	0	1	9.84	9.30	9.43	9.79	9.09	1	0	0	1	1	1	1	7.97	8.23	8.44	8.67	8.87	9.16	9.14
1386968_at	Ppp1r1a	1	0	1	0	1	1	10.06	10.40	10.92	9.94	10.54	1	0	0	0	0	1	1	10.50	10.49	10.56	10.53	10.46	9.90	9.14
1386981_at	Slc16a1	1	1	0	1	1	1	5.79	5.29	5.56	6.45	5.06	1	0	0	0	0	0	1	8.58	8.66	8.67	8.70	8.64	8.93	8.89
1386985_at	Gstm5	1	0	1	0	0	0	7.00	6.66	6.37	6.72	6.59	1	0	0	0	1	1	1	6.10	6.35	6.55	6.63	6.69	7.80	8.72
1386992_at	Pkn1	1	0	0	1	1	0	7.43	7.28	6.98	8.07	6.98	1	0	0	0	0	1	0	6.13	5.88	6.13	6.01	5.66	5.19	5.46
1387022_at	Aldh1a1	1	0	1	0	1	1	9.96	9.77	8.90	9.70	9.37	1	0	0	0	0	1	1	3.74	3.79	3.86	3.89	3.90	4.80	5.75
1387034_at	Pah	1	0	0	0	1	1	10.72	10.51	10.22	11.02	10.18	1	0	0	1	1	1	0	5.16	5.40	5.46	5.80	5.80	6.26	5.67
1387060_at	Klf6	1	1	0	0	0	0	8.77	9.34	8.50	8.56	8.56	1	0	0	0	0	1	0	9.00	8.93	8.97	8.75	8.65	8.40	8.59
1387074_at	Rgs2	1	1	0	1	1	0	8.39	9.01	8.69	7.82	8.71	1	0	0	0	0	0	1	6.11	6.10	6.09	5.92	5.97	6.61	7.71
1387122_at	Plagl1	1	0	0	0	0	1	5.33	5.43	5.67	5.38	6.17	1	0	0	0	0	1	1	5.16	5.10	5.39	5.27	5.16	5.79	6.12
1387134_at	Slfn12	1	0	0	0	0	1	4.66	4.99	4.50	4.29	5.17	1	0	0	0	0	1	1	4.04	4.07	4.13	4.10	4.28	5.17	4.70
1387171_at	Gria2	1	0	1	0	1	1	6.60	6.82	7.75	6.45	7.25	1	0	0	0	0	1	1	5.45	5.67	5.73	5.97	5.93	6.70	6.70
1387202_at	Icam1	1	0	1	0	0	0	4.93	4.91	4.33	4.84	5.08	1	0	0	0	0	1	1	6.04	6.03	6.23	6.33	6.59	7.78	7.92
1387205_at	Hla-g	1	0	0	0	0	1	6.94	7.27	7.42	7.02	7.53	1	0	0	0	0	1	0	8.48	8.64	8.60	8.65	8.79	9.30	8.55
1387212_at	Bhlha15	1	0	1	0	1	0	8.58	8.36	7.86	8.96	8.31	1	0	0	0	0	1	1	6.31	6.39	6.27	6.13	6.07	4.95	4.89
1387223_at	Aadat	1	0	1	0	0	1	5.85	5.49	5.15	5.53	5.24	1	0	0	1	1	0	0	4.63	4.60	4.81	5.22	5.26	4.98	4.73
1387228_at	Slc2a2	1	0	1	0	1	1	11.65	11.93	12.28	11.28	12.62	1	0	0	0	0	0	1	11.30	11.38	11.42	11.51	11.55	11.35	10.40
1387239_a_at	Padi4	1	0	1	0	1	1	5.36	5.21	4.47	5.70	4.75	1	0	0	0	0	1	1	3.81	3.95	4.04	3.95	3.90	4.85	4.57
1387247_at	LOC100911216 /	1	0	1	0	1	0	10.45	10.64	11.44	10.00	10.88	1	0	0	0	0	0	1	11.11	11.22	11.17	11.33	11.49	11.22	9.59
1387260_at	Klf4	1	0	0	1	0	1	7.22	7.64	6.73	6.52	6.71	1	0	0	0	0	1	1	5.30	5.46	5.16	5.35	5.33	5.90	6.09
1387270_at	Hhex	1	0	0	0	0	1	5.88	6.32	6.26	6.13	6.51	1	0	0	0	1	0	0	4.17	4.19	4.23	4.61	4.80	4.72	4.65
1387343_at	Cebpd	1	1	0	1	0	1	5.35	4.80	5.38	4.77	4.62	1	0	0	0	0	1	1	10.47	10.55	10.39	10.14	10.24	9.59	8.42
1387385_at	Cntn1	1	0	0	1	1	0	5.28	5.32	5.75	4.73	5.57	1	0	0	0	0	1	1	8.28	8.30	8.30	8.37	8.47	8.97	8.75
1387389_at	Ramp3	1	1	0	1	1	1	6.04	5.50	6.12	5.06	5.48	1	0	0	0	0	1	1	3.95	4.12	3.94	4.08	4.14	4.98	5.81
1387391_at	Cdkn1a	1	0	0	1	0	0	4.44	4.10	4.67	4.91	4.30	1	0	0	0	0	1	1	7.51	7.58	7.60	7.59	7.43	8.72	10.50
1387421_at	Kcnip3	1	0	0	1	0	0	5.11	5.06	5.26	4.62	4.74	1	0	0	0	0	0	1	7.31	7.42	7.52	7.65	7.43	7.18	6.23
1387448_at	Bet1l	1	0	0	1	1	0	6.25	5.95	6.31	5.43	6.04	1	0	0	0	0	0	1	7.88	7.96	7.91	7.88	7.75	7.35	6.48
1387455_a_at	Vldlr	1	0	1	0	0	1	7.21	6.91	7.92	7.59	7.84	1	0	0	0	0	1	1	10.57	10.49	10.31	10.02	10.01	9.34	8.83
1387517_at	Syt13	1	0	1	1	1	0	6.34	6.51	7.26	5.63	6.49	1	0	0	0	0	1	0	8.30	8.41	8.55	8.78	8.74	9.00	7.62
1387631_at	Hpgd	1	1	1	0	1	0	6.54	7.03	7.58	6.51	6.57	1	0	0	0	0	1	1	5.72	5.67	5.79	6.03	6.01	6.85	6.28
1387643_at	Fgf21	1	0	1	1	0	0	5.73	5.66	4.69	4.91	5.35	1	0	0	0	0	1	1	6.28	6.25	6.52	6.68	6.86	7.08	7.17
1387658_at	Eef2k	1	0	0	1	1	1	6.13	5.78	5.92	6.70	5.65	1	0	0	0	0	1	1	6.14	6.18	6.39	6.32	6.38	6.76	7.13
1387659_at	Gda	1	0	1	0	1	0	6.91	6.96	6.28	7.04	6.48	1	0	0	0	0	1	1	5.28	5.21	5.19	5.17	5.37	6.86	7.67
1387662_at	Syt4	1	1	1	1	1	1	6.38	7.12	7.32	5.89	7.21	1	0	0	0	0	1	1	9.63	9.50	9.50	9.45	9.36	9.03	7.74
1387670_at	Gpd2	1	0	1	0	0	0	6.50	6.49	7.16	6.59	6.86	1	0	0	0	0	1	1	7.78	7.78	7.91	7.97	7.92	8.54	8.89
1387672_at	Gnmt /// Loc10(	1	0	1	0	1	1	9.57	9.36	8.65	9.70	8.63	1	0	0	0	0	1	1	4.92	4.98	4.99	4.73	4.92	5.57	6.61
1387703_a_at	Usp2	1	0	0	1	1	0	5.10	4.90	4.67	5.64	4.75	1	0	0	0	0	1	1	4.63	4.67	4.68	4.66	4.61	5.26	5.79
1387763_at	Rab27a	1	0	1	1	1	0	6.15	6.21	6.81	5.37	5.91	1	0	0	0	0	0	1	7.09	7.30	7.31	7.39	7.31	6.99	5.64
1387769_a_at	Id3	1	0	0	1	1	0	6.38	5.96	6.48	5.56	6.29	1	0	0	1	0	1	0	9.01	9.11	8.92	8.40	8.52	8.22	9.37
1387770_at	Ifi27	1	0	0	0	0	1	8.25	8.69	8.27	7.92	9.92	1	0	0	0	0	1	0	11.20	11.35	11.40	11.51	11.58	11.95	11.83
1387793_at	Slc9a3r1	1	0	1	0	1	1	7.63	7.60	7.00	7.81	7.14	1	0	0	0	0	1	1	7.42	7.47	7.60	7.62	7.69	9.01	9.45
1387803_at	Ppp2r2b	1	0	0	1	0	0	7.09	6.90	7.11	7.56	6.93	1	0	0	0	0	0	1	8.28	8.22	8.29	8.38	8.34	8.73	8.65
1387808_at	Slc7a7	1	0	0	1	1	0	6.39	6.16	6.64	8.41	6.01	1	0	0	1	0	0	0	6.46	6.57	6.84	7.07	6.99	6.54	5.72
1387809_at	Map2k6	1	0	1	0	1	0	6.20	6.10	5.67	6.29	6.12	1	0	0	0	0	0	1	4.34	4.52	4.44	4.44	4.48	4.92	5.56
1387810_at	Keap1	1	0	0	0	1	0	5.78	5.58	6.26	5.46	5.86	1	0	0	0	0	0	1	8.39	8.40	8.48	8.53	8.40	8.03	7.14
1387870_at	Zfp36	1	0	1	1	0	0	8.58	8.56	7.90	7.48	8.42	1	0	0	0	0	0	1	8.02	7.98	8.20	7.87	7.93	8.00	6.26
1387885_at	Fcgrt	1	0	0	1	1	1	8.29	8.04	8.13	8.86	7.61	1	0	0	0	1	1	1	6.59	6.69	6.87	6.98	7.26	8.21	7.78
1387905_at	Dnajc12	1	0	1	0	1	0	7.38	7.46	8.14	7.11	7.65	1	0	0	0	0	1	1	8.41	8.36	8.56	8.69	8.83	9.70	9.63
1387908_at	Rasd1	1	1	0	0	1	0	7.60	8.30	8.12	7.37	7.85	1	0	0	0	0	1	0	9.26	9.41	9.60	9.51	9.75	9.96	8.18
1387969_at	Cxcl10	1	1	0	0	0	0	4.64	6.67	4.72	4.33	4.62	1	0	0	0	0	0	1	5.31	5.27	5.30	5.67	5.55	5.56	5.89
1387981_at	Or51e2	1	0	1	0	1	0	7.19	7.27	8.20	6.71	7.47	1	0	0	0	1	0	0	6.03	6.29	6.46	6.60	6.70	6.47	5.11
1387984_at	Cklf	1	0	1	0	0	1	4.74	4.89	5.27	4.67	5.28	1	0	0	0	0	1	1	6.59	6.67	6.81	6.90	7.00	8.10	7.77
1388060_at	Syt12	1	0	1	0	0	1	4.50	4.91	5.13	4.59	4.99	1	0	0	0	0	1	0	4.72	4.76	4.97	4.90	5.01	5.36	5.58
1388102_at	Ptgr1	1	0	0	1	0	0	6.03	5.83	6.24	6.60	5.70	1	0	0	0	1	1	1	5.65	5.59	5.86	5.87	6.26	8.80	9.24
1388122_at	Gstp1	1	0	0	0	1	1	9.96	9.69	9.57	10.19	9.46	1	0	0	1	1	1	1	8.39	8.64	8.79	9.08	9.21	10.17	10.58
1388132_at	Sfpq	1	0	0	0	1	0	7.44	7.09	7.96	7.21	7.73	1	0	0	0	0	0	1	10.32	10.27	10.43	10.51	10.46	10.05	9.51
1388143_at	Col18a1	1	0	0	0	1	0	6.16	6.08	5.73	6.32	5.72	1	0	0	0	0	0	1	7.24	7.23	7.25	7.04	7.15	7.52	7.68
1388152_at	Map2	1	0	1	0	1	0	9.46	9.75	10.09	9.23	9.90	1	0	0	0	0	1	1	8.72	8.89	8.93	9.05	9.19	9.79	9.98
1388196_at	Nckap1	1	0	1	1	1	0	9.12	9.10	9.73	8.52	9.24	1	0	0	0	0	0	1	10.37	10.37	10.37	10.37	10.29	9.86	9.19
1388271_at	Mt1m /// Mt2a	1	1	1	1	0	1	11.55	10.94	10.04	10.11	10.76	1	0	0	0	0	1	1	9.06	9.28	8.92	8.53	8.85	9.84	10.67
1388298_at	Myl9	1	0	1	0	1	1	7.46	7.52	6.88	7.46	6.99	1	0	0	0	0	0	1	5.53	5.47	5.57	5.37	5.48	5.83	6.27
1388340_at	Pclaf	1	0	0	1	0	0	6.60	6.50	6.73	7.21	7.03	1	0	0	0	0	1	1	9.87	9.76	9.76	9.83	9.70	9.11	8.64
1388393_at	Plp2	1	0	0	1	0	0	7.97	7.89	7.94	8.46	7.86	1	0	0	0	0	1	1	10.93	10.93	10.96	10.91	10.88	10.27	10.06
1388459_at	Col18a1	1	0	1	0	1	0	6.48	6.24	5.88	6.58	6.05	1	0	0	0	0	0	1	6.94	6.88	6.92	6.54	6.81	7.35	7.45
1388464_at	Cul7	1	0	0	1	0	0	7.53	7.82	7.72	8.08	7.96	1	0	0	0	0	1	1	9.48	9.45	9.48	9.42	9.34	8.78	8.31
1388569_at	Serpinf1	1	0	0	1	1	1	7.03	7.05	7.10	7.85	6.02	1	0	0	0	0	1	1	4.15	4.26	4.13	4.08	4.18	5.14	5.31
1388571_at	Syngr2	1	0	0	0	1	0	8.71	8.67	8.38	9.16	8.36	1	0	0	0	0	1	1	8.88	8.94	9.01	8.90	9.03	9.61	9.74
1388596_at	Cotl1	1	0	0	0	0	1	7.09	7.19	7.16	7.30	7.93	1	0	0	0	0	1	1	7.69	7.67	7.89	7.89	7.97	8.69	9.22
1388611_at	Tcea3	1	0	1	0	1	1	8.33	8.42	7.13	8.40	7.60	1	0	0	0	0	1	1	6.47	6.46	6.52	6.26	6.63	7.44	8.05
1388626_at	Ddrgk1	1	0	0	1	1	0	8.54	8.54	8.18	9.01	8.17	1	0	0	0	0	1	1	9.81	9.79	9.82	9.90	9.73	9.10	8.51
1388644_at	Mgll	1	0	1	0	1	1	6.94	6.70	6.31	7.15	6.40	1	0	0	0	0	0	1	5.41	5.59	5.50	5.37	5.45	5.91	7.35
1388650_at	Top2a	1	0	0	1	0	0	5.79	5.64	6.27	6.40	6.26	1	0	0	0	0	1	1	10.46	10.49	10.52	10.48	10.36	9.24	9.17
1388672_at	Zcchc24	1	0	0	0	1	1	5.79	5.63	5.42	6.02	5.31	1	0	0	0	0	1	1	6.98	7.06	7.19	7.30	7.35	8.15	8.25
1388674_at	Cdkn1a	1	0	0	1	1	0	7.30	7.19	7.20	8.72	7.10	1	0	0	0	0	1	1	8.29	8.28	8.32	8.22	8.27	8.98	9.55
1388693_at	Trabd	1	0	0	0	1	0	9.18	9.06	8.69	9.28	8.73	1	0	0	0	0	0	1	10.32	10.41	10.39	10.40	10.35	10.15	9.23
1388720_at	Rnf144b	1	0	1	0	1	0	6.77	6.62	6.23	7.10	6.51	1	0	0	1	0	1	0	7.69	7.61	7.44	7.07	7.12	6.65	7.39
1388728_at	Laptm4b	1	0	0	0	1	1	11.03	11.00	10.70	11.38	10.51	1	0	0	0	0	1	1	9.96	10.08	10.15	10.21	10.36	10.76	10.86
1388742_at	Bcl2l11	1	1	0	0	0	0	6.42	5.95	6.55	6.27	6.43	1	0	0	0	0	0	1	9.04	9.01	8.97	8.74	8.78	9.25	9.89
1388791_at	Spats2l	1	0	0	0	1	0	6.39	6.50	6.84	6.13	6.65	1	0	0	0	0	1	1	7.50	7.52	7.42	7.25	7.27	6.61	6.31
1388821_at	Trib2	1	0	0	1	1	0	6.67	6.48	7.03	6.17	6.77	1	0	0	0	0	1	1	8.59	8.67	8.82	9.04	9.11	9.59	9.93
1388853_at	Mrpl54	1	0	0	0	1	0	10.04	10.08	9.64	10.36	9.83	1	0	0	0	0	1	1	11.23	11.18	11.13	11.03	10.88	10.56	10.45
1388913_at	Plpp2	1	0	1	0	1	0	7.15	7.05	6.60	7.40	7.00	1	0	0	0	0	1	1	7.16	7.30	7.35	7.31	7.45	8.14	8.12
1388918_at	Hdlbp	1	0	0	0	1	0	9.26	9.13	9.03	9.60	9.34	1	0	0	0	0	1	1	9.34	9.28	9.33	9.22	9.23	8.69	8.49
1388921_at	Krt80	1	0	1	0	0	1	5.50	5.58	6.11	5.44	4.72	1	0	0	0	0	0	1	4.52	4.54	4.53	4.51	4.58	5.02	5.49
1388924_at	Angptl4	1	1	0	0	0	1	5.04	4.29	5.31	4.71	4.22	1	0	0	1	1	1	1	5.25	5.34	5.81	6.63	6.92	8.22	7.22
1388933_at	Itpk1	1	0	0	0	0	1	7.22	7.46	7.43	7.64	7.72	1	0	0	0	0	1	1	8.46	8.52	8.65	8.67	8.82	9.10	9.26
1388936_at	Cdh11	1	0	1	0	1	1	5.10	5.02	5.99	4.65	5.89	1	0	0	0	0	1	1	8.12	8.02	8.25	8.56	8.28	7.45	7.04
1388973_at	Col9a1	1	0	1	1	1	0	7.37	7.53	8.27	6.73	7.58	1	0	0	0	0	1	1	6.47	6.61	6.56	6.40	6.25	5.80	5.46
1388986_at	Klf6	1	1	0	0	0	0	7.59	8.48	7.71	7.22	7.98	1	0	0	0	0	1	0	8.08	8.13	8.02	7.89	7.81	7.36	7.64
1389004_at	Josd2	1	0	0	0	1	0	7.24	7.47	6.99	7.57	7.37	1	0	0	0	0	0	1	8.82	8.87	8.80	8.75	8.64	8.30	7.87
1389034_at	Usp18	1	1	0	0	1	0	6.22	7.01	5.78	6.50	6.30	1	0	0	0	0	1	1	5.73	5.91	5.91	5.87	5.89	6.59	6.41
1389042_at	Rnpepl1	1	0	1	1	1	0	8.24	8.33	7.66	9.03	8.16	1	0	0	0	0	1	1	7.38	7.34	7.45	7.39	7.22	6.77	6.79
1389049_at	---	1	0	0	0	1	0	6.06	5.85	6.59	5.62	6.20	1	0	0	0	0	1	1	4.84	4.86	5.04	5.08	5.12	5.83	6.39
1389107_at	Cgnl1	1	0	0	1	1	0	7.93	7.89	7.47	8.80	7.51	1	0	0	0	0	1	1	4.44	4.53	4.46	4.55	4.62	5.35	5.86
1389127_at	Smad3	1	0	0	1	1	0	5.86	5.77	5.57	6.57	5.61	1	0	0	0	0	0	1	5.93	5.90	6.08	6.06	6.12	6.37	6.67
1389137_at	---	1	0	0	0	0	1	5.09	5.05	5.12	5.05	6.01	1	0	0	0	0	1	0	8.39	8.29	8.42	8.34	8.18	7.76	8.29
1389142_at	Sqor	1	0	0	1	0	0	9.54	9.44	9.76	10.14	9.35	1	0	0	0	0	0	1	8.23	8.22	8.31	8.23	8.25	8.69	9.04

Probeset ID	Gene Symbol	Islet Data Set (log2 ratio ≥0.263; FDR <20%) union n=5325 1=YES 0=NO	Islet DRlyp/lyp ND vs ND+Lp299v (log2 ratio ≥0.263; FDR<20%) n=429 1=YES 0=NO	Islet DRlyp/lyp ND vs HCD (log2 ratio ≥0.263; FDR<20%) n=2247 1=YES 0=NO	Islet DRlyp/lyp ND vs HCD+Lp299v (log2 ratio ≥0.263; FDR<20%) n=1595 1=YES 0=NO	Islet DRlyp/lyp HCD vs HCD+Lp299v (log2 ratio ≥0.263; FDR<20%) n=3026 1=YES 0=NO	Islet DRlyp/lyp ND vs Flyp/lyp ND (log2 ratio ≥ 0.263; FDR<20%) n=1950 1=YES 0=NO	Islet Mean Log2 Intensity DRlyp/lyp ND	Islet Mean Log2 Intensity DRlyp/lyp ND+Lp299v	Islet Mean Log2 Intensity DRlyp/lyp HCD	Islet Mean Log2 Intensity DRlyp/lyp HCD+Lp299v	Islet Mean Log2 Intensity Flyp40 ND	Rinm5f data union of all conditions n=3705 1=YES 0=NO	Rinm5f 30 µM propionate/ 6 µM butyrate vs Media (FDR<20%; 1.5 fold) n=3 1=YES 0=NO	Rinm5f 60 µM propionate/ 12 µM butyrate vs Media (FDR<20%; 1.5 fold) n=18 1=YES 0=NO	Rinm5f 120 µM propionate/ 24 µM butyrate vs Media (FDR<20%; 1.5 fold) n=164 1=YES 0=NO	Rinm5f 240 µM propionate /48 µM butyrate vs Media (FDR<20%; 1.5 fold) n=268 1=YES 0=NO	Rinm5f 1500 µM propionate/ 300 µM butyrate vs Media (FDR<20%; 1.5 fold) n=2491 1=YES 0=NO	Rinm5f 500 µM propionate/ 500 µM butyrate vs Media (FDR<20%; 1.5 fold) n=3001 1=YES 0=NO	Rinm5f Mean Log2 Intensity Media only	Rinm5f Mean Log2 Intensity 30 µM propionate/ 6 µM butyrate	Rinm5f Mean Log2 Intensity 60 µM propionate/ 12 µM butyrate	Rinm5f Mean Log2 Intensity 120 µM propionate/ 24 µM butyrate	Rinm5f Mean Log2 Intensity 240 µM propionate/ 48 µM butyrate	Rinm5f Mean Log2 Intensity 1500 µM propionate /300 µM butyrate	Rinm5f Mean Log2 Intensity 500 µM propionate/ 500 µM butyrate
1389157_at	Cdc42ep1	1	0	1	0	1	1	9.15	8.78	8.44	9.56	8.47	1	0	0	0	0	0	1	5.88	5.83	5.57	5.59	5.62	6.09	6.88
1389159_at	Pde7b	1	0	1	0	0	1	6.60	6.89	7.28	6.89	7.26	1	0	0	0	0	0	1	6.96	6.96	6.99	6.79	6.62	6.53	6.22
1389176_at	Inpp5f	1	0	1	0	0	0	6.82	6.85	7.40	6.71	7.15	1	0	0	0	0	1	1	9.22	9.30	9.34	9.38	9.46	9.89	9.68
1389206_at	Efr3b	1	0	0	0	1	1	7.73	7.94	8.23	7.52	8.35	1	0	0	0	0	0	1	7.62	7.57	7.78	7.83	7.86	8.20	8.78
1389210_at	Lcp1	1	0	1	0	1	1	6.90	6.50	5.90	6.46	7.43	1	0	0	1	0	0	0	5.53	5.47	5.50	4.91	5.09	5.06	4.90
1389221_at	Mmd2	1	0	0	0	0	1	5.72	6.08	5.95	5.45	5.23	1	0	0	0	1	1	1	6.20	6.31	6.43	6.58	6.88	7.75	7.67
1389241_at	Ubr1	1	0	1	0	1	0	7.74	7.90	8.45	7.71	8.14	1	0	0	0	0	0	1	9.74	9.75	9.80	9.85	9.81	9.19	8.86
1389256_at	---	1	0	0	1	1	1	6.13	5.85	6.08	6.90	5.37	1	0	0	0	0	1	1	4.75	4.86	4.89	4.86	4.85	5.88	5.73
1389287_at	Tead1	1	0	1	1	0	0	7.46	7.50	8.02	8.08	7.72	1	0	0	0	0	1	1	8.84	8.88	8.91	8.95	9.03	9.54	9.86
1389304_at	Rest	1	0	0	0	1	0	6.23	5.87	5.87	6.65	5.85	1	0	0	0	0	0	1	4.72	4.76	4.68	4.60	4.73	5.25	5.54
1389385_at	B9d1 /// LOC100	1	0	0	0	0	1	6.90	6.72	6.77	6.82	6.32	1	0	0	0	0	1	1	7.83	7.77	7.81	7.70	7.63	7.01	6.39
1389408_at	Rrm2	1	0	0	0	0	1	7.08	6.81	7.37	7.29	7.63	1	0	0	0	0	1	1	12.12	12.00	12.07	12.06	11.95	11.36	11.03
1389411_at	FRMD5	1	0	0	0	1	1	5.82	5.94	6.32	5.58	6.52	1	0	0	0	0	0	1	7.36	7.54	7.55	7.59	7.72	7.89	7.64
1389457_at	Mybl2	1	0	1	0	0	0	4.86	4.61	4.26	4.59	4.56	1	0	0	0	0	1	1	7.22	7.19	7.24	7.23	7.06	6.52	6.04
1389474_at	Mylip	1	0	0	0	0	1	6.81	6.48	7.14	6.95	6.27	1	0	0	0	1	1	1	6.05	6.08	6.42	6.54	6.70	7.02	6.71
1389548_at	Adhfe1	1	0	0	0	0	1	8.59	8.21	8.11	8.44	7.85	1	0	0	0	0	1	1	4.95	4.87	5.09	4.87	4.85	5.76	6.27
1389557_at	Tex261	1	0	0	0	1	0	8.12	8.07	7.94	8.58	7.86	1	0	0	0	0	0	1	9.48	9.45	9.45	9.49	9.36	9.04	8.66
1389573_at	Chac1	1	0	1	1	1	0	9.66	9.66	7.80	8.54	9.47	1	0	0	0	0	0	1	9.82	9.82	9.98	10.20	10.32	10.12	8.25
1389579_at	Pappa2	1	0	0	1	1	0	4.77	4.77	4.91	4.18	4.78	1	0	1	1	1	0	1	7.09	7.41	7.84	8.60	8.97	7.58	5.63
1389618_at	---	1	0	0	0	0	1	6.16	6.01	6.36	6.35	5.62	1	0	0	0	0	1	0	7.69	7.88	7.66	7.26	7.51	7.01	6.61
1389632_at	Rhobtb1	1	0	1	0	0	1	7.92	8.28	8.50	7.99	8.74	1	0	0	0	0	1	1	8.24	8.30	8.22	8.19	8.21	7.31	6.82
1389649_at	Acss3	1	0	0	1	0	0	7.24	7.05	7.61	7.84	7.56	1	0	0	0	0	1	1	6.56	6.32	6.57	6.53	6.13	5.01	4.13
1389660_at	Amigo3	1	0	0	0	1	0	6.59	6.76	6.19	6.75	6.87	1	0	0	0	0	1	1	6.87	6.98	6.95	6.80	6.81	6.26	5.99
1389697_at	---	1	0	0	1	1	0	4.93	4.78	4.52	5.69	4.82	1	0	0	0	0	0	1	4.56	4.42	4.38	4.30	4.30	4.54	5.26
1389732_at	Dram1	1	0	0	1	1	0	8.93	8.70	8.84	9.60	8.62	1	0	0	0	0	1	1	7.37	7.46	7.58	7.62	7.71	8.00	7.95
1389756_at	Melk	1	0	0	1	0	0	5.88	5.93	6.04	6.50	5.97	1	0	0	0	0	1	1	9.24	9.15	9.25	9.24	9.10	8.16	8.36
1389763_at	Pcdh19	1	0	0	1	0	0	5.94	5.56	5.76	5.43	5.83	1	0	0	0	0	1	1	6.97	6.84	6.98	7.05	6.94	8.09	8.02
1389774_at	Znf23	1	0	1	0	1	1	6.63	6.57	7.36	6.46	7.68	1	0	0	0	0	1	0	8.57	8.62	8.81	8.99	9.06	9.23	8.63
1389779_at	Sh2d4a	1	0	1	0	1	1	8.53	8.23	7.42	8.46	7.31	1	0	0	0	0	0	1	5.42	5.35	5.25	5.23	5.28	5.81	5.96
1389781_at	Elmod1	1	0	0	1	1	0	4.24	4.08	4.46	3.72	3.95	1	0	0	0	0	1	0	6.83	6.92	7.07	7.25	7.32	7.52	6.34
1389908_at	---	1	0	0	1	1	0	6.55	6.52	6.35	7.43	6.11	1	0	0	0	0	0	1	4.47	4.46	4.60	4.57	4.58	4.93	5.25
1389922_at	Hadh	1	0	1	1	1	0	7.00	7.09	7.89	6.34	7.30	1	0	0	0	0	0	1	8.80	8.63	8.71	8.61	8.69	8.28	7.36
1389955_at	---	1	0	1	0	1	0	4.40	4.36	5.22	4.12	4.52	1	0	0	0	0	1	0	7.12	7.17	7.24	7.49	7.50	7.78	7.29
1389999_at	---	1	1	0	0	0	0	6.43	6.00	6.46	6.82	6.16	1	0	0	0	0	0	1	5.60	5.33	5.50	5.52	5.50	6.09	6.08
1390021_at	H2bc10	1	0	0	1	1	0	9.46	9.30	9.00	9.98	9.16	1	0	0	0	0	0	1	5.47	5.39	5.38	5.39	5.18	4.98	5.02
1390024_at	Clec2g	1	1	0	0	0	0	8.63	8.00	8.58	8.28	8.43	1	0	0	0	0	1	1	6.66	6.95	6.66	6.50	6.48	5.42	3.99
1390026_at	Bag3	1	0	0	0	0	1	5.01	4.94	4.78	4.94	5.61	1	0	0	0	0	1	1	9.25	9.21	9.38	9.46	9.46	10.17	10.29
1390032_at	Rbms2	1	0	1	0	1	0	6.92	6.98	6.39	7.37	6.52	1	0	0	0	0	0	1	4.50	4.29	4.30	4.36	4.27	4.76	5.05
1390036_at	Slc16a6	1	0	1	0	0	0	5.92	6.10	4.98	5.53	5.90	1	0	0	0	0	0	1	5.46	5.40	5.54	5.42	5.47	5.82	6.24
1390050_at	Golm1	1	1	0	0	0	1	7.26	7.84	7.72	7.50	8.04	1	0	0	0	0	1	1	8.68	8.65	8.82	8.83	8.98	9.46	9.43
1390116_at	---	1	0	0	1	1	1	7.29	6.90	7.00	6.14	6.17	1	0	0	0	0	1	1	5.47	5.58	5.61	5.76	5.93	6.60	5.87
1390145_at	Dmxl2	1	0	1	1	1	0	6.68	6.55	7.40	6.08	6.71	1	0	0	0	0	1	0	8.02	8.08	8.20	8.36	8.45	8.65	8.69
1390146_at	Tmem150c	1	0	1	0	1	0	6.59	6.86	7.30	6.46	6.97	1	0	0	0	0	1	1	7.80	7.90	7.83	7.76	7.56	6.98	6.74
1390156_a_at	Prickle2	1	0	1	0	1	1	4.73	4.70	5.40	4.63	5.33	1	0	0	0	0	0	1	7.09	7.16	7.24	7.28	7.38	7.45	7.38
1390172_at	Dhtkd1	1	0	1	0	1	1	8.14	7.78	7.40	7.97	7.34	1	0	0	0	0	1	1	4.77	4.79	5.00	4.89	5.14	6.10	6.48
1390173_at	Card10	1	0	0	0	1	0	7.07	6.96	6.76	7.38	6.68	1	0	0	0	0	1	1	8.22	8.15	8.28	8.25	8.34	9.02	9.41
1390226_at	Sting1	1	0	0	1	0	0	4.99	5.14	4.77	4.45	4.95	1	0	0	0	0	1	1	6.90	6.70	7.00	7.28	7.12	7.78	8.00
1390340_a_at	Eif4g1 /// LOC68	1	0	0	1	1	0	8.52	8.97	8.16	9.22	8.72	1	0	0	0	0	0	1	9.52	9.36	9.45	9.49	9.26	9.05	8.92
1390382_at	Hypk	1	0	0	0	1	0	9.61	9.65	9.26	9.84	9.46	1	0	0	0	0	0	1	10.71	10.69	10.67	10.69	10.59	10.14	9.71
1390393_at	Cntnap2	1	0	0	0	0	1	4.48	4.84	4.66	4.65	4.98	1	0	0	0	0	0	1	4.96	4.98	5.07	4.97	4.95	5.32	5.66
1390412_at	Slc40a1	1	0	0	1	0	0	6.77	6.84	6.97	7.25	6.97	1	0	0	0	0	1	1	5.44	5.39	5.46	5.59	5.65	6.25	6.97
1390561_at	Lig4	1	0	0	0	0	1	6.53	6.69	6.88	6.62	6.04	1	0	0	0	0	0	1	7.50	7.55	7.56	7.53	7.58	7.98	8.25
1390581_at	LOC103691977	1	0	0	1	0	0	4.01	4.09	4.05	4.48	3.92	1	0	0	0	0	0	1	4.43	4.52	4.39	4.35	4.22	4.04	3.72
1390647_at	Phtf2	1	0	0	0	0	1	6.11	6.23	6.57	6.25	6.61	1	0	0	0	0	0	1	8.35	8.25	8.33	8.47	8.45	8.60	9.05
1390653_at	Wdr89	1	0	1	0	1	0	4.70	4.74	5.34	4.48	5.18	1	0	0	0	0	0	1	6.61	6.56	6.67	6.76	6.78	7.19	7.22
1390659_at	Cd44	1	0	0	0	0	1	4.27	4.30	4.39	4.14	4.75	1	0	0	0	0	0	1	5.08	4.98	5.18	5.20	5.18	5.43	5.75
1390723_at	---	1	0	1	0	1	0	5.94	5.82	6.53	5.79	6.03	1	0	0	0	0	1	1	7.60	7.80	7.96	7.98	8.16	8.47	8.00
1390738_at	Bst2	1	0	1	0	1	1	8.57	8.59	7.38	8.73	7.84	1	0	0	0	0	1	1	7.85	7.81	8.04	8.01	8.31	9.49	10.04
1390786_at	Arhgef2	1	0	0	1	1	0	6.82	6.86	6.97	8.23	6.74	1	0	0	0	0	1	1	5.77	6.05	6.25	6.23	6.27	6.50	6.46
1390828_at	Npy1r	1	0	0	0	0	1	3.67	3.65	3.70	3.36	3.20	1	0	0	1	1	0	0	6.27	6.35	6.70	6.98	6.93	6.73	6.44
1390872_at	Ift80	1	0	0	0	0	1	6.25	6.39	6.76	6.21	6.78	1	0	0	0	0	1	1	7.92	7.96	8.06	8.11	8.24	8.65	8.66
1390913_at	Ttc30a	1	0	1	0	0	0	4.71	4.94	5.34	4.99	4.92	1	0	0	0	0	1	0	6.32	6.23	6.34	6.58	6.54	6.91	6.86
1390931_at	Adamts15	1	0	1	0	1	1	5.81	5.77	5.17	5.88	5.03	1	0	0	0	0	0	1	4.95	5.00	4.98	4.71	4.90	5.30	5.22
1391007_s_at	Kcnj11	1	0	1	0	1	0	9.30	9.50	9.93	9.12	9.43	1	0	0	0	0	1	1	11.86	11.80	11.78	11.63	11.51	10.55	9.60
1391062_at	Elp4	1	0	1	0	0	0	8.40	8.50	9.00	8.38	8.79	1	0	0	0	0	1	1	10.26	10.38	10.28	10.12	10.19	9.55	9.38
1391075_at	Rgs17	1	1	0	0	0	0	7.31	7.80	7.62	7.11	7.48	1	0	0	0	0	0	1	10.80	10.91	10.83	10.80	10.87	10.66	9.15
1391125_at	Ap1s3	1	0	1	0	1	0	6.36	6.35	7.23	6.32	6.62	1	0	0	0	0	1	1	7.78	7.86	7.96	8.03	8.17	8.58	8.62
1391206_at	Inava	1	0	0	1	0	1	6.32	6.79	6.55	6.84	7.17	1	0	0	0	0	0	1	7.03	6.97	7.01	7.05	6.93	6.45	6.50
1391274_at	Rab3ip	1	0	1	0	1	1	5.41	5.56	6.02	5.29	5.97	1	0	0	0	0	1	0	9.47	9.52	9.60	9.71	9.81	10.10	9.87
1391295_at	---	1	0	0	1	1	0	6.03	5.83	6.26	5.53	6.10	1	0	0	0	0	0	1	8.66	8.70	8.92	8.88	8.78	8.90	9.03
1391331_at	Sgpp2	1	0	1	0	1	0	5.52	5.47	6.33	5.63	5.98	1	0	0	0	0	1	1	7.71	7.67	7.98	8.18	8.15	8.56	8.37
1391334_at	---	1	0	0	0	0	1	4.66	4.58	4.84	4.48	5.50	1	0	0	0	1	1	1	5.67	5.95	6.00	6.18	6.37	7.14	7.45
1391411_at	Fmnl2	1	0	1	0	0	1	7.46	7.48	8.07	7.50	7.97	1	0	0	0	0	1	0	8.25	8.45	8.38	8.34	8.58	8.84	9.10
1391414_at	Tmem131l	1	0	0	0	1	0	7.92	7.81	7.64	8.26	7.73	1	0	0	0	0	1	0	9.08	9.00	9.00	8.93	8.88	8.46	8.54
1391450_at	Loxl2	1	0	1	0	0	1	5.58	5.79	6.17	5.67	6.44	1	0	0	0	0	1	1	6.53	6.39	6.47	6.51	6.59	5.81	5.45
1391463_at	Ddx58	1	0	0	0	0	1	4.50	4.95	4.98	4.49	5.00	1	0	0	1	1	1	1	5.55	5.81	6.03	6.29	6.29	6.84	6.59
1391466_at	Nucks1	1	0	1	0	0	0	6.65	6.40	7.29	6.96	7.12	1	0	0	0	0	1	1	9.83	9.76	9.82	9.85	9.79	9.16	8.37
1391512_at	---	1	0	0	0	0	1	3.17	3.22	3.32	3.35	4.31	1	0	0	0	0	0	1	7.78	7.65	7.78	7.67	7.62	7.19	5.92
1391551_at	---	1	0	1	0	1	0	6.84	6.73	5.94	7.24	6.69	1	0	0	0	0	1	0	7.33	7.37	7.43	7.52	7.72	7.92	7.10
1391572_at	Cars	1	0	0	0	0	1	8.02	7.81	7.79	7.73	7.55	1	0	0	0	0	0	1	9.01	9.03	9.13	9.23	9.12	8.73	7.54
1391605_at	Ptprt	1	0	1	0	0	0	5.34	5.42	5.94	5.44	5.26	1	0	0	0	1	0	0	6.88	7.18	7.20	7.32	7.58	7.37	7.17
1391626_at	Mnd1	1	0	0	0	1	0	4.25	3.80	4.05	4.70	4.15	1	0	0	0	0	1	0	6.44	6.43	6.49	6.52	6.29	5.60	6.02
1391648_at	Ripply3	1	0	1	0	1	1	7.50	7.72	8.30	7.41	7.98	1	0	0	0	0	0	1	10.16	10.02	10.06	10.05	9.99	9.65	8.99
1391656_at	Cpm	1	0	1	0	0	0	4.13	3.97	4.86	4.43	4.09	1	0	0	0	0	1	1	5.09	5.32	5.38	5.23	5.31	6.31	7.26
1391687_at	Dtna	1	0	0	1	0	0	3.74	3.80	3.70	4.23	3.91	1	0	0	0	0	1	0	6.04	5.95	6.00	5.84	5.66	5.34	4.58
1391727_at	Pak3	1	0	0	0	0	1	5.00	4.87	5.28	4.68	5.90	1	0	0	0	0	1	1	8.40	8.47	8.54	8.78	8.84	9.01	9.00
1391744_at	LOC102548389	1	0	1	0	1	1	4.88	5.02	5.80	4.83	5.38	1	0	0	0	0	1	0	7.01	7.16	7.35	7.45	7.50	7.61	6.55
1391808_at	Arrdc4	1	0	1	0	1	0	6.47	6.40	7.11	6.19	6.76	1	0	0	0	0	1	0	9.85	10.02	10.10	10.28	10.38	10.57	10.48
1391812_at	COL6A6	1	0	0	0	1	1	7.15	7.11	7.70	6.77	7.70	1	0	0	0	0	1	1	6.28	6.34	6.64	6.70	6.62	5.13	4.37
1391856_at	Sema3g	1	0	1	1	0	1	5.81	5.75	5.20	5.27	4.98	1	0	0	1	1	1	1	6.94	7.05	7.31	7.57	7.71	8.18	7.60
1391915_at	Hspa9	1	0	0	0	0	1	6.03	6.02	6.40	6.32	5.25	1	0	0	0	0	1	0	6.52	6.43	6.59	6.53	6.46	5.92	5.27
1391936_a_at	Vdr	1	0	0	0	1	0	8.59	8.98	9.11	8.41	9.06	1	0	0	0	1	1	1	6.32	6.55	6.51	6.67	7.05	8.01	8.90
1391968_at	Pdxdc1	1	0	0	1	1	0	7.56	7.84	7.46	8.10	7.71	1	0	0	0	0	1	0	7.11	6.79	7.03	6.74	6.73	6.19	6.25
1391979_at	Fyb1	1	0	0	0	0	1	4.02	3.93	3.99	3.83	4.86	1	0	0	0	0	1	1	9.78	9.82	9.81	9.69	9.70	9.09	8.64
1391994_at	C1galt1	1	0	1	0	1	0	6.52	6.63	7.27	6.47	6.61	1	0	0	0	0	1	0	9.02	9.27	9.01	9.19	9.35	9.87	9.45
1392025_x_at	LOC103695179	1	0	0	0	1	1	11.81	11.87	11.46	12.20	11.12	1	0	0	0	0	1	0	9.83	9.89	9.90	9.94	10.03	10.44	9.86
1392045_at	Slc35g2	1	0	1	0	1	1	6.62	7.00	7.86	6.15	7.52	1	0	0	0	0	1	1	9.59	9.56	9.49	9.44	9.31	8.13	7.18
1392077_at	Mpped2	1	0	0	1	0	0	4.77	4.68	5.10	5.36	5.02	1	0	0	0	0	0	1	7.63	7.67	7.91	7.97	7.77	7.15	6.93
1392118_at	Soga1	1	0	1	1	0	1	5.26	5.41	5.63	5.60	6.78	1	0	0	0	0	1	0	7.33	7.12	7.22	7.14	6.95	6.73	7.15
1392145_at	LOC102553190	1	0	0	0	0	1	7.41	7.44	7.68	7.16	8.16	1	0	0	0	0	1	0	9.09	9.14	9.26	9.30	9.37	9.94	9.61
1392148_at	---	1	0	1	0	1	0	5.78	5.77	6.80	5.81	5.98	1	0	0	0	0	1	1	8.87	8.87	8.69	8.36	8.36	7.67	7.40
1392245_at	---	1	0	1	0	1	1	4.98	4.98	5.76	4.82	5.84	1	0	0	0	0	1	1	8.56	8.43	8.66	8.89	8.61	7.14	6.75
1392295_a_at	---	1	0	0	1	1	0	4.70	4.48	4.74	5.44	4.81	1	0	0	0	0	0	1	7.94	8.07	8.14	8.26	8.22	7.87	7.39
1392359_at	---	1	0	0	0	0	1	3.48	3.67	3.78	3.66	4.17	1	0	0	0	0	0	1	4.51	4.55	4.57	4.68	4.38	4.07	3.75
1392404_at	---	1	0	0	0	0	1	4.25	4.42	4.03	4.15	5.05	1	0	0	0	0	1	0	6.01	6.11	5.99	5.60	5.82	5.37	4.60
1392406_at	Ipp	1	0	0	0	1	0	3.72	3.88	4.25	3.55	3.91	1	0	0	0	0	1	0	6.28	6.42	6.59	6.77	6.61	7.00	6.09
1392421_at	Baz2b	1	0	0	0	1	0	5.05	4.93	5.60	4.66	4.99	1	0	0	0	0	0	1	6.54	6.61	6.78	6.63	6.62	5.96	5.63
1392467_at	Impa2	1	1	0	0	0	1	7.10	6.40	6.94	7.22	6.02	1	0	0	0	0	1	1	5.89	5.82	5.94	6.14	6.09	6.81	6.83
1392473_at	Nmt2	1	0	1	0	0	0	7.78	7.93	8.36	7.70	8.13	1	0	0	0	0	1	0	9.69	9.70	9.80	9.87	9.91	10.43	10.33
1392501_at	---	1	0	1	0	1	1	6.18	6.26	6.85	6.11	6.73	1	0	0	0	0	1	1	11.37	11.43	11.32	11.14	11.18	10.73	10.62
1392580_at	---	1	0	1	1	1	0	7.42	7.21	8.03	6.84	7.87	1	0	0	0	0	1	1	9.09	9.11	9.29	9.47	9.54	10.05	10.29
1392612_at	---	1	1	1	0	1	1	6.54	7.26	7.73	6.36	7.47	1	0	0	0	0	1	1	7.76	7.76	7.75	7.94	7.85	7.15	6.39
1392618_at	---	1	0	1	0	1	0	4.91	4.87	5.75	4.66	5.21	1	0	0	0	0	1	1	9.26	9.24	9.26	9.26	9.18	8.45	8.29
1392716_at	---	1	1	0	0	0	1	5.14	5.68	5.11	5.04	5.66	1	0	0	1	0	0	1	7.45	7.17	7.30	6.86	7.09	6.94	7.33
1392758_at	Tra2a	1	0	1	0	1	0	5.52	5.50	6.57	5.11	5.63	1	0	0	0	0	1	0	9.33	9.26	9.46	9.34	9.35	8.73	8.29
1392778_at	LOC102552467 /	1	0	0	0	1	0	4.24	4.57	4.76	3.85	4.49	1	0	0	0	0	1	1	8.55	8.63	8.48	8.57	8.58	7.93	7.03
1392785_at	Kctd12	1	0	1	1	1	0	4.35	4.64	5.39	3.76	4.71	1	0	0	0	0	0	1	5.49	5.16	5.18	5.54	5.24	5.22	5.88
1392823_at	Snhg11	1	0	1	0	1	0	9.03	9.14	9.61	8.63	9.23	1	0	0	0	0	1	1	5.92	5.95	6.24	6.04	6.11	6.71	6.81
1392852_at	Smoc1	1	0	1	1	0	0	5.35	4.93	6.74	6.68	5.04	1	0	0	0	0	0	1	8.46	8.50	8.65	8.72	8.71	8.10	7.05
1392899_at	Prc1	1	0	0	0	0	1	6.28	6.36	6.78	6.47	6.77	1	0	0	0	0	1	1	10.46	10.37	10.36	10.29	10.21	9.30	9.23
1392905_at	Gng2	1	0	0	0	0	1	6.48	6.77	6.73	6.36	6.96	1	0	0	0	0	1	0	8.40	8.43	8.28	8.08	8.02	7.81	8.02
1392906_at	Ubl4a	1	0	0	0	1	0	8.46	8.46	8.11	8.79	8.16	1	0	0	0	0	1	1	9.86	9.80	9.75	9.74	9.61	9.19	9.07
1392915_at	Col11a1	1	0	1	1	1	0	4.86	5.01	5.59	6.61	5.24	1	0	0	0	0	1	1	4.45	4.39	4.39	4.37	4.54	5.87	6.53
1392925_at	ST7	1	0	0	0	1	1	8.19	7.97	7.69	8.57	7.60	1	0	0	0	0	0	1	6.40	6.43	6.57	6.47	6.33	5.87	5.56
1392952_at	Acsf2	1	0	0	0	1	0	6.22	6.11	5.84	6.40	6.05	1	0	0	0	0	1	1	6.23	6.41	6.46	6.44	6.49	7.26	7.61
1392965_a_at	Smoc2	1	0	0	0	0	1	5.64	5.70	5.88	5.30	5.08	1	0	0	0	0	0	1	3.83	3.85	3.81	3.73	3.77	4.21	4.09
1392969_at	Hnf4a	1	0	0	1	0	0	8.19	8.31	8.28	8.79	8.11	1	0	0	0	0	1	1	8.34	8.16	8.20	8.16	7.92	7.19	7.24
1392997_at	Ppp4r2	1	0	1	0	1	0	5.25	5.26	5.84	5.10	5.34	1	0	0	0	1	1	0	8.69	8.98	9.02	9.20	9.32	9.51	8.97
1393041_at	Smc2	1	0	1	0	0	1	4.57	4.37	5.29	4.93	5.23	1	0	0	0	0	1	1	8.22	8.30	8.22	8.29	8.13	7.14	6.69
1393048_at	Adra2a	1	0	0	0	0	1	7.41	7.74	7.68	7.24	8.07	1	0	0	0	0	0	1	8.43	8.38	8.55	8.70	8.71	7.94	7.73
1393063_at	Eif5a2	1	0	1	0	1	1	3.32	3.30	4.15	3.46	3.99	1	0	0	0	0	1	1	7.44	7.57	7.75	8.02	8.00	8.34	8.59
1393109_at	---	1	0	1	0	1	1	7.60	7.40	8.26	7.18	8.33	1	0	0	0	0	1	0	6.64	6.73	6.59	6.49	6.37	5.76	5.99
1393131_at	Fiz1	1	0	0	1	0	0	5.89	6.27	5.83	6.38	6.05	1	0	0	0	0	1	1	8.21	8.17	8.24	8.11	8.04	7.53	7.31
1393142_at	Cep70	1	0	1	0	1	0	5.18	5.25	6.08	4.90	5.43	1	0	0	0	0	1	1	7.25	7.35	7.47	7.59	7.56	7.87	7.94
1393149_at	Pcdha4	1	0	1	0	1	1	6.51	6.67	7.49	6.15	7.10	1	0	0	0	0	0	1	9.76	9.84	9.80	9.78	9.81	9.30	8.70

Probeset ID	Gene Symbol	Islet Data Set (log2 ratio ≥0.263; FDR <20%) union n=5325 1=YES 0=NO	Islet DRlyp/lyp ND vs ND+Lp299v (log2 ratio ≥0.263; FDR<20%) n=429 1=YES 0=NO	Islet DRlyp/lyp ND vs HCD (log2 ratio ≥0.263; FDR<20%) n=2247 1=YES 0=NO	Islet DRlyp/lyp ND vs HCD+Lp299v (log2 ratio ≥0.263; FDR<20%) n=1595 1=YES 0=NO	Islet DRlyp/lyp HCD vs HCD+Lp299v (log2 ratio ≥0.263; FDR<20%) n=3026 1=YES 0=NO	Islet DRlyp/lyp ND vs Flyp/lyp ND (log2 ratio ≥ 0.263; FDR<20%) n=1950 1=YES 0=NO	Islet Mean Log2 Intensity DRlyp/lyp ND	Islet Mean Log2 Intensity DRlyp/lyp ND+Lp299v	Islet Mean Log2 Intensity DRlyp/lyp HCD	Islet Mean Log2 Intensity DRlyp/lyp HCD+Lp299v	Islet Mean Log2 Intensity Flyp40 ND	Rinm5f data union of all conditions n=3705 1=YES 0=NO	Rinm5f 30 µM propionate/ 6 µM butyrate vs Media (FDR<20%; 1.5 fold) n=3 1=YES 0=NO	Rinm5f 60 µM propionate/ 12 µM butyrate vs Media (FDR<20%; 1.5 fold) n=18 1=YES 0=NO	Rinm5f 120 µM propionate/ 24 µM butyrate vs Media (FDR<20%; 1.5 fold) n=164 1=YES 0=NO	Rinm5f 240 µM propionate /48 µM butyrate vs Media (FDR<20%; 1.5 fold) n=268 1=YES 0=NO	Rinm5f 1500 µM propionate/ 300 µM butyrate vs Media (FDR<20%; 1.5 fold) n=2491 1=YES 0=NO	Rinm5f 500 µM propionate/ 500 µM butyrate vs Media (FDR<20%; 1.5 fold) n=3001 1=YES 0=NO	Rinm5f Mean Log2 Intensity Media only	Rinm5f Mean Log2 Intensity 30 µM propionate/ 6 µM butyrate	Rinm5f Mean Log2 Intensity 60 µM propionate/ 12 µM butyrate	Rinm5f Mean Log2 Intensity 120 µM propionate/ 24 µM butyrate	Rinm5f Mean Log2 Intensity 240 µM propionate/ 48 µM butyrate	Rinm5f Mean Log2 Intensity 1500 µM propionate /300 µM butyrate	Rinm5f Mean Log2 Intensity 500 µM propionate/ 500 µM butyrate
1393229_at	Pmel	1	0	1	0	1	0	5.29	5.31	4.76	5.45	5.08	1	0	0	0	0	1	1	5.75	5.67	5.91	5.95	5.97	6.90	6.88
1393252_at	---	1	0	0	0	0	1	5.18	5.05	4.96	5.16	4.53	1	0	0	0	0	0	1	6.69	6.57	6.78	6.65	6.64	7.21	7.52
1393263_at	---	1	1	0	0	1	0	9.10	9.66	9.58	8.83	9.37	1	0	0	0	0	0	1	4.62	4.51	4.64	4.68	4.75	5.15	5.67
1393285_at	---	1	0	0	0	0	1	8.43	8.29	8.60	8.83	7.75	1	0	0	0	0	1	0	7.72	7.64	7.89	8.05	8.00	8.42	7.76
1393330_at	LOC100909539	1	0	0	1	1	1	8.28	8.33	8.04	8.94	7.75	1	0	0	0	0	0	1	3.96	4.00	3.94	3.82	3.96	4.24	4.74
1393466_at	Cd164l2	1	0	0	0	1	1	7.00	6.80	6.60	7.34	6.43	1	0	0	0	0	1	1	7.83	7.94	7.97	8.03	8.22	9.01	8.89
1393469_at	---	1	0	0	1	0	0	4.34	4.56	4.39	4.89	3.88	1	0	0	0	0	0	1	9.31	9.59	9.35	9.04	9.25	8.91	7.96
1393510_at	Sybu	1	0	1	0	1	1	5.15	4.90	6.02	5.27	6.12	1	0	0	0	0	1	1	7.77	7.76	7.97	8.17	8.22	8.57	8.41
1393561_at	---	1	0	1	0	1	1	6.04	6.20	6.89	6.13	6.55	1	0	0	0	0	1	1	9.04	9.23	9.31	9.26	9.53	10.07	9.29
1393612_a_at	Depdc7	1	0	0	0	1	0	4.39	4.27	4.12	4.86	4.34	1	0	0	0	0	0	1	4.84	4.79	4.78	4.82	4.85	5.22	5.21
1393641_at	Blnk	1	0	1	0	1	0	5.86	5.88	6.49	5.74	6.04	1	0	0	1	1	1	1	10.00	10.10	10.34	10.68	10.74	11.33	11.03
1393643_at	Rcn1	1	0	0	1	0	0	6.61	6.92	7.17	7.39	6.70	1	0	0	0	0	0	1	12.42	12.50	12.44	12.35	12.41	11.85	11.34
1393657_at	Prcp	1	1	0	0	0	1	6.14	5.57	6.36	6.17	5.43	1	0	0	0	0	1	0	7.14	7.39	7.53	7.61	7.64	8.16	7.09
1393705_at	---	1	0	0	0	1	0	6.70	6.34	7.02	6.30	6.47	1	0	0	0	0	0	1	8.21	8.20	8.37	8.40	8.25	8.74	9.33
1393719_at	Osbpl3	1	0	1	0	0	0	4.30	4.06	4.86	4.55	4.59	1	0	0	0	0	1	1	4.36	4.38	4.84	4.55	4.61	5.84	5.97
1393847_at	Nkiras1	1	0	0	0	1	0	4.94	5.00	5.35	4.57	5.32	1	0	0	0	0	1	1	6.28	6.34	6.37	6.58	6.63	7.41	7.25
1393910_at	Fam13a	1	0	0	1	1	0	5.79	5.91	5.55	6.49	5.80	1	0	0	0	0	1	1	4.54	4.50	4.72	4.65	4.59	5.20	5.23
1393935_at	Tmem139	1	0	1	0	1	1	5.73	5.43	4.90	6.11	4.72	1	0	0	0	0	1	1	3.86	3.84	3.90	3.73	3.81	4.59	5.83
1393952_at	Ccdc68	1	0	1	1	0	0	4.06	3.97	4.81	5.08	4.39	1	0	0	0	0	1	0	5.50	4.97	5.25	5.24	4.98	4.46	4.69
1394003_at	Pole3	1	0	0	0	1	0	6.05	6.11	5.59	6.41	5.87	1	0	0	0	0	0	1	7.36	7.38	7.33	7.43	7.24	6.84	6.41
1394020_at	---	1	0	0	1	1	1	7.22	6.81	7.00	7.72	6.72	1	0	0	0	0	1	1	7.73	7.95	7.76	7.55	7.77	6.57	6.80
1394022_at	Id4	1	0	0	0	1	0	4.50	4.83	4.29	4.94	4.50	1	0	0	0	0	1	0	8.30	8.28	8.35	8.40	8.24	7.67	8.55
1394047_at	---	1	0	1	0	1	0	6.41	6.55	7.00	6.23	6.68	1	0	0	0	0	1	1	6.67	6.56	6.60	6.63	6.70	7.35	8.04
1394097_at	Kcnip1	1	0	1	0	1	0	5.56	5.45	6.26	5.46	5.33	1	0	0	0	0	1	1	7.06	7.19	7.15	7.01	7.03	6.30	6.00
1394107_at	Tbl1xr1	1	1	0	0	0	0	4.93	4.45	5.05	4.89	5.07	1	0	0	0	0	1	0	6.74	6.84	7.01	7.17	7.27	7.33	6.96
1394109_at	---	1	0	1	1	1	0	5.58	5.52	6.40	4.80	5.68	1	0	0	1	1	1	1	3.91	4.03	4.29	4.75	4.73	5.41	6.06
1394207_at	Slc16a10	1	0	1	0	1	0	8.32	8.42	9.08	7.86	8.50	1	0	0	0	0	1	1	6.49	6.50	6.63	6.75	6.86	7.36	7.66
1394395_at	---	1	0	0	1	1	0	6.67	6.53	7.14	5.95	7.14	1	0	0	1	1	1	1	9.60	9.72	10.01	10.26	10.28	10.49	10.10
1394419_at	Arhgap11a	1	0	0	0	0	1	5.45	5.53	5.81	5.78	5.98	1	0	0	0	0	1	1	10.53	10.53	10.51	10.51	10.41	9.62	9.60
1394459_at	FAM120C	1	1	0	1	0	0	7.07	7.57	7.18	7.62	7.40	1	0	0	0	0	1	1	5.71	5.65	5.93	5.90	5.87	6.69	7.50
1394541_at	---	1	0	1	0	1	1	5.60	5.68	4.80	5.92	4.98	1	0	0	0	0	1	0	4.65	4.61	4.73	5.05	4.85	5.89	3.47
1394578_at	---	1	0	1	0	1	0	8.26	8.25	9.39	7.97	8.60	1	0	0	0	0	1	1	6.49	6.62	6.65	6.82	6.85	7.32	7.11
1394585_at	---	1	1	0	0	0	0	5.90	6.48	5.97	6.21	6.28	1	0	0	0	0	1	1	6.01	5.81	6.13	5.59	5.70	5.31	5.46
1394596_at	---	1	0	1	1	1	0	5.32	5.31	6.01	4.59	5.44	1	0	0	0	0	0	1	7.29	7.41	7.50	7.58	7.51	6.80	5.80
1394600_at	Rgs17	1	0	0	1	1	0	5.71	5.49	6.24	5.10	5.80	1	0	0	0	0	0	1	9.65	9.83	9.79	9.72	9.89	9.46	7.86
1394712_at	---	1	0	0	0	0	1	5.16	4.94	4.76	5.00	4.68	1	0	0	0	0	1	0	5.68	5.94	5.90	5.71	5.90	6.28	5.84
1394726_at	---	1	0	1	0	1	0	3.33	3.27	3.91	3.20	3.49	1	0	0	1	1	1	0	6.51	6.76	6.89	7.22	7.33	7.43	7.10
1394760_at	Unc79	1	0	1	0	1	1	8.02	8.36	8.82	8.03	8.73	1	0	0	0	0	1	1	9.97	9.89	9.99	9.95	9.90	9.16	8.66
1394785_at	---	1	0	0	1	1	0	4.42	4.30	4.68	3.91	4.35	1	0	0	1	1	1	1	5.45	5.55	5.90	6.33	6.30	6.66	6.22
1394816_at	Impa2	1	1	0	0	0	1	5.86	5.29	5.47	5.75	5.19	1	0	0	0	0	1	1	6.14	6.18	6.27	6.24	6.36	6.85	6.71
1394906_at	C6orf120	1	0	1	0	1	1	6.48	6.69	7.18	6.28	7.04	1	0	0	0	0	0	1	8.66	8.67	8.68	8.58	8.55	8.23	7.56
1394954_at	Slc31a2	1	0	1	1	1	0	6.51	6.58	7.34	5.85	6.80	1	0	0	0	0	1	0	6.86	6.97	7.01	7.21	7.18	7.60	6.86
1395015_at	F8	1	0	0	0	0	1	5.64	5.78	6.15	5.83	6.15	1	0	0	0	0	1	0	6.03	5.82	5.95	5.88	5.76	5.28	4.68
1395072_at	---	1	0	0	0	0	1	5.54	5.73	5.53	5.75	6.14	1	0	0	0	0	1	1	6.04	5.90	6.18	5.78	5.85	5.44	5.55
1395081_at	Ncoa7	1	0	0	1	1	0	4.71	4.68	5.19	4.04	5.05	1	0	0	0	0	0	1	9.57	9.63	9.62	9.72	9.82	9.00	8.06
1395132_at	Utrn	1	0	1	0	1	1	5.66	6.07	6.47	5.27	6.31	1	0	0	0	0	1	0	5.64	5.54	5.89	6.04	6.14	6.37	5.96
1395173_at	Caprin1	1	0	1	1	1	1	5.78	5.77	6.51	4.31	5.27	1	0	0	0	0	0	1	9.11	9.01	9.19	9.31	9.20	8.76	7.47
1395240_at	---	1	0	0	0	0	1	3.95	4.16	3.96	4.01	4.56	1	0	0	0	0	0	1	7.57	7.62	7.74	7.57	7.65	7.91	8.22
1395249_at	Snhg11	1	0	0	0	1	0	9.39	9.58	9.94	9.20	9.61	1	0	0	0	1	1	1	4.56	4.92	5.03	4.99	5.25	5.53	5.83
1395255_at	Onecut1	1	0	1	1	1	0	6.97	7.01	6.36	7.78	6.77	1	0	0	0	0	1	1	5.07	5.16	5.52	5.50	5.50	6.24	6.57
1395336_at	Spats2l	1	0	1	0	0	0	6.08	6.32	6.74	6.25	6.36	1	0	0	0	0	1	1	7.03	6.85	6.85	6.61	6.51	5.97	6.28
1395344_at	Mtmr1	1	0	0	0	0	1	5.12	4.89	5.02	4.93	4.42	1	0	0	0	0	1	0	6.46	6.37	6.51	6.36	6.22	5.65	5.15
1395352_at	Cops3	1	0	1	1	1	0	6.72	6.73	7.38	6.08	6.77	1	0	0	0	0	0	1	9.07	9.06	9.09	9.17	9.14	8.89	7.81
1395355_at	Far1	1	0	1	0	1	1	6.61	6.84	7.21	6.38	7.40	1	0	0	0	0	0	1	9.09	9.16	9.20	9.30	9.32	9.57	9.13
1395357_at	Map1b	1	0	1	1	1	0	6.56	6.80	7.51	5.29	6.82	1	0	0	1	0	0	0	8.77	8.69	8.98	9.38	9.19	9.32	8.85
1395362_at	Xpr1	1	0	1	0	1	0	5.92	6.11	6.57	5.86	6.29	1	0	0	0	0	1	1	7.37	7.30	7.58	7.66	7.61	8.10	8.25
1395363_at	Mars1	1	0	0	1	1	1	7.07	6.74	7.15	5.86	6.38	1	0	0	0	0	0	1	9.32	9.41	9.42	9.59	9.52	9.45	7.55
1395409_at	Ptpa	1	0	0	1	1	0	6.08	5.91	6.47	5.26	6.00	1	0	0	0	0	1	1	9.14	9.10	9.15	9.12	8.83	8.54	7.68
1395415_at	Tcaf1	1	0	1	0	1	0	3.77	3.70	4.48	3.44	4.02	1	0	0	1	1	1	0	5.69	5.76	6.19	6.38	6.50	6.83	6.60
1395429_at	---	1	0	0	0	0	1	4.26	4.61	3.86	4.23	3.79	1	0	0	0	0	1	1	6.21	6.11	6.44	6.69	6.56	7.13	6.97
1395519_at	Mturn	1	0	1	0	0	0	4.35	4.18	4.99	4.33	4.13	1	0	0	0	0	1	1	6.36	6.36	6.36	6.29	6.24	7.09	7.42
1395557_at	Klf6	1	1	0	0	0	0	8.05	8.63	7.68	8.01	8.13	1	0	0	0	0	1	0	7.60	7.46	7.54	7.33	7.29	6.80	6.93
1395561_at	LOC499894	1	0	0	1	1	0	8.88	8.95	9.32	8.36	8.75	1	0	0	0	0	0	1	11.75	11.75	11.82	11.82	11.71	11.41	10.39
1395579_at	Dhx32	1	0	1	0	1	1	6.27	6.66	7.18	6.09	6.82	1	0	0	0	0	1	0	8.64	8.69	8.76	8.90	8.93	9.30	8.93
1395614_at	U2af2	1	0	0	1	0	0	5.65	5.43	5.74	5.16	5.35	1	0	0	0	0	0	1	8.21	8.26	8.39	8.51	8.34	8.14	6.69
1395645_at	Dancr	1	1	0	0	0	0	6.78	6.32	6.89	7.13	6.47	1	0	0	0	0	0	1	8.44	8.46	8.51	8.63	8.57	8.02	7.42
1395658_at	---	1	0	1	1	0	1	4.58	4.18	3.95	4.00	3.87	1	0	0	0	0	0	1	5.04	5.02	5.13	5.03	4.95	5.18	5.44
1395948_at	---	1	0	0	1	0	0	6.45	6.72	7.01	7.05	6.91	1	0	0	0	0	1	0	8.18	8.19	8.17	8.29	8.16	7.59	7.34
1395966_at	Kctd14	1	0	0	0	1	0	7.68	7.77	7.17	7.77	7.34	1	0	0	0	0	1	1	7.00	6.99	6.95	7.01	6.72	5.81	6.05
1395990_at	Srcap	1	0	0	0	1	0	5.74	5.75	5.48	6.05	5.98	1	0	0	0	0	0	1	6.91	6.93	6.82	6.82	6.67	6.40	6.19
1396101_at	Stc1	1	0	0	1	0	0	4.51	4.48	4.46	3.97	4.35	1	0	0	0	0	1	0	12.03	12.08	12.20	12.28	12.51	12.76	12.05
1396102_at	---	1	0	0	0	1	0	5.03	4.86	4.79	5.42	4.65	1	0	0	0	0	1	1	7.23	7.21	7.41	7.13	7.09	6.46	5.67
1396209_at	Slc25a53	1	0	0	0	1	0	7.55	7.68	8.02	7.09	7.84	1	0	0	1	1	1	0	7.68	7.73	8.09	8.43	8.34	8.79	8.54
1396217_at	Ap1s2	1	0	1	1	1	0	7.21	7.41	8.25	6.53	7.48	1	0	0	0	0	1	0	10.78	10.91	10.94	11.07	11.26	11.63	10.99
1396225_at	Cpeb2	1	1	0	0	0	0	6.23	5.73	6.18	6.36	6.06	1	0	0	0	0	1	1	5.55	5.25	5.67	5.73	5.61	6.53	7.47
1396300_at	Afmid	1	0	0	0	0	1	3.27	3.68	3.66	3.53	3.94	1	0	0	0	0	1	0	4.15	4.34	4.50	4.45	4.58	4.74	4.85
1396344_at	---	1	0	0	0	0	1	3.30	3.30	3.26	3.36	6.15	1	0	0	0	0	1	0	5.75	5.72	5.88	5.96	5.97	6.38	5.80
1396378_at	---	1	1	0	0	0	1	5.45	6.00	5.94	5.62	6.06	1	0	0	0	0	1	0	6.25	6.45	6.60	6.65	6.63	6.88	6.43
1396451_at	Scn3a	1	0	1	0	1	1	7.21	7.67	8.10	7.35	8.07	1	0	0	1	1	0	0	9.19	9.40	9.55	9.83	9.95	9.73	8.84
1396489_at	---	1	0	0	1	0	1	4.69	4.91	5.18	5.28	5.23	1	0	0	0	0	1	1	7.26	7.06	7.03	6.85	6.70	5.27	4.76
1396556_at	---	1	0	0	1	1	0	7.04	7.01	7.35	6.43	6.75	1	0	0	0	0	1	1	7.56	7.54	7.73	7.42	7.47	6.55	5.84
1396692_at	Pus10	1	0	0	0	1	0	4.52	4.58	4.94	4.17	4.45	1	0	0	0	0	0	1	8.10	8.17	8.20	8.23	8.19	7.76	6.87
1396717_at	---	1	0	0	1	0	0	3.63	3.66	3.72	4.14	3.68	1	0	0	0	0	0	1	4.80	4.58	5.01	4.79	4.88	5.14	4.79
1396771_at	Asxl1	1	0	0	1	1	0	4.37	4.64	4.25	4.98	4.21	1	0	0	0	0	0	1	5.52	5.61	5.69	5.50	5.40	5.00	4.70
1396820_at	Hdac1	1	0	1	1	1	0	6.54	6.53	7.56	5.45	6.75	1	0	0	0	0	1	0	10.21	10.22	10.34	10.49	10.52	10.97	10.28
1397164_at	---	1	0	1	0	1	0	7.99	7.90	6.68	7.62	7.71	1	0	0	0	0	0	1	10.85	10.79	11.14	10.80	11.00	11.02	9.70
1397220_at	LOC100911266	1	0	0	0	1	0	6.20	5.77	6.62	5.73	5.82	1	0	0	0	0	0	1	8.94	8.95	8.98	9.00	8.99	8.39	7.40
1397222_at	Trim39	1	0	0	1	0	0	4.99	5.14	5.07	5.51	5.32	1	0	0	0	0	0	1	7.40	7.37	7.48	7.30	7.33	6.85	6.27
1397356_at	Znf408	1	0	0	0	1	0	4.91	5.18	4.54	5.16	4.78	1	0	0	0	0	0	1	5.85	5.80	5.93	5.88	5.78	5.41	4.78
1397363_at	Nectin3	1	0	1	0	1	1	6.83	6.89	7.62	6.84	7.40	1	0	0	0	0	1	1	8.37	8.30	8.19	8.11	7.87	7.52	7.64
1397377_at	---	1	0	0	0	0	1	4.00	4.40	4.40	4.05	4.47	1	0	0	0	0	1	0	7.59	7.81	7.94	8.05	8.13	8.20	7.80
1397463_at	Rab14	1	1	0	0	0	0	3.16	3.72	3.18	3.23	3.54	1	1	0	0	0	1	1	4.25	3.64	4.73	3.96	4.38	3.58	3.47
1397468_at	Hsdl2	1	0	1	1	1	0	6.42	6.01	7.32	5.49	6.07	1	0	0	0	0	1	1	7.08	7.07	7.23	7.46	7.46	8.13	7.83
1397500_x_at	---	1	0	1	0	0	0	8.27	8.25	7.73	8.10	8.22	1	0	0	0	0	0	1	8.30	8.31	8.36	8.20	8.27	8.61	9.15
1397520_at	Eif4g2	1	0	0	1	1	0	8.49	8.50	8.97	7.67	8.40	1	0	0	0	0	0	1	10.93	10.88	10.98	11.07	10.98	10.50	9.46
1397526_at	Gcdh	1	1	0	1	0	1	7.42	6.76	7.12	6.71	6.38	1	0	0	0	0	1	0	6.84	7.00	7.22	7.21	7.10	7.49	6.33
1397573_at	---	1	0	1	0	0	0	8.67	8.77	9.11	8.57	8.45	1	0	0	0	0	0	1	9.34	9.27	9.50	9.23	9.48	9.54	9.62
1397631_at	---	1	0	1	0	0	0	5.05	4.81	4.29	4.67	4.74	1	0	0	0	0	0	1	7.17	7.07	7.03	6.92	6.86	6.64	5.96
1397644_at	Mtap	1	0	0	1	0	0	5.37	5.29	5.69	5.91	5.53	1	0	0	0	0	1	1	7.23	7.44	7.53	7.67	7.67	8.10	8.01
1397662_at	---	1	0	1	1	1	0	6.08	6.30	7.05	5.44	6.51	1	0	0	0	1	1	1	6.04	6.19	6.41	6.50	6.75	6.82	6.41
1397670_at	Atg12	1	0	1	1	1	0	6.74	6.76	7.47	5.91	6.78	1	0	0	0	0	1	0	9.39	9.49	9.59	9.69	9.72	10.07	9.41
1397676_at	---	1	0	0	1	0	0	7.80	7.83	7.78	8.29	7.67	1	0	0	0	0	0	1	4.89	4.80	4.98	4.75	4.87	4.36	3.42
1397788_at	Rimbp2	1	0	1	0	1	0	8.46	8.75	9.08	8.27	8.71	1	0	0	0	0	1	1	10.22	10.20	10.29	10.29	10.22	9.44	8.67
1397812_at	Senp6	1	0	0	0	1	0	7.43	7.40	7.94	7.07	7.43	1	0	0	0	0	0	1	7.32	7.36	7.50	7.54	7.48	7.04	6.64
1397819_at	Prr14	1	0	0	1	0	0	4.38	4.42	4.32	3.83	3.96	1	0	0	0	0	0	1	6.50	6.44	6.62	6.65	6.46	6.21	5.36
1397960_at	Armcx3	1	0	1	1	1	0	6.83	6.97	7.50	5.72	6.59	1	0	0	0	0	0	1	8.77	8.84	8.84	8.93	8.76	8.38	7.71
1397999_at	---	1	1	0	0	1	0	5.42	6.41	5.65	4.94	5.21	1	0	1	0	1	1	0	9.55	10.01	10.21	10.10	10.23	10.44	9.66
1398107_at	Ggct	1	0	0	1	0	0	7.30	7.74	6.91	6.63	6.91	1	0	0	0	0	0	1	5.72	5.63	5.80	5.49	5.72	6.04	6.08
1398219_at	Tmem215	1	0	1	0	1	1	9.68	10.03	10.63	9.49	10.58	1	0	0	1	1	1	0	5.43	5.61	5.82	6.13	6.10	6.03	5.66
1398295_at	Slc29a1	1	0	0	0	0	1	6.02	5.85	6.06	5.79	5.50	1	0	0	0	0	1	1	8.73	8.85	8.94	9.10	9.26	9.73	9.39
1398358_a_at	Itgb5	1	0	0	0	1	1	7.73	7.35	7.36	7.99	7.15	1	0	0	0	0	1	1	8.38	8.45	8.52	8.52	8.62	9.22	9.03
1398362_at	Notch2	1	0	0	0	1	0	6.05	5.78	5.70	6.49	5.91	1	0	0	0	0	1	1	7.53	7.55	7.57	7.50	7.59	8.29	9.46
1398373_at	B3galnt1	1	0	0	1	1	1	6.34	6.08	6.81	5.83	6.94	1	0	0	0	0	0	1	10.32	10.27	10.38	10.45	10.36	9.80	9.16
1398378_at	Gstk1	1	0	1	0	1	0	8.31	8.12	7.71	8.48	7.87	1	0	0	0	0	1	1	8.35	8.57	8.56	8.62	8.81	9.14	9.06
1398397_at	Arhgap29	1	0	0	0	0	1	8.46	8.22	8.28	8.33	7.90	1	0	0	0	0	0	1	7.57	7.61	7.69	7.63	7.74	8.15	8.47
1398429_at	Pdhx	1	1	0	0	0	0	5.55	5.07	5.82	5.70	5.76	1	0	0	0	0	1	1	8.48	8.46	8.49	8.48	8.37	7.89	7.49
1398431_at	Car8	1	0	1	0	0	1	6.52	6.32	7.37	6.70	7.63	1	0	0	0	0	1	1	4.57	4.66	4.90	4.81	4.91	5.82	6.74
1398483_at	Rgs17	1	0	1	0	1	1	6.68	6.85	7.53	6.31	7.28	1	0	0	0	0	0	1	10.36	10.54	10.47	10.53	10.60	10.44	8.39
1398484_at	Tbc1d9	1	0	0	1	0	0	7.27	7.27	7.66	7.74	7.46	1	0	0	0	0	1	0	7.41	7.26	7.47	7.50	7.23	6.73	7.19
1398511_at	Susd2	1	0	1	0	1	1	8.47	8.37	7.92	8.71	7.93	1	0	0	0	0	1	1	7.82	7.88	7.99	8.12	8.27	8.54	8.04
1398528_at	---	1	0	1	0	1	0	3.58	4.04	4.24	3.48	3.59	1	0	0	0	0	1	1	4.27	4.22	4.17	4.16	4.54	5.64	5.48
1398537_at	Amfr	1	0	0	0	1	0	7.84	7.81	8.41	7.62	7.95	1	0	0	0	0	0	1	9.21	9.18	9.27	9.29	9.24	8.91	7.92
1398582_at	Rps6ka5	1	0	1	0	1	0	4.99	5.00	6.05	5.28	5.43	1	0	0	0	0	0	1	7.08	7.05	7.14	7.15	7.17	7.66	7.69
1398590_at	Sass6	1	0	1	0	0	1	5.17	5.22	5.82	5.19	6.19	1	0	0	0	0	0	1	7.00	6.73	6.94	6.78	6.84	6.44	6.01
1398594_at	---	1	0	1	0	1	0	3.24	3.28	3.86	2.81	3.04	1	0	0	0	0	1	0	5.86	5.97	5.96	6.08	6.26	6.54	3.66
1398595_at	---	1	0	1	0	0	0	5.88	5.54	6.65	6.30	6.16	1	0	0	0	0	0	1	6.51	6.62	6.81	6.64	6.69	6.05	5.13
1398602_at	Mad2l1	1	0	0	0	0	1	5.03	5.33	5.51	5.26	5.60	1	0	0	0	0	1	1	9.03	8.97	8.94	8.90	8.73	7.99	7.83
1398613_at	---	1	0	1	0	1	1	3.68	4.00	4.52	3.57	5.00	1	0	0	0	1	1	1	6.11	6.16	6.34	6.53	6.81	7.60	6.95
1398646_at	Rgs22	1	0	0	1	1	1	7.18	7.11	7.63	6.36	6.20	1	0	0	0	0	0	1	8.04	8.00	8.21	8.31	8.12	7.95	7.03
1398664_at	Gramd2b	1	0	1	1	1	0	7.46	7.47	8.52	6.37	7.90	1	0	0	0	0	0	1	9.87	9.97	9.73	9.64	9.66	9.65	8.08
1398695_at	---	1	0	1	0	0	0	4.27	4.08	4.88	4.39	4.64	1	0	0	0	0	1	1	6.21	6.15	6.55	6.65	6.56	7.47	8.58
1398710_at	Cyp2u1	1	0	0	0	0	1	5.67	5.62	5.79	5.80	6.17	1	0	0	0	0	1	1	6.69	6.70	6.95	7.02	7.07	7.34	7.37
1398825_at	Rab11b	1	0	0	0	1	0	6.97	6.66	7.31	6.52	6.82	1	0	0	0	0	0	1	9.50	9.49	9.56	9.53	9.44	9.07	7.49
1398859_at	Hdlbp	1	0	0	1	1	0	9.42	9.36	9.08	10.00	9.22	1	0	0	0	0	1	1	8.88	8.88	8.95	8.86	8.74	8.09	7.82
1367453_at	Cdc37	1	0	0	1	0	0	9.14	9.38	9.22	9.65	9.11	0	0	0	0	0	0	0	11.50	11.52	11.59	11.53	11.50	11.72	11.44

Probeset ID	Gene Symbol	Islet Data Set (log2 ratio ≥0.263; FDR <20%) union n=5325 1=YES 0=NO	Islet DRlyp/lyp ND vs ND+Lp299v (log2 ratio ≥0.263; FDR<20%) n=429 1=YES 0=NO	Islet DRlyp/lyp ND vs HCD (log2 ratio ≥0.263; FDR<20%) n=2247 1=YES 0=NO	Islet DRlyp/lyp ND vs HCD+Lp299v (log2 ratio ≥0.263; FDR<20%) n=1595 1=YES 0=NO	Islet DRlyp/lyp HCD vs HCD+Lp299v (log2 ratio ≥0.263; FDR<20%) n=3026 1=YES 0=NO	Islet DRlyp/lyp ND vs Flyp/lyp ND (log2 ratio ≥ 0.263; FDR<20%) n=1950 1=YES 0=NO	Islet Mean Log2 Intensity DRlyp/lyp ND	Islet Mean Log2 Intensity DRlyp/lyp ND+Lp299v	Islet Mean Log2 Intensity DRlyp/lyp HCD	Islet Mean Log2 Intensity DRlyp/lyp HCD+Lp299v	Islet Mean Log2 Intensity Flyp40 ND	Rinm5f data union of all conditions n=3705 1=YES 0=NO	Rinm5f 30 µM propionate/ 6 µM butyrate vs Media (FDR<20%; 1.5 fold) n=3 1=YES 0=NO	Rinm5f 60 µM propionate/ 12 µM butyrate vs Media (FDR<20%; 1.5 fold) n=18 1=YES 0=NO	Rinm5f 120 µM propionate/ 24 µM butyrate vs Media (FDR<20%; 1.5 fold) n=164 1=YES 0=NO	Rinm5f 240 µM propionate /48 µM butyrate vs Media (FDR<20%; 1.5 fold) n=268 1=YES 0=NO	Rinm5f 1500 µM propionate/ 300 µM butyrate vs Media (FDR<20%; 1.5 fold) n=2491 1=YES 0=NO	Rinm5f 500 µM propionate/ 500 µM butyrate vs Media (FDR<20%; 1.5 fold) n=3001 1=YES 0=NO	Rinm5f Mean Log2 Intensity Media only	Rinm5f Mean Log2 Intensity 30 µM propionate/ 6 µM butyrate	Rinm5f Mean Log2 Intensity 60 µM propionate/ 12 µM butyrate	Rinm5f Mean Log2 Intensity 120 µM propionate/ 24 µM butyrate	Rinm5f Mean Log2 Intensity 240 µM propionate/ 48 µM butyrate	Rinm5f Mean Log2 Intensity 1500 µM propionate /300 µM butyrate	Rinm5f Mean Log2 Intensity 500 µM propionate/ 500 µM butyrate
1367472_at	Uba1	1	0	0	1	0	0	8.64	8.85	8.61	9.15	8.96	0	0	0	0	0	0	0	10.63	10.51	10.64	10.66	10.58	10.57	10.28
1367507_at	Nudt22	1	0	1	0	0	0	6.47	6.28	5.87	6.28	6.22	0	0	0	0	0	0	0	8.26	8.40	8.22	8.16	8.16	8.10	7.71
1367512_at	Chmp5	1	0	1	0	0	1	10.28	10.36	10.88	10.18	10.81	0	0	0	0	0	0	0	12.46	12.43	12.47	12.46	12.47	12.52	12.16
1367515_at	Cnot7	1	0	1	0	1	0	6.61	6.69	7.68	6.38	7.04	0	0	0	0	0	0	0	10.28	10.46	10.28	10.23	10.40	10.29	9.89
1367527_at	Ipo8	1	0	0	0	0	1	7.39	7.43	7.76	7.69	8.01	0	0	0	0	0	0	0	7.69	7.67	7.84	7.93	7.88	7.68	7.55
1367539_at	Gtf2a1	1	0	1	0	1	0	7.18	7.18	7.80	7.08	7.37	0	0	0	0	0	0	0	9.83	9.79	9.92	10.01	9.97	9.82	9.70
1367543_at	Sys1	1	0	0	0	0	1	8.52	8.36	8.28	8.72	8.03	0	0	0	0	0	0	0	9.96	9.91	9.95	9.88	9.85	9.64	9.29
1367545_at	Rabgap1	1	0	0	0	1	0	7.01	6.95	7.57	6.69	7.33	0	0	0	0	0	0	0	8.62	8.66	8.68	8.67	8.75	8.78	8.61
1367546_at	Mrpl43	1	0	0	0	1	0	8.71	8.64	8.49	9.07	8.42	0	0	0	0	0	0	0	11.09	11.07	11.13	11.08	11.13	11.17	10.70
1367547_at	Zmym2	1	0	1	0	1	0	8.03	8.12	8.70	7.85	8.13	0	0	0	0	0	0	0	8.75	8.73	8.77	8.92	8.74	8.37	7.96
1367550_a_at	Tm2d1	1	0	1	0	1	0	6.83	6.60	7.80	6.72	7.19	0	0	0	0	0	0	0	10.15	10.25	10.17	10.08	10.19	10.12	9.60
1367553_x_at	Hbb	1	1	0	1	1	1	10.23	9.74	9.96	9.16	9.22	0	0	0	0	0	0	0	6.11	6.19	6.03	5.91	5.95	6.03	5.58
1367555_at	Alb	1	0	1	1	0	1	4.39	4.56	5.09	5.45	6.64	0	0	0	0	0	0	0	3.61	3.66	3.63	3.64	3.50	3.62	3.15
1367556_s_at	Alb	1	0	0	0	0	1	5.52	5.63	5.49	5.60	6.09	0	0	0	0	0	0	0	4.38	4.26	4.23	4.19	4.19	4.18	3.76
1367562_at	Sparc	1	0	0	0	0	1	7.51	7.07	7.47	7.11	6.98	0	0	0	0	0	0	0	3.92	3.99	3.82	3.80	3.76	3.80	3.42
1367563_at	Sparc	1	1	0	1	0	0	9.98	9.52	10.05	9.48	9.59	0	0	0	0	0	0	0	6.65	6.54	6.55	6.36	6.35	6.45	6.33
1367568_a_at	Mgp	1	0	1	0	1	0	10.92	10.80	11.73	10.60	11.04	0	0	0	0	0	0	0	4.26	4.14	4.20	3.97	4.08	4.11	3.63
1367569_at	Rpsa	1	0	0	0	0	1	12.32	12.20	12.51	12.69	11.62	0	0	0	0	0	0	0	12.12	12.19	12.15	12.15	12.09	12.16	11.79
1367570_at	Tagln	1	0	1	0	1	1	6.55	6.91	5.87	6.89	5.95	0	0	0	0	0	0	0	3.84	3.93	3.62	3.63	3.57	3.66	3.30
1367574_at	Vim	1	0	0	0	0	1	8.62	8.61	8.27	8.50	8.09	0	0	0	0	0	0	0	3.83	3.79	3.90	3.75	3.87	3.92	4.36
1367576_at	Gpx1	1	0	1	1	0	0	11.66	11.64	9.91	10.44	11.22	0	0	0	0	0	0	0	5.13	5.12	5.21	4.90	5.08	5.19	4.40
1367580_at	Rpl10a	1	0	0	1	0	0	11.81	11.59	11.89	12.30	11.37	0	0	0	0	0	0	0	12.13	12.14	12.16	12.19	12.18	12.15	11.75
1367581_a_at	Spp1	1	1	0	0	0	1	7.37	6.89	7.53	7.45	6.76	0	0	0	0	0	0	0	5.11	5.04	5.03	4.93	4.93	5.05	4.58
1367582_at	Rpl29	1	0	0	1	0	0	11.67	11.56	11.89	12.16	11.36	0	0	0	0	0	0	0	12.33	12.31	12.31	12.34	12.33	12.41	12.13
1367586_at	Ldha /// RGD156	1	0	0	0	1	0	8.43	8.15	7.95	8.63	8.31	0	0	0	0	0	0	0	13.07	13.15	13.01	12.73	12.83	12.80	13.09
1367618_a_at	Rack1	1	0	0	1	0	0	12.25	12.18	12.53	12.79	11.88	0	0	0	0	0	0	0	11.92	11.93	11.94	11.92	11.93	12.08	12.06
1367619_at	Pgrmc1	1	0	1	0	1	0	10.29	10.39	10.95	9.97	10.65	0	0	0	0	0	0	0	11.82	11.82	11.88	11.91	11.94	11.77	11.45
1367621_at	Dapk3	1	0	0	0	1	1	8.72	8.89	8.23	9.11	8.26	0	0	0	0	0	0	0	9.70	9.72	9.72	9.72	9.65	9.85	9.71
1367623_at	Rpl18	1	0	0	1	0	0	12.28	12.21	12.43	12.76	11.95	0	0	0	0	0	0	0	13.05	13.07	13.08	13.12	13.07	13.12	12.89
1367631_at	Ccn2	1	0	0	1	0	0	7.56	7.45	7.58	6.98	7.59	0	0	0	0	0	0	0	3.65	3.68	3.64	3.70	3.61	3.70	3.09
1367649_at	Palm	1	0	1	0	0	0	5.31	4.94	4.75	5.03	5.02	0	0	0	0	0	0	0	7.59	7.56	7.72	7.67	7.68	7.88	7.45
1367653_a_at	Mdh1	1	0	0	0	0	1	10.91	11.09	11.41	10.75	11.60	0	0	0	0	0	0	0	12.90	12.89	12.88	12.86	12.86	12.99	12.86
1367659_s_at	Eci1	1	0	0	0	0	1	8.30	8.07	8.02	8.40	7.63	0	0	0	0	0	0	0	7.59	7.63	7.74	7.63	7.67	7.82	7.94
1367662_at	Hsd17b10	1	0	0	0	0	1	9.26	8.98	8.92	9.24	8.61	0	0	0	0	0	0	0	9.56	9.61	9.63	9.70	9.61	9.45	9.24
1367666_at	Hnrnph1	1	0	1	0	1	0	10.18	10.17	10.88	10.17	10.33	0	0	0	0	0	0	0	12.23	12.26	12.30	12.37	12.35	12.07	11.38
1367667_at	Fdps	1	0	1	0	0	0	9.04	8.87	8.46	8.89	8.96	0	0	0	0	0	0	0	10.18	10.32	10.39	10.47	10.54	10.44	10.22
1367668_a_at	Scd2	1	0	1	0	0	1	8.22	8.57	8.92	8.28	9.29	0	0	0	0	0	0	0	12.58	12.59	12.55	12.35	12.51	12.28	11.79
1367672_at	Hsd17b4	1	1	0	0	0	0	8.07	7.58	8.03	7.81	7.97	0	0	0	0	0	0	0	9.75	9.74	9.82	9.81	9.75	9.79	9.60
1367687_a_at	Pam	1	0	0	0	1	0	10.55	10.50	11.05	10.31	11.01	0	0	0	0	0	0	0	13.06	13.10	13.10	13.02	13.09	12.81	12.41
1367695_at	Qdpr	1	0	1	0	0	0	8.52	8.43	9.15	8.58	8.89	0	0	0	0	0	0	0	10.00	9.95	9.98	10.07	10.03	10.56	10.65
1367696_at	Ifitm2	1	0	0	0	1	1	8.74	8.60	8.30	8.91	8.24	0	0	0	0	0	0	0	12.27	12.33	12.30	12.30	12.31	12.16	11.70
1367725_at	Pim3	1	1	1	1	0	1	9.18	8.31	8.24	8.33	8.24	0	0	0	0	0	0	0	9.82	9.85	9.94	9.94	9.91	9.98	9.93
1367726_at	Thra	1	0	0	0	1	0	6.98	6.93	6.70	7.29	6.87	0	0	0	0	0	0	0	7.43	7.49	7.61	7.50	7.62	7.89	7.65
1367730_at	Uso1	1	0	0	0	0	1	9.10	9.06	9.56	8.88	9.59	0	0	0	0	0	0	0	10.72	10.75	10.80	10.77	10.77	10.64	10.60
1367732_at	Gnb1	1	0	0	0	1	0	8.28	8.27	8.78	7.89	8.34	0	0	0	0	0	0	0	10.14	10.14	10.14	10.14	10.10	9.79	9.60
1367735_at	Acadl	1	1	0	0	0	0	7.90	7.39	8.30	8.00	7.50	0	0	0	0	0	0	0	9.30	9.27	9.35	9.38	9.35	9.65	9.79
1367741_at	Herpud1	1	1	1	1	0	1	10.41	9.46	9.80	9.89	9.92	0	0	0	0	0	0	0	9.46	9.45	9.45	9.45	9.50	9.55	9.03
1367749_at	Lum	1	1	0	1	1	1	6.63	5.94	6.77	5.62	5.82	0	0	0	0	0	0	0	3.36	3.24	3.47	3.28	3.41	3.38	2.87
1367755_at	Cdo1	1	0	1	0	0	0	7.51	7.77	8.16	7.53	7.89	0	0	0	0	0	0	0	7.50	7.53	7.57	7.44	7.47	7.86	7.71
1367760_at	Map2k1	1	0	0	0	1	0	8.46	8.41	8.98	8.20	8.77	0	0	0	0	0	0	0	9.73	9.74	9.64	9.60	9.59	9.81	9.68
1367762_at	Sst	1	0	0	1	1	1	11.39	11.71	11.90	10.88	12.53	0	0	0	0	0	0	0	4.54	4.37	4.45	4.21	4.28	4.35	5.21
1367767_at	Hmgcl	1	0	0	0	0	1	7.80	7.47	7.45	7.75	7.23	0	0	0	0	0	0	0	9.34	9.52	9.47	9.41	9.37	9.73	9.40
1367774_at	Gsta1	1	1	0	1	1	1	6.56	5.98	6.90	7.61	6.00	0	0	0	0	0	0	0	3.80	3.75	3.76	3.76	3.71	3.89	3.81
1367778_at	Furin	1	0	0	0	1	0	7.96	7.97	7.65	8.29	7.95	0	0	0	0	0	0	0	8.37	8.38	8.47	8.31	8.36	8.26	7.92
1367783_at	Gabarapl2	1	0	0	0	1	0	10.58	10.52	11.14	10.28	10.73	0	0	0	0	0	0	0	11.24	11.24	11.28	11.28	11.29	11.55	11.51
1367784_a_at	Clu	1	0	0	1	0	0	11.49	11.15	11.13	10.97	11.05	0	0	0	0	0	0	0	12.47	12.55	12.59	12.58	12.64	12.90	12.23
1367790_at	Snd1	1	0	0	0	1	0	9.28	9.12	9.07	9.74	9.18	0	0	0	0	0	0	0	10.40	10.38	10.50	10.61	10.61	10.80	10.68
1367793_at	Ddt	1	0	0	0	0	1	9.60	9.34	9.42	9.88	8.97	0	0	0	0	0	0	0	9.64	9.62	9.67	9.68	9.70	10.02	9.80
1367795_at	Ifrd1	1	0	1	0	1	0	9.84	9.45	9.04	9.60	9.45	0	0	0	0	0	0	0	9.44	9.51	9.64	9.85	9.78	9.27	9.09
1367798_at	Ahcy	1	1	0	0	0	1	10.53	9.98	10.13	10.34	9.60	0	0	0	0	0	0	0	10.40	10.32	10.39	10.51	10.43	10.23	9.81
1367811_at	Phgdh	1	0	1	0	1	1	9.45	9.20	8.64	9.66	8.84	0	0	0	0	0	0	0	4.11	4.09	4.11	4.05	4.04	4.02	3.93
1367818_at	Coq3	1	1	0	0	0	0	6.67	6.19	6.48	6.83	6.45	0	0	0	0	0	0	0	7.71	7.79	7.80	7.79	7.71	7.51	7.41
1367826_at	Nfe2l2	1	0	0	1	0	0	7.75	7.64	8.13	8.35	7.75	0	0	0	0	0	0	0	8.91	8.90	8.97	9.03	8.98	9.10	9.24
1367828_at	Acads	1	0	1	0	0	1	8.47	8.28	7.92	8.40	7.89	0	0	0	0	0	0	0	8.79	8.91	8.94	8.81	8.86	9.17	9.09
1367829_at	Echs1	1	1	0	0	0	1	8.98	8.51	8.48	8.70	8.49	0	0	0	0	0	0	0	9.20	9.21	9.12	9.09	9.04	8.97	8.64
1367834_at	Srm	1	0	1	0	0	0	9.14	8.89	8.61	9.11	8.89	0	0	0	0	0	0	0	10.88	10.89	10.93	10.95	10.86	10.50	10.02
1367839_at	Fdft1	1	0	0	0	1	0	7.88	8.03	7.53	8.11	7.84	0	0	0	0	0	0	0	7.94	7.97	8.04	8.18	8.18	8.30	8.11
1367843_at	Akr7a2	1	0	0	0	1	1	8.15	8.24	7.80	8.51	7.64	0	0	0	0	0	0	0	8.78	8.71	8.80	8.80	8.70	8.91	8.92
1367849_at	Sdc1	1	0	1	0	1	0	6.50	6.56	5.91	6.93	6.42	0	0	0	0	0	0	0	10.47	10.54	10.62	10.61	10.70	10.62	10.31
1367851_at	Ptgds	1	0	0	0	0	1	5.25	5.46	5.31	5.44	5.96	0	0	0	0	0	0	0	6.15	6.34	6.50	6.44	6.67	6.60	6.00
1367854_at	Acly	1	0	1	0	0	1	11.09	11.31	11.69	11.22	11.59	0	0	0	0	0	0	0	12.79	12.78	12.79	12.72	12.72	12.88	12.82
1367855_at	Scarb1	1	0	0	1	0	0	6.78	6.60	6.81	6.23	6.73	0	0	0	0	0	0	0	8.52	8.49	8.62	8.58	8.48	8.56	8.04
1367857_at	Fads1	1	0	0	1	1	0	8.57	8.66	8.30	9.12	8.66	0	0	0	0	0	0	0	10.34	10.31	10.28	10.28	10.28	10.43	10.40
1367863_at	Bnip1	1	0	0	0	1	0	6.88	6.62	6.57	7.14	6.69	0	0	0	0	0	0	0	8.35	8.36	8.34	8.22	8.13	8.05	7.55
1367876_at	Ipo13	1	0	0	1	1	0	5.65	5.79	5.58	6.20	5.87	0	0	0	0	0	0	0	8.65	8.58	8.70	8.71	8.76	8.69	8.90
1367877_at	Slc11a2	1	0	0	1	1	0	8.00	7.74	7.84	8.83	8.03	0	0	0	0	0	0	0	8.33	8.34	8.43	8.50	8.45	8.30	8.00
1367878_at	Stx5	1	0	0	0	1	0	8.50	8.66	8.27	8.84	8.49	0	0	0	0	0	0	0	10.18	10.14	10.12	10.04	10.01	9.81	9.31
1367880_at	Lamb2	1	0	0	0	0	1	5.31	5.32	4.93	5.24	4.81	0	0	0	0	0	0	0	3.95	4.07	3.97	4.01	3.92	4.11	4.03
1367887_at	Lcat	1	0	0	0	1	0	4.23	4.10	3.88	4.56	4.24	0	0	0	0	0	0	0	5.41	5.42	5.45	5.66	5.58	5.49	5.23
1367890_at	Casp2	1	0	1	0	0	0	6.67	6.64	7.33	6.70	6.71	0	0	0	0	0	0	0	9.38	9.41	9.51	9.56	9.59	9.57	9.57
1367891_a_at	Casp2	1	0	1	0	1	0	4.91	4.96	5.43	4.61	4.68	0	0	0	0	0	0	0	7.32	7.29	7.46	7.53	7.55	7.50	6.62
1367895_at	Hnrnpk	1	0	1	1	1	0	9.89	9.83	10.49	9.38	10.02	0	0	0	0	0	0	0	12.09	12.12	12.12	12.16	12.12	11.95	11.42
1367897_at	Acadvl	1	0	0	0	0	1	7.33	7.13	7.16	7.39	6.87	0	0	0	0	0	0	0	8.50	8.58	8.64	8.71	8.68	8.83	8.41
1367902_at	Gng11	1	0	0	1	0	1	7.66	7.23	7.29	7.05	7.17	0	0	0	0	0	0	0	3.48	3.47	3.46	3.30	3.35	3.28	2.87
1367904_at	Resp18	1	0	0	0	1	0	13.00	13.06	13.41	12.64	13.36	0	0	0	0	0	0	0	13.44	13.48	13.46	13.48	13.51	13.57	13.06
1367908_at	Gcsh	1	0	0	0	1	0	9.65	9.60	9.31	9.88	9.32	0	0	0	0	0	0	0	9.04	9.09	9.06	9.10	9.12	9.57	9.54
1367910_at	Smad4	1	0	1	0	1	0	5.96	6.08	6.57	5.71	5.74	0	0	0	0	0	0	0	8.96	8.89	8.91	8.96	8.87	8.75	7.93
1367915_at	Dgat1	1	0	0	0	1	0	7.97	7.83	7.60	8.17	7.57	0	0	0	0	0	0	0	9.17	9.25	9.28	9.27	9.28	9.19	8.73
1367917_at	Cyp2d26	1	0	1	1	1	1	7.91	7.71	6.81	8.39	3.78	0	0	0	0	0	0	0	4.34	4.44	4.34	4.16	4.36	4.34	4.12
1367927_at	Phb	1	0	0	0	0	1	9.25	8.82	9.19	9.40	8.78	0	0	0	0	0	0	0	10.54	10.61	10.59	10.66	10.57	10.64	10.25
1367939_at	Rbp1	1	0	1	0	0	0	8.03	8.32	8.64	8.01	7.95	0	0	0	0	0	0	0	9.51	9.55	9.51	9.37	9.48	9.72	9.69
1367941_at	Tfam	1	0	1	0	0	0	8.11	7.99	8.75	8.15	8.10	0	0	0	0	0	0	0	10.52	10.55	10.55	10.63	10.61	10.83	10.54
1367956_at	Ncdn	1	0	0	1	0	1	4.33	4.76	4.54	4.83	5.24	0	0	0	0	0	0	0	7.84	7.79	8.02	8.02	7.94	8.08	7.24
1367970_at	Pfn2	1	0	1	1	1	0	5.82	6.03	6.96	4.72	5.98	0	0	0	0	0	0	0	10.02	10.10	10.15	10.21	10.16	10.18	9.23
1367974_at	Anxa3	1	0	1	1	0	1	5.93	5.66	4.97	5.30	5.46	0	0	0	0	0	0	0	3.94	3.97	3.92	3.81	3.92	4.05	4.01
1367980_at	Rabep1	1	0	1	0	1	0	7.11	7.21	7.75	7.04	7.32	0	0	0	0	0	0	0	8.28	8.23	8.32	8.42	8.40	8.50	8.60
1367981_at	Rabep1	1	0	1	0	1	0	5.97	6.26	6.67	5.56	6.11	0	0	0	0	0	0	0	7.12	7.13	7.38	7.48	7.31	7.41	7.37
1367986_at	Ptgfrn	1	0	0	0	1	0	5.80	5.67	5.42	6.25	5.60	0	0	0	0	0	0	0	7.09	7.14	7.23	7.27	7.20	7.18	7.42
1367992_at	Scg5	1	0	1	0	1	0	12.32	12.47	12.91	11.90	12.71	0	0	0	0	0	0	0	12.77	12.76	12.78	12.78	12.86	13.06	12.85
1368004_at	Mrpl23	1	0	0	1	1	0	9.22	9.14	9.15	9.78	8.92	0	0	0	0	0	0	0	10.71	10.75	10.75	10.77	10.71	10.45	10.05
1368005_at	Itpr3	1	0	0	0	1	0	6.84	6.79	6.71	7.29	6.61	0	0	0	0	0	0	0	9.00	8.96	8.95	8.81	8.92	9.07	9.33
1368007_at	Dmbt1	1	0	1	0	1	1	6.03	5.90	5.18	5.80	5.37	0	0	0	0	0	0	0	4.88	4.97	4.86	4.76	4.76	5.01	4.61
1368021_at	Adh1 /// Adh6	1	0	0	1	1	1	5.34	5.30	4.96	5.95	4.47	0	0	0	0	0	0	0	4.19	4.23	4.18	3.96	4.02	4.11	3.38
1368023_at	Qsox1	1	0	1	0	1	1	8.92	8.55	7.99	9.16	8.21	0	0	0	0	0	0	0	8.52	8.58	8.49	8.49	8.58	8.88	8.57
1368024_at	Qsox1	1	0	1	0	1	1	8.58	8.15	7.81	8.43	7.87	0	0	0	0	0	0	0	8.47	8.56	8.46	8.48	8.57	8.90	8.12
1368025_at	Ddit4	1	0	1	1	0	0	8.56	8.60	7.46	7.34	8.63	0	0	0	0	0	0	0	9.91	9.97	10.02	9.73	9.92	10.27	9.75
1368030_at	Gnai3	1	0	0	1	1	0	6.97	6.82	7.39	5.47	6.71	0	0	0	0	0	0	0	8.88	8.93	8.96	9.08	9.12	9.31	8.80
1368032_at	Nolc1	1	0	1	0	0	0	6.09	6.00	6.72	6.12	6.26	0	0	0	0	0	0	0	10.11	10.08	10.19	10.26	10.17	9.86	9.36
1368034_at	Chgb	1	0	1	0	1	0	9.11	9.21	10.17	9.10	9.26	0	0	0	0	0	0	0	14.43	14.47	14.46	14.43	14.46	14.47	14.14
1368040_at	Synj2bp	1	0	0	1	1	0	6.24	6.18	6.58	5.75	6.26	0	0	0	0	0	0	0	8.96	9.04	9.07	9.12	9.07	9.00	8.48
1368044_at	Scg2	1	0	1	0	1	1	12.42	12.52	13.04	12.14	12.93	0	0	0	0	0	0	0	11.65	11.78	11.72	11.81	12.01	11.50	10.48
1368050_at	Ccnl1 /// LOC10	1	1	0	0	0	0	7.42	7.93	7.69	7.31	7.59	0	0	0	0	0	0	0	9.68	9.72	9.72	9.80	9.77	9.31	8.74
1368051_at	Hsd17b12	1	0	0	0	0	1	8.16	8.36	8.57	8.45	8.79	0	0	0	0	0	0	0	10.30	10.28	10.32	10.37	10.34	10.09	10.09
1368052_at	Tspan8	1	0	0	0	0	1	6.99	6.53	6.96	7.22	6.34	0	0	0	0	0	0	0	3.36	3.32	3.37	3.28	3.36	3.32	3.10
1368053_at	Pard3	1	0	0	1	0	0	5.51	5.53	5.55	6.00	5.51	0	0	0	0	0	0	0	5.91	5.77	6.06	5.95	5.82	5.61	5.94
1368054_at	Lmna	1	0	1	1	1	0	6.89	7.10	6.36	7.66	6.81	0	0	0	0	0	0	0	8.16	8.18	8.22	8.29	8.19	7.85	7.52
1368064_a_at	Ddc	1	0	1	0	1	0	8.72	8.96	9.47	8.69	9.18	0	0	0	0	0	0	0	11.71	11.66	11.77	11.75	11.60	11.29	11.26
1368071_at	Mtarc2	1	0	1	0	0	1	9.50	9.09	8.94	9.38	8.65	0	0	0	0	0	0	0	7.20	7.28	7.46	7.56	7.53	7.65	7.72
1368072_at	Btg3	1	0	1	0	1	1	8.06	8.18	8.81	7.76	8.64	0	0	0	0	0	0	0	11.20	11.26	11.25	11.24	11.24	11.25	11.13
1368077_at	Fbp1	1	1	0	0	0	1	7.39	6.92	6.98	7.53	6.64	0	0	0	0	0	0	0	4.55	4.45	4.44	4.27	4.37	4.49	4.22
1368078_at	Esm1	1	0	1	0	1	1	6.80	6.61	7.51	6.49	7.96	0	0	0	0	0	0	0	3.45	3.42	3.36	3.45	3.37	3.34	2.87
1368080_at	Rgcc	1	1	0	1	1	0	8.13	7.30	8.42	7.22	7.78	0	0	0	0	0	0	0	5.19	5.37	5.15	4.92	4.85	4.98	5.53
1368082_at	Slc4a2	1	0	1	0	1	0	6.59	6.58	6.05	7.01	6.78	0	0	0	0	0	0	0	8.29	8.24	8.37	8.33	8.28	8.34	7.82
1368087_a_at	Ptpn21	1	0	0	1	1	0	5.61	5.58	5.44	6.38	5.76	0	0	0	0	0	0	0	6.85	6.87	6.98	7.10	7.01	7.02	7.24
1368091_at	Oplah	1	0	1	0	1	1	6.52	6.48	5.72	6.97	6.05	0	0	0	0	0	0	0	5.68	5.49	5.88	5.68	5.71	5.93	6.06
1368103_at	Abcg1	1	0	1	0	1	1	8.17	8.20	8.77	8.07	8.74	0	0	0	0	0	0	0	10.47	10.46	10.53	10.42	10.46	10.68	10.39
1368115_at	Cldn3	1	0	1	0	1	0	8.20	8.06	7.58	8.38	7.75	0	0	0	0	0	0	0	7.72	7.74	7.88	7.86	7.81	8.13	8.62
1368117_at	Gphn	1	0	1	0	0	0	6.54	6.55	7.12	6.65	6.90	0	0	0	0	0	0	0	8.84	8.88	8.90	8.93	8.88	8.78	8.63
1368119_at	Inpp5j	1	0	0	0	1	0	6.16	6.39	5.86	6.55	6.02	0	0	0	0	0	0	0	8.30	8.33	8.31	8.19	8.18	8.40	8.34
1368121_at	Akr7a3	1	0	0	0	1	0	5.39	5.30	4.94	5.51	5.12	0	0	0	0	0	0	0	4.82	4.80	4.84	4.72	4.66	5.04	5.69
1368126_at	Aacs	1	0	1	0	1	0	7.66	7.75	8.50	7.79	7.97	0	0	0	0	0	0	0	8.94	8.92	9.07	9.20	9.10	8.99	8.76
1368128_at	Pla2g2a	1	1	1	1	0	1	4.96	4.45	4.05	4.30	4.33	0	0	0	0	0	0	0	5.74	5.56	5.53	5.28	5.39	5.28	5.01
1368133_at	Mpdz	1	0	1	1	1	1	6.23	6.38	7.27	5.66	6.72	0	0	0	0	0	0	0	7.49	7.51	7.65	7.72	7.66	7.66	7.27
1368140_at	Wnk1	1	0	0	1	1	0	5.42	5.22	5.55	4.86	5.28	0	0	0	0	0	0	0	6.42	6.14	6.29	6.32	6.20	6.12	5.49
1368141_at	Cnbp	1	0	0	0	1	0	10.49	10.23	10.74	10.03	10.46	0	0	0	0	0	0	0	12.39	12.41	12.42	12.44	12.45	12.36	12.02

Probeset ID	Gene Symbol	Islet Data Set (log2 ratio ≥0.263; FDR <20%) union n=5325 1=YES 0=NO	Islet DRlyp/lyp ND vs ND+Lp299v (log2 ratio ≥0.263; FDR<20%) n=429 1=YES 0=NO	Islet DRlyp/lyp ND vs HCD (log2 ratio ≥0.263; FDR<20%) n=2247 1=YES 0=NO	Islet DRlyp/lyp ND vs HCD+Lp299v (log2 ratio ≥0.263; FDR<20%) n=1595 1=YES 0=NO	Islet DRlyp/lyp HCD vs HCD+Lp299v (log2 ratio ≥0.263; FDR<20%) n=3026 1=YES 0=NO	Islet DRlyp/lyp ND vs Flyp/lyp ND (log2 ratio ≥ 0.263; FDR<20%) n=1950 1=YES 0=NO	Islet Mean Log2 Intensity DRlyp/lyp ND	Islet Mean Log2 Intensity DRlyp/lyp ND+Lp299v	Islet Mean Log2 Intensity DRlyp/lyp HCD	Islet Mean Log2 Intensity DRlyp/lyp HCD+Lp299v	Islet Mean Log2 Intensity Flyp40 ND	Rinm5f data union of all conditions n=3705 1=YES 0=NO	Rinm5f 30 µM propionate/6 µM butyrate vs Media (FDR<20%; 1.5 fold) n=3 1=YES 0=NO	Rinm5f 60 µM propionate/12 µM butyrate vs Media (FDR<20%; 1.5 fold) n=18 1=YES 0=NO	Rinm5f 120 µM propionate/24 µM butyrate vs Media (FDR<20%; 1.5 fold) n=164 1=YES 0=NO	Rinm5f 240 µM propionate /48 µM butyrate vs Media (FDR<20%; 1.5 fold) n=268 1=YES 0=NO	Rinm5f 1500 µM propionate/300 µM butyrate vs Media (FDR<20%; 1.5 fold) n=2491 1=YES 0=NO	Rinm5f 500 µM propionate/500 µM butyrate vs Media (FDR<20%; 1.5 fold) n=3001 1=YES 0=NO	Rinm5f Mean Log2 Intensity Media only	Rinm5f Mean Log2 Intensity 30 µM propionate/6 µM butyrate	Rinm5f Mean Log2 Intensity 60 µM propionate/12 µM butyrate	Rinm5f Mean Log2 Intensity 120 µM propionate/24 µM butyrate	Rinm5f Mean Log2 Intensity 240 µM propionate/48 µM butyrate	Rinm5f Mean Log2 Intensity 1500 µM propionate /300 µM butyrate	Rinm5f Mean Log2 Intensity 500 µM propionate/500 µM butyrate
1368146_at	Dusp1	1	0	0	1	1	1	9.56	9.79	9.70	8.62	9.00	0	0	0	0	0	0	0	8.61	8.61	8.67	8.46	8.46	9.05	8.91
1368147_at	Dusp1	1	1	0	1	1	0	6.53	7.30	7.03	4.72	6.09	0	0	0	0	0	0	0	5.93	5.99	6.01	5.87	6.05	6.32	5.68
1368151_at	Matr3	1	0	1	0	0	0	10.03	10.09	10.60	10.00	10.29	0	0	0	0	0	0	0	12.14	12.19	12.22	12.31	12.28	12.28	11.87
1368157_at	Stmn3	1	0	0	0	0	1	5.76	6.11	6.13	5.65	6.27	0	0	0	0	0	0	0	10.83	10.90	10.90	10.96	10.94	10.65	10.01
1368159_at	Abcb6	1	0	1	0	0	0	7.97	7.85	7.44	7.88	7.66	0	0	0	0	0	0	0	8.08	8.07	8.31	8.29	8.24	8.35	8.13
1368162_at	Cst6	1	0	1	0	1	0	10.27	10.43	9.74	10.50	10.00	0	0	0	0	0	0	0	11.79	11.78	11.79	11.74	11.65	11.48	11.53
1368165_at	Prps1	1	0	0	0	1	1	8.65	8.69	9.13	8.39	9.31	0	0	0	0	0	0	0	11.09	11.05	11.13	11.22	11.19	11.32	10.98
1368168_at	Slc34a2	1	0	0	1	1	0	6.93	6.53	6.87	7.80	6.67	0	0	0	0	0	0	0	4.44	4.34	4.52	4.41	4.36	4.40	3.55
1368182_at	Acsl6	1	0	0	0	0	1	5.68	6.05	6.12	5.62	6.67	0	0	0	0	0	0	0	7.16	7.29	7.39	7.41	7.45	7.07	6.84
1368186_a_at	Syk	1	0	1	0	0	0	6.01	5.88	5.48	5.91	5.90	0	0	0	0	0	0	0	5.79	5.67	5.71	5.61	5.59	5.75	5.53
1368194_at	Agpat4	1	0	1	0	1	0	7.69	7.80	8.36	7.46	7.76	0	0	0	0	0	0	0	9.38	9.42	9.51	9.67	9.62	9.73	9.36
1368201_at	Npr1	1	0	1	0	0	0	5.29	5.14	4.66	4.84	4.96	0	0	0	0	0	0	0	5.79	5.88	5.99	5.96	5.99	5.74	5.18
1368205_at	Cfi	1	0	0	0	0	1	3.88	3.69	3.41	3.85	5.10	0	0	0	0	0	0	0	3.91	3.96	3.90	3.77	3.87	3.85	3.50
1368208_at	Nat8b	1	0	1	0	1	1	9.88	9.72	8.87	9.75	9.28	0	0	0	0	0	0	0	7.92	8.00	8.03	7.95	7.96	8.08	7.85
1368212_at	Srsf11	1	0	1	0	1	0	8.67	8.63	9.24	8.55	8.95	0	0	0	0	0	0	0	11.01	11.08	11.09	11.14	11.22	11.20	10.80
1368223_at	Adamts1	1	0	0	1	0	1	5.72	6.05	5.70	5.18	5.07	0	0	0	0	0	0	0	4.37	4.50	4.33	4.43	4.41	4.49	4.53
1368227_at	Slc28a2	1	0	0	0	0	1	6.01	5.80	5.64	5.78	5.55	0	0	0	0	0	0	0	4.41	4.47	4.38	4.35	4.33	4.31	4.22
1368245_at	Upb1	1	0	1	0	1	0	7.07	7.34	7.70	6.74	7.31	0	0	0	0	0	0	0	4.43	4.33	4.46	4.31	4.44	4.20	4.54
1368249_at	Klf15	1	1	1	1	0	1	8.72	7.57	8.18	8.06	7.75	0	0	0	0	0	0	0	3.48	3.44	3.56	3.50	3.53	3.51	3.32
1368251_at	Jak3	1	0	1	0	1	0	6.47	6.35	5.69	6.59	6.08	0	0	0	0	0	0	0	6.79	6.78	6.92	6.78	6.81	6.60	5.76
1368265_at	Cyp2t4	1	0	1	1	1	1	7.61	7.57	6.97	8.24	6.69	0	0	0	0	0	0	0	5.04	5.10	5.17	5.25	5.23	4.64	4.50
1368266_at	Arg1	1	0	1	0	1	0	7.82	8.04	8.74	7.60	7.57	0	0	0	0	0	0	0	8.59	8.51	8.70	8.95	8.86	8.79	8.64
1368271_a_at	Fabp4	1	1	1	1	0	1	8.19	7.32	7.30	6.86	7.03	0	0	0	0	0	0	0	3.39	3.54	3.42	3.33	3.35	3.32	2.74
1368276_at	Syp	1	0	0	0	0	1	8.00	8.41	8.49	8.36	8.48	0	0	0	0	0	0	0	9.24	9.18	9.34	9.28	9.25	9.13	9.13
1368277_at	Ppp3ca	1	0	0	1	1	0	9.80	9.79	9.69	10.27	9.57	0	0	0	0	0	0	0	10.54	10.54	10.64	10.65	10.61	10.74	10.68
1368278_at	Lgals2	1	0	1	1	1	1	7.90	8.02	7.37	8.39	7.20	0	0	0	0	0	0	0	4.69	4.61	4.57	4.53	4.64	4.49	5.94
1368280_at	Ctsc	1	0	0	0	0	1	8.65	8.20	8.51	8.73	7.86	0	0	0	0	0	0	0	4.43	4.41	4.63	4.38	4.40	4.65	4.43
1368281_at	Dpep1	1	0	0	1	1	1	9.69	9.55	9.44	10.30	9.06	0	0	0	0	0	0	0	6.04	5.96	5.78	5.77	5.69	5.88	5.52
1368282_at	Dpep1	1	0	0	0	1	0	5.69	5.61	5.40	6.00	5.28	0	0	0	0	0	0	0	4.39	4.24	4.42	4.14	4.25	4.21	3.78
1368284_at	Plvap	1	0	0	1	0	0	9.05	8.90	8.76	8.57	8.62	0	0	0	0	0	0	0	7.94	8.10	8.06	7.96	8.18	8.13	8.03
1368291_at	Birc2	1	0	0	0	1	0	7.68	7.64	8.07	7.35	7.65	0	0	0	0	0	0	0	8.05	7.97	7.89	8.07	8.00	8.03	7.80
1368294_at	Dnase1l3	1	0	0	0	0	1	4.39	4.70	3.96	4.38	5.56	0	0	0	0	0	0	0	4.73	4.76	4.83	4.67	4.80	4.97	4.52
1368300_at	Adora2a	1	0	0	0	0	1	5.56	5.32	5.21	5.10	4.92	0	0	0	0	0	0	0	6.97	7.10	7.12	7.01	7.05	7.20	7.21
1368303_at	Per2	1	0	1	1	0	0	7.14	7.08	7.83	7.75	7.49	0	0	0	0	0	0	0	8.78	8.75	8.88	8.78	8.79	8.47	7.85
1368305_at	Casp6	1	0	0	0	0	1	7.88	7.57	7.50	7.93	7.13	0	0	0	0	0	0	0	6.94	6.89	6.81	6.73	6.78	6.93	7.04
1368307_at	Ggt7	1	0	0	0	0	1	6.03	6.20	6.15	6.24	6.66	0	0	0	0	0	0	0	10.66	10.70	10.73	10.63	10.68	10.45	9.77
1368308_at	Myc	1	0	1	0	1	0	8.95	8.63	7.93	8.73	8.64	0	0	0	0	0	0	0	8.33	8.31	8.35	8.35	8.31	7.99	7.55
1368311_at	Mgmt	1	0	0	1	1	0	6.13	5.95	5.77	6.82	5.76	0	0	0	0	0	0	0	9.53	9.59	9.60	9.63	9.64	10.11	10.21
1368316_at	Aqp8	1	0	1	1	1	0	9.88	9.63	9.28	10.57	9.60	0	0	0	0	0	0	0	5.05	5.06	5.06	5.07	5.10	5.32	5.14
1368325_at	Egf	1	0	0	0	0	1	7.46	7.13	7.22	7.49	7.94	0	0	0	0	0	0	0	6.55	6.65	6.72	6.84	6.93	6.71	5.91
1368332_at	Gbp2	1	1	0	0	0	0	4.45	5.20	4.27	4.29	4.47	0	0	0	0	0	0	0	3.50	3.51	3.48	3.53	3.49	3.73	3.47
1368336_at	Fdx1	1	0	1	0	0	1	9.89	9.59	9.30	9.72	9.37	0	0	0	0	0	0	0	8.71	8.64	8.64	8.63	8.55	8.31	8.09
1368337_at	Glycam1	1	0	0	0	0	1	4.56	4.50	4.48	4.46	5.36	0	0	0	0	0	0	0	5.37	5.41	5.13	5.13	5.18	5.24	5.37
1368344_at	Gad1	1	0	1	0	1	0	9.14	9.20	10.08	8.72	9.57	0	0	0	0	0	0	0	7.61	7.57	7.62	7.40	7.47	7.24	7.23
1368366_at	Nat8b	1	0	0	0	1	0	6.97	6.94	6.56	7.33	6.74	0	0	0	0	0	0	0	4.15	4.27	4.18	4.08	4.17	4.29	3.52
1368367_at	Cuzd1	1	1	1	0	0	1	11.29	10.67	10.41	10.85	13.02	0	0	0	0	0	0	0	5.16	5.19	5.11	5.04	4.99	5.18	4.75
1368370_at	Adcy4	1	0	1	0	0	0	4.58	4.47	4.00	4.45	4.31	0	0	0	0	0	0	0	4.13	4.23	4.27	4.20	4.14	4.16	3.73
1368373_at	Rgs7	1	0	1	0	1	0	6.64	6.64	7.64	6.40	7.04	0	0	0	0	0	0	0	7.80	7.72	7.86	8.02	7.83	7.40	7.35
1368374_a_at	Ggt1	1	0	1	1	1	1	10.21	10.01	9.62	10.87	9.48	0	0	0	0	0	0	0	5.84	5.93	5.93	5.69	5.90	6.16	6.21
1368375_a_at	Il15	1	1	1	0	0	1	7.50	6.94	6.78	7.28	6.42	0	0	0	0	0	0	0	5.53	5.72	5.75	5.83	5.44	5.75	5.97
1368376_at	Nr0b2	1	0	0	0	1	0	7.11	7.15	6.66	7.48	6.73	0	0	0	0	0	0	0	7.80	7.87	8.03	8.19	8.30	8.04	7.30
1368378_at	Aldh1l1	1	0	1	0	1	1	8.40	8.19	7.43	8.53	7.72	0	0	0	0	0	0	0	4.26	4.37	4.29	4.16	4.26	4.37	4.41
1368389_at	Apba3	1	0	0	1	1	0	6.11	5.97	5.77	6.61	6.06	0	0	0	0	0	0	0	8.02	8.03	8.11	8.08	8.10	8.01	7.58
1368398_at	Cacna1h	1	1	0	0	0	1	4.72	5.26	5.19	5.00	5.79	0	0	0	0	0	0	0	7.72	7.91	8.01	7.76	8.00	7.75	7.48
1368399_a_at	Cpq	1	0	1	0	1	1	6.79	6.80	7.70	6.64	7.29	0	0	0	0	0	0	0	6.30	6.42	6.38	6.51	6.51	6.12	5.55
1368409_at	Gstt2 /// Gstt2b	1	0	0	1	0	0	6.38	6.37	6.45	6.92	6.04	0	0	0	0	0	0	0	6.90	7.04	6.95	6.76	6.87	6.96	6.55
1368410_at	Mpg	1	0	0	0	0	1	6.44	6.22	6.27	6.54	5.97	0	0	0	0	0	0	0	6.12	6.31	6.41	6.21	6.23	6.41	6.49
1368431_at	Hpn	1	0	1	0	1	1	9.04	8.75	8.20	9.46	8.09	0	0	0	0	0	0	0	4.28	4.28	4.25	4.20	4.24	4.27	3.90
1368432_a_at	Ros1	1	0	0	1	0	0	7.68	7.70	7.83	8.27	7.64	0	0	0	0	0	0	0	7.61	7.72	7.68	7.70	7.95	7.34	7.08
1368433_at	Sacm1l	1	0	0	0	0	1	9.06	9.18	9.37	9.07	8.41	0	0	0	0	0	0	0	9.43	9.50	9.48	9.45	9.45	9.46	9.73
1368440_at	Slc3a1	1	0	1	0	1	0	5.01	5.31	5.79	4.66	5.38	0	0	0	0	0	0	0	5.28	5.28	5.45	5.40	5.40	5.29	5.03
1368442_at	F2	1	0	1	0	1	1	7.25	6.83	6.57	7.16	7.75	0	0	0	0	0	0	0	5.33	5.49	5.53	5.60	5.53	5.41	4.95
1368446_at	Spink1 /// Spink	1	0	1	1	1	1	9.22	8.80	8.69	9.98	8.74	0	0	0	0	0	0	0	4.04	4.01	4.04	3.78	3.89	4.00	3.48
1368450_at	Myo5a	1	0	1	0	0	0	6.94	6.86	7.47	6.87	6.99	0	0	0	0	0	0	0	7.81	7.62	7.69	7.68	7.56	7.59	8.19
1368453_at	Fads2	1	0	0	1	0	0	6.79	6.98	6.84	7.35	6.63	0	0	0	0	0	0	0	8.14	8.13	8.18	8.14	8.07	7.70	7.40
1368464_at	Clec10a	1	0	1	0	0	0	5.18	4.84	4.43	4.75	5.03	0	0	0	0	0	0	0	4.38	4.39	4.37	3.99	4.30	4.26	4.05
1368465_at	Asic2	1	0	0	1	1	0	3.87	3.48	3.66	4.42	3.55	0	0	0	0	0	0	0	9.59	9.63	9.74	9.73	9.58	9.29	8.80
1368467_at	Cyp4f12	1	0	0	0	1	1	5.17	4.93	4.93	5.58	4.63	0	0	0	0	0	0	0	4.25	4.29	4.44	4.42	4.32	4.35	5.48
1368473_at	Gja5	1	0	0	0	0	1	5.48	5.52	5.01	5.23	4.98	0	0	0	0	0	0	0	4.84	4.76	4.61	4.63	4.49	4.50	4.16
1368474_at	Vcam1	1	0	0	0	0	1	4.11	4.34	3.92	4.18	4.96	0	0	0	0	0	0	0	3.35	3.29	3.55	3.24	3.32	3.34	3.04
1368503_at	Gch1	1	0	1	1	1	0	6.65	6.97	7.26	5.98	6.68	0	0	0	0	0	0	0	9.91	10.02	10.09	10.10	10.17	10.06	8.84
1368505_at	Rgs4	1	0	0	0	1	0	8.03	8.38	8.57	7.83	8.21	0	0	0	0	0	0	0	11.84	11.95	11.81	11.48	11.75	11.96	11.86
1368506_at	Rgs4	1	0	1	1	1	0	7.41	7.60	8.10	6.50	7.42	0	0	0	0	0	0	0	11.73	11.81	11.69	11.39	11.65	11.83	11.44
1368509_at	Bbs2	1	0	0	0	1	0	6.15	6.02	6.71	5.84	6.06	0	0	0	0	0	0	0	7.47	7.45	7.57	7.45	7.49	7.24	7.23
1368511_at	Bhlhe41	1	0	1	1	1	0	8.32	8.34	9.07	7.85	8.38	0	0	0	0	0	0	0	9.01	8.94	8.88	8.81	8.74	8.87	8.83
1368515_at	Epb41l3	1	0	1	0	1	1	7.18	7.43	7.81	6.99	7.79	0	0	0	0	0	0	0	7.48	7.53	7.55	7.56	7.54	7.47	7.66
1368518_at	Cd53	1	0	0	0	0	1	3.89	3.97	3.89	3.71	5.32	0	0	0	0	0	0	0	3.76	3.61	3.70	3.58	3.53	3.79	3.02
1368526_at	Pex3	1	0	0	0	1	0	6.23	5.92	6.63	5.90	6.40	0	0	0	0	0	0	0	8.45	8.43	8.64	8.67	8.58	8.19	7.76
1368529_at	Cd99l2	1	0	0	1	1	0	5.32	5.12	5.41	4.71	4.91	0	0	0	0	0	0	0	5.70	5.69	5.78	5.57	5.67	5.49	4.51
1368537_at	Dctn4	1	0	1	0	1	0	4.53	4.42	5.19	4.27	4.65	0	0	0	0	0	0	0	8.57	8.57	8.56	8.69	8.72	8.75	7.83
1368541_at	Emb	1	0	1	0	1	1	8.96	9.16	9.75	8.61	10.22	0	0	0	0	0	0	0	12.21	12.26	12.24	12.24	12.31	12.54	12.18
1368550_at	Foxq1	1	0	0	0	0	1	5.41	5.43	5.29	5.83	4.93	0	0	0	0	0	0	0	7.13	7.06	7.05	6.97	6.90	6.59	6.15
1368559_at	LOC100911216 /	1	0	1	0	1	1	9.42	9.62	10.31	9.02	9.90	0	0	0	0	0	0	0	10.48	10.48	10.50	10.68	10.69	10.39	9.33
1368565_at	Slc1a3	1	0	0	1	1	0	6.38	6.29	6.15	7.15	6.35	0	0	0	0	0	0	0	3.39	3.39	3.43	3.36	3.44	3.38	2.92
1368582_at	Slc7a3	1	0	0	1	1	0	4.52	4.56	5.06	6.12	4.54	0	0	0	0	0	0	0	5.72	5.76	5.78	5.55	5.50	5.53	5.23
1368585_at	Cartpt	1	0	0	1	0	1	9.16	9.57	9.21	8.57	10.23	0	0	0	0	0	0	0	12.21	12.16	12.08	12.01	11.99	11.91	11.45
1368596_at	Sik1	1	0	0	0	1	0	5.56	5.50	5.07	5.69	5.70	0	0	0	0	0	0	0	9.85	9.90	9.84	9.74	9.90	9.92	9.41
1368598_at	Sstr3	1	0	0	0	0	1	7.61	7.33	7.61	7.34	7.14	0	0	0	0	0	0	0	6.74	6.76	6.70	6.58	6.62	6.71	6.35
1368627_at	Rgn	1	0	0	1	1	0	3.59	3.33	3.31	4.17	3.50	0	0	0	0	0	0	0	3.26	3.34	3.26	3.17	3.30	3.23	2.78
1368629_at	Reg1a	1	1	1	1	0	0	14.32	13.80	13.05	13.30	14.40	0	0	0	0	0	0	0	6.27	6.48	6.30	5.99	6.16	5.91	4.28
1368642_at	Cdh2	1	0	1	0	1	1	6.56	6.82	7.57	6.59	7.09	0	0	0	0	0	0	0	10.22	10.13	10.18	10.24	10.27	10.25	9.98
1368646_at	Mapk9	1	0	0	1	0	0	5.64	5.65	5.81	5.14	5.40	0	0	0	0	0	0	0	6.24	6.20	6.19	6.20	6.19	6.65	5.81
1368649_at	Dkc1	1	0	0	1	1	0	7.42	7.60	7.12	7.95	7.08	0	0	0	0	0	0	0	9.86	9.85	9.96	9.84	9.66	9.33	9.06
1368655_at	Srgn	1	0	0	1	0	1	7.26	7.22	7.27	6.73	7.82	0	0	0	0	0	0	0	3.98	3.68	3.82	3.65	3.72	3.74	3.31
1368656_at	Scg3	1	0	1	0	1	0	10.97	11.38	11.59	10.89	11.39	0	0	0	0	0	0	0	12.28	12.35	12.36	12.38	12.47	12.45	12.12
1368665_at	Ufd1	1	0	0	1	1	0	6.84	6.86	7.14	6.27	6.83	0	0	0	0	0	0	0	9.71	9.74	9.80	9.88	9.86	9.70	9.20
1368670_a_at	Pde4a	1	0	1	0	1	0	5.69	5.87	5.07	5.99	5.59	0	0	0	0	0	0	0	7.12	7.06	7.24	7.05	7.07	7.16	6.65
1368677_at	Bdnf	1	0	0	0	1	0	3.79	3.92	3.55	4.20	3.78	0	0	0	0	0	0	0	5.48	5.43	5.37	5.21	5.05	5.66	6.33
1368699_at	Dscam	1	0	0	0	0	1	4.61	4.65	4.68	4.76	5.16	0	0	0	0	0	0	0	4.95	4.91	5.07	4.87	4.94	5.21	5.23
1368705_at	S1pr5	1	0	1	0	1	1	5.22	5.22	4.55	5.15	4.56	0	0	0	0	0	0	0	4.74	4.74	4.74	4.77	4.64	4.84	4.44
1368706_at	Tm4sf4	1	0	1	0	1	1	8.39	8.58	9.38	8.40	9.05	0	0	0	0	0	0	0	11.31	11.30	11.48	11.68	11.52	11.35	11.07
1368707_at	Itih4	1	0	1	0	0	0	4.23	3.86	3.65	3.95	3.99	0	0	0	0	0	0	0	4.16	3.97	4.02	4.02	3.89	4.10	3.77
1368712_at	Znf519	1	0	0	0	1	0	8.17	8.26	8.74	7.98	8.40	0	0	0	0	0	0	0	9.40	9.50	9.47	9.50	9.51	9.06	8.53
1368716_at	Ppp1r14c	1	0	0	0	1	0	4.69	4.68	4.97	4.27	4.61	0	0	0	0	0	0	0	5.62	5.60	5.68	5.53	5.41	5.51	5.30
1368726_a_at	Giot1 /// Zfp347	1	0	1	0	1	0	5.71	5.72	6.45	5.65	6.05	0	0	0	0	0	0	0	8.19	8.32	8.41	8.43	8.52	8.14	7.20
1368745_at	Slc10a2	1	0	0	0	1	0	3.99	4.23	3.75	4.36	4.19	0	0	0	0	0	0	0	3.65	3.63	3.55	3.46	3.66	3.65	3.06
1368747_at	Nup98	1	0	0	0	1	0	4.33	4.44	4.83	3.93	4.61	0	0	0	0	0	0	0	6.74	6.66	6.83	6.95	6.92	6.78	6.43
1368752_at	Tacr3	1	0	0	0	0	1	4.50	4.68	4.68	4.39	5.18	0	0	0	0	0	0	0	4.23	4.25	4.12	4.10	4.02	4.07	3.67
1368753_at	Camkk2	1	0	0	1	1	0	4.86	5.10	4.36	5.55	4.60	0	0	0	0	0	0	0	6.85	6.87	7.08	7.10	7.05	7.13	7.18
1368755_at	Clec4f	1	0	1	0	0	0	4.43	4.29	3.71	4.20	4.31	0	0	0	0	0	0	0	5.37	5.39	5.37	5.26	5.31	5.42	5.04
1368762_at	Ubd	1	1	1	1	0	0	6.24	8.49	5.23	5.70	6.01	0	0	0	0	0	0	0	4.22	4.35	4.14	4.15	4.15	4.25	3.86
1368770_at	Gcnt1	1	0	0	1	1	0	4.56	4.38	4.69	5.47	4.58	0	0	0	0	0	0	0	3.72	3.77	3.91	3.78	3.72	3.87	3.68
1368778_at	Slc6a6	1	0	1	0	1	0	6.69	6.84	7.40	6.46	6.97	0	0	0	0	0	0	0	8.37	8.24	8.36	8.37	8.24	8.22	8.40
1368790_at	Serpina10	1	0	1	0	1	1	7.73	7.59	6.91	7.78	7.18	0	0	0	0	0	0	0	4.74	4.66	4.52	4.48	4.43	4.40	3.84
1368808_at	Cap1	1	0	0	1	1	0	7.93	7.92	8.42	7.38	8.07	0	0	0	0	0	0	0	9.82	9.86	9.90	9.89	9.87	10.12	9.55
1368814_at	Aldh6a1	1	1	0	1	1	1	7.30	6.84	6.92	5.88	6.54	0	0	0	0	0	0	0	7.13	7.11	7.19	7.16	7.24	7.38	6.60
1368818_at	Psme4	1	0	0	0	0	1	6.08	6.13	6.28	6.27	6.56	0	0	0	0	0	0	0	8.89	8.91	9.10	9.10	9.10	9.23	8.92
1368821_at	Fstl1	1	0	0	1	0	0	5.27	4.91	5.24	4.61	4.81	0	0	0	0	0	0	0	4.67	4.85	4.70	4.62	4.53	4.67	4.40
1368822_at	Fstl1	1	0	0	1	0	1	7.18	6.85	7.13	6.53	6.65	0	0	0	0	0	0	0	4.78	4.65	4.75	4.62	4.68	4.73	4.14
1368829_at	Fbn1	1	1	0	1	1	1	6.43	5.76	6.28	5.49	5.72	0	0	0	0	0	0	0	3.78	3.76	3.81	3.83	3.77	3.83	3.89
1368835_at	Stat1	1	1	0	0	0	0	7.40	8.13	7.95	7.41	7.66	0	0	0	0	0	0	0	8.97	8.89	9.02	9.09	9.06	9.15	8.95
1368847_at	Rab10	1	0	0	0	1	0	8.09	8.11	8.46	7.73	8.14	0	0	0	0	0	0	0	9.53	9.49	9.53	9.66	9.60	9.71	9.07
1368848_at	Lman1	1	0	0	1	0	0	8.05	7.94	8.06	7.49	7.91	0	0	0	0	0	0	0	10.93	10.94	10.93	10.89	11.02	10.79	9.61
1368849_at	Csnk1g3	1	0	0	0	1	0	6.01	5.98	6.43	5.61	5.87	0	0	0	0	0	0	0	8.28	8.37	8.39	8.40	8.33	8.42	7.56
1368852_at	Dnaja1	1	0	1	0	1	0	8.22	8.41	8.99	7.78	8.67	0	0	0	0	0	0	0	10.52	10.54	10.61	10.71	10.68	10.36	9.99
1368858_at	Ugt8	1	0	1	1	1	1	5.37	5.49	6.53	4.44	5.91	0	0	0	0	0	0	0	9.53	9.58	9.67	9.89	9.75	9.54	8.93
1368871_at	Map3k1	1	0	0	1	0	0	6.61	6.53	6.67	7.22	6.69	0	0	0	0	0	0	0	8.68	8.53	8.55	8.22	8.32	8.17	8.21
1368872_a_at	Homer2	1	0	0	1	1	0	7.09	6.79	6.59	7.58	6.95	0	0	0	0	0	0	0	7.43	7.36	7.52	7.56	7.49	7.61	7.67
1368878_at	Idi1	1	0	0	0	0	1	7.00	7.00	7.12	6.96	7.52	0	0	0	0	0	0	0	8.57	8.55	8.60	8.78	8.65	8.51	8.34
1368879_a_at	Gnao1	1	0	0	0	0	1	5.94	6.26	6.36	5.91	6.42	0	0	0	0	0	0	0	8.56	8.53	8.68	8.65	8.72	8.54	8.19
1368887_at	RGD1566401	1	0	1	0	1	0	10.62	10.94	11.30	10.48	10.87	0	0	0	0	0	0	0	3.79	3.89	3.94	3.87	3.84	4.12	3.97
1368888_a_at	Rtn4	1	0	1	0	1	0	8.37	8.44	9.12	8.05	8.82	0	0	0	0	0	0	0	11.19	11.23	11.19	11.23	11.28	11.39	11.24
1368908_at	Anxa4	1	0	0	1	1	0	7.69	7.66	8.23	6.95	7.84	0	0	0	0	0	0	0	9.29	9.26	9.22	9.27	9.24	9.46	8.99
1368910_at	Pdp1	1	0	1	0	1	0	4.86	4.71	5.52	4.72	4.82	0	0	0	0	0	0	0	9.07	9.07	8.94	8.87	8.97	9.05	8.73
1368912_at	Trh	1	0	1	0	1	0	9.18	9.35	10.02	8.81	9.27	0	0	0	0	0	0	0	3.84	3.95	3.76	3.70	3.73	3.81	3.30

Probeset ID	Gene Symbol	Islet Data Set (log2 ratio ≥0.263; FDR <20%) union n=5325 1=YES 0=NO	Islet DRlyp/lyp ND vs ND+Lp299v (log2 ratio ≥0.263; FDR<20%) n=429 1=YES 0=NO	Islet DRlyp/lyp ND vs HCD (log2 ratio ≥0.263; FDR<20%) n=2247 1=YES 0=NO	Islet DRlyp/lyp ND vs HCD+Lp299v (log2 ratio ≥0.263; FDR<20%) n=1595 1=YES 0=NO	Islet DRlyp/lyp HCD vs HCD+Lp299v (log2 ratio ≥0.263; FDR<20%) n=3026 1=YES 0=NO	Islet DRlyp/lyp ND vs Flyp/lyp ND (log2 ratio ≥ 0.263; FDR<20%) n=1950 1=YES 0=NO	Islet Mean Log2 Intensity DRlyp/lyp ND	Islet Mean Log2 Intensity DRlyp/lyp ND+Lp299v	Islet Mean Log2 Intensity DRlyp/lyp HCD	Islet Mean Log2 Intensity DRlyp/lyp HCD+Lp299v	Islet Mean Log2 Intensity Flyp40 ND	Rinm5f data union of all conditions n=3705 1=YES 0=NO	Rinm5f 30 µM propionate/ 6 µM butyrate vs Media (FDR<20%; 1.5 fold) n=3 1=YES 0=NO	Rinm5f 60 µM propionate/ 12 µM butyrate vs Media (FDR<20%; 1.5 fold) n=18 1=YES 0=NO	Rinm5f 120 µM propionate/ 24 µM butyrate vs Media (FDR<20%; 1.5 fold) n=164 1=YES 0=NO	Rinm5f 240 µM propionate /48 µM butyrate vs Media (FDR<20%; 1.5 fold) n=268 1=YES 0=NO	Rinm5f 1500 µM propionate/ 300 µM butyrate vs Media (FDR<20%; 1.5 fold) n=2491 1=YES 0=NO	Rinm5f 500 µM propionate/ 500 µM butyrate vs Media (FDR<20%; 1.5 fold) n=3001 1=YES 0=NO	Rinm5f Mean Log2 Intensity Media only	Rinm5f Mean Log2 Intensity 30 µM propionate/ 6 µM butyrate	Rinm5f Mean Log2 Intensity 60 µM propionate/ 12 µM butyrate	Rinm5f Mean Log2 Intensity 120 µM propionate/ 24 µM butyrate	Rinm5f Mean Log2 Intensity 240 µM propionate/ 48 µM butyrate	Rinm5f Mean Log2 Intensity 1500 µM propionate /300 µM butyrate	Rinm5f Mean Log2 Intensity 500 µM propionate/ 500 µM butyrate
1368914_at	Runx1	1	0	0	0	1	0	4.22	4.34	3.76	4.48	4.06	0	0	0	0	0	0	0	6.55	6.63	6.64	6.53	6.56	6.52	6.32
1368924_at	Ghr	1	0	1	1	1	0	5.78	5.89	6.72	4.74	5.94	0	0	0	0	0	0	0	8.43	8.50	8.61	8.73	8.82	8.65	7.35
1368927_at	Esyt1	1	0	0	0	1	0	6.93	6.79	6.43	7.00	6.83	0	0	0	0	0	0	0	8.47	8.48	8.49	8.47	8.43	8.52	8.24
1368940_at	P2ry2	1	0	1	0	0	0	4.53	4.41	3.98	4.53	4.14	0	0	0	0	0	0	0	3.53	3.56	3.70	3.54	3.58	3.60	3.41
1368943_at	Rnase4	1	0	1	1	0	0	7.30	7.03	8.16	8.03	7.50	0	0	0	0	0	0	0	4.29	4.12	4.22	4.00	4.08	4.13	3.56
1368948_at	Msn	1	0	0	0	1	0	5.20	5.26	5.61	4.85	5.15	0	0	0	0	0	0	0	6.88	6.88	7.03	7.21	7.07	6.96	5.82
1368964_at	Lrrn3	1	0	1	0	1	0	3.97	3.80	4.67	3.59	4.09	0	0	0	0	0	0	0	7.62	7.57	7.69	7.67	7.57	7.38	7.63
1368969_at	Sost	1	0	1	0	0	0	3.83	3.75	3.27	3.56	3.60	0	0	0	0	0	0	0	4.17	4.23	4.30	4.24	4.16	4.11	3.79
1368977_a_at	Timm10b	1	0	0	1	0	0	6.25	6.54	6.60	6.72	6.04	0	0	0	0	0	0	0	8.99	9.09	8.98	8.96	8.95	8.86	8.40
1368984_at	Septin2	1	0	1	1	1	0	6.08	6.20	6.74	5.06	5.78	0	0	0	0	0	0	0	8.60	8.52	8.54	8.60	8.51	8.41	7.41
1368998_at	Nkx6-1	1	0	1	0	0	0	8.76	8.98	9.38	8.69	9.13	0	0	0	0	0	0	0	8.88	8.88	8.85	8.81	8.77	9.08	8.37
1369002_at	Soat1	1	0	1	1	1	0	6.65	6.71	7.23	5.90	6.45	0	0	0	0	0	0	0	7.32	7.21	7.41	7.48	7.27	7.67	6.81
1369003_at	Dedd	1	1	0	1	0	0	4.94	4.46	4.73	4.43	4.67	0	0	0	0	0	0	0	6.34	6.37	6.43	6.59	6.57	6.27	5.87
1369004_at	Rab26	1	0	1	0	1	1	8.65	8.27	7.94	9.04	7.84	0	0	0	0	0	0	0	9.25	9.41	9.32	9.19	9.31	9.37	8.76
1369026_at	Arfip1	1	0	0	1	1	0	6.16	6.25	6.25	5.54	6.07	0	0	0	0	0	0	0	8.29	8.28	8.28	8.39	8.28	8.35	8.01
1369029_at	Plscr1	1	0	0	1	0	0	7.47	7.32	7.61	8.08	7.28	0	0	0	0	0	0	0	3.62	3.59	3.64	3.63	3.64	3.55	3.13
1369032_at	Blcap	1	0	0	0	1	0	7.49	7.62	7.80	7.06	7.44	0	0	0	0	0	0	0	7.73	7.73	7.84	7.76	7.70	7.70	7.02
1369043_at	Kcna4	1	0	1	0	1	0	4.44	4.54	5.20	4.46	4.74	0	0	0	0	0	0	0	5.39	5.38	5.51	5.46	5.45	5.51	6.04
1369058_at	Syt3	1	0	0	0	1	0	4.80	4.66	4.31	4.90	4.55	0	0	0	0	0	0	0	5.62	5.74	5.74	5.81	5.81	5.65	4.56
1369059_at	Trpm7	1	0	0	1	1	0	6.37	5.99	6.55	5.70	6.17	0	0	0	0	0	0	0	7.58	7.64	7.66	7.69	7.71	7.64	7.33
1369061_at	Gsr	1	0	0	0	1	0	6.25	6.06	6.62	5.82	6.10	0	0	0	0	0	0	0	9.65	9.61	9.68	9.77	9.70	9.65	8.89
1369063_at	Anp32a	1	1	0	0	0	1	8.30	7.75	7.88	8.22	7.56	0	0	0	0	0	0	0	9.19	9.39	9.36	9.06	9.22	9.15	8.65
1369065_a_at	Atp2a2	1	0	0	0	0	1	9.04	9.08	9.39	8.94	9.53	0	0	0	0	0	0	0	10.17	10.11	10.25	10.27	10.32	10.03	10.36
1369069_at	Akap1	1	0	0	0	0	1	6.27	5.86	6.43	5.83	5.63	0	0	0	0	0	0	0	7.58	7.59	7.75	7.89	7.81	8.01	7.30
1369074_at	Slc38a4	1	0	1	0	1	0	9.00	9.00	9.66	8.62	9.23	0	0	0	0	0	0	0	11.72	11.68	11.68	11.61	11.53	11.55	10.68
1369076_at	Tmem33	1	1	0	1	0	1	7.33	7.85	7.47	7.90	7.96	0	0	0	0	0	0	0	8.67	8.33	8.74	8.53	8.53	8.38	7.79
1369078_at	Mapk1	1	0	0	1	1	0	6.87	6.85	7.23	6.26	6.81	0	0	0	0	0	0	0	9.12	9.21	9.23	9.26	9.20	9.34	8.49
1369083_at	Cirbp	1	0	1	1	1	0	6.71	6.50	7.57	5.89	6.27	0	0	0	0	0	0	0	8.86	9.01	9.01	8.93	8.95	8.73	7.96
1369085_s_at	Snurf	1	0	1	1	1	0	7.47	7.31	8.31	6.34	7.50	0	0	0	0	0	0	0	10.18	10.26	10.39	10.47	10.59	10.66	9.70
1369086_a_at	Cacna1d	1	0	1	0	1	0	6.43	6.57	7.35	6.14	6.89	0	0	0	0	0	0	0	8.27	8.28	8.45	8.54	8.59	8.27	7.67
1369092_at	Sec22a	1	0	0	0	1	0	5.74	5.64	6.17	5.33	5.85	0	0	0	0	0	0	0	7.86	8.02	7.95	7.93	7.97	7.91	7.28
1369099_at	Slc30a1	1	0	0	1	1	0	4.46	4.46	4.86	3.67	4.14	0	0	0	0	0	0	0	5.96	5.67	6.04	6.26	6.26	6.52	5.71
1369105_a_at	Pkib	1	0	0	0	0	1	6.35	6.08	6.38	6.18	4.86	0	0	0	0	0	0	0	10.34	10.47	10.39	10.31	10.41	10.32	9.38
1369113_at	Grem1	1	0	1	0	0	0	3.40	3.62	4.17	3.53	3.32	0	0	0	0	0	0	0	3.28	3.26	3.33	3.32	3.21	3.27	2.97
1369117_at	Calca	1	0	0	1	0	0	5.15	5.01	5.50	5.68	5.04	0	0	0	0	0	0	0	4.53	4.59	4.58	4.40	4.48	4.86	5.12
1369127_a_at	Ptgfr	1	0	1	0	0	0	5.58	5.48	5.10	5.49	5.48	0	0	0	0	0	0	0	4.60	4.61	4.45	4.33	4.42	4.45	4.20
1369150_at	Pdk4	1	0	1	1	1	1	6.52	6.11	7.14	5.50	5.89	0	0	0	0	0	0	0	5.60	5.55	5.44	5.25	5.46	5.76	4.94
1369152_at	Ppp3r1	1	0	1	1	1	0	5.95	5.97	6.55	3.79	5.54	0	0	0	0	0	0	0	9.40	9.39	9.44	9.57	9.53	9.69	8.65
1369154_at	Nphs1	1	0	1	0	0	1	4.22	3.87	3.50	4.01	3.72	0	0	0	0	0	0	0	3.87	3.90	3.92	3.74	3.91	3.95	3.50
1369158_at	Casr	1	0	0	1	0	0	6.42	6.69	6.83	7.10	6.46	0	0	0	0	0	0	0	6.48	6.55	6.69	6.75	6.72	6.19	6.22
1369178_a_at	P2rx1	1	0	1	1	0	0	9.62	9.24	8.88	9.05	9.38	0	0	0	0	0	0	0	4.25	4.37	4.49	4.19	4.27	4.41	4.07
1369196_at	Ppy	1	0	0	0	0	1	13.13	13.44	13.43	12.83	12.38	0	0	0	0	0	0	0	4.98	5.09	4.93	4.79	4.75	4.88	4.86
1369200_at	Nt5e	1	0	1	1	0	0	6.68	6.53	7.34	7.16	7.06	0	0	0	0	0	0	0	7.06	7.07	7.22	7.13	7.24	7.22	6.97
1369207_at	Il7	1	0	1	1	0	1	5.63	5.53	4.93	4.98	5.02	0	0	0	0	0	0	0	4.53	4.59	4.67	4.72	4.65	4.44	4.05
1369208_at	Il7	1	0	1	1	0	1	6.12	6.09	5.11	5.34	5.56	0	0	0	0	0	0	0	5.29	5.24	5.24	5.16	4.99	4.94	4.40
1369220_at	Dnm1l	1	0	1	1	1	0	6.16	6.47	6.97	5.32	6.36	0	0	0	0	0	0	0	9.65	9.62	9.68	9.83	9.74	9.46	8.69
1369227_at	Chm	1	0	1	0	1	0	4.37	4.41	5.22	4.04	4.32	0	0	0	0	0	0	0	7.02	7.07	7.14	7.27	7.20	7.12	6.09
1369242_at	Pax6	1	0	1	0	1	1	9.54	9.83	10.41	9.08	10.28	0	0	0	0	0	0	0	12.21	12.27	12.22	12.10	12.27	12.32	11.93
1369248_a_at	Xiap	1	0	0	0	1	0	4.60	4.78	5.08	4.18	4.47	0	0	0	0	0	0	0	6.25	5.99	6.38	6.41	6.20	6.01	5.04
1369249_at	Ankh	1	0	1	1	1	1	7.49	7.54	8.12	7.01	8.01	0	0	0	0	0	0	0	8.53	8.52	8.58	8.70	8.60	8.76	8.65
1369250_at	Slc28a1	1	0	1	0	0	0	4.58	4.32	4.03	4.43	4.36	0	0	0	0	0	0	0	4.09	4.50	4.25	4.10	4.14	4.04	4.03
1369256_at	Bace1	1	0	1	0	1	0	4.97	5.01	5.64	4.81	5.17	0	0	0	0	0	0	0	6.70	6.70	6.78	6.85	6.81	6.79	5.89
1369265_at	Senp2	1	0	0	1	1	1	6.15	6.03	6.44	5.19	5.53	0	0	0	0	0	0	0	9.41	9.47	9.49	9.47	9.47	9.52	8.77
1369268_at	Atf3	1	1	1	0	1	0	7.21	8.16	5.44	6.88	7.46	0	0	0	0	0	0	0	5.34	5.42	5.46	5.51	5.38	5.62	6.58
1369269_at	Galnt1	1	0	1	1	1	0	7.02	7.33	7.61	6.17	6.86	0	0	0	0	0	0	0	9.87	9.85	9.93	10.01	9.93	9.94	8.80
1369278_at	Gna12	1	0	0	1	0	0	5.42	5.04	5.29	4.70	5.11	0	0	0	0	0	0	0	7.71	7.72	7.62	7.46	7.50	7.18	6.71
1369285_at	Pggt1b	1	0	1	1	1	0	5.90	6.01	6.91	5.11	5.98	0	0	0	0	0	0	0	7.72	7.59	7.79	8.07	7.91	7.44	6.62
1369286_at	Proc	1	0	0	1	1	0	5.60	5.43	5.72	6.80	5.50	0	0	0	0	0	0	0	3.92	4.02	4.01	3.89	3.96	3.97	3.81
1369310_at	Basp1	1	0	0	0	1	0	7.21	7.22	7.60	6.89	7.35	0	0	0	0	0	0	0	10.67	10.67	10.76	10.73	10.65	10.43	9.92
1369312_a_at	Csnk1a1	1	0	0	1	1	0	8.02	8.01	8.30	7.15	8.03	0	0	0	0	0	0	0	10.84	10.84	10.88	10.88	10.89	10.83	9.91
1369319_at	Arl6ip5	1	0	1	1	1	0	8.10	8.03	8.77	7.54	8.23	0	0	0	0	0	0	0	9.46	9.48	9.52	9.42	9.51	9.61	9.23
1369323_at	Leprot	1	0	1	0	1	0	5.31	5.32	5.91	5.04	5.60	0	0	0	0	0	0	0	8.05	8.02	8.06	8.02	8.00	8.20	7.56
1369347_s_at	Prom2	1	0	1	0	1	0	6.47	6.35	5.67	6.30	6.11	0	0	0	0	0	0	0	6.92	6.85	7.04	7.01	7.14	7.27	6.95
1369351_at	Cntn3	1	0	0	0	0	1	5.49	5.40	5.99	5.41	6.06	0	0	0	0	0	0	0	4.75	4.61	4.71	4.71	4.63	4.83	4.02
1369379_at	Epha3	1	0	1	0	0	1	4.88	4.52	4.07	4.60	4.03	0	0	0	0	0	0	0	4.08	4.07	4.08	3.99	3.96	4.03	3.72
1369400_a_at	Pfkfb2	1	0	1	0	1	0	5.62	5.88	6.53	5.16	5.79	0	0	0	0	0	0	0	6.26	6.18	6.24	6.19	6.18	6.43	5.59
1369402_at	Adnp	1	0	1	0	1	0	4.56	4.73	5.21	4.48	4.67	0	0	0	0	0	0	0	7.84	7.72	7.90	8.00	7.98	7.70	7.53
1369407_at	Tnfrsf11b	1	0	1	1	1	0	7.63	8.06	8.90	6.89	7.37	0	0	0	0	0	0	0	4.28	4.19	4.21	4.14	4.11	4.31	3.62
1369409_at	Nab1	1	0	1	1	1	0	6.19	6.24	6.94	5.60	6.34	0	0	0	0	0	0	0	8.28	8.19	8.29	8.38	8.33	8.66	8.31
1369411_at	Gfi1	1	0	1	0	0	0	5.17	5.08	4.62	5.11	4.90	0	0	0	0	0	0	0	3.78	3.94	3.95	3.78	3.84	3.77	3.99
1369417_a_at	Opcml	1	0	1	1	0	1	5.49	5.36	4.67	4.66	4.55	0	0	0	0	0	0	0	6.09	6.13	6.12	6.08	6.20	6.24	5.55
1369421_at	Top1	1	0	1	1	1	0	5.45	5.44	6.05	4.83	5.52	0	0	0	0	0	0	0	8.50	8.44	8.59	8.62	8.54	8.08	7.69
1369425_at	Cdh13	1	0	1	0	1	1	4.88	4.87	4.13	4.70	4.17	0	0	0	0	0	0	0	3.92	3.91	4.03	3.92	3.82	3.90	3.69
1369439_at	Agtr1b	1	0	0	1	0	0	3.20	3.41	3.31	3.77	3.13	0	0	0	0	0	0	0	3.73	3.66	3.87	3.62	3.68	3.58	3.22
1369452_a_at	Picalm	1	0	0	1	1	0	7.10	7.24	7.61	5.79	7.07	0	0	0	0	0	0	0	9.46	9.42	9.56	9.66	9.63	9.86	9.08
1369453_at	Epn1	1	0	1	0	0	0	6.52	6.40	5.88	6.41	6.28	0	0	0	0	0	0	0	7.71	7.59	7.80	7.67	7.66	7.79	6.87
1369454_at	Vdr	1	0	0	1	1	0	4.42	4.37	4.60	3.85	4.50	0	0	0	0	0	0	0	4.71	4.72	4.72	4.53	4.57	5.10	4.95
1369462_at	Gad2	1	0	1	1	1	0	6.58	6.76	7.57	6.04	6.77	0	0	0	0	0	0	0	5.26	5.15	5.35	5.41	5.35	5.52	6.70
1369472_a_at	Atf2	1	0	1	1	1	0	5.13	5.35	5.71	4.53	5.17	0	0	0	0	0	0	0	7.57	7.50	7.52	7.64	7.56	7.50	6.80
1369501_at	Znf260	1	0	1	1	1	0	5.17	5.22	5.84	4.26	5.06	0	0	0	0	0	0	0	7.53	7.67	7.64	7.73	7.65	7.51	6.41
1369517_at	Cyth1	1	0	0	0	1	0	4.32	4.08	4.67	3.88	4.19	0	0	0	0	0	0	0	6.73	6.87	7.00	7.09	6.99	7.03	6.07
1369526_at	Acadsb	1	0	0	1	1	1	6.28	6.22	6.30	5.16	5.31	0	0	0	0	0	0	0	4.52	4.51	4.43	4.52	4.38	4.52	3.75
1369541_at	Tmod2	1	0	0	0	1	0	4.92	5.04	5.48	4.59	4.81	0	0	0	0	0	0	0	6.92	6.95	7.02	7.19	7.05	7.08	6.14
1369571_at	Golph3	1	0	0	1	1	0	7.47	7.49	7.76	6.11	7.14	0	0	0	0	0	0	0	9.24	9.24	9.25	9.31	9.29	9.24	8.71
1369584_at	Socs3	1	1	1	0	0	0	6.01	6.81	5.22	5.72	5.96	0	0	0	0	0	0	0	7.50	7.63	7.64	7.20	7.39	7.58	6.29
1369597_at	Vapb	1	0	0	1	1	0	7.14	7.06	7.69	6.26	7.20	0	0	0	0	0	0	0	10.69	10.82	10.83	10.85	10.87	11.10	10.16
1369621_s_at	Fkbp1a	1	0	0	0	1	0	8.18	8.21	8.69	7.84	8.40	0	0	0	0	0	0	0	11.08	11.13	11.12	11.19	11.17	11.09	10.74
1369625_at	Aqp1	1	0	1	1	1	1	6.54	6.13	4.51	5.29	5.07	0	0	0	0	0	0	0	3.94	4.00	3.96	3.85	3.90	3.97	3.55
1369631_at	Myo1c	1	0	0	1	1	0	8.15	8.15	7.78	8.78	7.95	0	0	0	0	0	0	0	8.77	8.75	8.80	8.75	8.76	8.58	8.38
1369632_a_at	Abcc8	1	0	1	0	0	0	6.73	7.02	7.38	7.06	7.16	0	0	0	0	0	0	0	10.34	10.36	10.49	10.53	10.72	10.70	10.22
1369640_at	Gja1	1	0	0	1	0	0	5.57	5.60	5.46	5.02	5.52	0	0	0	0	0	0	0	5.36	5.48	5.47	5.27	5.36	5.43	4.82
1369641_at	Pafah1b2	1	0	0	0	1	0	7.67	7.65	8.21	7.45	7.90	0	0	0	0	0	0	0	9.22	9.24	9.25	9.30	9.27	9.14	8.54
1369642_at	Pafah1b2	1	0	1	1	1	0	7.69	7.73	8.32	7.20	7.78	0	0	0	0	0	0	0	9.35	9.41	9.48	9.57	9.48	9.21	8.58
1369649_at	Cacna2d1	1	0	1	1	1	0	7.99	7.93	8.60	7.23	8.31	0	0	0	0	0	0	0	11.30	11.37	11.48	11.47	11.47	11.42	10.60
1369650_at	Pak2	1	0	0	1	1	0	5.30	5.27	5.72	4.72	5.31	0	0	0	0	0	0	0	8.13	8.14	8.17	8.32	8.15	7.95	7.14
1369659_at	Cga	1	0	1	0	0	0	4.41	4.42	3.84	4.21	4.24	0	0	0	0	0	0	0	4.40	4.55	4.21	4.21	4.27	4.39	4.08
1369689_at	Nsf	1	0	0	1	1	0	6.69	6.60	7.07	6.01	6.61	0	0	0	0	0	0	0	9.25	9.18	9.34	9.35	9.24	9.43	9.18
1369691_at	Scn3a	1	0	1	0	1	1	7.02	7.36	8.02	6.82	8.09	0	0	0	0	0	0	0	9.75	9.67	9.90	10.12	10.15	9.92	9.06
1369696_at	LOC100909655 /	1	0	0	1	1	0	7.63	7.61	8.17	7.04	7.47	0	0	0	0	0	0	0	9.74	9.80	9.92	9.87	9.85	9.68	8.91
1369699_at	Glp1r	1	0	1	0	0	1	7.99	8.24	8.61	7.95	8.49	0	0	0	0	0	0	0	8.63	8.67	8.74	8.69	8.77	8.74	7.81
1369718_at	Ssr3	1	0	0	1	1	0	8.88	8.95	9.20	7.80	8.63	0	0	0	0	0	0	0	10.71	10.70	10.74	10.85	10.83	10.85	9.76
1369720_at	Myo1b	1	0	0	1	1	0	7.30	7.20	7.71	6.78	7.08	0	0	0	0	0	0	0	8.90	8.81	8.88	8.84	8.92	9.02	8.58
1369725_at	Adap2	1	0	0	0	1	0	6.95	6.83	6.53	7.20	6.54	0	0	0	0	0	0	0	7.18	7.17	7.32	7.32	7.27	7.50	7.29
1369728_at	H4-16	1	0	0	1	1	0	6.15	6.52	6.15	7.44	5.70	0	0	0	0	0	0	0	4.16	4.04	4.09	4.15	4.01	4.07	4.05
1369733_at	Ctnnb1	1	0	0	1	0	0	8.20	8.07	8.36	7.68	7.98	0	0	0	0	0	0	0	8.96	8.85	9.00	9.04	9.01	9.11	9.00
1369735_at	Gas6	1	0	1	1	0	1	7.15	7.43	7.89	7.65	8.33	0	0	0	0	0	0	0	8.83	8.80	8.81	8.66	8.70	8.56	8.91
1369736_at	Emp1	1	0	0	1	1	0	5.25	5.15	5.57	4.74	4.89	0	0	0	0	0	0	0	4.22	4.41	4.38	4.17	4.25	4.25	3.86
1369743_a_at	P2rx4	1	0	0	0	1	0	8.90	8.68	8.40	8.97	8.64	0	0	0	0	0	0	0	9.47	9.56	9.48	9.38	9.45	9.39	9.04
1369765_at	Ascl1	1	1	0	0	0	0	4.25	4.83	4.42	4.58	4.17	0	0	0	0	0	0	0	5.66	5.66	5.68	5.93	5.75	5.27	5.18
1369769_at	Kcne1b	1	0	0	0	0	1	9.78	9.41	9.27	9.80	8.98	0	0	0	0	0	0	0	4.38	4.31	4.33	4.28	4.28	4.31	4.13
1369772_at	Slc6a9	1	0	0	0	1	0	6.76	6.61	6.24	6.81	6.47	0	0	0	0	0	0	0	6.65	6.63	6.73	6.71	6.72	6.54	5.69
1369773_at	Bmp3	1	0	0	1	0	1	5.50	5.34	5.83	6.20	4.40	0	0	0	0	0	0	0	6.34	6.15	6.36	6.25	6.16	6.40	6.12
1369785_at	Ppat	1	0	1	0	1	0	6.86	6.51	7.48	6.51	6.68	0	0	0	0	0	0	0	9.19	9.08	9.25	9.50	9.38	9.61	9.02
1369788_s_at	Jun	1	1	0	0	0	0	9.17	9.69	9.07	9.03	9.04	0	0	0	0	0	0	0	8.23	8.07	8.21	8.04	7.81	8.01	7.74
1369793_a_at	Mcam	1	0	0	1	1	0	5.05	4.87	5.39	4.41	4.81	0	0	0	0	0	0	0	4.12	4.09	4.22	3.95	4.14	4.57	4.37
1369796_at	Gjd2	1	0	1	1	1	0	7.34	7.42	8.14	6.51	7.16	0	0	0	0	0	0	0	8.87	8.96	8.93	8.89	8.99	8.93	7.35
1369803_at	Ptf1a	1	0	0	0	0	1	9.94	9.67	9.87	10.24	9.43	0	0	0	0	0	0	0	4.01	4.09	4.12	3.94	4.06	4.30	3.82
1369804_a_at	Csnk1e	1	0	0	1	0	0	4.48	4.41	4.36	3.97	4.26	0	0	0	0	0	0	0	8.18	8.21	8.28	8.43	8.29	8.58	7.78
1369805_at	P3h4	1	0	0	0	1	1	5.09	5.03	5.61	4.74	5.67	0	0	0	0	0	0	0	8.88	8.94	8.95	8.97	8.97	8.91	7.62
1369831_at	Bcan	1	0	1	0	0	0	4.34	4.33	3.81	4.17	4.10	0	0	0	0	0	0	0	5.62	5.69	5.57	5.54	5.42	5.51	4.95
1369897_s_at	Gnas	1	0	0	0	0	1	12.31	12.32	12.76	12.25	13.30	0	0	0	0	0	0	0	14.04	14.06	14.02	14.04	14.10	14.06	13.54
1369903_at	Gabrb3	1	0	1	1	1	1	4.88	5.20	5.97	4.09	5.47	0	0	0	0	0	0	0	9.07	8.84	9.02	9.02	8.87	8.92	7.94
1369909_s_at	Tmem150a	1	0	0	0	1	0	7.70	7.45	7.35	8.09	7.50	0	0	0	0	0	0	0	7.71	7.76	7.87	7.85	7.90	7.80	7.12
1369912_at	Crk	1	0	0	1	1	0	5.67	5.61	5.95	4.95	5.44	0	0	0	0	0	0	0	8.52	8.41	8.57	8.66	8.58	8.72	6.82
1369931_at	Pkm	1	0	1	0	0	1	9.61	9.77	10.30	9.69	10.23	0	0	0	0	0	0	0	12.41	12.40	12.37	12.17	12.19	12.23	11.87
1369934_at	Ppib	1	0	0	0	1	0	10.96	10.77	10.78	11.37	10.77	0	0	0	0	0	0	0	12.32	12.37	12.36	12.37	12.43	12.38	11.85
1369939_at	Cycs	1	0	0	0	0	1	9.74	9.78	9.82	9.77	10.35	0	0	0	0	0	0	0	12.13	12.13	12.13	12.21	12.20	12.39	12.34
1369953_a_at	Cd24a	1	0	0	1	1	0	9.26	9.23	9.49	10.07	9.05	0	0	0	0	0	0	0	13.29	13.37	13.31	13.22	13.35	13.04	12.44
1369957_at	Rgs5	1	0	1	1	1	0	7.55	7.40	8.54	6.72	7.35	0	0	0	0	0	0	0	4.00	3.95	3.79	3.78	3.80	4.07	4.14
1369959_at	Zfp36l1	1	0	0	0	1	0	9.57	9.90	9.10	9.76	9.48	0	0	0	0	0	0	0	4.21	4.22	4.23	3.98	4.04	4.08	3.42
1369960_at	Fxyd1	1	0	0	1	1	1	8.74	8.58	8.27	9.38	7.85	0	0	0	0	0	0	0	5.79	5.80	5.89	5.65	5.73	5.89	5.58
1369961_at	Plpp1	1	0	0	1	0	0	9.15	8.72	9.13	8.57	8.71	0	0	0	0	0	0	0	12.30	12.37	12.34	12.25	12.41	12.49	11.12
1369968_at	Ptn	1	0	1	0	1	0	6.54	6.15	7.14	6.20	6.61	0	0	0	0	0	0	0	8.26	8.33	8.29	8.27	8.17	8.09	7.15
1369971_a_at	Hnrnpd	1	0	0	0	1	0	7.42	7.24	7.82	7.04	7.39	0	0	0	0	0	0	0	10.87	10.94	11.00	11.00	10.95	10.69	10.21
1369973_at	Xdh	1	1	1	0	1	0	6.90	6.44	6.32	7.11	6.44	0	0	0	0	0	0	0	3.82	3.79	3.73	3.62	3.70	3.80	3.03

Probeset ID	Gene Symbol	Islet Data Set (log2 ratio ≥0.263; FDR <20%) union n=5325 1=YES 0=NO	Islet DRlyp/lyp ND vs ND+Lp299v (log2 ratio ≥0.263; FDR<20%) n=429 1=YES 0=NO	Islet DRlyp/lyp ND vs HCD (log2 ratio ≥0.263; FDR<20%) n=2247 1=YES 0=NO	Islet DRlyp/lyp ND vs HCD+Lp299v (log2 ratio ≥0.263; FDR<20%) n=1595 1=YES 0=NO	Islet DRlyp/lyp HCD vs HCD+Lp299v (log2 ratio ≥0.263; FDR<20%) n=3026 1=YES 0=NO	Islet DRlyp/lyp ND vs Flyp/lyp ND (log2 ratio ≥ 0.263; FDR<20%) n=1950 1=YES 0=NO	Islet Mean Log2 Intensity DRlyp/lyp ND	Islet Mean Log2 Intensity DRlyp/lyp ND+Lp299v	Islet Mean Log2 Intensity DRlyp/lyp HCD	Islet Mean Log2 Intensity DRlyp/lyp HCD+Lp299v	Islet Mean Log2 Intensity Flyp40 ND	Rinm5f data union of all conditions n=3705 1=YES 0=NO	Rinm5f 30 µM propionate/ 6 µM butyrate vs Media (FDR<20%; 1.5 fold) n=3 1=YES 0=NO	Rinm5f 60 µM propionate/ 12 µM butyrate vs Media (FDR<20%; 1.5 fold) n=18 1=YES 0=NO	Rinm5f 120 µM propionate/ 24 µM butyrate vs Media (FDR<20%; 1.5 fold) n=164 1=YES 0=NO	Rinm5f 240 µM propionate /48 µM butyrate vs Media (FDR<20%; 1.5 fold) n=268 1=YES 0=NO	Rinm5f 1500 µM propionate/ 300 µM butyrate vs Media (FDR<20%; 1.5 fold) n=2491 1=YES 0=NO	Rinm5f 500 µM propionate/ 500 µM butyrate vs Media (FDR<20%; 1.5 fold) n=3001 1=YES 0=NO	Rinm5f Mean Log2 Intensity Media only	Rinm5f Mean Log2 Intensity 30 µM propionate/ 6 µM butyrate	Rinm5f Mean Log2 Intensity 60 µM propionate/ 12 µM butyrate	Rinm5f Mean Log2 Intensity 120 µM propionate/ 24 µM butyrate	Rinm5f Mean Log2 Intensity 240 µM propionate/ 48 µM butyrate	Rinm5f Mean Log2 Intensity 1500 µM propionate /300 µM butyrate	Rinm5f Mean Log2 Intensity 500 µM propionate/ 500 µM butyrate
1369977_at	Uchl1	1	0	1	0	1	1	7.98	8.12	8.64	7.80	8.49	0	0	0	0	0	0	0	11.20	11.13	11.25	11.28	11.16	10.94	10.46
1369978_at	Prpsap2	1	0	1	0	1	0	7.21	7.20	7.84	7.07	7.53	0	0	0	0	0	0	0	8.43	8.49	8.55	8.54	8.47	8.15	8.15
1370002_at	Arhgef1	1	0	0	1	0	0	7.06	7.14	7.17	7.54	7.28	0	0	0	0	0	0	0	8.30	8.28	8.51	8.42	8.44	8.32	7.86
1370003_at	Eef2	1	0	0	1	0	1	11.97	11.64	12.09	12.45	11.44	0	0	0	0	0	0	0	12.78	12.81	12.85	12.76	12.78	12.85	12.54
1370007_at	Pdia4	1	0	1	0	0	1	8.52	8.71	9.17	8.70	9.14	0	0	0	0	0	0	0	11.54	11.52	11.53	11.51	11.56	11.63	11.20
1370016_at	Nell2	1	0	1	0	1	0	4.81	4.82	5.39	4.68	4.58	0	0	0	0	0	0	0	9.14	9.12	9.13	9.16	9.10	8.77	8.67
1370019_at	Sult1a1	1	1	0	1	1	1	5.23	4.63	5.20	4.22	4.34	0	0	0	0	0	0	0	5.15	4.93	4.91	4.95	4.91	4.90	4.40
1370023_at	Gja4	1	0	0	0	0	1	6.82	6.88	6.54	6.62	6.32	0	0	0	0	0	0	0	6.21	6.41	6.31	6.41	6.37	6.63	6.75
1370036_at	Suox	1	0	0	0	0	1	8.64	8.80	8.83	8.30	9.11	0	0	0	0	0	0	0	9.16	9.12	9.33	9.51	9.49	9.56	9.31
1370041_at	Stmn2	1	0	1	0	0	1	5.01	5.23	5.71	5.03	5.77	0	0	0	0	0	0	0	9.54	9.65	9.69	9.64	9.73	9.72	9.57
1370042_at	Stmn2	1	0	1	0	1	1	4.96	5.09	5.94	4.74	5.96	0	0	0	0	0	0	0	10.69	10.76	10.74	10.63	10.66	10.68	10.59
1370049_at	Smpd2	1	0	0	1	1	0	5.85	5.99	5.46	6.35	5.77	0	0	0	0	0	0	0	6.27	6.27	6.39	6.40	6.46	6.57	6.67
1370055_at	Rab3d	1	0	0	0	1	1	9.69	9.54	9.38	9.96	9.21	0	0	0	0	0	0	0	10.54	10.61	10.64	10.68	10.72	10.44	10.07
1370064_at	Psen2	1	0	1	0	0	0	6.44	6.34	5.91	6.27	6.06	0	0	0	0	0	0	0	5.95	6.01	6.12	6.01	6.02	5.87	5.58
1370067_at	Me1	1	0	1	0	1	1	6.60	6.74	7.24	6.14	7.18	0	0	0	0	0	0	0	9.19	9.19	9.14	9.24	9.14	8.92	8.88
1370073_at	Dnajc3	1	0	0	0	1	0	10.58	10.61	10.27	10.96	10.37	0	0	0	0	0	0	0	9.64	9.64	9.61	9.63	9.68	9.39	9.17
1370081_a_at	Vegfa	1	0	0	0	1	0	8.05	8.32	8.57	7.86	7.97	0	0	0	0	0	0	0	11.04	11.02	10.97	10.74	10.83	10.83	9.47
1370091_at	Gnaq	1	0	1	1	1	0	5.60	6.01	6.50	4.86	5.63	0	0	0	0	0	0	0	8.79	8.63	8.76	8.93	8.73	8.58	7.04
1370096_at	Prf1	1	0	1	0	0	0	5.00	5.12	4.33	4.82	4.82	0	0	0	0	0	0	0	4.53	4.53	4.46	4.33	4.39	4.51	4.01
1370114_a_at	Pik3r1	1	0	0	0	0	1	5.94	5.67	5.91	5.53	5.47	0	0	0	0	0	0	0	6.85	6.79	6.93	6.74	6.76	6.71	6.02
1370123_a_at	Cttn	1	0	0	1	1	0	5.96	5.95	6.17	5.34	6.03	0	0	0	0	0	0	0	9.20	9.24	9.35	9.31	9.27	9.56	8.92
1370127_at	Pold1	1	0	0	0	1	0	6.12	6.26	5.69	6.28	5.98	0	0	0	0	0	0	0	7.98	7.91	7.98	7.90	7.90	7.72	7.54
1370130_at	Rhoa	1	0	1	1	1	0	8.32	8.21	8.96	7.84	8.49	0	0	0	0	0	0	0	10.27	10.24	10.15	10.10	10.14	10.11	9.53
1370131_at	Cav1	1	1	0	1	1	1	6.15	5.63	5.83	4.30	5.08	0	0	0	0	0	0	0	3.52	3.54	3.57	3.59	3.45	3.66	3.38
1370134_at	Slc33a1	1	0	0	1	1	0	8.46	8.44	8.77	7.94	8.49	0	0	0	0	0	0	0	10.22	10.23	10.28	10.24	10.27	10.28	9.60
1370135_at	Cav2	1	0	0	1	1	1	4.96	4.94	5.06	4.09	4.40	0	0	0	0	0	0	0	4.55	4.55	4.69	4.74	4.85	5.00	4.44
1370136_at	---	1	0	0	1	1	0	5.32	5.23	5.54	4.84	4.92	0	0	0	0	0	0	0	6.72	6.67	6.84	6.82	6.73	6.72	6.41
1370141_at	Mcl1	1	0	0	1	1	0	6.99	6.86	7.36	6.00	6.75	0	0	0	0	0	0	0	8.80	8.87	8.90	8.89	8.87	8.72	7.75
1370154_at	Lyz	1	0	0	1	0	1	8.58	8.44	8.19	8.00	9.44	0	0	0	0	0	0	0	5.92	6.01	5.85	5.60	5.60	5.81	5.90
1370155_at	Col1a2	1	1	0	1	1	1	7.32	6.78	7.39	6.62	6.85	0	0	0	0	0	0	0	5.46	5.52	5.73	5.79	5.92	5.55	5.68
1370156_at	Prnp	1	0	1	0	1	0	10.99	11.09	11.83	10.67	11.46	0	0	0	0	0	0	0	12.77	12.76	12.82	12.83	12.92	13.29	13.18
1370158_at	Myh10	1	0	1	0	1	0	9.61	9.77	10.44	9.33	9.51	0	0	0	0	0	0	0	10.14	10.09	10.18	10.27	10.25	10.11	9.74
1370161_at	Ccdc80	1	0	0	1	0	1	7.29	6.89	7.21	6.58	6.71	0	0	0	0	0	0	0	4.51	4.49	4.50	4.31	4.32	4.45	4.32
1370166_at	Sdc2	1	0	1	0	1	0	7.03	7.26	7.94	6.56	7.33	0	0	0	0	0	0	0	7.65	7.61	7.73	7.86	7.87	8.02	7.27
1370167_at	Sdc2	1	0	0	0	1	0	7.10	7.19	7.65	6.82	6.96	0	0	0	0	0	0	0	8.12	8.14	8.21	8.21	8.19	8.26	7.96
1370168_at	Ywhaq	1	0	0	1	1	0	9.80	9.77	10.34	9.17	9.96	0	0	0	0	0	0	0	12.17	12.20	12.23	12.29	12.27	12.35	11.95
1370174_at	Ppp1r15a	1	0	1	0	0	0	7.93	8.12	7.40	7.48	7.52	0	0	0	0	0	0	0	8.09	8.13	8.19	8.24	8.19	8.13	7.50
1370178_at	Cacnb2	1	0	1	0	1	0	7.00	7.16	7.74	6.80	7.38	0	0	0	0	0	0	0	7.30	7.28	7.51	7.51	7.38	7.24	6.94
1370180_at	Nudt4	1	0	0	0	0	1	11.53	11.12	11.27	11.51	10.87	0	0	0	0	0	0	0	10.80	10.78	10.83	10.83	10.90	11.03	11.29
1370182_at	Ptprn2	1	0	0	0	0	1	8.67	8.74	8.91	8.53	10.65	0	0	0	0	0	0	0	11.60	11.62	11.67	11.65	11.72	11.63	11.18
1370188_at	Tra2b	1	0	1	0	1	0	8.82	8.73	9.58	8.47	8.83	0	0	0	0	0	0	0	11.18	11.21	11.21	11.22	11.21	10.95	10.33
1370193_at	Ptp4a1	1	0	0	0	1	0	9.52	9.45	9.85	9.07	9.65	0	0	0	0	0	0	0	11.97	12.01	11.93	11.93	11.90	11.87	11.39
1370209_at	Klf9	1	0	0	0	0	1	10.07	9.83	9.87	10.42	9.47	0	0	0	0	0	0	0	9.56	9.59	9.69	9.65	9.65	10.08	10.40
1370219_at	Cyba	1	0	1	0	0	0	6.57	6.33	6.00	6.22	6.42	0	0	0	0	0	0	0	5.20	5.27	5.13	4.93	4.87	5.12	4.83
1370224_at	Stat3	1	0	0	0	0	1	7.64	7.72	7.82	7.87	8.34	0	0	0	0	0	0	0	10.46	10.29	10.46	10.35	10.25	10.64	10.90
1370225_at	Cited4	1	0	1	0	0	0	7.13	6.82	6.54	6.77	6.68	0	0	0	0	0	0	0	4.70	4.59	4.66	4.60	4.49	4.71	4.43
1370234_at	Fn1	1	0	1	1	0	1	7.28	7.01	6.54	6.71	6.77	0	0	0	0	0	0	0	8.98	9.07	9.08	8.96	9.37	9.38	9.76
1370237_at	Hadh	1	0	1	0	1	0	11.05	11.11	11.86	10.81	11.45	0	0	0	0	0	0	0	12.80	12.83	12.78	12.65	12.74	12.41	11.85
1370246_at	Calm2	1	0	1	0	1	0	10.72	10.72	11.39	10.52	11.01	0	0	0	0	0	0	0	12.73	12.77	12.78	12.79	12.75	12.62	12.45
1370247_a_at	Pmp22	1	0	0	0	0	1	6.16	5.95	6.13	5.71	5.56	0	0	0	0	0	0	0	9.30	9.27	9.28	9.31	9.29	9.02	8.71
1370253_at	Rpl22	1	0	0	1	0	0	11.40	11.34	11.67	11.95	11.12	0	0	0	0	0	0	0	12.30	12.25	12.22	12.17	12.13	11.99	11.81
1370255_at	Sftpc	1	0	0	0	1	0	4.15	4.37	3.76	4.34	4.02	0	0	0	0	0	0	0	4.61	4.65	4.56	4.56	4.55	4.56	4.07
1370264_at	Syne1	1	0	0	0	1	0	7.63	7.60	8.14	7.35	7.93	0	0	0	0	0	0	0	7.13	7.07	7.19	7.20	7.04	7.06	7.30
1370265_at	Arrb2	1	0	0	0	1	0	4.76	4.60	4.40	5.03	4.54	0	0	0	0	0	0	0	6.09	6.04	6.28	6.15	6.10	6.30	6.42
1370276_at	Atp5o	1	0	0	0	1	0	9.90	9.87	9.63	10.32	9.64	0	0	0	0	0	0	0	11.45	11.42	11.42	11.41	11.33	10.99	10.76
1370281_at	Fabp5	1	0	1	0	0	0	7.05	6.68	6.38	6.73	6.96	0	0	0	0	0	0	0	12.25	12.17	12.14	12.09	11.97	11.98	12.72
1370283_at	Hspa5	1	0	0	0	0	1	11.40	11.60	11.52	11.30	11.95	0	0	0	0	0	0	0	12.87	12.91	12.91	12.90	12.95	12.83	12.17
1370284_at	Atp5e	1	0	0	0	1	0	10.95	11.30	10.66	11.32	10.67	0	0	0	0	0	0	0	12.10	12.07	12.03	11.98	11.94	11.83	11.87
1370290_at	Tubb	1	0	0	1	0	0	8.59	8.54	8.66	9.08	8.81	0	0	0	0	0	0	0	11.64	11.54	11.65	11.64	11.57	11.33	10.93
1370291_at	Pdlim3	1	0	1	0	0	0	6.46	6.34	5.68	6.12	6.65	0	0	0	0	0	0	0	7.65	7.62	7.86	7.87	7.66	7.73	7.45
1370295_at	Nme1	1	0	0	0	1	0	8.76	8.81	8.36	9.12	8.57	0	0	0	0	0	0	0	10.97	10.96	11.02	11.08	11.03	10.96	10.90
1370299_at	Aldob	1	1	1	1	0	1	8.09	7.10	7.14	7.40	6.69	0	0	0	0	0	0	0	6.60	6.75	6.98	6.68	6.71	6.68	8.56
1370308_at	Dctpp1	1	0	0	0	1	0	7.39	7.30	7.01	7.72	6.99	0	0	0	0	0	0	0	9.33	9.35	9.31	9.36	9.30	9.67	10.15
1370319_at	Ppif	1	0	0	1	0	0	6.13	5.90	5.69	5.54	6.09	0	0	0	0	0	0	0	9.61	9.57	9.67	9.69	9.69	9.98	9.40
1370331_at	Il11ra	1	0	1	0	1	1	7.31	7.13	6.71	7.44	6.56	0	0	0	0	0	0	0	4.12	3.97	3.90	3.83	3.97	3.99	3.95
1370333_a_at	Igf1	1	1	0	0	1	1	7.55	7.04	7.16	7.78	6.80	0	0	0	0	0	0	0	4.07	4.01	4.03	3.88	3.99	4.02	3.57
1370354_at	Parg	1	0	0	0	0	1	7.43	7.25	7.82	7.67	8.14	0	0	0	0	0	0	0	9.54	9.58	9.61	9.60	9.65	9.63	9.51
1370359_at	Amy1a	1	0	1	1	1	1	10.61	10.22	8.94	9.84	9.71	0	0	0	0	0	0	0	3.37	3.29	3.37	3.39	3.32	3.28	2.76
1370362_at	Ptprn	1	0	0	0	0	1	9.11	9.30	9.28	9.18	9.89	0	0	0	0	0	0	0	12.02	12.03	12.09	11.98	12.02	11.99	11.23
1370376_a_at	Ybx3	1	0	0	0	1	0	9.04	8.96	8.72	9.38	8.79	0	0	0	0	0	0	0	9.74	9.73	9.85	9.84	9.82	9.78	9.80
1370379_at	Prss8	1	0	0	0	1	1	7.63	7.43	7.14	7.94	7.14	0	0	0	0	0	0	0	5.14	5.01	5.14	4.91	4.86	5.30	5.09
1370383_s_at	Hla-drb5	1	0	0	0	0	1	5.42	5.77	5.05	5.35	6.33	0	0	0	0	0	0	0	4.59	4.57	4.70	4.46	4.47	4.63	3.91
1370407_at	Pcyox1	1	0	1	0	1	1	7.54	7.53	8.35	7.54	8.32	0	0	0	0	0	0	0	10.87	10.98	10.86	10.88	11.12	11.39	10.84
1370408_at	Smim3	1	0	0	0	1	1	4.52	4.45	4.20	4.98	4.01	0	0	0	0	0	0	0	6.64	6.79	6.72	6.64	6.70	6.94	6.92
1370410_at	Igsf1	1	0	1	0	1	1	5.94	6.10	6.68	5.87	6.93	0	0	0	0	0	0	0	5.47	5.56	5.48	5.43	5.52	5.61	5.32
1370411_at	Trpc1	1	0	1	0	0	0	6.45	6.48	7.05	6.37	6.64	0	0	0	0	0	0	0	7.62	7.62	7.67	7.56	7.58	7.42	7.45
1370418_s_at	Syt17	1	0	1	0	1	0	7.35	7.33	7.95	7.11	7.65	0	0	0	0	0	0	0	8.60	8.58	8.66	8.71	8.75	8.98	8.77
1370426_a_at	Atp2a2	1	0	0	0	0	1	10.36	10.48	10.81	10.37	10.91	0	0	0	0	0	0	0	11.30	11.17	11.28	11.28	11.19	10.93	11.01
1370427_at	Pdgfa	1	0	0	0	1	0	6.68	6.84	6.19	7.05	6.33	0	0	0	0	0	0	0	7.51	7.59	7.69	7.66	7.78	8.00	7.40
1370435_a_at	Nudt6	1	0	0	0	0	1	5.15	4.96	4.87	5.35	4.60	0	0	0	0	0	0	0	4.92	5.19	4.86	4.81	5.02	4.92	4.84
1370440_at	Slc15a4	1	0	0	0	1	0	6.23	6.08	6.63	5.92	6.25	0	0	0	0	0	0	0	9.05	9.02	9.09	9.08	9.09	9.23	8.88
1370444_at	Cacna1b	1	0	1	0	0	1	4.61	4.99	5.43	4.83	5.27	0	0	0	0	0	0	0	6.98	7.10	7.14	7.26	7.38	7.42	7.26
1370449_at	P2ry14	1	0	0	1	1	1	7.57	7.25	7.16	8.23	7.00	0	0	0	0	0	0	0	4.39	4.37	4.39	4.15	4.21	4.25	3.97
1370452_at	Cacna1c	1	0	1	0	0	1	5.11	5.44	5.67	5.20	5.60	0	0	0	0	0	0	0	6.62	6.65	6.67	6.58	6.58	6.39	6.37
1370463_x_at	Hla-a	1	0	1	0	0	0	5.86	5.85	5.22	5.60	5.60	0	0	0	0	0	0	0	9.97	10.08	10.06	10.07	10.21	10.36	9.78
1370474_at	Thrb	1	0	0	1	1	0	4.53	4.81	4.55	5.23	4.65	0	0	0	0	0	0	0	5.08	4.88	4.84	4.72	4.60	4.68	4.98
1370501_at	Ube2g1	1	0	0	1	0	0	7.53	7.16	7.58	6.92	7.12	0	0	0	0	0	0	0	9.31	9.30	9.31	9.29	9.27	9.10	8.44
1370503_s_at	Epb41l3	1	0	1	0	1	1	6.16	6.41	7.10	6.05	6.80	0	0	0	0	0	0	0	7.81	7.66	7.81	7.82	7.71	7.61	7.88
1370518_a_at	Stxbp1	1	0	0	1	1	0	4.90	5.04	5.32	4.37	5.14	0	0	0	0	0	0	0	8.07	8.00	8.12	8.18	8.07	7.91	6.98
1370540_at	Nr1d2	1	0	0	1	1	1	4.45	4.44	4.66	3.42	3.63	0	0	0	0	0	0	0	7.22	7.08	7.28	7.37	7.18	6.91	5.72
1370541_at	Nr1d2	1	0	0	1	1	1	7.76	7.53	8.22	6.32	6.68	0	0	0	0	0	0	0	9.56	9.59	9.60	9.67	9.59	9.38	8.97
1370543_at	Ogt	1	0	0	0	1	0	7.41	7.34	7.92	7.01	7.31	0	0	0	0	0	0	0	6.96	7.00	7.14	7.34	7.27	7.35	6.94
1370552_at	Ppm1f	1	0	0	1	0	0	4.63	4.34	4.50	4.13	4.29	0	0	0	0	0	0	0	6.67	6.68	6.79	6.86	6.70	6.72	5.55
1370555_at	Nalcn	1	0	0	0	1	0	6.04	5.93	6.48	5.62	5.91	0	0	0	0	0	0	0	4.40	4.31	4.32	4.21	4.21	4.25	3.80
1370562_at	Calcb	1	0	0	1	0	1	7.76	8.02	7.75	7.12	8.26	0	0	0	0	0	0	0	4.30	4.24	4.26	4.14	4.17	4.54	3.84
1370565_at	---	1	0	0	1	1	1	5.23	5.42	4.90	5.81	4.44	0	0	0	0	0	0	0	6.61	6.48	6.68	6.77	6.76	6.88	6.79
1370571_at	Slco3a1	1	0	1	0	1	0	6.44	6.46	7.20	6.38	6.90	0	0	0	0	0	0	0	9.89	9.86	9.91	9.89	9.95	9.70	8.79
1370573_at	Sardh	1	1	0	0	0	0	6.68	6.14	6.26	6.39	6.29	0	0	0	0	0	0	0	7.45	7.47	7.56	7.55	7.47	6.99	6.14
1370578_at	C16orf70	1	0	0	0	1	0	5.97	5.94	6.46	5.65	6.13	0	0	0	0	0	0	0	7.01	7.07	7.11	7.09	7.01	7.05	6.26
1370585_a_at	Prkcb	1	0	0	0	1	1	6.03	6.06	6.55	5.66	7.02	0	0	0	0	0	0	0	9.47	9.57	9.66	9.74	9.85	9.98	9.81
1370589_at	Znf443	1	0	1	0	1	0	4.65	4.60	5.31	4.58	4.95	0	0	0	0	0	0	0	7.14	7.09	7.21	7.35	7.15	6.86	6.32
1370590_at	Gpsm1	1	0	0	1	1	0	7.99	7.69	8.51	7.33	7.71	0	0	0	0	0	0	0	7.73	7.71	7.83	7.73	7.58	7.64	7.16
1370606_at	P2ry1	1	1	1	1	1	0	6.56	6.08	7.68	5.11	6.57	0	0	0	0	0	0	0	6.96	6.81	6.99	7.12	6.95	6.64	6.09
1370663_at	Wee1	1	0	0	1	0	0	6.96	7.10	7.28	7.49	7.35	0	0	0	0	0	0	0	9.00	8.95	9.02	9.08	9.05	8.88	8.36
1370665_at	Hyou1	1	0	0	1	1	1	6.13	6.24	6.56	5.55	5.12	0	0	0	0	0	0	0	5.92	5.98	6.06	6.02	5.92	5.79	4.88
1370680_at	Stau2	1	0	1	1	1	0	6.23	6.18	6.92	5.49	6.22	0	0	0	0	0	0	0	8.59	8.69	8.68	8.67	8.59	8.26	7.82
1370693_a_at	Cnp	1	0	0	0	0	1	6.17	5.92	5.89	6.10	5.59	0	0	0	0	0	0	0	6.93	7.11	7.17	7.09	7.15	7.16	6.84
1370695_s_at	Trib3	1	0	0	1	1	0	6.38	6.35	6.25	7.19	6.29	0	0	0	0	0	0	0	8.67	8.71	8.68	8.86	8.84	8.39	7.97
1370697_a_at	Nexn	1	0	0	0	1	0	5.55	5.46	5.11	5.85	5.34	0	0	0	0	0	0	0	3.50	3.62	3.57	3.54	3.52	3.54	3.20
1370751_at	LOC257642	1	0	0	1	0	0	12.51	12.34	12.43	11.91	12.11	0	0	0	0	0	0	0	13.09	12.93	13.17	13.04	13.02	13.09	12.24
1370760_a_at	Gad1	1	0	1	1	1	0	5.61	5.89	6.46	5.03	5.61	0	0	0	0	0	0	0	5.40	5.54	5.53	5.12	5.14	5.07	4.13
1370772_a_at	Hfe	1	0	0	1	1	1	7.04	7.00	7.49	6.44	6.56	0	0	0	0	0	0	0	7.94	8.02	8.07	7.99	7.91	7.84	7.06
1370781_a_at	Kcnip1	1	0	0	0	1	0	3.83	3.73	4.16	3.42	3.70	0	0	0	0	0	0	0	5.36	5.48	5.45	5.40	5.47	4.80	3.82
1370785_s_at	Tomm20	1	0	0	0	1	0	9.79	9.54	10.29	9.42	9.76	0	0	0	0	0	0	0	11.16	11.17	11.19	11.26	11.31	11.56	11.13
1370792_at	Mapre1	1	0	1	1	1	0	6.53	6.59	7.44	5.44	6.72	0	0	0	0	0	0	0	10.13	10.16	10.21	10.27	10.20	10.25	9.74
1370803_at	Zwint	1	0	1	1	1	0	9.30	9.26	9.99	8.70	9.49	0	0	0	0	0	0	0	12.87	12.85	12.90	12.92	12.89	12.58	12.08
1370811_at	Mpst	1	0	0	1	1	1	8.51	8.46	8.20	9.14	7.85	0	0	0	0	0	0	0	8.78	8.83	8.86	8.78	8.84	9.26	9.33
1370816_at	Nr1d1	1	0	0	1	1	1	6.51	6.80	6.10	5.22	5.34	0	0	0	0	0	0	0	7.93	7.95	8.03	8.05	8.09	8.45	7.95
1370822_at	Hla-dqa1	1	0	1	0	0	1	7.06	6.91	6.44	6.62	8.05	0	0	0	0	0	0	0	5.36	5.36	5.39	5.02	5.07	5.23	4.95
1370824_at	Slc38a3	1	0	1	1	1	1	7.11	7.05	6.46	7.73	6.63	0	0	0	0	0	0	0	4.65	4.73	4.71	4.52	4.52	4.56	4.54
1370828_at	Zdhhc2	1	0	1	0	1	0	6.82	6.68	7.42	6.71	6.99	0	0	0	0	0	0	0	11.76	11.77	11.61	11.40	11.51	11.37	11.11
1370833_at	Pex5l	1	1	1	1	0	1	6.19	7.05	6.87	6.80	6.98	0	0	0	0	0	0	0	6.52	6.25	6.55	6.73	6.49	6.75	7.16
1370842_at	Bckdk	1	0	1	1	1	0	9.62	9.29	9.05	10.17	9.25	0	0	0	0	0	0	0	10.14	10.26	10.27	10.30	10.31	10.45	10.26
1370844_at	Hnrnpf	1	0	0	1	1	0	8.32	8.07	8.60	7.64	8.16	0	0	0	0	0	0	0	10.13	10.14	10.25	10.21	10.16	10.09	9.64
1370847_at	Spon2	1	0	1	1	0	0	5.69	5.46	5.12	5.21	5.46	0	0	0	0	0	0	0	8.29	8.27	8.32	8.31	8.09	7.97	7.84
1370850_at	Scn3b	1	0	0	0	0	1	7.49	7.60	7.62	7.30	8.06	0	0	0	0	0	0	0	8.65	8.72	8.80	8.73	8.74	8.67	8.36
1370854_at	Nexn	1	0	1	0	1	0	6.79	6.59	6.24	7.00	6.42	0	0	0	0	0	0	0	3.73	3.61	3.71	3.61	3.57	3.63	3.60
1370859_at	Pdia6	1	0	0	0	0	1	11.30	11.38	11.68	11.57	11.78	0	0	0	0	0	0	0	13.09	13.16	13.14	13.14	13.25	13.28	12.72
1370863_at	Krt5	1	0	0	0	1	0	4.51	4.33	3.98	4.58	4.38	0	0	0	0	0	0	0	4.67	4.64	4.47	4.46	4.54	4.60	4.03
1370864_at	Col1a1	1	0	0	1	0	0	7.07	6.66	6.61	6.48	6.69	0	0	0	0	0	0	0	5.12	5.08	5.15	4.85	4.96	5.03	5.03
1370870_at	Me1	1	0	1	0	1	1	6.79	6.65	7.50	6.47	7.49	0	0	0	0	0	0	0	9.77	9.77	9.84	9.89	9.83	9.59	9.72
1370883_at	Hla-dra	1	0	0	1	0	1	7.46	7.63	7.25	6.87	8.83	0	0	0	0	0	0	0	4.37	4.41	4.25	4.32	4.21	4.34	3.76
1370891_at	Cd48	1	0	0	1	1	0	6.44	6.23	6.85	5.49	6.70	0	0	0	0	0	0	0	3.56	3.56	3.55	3.54	3.58	3.54	3.10
1370892_at	C4a /// C4b /// L	1	0	1	1	1	0	5.79	5.42	4.56	5.13	5.79	0	0	0	0	0	0	0	5.33	5.52	5.35	5.40	5.31	5.41	4.70
1370895_at	Col5a2	1	0	0	1	1	0	6.85	6.54	7.14	6.17	6.67	0	0	0	0	0	0	0	4.70	4.64	4.55	4.45	4.48	4.18	3.75

Probeset ID	Gene Symbol	Islet Data Set (log2 ratio ≥0.263; FDR <20%) union n=5325 1=YES 0=NO	Islet DRlyp/lyp ND vs ND+Lp299v (log2 ratio ≥0.263; FDR<20%) n=429 1=YES 0=NO	Islet DRlyp/lyp ND vs HCD (log2 ratio ≥0.263; FDR<20%) n=2247 1=YES 0=NO	Islet DRlyp/lyp ND vs HCD+Lp299v (log2 ratio ≥0.263; FDR<20%) n=1595 1=YES 0=NO	Islet DRlyp/lyp HCD vs HCD+Lp299v (log2 ratio ≥0.263; FDR<20%) n=3026 1=YES 0=NO	Islet DRlyp/lyp ND vs Flyp/lyp ND (log2 ratio ≥ 0.263; FDR<20%) n=1950 1=YES 0=NO	Islet Mean Log2 Intensity DRlyp/lyp ND	Islet Mean Log2 Intensity DRlyp/lyp ND+Lp299v	Islet Mean Log2 Intensity DRlyp/lyp HCD	Islet Mean Log2 Intensity DRlyp/lyp HCD+Lp299v	Islet Mean Log2 Intensity Flyp40 ND	Rinm5f data union of all conditions n=3705 1=YES 0=NO	Rinm5f 30 µM propionate/ 6 µM butyrate vs Media (FDR<20%; 1.5 fold) n=3 1=YES 0=NO	Rinm5f 60 µM propionate/ 12 µM butyrate vs Media (FDR<20%; 1.5 fold) n=18 1=YES 0=NO	Rinm5f 120 µM propionate/ 24 µM butyrate vs Media (FDR<20%; 1.5 fold) n=164 1=YES 0=NO	Rinm5f 240 µM propionate /48 µM butyrate vs Media (FDR<20%; 1.5 fold) n=268 1=YES 0=NO	Rinm5f 1500 µM propionate/ 300 µM butyrate vs Media (FDR<20%; 1.5 fold) n=2491 1=YES 0=NO	Rinm5f 500 µM propionate/ 500 µM butyrate vs Media (FDR<20%; 1.5 fold) n=3001 1=YES 0=NO	Rinm5f Mean Log2 Intensity Media only	Rinm5f Mean Log2 Intensity 30 µM propionate/ 6 µM butyrate	Rinm5f Mean Log2 Intensity 60 µM propionate/ 12 µM butyrate	Rinm5f Mean Log2 Intensity 120 µM propionate/ 24 µM butyrate	Rinm5f Mean Log2 Intensity 240 µM propionate/ 48 µM butyrate	Rinm5f Mean Log2 Intensity 1500 µM propionate /300 µM butyrate	Rinm5f Mean Log2 Intensity 500 µM propionate/ 500 µM butyrate
1370896_a_at	Myh11	1	0	1	0	1	1	6.52	6.78	5.88	6.84	5.92	0	0	0	0	0	0	0	5.46	5.54	5.57	5.33	5.37	5.31	5.07
1370897_at	Bckdha	1	0	1	0	1	1	8.41	8.03	7.73	8.57	7.19	0	0	0	0	0	0	0	7.23	7.37	7.36	7.38	7.22	7.25	7.00
1370905_at	Dock9	1	0	0	0	0	1	9.36	9.20	9.67	8.99	7.64	0	0	0	0	0	0	0	7.95	8.04	8.11	8.25	8.27	8.34	8.17
1370906_at	Bckdhb	1	0	0	0	1	1	10.13	9.75	9.63	10.29	9.36	0	0	0	0	0	0	0	8.16	8.26	8.32	8.51	8.49	8.67	8.88
1370912_at	Hspa1a /// Hspa	1	1	0	1	1	0	8.77	9.99	8.64	7.65	8.51	0	0	0	0	0	0	0	7.07	7.22	7.22	7.23	7.30	7.34	7.50
1370924_at	Trb	1	0	0	0	0	1	3.95	4.00	3.78	3.95	4.52	0	0	0	0	0	0	0	4.49	4.55	4.34	4.37	4.31	4.48	4.06
1370928_at	Litaf	1	0	0	0	1	0	10.07	9.87	9.64	10.30	9.68	0	0	0	0	0	0	0	10.44	10.40	10.36	10.27	10.39	10.67	10.92
1370934_at	Nup153	1	0	1	0	1	0	7.92	7.78	8.65	7.87	8.23	0	0	0	0	0	0	0	10.09	10.08	10.13	10.21	10.20	10.12	9.67
1370940_at	Tjp2	1	0	0	0	0	1	5.60	5.64	5.53	5.99	6.22	0	0	0	0	0	0	0	8.10	7.97	8.12	8.19	8.22	8.33	8.65
1370942_at	Rasa3	1	0	0	1	0	0	4.56	4.19	4.10	4.07	4.37	0	0	0	0	0	0	0	8.51	8.49	8.56	8.50	8.56	8.74	9.01
1370943_at	Sult1c2	1	0	0	0	0	1	4.97	4.78	5.11	5.20	5.44	0	0	0	0	0	0	0	3.67	3.59	3.53	3.45	3.39	3.57	2.88
1370946_at	Nfix	1	0	0	1	1	0	6.74	6.67	6.46	7.28	6.39	0	0	0	0	0	0	0	6.69	6.61	6.76	6.57	6.48	6.50	6.80
1370950_at	Plpp3	1	1	0	1	1	1	8.96	8.37	9.23	8.45	8.38	0	0	0	0	0	0	0	4.12	4.03	4.05	3.91	3.96	4.32	4.44
1370951_at	Plpp3	1	0	0	0	1	0	5.90	5.82	6.33	5.49	5.59	0	0	0	0	0	0	0	5.13	5.05	4.97	4.88	4.99	4.90	4.47
1370955_at	Adam10	1	0	0	1	1	0	5.55	5.60	5.57	6.29	5.34	0	0	0	0	0	0	0	7.15	7.08	7.14	7.20	7.17	7.26	5.92
1370956_at	Dcn	1	1	0	1	1	1	7.52	6.45	7.65	6.25	6.66	0	0	0	0	0	0	0	4.17	4.10	4.07	4.09	4.09	4.04	3.49
1370959_at	Col3a1	1	1	0	1	1	0	8.54	7.83	8.43	7.56	8.27	0	0	0	0	0	0	0	4.37	4.32	4.25	4.20	4.21	4.35	3.69
1370967_at	LOC680329	1	1	0	0	0	1	3.96	4.92	3.65	3.86	6.42	0	0	0	0	0	0	0	3.62	3.65	3.64	3.45	3.52	3.57	3.13
1370985_at	Mapk7	1	0	1	0	0	0	5.62	5.31	5.07	5.30	5.42	0	0	0	0	0	0	0	7.01	6.97	7.00	6.88	6.91	6.88	6.47
1370990_at	Crlf3	1	0	0	0	0	1	5.28	5.20	5.62	5.02	5.82	0	0	0	0	0	0	0	8.50	8.46	8.54	8.49	8.52	8.69	8.92
1370995_at	Pou2f1	1	0	0	0	0	1	5.99	6.06	5.79	6.32	6.47	0	0	0	0	0	0	0	6.17	6.20	6.35	6.28	6.18	6.21	5.61
1371005_at	Abcc1	1	0	0	0	1	0	7.47	7.64	7.18	7.78	7.47	0	0	0	0	0	0	0	8.17	8.05	8.13	8.00	7.94	7.95	8.38
1371012_at	Hacl1	1	0	0	0	0	1	5.16	5.23	5.47	5.19	5.80	0	0	0	0	0	0	0	7.82	7.85	7.89	7.95	7.94	8.21	8.29
1371024_at	Cux1	1	0	1	1	0	1	6.06	6.03	6.68	6.66	6.67	0	0	0	0	0	0	0	8.96	8.98	9.04	9.05	8.99	8.89	8.80
1371027_at	Cblb	1	0	0	1	1	0	7.08	7.39	6.86	7.71	7.21	0	0	0	0	0	0	0	6.87	6.78	6.95	6.99	6.85	7.02	7.14
1371028_at	Tgoln1	1	0	1	1	1	0	7.21	7.15	7.91	6.12	7.15	0	0	0	0	0	0	0	9.21	9.11	9.14	9.35	9.39	9.48	8.55
1371032_at	Nid1	1	0	0	0	1	0	7.22	7.23	7.08	7.66	7.27	0	0	0	0	0	0	0	7.28	7.33	7.34	7.19	7.20	7.31	7.42
1371035_at	Gtf3a	1	0	0	0	1	1	6.36	6.22	5.93	6.71	5.88	0	0	0	0	0	0	0	8.77	8.74	8.81	8.75	8.73	8.31	8.52
1371036_at	Nrcam	1	0	1	0	1	0	7.25	7.48	7.91	7.04	7.07	0	0	0	0	0	0	0	9.78	9.80	9.89	9.99	9.99	9.60	9.65
1371038_at	Cebpg	1	0	0	1	1	0	6.65	6.43	7.06	5.85	6.41	0	0	0	0	0	0	0	8.83	8.83	8.85	8.97	8.87	8.99	7.81
1371040_at	Slc1a5	1	0	0	0	1	0	6.37	6.29	5.98	6.68	6.28	0	0	0	0	0	0	0	9.96	9.95	10.04	10.04	10.12	10.09	9.23
1371042_at	Map4k3	1	0	0	0	1	0	6.20	6.11	6.70	5.80	6.16	0	0	0	0	0	0	0	8.46	8.50	8.47	8.47	8.49	8.34	7.53
1371051_at	Myzap /// Polr2	1	0	0	1	1	0	6.15	6.07	6.73	5.26	5.99	0	0	0	0	0	0	0	8.54	8.68	8.71	8.73	8.65	8.34	7.36
1371055_at	Rab12	1	0	1	1	1	0	5.91	5.88	6.59	5.41	6.09	0	0	0	0	0	0	0	8.36	8.38	8.50	8.53	8.54	8.86	8.19
1371060_at	Trim23	1	0	1	1	1	0	3.95	4.03	5.32	3.16	3.96	0	0	0	0	0	0	0	8.26	8.36	8.45	8.53	8.44	8.40	7.10
1371065_at	Hla-dqb1	1	0	0	0	0	1	5.15	5.42	4.99	4.95	6.25	0	0	0	0	0	0	0	4.06	4.05	3.97	3.81	3.92	3.75	3.27
1371074_a_at	Mcm6	1	0	0	1	0	0	5.95	5.98	6.35	6.47	6.36	0	0	0	0	0	0	0	12.15	12.18	12.26	12.30	12.33	12.40	12.10
1371075_at	Myh13	1	0	1	0	0	0	4.69	4.56	4.08	4.49	4.31	0	0	0	0	0	0	0	4.70	4.79	4.83	4.63	4.59	4.58	4.43
1371081_at	Rapgef4	1	0	1	0	1	1	5.75	5.96	6.81	5.42	6.54	0	0	0	0	0	0	0	5.27	5.38	5.40	5.73	5.74	5.54	5.28
1371092_at	LOC286960	1	1	0	1	0	1	9.63	8.74	9.86	10.37	12.16	0	0	0	0	0	0	0	4.19	4.37	4.15	4.08	3.92	4.14	3.54
1371102_x_at	Hbb	1	1	0	1	1	1	8.78	8.17	8.52	7.60	7.49	0	0	0	0	0	0	0	3.46	3.55	3.46	3.38	3.37	3.54	3.17
1371103_at	Rab6a	1	0	0	1	1	0	7.06	7.19	7.63	6.20	7.11	0	0	0	0	0	0	0	9.90	9.82	9.88	9.92	9.86	9.80	8.89
1371104_at	Srebf1	1	0	0	0	1	0	5.30	5.10	5.03	5.60	5.13	0	0	0	0	0	0	0	6.88	6.85	6.99	6.63	6.66	6.53	6.03
1371108_a_at	Atp1a1	1	0	1	1	1	0	8.54	8.48	9.35	7.93	8.25	0	0	0	0	0	0	0	10.99	11.02	11.10	11.05	11.11	11.38	10.80
1371131_a_at	Txnip	1	1	0	1	0	0	11.41	10.49	11.17	10.59	10.98	0	0	0	0	0	0	0	13.13	13.14	13.16	13.09	13.19	13.54	13.15
1371136_at	Ppm1b	1	0	1	0	1	1	7.68	7.74	8.58	7.53	8.23	0	0	0	0	0	0	0	9.86	9.81	9.87	9.78	9.76	10.01	9.79
1371150_at	Ccnd1	1	0	0	0	0	1	7.72	7.94	8.23	7.85	8.41	0	0	0	0	0	0	0	6.37	6.49	6.52	6.49	6.57	6.26	6.04
1371151_at	Cpa3	1	0	0	0	0	1	5.51	5.56	5.42	5.59	4.90	0	0	0	0	0	0	0	4.35	4.28	4.35	4.19	4.23	4.31	3.99
1371173_a_at	Cast	1	0	1	0	0	0	6.35	6.63	6.97	6.44	6.33	0	0	0	0	0	0	0	7.41	7.24	7.34	7.50	7.21	7.07	6.14
1371189_x_at	Rpsa	1	0	0	0	1	1	11.01	11.05	10.86	11.46	10.33	0	0	0	0	0	0	0	10.97	10.99	10.94	10.90	10.87	10.82	10.51
1371190_at	---	1	0	0	0	1	1	8.62	8.68	8.14	8.90	7.83	0	0	0	0	0	0	0	7.00	6.90	6.87	6.86	6.86	6.87	6.88
1371229_at	Atpif1	1	0	0	0	0	1	8.11	8.21	8.20	8.18	8.60	0	0	0	0	0	0	0	5.81	5.67	5.74	5.70	5.74	5.70	5.40
1371245_a_at	Hbb	1	0	0	1	1	1	8.71	8.36	8.69	7.98	5.23	0	0	0	0	0	0	0	3.45	3.43	3.48	3.42	3.46	3.46	3.07
1371262_at	IgG-2a /// Igha	1	0	1	0	1	0	5.56	5.36	4.60	5.25	5.76	0	0	0	0	0	0	0	4.13	4.26	4.05	4.03	3.89	4.07	3.77
1371266_at	Afm	1	0	0	0	0	1	7.54	7.47	7.21	7.53	6.98	0	0	0	0	0	0	0	6.15	6.21	6.12	5.97	5.85	5.98	5.90
1371303_at	Tdh	1	0	1	1	1	1	10.55	10.70	5.36	6.18	8.68	0	0	0	0	0	0	0	4.91	4.99	4.96	4.68	4.86	4.76	4.11
1371310_s_at	Serpinh1	1	0	0	0	0	1	8.89	8.56	8.81	9.27	8.29	0	0	0	0	0	0	0	3.77	3.69	3.73	3.70	3.65	3.80	3.14
1371313_at	Rpl23a	1	0	0	0	0	1	11.78	11.66	11.98	12.13	11.31	0	0	0	0	0	0	0	12.68	12.68	12.65	12.61	12.58	12.55	12.24
1371320_at	Itm2b	1	0	1	0	1	0	11.13	11.15	11.77	10.81	11.41	0	0	0	0	0	0	0	12.39	12.37	12.32	12.25	12.38	12.47	12.01
1371327_a_at	Actg1 /// LOC10	1	0	0	0	1	0	11.52	11.49	11.92	11.12	11.88	0	0	0	0	0	0	0	13.24	13.25	13.28	13.28	13.24	13.16	13.13
1371332_at	H1f2	1	0	0	1	1	0	10.50	10.49	10.58	11.74	10.59	0	0	0	0	0	0	0	6.54	6.53	6.64	6.62	6.69	6.60	6.43
1371337_at	Cox7a2l	1	0	0	1	0	0	10.18	10.01	10.58	10.78	9.85	0	0	0	0	0	0	0	11.86	11.88	11.84	11.74	11.73	11.80	11.50
1371341_at	Snrpd2	1	0	0	1	0	0	9.70	9.76	9.91	10.39	9.68	0	0	0	0	0	0	0	11.54	11.49	11.50	11.52	11.46	11.56	11.52
1371368_at	Sec61a1	1	0	0	0	1	0	10.80	10.77	10.54	11.19	10.81	0	0	0	0	0	0	0	11.42	11.45	11.49	11.50	11.51	11.33	11.13
1371373_at	Rnf181	1	0	0	0	0	1	8.26	8.37	8.60	8.04	8.84	0	0	0	0	0	0	0	9.34	9.34	9.32	9.31	9.31	9.10	8.69
1371376_at	H1-10	1	0	0	1	1	0	6.65	6.91	6.48	7.15	6.64	0	0	0	0	0	0	0	7.56	7.45	7.57	7.49	7.40	7.26	7.25
1371377_at	Rps19	1	0	0	1	0	0	12.30	12.14	12.63	12.94	11.84	0	0	0	0	0	0	0	12.84	12.86	12.83	12.82	12.79	12.66	12.32
1371394_x_at	---	1	0	0	0	0	1	6.91	6.91	6.91	7.07	7.50	0	0	0	0	0	0	0	7.77	7.79	7.83	7.69	7.75	7.87	7.93
1371397_at	Nosip	1	0	0	0	1	0	6.34	6.44	6.12	6.70	5.93	0	0	0	0	0	0	0	8.12	8.23	8.24	8.28	8.21	8.33	8.23
1371398_at	Atp5j2 /// LOC68	1	0	0	0	1	0	10.52	10.62	10.15	10.79	10.18	0	0	0	0	0	0	0	12.14	12.14	12.13	12.15	12.14	12.09	11.91
1371399_at	Tomm6	1	0	0	0	1	0	9.97	9.94	9.61	10.29	9.74	0	0	0	0	0	0	0	11.17	11.16	11.10	11.07	10.99	10.88	10.68
1371402_at	Atp6v1b2	1	0	0	0	1	0	9.47	9.45	9.98	9.10	9.81	0	0	0	0	0	0	0	11.72	11.72	11.75	11.74	11.73	11.73	11.58
1371404_at	Eif4b	1	0	0	0	0	1	9.37	8.94	9.59	9.48	8.89	0	0	0	0	0	0	0	10.78	10.77	10.83	10.82	10.81	10.52	10.27
1371417_at	Uqcrq	1	0	0	0	1	0	10.99	11.30	10.70	11.43	10.96	0	0	0	0	0	0	0	11.44	11.43	11.40	11.48	11.42	11.57	11.39
1371418_at	Cct2	1	0	0	0	1	0	8.81	8.63	9.26	8.54	8.68	0	0	0	0	0	0	0	11.45	11.45	11.48	11.55	11.56	11.81	11.69
1371422_at	Morf4l2	1	0	0	0	0	1	11.49	11.49	11.92	11.28	11.99	0	0	0	0	0	0	0	12.85	12.90	12.89	12.88	12.86	12.69	12.38
1371432_at	Vat1	1	0	0	0	1	0	7.53	7.51	7.12	7.93	7.55	0	0	0	0	0	0	0	8.47	8.41	8.58	8.36	8.45	8.35	8.61
1371442_at	Hyou1	1	0	0	0	0	1	9.20	9.34	9.40	9.51	8.20	0	0	0	0	0	0	0	7.63	7.67	7.70	7.67	7.66	7.44	6.99
1371446_at	Mapkapk2	1	0	0	0	1	0	8.66	8.78	8.25	8.89	8.55	0	0	0	0	0	0	0	10.64	10.59	10.60	10.45	10.52	10.36	10.12
1371448_at	---	1	0	0	1	1	0	9.03	8.96	8.82	9.51	8.93	0	0	0	0	0	0	0	8.82	8.82	8.91	8.85	8.87	9.10	9.34
1371452_at	Ubl7	1	0	0	0	1	0	6.58	6.83	6.24	6.87	6.59	0	0	0	0	0	0	0	6.95	6.87	7.05	7.14	6.98	6.95	7.17
1371466_at	Trappc2	1	0	0	0	1	1	7.88	8.00	8.43	7.69	8.37	0	0	0	0	0	0	0	8.57	8.60	8.69	8.71	8.77	8.98	8.90
1371468_at	Rab11fip5	1	0	0	1	1	0	7.13	7.18	6.93	7.75	7.02	0	0	0	0	0	0	0	7.39	7.38	7.39	7.29	7.35	7.74	7.92
1371472_at	---	1	1	0	1	1	0	8.44	7.97	8.96	7.75	8.26	0	0	0	0	0	0	0	3.49	3.40	3.57	3.41	3.45	3.59	3.39
1371475_at	Rnase4	1	0	1	1	0	0	7.55	7.17	8.35	8.82	7.96	0	0	0	0	0	0	0	4.10	3.98	4.08	3.98	3.95	4.03	3.89
1371478_at	Rab5if	1	0	1	0	1	0	9.92	9.80	9.37	10.02	9.75	0	0	0	0	0	0	0	11.45	11.40	11.44	11.43	11.39	11.31	11.25
1371481_at	Reep5	1	0	1	0	1	1	11.65	11.49	10.85	11.58	11.08	0	0	0	0	0	0	0	11.72	11.66	11.68	11.61	11.57	11.33	11.48
1371494_at	Lancl1	1	0	0	0	0	1	7.53	7.42	7.67	7.64	8.04	0	0	0	0	0	0	0	7.69	7.80	7.84	7.73	7.93	8.09	8.56
1371496_at	Smim24	1	1	0	0	0	0	6.98	7.65	7.04	7.44	7.21	0	0	0	0	0	0	0	7.91	7.82	7.93	7.79	7.78	7.73	7.68
1371502_at	Rbm42	1	0	0	0	1	0	8.36	8.59	8.17	8.74	8.44	0	0	0	0	0	0	0	10.33	10.22	10.31	10.31	10.20	9.99	9.83
1371517_at	Grb10	1	0	0	1	1	0	7.14	6.77	7.04	8.03	6.90	0	0	0	0	0	0	0	3.79	3.67	3.82	3.64	3.65	3.59	3.15
1371519_at	Etfdh	1	1	0	0	0	1	7.59	6.89	7.61	7.13	6.94	0	0	0	0	0	0	0	8.77	8.82	8.92	8.98	9.07	9.23	8.79
1371521_at	Sin3b	1	0	0	0	0	1	8.80	8.51	8.74	8.73	8.32	0	0	0	0	0	0	0	11.37	11.36	11.30	11.20	11.14	11.06	11.16
1371527_at	Emp1	1	0	0	1	1	0	7.85	7.74	8.31	7.31	7.60	0	0	0	0	0	0	0	3.66	3.64	3.76	3.69	3.67	3.65	3.26
1371536_at	Carhsp1	1	0	0	0	0	1	8.71	8.51	8.49	8.97	8.22	0	0	0	0	0	0	0	10.65	10.70	10.68	10.66	10.75	11.12	11.08
1371540_at	Krtcap2	1	0	0	0	1	1	11.06	10.92	10.70	11.28	10.60	0	0	0	0	0	0	0	11.43	11.48	11.45	11.49	11.52	11.40	11.20
1371542_at	Tuba4a	1	0	0	0	1	0	9.06	9.02	8.84	9.47	9.16	0	0	0	0	0	0	0	10.73	10.79	10.76	10.73	10.69	10.31	10.01
1371551_at	Traf4	1	0	0	0	1	0	7.17	7.16	7.03	7.60	6.94	0	0	0	0	0	0	0	8.48	8.48	8.54	8.47	8.45	8.29	8.46
1371556_at	Pias4	1	0	0	0	1	0	5.94	6.32	5.63	6.26	5.94	0	0	0	0	0	0	0	7.09	7.06	7.14	7.06	7.09	7.24	7.19
1371561_at	G6pc3	1	0	0	1	0	0	7.42	7.63	7.43	7.89	7.50	0	0	0	0	0	0	0	9.10	9.10	9.14	9.11	9.07	9.02	8.79
1371563_at	Rcc2	1	0	0	1	0	0	8.42	8.30	8.40	8.91	8.38	0	0	0	0	0	0	0	9.51	9.53	9.63	9.64	9.62	9.61	9.24
1371567_at	Aldh7a1	1	0	0	0	0	1	10.08	9.77	9.58	10.09	9.29	0	0	0	0	0	0	0	11.08	11.03	10.96	10.93	10.89	11.15	11.10
1371570_at	---	1	0	0	0	0	1	4.47	4.62	4.52	4.60	5.00	0	0	0	0	0	0	0	8.86	8.83	8.97	8.85	8.80	8.36	8.37
1371573_at	Rpl36a	1	0	0	1	0	0	11.42	11.32	11.69	12.03	11.10	0	0	0	0	0	0	0	12.52	12.57	12.54	12.60	12.62	12.65	12.25
1371574_at	Ghitm	1	0	0	1	0	0	9.18	8.96	9.56	9.72	9.24	0	0	0	0	0	0	0	11.50	11.52	11.52	11.51	11.49	11.51	11.25
1371587_at	Lamtor3	1	0	0	0	1	0	7.60	7.45	8.10	7.30	7.65	0	0	0	0	0	0	0	10.53	10.52	10.55	10.55	10.55	10.52	10.28
1371595_at	---	1	0	1	0	1	0	9.43	9.40	8.32	9.75	9.30	0	0	0	0	0	0	0	11.95	11.99	12.18	11.90	12.06	12.02	11.25
1371599_at	LOC294154	1	0	0	1	1	0	9.16	9.07	9.00	9.72	8.98	0	0	0	0	0	0	0	10.18	10.17	10.22	10.10	10.07	10.19	10.48
1371601_at	Man1b1	1	0	0	0	1	0	9.36	9.19	8.98	9.68	9.15	0	0	0	0	0	0	0	10.19	10.25	10.22	10.16	10.17	9.97	9.61
1371608_at	Mrps34	1	0	0	0	1	0	8.73	8.72	8.26	8.93	8.45	0	0	0	0	0	0	0	9.61	9.58	9.62	9.61	9.50	9.26	8.94
1371610_at	Tnks	1	0	1	0	0	1	8.21	8.25	8.85	8.26	8.78	0	0	0	0	0	0	0	11.01	11.01	11.02	10.97	11.00	10.99	10.86
1371616_at	---	1	0	1	0	1	0	7.19	7.04	7.92	6.96	7.54	0	0	0	0	0	0	0	9.56	9.65	9.65	9.61	9.72	9.49	9.34
1371619_at	C5orf15	1	0	0	0	0	1	8.24	8.27	8.61	8.27	8.90	0	0	0	0	0	0	0	9.85	9.84	9.89	9.84	9.97	9.91	9.73
1371625_at	Pygb	1	0	0	0	1	0	6.35	6.32	5.95	6.62	6.32	0	0	0	0	0	0	0	9.53	9.54	9.61	9.53	9.60	9.90	9.85
1371642_at	Eif4a2	1	0	0	0	1	0	11.61	11.44	11.95	11.14	11.68	0	0	0	0	0	0	0	12.96	13.00	12.97	12.96	13.04	12.84	12.26
1371649_at	Mrps24	1	0	0	0	1	1	9.82	9.80	9.33	10.19	9.25	0	0	0	0	0	0	0	10.72	10.72	10.72	10.74	10.72	10.74	10.73
1371658_at	Emc8	1	0	0	0	1	0	7.11	7.05	6.97	7.55	6.96	0	0	0	0	0	0	0	8.45	8.52	8.60	8.68	8.66	8.58	8.43
1371668_at	---	1	0	0	1	1	0	6.45	6.57	6.17	6.96	6.33	0	0	0	0	0	0	0	8.41	8.47	8.41	8.24	8.39	8.32	8.46
1371671_at	---	1	0	1	0	1	0	6.17	6.39	6.87	5.80	6.55	0	0	0	0	0	0	0	8.10	8.04	8.16	8.04	8.09	8.31	7.73
1371679_at	Synpo2	1	0	0	0	0	1	6.56	6.70	7.08	6.47	7.05	0	0	0	0	0	0	0	7.67	7.67	7.60	7.37	7.44	7.11	6.50
1371681_at	Tox4	1	0	0	0	0	1	5.84	5.85	6.28	6.03	6.52	0	0	0	0	0	0	0	8.43	8.48	8.45	8.52	8.44	8.51	8.38
1371682_at	Map1lc3a	1	0	0	0	0	1	8.70	8.66	8.30	8.84	8.23	0	0	0	0	0	0	0	9.75	9.78	9.84	9.80	9.81	10.06	9.79
1371686_at	Canx	1	0	1	0	0	0	10.45	10.56	11.04	10.36	10.85	0	0	0	0	0	0	0	11.26	11.36	11.39	11.43	11.51	11.60	11.26
1371687_at	Canx	1	1	0	0	0	0	9.32	9.84	9.62	9.45	9.28	0	0	0	0	0	0	0	10.41	10.51	10.50	10.55	10.63	10.57	9.72
1371694_at	Dpysl2	1	0	0	0	1	0	10.19	10.25	10.59	9.88	10.26	0	0	0	0	0	0	0	11.84	11.88	11.94	11.96	11.95	11.95	11.70
1371700_at	Mfap4	1	0	0	0	0	1	6.69	6.31	6.84	6.22	6.10	0	0	0	0	0	0	0	3.71	3.76	3.72	3.60	3.77	3.71	3.33
1371702_at	Tspan7	1	0	1	0	1	0	11.02	11.10	11.63	10.77	11.40	0	0	0	0	0	0	0	11.79	11.83	11.88	11.90	11.90	12.26	12.02
1371711_at	Reep5	1	0	1	0	1	0	8.99	8.77	8.41	9.38	8.92	0	0	0	0	0	0	0	9.98	9.99	10.03	10.06	10.04	9.87	10.03
1371716_at	Smarcc2	1	0	0	0	1	0	8.12	8.00	7.89	8.56	8.15	0	0	0	0	0	0	0	8.77	8.81	8.84	8.76	8.83	8.48	8.41
1371724_at	Rexo2	1	0	1	0	1	0	10.45	10.49	9.90	10.55	10.25	0	0	0	0	0	0	0	9.61	9.58	9.68	9.84	9.79	9.56	9.48
1371732_at	Dpt	1	1	0	1	1	1	8.00	7.26	8.13	7.34	6.75	0	0	0	0	0	0	0	4.11	4.13	4.08	4.03	4.16	4.32	3.65
1371739_at	LETM1	1	0	0	0	1	0	6.94	6.95	6.75	7.32	7.17	0	0	0	0	0	0	0	9.62	9.58	9.75	9.79	9.84	9.82	10.01
1371748_at	Agpat1	1	0	0	1	1	0	6.68	6.73	6.56	7.16	6.80	0	0	0	0	0	0	0	7.58	7.58	7.71	7.72	7.74	7.77	8.11
1371755_at	C5orf24	1	0	1	0	1	1	9.30	9.32	10.03	9.20	9.79	0	0	0	0	0	0	0	11.05	11.12	11.13	11.15	11.19	11.13	11.04
1371757_s_at	LOC683313	1	0	0	0	0	1	4.07	4.15	3.97	4.09	4.60	0	0	0	0	0	0	0	4.44	4.29	4.34	4.29	4.33	4.53	4.04

Probeset ID	Gene Symbol	Islet Data Set (log2 ratio ≥0.263; FDR <20%) union n=5325 1=YES 0=NO	Islet DRlyp/lyp ND vs ND+Lp299v (log2 ratio ≥0.263; FDR<20%) n=429 1=YES 0=NO	Islet DRlyp/lyp ND vs HCD (log2 ratio ≥0.263; FDR<20%) n=2247 1=YES 0=NO	Islet DRlyp/lyp ND vs HCD+Lp299v (log2 ratio ≥0.263; FDR<20%) n=1595 1=YES 0=NO	Islet DRlyp/lyp HCD vs HCD+Lp299v (log2 ratio ≥0.263; FDR<20%) n=3026 1=YES 0=NO	Islet DRlyp/lyp ND vs Flyp/lyp ND (log2 ratio ≥ 0.263; FDR<20%) n=1950 1=YES 0=NO	Islet Mean Log2 Intensity DRlyp/lyp ND	Islet Mean Log2 Intensity DRlyp/lyp ND+Lp299v	Islet Mean Log2 Intensity DRlyp/lyp HCD	Islet Mean Log2 Intensity DRlyp/lyp HCD+Lp299v	Islet Mean Log2 Intensity Flyp40 ND	Rinm5f data union of all conditions n=3705 1=YES 0=NO	Rinm5f 30 µM propionate/6 µM butyrate vs Media (FDR<20%; 1.5 fold) n=3 1=YES 0=NO	Rinm5f 60 µM propionate/12 µM butyrate vs Media (FDR<20%; 1.5 fold) n=18 1=YES 0=NO	Rinm5f 120 µM propionate/24 µM butyrate vs Media (FDR<20%; 1.5 fold) n=164 1=YES 0=NO	Rinm5f 240 µM propionate/48 µM butyrate vs Media (FDR<20%; 1.5 fold) n=268 1=YES 0=NO	Rinm5f 1500 µM propionate/300 µM butyrate vs Media (FDR<20%; 1.5 fold) n=2491 1=YES 0=NO	Rinm5f 500 µM propionate/500 µM butyrate vs Media (FDR<20%; 1.5 fold) n=3001 1=YES 0=NO	Rinm5f Mean Log2 Intensity Media only	Rinm5f Mean Log2 Intensity 30 µM propionate/6 µM butyrate	Rinm5f Mean Log2 Intensity 60 µM propionate/12 µM butyrate	Rinm5f Mean Log2 Intensity 120 µM propionate/24 µM butyrate	Rinm5f Mean Log2 Intensity 240 µM propionate/48 µM butyrate	Rinm5f Mean Log2 Intensity 1500 µM propionate/300 µM butyrate	Rinm5f Mean Log2 Intensity 500 µM propionate/500 µM butyrate
1371765_at	H2ac6	1	0	1	0	0	0	6.24	6.13	6.83	6.50	6.57	0	0	0	0	0	0	0	3.47	3.46	3.47	3.52	3.43	3.46	3.27
1371776_at	---	1	1	0	0	0	1	5.76	5.19	5.99	5.43	6.60	0	0	0	0	0	0	0	6.65	6.35	6.58	6.45	6.55	6.59	5.99
1371777_at	Pabpc4	1	0	0	0	0	1	11.36	10.97	10.98	11.46	10.85	0	0	0	0	0	0	0	9.93	9.90	9.94	10.03	9.95	9.57	9.15
1371779_at	Snx6	1	0	1	0	1	0	7.04	7.06	7.67	6.82	7.36	0	0	0	0	0	0	0	9.99	9.99	10.05	10.06	9.98	9.79	9.67
1371782_at	Nipsnap3a	1	1	0	0	0	1	8.06	7.57	7.76	8.19	6.71	0	0	0	0	0	0	0	9.00	8.91	8.94	8.97	8.86	8.89	8.93
1371784_at	Dnajc7	1	0	0	0	1	0	7.71	7.58	7.52	8.14	7.44	0	0	0	0	0	0	0	10.67	10.68	10.73	10.71	10.73	10.45	10.16
1371790_at	Mrpl45	1	0	0	1	0	0	6.92	7.07	6.99	7.41	6.76	0	0	0	0	0	0	0	9.13	9.13	9.15	9.03	8.93	8.61	8.57
1371791_at	Surf4	1	0	0	0	1	0	8.63	8.71	8.27	9.02	8.70	0	0	0	0	0	0	0	10.27	10.24	10.25	10.22	10.26	9.95	9.72
1371797_at	Cad	1	0	0	1	1	0	6.32	6.44	6.16	6.79	6.72	0	0	0	0	0	0	0	7.76	7.65	7.75	7.79	7.66	7.42	7.63
1371798_at	Gna12	1	0	0	0	0	1	7.15	7.21	7.33	7.14	7.65	0	0	0	0	0	0	0	10.14	10.09	10.04	9.77	9.84	9.59	9.96
1371802_at	Cope	1	0	0	0	1	0	9.78	9.97	9.41	10.01	9.65	0	0	0	0	0	0	0	11.21	11.25	11.20	11.14	11.11	11.08	10.94
1371803_at	Gm2a	1	0	1	0	1	1	7.98	8.04	7.30	8.17	7.26	0	0	0	0	0	0	0	7.50	7.58	7.54	7.51	7.54	7.68	7.87
1371817_at	Isyna1	1	0	0	1	0	0	7.52	7.76	7.75	7.99	7.93	0	0	0	0	0	0	0	10.15	10.22	10.25	10.26	10.20	10.11	9.38
1371819_at	Hdac5	1	0	0	1	0	0	7.57	7.77	7.54	8.06	7.50	0	0	0	0	0	0	0	9.64	9.55	9.54	9.41	9.39	9.40	9.36
1371823_at	Stag2	1	0	1	0	0	0	8.94	8.91	9.53	8.90	9.09	0	0	0	0	0	0	0	10.53	10.56	10.58	10.60	10.56	10.37	10.00
1371828_at	Fam216a	1	0	0	0	1	0	6.95	6.93	7.38	6.64	7.07	0	0	0	0	0	0	0	10.35	10.33	10.33	10.36	10.30	10.08	9.76
1371835_at	Prkacb	1	0	0	0	1	0	9.48	9.49	10.04	9.17	9.71	0	0	0	0	0	0	0	10.91	10.90	10.96	10.94	10.89	10.60	10.55
1371837_at	Ddx5	1	0	1	0	1	0	10.29	10.37	10.91	10.13	10.55	0	0	0	0	0	0	0	12.62	12.64	12.62	12.66	12.65	12.34	11.87
1371839_at	Srsf2	1	0	0	0	1	0	7.74	7.82	8.18	7.43	7.85	0	0	0	0	0	0	0	10.67	10.67	10.78	10.83	10.77	10.62	9.95
1371840_at	S1pr1	1	0	0	1	1	0	7.13	6.69	7.14	6.43	6.94	0	0	0	0	0	0	0	3.58	3.74	3.75	3.79	3.74	3.82	3.34
1371864_at	---	1	1	0	0	0	1	10.30	9.73	10.40	10.35	9.78	0	0	0	0	0	0	0	9.65	9.69	9.74	9.77	9.80	10.09	10.30
1371867_at	---	1	0	0	0	1	0	7.93	7.84	8.30	7.57	7.96	0	0	0	0	0	0	0	9.82	9.83	9.83	9.75	9.74	9.86	9.42
1371871_at	Rab12	1	0	0	0	1	0	8.51	8.55	8.96	8.19	8.91	0	0	0	0	0	0	0	10.31	10.34	10.35	10.43	10.45	10.79	10.76
1371873_at	Anp32e	1	0	0	0	1	0	7.93	7.98	8.41	7.69	8.38	0	0	0	0	0	0	0	10.80	10.85	10.92	10.92	10.94	10.76	10.41
1371896_at	Gadd45gip1	1	0	0	1	1	0	7.76	8.13	7.47	8.32	7.36	0	0	0	0	0	0	0	8.19	8.13	8.17	8.31	8.12	7.93	7.77
1371909_at	Med23	1	0	0	0	0	1	6.36	6.42	6.55	6.34	6.99	0	0	0	0	0	0	0	8.67	8.58	8.68	8.75	8.68	8.58	8.39
1371916_at	Msrb1	1	0	1	0	1	1	9.26	9.13	8.48	9.34	8.69	0	0	0	0	0	0	0	8.35	8.43	8.40	8.49	8.49	8.77	8.83
1371920_at	Poldip2	1	0	0	0	1	0	8.18	8.08	7.80	8.41	8.03	0	0	0	0	0	0	0	8.99	8.96	9.03	9.07	9.04	8.85	8.45
1371921_at	Ctnna1	1	0	1	0	0	0	10.00	10.02	10.60	9.97	10.26	0	0	0	0	0	0	0	11.97	11.96	11.98	11.99	12.03	12.31	12.11
1371922_at	Car12	1	0	1	1	1	0	6.38	6.33	7.42	9.42	6.44	0	0	0	0	0	0	0	3.69	3.75	3.67	3.51	3.48	3.72	5.78
1371925_at	Atp13a1	1	0	0	0	1	0	8.29	8.43	7.79	8.71	8.04	0	0	0	0	0	0	0	9.97	9.96	10.09	9.99	9.96	9.84	9.77
1371930_at	Qars	1	0	0	0	0	1	9.22	8.90	9.21	9.60	8.65	0	0	0	0	0	0	0	8.74	8.74	8.86	8.86	8.82	8.95	8.67
1371933_at	Pde4dip	1	0	0	0	0	1	5.29	5.67	5.74	5.28	6.23	0	0	0	0	0	0	0	7.40	7.43	7.54	7.48	7.51	7.23	7.35
1371939_at	Caprin1	1	0	0	0	1	0	8.65	8.54	9.19	8.31	8.80	0	0	0	0	0	0	0	10.96	10.91	10.98	11.07	10.96	10.71	10.27
1371942_at	Gstt3	1	0	1	1	1	1	7.07	6.98	5.79	6.43	6.33	0	0	0	0	0	0	0	3.73	3.68	3.60	3.61	3.58	3.63	3.51
1371947_at	Ndn	1	0	1	0	1	0	7.57	7.55	8.17	7.28	7.73	0	0	0	0	0	0	0	10.56	10.62	10.66	10.75	10.73	10.46	10.31
1371949_at	Bzw1	1	0	0	1	1	0	8.02	7.83	8.33	7.52	7.83	0	0	0	0	0	0	0	9.67	9.65	9.72	9.75	9.65	9.55	9.07
1371951_at	Fhl2	1	0	0	0	0	1	5.10	5.30	5.43	4.99	5.60	0	0	0	0	0	0	0	8.15	8.09	8.04	8.14	8.04	8.09	8.12
1371952_at	Rbm18	1	0	1	0	0	0	6.45	6.38	7.08	6.44	6.80	0	0	0	0	0	0	0	9.64	9.74	9.75	9.87	9.84	9.82	9.70
1371953_at	Ccng2	1	0	1	0	0	1	5.75	6.02	6.50	5.87	6.28	0	0	0	0	0	0	0	9.46	9.48	9.31	9.03	8.98	8.95	8.82
1371956_at	Pja1	1	0	1	0	1	1	8.65	8.79	9.42	8.50	9.77	0	0	0	0	0	0	0	11.97	11.99	11.94	11.89	11.93	11.81	11.33
1371959_at	H2ac18 /// H2ac	1	0	1	1	1	0	9.14	9.05	8.30	10.02	8.69	0	0	0	0	0	0	0	5.43	5.49	5.54	5.40	5.44	5.12	5.02
1371960_at	Ythdf2	1	0	0	0	0	1	7.19	7.13	7.53	7.26	6.60	0	0	0	0	0	0	0	9.95	9.85	10.03	10.03	10.02	10.04	9.55
1371970_at	Fam111a	1	0	0	0	0	1	11.39	11.44	11.50	11.21	4.28	0	0	0	0	0	0	0	9.63	9.59	9.67	9.80	9.74	9.13	9.07
1371976_at	Mcrip2	1	0	1	0	1	1	8.04	7.69	7.17	8.15	7.49	0	0	0	0	0	0	0	7.30	7.33	7.31	7.57	7.51	7.77	7.07
1371981_at	Vps26c	1	0	1	0	1	0	6.81	6.70	7.41	6.72	7.22	0	0	0	0	0	0	0	8.74	8.86	8.84	8.77	8.79	8.90	8.52
1371989_at	Hmgn3	1	0	1	1	1	0	10.66	10.69	11.38	10.17	11.00	0	0	0	0	0	0	0	10.53	10.55	10.69	10.90	10.90	10.91	10.52
1371990_at	Mpnd	1	0	1	0	1	1	8.79	8.76	8.18	8.95	8.23	0	0	0	0	0	0	0	9.21	9.28	9.28	9.28	9.30	9.32	9.16
1371992_at	Babam1	1	0	0	0	1	0	8.83	9.09	8.51	9.19	8.58	0	0	0	0	0	0	0	10.57	10.53	10.62	10.64	10.59	10.51	10.19
1371996_at	Aebp2	1	0	1	0	0	0	7.91	7.92	8.52	7.92	8.31	0	0	0	0	0	0	0	9.41	9.44	9.42	9.28	9.33	9.17	8.87
1372003_at	Tmem87a	1	0	1	0	1	0	7.90	7.89	8.51	7.81	8.12	0	0	0	0	0	0	0	10.14	10.19	10.25	10.32	10.29	10.15	9.67
1372006_at	---	1	0	0	0	0	1	7.21	7.26	7.49	7.16	8.13	0	0	0	0	0	0	0	6.93	6.93	6.94	7.06	7.14	6.74	6.00
1372012_at	Dhcr24	1	0	1	0	1	0	7.16	7.35	6.48	7.36	7.45	0	0	0	0	0	0	0	9.39	9.37	9.42	9.54	9.57	9.78	9.96
1372025_at	---	1	0	0	0	0	1	3.70	3.93	4.05	3.58	4.34	0	0	0	0	0	0	0	5.46	5.57	5.75	5.49	5.43	5.69	5.62
1372033_at	Isoc2b	1	0	0	0	1	0	7.25	7.22	6.81	7.47	7.15	0	0	0	0	0	0	0	7.48	7.48	7.44	7.25	7.27	7.74	7.67
1372044_at	Tango2	1	0	0	0	1	0	7.71	7.45	7.29	7.96	7.34	0	0	0	0	0	0	0	8.93	9.02	9.02	9.01	8.96	9.13	8.83
1372046_at	Gtf3c3	1	0	1	0	1	1	5.87	5.84	6.76	5.99	6.39	0	0	0	0	0	0	0	8.12	8.10	8.19	8.22	8.26	7.85	7.48
1372048_at	Podxl2	1	1	0	0	0	0	7.25	7.74	7.39	7.69	7.44	0	0	0	0	0	0	0	8.99	9.09	9.04	8.97	9.05	9.36	8.99
1372060_at	Lysmd4	1	0	0	0	0	1	5.14	5.21	5.52	5.35	3.49	0	0	0	0	0	0	0	8.01	8.04	8.13	8.10	8.07	8.09	7.65
1372067_at	Tmx1	1	0	0	0	1	0	9.08	9.15	9.64	8.77	9.46	0	0	0	0	0	0	0	11.78	11.85	11.80	11.81	11.87	11.90	11.53
1372071_at	Cd320 /// LOC10	1	0	0	0	0	1	8.14	7.95	7.90	8.21	7.57	0	0	0	0	0	0	0	9.79	9.82	9.78	9.79	9.80	9.96	9.63
1372079_at	Mbnl1	1	0	0	1	0	0	9.34	9.29	9.79	10.02	9.40	0	0	0	0	0	0	0	10.09	10.05	10.15	10.21	10.11	9.94	9.96
1372102_at	Ncor1	1	0	0	0	0	1	7.38	7.14	7.29	7.41	8.21	0	0	0	0	0	0	0	7.81	7.79	7.77	7.76	7.74	7.77	8.11
1372108_at	Ptcd3	1	0	0	0	1	1	8.53	8.27	9.10	8.17	8.02	0	0	0	0	0	0	0	9.62	9.60	9.69	9.84	9.77	9.50	9.05
1372110_at	Limch1	1	0	1	0	1	1	7.60	7.95	8.34	7.57	8.56	0	0	0	0	0	0	0	8.55	8.51	8.56	8.61	8.56	8.42	8.43
1372118_at	Nsg2	1	0	1	0	1	0	6.88	7.02	7.54	6.80	7.26	0	0	0	0	0	0	0	8.37	8.41	8.56	8.54	8.59	8.40	8.07
1372120_at	Uba5	1	0	0	0	0	1	7.46	7.49	7.54	7.41	8.39	0	0	0	0	0	0	0	7.72	7.76	7.93	8.14	8.01	7.66	7.34
1372121_at	Usp9x	1	0	0	0	1	1	8.33	8.39	8.88	8.08	8.91	0	0	0	0	0	0	0	10.04	10.14	10.20	10.23	10.24	10.07	9.78
1372124_at	Eif4b	1	0	0	0	0	1	8.68	8.45	8.76	8.82	7.89	0	0	0	0	0	0	0	10.39	10.46	10.39	10.33	10.26	10.21	9.50
1372128_at	Mrps12	1	0	0	0	1	0	8.49	8.55	8.11	8.77	8.27	0	0	0	0	0	0	0	10.21	10.21	10.21	10.25	10.19	10.46	10.53
1372131_at	Ubqln2	1	0	0	0	0	1	9.40	9.60	9.90	9.34	9.89	0	0	0	0	0	0	0	11.30	11.26	11.30	11.35	11.29	11.10	11.07
1372138_at	Cux1	1	0	0	1	1	0	8.14	8.25	7.97	8.65	7.99	0	0	0	0	0	0	0	9.54	9.55	9.62	9.54	9.52	9.41	9.38
1372146_at	---	1	0	1	0	1	0	6.69	6.69	7.35	6.64	6.99	0	0	0	0	0	0	0	8.80	8.80	8.86	8.84	8.90	8.87	8.91
1372151_at	Tcerg1	1	0	1	0	1	0	7.40	7.41	8.30	7.30	7.87	0	0	0	0	0	0	0	10.72	10.84	10.85	10.98	11.01	10.87	10.18
1372156_at	Tmem97	1	0	1	0	1	0	11.26	11.39	10.47	11.57	10.97	0	0	0	0	0	0	0	9.87	9.88	9.88	9.85	9.87	9.85	9.73
1372158_at	Macrod1	1	0	1	0	1	1	8.32	8.20	7.41	8.37	7.65	0	0	0	0	0	0	0	8.63	8.60	8.59	8.41	8.43	8.64	8.46
1372162_at	Acss1	1	1	1	0	1	1	7.59	7.13	6.68	7.43	6.97	0	0	0	0	0	0	0	3.70	3.69	3.78	3.65	3.52	3.56	3.38
1372164_at	Wbp1l	1	0	0	1	1	0	7.22	7.17	7.23	7.79	7.03	0	0	0	0	0	0	0	9.74	9.75	9.75	9.73	9.77	9.59	9.75
1372183_at	---	1	0	1	1	1	0	6.62	6.60	7.35	5.94	6.85	0	0	0	0	0	0	0	9.52	9.45	9.59	9.64	9.62	9.52	9.20
1372189_at	Dnajc13	1	0	1	0	1	0	7.25	7.31	7.87	7.14	7.62	0	0	0	0	0	0	0	7.86	7.92	8.01	8.07	7.97	7.97	8.07
1372191_at	---	1	0	0	0	1	0	5.82	5.74	5.49	6.10	5.50	0	0	0	0	0	0	0	6.32	6.27	6.49	6.55	6.49	6.58	6.75
1372193_at	Impact	1	0	1	0	1	0	6.86	6.89	7.50	6.45	7.07	0	0	0	0	0	0	0	8.87	9.13	9.11	9.17	9.27	9.37	8.83
1372196_at	Ncor2	1	0	0	1	0	0	8.24	8.26	8.25	8.78	8.22	0	0	0	0	0	0	0	9.08	9.03	9.06	8.96	9.03	9.08	9.69
1372197_at	Rictor	1	0	1	0	0	0	7.70	7.71	8.29	7.66	7.77	0	0	0	0	0	0	0	8.70	8.81	8.84	8.87	8.85	8.78	8.50
1372199_at	---	1	0	0	0	1	1	6.68	6.66	6.21	6.91	6.20	0	0	0	0	0	0	0	5.92	5.95	5.96	5.84	5.86	5.70	5.89
1372204_at	Elk4	1	1	0	0	0	0	5.73	5.26	5.67	6.05	5.65	0	0	0	0	0	0	0	7.56	7.55	7.59	7.67	7.60	7.47	7.57
1372212_at	Paox	1	1	1	0	1	1	9.03	8.41	8.36	9.00	8.21	0	0	0	0	0	0	0	8.16	8.23	8.29	8.25	8.31	8.39	7.94
1372213_at	Depp1	1	1	0	1	0	1	7.63	6.94	7.26	6.83	6.80	0	0	0	0	0	0	0	5.81	6.01	5.78	5.71	5.74	5.73	5.73
1372223_at	Cpeb4	1	0	1	0	1	1	8.43	8.33	9.09	8.04	8.92	0	0	0	0	0	0	0	10.97	11.14	11.25	11.44	11.45	11.51	11.14
1372229_at	Pdk3	1	0	0	0	0	1	5.45	5.68	5.97	5.73	6.01	0	0	0	0	0	0	0	9.41	9.40	9.41	9.44	9.40	9.55	9.91
1372230_at	Tmem147	1	0	0	1	1	1	9.95	9.90	9.56	10.55	9.48	0	0	0	0	0	0	0	11.09	11.10	11.14	11.18	11.16	11.12	10.93
1372233_at	Ergic3	1	0	0	0	1	0	8.65	8.54	8.40	9.11	8.29	0	0	0	0	0	0	0	10.40	10.48	10.49	10.42	10.48	10.43	10.13
1372237_at	Fam219a	1	0	0	0	0	1	7.63	7.71	7.88	7.49	8.26	0	0	0	0	0	0	0	10.75	10.79	10.76	10.65	10.75	10.62	10.13
1372242_at	Ddx3x	1	0	0	0	1	0	9.73	9.75	10.29	9.42	9.77	0	0	0	0	0	0	0	11.59	11.61	11.63	11.71	11.67	11.24	11.06
1372245_at	Ciao1	1	0	1	1	1	1	9.07	8.75	7.98	8.54	8.52	0	0	0	0	0	0	0	9.63	9.56	9.61	9.68	9.60	9.44	9.12
1372248_at	Sesn1	1	1	0	0	0	0	7.92	7.17	7.66	7.77	7.60	0	0	0	0	0	0	0	8.26	8.31	8.26	8.15	8.17	8.41	8.91
1372253_at	Cnot8	1	0	0	0	1	0	7.16	6.78	7.59	6.83	6.94	0	0	0	0	0	0	0	8.60	8.65	8.74	8.66	8.67	8.67	8.29
1372256_at	Crip1	1	0	1	0	0	0	8.17	8.32	7.62	7.99	7.75	0	0	0	0	0	0	0	8.31	8.34	8.37	8.27	8.24	8.26	8.38
1372261_at	---	1	0	1	0	1	0	7.75	7.91	8.36	7.65	8.07	0	0	0	0	0	0	0	9.43	9.38	9.55	9.81	9.74	9.77	9.65
1372264_at	Pck1	1	0	0	0	0	1	4.70	4.79	4.43	4.57	5.27	0	0	0	0	0	0	0	4.51	4.51	4.48	4.27	4.35	4.47	4.16
1372266_at	Rev3l	1	0	1	0	1	0	6.86	6.82	7.77	6.75	7.09	0	0	0	0	0	0	0	9.97	10.00	10.00	10.01	10.00	9.96	9.52
1372268_at	Opcml	1	0	1	1	0	1	5.63	5.43	4.67	4.66	4.83	0	0	0	0	0	0	0	7.34	7.34	7.39	7.40	7.30	7.57	6.95
1372271_at	Rbm38	1	0	0	1	0	0	5.95	5.91	6.15	6.62	5.74	0	0	0	0	0	0	0	7.57	7.55	7.68	7.65	7.71	7.95	7.80
1372283_at	Trappc6b	1	0	1	0	1	0	7.73	7.67	8.40	7.36	8.02	0	0	0	0	0	0	0	10.08	10.13	10.13	10.24	10.24	9.96	9.44
1372288_at	Zmiz2	1	0	0	1	1	0	6.35	6.56	6.25	6.85	6.49	0	0	0	0	0	0	0	8.29	8.37	8.35	8.29	8.30	8.49	8.81
1372291_at	Oxa1l	1	0	0	0	0	1	8.90	8.62	8.77	9.19	8.33	0	0	0	0	0	0	0	8.76	8.80	8.80	8.78	8.75	8.81	8.51
1372294_at	Pxdn	1	1	0	0	0	1	5.90	5.45	5.62	5.52	5.30	0	0	0	0	0	0	0	6.51	6.61	6.70	6.69	6.79	6.82	6.51
1372303_at	Cyrib	1	0	0	0	1	0	7.27	7.14	7.81	7.04	7.62	0	0	0	0	0	0	0	10.75	10.79	10.76	10.79	10.75	10.82	10.56
1372305_at	Copz2	1	0	1	0	1	0	7.79	7.65	7.07	7.86	7.47	0	0	0	0	0	0	0	8.70	8.82	8.80	8.81	9.00	9.16	8.84
1372311_at	Gnpda1	1	0	0	0	0	1	7.27	6.88	7.54	7.03	7.83	0	0	0	0	0	0	0	9.83	9.83	9.83	9.81	9.86	10.26	9.91
1372318_at	Elovl6	1	0	0	0	1	1	6.83	6.87	7.19	6.44	7.33	0	0	0	0	0	0	0	7.41	7.47	7.59	7.70	7.67	7.45	7.01
1372325_at	Emilin1	1	0	0	0	0	1	7.35	7.62	7.28	7.52	8.36	0	0	0	0	0	0	0	7.35	7.47	7.56	7.56	7.62	7.59	7.13
1372330_at	Rnf130	1	0	0	0	0	1	10.00	10.14	10.42	9.84	10.51	0	0	0	0	0	0	0	10.17	10.23	10.24	10.26	10.37	10.47	10.07
1372333_at	Snrpe	1	0	0	1	0	0	10.08	9.96	10.31	10.61	9.98	0	0	0	0	0	0	0	12.15	12.18	12.17	12.16	12.17	12.11	11.70
1372338_at	Immp1l	1	0	0	0	0	1	7.93	8.07	8.45	8.07	8.54	0	0	0	0	0	0	0	10.53	10.62	10.55	10.66	10.75	11.07	10.86
1372341_at	Slc25a36	1	0	1	0	1	0	8.71	8.64	9.40	8.66	8.68	0	0	0	0	0	0	0	10.66	10.76	10.71	10.67	10.72	10.90	10.98
1372347_at	Skil	1	0	1	1	0	0	7.50	7.71	8.24	8.18	7.75	0	0	0	0	0	0	0	9.38	9.40	9.42	9.49	9.48	9.53	9.45
1372368_at	Hspa13	1	0	0	0	0	1	9.50	9.81	9.94	9.36	10.17	0	0	0	0	0	0	0	11.40	11.45	11.47	11.54	11.62	11.61	11.13
1372381_at	---	1	0	0	0	0	1	7.99	7.97	8.54	7.92	8.51	0	0	0	0	0	0	0	9.90	9.83	9.88	9.91	9.90	9.54	9.22
1372383_at	Gpsm1	1	0	0	1	1	0	9.08	8.67	9.52	8.52	8.82	0	0	0	0	0	0	0	8.42	8.36	8.53	8.55	8.45	8.37	7.94
1372385_at	---	1	0	1	0	1	0	7.34	7.35	6.76	7.33	7.17	0	0	0	0	0	0	0	8.94	8.92	8.96	8.83	8.82	8.62	8.75
1372389_at	Ier2	1	0	0	0	0	1	9.05	9.34	8.82	8.90	8.32	0	0	0	0	0	0	0	7.74	7.68	7.84	7.78	7.83	8.10	7.39
1372390_at	Peg3	1	0	0	0	1	1	10.45	10.56	10.93	10.12	11.36	0	0	0	0	0	0	0	11.25	11.25	11.34	11.44	11.42	11.31	11.14
1372397_at	Tiprl	1	0	0	0	0	1	6.34	6.37	6.87	6.25	7.19	0	0	0	0	0	0	0	7.90	7.92	7.95	7.98	8.06	8.15	7.99
1372404_at	Rac2	1	0	0	0	0	1	6.08	6.43	5.78	6.11	6.68	0	0	0	0	0	0	0	4.84	4.66	4.61	4.61	4.51	4.75	4.80
1372405_at	Ppp1r37	1	0	0	0	1	0	6.79	6.67	6.37	6.97	6.57	0	0	0	0	0	0	0	7.87	7.88	7.91	7.79	7.81	7.74	7.67
1372407_at	Tmod4	1	0	1	0	0	0	5.26	5.21	4.58	4.99	5.07	0	0	0	0	0	0	0	6.58	6.63	6.72	6.69	6.77	6.83	6.52
1372428_at	Dnajc4	1	0	0	0	0	1	6.90	6.91	6.62	6.90	6.35	0	0	0	0	0	0	0	7.29	7.28	7.36	7.20	7.14	6.82	6.82
1372431_at	Mrpl12	1	0	0	0	1	0	8.58	8.60	8.30	8.95	8.19	0	0	0	0	0	0	0	10.53	10.54	10.51	10.55	10.52	10.38	10.20
1372437_at	Skp1	1	0	1	0	0	0	9.95	10.02	10.54	9.84	10.33	0	0	0	0	0	0	0	11.74	11.75	11.70	11.76	11.70	11.85	11.74
1372439_at	Col4a1	1	1	0	0	0	0	8.10	7.60	7.90	7.75	8.05	0	0	0	0	0	0	0	4.08	4.08	3.98	3.96	4.03	4.12	3.62
1372443_at	Lrp11	1	0	1	0	1	1	9.43	9.71	10.38	9.20	10.06	0	0	0	0	0	0	0	12.08	12.07	12.06	11.99	12.00	12.21	12.04
1372446_at	Ppp1r2	1	0	0	0	0	1	9.30	9.18	9.72	9.06	9.77	0	0	0	0	0	0	0	11.09	11.06	11.14	11.19	11.15	11.25	11.17
1372449_at	Slc8a1	1	0	1	0	1	1	8.43	8.61	9.10	8.26	9.11	0	0	0	0	0	0	0	9.55	9.49	9.65	9.83	9.81	9.70	9.60
1372459_at	Vasp	1	0	0	1	0	0	7.55	7.61	8.00	8.26	7.87	0	0	0	0	0	0	0	9.52	9.61	9.67	9.64	9.70	9.48	9.19

Probeset ID	Gene Symbol	Islet Data Set (log2 ratio ≥0.263; FDR <20%) union n=5325 1=YES 0=NO	Islet DRlyp/lyp ND vs ND+Lp299v (log2 ratio ≥0.263; FDR<20%) n=429 1=YES 0=NO	Islet DRlyp/lyp ND vs HCD (log2 ratio ≥0.263; FDR<20%) n=2247 1=YES 0=NO	Islet DRlyp/lyp ND vs HCD+Lp299v (log2 ratio ≥0.263; FDR<20%) n=1595 1=YES 0=NO	Islet DRlyp/lyp HCD vs HCD+Lp299v (log2 ratio ≥0.263; FDR<20%) n=3026 1=YES 0=NO	Islet DRlyp/lyp ND vs Flyp/lyp ND (log2 ratio ≥ 0.263; FDR<20%) n=1950 1=YES 0=NO	Islet Mean Log2 Intensity DRlyp/lyp ND	Islet Mean Log2 Intensity DRlyp/lyp ND+Lp299v	Islet Mean Log2 Intensity DRlyp/lyp HCD	Islet Mean Log2 Intensity DRlyp/lyp HCD+Lp299v	Islet Mean Log2 Intensity Flyp40 ND	Rinm5f data union of all conditions n=3705 1=YES 0=NO	Rinm5f 30 µM propionate/ 6 µM butyrate vs Media (FDR<20%; 1.5 fold) n=3 1=YES 0=NO	Rinm5f 60 µM propionate/ 12 µM butyrate vs Media (FDR<20%; 1.5 fold) n=18 1=YES 0=NO	Rinm5f 120 µM propionate/ 24 µM butyrate vs Media (FDR<20%; 1.5 fold) n=164 1=YES 0=NO	Rinm5f 240 µM propionate /48 µM butyrate vs Media (FDR<20%; 1.5 fold) n=268 1=YES 0=NO	Rinm5f 1500 µM propionate/ 300 µM butyrate vs Media (FDR<20%; 1.5 fold) n=2491 1=YES 0=NO	Rinm5f 500 µM propionate/ 500 µM butyrate vs Media (FDR<20%; 1.5 fold) n=3001 1=YES 0=NO	Rinm5f Mean Log2 Intensity Media only	Rinm5f Mean Log2 Intensity 30 µM propionate/ 6 µM butyrate	Rinm5f Mean Log2 Intensity 60 µM propionate/ 12 µM butyrate	Rinm5f Mean Log2 Intensity 120 µM propionate/ 24 µM butyrate	Rinm5f Mean Log2 Intensity 240 µM propionate/ 48 µM butyrate	Rinm5f Mean Log2 Intensity 1500 µM propionate /300 µM butyrate	Rinm5f Mean Log2 Intensity 500 µM propionate/ 500 µM butyrate
1372462_at	Acat2	1	0	0	0	0	1	7.64	7.53	7.77	7.58	6.92	0	0	0	0	0	0	0	8.95	8.93	9.06	9.18	9.13	8.80	8.29
1372463_at	Fcho2	1	0	1	0	0	0	7.30	7.22	7.97	7.43	7.43	0	0	0	0	0	0	0	9.70	9.78	9.74	9.83	9.87	10.19	10.08
1372469_at	Ephb4	1	0	0	1	1	0	5.40	5.70	5.63	6.27	5.39	0	0	0	0	0	0	0	4.37	4.16	4.24	4.10	4.17	4.23	4.33
1372475_at	Pink1	1	0	0	0	0	1	8.55	8.35	8.09	8.63	7.82	0	0	0	0	0	0	0	8.33	8.38	8.39	8.31	8.24	7.97	7.67
1372481_at	---	1	1	0	1	0	1	6.88	6.36	6.47	6.35	6.32	0	0	0	0	0	0	0	4.21	4.32	4.21	4.17	4.19	4.25	3.70
1372489_at	Slmap	1	0	1	0	0	0	7.60	7.49	8.18	7.56	7.71	0	0	0	0	0	0	0	9.64	9.72	9.80	9.81	9.84	9.69	9.35
1372492_at	Cldn10	1	0	0	0	1	1	9.29	8.99	8.85	9.66	8.35	0	0	0	0	0	0	0	3.87	3.74	3.85	3.75	3.86	4.02	3.65
1372494_a_at	Hmg20b	1	0	0	0	1	0	5.78	5.82	5.58	6.20	5.72	0	0	0	0	0	0	0	7.93	7.98	8.08	8.03	8.05	8.36	8.40
1372504_at	Dpm3 /// LOC10	1	0	0	0	1	0	9.06	9.16	8.85	9.47	8.88	0	0	0	0	0	0	0	11.11	11.13	11.01	10.95	10.96	10.96	10.80
1372510_at	Srxn1	1	0	0	0	0	1	6.44	6.73	6.93	6.52	7.29	0	0	0	0	0	0	0	9.05	8.99	9.09	9.20	9.11	9.07	9.45
1372527_at	Rtn2	1	0	0	0	0	1	5.51	5.71	5.82	5.68	5.98	0	0	0	0	0	0	0	9.57	9.60	9.70	9.64	9.66	9.54	9.26
1372533_at	Edem1	1	1	0	1	0	0	9.32	8.52	9.46	8.78	8.99	0	0	0	0	0	0	0	10.62	10.65	10.69	10.68	10.74	10.98	10.53
1372536_at	Adck3	1	0	1	1	1	1	7.65	7.57	7.09	8.34	7.10	0	0	0	0	0	0	0	4.72	4.87	4.75	4.77	4.85	5.00	5.29
1372544_at	---	1	0	0	0	1	0	8.61	8.61	9.08	8.26	8.92	0	0	0	0	0	0	0	9.67	9.74	9.70	9.69	9.76	9.62	9.41
1372551_at	Fastk	1	0	0	0	1	0	7.65	7.67	7.39	8.02	7.44	0	0	0	0	0	0	0	8.42	8.45	8.39	8.35	8.28	8.01	7.31
1372555_at	Flywch1	1	0	0	0	0	1	7.19	7.55	7.40	7.42	7.72	0	0	0	0	0	0	0	9.54	9.56	9.64	9.59	9.65	9.72	9.50
1372556_at	Polr2l	1	0	1	0	0	0	10.13	10.09	9.60	10.12	10.00	0	0	0	0	0	0	0	11.34	11.33	11.33	11.36	11.36	11.51	11.65
1372557_at	Arl6	1	0	1	0	1	1	6.30	6.20	7.14	6.13	6.78	0	0	0	0	0	0	0	8.81	8.84	8.92	9.05	9.12	9.34	9.42
1372558_at	Naa15	1	0	1	0	1	0	7.07	6.99	7.68	6.92	7.21	0	0	0	0	0	0	0	9.52	9.47	9.60	9.80	9.67	9.59	9.41
1372582_at	Csnk1g3	1	0	1	0	0	0	6.64	6.65	7.37	6.70	7.06	0	0	0	0	0	0	0	10.07	10.11	10.17	10.21	10.22	10.31	10.01
1372583_at	Tmtc1	1	0	1	0	1	0	6.97	6.93	7.59	6.88	7.04	0	0	0	0	0	0	0	8.31	8.28	8.28	8.10	8.07	8.11	8.15
1372585_at	LOC100910973	1	1	0	1	0	0	7.60	8.13	7.16	7.06	7.89	0	0	0	0	0	0	0	4.70	4.74	4.75	4.53	4.67	4.98	4.39
1372586_at	---	1	0	1	0	1	0	8.43	8.33	9.02	8.14	8.65	0	0	0	0	0	0	0	10.18	10.25	10.29	10.36	10.36	10.49	10.07
1372587_at	Emcn	1	0	0	1	1	0	7.85	7.54	8.04	7.23	7.70	0	0	0	0	0	0	0	3.82	3.86	3.89	3.85	3.91	3.82	3.22
1372588_at	Bles03	1	0	0	1	0	0	7.23	7.26	7.23	7.70	7.22	0	0	0	0	0	0	0	7.97	7.97	7.94	7.79	7.71	7.47	7.39
1372597_at	Mrpl14	1	0	0	0	1	0	6.91	6.86	6.65	7.23	6.58	0	0	0	0	0	0	0	9.52	9.56	9.51	9.52	9.45	9.78	9.91
1372598_at	C16orf74	1	0	1	0	0	0	5.31	5.23	4.79	5.07	5.07	0	0	0	0	0	0	0	5.36	5.52	5.46	5.58	5.58	5.68	6.05
1372607_at	Nubp2	1	0	0	0	1	0	8.59	8.54	8.26	8.83	8.42	0	0	0	0	0	0	0	8.32	8.33	8.32	8.37	8.27	8.14	8.07
1372615_at	Aoc3	1	1	0	1	0	1	7.28	6.64	6.86	6.53	5.72	0	0	0	0	0	0	0	3.99	4.01	3.98	3.87	3.94	4.02	3.49
1372618_at	Rabl6	1	0	0	0	1	0	7.43	7.50	7.05	7.76	7.38	0	0	0	0	0	0	0	8.76	8.66	8.74	8.62	8.59	8.34	8.54
1372619_at	Mrpl49	1	0	1	0	1	1	5.18	5.24	5.88	4.97	5.69	0	0	0	0	0	0	0	9.42	9.49	9.52	9.62	9.59	9.66	9.39
1372620_at	Anp32e	1	0	1	0	1	0	6.43	6.51	7.07	6.21	6.57	0	0	0	0	0	0	0	9.65	9.70	9.69	9.71	9.61	9.32	8.96
1372623_at	---	1	0	0	0	1	0	6.90	6.93	6.60	7.17	6.50	0	0	0	0	0	0	0	7.79	7.80	7.79	7.80	7.79	7.58	7.15
1372629_at	Coro2b	1	0	0	0	0	1	6.80	6.88	6.79	6.81	7.30	0	0	0	0	0	0	0	8.17	8.08	8.16	8.11	8.14	8.14	8.24
1372630_at	Rad23a	1	0	0	1	1	0	7.05	7.24	6.71	7.53	6.80	0	0	0	0	0	0	0	7.43	7.44	7.49	7.37	7.38	7.27	7.52
1372634_at	Adprhl2	1	0	0	1	0	0	6.63	6.82	6.71	7.12	6.65	0	0	0	0	0	0	0	9.45	9.44	9.54	9.52	9.34	9.38	9.41
1372635_at	G3bp1	1	0	0	0	0	1	7.13	7.30	7.33	7.29	7.66	0	0	0	0	0	0	0	7.02	6.99	7.10	7.06	7.20	7.41	7.71
1372642_at	---	1	0	0	1	0	0	8.25	7.89	8.37	8.82	7.99	0	0	0	0	0	0	0	10.43	10.44	10.53	10.80	10.73	10.66	10.32
1372650_at	Dnmbp	1	0	0	1	1	1	6.65	6.61	6.55	7.16	6.12	0	0	0	0	0	0	0	7.10	7.04	7.18	7.22	7.11	7.13	6.96
1372668_at	---	1	0	1	0	1	0	7.13	7.13	7.75	6.87	7.54	0	0	0	0	0	0	0	7.23	7.21	7.11	7.11	7.22	7.61	7.51
1372671_at	Rfk	1	0	0	0	0	1	7.58	7.46	7.79	7.48	8.09	0	0	0	0	0	0	0	10.47	10.43	10.46	10.51	10.52	10.66	10.32
1372674_at	Rybp	1	0	1	0	1	0	8.92	8.87	9.58	8.80	8.94	0	0	0	0	0	0	0	11.90	11.89	11.91	11.92	11.97	12.26	11.85
1372696_at	Zkscan1	1	0	1	0	1	0	8.01	8.13	8.65	7.69	8.32	0	0	0	0	0	0	0	9.82	9.87	9.93	9.96	9.95	10.10	10.27
1372701_at	Hsp90aa1	1	0	0	0	0	1	10.61	10.69	11.10	10.44	11.14	0	0	0	0	0	0	0	12.94	12.97	13.03	13.06	13.05	12.96	12.74
1372706_at	Hexb	1	0	1	0	1	0	8.36	8.47	8.98	8.24	8.53	0	0	0	0	0	0	0	11.32	11.35	11.35	11.37	11.48	11.71	11.50
1372707_at	Rab6a	1	0	1	0	1	1	9.04	9.14	9.63	8.84	9.64	0	0	0	0	0	0	0	11.38	11.40	11.38	11.39	11.37	11.29	11.09
1372708_at	Thsd4	1	0	0	1	1	0	5.09	5.17	5.11	6.18	5.01	0	0	0	0	0	0	0	6.65	6.69	6.65	6.61	6.59	6.75	6.91
1372710_at	Bet1	1	0	0	0	0	1	8.78	8.81	9.06	8.74	9.34	0	0	0	0	0	0	0	9.78	9.89	9.90	9.94	9.95	9.95	9.70
1372719_at	Susd6	1	0	1	0	1	0	8.13	8.01	8.71	7.88	8.33	0	0	0	0	0	0	0	11.01	11.04	11.06	10.94	11.03	11.05	10.35
1372720_at	Btbd1	1	0	1	0	1	0	8.38	8.33	9.23	8.31	8.73	0	0	0	0	0	0	0	10.87	10.95	10.92	10.90	10.92	10.89	10.48
1372721_at	Tmem167a	1	0	0	0	0	1	9.04	9.12	9.30	9.16	9.52	0	0	0	0	0	0	0	10.87	10.87	10.83	10.85	10.84	10.80	10.46
1372722_at	Dnajb4	1	0	0	0	1	1	7.71	8.10	8.25	7.36	8.18	0	0	0	0	0	0	0	9.54	9.51	9.55	9.60	9.53	9.40	9.14
1372723_at	Ipo9	1	0	0	0	0	1	7.34	7.60	7.72	7.49	7.81	0	0	0	0	0	0	0	10.05	10.03	10.11	10.14	10.14	10.17	10.19
1372724_at	Grina	1	0	0	0	1	0	8.60	8.61	8.21	8.92	8.47	0	0	0	0	0	0	0	10.91	10.89	10.96	10.90	10.87	10.91	10.65
1372725_at	Plscr2	1	0	0	0	0	1	4.55	4.54	4.75	5.02	5.10	0	0	0	0	0	0	0	3.17	3.13	3.25	3.22	3.17	3.25	2.71
1372726_at	H4c11	1	0	0	1	1	0	7.74	7.61	8.01	8.72	7.76	0	0	0	0	0	0	0	3.71	3.55	3.65	3.43	3.65	3.66	3.11
1372727_at	---	1	0	0	1	0	0	8.74	8.98	8.84	9.32	8.49	0	0	0	0	0	0	0	9.40	9.34	9.44	9.48	9.45	9.41	9.39
1372735_at	Eif3k	1	0	0	1	0	0	9.42	9.28	9.45	9.90	9.04	0	0	0	0	0	0	0	10.80	10.80	10.77	10.76	10.75	10.87	10.47
1372736_at	Agl	1	0	1	0	0	0	6.48	6.32	7.07	6.47	6.54	0	0	0	0	0	0	0	7.56	7.54	7.66	7.73	7.69	7.88	7.92
1372747_at	Slc20a2	1	0	0	1	0	0	5.39	5.24	5.48	6.02	5.49	0	0	0	0	0	0	0	7.77	7.56	7.66	7.70	7.52	7.20	7.37
1372752_at	Tspan4	1	0	1	0	1	1	7.02	6.83	6.35	7.03	6.47	0	0	0	0	0	0	0	10.04	10.22	10.23	10.27	10.32	10.29	9.71
1372754_at	Appl2	1	0	0	0	0	1	9.46	9.26	9.40	9.57	8.07	0	0	0	0	0	0	0	9.43	9.43	9.53	9.56	9.54	9.77	9.90
1372757_at	Stat1	1	0	0	0	1	0	7.79	8.09	8.28	7.58	8.12	0	0	0	0	0	0	0	9.16	9.19	9.26	9.31	9.24	9.38	9.06
1372762_at	Hddc3	1	0	0	0	0	1	8.09	7.93	7.78	8.23	7.36	0	0	0	0	0	0	0	9.24	9.37	9.40	9.43	9.53	9.72	9.35
1372769_at	Eif2d	1	0	0	0	1	0	7.83	7.68	7.36	8.07	7.54	0	0	0	0	0	0	0	10.46	10.47	10.47	10.33	10.29	10.18	9.88
1372770_at	Sri	1	0	0	0	1	0	7.28	7.23	7.74	6.96	7.14	0	0	0	0	0	0	0	9.19	9.22	9.23	9.34	9.26	9.12	8.50
1372778_at	Slc39a1	1	0	0	1	1	0	6.54	6.49	6.37	7.18	6.51	0	0	0	0	0	0	0	8.19	8.18	8.14	8.12	8.24	8.35	8.02
1372781_at	Wdr47	1	0	1	0	1	0	6.16	6.01	6.82	5.72	6.44	0	0	0	0	0	0	0	8.91	8.96	9.01	9.09	9.12	9.14	8.90
1372782_a_at	Ampd2	1	0	0	0	0	1	8.80	8.99	9.05	8.74	9.35	0	0	0	0	0	0	0	9.47	9.43	9.59	9.61	9.64	9.87	9.96
1372794_at	Dapk2	1	0	0	0	0	1	6.26	6.08	6.37	6.66	5.74	0	0	0	0	0	0	0	4.99	4.97	5.00	4.83	5.05	5.18	5.69
1372801_at	Commd10	1	0	1	0	0	1	7.60	7.62	8.31	7.66	8.25	0	0	0	0	0	0	0	10.37	10.30	10.27	10.31	10.26	10.20	9.81
1372804_at	Noa1	1	0	0	1	0	0	4.42	4.41	4.88	5.01	4.59	0	0	0	0	0	0	0	7.48	7.45	7.43	7.45	7.37	7.31	7.35
1372806_at	Vps35	1	0	1	0	1	0	9.15	9.12	9.78	8.83	9.55	0	0	0	0	0	0	0	10.09	10.11	10.15	10.25	10.21	10.17	10.12
1372811_at	Ktn1	1	0	1	0	0	0	7.63	7.67	8.30	7.66	7.77	0	0	0	0	0	0	0	8.82	8.93	8.95	9.10	9.04	8.86	8.81
1372816_at	---	1	0	1	0	1	1	7.25	7.45	7.92	7.17	7.86	0	0	0	0	0	0	0	8.48	8.47	8.56	8.64	8.72	8.83	9.04
1372817_at	Znf260	1	0	1	0	0	0	8.71	8.82	9.39	8.77	9.04	0	0	0	0	0	0	0	10.06	10.10	10.10	10.08	10.08	10.04	9.86
1372818_at	Colec12	1	1	1	1	1	0	5.09	4.39	5.68	3.95	4.77	0	0	0	0	0	0	0	3.56	3.56	3.48	3.49	3.58	3.66	3.51
1372824_at	Plekhf2	1	0	0	1	0	0	6.94	7.04	7.12	7.45	6.99	0	0	0	0	0	0	0	8.50	8.55	8.57	8.57	8.66	8.91	8.97
1372843_at	Smco4	1	0	1	0	0	0	8.70	8.59	8.18	8.65	8.57	0	0	0	0	0	0	0	9.91	9.92	9.87	9.84	9.80	9.84	9.59
1372852_at	Ptprcap	1	0	0	0	0	1	4.33	4.37	4.09	4.28	4.83	0	0	0	0	0	0	0	6.13	6.16	6.08	5.93	5.96	6.02	6.03
1372859_at	Gmppa	1	0	0	0	1	0	8.18	8.47	7.89	8.51	8.14	0	0	0	0	0	0	0	8.40	8.42	8.37	8.21	8.24	8.15	7.77
1372866_at	Slc25a38	1	0	1	0	0	0	7.37	7.13	6.76	7.27	6.92	0	0	0	0	0	0	0	5.92	5.86	5.91	5.63	5.68	5.75	5.57
1372875_at	---	1	0	0	0	0	1	7.76	7.71	8.29	7.61	8.39	0	0	0	0	0	0	0	10.51	10.53	10.56	10.54	10.57	10.46	10.51
1372878_at	Zfr	1	0	1	0	0	0	9.71	9.91	10.28	9.79	10.10	0	0	0	0	0	0	0	11.43	11.43	11.46	11.53	11.52	11.45	11.08
1372887_at	Scarf2	1	0	1	0	1	1	5.70	5.42	4.90	6.06	5.19	0	0	0	0	0	0	0	7.34	7.32	7.39	7.32	7.31	6.96	6.50
1372900_at	Pigt	1	0	0	0	1	0	8.18	8.03	7.79	8.40	7.92	0	0	0	0	0	0	0	10.53	10.52	10.51	10.37	10.38	10.34	9.79
1372920_at	LOC102724788 /	1	0	1	0	0	0	6.58	6.38	5.94	6.22	6.24	0	0	0	0	0	0	0	6.21	6.23	6.36	6.16	6.26	6.78	6.78
1372923_at	---	1	0	0	0	0	1	4.45	4.32	4.34	4.29	6.10	0	0	0	0	0	0	0	8.86	8.88	8.88	8.94	8.88	8.77	8.44
1372926_at	Timp3	1	0	1	0	1	0	5.27	5.39	6.05	4.96	5.17	0	0	0	0	0	0	0	3.24	3.20	3.20	3.14	3.21	3.13	2.86
1372927_at	Mrpl50	1	0	0	0	0	1	5.73	5.74	5.96	6.10	6.93	0	0	0	0	0	0	0	7.49	7.62	7.64	7.64	7.58	7.64	7.55
1372934_at	Ift43	1	0	1	0	0	0	5.08	5.09	5.66	5.09	5.32	0	0	0	0	0	0	0	9.10	9.10	9.09	8.91	8.99	8.91	8.84
1372938_at	Smarce1	1	0	1	0	1	0	8.35	8.25	8.97	8.21	8.58	0	0	0	0	0	0	0	11.17	11.21	11.25	11.32	11.33	11.45	11.29
1372954_at	---	1	0	1	0	1	1	4.70	4.86	5.53	4.79	5.37	0	0	0	0	0	0	0	8.43	8.42	8.51	8.64	8.58	8.32	8.05
1372973_at	Lss	1	0	0	0	0	1	6.32	6.35	6.53	6.23	6.81	0	0	0	0	0	0	0	8.89	8.87	8.94	8.89	8.89	8.50	8.08
1372976_at	Abhd14a	1	0	1	0	1	1	7.71	7.37	6.77	7.56	7.23	0	0	0	0	0	0	0	6.04	6.08	6.14	5.94	6.10	6.38	5.78
1372994_at	Fance	1	0	0	0	1	0	6.89	6.86	6.70	7.27	6.76	0	0	0	0	0	0	0	7.65	7.75	7.85	7.73	7.72	7.57	7.29
1372996_at	Isoc2	1	0	1	0	1	1	9.10	8.86	8.27	9.32	8.29	0	0	0	0	0	0	0	7.91	7.99	7.91	7.66	7.72	7.92	8.06
1372997_at	Gucd1	1	1	0	0	0	0	7.58	6.92	7.37	7.87	7.22	0	0	0	0	0	0	0	8.04	8.08	8.09	8.10	8.03	7.74	7.96
1372998_at	---	1	0	1	0	1	1	8.38	8.76	9.07	8.34	9.24	0	0	0	0	0	0	0	10.89	10.86	10.88	10.80	10.86	10.89	10.61
1373011_at	Retreg1	1	1	0	0	0	1	9.96	9.42	9.61	9.68	9.35	0	0	0	0	0	0	0	9.89	9.89	9.89	9.97	9.85	9.71	9.72
1373012_at	Retreg1	1	0	0	0	0	1	7.05	6.73	6.71	6.60	6.55	0	0	0	0	0	0	0	7.11	7.05	7.06	7.08	7.03	6.87	6.65
1373015_at	Rnf11 /// Rnf11l	1	0	1	0	1	0	8.11	8.12	8.73	7.90	8.48	0	0	0	0	0	0	0	10.51	10.59	10.58	10.61	10.60	10.67	10.49
1373038_at	Mllt10	1	0	0	0	1	0	6.23	6.14	6.78	5.84	6.30	0	0	0	0	0	0	0	8.75	8.72	8.78	8.72	8.74	8.38	8.12
1373040_at	Eif3f	1	0	0	1	1	0	9.93	9.98	10.00	10.65	9.51	0	0	0	0	0	0	0	11.29	11.27	11.28	11.19	11.14	11.10	11.29
1373056_at	Tmed4	1	0	1	0	1	0	8.44	8.51	9.08	8.24	8.90	0	0	0	0	0	0	0	11.70	11.68	11.73	11.69	11.75	11.78	11.53
1373057_at	Lzts3	1	0	1	0	0	0	6.40	6.20	5.78	6.26	6.09	0	0	0	0	0	0	0	6.59	6.67	6.66	6.71	6.65	6.56	6.37
1373068_at	Ifrd2	1	0	0	0	1	0	9.36	9.27	9.04	9.66	9.09	0	0	0	0	0	0	0	8.59	8.59	8.56	8.56	8.58	8.49	8.14
1373075_at	Cdk8	1	0	0	0	1	0	7.40	7.36	7.90	7.13	7.49	0	0	0	0	0	0	0	10.14	10.15	10.21	10.27	10.26	10.13	9.84
1373078_at	Ahcyl2	1	0	0	0	0	1	6.99	7.13	7.22	6.90	7.52	0	0	0	0	0	0	0	8.07	7.97	7.99	7.93	7.85	7.81	8.27
1373080_at	Papola	1	0	0	0	1	0	8.11	8.08	8.55	7.75	8.19	0	0	0	0	0	0	0	10.51	10.45	10.47	10.47	10.43	10.20	9.72
1373082_at	Pkia	1	0	1	0	1	1	5.21	5.15	5.83	4.87	5.80	0	0	0	0	0	0	0	8.58	8.62	8.72	8.75	8.84	9.02	9.23
1373100_at	Swi5	1	0	1	0	0	0	7.39	7.65	8.00	7.67	7.56	0	0	0	0	0	0	0	12.11	12.08	12.08	11.97	11.98	11.78	11.69
1373102_at	Cdh13	1	0	0	0	0	1	4.95	4.75	4.50	4.73	4.38	0	0	0	0	0	0	0	4.21	4.20	4.13	4.15	4.04	4.21	4.24
1373118_at	Camsap3	1	0	0	0	1	0	5.97	6.13	5.67	6.29	5.87	0	0	0	0	0	0	0	7.51	7.43	7.57	7.48	7.52	7.20	7.32
1373134_at	Fahd2b	1	0	0	0	1	0	6.31	6.46	6.14	6.74	6.17	0	0	0	0	0	0	0	7.21	7.26	7.28	7.11	7.15	6.89	6.72
1373146_at	Ssx2ip	1	0	1	0	0	0	6.52	6.60	7.11	6.77	6.97	0	0	0	0	0	0	0	9.48	9.47	9.47	9.55	9.62	9.57	9.47
1373150_at	Comtd1	1	0	1	0	1	1	7.67	7.61	7.05	7.85	7.07	0	0	0	0	0	0	0	8.28	8.28	8.24	8.29	8.09	7.82	7.55
1373156_at	Armcx2	1	0	1	0	1	1	7.52	7.72	8.18	7.43	8.34	0	0	0	0	0	0	0	10.90	10.92	11.01	11.11	11.13	10.88	10.72
1373157_at	Usp47	1	0	0	1	1	0	6.71	6.71	6.99	6.15	6.61	0	0	0	0	0	0	0	9.12	9.13	9.19	9.27	9.20	9.21	8.83
1373163_at	Usp28	1	0	1	0	1	0	5.17	5.19	5.90	5.19	5.25	0	0	0	0	0	0	0	6.98	7.01	7.15	6.96	7.06	7.04	7.31
1373164_at	Stk17b	1	0	0	0	0	1	6.16	5.86	6.11	6.05	6.74	0	0	0	0	0	0	0	8.00	8.10	8.12	8.13	8.09	7.78	7.75
1373165_at	Zmat1	1	0	1	0	1	0	5.79	5.78	6.63	5.52	6.16	0	0	0	0	0	0	0	7.44	7.45	7.49	7.55	7.47	7.36	7.04
1373171_at	Cep85	1	1	0	0	0	0	6.04	5.58	6.20	5.98	5.92	0	0	0	0	0	0	0	9.03	9.10	9.15	9.12	9.15	9.22	9.10
1373180_at	---	1	0	0	0	1	0	7.70	7.72	8.22	7.32	8.09	0	0	0	0	0	0	0	9.07	9.05	9.11	9.09	9.08	9.10	9.36
1373185_at	Ssr2	1	0	0	1	1	0	10.34	10.27	10.23	10.85	10.25	0	0	0	0	0	0	0	12.11	12.16	12.08	12.06	12.10	11.82	11.30
1373196_at	Micu3	1	0	1	0	1	0	7.35	7.44	8.05	7.17	7.70	0	0	0	0	0	0	0	10.30	10.33	10.34	10.38	10.39	10.39	10.31
1373201_at	Dbt	1	1	1	0	0	0	9.31	8.84	8.71	9.24	9.02	0	0	0	0	0	0	0	7.19	7.20	7.23	7.33	7.37	7.57	7.35
1373215_at	Abr	1	0	0	0	0	1	6.35	6.44	6.85	6.49	6.94	0	0	0	0	0	0	0	8.43	8.31	8.46	8.39	8.29	7.89	7.95
1373221_at	Diaph1	1	0	0	1	1	0	7.07	7.25	6.88	7.70	7.42	0	0	0	0	0	0	0	7.42	7.34	7.36	7.25	7.28	7.29	7.76
1373222_at	Hexa	1	0	0	1	0	0	9.83	10.05	9.88	10.36	9.81	0	0	0	0	0	0	0	10.85	10.90	10.86	10.76	10.83	10.94	10.65
1373226_at	Tor4a	1	0	0	0	0	1	4.06	4.17	4.41	4.20	4.76	0	0	0	0	0	0	0	7.11	7.19	7.25	7.40	7.36	7.57	7.34
1373228_at	Hgsnat	1	0	0	1	1	0	8.14	8.19	8.04	8.73	8.07	0	0	0	0	0	0	0	10.39	10.44	10.42	10.35	10.40	10.44	10.16
1373234_at	Extl3 /// LOC102	1	0	1	0	1	0	8.80	8.68	8.10	9.12	8.37	0	0	0	0	0	0	0	7.88	7.92	7.88	7.82	7.77	7.78	8.03
1373236_at	Ppcdc	1	0	1	1	0	1	7.24	6.90	6.65	6.66	6.49	0	0	0	0	0	0	0	8.15	8.27	8.27	8.22	8.24	7.80	7.41
1373237_at	Rufy3	1	0	0	0	0	1	5.21	5.31	5.74	5.35	5.73	0	0	0	0	0	0	0	8.02	8.12	8.34	8.38	8.50	8.59	8.50

Probeset ID	Gene Symbol	Islet Data Set (log2 ratio ≥0.263; FDR <20%) union n=5325 1=YES 0=NO	Islet DRlyp/lyp ND vs ND+Lp299v (log2 ratio ≥0.263; FDR<20%) n=429 1=YES 0=NO	Islet DRlyp/lyp ND vs HCD (log2 ratio ≥0.263; FDR<20%) n=2247 1=YES 0=NO	Islet DRlyp/lyp ND vs HCD+Lp299v (log2 ratio ≥0.263; FDR<20%) n=1595 1=YES 0=NO	Islet DRlyp/lyp HCD vs HCD+Lp299v (log2 ratio ≥0.263; FDR<20%) n=3026 1=YES 0=NO	Islet DRlyp/lyp ND vs Flyp/lyp ND (log2 ratio ≥ 0.263; FDR<20%) n=1950 1=YES 0=NO	Islet Mean Log2 Intensity DRlyp/lyp ND	Islet Mean Log2 Intensity DRlyp/lyp ND+Lp299v	Islet Mean Log2 Intensity DRlyp/lyp HCD	Islet Mean Log2 Intensity DRlyp/lyp HCD+Lp299v	Islet Mean Log2 Intensity Flyp40 ND	Rinm5f data union of all conditions n=3705 1=YES 0=NO	Rinm5f 30 µM propionate/ 6 µM butyrate vs Media (FDR<20%; 1.5 fold) n=3 1=YES 0=NO	Rinm5f 60 µM propionate/ 12 µM butyrate vs Media (FDR<20%; 1.5 fold) n=18 1=YES 0=NO	Rinm5f 120 µM propionate/ 24 µM butyrate vs Media (FDR<20%; 1.5 fold) n=164 1=YES 0=NO	Rinm5f 240 µM propionate /48 µM butyrate vs Media (FDR<20%; 1.5 fold) n=268 1=YES 0=NO	Rinm5f 1500 µM propionate/ 300 µM butyrate vs Media (FDR<20%; 1.5 fold) n=2491 1=YES 0=NO	Rinm5f 500 µM propionate/ 500 µM butyrate vs Media (FDR<20%; 1.5 fold) n=3001 1=YES 0=NO	Rinm5f Mean Log2 Intensity Media only	Rinm5f Mean Log2 Intensity 30 µM propionate/ 6 µM butyrate	Rinm5f Mean Log2 Intensity 60 µM propionate/ 12 µM butyrate	Rinm5f Mean Log2 Intensity 120 µM propionate/ 24 µM butyrate	Rinm5f Mean Log2 Intensity 240 µM propionate/ 48 µM butyrate	Rinm5f Mean Log2 Intensity 1500 µM propionate /300 µM butyrate	Rinm5f Mean Log2 Intensity 500 µM propionate/ 500 µM butyrate
1373239_at	Snx33	1	0	0	0	1	0	6.79	6.72	6.33	7.25	6.55	0	0	0	0	0	0	0	5.78	5.87	5.69	5.56	5.64	5.68	5.59
1373248_at	Vsig10	1	0	0	0	1	0	7.55	7.55	8.12	7.34	7.76	0	0	0	0	0	0	0	9.01	9.04	9.15	9.20	9.25	9.35	9.05
1373258_at	Ctsf	1	0	0	0	1	0	7.51	7.44	8.06	7.16	7.57	0	0	0	0	0	0	0	11.26	11.24	11.27	11.26	11.33	11.44	10.71
1373264_at	Zbed3	1	0	0	0	1	0	9.00	8.79	8.70	9.33	8.63	0	0	0	0	0	0	0	7.70	7.84	7.82	7.85	7.80	7.53	7.24
1373282_at	Slc25a33	1	1	0	0	0	1	5.98	5.47	5.77	6.27	5.32	0	0	0	0	0	0	0	7.23	7.25	7.36	7.49	7.42	7.36	7.22
1373284_at	Sav1	1	1	0	0	0	1	6.70	6.23	6.53	6.62	6.24	0	0	0	0	0	0	0	8.69	8.58	8.67	8.62	8.51	8.47	8.42
1373296_a_at	Anapc15	1	0	0	1	1	0	6.47	6.64	6.26	7.01	6.17	0	0	0	0	0	0	0	9.02	8.99	9.03	9.00	8.87	8.62	8.64
1373300_at	Fam234b	1	0	0	0	1	0	6.07	6.04	5.83	6.40	6.00	0	0	0	0	0	0	0	8.60	8.56	8.66	8.53	8.60	8.63	8.14
1373301_at	Ppp1r13b	1	0	0	1	0	0	6.78	6.84	6.83	7.28	6.68	0	0	0	0	0	0	0	7.97	8.00	8.04	8.07	8.07	8.09	8.31
1373313_at	Sel1l	1	0	0	1	0	0	10.94	10.73	11.09	11.44	10.86	0	0	0	0	0	0	0	10.21	10.27	10.29	10.35	10.42	10.52	10.32
1373315_at	Arnt2	1	0	0	0	0	1	5.98	5.97	6.44	6.02	6.55	0	0	0	0	0	0	0	9.02	8.94	9.05	9.07	9.03	9.30	9.53
1373316_at	LOC100911887	1	0	0	1	0	0	3.36	3.35	3.54	3.85	3.37	0	0	0	0	0	0	0	3.57	3.46	3.68	3.72	3.65	3.64	3.13
1373337_at	Grhpr	1	0	0	1	1	0	7.86	7.78	7.39	8.39	7.42	0	0	0	0	0	0	0	8.29	8.24	8.23	8.20	8.15	7.86	7.82
1373347_at	Acbd3	1	0	0	1	0	0	8.15	8.31	8.17	8.62	8.37	0	0	0	0	0	0	0	8.80	8.57	8.61	8.55	8.44	8.45	8.61
1373350_at	Psip1	1	0	1	0	0	1	5.88	5.87	6.61	6.05	6.47	0	0	0	0	0	0	0	8.53	8.68	8.67	8.80	8.86	8.75	7.74
1373363_at	Map1b	1	0	0	0	1	0	9.90	9.96	10.30	9.51	10.18	0	0	0	0	0	0	0	11.54	11.56	11.62	11.69	11.64	11.64	11.87
1373366_at	Ganab	1	0	0	0	0	1	7.76	7.86	8.02	8.09	8.34	0	0	0	0	0	0	0	10.93	10.78	10.93	10.90	10.93	10.70	10.13
1373372_at	Mydgf	1	0	0	0	1	0	8.87	8.74	8.52	9.21	8.49	0	0	0	0	0	0	0	10.71	10.74	10.70	10.65	10.63	10.58	10.39
1373373_at	Gfpt1	1	0	1	0	0	1	9.34	9.56	10.00	9.54	10.04	0	0	0	0	0	0	0	10.73	10.71	10.75	10.69	10.67	10.48	10.37
1373374_at	Lmo4	1	0	1	0	1	1	10.16	10.02	9.52	10.23	9.65	0	0	0	0	0	0	0	10.93	10.84	10.88	10.85	10.83	11.07	10.93
1373386_at	Gjb2	1	0	1	0	1	0	8.70	8.68	8.11	9.07	8.62	0	0	0	0	0	0	0	4.76	4.70	4.51	4.55	4.53	4.63	3.80
1373389_at	Acad9	1	0	0	0	0	1	6.80	6.90	7.04	6.90	7.44	0	0	0	0	0	0	0	9.53	9.46	9.56	9.61	9.51	9.31	8.86
1373391_at	Tmco1	1	0	1	0	0	0	8.50	8.50	9.10	8.57	8.88	0	0	0	0	0	0	0	10.51	10.58	10.61	10.68	10.69	10.74	10.45
1373392_at	Tmem165	1	0	0	0	1	0	7.54	7.46	8.02	7.31	7.92	0	0	0	0	0	0	0	9.79	9.85	9.82	9.80	9.80	9.59	9.21
1373393_at	Ext1	1	0	0	0	1	0	6.60	6.50	6.23	6.86	6.58	0	0	0	0	0	0	0	8.65	8.56	8.65	8.68	8.67	9.04	9.04
1373397_at	Mapre1	1	0	1	1	1	0	7.78	7.71	8.40	7.21	8.12	0	0	0	0	0	0	0	10.90	10.91	10.97	11.00	10.95	11.00	11.03
1373398_at	Trim37	1	0	0	0	1	0	6.83	6.79	7.32	6.62	7.22	0	0	0	0	0	0	0	8.59	8.54	8.57	8.62	8.58	8.84	9.20
1373403_at	Tcim	1	1	0	1	0	1	9.10	8.19	8.59	8.41	8.61	0	0	0	0	0	0	0	5.01	4.88	4.98	4.74	4.86	5.16	5.83
1373407_at	Gramd4	1	0	1	0	1	0	7.18	6.86	6.48	7.08	6.76	0	0	0	0	0	0	0	8.90	8.91	9.04	9.03	9.12	9.32	9.11
1373416_at	Fndc3b	1	0	0	0	0	1	7.24	7.27	7.62	7.38	7.83	0	0	0	0	0	0	0	11.52	11.58	11.53	11.42	11.56	11.57	10.69
1373417_at	Plscr3	1	0	0	1	0	0	6.52	6.72	6.75	7.01	6.81	0	0	0	0	0	0	0	7.48	7.46	7.54	7.41	7.42	7.45	7.01
1373421_at	Tgif1	1	0	0	1	1	0	6.44	6.34	6.53	7.44	6.52	0	0	0	0	0	0	0	7.51	7.49	7.47	7.49	7.30	7.03	7.07
1373427_at	Rragd	1	0	0	0	0	1	6.98	6.63	7.00	6.86	6.46	0	0	0	0	0	0	0	7.83	7.82	7.79	7.69	7.64	7.82	8.02
1373432_at	Marcks	1	0	0	0	0	1	9.36	9.42	9.61	9.79	10.15	0	0	0	0	0	0	0	10.43	10.40	10.50	10.55	10.47	10.20	9.83
1373433_at	Hmgn5 /// Hmgi	1	0	1	0	1	0	7.21	7.15	7.88	7.10	7.48	0	0	0	0	0	0	0	8.87	8.96	9.01	9.13	9.09	9.03	8.86
1373436_at	Cabcoco1	1	0	0	0	1	0	5.22	5.07	5.69	4.98	5.12	0	0	0	0	0	0	0	8.59	8.53	8.52	8.50	8.51	9.12	8.94
1373442_at	Os9	1	0	0	1	1	0	8.18	8.16	7.74	8.82	7.98	0	0	0	0	0	0	0	9.33	9.41	9.41	9.37	9.41	9.52	8.53
1373447_at	Jpt2	1	0	0	0	0	1	7.32	7.25	7.50	7.23	7.84	0	0	0	0	0	0	0	8.63	8.77	8.78	8.92	8.88	8.71	8.45
1373458_at	Bex4	1	0	1	0	1	0	7.06	7.00	7.90	7.07	7.48	0	0	0	0	0	0	0	10.14	10.29	10.32	10.53	10.61	10.65	10.25
1373460_at	Slc35b2	1	0	0	1	0	0	7.22	7.12	7.21	7.72	7.10	0	0	0	0	0	0	0	9.62	9.71	9.68	9.66	9.67	9.68	9.33
1373463_at	Col5a2	1	1	0	1	1	1	6.32	5.60	6.20	5.26	5.43	0	0	0	0	0	0	0	3.70	3.69	3.68	3.62	3.64	3.61	3.02
1373466_at	Cast	1	0	1	0	0	0	7.90	7.89	8.57	7.90	7.91	0	0	0	0	0	0	0	8.79	8.78	8.79	8.86	8.82	8.75	8.47
1373472_at	Actr6	1	0	1	0	1	1	5.91	6.04	6.69	5.96	6.46	0	0	0	0	0	0	0	9.18	9.26	9.27	9.33	9.31	9.50	9.18
1373474_at	Khdc4	1	0	0	0	1	0	8.33	8.11	8.85	8.09	8.40	0	0	0	0	0	0	0	10.45	10.50	10.57	10.56	10.56	10.27	10.00
1373502_at	Dym	1	0	0	0	1	0	7.36	7.30	7.05	7.68	7.27	0	0	0	0	0	0	0	9.50	9.52	9.51	9.50	9.51	9.48	9.42
1373512_at	Ilvbl	1	0	1	0	0	1	6.41	5.98	5.80	6.27	5.49	0	0	0	0	0	0	0	7.24	7.40	7.39	7.34	7.41	7.63	6.98
1373513_at	---	1	0	1	1	1	0	8.01	8.05	8.62	7.52	8.35	0	0	0	0	0	0	0	10.41	10.55	10.66	10.73	10.88	10.91	10.36
1373517_at	Chuk	1	0	1	0	1	0	8.64	8.64	9.23	8.29	9.02	0	0	0	0	0	0	0	11.17	11.17	11.19	11.18	11.19	11.04	10.55
1373519_at	Tmed8	1	0	0	1	1	0	8.86	8.72	9.16	8.38	8.88	0	0	0	0	0	0	0	9.61	9.67	9.82	9.91	9.94	10.06	10.02
1373524_at	---	1	0	1	0	0	0	6.25	6.37	6.91	6.60	6.53	0	0	0	0	0	0	0	8.82	8.81	8.90	9.02	9.01	9.05	9.16
1373534_at	Pnisr	1	0	1	0	1	0	8.15	8.19	8.81	8.11	8.26	0	0	0	0	0	0	0	9.66	9.73	9.73	9.64	9.69	9.29	8.48
1373535_at	Enah	1	0	0	0	0	1	7.61	7.96	8.16	7.65	8.29	0	0	0	0	0	0	0	7.32	7.23	7.48	7.48	7.44	7.32	7.53
1373538_at	Usp1	1	0	1	0	0	1	6.11	6.28	6.72	6.40	6.59	0	0	0	0	0	0	0	9.05	8.88	8.99	9.12	9.08	8.99	8.53
1373544_at	Cxcl9	1	0	1	0	0	1	4.31	4.47	3.64	3.98	3.78	0	0	0	0	0	0	0	4.12	4.09	4.15	4.06	4.04	4.16	3.60
1373545_at	Ttll7	1	0	0	0	1	1	6.93	7.06	7.44	6.47	7.49	0	0	0	0	0	0	0	8.81	8.82	8.99	9.14	9.18	9.23	8.58
1373548_at	Dcaf7	1	0	1	0	1	0	8.46	8.47	9.11	8.17	8.91	0	0	0	0	0	0	0	9.90	9.80	9.92	9.94	9.91	10.01	10.49
1373557_at	Mcm4	1	0	0	0	0	1	6.52	6.45	6.91	6.66	7.02	0	0	0	0	0	0	0	10.33	10.30	10.39	10.54	10.51	10.38	9.85
1373560_at	Mgat4b	1	0	0	1	0	0	8.18	8.05	8.32	8.79	8.19	0	0	0	0	0	0	0	9.41	9.43	9.41	9.35	9.35	9.25	8.77
1373562_at	Ints5	1	0	0	0	1	0	7.82	7.70	7.42	8.08	7.49	0	0	0	0	0	0	0	8.12	8.06	8.07	8.03	8.04	8.05	7.99
1373568_at	Bmi1	1	0	1	0	0	0	8.03	8.01	8.66	8.13	8.21	0	0	0	0	0	0	0	10.28	10.29	10.36	10.46	10.41	10.03	9.86
1373570_at	Npepl1	1	0	0	0	1	0	7.20	7.16	6.86	7.62	7.13	0	0	0	0	0	0	0	7.61	7.53	7.52	7.51	7.48	7.19	7.19
1373576_at	CNOT6	1	0	0	0	1	0	7.52	7.51	8.03	7.17	7.86	0	0	0	0	0	0	0	9.81	9.85	9.90	9.97	9.99	10.07	10.09
1373577_at	Nrp1	1	0	0	0	1	0	7.61	7.56	8.18	7.30	7.60	0	0	0	0	0	0	0	7.85	7.88	7.78	7.75	7.78	7.82	7.38
1373581_at	Arhgap44	1	0	0	0	0	1	5.23	5.07	5.41	4.97	5.74	0	0	0	0	0	0	0	6.79	6.79	6.83	6.84	6.85	6.82	6.78
1373582_at	Clip3 /// LOC100	1	0	1	0	0	0	5.56	5.56	4.98	5.29	5.37	0	0	0	0	0	0	0	7.72	7.70	7.86	7.89	7.83	7.86	8.17
1373591_at	Arfip2	1	0	0	0	1	0	8.49	8.44	8.18	8.80	8.33	0	0	0	0	0	0	0	10.12	10.15	10.15	10.15	10.14	10.10	9.80
1373607_at	St3gal3	1	0	0	0	1	0	6.91	7.25	6.55	7.30	7.06	0	0	0	0	0	0	0	8.95	8.98	8.92	8.77	8.75	8.87	8.54
1373609_at	Mrps21	1	0	0	0	1	0	9.88	9.77	9.43	10.16	9.68	0	0	0	0	0	0	0	10.57	10.65	10.62	10.63	10.67	10.66	10.37
1373615_at	Frzb	1	0	1	0	1	1	6.77	7.05	7.53	6.46	5.65	0	0	0	0	0	0	0	4.56	4.53	4.46	4.52	4.40	4.55	4.38
1373626_at	---	1	0	0	0	1	0	8.54	8.41	9.10	8.37	8.72	0	0	0	0	0	0	0	8.99	8.97	9.05	9.07	8.97	8.70	8.62
1373628_at	---	1	0	0	0	1	0	5.12	5.19	5.59	4.71	4.97	0	0	0	0	0	0	0	3.46	3.53	3.51	3.49	3.49	3.47	3.22
1373631_at	Rap1gap	1	0	0	1	1	0	8.87	8.73	8.53	9.53	8.65	0	0	0	0	0	0	0	8.57	8.57	8.63	8.59	8.64	8.84	9.02
1373634_at	---	1	0	1	0	1	0	9.08	8.95	9.76	8.74	9.12	0	0	0	0	0	0	0	11.51	11.54	11.54	11.47	11.54	11.60	11.54
1373636_at	Spock1	1	0	1	0	0	1	4.53	4.82	5.25	4.67	5.67	0	0	0	0	0	0	0	7.36	7.37	7.45	7.53	7.60	7.85	7.27
1373643_at	---	1	0	1	0	1	0	7.79	7.96	8.44	7.67	8.12	0	0	0	0	0	0	0	9.22	9.29	9.31	9.33	9.27	9.26	9.22
1373644_at	Nfib	1	0	0	1	1	0	7.25	6.85	6.94	7.73	6.95	0	0	0	0	0	0	0	4.80	4.98	5.10	5.05	5.22	5.02	5.08
1373646_at	Rab15	1	0	0	0	0	1	5.04	5.11	5.04	5.08	5.89	0	0	0	0	0	0	0	7.78	7.85	7.85	7.82	7.84	7.99	8.22
1373650_at	Cmas	1	0	0	0	0	1	9.53	9.63	9.84	9.59	10.25	0	0	0	0	0	0	0	10.95	10.93	10.98	10.97	11.02	11.10	10.98
1373656_at	Ppp1r3d	1	0	1	0	1	1	5.87	6.32	6.65	5.65	6.41	0	0	0	0	0	0	0	7.97	7.98	8.03	8.07	8.01	7.79	7.96
1373659_at	Smim5	1	0	1	0	1	0	6.83	6.91	7.71	6.89	6.86	0	0	0	0	0	0	0	6.40	6.44	6.45	6.32	6.34	6.19	6.31
1373669_at	Gnpda2	1	0	1	0	1	0	5.97	5.95	6.78	5.62	6.21	0	0	0	0	0	0	0	8.07	8.15	7.95	8.14	8.19	8.25	7.72
1373677_at	Slc39a10	1	0	1	0	1	0	5.97	6.13	6.82	5.94	6.32	0	0	0	0	0	0	0	9.44	9.49	9.51	9.44	9.48	9.48	9.40
1373689_at	ESRRB	1	0	1	0	0	0	5.88	5.94	5.36	5.85	5.81	0	0	0	0	0	0	0	7.55	7.55	7.64	7.39	7.45	7.28	7.12
1373695_at	---	1	0	0	0	1	0	7.93	7.83	8.26	7.48	7.98	0	0	0	0	0	0	0	9.59	9.67	9.69	9.79	9.75	9.74	9.47
1373710_at	Slc38a4	1	0	1	1	1	0	8.56	8.72	9.27	8.04	8.61	0	0	0	0	0	0	0	10.77	10.79	10.84	10.82	10.79	10.89	10.21
1373717_at	---	1	0	0	0	1	0	3.87	3.96	3.50	4.14	3.78	0	0	0	0	0	0	0	3.95	3.97	3.82	3.93	3.95	3.99	3.73
1373718_at	Tubb2a	1	0	0	0	0	1	7.40	7.62	7.89	7.36	8.20	0	0	0	0	0	0	0	11.82	11.87	11.86	11.89	11.91	11.81	11.40
1373723_at	Gpihbp1	1	0	1	0	0	1	7.63	7.27	6.82	7.25	7.10	0	0	0	0	0	0	0	5.14	5.31	5.26	5.03	4.92	5.04	5.03
1373725_at	---	1	0	0	0	0	1	3.63	3.73	3.60	3.76	4.13	0	0	0	0	0	0	0	4.33	4.45	4.48	4.62	4.81	4.49	4.65
1373733_at	Bok	1	0	0	0	1	0	7.96	7.81	7.70	8.41	7.83	0	0	0	0	0	0	0	8.97	8.99	8.97	8.93	8.94	8.75	8.39
1373734_at	Slco3a1	1	0	0	0	0	1	6.89	6.99	7.41	6.89	7.49	0	0	0	0	0	0	0	10.38	10.40	10.38	10.36	10.47	10.11	9.58
1373739_at	---	1	0	0	1	0	0	5.01	5.17	4.96	5.45	5.34	0	0	0	0	0	0	0	5.20	5.30	5.33	4.93	5.05	5.18	5.53
1373740_at	---	1	0	1	0	1	1	5.33	5.51	6.05	4.89	6.16	0	0	0	0	0	0	0	5.42	5.53	5.54	5.60	5.51	6.00	6.34
1373743_at	Rwdd2b	1	0	0	1	0	0	5.71	5.71	5.90	6.18	5.95	0	0	0	0	0	0	0	7.78	7.81	7.96	8.03	7.92	7.53	7.06
1373744_at	Anapc1	1	0	0	0	0	1	4.69	4.60	4.62	4.80	4.22	0	0	0	0	0	0	0	7.69	7.69	7.71	7.60	7.66	7.24	7.07
1373755_at	Lrrc39	1	1	0	1	1	1	5.93	5.41	6.27	5.42	5.16	0	0	0	0	0	0	0	5.38	5.19	5.36	5.34	5.26	5.01	5.59
1373759_at	Fosb	1	1	1	0	0	0	8.35	9.41	7.77	8.02	7.92	0	0	0	0	0	0	0	4.38	4.43	4.33	4.32	4.30	4.72	4.70
1373763_at	Zbtb43	1	0	1	0	1	0	6.28	5.86	6.85	6.09	6.31	0	0	0	0	0	0	0	8.26	8.25	8.31	8.28	8.22	7.97	7.49
1373771_at	Zbtb34	1	0	1	0	0	0	4.66	4.77	5.51	5.06	4.95	0	0	0	0	0	0	0	6.66	6.75	6.84	6.86	6.85	6.78	6.54
1373776_at	---	1	0	0	0	1	1	4.92	4.84	5.44	4.60	5.41	0	0	0	0	0	0	0	8.63	8.86	8.79	8.76	8.98	9.01	8.67
1373778_at	Acacb	1	0	0	0	1	1	6.54	6.56	6.16	6.79	5.85	0	0	0	0	0	0	0	5.56	5.50	5.55	5.63	5.52	5.43	5.44
1373780_at	Tspan1	1	0	1	0	1	1	6.52	6.51	5.97	6.58	6.02	0	0	0	0	0	0	0	5.64	5.67	5.70	5.67	5.71	5.72	5.95
1373784_at	Cct8	1	0	0	0	1	0	5.69	5.62	6.25	5.32	5.89	0	0	0	0	0	0	0	8.96	8.96	9.10	9.25	9.24	9.16	8.84
1373786_at	Znf703	1	0	1	0	1	0	5.22	5.26	4.64	5.26	4.85	0	0	0	0	0	0	0	9.82	9.86	9.87	9.73	9.81	9.56	9.66
1373787_at	Slc6a9	1	0	0	0	1	0	7.59	7.64	7.20	7.98	7.33	0	0	0	0	0	0	0	7.78	7.75	7.70	7.66	7.62	7.50	7.15
1373794_at	Egln2	1	0	0	0	1	1	8.54	8.35	8.11	8.71	8.07	0	0	0	0	0	0	0	9.62	9.60	9.62	9.50	9.44	9.23	9.10
1373804_at	Foxp1	1	0	0	1	0	0	6.92	7.23	6.96	7.41	7.07	0	0	0	0	0	0	0	7.05	7.01	7.18	7.10	7.03	7.20	7.48
1373806_at	Cfap36	1	0	0	0	1	0	6.96	7.00	7.34	6.62	7.16	0	0	0	0	0	0	0	9.56	9.62	9.67	9.74	9.75	9.85	9.62
1373807_at	Vegfa	1	0	0	0	1	0	9.28	9.27	9.81	9.10	9.54	0	0	0	0	0	0	0	11.96	12.03	11.99	11.80	11.95	11.99	11.17
1373810_at	Pla2g12a	1	0	0	0	1	0	7.81	7.51	7.45	8.18	7.44	0	0	0	0	0	0	0	8.92	8.98	8.95	8.86	8.88	8.88	8.85
1373813_at	Dnajc10	1	0	0	0	0	1	7.44	7.52	7.99	7.38	8.29	0	0	0	0	0	0	0	9.95	9.94	9.97	10.06	10.05	9.85	9.61
1373822_at	Bola2 /// RGD15	1	0	0	0	1	0	8.48	8.71	8.24	8.86	8.41	0	0	0	0	0	0	0	10.79	10.81	10.80	10.78	10.76	10.89	10.73
1373834_at	Borcs8	1	0	0	0	0	1	6.47	6.21	6.35	6.58	6.00	0	0	0	0	0	0	0	8.52	8.53	8.54	8.57	8.51	8.24	7.86
1373835_at	Fbxo8	1	0	1	1	1	0	6.99	6.84	7.69	6.46	7.20	0	0	0	0	0	0	0	9.98	10.03	10.01	10.04	10.03	10.12	9.63
1373840_at	Nudt9	1	1	0	0	0	0	6.37	5.89	6.06	6.17	5.98	0	0	0	0	0	0	0	7.01	7.05	7.21	7.19	7.20	7.12	7.00
1373847_at	Tm4sf1	1	0	0	1	1	0	7.34	7.32	7.79	6.79	7.38	0	0	0	0	0	0	0	3.94	3.88	4.00	3.91	3.82	3.95	3.16
1373862_at	Tmed7	1	0	0	0	0	1	10.26	10.28	10.76	10.30	10.91	0	0	0	0	0	0	0	12.08	12.14	12.11	12.15	12.19	12.09	11.48
1373865_at	Snap91	1	0	0	1	1	1	7.71	7.58	8.28	7.19	8.34	0	0	0	0	0	0	0	11.13	11.09	11.18	11.24	11.22	11.06	10.64
1373866_at	Coq10b	1	0	0	1	1	0	7.35	7.29	7.64	6.63	7.70	0	0	0	0	0	0	0	9.77	9.81	9.79	9.78	9.88	10.10	9.79
1373867_at	Kdm5a	1	0	1	0	0	1	7.04	7.18	7.61	7.09	7.53	0	0	0	0	0	0	0	7.68	7.82	7.88	7.96	7.93	8.02	7.77
1373872_at	Mgat4a	1	1	0	1	0	0	8.38	7.91	8.73	8.89	7.95	0	0	0	0	0	0	0	8.96	8.95	8.98	8.85	8.95	8.97	8.75
1373881_at	Arhgdib	1	0	0	0	0	1	6.56	6.73	6.49	6.54	7.06	0	0	0	0	0	0	0	5.03	5.05	5.07	4.86	4.91	4.99	4.81
1373893_at	Cdc23	1	0	1	0	0	0	6.05	6.25	6.69	6.25	6.32	0	0	0	0	0	0	0	8.90	8.90	8.90	8.87	8.90	8.81	8.22
1373895_at	LOC100912002	1	0	0	1	0	1	6.92	7.23	7.24	7.39	7.70	0	0	0	0	0	0	0	10.31	10.28	10.34	10.39	10.36	10.56	10.63
1373901_at	Cdk2ap2	1	0	1	0	1	0	9.00	8.83	8.32	9.06	8.75	0	0	0	0	0	0	0	9.59	9.58	9.57	9.59	9.55	9.51	9.17
1373902_at	Rimklb	1	0	1	0	1	0	4.94	5.17	5.66	4.92	5.25	0	0	0	0	0	0	0	6.00	6.02	6.17	6.22	6.17	6.26	6.39
1373905_at	Hnrnpr	1	0	1	0	1	0	7.88	7.90	8.48	7.77	8.15	0	0	0	0	0	0	0	10.88	10.94	10.96	11.03	11.08	11.05	10.78
1373908_at	---	1	0	1	0	1	0	8.11	7.94	6.99	8.43	7.94	0	0	0	0	0	0	0	8.00	7.96	7.99	7.99	8.03	8.31	8.57
1373911_at	Postn	1	1	1	1	0	0	4.74	4.18	4.17	4.22	4.65	0	0	0	0	0	0	0	3.83	3.78	3.90	3.77	3.90	3.65	3.22
1373919_at	Cisd2	1	0	0	0	0	1	7.75	7.87	8.25	7.81	8.28	0	0	0	0	0	0	0	9.51	9.55	9.58	9.62	9.58	9.55	9.14
1373921_at	Echdc3	1	0	1	0	1	0	8.31	8.24	7.50	8.57	7.95	0	0	0	0	0	0	0	4.84	4.84	5.02	4.79	4.79	4.86	4.65
1373933_at	Rapgef2	1	0	1	0	0	0	7.83	7.79	8.42	7.73	8.23	0	0	0	0	0	0	0	9.40	9.32	9.33	9.40	9.30	9.38	9.49
1373935_at	Pold2	1	0	1	0	0	0	7.09	6.89	6.50	6.93	6.75	0	0	0	0	0	0	0	9.09	9.08	9.16	9.27	9.23	9.49	9.39
1373940_at	Nck1	1	0	1	0	1	0	8.46	8.35	9.07	8.18	8.61	0	0	0	0	0	0	0	8.73	8.77	8.81	8.88	8.86	8.72	8.26
1373947_at	Dpt	1	0	0	1	1	1	6.07	5.63	6.49	5.38	5.47	0	0	0	0	0	0	0	4.12	4.09	4.12	4.01	3.97	3.91	3.44

Probeset ID	Gene Symbol	Islet Data Set (log2 ratio ≥0.263; FDR <20%) union n=5325 1=YES 0=NO	Islet DRlyp/lyp ND vs ND+Lp299v (log2 ratio ≥0.263; FDR<20%) n=429 1=YES 0=NO	Islet DRlyp/lyp ND vs HCD (log2 ratio ≥0.263; FDR<20%) n=2247 1=YES 0=NO	Islet DRlyp/lyp ND vs HCD+Lp299v (log2 ratio ≥0.263; FDR<20%) n=1595 1=YES 0=NO	Islet DRlyp/lyp HCD vs HCD+Lp299v (log2 ratio ≥0.263; FDR<20%) n=3026 1=YES 0=NO	Islet DRlyp/lyp ND vs Flyp/lyp ND (log2 ratio ≥ 0.263; FDR<20%) n=1950 1=YES 0=NO	Islet Mean Log2 Intensity DRlyp/lyp ND	Islet Mean Log2 Intensity DRlyp/lyp ND+Lp299v	Islet Mean Log2 Intensity DRlyp/lyp HCD	Islet Mean Log2 Intensity DRlyp/lyp HCD+Lp299v	Islet Mean Log2 Intensity Flyp40 ND	Rinm5f data union of all conditions n=3705 1=YES 0=NO	Rinm5f 30 µM propionate/ 6 µM butyrate vs Media (FDR<20%; 1.5 fold) n=3 1=YES 0=NO	Rinm5f 60 µM propionate/ 12 µM butyrate vs Media (FDR<20%; 1.5 fold) n=18 1=YES 0=NO	Rinm5f 120 µM propionate/ 24 µM butyrate vs Media (FDR<20%; 1.5 fold) n=164 1=YES 0=NO	Rinm5f 240 µM propionate /48 µM butyrate vs Media (FDR<20%; 1.5 fold) n=268 1=YES 0=NO	Rinm5f 1500 µM propionate/ 300 µM butyrate vs Media (FDR<20%; 1.5 fold) n=2491 1=YES 0=NO	Rinm5f 500 µM propionate/ 500 µM butyrate vs Media (FDR<20%; 1.5 fold) n=3001 1=YES 0=NO	Rinm5f Mean Log2 Intensity Media only	Rinm5f Mean Log2 Intensity 30 µM propionate/ 6 µM butyrate	Rinm5f Mean Log2 Intensity 60 µM propionate/ 12 µM butyrate	Rinm5f Mean Log2 Intensity 120 µM propionate/ 24 µM butyrate	Rinm5f Mean Log2 Intensity 240 µM propionate/ 48 µM butyrate	Rinm5f Mean Log2 Intensity 1500 µM propionate /300 µM butyrate	Rinm5f Mean Log2 Intensity 500 µM propionate/ 500 µM butyrate
1373949_at	Atp13a2	1	0	1	0	1	0	7.27	7.31	6.68	7.68	7.07	0	0	0	0	0	0	0	7.28	7.32	7.27	7.34	7.34	7.35	7.37
1373951_at	Prkar1a	1	0	0	0	1	0	10.44	10.41	10.93	10.03	10.68	0	0	0	0	0	0	0	11.53	11.55	11.56	11.63	11.60	11.60	10.98
1373956_at	Fundc1	1	0	1	0	1	0	6.04	5.82	6.68	5.91	6.34	0	0	0	0	0	0	0	8.11	8.31	8.29	8.43	8.48	8.26	7.59
1373964_at	C5orf63	1	0	0	0	0	1	6.71	6.60	7.08	6.71	7.20	0	0	0	0	0	0	0	8.40	8.49	8.55	8.73	8.73	8.84	8.41
1373966_at	Rai1	1	0	1	0	1	0	8.11	8.15	8.78	8.08	8.41	0	0	0	0	0	0	0	8.27	8.27	8.25	8.20	8.16	7.85	7.45
1373968_at	Ranbp9	1	0	1	0	1	0	8.60	8.58	9.19	8.44	8.92	0	0	0	0	0	0	0	11.40	11.43	11.41	11.37	11.42	11.37	11.11
1373977_at	Kif5c	1	0	1	0	1	1	7.61	7.85	8.36	7.47	8.29	0	0	0	0	0	0	0	10.24	10.23	10.29	10.29	10.28	10.30	10.78
1373986_at	Fnip2	1	0	0	0	1	0	8.81	8.75	9.30	8.59	8.69	0	0	0	0	0	0	0	9.31	9.30	9.29	9.35	9.33	9.37	9.82
1373992_at	Mgc108823	1	1	0	0	0	0	3.19	5.35	3.01	2.96	3.05	0	0	0	0	0	0	0	3.15	3.25	3.23	3.15	3.09	3.25	2.91
1374002_at	Atxn7l3b	1	0	1	0	0	0	8.55	8.51	9.16	8.56	8.77	0	0	0	0	0	0	0	10.11	9.98	10.11	10.09	10.02	9.95	10.24
1374004_at	Prepl	1	0	1	0	1	0	7.22	7.46	7.81	6.95	7.52	0	0	0	0	0	0	0	9.49	9.50	9.51	9.53	9.40	9.14	9.03
1374017_at	Trim72	1	0	0	0	1	0	5.34	5.16	5.74	5.05	5.45	0	0	0	0	0	0	0	5.32	5.38	5.51	5.58	5.47	5.47	4.87
1374018_at	Ascc2	1	0	0	0	1	0	5.99	6.03	5.49	6.13	5.86	0	0	0	0	0	0	0	7.34	7.34	7.37	7.24	7.29	7.14	7.40
1374027_at	Zscan26	1	0	1	0	1	0	8.07	7.93	8.77	7.73	8.23	0	0	0	0	0	0	0	7.91	8.04	8.10	8.23	8.38	8.36	7.80
1374030_at	Sik3	1	0	1	0	0	1	7.90	8.13	8.54	7.88	8.39	0	0	0	0	0	0	0	7.95	7.86	8.01	8.02	7.94	7.78	7.34
1374035_at	Rem2	1	0	0	0	0	1	6.47	6.80	6.81	6.30	7.02	0	0	0	0	0	0	0	8.38	8.39	8.56	8.79	8.68	8.61	8.68
1374036_at	Mcm2	1	0	0	1	0	0	6.22	6.55	6.43	6.76	6.62	0	0	0	0	0	0	0	10.22	10.25	10.26	10.28	10.32	10.33	10.13
1374039_at	Car14	1	0	1	0	0	0	6.01	5.97	5.42	5.87	5.74	0	0	0	0	0	0	0	5.14	5.10	5.15	4.85	5.01	4.85	4.59
1374042_at	Camkmt	1	0	0	0	0	1	4.78	5.21	5.33	4.89	5.49	0	0	0	0	0	0	0	7.44	7.47	7.55	7.58	7.54	7.40	7.49
1374065_at	Met	1	1	1	0	0	1	8.61	8.12	8.05	8.55	8.04	0	0	0	0	0	0	0	8.90	8.88	8.96	8.95	8.89	9.03	9.13
1374069_at	Rab11a	1	0	1	0	1	0	7.51	7.52	8.22	7.27	7.93	0	0	0	0	0	0	0	9.14	9.30	9.29	9.45	9.44	9.55	9.09
1374070_at	Gpx2	1	0	1	0	0	0	4.96	4.94	4.34	4.84	5.34	0	0	0	0	0	0	0	4.31	4.34	4.28	4.07	4.28	4.16	4.06
1374073_at	Slc46a1	1	0	0	1	1	0	5.64	5.60	5.42	6.13	5.32	0	0	0	0	0	0	0	6.36	6.31	6.52	6.57	6.50	6.50	6.25
1374080_at	Six5	1	0	0	0	1	0	6.40	6.42	5.95	6.61	6.21	0	0	0	0	0	0	0	6.79	6.74	6.71	6.45	6.41	6.30	5.94
1374081_at	Casc4	1	0	1	0	1	1	8.75	8.94	9.69	8.32	9.87	0	0	0	0	0	0	0	10.69	10.76	10.77	10.78	10.83	10.82	10.64
1374085_at	Mxd4	1	0	0	0	0	1	6.61	6.19	6.72	6.37	6.12	0	0	0	0	0	0	0	8.35	8.32	8.33	8.36	8.21	8.26	7.58
1374089_at	---	1	0	1	0	1	0	5.06	5.19	5.85	4.90	5.32	0	0	0	0	0	0	0	5.88	5.73	6.11	6.07	5.93	6.17	5.86
1374095_at	Galnt6	1	0	1	0	0	0	5.06	5.02	4.48	4.72	4.71	0	0	0	0	0	0	0	4.66	4.56	4.58	4.35	4.49	4.61	4.67
1374104_at	Cldn5	1	0	1	0	0	0	4.74	4.74	4.16	4.48	4.36	0	0	0	0	0	0	0	4.18	4.18	4.21	4.16	4.01	4.12	3.59
1374105_at	Higd1a	1	0	0	1	0	0	5.47	5.37	5.43	4.98	5.73	0	0	0	0	0	0	0	8.35	8.54	8.42	8.30	8.43	8.57	8.44
1374111_at	Pnn	1	0	1	0	1	0	7.36	7.08	8.21	7.34	7.44	0	0	0	0	0	0	0	9.81	9.89	9.92	9.90	10.02	9.70	9.07
1374117_at	Baiap2	1	0	1	0	0	1	6.40	6.15	5.75	6.15	5.73	0	0	0	0	0	0	0	8.40	8.44	8.44	8.54	8.47	8.53	8.26
1374122_at	Myo5c	1	0	0	0	1	1	8.48	8.38	8.16	8.89	7.34	0	0	0	0	0	0	0	4.01	3.89	3.97	3.82	3.75	3.91	3.32
1374129_at	Oaz2	1	0	0	1	0	0	8.13	7.97	8.17	7.53	7.88	0	0	0	0	0	0	0	9.53	9.67	9.68	9.71	9.71	9.70	9.19
1374134_at	Elk3	1	0	1	0	0	1	6.30	6.16	5.55	6.09	5.70	0	0	0	0	0	0	0	5.92	5.95	6.05	5.75	6.07	6.44	6.37
1374135_at	Ipo4	1	0	0	0	1	0	8.27	8.13	7.95	8.59	8.13	0	0	0	0	0	0	0	8.57	8.49	8.60	8.62	8.54	8.14	7.82
1374142_at	Prr15	1	0	0	1	0	0	4.99	4.58	5.17	5.60	4.56	0	0	0	0	0	0	0	6.47	6.55	6.37	6.06	6.17	6.44	7.04
1374150_at	---	1	0	1	0	0	0	7.20	7.24	7.87	7.19	7.65	0	0	0	0	0	0	0	9.45	9.39	9.45	9.51	9.44	9.01	8.58
1374158_at	---	1	0	1	0	0	0	5.81	5.75	6.46	5.92	6.18	0	0	0	0	0	0	0	7.92	7.95	8.05	7.94	7.96	7.69	7.25
1374168_at	Sphkap	1	0	1	0	1	0	10.59	10.84	11.31	10.33	11.02	0	0	0	0	0	0	0	10.51	10.58	10.58	10.59	10.69	10.48	9.98
1374170_at	Tdp2	1	0	1	0	0	0	5.97	5.95	6.56	6.18	6.31	0	0	0	0	0	0	0	8.39	8.41	8.43	8.45	8.47	8.38	8.07
1374171_at	Abcc9	1	0	1	1	1	0	6.08	5.65	6.74	5.39	6.01	0	0	0	0	0	0	0	3.48	3.42	3.53	3.29	3.48	3.49	3.02
1374187_at	Adam22	1	1	1	1	0	1	5.23	4.76	6.12	5.76	6.12	0	0	0	0	0	0	0	8.49	8.58	8.65	8.83	8.92	8.88	8.64
1374191_at	Rhbdf1	1	0	0	0	1	1	7.56	7.29	7.12	7.96	6.62	0	0	0	0	0	0	0	6.47	6.64	6.67	6.46	6.55	6.70	6.44
1374193_at	Galnt7	1	0	1	0	0	0	6.30	6.01	7.04	6.36	6.37	0	0	0	0	0	0	0	8.02	7.98	8.07	8.06	7.98	7.49	7.52
1374194_at	Slc39a11	1	0	1	0	1	0	8.75	8.75	8.02	9.05	8.79	0	0	0	0	0	0	0	10.30	10.32	10.24	10.08	10.22	10.18	9.84
1374221_at	Slc29a3	1	0	1	0	0	0	6.58	6.44	6.06	6.59	6.42	0	0	0	0	0	0	0	7.12	7.22	7.22	7.20	7.19	6.97	7.02
1374230_at	Virma	1	0	0	0	1	0	6.21	6.25	6.70	5.99	6.52	0	0	0	0	0	0	0	7.93	7.87	8.04	7.99	7.98	7.79	7.85
1374232_at	Pik3ca	1	0	1	0	0	0	6.28	6.23	7.07	6.48	6.50	0	0	0	0	0	0	0	8.55	8.34	8.51	8.42	8.42	8.51	8.44
1374235_at	Rcan2	1	0	1	0	1	1	6.25	6.63	7.13	6.13	6.79	0	0	0	0	0	0	0	7.61	7.54	7.66	7.64	7.62	7.72	7.36
1374247_at	Stab1	1	0	0	0	1	0	4.37	4.53	3.96	4.62	4.37	0	0	0	0	0	0	0	3.98	3.96	4.05	3.86	3.88	3.89	3.38
1374266_at	---	1	0	0	0	1	0	5.47	5.58	5.32	5.88	5.74	0	0	0	0	0	0	0	7.74	7.63	7.82	7.72	7.81	7.94	7.40
1374269_at	Apba1	1	0	0	0	0	1	5.34	5.81	5.70	5.37	5.99	0	0	0	0	0	0	0	8.87	8.85	8.97	8.95	8.90	8.71	8.86
1374287_at	Pald1	1	0	0	0	1	0	6.93	6.90	6.68	7.26	6.82	0	0	0	0	0	0	0	7.73	7.68	7.75	7.63	7.62	7.88	8.02
1374293_at	H2aj	1	0	0	1	1	0	8.61	8.98	8.35	9.14	8.41	0	0	0	0	0	0	0	9.38	9.35	9.33	9.24	9.12	9.42	9.63
1374299_at	Dhx9	1	0	1	0	0	0	7.09	7.35	7.76	7.27	7.36	0	0	0	0	0	0	0	9.62	9.81	9.96	9.89	10.13	9.87	9.39
1374304_at	Xrcc4	1	0	1	0	0	0	4.38	4.59	5.00	4.50	4.84	0	0	0	0	0	0	0	6.57	6.75	6.79	6.91	6.70	6.50	6.19
1374307_at	---	1	0	0	0	0	1	9.55	9.67	10.09	9.54	10.04	0	0	0	0	0	0	0	11.50	11.44	11.49	11.45	11.39	11.14	10.90
1374315_at	---	1	0	0	0	0	1	6.65	7.02	7.07	6.57	7.19	0	0	0	0	0	0	0	7.48	7.55	7.67	7.82	7.86	7.85	7.92
1374325_at	Itga1	1	0	0	0	0	1	6.70	6.67	6.65	6.58	6.10	0	0	0	0	0	0	0	5.21	5.14	5.12	5.05	5.05	5.42	5.56
1374327_at	Bbs4	1	0	1	0	1	0	5.34	5.47	6.00	5.20	5.57	0	0	0	0	0	0	0	7.25	7.43	7.43	7.50	7.53	7.29	7.22
1374328_at	---	1	0	1	0	0	1	4.54	4.63	5.19	4.80	5.34	0	0	0	0	0	0	0	8.47	8.44	8.53	8.58	8.56	8.46	7.94
1374334_at	Igha /// LOC366	1	0	1	1	0	0	5.03	5.02	3.90	4.20	4.94	0	0	0	0	0	0	0	4.82	4.76	4.74	4.71	4.64	4.75	4.31
1374341_at	Thap7	1	0	0	0	1	0	6.26	6.34	5.96	6.59	6.02	0	0	0	0	0	0	0	8.04	8.14	8.17	8.08	7.97	7.61	7.36
1374349_at	Ctdspl	1	0	0	0	0	1	8.14	8.13	8.23	8.59	7.54	0	0	0	0	0	0	0	8.27	8.27	8.23	8.15	8.12	8.22	8.17
1374351_at	Mlxip	1	0	0	0	1	0	7.85	7.61	7.36	8.04	7.48	0	0	0	0	0	0	0	7.27	7.28	7.34	7.34	7.27	6.96	7.00
1374359_at	Ccne2	1	0	1	0	0	1	4.51	4.77	5.13	4.92	4.99	0	0	0	0	0	0	0	10.42	10.48	10.45	10.57	10.63	10.81	10.48
1374382_at	---	1	0	0	1	1	1	6.77	6.55	6.40	7.26	6.15	0	0	0	0	0	0	0	5.23	5.32	5.18	5.21	5.05	5.28	4.77
1374395_at	Xpo1	1	0	0	0	1	0	7.57	7.45	8.11	7.32	7.97	0	0	0	0	0	0	0	9.68	9.68	9.68	9.72	9.66	9.55	9.39
1374396_at	Atp6v1c1	1	0	0	0	1	0	8.52	8.49	8.90	8.14	8.82	0	0	0	0	0	0	0	11.00	11.05	11.04	11.07	11.06	11.16	10.91
1374400_at	Efr3a	1	0	0	0	0	1	6.21	5.89	6.69	6.39	6.76	0	0	0	0	0	0	0	9.55	9.55	9.66	9.71	9.74	9.98	10.01
1374404_at	Jun	1	0	0	1	0	1	7.98	8.25	7.74	7.30	7.38	0	0	0	0	0	0	0	6.53	6.53	6.66	6.46	6.21	6.31	5.66
1374405_at	Hnrnpa3	1	0	1	0	0	0	8.96	8.92	9.54	9.09	9.16	0	0	0	0	0	0	0	9.96	10.13	10.11	10.25	10.29	10.33	10.01
1374410_at	Dock5	1	0	0	0	0	1	6.27	6.34	6.40	6.37	6.78	0	0	0	0	0	0	0	6.89	6.90	6.93	6.79	6.91	7.29	7.44
1374423_at	Mfsd14a	1	0	0	0	1	0	8.94	8.85	9.43	8.66	9.06	0	0	0	0	0	0	0	10.85	10.83	10.84	10.79	10.78	10.42	9.52
1374427_at	Syt13	1	0	1	0	0	1	9.85	10.10	10.54	9.85	10.51	0	0	0	0	0	0	0	10.62	10.67	10.80	10.91	10.92	11.07	10.97
1374431_at	Atp6v1a	1	0	0	0	1	0	8.64	8.62	9.17	8.31	9.04	0	0	0	0	0	0	0	10.29	10.22	10.27	10.23	10.21	10.28	10.34
1374433_at	LOC103690154	1	1	0	0	0	0	7.63	6.97	7.55	7.75	7.37	0	0	0	0	0	0	0	7.86	7.78	7.87	7.78	7.75	7.65	8.13
1374436_at	Sbds	1	0	0	0	1	0	7.63	7.50	8.03	7.32	7.70	0	0	0	0	0	0	0	9.77	9.80	9.84	9.91	9.89	9.88	9.40
1374440_at	Hsd17b11	1	1	0	0	0	1	7.36	6.89	7.27	7.62	6.51	0	0	0	0	0	0	0	5.20	5.20	5.41	5.41	5.24	5.30	5.52
1374447_at	Usp9x	1	0	0	0	1	0	8.04	8.02	8.49	7.67	8.22	0	0	0	0	0	0	0	9.47	9.44	9.51	9.54	9.47	9.13	9.35
1374456_at	Tmx3	1	0	1	0	1	0	8.08	8.12	8.76	7.77	8.47	0	0	0	0	0	0	0	10.92	11.02	10.95	11.02	11.02	10.74	9.98
1374462_at	Kifap3	1	0	1	0	1	0	7.12	7.13	7.72	6.97	7.45	0	0	0	0	0	0	0	8.47	8.52	8.57	8.59	8.53	8.19	8.16
1374472_at	Vps37a	1	0	1	1	1	0	7.03	7.31	7.79	6.54	7.39	0	0	0	0	0	0	0	9.69	9.68	9.65	9.68	9.66	9.52	9.07
1374489_at	Gtpbp2	1	0	0	1	0	0	6.68	6.94	6.91	7.31	7.12	0	0	0	0	0	0	0	7.67	7.78	7.79	7.76	7.78	7.81	7.46
1374491_at	Cmtm8	1	0	0	0	0	1	8.31	8.04	8.20	8.66	7.83	0	0	0	0	0	0	0	8.22	8.25	8.30	8.34	8.35	8.48	8.43
1374497_at	Ncln	1	0	0	0	1	0	8.93	9.04	8.56	9.40	9.02	0	0	0	0	0	0	0	9.79	9.76	9.82	9.90	9.84	9.76	9.35
1374501_at	G3bp2	1	0	1	1	1	0	8.27	8.22	8.87	7.66	8.15	0	0	0	0	0	0	0	9.94	9.97	10.04	10.21	10.06	9.90	9.59
1374508_at	Mtln	1	0	0	0	1	0	8.61	8.55	8.30	8.96	8.41	0	0	0	0	0	0	0	9.79	9.74	9.66	9.68	9.56	9.38	9.02
1374513_at	Septin7	1	0	1	0	1	0	8.70	8.74	9.36	8.33	9.06	0	0	0	0	0	0	0	11.83	11.84	11.88	11.97	11.91	11.86	11.27
1374515_at	Spryd7	1	0	1	0	1	0	7.55	7.66	8.22	7.50	7.90	0	0	0	0	0	0	0	8.55	8.48	8.51	8.57	8.58	8.49	8.24
1374526_at	Ankfy1	1	0	0	0	1	0	7.52	7.55	8.02	7.32	7.81	0	0	0	0	0	0	0	10.19	10.16	10.24	10.19	10.21	10.10	10.29
1374531_at	Slc6a6	1	0	1	0	1	0	8.38	8.52	9.00	8.18	8.66	0	0	0	0	0	0	0	10.36	10.32	10.44	10.27	10.28	10.35	10.32
1374541_at	Gys1	1	0	0	0	1	0	5.53	5.66	5.24	5.94	5.53	0	0	0	0	0	0	0	8.84	8.83	8.85	8.83	8.73	8.54	8.38
1374545_at	Mex3c	1	0	1	0	0	0	6.72	6.67	7.48	6.80	7.04	0	0	0	0	0	0	0	9.28	9.31	9.23	9.24	9.22	9.03	8.86
1374548_at	Aff4	1	0	0	0	1	0	8.85	8.72	9.43	8.45	9.11	0	0	0	0	0	0	0	10.11	10.09	10.18	10.23	10.26	10.01	9.66
1374554_at	Pithd1	1	0	0	0	0	1	8.39	8.28	8.54	8.38	7.83	0	0	0	0	0	0	0	10.75	10.79	10.78	10.75	10.70	10.74	10.49
1374556_at	Smox	1	0	1	0	1	1	7.67	7.45	7.13	7.95	7.10	0	0	0	0	0	0	0	6.72	6.70	6.59	6.44	6.43	6.50	6.49
1374563_at	---	1	0	1	0	0	0	4.96	5.08	5.68	5.09	5.34	0	0	0	0	0	0	0	8.50	8.60	8.66	8.77	8.82	8.39	7.64
1374565_at	Nek6	1	0	0	1	1	0	6.21	5.93	6.08	6.75	6.01	0	0	0	0	0	0	0	8.87	8.86	8.81	8.60	8.75	8.73	8.65
1374570_at	Agpat2	1	0	0	0	0	1	5.41	5.40	5.35	5.87	4.91	0	0	0	0	0	0	0	8.58	8.65	8.67	8.74	8.67	8.57	8.28
1374574_at	Fam214a	1	0	1	0	0	1	4.69	4.85	5.33	5.11	5.59	0	0	0	0	0	0	0	4.84	4.92	4.97	5.16	5.22	5.25	5.07
1374575_at	Creb3l1	1	0	0	0	1	0	7.47	7.42	7.17	7.81	7.03	0	0	0	0	0	0	0	9.83	9.86	9.93	9.82	9.82	9.30	9.23
1374582_at	Kctd9	1	0	1	0	1	0	6.74	6.79	7.37	6.45	7.01	0	0	0	0	0	0	0	7.86	7.80	7.90	7.86	7.81	7.72	7.63
1374587_at	Tmtc3	1	0	1	0	1	0	6.35	6.15	7.03	6.15	6.57	0	0	0	0	0	0	0	8.58	8.58	8.61	8.71	8.74	8.88	8.58
1374591_at	Ptprd	1	0	0	0	0	1	6.91	7.05	6.93	7.25	6.07	0	0	0	0	0	0	0	5.44	5.32	5.52	5.65	5.54	5.59	5.41
1374600_at	H4c11	1	0	0	1	1	0	7.85	7.76	7.65	9.08	7.46	0	0	0	0	0	0	0	4.96	4.82	4.91	4.66	4.55	4.81	4.46
1374606_at	---	1	0	0	0	1	0	6.09	5.92	5.92	6.52	5.88	0	0	0	0	0	0	0	6.71	6.68	6.93	6.97	7.00	6.96	6.79
1374609_at	Rab3gap2	1	0	0	0	1	0	7.43	7.55	7.89	7.17	7.76	0	0	0	0	0	0	0	8.31	8.33	8.41	8.39	8.42	8.29	8.34
1374615_at	Ankrd44	1	0	0	0	1	0	7.51	7.34	8.03	7.11	7.79	0	0	0	0	0	0	0	8.10	8.06	8.24	8.22	8.24	8.07	8.26
1374620_at	Ceacam1	1	0	0	0	0	1	4.29	4.68	4.61	4.47	4.90	0	0	0	0	0	0	0	8.80	8.92	8.99	9.13	9.19	8.64	8.11
1374626_at	Lrg1	1	0	1	0	0	0	5.27	5.06	4.65	5.05	4.97	0	0	0	0	0	0	0	4.37	4.28	4.35	4.19	4.21	4.31	3.62
1374630_at	Clic3	1	0	1	0	1	1	5.22	5.15	4.25	4.85	4.48	0	0	0	0	0	0	0	4.93	5.19	5.13	5.27	5.11	4.74	4.76
1374647_at	Rsbn1	1	0	1	0	1	0	6.45	6.26	7.18	5.98	6.49	0	0	0	0	0	0	0	9.08	9.18	9.09	9.05	9.05	8.94	8.57
1374675_at	Tmem9b	1	0	0	0	0	1	7.08	7.03	7.19	6.96	7.57	0	0	0	0	0	0	0	9.67	9.64	9.68	9.74	9.76	9.94	9.74
1374676_at	Sgms1	1	0	1	0	1	0	7.09	7.15	8.24	7.45	7.51	0	0	0	0	0	0	0	10.09	10.09	10.16	10.25	10.32	10.33	9.91
1374683_at	Sgcg	1	0	1	0	1	1	6.51	6.61	5.30	6.64	4.85	0	0	0	0	0	0	0	4.28	4.14	4.34	4.11	4.03	4.00	3.85
1374684_at	LOC100911498	1	1	1	1	0	1	10.02	11.07	10.79	11.05	10.76	0	0	0	0	0	0	0	3.38	3.33	3.42	3.39	3.38	3.37	2.83
1374686_at	Slc30a7	1	0	0	0	0	1	5.59	5.74	6.01	6.03	6.21	0	0	0	0	0	0	0	8.36	8.42	8.33	8.55	8.48	8.17	7.81
1374688_at	---	1	0	0	0	0	1	7.85	7.94	8.29	7.78	8.35	0	0	0	0	0	0	0	9.72	9.78	9.91	9.96	10.05	10.29	10.01
1374692_at	Snx14	1	0	1	0	1	1	7.25	7.22	8.16	7.11	8.33	0	0	0	0	0	0	0	8.38	8.44	8.46	8.40	8.51	8.59	8.37
1374693_at	Parp16	1	0	1	1	1	0	7.19	7.00	6.57	7.87	6.83	0	0	0	0	0	0	0	4.81	4.86	4.93	4.74	4.76	4.90	4.88
1374700_at	Spred1	1	0	1	0	0	0	5.78	5.43	6.60	5.98	5.73	0	0	0	0	0	0	0	7.92	8.00	8.06	8.18	8.26	8.14	7.73
1374718_at	Dtx3l	1	0	0	1	1	0	6.81	6.93	6.78	7.37	6.50	0	0	0	0	0	0	0	6.56	6.55	6.68	6.85	6.80	6.95	6.82
1374732_at	Hexim2	1	0	0	0	0	1	4.83	4.81	4.95	4.85	5.41	0	0	0	0	0	0	0	8.03	8.21	8.27	8.46	8.46	8.34	7.80
1374733_at	Sympk	1	0	0	0	1	0	6.59	6.58	6.21	6.92	6.56	0	0	0	0	0	0	0	8.10	8.05	8.18	8.09	8.07	8.05	8.30
1374738_at	Cwc27	1	0	0	1	0	0	5.68	5.65	5.88	6.31	5.29	0	0	0	0	0	0	0	7.56	7.66	7.64	7.65	7.67	7.30	6.84
1374749_at	Lclat1	1	0	0	1	1	0	7.20	7.04	6.81	7.71	7.06	0	0	0	0	0	0	0	7.26	7.17	7.21	7.32	7.29	6.97	6.81
1374752_at	Mdfic	1	0	0	1	0	0	5.62	5.16	5.91	6.35	5.40	0	0	0	0	0	0	0	7.27	7.32	7.38	7.41	7.31	7.64	7.45
1374753_at	Tent2	1	0	1	0	0	1	4.78	4.74	5.44	4.82	5.26	0	0	0	0	0	0	0	8.24	8.36	8.52	8.72	8.78	8.62	8.10
1374761_at	Gga1	1	0	0	1	1	0	7.66	7.68	7.17	8.17	7.47	0	0	0	0	0	0	0	8.11	8.15	8.28	8.26	8.24	8.34	8.41
1374766_at	Fam91a1	1	0	0	0	1	0	7.62	7.48	8.11	7.27	7.94	0	0	0	0	0	0	0	10.00	10.05	10.12	10.21	10.30	10.58	10.39
1374777_at	Tigar	1	0	1	0	0	1	6.29	6.48	6.94	6.38	7.03	0	0	0	0	0	0	0	7.13	7.13	7.27	7.25	7.37	7.34	7.23
1374778_at	Ctsc	1	0	0	1	0	1	8.35	8.06	8.33	8.89	7.87	0	0	0	0	0	0	0	4.69	4.79	4.68	4.74	4.76	5.07	4.59
1374784_at	Prtfdc1	1	0	1	0	1	0	5.53	5.53	6.16	5.16	5.52	0	0	0	0	0	0	0	10.46	10.49	10.50	10.53	10.45	10.34	10.16

Probeset ID	Gene Symbol	Islet Data Set (log2 ratio ≥0.263; FDR <20%) union n=5325 1=YES 0=NO	Islet DRlyp/lyp ND vs ND+Lp299v (log2 ratio ≥0.263; FDR<20%) n=429 1=YES 0=NO	Islet DRlyp/lyp ND vs HCD (log2 ratio ≥0.263; FDR<20%) n=2247 1=YES 0=NO	Islet DRlyp/lyp ND vs HCD+Lp299v (log2 ratio ≥0.263; FDR<20%) n=1595 1=YES 0=NO	Islet DRlyp/lyp HCD vs HCD+Lp299v (log2 ratio ≥0.263; FDR<20%) n=3026 1=YES 0=NO	Islet DRlyp/lyp ND vs Flyp/lyp ND (log2 ratio ≥ 0.263; FDR<20%) n=1950 1=YES 0=NO	Islet Mean Log2 Intensity DRlyp/lyp ND	Islet Mean Log2 Intensity DRlyp/lyp ND+Lp299v	Islet Mean Log2 Intensity DRlyp/lyp HCD	Islet Mean Log2 Intensity DRlyp/lyp HCD+Lp299v	Islet Mean Log2 Intensity Flyp40 ND	Rinm5f data union of all conditions n=3705 1=YES 0=NO	Rinm5f 30 µM propionate/ 6 µM butyrate vs Media (FDR<20%; 1.5 fold) n=3 1=YES 0=NO	Rinm5f 60 µM propionate/ 12 µM butyrate vs Media (FDR<20%; 1.5 fold) n=18 1=YES 0=NO	Rinm5f 120 µM propionate/ 24 µM butyrate vs Media (FDR<20%; 1.5 fold) n=164 1=YES 0=NO	Rinm5f 240 µM propionate /48 µM butyrate vs Media (FDR<20%; 1.5 fold) n=268 1=YES 0=NO	Rinm5f 1500 µM propionate/ 300 µM butyrate vs Media (FDR<20%; 1.5 fold) n=2491 1=YES 0=NO	Rinm5f 500 µM propionate/ 500 µM butyrate vs Media (FDR<20%; 1.5 fold) n=3001 1=YES 0=NO	Rinm5f Mean Log2 Intensity Media only	Rinm5f Mean Log2 Intensity 30 µM propionate/ 6 µM butyrate	Rinm5f Mean Log2 Intensity 60 µM propionate/ 12 µM butyrate	Rinm5f Mean Log2 Intensity 120 µM propionate/ 24 µM butyrate	Rinm5f Mean Log2 Intensity 240 µM propionate/ 48 µM butyrate	Rinm5f Mean Log2 Intensity 1500 µM propionate /300 µM butyrate	Rinm5f Mean Log2 Intensity 500 µM propionate/ 500 µM butyrate
1374789_at	Dcun1d2	1	0	1	0	0	0	5.79	5.92	6.42	5.86	6.14	0	0	0	0	0	0	0	8.69	8.70	8.80	8.89	8.80	8.67	8.63
1374796_at	Sgcb	1	0	1	0	1	0	7.89	7.97	8.48	7.59	8.15	0	0	0	0	0	0	0	10.64	10.72	10.69	10.70	10.75	10.79	10.08
1374803_at	Znf207	1	0	1	0	0	1	6.46	6.23	7.37	6.70	7.05	0	0	0	0	0	0	0	8.42	8.42	8.62	8.78	8.78	8.42	7.63
1374806_at	Sfn	1	0	0	0	1	0	5.38	5.59	4.88	5.57	5.46	0	0	0	0	0	0	0	5.20	5.33	5.25	5.11	5.16	5.44	5.84
1374809_at	---	1	0	1	0	1	1	8.06	8.09	8.72	7.98	8.62	0	0	0	0	0	0	0	10.82	10.84	10.82	10.85	10.94	10.99	10.36
1374819_at	Tmem201	1	0	0	0	1	0	6.49	6.39	6.15	6.79	6.31	0	0	0	0	0	0	0	7.26	7.26	7.34	7.33	7.30	7.64	7.80
1374832_at	LOC102548682	1	0	1	0	0	1	5.76	5.50	5.21	5.34	6.34	0	0	0	0	0	0	0	4.32	4.37	4.45	4.20	4.08	4.23	3.79
1374837_at	Bcl7c	1	0	0	0	1	0	7.11	7.01	6.78	7.47	6.74	0	0	0	0	0	0	0	7.84	7.86	7.97	7.95	7.94	7.81	7.63
1374844_at	Rbm14	1	0	0	1	0	0	6.72	6.85	6.74	7.27	7.06	0	0	0	0	0	0	0	9.20	9.14	9.27	9.28	9.14	8.66	8.69
1374851_at	Med29	1	0	0	0	1	0	6.97	7.20	6.51	7.40	6.67	0	0	0	0	0	0	0	8.56	8.54	8.54	8.58	8.53	8.45	8.29
1374855_at	Per1	1	0	0	0	0	1	8.05	7.77	7.70	7.76	7.50	0	0	0	0	0	0	0	7.33	7.28	7.26	7.02	7.04	7.09	6.89
1374859_at	---	1	0	1	0	0	0	3.65	3.98	4.28	3.65	3.74	0	0	0	0	0	0	0	5.12	5.18	5.49	5.21	5.46	5.33	4.09
1374863_at	Rbp7	1	0	1	0	0	1	4.78	4.57	4.14	4.37	4.08	0	0	0	0	0	0	0	3.32	3.35	3.42	3.41	3.31	3.38	2.89
1374864_at	Spry2	1	0	1	0	0	0	5.04	4.66	5.71	5.20	4.85	0	0	0	0	0	0	0	6.12	6.08	6.08	5.96	5.89	5.83	5.96
1374868_at	Zfhx3	1	1	1	1	0	1	6.41	7.09	7.02	7.10	7.09	0	0	0	0	0	0	0	9.07	9.08	9.10	9.05	9.25	9.01	8.01
1374870_at	Col27a1	1	0	0	0	0	1	9.38	9.52	9.88	9.19	9.93	0	0	0	0	0	0	0	9.69	9.76	9.80	9.89	9.93	9.51	8.85
1374879_x_at	Larp4b	1	0	1	0	0	1	6.90	6.94	7.45	7.08	7.48	0	0	0	0	0	0	0	8.40	8.45	8.41	8.52	8.53	8.68	7.96
1374883_at	Mtmr7	1	0	1	0	1	0	7.71	7.79	8.38	7.34	8.05	0	0	0	0	0	0	0	10.37	10.37	10.43	10.55	10.50	10.45	10.13
1374884_at	Ppm1d	1	0	1	0	0	0	6.86	6.98	7.46	7.16	7.09	0	0	0	0	0	0	0	8.35	8.34	8.38	8.47	8.40	8.49	8.52
1374888_at	Cwc25	1	0	0	0	0	1	6.09	6.08	6.22	5.99	5.63	0	0	0	0	0	0	0	5.92	5.81	5.88	5.88	5.75	5.90	5.72
1374891_at	Brf2	1	0	0	0	0	1	4.45	4.59	4.84	4.39	4.94	0	0	0	0	0	0	0	7.53	7.51	7.59	7.63	7.59	7.34	6.82
1374898_at	Hid1	1	0	1	1	1	0	9.51	9.70	8.86	10.11	9.41	0	0	0	0	0	0	0	9.85	9.76	9.87	9.94	9.86	9.52	9.31
1374913_at	Setd3	1	0	0	1	1	0	7.12	6.93	7.51	6.59	6.90	0	0	0	0	0	0	0	9.61	9.66	9.70	9.69	9.67	9.69	9.06
1374914_at	Ppard	1	0	1	0	1	0	7.53	7.43	6.76	7.51	7.16	0	0	0	0	0	0	0	6.93	6.95	6.96	6.66	6.67	6.89	7.07
1374920_at	---	1	0	0	0	0	1	6.88	7.06	7.30	7.28	7.66	0	0	0	0	0	0	0	10.28	10.22	10.34	10.32	10.36	10.73	10.89
1374929_at	Ccdc126	1	0	1	0	1	0	6.56	6.68	7.20	6.25	6.82	0	0	0	0	0	0	0	8.67	8.64	8.71	8.74	8.77	8.82	8.47
1374935_at	Fsd1l	1	0	1	0	1	1	6.50	6.90	7.32	6.42	7.10	0	0	0	0	0	0	0	9.66	9.74	9.78	9.94	9.88	10.00	9.98
1374939_at	Cyfip2	1	0	0	0	0	1	6.39	6.54	6.91	6.44	7.10	0	0	0	0	0	0	0	7.98	8.02	8.08	7.98	8.08	8.01	8.43
1374947_at	Bcar3	1	0	0	0	0	1	5.40	5.03	5.68	5.53	4.83	0	0	0	0	0	0	0	3.80	3.69	3.76	3.78	3.67	3.96	4.21
1374954_at	Hdac11	1	0	0	1	1	0	7.26	7.33	7.09	7.81	7.04	0	0	0	0	0	0	0	7.25	7.27	7.26	7.34	7.42	7.51	7.13
1374956_at	Pcm1	1	0	1	0	0	0	7.76	7.82	8.38	7.81	7.94	0	0	0	0	0	0	0	10.75	10.77	10.76	10.81	10.78	10.55	10.13
1374959_at	Nqo2	1	0	0	1	0	1	5.45	5.49	5.95	5.98	7.31	0	0	0	0	0	0	0	3.82	3.76	3.72	3.68	3.71	3.78	3.32
1374966_at	Dcx	1	0	1	0	1	1	8.07	8.40	9.02	7.83	9.07	0	0	0	0	0	0	0	3.95	3.89	4.12	4.10	4.05	4.10	3.80
1374972_at	Tbc1d2	1	0	0	0	1	0	5.10	4.93	4.68	5.31	4.94	0	0	0	0	0	0	0	5.51	5.58	5.70	5.49	5.63	5.68	5.91
1374982_at	Ppp1r16a	1	0	0	0	1	0	5.26	5.11	4.93	5.49	5.10	0	0	0	0	0	0	0	7.33	7.41	7.45	7.28	7.31	7.30	7.10
1374987_at	Pank3	1	0	0	0	1	0	8.21	8.11	8.68	7.88	8.47	0	0	0	0	0	0	0	9.13	9.14	9.21	9.23	9.21	9.04	9.21
1374993_at	Rpusd1	1	0	0	0	0	1	6.20	6.18	5.93	5.95	5.71	0	0	0	0	0	0	0	7.10	6.98	7.04	6.98	6.95	6.98	6.90
1374996_at	Nploc4	1	0	0	0	0	1	6.56	6.53	6.71	6.68	7.17	0	0	0	0	0	0	0	9.37	9.30	9.41	9.45	9.32	9.14	9.09
1375001_at	Cytl1	1	0	1	0	0	1	5.52	5.62	4.99	5.45	4.94	0	0	0	0	0	0	0	4.03	4.06	3.93	3.87	3.86	3.98	3.43
1375005_at	---	1	0	1	0	1	0	5.65	5.86	6.27	5.18	5.84	0	0	0	0	0	0	0	5.09	5.32	5.57	5.58	5.61	5.21	5.24
1375006_at	Zc3hav1	1	0	0	0	0	1	7.97	8.10	8.52	8.08	8.68	0	0	0	0	0	0	0	9.54	9.49	9.54	9.59	9.44	9.15	9.19
1375023_at	Prr12	1	0	1	0	1	0	6.39	6.32	5.80	6.39	6.25	0	0	0	0	0	0	0	7.31	7.42	7.37	7.15	7.20	7.16	6.88
1375025_at	Camkk2	1	0	0	1	1	0	6.08	6.32	5.69	6.58	5.96	0	0	0	0	0	0	0	8.17	8.06	8.16	8.23	8.09	8.18	8.35
1375028_at	Tmem154	1	0	1	0	0	1	5.08	5.26	5.72	5.52	5.78	0	0	0	0	0	0	0	3.93	3.80	3.90	3.77	3.72	3.92	3.58
1375043_at	---	1	1	1	1	1	0	11.40	12.31	12.06	10.47	11.28	0	0	0	0	0	0	0	9.16	9.03	9.10	8.65	8.74	9.04	8.26
1375049_at	Ltbp3	1	0	0	0	1	0	7.46	7.40	7.19	7.78	7.26	0	0	0	0	0	0	0	10.33	10.26	10.26	10.18	10.19	9.83	9.64
1375056_at	Ociad2	1	0	1	0	1	1	9.91	10.04	10.74	9.84	10.43	0	0	0	0	0	0	0	10.19	10.25	10.24	10.34	10.38	10.16	9.44
1375067_at	---	1	0	0	1	0	0	6.83	6.64	6.90	7.31	6.77	0	0	0	0	0	0	0	8.31	8.40	8.39	8.30	8.38	8.26	8.18
1375072_at	Tmem63b	1	0	1	0	1	1	7.77	7.74	6.85	7.99	7.06	0	0	0	0	0	0	0	8.77	8.80	8.87	8.78	8.86	8.82	9.06
1375073_at	Samd11	1	0	0	1	0	0	3.38	3.76	3.79	3.86	3.60	0	0	0	0	0	0	0	5.18	5.05	5.24	5.04	5.06	4.96	5.05
1375080_at	Slc25a45	1	0	1	0	1	0	8.54	8.29	7.92	9.00	8.23	0	0	0	0	0	0	0	5.42	5.46	5.60	5.60	5.65	5.84	5.69
1375083_at	C9orf152	1	0	1	1	1	1	7.38	7.56	6.52	8.32	6.90	0	0	0	0	0	0	0	4.92	5.00	4.90	4.81	4.83	4.94	4.94
1375085_at	Slc25a35	1	0	1	1	0	0	5.48	5.09	6.75	6.18	5.14	0	0	0	0	0	0	0	4.10	4.03	4.02	3.84	3.91	3.91	3.86
1375092_at	Myo18b	1	0	1	0	0	0	3.90	3.74	3.35	3.66	3.71	0	0	0	0	0	0	0	4.89	4.86	4.92	4.76	4.85	5.02	5.05
1375104_at	---	1	0	0	1	0	0	5.49	5.64	5.84	5.98	5.96	0	0	0	0	0	0	0	7.43	7.38	7.44	7.55	7.65	7.34	6.72
1375112_at	Nr2f6	1	0	0	0	0	1	5.00	4.73	4.52	4.64	4.53	0	0	0	0	0	0	0	5.12	5.04	5.09	5.01	4.92	4.97	4.88
1375122_at	Yaf2	1	0	1	0	0	0	3.61	3.61	4.19	3.54	3.83	0	0	0	0	0	0	0	7.66	7.63	7.68	7.85	7.84	7.87	7.65
1375127_at	Cox5a	1	0	0	0	1	0	7.46	7.42	7.20	7.79	7.42	0	0	0	0	0	0	0	8.09	7.90	7.99	8.02	8.12	8.12	7.70
1375130_at	Zfand5	1	0	0	1	0	0	6.66	6.73	6.79	6.17	6.65	0	0	0	0	0	0	0	8.56	8.57	8.71	8.74	8.60	8.70	7.78
1375135_at	Gcn1	1	0	0	1	1	0	6.76	6.58	6.39	7.39	6.93	0	0	0	0	0	0	0	8.33	8.27	8.41	8.25	8.31	8.03	7.83
1375137_at	Arpc2	1	0	0	1	1	0	8.11	7.89	8.62	7.28	8.10	0	0	0	0	0	0	0	9.47	9.40	9.42	9.42	9.33	9.15	8.60
1375140_at	Mbnl2	1	0	0	1	1	0	7.33	7.25	7.81	6.56	7.20	0	0	0	0	0	0	0	8.97	9.07	9.18	9.32	9.16	9.33	8.71
1375146_at	Slc25a39	1	0	0	0	1	0	9.87	9.84	9.45	10.33	9.60	0	0	0	0	0	0	0	11.78	11.80	11.80	11.79	11.79	11.86	11.56
1375173_at	Plbd2	1	0	0	1	1	0	8.72	8.90	8.53	9.31	8.55	0	0	0	0	0	0	0	10.97	10.98	10.97	10.86	10.84	10.73	10.63
1375181_at	Rpl12	1	0	0	1	0	0	12.47	12.45	12.86	12.94	12.34	0	0	0	0	0	0	0	12.40	12.38	12.39	12.42	12.38	12.30	12.06
1375182_at	---	1	0	0	0	1	1	8.49	8.48	9.01	8.05	8.97	0	0	0	0	0	0	0	8.84	8.84	8.93	9.03	9.02	8.91	8.47
1375189_at	Edf1	1	0	0	0	1	0	10.28	10.42	9.95	10.71	9.94	0	0	0	0	0	0	0	11.52	11.56	11.53	11.53	11.52	11.52	11.33
1375193_at	Lrp11	1	0	1	1	1	0	5.91	6.17	7.02	5.12	6.05	0	0	0	0	0	0	0	9.53	9.60	9.55	9.59	9.58	9.78	8.97
1375197_at	Uqcr11	1	0	0	0	1	0	9.25	9.40	9.03	9.63	9.22	0	0	0	0	0	0	0	11.89	11.85	11.86	11.79	11.82	11.98	11.79
1375199_at	Igf1r	1	0	0	0	0	1	6.26	6.23	6.64	6.24	7.25	0	0	0	0	0	0	0	8.07	7.93	8.12	8.28	8.18	8.20	8.04
1375221_at	Tmx4	1	0	1	0	1	0	8.95	8.95	9.57	8.57	9.15	0	0	0	0	0	0	0	11.82	11.87	11.84	11.82	11.81	11.64	11.38
1375246_at	Tma7	1	0	0	0	0	1	10.42	10.51	10.78	10.55	10.92	0	0	0	0	0	0	0	11.62	11.62	11.57	11.58	11.57	11.45	11.09
1375254_at	Slc35a1	1	0	0	0	1	0	7.04	7.03	7.45	6.62	6.91	0	0	0	0	0	0	0	8.92	8.99	9.18	9.26	9.16	8.83	8.01
1375266_at	---	1	0	0	0	0	1	10.94	10.99	11.27	11.07	11.45	0	0	0	0	0	0	0	3.55	3.52	3.56	3.55	3.57	3.64	3.40
1375268_at	Rps27l	1	0	0	0	1	0	10.10	9.77	9.60	10.26	9.70	0	0	0	0	0	0	0	9.33	9.30	9.34	9.44	9.35	9.37	8.64
1375270_at	Kiaa1671	1	0	0	0	1	0	5.96	5.97	5.57	6.25	6.30	0	0	0	0	0	0	0	6.87	6.85	7.02	6.88	6.95	7.36	7.89
1375271_at	Nek9	1	0	0	1	0	0	4.27	4.14	4.46	3.79	4.14	0	0	0	0	0	0	0	6.88	6.74	7.05	7.03	6.89	6.92	5.84
1375275_at	---	1	0	1	1	0	0	7.45	7.29	6.80	6.95	7.53	0	0	0	0	0	0	0	5.15	5.15	5.08	4.76	4.73	4.76	4.53
1375334_at	Hm13	1	0	1	0	1	0	6.81	6.83	6.22	7.18	6.55	0	0	0	0	0	0	0	6.78	6.75	6.78	6.90	6.94	6.69	6.45
1375342_at	Nfic	1	0	0	0	1	0	7.27	7.55	6.98	7.60	7.28	0	0	0	0	0	0	0	7.75	7.76	7.76	7.62	7.54	7.54	7.00
1375345_at	Cnpy2	1	0	0	0	0	1	10.27	10.01	10.34	10.41	9.74	0	0	0	0	0	0	0	11.74	11.82	11.82	11.81	11.77	11.60	10.98
1375346_at	C11orf71	1	0	0	1	0	0	7.42	7.71	7.38	7.92	7.24	0	0	0	0	0	0	0	8.39	8.42	8.39	8.40	8.37	8.37	8.68
1375356_at	Misp3	1	0	1	0	0	0	6.14	5.90	5.58	5.78	5.88	0	0	0	0	0	0	0	7.42	7.42	7.39	7.17	7.24	6.95	6.38
1375363_at	Asxl2	1	0	0	0	1	0	7.24	7.07	7.73	7.01	7.18	0	0	0	0	0	0	0	9.33	9.35	9.38	9.35	9.42	9.23	8.78
1375364_at	Stk11	1	0	0	0	0	1	4.97	4.81	4.95	4.73	4.03	0	0	0	0	0	0	0	6.68	6.79	6.68	6.85	6.77	6.87	5.71
1375367_at	Pdlim2	1	0	1	0	0	1	5.09	5.09	4.57	4.89	4.60	0	0	0	0	0	0	0	5.32	5.56	5.60	5.76	5.91	5.74	5.97
1375369_at	Wbp1l	1	0	0	1	1	0	8.64	8.68	8.73	9.29	8.58	0	0	0	0	0	0	0	9.87	9.84	9.88	9.75	9.75	9.71	9.74
1375371_at	Ranbp2	1	0	0	0	1	0	6.95	7.11	7.41	6.55	7.06	0	0	0	0	0	0	0	8.27	8.20	8.43	8.54	8.51	8.30	7.69
1375382_at	Mdc1	1	0	0	0	1	0	5.33	5.55	4.98	5.78	5.61	0	0	0	0	0	0	0	8.56	8.34	8.54	8.45	8.44	8.41	8.63
1375393_at	---	1	0	0	0	1	0	7.19	7.28	6.92	7.53	7.13	0	0	0	0	0	0	0	6.98	7.12	7.24	7.05	7.03	6.99	7.06
1375419_at	Lrrc28	1	0	0	0	0	1	4.73	4.69	4.55	5.07	5.24	0	0	0	0	0	0	0	6.37	6.45	6.43	6.34	6.44	6.70	6.29
1375421_a_at	Pja2	1	0	1	0	1	1	9.07	9.29	9.85	9.10	9.63	0	0	0	0	0	0	0	9.65	9.55	9.57	9.55	9.49	9.70	9.98
1375422_at	---	1	0	0	0	0	1	10.23	10.13	10.31	10.67	8.95	0	0	0	0	0	0	0	10.05	9.91	9.90	9.91	9.80	9.80	9.82
1375424_at	Actr2	1	0	0	0	0	1	8.80	8.65	9.27	8.68	9.33	0	0	0	0	0	0	0	11.92	11.97	11.98	11.98	11.96	11.90	11.70
1375426_a_at	Khsrp	1	0	0	1	1	0	8.37	8.63	8.11	8.99	8.09	0	0	0	0	0	0	0	9.47	9.41	9.43	9.38	9.40	9.31	9.10
1375428_at	Creg1	1	0	0	0	0	1	7.10	6.74	6.70	7.04	6.30	0	0	0	0	0	0	0	6.61	6.47	6.62	6.44	6.45	6.82	6.50
1375433_at	Disp2	1	0	0	0	0	1	8.29	8.29	8.70	8.15	8.92	0	0	0	0	0	0	0	10.29	10.26	10.39	10.36	10.45	10.12	10.00
1375439_at	Wdr18	1	0	0	0	1	0	6.69	6.82	6.39	6.99	6.50	0	0	0	0	0	0	0	7.93	7.96	8.03	8.02	7.89	7.87	7.72
1375441_at	Sars1	1	0	0	0	1	0	9.22	9.23	9.02	9.66	8.82	0	0	0	0	0	0	0	10.27	10.26	10.33	10.38	10.31	10.22	10.15
1375462_at	---	1	0	1	0	0	0	5.73	5.67	5.17	5.72	5.57	0	0	0	0	0	0	0	5.22	5.29	5.21	4.95	5.05	5.18	4.68
1375463_at	Ski	1	0	0	1	1	0	7.72	7.62	7.45	8.33	7.60	0	0	0	0	0	0	0	6.79	6.62	6.79	6.64	6.66	6.77	7.34
1375468_at	Abcc5	1	0	1	1	1	0	4.79	4.74	5.61	3.78	4.63	0	0	0	0	0	0	0	6.57	6.40	6.68	6.80	6.66	6.72	6.41
1375469_at	Smarca4	1	1	0	1	1	0	7.89	8.50	7.76	8.53	8.01	0	0	0	0	0	0	0	10.05	9.89	10.07	9.95	9.83	9.76	10.17
1375508_at	Kif2a	1	0	0	0	0	1	4.44	4.75	4.67	4.78	4.91	0	0	0	0	0	0	0	5.92	5.82	6.04	5.94	5.96	5.88	5.10
1375519_at	Hba1 /// Hba2	1	0	1	1	0	1	8.25	7.81	7.60	7.65	7.24	0	0	0	0	0	0	0	6.49	6.59	6.42	6.38	6.40	6.75	6.29
1375520_at	Copz1	1	0	0	1	1	0	9.87	10.18	9.50	10.39	9.84	0	0	0	0	0	0	0	10.94	10.95	10.98	10.88	10.84	10.83	10.83
1375523_at	Marcks	1	0	0	0	0	1	8.57	8.65	8.93	8.66	9.39	0	0	0	0	0	0	0	9.18	9.10	9.30	9.39	9.27	9.18	9.06
1375529_at	Cbr4	1	1	0	0	0	1	6.58	6.04	6.52	6.69	5.54	0	0	0	0	0	0	0	8.33	8.40	8.39	8.45	8.45	8.69	8.41
1375534_at	Pum2	1	0	1	0	0	0	6.78	6.92	7.48	7.10	7.24	0	0	0	0	0	0	0	10.07	10.09	10.15	10.14	10.13	10.21	10.29
1375535_at	LOC100911498	1	1	1	1	0	1	9.48	10.62	10.29	10.76	10.30	0	0	0	0	0	0	0	4.74	4.66	4.57	4.42	4.56	4.56	4.12
1375538_at	Vcl	1	0	1	1	1	1	6.40	6.64	7.29	5.78	7.02	0	0	0	0	0	0	0	8.08	7.95	8.06	7.98	7.82	8.01	7.74
1375540_at	Cdkn2aipnl	1	0	1	1	1	0	6.71	6.56	7.30	6.21	6.71	0	0	0	0	0	0	0	7.96	7.97	8.06	8.09	8.08	8.32	7.82
1375548_at	Efcab14	1	0	0	0	0	1	8.37	8.72	8.81	8.56	8.88	0	0	0	0	0	0	0	9.07	9.04	9.05	8.99	9.04	9.40	9.47
1375550_at	Bag1	1	0	0	0	1	0	8.43	8.34	8.02	8.66	8.07	0	0	0	0	0	0	0	9.98	9.97	9.97	10.00	10.01	10.30	10.44
1375563_at	Ubtd2	1	0	1	0	1	0	6.63	6.64	7.51	6.42	6.92	0	0	0	0	0	0	0	7.20	7.18	7.22	7.30	7.20	7.42	7.37
1375566_at	Agtrap	1	0	0	0	1	0	6.73	6.82	6.54	7.11	6.37	0	0	0	0	0	0	0	7.80	7.86	7.86	7.78	7.85	7.95	7.40
1375590_at	Zc3h11a	1	0	1	0	0	0	7.54	7.70	8.01	7.67	7.76	0	0	0	0	0	0	0	9.62	9.50	9.69	9.64	9.61	9.53	9.47
1375595_at	Arih1	1	0	0	1	0	0	5.44	5.22	5.49	4.90	5.30	0	0	0	0	0	0	0	6.79	6.74	6.81	6.97	6.97	6.81	6.24
1375613_at	Gata6	1	0	1	0	0	1	6.17	5.87	5.41	5.79	5.68	0	0	0	0	0	0	0	5.49	5.55	5.44	5.32	5.30	5.30	5.20
1375622_at	Rbsn	1	0	0	1	0	0	3.95	4.29	4.28	4.40	4.13	0	0	0	0	0	0	0	6.63	6.62	6.55	6.78	6.61	6.56	6.41
1375627_at	Akirin2	1	0	0	1	1	0	6.66	6.51	7.03	6.05	6.36	0	0	0	0	0	0	0	9.26	9.29	9.33	9.36	9.26	9.06	8.49
1375629_at	LAMP1	1	0	1	0	1	0	5.71	5.69	6.37	5.26	6.19	0	0	0	0	0	0	0	8.49	8.45	8.47	8.56	8.72	8.51	7.33
1375630_at	Snu13	1	0	0	0	1	0	8.80	8.62	9.28	8.50	8.82	0	0	0	0	0	0	0	11.58	11.58	11.62	11.64	11.62	11.62	11.24
1375631_at	Plekhb2	1	0	0	0	1	0	9.01	8.95	9.54	8.75	9.15	0	0	0	0	0	0	0	12.14	12.14	12.19	12.14	12.19	12.25	12.08
1375638_at	Cavin2	1	0	0	0	0	1	7.80	7.43	7.53	7.35	7.28	0	0	0	0	0	0	0	3.52	3.54	3.45	3.55	3.45	3.46	2.95
1375642_at	Ube2b	1	0	1	0	1	0	8.62	8.59	9.25	8.44	8.84	0	0	0	0	0	0	0	9.61	9.65	9.71	9.79	9.80	10.04	9.71
1375647_at	Efna5	1	0	0	0	0	1	6.76	6.84	7.23	6.77	7.32	0	0	0	0	0	0	0	6.77	6.90	6.80	6.74	6.76	7.06	7.27
1375655_at	Creb1	1	0	0	0	0	1	6.26	6.31	6.61	6.35	7.03	0	0	0	0	0	0	0	6.41	6.38	6.44	6.50	6.45	6.62	6.59
1375670_at	---	1	0	1	0	0	0	5.82	5.64	5.25	5.59	5.69	0	0	0	0	0	0	0	4.93	4.88	4.85	4.71	4.79	4.87	4.38
1375676_at	---	1	0	1	1	1	0	7.25	7.21	8.17	6.39	7.22	0	0	0	0	0	0	0	10.03	9.89	10.02	10.16	10.05	9.91	8.96
1375685_at	Clasp1	1	0	0	0	0	1	6.69	6.51	6.82	6.60	7.22	0	0	0	0	0	0	0	8.86	8.87	8.92	8.92	8.97	8.82	8.97
1375689_at	---	1	0	1	1	0	0	8.26	8.30	8.89	8.68	8.46	0	0	0	0	0	0	0	9.94	9.88	10.01	9.95	9.92	9.90	9.49
1375692_at	Mapk1	1	0	1	0	0	0	8.08	8.11	8.68	8.02	8.40	0	0	0	0	0	0	0	10.17	10.17	10.23	10.22	10.25	10.42	10.22
1375697_at	Mlec	1	0	0	1	1	1	7.63	7.69	7.34	8.28	7.01	0	0	0	0	0	0	0	8.43	8.35	8.47	8.35	8.32	8.10	7.71
1375703_at	Kmt2e	1	0	0	1	0	0	6.35	6.43	6.55	6.99	6.75	0	0	0	0	0	0	0	7.46	7.44	7.63	7.50	7.45	7.24	8.06

Probeset ID	Gene Symbol	Islet Data Set (log2 ratio ≥0.263; FDR <20%) union n=5325 1=YES 0=NO	Islet DRlyp/lyp ND vs ND+Lp299v (log2 ratio ≥0.263; FDR<20%) n=429 1=YES 0=NO	Islet DRlyp/lyp ND vs HCD (log2 ratio ≥0.263; FDR<20%) n=2247 1=YES 0=NO	Islet DRlyp/lyp ND vs HCD+Lp299v (log2 ratio ≥0.263; FDR<20%) n=1595 1=YES 0=NO	Islet DRlyp/lyp HCD vs HCD+Lp299v (log2 ratio ≥0.263; FDR<20%) n=3026 1=YES 0=NO	Islet DRlyp/lyp ND vs Flyp/lyp ND (log2 ratio ≥ 0.263; FDR<20%) n=1950 1=YES 0=NO	Islet Mean Log2 Intensity DRlyp/lyp ND	Islet Mean Log2 Intensity DRlyp/lyp ND+Lp299v	Islet Mean Log2 Intensity DRlyp/lyp HCD	Islet Mean Log2 Intensity DRlyp/lyp HCD+Lp299v	Islet Mean Log2 Intensity Flyp40 ND	Rinm5f data union of all conditions n=3705 1=YES 0=NO	Rinm5f 30 µM propionate/ 6 µM butyrate vs Media (FDR<20%; 1.5 fold) n=3 1=YES 0=NO	Rinm5f 60 µM propionate/ 12 µM butyrate vs Media (FDR<20%; 1.5 fold) n=18 1=YES 0=NO	Rinm5f 120 µM propionate/ 24 µM butyrate vs Media (FDR<20%; 1.5 fold) n=164 1=YES 0=NO	Rinm5f 240 µM propionate /48 µM butyrate vs Media (FDR<20%; 1.5 fold) n=268 1=YES 0=NO	Rinm5f 1500 µM propionate/ 300 µM butyrate vs Media (FDR<20%; 1.5 fold) n=2491 1=YES 0=NO	Rinm5f 500 µM propionate/ 500 µM butyrate vs Media (FDR<20%; 1.5 fold) n=3001 1=YES 0=NO	Rinm5f Mean Log2 Intensity Media only	Rinm5f Mean Log2 Intensity 30 µM propionate/ 6 µM butyrate	Rinm5f Mean Log2 Intensity 60 µM propionate/ 12 µM butyrate	Rinm5f Mean Log2 Intensity 120 µM propionate/ 24 µM butyrate	Rinm5f Mean Log2 Intensity 240 µM propionate/ 48 µM butyrate	Rinm5f Mean Log2 Intensity 1500 µM propionate /300 µM butyrate	Rinm5f Mean Log2 Intensity 500 µM propionate/ 500 µM butyrate
1375705_at	Gnb1	1	0	1	1	1	0	7.72	7.59	8.41	6.97	7.99	0	0	0	0	0	0	0	10.17	10.10	10.28	10.31	10.29	10.26	9.61
1375706_at	LOC100361913 /	1	0	1	0	1	1	5.93	5.82	5.30	5.94	5.36	0	0	0	0	0	0	0	5.90	5.77	6.04	5.71	5.84	5.86	5.75
1375707_at	---	1	0	1	0	1	0	6.75	6.79	5.99	6.85	6.71	0	0	0	0	0	0	0	7.01	7.02	7.19	7.25	7.28	7.53	7.66
1375708_at	Col27a1	1	0	0	0	0	1	8.32	8.39	8.44	8.18	8.90	0	0	0	0	0	0	0	8.44	8.43	8.58	8.55	8.55	8.24	7.27
1375716_at	Ifngr2	1	0	0	1	0	0	5.46	5.12	5.19	4.91	5.36	0	0	0	0	0	0	0	8.90	8.89	8.90	8.76	8.72	8.84	7.92
1375719_s_at	Cdh13	1	0	1	0	0	1	6.48	6.32	5.91	6.42	5.92	0	0	0	0	0	0	0	4.91	4.84	4.89	4.82	4.84	4.94	4.79
1375721_at	---	1	0	0	1	1	0	6.00	5.76	5.98	6.74	6.20	0	0	0	0	0	0	0	6.86	6.60	6.84	6.85	6.65	6.47	6.92
1375733_at	---	1	0	0	0	0	1	6.75	6.69	6.99	7.20	7.28	0	0	0	0	0	0	0	6.02	6.05	6.10	5.99	5.80	5.69	5.45
1375740_at	---	1	1	1	0	1	1	6.66	6.11	6.05	6.69	6.15	0	0	0	0	0	0	0	4.88	4.92	4.79	4.96	4.77	4.95	4.32
1375745_at	---	1	0	0	0	1	0	9.03	8.93	9.61	8.82	9.49	0	0	0	0	0	0	0	11.02	11.01	11.05	11.05	11.02	10.76	10.55
1375749_at	Exoc1	1	0	0	1	1	0	5.14	5.09	5.37	4.54	4.94	0	0	0	0	0	0	0	7.56	7.56	7.72	7.65	7.58	7.59	6.99
1375773_at	LOC100912503	1	0	0	0	1	0	4.96	5.03	5.43	4.48	4.81	0	0	0	0	0	0	0	6.78	6.77	6.87	6.93	6.86	6.87	6.35
1375799_at	Tmem121	1	0	0	1	0	0	4.40	4.86	4.62	5.14	4.46	0	0	0	0	0	0	0	8.15	8.20	8.25	8.28	8.31	8.31	7.86
1375815_at	---	1	0	0	0	0	1	5.05	5.33	5.14	5.07	6.69	0	0	0	0	0	0	0	6.08	6.16	6.34	6.30	6.22	6.54	7.20
1375818_at	Lactb	1	0	0	1	0	0	5.44	5.27	5.20	4.95	5.07	0	0	0	0	0	0	0	6.03	6.10	6.03	6.10	5.98	6.08	5.66
1375824_at	---	1	0	0	1	1	0	6.53	6.64	6.50	7.18	6.66	0	0	0	0	0	0	0	6.84	6.76	6.84	6.60	6.67	6.83	6.56
1375829_at	Dennd10	1	0	0	1	1	0	5.54	5.65	5.90	4.97	5.57	0	0	0	0	0	0	0	8.36	8.35	8.37	8.46	8.43	8.35	7.92
1375831_at	Rbmxl2	1	0	1	0	0	0	4.67	4.81	4.06	4.53	4.24	0	0	0	0	0	0	0	4.03	4.09	4.11	3.87	3.86	3.95	3.71
1375843_at	Ids	1	0	1	0	1	0	10.26	10.39	11.04	10.24	10.54	0	0	0	0	0	0	0	11.91	11.92	11.87	11.82	11.84	11.80	11.34
1375845_at	Aig1	1	0	1	0	0	0	7.72	8.06	8.32	7.99	8.13	0	0	0	0	0	0	0	10.67	10.70	10.67	10.61	10.60	10.35	10.30
1375847_at	Nudt16	1	0	1	0	0	0	7.68	7.52	7.05	7.58	7.31	0	0	0	0	0	0	0	6.36	6.21	6.21	6.04	6.02	6.16	5.79
1375852_at	Hmgcr	1	0	0	0	1	0	8.16	8.02	8.58	7.71	8.42	0	0	0	0	0	0	0	10.51	10.52	10.58	10.63	10.65	10.22	9.76
1375853_at	Washc4	1	0	1	0	1	0	7.51	7.37	8.18	7.32	7.64	0	0	0	0	0	0	0	9.92	9.93	9.96	10.10	10.01	9.94	9.63
1375860_at	---	1	0	0	1	0	0	5.49	5.23	5.56	4.95	5.41	0	0	0	0	0	0	0	7.87	7.82	7.88	7.93	7.97	7.75	6.79
1375861_at	Nap1l5	1	0	1	0	1	1	7.84	8.22	8.79	7.50	8.47	0	0	0	0	0	0	0	4.38	4.32	4.29	4.21	4.11	4.36	3.80
1375862_at	Pxdn	1	1	0	0	0	0	5.87	5.35	5.41	5.56	5.48	0	0	0	0	0	0	0	5.43	5.43	5.57	5.69	5.42	5.57	5.74
1375866_at	---	1	0	0	1	0	0	6.21	6.32	6.25	6.79	6.32	0	0	0	0	0	0	0	7.18	7.14	7.28	7.10	7.09	6.90	7.11
1375868_at	---	1	0	0	1	1	0	4.77	4.91	4.60	5.75	4.77	0	0	0	0	0	0	0	7.32	7.39	7.63	7.67	7.69	7.90	7.31
1375869_at	Ulk1	1	0	0	1	1	0	6.51	6.32	6.33	6.99	6.57	0	0	0	0	0	0	0	7.81	7.78	7.80	7.69	7.74	8.09	8.48
1375870_a_at	Rbms1	1	0	0	0	0	1	6.20	6.39	6.71	6.02	6.72	0	0	0	0	0	0	0	7.51	7.68	7.60	7.75	7.80	7.70	7.29
1375879_at	---	1	0	1	0	1	0	8.00	7.90	8.63	7.93	8.34	0	0	0	0	0	0	0	8.17	8.12	8.21	8.25	8.20	8.62	8.71
1375880_at	Appbp2	1	0	1	0	1	0	6.46	6.16	7.09	6.34	6.84	0	0	0	0	0	0	0	8.71	8.79	8.88	8.89	8.85	8.81	8.79
1375881_at	Dstn /// Dstnl1	1	0	0	1	1	0	10.61	10.51	11.15	9.85	10.62	0	0	0	0	0	0	0	12.25	12.25	12.24	12.24	12.22	12.27	11.96
1375882_at	Ppp3cb	1	0	1	0	0	0	7.69	7.79	8.28	7.73	7.95	0	0	0	0	0	0	0	9.36	9.44	9.50	9.51	9.49	9.44	9.60
1375883_at	Tut7	1	0	0	1	1	0	5.61	5.74	5.43	6.09	5.72	0	0	0	0	0	0	0	5.98	5.97	6.02	6.07	5.90	5.88	6.20
1375884_at	Msantd4	1	0	1	0	1	0	7.92	7.80	8.57	7.73	8.12	0	0	0	0	0	0	0	8.92	8.96	9.00	9.16	9.17	9.38	8.99
1375887_at	LOC100910807 /	1	0	1	0	0	0	6.79	6.84	7.49	6.82	7.20	0	0	0	0	0	0	0	8.98	9.05	8.99	9.08	9.02	8.71	8.51
1375898_at	Rbpms	1	0	0	0	1	0	7.53	7.55	7.90	7.11	7.84	0	0	0	0	0	0	0	8.42	8.45	8.47	8.42	8.51	8.44	8.79
1375903_a_at	Yaf2	1	0	1	0	1	0	4.34	4.65	5.21	4.27	4.44	0	0	0	0	0	0	0	9.52	9.54	9.62	9.68	9.64	9.64	9.03
1375908_at	Mpzl2	1	0	0	1	1	1	7.31	7.01	7.07	7.81	6.56	0	0	0	0	0	0	0	3.96	3.97	4.07	3.89	3.99	4.02	3.58
1375915_at	Irak1bp1	1	0	1	0	1	0	7.09	7.23	7.78	6.99	7.02	0	0	0	0	0	0	0	8.49	8.48	8.42	8.55	8.52	8.28	8.16
1375928_at	Kmt5a	1	0	0	0	1	0	7.80	7.74	7.31	8.00	7.65	0	0	0	0	0	0	0	9.01	9.03	9.08	9.04	9.01	8.76	8.74
1375958_at	Tsc22d4	1	0	0	0	1	0	7.06	7.25	6.58	7.30	6.99	0	0	0	0	0	0	0	8.30	8.29	8.36	8.15	8.16	8.20	8.55
1375961_at	Frzb	1	0	0	0	0	1	7.76	7.95	8.25	7.70	6.65	0	0	0	0	0	0	0	4.07	4.01	3.94	4.02	4.00	4.21	3.65
1375967_a_at	Dusp22	1	0	0	0	1	0	7.76	7.63	7.50	8.08	7.52	0	0	0	0	0	0	0	8.41	8.41	8.48	8.52	8.48	8.68	9.01
1375969_at	Tor1aip2	1	0	1	0	1	0	5.14	5.15	5.69	4.71	5.01	0	0	0	0	0	0	0	5.54	5.47	5.59	5.52	5.49	5.52	5.51
1375974_at	G3bp2	1	0	1	0	1	0	8.74	8.77	9.39	8.67	8.93	0	0	0	0	0	0	0	10.42	10.38	10.42	10.48	10.48	10.41	10.33
1375980_at	Nxn	1	0	1	0	1	1	6.99	6.91	6.33	7.38	6.50	0	0	0	0	0	0	0	7.99	7.96	8.04	7.95	8.00	8.31	8.10
1375990_a_at	Ppp2r3a	1	0	0	1	0	0	6.29	6.19	6.27	6.81	6.32	0	0	0	0	0	0	0	5.28	5.26	5.33	5.40	5.33	5.30	6.04
1375995_at	C2cd5	1	0	1	0	0	1	7.77	7.88	8.36	7.94	8.31	0	0	0	0	0	0	0	7.98	7.99	8.04	8.01	8.05	7.97	7.85
1375997_at	Dglucy	1	0	0	0	1	0	5.93	5.79	5.43	6.18	5.54	0	0	0	0	0	0	0	4.63	4.53	4.30	4.37	4.37	4.56	4.64
1376029_at	---	1	1	0	0	0	0	7.10	7.71	7.16	7.15	7.27	0	0	0	0	0	0	0	10.48	10.52	10.57	10.57	10.67	10.83	9.99
1376034_at	Tcaf1	1	0	1	0	1	0	6.11	6.42	6.74	5.68	6.43	0	0	0	0	0	0	0	9.18	9.13	9.18	9.27	9.20	9.21	8.96
1376047_at	Papss2	1	0	1	0	0	1	9.26	9.68	9.87	9.22	9.86	0	0	0	0	0	0	0	7.76	7.76	7.81	7.82	7.87	8.23	8.13
1376058_at	Mtus1	1	0	0	0	0	1	7.21	7.14	7.31	7.00	6.65	0	0	0	0	0	0	0	8.84	8.84	8.95	8.95	8.99	9.13	8.53
1376059_at	Gigyf2	1	0	0	0	0	1	6.57	6.42	6.54	6.29	5.92	0	0	0	0	0	0	0	6.69	6.65	6.81	6.79	6.81	6.75	6.50
1376062_at	Sdc1	1	0	1	0	1	0	4.78	4.85	4.21	5.24	4.73	0	0	0	0	0	0	0	7.87	7.98	8.14	8.19	8.25	8.31	8.35
1376065_at	Rrs1	1	0	0	1	0	0	6.95	6.73	7.03	7.44	6.78	0	0	0	0	0	0	0	8.09	7.94	8.03	8.17	8.03	7.79	7.25
1376073_at	Sel1l	1	0	0	1	1	1	9.98	9.91	9.73	10.63	9.41	0	0	0	0	0	0	0	8.38	8.31	8.45	8.42	8.35	8.45	8.29
1376080_at	Rcor1	1	0	0	1	1	0	6.68	6.73	6.48	7.33	6.64	0	0	0	0	0	0	0	7.37	7.31	7.45	7.37	7.44	7.73	7.89
1376087_at	Retreg3	1	0	0	0	1	0	6.12	5.79	5.76	6.43	5.67	0	0	0	0	0	0	0	8.57	8.58	8.70	8.40	8.60	8.33	8.72
1376102_at	Tmbim1	1	0	1	0	1	1	6.42	6.05	5.76	6.55	5.85	0	0	0	0	0	0	0	3.61	3.52	3.53	3.36	3.39	3.35	3.03
1376103_at	Erc1	1	0	0	0	0	1	6.03	6.08	6.21	6.16	6.53	0	0	0	0	0	0	0	6.52	6.43	6.55	6.49	6.52	6.63	6.68
1376104_at	Mtcl1	1	0	1	0	0	1	5.81	6.02	6.44	5.98	6.50	0	0	0	0	0	0	0	7.27	7.27	7.38	7.34	7.46	7.62	7.93
1376105_at	Col14a1	1	0	0	1	0	1	7.11	6.90	6.90	6.51	8.15	0	0	0	0	0	0	0	4.31	4.27	4.42	4.17	4.24	4.38	4.30
1376117_at	Slc44a4	1	0	1	0	1	1	7.06	6.77	6.31	6.99	6.41	0	0	0	0	0	0	0	8.42	8.51	8.44	8.54	8.70	8.87	8.92
1376121_at	---	1	1	1	0	1	0	6.52	5.99	7.14	6.05	6.57	0	0	0	0	0	0	0	7.98	7.91	8.09	8.26	8.27	7.98	7.51
1376128_at	---	1	0	1	1	1	0	7.49	7.13	6.59	8.07	7.13	0	0	0	0	0	0	0	4.37	4.46	4.27	4.17	4.40	4.52	4.42
1376130_a_at	Dtnb	1	0	1	0	1	1	8.68	8.64	7.87	8.94	8.16	0	0	0	0	0	0	0	7.13	7.05	7.26	7.07	7.04	7.17	7.60
1376131_a_at	Pelp1	1	0	0	0	1	0	6.64	6.82	6.16	6.80	6.51	0	0	0	0	0	0	0	7.35	7.29	7.33	7.28	7.19	7.00	6.95
1376137_at	Plekhb2	1	0	1	1	1	0	5.65	5.70	6.73	4.55	5.33	0	0	0	0	0	0	0	10.73	10.82	10.82	10.87	10.89	11.00	9.44
1376140_at	Abcd1	1	0	1	0	0	0	6.72	6.75	6.15	6.70	6.58	0	0	0	0	0	0	0	7.69	7.72	7.53	7.53	7.50	7.54	7.17
1376151_a_at	Irgm	1	1	0	0	0	0	7.11	8.00	7.54	7.33	7.51	0	0	0	0	0	0	0	6.59	6.61	6.61	6.60	6.66	6.95	6.95
1376153_at	---	1	0	0	0	1	1	7.97	7.97	8.43	7.71	8.45	0	0	0	0	0	0	0	9.60	9.65	9.66	9.71	9.63	9.34	8.92
1376157_at	---	1	0	1	1	1	0	5.78	6.07	6.60	4.56	5.91	0	0	0	0	0	0	0	7.51	7.51	7.62	7.61	7.59	7.46	5.77
1376158_at	---	1	0	0	0	0	1	6.83	7.21	7.20	7.07	7.31	0	0	0	0	0	0	0	8.96	8.93	9.02	8.96	8.92	8.75	8.54
1376161_at	Tmed4	1	0	1	0	1	0	7.56	7.66	8.26	7.13	7.65	0	0	0	0	0	0	0	10.66	10.71	10.72	10.72	10.75	10.82	10.14
1376165_at	Slc24a3	1	0	0	0	1	0	5.84	5.87	5.39	6.05	5.79	0	0	0	0	0	0	0	9.78	9.85	10.00	10.09	10.29	10.30	10.14
1376168_at	Mmgt1	1	0	0	1	0	0	5.96	6.06	6.21	6.45	6.33	0	0	0	0	0	0	0	8.90	9.01	9.05	9.19	9.23	9.39	9.30
1376169_at	Phf20l1	1	0	1	0	0	1	6.88	6.99	7.53	7.05	7.41	0	0	0	0	0	0	0	8.45	8.61	8.65	8.79	8.75	8.70	8.26
1376170_at	---	1	0	0	0	0	1	5.54	5.38	5.34	5.15	7.03	0	0	0	0	0	0	0	3.98	3.97	3.90	3.78	3.85	4.08	4.19
1376176_at	Mast3	1	0	0	0	1	0	7.96	7.98	7.50	8.26	7.73	0	0	0	0	0	0	0	8.81	8.78	8.87	8.88	8.91	8.93	8.96
1376180_at	LOC102556224	1	0	1	0	0	0	6.40	6.44	7.10	6.48	6.48	0	0	0	0	0	0	0	8.39	8.44	8.51	8.62	8.62	8.85	8.95
1376189_at	Zmym1	1	0	1	0	1	0	5.79	5.73	6.59	5.79	6.05	0	0	0	0	0	0	0	8.31	8.47	8.58	8.72	8.74	8.81	8.68
1376194_at	---	1	0	1	0	0	0	8.17	8.24	8.76	8.24	8.42	0	0	0	0	0	0	0	7.29	7.46	7.63	7.71	7.71	7.69	7.49
1376204_at	---	1	0	0	0	0	1	4.78	4.89	5.31	4.68	5.79	0	0	0	0	0	0	0	7.56	7.59	7.60	7.42	7.39	7.51	7.35
1376208_at	Dlg2	1	1	0	1	0	1	5.45	6.05	5.61	5.97	5.99	0	0	0	0	0	0	0	5.09	5.08	5.07	4.75	4.88	4.85	5.04
1376213_at	Rundc3b	1	0	0	0	1	0	6.51	6.38	6.30	6.87	6.78	0	0	0	0	0	0	0	7.64	7.66	7.76	7.80	7.71	7.50	7.52
1376218_a_at	Atp8b2	1	0	1	0	1	1	4.19	4.27	4.81	4.01	4.82	0	0	0	0	0	0	0	5.55	5.78	5.57	5.68	5.85	5.95	5.80
1376219_at	Atp8b2	1	0	1	0	1	1	6.41	6.58	7.08	6.15	7.18	0	0	0	0	0	0	0	7.79	7.78	7.84	7.82	7.86	7.84	7.89
1376226_at	---	1	0	0	0	0	1	7.25	7.08	7.35	7.41	4.35	0	0	0	0	0	0	0	3.64	3.72	3.63	3.54	3.53	3.71	3.77
1376241_at	Cul3	1	0	1	1	1	0	5.30	5.38	5.85	4.70	5.33	0	0	0	0	0	0	0	5.86	5.82	6.02	6.10	6.10	6.09	5.64
1376242_at	---	1	0	1	0	1	0	8.09	8.11	8.73	7.93	8.29	0	0	0	0	0	0	0	10.23	10.34	10.30	10.31	10.38	10.38	10.03
1376260_at	Mettl14	1	0	1	0	0	1	5.91	6.15	6.75	6.25	6.51	0	0	0	0	0	0	0	8.73	8.78	8.82	8.93	8.95	8.90	8.42
1376275_at	Arl5b	1	1	0	1	0	0	8.30	7.54	8.15	7.82	8.02	0	0	0	0	0	0	0	7.49	7.49	7.54	7.49	7.49	7.65	8.20
1376285_at	Gulp1	1	0	1	0	1	0	7.55	7.75	8.61	7.12	8.01	0	0	0	0	0	0	0	7.88	7.97	8.06	8.21	8.14	8.20	7.44
1376293_at	Engase	1	0	1	0	0	0	6.85	6.72	6.32	6.66	6.63	0	0	0	0	0	0	0	6.13	6.20	6.22	6.06	6.08	6.23	6.05
1376302_at	Dgke	1	1	0	1	1	1	6.78	6.28	6.97	6.22	6.26	0	0	0	0	0	0	0	8.00	7.97	7.97	8.01	8.03	8.25	8.50
1376304_at	LOC103690511 /	1	0	0	0	0	1	6.23	6.38	6.55	6.51	6.77	0	0	0	0	0	0	0	7.87	7.91	8.02	8.05	8.06	7.32	7.23
1376310_at	---	1	0	0	0	0	1	6.90	7.12	7.46	6.89	7.60	0	0	0	0	0	0	0	8.72	8.77	8.82	8.73	8.74	8.56	8.33
1376315_at	Edem2	1	0	1	0	1	0	8.80	8.61	8.27	8.85	8.50	0	0	0	0	0	0	0	10.61	10.65	10.65	10.61	10.66	10.66	10.08
1376317_at	Orc6	1	0	0	0	0	1	5.77	5.64	6.27	5.68	6.47	0	0	0	0	0	0	0	8.61	8.54	8.71	8.75	8.68	8.43	7.98
1376319_at	Sema3c	1	0	0	1	1	0	7.51	7.31	7.12	7.99	7.45	0	0	0	0	0	0	0	7.36	7.38	7.49	7.39	7.08	7.37	8.22
1376328_at	Cracdl	1	0	0	1	1	0	6.26	6.25	6.23	6.90	6.17	0	0	0	0	0	0	0	7.21	7.22	7.36	7.17	7.31	7.55	7.89
1376333_at	Btbd1	1	0	1	0	1	0	6.95	7.15	7.74	6.84	7.16	0	0	0	0	0	0	0	9.92	9.93	9.88	9.78	9.76	9.66	8.95
1376336_at	LOC103690175 /	1	0	0	0	1	0	6.03	5.86	6.55	5.56	6.40	0	0	0	0	0	0	0	8.61	8.65	8.67	8.86	8.96	9.05	9.00
1376337_at	Smarca2	1	0	0	0	1	0	8.85	8.88	9.41	8.64	9.18	0	0	0	0	0	0	0	10.32	10.35	10.32	10.10	10.18	9.92	9.28
1376363_at	---	1	0	0	0	0	1	6.49	6.77	6.86	6.56	7.08	0	0	0	0	0	0	0	8.04	8.05	8.09	8.20	8.21	8.16	7.90
1376367_at	---	1	0	1	0	0	0	4.76	4.57	4.24	4.59	4.40	0	0	0	0	0	0	0	4.47	4.55	4.48	4.25	4.23	4.31	3.86
1376371_at	LOC100912143	1	0	0	0	1	1	5.63	5.41	5.15	5.84	5.15	0	0	0	0	0	0	0	5.43	5.38	5.32	5.38	5.32	5.33	4.36
1376374_at	Thoc2l	1	0	0	0	1	0	5.13	5.08	5.53	4.82	4.91	0	0	0	0	0	0	0	5.55	5.73	5.85	5.77	5.64	5.51	5.38
1376379_a_at	Adprm	1	1	0	1	0	1	6.08	5.58	5.99	5.54	5.53	0	0	0	0	0	0	0	6.59	6.71	6.80	6.91	6.93	6.72	6.18
1376395_at	Zdbf2	1	0	0	1	1	0	8.37	8.49	8.82	7.84	8.49	0	0	0	0	0	0	0	8.79	8.90	9.09	9.20	9.24	9.35	9.29
1376398_at	---	1	0	0	0	1	1	7.52	7.46	7.97	7.15	8.18	0	0	0	0	0	0	0	6.41	6.46	6.29	6.13	6.11	5.89	5.72
1376411_at	Arl13b	1	0	1	0	1	0	6.22	6.10	6.89	6.18	6.33	0	0	0	0	0	0	0	8.80	8.89	8.89	8.89	8.97	8.88	8.02
1376431_at	Sil1	1	0	0	0	1	0	7.33	7.32	7.02	7.65	6.92	0	0	0	0	0	0	0	9.43	9.55	9.59	9.61	9.72	9.89	9.18
1376441_at	Pcnx4	1	0	1	0	1	0	6.39	6.34	7.09	6.38	6.78	0	0	0	0	0	0	0	8.64	8.70	8.72	8.74	8.82	8.48	8.08
1376442_at	---	1	0	0	1	0	0	5.41	5.66	5.57	5.90	5.57	0	0	0	0	0	0	0	4.87	4.73	4.71	4.70	4.69	4.81	3.99
1376444_at	---	1	0	0	0	1	0	5.57	5.46	5.10	5.98	5.17	0	0	0	0	0	0	0	7.77	7.77	7.91	7.81	7.66	7.56	7.26
1376451_at	Krit1	1	0	1	0	1	0	6.42	6.46	7.23	6.29	6.74	0	0	0	0	0	0	0	8.72	8.84	8.73	8.90	8.97	8.97	8.30
1376455_at	Mfsd3	1	0	0	1	1	0	6.02	6.05	5.82	6.51	6.01	0	0	0	0	0	0	0	8.08	8.06	8.16	8.14	8.12	8.12	7.83
1376470_at	Fnbp4	1	0	0	0	1	0	4.42	4.44	4.98	4.21	4.72	0	0	0	0	0	0	0	7.79	7.82	7.87	7.84	7.92	7.67	7.31
1376476_at	Telo2	1	0	0	0	1	0	5.85	5.72	5.46	6.05	5.67	0	0	0	0	0	0	0	6.55	6.62	6.64	6.68	6.59	6.62	6.25
1376481_at	Adamts9	1	0	1	0	1	0	6.34	6.23	5.77	6.61	5.99	0	0	0	0	0	0	0	5.02	5.16	5.16	4.91	5.15	5.41	6.22
1376501_at	Arhgap8	1	0	0	0	1	0	6.65	6.63	6.15	6.92	6.52	0	0	0	0	0	0	0	7.93	7.97	8.04	8.05	8.06	8.32	8.20
1376502_at	Slc35f6	1	0	0	1	0	0	5.07	5.17	5.07	5.62	5.29	0	0	0	0	0	0	0	6.80	6.75	6.84	6.89	6.91	7.14	7.37
1376516_at	Flywch1	1	1	0	0	0	0	6.07	6.66	5.87	6.41	5.82	0	0	0	0	0	0	0	7.69	7.75	7.82	7.81	7.65	7.74	7.81
1376518_at	Palm3	1	0	1	0	0	1	6.39	6.34	5.70	6.13	5.81	0	0	0	0	0	0	0	5.89	5.92	5.91	5.69	5.70	5.85	6.06
1376531_at	---	1	0	1	1	1	0	8.21	8.27	8.82	7.38	8.23	0	0	0	0	0	0	0	8.79	8.89	8.98	8.90	8.96	8.35	7.55
1376546_at	Svip	1	0	1	0	1	1	10.55	10.38	9.97	10.57	10.05	0	0	0	0	0	0	0	10.42	10.35	10.37	10.37	10.36	10.45	10.54
1376553_at	Nthl1	1	0	1	0	1	0	7.29	7.02	6.54	7.29	7.00	0	0	0	0	0	0	0	7.26	7.21	7.27	7.06	7.22	7.37	7.22
1376559_at	RPIA	1	0	1	0	0	0	7.62	7.74	7.04	7.53	7.47	0	0	0	0	0	0	0	8.15	8.01	8.01	7.72	7.82	8.00	7.90
1376562_at	Tnik	1	0	0	0	0	1	3.61	4.04	3.80	3.84	4.08	0	0	0	0	0	0	0	6.41	6.33	6.37	6.21	6.29	6.19	6.34
1376569_at	Klf2	1	0	0	1	0	0	7.61	7.74	7.17	6.87	7.45	0	0	0	0	0	0	0	5.36	5.34	5.37	5.21	5.09	5.19	5.36
1376572_a_at	Svil	1	0	1	1	0	0	6.81	6.90	7.49	7.45	7.07	0	0	0	0	0	0	0	7.34	7.12	7.24	7.31	7.22	7.15	7.13

Probeset ID	Gene Symbol	Islet Data Set (log2 ratio ≥0.263; FDR <20%) union n=5325 1=YES 0=NO	Islet DRlyp/lyp ND vs ND+Lp299v (log2 ratio ≥0.263; FDR<20%) n=429 1=YES 0=NO	Islet DRlyp/lyp ND vs HCD (log2 ratio ≥0.263; FDR<20%) n=2247 1=YES 0=NO	Islet DRlyp/lyp ND vs HCD+Lp299v (log2 ratio ≥0.263; FDR<20%) n=1595 1=YES 0=NO	Islet DRlyp/lyp HCD vs HCD+Lp299v (log2 ratio ≥0.263; FDR<20%) n=3026 1=YES 0=NO	Islet DRlyp/lyp ND vs Flyp/lyp ND (log2 ratio ≥ 0.263; FDR<20%) n=1950 1=YES 0=NO	Islet Mean Log2 Intensity DRlyp/lyp ND	Islet Mean Log2 Intensity DRlyp/lyp ND+Lp299v	Islet Mean Log2 Intensity DRlyp/lyp HCD	Islet Mean Log2 Intensity DRlyp/lyp HCD+Lp299v	Islet Mean Log2 Intensity Flyp40 ND	Rinm5f data union of all conditions n=3705 1=YES 0=NO	Rinm5f 30 µM propionate/ 6 µM butyrate vs Media (FDR<20%; 1.5 fold) n=3 1=YES 0=NO	Rinm5f 60 µM propionate/ 12 µM butyrate vs Media (FDR<20%; 1.5 fold) n=18 1=YES 0=NO	Rinm5f 120 µM propionate/ 24 µM butyrate vs Media (FDR<20%; 1.5 fold) n=164 1=YES 0=NO	Rinm5f 240 µM propionate /48 µM butyrate vs Media (FDR<20%; 1.5 fold) n=268 1=YES 0=NO	Rinm5f 1500 µM propionate/ 300 µM butyrate vs Media (FDR<20%; 1.5 fold) n=2491 1=YES 0=NO	Rinm5f 500 µM propionate/ 500 µM butyrate vs Media (FDR<20%; 1.5 fold) n=3001 1=YES 0=NO	Rinm5f Mean Log2 Intensity Media only	Rinm5f Mean Log2 Intensity 30 µM propionate/ 6 µM butyrate	Rinm5f Mean Log2 Intensity 60 µM propionate/ 12 µM butyrate	Rinm5f Mean Log2 Intensity 120 µM propionate/ 24 µM butyrate	Rinm5f Mean Log2 Intensity 240 µM propionate/ 48 µM butyrate	Rinm5f Mean Log2 Intensity 1500 µM propionate /300 µM butyrate	Rinm5f Mean Log2 Intensity 500 µM propionate/ 500 µM butyrate
1376578_at	Ehmt1	1	0	1	1	0	0	6.96	7.28	7.54	7.43	7.25	0	0	0	0	0	0	0	9.16	9.14	9.24	9.23	9.17	9.14	8.67
1376579_at	Lap3	1	0	0	1	0	0	6.90	7.12	7.16	7.42	7.34	0	0	0	0	0	0	0	7.97	8.06	8.03	8.00	8.00	8.06	7.66
1376580_at	Fxn	1	0	0	0	1	0	7.87	7.88	7.61	8.27	7.54	0	0	0	0	0	0	0	9.39	9.33	9.40	9.39	9.27	9.17	9.04
1376587_at	Fbxo11	1	0	1	0	1	0	7.68	7.55	8.40	7.30	7.87	0	0	0	0	0	0	0	9.85	9.90	9.95	9.89	10.02	9.63	9.35
1376593_at	Foxo3	1	1	0	0	0	0	9.59	9.04	9.61	9.78	9.15	0	0	0	0	0	0	0	9.91	9.90	9.85	9.74	9.75	9.87	9.82
1376594_at	Vezf1	1	0	1	0	1	0	6.94	7.01	7.85	7.12	7.13	0	0	0	0	0	0	0	9.14	9.01	9.14	9.11	9.07	8.90	8.85
1376605_at	Slc17a5	1	0	0	1	1	0	8.44	8.44	8.45	9.04	8.13	0	0	0	0	0	0	0	8.72	8.72	8.70	8.62	8.73	8.61	7.99
1376613_at	Ttc9c	1	0	0	0	1	0	7.36	7.36	7.92	7.08	7.53	0	0	0	0	0	0	0	10.53	10.47	10.43	10.52	10.41	10.36	10.20
1376619_at	Ptprd	1	0	0	0	0	1	5.83	5.45	5.76	6.19	5.26	0	0	0	0	0	0	0	4.56	4.32	4.69	4.68	4.51	4.57	4.36
1376623_at	Tmem204	1	0	0	0	1	0	6.55	6.21	6.85	6.11	6.41	0	0	0	0	0	0	0	4.82	4.76	4.94	4.56	4.80	4.76	4.44
1376624_at	---	1	0	0	1	0	0	5.19	4.83	5.19	4.55	4.90	0	0	0	0	0	0	0	6.79	6.69	6.83	6.94	6.60	6.52	6.75
1376630_at	Tars3	1	0	0	0	1	0	7.29	7.13	7.70	6.93	6.84	0	0	0	0	0	0	0	7.62	7.50	7.57	7.71	7.55	7.35	7.19
1376633_at	Shisal1	1	0	0	0	0	1	5.52	5.72	5.72	5.81	6.15	0	0	0	0	0	0	0	5.53	5.72	5.71	5.70	5.88	6.08	5.33
1376636_at	Tgfbr1	1	0	1	0	1	0	8.68	8.73	9.48	8.46	9.01	0	0	0	0	0	0	0	8.55	8.47	8.61	8.66	8.57	8.84	8.52
1376637_at	Edil3	1	0	1	0	1	1	7.18	7.38	8.12	7.06	7.93	0	0	0	0	0	0	0	6.91	7.13	7.19	7.25	7.10	6.93	6.47
1376641_at	Thoc1	1	0	1	0	1	0	7.44	7.55	8.10	7.38	7.46	0	0	0	0	0	0	0	9.55	9.64	9.71	9.83	9.82	9.57	8.78
1376648_at	Mycn	1	0	1	0	0	1	4.81	5.19	5.42	5.14	5.39	0	0	0	0	0	0	0	11.59	11.73	11.60	11.43	11.55	11.27	9.92
1376653_at	Phtf1	1	0	1	0	0	0	5.91	5.94	6.62	5.98	6.37	0	0	0	0	0	0	0	9.69	9.72	9.71	9.67	9.70	9.67	9.64
1376658_at	Raph1	1	0	0	1	0	1	5.76	5.92	6.12	6.24	6.39	0	0	0	0	0	0	0	5.56	5.35	5.70	5.53	5.61	5.59	5.80
1376665_at	---	1	0	1	0	1	0	7.37	7.42	8.29	7.18	7.70	0	0	0	0	0	0	0	8.84	8.80	8.94	9.01	9.08	8.88	7.95
1376674_at	Man2a2	1	0	1	0	0	1	6.27	6.59	6.93	6.52	6.88	0	0	0	0	0	0	0	8.86	8.69	8.84	8.67	8.65	8.56	8.84
1376681_at	Rrp8	1	0	1	0	0	0	5.14	5.37	5.84	5.20	5.44	0	0	0	0	0	0	0	7.27	7.22	7.25	7.50	7.35	7.10	6.85
1376685_at	---	1	0	0	0	0	1	6.09	6.04	6.54	6.36	6.74	0	0	0	0	0	0	0	7.31	7.02	7.16	7.21	7.22	7.08	7.29
1376686_at	---	1	0	1	0	1	1	6.06	6.30	6.96	5.91	6.99	0	0	0	0	0	0	0	8.96	8.93	9.01	9.04	9.06	9.37	9.69
1376687_at	Usp1	1	0	1	0	0	1	6.89	7.08	7.56	7.07	7.39	0	0	0	0	0	0	0	10.08	10.12	10.15	10.27	10.23	10.30	10.10
1376693_at	Samd9	1	1	0	0	0	0	4.68	5.46	4.66	4.56	4.65	0	0	0	0	0	0	0	6.19	6.11	6.45	6.70	6.44	6.25	5.64
1376694_at	Lmbr1l	1	0	0	0	1	0	5.32	5.04	4.87	5.58	4.93	0	0	0	0	0	0	0	7.85	8.01	7.93	7.52	7.75	7.86	7.66
1376709_at	Slc39a8	1	0	0	1	1	0	6.86	6.80	6.63	7.53	6.77	0	0	0	0	0	0	0	5.43	5.42	5.64	5.67	5.83	5.93	6.22
1376716_at	LOC102547411	1	0	1	0	0	0	4.59	4.52	4.02	4.21	4.31	0	0	0	0	0	0	0	4.21	4.26	4.14	4.04	4.12	4.17	3.75
1376722_at	Nup205	1	0	1	0	0	1	6.29	6.57	7.18	6.52	7.11	0	0	0	0	0	0	0	10.20	10.15	10.27	10.34	10.29	10.11	9.86
1376727_at	Yipf4	1	0	0	1	1	0	9.20	8.96	9.60	8.61	9.02	0	0	0	0	0	0	0	10.70	10.75	10.74	10.78	10.74	10.62	9.70
1376728_at	Rbm47	1	0	0	1	0	0	9.47	9.46	9.61	10.00	9.52	0	0	0	0	0	0	0	8.80	8.81	8.87	8.85	8.93	9.12	9.33
1376729_at	Appl1	1	0	1	0	1	0	5.86	6.04	6.69	5.60	6.08	0	0	0	0	0	0	0	8.14	8.01	8.15	8.27	8.20	8.17	7.81
1376732_at	Calr3	1	0	0	0	1	0	5.09	5.01	4.95	5.54	4.87	0	0	0	0	0	0	0	6.70	6.81	6.86	6.84	6.96	7.23	6.89
1376744_at	Rnpc3	1	1	0	1	0	0	7.05	6.57	7.13	7.52	6.88	0	0	0	0	0	0	0	6.27	6.37	6.40	6.51	6.43	6.22	5.57
1376748_at	Arx	1	0	0	1	1	0	8.29	8.24	8.59	7.62	8.55	0	0	0	0	0	0	0	9.91	9.86	9.89	9.96	9.95	9.70	9.19
1376749_at	Ogn	1	1	0	1	1	1	5.31	4.59	5.74	4.38	4.68	0	0	0	0	0	0	0	3.51	3.47	3.50	3.47	3.40	3.38	2.86
1376753_at	Fpgt	1	0	1	1	1	0	6.14	5.81	7.08	5.53	6.50	0	0	0	0	0	0	0	8.16	8.26	8.30	8.35	8.36	8.32	7.58
1376760_at	Paxbp1	1	0	1	0	1	0	7.35	7.64	8.13	7.40	7.48	0	0	0	0	0	0	0	9.66	9.70	9.69	9.72	9.71	9.56	8.90
1376763_at	Tet2	1	0	1	0	1	0	6.55	6.46	7.24	6.20	7.00	0	0	0	0	0	0	0	8.33	8.45	8.30	8.25	8.34	8.53	8.57
1376764_at	---	1	0	1	0	0	1	6.83	7.01	7.49	6.84	7.36	0	0	0	0	0	0	0	6.69	6.80	6.70	6.92	7.00	7.02	7.33
1376771_at	Ppm1l	1	0	0	0	0	1	7.44	7.29	7.25	7.33	7.93	0	0	0	0	0	0	0	8.35	8.36	8.41	8.37	8.43	8.74	8.92
1376773_at	---	1	0	0	0	1	0	6.79	6.45	7.07	6.37	6.77	0	0	0	0	0	0	0	8.61	8.60	8.79	8.84	8.87	8.82	8.18
1376780_at	Ramac	1	0	1	0	0	0	4.51	4.56	5.08	4.43	4.77	0	0	0	0	0	0	0	8.53	8.62	8.67	8.77	8.79	9.04	8.82
1376792_at	Eva1a	1	0	1	0	1	0	3.69	3.73	4.38	3.64	4.09	0	0	0	0	0	0	0	5.74	5.81	5.78	5.71	5.92	6.07	6.51
1376796_at	Rab14	1	0	1	1	1	0	7.59	7.68	8.25	7.05	7.46	0	0	0	0	0	0	0	9.54	9.51	9.52	9.66	9.63	9.51	8.82
1376805_at	Rnf2	1	0	1	0	1	0	6.69	6.61	7.39	6.45	6.70	0	0	0	0	0	0	0	9.58	9.64	9.63	9.74	9.73	9.77	9.51
1376806_at	---	1	0	0	1	0	0	4.34	4.31	4.34	4.85	4.37	0	0	0	0	0	0	0	7.40	7.41	7.57	7.72	7.59	7.21	6.78
1376811_a_at	Cpsf6	1	0	1	0	1	0	5.16	5.10	6.00	5.13	5.52	0	0	0	0	0	0	0	8.81	8.84	8.91	9.02	8.91	8.55	7.63
1376845_at	Ifi27l2b	1	1	0	0	0	1	7.57	8.71	7.44	7.77	8.05	0	0	0	0	0	0	0	11.14	11.13	11.30	11.58	11.45	11.25	10.88
1376848_at	---	1	0	1	0	0	1	6.09	6.34	6.66	6.39	6.73	0	0	0	0	0	0	0	7.48	7.29	7.49	7.36	7.37	7.45	7.10
1376852_at	Mccc1	1	0	0	0	0	1	8.45	8.17	8.05	8.28	7.91	0	0	0	0	0	0	0	7.41	7.56	7.46	7.46	7.51	7.25	7.11
1376861_at	Mospd1	1	0	1	0	1	1	7.51	7.26	6.89	7.91	7.00	0	0	0	0	0	0	0	7.23	7.25	7.30	7.58	7.49	7.62	7.92
1376862_at	Ube4b	1	0	0	1	1	0	8.69	9.03	8.59	9.32	8.72	0	0	0	0	0	0	0	9.58	9.56	9.64	9.64	9.64	9.54	9.29
1376864_at	Ccar1	1	0	1	0	0	0	5.75	5.90	6.64	5.97	6.17	0	0	0	0	0	0	0	8.42	8.47	8.73	8.83	8.84	8.63	8.31
1376867_at	Lgalsl	1	0	1	0	1	0	4.74	5.04	5.62	4.55	4.69	0	0	0	0	0	0	0	8.90	8.89	8.87	8.74	8.76	8.43	7.84
1376869_at	Tle4	1	0	1	1	0	0	5.93	6.03	6.64	6.58	6.36	0	0	0	0	0	0	0	8.60	8.51	8.62	8.72	8.64	8.87	9.11
1376881_at	LOC100909655 /	1	0	0	1	1	0	8.63	8.38	8.97	7.92	8.34	0	0	0	0	0	0	0	9.12	9.24	9.36	9.53	9.58	9.53	8.69
1376885_at	---	1	0	0	0	1	0	8.13	8.09	7.92	8.50	8.16	0	0	0	0	0	0	0	9.02	9.00	9.06	8.98	8.97	9.18	9.47
1376887_at	Znf445	1	0	1	0	1	0	7.15	7.17	7.95	7.19	7.33	0	0	0	0	0	0	0	8.19	8.23	8.38	8.48	8.47	8.37	8.08
1376888_a_at	Mllt1	1	0	0	1	0	0	4.44	4.63	4.39	4.91	4.57	0	0	0	0	0	0	0	7.25	7.21	7.38	7.29	7.31	7.37	7.81
1376892_at	Gria3	1	0	0	1	1	0	7.50	7.10	7.68	6.70	7.29	0	0	0	0	0	0	0	3.61	3.56	3.58	3.62	3.52	3.55	3.26
1376894_at	Bloc1s6	1	0	1	0	0	0	6.38	6.33	6.99	6.32	6.18	0	0	0	0	0	0	0	8.46	8.48	8.49	8.58	8.56	8.80	8.53
1376913_at	B3gat1	1	0	0	0	0	1	4.32	4.53	4.60	4.46	4.82	0	0	0	0	0	0	0	8.18	8.21	8.27	8.16	8.17	8.01	7.63
1376946_at	---	1	0	0	0	1	0	7.64	7.87	8.18	7.31	7.75	0	0	0	0	0	0	0	7.96	7.93	8.03	8.09	8.17	8.00	8.02
1376965_at	---	1	0	0	0	0	1	6.43	6.66	6.59	6.64	6.94	0	0	0	0	0	0	0	8.44	8.45	8.53	8.38	8.47	8.50	8.47
1376991_at	Orc3	1	0	0	1	0	0	5.65	5.54	5.84	5.16	5.54	0	0	0	0	0	0	0	7.08	7.02	7.13	7.18	7.26	7.33	7.02
1376993_at	Amotl1	1	0	0	0	1	0	5.18	5.34	4.78	5.35	5.19	0	0	0	0	0	0	0	4.91	4.74	4.81	4.68	4.89	4.88	4.42
1377001_at	Nrcam	1	0	0	0	1	0	5.15	5.20	5.72	5.02	5.13	0	0	0	0	0	0	0	6.67	6.76	6.86	6.98	7.06	6.81	6.62
1377005_at	Cul4b	1	0	1	0	0	0	7.12	7.22	7.80	7.13	7.34	0	0	0	0	0	0	0	8.82	8.93	9.01	9.16	9.09	9.19	9.17
1377008_at	RGD1566401	1	0	1	0	1	0	8.92	9.25	9.82	8.82	9.19	0	0	0	0	0	0	0	3.94	3.71	4.00	3.78	3.85	3.74	3.28
1377011_at	Fry	1	0	1	0	1	0	7.71	7.74	8.33	7.40	7.97	0	0	0	0	0	0	0	9.11	9.17	9.15	9.17	9.15	9.46	9.37
1377016_at	Creld2	1	0	0	0	1	0	9.56	9.71	9.21	9.96	9.90	0	0	0	0	0	0	0	10.81	10.86	10.82	10.80	10.89	10.78	10.18
1377053_at	Pomk	1	0	0	1	1	0	7.37	7.49	7.32	7.92	7.64	0	0	0	0	0	0	0	9.13	9.16	9.23	9.25	9.29	9.26	9.27
1377060_at	Mccc2	1	0	1	0	1	1	8.73	8.52	8.09	8.67	8.10	0	0	0	0	0	0	0	8.18	8.18	8.26	8.22	8.13	8.25	8.60
1377061_at	Arhgap32	1	0	0	1	1	0	7.32	7.55	7.12	8.17	7.25	0	0	0	0	0	0	0	8.54	8.42	8.58	8.41	8.38	8.48	8.61
1377064_at	Dusp6	1	1	1	1	0	0	6.76	6.01	8.38	8.02	6.42	0	0	0	0	0	0	0	9.57	9.74	9.69	9.72	10.06	9.96	9.28
1377065_at	C10orf71	1	0	1	0	0	0	4.93	4.69	4.35	4.64	4.47	0	0	0	0	0	0	0	4.92	4.94	4.75	4.58	4.70	4.67	4.92
1377070_at	Sf3b2	1	1	0	0	0	0	8.32	8.84	8.44	8.70	8.57	0	0	0	0	0	0	0	10.46	10.35	10.36	10.25	10.16	9.88	9.88
1377071_at	Rsf1	1	0	1	0	1	0	5.17	5.21	5.97	5.18	5.47	0	0	0	0	0	0	0	7.27	7.41	7.44	7.55	7.45	6.97	6.36
1377072_at	Kank2	1	0	0	0	1	0	6.70	6.85	6.59	7.15	6.37	0	0	0	0	0	0	0	7.33	7.13	7.31	7.28	7.26	7.40	7.69
1377084_at	---	1	0	1	1	0	1	7.72	7.54	6.88	7.22	6.84	0	0	0	0	0	0	0	8.79	8.86	9.05	9.09	9.12	9.25	8.84
1377092_at	Socs3	1	1	1	1	0	0	6.53	7.68	5.33	5.14	6.61	0	0	0	0	0	0	0	7.98	7.99	7.94	7.64	7.81	7.98	7.31
1377100_at	Pds5b	1	0	0	0	1	0	5.59	5.49	6.15	5.31	5.69	0	0	0	0	0	0	0	7.54	7.42	7.66	7.67	7.63	7.55	7.52
1377111_at	Tab3	1	0	0	0	0	1	3.56	3.80	3.74	3.96	4.94	0	0	0	0	0	0	0	7.36	7.34	7.53	7.57	7.61	7.61	7.63
1377115_at	---	1	0	1	0	0	0	6.07	5.98	5.48	5.95	5.83	0	0	0	0	0	0	0	6.54	6.52	6.53	6.30	6.39	6.34	5.73
1377125_at	Dnajc6	1	0	1	0	1	1	8.98	9.10	9.77	8.64	9.57	0	0	0	0	0	0	0	10.13	10.09	10.14	10.19	10.11	9.83	9.26
1377130_at	Phip	1	0	1	0	1	0	8.20	8.33	8.98	8.00	8.54	0	0	0	0	0	0	0	9.07	9.18	9.28	9.40	9.31	9.36	9.14
1377133_at	LOC100912622	1	0	0	0	0	1	3.13	3.11	3.06	3.21	6.09	0	0	0	0	0	0	0	4.55	4.26	4.33	4.46	4.15	4.18	3.33
1377141_at	Aaed1	1	0	1	0	0	0	8.12	8.20	8.69	8.10	8.27	0	0	0	0	0	0	0	9.09	9.04	9.18	9.32	9.25	9.12	8.63
1377162_at	Zbtb41	1	0	0	0	1	0	6.65	6.61	7.17	6.43	6.87	0	0	0	0	0	0	0	9.12	9.22	9.18	9.29	9.22	9.05	8.50
1377165_at	LOC102547903	1	0	0	0	0	1	6.17	6.35	6.38	6.11	6.85	0	0	0	0	0	0	0	6.78	6.66	6.77	6.68	6.73	6.94	7.29
1377167_at	---	1	0	1	0	1	0	3.91	4.21	4.70	3.98	4.17	0	0	0	0	0	0	0	6.54	6.36	6.47	6.46	6.51	6.44	6.11
1377187_at	Rbm12b	1	0	1	0	0	0	5.16	5.08	6.05	5.46	5.54	0	0	0	0	0	0	0	7.91	7.97	8.04	8.04	8.05	7.88	7.15
1377192_a_at	Clpx	1	0	0	0	0	1	7.39	6.97	7.13	7.29	6.89	0	0	0	0	0	0	0	8.35	8.43	8.37	8.40	8.35	8.34	7.94
1377194_a_at	Ccdc90b	1	0	1	0	0	1	6.80	7.16	7.43	7.05	7.42	0	0	0	0	0	0	0	9.28	9.23	9.23	9.26	9.23	9.23	9.08
1377198_at	Skap2	1	0	0	0	0	1	5.28	5.59	5.75	5.65	5.89	0	0	0	0	0	0	0	8.46	8.49	8.50	8.55	8.59	8.72	8.36
1377199_at	CBFA2T3	1	0	0	0	1	0	7.12	6.98	6.74	7.57	7.02	0	0	0	0	0	0	0	5.51	5.47	5.60	5.66	5.66	5.88	5.75
1377224_at	Taf1	1	0	1	0	1	1	3.76	3.89	4.55	3.54	4.37	0	0	0	0	0	0	0	7.12	7.27	7.14	7.33	7.44	7.05	5.35
1377232_at	Limch1	1	1	1	0	1	1	8.48	8.97	9.12	8.27	9.28	0	0	0	0	0	0	0	9.14	9.10	9.08	9.14	9.08	8.98	9.33
1377243_at	Wbp4	1	0	1	0	1	0	6.67	6.81	7.42	6.68	6.97	0	0	0	0	0	0	0	6.96	6.97	7.00	7.06	7.09	6.77	6.44
1377249_at	Gspt2	1	0	1	0	1	1	5.94	6.34	6.57	5.73	6.45	0	0	0	0	0	0	0	7.26	7.21	7.40	7.43	7.39	7.31	6.85
1377250_at	Fam53b	1	0	0	0	1	0	5.45	5.38	5.15	5.77	5.46	0	0	0	0	0	0	0	5.63	5.51	5.67	5.66	5.60	5.69	6.04
1377263_at	Med7	1	0	1	0	1	0	6.98	6.85	7.73	6.89	7.41	0	0	0	0	0	0	0	8.47	8.57	8.63	8.82	8.76	8.79	8.48
1377268_at	Poc5	1	0	0	0	0	1	4.80	4.92	5.22	4.94	5.38	0	0	0	0	0	0	0	7.80	7.85	7.86	7.75	7.81	7.47	7.15
1377273_at	Nsd2	1	0	1	0	1	0	5.57	5.72	6.35	5.45	5.92	0	0	0	0	0	0	0	8.95	9.06	9.10	9.22	9.27	9.17	9.04
1377282_at	---	1	0	0	0	0	1	4.28	4.27	4.24	4.29	4.86	0	0	0	0	0	0	0	4.65	4.56	4.49	4.33	4.39	4.50	4.24
1377321_at	---	1	0	0	1	0	0	5.28	5.59	5.61	5.85	5.54	0	0	0	0	0	0	0	6.04	6.04	6.18	6.08	5.93	5.90	6.29
1377333_at	---	1	0	1	0	1	1	5.56	5.68	6.33	5.23	6.25	0	0	0	0	0	0	0	8.44	8.52	8.63	8.65	8.71	8.48	8.14
1377334_at	Hla-dqa1	1	0	0	0	0	1	6.28	6.72	5.87	5.99	7.19	0	0	0	0	0	0	0	4.16	4.10	4.19	3.93	3.90	3.99	3.80
1377354_at	---	1	0	0	1	1	0	4.67	4.85	4.39	5.17	4.70	0	0	0	0	0	0	0	7.22	7.24	7.22	7.37	7.32	7.46	7.41
1377366_at	Syt7	1	0	0	0	0	1	9.00	9.09	9.19	8.95	8.52	0	0	0	0	0	0	0	11.13	11.12	11.16	11.11	11.15	10.88	10.65
1377367_at	Kazald1	1	0	1	0	1	1	6.15	5.80	5.44	6.53	5.44	0	0	0	0	0	0	0	5.48	5.71	5.79	5.72	5.88	5.89	6.01
1377376_at	---	1	0	0	1	1	0	4.59	4.50	4.44	5.12	4.39	0	0	0	0	0	0	0	4.99	5.12	5.12	5.03	5.11	5.08	4.80
1377379_at	Irf6	1	0	0	1	1	0	8.61	8.66	8.52	9.33	8.46	0	0	0	0	0	0	0	8.41	8.49	8.47	8.55	8.60	8.54	8.53
1377393_at	Frmd5	1	0	0	0	0	1	5.12	5.21	5.49	5.02	5.74	0	0	0	0	0	0	0	6.92	7.07	6.99	7.02	7.01	7.07	7.11
1377408_at	Pla2g6	1	0	0	0	1	0	5.99	6.03	5.74	6.31	5.99	0	0	0	0	0	0	0	8.59	8.58	8.66	8.63	8.56	8.49	8.14
1377412_at	Chic1	1	0	1	0	0	1	9.08	9.35	9.73	9.05	9.63	0	0	0	0	0	0	0	8.64	8.69	8.79	8.90	8.93	9.20	9.29
1377424_at	---	1	0	0	0	0	1	6.06	6.14	6.54	5.99	6.57	0	0	0	0	0	0	0	10.10	10.15	10.22	10.42	10.44	10.66	9.82
1377427_at	Ptpn14	1	0	0	1	1	0	5.13	5.02	4.92	5.61	4.92	0	0	0	0	0	0	0	4.09	4.14	3.98	4.15	3.91	4.15	4.21
1377445_at	---	1	1	1	1	1	1	6.58	7.12	7.52	5.94	7.25	0	0	0	0	0	0	0	3.43	3.43	3.55	3.40	3.41	3.37	2.98
1377455_at	---	1	0	0	0	0	1	3.50	3.47	3.31	3.44	4.61	0	0	0	0	0	0	0	3.92	3.62	3.79	3.66	3.80	3.66	3.56
1377457_a_at	Sorl1	1	0	1	0	1	0	9.09	9.30	9.92	8.87	9.50	0	0	0	0	0	0	0	10.46	10.42	10.48	10.45	10.56	10.38	10.05
1377460_at	LOC102552398	1	0	1	1	0	1	6.83	6.39	5.99	6.02	5.91	0	0	0	0	0	0	0	3.57	3.57	3.64	3.64	3.52	3.65	3.23
1377466_a_at	---	1	0	1	0	0	0	4.96	4.74	4.29	4.63	4.55	0	0	0	0	0	0	0	5.09	5.02	4.93	4.90	4.80	4.83	4.65
1377470_at	---	1	0	0	1	1	0	6.79	6.80	6.74	5.96	6.60	0	0	0	0	0	0	0	5.01	4.91	4.89	5.03	4.91	5.31	4.06
1377487_at	Foxc2	1	0	1	0	0	0	6.95	6.88	6.34	6.67	6.64	0	0	0	0	0	0	0	6.48	6.53	6.49	6.27	6.35	6.29	5.90
1377492_at	Rprml	1	0	0	0	0	1	5.17	5.31	5.29	4.93	4.36	0	0	0	0	0	0	0	9.08	9.05	9.07	9.05	9.03	9.15	9.25
1377501_at	Znf317	1	0	0	0	0	1	5.07	5.19	5.35	5.48	5.74	0	0	0	0	0	0	0	7.05	7.07	7.22	7.24	7.20	6.84	5.95
1377558_at	---	1	0	0	1	0	0	3.93	3.96	4.27	4.40	4.06	0	0	0	0	0	0	0	6.23	6.05	6.33	6.16	6.18	5.87	5.67
1377572_at	Uggt2	1	0	1	0	1	0	3.65	3.88	4.50	3.59	4.10	0	0	0	0	0	0	0	8.47	8.58	8.68	8.83	8.91	8.60	7.93
1377574_at	---	1	0	0	1	1	0	4.48	4.53	4.34	5.06	4.22	0	0	0	0	0	0	0	4.03	4.13	4.29	4.07	4.02	4.10	3.65
1377580_at	Tcaf1	1	0	1	1	1	0	4.42	4.27	5.08	3.84	4.18	0	0	0	0	0	0	0	7.73	7.84	7.84	7.96	7.82	7.93	6.62
1377595_at	Itprid2	1	0	0	1	1	0	9.07	9.07	9.12	9.80	9.27	0	0	0	0	0	0	0	9.34	9.23	9.20	9.15	9.13	9.08	9.12
1377615_at	Tmem130	1	0	0	0	0	1	8.79	9.24	9.23	8.90	9.57	0	0	0	0	0	0	0	11.58	11.60	11.67	11.69	11.79	11.71	11.26

Probeset ID	Gene Symbol	Islet Data Set (log2 ratio ≥0.263; FDR <20%) union n=5325 1=YES 0=NO	Islet DRlyp/lyp ND vs ND+Lp299v (log2 ratio ≥0.263; FDR<20%) n=429 1=YES 0=NO	Islet DRlyp/lyp ND vs HCD (log2 ratio ≥0.263; FDR<20%) n=2247 1=YES 0=NO	Islet DRlyp/lyp ND vs HCD+Lp299v (log2 ratio ≥0.263; FDR<20%) n=1595 1=YES 0=NO	Islet DRlyp/lyp HCD vs HCD+Lp299v (log2 ratio ≥0.263; FDR<20%) n=3026 1=YES 0=NO	Islet DRlyp/lyp ND vs Flyp/lyp ND (log2 ratio ≥ 0.263; FDR<20%) n=1950 1=YES 0=NO	Islet Mean Log2 Intensity DRlyp/lyp ND	Islet Mean Log2 Intensity DRlyp/lyp ND+Lp299v	Islet Mean Log2 Intensity DRlyp/lyp HCD	Islet Mean Log2 Intensity DRlyp/lyp HCD+Lp299v	Islet Mean Log2 Intensity Flyp40 ND	Rinm5f data union of all conditions n=3705 1=YES 0=NO	Rinm5f 30 µM propionate/ 6 µM butyrate vs Media (FDR<20%; 1.5 fold) n=3 1=YES 0=NO	Rinm5f 60 µM propionate/ 12 µM butyrate vs Media (FDR<20%; 1.5 fold) n=18 1=YES 0=NO	Rinm5f 120 µM propionate/ 24 µM butyrate vs Media (FDR<20%; 1.5 fold) n=164 1=YES 0=NO	Rinm5f 240 µM propionate /48 µM butyrate vs Media (FDR<20%; 1.5 fold) n=268 1=YES 0=NO	Rinm5f 1500 µM propionate/ 300 µM butyrate vs Media (FDR<20%; 1.5 fold) n=2491 1=YES 0=NO	Rinm5f 500 µM propionate/ 500 µM butyrate vs Media (FDR<20%; 1.5 fold) n=3001 1=YES 0=NO	Rinm5f Mean Log2 Intensity Media only	Rinm5f Mean Log2 Intensity 30 µM propionate/ 6 µM butyrate	Rinm5f Mean Log2 Intensity 60 µM propionate/ 12 µM butyrate	Rinm5f Mean Log2 Intensity 120 µM propionate/ 24 µM butyrate	Rinm5f Mean Log2 Intensity 240 µM propionate/ 48 µM butyrate	Rinm5f Mean Log2 Intensity 1500 µM propionate /300 µM butyrate	Rinm5f Mean Log2 Intensity 500 µM propionate/ 500 µM butyrate
1377616_at	Cibar1	1	0	0	0	0	1	7.39	7.73	7.77	7.16	8.04	0	0	0	0	0	0	0	10.08	10.09	10.11	10.20	10.11	9.75	9.29
1377617_at	Pura	1	0	0	0	1	0	8.32	8.11	8.87	8.15	8.56	0	0	0	0	0	0	0	10.20	10.25	10.36	10.33	10.35	10.33	10.05
1377619_at	Wdr36	1	0	0	0	0	1	4.88	4.82	5.34	4.88	5.74	0	0	0	0	0	0	0	8.13	8.16	8.33	8.42	8.44	8.26	7.99
1377622_at	Rsf1	1	0	0	0	1	0	5.68	5.64	6.13	5.38	5.84	0	0	0	0	0	0	0	7.96	7.98	8.07	8.15	8.07	7.67	7.45
1377626_at	LOC100911030	1	1	0	0	0	0	4.34	6.09	4.13	4.35	4.20	0	0	0	0	0	0	0	4.53	4.41	4.46	4.45	4.55	4.57	4.15
1377635_at	Fmo2	1	0	0	0	0	1	4.01	3.83	3.81	3.64	3.50	0	0	0	0	0	0	0	4.11	4.06	4.02	3.95	4.01	3.91	3.39
1377637_at	B3gnt5	1	0	0	0	0	1	3.54	3.67	3.41	3.58	4.59	0	0	0	0	0	0	0	3.61	3.37	3.67	3.49	3.65	3.55	3.20
1377640_at	Calcrl	1	0	1	0	1	0	7.10	6.97	7.71	6.87	6.82	0	0	0	0	0	0	0	3.61	3.54	3.59	3.63	3.56	3.58	2.88
1377649_at	---	1	0	1	0	0	0	6.32	6.20	6.90	6.25	6.52	0	0	0	0	0	0	0	7.34	7.34	7.47	7.53	7.54	7.87	7.71
1377651_at	---	1	0	0	0	0	1	3.22	3.14	3.64	3.08	4.76	0	0	0	0	0	0	0	9.04	8.93	9.02	9.06	8.98	8.74	7.55
1377653_at	Syne2	1	0	0	0	0	1	7.69	7.84	7.98	7.65	8.32	0	0	0	0	0	0	0	8.53	8.52	8.56	8.64	8.67	8.80	8.99
1377660_at	Urah	1	1	1	0	1	1	6.90	6.33	6.28	6.93	6.36	0	0	0	0	0	0	0	4.56	4.58	4.60	4.37	4.32	4.42	4.35
1377662_at	Pir	1	0	1	0	0	1	3.69	4.05	4.30	3.81	4.26	0	0	0	0	0	0	0	8.28	8.22	8.43	8.68	8.65	8.70	8.29
1377664_at	Cep170	1	0	1	0	1	1	7.59	7.70	8.47	7.27	8.10	0	0	0	0	0	0	0	10.06	10.17	10.18	10.25	10.18	10.06	9.97
1377669_at	Rab27a	1	0	1	0	1	0	7.89	7.92	8.51	7.75	7.94	0	0	0	0	0	0	0	8.70	8.74	8.74	8.83	8.85	8.52	8.07
1377671_at	---	1	0	1	0	0	0	5.86	5.84	5.33	5.64	5.95	0	0	0	0	0	0	0	5.50	5.32	5.16	5.13	5.10	5.18	4.49
1377678_at	PLXNA4	1	0	0	1	1	0	6.77	6.60	6.36	7.63	6.33	0	0	0	0	0	0	0	6.33	6.18	6.29	6.16	6.05	6.10	6.24
1377681_at	---	1	0	1	0	1	0	4.01	3.90	4.87	3.71	3.93	0	0	0	0	0	0	0	7.42	7.45	7.57	7.66	7.65	7.73	7.50
1377685_at	Esco1	1	0	0	0	1	0	7.41	7.37	7.90	7.11	7.58	0	0	0	0	0	0	0	9.68	9.77	9.74	9.85	9.88	9.75	9.23
1377700_at	Atrx	1	0	1	0	1	0	8.41	8.39	9.00	8.30	8.62	0	0	0	0	0	0	0	9.51	9.63	9.71	9.85	9.82	9.56	8.94
1377701_at	Zmym6	1	0	1	0	1	0	6.47	6.43	7.20	6.45	6.68	0	0	0	0	0	0	0	8.84	8.96	8.99	9.09	9.05	9.12	9.02
1377703_at	Fam122a	1	0	1	0	0	0	6.78	6.89	7.40	6.76	7.08	0	0	0	0	0	0	0	9.37	9.32	9.34	9.47	9.43	9.64	9.22
1377719_a_at	---	1	0	0	0	0	1	8.99	9.29	9.31	8.92	9.87	0	0	0	0	0	0	0	11.74	11.79	11.68	11.57	11.67	11.68	11.01
1377720_x_at	---	1	0	0	0	0	1	9.32	9.74	9.68	9.36	9.99	0	0	0	0	0	0	0	11.73	11.86	11.71	11.61	11.71	11.84	11.32
1377728_at	Rbis	1	0	0	1	1	0	6.52	6.57	6.31	7.14	6.45	0	0	0	0	0	0	0	9.00	9.15	9.12	9.19	9.28	9.31	8.84
1377731_at	C1d	1	0	0	0	0	1	8.72	8.76	9.25	8.73	9.29	0	0	0	0	0	0	0	11.53	11.58	11.55	11.56	11.58	11.61	11.16
1377735_at	PLEKHA3	1	0	1	0	1	0	7.39	7.46	8.01	7.25	7.52	0	0	0	0	0	0	0	8.33	8.27	8.36	8.39	8.39	8.33	8.11
1377741_at	Phf20l1	1	0	1	0	1	0	4.11	4.25	4.76	3.77	4.30	0	0	0	0	0	0	0	5.96	5.61	6.11	6.16	5.95	5.70	4.36
1377743_at	---	1	0	1	1	1	0	5.72	5.56	7.09	4.79	5.65	0	0	0	0	0	0	0	9.09	9.07	9.17	9.22	9.26	9.13	8.36
1377745_at	Lrrc40	1	0	1	0	0	0	6.02	6.18	6.84	6.19	6.45	0	0	0	0	0	0	0	9.30	9.26	9.26	9.34	9.32	9.10	8.52
1377748_at	Zfp955a /// Zfp9	1	0	1	0	1	0	6.77	7.13	7.39	6.57	6.97	0	0	0	0	0	0	0	8.47	8.47	8.48	8.40	8.43	8.08	7.48
1377753_at	---	1	0	0	1	1	0	7.02	6.61	6.59	8.16	6.75	0	0	0	0	0	0	0	8.28	8.33	8.44	8.52	8.43	8.66	8.50
1377755_at	Ascc3	1	0	0	0	0	1	6.63	6.52	7.08	6.66	7.13	0	0	0	0	0	0	0	9.07	9.01	9.11	9.15	9.10	9.01	8.43
1377757_at	Shprh	1	0	1	0	1	0	6.98	6.94	7.65	6.80	7.31	0	0	0	0	0	0	0	8.18	8.20	8.36	8.41	8.39	8.31	8.37
1377758_at	Hsd17b13	1	1	1	0	1	1	9.70	9.11	9.14	9.82	8.79	0	0	0	0	0	0	0	3.41	3.47	3.50	3.39	3.35	3.41	3.02
1377761_at	Gfpt2	1	0	0	0	1	0	7.59	7.74	8.10	7.36	7.75	0	0	0	0	0	0	0	7.08	7.13	7.28	7.30	7.26	7.28	7.43
1377762_at	---	1	0	0	1	0	0	7.10	7.01	7.17	7.58	7.14	0	0	0	0	0	0	0	8.08	8.23	8.26	8.08	8.15	7.76	6.78
1377765_at	Clic4	1	0	0	1	1	1	5.76	5.54	5.90	4.68	5.28	0	0	0	0	0	0	0	8.22	8.23	8.37	8.57	8.47	8.73	7.47
1377769_at	Ap1s1	1	0	0	0	1	0	7.12	6.87	7.39	6.64	7.00	0	0	0	0	0	0	0	11.06	11.11	11.10	11.10	11.05	11.04	10.40
1377770_at	---	1	0	1	0	1	0	5.86	5.96	6.53	5.75	5.99	0	0	0	0	0	0	0	7.10	7.13	7.20	7.22	7.13	6.86	6.28
1377781_at	---	1	0	0	1	1	0	5.18	5.04	5.59	4.25	4.98	0	0	0	0	0	0	0	5.49	5.30	5.63	5.76	5.79	5.95	5.33
1377791_at	LOC100909655 /	1	0	0	1	1	0	9.41	9.40	9.90	8.85	9.28	0	0	0	0	0	0	0	10.48	10.66	10.72	10.83	10.87	10.97	10.10
1377809_at	Pet100	1	0	0	0	1	0	8.09	8.49	7.60	8.30	7.85	0	0	0	0	0	0	0	9.54	9.50	9.44	9.46	9.41	9.27	9.19
1377812_a_at	C2cd2l	1	0	0	0	0	1	7.58	7.69	7.75	7.47	8.29	0	0	0	0	0	0	0	8.22	8.28	8.30	8.30	8.38	8.27	8.42
1377820_a_at	Ate1	1	0	0	0	1	0	7.70	7.62	8.13	7.42	8.00	0	0	0	0	0	0	0	9.18	9.16	9.28	9.39	9.45	9.65	9.30
1377823_at	---	1	0	0	0	1	1	5.46	5.39	6.00	5.11	6.07	0	0	0	0	0	0	0	9.91	9.73	9.85	9.83	9.82	9.53	9.16
1377826_at	Trim21	1	0	1	0	0	0	5.70	5.82	6.32	5.68	5.84	0	0	0	0	0	0	0	6.65	6.82	6.86	6.94	7.01	7.23	6.78
1377830_a_at	Fuom	1	0	0	0	1	0	7.01	7.07	6.70	7.28	6.70	0	0	0	0	0	0	0	8.65	8.66	8.71	8.65	8.61	8.40	8.10
1377833_at	Rif1	1	0	1	0	0	0	5.71	5.73	6.52	5.84	5.92	0	0	0	0	0	0	0	7.55	7.65	7.84	8.02	7.89	7.76	7.67
1377840_at	Ccm2l	1	0	0	0	0	1	5.58	5.30	5.07	5.61	5.09	0	0	0	0	0	0	0	5.17	5.28	5.19	5.18	5.29	5.31	5.73
1377841_at	Sprn	1	0	1	1	1	0	7.84	7.83	7.28	8.66	7.56	0	0	0	0	0	0	0	6.78	6.89	6.95	6.92	6.94	6.75	6.80
1377842_at	Twistnb	1	0	0	1	0	0	6.92	6.97	7.10	7.40	6.89	0	0	0	0	0	0	0	9.58	9.57	9.63	9.70	9.63	9.61	9.37
1377844_at	Ptgr2	1	0	1	0	1	0	6.64	6.61	7.39	6.33	6.93	0	0	0	0	0	0	0	4.05	3.99	3.96	3.86	3.93	4.03	3.35
1377860_at	Ttc39b	1	0	0	1	0	0	6.11	6.17	6.19	6.64	6.19	0	0	0	0	0	0	0	7.11	7.27	7.22	7.15	7.28	6.96	6.84
1377867_at	Qpct	1	0	1	0	1	0	4.23	4.27	4.99	4.18	3.81	0	0	0	0	0	0	0	9.95	10.14	10.15	10.26	10.40	10.42	9.73
1377868_at	Gprasp2	1	0	1	0	1	0	7.39	7.69	8.27	7.20	7.78	0	0	0	0	0	0	0	10.26	10.25	10.34	10.44	10.43	10.01	9.30
1377875_at	---	1	0	0	1	0	0	5.78	5.39	5.59	5.29	5.74	0	0	0	0	0	0	0	7.83	7.95	8.14	8.19	8.27	7.82	7.32
1377898_at	Slc39a5	1	0	1	0	1	1	6.94	6.55	6.20	6.81	6.04	0	0	0	0	0	0	0	5.44	5.60	5.37	5.28	5.30	5.37	4.86
1377899_at	Cryzl2	1	0	0	0	0	1	5.61	5.68	5.99	5.53	6.35	0	0	0	0	0	0	0	6.59	6.68	6.76	6.70	6.68	6.21	6.25
1377900_at	Abhd14b	1	0	0	1	1	0	8.76	8.58	9.27	8.04	8.58	0	0	0	0	0	0	0	9.93	9.92	9.95	10.03	9.92	9.98	9.19
1377905_at	Lsm14a	1	0	0	0	1	0	6.21	6.10	6.59	5.81	6.09	0	0	0	0	0	0	0	8.56	8.58	8.63	8.66	8.62	8.41	7.68
1377906_at	Dhx36	1	0	1	0	0	0	5.46	5.80	6.32	5.69	5.66	0	0	0	0	0	0	0	7.98	7.87	8.00	8.13	8.02	8.01	7.44
1377910_at	---	1	0	0	0	0	1	4.09	4.18	4.51	4.28	4.71	0	0	0	0	0	0	0	5.83	5.85	5.98	6.15	6.11	5.80	4.56
1377911_at	Osbpl8	1	0	0	0	1	0	6.22	6.12	6.79	6.05	6.60	0	0	0	0	0	0	0	8.37	8.41	8.52	8.55	8.57	8.73	8.87
1377924_at	---	1	0	0	0	1	0	5.75	5.47	5.27	6.08	5.36	0	0	0	0	0	0	0	5.74	5.61	5.87	5.88	5.85	6.05	5.83
1377926_at	Agap1	1	0	0	0	1	0	6.05	6.06	5.82	6.47	6.12	0	0	0	0	0	0	0	6.80	6.93	6.98	6.80	6.87	6.94	7.56
1377932_at	---	1	0	1	0	0	0	6.03	5.84	5.47	5.87	5.81	0	0	0	0	0	0	0	5.01	4.84	4.95	4.85	4.85	5.12	4.59
1377938_at	LOC100910875 /	1	0	0	1	0	0	5.96	5.99	6.00	6.48	5.96	0	0	0	0	0	0	0	7.32	7.28	7.41	7.41	7.26	6.99	6.61
1377946_at	---	1	0	0	1	1	0	7.41	7.42	7.18	7.98	7.36	0	0	0	0	0	0	0	4.14	3.97	4.07	3.95	3.94	3.98	3.64
1377950_at	Mgc108823	1	1	0	0	0	1	4.64	5.70	4.32	4.41	4.00	0	0	0	0	0	0	0	3.34	3.35	3.24	3.21	3.23	3.21	2.71
1377955_at	Orai2	1	0	1	0	1	1	7.16	7.07	6.53	7.36	6.64	0	0	0	0	0	0	0	8.39	8.44	8.48	8.44	8.45	8.27	7.91
1377964_at	---	1	0	0	1	1	0	7.16	7.20	7.54	6.43	7.00	0	0	0	0	0	0	0	8.87	8.95	8.90	8.86	8.86	8.83	7.90
1377968_at	Mbnl3	1	1	1	0	1	0	6.65	6.21	6.08	6.68	6.35	0	0	0	0	0	0	0	6.64	6.59	6.73	6.82	6.62	6.48	6.30
1377971_at	LOC100911498	1	1	0	1	0	0	8.52	9.06	8.91	9.33	8.89	0	0	0	0	0	0	0	3.88	3.73	3.79	3.77	3.85	3.82	3.20
1377975_at	---	1	0	1	0	1	0	7.18	7.00	8.08	7.00	6.78	0	0	0	0	0	0	0	10.37	10.40	10.47	10.46	10.53	10.52	10.14
1377979_at	Pyurf	1	0	0	0	0	1	5.66	5.36	5.65	5.80	5.16	0	0	0	0	0	0	0	6.85	6.85	6.92	6.99	7.03	6.78	6.05
1377980_at	Vstm2l	1	1	0	0	0	1	7.43	7.94	7.94	7.70	8.23	0	0	0	0	0	0	0	9.20	9.25	9.38	9.28	9.33	9.33	8.40
1377986_at	Scoc	1	0	1	1	1	0	6.94	7.15	7.77	6.12	7.12	0	0	0	0	0	0	0	9.31	9.35	9.41	9.48	9.45	9.41	8.80
1377992_at	Dusp7	1	0	1	1	0	1	7.12	7.29	7.88	7.60	7.59	0	0	0	0	0	0	0	7.82	7.82	7.92	7.99	8.01	8.25	8.00
1378000_at	Tmem178b	1	0	1	1	1	0	6.66	6.65	7.34	6.17	6.89	0	0	0	0	0	0	0	8.03	8.05	8.09	8.14	8.09	7.93	7.75
1378002_at	Hspa4l	1	0	1	0	1	1	5.91	6.09	6.64	5.61	6.64	0	0	0	0	0	0	0	8.24	8.22	8.32	8.32	8.36	8.21	8.43
1378007_at	Ecpas	1	0	1	0	0	1	5.35	5.51	6.08	5.58	5.88	0	0	0	0	0	0	0	7.02	6.89	7.11	7.28	7.12	6.79	6.15
1378015_at	Ccl21	1	0	0	0	0	1	4.96	4.87	4.55	4.85	6.84	0	0	0	0	0	0	0	5.22	5.15	5.19	5.15	5.21	5.29	5.04
1378021_at	Hspa4l	1	0	1	0	1	1	4.60	4.88	5.74	4.39	5.39	0	0	0	0	0	0	0	7.23	7.32	7.36	7.47	7.39	7.42	7.57
1378029_at	---	1	1	0	0	0	1	6.66	5.79	6.29	6.26	5.94	0	0	0	0	0	0	0	4.95	4.88	5.14	5.06	5.03	5.48	5.52
1378048_at	Tcf7l2	1	0	0	0	1	0	4.34	4.42	4.11	4.74	4.45	0	0	0	0	0	0	0	4.67	4.83	4.69	4.47	4.56	5.02	4.25
1378055_at	---	1	0	0	0	1	0	4.88	4.97	4.50	5.18	4.79	0	0	0	0	0	0	0	5.35	5.48	5.46	5.17	5.32	5.24	5.09
1378065_at	RGD1566401	1	0	1	0	0	1	5.90	6.24	6.55	5.96	6.39	0	0	0	0	0	0	0	4.05	4.02	4.07	3.92	4.11	4.20	3.95
1378077_at	---	1	0	1	0	0	0	4.47	4.42	3.94	4.39	4.24	0	0	0	0	0	0	0	4.81	4.76	4.71	4.72	4.64	4.75	4.21
1378094_at	Phldb2	1	0	1	0	1	1	7.56	7.48	8.26	7.43	8.12	0	0	0	0	0	0	0	7.03	7.12	7.21	7.41	7.31	6.59	6.61
1378096_at	Kif5b	1	0	0	0	1	0	6.30	6.42	6.74	5.85	6.57	0	0	0	0	0	0	0	7.96	7.83	8.07	8.22	8.14	7.82	7.34
1378097_at	---	1	1	1	0	0	0	6.31	7.22	6.89	6.32	6.51	0	0	0	0	0	0	0	4.36	4.08	4.51	4.21	4.49	4.43	4.06
1378098_at	---	1	0	0	0	0	1	7.37	7.34	7.71	7.34	8.21	0	0	0	0	0	0	0	9.12	9.20	9.29	9.32	9.34	8.96	8.74
1378099_at	---	1	0	1	0	1	0	6.33	6.48	7.04	5.87	6.44	0	0	0	0	0	0	0	3.90	3.87	3.94	3.73	3.73	3.94	3.40
1378110_at	Mtdh	1	0	1	0	0	0	6.18	6.12	6.85	6.18	6.24	0	0	0	0	0	0	0	8.87	8.85	8.96	9.10	9.03	8.63	8.32
1378137_at	Lrrc59	1	0	0	1	0	0	8.06	7.76	7.70	7.39	7.70	0	0	0	0	0	0	0	10.31	10.34	10.39	10.46	10.34	10.27	9.61
1378139_at	---	1	0	1	1	1	0	6.16	6.16	7.02	5.60	6.50	0	0	0	0	0	0	0	9.01	9.04	9.16	9.27	9.38	9.56	8.85
1378141_at	Tmem104	1	0	0	1	1	0	5.44	5.73	5.20	6.04	5.34	0	0	0	0	0	0	0	5.80	5.81	5.81	5.69	5.74	5.77	6.31
1378144_at	Kank4	1	0	0	1	0	0	4.90	4.60	5.03	5.46	4.87	0	0	0	0	0	0	0	4.30	4.34	4.26	4.24	4.14	4.41	3.99
1378163_at	Grem2	1	0	1	1	0	0	4.99	4.80	4.13	4.47	5.34	0	0	0	0	0	0	0	4.08	4.02	4.04	3.90	3.90	4.06	4.24
1378180_at	Dctd	1	0	1	0	1	1	6.79	6.92	7.41	6.67	7.38	0	0	0	0	0	0	0	9.51	9.56	9.64	9.75	9.71	9.65	8.89
1378217_at	Zfhx2	1	0	0	0	0	1	6.19	6.53	6.33	6.40	6.70	0	0	0	0	0	0	0	6.43	6.47	6.67	6.41	6.45	6.52	5.80
1378249_x_at	LOC102554740 /	1	0	1	0	1	1	9.36	9.27	8.44	9.21	8.89	0	0	0	0	0	0	0	8.54	8.50	8.47	8.39	8.41	8.81	8.28
1378255_at	Smad5	1	0	0	0	1	0	6.78	6.93	6.54	7.11	6.68	0	0	0	0	0	0	0	7.04	6.99	7.07	7.05	7.02	7.04	7.25
1378282_at	Csnk2a2	1	0	0	1	0	0	7.17	7.42	7.23	7.76	7.38	0	0	0	0	0	0	0	8.06	7.89	8.07	8.09	7.97	7.85	8.12
1378301_at	Slc16a6	1	0	1	0	0	0	4.57	4.46	3.92	4.19	4.23	0	0	0	0	0	0	0	4.63	4.75	4.91	4.90	4.87	4.79	3.92
1378303_at	Tnrc6b	1	0	0	1	0	0	7.30	7.46	7.60	7.86	7.62	0	0	0	0	0	0	0	7.95	7.80	7.80	7.70	7.68	7.71	8.10
1378332_at	Klf3	1	0	0	1	1	0	6.34	6.15	6.03	6.88	6.06	0	0	0	0	0	0	0	6.26	6.46	6.45	6.44	6.40	6.56	7.01
1378352_at	Golph3l	1	0	0	0	1	0	8.12	8.30	7.83	8.46	8.25	0	0	0	0	0	0	0	8.13	8.12	8.22	8.18	8.13	8.17	8.22
1378384_at	Ints6	1	0	0	1	0	0	5.20	5.25	5.18	4.60	5.17	0	0	0	0	0	0	0	5.76	5.87	6.02	6.16	5.95	5.89	4.55
1378391_at	---	1	0	0	0	1	0	6.22	6.17	6.73	5.99	6.22	0	0	0	0	0	0	0	8.37	8.28	8.38	8.38	8.40	8.36	8.75
1378416_at	Ttc8	1	0	1	0	1	1	6.39	6.44	7.43	6.17	6.87	0	0	0	0	0	0	0	8.39	8.46	8.56	8.56	8.62	8.90	8.38
1378421_at	LOC100910996	1	0	1	0	1	1	9.74	9.92	10.48	9.46	9.05	0	0	0	0	0	0	0	10.79	10.84	10.85	10.75	10.72	10.57	10.50
1378441_at	U2surp	1	0	0	0	1	0	6.84	6.73	7.28	6.50	6.86	0	0	0	0	0	0	0	7.84	7.95	7.96	7.98	8.04	8.08	7.73
1378447_at	---	1	0	0	1	1	0	5.13	5.17	5.58	4.57	5.55	0	0	0	0	0	0	0	6.91	6.98	7.26	7.27	7.31	7.01	6.71
1378458_at	---	1	0	0	1	0	0	5.54	5.59	5.80	6.03	5.89	0	0	0	0	0	0	0	5.73	5.93	5.97	5.75	5.74	5.80	6.07
1378467_at	Lrch1	1	0	1	0	1	0	5.04	4.92	5.63	4.78	4.87	0	0	0	0	0	0	0	4.81	4.81	4.89	4.92	4.90	4.78	4.64
1378493_at	Fam29a	1	0	0	0	0	1	4.72	4.65	5.11	4.53	5.20	0	0	0	0	0	0	0	7.45	7.41	7.44	7.61	7.54	7.28	7.28
1378511_at	---	1	0	1	0	1	1	6.89	6.80	6.13	6.78	6.28	0	0	0	0	0	0	0	5.96	6.06	6.08	5.94	6.10	6.16	6.09
1378515_at	---	1	0	1	0	1	0	5.93	6.24	6.63	5.73	6.04	0	0	0	0	0	0	0	5.88	5.69	5.96	5.73	5.72	5.60	5.63
1378517_at	Trub1	1	0	1	0	0	0	5.43	5.32	6.03	5.48	5.55	0	0	0	0	0	0	0	7.09	7.06	7.25	7.28	7.27	7.29	6.58
1378524_at	Rnf19a	1	0	1	1	1	0	6.61	6.61	7.26	5.96	6.65	0	0	0	0	0	0	0	9.05	9.08	9.07	9.12	9.07	9.31	9.04
1378544_at	Rbbp6	1	0	0	1	0	0	6.67	6.88	6.85	7.09	6.79	0	0	0	0	0	0	0	8.09	7.96	8.12	8.05	7.87	7.94	8.05
1378547_at	---	1	0	0	0	0	1	6.48	6.55	6.81	6.42	7.47	0	0	0	0	0	0	0	7.62	7.69	7.89	8.06	7.98	7.92	7.28
1378568_a_at	---	1	0	0	0	1	0	8.48	8.32	7.98	8.87	8.21	0	0	0	0	0	0	0	9.72	9.74	9.75	9.87	9.88	9.92	9.83
1378578_at	Bclaf3	1	0	1	0	0	0	4.91	4.83	5.57	4.96	4.85	0	0	0	0	0	0	0	8.25	8.34	8.39	8.48	8.45	8.38	7.90
1378582_at	Trabd	1	0	1	0	1	0	7.96	7.82	7.43	8.06	7.64	0	0	0	0	0	0	0	8.83	8.95	8.94	9.03	9.00	8.69	7.92
1378586_at	Cish	1	0	0	1	1	1	6.43	6.06	6.18	7.29	5.64	0	0	0	0	0	0	0	6.96	6.91	7.08	6.90	6.79	6.86	7.00
1378587_at	Ankrd40	1	0	0	1	0	1	5.87	5.43	5.88	5.29	5.40	0	0	0	0	0	0	0	8.66	8.62	8.70	8.69	8.65	8.54	7.54
1378589_at	Edn3	1	0	0	0	0	1	5.97	6.43	6.26	6.09	6.76	0	0	0	0	0	0	0	6.13	6.12	6.06	6.04	6.11	5.96	5.50
1378605_at	Sigirr	1	0	1	0	1	1	7.48	7.21	6.85	7.58	6.95	0	0	0	0	0	0	0	6.64	6.70	6.79	6.88	6.82	6.78	6.37
1378606_at	Fam13b	1	0	1	0	0	0	5.87	5.77	6.48	5.99	6.17	0	0	0	0	0	0	0	8.21	8.25	8.39	8.57	8.51	8.27	7.99
1378629_at	Satb1	1	0	0	1	0	0	5.10	5.06	5.36	5.72	5.45	0	0	0	0	0	0	0	8.47	8.43	8.52	8.37	8.47	8.69	8.89
1378633_at	Lpxn	1	0	0	0	0	1	4.69	4.83	4.78	4.89	5.35	0	0	0	0	0	0	0	6.41	6.34	6.64	6.76	6.67	6.95	6.66
1378644_at	---	1	0	0	0	1	0	4.92	4.68	4.43	5.12	4.55	0	0	0	0	0	0	0	5.58	5.79	5.90	5.81	5.69	5.69	5.59

Probeset ID	Gene Symbol	Islet Data Set (log2 ratio ≥0.263; FDR <20%) union n=5325 1=YES 0=NO	Islet DRlyp/lyp ND vs ND+Lp299v (log2 ratio ≥0.263; FDR<20%) n=429 1=YES 0=NO	Islet DRlyp/lyp ND vs HCD (log2 ratio ≥0.263; FDR<20%) n=2247 1=YES 0=NO	Islet DRlyp/lyp ND vs HCD+Lp299v (log2 ratio ≥0.263; FDR<20%) n=1595 1=YES 0=NO	Islet DRlyp/lyp HCD vs HCD+Lp299v (log2 ratio ≥0.263; FDR<20%) n=3026 1=YES 0=NO	Islet DRlyp/lyp ND vs Flyp/lyp ND (log2 ratio ≥ 0.263; FDR<20%) n=1950 1=YES 0=NO	Islet Mean Log2 Intensity DRlyp/lyp ND	Islet Mean Log2 Intensity DRlyp/lyp ND+Lp299v	Islet Mean Log2 Intensity DRlyp/lyp HCD	Islet Mean Log2 Intensity DRlyp/lyp HCD+Lp299v	Islet Mean Log2 Intensity Flyp40 ND	Rinm5f data union of all conditions n=3705 1=YES 0=NO	Rinm5f 30 µM propionate/6 µM butyrate vs Media (FDR<20%; 1.5 fold) n=3 1=YES 0=NO	Rinm5f 60 µM propionate/12 µM butyrate vs Media (FDR<20%; 1.5 fold) n=18 1=YES 0=NO	Rinm5f 120 µM propionate/24 µM butyrate vs Media (FDR<20%; 1.5 fold) n=164 1=YES 0=NO	Rinm5f 240 µM propionate/48 µM butyrate vs Media (FDR<20%; 1.5 fold) n=268 1=YES 0=NO	Rinm5f 1500 µM propionate/300 µM butyrate vs Media (FDR<20%; 1.5 fold) n=2491 1=YES 0=NO	Rinm5f 500 µM propionate/500 µM butyrate vs Media (FDR<20%; 1.5 fold) n=3001 1=YES 0=NO	Rinm5f Mean Log2 Intensity Media only	Rinm5f Mean Log2 Intensity 30 µM propionate/6 µM butyrate	Rinm5f Mean Log2 Intensity 60 µM propionate/12 µM butyrate	Rinm5f Mean Log2 Intensity 120 µM propionate/24 µM butyrate	Rinm5f Mean Log2 Intensity 240 µM propionate/48 µM butyrate	Rinm5f Mean Log2 Intensity 1500 µM propionate/300 µM butyrate	Rinm5f Mean Log2 Intensity 500 µM propionate/500 µM butyrate
1378656_at	Bbs5	1	0	0	0	0	1	5.16	5.07	5.63	5.06	5.72	0	0	0	0	0	0	0	7.65	7.66	7.61	7.65	7.58	7.48	6.81
1378666_at	Arhgap21	1	0	0	1	0	0	5.40	5.77	5.43	5.94	5.36	0	0	0	0	0	0	0	5.39	5.09	5.17	5.22	5.04	5.12	5.09
1378692_at	Pax8	1	0	1	0	0	0	6.70	6.73	6.19	6.39	6.50	0	0	0	0	0	0	0	5.97	5.97	5.76	5.69	5.83	5.96	6.12
1378714_at	Mrap2	1	0	1	0	1	0	6.98	7.00	5.93	7.07	6.68	0	0	0	0	0	0	0	4.32	4.32	4.30	4.20	4.34	4.49	4.86
1378742_at	---	1	0	0	0	0	1	5.40	5.57	5.78	5.43	5.87	0	0	0	0	0	0	0	7.05	6.96	7.11	7.04	6.99	6.49	5.61
1378745_at	Per3	1	0	0	1	0	1	6.55	6.52	6.57	5.94	5.84	0	0	0	0	0	0	0	7.43	7.50	7.62	7.32	7.44	7.52	6.59
1378765_at	Mul1	1	0	0	0	1	0	5.74	5.71	5.39	6.03	5.69	0	0	0	0	0	0	0	5.91	5.87	6.01	6.12	6.04	6.02	5.93
1378797_at	---	1	0	1	0	0	0	5.21	5.08	4.51	5.06	4.85	0	0	0	0	0	0	0	4.71	4.84	4.79	4.59	4.63	4.98	4.45
1378800_at	---	1	0	0	1	0	0	4.19	4.60	4.38	4.69	4.60	0	0	0	0	0	0	0	6.69	6.54	6.69	6.73	6.66	6.47	6.58
1378818_at	Lgi1	1	1	0	0	0	0	5.23	5.71	5.25	5.42	5.22	0	0	0	0	0	0	0	5.01	4.91	4.96	4.79	4.83	4.83	4.68
1378842_at	Gabarapl1	1	0	1	0	1	0	6.81	6.64	7.47	6.45	6.75	0	0	0	0	0	0	0	7.33	7.46	7.49	7.46	7.47	7.38	6.40
1378863_at	---	1	0	1	0	0	0	4.86	4.76	5.40	4.77	4.97	0	0	0	0	0	0	0	7.24	7.06	7.34	7.34	7.35	7.53	6.82
1378875_a_at	Atat1	1	0	0	0	0	1	6.09	6.40	6.31	6.35	6.61	0	0	0	0	0	0	0	7.16	7.21	7.34	7.26	7.21	6.97	6.91
1378881_at	---	1	0	0	1	1	0	4.61	4.87	4.95	4.09	4.73	0	0	0	0	0	0	0	5.53	5.34	5.57	5.61	5.61	5.56	4.28
1378896_at	Slc30a2	1	1	1	1	1	1	6.63	5.82	4.54	5.45	5.13	0	0	0	0	0	0	0	4.63	4.59	4.57	4.50	4.58	4.53	4.30
1378899_at	Slc35d3	1	0	1	0	1	0	8.04	8.27	8.65	7.79	8.14	0	0	0	0	0	0	0	4.45	4.56	4.69	4.56	4.66	4.46	4.32
1378900_at	Kat2a	1	0	0	0	1	0	7.54	7.47	7.23	7.85	7.38	0	0	0	0	0	0	0	9.28	9.24	9.41	9.37	9.32	9.04	8.96
1378921_at	Cbfa2t3	1	0	0	1	1	0	6.57	6.51	6.26	7.08	6.38	0	0	0	0	0	0	0	5.04	5.13	5.31	5.03	5.24	5.27	5.30
1378927_at	---	1	0	1	0	1	0	11.43	11.41	12.05	11.25	11.78	0	0	0	0	0	0	0	9.30	9.35	9.44	9.55	9.57	9.77	9.64
1378930_a_at	LOC103692220 /	1	0	1	0	0	0	4.69	5.07	5.35	4.94	5.05	0	0	0	0	0	0	0	6.14	6.32	6.36	6.28	6.49	6.38	6.06
1378936_at	Cstf2	1	0	1	0	0	0	3.50	3.67	4.06	3.64	3.85	0	0	0	0	0	0	0	8.69	8.62	8.87	8.94	8.89	8.64	8.10
1378943_at	---	1	0	0	0	1	0	5.28	5.47	4.92	5.72	5.02	0	0	0	0	0	0	0	5.17	5.27	5.49	5.32	5.41	5.72	5.59
1378958_at	---	1	0	1	0	1	0	7.76	7.72	8.43	7.60	7.94	0	0	0	0	0	0	0	8.31	8.25	8.40	8.52	8.44	8.06	8.05
1378972_at	Wwc1	1	0	0	1	1	0	5.62	5.36	5.50	6.10	5.47	0	0	0	0	0	0	0	6.27	6.21	6.24	6.35	6.26	6.28	5.93
1378993_at	---	1	0	1	0	0	0	4.99	4.89	4.44	4.76	4.93	0	0	0	0	0	0	0	6.30	6.19	6.38	6.27	6.30	6.39	6.12
1379008_at	---	1	0	0	1	1	0	5.96	5.97	6.29	5.34	6.19	0	0	0	0	0	0	0	7.18	7.12	7.15	7.06	7.18	7.65	7.30
1379027_at	Wwc1	1	0	0	1	0	0	5.62	5.61	5.82	6.27	5.83	0	0	0	0	0	0	0	6.87	6.76	6.82	6.93	6.95	6.95	6.52
1379036_at	Trdmt1	1	0	1	0	0	0	4.36	4.66	4.98	4.43	4.53	0	0	0	0	0	0	0	6.64	6.70	6.89	7.01	6.95	6.82	5.73
1379057_at	Proser2	1	0	0	1	1	1	7.11	7.01	6.85	7.72	6.65	0	0	0	0	0	0	0	6.35	6.21	6.29	6.27	6.10	5.96	5.88
1379058_at	---	1	0	1	0	1	0	5.87	5.94	6.75	5.41	6.14	0	0	0	0	0	0	0	9.39	9.24	9.38	9.42	9.29	9.21	8.72
1379062_at	Nkapd1	1	0	1	0	0	0	5.01	5.19	5.63	5.31	5.21	0	0	0	0	0	0	0	7.45	7.37	7.44	7.48	7.48	7.29	7.05
1379073_at	Slc25a23	1	0	0	1	1	0	7.55	7.57	7.20	8.09	7.29	0	0	0	0	0	0	0	8.43	8.41	8.40	8.17	8.26	8.41	8.31
1379074_at	Lto1	1	0	0	1	0	0	5.35	5.54	5.84	5.84	5.64	0	0	0	0	0	0	0	8.73	8.79	8.89	8.98	8.97	8.67	8.02
1379089_at	---	1	0	0	0	1	0	5.94	6.29	5.50	6.22	5.73	0	0	0	0	0	0	0	6.25	6.46	6.56	6.17	6.23	6.50	6.27
1379092_at	Kcna2	1	0	0	1	0	1	5.25	5.39	5.76	6.24	6.25	0	0	0	0	0	0	0	7.02	7.06	7.13	7.07	7.11	6.92	6.86
1379101_at	Dhx36	1	0	1	0	1	0	6.86	6.86	7.81	6.86	7.32	0	0	0	0	0	0	0	9.19	9.21	9.27	9.33	9.38	9.37	8.64
1379112_at	Arid4a	1	0	0	1	0	0	4.56	4.69	4.69	5.03	4.56	0	0	0	0	0	0	0	4.63	4.52	4.63	4.66	4.68	4.94	5.10
1379117_at	---	1	0	0	1	1	0	4.46	4.67	4.34	5.25	4.32	0	0	0	0	0	0	0	4.75	4.69	4.83	4.86	4.90	5.05	4.73
1379122_at	---	1	0	1	0	0	0	4.98	4.65	4.44	4.86	4.69	0	0	0	0	0	0	0	4.73	4.89	4.80	4.59	4.52	4.71	4.58
1379125_at	---	1	0	0	1	1	0	4.00	4.11	4.01	4.61	4.00	0	0	0	0	0	0	0	4.20	4.22	4.38	4.18	4.16	4.31	4.17
1379126_at	Prune2	1	0	0	1	1	1	7.87	8.06	7.62	8.38	8.39	0	0	0	0	0	0	0	7.53	7.48	7.54	7.75	7.67	8.00	8.11
1379150_at	---	1	0	1	0	1	0	4.65	4.93	5.23	4.50	4.81	0	0	0	0	0	0	0	6.12	6.23	6.33	6.18	6.25	6.10	6.17
1379170_at	---	1	0	0	0	0	1	3.64	3.62	3.52	3.56	4.18	0	0	0	0	0	0	0	4.89	4.84	5.01	4.76	4.85	4.86	4.87
1379175_at	---	1	0	0	1	0	1	4.54	4.74	4.61	5.09	5.38	0	0	0	0	0	0	0	4.99	4.81	5.03	5.17	4.92	4.90	4.44
1379195_s_at	Lig1	1	0	0	0	1	0	6.24	6.18	5.77	6.57	5.89	0	0	0	0	0	0	0	9.21	9.18	9.29	9.21	9.21	9.05	8.81
1379200_at	Dzip3	1	0	0	0	0	1	4.51	4.53	4.62	4.71	5.13	0	0	0	0	0	0	0	4.90	4.74	4.96	5.00	4.89	4.72	5.10
1379206_at	Tmem38a	1	0	0	1	1	0	6.99	6.98	7.15	6.40	6.95	0	0	0	0	0	0	0	7.33	7.29	7.28	7.19	7.22	7.29	6.40
1379214_at	Grik3	1	0	0	0	0	1	2.91	2.87	2.90	2.82	3.58	0	0	0	0	0	0	0	7.47	7.76	7.48	7.52	7.83	7.87	7.28
1379217_at	Acvr1	1	0	1	0	0	0	6.15	6.44	6.76	6.42	6.48	0	0	0	0	0	0	0	6.06	6.02	6.15	5.91	6.17	6.08	7.11
1379223_x_at	LOC100911077 /	1	0	1	0	1	1	5.68	5.64	5.01	5.80	4.66	0	0	0	0	0	0	0	4.27	4.23	4.23	4.09	4.11	4.25	3.85
1379236_at	---	1	0	1	0	0	1	6.51	6.76	7.26	6.88	7.05	0	0	0	0	0	0	0	9.71	9.76	9.84	9.92	9.92	9.60	9.03
1379238_at	Ctdspl	1	0	0	0	0	1	6.28	6.03	6.78	6.39	5.60	0	0	0	0	0	0	0	7.12	7.09	7.27	7.22	7.16	7.28	6.87
1379246_at	RGD1308742	1	0	1	0	1	1	5.63	5.36	5.08	5.93	6.19	0	0	0	0	0	0	0	4.84	4.84	4.78	4.74	4.76	4.76	3.99
1379248_at	Prcp	1	0	0	1	1	1	8.60	8.22	8.52	9.18	8.04	0	0	0	0	0	0	0	7.98	8.07	8.07	8.05	8.16	8.51	8.18
1379251_at	MYSM1	1	0	1	0	1	0	6.82	6.62	7.44	6.61	7.19	0	0	0	0	0	0	0	7.35	7.45	7.50	7.47	7.52	7.33	6.57
1379255_at	Atp6ap2	1	0	1	0	1	1	9.62	9.77	10.42	9.15	10.20	0	0	0	0	0	0	0	11.37	11.37	11.38	11.38	11.38	11.42	11.19
1379259_at	Mdm4	1	0	0	0	0	1	7.09	7.00	7.14	7.35	7.67	0	0	0	0	0	0	0	9.36	9.33	9.40	9.36	9.38	9.21	9.45
1379261_at	Ska2	1	0	1	0	1	0	6.43	6.25	7.02	6.18	6.70	0	0	0	0	0	0	0	8.26	8.20	8.23	8.25	8.10	7.89	7.85
1379271_at	Socs5	1	0	1	0	1	0	8.39	8.44	9.19	8.21	8.87	0	0	0	0	0	0	0	8.66	8.72	8.83	8.85	8.87	9.09	9.23
1379272_at	Paip1	1	0	1	0	1	0	6.52	6.51	7.09	6.16	6.61	0	0	0	0	0	0	0	8.30	8.21	8.31	8.35	8.39	8.06	7.30
1379281_at	Sostdc1	1	0	0	1	1	0	8.51	8.48	8.34	9.16	8.24	0	0	0	0	0	0	0	4.79	4.79	4.83	4.63	4.70	4.82	4.37
1379283_at	Braf	1	0	0	0	1	0	8.20	8.02	8.64	7.81	8.56	0	0	0	0	0	0	0	9.56	9.61	9.68	9.73	9.65	9.47	9.32
1379296_at	PA2G4	1	0	0	0	1	0	6.07	5.87	5.66	6.23	6.04	0	0	0	0	0	0	0	7.89	7.90	8.01	8.07	8.02	8.20	8.15
1379326_at	Adtrp	1	1	0	0	0	0	4.68	4.00	5.09	4.68	4.56	0	0	0	0	0	0	0	4.38	4.32	4.50	4.23	4.34	4.47	4.09
1379327_at	Smarca5	1	0	1	0	0	1	7.62	7.67	8.28	7.61	8.25	0	0	0	0	0	0	0	9.13	9.08	9.17	9.28	9.17	8.99	8.86
1379335_at	Paip1	1	0	1	0	1	0	8.28	8.34	8.87	8.13	8.67	0	0	0	0	0	0	0	10.54	10.56	10.56	10.58	10.57	10.27	9.77
1379344_at	Cybb	1	0	0	0	0	1	4.44	4.56	4.43	4.15	5.34	0	0	0	0	0	0	0	3.42	3.50	3.46	3.47	3.42	3.55	3.19
1379345_at	Col15a1 /// LOC	1	0	0	1	1	0	6.29	6.02	6.32	5.54	6.22	0	0	0	0	0	0	0	3.75	3.74	3.75	3.65	3.74	3.76	3.32
1379346_at	Cdc73	1	0	1	0	0	0	7.93	7.90	8.52	8.03	8.17	0	0	0	0	0	0	0	9.84	9.90	9.94	10.04	10.06	10.20	10.03
1379363_at	LOC103690059	1	0	0	0	0	1	3.57	3.49	3.49	3.53	3.09	0	0	0	0	0	0	0	3.57	3.50	3.66	3.47	3.49	3.39	3.59
1379365_at	Cxcl11	1	1	0	0	0	0	6.85	8.12	7.28	6.66	7.00	0	0	0	0	0	0	0	6.70	6.65	6.66	6.67	6.53	6.41	7.05
1379366_a_at	Bspry	1	0	1	0	1	1	7.61	7.47	6.81	7.92	7.09	0	0	0	0	0	0	0	6.91	6.95	7.12	7.11	7.00	7.15	7.51
1379374_at	Plppr4	1	0	1	0	1	0	8.41	8.47	9.55	8.19	8.61	0	0	0	0	0	0	0	9.49	9.38	9.45	9.49	9.28	8.96	8.58
1379375_at	Pdgfa	1	0	0	0	1	0	6.19	6.24	5.82	6.51	6.01	0	0	0	0	0	0	0	6.49	6.53	6.61	6.71	6.72	6.90	6.52
1379382_at	Sema3e	1	0	1	0	1	1	5.57	5.41	6.49	5.31	6.57	0	0	0	0	0	0	0	8.43	8.42	8.24	8.05	8.15	8.55	7.39
1379399_at	Cc2d1a	1	0	0	0	1	0	6.36	6.67	6.12	6.82	6.43	0	0	0	0	0	0	0	6.45	6.46	6.59	6.50	6.53	6.40	6.11
1379400_at	Aldh1l2	1	0	1	0	1	0	9.25	9.07	8.68	9.60	8.86	0	0	0	0	0	0	0	7.09	7.10	7.22	7.25	7.32	6.75	6.81
1379401_a_at	Fbxo33	1	0	0	0	1	0	6.87	6.65	7.43	6.70	6.90	0	0	0	0	0	0	0	8.93	9.03	8.98	8.96	9.05	9.19	8.68
1379402_at	Abcc4	1	0	0	0	0	1	5.18	5.10	5.27	5.14	5.71	0	0	0	0	0	0	0	7.12	7.04	7.28	7.41	7.48	7.53	8.13
1379405_at	Arhgap35	1	0	0	0	1	0	5.64	5.64	6.19	5.48	5.78	0	0	0	0	0	0	0	8.67	8.62	8.84	8.93	8.95	8.93	9.00
1379412_at	---	1	0	0	1	1	0	5.72	5.39	6.22	5.04	5.48	0	0	0	0	0	0	0	8.16	8.21	8.15	8.30	8.21	8.13	6.92
1379414_at	Ube2ql1	1	0	0	0	0	1	5.60	6.02	5.68	5.57	6.08	0	0	0	0	0	0	0	8.51	8.50	8.50	8.39	8.48	8.58	8.64
1379416_at	Auts2	1	0	1	0	0	0	5.97	6.39	6.59	6.10	5.95	0	0	0	0	0	0	0	9.41	9.37	9.44	9.33	9.41	8.93	8.33
1379419_at	Tmem184c	1	0	0	0	0	1	7.35	7.24	7.83	7.29	8.03	0	0	0	0	0	0	0	8.82	8.83	8.91	9.03	9.04	8.85	8.63
1379420_at	Dhrs7	1	0	1	0	1	1	7.58	7.67	8.23	7.52	8.33	0	0	0	0	0	0	0	11.24	11.19	11.09	11.00	11.03	10.71	10.62
1379425_at	Exo5	1	0	1	0	0	1	5.29	5.33	5.95	5.52	6.00	0	0	0	0	0	0	0	7.08	7.16	7.21	7.17	7.12	6.71	6.22
1379427_at	Pgrmc2	1	0	0	1	1	0	8.21	8.09	8.55	7.45	8.09	0	0	0	0	0	0	0	9.95	10.04	10.01	10.01	10.10	10.36	9.70
1379429_at	---	1	0	0	0	1	0	6.74	6.60	7.17	6.30	6.71	0	0	0	0	0	0	0	8.02	8.01	8.00	8.14	8.10	7.89	7.22
1379439_at	Zbtb44	1	0	0	1	1	0	5.73	5.80	5.64	6.46	5.44	0	0	0	0	0	0	0	7.54	7.54	7.65	7.80	7.59	7.36	7.44
1379443_at	Hps3	1	0	1	0	0	1	5.14	5.52	5.94	5.39	5.83	0	0	0	0	0	0	0	7.64	7.67	7.73	7.58	7.71	7.51	7.32
1379446_at	Cr2	1	0	0	0	0	1	3.20	3.30	3.14	3.22	5.55	0	0	0	0	0	0	0	4.11	3.96	3.99	3.81	3.98	3.94	3.36
1379452_at	Gas2	1	0	1	0	0	0	4.99	5.02	5.67	5.07	4.58	0	0	0	0	0	0	0	5.78	5.71	5.82	6.04	5.93	5.96	6.05
1379453_a_at	Lysmd4	1	0	0	0	0	1	3.02	3.08	3.36	3.14	4.21	0	0	0	0	0	0	0	4.56	4.62	4.51	4.46	4.46	4.17	3.59
1379461_at	Znf445	1	0	1	0	0	0	7.41	7.52	7.99	7.75	7.66	0	0	0	0	0	0	0	8.15	8.10	8.16	8.24	8.16	8.16	8.25
1379466_at	Sprn	1	0	1	0	1	1	6.05	5.85	5.42	6.08	5.58	0	0	0	0	0	0	0	6.04	6.14	6.26	6.23	6.19	5.87	5.02
1379471_at	Uba6	1	0	1	0	1	0	7.60	7.52	8.17	7.41	7.86	0	0	0	0	0	0	0	9.09	9.02	9.07	9.06	9.03	8.99	8.30
1379493_at	---	1	0	1	0	0	1	4.90	4.75	4.35	4.88	4.17	0	0	0	0	0	0	0	4.09	4.04	4.11	3.86	4.00	4.36	3.81
1379496_at	---	1	0	1	0	1	0	5.03	5.04	4.46	5.04	4.92	0	0	0	0	0	0	0	5.89	5.62	6.01	5.82	5.92	6.27	6.04
1379497_at	---	1	0	0	0	0	1	5.48	5.34	5.17	5.44	6.24	0	0	0	0	0	0	0	5.53	5.77	5.86	5.80	5.79	5.81	5.56
1379500_at	Ccdc117	1	0	0	0	0	1	7.08	6.74	7.37	7.02	6.58	0	0	0	0	0	0	0	8.76	8.80	8.84	8.94	8.89	9.01	8.44
1379506_at	---	1	1	0	0	0	0	7.46	6.77	7.57	7.16	7.02	0	0	0	0	0	0	0	7.72	7.60	7.78	7.60	7.59	7.64	7.23
1379510_at	KCNQ5	1	0	0	0	1	1	6.25	6.05	5.93	6.66	5.56	0	0	0	0	0	0	0	5.84	5.87	6.00	6.05	5.91	5.96	5.54
1379511_at	---	1	0	1	0	1	0	4.15	4.19	4.93	3.81	4.56	0	0	0	0	0	0	0	6.30	6.16	6.41	6.56	6.36	6.57	6.05
1379515_at	Pigt	1	0	0	1	1	0	6.52	6.26	6.60	5.81	6.17	0	0	0	0	0	0	0	10.07	10.13	10.09	10.02	10.03	9.99	8.44
1379521_at	Alg3	1	0	0	1	1	0	8.05	8.21	7.56	8.57	7.79	0	0	0	0	0	0	0	8.62	8.56	8.66	8.66	8.55	8.31	7.98
1379526_at	---	1	0	0	1	0	0	8.32	8.12	8.41	8.81	8.23	0	0	0	0	0	0	0	9.80	9.75	9.88	9.85	9.86	9.72	9.90
1379529_at	Hmgn5 /// Hmgi	1	0	1	0	0	0	5.64	5.72	6.22	5.60	5.71	0	0	0	0	0	0	0	6.93	6.86	7.15	7.25	7.08	7.04	6.93
1379541_at	Tmtc4	1	0	1	0	0	1	8.25	8.32	8.83	8.30	8.85	0	0	0	0	0	0	0	9.50	9.57	9.52	9.55	9.65	9.90	9.39
1379546_at	---	1	0	0	1	1	0	7.63	7.69	7.60	8.17	7.37	0	0	0	0	0	0	0	6.73	6.77	6.84	6.63	6.74	6.71	6.42
1379548_at	---	1	0	1	0	1	0	5.82	5.74	6.51	5.38	5.91	0	0	0	0	0	0	0	7.90	7.77	7.98	8.00	8.01	7.54	6.56
1379550_a_at	Gtf2ird1	1	0	1	0	0	0	7.38	7.39	7.96	7.54	7.57	0	0	0	0	0	0	0	10.29	10.34	10.38	10.42	10.43	10.40	10.24
1379556_a_at	Ggps1	1	0	1	0	0	0	5.03	5.19	5.61	5.43	5.43	0	0	0	0	0	0	0	6.44	6.27	6.52	6.47	6.46	6.14	5.63
1379561_at	Atad1	1	0	1	0	1	0	7.95	8.08	8.59	7.83	8.08	0	0	0	0	0	0	0	10.97	10.98	11.04	11.07	11.09	11.08	10.85
1379571_at	Pkp4	1	0	0	0	1	0	7.27	7.28	7.09	7.69	7.11	0	0	0	0	0	0	0	7.08	7.09	7.09	7.03	6.98	7.26	7.74
1379574_at	Lemd3	1	0	1	0	0	0	6.21	6.24	6.82	6.13	6.49	0	0	0	0	0	0	0	8.56	8.57	8.57	8.67	8.59	8.52	8.12
1379580_at	Chd6	1	0	1	0	0	0	6.60	6.58	7.32	6.63	6.89	0	0	0	0	0	0	0	8.44	8.56	8.56	8.57	8.61	8.46	8.38
1379588_at	---	1	0	0	0	0	1	6.90	6.95	7.33	7.12	7.48	0	0	0	0	0	0	0	9.36	9.23	9.43	9.39	9.38	9.02	8.47
1379595_at	Tmem60	1	0	0	0	1	0	7.24	7.12	7.61	6.91	7.46	0	0	0	0	0	0	0	9.43	9.48	9.43	9.43	9.46	9.69	9.35
1379606_at	Rab30	1	1	1	1	0	1	6.98	5.99	5.99	6.01	6.14	0	0	0	0	0	0	0	7.26	7.16	7.13	7.20	7.14	7.03	7.01
1379607_at	---	1	0	0	0	0	1	5.04	5.44	5.47	5.14	6.15	0	0	0	0	0	0	0	9.22	9.12	9.23	9.25	9.27	9.19	9.14
1379625_at	Zc2hc1a	1	0	1	0	1	0	5.88	5.81	6.51	5.61	6.02	0	0	0	0	0	0	0	8.23	8.26	8.34	8.47	8.43	8.43	8.21
1379626_at	Satb1	1	0	1	0	0	1	6.69	6.97	7.45	7.14	7.36	0	0	0	0	0	0	0	9.88	9.83	9.89	9.80	9.85	10.13	9.95
1379628_at	LOC100911090 /	1	0	0	1	0	0	4.17	4.34	4.41	4.72	4.52	0	0	0	0	0	0	0	6.23	6.23	6.41	6.52	6.48	6.70	6.59
1379632_at	---	1	0	1	0	1	0	4.73	4.72	5.34	4.63	5.17	0	0	0	0	0	0	0	8.60	8.67	8.76	8.82	8.98	9.17	8.92
1379633_a_at	Uba7	1	1	0	0	0	0	4.54	5.16	4.47	4.62	4.32	0	0	0	0	0	0	0	4.80	4.83	5.04	4.81	4.76	4.62	4.48
1379650_at	Pdzd8	1	1	0	1	0	0	4.91	5.44	4.95	5.38	5.28	0	0	0	0	0	0	0	5.23	5.03	5.21	5.08	5.10	5.19	4.70
1379652_at	Ahctf1	1	0	1	0	0	0	6.09	5.99	6.83	6.19	6.40	0	0	0	0	0	0	0	7.96	7.92	8.07	8.15	8.08	7.95	8.04
1379654_at	Slx1a /// Slx1b	1	0	0	0	0	1	6.38	6.27	6.20	6.42	5.88	0	0	0	0	0	0	0	7.23	7.20	7.33	7.27	7.08	7.01	6.47
1379674_at	Cbfa2t3	1	0	0	1	1	0	6.57	6.97	6.44	7.77	6.67	0	0	0	0	0	0	0	5.92	5.91	6.08	6.00	6.02	5.98	5.89
1379687_at	Actr2	1	0	1	1	1	0	6.54	6.59	7.14	5.29	6.53	0	0	0	0	0	0	0	10.43	10.35	10.48	10.45	10.45	10.20	9.28
1379689_at	Rc3h2	1	0	1	1	1	0	6.66	6.61	7.50	5.85	6.98	0	0	0	0	0	0	0	9.40	9.28	9.48	9.53	9.46	9.33	8.94
1379690_at	---	1	0	0	1	1	0	6.23	6.09	6.53	5.41	6.02	0	0	0	0	0	0	0	8.80	8.80	8.91	8.92	8.77	8.72	8.20
1379693_at	Robo2	1	0	1	0	1	1	7.82	8.27	8.73	7.57	8.55	0	0	0	0	0	0	0	5.61	5.52	5.81	5.94	5.84	6.19	6.07
1379698_at	Nfam1	1	0	0	0	0	1	4.89	5.15	5.38	5.05	5.62	0	0	0	0	0	0	0	6.49	6.44	6.54	6.44	6.54	6.39	5.97
1379706_at	Fnbp1l	1	0	1	0	1	0	4.64	4.75	5.35	4.51	4.77	0	0	0	0	0	0	0	7.86	7.83	7.94	8.02	7.95	8.00	7.86

Probeset ID	Gene Symbol	Islet Data Set (log2 ratio ≥0.263; FDR <20%) union n=5325 1=YES 0=NO	Islet DRlyp/lyp ND vs ND+Lp299v (log2 ratio ≥0.263; FDR<20%) n=429 1=YES 0=NO	Islet DRlyp/lyp ND vs HCD (log2 ratio ≥0.263; FDR<20%) n=2247 1=YES 0=NO	Islet DRlyp/lyp ND vs HCD+Lp299v (log2 ratio ≥0.263; FDR<20%) n=1595 1=YES 0=NO	Islet DRlyp/lyp HCD vs HCD+Lp299v (log2 ratio ≥0.263; FDR<20%) n=3026 1=YES 0=NO	Islet DRlyp/lyp ND vs Flyp/lyp ND (log2 ratio ≥ 0.263; FDR<20%) n=1950 1=YES 0=NO	Islet Mean Log2 Intensity DRlyp/lyp ND	Islet Mean Log2 Intensity DRlyp/lyp ND+Lp299v	Islet Mean Log2 Intensity DRlyp/lyp HCD	Islet Mean Log2 Intensity DRlyp/lyp HCD+Lp299v	Islet Mean Log2 Intensity Flyp40 ND	Rinm5f data union of all conditions n=3705 1=YES 0=NO	Rinm5f 30 µM propionate/ 6 µM butyrate vs Media (FDR<20%; 1.5 fold) n=3 1=YES 0=NO	Rinm5f 60 µM propionate/ 12 µM butyrate vs Media (FDR<20%; 1.5 fold) n=18 1=YES 0=NO	Rinm5f 120 µM propionate/ 24 µM butyrate vs Media (FDR<20%; 1.5 fold) n=164 1=YES 0=NO	Rinm5f 240 µM propionate /48 µM butyrate vs Media (FDR<20%; 1.5 fold) n=268 1=YES 0=NO	Rinm5f 1500 µM propionate/ 300 µM butyrate vs Media (FDR<20%; 1.5 fold) n=2491 1=YES 0=NO	Rinm5f 500 µM propionate/ 500 µM butyrate vs Media (FDR<20%; 1.5 fold) n=3001 1=YES 0=NO	Rinm5f Mean Log2 Intensity Media only	Rinm5f Mean Log2 Intensity 30 µM propionate/ 6 µM butyrate	Rinm5f Mean Log2 Intensity 60 µM propionate/ 12 µM butyrate	Rinm5f Mean Log2 Intensity 120 µM propionate/ 24 µM butyrate	Rinm5f Mean Log2 Intensity 240 µM propionate/ 48 µM butyrate	Rinm5f Mean Log2 Intensity 1500 µM propionate /300 µM butyrate	Rinm5f Mean Log2 Intensity 500 µM propionate/ 500 µM butyrate
1379715_at	U2surp	1	0	1	0	0	0	7.10	7.26	7.67	7.46	7.32	0	0	0	0	0	0	0	8.72	8.71	8.74	8.69	8.66	8.53	8.39
1379720_at	---	1	1	1	0	0	1	5.44	5.93	6.11	5.87	6.28	0	0	0	0	0	0	0	9.05	9.14	9.15	9.19	9.21	8.86	8.61
1379723_at	Crebzf	1	0	1	0	1	0	7.50	7.37	8.20	7.49	7.74	0	0	0	0	0	0	0	9.66	9.69	9.71	9.71	9.72	9.41	8.89
1379724_at	LOC100912785	1	0	1	0	1	1	7.46	7.58	8.17	7.39	8.28	0	0	0	0	0	0	0	7.16	7.12	7.16	7.28	7.15	6.86	6.79
1379728_at	Pabpc1	1	0	1	0	1	0	7.12	7.12	6.61	7.18	6.98	0	0	0	0	0	0	0	8.19	8.24	8.11	7.89	7.99	7.98	7.68
1379729_at	Cpsf6	1	0	1	1	1	0	6.18	6.17	7.15	5.17	6.38	0	0	0	0	0	0	0	9.72	9.69	9.74	9.82	9.81	9.58	8.84
1379730_at	Atp6v1h	1	0	0	0	1	0	7.78	7.52	8.26	7.50	7.87	0	0	0	0	0	0	0	10.26	10.27	10.27	10.35	10.26	10.26	10.03
1379739_at	Slc4a7	1	0	1	0	1	0	6.88	7.03	7.74	7.03	7.33	0	0	0	0	0	0	0	8.45	8.53	8.46	8.31	8.34	8.76	8.59
1379748_at	Ifi44l	1	0	0	0	0	1	3.62	3.89	3.85	3.84	5.07	0	0	0	0	0	0	0	6.69	6.69	6.91	7.23	6.99	6.90	6.61
1379750_at	Robo2	1	0	1	0	1	1	5.31	5.46	6.15	5.28	5.96	0	0	0	0	0	0	0	4.71	4.67	4.70	4.72	4.72	4.84	4.76
1379754_at	Stau2	1	0	1	0	1	0	5.93	6.09	6.68	5.98	5.92	0	0	0	0	0	0	0	8.14	8.31	8.31	8.28	8.31	7.79	7.64
1379768_at	---	1	0	0	1	1	0	5.48	5.12	5.46	6.03	5.18	0	0	0	0	0	0	0	4.54	4.50	4.67	4.49	4.65	4.57	4.91
1379771_at	---	1	0	1	0	0	0	6.08	6.04	5.54	5.75	5.91	0	0	0	0	0	0	0	4.67	4.71	4.70	4.48	4.51	4.62	3.99
1379772_at	Aplnr	1	0	0	0	0	1	5.02	5.06	4.58	5.09	4.55	0	0	0	0	0	0	0	4.03	3.91	3.94	3.91	3.77	3.86	3.54
1379795_at	Trappc13	1	0	0	1	1	0	6.52	6.42	6.93	5.83	6.49	0	0	0	0	0	0	0	9.40	9.46	9.49	9.49	9.47	9.39	8.53
1379810_at	Aimp1	1	0	0	1	0	0	7.82	8.02	7.80	8.33	7.73	0	0	0	0	0	0	0	9.60	9.62	9.70	9.80	9.69	9.61	9.00
1379817_at	Purg	1	0	1	0	1	1	5.28	5.46	6.26	5.50	5.96	0	0	0	0	0	0	0	8.31	8.52	8.57	8.57	8.65	8.89	8.96
1379822_at	Pnisr	1	0	1	1	1	0	5.59	5.55	6.23	4.56	5.53	0	0	0	0	0	0	0	8.04	7.98	8.07	8.10	8.02	7.73	6.65
1379825_at	Poc1b	1	0	1	0	0	0	6.09	6.25	6.73	6.15	6.50	0	0	0	0	0	0	0	8.07	8.07	8.13	8.12	8.14	8.10	7.80
1379834_at	---	1	1	1	1	1	1	7.91	9.41	8.50	9.56	8.42	0	0	0	0	0	0	0	6.62	6.67	6.62	6.61	6.49	6.44	5.62
1379842_at	St7l	1	0	0	0	1	0	4.51	4.63	4.84	4.09	4.49	0	0	0	0	0	0	0	8.52	8.53	8.57	8.52	8.59	8.32	7.34
1379850_at	Psmc6	1	0	0	0	0	1	6.96	7.02	7.21	7.24	7.48	0	0	0	0	0	0	0	9.28	9.41	9.41	9.49	9.46	9.29	8.54
1379859_at	---	1	0	1	0	1	0	4.89	5.03	5.79	4.45	4.46	0	0	0	0	0	0	0	7.18	7.34	7.38	7.35	7.33	6.90	5.38
1379874_at	Mrpl51	1	0	0	0	1	0	6.55	6.45	6.94	6.16	6.88	0	0	0	0	0	0	0	8.50	8.44	8.52	8.59	8.59	8.51	7.82
1379903_at	---	1	0	0	0	0	1	8.03	8.30	8.41	8.10	7.50	0	0	0	0	0	0	0	9.57	9.57	9.66	9.67	9.68	9.62	9.52
1379912_at	Cnot6l	1	0	1	0	0	0	7.12	6.99	7.74	7.33	7.42	0	0	0	0	0	0	0	9.09	9.00	9.11	9.14	9.08	8.72	8.85
1379917_at	---	1	0	1	0	0	0	6.07	5.77	6.72	6.23	6.36	0	0	0	0	0	0	0	8.61	8.79	8.83	8.82	8.90	9.00	8.54
1379936_at	---	1	0	1	0	1	0	4.73	4.88	4.04	4.65	4.52	0	0	0	0	0	0	0	3.94	3.95	4.00	3.88	3.99	3.97	3.60
1379948_at	---	1	0	0	0	0	1	5.85	5.94	5.64	6.07	6.40	0	0	0	0	0	0	0	6.58	6.33	6.49	6.44	6.33	6.33	6.57
1379951_at	Shprh	1	0	1	0	1	0	5.43	5.58	6.08	5.12	5.75	0	0	0	0	0	0	0	7.24	7.23	7.49	7.56	7.55	7.39	7.17
1379959_at	Get1	1	0	1	1	1	0	6.02	6.01	6.63	5.43	6.32	0	0	0	0	0	0	0	8.71	8.76	8.80	8.86	8.80	8.81	7.72
1379960_at	---	1	1	1	1	0	1	6.97	6.15	6.01	6.43	6.25	0	0	0	0	0	0	0	4.00	3.93	3.95	3.89	3.90	4.35	4.06
1379964_at	---	1	0	0	0	0	1	4.38	4.50	4.78	4.52	5.78	0	0	0	0	0	0	0	6.76	6.76	6.94	6.99	7.03	7.14	6.67
1379967_at	Znf367	1	0	1	0	0	1	5.37	5.40	6.11	5.50	5.95	0	0	0	0	0	0	0	9.32	9.27	9.38	9.46	9.39	9.17	8.87
1379982_at	Nrip1	1	0	0	1	0	0	6.19	6.39	6.64	6.87	6.49	0	0	0	0	0	0	0	8.38	8.51	8.51	8.51	8.62	8.57	7.96
1379996_at	---	1	0	0	0	1	0	6.65	6.52	7.20	6.36	6.93	0	0	0	0	0	0	0	8.35	8.28	8.54	8.59	8.56	8.69	8.56
1380001_at	Pnn	1	0	1	0	1	0	7.70	7.68	8.43	7.44	7.71	0	0	0	0	0	0	0	9.60	9.51	9.64	9.64	9.60	9.30	7.95
1380021_at	Dennd2d	1	0	1	0	1	0	5.94	5.86	5.26	6.11	5.61	0	0	0	0	0	0	0	6.44	6.50	6.52	6.62	6.70	6.88	6.83
1380033_at	---	1	0	0	1	1	0	6.27	5.93	6.65	5.69	6.51	0	0	0	0	0	0	0	7.73	7.72	7.80	7.91	7.92	8.05	7.58
1380035_at	Caap1	1	0	1	0	0	0	6.45	6.59	7.21	6.65	6.80	0	0	0	0	0	0	0	8.29	8.26	8.35	8.48	8.44	8.63	8.29
1380041_at	---	1	0	1	0	0	0	4.58	4.52	5.19	4.97	4.95	0	0	0	0	0	0	0	5.67	5.38	5.89	5.77	5.71	5.61	5.84
1380045_at	Pdp2	1	0	1	0	1	0	5.96	6.22	5.40	6.04	6.01	0	0	0	0	0	0	0	5.99	5.98	6.15	5.95	6.01	6.04	5.88
1380050_at	LOC102546810	1	0	0	0	0	1	4.54	4.39	4.74	4.29	5.45	0	0	0	0	0	0	0	4.93	4.77	4.85	4.85	4.85	4.74	4.27
1380053_at	Tm9sf3	1	0	0	1	1	0	7.88	7.83	8.27	7.31	7.78	0	0	0	0	0	0	0	9.72	9.71	9.90	10.04	10.04	10.08	9.29
1380067_at	Myo5c	1	0	1	0	1	1	6.75	6.56	6.13	7.12	5.54	0	0	0	0	0	0	0	4.75	4.82	4.65	4.63	4.58	4.68	4.13
1380091_at	Zfand4	1	0	1	0	1	0	3.60	3.67	4.19	3.42	3.84	0	0	0	0	0	0	0	5.92	5.99	6.18	6.13	6.11	5.76	5.71
1380098_at	Clock	1	0	0	0	1	0	4.27	4.10	4.65	3.96	4.28	0	0	0	0	0	0	0	7.20	7.11	7.13	7.21	7.30	7.05	6.61
1380101_at	Papolg	1	0	1	0	1	0	5.03	4.97	5.75	4.80	5.24	0	0	0	0	0	0	0	8.29	8.26	8.31	8.37	8.33	7.97	7.54
1380116_at	Foxn2	1	0	1	0	1	0	6.94	6.79	7.68	6.95	7.30	0	0	0	0	0	0	0	9.05	9.06	9.06	9.07	9.07	8.95	8.82
1380123_at	Tmem79	1	0	0	0	1	0	5.60	5.56	5.09	5.81	5.43	0	0	0	0	0	0	0	4.51	4.47	4.49	4.41	4.42	4.58	4.24
1380131_at	Wdr37	1	0	1	0	0	0	4.87	4.89	5.57	5.01	5.13	0	0	0	0	0	0	0	7.22	7.19	7.23	7.21	7.27	7.25	7.03
1380143_at	---	1	0	1	0	0	0	4.44	4.19	3.91	4.29	3.99	0	0	0	0	0	0	0	5.06	5.05	5.06	4.88	4.98	4.95	4.89
1380144_at	---	1	0	0	1	0	0	6.55	6.58	6.67	7.04	6.56	0	0	0	0	0	0	0	7.94	7.83	8.00	8.13	8.05	8.25	8.73
1380154_at	Immt	1	0	1	0	0	0	5.79	5.83	6.37	5.84	5.88	0	0	0	0	0	0	0	6.53	6.71	6.85	6.69	6.86	6.10	6.17
1380157_at	Ube2g2	1	0	0	1	1	0	6.04	5.74	6.49	5.56	5.72	0	0	0	0	0	0	0	8.54	8.66	8.73	8.81	8.76	8.89	8.28
1380159_at	Twnk	1	0	0	1	1	0	5.23	5.31	5.10	5.80	4.96	0	0	0	0	0	0	0	7.54	7.44	7.57	7.63	7.68	7.40	7.52
1380172_at	Kif5c	1	1	0	0	0	0	6.33	6.91	6.73	6.65	6.42	0	0	0	0	0	0	0	9.26	9.11	9.25	9.16	9.07	9.22	9.91
1380174_at	---	1	0	1	0	0	0	5.91	5.70	6.67	6.02	6.16	0	0	0	0	0	0	0	7.76	7.92	7.92	8.01	8.12	8.28	7.92
1380183_at	Prss3	1	0	0	0	0	1	13.55	13.35	13.22	13.21	14.11	0	0	0	0	0	0	0	5.23	5.46	5.26	5.12	5.22	5.11	5.10
1380205_at	Clint1	1	0	0	0	0	1	5.42	5.62	5.54	5.66	5.84	0	0	0	0	0	0	0	5.60	5.68	5.67	5.81	5.76	5.73	5.01
1380222_at	---	1	0	0	0	1	0	5.68	5.62	5.18	5.77	5.61	0	0	0	0	0	0	0	5.80	5.73	5.75	5.42	5.46	5.64	5.38
1380238_at	---	1	0	0	1	1	0	6.17	6.50	5.97	6.77	6.16	0	0	0	0	0	0	0	6.59	6.64	6.80	6.56	6.58	6.90	6.48
1380242_at	Lrp5	1	0	0	0	1	0	5.94	5.68	5.49	6.12	5.64	0	0	0	0	0	0	0	4.80	4.73	4.47	4.49	4.55	4.69	4.72
1380251_at	---	1	1	0	1	1	1	4.69	4.17	4.67	3.91	4.17	0	0	0	0	0	0	0	4.06	3.85	4.00	4.19	4.16	4.17	3.47
1380255_at	Ubxn2b	1	0	1	0	1	1	6.38	6.34	7.10	6.20	6.85	0	0	0	0	0	0	0	8.00	8.04	8.18	8.34	8.38	8.45	8.32
1380256_at	Polr3c	1	0	0	0	0	1	6.72	6.42	6.75	6.76	6.16	0	0	0	0	0	0	0	8.41	8.47	8.49	8.42	8.44	8.53	8.25
1380283_at	Tshz1	1	0	0	0	0	1	7.32	7.67	7.62	7.72	7.85	0	0	0	0	0	0	0	9.11	9.09	9.18	9.20	9.23	9.63	9.51
1380327_at	Helb	1	0	1	0	0	1	5.92	6.04	6.55	6.06	6.41	0	0	0	0	0	0	0	7.41	7.39	7.49	7.49	7.47	7.49	7.63
1380332_at	---	1	0	1	0	1	0	5.42	5.63	6.07	4.94	5.39	0	0	0	0	0	0	0	5.85	5.77	5.99	5.91	6.02	5.86	5.49
1380336_at	Irak3	1	0	1	0	0	0	4.63	4.32	4.07	4.25	4.35	0	0	0	0	0	0	0	7.57	7.52	7.61	7.65	7.64	7.51	7.27
1380340_at	---	1	0	0	0	0	1	5.24	5.19	4.89	5.21	4.48	0	0	0	0	0	0	0	3.83	3.98	4.07	3.87	3.91	3.85	3.61
1380345_at	Rimkla	1	0	0	0	0	1	4.80	5.06	5.02	4.89	5.43	0	0	0	0	0	0	0	10.68	10.67	10.73	10.65	10.65	10.82	10.76
1380346_at	Serpinb1	1	1	0	0	0	1	9.58	9.11	9.38	9.36	8.74	0	0	0	0	0	0	0	6.09	6.15	6.07	5.96	5.88	6.31	5.78
1380347_at	Bche	1	0	0	0	0	1	5.08	5.13	5.40	5.13	5.72	0	0	0	0	0	0	0	3.76	3.77	3.87	3.75	3.67	3.97	3.58
1380366_at	Igh-6	1	0	0	0	0	1	3.52	3.51	3.37	3.63	5.11	0	0	0	0	0	0	0	3.76	3.69	3.60	3.43	3.50	3.56	2.94
1380371_at	Nipbl	1	0	0	1	0	0	8.19	8.53	8.54	8.72	8.38	0	0	0	0	0	0	0	9.23	9.09	9.24	9.25	9.15	9.16	9.24
1380373_at	Gmps	1	0	0	1	1	1	6.71	6.47	6.75	5.70	6.18	0	0	0	0	0	0	0	8.76	8.75	8.77	8.91	8.75	8.61	7.67
1380376_at	Osbpl6	1	0	0	0	0	1	5.21	5.23	5.51	5.42	4.74	0	0	0	0	0	0	0	6.14	6.12	6.11	6.06	6.19	6.06	6.59
1380381_at	Alg2	1	0	1	1	1	0	5.59	5.75	6.28	4.69	5.58	0	0	0	0	0	0	0	7.96	7.99	8.15	8.29	8.19	8.08	6.62
1380387_at	Foxp2	1	0	0	1	1	1	5.53	5.21	5.25	6.34	5.00	0	0	0	0	0	0	0	6.29	6.32	6.54	6.46	6.37	6.77	6.70
1380389_at	---	1	0	1	0	1	0	9.08	8.69	8.37	9.48	8.63	0	0	0	0	0	0	0	9.04	9.17	9.05	8.95	9.15	9.30	8.78
1380398_at	---	1	0	1	0	1	0	5.93	5.82	5.39	6.09	5.59	0	0	0	0	0	0	0	6.14	6.33	6.27	6.06	6.11	5.80	5.67
1380399_at	---	1	0	0	1	1	1	6.26	6.38	6.82	5.70	5.74	0	0	0	0	0	0	0	7.39	7.28	7.55	7.21	7.29	7.01	5.94
1380406_at	---	1	0	0	0	0	1	4.77	5.16	4.98	4.93	5.25	0	0	0	0	0	0	0	5.72	5.76	5.87	5.78	5.80	5.59	5.62
1380407_at	C5orf15	1	0	0	0	0	1	8.89	9.07	9.10	8.94	7.77	0	0	0	0	0	0	0	9.88	9.97	9.97	9.95	10.04	10.07	9.57
1380416_at	Znf24	1	0	1	0	1	0	6.48	6.46	7.15	6.36	6.73	0	0	0	0	0	0	0	9.06	9.09	9.03	9.15	9.17	8.98	8.46
1380426_at	Ankrd52	1	0	0	0	0	1	3.90	3.59	3.44	3.50	3.41	0	0	0	0	0	0	0	3.70	3.75	3.73	3.58	3.62	3.58	3.35
1380439_at	LOC100912143	1	0	1	0	0	1	5.05	4.72	4.37	4.89	4.49	0	0	0	0	0	0	0	4.26	4.15	4.24	4.25	4.15	4.14	3.64
1380447_a_at	Mrps18c	1	1	0	0	0	0	5.87	5.35	6.14	5.75	5.93	0	0	0	0	0	0	0	8.52	8.66	8.53	8.71	8.70	8.70	7.94
1380459_at	Nacc2	1	0	0	0	1	0	7.54	7.81	7.31	8.01	7.67	0	0	0	0	0	0	0	8.65	8.68	8.65	8.62	8.59	8.84	8.56
1380464_at	---	1	0	0	1	0	1	3.90	4.02	3.92	4.44	5.29	0	0	0	0	0	0	0	4.48	4.28	4.31	4.48	4.52	4.34	3.62
1380486_at	Sec22c	1	0	0	0	1	0	8.75	8.74	8.57	9.20	8.63	0	0	0	0	0	0	0	8.18	8.24	8.31	8.18	8.26	8.49	8.64
1380490_at	Ipo8	1	0	0	0	0	1	6.13	6.45	6.68	6.57	6.96	0	0	0	0	0	0	0	7.98	7.90	8.14	8.21	8.15	7.84	7.32
1380493_at	Tet3	1	1	0	1	1	0	5.51	6.10	5.50	6.36	5.79	0	0	0	0	0	0	0	6.44	6.33	6.38	6.25	6.18	6.17	5.94
1380497_at	Ash1l	1	0	0	1	0	1	4.11	4.36	4.44	4.77	4.40	0	0	0	0	0	0	0	5.43	5.36	5.51	5.26	5.29	4.89	5.27
1380501_x_at	Fkbp2	1	0	1	0	0	0	7.22	7.23	6.66	7.15	7.06	0	0	0	0	0	0	0	7.56	7.54	7.49	7.32	7.38	7.46	7.26
1380504_at	---	1	0	0	0	0	1	4.34	4.23	4.19	4.39	3.62	0	0	0	0	0	0	0	7.55	7.44	7.53	7.54	7.38	7.39	7.04
1380526_at	Gcdh	1	1	1	1	0	1	6.52	5.96	5.95	5.81	5.80	0	0	0	0	0	0	0	6.41	6.36	6.42	6.22	6.19	6.38	5.94
1380529_at	---	1	0	1	1	1	0	4.47	4.48	5.12	3.89	4.52	0	0	0	0	0	0	0	7.26	7.17	7.32	7.43	7.26	7.11	6.44
1380533_at	App	1	0	0	1	1	1	8.73	8.31	8.71	7.27	8.13	0	0	0	0	0	0	0	11.03	11.11	11.12	11.14	11.15	11.52	10.55
1380536_at	RGD1305928	1	0	0	1	1	1	5.58	5.60	5.33	6.11	5.02	0	0	0	0	0	0	0	4.30	4.38	4.45	4.32	4.37	4.45	3.81
1380544_at	Foxp1	1	0	0	1	0	0	7.17	7.59	7.28	7.76	7.21	0	0	0	0	0	0	0	7.85	7.86	7.91	8.02	8.00	8.31	7.75
1380547_at	Clcn3	1	0	0	1	1	0	7.39	7.44	7.27	7.91	7.19	0	0	0	0	0	0	0	9.52	9.46	9.56	9.57	9.48	9.45	9.57
1380549_at	Ccar1 /// LOC10(	1	0	0	1	1	0	4.17	4.13	4.47	3.30	3.78	0	0	0	0	0	0	0	7.82	7.67	7.92	8.14	7.97	7.89	6.99
1380557_at	Bckdha	1	0	1	0	1	1	6.64	6.30	6.03	6.80	5.90	0	0	0	0	0	0	0	5.19	5.18	5.38	5.22	4.99	5.16	4.50
1380567_at	---	1	0	1	1	1	1	6.15	5.93	6.90	5.45	5.04	0	0	0	0	0	0	0	3.37	3.27	3.32	3.22	3.19	3.31	2.74
1380569_at	Rnf41	1	0	0	1	1	0	5.80	5.93	6.26	5.29	5.79	0	0	0	0	0	0	0	8.43	8.41	8.54	8.57	8.48	8.53	7.83
1380582_at	Csf1	1	0	1	0	0	0	7.89	7.86	7.23	7.71	7.77	0	0	0	0	0	0	0	8.08	8.04	8.11	7.63	7.78	8.04	7.74
1380611_at	Fkbp5	1	0	0	1	1	0	4.92	4.76	5.31	4.34	4.71	0	0	0	0	0	0	0	8.30	8.24	8.41	8.60	8.45	8.58	8.32
1380617_at	Gimap1-gimap5	1	0	0	1	0	0	4.82	4.41	4.49	4.23	4.47	0	0	0	0	0	0	0	4.04	4.06	4.15	3.87	3.99	4.00	3.44
1380623_at	Taz	1	0	0	0	1	0	6.85	6.89	7.36	6.67	7.06	0	0	0	0	0	0	0	8.70	8.73	8.78	8.76	8.75	8.75	8.31
1380646_at	Elmod2	1	0	0	1	1	0	4.54	4.44	4.73	3.80	4.40	0	0	0	0	0	0	0	5.82	5.84	6.06	5.96	5.84	5.85	5.53
1380651_at	Rnf6	1	0	0	0	0	1	6.16	5.94	6.52	6.19	6.89	0	0	0	0	0	0	0	8.92	8.84	9.03	9.06	9.01	8.88	8.39
1380658_at	---	1	0	0	1	1	0	4.12	4.21	4.01	4.88	3.77	0	0	0	0	0	0	0	4.07	4.01	4.05	3.78	3.87	3.84	3.37
1380668_at	---	1	0	0	0	0	1	4.52	4.43	4.51	4.49	4.06	0	0	0	0	0	0	0	4.21	4.20	4.08	3.98	4.03	4.11	3.94
1380681_at	Rnf180	1	0	1	0	0	0	3.63	3.58	4.20	3.57	3.72	0	0	0	0	0	0	0	6.09	6.01	6.26	6.19	5.99	5.75	5.83
1380685_at	---	1	0	1	1	1	1	4.94	4.80	5.57	4.28	5.63	0	0	0	0	0	0	0	8.22	8.09	8.32	8.36	8.29	8.12	7.44
1380692_at	Rbl1	1	0	1	0	1	1	5.42	5.39	6.14	5.34	6.05	0	0	0	0	0	0	0	8.60	8.61	8.72	8.84	8.98	8.95	8.52
1380693_at	---	1	0	1	0	1	1	5.46	5.61	6.34	5.39	4.62	0	0	0	0	0	0	0	7.22	7.20	7.28	7.01	7.10	7.41	7.04
1380701_at	---	1	1	0	1	1	0	6.10	6.59	5.76	6.85	6.33	0	0	0	0	0	0	0	6.21	6.10	6.20	5.97	5.68	5.68	5.44
1380725_at	Tspan5	1	0	1	0	0	0	6.45	6.70	7.04	6.36	6.22	0	0	0	0	0	0	0	8.86	8.85	8.96	9.03	8.88	8.83	8.21
1380726_at	Aspn /// NEWGE	1	1	1	0	1	0	4.02	3.54	5.09	3.60	3.83	0	0	0	0	0	0	0	3.27	3.13	3.25	3.31	3.15	3.24	2.78
1380730_at	Atp6v0a2 /// Tct	1	0	0	0	1	0	6.95	6.72	6.50	7.07	6.89	0	0	0	0	0	0	0	7.68	7.62	7.64	7.50	7.54	7.21	6.67
1380743_at	Cecr2	1	0	0	0	1	0	5.35	5.34	5.09	5.80	5.15	0	0	0	0	0	0	0	5.50	5.53	5.64	5.53	5.68	5.88	5.74
1380770_at	---	1	0	1	0	1	0	5.37	5.36	4.62	5.63	5.35	0	0	0	0	0	0	0	5.67	5.63	5.82	5.61	5.54	5.40	5.71
1380774_at	---	1	0	0	1	0	0	5.58	5.60	5.58	6.06	5.52	0	0	0	0	0	0	0	6.63	6.59	6.64	6.58	6.58	6.31	6.54
1380794_at	Znf770	1	0	1	0	1	0	6.33	6.07	7.04	6.05	6.60	0	0	0	0	0	0	0	8.48	8.54	8.52	8.67	8.56	8.42	8.08
1380796_at	Spire1	1	0	0	0	1	0	6.29	6.30	6.68	5.98	6.39	0	0	0	0	0	0	0	7.67	7.70	7.66	7.60	7.64	7.91	7.98
1380811_at	Ptpn4	1	0	1	0	0	0	6.36	6.19	6.92	6.27	6.48	0	0	0	0	0	0	0	8.34	8.51	8.57	8.60	8.51	8.66	8.64
1380815_at	Gorab	1	0	1	0	1	1	6.13	6.13	6.74	6.04	6.61	0	0	0	0	0	0	0	6.76	6.84	6.81	6.82	6.82	6.50	6.15
1380824_at	---	1	0	1	0	0	1	6.52	6.27	7.26	6.97	7.68	0	0	0	0	0	0	0	8.80	8.74	8.83	8.91	8.81	8.57	8.73
1380825_at	---	1	0	1	0	1	0	5.03	5.28	5.82	4.78	5.50	0	0	0	0	0	0	0	6.10	6.06	6.27	6.23	6.27	6.03	5.74
1380850_at	---	1	0	0	0	0	1	4.31	4.26	4.60	4.34	5.07	0	0	0	0	0	0	0	8.12	8.04	7.92	8.06	8.16	8.33	8.45
1380860_at	---	1	0	0	1	0	0	4.89	5.03	5.20	5.36	4.75	0	0	0	0	0	0	0	6.63	6.68	6.58	6.66	6.58	6.61	6.61
1380866_at	Ak5	1	1	1	0	1	1	7.82	7.13	7.03	7.73	5.03	0	0	0	0	0	0	0	3.52	3.46	3.39	3.40	3.39	3.42	3.27

Probeset ID	Gene Symbol	Islet Data Set (log2 ratio ≥0.263; FDR <20%) union n=5325 1=YES 0=NO	Islet DRlyp/lyp ND vs ND+Lp299v (log2 ratio ≥0.263; FDR<20%) n=429 1=YES 0=NO	Islet DRlyp/lyp ND vs HCD (log2 ratio ≥0.263; FDR<20%) n=2247 1=YES 0=NO	Islet DRlyp/lyp ND vs HCD+Lp299v (log2 ratio ≥0.263; FDR<20%) n=1595 1=YES 0=NO	Islet DRlyp/lyp HCD vs HCD+Lp299v (log2 ratio ≥0.263; FDR<20%) n=3026 1=YES 0=NO	Islet DRlyp/lyp ND vs Flyp/lyp ND (log2 ratio ≥ 0.263; FDR<20%) n=1950 1=YES 0=NO	Islet Mean Log2 Intensity DRlyp/lyp ND	Islet Mean Log2 Intensity DRlyp/lyp ND+Lp299v	Islet Mean Log2 Intensity DRlyp/lyp HCD	Islet Mean Log2 Intensity DRlyp/lyp HCD+Lp299v	Islet Mean Log2 Intensity Flyp40 ND	Rinm5f data union of all conditions n=3705 1=YES 0=NO	Rinm5f 30 µM propionate/ 6 µM butyrate vs Media (FDR<20%; 1.5 fold) n=3 1=YES 0=NO	Rinm5f 60 µM propionate/ 12 µM butyrate vs Media (FDR<20%; 1.5 fold) n=18 1=YES 0=NO	Rinm5f 120 µM propionate/ 24 µM butyrate vs Media (FDR<20%; 1.5 fold) n=164 1=YES 0=NO	Rinm5f 240 µM propionate /48 µM butyrate vs Media (FDR<20%; 1.5 fold) n=268 1=YES 0=NO	Rinm5f 1500 µM propionate/ 300 µM butyrate vs Media (FDR<20%; 1.5 fold) n=2491 1=YES 0=NO	Rinm5f 500 µM propionate/ 500 µM butyrate vs Media (FDR<20%; 1.5 fold) n=3001 1=YES 0=NO	Rinm5f Mean Log2 Intensity Media only	Rinm5f Mean Log2 Intensity 30 µM propionate/ 6 µM butyrate	Rinm5f Mean Log2 Intensity 60 µM propionate/ 12 µM butyrate	Rinm5f Mean Log2 Intensity 120 µM propionate/ 24 µM butyrate	Rinm5f Mean Log2 Intensity 240 µM propionate/ 48 µM butyrate	Rinm5f Mean Log2 Intensity 1500 µM propionate /300 µM butyrate	Rinm5f Mean Log2 Intensity 500 µM propionate/ 500 µM butyrate
1380877_at	---	1	1	1	0	0	0	5.38	4.88	4.84	5.36	5.15	0	0	0	0	0	0	0	4.98	5.05	5.16	5.02	5.05	5.05	5.60
1380882_at	---	1	0	1	0	1	0	5.26	4.89	4.63	5.20	5.11	0	0	0	0	0	0	0	4.65	4.53	4.83	4.48	4.53	4.58	4.32
1380892_at	---	1	0	1	0	0	0	5.22	4.94	4.62	4.89	4.89	0	0	0	0	0	0	0	5.82	5.81	5.81	5.63	5.65	5.80	5.41
1380903_at	LOC691170	1	1	1	0	1	0	4.99	5.75	5.98	5.15	5.37	0	0	0	0	0	0	0	6.48	6.47	6.73	6.74	6.74	6.27	5.50
1380909_at	Slc25a24	1	0	1	0	1	1	6.46	6.45	7.11	6.31	7.28	0	0	0	0	0	0	0	8.91	8.85	8.94	8.96	8.77	8.50	8.83
1380938_at	Dstyk	1	0	1	0	1	0	6.76	6.69	7.38	6.51	6.96	0	0	0	0	0	0	0	8.62	8.66	8.71	8.62	8.57	8.41	8.59
1380997_at	Ccnj /// LOC100	1	0	1	0	0	0	4.98	5.27	5.65	5.10	5.17	0	0	0	0	0	0	0	8.35	8.34	8.48	8.53	8.51	8.12	7.86
1381006_at	Hgfac	1	0	1	0	1	1	9.42	9.35	8.33	9.15	8.85	0	0	0	0	0	0	0	10.50	10.55	10.53	10.49	10.50	10.22	9.76
1381020_at	---	1	0	0	0	1	0	3.78	3.82	3.38	3.95	3.68	0	0	0	0	0	0	0	4.00	3.92	3.82	3.82	3.84	3.66	3.55
1381028_at	---	1	0	1	0	0	0	6.38	6.34	5.72	6.19	6.19	0	0	0	0	0	0	0	6.31	6.45	6.39	6.37	6.38	6.44	5.91
1381030_at	Slu7	1	0	1	0	1	0	6.93	6.80	7.57	6.46	6.82	0	0	0	0	0	0	0	8.53	8.57	8.64	8.59	8.57	8.53	7.92
1381036_at	---	1	0	1	0	1	0	4.13	4.57	4.92	3.84	4.34	0	0	0	0	0	0	0	6.38	6.35	6.50	6.20	6.27	6.36	5.04
1381044_at	---	1	1	0	0	0	0	3.75	4.28	3.93	4.04	4.10	0	0	0	0	0	0	0	5.27	5.07	5.26	5.47	5.19	5.48	5.40
1381053_at	---	1	0	0	0	1	0	5.00	4.89	4.65	5.32	4.83	0	0	0	0	0	0	0	5.23	5.35	5.27	5.18	5.27	5.41	5.16
1381065_at	LOC103690114 /	1	0	1	0	1	0	6.76	6.83	7.62	6.58	7.12	0	0	0	0	0	0	0	9.14	9.22	9.21	9.16	9.28	9.04	8.56
1381069_at	Kmt2a	1	0	0	0	1	0	4.31	4.51	4.18	4.76	4.11	0	0	0	0	0	0	0	3.84	3.77	3.92	3.91	3.85	3.99	3.45
1381077_at	---	1	0	1	0	1	0	5.57	5.58	6.22	5.47	5.81	0	0	0	0	0	0	0	7.31	7.45	7.57	7.79	7.85	7.77	7.32
1381100_at	---	1	0	0	1	1	0	7.31	7.60	7.03	7.86	7.21	0	0	0	0	0	0	0	6.81	6.74	6.67	6.57	6.49	6.56	6.48
1381121_at	---	1	0	1	1	0	1	6.55	6.70	5.50	5.92	5.92	0	0	0	0	0	0	0	9.11	9.13	9.25	8.61	9.20	9.06	5.53
1381144_at	Bpnt2	1	0	0	0	1	0	4.94	4.96	5.32	4.59	5.03	0	0	0	0	0	0	0	8.36	8.43	8.47	8.56	8.62	8.53	7.77
1381153_at	Anapc4	1	0	0	0	1	0	5.66	5.61	6.18	5.31	6.02	0	0	0	0	0	0	0	7.43	7.51	7.64	7.64	7.71	7.46	6.72
1381162_at	Septin10	1	0	1	0	0	1	6.61	6.81	7.24	6.68	7.18	0	0	0	0	0	0	0	9.06	9.16	9.03	8.88	8.86	9.06	8.71
1381163_at	---	1	0	0	0	0	1	6.12	6.10	6.57	6.41	6.95	0	0	0	0	0	0	0	8.90	8.87	9.04	8.98	9.06	9.40	8.96
1381205_at	Snapc5	1	0	1	1	1	0	5.44	5.54	6.24	4.62	5.48	0	0	0	0	0	0	0	6.83	6.92	7.06	7.11	7.13	7.17	6.08
1381228_at	---	1	0	0	0	0	1	4.64	5.08	4.61	4.30	4.14	0	0	0	0	0	0	0	5.30	5.42	5.59	5.57	5.64	5.44	4.99
1381229_at	---	1	0	1	0	0	0	3.86	3.82	4.42	3.90	4.34	0	0	0	0	0	0	0	6.99	6.96	7.05	6.93	6.99	6.89	6.62
1381252_at	Eri2	1	0	1	0	0	1	5.24	5.41	5.80	5.52	6.57	0	0	0	0	0	0	0	7.52	7.49	7.62	7.72	7.68	7.52	7.49
1381253_at	---	1	0	0	0	1	0	6.21	6.22	5.85	6.45	6.04	0	0	0	0	0	0	0	5.95	5.98	6.00	5.86	5.91	6.04	5.69
1381269_at	LOC100911661	1	0	1	0	1	1	7.58	7.39	6.21	7.60	7.11	0	0	0	0	0	0	0	3.68	3.62	3.60	3.59	3.51	3.61	3.08
1381279_at	Ripk2	1	0	1	0	1	0	3.81	3.97	4.45	3.73	3.93	0	0	0	0	0	0	0	5.81	5.86	6.07	6.19	6.14	6.17	6.00
1381327_a_at	Cr2	1	0	0	0	0	1	2.66	2.59	2.58	2.74	3.44	0	0	0	0	0	0	0	3.18	3.17	3.10	3.19	3.17	3.22	2.65
1381336_at	Insr	1	0	0	0	0	1	7.86	7.63	7.71	8.21	7.27	0	0	0	0	0	0	0	7.60	7.62	7.62	7.77	7.79	7.65	7.51
1381340_at	Rsph10b /// Rsp	1	0	1	0	0	0	4.06	3.86	3.50	3.66	3.69	0	0	0	0	0	0	0	3.88	3.98	3.94	3.74	3.81	3.97	3.55
1381386_at	---	1	0	0	0	0	1	3.65	3.44	3.58	3.66	4.58	0	0	0	0	0	0	0	7.08	7.11	7.22	7.23	7.18	7.19	7.27
1381396_s_at	Klf15	1	1	0	1	0	1	6.84	5.95	6.45	6.17	6.06	0	0	0	0	0	0	0	3.78	3.87	3.89	3.67	3.73	3.72	3.50
1381428_a_at	---	1	0	0	1	1	1	8.83	9.06	8.94	9.96	9.66	0	0	0	0	0	0	0	4.55	4.51	4.74	4.56	4.52	4.61	4.78
1381434_s_at	LOC302022	1	0	0	1	0	0	4.32	4.34	4.74	4.93	4.45	0	0	0	0	0	0	0	3.63	3.73	3.69	3.58	3.63	3.74	3.54
1381443_at	Hapln4	1	0	1	0	0	0	6.79	6.69	6.19	6.58	6.51	0	0	0	0	0	0	0	7.11	7.23	7.23	7.12	7.16	7.35	6.80
1381444_at	---	1	0	1	0	0	0	4.39	4.07	3.75	4.11	3.99	0	0	0	0	0	0	0	3.69	3.79	3.66	3.71	3.66	3.62	3.25
1381467_at	Neo1	1	0	0	0	1	0	7.09	7.09	7.49	6.68	6.97	0	0	0	0	0	0	0	6.32	6.25	6.22	6.16	6.08	6.39	6.97
1381469_a_at	Gigyf1	1	0	0	0	1	0	6.07	6.05	5.72	6.36	6.08	0	0	0	0	0	0	0	7.64	7.53	7.70	7.65	7.53	7.31	7.20
1381470_at	Kit	1	0	0	0	0	1	8.37	8.15	8.48	8.30	7.81	0	0	0	0	0	0	0	12.14	12.19	12.26	12.28	12.36	12.71	12.49
1381489_at	Sox6	1	0	0	1	0	1	5.78	6.07	6.11	6.32	6.27	0	0	0	0	0	0	0	3.55	3.49	3.52	3.52	3.45	3.60	3.13
1381496_x_at	---	1	0	0	0	1	0	4.21	4.16	4.71	4.02	4.33	0	0	0	0	0	0	0	6.57	6.54	6.65	6.63	6.56	6.45	5.88
1381504_at	Aspn /// NEWGE	1	0	0	0	1	0	4.17	3.99	4.60	3.87	3.99	0	0	0	0	0	0	0	3.19	3.18	3.27	3.30	3.12	3.24	2.93
1381515_at	---	1	0	1	0	1	0	4.42	3.98	5.30	4.43	4.46	0	0	0	0	0	0	0	7.20	7.15	7.25	7.26	7.16	6.79	6.51
1381525_at	Adar	1	0	0	0	0	1	5.54	6.00	5.88	5.86	6.27	0	0	0	0	0	0	0	6.85	6.86	6.98	6.87	6.86	6.61	6.45
1381543_at	Slc38a7	1	0	0	0	1	0	6.19	6.47	5.94	6.60	6.08	0	0	0	0	0	0	0	5.93	5.84	6.01	5.80	5.78	5.73	5.81
1381551_at	Alkbh1	1	0	0	1	0	0	4.94	4.98	5.14	5.43	4.93	0	0	0	0	0	0	0	6.57	6.61	6.63	6.74	6.64	6.70	6.39
1381553_at	---	1	1	0	1	0	1	4.42	3.36	3.91	3.54	3.61	0	0	0	0	0	0	0	3.42	3.49	3.48	3.26	3.29	3.48	2.92
1381557_at	Gna14	1	0	1	1	1	1	5.25	5.04	6.07	6.86	4.69	0	0	0	0	0	0	0	4.20	4.36	4.23	4.25	4.22	4.04	4.46
1381560_at	Clec2d	1	0	1	0	0	0	5.02	4.79	4.41	4.91	4.71	0	0	0	0	0	0	0	4.70	4.64	4.68	4.63	4.51	4.55	4.52
1381574_at	Agmo	1	0	1	1	0	0	5.38	5.17	6.17	6.53	5.33	0	0	0	0	0	0	0	4.30	4.16	4.25	4.04	4.08	4.19	3.93
1381590_at	---	1	0	0	1	0	0	5.55	5.74	5.58	6.10	5.33	0	0	0	0	0	0	0	4.53	4.63	4.73	4.37	4.49	4.54	4.24
1381593_x_at	Hla-dqa1	1	0	1	0	0	0	8.73	8.70	8.14	8.48	8.73	0	0	0	0	0	0	0	8.00	7.92	7.83	7.68	7.79	7.93	7.34
1381598_at	Ddx50	1	0	1	0	1	0	6.11	6.08	5.46	6.11	5.86	0	0	0	0	0	0	0	6.11	6.20	6.05	5.98	6.03	6.06	5.70
1381621_at	---	1	0	1	0	1	0	5.12	5.25	5.90	4.87	5.44	0	0	0	0	0	0	0	8.49	8.57	8.67	8.75	8.73	8.23	7.80
1381630_at	C11orf91	1	0	0	0	1	0	4.09	4.11	3.79	4.36	3.98	0	0	0	0	0	0	0	4.70	4.65	4.66	4.55	4.67	4.68	4.75
1381655_at	---	1	0	1	0	1	0	6.54	6.54	7.30	6.10	6.85	0	0	0	0	0	0	0	10.07	10.21	10.27	10.10	10.15	9.62	9.15
1381703_at	---	1	0	0	0	0	1	6.79	6.60	6.82	6.75	6.32	0	0	0	0	0	0	0	6.56	6.50	6.83	6.53	6.56	6.45	5.77
1381705_at	Pank3	1	0	0	0	1	0	5.72	5.63	6.00	5.28	5.68	0	0	0	0	0	0	0	6.63	6.63	6.66	6.76	6.68	6.60	6.64
1381737_at	---	1	0	1	0	0	0	5.38	5.36	6.07	5.65	5.59	0	0	0	0	0	0	0	6.10	6.11	6.36	6.28	6.29	6.14	5.66
1381746_at	---	1	0	1	0	0	1	6.38	6.00	5.48	5.91	5.44	0	0	0	0	0	0	0	5.16	5.08	5.11	4.95	4.97	5.14	5.01
1381749_at	---	1	0	0	0	0	1	8.33	7.98	7.89	8.37	7.55	0	0	0	0	0	0	0	8.50	8.45	8.61	8.82	8.88	8.61	7.70
1381764_s_at	Rnf126	1	0	1	0	1	1	6.49	6.53	5.89	6.69	6.02	0	0	0	0	0	0	0	8.02	8.01	8.09	7.91	7.85	7.74	8.21
1381774_at	---	1	0	0	0	1	0	6.19	6.07	5.72	6.41	5.74	0	0	0	0	0	0	0	6.13	6.12	6.28	6.18	6.24	6.43	6.28
1381776_at	Tmed6	1	0	1	0	1	1	11.69	11.38	11.08	11.74	10.92	0	0	0	0	0	0	0	3.90	3.89	4.00	3.82	3.85	3.81	3.76
1381787_at	---	1	0	1	0	0	0	4.76	4.68	4.08	4.47	4.43	0	0	0	0	0	0	0	4.40	4.24	4.43	4.30	4.34	4.18	3.71
1381817_at	---	1	0	1	0	1	0	7.22	6.95	7.83	6.96	7.30	0	0	0	0	0	0	0	9.20	9.13	9.29	9.30	9.27	9.39	8.61
1381829_at	Znf318	1	0	0	1	1	0	4.12	4.42	3.92	4.87	4.42	0	0	0	0	0	0	0	4.28	4.27	4.36	4.16	3.93	3.83	4.13
1381845_at	---	1	0	1	0	0	0	5.68	5.49	5.13	5.39	5.48	0	0	0	0	0	0	0	5.06	4.99	4.95	5.02	4.96	4.93	4.73
1381850_at	Ppp1r12a	1	0	0	1	0	0	6.29	6.28	6.57	6.76	6.46	0	0	0	0	0	0	0	7.22	7.06	7.34	7.36	7.21	6.94	7.27
1381878_at	Ubn1	1	0	0	1	0	0	6.71	6.78	7.16	7.24	7.00	0	0	0	0	0	0	0	9.13	8.94	9.02	8.95	8.93	8.63	8.43
1381903_at	Fbxo33	1	0	1	0	1	0	7.65	7.74	8.30	7.54	7.67	0	0	0	0	0	0	0	9.85	9.82	9.80	9.81	9.76	9.88	9.70
1381915_at	RGD1305938	1	0	1	0	0	0	7.53	7.57	8.11	7.49	7.76	0	0	0	0	0	0	0	8.27	8.24	8.29	8.38	8.34	8.68	8.56
1381916_at	---	1	0	1	1	0	1	6.30	6.13	5.71	5.80	5.81	0	0	0	0	0	0	0	7.00	7.00	7.20	7.07	7.11	6.91	6.32
1381957_at	---	1	0	1	0	0	0	5.08	5.02	4.56	4.84	4.88	0	0	0	0	0	0	0	4.83	4.62	4.68	4.52	4.65	4.70	4.26
1381972_at	Crbn	1	0	0	0	1	0	8.00	7.91	8.40	7.62	7.92	0	0	0	0	0	0	0	8.94	8.97	8.98	9.07	9.02	8.99	8.63
1381976_at	Kif21a	1	0	1	1	1	0	8.13	8.15	8.95	7.64	8.44	0	0	0	0	0	0	0	10.07	10.07	10.11	10.21	10.18	10.07	9.84
1381987_at	---	1	0	0	0	0	1	7.00	6.90	7.09	7.10	7.67	0	0	0	0	0	0	0	8.29	8.38	8.40	8.29	8.35	8.35	7.59
1381990_at	Galnt1	1	0	0	0	0	1	6.22	5.94	6.36	5.93	5.23	0	0	0	0	0	0	0	6.98	6.95	7.07	7.20	7.14	7.09	6.73
1381993_at	Clic2	1	0	0	1	1	0	6.61	6.58	6.79	6.00	6.71	0	0	0	0	0	0	0	3.69	3.61	3.74	3.60	3.67	3.59	3.14
1382013_at	Fam8a1	1	0	1	1	1	0	6.71	6.66	7.33	6.08	6.68	0	0	0	0	0	0	0	9.61	9.63	9.65	9.67	9.70	9.50	9.02
1382021_at	Pkd2	1	0	0	0	1	0	8.28	8.18	8.74	7.96	8.35	0	0	0	0	0	0	0	7.99	7.99	8.10	8.20	8.27	7.94	7.07
1382024_at	Dnajb6 /// LOC6	1	0	1	0	1	1	7.18	7.15	7.77	6.83	7.96	0	0	0	0	0	0	0	10.72	10.84	10.80	10.96	10.95	11.14	10.57
1382052_at	Srsf10	1	0	1	0	1	0	6.36	6.33	6.94	6.20	6.78	0	0	0	0	0	0	0	9.41	9.42	9.50	9.64	9.67	9.57	9.01
1382059_at	Fbxo30	1	0	1	0	1	1	4.00	4.22	4.81	3.95	4.50	0	0	0	0	0	0	0	7.29	7.34	7.37	7.59	7.60	7.45	6.32
1382060_at	Asph	1	0	1	0	1	0	6.22	6.48	7.02	6.19	6.60	0	0	0	0	0	0	0	7.55	7.45	7.53	7.44	7.34	7.65	7.68
1382068_at	Tulp4	1	0	1	1	0	1	7.63	7.90	8.35	7.96	8.39	0	0	0	0	0	0	0	8.05	7.91	7.92	7.89	7.83	7.89	8.18
1382083_at	Coch	1	0	1	0	1	0	3.48	3.49	4.28	3.47	3.17	0	0	0	0	0	0	0	3.66	3.61	3.79	3.53	3.57	3.57	3.09
1382088_at	Ryr2	1	0	0	0	0	1	5.04	5.12	5.40	4.73	4.19	0	0	0	0	0	0	0	6.23	6.31	6.40	6.36	6.45	5.68	5.72
1382094_at	Ptprj	1	0	1	0	0	1	7.64	7.80	8.33	7.68	8.35	0	0	0	0	0	0	0	8.46	8.63	8.61	8.73	8.79	8.94	8.16
1382100_at	Mindy3	1	0	1	0	0	0	6.01	6.10	6.70	6.22	6.48	0	0	0	0	0	0	0	8.78	8.87	8.89	8.87	8.93	9.08	8.73
1382103_at	Pgm3	1	0	0	0	0	1	5.87	6.08	6.24	6.19	6.35	0	0	0	0	0	0	0	6.07	6.03	6.17	6.13	6.11	5.98	6.31
1382104_at	---	1	0	1	0	1	0	6.01	6.15	6.75	6.01	6.31	0	0	0	0	0	0	0	8.88	8.88	8.91	9.02	8.95	8.95	8.08
1382105_at	Gnb5	1	0	1	0	1	0	6.16	6.58	6.87	6.11	6.55	0	0	0	0	0	0	0	8.46	8.53	8.57	8.70	8.66	8.85	9.09
1382108_at	---	1	0	0	1	1	0	8.78	8.53	8.98	7.90	8.76	0	0	0	0	0	0	0	7.19	7.23	7.36	7.33	7.16	7.56	7.79
1382118_at	Peg3	1	0	0	0	0	1	10.18	10.14	10.50	10.05	10.77	0	0	0	0	0	0	0	9.50	9.33	9.45	9.44	9.37	9.39	10.05
1382128_at	Kdm7a	1	0	0	0	1	0	6.99	6.82	7.54	6.68	6.91	0	0	0	0	0	0	0	8.68	8.87	8.88	8.94	8.92	9.11	9.04
1382131_at	Arhgap5	1	0	1	0	0	1	6.42	6.47	7.05	6.44	6.90	0	0	0	0	0	0	0	7.21	7.41	7.43	7.59	7.57	7.51	7.33
1382132_at	---	1	0	1	0	1	0	6.94	7.02	7.66	6.58	7.34	0	0	0	0	0	0	0	10.22	10.19	10.27	10.26	10.29	10.28	10.31
1382142_at	---	1	0	1	0	1	0	4.74	4.93	5.66	4.32	4.97	0	0	0	0	0	0	0	6.34	6.19	6.44	6.42	6.30	6.40	6.39
1382144_at	Mrpl47	1	0	0	0	0	1	6.29	6.42	6.67	6.37	6.84	0	0	0	0	0	0	0	9.03	8.91	9.00	9.02	8.96	8.76	7.91
1382146_at	Tspan6	1	0	1	0	1	0	6.80	6.82	7.62	6.64	7.15	0	0	0	0	0	0	0	8.64	8.67	8.75	8.83	8.88	9.09	8.79
1382155_at	Cox15	1	0	0	1	1	0	5.84	5.82	6.38	5.23	5.70	0	0	0	0	0	0	0	8.57	8.61	8.72	8.81	8.72	8.70	7.35
1382161_at	Mphosph10	1	0	1	0	0	0	4.82	4.99	5.60	4.98	5.25	0	0	0	0	0	0	0	7.90	7.98	8.08	8.27	8.23	8.03	7.73
1382171_at	Tsc22d2	1	0	0	0	0	1	4.78	4.91	5.27	5.14	5.62	0	0	0	0	0	0	0	8.63	8.50	8.59	8.64	8.51	8.30	7.86
1382173_at	LOC684828	1	0	0	1	1	0	6.58	6.28	6.08	7.93	6.55	0	0	0	0	0	0	0	3.55	3.61	3.71	3.56	3.60	3.60	3.25
1382179_at	Rdh14	1	0	1	0	0	0	6.90	6.99	7.48	6.89	7.16	0	0	0	0	0	0	0	8.14	8.21	8.25	8.32	8.24	8.15	7.49
1382184_at	Fcmr	1	0	0	0	0	1	4.11	4.17	3.88	4.22	4.93	0	0	0	0	0	0	0	6.22	6.29	6.32	6.31	6.11	6.13	6.31
1382189_at	Sdc2	1	0	1	0	1	0	8.43	8.53	9.21	8.08	8.72	0	0	0	0	0	0	0	8.41	8.43	8.52	8.57	8.52	8.78	8.37
1382193_at	Cisd2	1	0	1	0	0	1	7.90	8.25	8.56	8.19	8.44	0	0	0	0	0	0	0	9.97	9.97	9.95	10.03	9.92	9.89	9.86
1382196_at	Mfsd11	1	0	0	1	1	0	6.72	6.58	7.09	6.12	7.06	0	0	0	0	0	0	0	9.16	9.27	9.36	9.37	9.39	9.57	8.72
1382198_at	Pum2	1	0	1	1	1	0	7.77	7.71	8.50	7.13	7.86	0	0	0	0	0	0	0	10.11	10.13	10.08	10.22	10.22	10.08	9.68
1382203_at	Gdf1	1	0	0	0	0	1	8.27	8.32	8.23	8.26	7.55	0	0	0	0	0	0	0	10.53	10.41	10.57	10.59	10.48	10.55	10.01
1382206_a_at	Akap2	1	0	0	1	0	0	4.88	4.64	4.91	4.37	4.55	0	0	0	0	0	0	0	4.27	4.27	4.37	4.11	4.10	4.26	4.03
1382222_at	Tmem163	1	0	0	0	0	1	6.08	6.35	6.61	6.15	7.00	0	0	0	0	0	0	0	10.97	11.04	10.96	10.76	10.89	10.62	10.36
1382223_at	Zmym4	1	0	1	0	0	0	6.64	6.66	7.24	6.56	7.01	0	0	0	0	0	0	0	8.92	8.91	8.97	9.00	8.98	8.82	8.81
1382231_at	LOC102546992	1	1	1	0	1	1	8.59	7.97	7.47	8.36	7.35	0	0	0	0	0	0	0	4.12	4.03	3.90	3.83	3.95	3.96	3.84
1382232_at	Gpt2	1	0	1	0	0	0	7.11	7.07	6.56	7.08	6.93	0	0	0	0	0	0	0	7.82	7.88	7.91	7.87	7.85	7.96	7.67
1382236_at	---	1	0	1	0	0	1	3.85	3.83	4.45	3.92	4.35	0	0	0	0	0	0	0	8.13	8.14	8.18	8.27	8.25	8.14	7.36
1382245_at	---	1	0	1	0	1	0	7.06	7.32	7.92	6.68	7.39	0	0	0	0	0	0	0	8.79	8.78	8.85	8.99	8.97	9.03	8.45
1382247_a_at	Iqcc	1	0	0	0	0	1	5.27	5.50	5.50	5.17	4.66	0	0	0	0	0	0	0	5.33	5.40	5.29	5.22	5.15	4.93	4.75
1382273_at	Bcl2l14	1	1	0	0	0	1	5.23	4.76	4.77	5.07	4.72	0	0	0	0	0	0	0	5.67	5.58	5.80	5.79	5.74	5.83	5.84
1382274_at	Rarres1	1	0	0	0	1	1	7.19	7.08	6.73	7.43	5.81	0	0	0	0	0	0	0	5.82	5.80	5.71	5.61	5.58	5.63	5.42
1382276_at	Ssh1	1	0	0	0	1	0	5.80	5.88	5.65	6.23	5.80	0	0	0	0	0	0	0	6.84	6.82	6.92	6.77	6.64	6.69	7.28
1382278_at	---	1	1	1	1	0	0	9.05	9.80	8.07	8.18	9.19	0	0	0	0	0	0	0	10.27	10.09	10.71	10.25	10.40	10.47	9.05
1382280_at	Uqcrc2	1	0	0	1	1	0	7.85	7.71	8.24	7.05	7.58	0	0	0	0	0	0	0	9.40	9.43	9.38	9.37	9.37	9.27	8.48
1382282_at	Lix1l	1	0	0	0	1	0	5.63	5.71	6.03	5.33	5.83	0	0	0	0	0	0	0	8.60	8.56	8.46	8.38	8.37	8.54	8.45
1382287_at	Rnps1	1	0	1	0	1	0	6.53	6.64	7.25	6.37	6.68	0	0	0	0	0	0	0	9.54	9.57	9.64	9.72	9.68	9.62	8.92
1382290_at	Eef1akmt2	1	0	1	0	0	0	4.18	4.21	4.86	4.29	4.59	0	0	0	0	0	0	0	8.12	8.10	8.17	8.17	8.09	7.92	7.57
1382309_at	Tulp4	1	0	1	1	1	1	7.22	7.20	7.98	6.68	7.75	0	0	0	0	0	0	0	8.92	8.86	8.94	9.00	8.91	8.89	9.02
1382314_at	Isg15	1	1	0	0	0	0	5.03	5.64	4.55	4.69	4.79	0	0	0	0	0	0	0	5.71	5.71	5.87	6.10	5.91	5.75	5.10
1382317_at	Eps8	1	0	1	0	0	0	5.46	5.55	6.38	5.77	5.88	0	0	0	0	0	0	0	6.69	6.59	6.79	6.86	6.82	6.74	6.90
1382320_at	Clba1	1	0	1	0	0	0	5.35	5.20	4.81	4.89	5.17	0	0	0	0	0	0	0	6.74	6.76	6.73	6.68	6.72	6.95	6.74

Probeset ID	Gene Symbol	Islet Data Set (log2 ratio ≥0.263; FDR <20%) union n=5325 1=YES 0=NO	Islet DRlyp/lyp ND vs ND+Lp299v (log2 ratio ≥0.263; FDR<20%) n=429 1=YES 0=NO	Islet DRlyp/lyp ND vs HCD (log2 ratio ≥0.263; FDR<20%) n=2247 1=YES 0=NO	Islet DRlyp/lyp ND vs HCD+Lp299v (log2 ratio ≥0.263; FDR<20%) n=1595 1=YES 0=NO	Islet DRlyp/lyp HCD vs HCD+Lp299v (log2 ratio ≥0.263; FDR<20%) n=3026 1=YES 0=NO	Islet DRlyp/lyp ND vs Flyp/lyp ND (log2 ratio ≥ 0.263; FDR<20%) n=1950 1=YES 0=NO	Islet Mean Log2 Intensity DRlyp/lyp ND	Islet Mean Log2 Intensity DRlyp/lyp ND+Lp299v	Islet Mean Log2 Intensity DRlyp/lyp HCD	Islet Mean Log2 Intensity DRlyp/lyp HCD+Lp299v	Islet Mean Log2 Intensity Flyp40 ND	Rinm5f data union of all conditions n=3705 1=YES 0=NO	Rinm5f 30 µM propionate/ 6 µM butyrate vs Media (FDR<20%; 1.5 fold) n=3 1=YES 0=NO	Rinm5f 60 µM propionate/ 12 µM butyrate vs Media (FDR<20%; 1.5 fold) n=18 1=YES 0=NO	Rinm5f 120 µM propionate/ 24 µM butyrate vs Media (FDR<20%; 1.5 fold) n=164 1=YES 0=NO	Rinm5f 240 µM propionate /48 µM butyrate vs Media (FDR<20%; 1.5 fold) n=268 1=YES 0=NO	Rinm5f 1500 µM propionate/ 300 µM butyrate vs Media (FDR<20%; 1.5 fold) n=2491 1=YES 0=NO	Rinm5f 500 µM propionate/ 500 µM butyrate vs Media (FDR<20%; 1.5 fold) n=3001 1=YES 0=NO	Rinm5f Mean Log2 Intensity Media only	Rinm5f Mean Log2 Intensity 30 µM propionate/ 6 µM butyrate	Rinm5f Mean Log2 Intensity 60 µM propionate/ 12 µM butyrate	Rinm5f Mean Log2 Intensity 120 µM propionate/ 24 µM butyrate	Rinm5f Mean Log2 Intensity 240 µM propionate/ 48 µM butyrate	Rinm5f Mean Log2 Intensity 1500 µM propionate /300 µM butyrate	Rinm5f Mean Log2 Intensity 500 µM propionate/ 500 µM butyrate
1382331_at	Rfk	1	0	0	0	1	0	7.31	7.29	7.60	6.85	7.50	0	0	0	0	0	0	0	10.29	10.23	10.29	10.34	10.32	10.48	10.12
1382332_at	Stag2	1	0	0	1	1	0	7.99	8.11	8.52	7.52	7.99	0	0	0	0	0	0	0	8.90	8.91	9.00	9.01	8.90	8.70	8.16
1382349_at	Tbccd1	1	0	0	0	0	1	5.63	5.69	6.04	5.53	6.11	0	0	0	0	0	0	0	7.33	7.42	7.44	7.47	7.54	7.66	7.49
1382351_at	Gem	1	0	1	0	1	1	4.30	4.36	5.32	4.30	6.17	0	0	0	0	0	0	0	9.68	9.82	9.65	9.43	9.71	9.59	8.10
1382358_at	Sox5	1	0	0	1	1	0	4.08	3.82	4.11	5.23	3.91	0	0	0	0	0	0	0	3.48	3.58	3.57	3.46	3.53	3.55	3.20
1382368_at	Ro60	1	0	0	0	0	1	3.97	4.26	4.29	3.90	4.92	0	0	0	0	0	0	0	5.99	6.03	6.28	6.10	6.23	6.39	6.22
1382379_at	Rnf138	1	0	0	0	1	0	6.84	6.65	7.38	6.66	6.74	0	0	0	0	0	0	0	8.54	8.63	8.63	8.69	8.77	8.44	7.46
1382384_at	Rassf6	1	0	1	0	1	0	7.83	7.93	8.78	7.74	8.18	0	0	0	0	0	0	0	8.46	8.48	8.48	8.51	8.41	8.44	8.56
1382385_at	Psmc6	1	0	1	0	0	0	5.10	5.04	5.75	5.26	5.48	0	0	0	0	0	0	0	7.42	7.55	7.51	7.72	7.75	7.51	7.34
1382417_at	Mdm4	1	0	0	0	1	0	4.86	4.92	5.31	4.42	5.07	0	0	0	0	0	0	0	8.23	8.23	8.29	8.28	8.26	7.89	7.53
1382419_at	Cenpk	1	0	0	1	1	0	4.09	4.32	4.12	4.74	4.53	0	0	0	0	0	0	0	8.72	8.73	8.78	8.88	8.78	8.30	8.22
1382420_at	Tmem87b	1	0	0	0	1	0	7.25	7.07	7.71	6.86	7.45	0	0	0	0	0	0	0	9.29	9.36	9.32	9.34	9.36	9.03	8.62
1382423_at	---	1	0	1	0	1	1	5.06	4.89	5.81	4.87	5.70	0	0	0	0	0	0	0	7.50	7.49	7.60	7.68	7.58	7.38	7.31
1382431_at	Abca1	1	0	1	1	1	0	6.99	6.88	7.73	6.48	7.05	0	0	0	0	0	0	0	8.03	8.01	8.16	8.09	7.97	8.28	8.16
1382439_at	Itgb6	1	0	0	1	0	0	5.55	5.53	5.72	6.02	5.64	0	0	0	0	0	0	0	3.20	3.23	3.25	3.16	3.13	3.20	2.82
1382452_at	Cavin2	1	1	0	1	0	0	6.78	6.28	6.47	6.08	6.37	0	0	0	0	0	0	0	3.22	3.14	3.25	3.19	3.15	3.22	2.79
1382454_at	Cxcl9	1	1	1	0	0	1	4.69	5.87	3.92	4.23	4.14	0	0	0	0	0	0	0	3.65	3.73	3.79	3.66	3.72	3.85	3.64
1382463_at	---	1	0	1	0	1	0	6.02	5.84	6.77	5.93	6.01	0	0	0	0	0	0	0	6.85	6.82	6.89	7.15	7.05	6.80	5.84
1382467_at	Bex1 /// Bex2	1	0	1	0	1	1	8.98	9.17	9.99	9.14	9.71	0	0	0	0	0	0	0	11.63	11.64	11.64	11.76	11.77	11.57	11.05
1382482_at	---	1	1	0	1	0	0	4.68	4.18	4.40	4.19	4.28	0	0	0	0	0	0	0	4.33	4.28	4.40	4.56	4.58	4.77	4.89
1382494_at	---	1	0	1	0	0	0	6.92	6.90	6.33	6.81	6.70	0	0	0	0	0	0	0	5.37	5.37	5.27	5.15	5.23	5.13	4.58
1382495_x_at	---	1	0	1	0	1	0	6.21	5.97	5.51	6.19	5.90	0	0	0	0	0	0	0	5.05	5.15	5.23	4.97	4.97	5.10	4.97
1382497_at	---	1	1	1	1	1	0	7.12	7.78	7.69	6.50	7.11	0	0	0	0	0	0	0	7.01	7.00	7.30	7.11	7.16	7.54	6.61
1382509_at	CNOT6	1	0	1	0	1	0	7.03	6.95	7.63	6.71	7.09	0	0	0	0	0	0	0	9.55	9.50	9.58	9.72	9.56	9.53	9.35
1382510_at	---	1	0	1	0	1	1	5.65	5.91	6.25	5.42	6.62	0	0	0	0	0	0	0	6.54	6.34	6.59	6.51	6.47	6.51	7.46
1382516_at	---	1	0	1	0	1	0	4.12	4.01	4.89	4.16	4.16	0	0	0	0	0	0	0	6.68	6.69	6.92	7.00	6.92	6.98	6.79
1382518_at	Pde8a	1	0	0	1	0	1	4.39	4.60	4.59	4.75	4.85	0	0	0	0	0	0	0	5.44	5.37	5.50	5.51	5.44	5.17	4.98
1382524_at	Zbtb20	1	0	1	0	0	1	4.77	4.57	5.51	4.84	5.06	0	0	0	0	0	0	0	4.38	4.49	4.75	4.73	4.72	4.72	4.19
1382533_at	Agfg1	1	0	1	1	1	0	5.53	5.35	6.14	5.00	5.53	0	0	0	0	0	0	0	8.26	8.21	8.34	8.27	8.23	8.37	8.15
1382535_at	Kctd12	1	0	1	0	1	0	4.21	4.48	4.85	3.86	4.58	0	0	0	0	0	0	0	4.79	4.72	4.77	4.65	4.59	4.76	4.72
1382538_at	LOC100912031	1	0	1	1	1	0	5.26	5.32	5.98	4.68	5.22	0	0	0	0	0	0	0	9.26	9.21	9.30	9.37	9.31	9.38	8.56
1382540_at	Prpf40a	1	0	1	0	1	0	5.43	5.46	6.02	5.31	5.64	0	0	0	0	0	0	0	7.36	7.55	7.48	7.69	7.70	7.66	7.07
1382561_at	---	1	0	0	1	1	0	4.96	4.97	5.26	4.40	4.95	0	0	0	0	0	0	0	7.33	7.21	7.36	7.33	7.13	6.91	5.39
1382565_at	Mapk1ip1l	1	0	1	0	0	0	4.16	4.12	4.86	4.18	4.46	0	0	0	0	0	0	0	6.92	7.07	7.03	6.96	7.06	6.90	6.77
1382578_at	Zbtb41	1	0	1	0	1	0	5.04	4.80	5.83	4.79	5.11	0	0	0	0	0	0	0	7.90	7.89	7.98	8.10	8.04	7.75	7.57
1382579_at	Tox3	1	0	0	0	1	0	8.67	8.60	9.21	8.33	8.90	0	0	0	0	0	0	0	10.75	10.72	10.79	10.79	10.86	11.04	10.96
1382593_at	Etv1	1	0	0	1	1	0	5.87	5.81	6.16	5.31	5.70	0	0	0	0	0	0	0	11.59	11.63	11.56	11.50	11.66	11.56	10.87
1382594_at	Man2a1	1	0	0	1	0	1	4.98	4.75	4.74	4.47	4.39	0	0	0	0	0	0	0	5.26	5.14	5.18	5.31	5.38	5.34	4.69
1382599_at	Igf1	1	1	0	0	0	0	4.14	3.63	4.05	4.24	3.89	0	0	0	0	0	0	0	3.20	3.22	3.20	3.25	3.12	3.25	2.73
1382602_at	Ubr3	1	0	1	0	1	0	7.72	8.04	8.65	7.43	8.19	0	0	0	0	0	0	0	6.92	6.87	7.08	7.15	6.99	6.78	6.73
1382610_at	---	1	0	1	1	1	0	5.65	5.75	6.52	4.95	5.93	0	0	0	0	0	0	0	8.74	8.52	8.74	8.84	8.82	8.64	7.89
1382614_at	Zfyve16	1	0	0	0	1	0	4.86	4.81	5.42	4.66	5.07	0	0	0	0	0	0	0	6.63	6.62	6.67	6.56	6.57	6.41	5.78
1382615_at	Sec61a1	1	0	0	1	0	0	9.46	9.23	9.42	8.97	9.28	0	0	0	0	0	0	0	10.80	10.82	10.89	10.96	10.90	10.82	9.77
1382620_at	Ankrd11	1	0	0	1	1	0	6.75	6.99	6.82	7.40	6.96	0	0	0	0	0	0	0	8.14	8.10	8.30	8.12	8.03	8.07	8.47
1382629_at	Far1	1	0	1	1	1	0	5.18	5.46	5.81	4.26	5.53	0	0	0	0	0	0	0	8.21	8.18	8.27	8.38	8.35	8.51	8.14
1382632_at	Robo2	1	0	1	0	1	1	5.11	5.41	6.05	5.04	6.10	0	0	0	0	0	0	0	3.92	3.92	4.02	4.04	3.93	4.07	4.28
1382633_at	Creb1	1	0	1	0	0	1	5.84	5.71	6.37	6.01	6.20	0	0	0	0	0	0	0	6.43	6.51	6.58	6.52	6.56	6.69	6.47
1382670_at	Larp1	1	0	1	0	1	0	8.47	8.21	7.85	8.72	8.29	0	0	0	0	0	0	0	8.07	8.07	8.22	8.32	8.22	8.40	7.34
1382679_at	Wdr43	1	0	0	0	0	1	6.69	6.55	6.55	6.65	6.21	0	0	0	0	0	0	0	8.42	8.51	8.54	8.55	8.48	8.26	7.83
1382682_at	---	1	1	0	0	0	0	4.97	4.50	5.38	4.88	4.56	0	0	0	0	0	0	0	6.45	6.53	6.59	6.50	6.47	6.68	6.62
1382691_at	Sf3b1	1	0	1	0	1	0	6.33	6.09	7.23	6.38	6.59	0	0	0	0	0	0	0	8.61	8.63	8.59	8.61	8.60	8.63	7.62
1382707_at	Rps6ka3	1	0	1	0	0	1	6.05	6.10	6.77	6.21	6.63	0	0	0	0	0	0	0	7.25	7.18	7.27	7.20	7.27	7.29	6.81
1382711_at	Zkscan1	1	0	0	0	1	0	4.33	4.42	4.86	3.89	4.24	0	0	0	0	0	0	0	6.43	6.32	6.48	6.51	6.42	6.65	5.92
1382712_at	Pfkfb2	1	0	1	1	0	0	7.47	7.59	8.38	8.00	7.44	0	0	0	0	0	0	0	6.41	6.11	6.57	6.54	6.50	6.65	6.81
1382734_at	Jag2	1	0	1	0	0	0	5.50	5.09	4.96	5.24	5.16	0	0	0	0	0	0	0	8.77	8.87	8.93	8.85	8.96	8.79	8.58
1382735_at	Atg12	1	0	0	0	0	1	5.35	5.49	5.62	5.13	5.99	0	0	0	0	0	0	0	8.93	8.99	9.03	9.17	9.19	9.42	8.52
1382741_at	Ube3a	1	0	1	0	1	0	7.57	7.58	8.22	7.38	7.79	0	0	0	0	0	0	0	10.17	10.29	10.27	10.37	10.37	10.11	9.14
1382742_at	---	1	0	0	0	1	0	5.65	5.55	5.24	5.86	5.48	0	0	0	0	0	0	0	4.17	4.29	4.39	4.01	4.07	4.18	3.68
1382763_at	C12orf73	1	0	0	0	1	0	7.39	7.39	7.06	7.66	7.09	0	0	0	0	0	0	0	8.06	8.01	8.03	8.01	7.94	7.67	7.31
1382778_at	Dusp6	1	0	1	1	0	0	6.48	6.08	7.59	7.24	6.17	0	0	0	0	0	0	0	8.35	8.40	8.48	8.48	8.78	8.72	7.81
1382779_at	Hace1	1	0	1	0	1	1	4.87	4.88	6.00	4.66	5.49	0	0	0	0	0	0	0	9.53	9.59	9.69	9.69	9.70	9.60	9.38
1382807_at	Atg9a	1	0	0	1	1	0	7.31	7.52	6.88	7.88	7.17	0	0	0	0	0	0	0	7.86	7.88	7.82	7.71	7.76	7.49	7.38
1382809_at	Cirbp	1	1	1	1	1	1	7.69	7.22	8.31	6.86	7.21	0	0	0	0	0	0	0	9.47	9.56	9.61	9.60	9.61	9.48	8.52
1382813_at	Pomk	1	0	0	1	0	1	5.83	5.82	6.06	6.38	6.56	0	0	0	0	0	0	0	8.10	8.04	8.21	8.24	8.20	8.25	8.03
1382814_at	Tenm3	1	0	1	0	1	1	5.68	5.95	6.60	5.67	6.62	0	0	0	0	0	0	0	7.02	6.93	7.02	6.92	6.93	7.02	7.21
1382835_at	---	1	0	1	0	1	1	6.25	6.30	7.12	6.16	6.75	0	0	0	0	0	0	0	8.63	8.54	8.72	8.69	8.70	8.88	9.04
1382839_at	---	1	0	0	0	1	0	4.00	4.03	4.47	3.73	4.17	0	0	0	0	0	0	0	6.51	6.39	6.64	6.59	6.55	6.45	6.15
1382843_at	Sgpl1	1	0	0	0	1	0	4.91	4.96	5.35	4.60	4.85	0	0	0	0	0	0	0	7.57	7.60	7.72	7.81	7.68	7.96	6.96
1382855_at	Tcfl5	1	0	0	0	0	1	5.30	5.61	5.75	5.33	5.91	0	0	0	0	0	0	0	4.56	4.46	4.46	4.27	4.48	4.55	3.66
1382856_at	Atp23	1	0	1	0	1	0	6.45	6.66	7.18	6.36	6.77	0	0	0	0	0	0	0	9.32	9.24	9.34	9.35	9.37	9.57	9.24
1382861_at	Tcea1	1	0	0	1	1	0	7.65	7.61	8.12	7.06	7.62	0	0	0	0	0	0	0	10.11	10.10	10.19	10.27	10.18	10.06	9.41
1382864_at	Palmd	1	0	1	0	0	0	5.95	5.89	5.39	5.79	5.70	0	0	0	0	0	0	0	3.94	3.87	3.91	3.70	3.68	3.82	3.57
1382866_at	Gatc	1	0	0	1	1	0	6.55	6.77	6.39	7.07	6.52	0	0	0	0	0	0	0	7.89	7.91	7.97	7.98	7.95	8.00	8.11
1382872_at	---	1	0	0	0	0	1	4.03	4.05	4.31	4.11	4.52	0	0	0	0	0	0	0	7.01	6.94	7.10	7.12	6.93	6.72	6.53
1382873_at	Cttnbp2nl	1	0	0	1	0	1	4.75	4.44	4.47	4.21	4.16	0	0	0	0	0	0	0	5.44	5.46	5.58	5.55	5.47	5.74	5.27
1382903_at	Zzz3	1	0	1	0	0	0	6.70	6.80	7.47	6.83	6.89	0	0	0	0	0	0	0	9.03	8.94	9.00	9.12	8.97	8.67	8.35
1382907_at	Tent5c	1	0	0	0	0	1	10.45	10.26	10.23	10.45	9.92	0	0	0	0	0	0	0	4.34	4.40	4.35	4.28	4.46	4.72	5.49
1382913_at	Cttnbp2	1	0	0	1	0	0	2.90	3.05	3.20	3.59	3.12	0	0	0	0	0	0	0	3.43	3.29	3.63	3.44	3.50	3.59	3.30
1382937_at	Spdef	1	0	1	1	1	1	6.36	6.25	5.36	6.86	5.66	0	0	0	0	0	0	0	4.53	4.66	4.50	4.39	4.39	4.46	4.15
1382940_at	Glb1l2	1	0	0	1	1	0	6.11	6.18	6.65	5.54	6.38	0	0	0	0	0	0	0	7.99	7.83	7.93	7.89	7.77	7.60	7.07
1382950_at	Gbp7	1	1	0	0	0	0	3.80	4.68	3.59	3.40	4.01	0	0	0	0	0	0	0	4.37	4.22	4.40	4.39	4.24	4.10	3.77
1382964_at	Glb1l2	1	0	1	0	1	0	7.83	8.11	8.58	7.71	8.27	0	0	0	0	0	0	0	9.51	9.42	9.40	9.29	9.26	9.15	8.79
1382978_at	---	1	0	1	0	0	0	6.80	6.65	7.39	6.87	7.08	0	0	0	0	0	0	0	9.54	9.61	9.80	9.80	9.90	9.75	9.07
1382981_at	Ahi1	1	1	0	1	0	1	5.85	6.42	6.16	6.37	6.33	0	0	0	0	0	0	0	6.31	6.05	6.34	6.16	6.25	6.38	6.36
1382982_at	---	1	0	1	0	0	0	4.31	4.26	4.84	4.20	4.54	0	0	0	0	0	0	0	6.32	6.33	6.58	6.54	6.56	6.35	6.54
1382994_at	LOC102548472	1	0	0	0	1	0	4.46	4.46	4.27	4.90	4.46	0	0	0	0	0	0	0	4.08	3.93	3.99	3.96	3.90	3.99	3.59
1383002_at	Phf20l1	1	0	1	0	1	1	5.10	5.26	5.86	5.16	5.65	0	0	0	0	0	0	0	6.59	6.66	6.81	7.08	7.01	6.61	5.56
1383004_at	Ahcyl1	1	0	0	1	1	0	7.87	7.80	8.41	6.97	7.76	0	0	0	0	0	0	0	10.37	10.24	10.25	10.30	10.21	10.26	9.92
1383018_at	Angptl6	1	0	0	1	1	0	7.70	7.68	7.36	8.20	7.37	0	0	0	0	0	0	0	6.15	6.16	6.27	6.20	6.22	6.25	5.81
1383023_at	Ube2h	1	0	0	1	1	0	4.05	3.82	4.39	3.48	3.88	0	0	0	0	0	0	0	7.23	7.09	7.26	7.29	7.13	7.38	6.38
1383028_at	Cdv3	1	0	0	1	0	0	6.65	6.44	6.26	6.13	6.25	0	0	0	0	0	0	0	7.85	7.77	7.93	7.84	7.72	7.61	6.64
1383037_at	Poldip2	1	0	0	0	0	1	8.31	8.03	7.81	8.01	7.85	0	0	0	0	0	0	0	8.83	8.83	8.91	9.01	8.89	8.73	7.82
1383044_at	Fryl	1	0	0	0	0	1	4.25	4.37	4.58	4.45	4.75	0	0	0	0	0	0	0	5.41	5.55	5.57	5.42	5.39	5.68	5.86
1383047_at	Gas6	1	0	0	0	0	1	9.17	8.96	9.07	9.04	8.54	0	0	0	0	0	0	0	11.57	11.65	11.56	11.21	11.45	11.17	11.03
1383055_at	---	1	0	1	0	0	1	6.81	7.11	7.48	6.96	7.29	0	0	0	0	0	0	0	9.55	9.60	9.60	9.67	9.69	9.24	9.14
1383061_at	---	1	0	0	1	0	0	8.15	8.27	8.58	8.61	8.50	0	0	0	0	0	0	0	8.14	8.05	8.21	8.21	8.08	8.30	8.54
1383062_at	---	1	0	1	0	1	0	7.91	7.82	8.69	7.71	8.32	0	0	0	0	0	0	0	10.26	10.28	10.37	10.44	10.46	10.66	10.62
1383063_a_at	---	1	0	1	0	0	0	8.97	9.03	9.59	9.24	9.42	0	0	0	0	0	0	0	9.26	9.10	9.28	9.22	9.30	9.59	9.97
1383075_at	Ccnd1	1	0	0	0	0	1	8.43	8.38	8.98	8.33	9.10	0	0	0	0	0	0	0	7.03	7.19	7.26	7.46	7.46	6.99	6.36
1383079_at	Vma21	1	0	1	0	1	0	4.32	4.46	4.98	4.18	4.55	0	0	0	0	0	0	0	7.72	7.82	7.80	7.95	7.84	7.70	6.38
1383084_at	Ttll7	1	0	1	0	1	1	7.35	7.72	8.07	7.02	8.05	0	0	0	0	0	0	0	8.65	8.75	8.77	8.84	8.93	8.94	8.37
1383085_at	Sh3bgrl	1	0	0	0	1	0	8.08	7.94	8.59	7.89	8.22	0	0	0	0	0	0	0	7.30	7.27	7.28	7.24	7.15	7.23	7.38
1383095_at	Tasor	1	0	1	0	1	0	6.12	6.09	6.92	6.13	6.40	0	0	0	0	0	0	0	8.60	8.76	8.77	8.94	8.96	8.98	8.68
1383096_at	Aplp2	1	0	0	1	1	0	5.69	5.32	5.81	4.61	5.37	0	0	0	0	0	0	0	9.66	9.62	9.80	9.84	9.83	10.11	8.72
1383098_at	Fyttd1	1	0	1	1	1	0	7.01	7.06	7.73	6.42	7.05	0	0	0	0	0	0	0	9.52	9.57	9.63	9.77	9.78	9.84	9.07
1383114_at	Mark2	1	0	0	0	1	0	6.63	6.80	6.29	7.10	6.50	0	0	0	0	0	0	0	7.06	7.08	7.09	6.97	6.96	6.93	7.01
1383115_at	Cstf2t	1	0	1	0	0	0	8.31	8.37	8.94	8.47	8.71	0	0	0	0	0	0	0	11.05	11.08	11.08	11.16	11.07	11.04	10.84
1383117_at	Pxmp4	1	0	0	0	0	1	7.49	7.42	7.18	7.62	4.59	0	0	0	0	0	0	0	9.09	9.03	9.13	9.08	9.07	9.28	9.24
1383118_at	Tmem209	1	1	1	1	0	1	8.91	8.41	7.55	7.87	8.33	0	0	0	0	0	0	0	8.84	8.84	9.02	9.16	9.09	9.16	8.90
1383126_at	Akt1	1	0	0	0	1	0	9.83	9.75	9.69	10.26	9.94	0	0	0	0	0	0	0	10.66	10.65	10.68	10.62	10.58	10.46	10.25
1383133_at	---	1	0	0	0	0	1	7.18	7.38	7.49	7.19	7.72	0	0	0	0	0	0	0	8.16	8.10	8.18	8.22	8.26	8.18	8.20
1383137_at	Sox4	1	0	1	1	0	0	9.01	9.12	9.78	9.57	9.30	0	0	0	0	0	0	0	11.96	12.00	12.08	12.07	12.13	12.48	12.32
1383139_at	rnf141	1	0	1	0	0	0	7.34	7.32	7.99	7.52	7.57	0	0	0	0	0	0	0	9.19	9.19	9.23	9.19	9.20	9.34	9.24
1383140_at	Spock2	1	0	0	0	0	1	4.74	4.96	5.10	4.84	5.25	0	0	0	0	0	0	0	5.46	5.48	5.52	5.39	5.31	5.30	4.85
1383147_at	---	1	0	1	0	1	0	7.33	7.39	8.10	6.95	7.80	0	0	0	0	0	0	0	9.23	9.25	9.37	9.43	9.50	9.56	9.41
1383152_at	Chid1 /// LOC10	1	0	1	1	1	0	8.25	8.34	7.71	8.84	7.99	0	0	0	0	0	0	0	8.87	8.92	8.91	8.93	8.98	8.92	8.51
1383160_at	Chordc1	1	0	1	0	0	0	7.25	7.31	7.94	7.34	7.65	0	0	0	0	0	0	0	9.91	9.92	9.96	10.15	10.05	10.09	9.85
1383161_a_at	---	1	0	0	0	0	1	9.11	9.47	9.49	9.01	10.15	0	0	0	0	0	0	0	11.97	12.01	11.90	11.84	11.93	11.95	11.36
1383162_at	---	1	0	0	0	0	1	9.42	9.89	9.80	9.26	10.41	0	0	0	0	0	0	0	11.59	11.57	11.59	11.47	11.65	11.56	11.05
1383165_at	RGD1310209	1	0	0	1	1	0	9.92	9.84	10.06	10.62	9.99	0	0	0	0	0	0	0	10.58	10.60	10.63	10.57	10.59	10.54	10.39
1383179_at	CTDSPL2	1	0	1	1	0	1	5.40	5.30	5.97	5.90	6.13	0	0	0	0	0	0	0	9.29	9.32	9.31	9.25	9.28	9.28	8.87
1383180_at	Ppp1r2	1	0	0	1	1	0	8.56	8.51	8.88	7.84	8.64	0	0	0	0	0	0	0	10.93	10.91	10.93	11.00	10.97	10.99	10.65
1383183_at	---	1	0	1	0	0	1	4.34	4.73	5.02	4.41	4.89	0	0	0	0	0	0	0	8.31	8.36	8.52	8.62	8.58	8.39	8.17
1383185_at	---	1	0	1	0	1	0	6.68	6.79	7.82	6.33	7.06	0	0	0	0	0	0	0	4.01	4.06	3.94	3.84	4.08	3.96	3.87
1383192_at	Spast	1	0	1	0	1	0	5.77	5.66	6.44	5.70	5.83	0	0	0	0	0	0	0	7.92	7.91	8.06	8.19	8.11	7.76	7.36
1383197_at	Jam2	1	0	0	0	0	1	6.70	6.62	6.56	6.55	6.09	0	0	0	0	0	0	0	5.15	5.16	5.15	4.94	4.95	5.15	4.96
1383215_at	Ttc28	1	0	1	0	0	0	5.63	5.59	5.10	5.48	5.68	0	0	0	0	0	0	0	5.09	5.15	5.11	5.11	5.18	5.32	5.58
1383223_at	Ubl3	1	0	1	1	1	0	7.51	7.76	8.10	6.69	7.41	0	0	0	0	0	0	0	10.68	10.75	10.77	10.78	10.76	10.89	10.28
1383225_at	Scube3	1	0	1	1	0	1	6.32	6.03	4.66	5.07	5.77	0	0	0	0	0	0	0	4.20	4.11	4.07	4.18	4.11	4.04	3.67
1383226_at	Zfx	1	0	0	0	1	0	5.85	5.61	6.38	5.68	6.00	0	0	0	0	0	0	0	7.35	7.40	7.50	7.51	7.42	7.09	6.95
1383242_a_at	Cebpa	1	0	1	1	1	1	5.64	5.68	4.59	6.63	5.04	0	0	0	0	0	0	0	3.84	3.80	3.99	3.99	3.83	4.18	3.92
1383243_at	Cebpa	1	0	0	1	1	0	5.80	6.02	5.32	6.41	5.42	0	0	0	0	0	0	0	4.79	4.84	4.86	4.71	4.81	4.91	5.14
1383254_at	---	1	0	1	0	0	1	5.63	6.01	6.31	5.64	6.33	0	0	0	0	0	0	0	9.06	9.20	9.28	9.38	9.49	9.47	8.58
1383261_at	Arrb1	1	0	1	0	1	0	6.35	6.25	5.82	6.39	6.01	0	0	0	0	0	0	0	7.88	7.81	7.90	7.66	7.74	7.70	7.57
1383263_at	Ogn	1	0	0	1	1	1	5.37	5.06	5.50	4.57	4.84	0	0	0	0	0	0	0	4.51	4.51	4.49	4.41	4.28	4.39	3.83

Probeset ID	Gene Symbol	Islet Data Set (log2 ratio ≥0.263; FDR <20%) union n=5325 1=YES 0=NO	Islet DRlyp/lyp ND vs ND+Lp299v (log2 ratio ≥0.263; FDR<20%) n=429 1=YES 0=NO	Islet DRlyp/lyp ND vs HCD (log2 ratio ≥0.263; FDR<20%) n=2247 1=YES 0=NO	Islet DRlyp/lyp ND vs HCD+Lp299v (log2 ratio ≥0.263; FDR<20%) n=1595 1=YES 0=NO	Islet DRlyp/lyp HCD vs HCD+Lp299v (log2 ratio ≥0.263; FDR<20%) n=3026 1=YES 0=NO	Islet DRlyp/lyp ND vs Flyp/lyp ND (log2 ratio ≥ 0.263; FDR<20%) n=1950 1=YES 0=NO	Islet Mean Log2 Intensity DRlyp/lyp ND	Islet Mean Log2 Intensity DRlyp/lyp ND+Lp299v	Islet Mean Log2 Intensity DRlyp/lyp HCD	Islet Mean Log2 Intensity DRlyp/lyp HCD+Lp299v	Islet Mean Log2 Intensity Flyp40 ND	Rinm5f data union of all conditions n=3705 1=YES 0=NO	Rinm5f 30 µM propionate/ 6 µM butyrate vs Media (FDR<20%; 1.5 fold) n=3 1=YES 0=NO	Rinm5f 60 µM propionate/ 12 µM butyrate vs Media (FDR<20%; 1.5 fold) n=18 1=YES 0=NO	Rinm5f 120 µM propionate/ 24 µM butyrate vs Media (FDR<20%; 1.5 fold) n=164 1=YES 0=NO	Rinm5f 240 µM propionate /48 µM butyrate vs Media (FDR<20%; 1.5 fold) n=268 1=YES 0=NO	Rinm5f 1500 µM propionate/ 300 µM butyrate vs Media (FDR<20%; 1.5 fold) n=2491 1=YES 0=NO	Rinm5f 500 µM propionate/ 500 µM butyrate vs Media (FDR<20%; 1.5 fold) n=3001 1=YES 0=NO	Rinm5f Mean Log2 Intensity Media only	Rinm5f Mean Log2 Intensity 30 µM propionate/ 6 µM butyrate	Rinm5f Mean Log2 Intensity 60 µM propionate/ 12 µM butyrate	Rinm5f Mean Log2 Intensity 120 µM propionate/ 24 µM butyrate	Rinm5f Mean Log2 Intensity 240 µM propionate/ 48 µM butyrate	Rinm5f Mean Log2 Intensity 1500 µM propionate /300 µM butyrate	Rinm5f Mean Log2 Intensity 500 µM propionate/ 500 µM butyrate
1383265_at	Tbc1d23	1	0	1	0	0	0	7.78	7.97	8.38	7.73	8.17	0	0	0	0	0	0	0	9.70	9.74	9.74	9.81	9.80	9.83	9.53
1383269_at	Rnf2	1	0	1	0	1	0	6.80	6.73	7.42	6.41	6.77	0	0	0	0	0	0	0	9.32	9.37	9.38	9.45	9.46	9.49	9.17
1383289_at	Nif3l1	1	0	0	0	0	1	5.77	5.97	6.25	5.70	6.32	0	0	0	0	0	0	0	7.79	7.80	7.84	7.80	7.89	7.84	7.32
1383291_at	C7	1	1	0	1	1	0	5.47	5.00	5.81	4.08	5.19	0	0	0	0	0	0	0	3.88	4.02	3.98	3.96	3.88	3.92	3.50
1383293_at	---	1	0	0	0	0	1	7.13	7.32	7.64	7.35	7.68	0	0	0	0	0	0	0	7.84	7.80	7.91	7.81	7.89	7.66	7.46
1383295_at	Fam110a	1	0	1	0	0	1	6.52	6.07	5.96	6.20	5.92	0	0	0	0	0	0	0	7.85	7.86	7.90	7.81	7.84	7.52	7.63
1383302_at	Dnajb1	1	1	0	1	1	0	7.46	8.38	7.66	6.86	7.46	0	0	0	0	0	0	0	8.63	8.59	8.58	8.63	8.50	8.48	7.99
1383305_at	---	1	0	1	1	1	0	5.82	5.80	6.53	5.31	6.07	0	0	0	0	0	0	0	7.73	7.85	7.90	8.01	7.93	7.97	7.71
1383309_at	St3gal6	1	0	0	1	1	0	6.43	6.41	6.98	5.90	6.70	0	0	0	0	0	0	0	7.58	7.70	7.81	7.81	7.79	7.82	7.59
1383311_at	Mtmr11	1	0	1	0	1	0	4.98	4.93	5.64	4.87	5.34	0	0	0	0	0	0	0	8.31	8.35	8.37	8.30	8.31	7.96	7.60
1383314_at	Tmem51	1	0	0	0	1	0	6.16	6.20	5.77	6.42	5.88	0	0	0	0	0	0	0	8.62	8.80	8.64	8.37	8.49	8.52	8.01
1383317_at	LOC102554996	1	0	1	0	0	0	5.61	5.37	5.02	5.29	5.31	0	0	0	0	0	0	0	4.89	4.92	4.91	4.68	4.80	5.03	4.22
1383325_at	Smim13	1	0	0	0	1	1	5.90	5.68	5.61	6.30	5.38	0	0	0	0	0	0	0	6.86	6.91	6.82	6.66	6.71	6.59	7.05
1383327_at	Pdcd4	1	0	0	0	0	1	9.04	8.77	9.29	8.88	8.48	0	0	0	0	0	0	0	8.64	8.63	8.76	8.91	8.94	9.11	8.49
1383331_at	---	1	0	0	0	0	1	4.40	4.32	4.21	4.35	7.24	0	0	0	0	0	0	0	7.87	7.89	8.06	8.29	8.30	8.21	8.16
1383336_at	Pnn	1	0	1	0	1	0	7.65	7.41	8.43	7.38	7.85	0	0	0	0	0	0	0	9.76	9.81	9.84	9.79	9.91	9.58	8.47
1383337_at	Gse1	1	0	0	0	0	1	5.96	6.13	6.38	6.39	6.61	0	0	0	0	0	0	0	8.72	8.73	8.80	8.79	8.88	8.98	9.05
1383338_at	Pggt1b	1	0	1	0	1	0	5.00	5.16	5.77	5.24	5.17	0	0	0	0	0	0	0	7.28	7.32	7.39	7.55	7.54	7.17	6.18
1383339_at	Uri1	1	0	0	0	0	1	8.35	8.29	8.62	8.66	7.46	0	0	0	0	0	0	0	8.78	8.80	8.83	8.98	8.88	8.96	9.13
1383341_at	Cab39l	1	0	1	0	0	0	4.89	5.35	5.51	5.19	5.16	0	0	0	0	0	0	0	6.77	6.73	6.68	6.59	6.47	6.44	6.10
1383348_at	Ndst1	1	0	0	1	1	0	5.99	5.99	5.92	6.71	5.80	0	0	0	0	0	0	0	7.64	7.71	7.76	7.87	7.81	7.63	7.22
1383350_at	Slc35c2	1	0	0	0	1	0	7.08	6.95	6.71	7.37	6.79	0	0	0	0	0	0	0	8.40	8.47	8.53	8.45	8.45	8.38	7.90
1383352_at	Ttf1	1	0	0	0	0	1	5.99	5.86	6.02	6.21	5.48	0	0	0	0	0	0	0	6.96	6.94	6.97	7.05	7.01	6.93	6.77
1383354_a_at	Fbxo33	1	0	1	1	1	0	5.62	5.47	6.68	4.94	5.73	0	0	0	0	0	0	0	8.35	8.42	8.38	8.47	8.45	8.60	7.99
1383355_at	Abca1	1	0	1	0	1	0	5.92	6.07	6.54	5.73	5.98	0	0	0	0	0	0	0	7.95	7.86	7.86	7.58	7.62	7.82	8.11
1383371_at	Ssc4d	1	0	0	0	0	1	5.13	5.23	5.10	5.38	4.62	0	0	0	0	0	0	0	6.35	6.36	6.55	6.41	6.34	5.87	6.13
1383377_at	Gabpa	1	0	1	0	1	0	6.83	6.91	7.63	6.90	7.14	0	0	0	0	0	0	0	9.29	9.30	9.22	9.33	9.32	9.20	8.76
1383380_at	Rpp38	1	1	0	0	0	0	6.72	6.22	6.40	6.78	6.31	0	0	0	0	0	0	0	7.79	7.85	7.91	7.93	7.82	7.70	7.22
1383391_a_at	C2 /// Cfb	1	0	1	0	0	0	5.83	5.79	5.26	5.70	5.53	0	0	0	0	0	0	0	4.70	4.74	4.54	4.41	4.47	4.66	4.61
1383399_at	Sv2a	1	0	0	0	0	1	7.42	7.70	7.69	7.50	7.94	0	0	0	0	0	0	0	10.05	10.06	10.19	10.09	10.10	10.21	10.07
1383415_at	Ppp3ca	1	0	0	0	1	0	7.29	7.23	7.04	7.75	7.27	0	0	0	0	0	0	0	7.70	7.70	7.77	7.86	7.90	7.99	8.29
1383416_at	Acat1	1	0	0	1	1	0	7.14	6.91	6.99	7.67	7.21	0	0	0	0	0	0	0	4.22	4.25	4.30	4.39	4.28	4.60	4.35
1383417_at	Bbs1	1	0	0	0	0	1	4.19	4.37	4.74	4.09	4.74	0	0	0	0	0	0	0	7.80	7.84	7.93	7.90	7.94	7.82	7.85
1383418_at	Adam11	1	0	1	1	1	0	7.31	7.25	6.47	7.99	6.97	0	0	0	0	0	0	0	5.40	5.48	5.54	5.37	5.61	5.80	5.79
1383424_at	Cmpk2	1	1	0	1	0	0	6.08	6.71	6.42	6.57	6.12	0	0	0	0	0	0	0	7.21	7.28	7.41	7.59	7.40	7.76	7.55
1383425_at	C5	1	0	0	1	1	1	7.05	6.86	6.95	7.79	6.42	0	0	0	0	0	0	0	3.55	3.59	3.67	3.44	3.49	3.49	3.48
1383433_at	Klhl23 /// Phosp	1	0	0	1	0	0	6.75	7.03	7.00	7.34	6.79	0	0	0	0	0	0	0	7.58	7.54	7.80	7.90	7.79	7.56	7.78
1383434_at	Pycr1	1	0	1	0	1	0	10.42	10.43	9.74	10.86	10.01	0	0	0	0	0	0	0	9.43	9.49	9.45	9.51	9.59	9.69	9.59
1383435_at	Scn3b	1	0	1	0	1	0	8.34	8.63	8.92	8.03	8.73	0	0	0	0	0	0	0	9.80	9.87	9.89	9.84	9.87	9.75	9.37
1383442_at	Morn1	1	0	1	0	0	0	5.44	5.33	4.66	5.19	5.07	0	0	0	0	0	0	0	6.09	6.04	6.22	5.90	6.03	6.05	5.97
1383443_at	Aaed1	1	0	1	1	1	0	5.62	6.00	7.02	5.03	5.75	0	0	0	0	0	0	0	7.69	7.61	7.71	7.85	7.71	7.63	6.24
1383444_at	Slc24a2	1	0	0	0	1	0	8.57	8.68	9.05	8.20	8.76	0	0	0	0	0	0	0	10.21	10.31	10.38	10.44	10.58	10.39	9.41
1383445_at	Znf24	1	0	1	1	1	0	4.71	4.82	5.57	4.14	4.69	0	0	0	0	0	0	0	8.24	8.26	8.24	8.43	8.37	8.06	6.94
1383450_at	Cnot6	1	0	0	0	1	0	5.33	5.39	5.78	4.99	5.29	0	0	0	0	0	0	0	7.79	7.69	7.84	7.92	7.84	7.86	7.28
1383451_at	Wsb2	1	0	0	1	1	0	6.60	6.52	7.02	5.97	6.42	0	0	0	0	0	0	0	9.57	9.60	9.56	9.53	9.69	9.39	8.71
1383455_at	Eprs1	1	0	0	1	0	0	9.17	9.16	9.17	9.64	9.19	0	0	0	0	0	0	0	10.05	9.97	10.06	10.06	9.97	9.59	9.38
1383464_at	Pex19	1	0	0	1	1	0	6.27	6.08	6.52	5.68	6.12	0	0	0	0	0	0	0	7.70	7.72	7.78	7.71	7.74	7.69	7.33
1383466_at	Srsf6	1	0	0	1	1	0	7.00	6.71	7.30	6.42	7.05	0	0	0	0	0	0	0	8.45	8.48	8.61	8.72	8.69	8.50	7.76
1383468_at	---	1	0	0	0	1	0	5.34	5.74	5.77	5.06	5.38	0	0	0	0	0	0	0	7.35	7.48	7.69	7.74	7.75	7.15	7.16
1383470_at	Zbtb6	1	0	1	0	0	0	6.61	6.83	7.36	6.71	7.04	0	0	0	0	0	0	0	8.44	8.56	8.53	8.67	8.63	8.62	8.16
1383475_at	---	1	0	1	0	1	0	5.73	5.54	6.38	5.59	5.86	0	0	0	0	0	0	0	7.36	7.38	7.56	7.66	7.66	7.31	7.32
1383485_at	Mdm2	1	0	1	0	1	0	5.34	5.80	5.91	5.02	5.28	0	0	0	0	0	0	0	8.39	8.23	8.44	8.52	8.43	8.27	7.43
1383487_at	Traf3ip1	1	0	1	0	1	0	6.24	6.14	6.93	5.79	6.51	0	0	0	0	0	0	0	7.69	7.62	7.84	7.96	7.87	8.06	7.81
1383489_at	Il6st	1	0	0	0	1	0	7.05	7.26	7.39	6.60	7.47	0	0	0	0	0	0	0	6.94	6.71	7.08	7.10	7.01	7.39	7.24
1383500_at	Rrbp1	1	0	1	0	1	1	10.85	10.83	10.21	11.07	10.24	0	0	0	0	0	0	0	9.40	9.46	9.51	9.50	9.58	9.55	9.43
1383504_at	Cpsf6	1	0	0	0	1	0	7.54	7.34	8.00	7.26	7.58	0	0	0	0	0	0	0	9.05	9.18	9.19	9.18	9.30	9.22	8.67
1383510_at	LOC103691125	1	0	1	0	1	0	6.16	6.28	6.81	5.87	5.96	0	0	0	0	0	0	0	9.12	9.14	9.20	9.29	9.24	9.08	7.89
1383514_s_at	Naa15	1	0	1	0	1	0	5.37	5.22	6.17	4.99	5.53	0	0	0	0	0	0	0	8.41	8.45	8.57	8.80	8.70	8.53	8.05
1383528_at	---	1	0	1	0	1	0	6.84	6.89	7.54	6.68	7.14	0	0	0	0	0	0	0	7.68	7.68	7.76	7.92	7.80	8.08	8.27
1383531_at	Crebrf	1	0	1	0	1	0	6.36	6.27	7.06	6.14	6.22	0	0	0	0	0	0	0	6.35	6.32	6.23	6.21	6.17	6.24	5.79
1383533_at	LOC102551140	1	0	1	0	0	0	7.22	7.38	7.86	7.31	7.45	0	0	0	0	0	0	0	8.43	8.41	8.49	8.51	8.50	8.07	7.63
1383538_at	Ubr3	1	0	1	0	1	0	8.52	8.70	9.35	8.14	8.93	0	0	0	0	0	0	0	8.89	8.93	8.94	9.01	9.07	8.87	8.44
1383543_at	Golt1b	1	0	0	0	1	0	6.89	7.10	7.35	6.59	7.04	0	0	0	0	0	0	0	8.78	8.75	8.77	8.78	8.78	8.75	7.71
1383552_at	---	1	0	0	0	0	1	7.01	7.27	7.36	7.07	8.06	0	0	0	0	0	0	0	5.98	5.98	5.99	6.07	6.15	6.20	5.61
1383553_at	Nlrx1	1	0	0	0	0	0	6.79	6.91	6.53	7.10	6.70	0	0	0	0	0	0	0	7.12	7.01	7.09	7.08	7.18	7.14	7.13
1383564_at	Irf7	1	1	0	0	0	0	5.55	6.31	5.14	5.51	5.60	0	0	0	0	0	0	0	6.76	6.75	6.55	6.60	6.60	6.97	7.37
1383566_at	---	1	0	0	0	0	1	5.31	5.56	5.69	5.25	4.74	0	0	0	0	0	0	0	9.46	9.51	9.67	9.65	9.82	10.03	9.99
1383571_at	Rapgefl1	1	0	0	1	1	0	4.31	4.18	4.01	4.98	4.32	0	0	0	0	0	0	0	5.72	5.88	5.86	5.72	5.77	5.87	6.01
1383573_at	Tshz1	1	0	0	0	0	1	8.49	8.64	9.03	8.59	9.06	0	0	0	0	0	0	0	10.54	10.57	10.63	10.68	10.78	11.08	10.81
1383575_at	Ctnnd2	1	0	1	0	0	1	7.55	7.81	8.26	7.71	8.30	0	0	0	0	0	0	0	8.76	8.76	8.76	8.81	8.74	8.64	8.51
1383578_at	Rad51	1	0	0	1	0	1	4.96	5.09	5.23	5.61	5.79	0	0	0	0	0	0	0	10.09	10.08	10.09	10.04	9.98	9.73	9.46
1383579_at	Znf665	1	0	1	0	0	1	4.02	4.13	4.65	4.30	4.53	0	0	0	0	0	0	0	6.72	6.79	6.87	6.99	6.98	6.72	6.41
1383587_at	Hacd3	1	0	0	0	1	0	8.42	8.54	8.94	8.15	8.74	0	0	0	0	0	0	0	9.00	9.03	9.09	9.15	9.12	9.00	8.40
1383592_at	---	1	0	1	0	1	0	4.83	4.76	5.49	4.70	5.18	0	0	0	0	0	0	0	7.11	7.13	7.15	7.26	7.12	7.05	6.85
1383608_at	Arrdc2	1	1	0	0	0	0	5.15	5.66	5.06	5.27	5.20	0	0	0	0	0	0	0	7.48	7.56	7.63	7.51	7.64	7.95	7.79
1383618_at	Mrpl38	1	0	0	0	1	0	5.90	6.06	5.47	6.23	5.81	0	0	0	0	0	0	0	7.47	7.48	7.52	7.53	7.41	6.92	6.52
1383623_at	Thyn1	1	0	0	0	1	0	7.19	6.96	7.04	7.60	6.92	0	0	0	0	0	0	0	9.52	9.53	9.50	9.49	9.55	9.24	8.82
1383641_at	Ednra	1	0	0	1	0	0	5.64	5.48	5.59	5.09	5.35	0	0	0	0	0	0	0	3.93	4.02	4.02	3.89	3.83	3.97	3.74
1383651_a_at	Wdr45b	1	0	0	0	0	1	8.85	9.03	9.28	9.09	9.41	0	0	0	0	0	0	0	10.44	10.45	10.48	10.42	10.44	10.31	10.05
1383658_at	Laptm5	1	0	1	0	0	0	7.62	7.59	7.10	7.48	7.87	0	0	0	0	0	0	0	6.25	6.16	6.17	6.04	6.19	6.21	5.89
1383659_a_at	---	1	0	0	1	0	0	6.73	6.72	7.29	7.23	6.78	0	0	0	0	0	0	0	7.51	7.63	7.66	7.70	7.74	7.58	6.85
1383668_at	Mmp15	1	0	0	0	1	0	6.71	6.74	6.30	7.11	6.47	0	0	0	0	0	0	0	7.78	7.76	7.87	7.87	8.03	7.78	7.21
1383673_at	Nap1l2	1	0	1	0	1	1	5.29	5.64	6.28	5.43	6.22	0	0	0	0	0	0	0	8.60	8.67	8.62	8.81	8.72	8.51	7.57
1383677_at	Rnf168	1	0	0	0	0	1	5.30	5.50	5.78	5.61	5.92	0	0	0	0	0	0	0	8.30	8.30	8.38	8.55	8.49	8.56	8.25
1383691_at	---	1	0	1	0	0	0	5.23	5.22	5.81	5.36	5.34	0	0	0	0	0	0	0	5.76	5.88	6.01	6.00	6.01	6.00	5.09
1383692_at	Prelid2	1	0	1	0	1	0	5.99	6.23	5.03	6.29	5.78	0	0	0	0	0	0	0	5.02	5.01	4.94	4.74	4.69	4.92	4.93
1383698_at	Pdha1	1	0	0	1	0	0	7.87	7.71	8.00	7.37	7.48	0	0	0	0	0	0	0	9.96	9.95	9.99	9.95	9.85	9.76	8.95
1383708_at	Itgbl1	1	0	0	0	1	0	3.65	3.58	4.12	3.38	3.41	0	0	0	0	0	0	0	3.67	3.67	3.76	3.65	3.81	3.63	3.20
1383711_at	Nmt1	1	0	0	1	0	0	6.04	5.83	6.18	5.51	5.87	0	0	0	0	0	0	0	8.97	9.03	9.03	9.01	8.93	8.88	8.12
1383720_at	Kdm6a	1	0	1	0	0	1	7.86	8.10	8.60	8.15	8.35	0	0	0	0	0	0	0	8.06	8.24	8.22	8.27	8.28	8.21	7.95
1383722_at	Plpbp	1	0	0	1	1	0	7.43	7.38	7.97	6.55	7.15	0	0	0	0	0	0	0	9.40	9.45	9.60	9.78	9.69	9.66	8.75
1383731_at	Tmem106b	1	0	1	0	1	0	8.24	8.19	8.88	8.12	8.22	0	0	0	0	0	0	0	8.54	8.48	8.57	8.69	8.58	8.60	8.47
1383739_at	LOC100909782 /	1	0	0	0	0	1	6.08	5.72	5.73	6.03	5.60	0	0	0	0	0	0	0	4.46	4.51	4.39	4.27	4.34	4.48	3.82
1383745_at	Emilin3	1	0	1	0	1	1	5.39	5.04	4.01	5.41	4.34	0	0	0	0	0	0	0	3.78	3.70	3.73	3.78	3.73	3.71	3.16
1383750_at	---	1	0	0	0	0	1	6.66	6.82	6.95	6.87	7.72	0	0	0	0	0	0	0	4.79	4.87	5.00	4.89	5.13	5.34	4.77
1383766_at	Sgcb	1	0	1	1	1	0	6.17	6.20	7.23	5.21	6.57	0	0	0	0	0	0	0	9.59	9.66	9.61	9.61	9.64	9.59	8.95
1383767_at	---	1	0	0	0	0	1	5.65	5.67	5.88	5.26	6.92	0	0	0	0	0	0	0	7.02	6.92	7.05	7.53	7.37	7.38	6.91
1383769_at	Bmpr2	1	0	1	0	1	0	6.82	6.87	7.40	6.77	7.15	0	0	0	0	0	0	0	6.77	6.82	6.80	6.84	6.86	6.79	6.55
1383775_at	---	1	1	0	0	0	0	4.42	4.92	4.55	4.63	4.64	0	0	0	0	0	0	0	5.24	4.97	5.01	4.96	5.00	4.86	4.81
1383776_at	---	1	0	0	0	1	0	7.24	7.37	7.75	6.95	7.43	0	0	0	0	0	0	0	10.00	9.94	9.98	9.93	10.04	10.05	10.00
1383785_at	Lef1	1	0	0	0	0	1	4.61	4.54	4.92	4.85	5.55	0	0	0	0	0	0	0	5.83	5.79	5.64	5.63	5.47	5.64	6.02
1383791_at	Vma21	1	0	0	0	1	0	6.15	6.29	6.89	5.74	6.39	0	0	0	0	0	0	0	8.90	9.05	9.04	9.12	9.10	8.86	7.86
1383792_at	Sytl1	1	0	0	0	1	1	7.85	7.63	7.26	8.04	7.20	0	0	0	0	0	0	0	6.89	7.00	6.93	6.96	7.13	7.30	6.90
1383810_at	Zmym2	1	0	0	0	1	0	5.74	5.73	6.32	5.69	5.95	0	0	0	0	0	0	0	7.27	7.32	7.45	7.55	7.49	7.16	6.82
1383813_at	Arl2bp	1	0	0	1	1	0	7.37	7.34	7.80	6.63	7.31	0	0	0	0	0	0	0	9.54	9.50	9.55	9.53	9.42	9.11	8.38
1383817_at	LOC102552343	1	0	0	1	1	0	4.58	4.50	4.64	5.20	4.70	0	0	0	0	0	0	0	4.92	4.82	5.12	4.98	4.97	5.15	5.75
1383819_at	Cops2	1	0	1	0	1	0	7.03	6.87	7.64	6.59	7.21	0	0	0	0	0	0	0	9.33	9.40	9.46	9.64	9.66	9.55	8.83
1383823_at	---	1	0	0	0	0	1	4.66	4.78	4.71	4.82	4.04	0	0	0	0	0	0	0	4.52	4.47	4.40	4.27	4.29	4.29	3.85
1383825_at	Rdx	1	0	0	1	0	0	8.63	8.87	8.86	9.49	8.93	0	0	0	0	0	0	0	8.60	8.48	8.61	8.59	8.35	8.10	7.67
1383837_at	Yipf3	1	0	0	0	1	0	6.67	6.31	6.70	6.37	6.04	0	0	0	0	0	0	0	8.77	8.83	8.79	8.83	8.81	8.77	8.36
1383841_at	Pwwp2a	1	0	1	0	1	0	5.23	5.32	5.92	5.21	5.41	0	0	0	0	0	0	0	7.07	6.99	7.14	7.17	7.11	7.06	6.85
1383849_at	Rfx7	1	0	0	0	0	1	5.57	5.48	5.82	5.67	6.30	0	0	0	0	0	0	0	5.81	5.67	5.92	5.88	5.66	5.31	5.47
1383852_at	---	1	0	0	0	0	0	5.34	5.07	5.00	5.65	5.13	0	0	0	0	0	0	0	7.98	8.03	8.07	8.05	8.11	8.46	7.73
1383855_at	Degs2	1	0	1	0	1	0	6.80	6.57	5.79	6.85	6.85	0	0	0	0	0	0	0	4.01	3.97	4.05	3.79	3.84	3.95	4.36
1383861_at	Trim9	1	0	1	0	0	0	6.13	6.47	6.79	6.47	6.55	0	0	0	0	0	0	0	7.93	7.99	7.90	7.84	7.92	8.17	8.14
1383867_at	Eif5a2	1	0	1	0	1	0	3.99	3.61	4.60	3.90	4.34	0	0	0	0	0	0	0	7.89	7.83	8.01	8.07	7.98	8.11	8.39
1383869_at	Eml6	1	0	1	0	1	1	6.86	6.88	7.67	6.40	7.48	0	0	0	0	0	0	0	10.28	10.34	10.38	10.45	10.42	10.50	10.08
1383885_at	Pbrm1	1	0	1	0	1	0	5.80	6.01	6.55	5.53	6.16	0	0	0	0	0	0	0	9.24	9.16	9.25	9.34	9.23	9.09	9.04
1383899_at	Nedd4	1	0	0	1	0	0	7.22	7.37	7.69	6.13	7.16	0	0	0	0	0	0	0	9.92	9.87	10.03	10.09	10.07	9.82	9.02
1383906_at	Neurl3	1	1	1	0	0	0	5.37	5.86	4.73	5.15	5.50	0	0	0	0	0	0	0	5.86	5.96	5.86	5.87	5.89	6.18	6.25
1383907_at	Slc35e2b	1	0	1	0	1	1	7.42	7.53	8.32	7.47	7.90	0	0	0	0	0	0	0	8.26	8.30	8.39	8.46	8.50	8.66	8.27
1383909_at	Ube3a	1	0	1	0	0	0	5.68	5.74	6.27	5.99	5.76	0	0	0	0	0	0	0	7.86	7.85	8.03	8.07	7.97	7.55	6.93
1383910_at	Sh3rf1	1	0	1	0	1	0	8.21	8.01	9.07	8.51	8.01	0	0	0	0	0	0	0	9.47	9.49	9.50	9.51	9.53	9.39	9.24
1383912_at	Abracl	1	0	0	0	0	1	5.46	5.41	5.75	5.83	6.35	0	0	0	0	0	0	0	9.01	9.15	9.13	9.25	9.37	8.87	8.12
1383914_at	Irag1	1	0	0	0	0	1	5.85	5.75	5.39	5.86	5.35	0	0	0	0	0	0	0	5.10	5.05	5.01	4.98	4.85	5.07	4.53
1383919_at	---	1	0	0	0	0	1	5.39	5.15	5.49	5.82	4.57	0	0	0	0	0	0	0	4.82	4.44	4.86	4.60	4.49	4.29	3.83
1383920_at	Amt	1	0	0	0	1	0	6.23	6.35	6.02	6.61	6.42	0	0	0	0	0	0	0	6.47	6.37	6.62	6.48	6.52	6.48	6.11
1383922_a_at	Naa10	1	0	0	1	1	0	5.43	5.54	5.30	6.02	5.42	0	0	0	0	0	0	0	6.80	6.84	6.95	7.14	6.85	6.45	6.66
1383929_at	---	1	1	0	0	0	0	8.04	7.54	8.49	7.87	8.35	0	0	0	0	0	0	0	9.87	10.01	9.94	10.11	10.22	10.08	9.51
1383934_at	---	1	0	1	0	1	1	6.71	6.96	7.43	6.66	7.21	0	0	0	0	0	0	0	7.77	7.70	7.69	7.63	7.62	7.78	7.46
1383938_at	Ncoa7	1	0	1	0	0	0	7.77	7.86	8.22	7.37	8.11	0	0	0	0	0	0	0	11.32	11.36	11.38	11.39	11.45	10.97	10.63
1383939_at	---	1	0	0	1	0	0	4.05	4.19	4.16	4.52	3.88	0	0	0	0	0	0	0	5.45	5.62	5.41	5.52	5.60	5.92	5.48
1383951_at	Rnf145	1	0	1	0	1	0	7.09	7.35	7.68	6.89	7.53	0	0	0	0	0	0	0	9.83	9.89	9.98	9.97	9.94	9.78	9.75
1383964_at	Ankrd50	1	0	0	0	0	1	6.48	6.50	6.80	6.52	7.14	0	0	0	0	0	0	0	7.43	7.46	7.58	7.64	7.66	7.83	7.79
1383965_at	Ncbp2	1	0	1	0	0	1	7.48	7.68	8.31	7.62	8.15	0	0	0	0	0	0	0	9.91	10.01	9.96	10.08	10.13	10.10	9.75

Probeset ID	Gene Symbol	Islet Data Set (log2 ratio ≥0.263; FDR <20%) union n=5325 1=YES 0=NO	Islet DRlyp/lyp ND vs ND+Lp299v (log2 ratio ≥0.263; FDR<20%) n=429 1=YES 0=NO	Islet DRlyp/lyp ND vs HCD (log2 ratio ≥0.263; FDR<20%) n=2247 1=YES 0=NO	Islet DRlyp/lyp ND vs HCD+Lp299v (log2 ratio ≥0.263; FDR<20%) n=1595 1=YES 0=NO	Islet DRlyp/lyp HCD vs HCD+Lp299v (log2 ratio ≥0.263; FDR<20%) n=3026 1=YES 0=NO	Islet DRlyp/lyp ND vs Flyp/lyp ND (log2 ratio ≥ 0.263; FDR<20%) n=1950 1=YES 0=NO	Islet Mean Log2 Intensity DRlyp/lyp ND	Islet Mean Log2 Intensity DRlyp/lyp ND+Lp299v	Islet Mean Log2 Intensity DRlyp/lyp HCD	Islet Mean Log2 Intensity DRlyp/lyp HCD+Lp299v	Islet Mean Log2 Intensity Flyp40 ND	Rinm5f data union of all conditions n=3705 1=YES 0=NO	Rinm5f 30 µM propionate/6 µM butyrate vs Media (FDR<20%; 1.5 fold) n=3 1=YES 0=NO	Rinm5f 60 µM propionate/12 µM butyrate vs Media (FDR<20%; 1.5 fold) n=18 1=YES 0=NO	Rinm5f 120 µM propionate/24 µM butyrate vs Media (FDR<20%; 1.5 fold) n=164 1=YES 0=NO	Rinm5f 240 µM propionate/48 µM butyrate vs Media (FDR<20%; 1.5 fold) n=268 1=YES 0=NO	Rinm5f 1500 µM propionate/300 µM butyrate vs Media (FDR<20%; 1.5 fold) n=2491 1=YES 0=NO	Rinm5f 500 µM propionate/500 µM butyrate vs Media (FDR<20%; 1.5 fold) n=3001 1=YES 0=NO	Rinm5f Mean Log2 Intensity Media only	Rinm5f Mean Log2 Intensity 30 µM propionate/6 µM butyrate	Rinm5f Mean Log2 Intensity 60 µM propionate/12 µM butyrate	Rinm5f Mean Log2 Intensity 120 µM propionate/24 µM butyrate	Rinm5f Mean Log2 Intensity 240 µM propionate/48 µM butyrate	Rinm5f Mean Log2 Intensity 1500 µM propionate/300 µM butyrate	Rinm5f Mean Log2 Intensity 500 µM propionate/500 µM butyrate
1383973_at	Tango6	1	0	0	0	1	0	5.78	5.78	5.46	6.08	5.91	0	0	0	0	0	0	0	5.79	5.65	5.64	5.62	5.52	5.54	5.84
1383978_at	Ficd	1	0	0	0	0	1	8.36	8.67	8.23	8.38	9.04	0	0	0	0	0	0	0	8.17	8.17	8.24	8.28	8.23	8.14	8.20
1383980_at	Unc5a	1	0	1	0	1	1	6.18	6.01	5.64	6.21	5.62	0	0	0	0	0	0	0	8.20	8.14	8.25	8.05	8.21	8.35	8.14
1383985_at	Cdh15	1	0	1	0	0	0	5.87	6.01	5.33	5.76	5.78	0	0	0	0	0	0	0	6.32	6.41	6.28	6.20	6.15	6.35	6.31
1383986_at	Tpm3	1	0	0	0	1	0	8.93	9.16	8.64	9.21	8.99	0	0	0	0	0	0	0	10.89	10.84	10.89	10.92	10.89	10.73	10.28
1383998_at	Ppp4r3b	1	0	1	0	1	0	7.68	7.72	8.38	7.49	7.79	0	0	0	0	0	0	0	9.88	9.95	9.96	10.01	9.95	9.75	9.34
1383999_at	Sppl2a	1	0	1	0	1	0	7.19	7.26	7.82	6.99	7.55	0	0	0	0	0	0	0	8.74	8.76	8.81	8.89	8.95	8.97	8.30
1384004_at	Gpr158	1	0	1	0	1	1	6.92	7.35	7.79	6.84	7.83	0	0	0	0	0	0	0	8.16	8.15	8.18	8.16	8.30	8.47	8.33
1384005_at	Dr1	1	0	0	1	1	0	5.87	5.82	6.32	5.29	5.74	0	0	0	0	0	0	0	7.59	7.56	7.69	7.75	7.55	7.46	6.63
1384007_at	Atl1	1	0	1	1	0	1	6.93	6.67	4.87	5.19	5.61	0	0	0	0	0	0	0	5.68	5.63	5.94	5.77	5.70	5.79	6.13
1384017_at	Lrrk1	1	0	0	0	1	0	5.14	5.17	4.94	5.51	5.14	0	0	0	0	0	0	0	4.67	4.67	4.75	4.45	4.63	4.56	4.73
1384018_at	---	1	0	0	1	1	0	6.32	6.15	6.54	5.79	6.02	0	0	0	0	0	0	0	8.60	8.51	8.54	8.55	8.51	8.64	8.46
1384022_at	Agfg1	1	0	1	0	1	0	7.15	7.22	7.83	6.87	7.49	0	0	0	0	0	0	0	9.60	9.57	9.64	9.68	9.68	9.83	10.18
1384025_at	Pcsk2	1	0	1	1	1	1	11.32	11.57	12.06	10.83	11.86	0	0	0	0	0	0	0	11.15	11.16	11.21	11.32	11.43	11.50	11.10
1384039_at	Camsap2	1	0	0	0	1	0	6.09	6.12	6.65	5.73	6.39	0	0	0	0	0	0	0	8.96	9.02	9.10	9.15	9.10	8.78	8.75
1384043_at	---	1	0	0	0	1	0	4.85	4.60	5.09	4.40	4.43	0	0	0	0	0	0	0	7.49	7.42	7.51	7.62	7.54	7.51	6.90
1384044_at	Sgcb	1	0	1	1	1	0	5.49	5.86	6.65	4.55	5.63	0	0	0	0	0	0	0	9.75	9.71	9.70	9.66	9.66	9.68	8.51
1384046_at	Mtfr1	1	0	0	0	1	0	4.87	4.66	5.26	4.54	4.84	0	0	0	0	0	0	0	7.77	7.78	7.94	8.00	8.02	7.96	7.55
1384069_at	Lrp11	1	1	1	1	1	0	7.66	8.23	8.72	7.05	8.08	0	0	0	0	0	0	0	9.83	9.80	9.83	9.75	9.79	9.98	9.69
1384073_at	Adhfe1	1	1	0	1	0	1	7.20	6.69	6.75	6.67	6.38	0	0	0	0	0	0	0	4.36	4.43	4.45	4.34	4.35	4.74	4.45
1384080_at	Greb1l	1	0	1	1	1	0	7.34	7.28	6.72	7.91	7.13	0	0	0	0	0	0	0	5.40	5.55	5.70	5.83	5.78	5.83	5.31
1384097_at	Fam3b	1	0	1	0	0	0	4.26	4.02	3.63	3.98	3.85	0	0	0	0	0	0	0	3.85	3.86	3.82	3.77	3.79	3.94	3.98
1384100_at	---	1	0	1	0	0	0	5.76	5.73	5.22	5.75	5.71	0	0	0	0	0	0	0	6.09	6.03	6.06	6.01	6.00	6.42	6.03
1384104_at	---	1	0	0	0	0	1	3.43	3.66	3.71	3.76	4.04	0	0	0	0	0	0	0	5.31	5.29	5.42	5.15	5.28	5.52	6.26
1384106_at	Sgms1	1	0	1	0	0	0	6.50	6.63	7.30	6.72	6.38	0	0	0	0	0	0	0	8.11	8.13	8.26	8.33	8.24	8.29	7.57
1384110_at	Dock4	1	0	0	0	0	1	5.07	5.07	5.50	4.95	5.93	0	0	0	0	0	0	0	8.51	8.57	8.74	8.83	8.81	8.99	8.60
1384112_at	---	1	0	1	1	0	1	6.45	6.48	7.24	7.04	7.20	0	0	0	0	0	0	0	7.12	7.16	7.29	7.37	7.50	7.36	6.57
1384116_at	Vps37d	1	1	0	0	0	0	5.72	6.27	6.04	6.08	5.95	0	0	0	0	0	0	0	7.68	7.73	7.78	7.72	7.71	7.70	7.48
1384118_at	Znf329	1	0	0	0	1	0	4.42	4.37	4.90	4.10	4.76	0	0	0	0	0	0	0	5.58	5.69	5.79	5.71	5.57	5.59	4.92
1384126_a_at	Cebpa	1	0	1	1	1	1	5.80	5.59	5.02	6.71	4.98	0	0	0	0	0	0	0	3.33	3.42	3.44	3.41	3.40	3.67	3.58
1384128_at	Cep104	1	0	1	0	0	1	5.60	5.63	6.33	5.75	6.46	0	0	0	0	0	0	0	7.44	7.46	7.55	7.51	7.63	7.27	6.48
1384131_at	Atl2	1	0	1	1	1	0	6.93	6.94	7.61	6.03	6.63	0	0	0	0	0	0	0	9.39	9.35	9.35	9.32	9.26	8.93	7.75
1384139_at	Gjc1	1	0	0	0	1	0	5.35	5.22	5.78	5.05	5.35	0	0	0	0	0	0	0	4.68	4.66	4.74	4.61	4.63	4.79	4.74
1384144_at	Nxn	1	0	0	0	1	0	4.79	4.80	4.30	5.10	4.52	0	0	0	0	0	0	0	5.63	5.60	5.56	5.49	5.48	5.71	6.05
1384146_at	---	1	0	0	1	1	0	6.89	6.96	6.70	7.71	6.70	0	0	0	0	0	0	0	7.61	7.62	7.66	7.58	7.53	7.32	6.68
1384147_at	Eif1ax	1	0	1	0	1	1	6.35	6.26	6.94	6.06	6.86	0	0	0	0	0	0	0	8.55	8.61	8.54	8.74	8.89	8.63	7.37
1384157_at	Arl8b	1	0	0	1	1	0	7.52	7.58	8.03	6.52	7.59	0	0	0	0	0	0	0	10.21	10.23	10.23	10.30	10.30	10.41	9.63
1384164_at	Wwp1	1	0	0	1	0	0	5.54	5.75	5.80	6.02	5.83	0	0	0	0	0	0	0	6.43	6.51	6.61	6.51	6.59	6.49	6.32
1384169_a_at	Vav2	1	0	1	0	0	1	5.01	5.29	5.61	5.41	5.69	0	0	0	0	0	0	0	8.44	8.62	8.68	8.75	8.92	9.02	8.92
1384177_at	Gramd2b	1	0	0	0	1	0	6.26	6.27	6.72	5.84	6.40	0	0	0	0	0	0	0	7.81	7.90	7.78	7.61	7.55	7.46	6.40
1384184_at	---	1	0	0	0	0	1	4.96	5.20	5.45	4.93	5.66	0	0	0	0	0	0	0	7.61	7.50	7.71	7.69	7.71	7.66	7.21
1384187_at	Ap1s2	1	0	1	1	1	0	9.23	9.24	10.09	8.14	9.60	0	0	0	0	0	0	0	12.97	13.05	13.07	13.14	13.21	13.50	13.13
1384204_at	Srsf10	1	0	0	0	0	1	6.21	5.95	6.27	6.35	6.78	0	0	0	0	0	0	0	7.96	8.02	8.17	8.42	8.34	8.52	8.20
1384213_at	Pdcd6ip	1	0	0	0	0	1	7.41	7.54	7.90	7.51	8.00	0	0	0	0	0	0	0	8.77	8.83	8.92	8.94	9.00	8.97	9.03
1384226_at	---	1	0	0	0	0	1	6.62	6.81	7.09	6.45	7.14	0	0	0	0	0	0	0	4.09	4.08	4.02	3.89	3.94	4.27	4.20
1384251_at	Lrrc27	1	0	1	0	0	0	6.06	5.96	5.47	5.87	5.81	0	0	0	0	0	0	0	5.37	5.47	5.49	5.29	5.39	5.23	5.28
1384255_at	Napg	1	0	0	0	1	0	4.72	4.81	5.06	4.26	4.60	0	0	0	0	0	0	0	8.35	8.34	8.38	8.47	8.44	8.54	7.14
1384257_at	Nono	1	0	1	0	1	0	7.95	8.02	8.79	7.66	7.87	0	0	0	0	0	0	0	10.58	10.63	10.68	10.77	10.69	10.69	10.20
1384260_at	FRAT2	1	0	1	0	1	0	6.35	6.37	5.77	6.39	6.07	0	0	0	0	0	0	0	7.58	7.52	7.52	7.33	7.41	7.80	6.79
1384263_at	LOC103691414 /	1	0	0	1	1	0	5.89	6.08	5.72	6.47	5.71	0	0	0	0	0	0	0	8.32	8.29	8.35	8.44	8.33	8.34	7.82
1384270_at	---	1	0	1	0	0	0	3.80	3.97	4.52	3.84	4.16	0	0	0	0	0	0	0	7.87	7.87	8.01	8.15	8.16	7.94	7.73
1384271_at	IgG-2a	1	0	0	0	0	1	3.71	3.80	3.67	3.76	4.63	0	0	0	0	0	0	0	3.70	3.77	3.84	3.63	3.72	3.70	3.49
1384279_at	Fam117b	1	0	1	0	0	1	5.51	5.81	6.05	5.62	6.18	0	0	0	0	0	0	0	6.73	6.73	6.76	6.74	6.67	6.78	6.34
1384287_at	Ctif	1	0	0	0	1	0	4.40	4.32	3.96	4.60	4.12	0	0	0	0	0	0	0	6.38	6.37	6.49	6.37	6.30	6.22	6.36
1384290_at	---	1	0	1	0	1	0	6.60	6.83	7.39	6.44	6.96	0	0	0	0	0	0	0	9.09	9.06	9.07	9.11	9.10	8.92	8.29
1384301_at	---	1	0	1	0	0	0	4.17	4.31	4.73	4.50	4.30	0	0	0	0	0	0	0	4.48	4.37	4.65	4.70	4.71	4.22	3.77
1384302_at	Slc6a17	1	0	1	0	0	0	7.30	7.54	7.90	7.36	7.53	0	0	0	0	0	0	0	10.84	10.87	10.98	10.94	10.89	10.80	10.54
1384306_at	---	1	0	1	1	1	0	6.78	7.21	4.98	6.14	6.41	0	0	0	0	0	0	0	4.70	4.63	4.57	4.36	4.44	4.55	4.27
1384307_at	---	1	0	1	1	1	1	5.88	5.92	5.14	6.48	5.38	0	0	0	0	0	0	0	4.09	4.08	3.99	3.96	3.83	4.12	3.51
1384326_at	Nedd4	1	0	0	1	0	0	4.54	4.41	4.48	3.88	4.19	0	0	0	0	0	0	0	5.95	5.93	6.15	6.15	6.12	5.82	4.81
1384340_a_at	Naa10	1	0	0	0	1	0	5.60	5.68	5.17	5.92	5.44	0	0	0	0	0	0	0	7.32	7.33	7.39	7.57	7.27	7.03	6.87
1384345_at	Ptgr2	1	0	1	0	1	0	4.16	4.07	4.94	3.83	4.05	0	0	0	0	0	0	0	3.27	3.36	3.37	3.35	3.36	3.27	2.93
1384362_at	Zbtb24	1	0	0	0	1	0	5.43	5.47	5.24	5.83	5.53	0	0	0	0	0	0	0	6.23	6.25	6.28	6.22	6.20	6.09	5.90
1384366_at	Cops2	1	0	0	1	1	0	7.02	6.94	7.40	6.28	6.90	0	0	0	0	0	0	0	9.47	9.52	9.56	9.71	9.67	9.65	9.11
1384367_at	Hist2h3c2 /// LC	1	0	0	1	1	0	5.81	5.87	5.42	6.98	5.93	0	0	0	0	0	0	0	4.34	4.28	4.24	4.18	4.19	4.13	4.03
1384368_at	Slc25a16	1	0	0	0	1	0	6.29	6.19	6.75	5.86	6.57	0	0	0	0	0	0	0	8.81	8.83	8.84	8.91	8.98	8.77	8.65
1384384_at	C2cd5	1	0	1	0	0	0	4.86	4.79	5.45	4.79	5.12	0	0	0	0	0	0	0	7.08	7.04	7.32	7.24	7.16	7.13	6.75
1384387_at	Gtf2a1	1	0	1	0	1	0	4.79	4.76	5.57	4.81	5.02	0	0	0	0	0	0	0	8.11	8.20	8.36	8.54	8.46	8.34	7.73
1384400_at	Pde4c	1	0	0	0	1	0	7.65	7.67	7.13	7.75	7.26	0	0	0	0	0	0	0	7.41	7.33	7.34	7.40	7.42	7.80	7.50
1384407_at	Nmral1	1	0	0	1	0	0	6.92	7.20	7.01	7.49	6.52	0	0	0	0	0	0	0	8.93	8.90	9.01	9.05	8.98	8.92	8.68
1384411_at	Glcci1	1	0	1	0	0	1	6.11	6.47	6.70	6.24	6.90	0	0	0	0	0	0	0	7.49	7.57	7.58	7.61	7.65	7.63	6.98
1384423_at	Nt5c2	1	0	1	1	1	0	6.50	6.60	7.38	5.99	6.70	0	0	0	0	0	0	0	10.48	10.59	10.48	10.39	10.40	10.29	9.67
1384427_at	Mdm2	1	0	1	1	1	0	6.44	6.56	7.07	5.83	6.24	0	0	0	0	0	0	0	9.55	9.56	9.61	9.71	9.60	9.52	9.01
1384430_at	Dph3	1	0	0	0	0	1	4.82	5.14	5.09	4.97	5.32	0	0	0	0	0	0	0	6.51	6.57	6.60	6.59	6.50	6.43	6.17
1384449_at	Pvt1	1	0	0	0	1	1	6.31	6.16	5.98	6.64	5.62	0	0	0	0	0	0	0	7.62	7.63	7.68	7.75	7.65	7.66	7.46
1384460_at	Mafa	1	0	0	0	0	1	10.64	11.06	11.15	10.60	11.65	0	0	0	0	0	0	0	10.70	10.69	10.82	10.89	10.92	11.22	10.68
1384470_at	Maneal	1	0	1	0	1	1	5.80	6.03	6.69	5.84	6.79	0	0	0	0	0	0	0	10.05	9.98	10.00	10.00	9.91	9.71	9.15
1384499_at	Acsm1 /// Acsm	1	0	1	0	1	1	8.64	8.37	6.56	8.21	6.30	0	0	0	0	0	0	0	4.28	4.39	4.29	4.25	4.24	4.30	3.79
1384517_at	Znf496	1	0	0	0	0	1	5.64	5.38	5.44	5.98	5.15	0	0	0	0	0	0	0	6.65	6.48	6.57	6.46	6.48	6.29	6.41
1384540_at	Lrfn3	1	1	0	0	0	0	6.25	6.86	6.27	6.65	6.45	0	0	0	0	0	0	0	7.78	7.84	7.86	7.72	7.85	8.02	8.46
1384546_at	Gpr161	1	0	1	0	1	0	6.62	6.54	7.22	6.43	6.84	0	0	0	0	0	0	0	7.29	7.27	7.47	7.58	7.60	7.76	7.62
1384550_at	Cxorf38	1	0	0	0	1	0	3.90	3.95	4.47	3.59	3.95	0	0	0	0	0	0	0	7.00	7.02	7.10	7.13	7.10	6.69	6.21
1384558_at	Plac9	1	0	0	0	0	1	4.68	4.70	4.44	4.45	4.09	0	0	0	0	0	0	0	4.77	4.78	4.48	4.46	4.59	4.58	4.36
1384559_at	Psx1	1	0	1	0	0	0	6.98	6.94	6.37	6.77	6.85	0	0	0	0	0	0	0	5.13	5.23	5.12	5.09	4.97	5.14	4.67
1384584_at	---	1	0	1	0	1	0	6.48	6.50	7.38	6.48	6.72	0	0	0	0	0	0	0	8.54	8.56	8.49	8.48	8.38	8.27	7.94
1384599_at	---	1	0	0	0	0	1	4.76	4.65	4.76	5.02	3.96	0	0	0	0	0	0	0	4.59	4.39	4.70	4.70	4.50	4.45	3.82
1384615_at	---	1	0	1	0	1	0	6.34	6.42	7.38	5.91	6.52	0	0	0	0	0	0	0	7.47	7.26	7.50	7.41	7.54	7.29	6.45
1384622_at	Fra10ac1	1	0	1	0	0	0	4.87	4.78	4.32	4.63	4.57	0	0	0	0	0	0	0	5.05	4.96	5.06	4.85	4.87	5.00	4.38
1384638_at	---	1	0	1	0	0	0	6.98	6.85	6.39	6.91	6.56	0	0	0	0	0	0	0	6.83	6.85	6.93	7.00	7.02	7.35	7.11
1384675_at	Apeg3	1	0	0	0	0	1	7.68	7.72	7.76	7.62	8.19	0	0	0	0	0	0	0	5.99	5.85	5.89	6.09	5.87	5.80	6.05
1384679_at	Slc9b2	1	0	0	1	0	0	3.30	3.58	3.48	3.86	3.51	0	0	0	0	0	0	0	6.07	5.98	6.08	5.97	5.99	6.08	6.62
1384703_at	---	1	0	1	0	0	1	6.21	6.48	6.89	6.29	6.69	0	0	0	0	0	0	0	8.49	8.53	8.65	8.87	8.79	8.84	8.35
1384714_x_at	Rpl8	1	0	0	1	0	0	9.17	8.99	9.40	9.80	8.91	0	0	0	0	0	0	0	10.10	10.15	10.01	10.00	10.03	9.90	9.74
1384726_at	---	1	0	0	1	0	0	3.51	3.71	3.65	4.14	3.61	0	0	0	0	0	0	0	3.29	3.37	3.36	3.33	3.34	3.31	2.99
1384756_at	Slc43a2	1	1	1	0	0	1	7.82	8.36	8.41	7.94	8.59	0	0	0	0	0	0	0	7.27	7.15	7.55	7.70	7.70	7.71	7.81
1384765_at	Rhbdf2	1	0	0	0	1	0	5.89	5.71	5.59	6.15	5.76	0	0	0	0	0	0	0	6.93	6.99	7.02	6.86	6.94	7.08	7.11
1384772_at	Prkacb	1	0	1	1	1	0	7.51	7.70	8.38	6.41	7.57	0	0	0	0	0	0	0	8.71	8.61	8.72	8.77	8.70	8.33	7.62
1384794_at	Gmeb1	1	0	0	0	0	1	4.05	3.92	3.88	4.20	3.57	0	0	0	0	0	0	0	6.20	6.33	6.36	6.26	6.21	6.26	6.73
1384795_at	Znf638	1	0	1	0	1	0	4.97	5.07	5.66	4.77	5.19	0	0	0	0	0	0	0	7.08	7.18	7.27	7.46	7.42	7.59	6.48
1384797_at	Atl3	1	0	0	1	1	0	5.72	5.73	6.11	4.93	5.43	0	0	0	0	0	0	0	8.85	8.70	8.87	8.98	8.87	8.76	7.79
1384809_at	Adgra1	1	0	1	0	0	0	3.94	3.77	3.38	3.70	3.72	0	0	0	0	0	0	0	6.37	6.24	6.48	6.52	6.52	6.64	6.32
1384815_at	---	1	0	0	1	1	0	6.62	6.84	6.34	7.11	6.66	0	0	0	0	0	0	0	6.91	6.99	7.16	6.99	7.07	7.13	6.70
1384816_at	Cxadr	1	0	0	0	0	1	3.64	3.81	4.07	3.67	4.36	0	0	0	0	0	0	0	4.36	4.27	4.40	4.34	4.40	4.25	3.87
1384824_at	Pcdh18	1	0	0	0	1	0	5.04	4.86	5.52	4.77	4.94	0	0	0	0	0	0	0	3.70	3.55	3.79	3.54	3.59	3.63	3.01
1384833_at	---	1	0	0	0	0	1	5.65	5.76	5.37	5.92	6.27	0	0	0	0	0	0	0	6.15	6.14	6.41	6.35	6.34	6.29	6.62
1384852_at	Rab27a	1	0	1	1	1	0	6.91	6.88	7.81	6.02	6.85	0	0	0	0	0	0	0	8.04	8.14	8.21	8.28	8.24	7.97	6.90
1384854_at	C5orf24	1	0	1	1	1	0	6.06	6.20	6.72	5.15	6.32	0	0	0	0	0	0	0	8.15	8.10	8.17	8.22	8.16	8.05	6.97
1384858_a_at	Utp23	1	0	0	0	1	0	7.06	6.99	6.68	7.42	6.86	0	0	0	0	0	0	0	8.74	8.67	8.64	8.51	8.40	8.27	8.10
1384859_at	---	1	0	1	1	1	0	5.86	6.10	6.47	5.10	5.64	0	0	0	0	0	0	0	5.31	5.21	5.61	5.62	5.49	5.55	4.56
1384866_at	Entpd3	1	0	1	0	1	1	9.00	9.16	9.65	8.77	9.53	0	0	0	0	0	0	0	9.48	9.55	9.62	9.63	9.71	9.49	8.76
1384874_at	Cnpy1	1	0	0	1	0	0	4.87	4.95	5.00	5.45	4.78	0	0	0	0	0	0	0	4.51	4.55	4.48	4.34	4.47	4.41	4.05
1384899_at	Spin4	1	0	1	0	0	1	3.92	4.17	4.56	4.06	4.47	0	0	0	0	0	0	0	6.01	5.94	5.94	5.94	5.90	6.13	6.08
1384901_at	Znf451	1	0	1	0	1	0	5.16	4.94	5.96	4.95	5.33	0	0	0	0	0	0	0	8.66	8.67	8.65	8.79	8.78	8.74	8.55
1384903_at	Gpt2	1	0	1	0	0	0	6.50	6.31	5.85	6.30	6.24	0	0	0	0	0	0	0	5.77	5.82	5.82	5.67	5.80	5.70	5.16
1384907_at	LOC103693761	1	0	1	0	1	1	6.93	7.20	7.88	6.64	7.69	0	0	0	0	0	0	0	8.87	8.91	8.98	9.06	9.12	9.11	8.76
1384922_at	LOC102551114	1	0	0	1	1	0	6.58	6.63	6.99	5.82	6.81	0	0	0	0	0	0	0	4.41	4.36	4.40	4.58	4.47	4.68	4.93
1384943_at	C21orf91	1	0	0	1	1	0	4.84	4.98	5.26	4.22	4.72	0	0	0	0	0	0	0	6.00	6.03	6.21	6.35	6.21	6.55	6.25
1384945_at	Fyttd1	1	0	0	1	1	0	6.07	5.96	6.63	5.48	5.92	0	0	0	0	0	0	0	8.17	8.08	8.19	8.27	8.27	8.29	7.66
1384950_at	Pi4k2b	1	0	0	1	0	1	5.73	5.47	5.68	5.03	5.07	0	0	0	0	0	0	0	7.74	7.74	7.78	7.82	7.80	7.69	7.26
1384957_at	Atf6	1	0	0	1	1	0	5.78	6.05	5.58	6.33	5.83	0	0	0	0	0	0	0	5.98	5.84	5.75	5.77	5.74	5.61	5.39
1384960_at	Cftr	1	1	0	0	0	1	6.29	5.62	5.97	6.05	5.67	0	0	0	0	0	0	0	3.53	3.66	3.67	3.58	3.74	3.63	3.45
1384963_at	Cecr2	1	0	0	0	1	0	4.39	4.38	4.24	4.84	4.18	0	0	0	0	0	0	0	5.43	5.37	5.55	5.48	5.39	5.86	5.56
1384968_at	Sh3bp2	1	0	0	0	1	0	5.23	5.27	4.76	5.38	4.80	0	0	0	0	0	0	0	5.03	5.10	5.01	5.01	5.11	4.91	4.81
1384977_at	Spen	1	0	0	1	0	0	5.37	5.69	5.38	5.91	5.72	0	0	0	0	0	0	0	5.16	5.20	5.13	4.93	5.01	5.17	5.66
1384996_at	Adrbk2	1	0	0	0	1	0	6.77	6.76	7.26	6.51	6.82	0	0	0	0	0	0	0	9.26	9.22	9.29	9.26	9.23	9.10	9.09
1385014_at	Nudt10 /// Nudt	1	0	1	0	1	1	6.75	6.93	7.57	6.29	7.23	0	0	0	0	0	0	0	10.97	10.93	11.13	11.41	11.30	10.90	10.19
1385017_at	Znf770	1	0	0	1	0	0	3.86	4.26	4.06	4.38	4.08	0	0	0	0	0	0	0	5.08	4.97	5.26	5.20	5.14	5.06	4.66
1385025_a_at	Gpr137b	1	0	1	0	1	1	4.46	4.37	5.13	4.37	5.33	0	0	0	0	0	0	0	6.36	6.31	6.38	6.43	6.37	6.86	6.71
1385053_at	Epha3	1	0	1	0	1	1	6.42	6.38	5.80	6.81	4.50	0	0	0	0	0	0	0	3.95	3.94	4.02	3.79	4.00	4.16	3.73
1385054_at	Zfp17	1	0	1	0	1	0	5.42	5.39	5.98	5.26	5.47	0	0	0	0	0	0	0	7.38	7.38	7.49	7.48	7.48	7.47	6.98
1385059_at	Znf655	1	0	1	1	1	0	4.29	4.32	5.12	3.52	4.00	0	0	0	0	0	0	0	7.61	7.65	7.70	7.89	7.76	7.61	6.36
1385073_at	LOC102551114	1	0	0	1	1	0	7.87	7.98	8.27	6.52	8.28	0	0	0	0	0	0	0	6.67	6.67	6.73	6.79	6.65	6.61	6.90
1385076_at	Zbed6	1	0	0	1	1	0	5.51	5.80	5.27	6.07	5.71	0	0	0	0	0	0	0	6.35	6.31	6.30	6.16	6.15	6.12	5.58
1385082_at	Epha3	1	0	1	0	1	1	5.83	5.54	4.97	6.12	4.27	0	0	0	0	0	0	0	3.67	3.66	3.76	3.55	3.65	3.82	3.20
1385087_at	ZBTB20	1	1	0	0	0	0	4.74	5.22	5.13	4.91	5.02	0	0	0	0	0	0	0	5.22	5.30	5.35	5.23	5.14	5.26	5.16

Probeset ID	Gene Symbol	Islet Data Set (log2 ratio ≥0.263; FDR <20%) union n=5325 1=YES 0=NO	Islet DRlyp/lyp ND vs ND+Lp299v (log2 ratio ≥0.263; FDR<20%) n=429 1=YES 0=NO	Islet DRlyp/lyp ND vs HCD (log2 ratio ≥0.263; FDR<20%) n=2247 1=YES 0=NO	Islet DRlyp/lyp ND vs HCD+Lp299v (log2 ratio ≥0.263; FDR<20%) n=1595 1=YES 0=NO	Islet DRlyp/lyp HCD vs HCD+Lp299v (log2 ratio ≥0.263; FDR<20%) n=3026 1=YES 0=NO	Islet DRlyp/lyp ND vs Flyp/lyp ND (log2 ratio ≥ 0.263; FDR<20%) n=1950 1=YES 0=NO	Islet Mean Log2 Intensity DRlyp/lyp ND	Islet Mean Log2 Intensity DRlyp/lyp ND+Lp299v	Islet Mean Log2 Intensity DRlyp/lyp HCD	Islet Mean Log2 Intensity DRlyp/lyp HCD+Lp299v	Islet Mean Log2 Intensity Flyp40 ND	Rinm5f data union of all conditions n=3705 1=YES 0=NO	Rinm5f 30 µM propionate/ 6 µM butyrate vs Media (FDR<20%; 1.5 fold) n=3 1=YES 0=NO	Rinm5f 60 µM propionate/ 12 µM butyrate vs Media (FDR<20%; 1.5 fold) n=18 1=YES 0=NO	Rinm5f 120 µM propionate/ 24 µM butyrate vs Media (FDR<20%; 1.5 fold) n=164 1=YES 0=NO	Rinm5f 240 µM propionate /48 µM butyrate vs Media (FDR<20%; 1.5 fold) n=268 1=YES 0=NO	Rinm5f 1500 µM propionate/ 300 µM butyrate vs Media (FDR<20%; 1.5 fold) n=2491 1=YES 0=NO	Rinm5f 500 µM propionate/ 500 µM butyrate vs Media (FDR<20%; 1.5 fold) n=3001 1=YES 0=NO	Rinm5f Mean Log2 Intensity Media only	Rinm5f Mean Log2 Intensity 30 µM propionate/ 6 µM butyrate	Rinm5f Mean Log2 Intensity 60 µM propionate/ 12 µM butyrate	Rinm5f Mean Log2 Intensity 120 µM propionate/ 24 µM butyrate	Rinm5f Mean Log2 Intensity 240 µM propionate/ 48 µM butyrate	Rinm5f Mean Log2 Intensity 1500 µM propionate /300 µM butyrate	Rinm5f Mean Log2 Intensity 500 µM propionate/ 500 µM butyrate
1385088_at	Misp3	1	0	1	0	0	0	6.81	7.06	6.06	6.57	6.64	0	0	0	0	0	0	0	7.77	7.76	7.70	7.37	7.41	7.36	7.28
1385090_at	Rad17	1	0	0	0	1	0	6.33	6.27	6.75	5.94	6.46	0	0	0	0	0	0	0	9.47	9.48	9.49	9.54	9.50	9.23	8.79
1385103_at	Aktip	1	0	0	1	1	0	7.33	7.15	7.70	6.72	7.16	0	0	0	0	0	0	0	8.70	8.74	8.87	8.83	8.82	9.02	8.31
1385119_at	---	1	0	1	0	1	0	4.42	4.82	5.56	4.13	4.33	0	0	0	0	0	0	0	7.68	7.47	7.63	7.66	7.70	7.52	5.75
1385120_at	Pof1b	1	0	1	0	0	0	5.24	4.96	4.62	4.98	4.92	0	0	0	0	0	0	0	3.63	3.44	3.54	3.43	3.49	3.47	3.05
1385126_at	---	1	0	1	0	1	0	4.31	4.26	5.16	4.29	4.75	0	0	0	0	0	0	0	6.50	6.43	6.64	6.67	6.52	6.38	6.35
1385132_at	Mybl1	1	0	1	0	1	1	3.80	3.93	4.74	3.88	4.28	0	0	0	0	0	0	0	9.35	9.33	9.47	9.51	9.44	9.17	9.23
1385138_at	Tbc1d4	1	1	0	0	0	1	8.78	8.14	8.65	8.71	8.20	0	0	0	0	0	0	0	6.81	6.76	7.02	6.83	6.89	7.06	7.03
1385143_at	Dock10	1	0	1	0	1	0	4.95	4.96	5.57	4.59	5.23	0	0	0	0	0	0	0	4.28	4.20	4.44	4.10	4.25	4.34	4.51
1385148_at	P2rx1	1	0	1	0	1	1	8.70	8.50	7.80	8.74	7.97	0	0	0	0	0	0	0	4.50	4.40	4.48	4.22	4.39	4.45	4.40
1385153_at	Zfp68	1	0	1	0	1	0	5.67	5.74	6.39	5.33	5.78	0	0	0	0	0	0	0	8.49	8.58	8.59	8.73	8.72	8.77	8.29
1385158_at	Chmp1b	1	0	1	0	0	0	5.77	5.98	6.34	5.88	5.34	0	0	0	0	0	0	0	8.96	9.07	8.98	9.02	9.08	9.11	8.39
1385168_at	Lrif1	1	0	1	0	0	0	5.31	5.65	6.07	5.48	5.69	0	0	0	0	0	0	0	7.51	7.73	7.55	7.67	7.76	7.43	6.84
1385191_at	---	1	1	1	0	1	1	4.68	5.32	5.71	4.36	5.48	0	0	0	0	0	0	0	6.46	6.40	6.59	6.76	6.69	6.80	6.75
1385205_at	---	1	0	1	1	1	0	5.11	5.01	6.68	5.78	4.96	0	0	0	0	0	0	0	3.32	3.23	3.28	3.33	3.29	3.41	2.78
1385214_at	LOC498906	1	0	0	1	1	0	7.71	7.93	8.21	6.61	7.45	0	0	0	0	0	0	0	9.43	9.37	9.49	9.61	9.49	9.07	7.89
1385215_at	Runx1t1	1	0	0	0	0	1	3.30	3.20	3.19	3.24	3.95	0	0	0	0	0	0	0	5.86	5.73	5.90	5.79	5.51	5.29	6.10
1385253_at	Atg14	1	0	0	1	0	0	4.44	4.69	4.73	4.92	4.51	0	0	0	0	0	0	0	5.91	5.98	5.95	6.08	6.01	5.97	5.28
1385262_at	LOC102553760	1	0	1	0	1	0	3.63	3.40	4.27	3.45	3.42	0	0	0	0	0	0	0	4.22	4.12	4.40	4.55	4.41	4.28	3.43
1385270_s_at	Rab1b	1	0	0	1	1	0	5.33	5.02	5.83	4.76	5.18	0	0	0	0	0	0	0	9.32	9.40	9.38	9.28	9.25	9.16	8.44
1385291_a_at	P3h4	1	0	1	0	1	1	5.24	5.53	6.12	5.31	5.89	0	0	0	0	0	0	0	9.38	9.31	9.33	9.29	9.30	9.20	8.81
1385314_at	Rab14	1	0	0	1	1	0	7.82	7.74	8.29	6.91	7.38	0	0	0	0	0	0	0	9.81	9.85	9.89	9.98	9.97	9.81	8.90
1385320_at	Magix	1	0	0	0	1	0	4.91	4.88	4.54	5.14	4.62	0	0	0	0	0	0	0	4.76	4.89	4.64	4.59	4.59	4.62	4.32
1385367_at	Myo10	1	0	0	0	0	1	4.49	4.65	4.76	4.80	5.20	0	0	0	0	0	0	0	5.08	5.06	4.99	4.93	4.91	4.78	5.04
1385381_at	---	1	1	1	0	1	1	4.55	5.13	5.61	4.41	5.66	0	0	0	0	0	0	0	9.63	9.83	9.66	9.62	9.91	10.02	8.75
1385397_at	Steap4	1	1	0	1	1	1	5.68	4.62	6.04	4.33	5.19	0	0	0	0	0	0	0	3.38	3.40	3.57	3.37	3.38	3.53	2.72
1385408_at	Mga	1	0	1	0	0	0	6.11	5.88	6.65	6.24	6.48	0	0	0	0	0	0	0	6.90	6.92	7.11	7.18	7.17	7.21	7.32
1385442_at	---	1	0	0	0	0	1	6.50	6.81	6.52	6.61	7.07	0	0	0	0	0	0	0	9.09	9.14	9.08	8.94	9.02	9.31	8.61
1385443_at	Mlx	1	0	0	0	1	0	7.68	7.62	8.04	7.22	7.91	0	0	0	0	0	0	0	10.27	10.26	10.36	10.39	10.31	10.12	9.63
1385445_at	---	1	0	0	1	0	0	3.68	3.61	4.00	4.20	3.74	0	0	0	0	0	0	0	4.64	5.01	4.89	4.83	5.17	4.94	4.66
1385455_at	Prpf38b	1	1	0	0	1	0	6.53	6.23	6.97	6.16	6.86	0	0	0	0	0	0	0	8.79	8.71	8.90	8.98	8.88	8.98	8.33
1385481_at	Dsel	1	1	1	0	0	1	5.70	6.24	6.39	6.07	6.47	0	0	0	0	0	0	0	10.19	10.25	10.19	10.32	10.22	10.16	9.10
1385486_at	Bnc2	1	0	1	0	1	1	6.50	6.56	5.14	6.67	5.43	0	0	0	0	0	0	0	3.90	3.94	3.95	4.02	4.09	4.17	4.04
1385491_at	Pnma8b	1	0	1	0	1	1	6.86	6.85	7.86	6.58	7.90	0	0	0	0	0	0	0	7.61	7.47	7.72	7.74	7.80	7.93	8.20
1385493_at	Thoc2	1	0	0	1	1	0	3.54	3.30	3.92	2.96	3.36	0	0	0	0	0	0	0	6.72	6.52	6.77	6.89	6.86	6.87	5.69
1385494_at	Tbc1d23	1	1	1	0	0	0	5.96	6.59	6.54	6.25	6.27	0	0	0	0	0	0	0	7.88	7.89	7.95	8.07	7.96	7.99	7.29
1385512_at	Atg4d	1	0	0	0	0	1	6.34	6.10	6.09	6.49	5.87	0	0	0	0	0	0	0	7.84	7.98	8.05	8.09	8.05	8.01	7.53
1385529_at	Fam3b	1	0	1	1	1	1	5.87	5.66	3.92	4.98	5.09	0	0	0	0	0	0	0	3.12	3.21	3.34	3.28	3.38	3.30	3.12
1385543_at	Akap10	1	0	1	0	0	0	3.77	3.92	4.41	3.88	4.21	0	0	0	0	0	0	0	4.78	5.01	5.04	5.02	5.00	5.06	4.28
1385553_at	Ncbp2	1	0	1	1	1	0	7.18	7.16	7.91	6.53	7.41	0	0	0	0	0	0	0	9.76	9.83	9.83	9.92	9.86	9.73	8.84
1385577_at	---	1	0	1	0	1	1	4.66	4.87	5.59	4.53	5.32	0	0	0	0	0	0	0	9.28	9.44	9.43	9.44	9.55	9.62	8.94
1385582_at	Osbpl2	1	0	0	1	1	0	5.23	5.29	5.56	4.62	4.97	0	0	0	0	0	0	0	7.83	7.94	7.78	7.82	7.78	7.99	6.83
1385594_at	---	1	0	0	1	1	0	4.89	4.86	5.07	4.29	4.66	0	0	0	0	0	0	0	6.74	6.44	6.75	6.78	6.70	6.73	5.35
1385595_at	Kin	1	0	1	0	0	0	4.81	4.80	5.55	4.86	5.20	0	0	0	0	0	0	0	7.72	7.75	7.84	7.80	7.85	7.73	7.29
1385596_at	Tbc1d15	1	0	0	1	1	0	7.10	7.10	7.65	6.61	6.93	0	0	0	0	0	0	0	9.97	9.99	10.02	10.09	10.06	9.99	9.35
1385597_at	Zfp763	1	0	0	0	1	0	4.33	4.27	4.75	4.03	4.49	0	0	0	0	0	0	0	5.92	5.93	6.06	5.98	5.98	5.81	5.17
1385614_at	Mlph	1	0	0	0	1	0	6.15	6.18	5.73	6.41	6.04	0	0	0	0	0	0	0	5.62	5.54	5.48	5.26	5.23	5.40	5.07
1385620_at	Hsph1	1	1	0	1	1	0	6.17	6.65	6.41	5.62	6.61	0	0	0	0	0	0	0	8.11	8.09	8.33	8.51	8.46	8.54	8.14
1385641_at	Tnfaip3	1	1	0	0	0	0	5.93	6.62	5.82	5.80	6.40	0	0	0	0	0	0	0	6.81	6.90	6.83	6.58	6.46	6.40	6.44
1385658_at	Rnf114	1	0	0	1	1	1	7.24	6.86	7.66	6.47	6.64	0	0	0	0	0	0	0	9.17	9.37	9.33	9.41	9.32	9.53	8.55
1385663_at	Usp13	1	0	0	1	1	1	6.30	6.32	6.74	5.76	6.81	0	0	0	0	0	0	0	7.04	7.12	7.16	7.06	7.11	6.67	6.15
1385668_at	Proser2	1	1	0	0	0	1	6.73	6.20	6.60	7.08	6.13	0	0	0	0	0	0	0	4.84	4.94	5.19	5.26	5.28	5.06	5.00
1385676_at	Cd2bp2	1	0	0	1	1	1	6.78	6.50	6.79	6.05	6.20	0	0	0	0	0	0	0	8.41	8.56	8.62	8.72	8.65	8.88	8.29
1385687_at	---	1	0	1	1	1	1	9.19	9.16	6.52	7.45	8.67	0	0	0	0	0	0	0	5.14	5.12	5.28	5.00	4.98	5.19	5.06
1385690_at	Mmut	1	0	0	1	1	0	6.81	6.71	6.88	5.97	6.62	0	0	0	0	0	0	0	6.43	6.42	6.63	6.77	6.74	6.82	6.18
1385697_at	Ccnt2	1	0	1	0	0	0	5.70	5.62	6.35	5.81	5.89	0	0	0	0	0	0	0	7.47	7.60	7.62	7.70	7.81	7.63	6.81
1385699_at	Ythdf1	1	0	0	1	1	0	6.43	6.40	6.68	5.66	6.42	0	0	0	0	0	0	0	8.36	8.39	8.45	8.48	8.45	8.52	8.21
1385700_at	Cr2	1	0	0	0	0	1	3.40	3.60	3.41	3.50	4.01	0	0	0	0	0	0	0	3.56	3.61	3.72	3.49	3.57	3.59	3.34
1385709_x_at	---	1	0	0	0	0	1	7.60	7.81	7.94	7.74	8.34	0	0	0	0	0	0	0	8.62	8.60	8.75	8.75	8.76	9.02	9.34
1385717_at	Gdap1	1	0	1	0	1	1	6.78	6.76	7.70	6.47	7.72	0	0	0	0	0	0	0	9.13	9.20	9.28	9.43	9.47	9.38	8.56
1385729_at	Klhdc7a	1	0	0	0	1	0	4.39	4.52	4.09	4.66	4.83	0	0	0	0	0	0	0	3.81	3.80	3.75	3.55	3.69	3.79	3.28
1385731_at	---	1	0	1	0	1	1	6.14	6.19	7.05	5.90	6.85	0	0	0	0	0	0	0	4.57	4.69	4.74	4.59	4.50	4.54	4.35
1385734_at	---	1	0	1	0	1	0	5.55	5.57	6.29	5.23	5.77	0	0	0	0	0	0	0	6.09	6.13	6.24	6.23	6.20	5.96	4.85
1385737_at	---	1	0	1	0	0	0	3.66	3.72	4.24	3.78	3.70	0	0	0	0	0	0	0	6.53	6.52	6.87	6.76	6.86	6.80	6.00
1385760_at	Zbtb42	1	0	1	1	1	0	7.37	7.34	6.62	7.92	7.19	0	0	0	0	0	0	0	6.80	6.68	6.73	6.51	6.60	6.70	6.47
1385761_s_at	Zbtb42	1	0	1	1	1	0	8.09	8.13	7.45	8.70	7.73	0	0	0	0	0	0	0	8.06	8.00	8.03	7.81	7.86	7.89	7.92
1385773_at	Zkscan1	1	0	1	0	1	1	5.15	5.06	5.92	4.84	5.62	0	0	0	0	0	0	0	7.74	7.71	7.78	7.86	7.81	7.98	7.63
1385791_at	Vill	1	0	1	0	1	1	6.47	6.35	5.72	6.55	5.99	0	0	0	0	0	0	0	6.14	6.23	6.08	6.04	6.03	6.23	5.93
1385796_at	---	1	0	1	0	0	0	5.73	5.42	4.99	5.38	5.48	0	0	0	0	0	0	0	5.35	5.23	5.29	5.19	5.24	5.39	4.84
1385828_at	Pradc1	1	1	0	0	0	1	6.13	5.64	5.75	6.28	5.52	0	0	0	0	0	0	0	7.52	7.55	7.58	7.72	7.70	7.95	7.67
1385832_s_at	Sash3	1	0	0	0	0	1	3.75	3.88	3.71	3.75	4.35	0	0	0	0	0	0	0	3.59	3.62	3.57	3.54	3.60	3.72	3.27
1385836_at	---	1	0	1	0	1	1	6.28	6.71	7.03	6.13	6.93	0	0	0	0	0	0	0	7.30	7.38	7.38	7.44	7.53	7.39	7.09
1385838_a_at	Tm2d1	1	0	1	0	1	0	6.83	6.66	7.68	6.70	7.25	0	0	0	0	0	0	0	9.77	9.86	9.71	9.68	9.76	9.68	9.30
1385839_x_at	Tm2d1	1	0	1	0	1	0	6.93	6.79	7.62	6.71	7.15	0	0	0	0	0	0	0	9.62	9.68	9.59	9.51	9.58	9.50	8.80
1385866_at	---	1	0	1	1	0	0	4.37	4.22	3.79	3.70	4.26	0	0	0	0	0	0	0	3.80	3.77	3.90	3.72	3.64	3.75	3.31
1385871_at	Dhx36	1	0	1	1	1	0	6.58	6.62	7.62	5.86	6.26	0	0	0	0	0	0	0	8.19	8.08	8.32	8.40	8.49	8.26	6.84
1385872_at	Dhx36	1	0	0	0	1	0	6.69	6.71	7.14	6.40	6.84	0	0	0	0	0	0	0	8.85	8.90	8.98	8.94	9.00	8.95	8.46
1385883_at	Zbtb11	1	0	1	0	1	0	7.15	7.09	7.85	7.06	7.39	0	0	0	0	0	0	0	9.58	9.55	9.63	9.75	9.70	9.58	9.08
1385887_at	Cux1	1	0	0	0	0	1	5.93	6.13	6.18	6.22	6.56	0	0	0	0	0	0	0	8.48	8.37	8.46	8.44	8.38	8.23	7.57
1385889_at	---	1	0	1	1	1	0	6.25	6.54	7.18	4.47	6.44	0	0	0	0	0	0	0	8.20	8.04	8.27	8.34	8.32	7.98	7.00
1385899_at	Trim16	1	0	1	0	1	0	5.24	4.85	4.68	5.50	5.63	0	0	0	0	0	0	0	3.79	3.72	3.94	3.85	3.90	3.75	3.69
1385924_at	---	1	0	1	0	1	0	5.94	6.12	6.83	5.83	6.16	0	0	0	0	0	0	0	7.90	7.96	8.14	8.19	8.21	8.33	7.84
1385925_at	---	1	0	0	0	1	0	4.64	4.72	5.05	4.15	4.50	0	0	0	0	0	0	0	9.48	9.48	9.61	9.66	9.78	9.76	8.79
1385931_at	Hook3	1	0	1	1	0	0	4.15	4.41	4.68	5.03	4.47	0	0	0	0	0	0	0	6.26	6.20	6.23	6.39	6.08	5.74	5.98
1385933_at	Mbnl1	1	0	1	0	0	0	7.38	7.27	8.21	7.73	7.17	0	0	0	0	0	0	0	7.32	7.31	7.47	7.65	7.44	7.23	6.44
1385965_at	Ankhd1	1	0	0	0	1	0	4.08	4.10	4.55	3.85	4.47	0	0	0	0	0	0	0	6.99	6.88	7.01	7.22	7.08	6.79	5.95
1385974_at	Pard6g	1	0	0	1	1	0	5.40	5.47	5.30	6.58	5.23	0	0	0	0	0	0	0	5.88	5.86	5.93	6.01	6.01	6.12	5.19
1385977_at	Znf644	1	0	0	0	1	0	5.69	5.58	6.08	5.37	5.69	0	0	0	0	0	0	0	6.36	6.35	6.37	6.43	6.44	6.23	5.93
1386041_a_at	Klf2	1	0	0	1	0	0	3.80	3.77	3.82	3.21	3.79	0	0	0	0	0	0	0	3.46	3.38	3.54	3.24	3.40	3.35	3.10
1386061_at	Ccdc88a	1	0	0	0	0	1	3.90	4.06	4.39	3.79	4.42	0	0	0	0	0	0	0	8.59	8.57	8.74	8.79	8.84	8.76	8.69
1386065_at	Sowahc	1	0	1	0	1	1	5.66	6.04	6.44	5.68	6.20	0	0	0	0	0	0	0	9.88	9.93	9.92	9.98	10.00	10.07	9.60
1386088_at	Slc25a46	1	0	1	1	1	0	6.41	6.51	7.17	5.52	6.64	0	0	0	0	0	0	0	9.66	9.72	9.71	9.81	9.86	9.73	8.94
1386102_at	LOC100912031	1	0	1	0	0	0	6.01	6.13	6.60	6.17	6.35	0	0	0	0	0	0	0	9.07	9.27	9.28	9.25	9.40	9.30	8.46
1386120_at	LOC100912373	1	0	1	0	1	0	5.00	5.06	6.06	4.95	5.18	0	0	0	0	0	0	0	6.10	6.08	6.25	6.33	6.14	6.15	6.08
1386123_at	---	1	0	1	1	1	0	9.41	9.28	10.18	8.68	9.27	0	0	0	0	0	0	0	10.14	10.24	10.17	10.18	10.28	10.31	9.52
1386154_at	Fam135a	1	0	1	0	1	0	8.14	8.17	9.10	7.75	8.51	0	0	0	0	0	0	0	10.23	10.22	10.14	10.13	10.03	9.99	9.83
1386263_at	Nedd4	1	0	0	1	1	0	6.80	6.65	7.15	6.10	6.66	0	0	0	0	0	0	0	8.75	8.70	8.81	8.77	8.82	8.66	8.05
1386269_x_at	Sorl1	1	0	0	0	1	0	3.52	3.69	4.05	3.31	3.74	0	0	0	0	0	0	0	4.52	4.52	4.52	4.43	4.54	4.46	4.34
1386277_at	Uba7	1	1	0	0	0	0	5.55	6.04	5.64	5.78	5.76	0	0	0	0	0	0	0	6.58	6.55	6.43	6.42	6.49	6.40	5.82
1386337_at	---	1	0	0	0	1	0	6.28	6.65	5.91	6.56	6.04	0	0	0	0	0	0	0	6.41	6.32	6.37	6.36	6.34	5.92	5.68
1386344_at	---	1	0	1	0	1	1	9.20	9.33	9.92	9.04	9.80	0	0	0	0	0	0	0	10.09	10.06	10.11	10.17	10.14	10.28	10.36
1386382_at	Osbpl1a	1	0	0	0	0	1	5.67	5.60	5.17	5.44	5.17	0	0	0	0	0	0	0	6.44	6.21	6.21	6.39	6.23	6.15	5.36
1386401_at	---	1	0	1	0	0	0	4.72	4.84	4.15	4.57	4.57	0	0	0	0	0	0	0	5.29	5.18	5.28	5.14	5.14	5.14	5.46
1386410_at	Cytip	1	0	0	0	1	0	6.09	5.70	5.70	6.45	5.94	0	0	0	0	0	0	0	4.09	4.12	4.25	4.17	4.18	4.10	3.69
1386479_at	---	1	0	0	1	0	0	3.66	3.94	3.82	4.18	3.78	0	0	0	0	0	0	0	4.13	4.09	3.99	4.09	3.90	4.17	3.96
1386529_at	---	1	1	0	0	0	1	4.72	4.19	4.42	4.93	4.10	0	0	0	0	0	0	0	3.88	3.90	4.04	3.63	3.76	3.81	3.56
1386550_at	Ikzf5	1	0	1	1	1	0	4.70	4.91	5.43	4.12	4.72	0	0	0	0	0	0	0	7.52	7.49	7.66	7.72	7.68	7.57	6.60
1386568_at	Irf6	1	0	0	1	1	0	7.51	7.33	7.27	8.04	7.37	0	0	0	0	0	0	0	7.03	7.11	7.19	7.16	7.23	7.20	7.14
1386621_at	---	1	0	1	0	1	1	4.92	4.85	5.54	4.72	5.58	0	0	0	0	0	0	0	6.29	6.27	6.38	6.53	6.51	6.26	5.62
1386633_at	Znf91	1	0	0	1	1	0	5.74	5.53	6.28	5.13	6.01	0	0	0	0	0	0	0	7.96	8.07	8.02	8.18	8.11	7.93	7.28
1386641_at	Hlf	1	1	1	1	0	1	4.69	5.17	5.62	5.28	5.22	0	0	0	0	0	0	0	8.10	7.98	8.04	8.07	8.08	8.16	8.20
1386648_at	Dnajc6	1	0	1	0	1	1	5.87	5.87	6.73	5.48	6.57	0	0	0	0	0	0	0	8.41	8.32	8.45	8.51	8.56	8.19	8.16
1386660_at	Gng2	1	0	0	0	1	0	4.65	4.59	4.98	4.20	4.87	0	0	0	0	0	0	0	6.74	6.75	6.87	6.81	6.73	6.59	6.57
1386683_at	Atrx	1	0	1	1	1	0	6.29	6.57	7.02	5.79	6.48	0	0	0	0	0	0	0	7.67	7.79	7.94	8.15	8.10	7.79	7.16
1386718_at	Akr1c19	1	0	1	1	1	0	8.01	8.02	8.72	7.52	8.00	0	0	0	0	0	0	0	8.44	8.60	8.67	8.72	8.75	9.02	8.97
1386733_at	Ccdc88a	1	0	1	0	1	1	3.78	3.96	4.78	3.63	4.41	0	0	0	0	0	0	0	8.04	8.02	8.29	8.43	8.38	8.47	8.19
1386744_x_at	---	1	0	1	0	1	0	5.49	5.34	4.79	5.49	5.20	0	0	0	0	0	0	0	4.78	4.67	4.82	4.71	4.88	4.94	4.18
1386745_at	---	1	0	1	0	1	0	6.13	6.12	6.70	5.70	5.96	0	0	0	0	0	0	0	8.32	8.35	8.45	8.53	8.52	8.34	7.92
1386755_at	Ccdc91 /// NEW	1	0	1	0	1	0	5.78	5.58	6.37	5.59	5.88	0	0	0	0	0	0	0	6.38	6.56	6.65	6.80	6.82	6.63	6.23
1386785_a_at	LOC100911498	1	1	1	1	0	1	8.19	8.75	8.75	9.12	8.84	0	0	0	0	0	0	0	3.41	3.33	3.53	3.36	3.40	3.36	2.87
1386786_at	LOC100911498	1	1	0	1	0	1	7.17	7.80	7.59	7.92	7.72	0	0	0	0	0	0	0	3.65	3.61	3.62	3.40	3.41	3.57	3.15
1386793_at	Zfp61	1	0	1	0	1	0	6.02	5.80	6.74	5.89	6.23	0	0	0	0	0	0	0	7.50	7.56	7.62	7.76	7.73	7.30	6.57
1386795_at	Hepacam2	1	0	1	0	1	0	6.88	7.16	7.52	6.80	7.18	0	0	0	0	0	0	0	11.39	11.48	11.45	11.49	11.58	11.38	10.28
1386832_a_at	Bcl6b	1	0	1	0	0	0	4.97	4.93	4.34	4.66	4.69	0	0	0	0	0	0	0	6.43	6.42	6.40	6.60	6.54	6.23	5.87
1386850_x_at	Tlk1	1	0	1	0	0	0	7.96	7.89	7.37	7.87	7.93	0	0	0	0	0	0	0	7.56	7.53	7.45	7.26	7.29	7.25	7.19
1386855_at	---	1	0	1	0	0	0	5.38	5.65	5.98	5.57	5.41	0	0	0	0	0	0	0	9.37	9.30	9.51	9.52	9.51	9.34	8.19
1386857_at	Stmn1	1	0	0	0	0	1	8.32	8.37	8.83	8.57	8.81	0	0	0	0	0	0	0	12.76	12.74	12.78	12.79	12.77	12.68	12.45
1386858_at	Rpl13	1	0	0	0	0	1	12.62	12.44	12.72	13.07	12.14	0	0	0	0	0	0	0	12.21	12.21	12.21	12.22	12.18	12.10	11.91
1386865_at	Sparcl1	1	0	0	1	1	0	8.54	8.34	8.94	7.83	8.47	0	0	0	0	0	0	0	3.94	3.82	3.91	3.68	3.70	3.79	3.17
1386866_at	Ywhag	1	0	0	0	0	1	10.09	10.21	10.46	10.08	10.60	0	0	0	0	0	0	0	11.96	11.96	12.02	12.05	12.08	12.09	12.01
1386868_at	Rps10	1	0	0	1	0	0	11.45	11.23	11.67	11.92	11.06	0	0	0	0	0	0	0	12.49	12.52	12.53	12.55	12.57	12.73	12.61
1386874_at	Rps15	1	0	0	1	0	0	12.54	12.67	12.65	13.05	12.13	0	0	0	0	0	0	0	12.49	12.49	12.45	12.48	12.42	12.43	12.24
1386885_at	Ech1	1	1	0	0	0	1	9.04	8.38	9.36	8.77	8.30	0	0	0	0	0	0	0	10.59	10.56	10.69	10.92	10.82	10.73	10.04
1386888_at	Eif4ebp1	1	0	1	0	1	1	9.09	8.68	8.29	8.99	8.18	0	0	0	0	0	0	0	9.80	9.76	9.73	9.72	9.76	9.55	9.30
1386892_at	Ptms	1	0	0	0	1	0	9.15	9.13	8.96	9.55	9.11	0	0	0	0	0	0	0	9.84	9.80	9.95	9.82	9.72	9.35	9.02

Probeset ID	Gene Symbol	Islet Data Set (log2 ratio ≥0.263; FDR <20%) union n=5325 1=YES 0=NO	Islet DRlyp/lyp ND vs ND+Lp299v (log2 ratio ≥0.263; FDR<20%) n=429 1=YES 0=NO	Islet DRlyp/lyp ND vs HCD (log2 ratio ≥0.263; FDR<20%) n=2247 1=YES 0=NO	Islet DRlyp/lyp ND vs HCD+Lp299v (log2 ratio ≥0.263; FDR<20%) n=1595 1=YES 0=NO	Islet DRlyp/lyp HCD vs HCD+Lp299v (log2 ratio ≥0.263; FDR<20%) n=3026 1=YES 0=NO	Islet DRlyp/lyp ND vs Flyp/lyp ND (log2 ratio ≥ 0.263; FDR<20%) n=1950 1=YES 0=NO	Islet Mean Log2 Intensity DRlyp/lyp ND	Islet Mean Log2 Intensity DRlyp/lyp ND+Lp299v	Islet Mean Log2 Intensity DRlyp/lyp HCD	Islet Mean Log2 Intensity DRlyp/lyp HCD+Lp299v	Islet Mean Log2 Intensity Flyp40 ND	Rinm5f data union of all conditions n=3705 1=YES 0=NO	Rinm5f 30 µM propionate/ 6 µM butyrate vs Media (FDR<20%; 1.5 fold) n=3 1=YES 0=NO	Rinm5f 60 µM propionate/ 12 µM butyrate vs Media (FDR<20%; 1.5 fold) n=18 1=YES 0=NO	Rinm5f 120 µM propionate/ 24 µM butyrate vs Media (FDR<20%; 1.5 fold) n=164 1=YES 0=NO	Rinm5f 240 µM propionate /48 µM butyrate vs Media (FDR<20%; 1.5 fold) n=268 1=YES 0=NO	Rinm5f 1500 µM propionate/ 300 µM butyrate vs Media (FDR<20%; 1.5 fold) n=2491 1=YES 0=NO	Rinm5f 500 µM propionate/ 500 µM butyrate vs Media (FDR<20%; 1.5 fold) n=3001 1=YES 0=NO	Rinm5f Mean Log2 Intensity Media only	Rinm5f Mean Log2 Intensity 30 µM propionate/ 6 µM butyrate	Rinm5f Mean Log2 Intensity 60 µM propionate/ 12 µM butyrate	Rinm5f Mean Log2 Intensity 120 µM propionate/ 24 µM butyrate	Rinm5f Mean Log2 Intensity 240 µM propionate/ 48 µM butyrate	Rinm5f Mean Log2 Intensity 1500 µM propionate /300 µM butyrate	Rinm5f Mean Log2 Intensity 500 µM propionate/ 500 µM butyrate
1386896_at	Khdrbs1	1	0	1	0	1	0	6.94	7.00	7.57	6.85	7.05	0	0	0	0	0	0	0	9.61	9.61	9.66	9.63	9.57	9.45	9.02
1386900_at	Serp1	1	0	0	1	0	0	11.26	11.06	11.05	10.61	10.96	0	0	0	0	0	0	0	11.82	11.78	11.82	11.83	11.81	11.67	10.93
1386905_at	Prkar1a	1	0	1	1	1	0	8.92	8.94	9.59	8.31	8.98	0	0	0	0	0	0	0	11.85	11.85	11.85	11.85	11.77	11.76	11.41
1386909_a_at	Vdac1	1	0	0	1	1	0	8.76	8.41	9.08	7.94	8.33	0	0	0	0	0	0	0	11.35	11.47	11.39	11.32	11.39	11.53	10.82
1386911_at	Atp1a2	1	0	0	1	1	0	5.00	4.76	5.21	4.46	4.70	0	0	0	0	0	0	0	5.72	5.78	5.79	5.90	5.93	6.19	6.40
1386913_at	Pdpn	1	0	1	0	0	1	5.24	5.22	5.83	5.20	6.05	0	0	0	0	0	0	0	4.19	4.11	4.09	3.83	4.05	3.99	3.86
1386928_at	Bcat2	1	0	1	0	1	1	9.91	9.47	8.71	9.53	8.96	0	0	0	0	0	0	0	7.36	7.43	7.53	7.71	7.73	7.77	7.51
1386929_at	Hk1	1	0	1	0	1	1	6.85	6.65	6.13	7.12	6.33	0	0	0	0	0	0	0	7.81	7.74	7.88	7.68	7.59	7.71	7.47
1386935_at	Nr4a1	1	0	0	1	1	0	7.36	7.54	7.52	6.71	6.92	0	0	0	0	0	0	0	7.96	7.85	7.86	7.68	7.54	7.68	7.71
1386940_at	Timp2	1	0	1	0	0	0	8.07	8.12	7.53	8.04	8.47	0	0	0	0	0	0	0	10.44	10.53	10.51	10.44	10.49	10.60	10.43
1386941_at	Plec	1	0	0	0	1	0	6.36	6.46	5.86	6.70	6.24	0	0	0	0	0	0	0	7.83	7.83	7.80	7.66	7.77	7.83	7.99
1386949_a_at	Mta1	1	0	0	1	0	0	6.23	6.00	6.29	6.71	6.23	0	0	0	0	0	0	0	8.23	8.24	8.31	8.30	8.21	8.31	7.76
1386952_a_at	Dync1i2	1	0	1	0	1	0	7.96	7.89	8.61	7.86	8.29	0	0	0	0	0	0	0	9.55	9.65	9.63	9.73	9.65	9.44	9.14
1386958_at	Txnrd1	1	0	0	1	1	0	6.43	6.38	6.96	5.87	6.40	0	0	0	0	0	0	0	10.03	10.05	10.14	10.24	10.13	10.18	9.59
1386961_at	Pfkm	1	0	0	1	1	0	6.48	6.17	6.79	5.59	6.23	0	0	0	0	0	0	0	9.53	9.73	9.74	9.81	9.75	9.99	8.96
1386962_at	Plcb4	1	0	1	0	1	0	6.79	6.99	7.40	6.69	7.12	0	0	0	0	0	0	0	8.45	8.47	8.55	8.61	8.66	8.91	9.32
1386964_at	Bpifa2f	1	0	0	0	1	1	6.24	6.56	6.78	5.98	6.84	0	0	0	0	0	0	0	6.10	6.10	6.00	6.19	6.19	5.77	4.80
1386987_at	Il6r	1	0	0	0	1	0	7.13	6.71	7.46	6.70	6.72	0	0	0	0	0	0	0	7.46	7.61	7.65	7.66	7.72	7.98	7.60
1386988_at	Deaf1	1	0	0	0	1	0	6.83	6.81	6.55	7.14	6.53	0	0	0	0	0	0	0	8.52	8.47	8.55	8.53	8.58	8.54	8.42
1386999_at	Ywhab	1	0	0	0	0	1	8.08	8.27	8.54	8.07	8.61	0	0	0	0	0	0	0	10.89	10.80	10.81	10.82	10.74	10.75	10.44
1387013_at	Cltrn	1	0	1	0	1	0	10.67	10.86	11.50	10.40	11.05	0	0	0	0	0	0	0	4.73	4.59	4.63	4.95	4.89	4.55	5.49
1387015_at	Pfn2	1	0	1	0	1	0	7.54	7.71	8.43	7.09	7.95	0	0	0	0	0	0	0	11.10	11.23	11.21	11.24	11.25	11.29	10.98
1387016_a_at	Nptn	1	0	1	0	1	0	10.22	10.21	10.93	9.99	10.52	0	0	0	0	0	0	0	11.28	11.33	11.33	11.43	11.49	11.75	11.46
1387017_at	Sqle	1	0	0	0	0	1	6.57	6.55	6.93	6.48	7.27	0	0	0	0	0	0	0	9.97	10.00	10.10	10.17	10.32	10.20	10.05
1387024_at	Dusp6	1	1	1	1	0	0	7.58	6.91	8.90	8.31	7.21	0	0	0	0	0	0	0	10.27	10.46	10.36	10.40	10.68	10.73	10.16
1387028_a_at	Id1	1	0	0	1	0	1	7.07	6.80	6.78	6.36	6.53	0	0	0	0	0	0	0	10.06	10.11	10.01	9.54	9.69	9.80	10.72
1387041_at	Ubqln1	1	0	1	0	1	0	8.17	8.20	8.78	7.99	8.47	0	0	0	0	0	0	0	10.08	10.05	10.10	10.16	10.05	9.83	9.40
1387044_at	Gpha2	1	0	1	0	0	1	6.46	6.53	5.65	6.09	5.98	0	0	0	0	0	0	0	6.10	6.13	6.07	5.92	5.99	6.17	5.62
1387052_at	Gpt	1	0	1	0	1	1	7.23	7.01	6.54	7.61	6.56	0	0	0	0	0	0	0	6.87	6.82	6.97	7.06	6.98	6.86	6.17
1387055_at	Nae1	1	0	0	0	1	0	6.55	6.38	6.90	6.18	6.67	0	0	0	0	0	0	0	8.71	8.71	8.81	8.90	8.84	8.82	8.53
1387063_at	Ip6k2	1	1	0	0	0	0	6.91	6.45	7.04	6.62	7.27	0	0	0	0	0	0	0	7.80	7.89	7.93	8.03	8.02	7.94	7.48
1387073_at	Snap25	1	1	1	0	1	1	9.42	9.90	10.46	8.96	10.65	0	0	0	0	0	0	0	11.75	11.76	11.87	11.97	12.05	12.31	11.98
1387076_at	Hif1a	1	0	1	0	1	0	9.07	9.11	9.74	8.83	9.46	0	0	0	0	0	0	0	10.44	10.35	10.47	10.60	10.49	10.61	10.65
1387085_at	Prps1	1	0	1	1	1	0	7.39	7.41	8.07	6.63	7.69	0	0	0	0	0	0	0	10.76	10.78	10.86	11.03	10.93	11.06	10.42
1387087_at	Cebpb	1	0	1	0	0	0	7.47	7.44	6.93	7.45	7.20	0	0	0	0	0	0	0	10.09	10.13	10.19	10.26	10.28	9.93	9.58
1387095_at	Gnaz	1	0	1	1	1	0	6.20	6.07	6.77	5.43	6.42	0	0	0	0	0	0	0	7.75	7.70	7.80	7.84	7.76	7.72	7.05
1387097_at	Fut2	1	0	0	0	1	0	5.67	5.87	5.50	6.11	5.87	0	0	0	0	0	0	0	5.67	5.62	5.56	5.38	5.50	5.85	5.51
1387111_at	Ddah1	1	0	1	1	1	1	9.18	8.80	8.54	9.77	8.71	0	0	0	0	0	0	0	7.71	7.76	7.77	7.76	7.79	7.83	8.18
1387113_at	Ctbp2	1	0	0	1	1	0	9.00	9.08	8.90	9.56	8.74	0	0	0	0	0	0	0	9.42	9.42	9.49	9.49	9.42	9.40	9.19
1387116_at	Dnajb9	1	0	0	0	0	1	9.42	9.58	9.74	9.42	10.29	0	0	0	0	0	0	0	9.77	9.90	9.83	9.96	9.93	9.96	9.27
1387121_a_at	Ndrg2	1	0	0	1	0	1	6.85	6.44	6.47	6.19	5.84	0	0	0	0	0	0	0	9.04	9.11	9.07	8.95	8.93	9.21	9.18
1387126_at	Atp2c1	1	0	1	0	1	0	6.41	6.57	7.09	6.10	6.73	0	0	0	0	0	0	0	7.14	7.15	7.15	7.27	7.23	7.28	7.14
1387132_at	Lipe	1	0	1	0	0	0	6.14	5.97	5.61	5.89	5.82	0	0	0	0	0	0	0	6.58	6.71	6.68	6.68	6.51	6.64	6.45
1387145_at	Gjb1	1	0	1	0	1	1	8.09	7.84	7.24	8.39	7.49	0	0	0	0	0	0	0	5.39	5.34	5.30	5.18	5.10	5.21	4.83
1387146_a_at	Ednrb	1	0	0	1	1	0	6.78	6.46	7.14	6.10	6.77	0	0	0	0	0	0	0	4.30	4.28	4.11	3.99	4.14	4.21	3.82
1387147_at	Rab3c	1	0	0	0	0	1	4.71	4.68	4.97	4.47	5.22	0	0	0	0	0	0	0	8.98	8.97	9.07	9.17	9.04	8.98	8.89
1387148_at	Gprasp1	1	0	0	0	1	0	8.89	8.93	9.46	8.44	8.87	0	0	0	0	0	0	0	9.65	9.68	9.78	9.91	9.85	9.73	9.75
1387154_at	Npy	1	0	1	0	1	1	8.33	8.45	9.27	8.41	10.27	0	0	0	0	0	0	0	11.32	11.36	11.34	11.33	11.34	11.47	10.71
1387155_at	Pcsk2	1	0	0	0	1	0	11.31	11.30	11.76	10.91	11.65	0	0	0	0	0	0	0	11.45	11.46	11.44	11.38	11.48	11.59	10.95
1387170_at	Csnk2a1	1	0	0	1	1	0	6.95	6.93	7.36	6.31	6.90	0	0	0	0	0	0	0	9.74	9.71	9.77	9.78	9.78	9.82	9.24
1387178_a_at	Cbs /// Cbsl	1	0	1	0	1	1	9.47	9.10	8.82	9.73	8.82	0	0	0	0	0	0	0	4.20	4.15	3.96	3.99	3.98	4.10	3.91
1387183_at	Crot	1	0	0	1	1	0	6.27	5.85	6.50	5.57	6.15	0	0	0	0	0	0	0	6.19	6.17	6.30	6.15	6.24	6.75	6.46
1387184_at	Axin2	1	0	0	0	1	0	5.32	5.33	5.10	5.67	5.38	0	0	0	0	0	0	0	6.14	6.12	6.27	6.33	6.29	6.59	6.76
1387195_at	St14	1	0	0	1	1	0	7.22	7.27	6.93	7.91	7.02	0	0	0	0	0	0	0	9.96	9.96	9.97	9.88	9.87	9.88	9.90
1387196_at	Khdrbs3	1	0	0	0	0	1	8.21	8.49	8.73	8.14	8.69	0	0	0	0	0	0	0	11.04	11.02	11.04	11.06	11.02	11.07	11.16
1387206_at	B4galt6	1	0	1	0	0	0	8.46	8.48	9.07	8.52	8.54	0	0	0	0	0	0	0	10.01	10.08	10.07	10.05	10.01	9.99	9.74
1387221_at	Gch1	1	0	1	1	1	1	8.22	8.51	9.12	7.55	8.75	0	0	0	0	0	0	0	11.39	11.49	11.49	11.56	11.64	11.53	10.84
1387226_at	Ina	1	0	0	0	0	1	5.91	6.22	6.24	6.01	6.42	0	0	0	0	0	0	0	8.70	8.64	8.73	8.68	8.55	8.52	9.03
1387234_at	Azgp1	1	0	1	0	0	1	5.72	5.51	5.03	5.35	5.20	0	0	0	0	0	0	0	3.90	3.95	3.89	3.55	3.83	3.88	3.53
1387235_at	Chga	1	0	0	0	1	0	10.34	10.40	10.87	10.09	10.57	0	0	0	0	0	0	0	13.43	13.53	13.52	13.56	13.58	13.61	13.30
1387243_at	Cyp1a2	1	0	0	0	1	0	4.87	5.15	4.48	5.15	4.80	0	0	0	0	0	0	0	4.87	5.08	4.69	4.57	4.69	4.81	4.42
1387244_at	Cgrrf1	1	0	0	0	1	0	7.61	7.51	7.20	7.85	7.32	0	0	0	0	0	0	0	8.60	8.69	8.62	8.54	8.60	8.73	8.56
1387254_at	Ghrl	1	0	0	0	0	1	6.92	7.17	7.24	7.05	6.11	0	0	0	0	0	0	0	4.48	4.44	4.42	4.29	4.43	4.39	4.01
1387258_a_at	Pcmt1	1	0	0	0	1	0	7.45	7.40	7.95	7.05	7.45	0	0	0	0	0	0	0	10.57	10.62	10.62	10.64	10.60	10.66	10.43
1387259_at	Cdh2	1	0	1	0	1	0	5.98	6.04	6.77	5.68	6.32	0	0	0	0	0	0	0	9.89	9.74	9.83	9.91	9.88	9.79	9.55
1387261_at	Ppp3cb	1	0	1	0	0	0	6.52	6.50	7.22	6.56	6.45	0	0	0	0	0	0	0	8.40	8.55	8.58	8.61	8.57	8.53	7.85
1387266_at	Siah1	1	0	1	0	1	0	6.05	5.97	6.70	5.97	6.32	0	0	0	0	0	0	0	7.66	7.64	7.80	7.76	7.67	7.47	7.20
1387288_at	Neurod1	1	0	1	0	1	0	11.02	11.40	11.82	10.81	11.28	0	0	0	0	0	0	0	12.15	12.15	12.29	12.33	12.32	12.44	12.14
1387294_at	Sh3bp5	1	0	0	0	1	0	6.57	6.46	7.07	6.22	6.29	0	0	0	0	0	0	0	7.68	7.80	7.99	8.02	8.00	8.05	7.82
1387300_at	Crnkl1	1	0	0	0	1	0	5.71	5.73	6.25	5.54	5.83	0	0	0	0	0	0	0	8.77	8.78	8.81	8.80	8.79	8.67	8.50
1387316_at	Cxcl2	1	1	0	0	0	0	4.67	6.11	4.45	4.23	4.41	0	0	0	0	0	0	0	4.41	4.47	4.50	4.37	4.33	4.39	3.80
1387319_at	Ccl11	1	0	0	0	0	1	3.99	3.78	3.94	3.54	3.46	0	0	0	0	0	0	0	3.77	3.66	3.72	3.54	3.49	3.68	3.19
1387323_at	Klkb1	1	0	0	0	0	1	3.03	3.13	3.01	3.18	4.08	0	0	0	0	0	0	0	3.76	3.67	3.54	3.53	3.58	3.72	3.21
1387335_s_at	Abo	1	1	0	0	0	0	4.43	3.96	3.99	4.27	4.00	0	0	0	0	0	0	0	4.96	5.17	5.13	4.94	4.91	4.84	4.80
1387342_at	Gng5	1	0	0	1	0	0	9.99	9.83	10.19	10.48	9.97	0	0	0	0	0	0	0	10.89	10.87	10.84	10.90	10.88	11.02	10.80
1387344_at	Aldh6a1	1	1	0	0	0	1	9.57	9.11	9.32	9.20	9.10	0	0	0	0	0	0	0	8.31	8.29	8.31	8.49	8.46	8.54	8.52
1387346_at	Itgb1	1	0	0	1	1	0	8.17	8.14	8.68	7.46	8.06	0	0	0	0	0	0	0	9.46	9.52	9.52	9.52	9.46	9.31	8.64
1387351_at	Fbn1	1	0	0	0	0	1	5.89	5.64	5.78	5.60	5.42	0	0	0	0	0	0	0	3.89	3.84	3.88	3.80	3.89	3.96	3.64
1387353_at	Akt2	1	0	0	1	1	0	5.81	5.41	5.93	5.02	5.38	0	0	0	0	0	0	0	7.51	7.52	7.63	7.63	7.55	7.32	6.56
1387354_at	Stat1	1	0	0	0	1	0	6.93	7.38	7.46	6.46	7.05	0	0	0	0	0	0	0	8.76	8.79	8.88	8.95	8.90	9.04	8.58
1387358_at	Arl1	1	0	0	1	1	0	9.10	8.93	9.32	8.37	9.17	0	0	0	0	0	0	0	10.59	10.65	10.60	10.63	10.59	10.36	9.75
1387365_at	Nr1h3	1	0	1	0	1	1	5.10	5.08	4.50	5.49	4.39	0	0	0	0	0	0	0	3.82	3.79	3.76	3.73	3.83	3.79	3.30
1387366_at	Ilf3	1	0	0	0	0	1	5.26	5.04	5.61	5.39	6.57	0	0	0	0	0	0	0	8.69	8.64	8.83	8.86	8.74	8.50	7.90
1387370_at	Tmod1	1	0	1	0	1	0	5.33	5.55	6.39	5.32	5.50	0	0	0	0	0	0	0	5.44	5.36	5.29	5.21	5.34	5.10	4.41
1387374_at	Tcf12	1	0	0	1	1	0	5.76	5.86	5.65	6.30	5.65	0	0	0	0	0	0	0	5.64	5.49	5.50	5.70	5.59	5.22	5.05
1387388_at	Chp1 /// RGD15	1	0	0	1	1	1	7.14	6.87	7.48	6.02	6.62	0	0	0	0	0	0	0	9.24	9.21	9.31	9.41	9.42	9.67	8.71
1387398_at	Pkia	1	0	0	0	1	0	5.39	5.53	5.89	4.99	5.37	0	0	0	0	0	0	0	8.57	8.54	8.62	8.72	8.68	8.83	8.49
1387399_at	Pclo	1	1	1	0	0	1	3.97	4.45	4.46	4.18	4.72	0	0	0	0	0	0	0	4.06	3.90	4.13	4.04	3.96	4.10	3.98
1387405_at	Ralgapa1	1	0	0	0	1	0	5.70	5.73	6.14	5.23	6.01	0	0	0	0	0	0	0	7.58	7.58	7.61	7.57	7.58	7.38	7.26
1387407_at	Nap1l3	1	0	1	0	1	1	5.72	5.82	6.70	5.60	6.39	0	0	0	0	0	0	0	8.50	8.58	8.55	8.59	8.59	8.27	7.62
1387410_at	Nr4a2	1	1	0	0	0	0	5.08	5.60	5.24	5.22	4.65	0	0	0	0	0	0	0	4.14	4.12	4.19	4.05	4.08	4.09	3.89
1387420_at	Clic4	1	0	0	1	1	0	5.85	5.75	5.86	5.16	5.59	0	0	0	0	0	0	0	8.32	8.32	8.50	8.62	8.61	8.88	7.42
1387430_at	Hsf2	1	0	1	0	1	0	6.19	6.21	6.77	5.88	6.32	0	0	0	0	0	0	0	8.87	8.88	8.97	9.02	9.02	9.11	8.74
1387436_at	Septin7	1	0	1	0	1	0	7.32	7.55	8.07	6.85	7.34	0	0	0	0	0	0	0	10.10	10.13	10.19	10.28	10.20	10.12	9.70
1387437_at	Fbxo2	1	0	0	0	0	1	4.09	4.31	4.65	4.16	4.78	0	0	0	0	0	0	0	10.33	10.45	10.49	10.49	10.53	10.66	9.89
1387440_at	Ireb2	1	0	0	1	1	0	5.33	5.46	5.85	4.69	5.41	0	0	0	0	0	0	0	6.90	6.93	6.98	7.20	7.01	6.90	6.42
1387447_at	Arf3 /// Fkbp11	1	0	1	1	1	0	6.11	6.19	6.93	5.39	6.15	0	0	0	0	0	0	0	9.75	9.74	9.75	9.86	9.74	9.73	8.70
1387452_a_at	Nfyb	1	0	0	0	1	0	4.69	4.60	5.01	4.25	4.48	0	0	0	0	0	0	0	8.19	8.29	8.31	8.43	8.52	8.68	7.88
1387458_at	Rnf4	1	0	0	1	1	0	7.64	7.40	8.04	6.65	7.36	0	0	0	0	0	0	0	10.62	10.59	10.63	10.75	10.71	10.63	9.87
1387459_at	Pkib	1	0	0	1	0	1	5.83	5.75	6.02	6.32	4.47	0	0	0	0	0	0	0	9.98	10.11	10.01	9.98	10.05	10.01	9.16
1387499_a_at	Pdcl	1	0	1	0	1	0	6.50	6.61	7.22	6.40	6.76	0	0	0	0	0	0	0	8.28	8.33	8.42	8.53	8.53	8.78	8.48
1387502_at	Stk17b	1	0	0	1	1	0	4.78	4.84	4.85	4.14	5.09	0	0	0	0	0	0	0	7.26	7.33	7.39	7.46	7.41	7.03	6.78
1387505_at	Gnai1	1	0	1	1	1	0	4.94	5.00	5.78	4.12	5.06	0	0	0	0	0	0	0	10.19	10.29	10.26	10.24	10.31	10.50	9.18
1387508_at	Baat	1	0	0	0	0	1	3.59	3.40	3.43	3.85	3.11	0	0	0	0	0	0	0	4.13	3.98	4.01	3.76	4.01	4.01	4.02
1387510_at	Snx16	1	0	0	0	1	0	5.90	5.77	6.32	5.49	5.78	0	0	0	0	0	0	0	8.06	8.19	8.25	8.34	8.31	8.14	7.90
1387512_at	Zbtb18	1	0	0	1	0	0	6.11	6.05	6.20	5.61	5.91	0	0	0	0	0	0	0	5.92	5.85	6.03	5.83	5.84	6.14	5.79
1387514_at	Cbwd1	1	0	0	1	0	0	4.80	4.35	4.82	4.17	4.60	0	0	0	0	0	0	0	8.64	8.71	8.69	8.70	8.69	8.59	8.11
1387521_at	Pdcd4	1	0	0	1	1	1	9.69	9.41	9.57	8.86	9.15	0	0	0	0	0	0	0	9.28	9.29	9.40	9.55	9.54	9.72	8.91
1387529_a_at	Tagln3	1	0	0	0	0	1	5.95	5.97	5.86	5.74	6.86	0	0	0	0	0	0	0	10.02	10.07	10.16	10.35	10.22	9.89	9.26
1387545_at	Mtr	1	0	1	0	1	0	4.83	4.75	4.25	4.92	4.67	0	0	0	0	0	0	0	5.45	5.25	5.24	5.17	5.08	5.12	4.78
1387554_at	Galnt5	1	0	0	0	0	1	6.54	6.54	6.41	6.55	5.23	0	0	0	0	0	0	0	5.70	5.90	5.72	5.67	5.70	6.07	6.14
1387573_a_at	Nr5a2	1	1	1	0	0	1	7.78	7.30	7.20	7.68	7.04	0	0	0	0	0	0	0	3.72	3.80	3.82	3.69	3.62	3.82	3.20
1387599_a_at	Nqo1	1	0	0	1	0	0	6.24	6.27	6.19	5.73	6.58	0	0	0	0	0	0	0	5.72	5.60	5.74	5.58	5.47	5.52	6.09
1387629_at	Bspry	1	0	0	0	0	1	7.36	7.16	6.96	7.04	6.85	0	0	0	0	0	0	0	7.34	7.34	7.39	7.46	7.41	7.58	7.11
1387630_at	Elovl5	1	0	0	1	0	0	6.18	6.16	6.18	5.56	5.99	0	0	0	0	0	0	0	7.60	7.58	7.64	7.78	7.77	7.81	6.77
1387641_at	Rab5a	1	0	1	1	1	0	6.80	6.95	7.61	5.51	6.79	0	0	0	0	0	0	0	9.41	9.49	9.49	9.54	9.49	9.33	8.34
1387651_at	Aqp1	1	0	1	1	0	1	6.46	6.09	5.52	5.60	5.68	0	0	0	0	0	0	0	5.97	5.97	5.92	5.64	5.68	5.89	5.79
1387660_at	Iapp	1	0	0	0	1	0	13.60	13.76	14.16	13.30	13.99	0	0	0	0	0	0	0	13.44	13.50	13.41	13.38	13.52	13.31	12.37
1387664_at	Atp6v1b2	1	0	1	1	1	0	8.74	8.75	9.42	7.63	8.75	0	0	0	0	0	0	0	11.44	11.45	11.48	11.51	11.44	11.46	10.77
1387671_at	Sctr	1	0	1	0	1	1	8.82	8.38	8.11	9.09	8.05	0	0	0	0	0	0	0	4.14	4.12	4.06	4.03	4.04	4.06	3.80
1387673_a_at	Anxa6	1	0	0	0	1	0	7.71	7.82	7.45	8.09	7.35	0	0	0	0	0	0	0	7.82	7.76	7.85	7.84	7.90	8.14	8.12
1387690_at	Casp3	1	0	0	1	1	1	6.72	6.54	6.91	5.60	6.23	0	0	0	0	0	0	0	9.33	9.33	9.38	9.45	9.36	9.31	8.53
1387700_at	Cckar	1	1	0	1	1	1	8.27	7.73	8.26	7.44	7.44	0	0	0	0	0	0	0	4.29	4.29	4.40	4.36	4.43	4.49	4.14
1387716_at	Utrn	1	1	0	0	0	0	4.89	5.48	5.13	4.70	5.15	0	0	0	0	0	0	0	5.63	5.75	5.80	5.81	5.74	6.16	5.41
1387737_at	Mat2a	1	0	0	1	1	0	6.66	6.59	7.14	5.69	6.44	0	0	0	0	0	0	0	9.52	9.50	9.58	9.80	9.80	10.08	9.13
1387749_at	Cd79b	1	0	0	0	0	1	4.75	4.80	4.48	4.64	5.38	0	0	0	0	0	0	0	4.65	4.75	4.80	4.59	4.59	4.64	4.46
1387755_s_at	Tgoln1	1	0	1	1	1	0	6.75	6.77	7.41	5.79	6.65	0	0	0	0	0	0	0	8.79	8.69	8.74	8.99	8.89	9.02	8.04
1387757_at	Lmbrd1	1	0	1	1	1	0	7.45	7.63	8.15	6.84	7.64	0	0	0	0	0	0	0	9.63	9.64	9.67	9.72	9.74	9.68	9.08
1387759_s_at	Ugt1a6	1	0	0	1	1	0	7.13	7.03	7.38	6.54	7.21	0	0	0	0	0	0	0	9.31	9.38	9.44	9.54	9.68	9.77	9.04
1387760_a_at	Onecut1	1	0	0	0	1	0	6.00	6.03	5.68	6.38	6.01	0	0	0	0	0	0	0	4.20	4.34	4.25	4.21	4.27	4.60	4.45
1387772_at	Calm1	1	0	0	0	0	1	9.97	10.03	10.47	9.88	10.53	0	0	0	0	0	0	0	11.50	11.53	11.61	11.69	11.73	11.76	11.88
1387774_at	Ywhaz	1	0	1	1	1	0	9.64	9.66	10.25	9.17	9.94	0	0	0	0	0	0	0	13.04	13.05	13.10	13.12	13.06	12.92	12.43
1387780_at	Dnaja2	1	0	0	0	1	0	8.93	8.74	9.48	8.73	8.99	0	0	0	0	0	0	0	10.72	10.68	10.74	10.77	10.77	10.74	10.39
1387783_a_at	Acaa1 /// Acaa1	1	0	0	0	0	1	8.83	8.49	8.45	8.58	8.15	0	0	0	0	0	0	0	8.14	8.19	8.23	8.26	8.20	8.39	8.39
1387785_at	Mtpn	1	0	1	1	1	0	6.85	6.96	7.50	6.08	6.96	0	0	0	0	0	0	0	9.85	9.81	9.88	9.93	9.85	9.97	8.95

Probeset ID	Gene Symbol	Islet Data Set (log2 ratio ≥0.263; FDR <20%) union n=5325 1=YES 0=NO	Islet DRlyp/lyp ND vs ND+Lp299v (log2 ratio ≥0.263; FDR<20%) n=429 1=YES 0=NO	Islet DRlyp/lyp ND vs HCD (log2 ratio ≥0.263; FDR<20%) n=2247 1=YES 0=NO	Islet DRlyp/lyp ND vs HCD+Lp299v (log2 ratio ≥0.263; FDR<20%) n=1595 1=YES 0=NO	Islet DRlyp/lyp HCD vs HCD+Lp299v (log2 ratio ≥0.263; FDR<20%) n=3026 1=YES 0=NO	Islet DRlyp/lyp ND vs Flyp/lyp ND (log2 ratio ≥ 0.263; FDR<20%) n=1950 1=YES 0=NO	Islet Mean Log2 Intensity DRlyp/lyp ND	Islet Mean Log2 Intensity DRlyp/lyp ND+Lp299v	Islet Mean Log2 Intensity DRlyp/lyp HCD	Islet Mean Log2 Intensity DRlyp/lyp HCD+Lp299v	Islet Mean Log2 Intensity Flyp40 ND	Rinm5f data union of all conditions n=3705 1=YES 0=NO	Rinm5f 30 µM propionate/ 6 µM butyrate vs Media (FDR<20%; 1.5 fold) n=3 1=YES 0=NO	Rinm5f 60 µM propionate/ 12 µM butyrate vs Media (FDR<20%; 1.5 fold) n=18 1=YES 0=NO	Rinm5f 120 µM propionate/ 24 µM butyrate vs Media (FDR<20%; 1.5 fold) n=164 1=YES 0=NO	Rinm5f 240 µM propionate /48 µM butyrate vs Media (FDR<20%; 1.5 fold) n=268 1=YES 0=NO	Rinm5f 1500 µM propionate/ 300 µM butyrate vs Media (FDR<20%; 1.5 fold) n=2491 1=YES 0=NO	Rinm5f 500 µM propionate/ 500 µM butyrate vs Media (FDR<20%; 1.5 fold) n=3001 1=YES 0=NO	Rinm5f Mean Log2 Intensity Media only	Rinm5f Mean Log2 Intensity 30 µM propionate/ 6 µM butyrate	Rinm5f Mean Log2 Intensity 60 µM propionate/ 12 µM butyrate	Rinm5f Mean Log2 Intensity 120 µM propionate/ 24 µM butyrate	Rinm5f Mean Log2 Intensity 240 µM propionate/ 48 µM butyrate	Rinm5f Mean Log2 Intensity 1500 µM propionate /300 µM butyrate	Rinm5f Mean Log2 Intensity 500 µM propionate/ 500 µM butyrate
1387786_at	Mtpn	1	0	1	1	1	0	7.66	7.71	8.29	7.18	7.87	0	0	0	0	0	0	0	9.87	9.88	9.96	10.02	9.93	9.99	9.27
1387788_at	Junb	1	0	0	1	1	1	7.81	8.17	7.76	6.99	7.28	0	0	0	0	0	0	0	8.39	8.36	8.41	7.93	8.08	8.30	7.55
1387805_at	Bnip3	1	0	0	0	1	0	9.29	9.30	9.82	8.97	9.41	0	0	0	0	0	0	0	11.88	11.95	11.77	11.52	11.58	11.45	11.46
1387806_at	Rap1b	1	0	0	0	1	0	9.14	9.02	9.66	8.82	9.33	0	0	0	0	0	0	0	11.45	11.47	11.55	11.70	11.72	11.94	11.85
1387812_at	Pcsk6	1	0	0	1	0	0	4.14	4.32	4.51	4.63	4.25	0	0	0	0	0	0	0	9.16	9.08	9.20	9.10	9.10	9.13	8.81
1387813_at	Erbb2	1	0	0	0	0	1	4.95	4.70	4.48	4.67	4.41	0	0	0	0	0	0	0	4.58	4.56	4.45	4.28	4.48	4.31	4.25
1387817_at	Nsg1	1	0	0	0	0	1	7.48	7.57	7.71	7.10	8.00	0	0	0	0	0	0	0	12.28	12.35	12.35	12.25	12.26	12.17	11.70
1387822_at	Gna11	1	0	0	0	1	0	7.00	7.14	6.75	7.35	6.67	0	0	0	0	0	0	0	8.15	8.01	8.16	8.10	8.00	8.06	7.89
1387830_at	Crp	1	0	1	0	1	0	5.56	5.58	6.16	5.11	5.46	0	0	0	0	0	0	0	6.28	5.98	6.27	6.09	6.17	6.21	4.84
1387833_at	Tmprss2	1	0	1	0	0	0	6.08	5.93	5.54	5.84	5.75	0	0	0	0	0	0	0	6.28	6.23	6.39	6.26	6.42	6.47	6.11
1387840_at	Acp1	1	0	0	1	1	0	6.50	6.43	7.04	5.89	6.52	0	0	0	0	0	0	0	10.33	10.37	10.38	10.45	10.40	10.18	9.41
1387841_at	Exoc5	1	0	1	1	1	0	5.50	5.56	6.07	4.64	5.33	0	0	0	0	0	0	0	8.00	7.95	8.04	8.19	8.07	7.89	7.21
1387848_at	Hmgcr	1	0	1	1	1	0	5.88	5.95	6.52	5.15	5.74	0	0	0	0	0	0	0	8.38	8.32	8.47	8.50	8.51	7.98	7.38
1387851_at	Pter	1	0	0	0	0	1	5.56	5.49	5.04	5.45	4.91	0	0	0	0	0	0	0	6.06	5.89	5.99	5.84	5.71	5.84	5.91
1387854_at	NEWGENE_6213	1	1	0	1	1	1	7.11	6.49	7.08	6.06	6.34	0	0	0	0	0	0	0	4.66	4.90	4.96	5.14	5.20	5.18	5.22
1387856_at	Cnn3	1	0	1	0	1	0	8.56	8.42	9.24	8.12	8.30	0	0	0	0	0	0	0	10.36	10.38	10.45	10.53	10.52	10.75	10.72
1387864_at	Kidins220	1	0	1	0	0	0	8.97	9.11	9.58	9.17	9.36	0	0	0	0	0	0	0	10.24	10.21	10.37	10.36	10.37	10.24	10.30
1387872_at	Hnrnpa1	1	0	1	0	1	0	8.78	8.59	9.48	8.69	8.67	0	0	0	0	0	0	0	12.34	12.30	12.34	12.33	12.27	12.06	11.58
1387874_at	Dbp	1	1	0	1	1	1	6.79	6.33	6.91	6.16	5.39	0	0	0	0	0	0	0	10.35	10.42	10.40	10.33	10.33	9.98	9.48
1387878_at	Glud1	1	0	0	1	0	1	10.53	10.19	10.17	9.92	10.04	0	0	0	0	0	0	0	11.41	11.39	11.42	11.41	11.37	11.37	11.10
1387879_a_at	Celf2	1	0	0	0	1	0	4.57	4.58	5.06	4.22	4.79	0	0	0	0	0	0	0	6.59	6.57	6.66	6.75	6.53	6.25	5.35
1387882_at	Klf9	1	1	0	0	0	1	6.79	6.32	6.80	6.45	6.05	0	0	0	0	0	0	0	7.41	7.42	7.49	7.42	7.39	7.90	7.50
1387893_at	C1s	1	0	1	1	0	0	6.12	5.73	5.43	4.86	6.20	0	0	0	0	0	0	0	5.77	5.72	5.87	5.88	5.84	5.74	5.14
1387894_at	Gata4	1	0	1	1	1	1	6.75	6.47	5.80	7.26	6.20	0	0	0	0	0	0	0	6.56	6.61	6.76	6.61	6.68	6.87	7.04
1387897_at	Cnp	1	0	0	0	1	1	8.21	8.23	7.89	8.65	7.70	0	0	0	0	0	0	0	9.19	9.27	9.25	9.27	9.27	9.27	9.20
1387899_at	Crmp1	1	0	0	0	0	1	6.09	6.06	6.50	5.83	6.91	0	0	0	0	0	0	0	10.86	10.82	11.02	11.10	11.04	11.17	11.23
1387902_a_at	Igkc	1	1	1	1	1	1	6.01	5.32	4.38	5.07	7.51	0	0	0	0	0	0	0	3.94	4.15	3.97	3.96	4.05	3.99	3.62
1387904_at	Ip6k1	1	0	0	0	1	0	8.26	8.29	8.06	8.71	8.29	0	0	0	0	0	0	0	7.78	7.76	7.74	7.68	7.64	7.55	7.74
1387906_a_at	Gnas	1	0	0	1	1	1	9.25	9.17	9.72	8.64	10.76	0	0	0	0	0	0	0	13.96	13.98	13.94	13.91	13.97	13.88	13.28
1387909_at	Abi2	1	0	1	0	0	0	7.29	7.56	7.88	7.33	7.47	0	0	0	0	0	0	0	8.87	8.96	9.03	9.05	9.02	8.91	9.26
1387914_at	Cyp27a1	1	0	0	0	0	1	5.25	5.02	4.84	5.34	4.77	0	0	0	0	0	0	0	4.37	4.37	4.32	4.29	4.26	4.21	3.84
1387918_at	Tpm3	1	0	0	1	0	0	7.84	7.82	7.79	7.30	7.64	0	0	0	0	0	0	0	10.62	10.63	10.67	10.73	10.58	10.59	10.26
1387926_at	Sc5d	1	0	0	1	1	0	6.57	6.54	6.96	5.97	6.41	0	0	0	0	0	0	0	8.30	8.31	8.40	8.51	8.50	8.19	7.76
1387930_at	Reg3a	1	1	1	1	1	1	11.19	9.71	7.55	8.68	12.57	0	0	0	0	0	0	0	3.75	3.81	3.94	3.79	3.83	3.80	3.01
1387938_at	Baalc	1	0	1	0	1	0	4.25	4.55	5.04	4.33	4.54	0	0	0	0	0	0	0	6.95	6.96	7.24	7.28	7.14	6.61	5.82
1387947_at	Mafb	1	0	0	1	0	0	7.44	7.37	7.41	6.91	7.44	0	0	0	0	0	0	0	5.85	5.86	6.07	5.72	5.81	5.95	6.26
1387948_at	Cilk1	1	0	0	0	1	0	6.98	6.85	6.63	7.32	6.75	0	0	0	0	0	0	0	6.71	6.55	6.65	6.48	6.54	6.53	6.39
1387961_at	Opcml	1	0	1	1	0	1	6.85	6.65	5.77	6.18	6.06	0	0	0	0	0	0	0	7.31	7.50	7.36	7.31	7.30	7.56	7.06
1387964_a_at	Ero1a	1	0	1	0	0	0	6.41	6.44	7.12	6.50	6.72	0	0	0	0	0	0	0	7.53	7.62	7.51	7.22	7.32	7.56	7.58
1387976_at	Slc9a3r2	1	0	0	0	0	1	6.35	6.30	6.09	6.42	5.86	0	0	0	0	0	0	0	6.01	6.01	6.05	5.90	5.88	5.98	5.53
1387994_at	Hsd17b6 /// LOC	1	0	1	0	0	0	3.99	3.63	3.40	3.59	3.69	0	0	0	0	0	0	0	3.78	3.92	3.79	3.77	3.72	3.83	3.55
1388000_at	Slc24a2	1	0	1	1	1	1	6.04	6.34	7.18	4.93	6.55	0	0	0	0	0	0	0	7.86	7.91	8.14	8.25	8.43	8.16	7.52
1388002_at	Taok1	1	0	1	0	1	0	6.41	6.45	7.06	6.08	6.73	0	0	0	0	0	0	0	7.94	7.86	8.01	8.06	7.96	7.77	7.69
1388016_a_at	Grip2	1	0	1	0	0	0	5.47	5.36	4.88	5.22	5.11	0	0	0	0	0	0	0	5.11	5.13	5.05	4.87	4.98	5.10	4.69
1388022_a_at	Dnm1l	1	0	1	0	1	0	4.03	4.26	4.77	3.59	4.19	0	0	0	0	0	0	0	7.79	7.80	7.90	7.97	7.96	7.62	6.58
1388027_a_at	Rtn4	1	0	1	1	1	0	7.50	7.47	8.48	6.49	7.65	0	0	0	0	0	0	0	10.85	10.89	10.93	10.84	10.95	10.94	10.58
1388036_a_at	Afdn	1	0	1	0	0	0	5.42	5.23	4.85	5.16	5.14	0	0	0	0	0	0	0	4.61	4.40	4.37	4.72	4.56	4.55	3.97
1388044_at	Pfkfb2	1	0	1	0	1	0	7.68	7.71	8.59	7.28	8.02	0	0	0	0	0	0	0	8.27	8.26	8.38	8.55	8.56	8.69	8.33
1388045_a_at	Cdh22	1	1	0	0	0	0	5.89	6.40	6.02	6.28	6.19	0	0	0	0	0	0	0	7.74	7.77	7.82	7.60	7.70	7.48	7.20
1388063_a_at	Pfkfb2	1	0	1	1	1	0	5.46	5.64	6.59	4.86	5.49	0	0	0	0	0	0	0	6.37	6.26	6.52	6.65	6.61	6.62	5.54
1388070_a_at	Akap1	1	0	0	1	0	0	5.17	4.80	4.98	4.66	4.72	0	0	0	0	0	0	0	7.33	7.29	7.39	7.47	7.42	7.56	6.74
1388089_a_at	Rnf4	1	0	0	1	1	0	7.86	7.68	7.99	7.16	7.62	0	0	0	0	0	0	0	10.52	10.50	10.53	10.63	10.55	10.44	9.45
1388103_at	Tmem37	1	0	1	0	1	0	5.52	5.60	4.81	5.80	5.28	0	0	0	0	0	0	0	4.29	4.38	4.34	4.34	4.34	4.42	4.22
1388108_at	Elovl6	1	0	0	0	1	0	5.99	5.77	6.45	5.62	6.37	0	0	0	0	0	0	0	7.91	7.78	7.94	8.08	7.94	7.83	7.59
1388109_at	Adgrf5	1	0	0	1	0	0	6.30	6.12	6.18	5.77	6.27	0	0	0	0	0	0	0	4.07	4.04	4.11	3.99	4.10	4.03	3.30
1388112_at	Slc25a4	1	0	1	0	1	0	9.14	8.95	9.82	8.69	9.30	0	0	0	0	0	0	0	13.15	13.13	13.16	13.19	13.14	13.06	12.76
1388127_at	Cyp4v2	1	0	0	0	0	1	7.47	7.74	7.94	7.38	6.24	0	0	0	0	0	0	0	8.04	7.93	8.07	8.25	8.17	8.16	7.86
1388131_at	Tubb2b	1	0	1	0	0	0	5.79	5.82	5.25	5.70	5.79	0	0	0	0	0	0	0	5.08	4.98	5.09	4.93	5.09	5.20	4.88
1388134_at	Eef1d	1	0	0	1	0	0	9.90	9.73	10.10	10.51	9.49	0	0	0	0	0	0	0	11.40	11.35	11.43	11.46	11.36	11.24	11.15
1388138_at	Thbs4	1	0	1	0	1	1	5.24	5.37	6.02	5.11	4.78	0	0	0	0	0	0	0	4.28	4.29	4.28	4.13	4.17	4.05	4.03
1388149_at	Tap1	1	1	0	0	0	0	5.55	6.82	5.85	5.87	5.90	0	0	0	0	0	0	0	7.64	7.63	7.66	7.64	7.60	7.87	7.84
1388153_at	Acsl1	1	0	0	0	1	0	8.00	7.87	7.82	8.39	7.83	0	0	0	0	0	0	0	7.78	7.75	7.80	7.76	7.83	8.20	8.66
1388154_at	E2f5	1	0	1	0	1	0	6.04	5.96	6.71	5.89	6.27	0	0	0	0	0	0	0	8.74	8.69	8.76	8.82	8.71	8.61	8.57
1388166_at	Igh-6	1	0	0	0	0	1	4.41	4.51	4.00	4.38	5.40	0	0	0	0	0	0	0	4.56	4.50	4.64	4.52	4.52	4.56	4.04
1388167_at	Nfib	1	0	0	0	1	0	6.95	6.61	6.58	7.29	6.52	0	0	0	0	0	0	0	5.50	5.50	5.53	5.67	5.56	5.53	5.17
1388169_at	Jmjd1c	1	0	1	0	0	0	8.18	8.20	8.86	8.27	8.31	0	0	0	0	0	0	0	10.00	9.98	9.94	9.94	10.08	10.17	9.81
1388176_at	Nat8f5	1	0	1	0	1	0	4.70	4.50	3.77	4.77	4.29	0	0	0	0	0	0	0	3.15	3.17	3.15	3.04	3.13	3.04	2.79
1388179_at	Acvr2b	1	0	0	1	1	0	5.40	5.55	5.24	6.13	5.37	0	0	0	0	0	0	0	5.75	5.87	5.79	5.60	5.57	5.53	5.53
1388182_at	Prim1	1	0	0	0	0	1	6.75	6.94	7.22	7.16	6.22	0	0	0	0	0	0	0	12.03	12.12	12.12	12.17	12.27	12.39	12.00
1388185_at	Rb1	1	0	1	0	1	0	7.13	7.15	7.72	6.98	7.56	0	0	0	0	0	0	0	3.73	3.70	3.68	3.69	3.63	3.55	3.44
1388188_at	Cyp7b1	1	0	1	0	0	0	3.57	3.63	3.04	3.53	3.26	0	0	0	0	0	0	0	3.27	3.26	3.37	3.22	3.30	3.34	2.81
1388190_at	Apob	1	0	1	0	1	1	8.94	8.53	8.04	8.66	8.12	0	0	0	0	0	0	0	3.88	4.01	3.94	3.80	3.82	3.89	3.59
1388200_at	Myl2	1	0	1	0	0	0	4.13	3.97	3.58	3.71	3.79	0	0	0	0	0	0	0	4.30	4.29	4.18	4.07	3.94	4.10	4.00
1388206_a_at	Eif5	1	0	0	1	1	0	8.08	8.01	8.18	7.12	7.74	0	0	0	0	0	0	0	10.08	10.05	10.15	10.25	10.17	9.90	9.02
1388210_at	Acot2	1	0	0	0	1	0	3.72	3.41	4.12	3.40	3.43	0	0	0	0	0	0	0	4.21	4.09	4.22	4.33	4.17	4.15	3.87
1388213_a_at	Hla-e	1	0	0	1	0	0	5.52	5.73	5.67	5.02	5.85	0	0	0	0	0	0	0	6.96	7.01	7.09	6.94	7.00	7.08	6.19
1388223_at	Gnat3	1	0	0	0	1	0	4.07	3.83	4.62	3.84	3.81	0	0	0	0	0	0	0	3.92	3.89	3.89	3.84	3.69	4.29	3.85
1388233_at	Cish	1	1	0	0	0	1	5.90	5.36	5.80	6.09	4.81	0	0	0	0	0	0	0	7.12	7.08	7.15	6.99	7.01	7.09	6.34
1388241_at	Insl3	1	0	1	0	0	0	5.51	5.60	4.92	5.29	5.40	0	0	0	0	0	0	0	5.19	5.36	5.19	5.13	5.09	5.07	5.09
1388244_s_at	Rpsa	1	0	0	1	0	1	12.21	12.32	12.26	12.69	11.42	0	0	0	0	0	0	0	11.49	11.51	11.47	11.47	11.40	11.39	11.30
1388246_at	Clu	1	0	1	1	0	0	8.27	8.06	7.71	7.78	7.95	0	0	0	0	0	0	0	8.38	8.30	8.45	8.52	8.66	8.76	7.71
1388267_a_at	---	1	0	1	1	1	1	7.83	7.40	6.35	7.01	6.90	0	0	0	0	0	0	0	5.79	5.68	5.71	5.31	5.53	5.55	6.86
1388277_at	LOC100361706	1	0	1	0	0	0	5.60	5.43	4.93	5.24	5.81	0	0	0	0	0	0	0	4.74	4.65	4.47	4.52	4.52	4.61	4.51
1388280_a_at	Dlg3	1	0	0	1	1	0	5.75	5.89	5.92	5.04	5.41	0	0	0	0	0	0	0	6.24	6.25	6.39	6.52	6.58	6.45	5.41
1388297_at	Eef1g	1	0	0	1	0	0	11.98	11.84	12.34	12.58	11.73	0	0	0	0	0	0	0	13.10	13.14	13.17	13.15	13.15	13.14	12.82
1388301_at	Uqcrc1	1	0	0	0	1	0	10.17	10.37	9.90	10.51	9.91	0	0	0	0	0	0	0	10.53	10.49	10.53	10.52	10.44	10.39	10.35
1388304_at	Ndufb5	1	0	0	1	0	0	10.76	10.89	10.75	11.23	10.59	0	0	0	0	0	0	0	11.73	11.75	11.71	11.68	11.71	11.70	11.32
1388311_at	Mrfap1	1	0	0	0	1	0	11.60	11.61	12.11	11.26	11.98	0	0	0	0	0	0	0	13.68	13.64	13.68	13.67	13.66	13.62	13.29
1388322_at	Eif4g1	1	0	0	1	1	0	8.22	8.10	7.96	8.79	8.46	0	0	0	0	0	0	0	9.75	9.69	9.80	9.74	9.70	9.51	9.69
1388330_at	Vkorc1	1	0	0	1	1	0	10.58	10.49	10.70	11.26	10.50	0	0	0	0	0	0	0	11.70	11.74	11.69	11.68	11.77	12.14	11.95
1388331_at	Hsp90b1	1	0	0	0	0	1	11.32	11.64	11.78	11.60	11.83	0	0	0	0	0	0	0	12.83	12.88	12.88	12.88	12.94	12.86	12.54
1388336_at	Atg13	1	0	0	0	1	0	7.49	7.31	7.22	7.86	7.53	0	0	0	0	0	0	0	7.97	8.03	7.97	7.95	7.91	7.95	7.88
1388339_at	Pea15	1	0	0	0	1	0	6.10	6.20	6.63	5.81	6.22	0	0	0	0	0	0	0	7.72	7.70	7.76	7.83	7.80	7.41	7.18
1388342_at	Etv3	1	0	1	0	1	0	6.82	6.68	7.43	6.72	7.07	0	0	0	0	0	0	0	9.04	8.98	9.07	9.14	9.07	9.11	8.38
1388343_at	Ndufb7	1	0	0	1	1	0	8.97	9.31	8.79	9.46	8.82	0	0	0	0	0	0	0	10.38	10.32	10.36	10.35	10.30	10.23	10.17
1388347_at	Ly6e	1	0	0	0	1	0	9.11	9.25	8.83	9.41	8.87	0	0	0	0	0	0	0	10.86	10.87	10.89	10.90	10.91	10.76	9.72
1388352_at	Naa20	1	0	1	0	1	1	7.00	7.19	7.77	6.87	7.55	0	0	0	0	0	0	0	10.11	10.13	10.20	10.23	10.22	10.33	9.99
1388353_at	Pa2g4	1	0	0	0	1	0	9.46	9.61	9.16	9.86	9.24	0	0	0	0	0	0	0	11.12	11.09	11.14	11.12	11.03	11.14	11.18
1388356_at	S100a16	1	0	1	0	1	1	8.98	8.78	8.32	9.26	8.42	0	0	0	0	0	0	0	7.08	7.12	7.02	6.96	7.02	7.13	6.80
1388366_at	Mrpl4	1	0	0	0	0	1	7.93	8.09	7.93	8.26	7.19	0	0	0	0	0	0	0	10.43	10.44	10.41	10.39	10.34	10.20	9.68
1388369_at	Tmed9	1	0	0	1	1	0	10.34	10.27	10.28	10.85	10.35	0	0	0	0	0	0	0	11.21	11.22	11.24	11.08	11.17	11.02	10.86
1388371_at	Prkcsh	1	0	0	1	0	0	9.00	9.06	9.22	9.47	9.10	0	0	0	0	0	0	0	11.42	11.47	11.54	11.48	11.49	11.38	11.07
1388390_at	Eif3h	1	0	0	1	1	1	10.43	10.22	10.43	11.01	9.94	0	0	0	0	0	0	0	11.90	11.92	11.89	11.84	11.81	11.83	11.81
1388399_at	Mast2	1	0	0	0	1	0	5.84	6.00	5.53	6.11	5.75	0	0	0	0	0	0	0	7.23	7.12	7.33	7.16	7.15	7.26	7.93
1388400_at	Cdc34	1	0	0	0	1	0	8.40	8.49	7.90	8.81	8.07	0	0	0	0	0	0	0	9.47	9.46	9.57	9.60	9.55	9.53	9.36
1388405_at	Ckap5	1	0	1	0	1	1	7.53	7.52	8.32	7.59	8.10	0	0	0	0	0	0	0	9.94	9.96	9.96	9.94	9.91	9.75	9.44
1388410_at	Ugp2	1	0	0	0	0	1	7.75	7.82	8.27	7.78	8.25	0	0	0	0	0	0	0	11.08	11.12	11.15	11.21	11.25	11.40	11.16
1388413_at	Rrbp1	1	0	0	1	1	0	10.09	10.10	9.81	10.63	9.77	0	0	0	0	0	0	0	9.78	9.92	9.80	9.79	9.75	9.85	9.78
1388420_at	---	1	0	1	0	1	0	7.22	7.14	7.89	7.12	7.37	0	0	0	0	0	0	0	10.54	10.53	10.59	10.64	10.60	10.49	10.19
1388430_at	Ptov1	1	0	0	1	1	0	7.94	8.24	7.69	8.44	7.99	0	0	0	0	0	0	0	8.59	8.57	8.61	8.54	8.57	8.28	8.34
1388431_at	Ss18	1	0	1	0	1	0	8.55	8.50	9.14	8.38	8.89	0	0	0	0	0	0	0	10.83	10.80	10.87	10.84	10.80	10.75	10.71
1388433_at	Krt19	1	0	0	0	0	1	6.95	7.24	7.27	6.87	5.10	0	0	0	0	0	0	0	9.08	9.24	9.23	9.27	9.48	9.43	9.20
1388434_at	Myg1	1	0	0	0	0	1	7.59	7.49	7.46	7.65	7.10	0	0	0	0	0	0	0	9.88	9.91	9.93	9.89	9.89	9.90	9.86
1388447_at	Lbh	1	0	0	1	1	0	9.01	9.13	8.76	10.00	9.12	0	0	0	0	0	0	0	7.20	7.26	7.27	7.10	7.12	7.24	7.11
1388451_at	Cacna2d1	1	0	1	1	1	1	8.93	8.99	9.83	8.24	9.73	0	0	0	0	0	0	0	11.50	11.58	11.62	11.69	11.69	11.64	10.74
1388453_at	Myadm	1	0	0	0	1	0	8.19	7.88	7.96	8.56	8.10	0	0	0	0	0	0	0	6.51	6.50	6.51	6.49	6.50	6.64	6.91
1388462_at	Sbk1	1	0	0	1	1	0	7.35	7.43	7.19	7.82	6.93	0	0	0	0	0	0	0	8.21	8.19	8.25	8.19	8.21	8.48	8.91
1388463_at	Tex264	1	0	0	0	1	1	8.76	8.55	8.24	8.84	7.99	0	0	0	0	0	0	0	7.65	7.74	7.74	7.68	7.61	7.64	7.19
1388469_at	Igf1	1	1	0	0	0	0	6.41	5.78	6.49	6.45	6.09	0	0	0	0	0	0	0	3.68	3.65	3.65	3.62	3.57	3.55	3.26
1388470_at	Fbxo9	1	0	0	0	0	1	8.12	8.18	7.93	8.15	7.52	0	0	0	0	0	0	0	8.15	8.11	8.18	8.18	8.22	8.01	7.79
1388478_at	Tbl1x	1	1	0	0	0	0	7.91	8.40	8.30	8.34	8.20	0	0	0	0	0	0	0	9.56	9.64	9.71	9.70	9.80	9.65	9.80
1388483_at	Cfl2	1	0	1	0	1	1	5.60	5.59	6.29	5.47	6.14	0	0	0	0	0	0	0	10.44	10.47	10.52	10.59	10.52	10.41	10.17
1388489_at	Ndufa3	1	0	0	0	1	0	10.77	10.84	10.27	10.94	10.40	0	0	0	0	0	0	0	11.79	11.78	11.76	11.66	11.67	11.58	11.39
1388491_at	Marchf5	1	0	0	0	1	0	6.96	6.60	7.34	6.65	6.93	0	0	0	0	0	0	0	10.22	10.28	10.31	10.29	10.28	10.20	9.93
1388494_at	Col4a2	1	0	0	0	0	1	7.89	7.69	7.65	7.83	7.41	0	0	0	0	0	0	0	6.36	6.24	6.18	5.92	6.05	6.13	5.66
1388505_at	Cbl	1	0	1	0	1	0	6.44	6.44	7.09	6.22	6.84	0	0	0	0	0	0	0	8.82	8.87	8.86	8.86	8.90	8.79	8.63
1388506_at	Dsp	1	0	0	0	0	1	8.35	8.31	8.77	8.49	7.66	0	0	0	0	0	0	0	8.10	8.10	8.15	8.15	8.11	7.92	7.74
1388518_at	Fbxw5	1	0	0	1	1	0	7.20	7.40	7.01	7.68	7.12	0	0	0	0	0	0	0	9.29	9.25	9.28	9.24	9.23	9.23	8.83
1388533_at	Ctdsp1	1	0	0	1	1	0	7.80	7.69	7.59	8.45	7.71	0	0	0	0	0	0	0	7.34	7.29	7.32	7.31	7.28	7.17	6.92
1388537_at	Nipsnap1	1	0	0	0	1	0	7.06	7.24	7.53	6.84	7.36	0	0	0	0	0	0	0	9.25	9.31	9.38	9.43	9.37	9.30	9.16
1388539_at	Pkp2	1	0	0	1	1	0	6.16	6.33	6.21	6.78	6.16	0	0	0	0	0	0	0	6.95	6.88	6.94	6.77	6.73	7.07	7.20
1388542_at	Use1	1	0	0	0	0	1	8.29	8.33	8.29	8.60	7.83	0	0	0	0	0	0	0	9.73	9.67	9.67	9.64	9.57	9.55	9.17
1388545_at	Smoc1	1	0	1	1	0	0	7.16	6.97	7.79	8.33	6.87	0	0	0	0	0	0	0	9.02	9.07	9.12	9.03	9.11	8.56	8.45
1388546_at	Ablim1	1	0	0	0	0	1	6.95	6.93	7.06	6.96	7.52	0	0	0	0	0	0	0	7.59	7.54	7.64	7.66	7.69	7.45	7.58
1388547_at	Cldn4	1	0	1	0	1	0	6.12	6.24	7.22	6.25	5.87	0	0	0	0	0	0	0	3.88	3.93	4.02	3.78	3.80	3.91	3.80

Probeset ID	Gene Symbol	Islet Data Set (log2 ratio ≥0.263; FDR <20%) union n=5325 1=YES 0=NO	Islet DRlyp/lyp ND vs ND+Lp299v (log2 ratio ≥0.263; FDR<20%) n=429 1=YES 0=NO	Islet DRlyp/lyp ND vs HCD (log2 ratio ≥0.263; FDR<20%) n=2247 1=YES 0=NO	Islet DRlyp/lyp ND vs HCD+Lp299v (log2 ratio ≥0.263; FDR<20%) n=1595 1=YES 0=NO	Islet DRlyp/lyp HCD vs HCD+Lp299v (log2 ratio ≥0.263; FDR<20%) n=3026 1=YES 0=NO	Islet DRlyp/lyp ND vs Flyp/lyp ND (log2 ratio ≥ 0.263; FDR<20%) n=1950 1=YES 0=NO	Islet Mean Log2 Intensity DRlyp/lyp ND	Islet Mean Log2 Intensity DRlyp/lyp ND+Lp299v	Islet Mean Log2 Intensity DRlyp/lyp HCD	Islet Mean Log2 Intensity DRlyp/lyp HCD+Lp299v	Islet Mean Log2 Intensity Flyp40 ND	Rinm5f data union of all conditions n=3705 1=YES 0=NO	Rinm5f 30 µM propionate/ 6 µM butyrate vs Media (FDR<20%; 1.5 fold) n=3 1=YES 0=NO	Rinm5f 60 µM propionate/ 12 µM butyrate vs Media (FDR<20%; 1.5 fold) n=18 1=YES 0=NO	Rinm5f 120 µM propionate/ 24 µM butyrate vs Media (FDR<20%; 1.5 fold) n=164 1=YES 0=NO	Rinm5f 240 µM propionate /48 µM butyrate vs Media (FDR<20%; 1.5 fold) n=268 1=YES 0=NO	Rinm5f 1500 µM propionate/ 300 µM butyrate vs Media (FDR<20%; 1.5 fold) n=2491 1=YES 0=NO	Rinm5f 500 µM propionate/ 500 µM butyrate vs Media (FDR<20%; 1.5 fold) n=3001 1=YES 0=NO	Rinm5f Mean Log2 Intensity Media only	Rinm5f Mean Log2 Intensity 30 µM propionate/ 6 µM butyrate	Rinm5f Mean Log2 Intensity 60 µM propionate/ 12 µM butyrate	Rinm5f Mean Log2 Intensity 120 µM propionate/ 24 µM butyrate	Rinm5f Mean Log2 Intensity 240 µM propionate/ 48 µM butyrate	Rinm5f Mean Log2 Intensity 1500 µM propionate /300 µM butyrate	Rinm5f Mean Log2 Intensity 500 µM propionate/ 500 µM butyrate
1388549_at	Ncoa4	1	0	0	0	1	0	8.82	8.65	9.23	8.52	9.01	0	0	0	0	0	0	0	11.00	11.05	10.97	10.81	10.91	10.91	10.70
1388551_at	Pcdhga1 /// Pcd	1	0	0	0	0	1	8.60	8.73	8.98	8.45	9.12	0	0	0	0	0	0	0	10.10	10.06	10.12	10.09	10.03	9.74	9.62
1388557_at	C7	1	1	0	1	1	0	6.97	6.11	7.01	5.69	6.63	0	0	0	0	0	0	0	3.37	3.41	3.46	3.52	3.42	3.51	3.23
1388574_at	Wars1	1	0	0	0	0	1	7.88	8.18	7.72	8.26	8.42	0	0	0	0	0	0	0	11.03	11.05	11.11	11.07	11.08	11.11	10.83
1388578_at	Tatdn3	1	0	0	0	0	1	6.83	6.74	6.96	6.82	6.34	0	0	0	0	0	0	0	7.27	7.28	7.35	7.40	7.22	7.14	6.93
1388583_at	Cxcl12	1	0	0	1	0	0	7.38	7.09	6.92	6.65	7.41	0	0	0	0	0	0	0	4.41	4.36	4.20	4.22	4.20	4.29	3.87
1388586_at	Synj1	1	0	0	0	0	1	7.92	8.05	8.41	7.77	8.50	0	0	0	0	0	0	0	10.41	10.39	10.40	10.43	10.41	10.34	10.41
1388587_at	Ier3	1	1	0	1	0	0	6.39	7.18	6.16	5.47	6.41	0	0	0	0	0	0	0	7.38	7.44	7.45	7.28	7.35	7.61	7.63
1388603_a_at	Isca1	1	0	0	0	0	1	8.10	7.89	8.07	8.24	9.01	0	0	0	0	0	0	0	9.94	9.93	9.82	9.71	9.76	9.77	9.61
1388608_x_at	Hba-a1 /// Hba-a	1	0	0	1	0	1	11.08	10.73	10.77	10.40	10.13	0	0	0	0	0	0	0	6.96	6.98	6.82	6.71	6.84	7.06	6.43
1388613_at	Isca1	1	0	0	0	1	0	7.71	7.75	7.23	7.87	8.09	0	0	0	0	0	0	0	9.13	9.23	9.15	9.08	9.08	9.08	8.80
1388615_at	Rap1a	1	0	0	0	1	0	9.17	9.11	9.57	8.80	9.38	0	0	0	0	0	0	0	11.16	11.19	11.21	11.23	11.23	11.27	10.94
1388617_at	Bphl	1	0	0	0	0	1	6.36	6.47	6.77	6.60	5.77	0	0	0	0	0	0	0	7.19	7.26	7.22	7.19	7.22	7.14	6.65
1388623_at	Ehd1	1	0	0	0	1	0	5.79	5.94	5.52	6.10	5.64	0	0	0	0	0	0	0	7.93	8.04	8.06	8.03	7.94	8.11	8.14
1388641_at	Gart	1	0	0	0	0	1	5.16	5.17	5.45	5.39	5.68	0	0	0	0	0	0	0	7.25	7.25	7.46	7.55	7.59	7.69	7.56
1388643_at	Fut8	1	0	0	0	1	0	7.91	8.02	7.55	8.18	7.97	0	0	0	0	0	0	0	9.90	9.90	10.03	10.11	10.11	10.24	9.97
1388663_at	Dedd2	1	1	0	0	0	0	5.04	4.53	4.88	4.94	4.58	0	0	0	0	0	0	0	6.06	5.99	6.15	6.06	6.01	6.26	6.28
1388665_at	C17orf49	1	0	0	0	1	0	7.10	7.30	6.91	7.47	7.04	0	0	0	0	0	0	0	7.28	7.22	7.32	7.21	7.17	7.19	7.39
1388667_at	Man2a1	1	0	0	0	1	0	8.54	8.31	8.27	8.83	8.20	0	0	0	0	0	0	0	8.75	8.78	8.80	8.92	8.98	9.14	8.98
1388669_at	Sf3a3	1	0	0	0	1	0	6.79	6.71	7.30	6.59	6.83	0	0	0	0	0	0	0	9.51	9.49	9.60	9.66	9.62	9.48	9.32
1388679_at	Tbc1d14	1	0	0	0	0	1	7.03	7.06	7.49	7.14	7.50	0	0	0	0	0	0	0	10.00	9.93	9.98	9.94	9.93	9.79	9.78
1388680_at	C1galt1c1	1	0	1	0	1	1	9.77	9.79	10.45	9.63	10.32	0	0	0	0	0	0	0	10.83	10.87	10.87	11.01	10.98	11.00	10.62
1388683_at	Naa38	1	0	0	0	1	0	9.29	9.43	9.02	9.59	9.13	0	0	0	0	0	0	0	9.43	9.42	9.38	9.40	9.30	9.16	9.20
1388689_at	Acyp2	1	0	0	1	0	0	6.98	6.61	6.97	6.47	6.76	0	0	0	0	0	0	0	3.50	3.39	3.41	3.61	3.50	3.19	2.75
1388690_at	Brms1l /// LOC1	1	0	0	0	1	0	7.62	7.63	8.16	7.38	7.74	0	0	0	0	0	0	0	9.73	9.75	9.79	9.87	9.88	9.94	9.62
1388699_at	Man2b1	1	0	0	1	1	0	9.42	9.43	9.35	9.95	9.12	0	0	0	0	0	0	0	9.40	9.45	9.51	9.51	9.57	9.74	9.49
1388718_at	Tmod1	1	0	1	0	1	0	6.64	6.81	7.52	6.67	6.85	0	0	0	0	0	0	0	6.70	6.69	6.61	6.53	6.52	6.31	5.77
1388722_at	Dnajb1	1	1	0	0	0	0	8.40	9.17	8.68	8.12	8.25	0	0	0	0	0	0	0	9.00	9.00	8.99	8.99	8.99	8.91	8.75
1388733_at	Bfar	1	0	0	0	1	0	6.73	6.74	7.26	6.46	6.64	0	0	0	0	0	0	0	9.35	9.33	9.44	9.33	9.36	9.14	8.65
1388738_at	Atrnl1	1	0	0	1	1	0	5.96	5.67	5.70	6.58	5.65	0	0	0	0	0	0	0	8.60	8.64	8.76	8.78	8.79	9.06	8.92
1388743_at	Chic1	1	0	1	0	1	0	9.53	9.80	10.17	9.31	9.95	0	0	0	0	0	0	0	8.93	8.97	9.05	9.16	9.16	9.44	9.57
1388747_at	Lcmt1	1	0	0	0	0	1	7.78	7.76	7.44	7.86	8.43	0	0	0	0	0	0	0	9.62	9.62	9.68	9.69	9.73	10.02	10.07
1388754_at	LOC100911177	1	0	0	0	0	1	8.38	7.96	8.38	8.78	7.47	0	0	0	0	0	0	0	10.82	10.88	10.97	11.13	11.09	10.99	10.57
1388755_at	Sec23a	1	0	1	0	1	0	6.13	6.12	7.01	5.84	6.16	0	0	0	0	0	0	0	8.38	8.46	8.42	8.44	8.42	8.37	8.04
1388765_at	Akt2	1	0	0	0	1	0	7.83	7.65	7.56	8.14	7.59	0	0	0	0	0	0	0	8.61	8.59	8.62	8.60	8.59	8.46	8.27
1388780_at	Terf2ip	1	0	1	0	1	0	7.88	7.88	8.46	7.74	7.85	0	0	0	0	0	0	0	8.65	8.66	8.75	8.86	8.77	8.72	8.37
1388788_at	Gcdh	1	0	0	0	0	1	9.70	9.48	9.30	9.65	9.21	0	0	0	0	0	0	0	8.47	8.56	8.56	8.62	8.61	8.84	8.67
1388792_at	Gadd45g	1	0	0	1	0	0	8.62	8.82	8.29	7.83	8.50	0	0	0	0	0	0	0	9.49	9.63	9.69	9.48	9.67	9.91	8.93
1388795_at	Tppp	1	0	0	1	0	0	5.76	5.39	5.80	6.33	5.34	0	0	0	0	0	0	0	7.63	7.74	7.65	7.54	7.62	7.72	7.63
1388799_at	Klhl7	1	0	1	0	1	0	8.60	8.68	9.50	8.22	8.99	0	0	0	0	0	0	0	10.22	10.30	10.29	10.35	10.39	10.48	10.12
1388802_at	Bex1 /// LOC100	1	0	1	0	1	0	8.58	8.46	9.31	8.36	8.82	0	0	0	0	0	0	0	12.16	12.29	12.31	12.37	12.42	12.31	11.79
1388809_at	Smpdl3a	1	1	0	1	0	1	9.30	8.72	9.44	9.99	8.51	0	0	0	0	0	0	0	9.35	9.42	9.46	9.52	9.68	9.71	9.07
1388814_at	Ddx19a	1	0	1	0	0	0	7.48	7.54	8.14	7.55	7.85	0	0	0	0	0	0	0	8.75	8.83	8.82	8.92	8.86	8.88	8.74
1388822_at	Scoc	1	0	0	0	0	1	9.50	9.59	10.01	9.45	9.97	0	0	0	0	0	0	0	11.15	11.15	11.15	11.19	11.20	11.19	11.00
1388825_at	Prkca	1	0	1	1	0	1	6.83	7.30	7.43	7.35	7.57	0	0	0	0	0	0	0	9.06	9.06	9.08	9.10	9.09	9.30	9.50
1388828_at	Sidt2	1	0	1	1	1	0	8.38	8.34	7.84	9.12	8.00	0	0	0	0	0	0	0	7.98	7.95	7.94	7.82	7.94	8.02	7.66
1388830_at	Pkn2	1	0	1	0	0	1	7.34	7.56	8.01	7.51	7.86	0	0	0	0	0	0	0	9.98	10.02	10.04	10.13	10.09	9.99	9.82
1388831_at	Slc9a3r2	1	0	0	0	0	1	7.26	7.18	6.82	7.18	6.66	0	0	0	0	0	0	0	6.01	6.06	6.04	5.99	5.99	6.23	6.02
1388835_at	Strn4	1	0	0	0	0	1	8.18	8.30	8.46	8.20	8.66	0	0	0	0	0	0	0	10.42	10.45	10.60	10.57	10.56	10.37	10.12
1388848_at	Inf2	1	0	0	0	1	0	5.57	5.46	5.16	5.81	5.40	0	0	0	0	0	0	0	6.52	6.64	6.53	6.50	6.54	6.77	7.10
1388849_at	Aup1	1	0	0	0	1	0	7.78	7.71	7.28	8.18	7.34	0	0	0	0	0	0	0	8.52	8.57	8.64	8.63	8.61	8.39	8.26
1388850_at	Hsp90aa1	1	0	0	0	0	1	9.57	9.73	10.00	9.54	10.09	0	0	0	0	0	0	0	12.62	12.62	12.67	12.75	12.67	12.58	12.36
1388852_at	Nomo1	1	0	0	0	1	0	9.23	9.33	9.05	9.65	9.34	0	0	0	0	0	0	0	11.05	10.99	11.04	10.97	11.03	11.11	10.79
1388856_at	Kitlg	1	1	0	0	1	0	7.02	6.46	7.57	6.70	6.57	0	0	0	0	0	0	0	4.33	4.39	4.42	4.44	4.38	4.40	4.67
1388857_at	Sec23b	1	0	0	0	0	1	10.12	10.20	10.39	9.92	10.76	0	0	0	0	0	0	0	10.40	10.46	10.45	10.49	10.50	10.25	9.97
1388858_at	Map2k3	1	0	0	0	1	0	7.59	7.55	7.31	7.88	7.36	0	0	0	0	0	0	0	9.16	9.06	9.12	9.22	9.10	9.15	9.10
1388862_at	Txnl1	1	0	0	0	0	1	7.33	7.47	7.65	7.13	7.86	0	0	0	0	0	0	0	10.46	10.49	10.56	10.65	10.64	10.51	9.84
1388864_at	Ddx54	1	0	0	0	1	0	5.72	5.46	5.25	5.88	5.62	0	0	0	0	0	0	0	8.07	7.93	8.00	7.99	7.94	7.92	7.94
1388865_at	Ppp4r2	1	0	0	0	1	0	8.65	8.51	9.15	8.42	8.70	0	0	0	0	0	0	0	11.64	11.65	11.74	11.86	11.86	11.89	11.62
1388868_at	Zfand5	1	0	0	0	1	0	8.24	8.19	8.73	7.89	8.43	0	0	0	0	0	0	0	11.30	11.29	11.34	11.41	11.35	11.41	11.37
1388870_at	Msi2	1	0	1	0	1	0	9.53	9.56	10.14	9.37	9.72	0	0	0	0	0	0	0	11.12	11.04	11.06	10.83	10.87	10.83	10.97
1388874_at	Mtss1	1	0	1	0	0	0	9.06	9.16	9.76	9.17	9.25	0	0	0	0	0	0	0	10.74	10.81	10.78	10.72	10.81	11.17	11.04
1388880_at	Slfn5	1	1	0	1	0	0	6.09	5.61	5.76	5.19	5.80	0	0	0	0	0	0	0	3.33	3.42	3.43	3.36	3.31	3.45	2.90
1388896_at	Usp33	1	0	1	0	1	0	8.19	8.27	8.87	7.99	8.57	0	0	0	0	0	0	0	10.34	10.39	10.38	10.45	10.50	10.50	9.99
1388900_at	LOC102549726	1	0	0	0	0	1	7.52	7.24	7.53	7.92	6.84	0	0	0	0	0	0	0	9.44	9.45	9.50	9.51	9.47	9.22	9.04
1388901_at	Fkbp5	1	0	0	1	1	0	7.23	6.85	7.52	6.68	6.86	0	0	0	0	0	0	0	8.60	8.55	8.73	8.78	8.78	8.97	9.00
1388903_at	Dynlt3	1	0	1	0	1	0	8.49	8.58	9.07	8.28	8.87	0	0	0	0	0	0	0	10.72	10.76	10.76	10.80	10.77	10.71	10.43
1388906_at	Tns3	1	0	1	0	1	0	9.22	9.26	9.84	9.00	9.39	0	0	0	0	0	0	0	10.77	10.77	10.88	10.91	10.95	10.81	10.53
1388929_at	---	1	0	1	0	1	0	8.62	8.68	9.21	8.42	8.89	0	0	0	0	0	0	0	9.38	9.45	9.48	9.56	9.58	9.65	9.34
1388941_at	Amz2	1	0	1	0	1	0	5.88	5.78	6.52	5.76	5.97	0	0	0	0	0	0	0	9.43	9.42	9.41	9.42	9.31	9.37	9.07
1388942_at	---	1	0	0	0	0	1	9.10	9.19	9.56	9.09	9.69	0	0	0	0	0	0	0	9.49	9.56	9.65	9.68	9.81	9.82	9.15
1388948_at	Stard10	1	0	0	0	1	1	9.24	9.38	8.81	9.65	8.69	0	0	0	0	0	0	0	9.55	9.50	9.61	9.62	9.57	9.72	9.48
1388955_at	---	1	0	1	1	0	1	6.62	6.34	5.86	6.14	6.16	0	0	0	0	0	0	0	4.91	5.06	5.10	4.79	4.89	4.95	4.76
1388969_at	Acsf3	1	0	0	0	1	0	7.55	7.59	7.28	7.93	7.38	0	0	0	0	0	0	0	8.52	8.44	8.46	8.38	8.37	8.31	8.00
1388970_at	Rasip1	1	0	0	0	0	1	5.26	5.04	4.95	5.00	4.79	0	0	0	0	0	0	0	5.69	5.74	5.76	5.65	5.72	5.94	5.96
1388977_at	Cept1	1	0	0	0	0	1	9.15	8.89	8.78	9.17	8.65	0	0	0	0	0	0	0	9.46	9.50	9.52	9.53	9.53	9.44	9.03
1388978_at	Tmem30a	1	0	0	0	1	0	8.85	8.71	9.36	8.38	9.04	0	0	0	0	0	0	0	10.87	10.87	10.89	10.87	10.95	11.10	10.58
1388985_at	---	1	1	1	0	1	1	9.82	10.36	8.86	10.08	8.96	0	0	0	0	0	0	0	10.92	11.04	11.05	10.85	10.98	10.69	9.96
1388994_at	Fzd6	1	0	1	0	1	0	6.27	6.41	7.06	6.35	6.48	0	0	0	0	0	0	0	7.87	7.81	7.91	7.91	7.93	8.21	7.84
1389003_at	Rhobtb3	1	0	1	0	1	1	6.81	6.89	7.75	6.93	7.39	0	0	0	0	0	0	0	8.25	8.27	8.31	8.30	8.35	8.33	8.00
1389008_at	---	1	0	1	0	1	1	6.89	6.96	7.82	6.95	7.38	0	0	0	0	0	0	0	10.47	10.47	10.54	10.51	10.59	10.30	9.88
1389014_at	Nampt	1	0	1	0	1	0	6.49	6.62	7.19	6.09	6.81	0	0	0	0	0	0	0	9.33	9.37	9.33	9.17	9.14	8.96	8.63
1389023_at	---	1	0	0	0	0	1	10.31	10.46	10.71	10.62	10.84	0	0	0	0	0	0	0	11.01	11.05	11.07	11.07	11.11	11.12	11.30
1389028_at	Ncoa6	1	0	0	0	1	0	6.90	6.79	7.38	6.55	7.07	0	0	0	0	0	0	0	9.23	9.21	9.28	9.29	9.24	8.88	8.47
1389051_at	Rxrb	1	0	0	0	1	0	5.71	5.78	5.44	6.08	5.50	0	0	0	0	0	0	0	6.77	6.83	6.91	6.71	6.60	6.71	7.09
1389057_at	Arv1	1	0	0	1	0	0	7.34	7.20	7.46	7.97	7.36	0	0	0	0	0	0	0	7.82	7.87	7.92	7.93	7.89	7.70	7.46
1389059_at	Nfix	1	0	0	0	1	0	6.16	6.13	5.76	6.42	5.81	0	0	0	0	0	0	0	6.53	6.50	6.68	6.54	6.56	6.48	6.67
1389062_at	Ik	1	0	0	1	1	0	7.69	7.55	8.09	7.06	7.68	0	0	0	0	0	0	0	10.53	10.56	10.60	10.63	10.56	10.31	9.76
1389064_at	Fem1c	1	0	0	0	0	1	6.93	6.94	7.18	7.30	7.44	0	0	0	0	0	0	0	8.06	8.16	8.18	8.21	8.14	8.04	7.89
1389066_at	Rcan2	1	0	1	0	0	0	7.29	7.46	8.01	7.42	7.65	0	0	0	0	0	0	0	7.93	7.95	8.09	8.06	8.09	8.36	8.47
1389067_at	Slco4a1	1	0	1	0	1	0	6.41	6.60	7.12	6.39	6.72	0	0	0	0	0	0	0	8.57	8.59	8.66	8.67	8.73	8.81	8.18
1389068_at	Wipi2	1	0	0	0	0	1	6.66	6.58	6.77	6.69	5.85	0	0	0	0	0	0	0	7.93	7.91	7.98	7.87	7.91	7.90	8.24
1389074_at	Fchsd2	1	0	0	1	0	0	7.25	7.32	7.62	7.75	7.63	0	0	0	0	0	0	0	9.26	9.22	9.38	9.39	9.29	9.26	9.32
1389081_at	Vps37b	1	0	0	1	1	0	5.56	5.76	5.44	6.12	5.80	0	0	0	0	0	0	0	7.98	7.96	8.03	8.06	7.99	8.16	8.26
1389082_at	Mindy2	1	0	0	0	1	0	6.24	6.09	6.67	5.96	6.35	0	0	0	0	0	0	0	7.51	7.68	7.60	7.51	7.61	7.65	7.72
1389088_at	Adnp	1	0	1	0	1	0	8.63	8.66	9.28	8.44	8.80	0	0	0	0	0	0	0	11.20	11.15	11.28	11.40	11.38	11.11	10.61
1389089_at	Slc39a7	1	0	0	0	1	0	8.94	8.84	8.53	9.14	8.69	0	0	0	0	0	0	0	10.41	10.43	10.49	10.40	10.36	10.21	10.11
1389095_at	Boc	1	0	0	0	0	1	4.46	4.12	4.16	4.34	3.94	0	0	0	0	0	0	0	3.79	3.81	3.78	3.69	3.76	3.73	3.72
1389101_at	Ccnc	1	0	0	0	0	1	6.08	6.16	6.61	6.22	6.58	0	0	0	0	0	0	0	8.67	8.80	8.83	8.91	8.97	8.75	7.97
1389104_s_at	Prdm2	1	0	0	0	1	0	6.05	6.23	5.90	6.47	6.08	0	0	0	0	0	0	0	6.62	6.67	6.63	6.54	6.51	6.34	6.19
1389115_at	Evpl	1	0	0	0	0	1	4.86	5.01	4.82	4.99	5.67	0	0	0	0	0	0	0	7.23	7.23	7.46	7.47	7.42	7.51	7.57
1389134_at	ZDHHC17	1	0	0	0	1	0	6.34	6.20	6.81	6.04	6.35	0	0	0	0	0	0	0	8.17	8.12	8.27	8.33	8.21	7.94	8.09
1389145_at	Cdc42ep2	1	1	1	1	0	1	6.73	6.14	6.06	6.05	6.10	0	0	0	0	0	0	0	5.58	5.63	5.44	5.34	5.42	5.74	5.62
1389147_at	---	1	0	1	0	1	1	9.70	9.54	10.41	9.60	10.21	0	0	0	0	0	0	0	11.40	11.50	11.51	11.51	11.54	11.45	11.05
1389154_at	Lias	1	0	0	0	1	0	7.30	7.35	7.09	7.65	7.18	0	0	0	0	0	0	0	8.95	8.98	9.02	9.04	9.01	9.06	8.86
1389188_at	Gpr108	1	0	0	1	1	0	8.16	8.23	8.12	8.68	8.23	0	0	0	0	0	0	0	9.73	9.76	9.74	9.73	9.79	9.84	9.61
1389202_at	Rpe	1	0	0	0	0	1	6.72	6.51	7.29	6.67	7.24	0	0	0	0	0	0	0	8.27	8.32	8.42	8.56	8.54	8.60	8.07
1389208_at	Armcx4	1	0	1	0	1	0	7.27	7.36	7.93	6.94	7.73	0	0	0	0	0	0	0	8.88	8.83	8.96	9.01	8.91	8.38	8.42
1389218_at	Uggt1	1	0	0	1	0	1	9.01	9.33	9.03	9.50	9.58	0	0	0	0	0	0	0	8.70	8.76	8.88	8.90	9.09	9.15	8.99
1389220_at	Bbip1	1	0	0	0	0	1	7.12	7.21	7.47	7.16	7.66	0	0	0	0	0	0	0	9.27	9.29	9.29	9.31	9.26	9.10	8.70
1389223_at	Rimklb	1	0	0	0	1	0	5.13	5.25	5.64	4.83	5.25	0	0	0	0	0	0	0	5.80	5.93	6.01	5.97	6.12	6.18	6.29
1389226_at	Stag1	1	1	0	0	0	0	6.91	6.43	7.04	7.35	6.95	0	0	0	0	0	0	0	7.26	7.39	7.45	7.45	7.39	7.08	6.97
1389238_at	Pes1	1	0	0	0	0	1	6.40	6.04	6.53	6.46	5.91	0	0	0	0	0	0	0	8.83	8.88	8.87	8.96	8.81	8.68	8.26
1389255_at	Cdh5	1	0	0	1	0	0	7.12	6.90	6.82	6.55	6.98	0	0	0	0	0	0	0	5.58	5.62	5.55	5.37	5.36	5.57	5.40
1389268_at	Poll	1	0	0	1	1	1	6.82	6.75	6.61	7.30	6.35	0	0	0	0	0	0	0	7.74	7.89	7.89	7.87	7.86	7.85	7.68
1389271_at	Vasn	1	1	0	0	0	1	7.56	6.97	7.29	7.37	6.91	0	0	0	0	0	0	0	6.31	6.22	6.33	6.35	6.51	6.74	6.69
1389279_at	Ran	1	0	1	0	0	1	6.77	6.91	7.37	6.78	7.36	0	0	0	0	0	0	0	9.72	9.76	9.93	10.06	10.08	10.02	9.48
1389292_at	Rab18	1	0	0	1	1	0	9.20	9.04	9.41	8.56	9.05	0	0	0	0	0	0	0	9.49	9.48	9.50	9.49	9.48	9.30	8.59
1389306_at	Matn2	1	0	1	0	1	0	5.93	6.23	6.58	5.80	6.05	0	0	0	0	0	0	0	4.70	4.81	4.83	4.82	4.81	4.60	6.06
1389308_at	Dnajb11	1	0	0	1	1	0	8.79	8.81	8.51	9.30	9.04	0	0	0	0	0	0	0	10.96	11.00	11.07	11.13	11.22	11.12	10.62
1389316_at	Usp9x	1	0	0	0	1	0	8.14	8.24	8.61	7.80	8.24	0	0	0	0	0	0	0	9.71	9.71	9.82	9.91	9.85	9.54	9.44
1389318_at	Daam1	1	0	1	0	0	0	7.79	7.93	8.40	7.87	8.23	0	0	0	0	0	0	0	8.69	8.66	8.66	8.73	8.64	8.56	8.61
1389319_at	Ergic1	1	0	1	0	0	1	8.32	8.34	8.91	8.42	8.83	0	0	0	0	0	0	0	9.99	9.89	9.96	9.95	9.97	9.98	9.21
1389321_at	---	1	0	0	0	1	0	6.32	6.30	5.85	6.56	6.28	0	0	0	0	0	0	0	7.50	7.48	7.48	7.14	7.24	7.24	6.79
1389323_at	Wdr61	1	0	0	0	0	1	9.22	8.93	9.03	9.02	8.51	0	0	0	0	0	0	0	9.77	9.75	9.82	9.86	9.85	9.77	9.59
1389325_at	Pdcd10	1	0	1	0	1	0	7.87	7.78	8.56	7.72	8.25	0	0	0	0	0	0	0	10.62	10.65	10.58	10.56	10.65	10.65	10.27
1389332_at	Ptprb	1	0	0	1	0	0	7.63	7.42	7.68	7.01	7.46	0	0	0	0	0	0	0	3.85	3.71	3.65	3.74	3.63	3.75	3.49
1389333_at	Fbxo3	1	0	1	0	1	0	8.09	7.95	8.68	7.88	8.31	0	0	0	0	0	0	0	10.70	10.77	10.66	10.73	10.69	10.51	9.84
1389340_at	Znf318	1	0	0	1	0	0	6.20	6.38	6.18	6.69	6.25	0	0	0	0	0	0	0	8.16	8.15	8.23	8.13	8.14	7.93	8.12
1389345_at	Fbxo3	1	0	0	1	1	0	6.54	6.25	6.82	6.03	6.60	0	0	0	0	0	0	0	9.44	9.38	9.40	9.34	9.28	8.95	9.03
1389353_at	Sema6d	1	0	1	1	1	0	6.65	6.46	7.21	6.09	6.72	0	0	0	0	0	0	0	6.60	6.56	6.72	6.84	6.84	6.93	7.25
1389358_at	Lpgat1	1	0	0	0	1	0	7.17	7.38	7.73	6.99	7.50	0	0	0	0	0	0	0	8.46	8.39	8.58	8.68	8.54	8.28	8.00
1389360_at	Fxyd3	1	0	1	0	1	1	8.83	8.65	8.16	8.85	8.14	0	0	0	0	0	0	0	5.98	6.07	5.76	5.80	5.85	6.01	5.83
1389362_at	Ptpn3	1	0	0	1	0	0	6.49	6.52	6.77	7.08	6.58	0	0	0	0	0	0	0	8.25	8.24	8.32	8.34	8.29	8.13	7.65
1389367_at	Iqcj-schip1	1	0	1	0	1	0	6.19	6.35	6.78	5.83	6.49	0	0	0	0	0	0	0	8.60	8.63	8.62	8.62	8.65	8.82	8.31

Probeset ID	Gene Symbol	Islet Data Set (log2 ratio ≥0.263; FDR <20%) union n=5325 1=YES 0=NO	Islet DRlyp/lyp ND vs ND+Lp299v (log2 ratio ≥0.263; FDR<20%) n=429 1=YES 0=NO	Islet DRlyp/lyp ND vs HCD (log2 ratio ≥0.263; FDR<20%) n=2247 1=YES 0=NO	Islet DRlyp/lyp ND vs HCD+Lp299v (log2 ratio ≥0.263; FDR<20%) n=1595 1=YES 0=NO	Islet DRlyp/lyp HCD vs HCD+Lp299v (log2 ratio ≥0.263; FDR<20%) n=3026 1=YES 0=NO	Islet DRlyp/lyp ND vs Flyp/lyp ND (log2 ratio ≥ 0.263; FDR<20%) n=1950 1=YES 0=NO	Islet Mean Log2 Intensity DRlyp/lyp ND	Islet Mean Log2 Intensity DRlyp/lyp ND+Lp299v	Islet Mean Log2 Intensity DRlyp/lyp HCD	Islet Mean Log2 Intensity DRlyp/lyp HCD+Lp299v	Islet Mean Log2 Intensity Flyp40 ND	Rinm5f data union of all conditions n=3705 1=YES 0=NO	Rinm5f 30 µM propionate/ 6 µM butyrate vs Media (FDR<20%; 1.5 fold) n=3 1=YES 0=NO	Rinm5f 60 µM propionate/ 12 µM butyrate vs Media (FDR<20%; 1.5 fold) n=18 1=YES 0=NO	Rinm5f 120 µM propionate/ 24 µM butyrate vs Media (FDR<20%; 1.5 fold) n=164 1=YES 0=NO	Rinm5f 240 µM propionate /48 µM butyrate vs Media (FDR<20%; 1.5 fold) n=268 1=YES 0=NO	Rinm5f 1500 µM propionate/ 300 µM butyrate vs Media (FDR<20%; 1.5 fold) n=2491 1=YES 0=NO	Rinm5f 500 µM propionate/ 500 µM butyrate vs Media (FDR<20%; 1.5 fold) n=3001 1=YES 0=NO	Rinm5f Mean Log2 Intensity Media only	Rinm5f Mean Log2 Intensity 30 µM propionate/ 6 µM butyrate	Rinm5f Mean Log2 Intensity 60 µM propionate/ 12 µM butyrate	Rinm5f Mean Log2 Intensity 120 µM propionate/ 24 µM butyrate	Rinm5f Mean Log2 Intensity 240 µM propionate/ 48 µM butyrate	Rinm5f Mean Log2 Intensity 1500 µM propionate /300 µM butyrate	Rinm5f Mean Log2 Intensity 500 µM propionate/ 500 µM butyrate
1389372_at	Dennd4c /// LOC	1	0	0	0	1	0	8.08	7.91	8.62	7.81	8.28	0	0	0	0	0	0	0	9.34	9.41	9.35	9.27	9.41	9.57	9.79
1389377_at	Insig2	1	0	0	0	1	0	6.84	6.67	7.25	6.50	6.93	0	0	0	0	0	0	0	9.58	9.72	9.65	9.63	9.77	9.76	9.52
1389383_at	LOC103689920 /	1	0	1	0	1	0	7.22	7.11	7.80	7.04	7.60	0	0	0	0	0	0	0	8.83	8.80	8.85	8.86	8.95	8.77	8.16
1389390_at	Gatad1	1	0	0	0	1	0	7.79	7.70	8.11	7.35	7.75	0	0	0	0	0	0	0	10.06	9.96	10.00	10.02	9.91	9.76	9.50
1389403_at	Bmp7	1	0	0	1	1	1	6.38	6.33	6.00	7.14	5.82	0	0	0	0	0	0	0	7.38	7.37	7.44	7.28	7.34	7.51	7.33
1389412_at	---	1	0	0	0	0	1	7.66	7.74	7.92	7.83	8.74	0	0	0	0	0	0	0	8.69	8.71	8.79	8.80	8.84	8.92	9.36
1389413_at	Evi2a	1	0	0	0	1	0	6.78	6.39	6.31	6.94	6.36	0	0	0	0	0	0	0	3.51	3.47	3.51	3.42	3.53	3.48	3.15
1389414_at	---	1	0	0	1	0	0	7.01	7.01	7.08	7.48	7.15	0	0	0	0	0	0	0	9.40	9.43	9.44	9.41	9.47	9.60	9.72
1389419_at	LOC102553917	1	0	0	0	0	1	4.66	4.50	4.84	4.75	5.38	0	0	0	0	0	0	0	6.00	6.21	6.16	6.27	6.33	6.06	6.27
1389424_at	Spaca6	1	0	1	0	0	1	5.50	5.74	6.18	5.80	6.01	0	0	0	0	0	0	0	6.56	6.50	6.64	6.47	6.46	5.99	5.71
1389429_at	---	1	0	0	0	0	1	4.53	4.79	4.70	4.75	5.19	0	0	0	0	0	0	0	6.46	6.46	6.46	6.49	6.42	6.31	6.36
1389445_at	Znf688	1	0	0	0	0	1	5.60	5.94	5.86	5.82	6.14	0	0	0	0	0	0	0	8.42	8.48	8.45	8.43	8.41	8.20	7.96
1389447_at	Wdr6	1	0	0	0	0	1	6.00	6.26	6.52	6.15	6.52	0	0	0	0	0	0	0	8.37	8.42	8.49	8.55	8.52	8.08	7.67
1389460_at	---	1	0	1	0	1	0	3.71	3.47	4.49	3.72	3.89	0	0	0	0	0	0	0	7.09	7.17	7.28	7.37	7.44	7.47	7.46
1389464_at	Lnx1	1	1	0	0	0	0	6.11	5.58	6.03	6.09	5.76	0	0	0	0	0	0	0	7.90	7.97	7.96	7.76	7.75	7.79	7.57
1389467_at	Tmem100	1	0	1	0	1	1	5.47	5.60	6.05	5.02	6.02	0	0	0	0	0	0	0	3.83	3.96	3.87	3.76	3.82	3.82	3.46
1389469_at	Chd1l	1	0	1	0	0	0	6.55	6.66	7.26	6.60	6.64	0	0	0	0	0	0	0	7.87	7.78	7.90	7.91	7.82	8.03	7.94
1389470_at	Cfb	1	0	1	0	0	0	4.68	4.57	4.04	4.41	4.39	0	0	0	0	0	0	0	4.29	4.43	4.45	4.31	4.24	4.37	4.15
1389482_at	Krcc1	1	0	0	0	1	0	8.21	8.03	8.71	7.86	8.45	0	0	0	0	0	0	0	9.19	9.24	9.29	9.35	9.33	9.43	9.22
1389486_at	---	1	0	0	0	1	0	10.53	10.45	10.20	10.97	10.16	0	0	0	0	0	0	0	4.93	4.97	5.04	4.87	4.95	5.13	5.79
1389491_at	Sfxn5	1	0	1	0	1	0	6.38	6.19	5.77	6.35	5.98	0	0	0	0	0	0	0	7.88	7.87	7.92	7.81	7.79	7.47	7.39
1389496_at	Akap7	1	0	0	0	0	1	6.06	6.27	6.60	6.23	6.83	0	0	0	0	0	0	0	7.91	8.04	7.95	8.02	7.97	8.20	8.11
1389500_at	Rhou	1	0	1	0	1	1	7.92	7.94	7.21	8.11	7.18	0	0	0	0	0	0	0	8.65	8.75	8.72	8.62	8.73	8.27	8.52
1389515_at	Rfxank	1	0	0	0	1	0	5.44	5.79	5.19	5.77	5.19	0	0	0	0	0	0	0	6.19	6.19	6.35	6.49	6.49	6.38	6.67
1389521_at	Ivns1abp	1	0	1	0	0	0	8.18	8.05	8.77	8.12	8.55	0	0	0	0	0	0	0	11.13	11.14	11.15	11.16	11.17	11.24	10.80
1389524_at	Ythdf3	1	0	0	1	1	0	7.43	7.14	7.82	6.85	7.37	0	0	0	0	0	0	0	9.48	9.50	9.61	9.64	9.60	9.40	9.03
1389526_at	Efr3a	1	0	0	0	1	0	8.43	8.16	8.92	8.21	8.79	0	0	0	0	0	0	0	10.36	10.32	10.39	10.42	10.47	10.78	10.81
1389528_s_at	Jun	1	0	0	1	0	1	10.35	10.73	10.27	9.68	9.81	0	0	0	0	0	0	0	8.65	8.62	8.75	8.55	8.43	8.45	7.55
1389530_at	Slc35e1	1	0	0	0	1	1	6.63	6.61	7.03	6.19	7.12	0	0	0	0	0	0	0	10.59	10.63	10.55	10.39	10.43	10.32	10.06
1389533_at	Fbln2	1	0	1	0	1	0	5.68	5.79	5.11	5.89	5.45	0	0	0	0	0	0	0	5.38	5.18	5.48	5.07	4.98	5.22	4.89
1389534_at	Ube2e3	1	0	1	0	0	0	8.91	8.87	9.56	8.94	9.34	0	0	0	0	0	0	0	10.78	10.79	10.86	10.91	10.87	10.98	10.88
1389539_at	Appl1	1	0	0	0	1	0	7.58	7.50	8.09	7.30	7.85	0	0	0	0	0	0	0	9.87	9.85	9.89	9.83	9.82	9.78	9.44
1389540_at	Iqsec1	1	0	0	0	1	0	6.71	6.58	6.21	6.96	6.51	0	0	0	0	0	0	0	6.61	6.74	6.76	6.56	6.73	6.97	7.11
1389555_at	Tcf19	1	0	0	1	1	0	6.23	6.29	6.15	6.75	6.31	0	0	0	0	0	0	0	10.41	10.39	10.45	10.54	10.53	10.71	10.64
1389556_at	Kifap3	1	0	1	0	1	0	7.82	7.94	8.43	7.66	8.23	0	0	0	0	0	0	0	8.89	8.96	9.08	9.10	9.06	8.80	8.80
1389558_at	Bmt2	1	0	1	0	1	1	6.96	7.16	7.82	6.95	7.67	0	0	0	0	0	0	0	9.23	9.29	9.25	9.27	9.21	9.07	8.81
1389562_at	---	1	0	0	0	0	1	5.99	6.20	6.28	5.94	6.69	0	0	0	0	0	0	0	10.11	10.07	10.11	10.09	10.02	9.77	9.52
1389569_at	Brix1	1	0	0	0	1	0	7.25	7.07	7.56	6.80	7.19	0	0	0	0	0	0	0	9.20	9.23	9.34	9.40	9.40	9.18	8.76
1389575_at	C11orf58	1	0	0	0	1	0	8.84	8.83	9.34	8.57	9.07	0	0	0	0	0	0	0	11.02	11.03	11.09	11.06	11.05	10.96	10.56
1389580_at	Hltf	1	0	1	0	1	0	5.95	6.02	6.65	5.92	6.25	0	0	0	0	0	0	0	8.97	8.97	8.96	8.99	8.96	9.39	9.51
1389581_at	Il33	1	0	1	0	0	0	4.91	4.88	4.34	4.64	4.92	0	0	0	0	0	0	0	4.55	4.59	4.46	4.43	4.46	4.64	4.84
1389583_at	Slc25a34	1	1	0	1	0	0	4.33	3.74	3.91	3.83	3.88	0	0	0	0	0	0	0	4.50	4.39	4.51	4.30	4.39	4.59	4.29
1389584_at	---	1	0	1	0	1	1	6.52	6.95	7.43	6.16	7.42	0	0	0	0	0	0	0	7.08	7.15	7.29	7.48	7.47	7.54	7.11
1389586_at	Ednrb	1	0	0	0	1	0	8.12	7.87	8.51	7.65	7.94	0	0	0	0	0	0	0	5.12	5.14	4.95	4.83	4.93	5.02	4.69
1389587_at	Umps	1	0	0	0	0	1	8.46	8.37	8.18	8.63	7.97	0	0	0	0	0	0	0	9.97	9.92	10.04	10.08	10.05	10.17	10.10
1389600_at	Mgc116197	1	0	0	0	1	1	7.27	7.20	6.84	7.65	7.77	0	0	0	0	0	0	0	11.87	11.90	11.90	11.88	11.88	11.60	11.52
1389601_at	Nfib	1	0	0	1	1	0	7.59	7.40	7.42	8.17	7.23	0	0	0	0	0	0	0	4.95	5.04	5.12	5.29	5.36	5.26	5.29
1389616_at	Nsd2	1	0	1	0	0	1	4.64	4.54	5.25	4.70	5.14	0	0	0	0	0	0	0	9.34	9.22	9.37	9.37	9.32	9.16	9.20
1389622_at	Slc25a13	1	0	1	0	0	0	7.09	6.85	6.56	6.98	6.74	0	0	0	0	0	0	0	9.01	9.05	9.02	9.05	9.09	9.46	9.59
1389644_at	Tbc1d31	1	0	0	0	0	1	6.07	6.17	6.63	6.16	6.65	0	0	0	0	0	0	0	9.17	9.21	9.11	9.11	9.15	8.98	8.81
1389650_at	Idh2	1	0	0	0	0	1	6.11	6.13	6.18	6.18	5.35	0	0	0	0	0	0	0	4.73	4.74	4.91	4.87	4.85	5.07	5.26
1389652_at	Clcn3	1	0	0	0	0	1	8.61	8.40	8.76	8.86	9.11	0	0	0	0	0	0	0	10.37	10.41	10.46	10.52	10.45	10.45	10.65
1389654_at	Pls1	1	0	1	0	1	0	8.57	8.58	9.49	8.39	8.94	0	0	0	0	0	0	0	9.81	9.77	9.82	9.87	9.73	9.81	9.82
1389657_at	Znf524	1	0	0	0	1	0	6.86	7.04	6.66	7.32	6.77	0	0	0	0	0	0	0	7.69	7.64	7.63	7.64	7.59	7.40	7.25
1389667_at	Mtres1	1	0	0	0	1	0	6.63	6.57	6.41	7.01	6.42	0	0	0	0	0	0	0	8.08	8.21	8.19	8.27	8.29	8.35	8.09
1389678_at	SLC25A48	1	0	1	0	0	0	5.58	5.44	4.97	5.34	5.39	0	0	0	0	0	0	0	4.76	4.76	4.76	4.66	4.61	4.88	4.84
1389679_at	Glcci1	1	0	0	0	1	0	6.70	6.59	7.24	6.44	6.72	0	0	0	0	0	0	0	8.91	8.87	8.90	8.93	8.98	9.03	9.45
1389681_at	Nectin2	1	0	1	0	1	1	5.73	5.34	4.96	5.63	5.05	0	0	0	0	0	0	0	7.90	7.95	8.01	7.85	7.99	8.21	7.79
1389682_at	Chac2	1	0	1	0	0	1	5.13	5.21	5.86	5.17	5.69	0	0	0	0	0	0	0	8.54	8.62	8.59	8.64	8.72	8.54	7.88
1389698_at	Unc13b	1	0	0	0	0	1	7.33	7.51	7.82	7.29	7.97	0	0	0	0	0	0	0	8.61	8.60	8.70	8.67	8.74	8.95	9.04
1389699_at	Dhx34	1	0	0	0	1	0	6.12	6.32	5.69	6.30	6.05	0	0	0	0	0	0	0	6.70	6.66	6.74	6.66	6.69	6.52	6.17
1389716_at	---	1	0	0	1	0	0	5.38	5.35	5.38	5.87	5.82	0	0	0	0	0	0	0	7.33	7.42	7.46	7.61	7.56	7.87	7.79
1389730_at	---	1	0	1	0	1	0	6.47	6.44	7.25	6.21	6.75	0	0	0	0	0	0	0	9.50	9.57	9.59	9.67	9.63	9.46	9.13
1389733_at	Mars1	1	0	0	0	0	1	9.39	9.16	9.07	9.30	8.91	0	0	0	0	0	0	0	10.87	10.84	10.91	10.94	10.94	10.75	10.23
1389735_at	Rps6ka6	1	0	0	0	0	1	3.94	3.94	4.48	4.25	4.69	0	0	0	0	0	0	0	7.33	7.36	7.51	7.70	7.60	7.53	7.89
1389739_at	Neurl2	1	0	1	0	0	0	5.30	5.17	4.71	5.06	5.05	0	0	0	0	0	0	0	5.47	5.32	5.29	5.16	5.08	5.18	4.95
1389746_at	Naglu	1	0	1	0	1	1	6.81	6.78	6.21	6.93	6.05	0	0	0	0	0	0	0	8.89	8.99	9.00	8.87	8.99	8.91	8.32
1389747_at	Slc26a8	1	0	1	1	1	1	7.32	7.26	6.71	7.91	6.68	0	0	0	0	0	0	0	7.12	7.03	7.10	7.06	7.13	7.29	7.60
1389748_at	Runx1t1	1	0	0	0	0	1	4.21	4.05	4.35	4.02	5.34	0	0	0	0	0	0	0	7.83	7.77	7.90	7.88	7.61	7.30	7.61
1389760_at	Pcgf6	1	0	1	0	0	0	5.69	5.75	6.32	5.69	5.97	0	0	0	0	0	0	0	9.43	9.39	9.43	9.48	9.47	9.50	9.09
1389762_at	Fcgbp	1	0	1	0	1	0	7.12	7.03	6.26	7.36	6.77	0	0	0	0	0	0	0	6.46	6.49	6.48	6.60	6.52	6.37	6.30
1389765_at	Nle1	1	0	0	0	0	1	7.30	7.10	6.88	7.36	6.83	0	0	0	0	0	0	0	8.35	8.43	8.53	8.57	8.48	8.26	7.97
1389769_at	Ints1	1	0	0	0	1	0	5.65	5.83	5.24	6.00	5.51	0	0	0	0	0	0	0	8.03	8.00	8.03	7.98	7.89	7.80	7.91
1389783_s_at	Fcgbp /// Fcgbp	1	0	1	0	1	0	6.74	6.77	5.27	6.96	6.34	0	0	0	0	0	0	0	6.23	6.22	6.31	6.38	6.33	6.28	6.54
1389785_at	Acy3	1	0	0	1	1	0	5.94	5.67	5.79	6.46	5.86	0	0	0	0	0	0	0	4.26	4.40	4.29	4.24	4.25	4.28	4.30
1389800_at	Pwwp2b	1	0	0	1	1	0	5.84	6.03	5.60	6.32	5.86	0	0	0	0	0	0	0	7.96	7.98	8.08	8.03	8.02	7.99	7.85
1389806_at	Stard10	1	0	0	1	1	1	5.33	5.56	5.30	5.90	6.56	0	0	0	0	0	0	0	5.98	6.07	6.31	6.32	6.39	6.40	6.44
1389809_at	Zbp1	1	0	0	0	1	0	6.22	6.36	5.77	6.46	6.14	0	0	0	0	0	0	0	7.14	7.15	7.12	7.05	7.10	7.11	7.41
1389814_at	Huwe1	1	1	0	1	1	0	4.37	5.19	4.40	5.08	4.61	0	0	0	0	0	0	0	4.60	4.70	4.87	4.80	4.74	4.53	3.70
1389830_at	Ubtf	1	0	0	0	1	0	5.47	5.43	5.29	5.92	5.17	0	0	0	0	0	0	0	7.78	7.80	7.98	7.99	7.97	7.93	7.96
1389834_at	Nudt8	1	0	1	0	1	0	4.34	4.23	3.61	4.22	3.98	0	0	0	0	0	0	0	5.03	4.96	5.05	4.99	4.93	4.81	4.21
1389841_at	---	1	0	0	1	1	0	4.89	4.57	4.78	5.60	4.70	0	0	0	0	0	0	0	3.64	3.52	3.64	3.52	3.51	3.54	3.18
1389868_at	Ddx6	1	0	1	0	0	1	8.90	8.93	9.58	8.95	9.35	0	0	0	0	0	0	0	9.55	9.40	9.53	9.53	9.46	9.45	9.90
1389873_at	Pycard	1	0	0	0	0	1	6.14	6.31	6.10	6.31	6.68	0	0	0	0	0	0	0	5.91	5.85	5.90	5.92	5.84	5.90	6.02
1389876_at	Camk2n1	1	0	0	0	1	0	8.60	8.60	9.05	8.25	8.92	0	0	0	0	0	0	0	9.98	9.84	9.98	9.85	9.90	9.69	9.09
1389897_at	Cops7a	1	0	0	1	1	0	6.94	6.74	7.42	6.45	6.96	0	0	0	0	0	0	0	8.32	8.34	8.41	8.45	8.31	8.17	7.28
1389902_at	Hm13	1	0	0	1	1	0	11.17	11.15	10.74	11.67	10.79	0	0	0	0	0	0	0	10.96	11.02	11.06	11.11	11.11	10.87	10.61
1389904_at	Actr2	1	0	0	0	1	0	10.18	10.13	10.73	10.02	10.63	0	0	0	0	0	0	0	12.69	12.67	12.69	12.68	12.69	12.61	12.50
1389905_at	---	1	1	0	0	0	1	6.06	5.39	5.65	5.69	5.21	0	0	0	0	0	0	0	3.42	3.50	3.46	3.45	3.41	3.42	3.03
1389911_at	Metrnl	1	0	1	0	1	1	6.93	6.81	6.10	6.90	6.23	0	0	0	0	0	0	0	8.68	8.65	8.63	8.51	8.44	8.61	8.17
1389918_at	Palld	1	1	0	0	0	1	5.72	6.41	6.06	6.14	6.73	0	0	0	0	0	0	0	8.53	8.50	8.58	8.51	8.49	8.73	9.41
1389930_at	HSPA9	1	0	0	1	1	0	6.22	6.14	6.45	5.57	6.06	0	0	0	0	0	0	0	8.30	8.13	8.29	8.50	8.41	8.35	6.34
1389933_at	Ppp4r3a	1	0	1	0	0	0	5.78	5.66	5.24	5.67	5.41	0	0	0	0	0	0	0	5.72	5.66	5.70	5.38	5.44	5.43	5.12
1389961_at	Entr1	1	0	1	1	1	0	5.26	5.36	5.93	4.71	5.26	0	0	0	0	0	0	0	8.66	8.68	8.75	8.81	8.80	8.74	7.64
1389963_at	Mpp1	1	0	0	1	1	0	8.34	8.51	8.02	8.89	8.17	0	0	0	0	0	0	0	9.48	9.50	9.61	9.60	9.64	9.89	9.66
1389966_at	Col6a3	1	1	0	1	0	1	7.01	6.34	6.81	6.39	6.54	0	0	0	0	0	0	0	4.22	3.99	4.06	3.91	4.09	4.03	3.73
1389971_at	Sgce	1	0	0	1	0	0	5.47	5.15	5.38	4.83	5.23	0	0	0	0	0	0	0	8.09	8.19	8.28	8.42	8.52	8.27	7.62
1389972_at	Rgcc	1	1	0	1	1	1	5.56	4.96	5.58	4.69	5.05	0	0	0	0	0	0	0	4.20	4.16	4.06	4.00	4.09	4.16	3.74
1389974_at	---	1	0	0	1	1	0	4.78	4.74	5.27	4.16	4.81	0	0	0	0	0	0	0	8.48	8.45	8.64	8.51	8.59	8.66	7.67
1389975_at	Elavl4	1	0	0	0	1	1	7.10	7.22	7.52	6.79	7.68	0	0	0	0	0	0	0	8.24	8.36	8.45	8.54	8.55	8.30	7.68
1389985_at	Tfe3	1	0	0	0	0	1	4.54	4.31	4.27	4.29	4.04	0	0	0	0	0	0	0	6.06	6.19	6.27	6.19	6.07	6.15	5.01
1389989_at	Atrx	1	0	1	1	0	1	5.85	5.58	6.27	6.36	6.23	0	0	0	0	0	0	0	5.81	5.69	6.00	6.17	5.88	5.61	5.41
1389995_at	Srrm1	1	0	0	0	1	0	6.39	6.49	6.02	6.63	6.32	0	0	0	0	0	0	0	6.90	6.77	6.92	6.64	6.71	6.65	7.18
1390000_at	Kdm6b	1	0	0	1	1	0	7.16	7.29	6.81	8.00	7.35	0	0	0	0	0	0	0	7.45	7.38	7.53	7.20	7.19	7.10	7.23
1390019_at	H3-3a /// H3-3b	1	0	1	0	0	0	10.76	10.76	11.33	10.65	11.03	0	0	0	0	0	0	0	12.74	12.75	12.75	12.74	12.72	12.85	12.60
1390020_at	Ogdh	1	0	0	0	0	1	8.13	8.18	8.08	8.49	8.67	0	0	0	0	0	0	0	10.21	10.12	10.27	10.28	10.26	10.21	10.33
1390031_at	Orai1	1	0	0	0	1	0	6.58	6.56	6.28	6.97	6.34	0	0	0	0	0	0	0	7.31	7.33	7.41	7.34	7.34	7.51	7.51
1390040_at	Bre	1	0	0	1	1	0	7.02	7.06	7.57	6.29	7.11	0	0	0	0	0	0	0	9.54	9.58	9.59	9.57	9.55	9.68	9.38
1390043_at	Smarcc2	1	0	0	0	1	0	5.19	5.20	4.88	5.51	4.90	0	0	0	0	0	0	0	7.28	7.14	7.27	7.01	7.05	6.76	6.29
1390063_at	Mfap3	1	0	1	0	1	1	4.76	4.54	5.48	4.30	6.27	0	0	0	0	0	0	0	8.50	8.50	8.65	8.84	8.74	8.67	8.54
1390068_at	Nfe2l1	1	0	0	0	0	1	6.24	5.99	5.76	6.00	5.69	0	0	0	0	0	0	0	7.13	7.01	7.23	7.17	7.28	7.15	6.84
1390070_at	LOC100911253	1	0	0	1	0	0	5.39	5.17	5.78	5.87	5.47	0	0	0	0	0	0	0	4.01	4.04	3.95	3.84	3.93	3.81	3.35
1390077_at	Tram1	1	0	0	1	0	0	5.75	5.42	5.56	5.13	5.40	0	0	0	0	0	0	0	7.54	7.29	7.69	7.82	7.79	7.83	7.09
1390090_at	---	1	0	1	0	0	0	5.03	4.77	4.46	4.88	4.72	0	0	0	0	0	0	0	5.54	5.70	5.68	5.36	5.49	5.54	5.20
1390097_at	Tspyl4	1	0	0	0	0	1	7.24	7.36	7.70	7.41	8.17	0	0	0	0	0	0	0	9.52	9.43	9.54	9.58	9.44	9.06	8.98
1390099_at	Ube3a	1	0	0	0	1	0	5.11	5.13	5.50	4.78	5.09	0	0	0	0	0	0	0	7.70	7.62	7.70	7.80	7.75	7.51	6.18
1390109_at	LOC100910069 /	1	0	0	0	0	1	8.64	8.38	8.84	8.71	8.13	0	0	0	0	0	0	0	10.01	10.01	10.08	10.23	10.19	9.64	8.98
1390111_at	Fam91a1	1	0	1	0	1	0	7.71	7.63	8.43	7.27	7.96	0	0	0	0	0	0	0	10.82	10.83	10.86	10.88	10.90	10.99	10.79
1390113_a_at	Larp1	1	0	1	0	1	0	10.03	10.02	9.44	10.15	9.79	0	0	0	0	0	0	0	9.92	9.95	10.02	10.03	10.03	10.03	9.89
1390123_at	Tmem45b	1	0	1	0	0	0	4.51	4.44	3.97	4.31	4.48	0	0	0	0	0	0	0	4.79	4.84	4.71	4.56	4.56	4.68	4.67
1390127_at	Dixdc1	1	0	0	1	1	0	6.39	6.25	6.31	6.93	6.20	0	0	0	0	0	0	0	8.05	8.15	8.10	8.02	8.20	8.37	8.16
1390130_at	---	1	0	1	0	1	0	6.15	6.14	5.46	6.08	6.01	0	0	0	0	0	0	0	7.27	7.31	7.37	7.53	7.55	7.57	7.05
1390138_at	Ccdc8	1	0	0	0	0	1	5.69	5.85	6.11	5.68	6.49	0	0	0	0	0	0	0	6.20	6.22	6.29	6.23	6.29	6.50	6.50
1390142_at	Morc3	1	0	1	0	1	0	7.53	7.46	8.24	7.13	7.62	0	0	0	0	0	0	0	9.57	9.63	9.68	9.79	9.82	9.63	9.28
1390152_at	Tmed7	1	0	0	0	1	0	6.70	6.83	7.01	6.27	6.70	0	0	0	0	0	0	0	9.31	9.22	9.30	9.35	9.27	9.17	7.82
1390159_at	Rasgrp3	1	0	0	1	1	1	7.20	7.00	7.47	6.64	6.46	0	0	0	0	0	0	0	4.59	4.46	4.22	4.33	4.28	4.28	4.22
1390161_at	Cyyr1	1	0	0	1	1	0	7.31	7.08	7.65	6.58	7.17	0	0	0	0	0	0	0	3.30	3.33	3.36	3.39	3.33	3.46	2.85
1390162_at	Iqcb1	1	0	1	0	0	0	4.98	4.98	5.58	5.10	4.97	0	0	0	0	0	0	0	7.69	7.77	7.76	7.87	7.77	7.53	7.21
1390165_at	C2cd2	1	0	0	0	1	0	6.65	6.48	6.21	6.87	6.44	0	0	0	0	0	0	0	6.81	6.87	6.89	6.82	6.78	6.97	7.34
1390168_a_at	Dnajc24	1	0	1	0	1	1	7.12	7.17	7.84	7.01	7.70	0	0	0	0	0	0	0	9.80	9.85	9.70	9.61	9.70	9.50	9.38
1390191_at	Hunk	1	0	0	0	0	1	5.13	5.28	5.58	5.29	4.60	0	0	0	0	0	0	0	8.19	8.19	8.20	8.00	8.01	7.62	7.39
1390207_at	Lats2	1	0	0	1	1	0	7.39	7.46	7.79	6.89	7.76	0	0	0	0	0	0	0	8.83	8.82	8.89	8.78	8.80	8.65	8.22
1390221_at	Prdm1	1	0	0	1	0	0	5.86	5.66	5.77	5.37	5.89	0	0	0	0	0	0	0	5.20	5.16	5.01	4.93	4.99	5.09	4.77
1390231_at	---	1	0	0	0	0	1	6.36	6.18	6.73	6.35	5.79	0	0	0	0	0	0	0	9.09	9.04	9.13	9.10	9.12	9.23	9.53
1390234_at	Sf3b1	1	0	1	0	1	0	9.18	9.12	9.93	9.23	9.42	0	0	0	0	0	0	0	11.38	11.43	11.39	11.39	11.43	11.38	10.82

Probeset ID	Gene Symbol	Islet Data Set (log2 ratio ≥0.263; FDR <20%) union n=5325 1=YES 0=NO	Islet DRlyp/lyp ND vs ND+Lp299v (log2 ratio ≥0.263; FDR<20%) n=429 1=YES 0=NO	Islet DRlyp/lyp ND vs HCD (log2 ratio ≥0.263; FDR<20%) n=2247 1=YES 0=NO	Islet DRlyp/lyp ND vs HCD+Lp299v (log2 ratio ≥0.263; FDR<20%) n=1595 1=YES 0=NO	Islet DRlyp/lyp HCD vs HCD+Lp299v (log2 ratio ≥0.263; FDR<20%) n=3026 1=YES 0=NO	Islet DRlyp/lyp ND vs Flyp/lyp ND (log2 ratio ≥ 0.263; FDR<20%) n=1950 1=YES 0=NO	Islet Mean Log2 Intensity DRlyp/lyp ND	Islet Mean Log2 Intensity DRlyp/lyp ND+Lp299v	Islet Mean Log2 Intensity DRlyp/lyp HCD	Islet Mean Log2 Intensity DRlyp/lyp HCD+Lp299v	Islet Mean Log2 Intensity Flyp40 ND	Rinm5f data union of all conditions n=3705 1=YES 0=NO	Rinm5f 30 µM propionate/ 6 µM butyrate vs Media (FDR<20%; 1.5 fold) n=3 1=YES 0=NO	Rinm5f 60 µM propionate/ 12 µM butyrate vs Media (FDR<20%; 1.5 fold) n=18 1=YES 0=NO	Rinm5f 120 µM propionate/ 24 µM butyrate vs Media (FDR<20%; 1.5 fold) n=164 1=YES 0=NO	Rinm5f 240 µM propionate /48 µM butyrate vs Media (FDR<20%; 1.5 fold) n=268 1=YES 0=NO	Rinm5f 1500 µM propionate/ 300 µM butyrate vs Media (FDR<20%; 1.5 fold) n=2491 1=YES 0=NO	Rinm5f 500 µM propionate/ 500 µM butyrate vs Media (FDR<20%; 1.5 fold) n=3001 1=YES 0=NO	Rinm5f Mean Log2 Intensity Media only	Rinm5f Mean Log2 Intensity 30 µM propionate/ 6 µM butyrate	Rinm5f Mean Log2 Intensity 60 µM propionate/ 12 µM butyrate	Rinm5f Mean Log2 Intensity 120 µM propionate/ 24 µM butyrate	Rinm5f Mean Log2 Intensity 240 µM propionate/ 48 µM butyrate	Rinm5f Mean Log2 Intensity 1500 µM propionate /300 µM butyrate	Rinm5f Mean Log2 Intensity 500 µM propionate/ 500 µM butyrate
1390239_at	LOC100910996	1	0	0	1	1	0	5.23	5.22	5.40	4.62	4.87	0	0	0	0	0	0	0	6.31	6.23	6.31	6.34	6.31	6.07	5.94
1390240_at	---	1	0	0	1	1	1	7.00	6.71	6.72	7.78	6.52	0	0	0	0	0	0	0	7.25	7.22	7.26	7.26	7.25	7.34	7.64
1390241_at	Gigyf1	1	0	0	0	1	0	6.11	6.30	5.74	6.35	6.18	0	0	0	0	0	0	0	6.45	6.62	6.62	6.60	6.45	6.41	5.99
1390249_at	C15orf39	1	0	0	0	1	0	7.27	7.01	6.77	7.66	6.95	0	0	0	0	0	0	0	7.72	7.77	7.95	8.00	8.04	8.05	8.41
1390250_x_at	Atp8b2	1	0	0	0	1	1	5.92	5.97	6.30	5.48	6.44	0	0	0	0	0	0	0	7.19	7.19	7.31	7.26	7.27	7.36	7.29
1390259_at	Ube2d1	1	0	1	0	0	1	5.94	6.16	6.65	6.22	6.46	0	0	0	0	0	0	0	8.82	8.81	8.94	8.92	8.98	9.00	9.10
1390261_at	Smg5	1	0	0	0	1	0	5.97	6.12	5.61	6.23	5.85	0	0	0	0	0	0	0	7.21	7.24	7.27	7.30	7.19	7.10	7.12
1390263_at	Gnas	1	0	0	0	0	1	7.86	8.00	8.34	7.75	9.02	0	0	0	0	0	0	0	9.08	9.36	9.55	9.39	9.65	9.41	8.72
1390266_at	---	1	0	0	0	0	1	8.28	8.48	8.14	8.60	8.86	0	0	0	0	0	0	0	9.64	9.63	9.66	9.64	9.67	9.56	9.67
1390267_at	Fars2	1	0	0	0	1	0	5.98	6.14	5.71	6.34	6.00	0	0	0	0	0	0	0	8.81	9.02	8.89	8.88	8.97	8.75	9.16
1390276_at	---	1	0	0	0	0	1	4.55	4.65	4.70	4.71	5.04	0	0	0	0	0	0	0	7.01	7.15	7.23	7.24	7.24	7.30	7.06
1390278_at	Tra2a	1	0	0	0	1	0	7.13	6.92	7.58	6.88	7.23	0	0	0	0	0	0	0	9.67	9.72	9.76	9.81	9.82	9.54	8.48
1390284_at	Ccdc77	1	0	0	0	0	1	6.69	6.75	6.85	6.79	5.95	0	0	0	0	0	0	0	7.30	7.37	7.41	7.32	7.23	7.13	7.23
1390292_at	Pgap6	1	0	0	0	1	0	8.36	8.05	7.86	8.55	8.12	0	0	0	0	0	0	0	8.12	8.21	8.20	8.16	8.20	8.27	8.03
1390293_at	---	1	0	0	0	1	0	3.98	4.06	3.50	4.06	3.90	0	0	0	0	0	0	0	3.93	3.88	3.90	3.86	3.98	4.00	3.68
1390301_at	---	1	0	1	0	0	0	5.52	5.59	6.07	5.43	5.93	0	0	0	0	0	0	0	7.43	7.47	7.50	7.54	7.51	7.74	7.32
1390303_at	---	1	0	0	1	0	0	5.73	5.86	6.29	6.47	6.11	0	0	0	0	0	0	0	5.17	5.20	5.23	5.32	5.30	5.21	5.08
1390315_a_at	Zswim8	1	0	0	0	1	0	4.63	4.56	4.31	4.93	5.00	0	0	0	0	0	0	0	7.17	7.19	7.31	7.23	7.18	6.91	6.92
1390326_at	Ang	1	0	0	1	1	0	9.79	9.40	10.08	10.98	10.06	0	0	0	0	0	0	0	3.92	3.96	3.99	3.96	3.98	4.04	4.02
1390341_at	Ttbk2	1	0	1	0	1	0	6.40	6.55	7.14	6.32	6.82	0	0	0	0	0	0	0	9.00	9.07	9.04	9.03	9.11	9.06	8.94
1390347_at	---	1	0	0	0	1	0	5.40	5.41	5.03	5.64	5.10	0	0	0	0	0	0	0	5.12	5.10	5.07	4.86	5.00	4.90	5.08
1390350_at	Smap2	1	0	1	0	1	0	6.57	6.97	7.19	6.37	6.78	0	0	0	0	0	0	0	7.07	6.98	7.26	7.33	7.29	7.29	6.77
1390351_at	---	1	0	0	1	1	0	6.32	6.39	6.84	5.74	6.66	0	0	0	0	0	0	0	7.92	7.91	7.95	7.88	7.91	8.17	7.46
1390364_at	Runx1	1	0	0	0	1	0	4.63	4.63	4.21	4.79	4.45	0	0	0	0	0	0	0	6.93	6.94	6.96	6.79	6.73	6.78	6.61
1390373_at	Smad5	1	0	1	0	0	0	5.49	5.44	6.20	5.64	5.47	0	0	0	0	0	0	0	7.74	7.69	7.82	7.84	7.86	7.77	7.36
1390374_at	Fgfrl1	1	0	1	0	0	0	8.61	8.38	7.88	8.39	8.23	0	0	0	0	0	0	0	7.04	6.99	6.86	6.71	6.76	6.89	6.79
1390379_at	Lmo4	1	0	1	0	0	0	6.43	6.07	5.73	6.28	6.14	0	0	0	0	0	0	0	6.63	6.58	6.54	6.56	6.44	6.74	6.40
1390380_at	Ski	1	0	0	1	1	0	8.59	8.68	8.35	9.13	8.41	0	0	0	0	0	0	0	7.91	7.97	8.01	7.98	8.02	8.16	8.20
1390396_at	Eif4e3	1	0	0	1	0	0	5.40	5.44	5.48	4.89	5.42	0	0	0	0	0	0	0	8.77	8.73	8.65	8.51	8.53	8.65	8.43
1390403_at	Fam43a	1	0	0	0	1	0	6.75	6.45	7.23	6.49	7.14	0	0	0	0	0	0	0	7.87	7.84	7.85	7.90	7.98	8.02	8.08
1390404_at	Lama2	1	0	0	1	0	0	5.94	5.50	5.91	5.27	5.55	0	0	0	0	0	0	0	4.55	4.46	4.62	4.26	4.40	4.57	3.91
1390406_at	Arhgap18	1	0	0	0	0	1	8.37	8.30	8.08	8.44	7.91	0	0	0	0	0	0	0	8.61	8.66	8.58	8.49	8.56	8.70	8.88
1390419_a_at	Tusc3	1	0	1	0	1	1	9.26	9.43	10.15	9.03	9.86	0	0	0	0	0	0	0	12.21	12.28	12.21	12.20	12.20	12.10	11.68
1390422_at	Pxk	1	0	1	0	1	0	6.65	6.73	7.38	6.62	7.10	0	0	0	0	0	0	0	8.06	8.01	8.02	8.10	8.07	8.14	8.14
1390425_at	LOC100911224 /	1	0	1	0	1	0	4.55	4.54	5.27	4.54	4.91	0	0	0	0	0	0	0	6.60	6.70	6.88	7.07	7.00	6.97	6.11
1390427_at	Dzip3	1	0	1	0	1	1	5.84	5.93	6.55	5.71	6.61	0	0	0	0	0	0	0	7.86	7.86	8.01	8.04	8.06	7.97	7.62
1390430_at	Nr1d2	1	0	0	1	1	1	8.61	8.32	8.89	7.36	7.46	0	0	0	0	0	0	0	9.96	9.97	10.01	10.02	9.99	9.78	9.63
1390432_at	Tmco3	1	0	1	0	1	0	8.56	8.45	9.18	8.27	8.69	0	0	0	0	0	0	0	11.46	11.44	11.46	11.33	11.31	11.03	10.68
1390435_at	Smim4	1	0	0	0	1	1	7.99	7.99	7.52	8.29	7.28	0	0	0	0	0	0	0	8.25	8.28	8.19	8.21	8.16	8.37	8.41
1390453_at	Ldlrad3 /// LOC1	1	0	1	0	0	0	6.82	7.00	7.53	7.07	7.04	0	0	0	0	0	0	0	7.32	7.22	7.37	7.32	7.33	7.52	8.04
1390454_at	Nipsnap1	1	0	1	0	1	1	6.24	6.45	7.12	6.05	6.82	0	0	0	0	0	0	0	9.31	9.33	9.38	9.45	9.42	9.39	9.23
1390458_at	---	1	0	0	0	0	1	5.22	5.54	5.78	5.21	5.91	0	0	0	0	0	0	0	6.22	6.22	6.39	6.69	6.70	6.29	4.77
1390459_at	Adgrg6	1	0	0	1	1	0	4.27	4.26	4.02	4.82	4.37	0	0	0	0	0	0	0	4.30	4.24	4.34	4.20	4.15	4.20	3.74
1390482_at	Tmod2	1	0	1	0	1	1	4.77	5.02	5.50	4.66	5.76	0	0	0	0	0	0	0	5.96	6.17	6.15	6.17	6.05	5.98	6.15
1390489_at	Syncrip	1	0	1	0	0	0	6.96	6.96	7.65	7.03	7.24	0	0	0	0	0	0	0	8.40	8.50	8.54	8.71	8.69	8.36	8.15
1390491_at	Coq9	1	0	0	1	0	0	4.97	5.32	5.23	5.52	5.24	0	0	0	0	0	0	0	5.24	5.21	5.13	5.35	5.07	5.29	4.92
1390501_at	Scrn3	1	0	1	0	1	0	6.92	6.96	7.54	6.73	7.19	0	0	0	0	0	0	0	7.90	7.87	8.07	8.11	8.09	8.06	7.73
1390515_at	---	1	0	0	1	1	0	5.16	4.85	5.05	5.65	4.84	0	0	0	0	0	0	0	6.61	6.58	6.72	6.82	6.62	6.70	6.64
1390526_at	Klhl9	1	0	0	0	1	0	9.47	9.47	9.99	9.22	9.83	0	0	0	0	0	0	0	10.22	10.26	10.17	10.22	10.20	10.09	9.65
1390534_at	Smc5	1	0	1	0	1	0	6.68	6.64	7.49	6.58	7.09	0	0	0	0	0	0	0	9.80	9.93	9.99	10.12	10.11	10.22	9.71
1390543_at	---	1	0	1	0	1	1	8.54	8.28	7.86	8.78	7.91	0	0	0	0	0	0	0	6.56	6.56	6.56	6.48	6.56	6.64	6.76
1390546_at	LOC103693421	1	0	0	1	1	0	7.58	7.40	7.21	8.18	7.18	0	0	0	0	0	0	0	7.29	7.38	7.25	7.13	7.27	7.19	6.76
1390555_at	Socs5	1	0	0	0	1	0	6.89	6.96	7.41	6.54	6.86	0	0	0	0	0	0	0	8.45	8.40	8.47	8.44	8.40	8.38	8.24
1390558_at	Stau2	1	0	1	0	1	0	7.30	7.34	7.95	7.19	7.72	0	0	0	0	0	0	0	8.89	8.95	9.10	9.10	9.13	9.05	8.91
1390571_at	Gpr4	1	0	1	0	0	0	5.79	5.65	5.25	5.66	5.63	0	0	0	0	0	0	0	5.37	5.51	5.29	5.22	5.19	5.45	5.57
1390580_at	---	1	0	1	0	0	0	5.23	5.23	4.60	4.87	4.89	0	0	0	0	0	0	0	5.34	5.32	5.25	5.14	5.16	5.30	4.92
1390592_at	Znf106	1	0	0	1	1	0	7.14	7.34	7.25	7.83	7.38	0	0	0	0	0	0	0	7.89	7.66	7.86	7.66	7.71	7.64	8.26
1390615_at	---	1	0	0	0	0	1	4.90	4.76	4.81	4.87	5.40	0	0	0	0	0	0	0	6.97	7.06	7.23	7.18	7.17	7.26	7.28
1390642_at	---	1	0	0	0	0	1	4.60	4.50	4.57	4.67	4.06	0	0	0	0	0	0	0	4.98	5.14	5.05	5.04	5.17	5.19	4.97
1390645_at	---	1	0	1	1	1	1	5.15	5.42	6.03	4.61	5.70	0	0	0	0	0	0	0	6.60	6.44	6.70	6.73	6.76	7.05	6.79
1390648_at	Herc2	1	0	1	0	0	1	7.30	7.52	7.92	7.32	7.81	0	0	0	0	0	0	0	9.20	9.28	9.31	9.36	9.37	9.28	9.05
1390650_at	Nup85	1	1	0	0	0	0	6.30	5.82	6.07	6.24	5.92	0	0	0	0	0	0	0	9.08	9.03	9.15	9.20	9.06	8.73	8.29
1390655_at	Peg3	1	1	0	0	0	1	6.67	7.45	6.82	7.00	7.54	0	0	0	0	0	0	0	6.61	6.39	6.68	6.75	6.62	6.45	7.29
1390661_at	---	1	0	0	0	1	0	5.41	5.32	5.85	5.15	5.36	0	0	0	0	0	0	0	7.86	7.89	8.00	7.98	8.01	7.87	7.52
1390681_at	Tgif2	1	0	0	0	1	0	5.12	5.10	4.71	5.39	4.96	0	0	0	0	0	0	0	4.53	4.67	4.53	4.54	4.55	4.75	4.75
1390692_at	Ctps1	1	0	0	0	0	1	6.99	6.99	7.23	7.10	7.55	0	0	0	0	0	0	0	9.54	9.59	9.58	9.69	9.70	9.53	9.07
1390698_at	Mapk1ip1l	1	0	0	0	0	1	5.86	5.77	6.36	5.79	6.41	0	0	0	0	0	0	0	9.33	9.41	9.34	9.36	9.41	9.50	8.92
1390699_at	Kiaa2026	1	0	1	0	1	0	7.62	7.71	8.33	7.57	7.93	0	0	0	0	0	0	0	9.79	9.78	9.82	9.82	9.84	9.57	9.13
1390706_at	Sptbn1	1	1	0	0	1	0	8.86	9.37	8.68	9.24	8.97	0	0	0	0	0	0	0	9.22	9.14	9.21	9.11	9.08	8.98	8.92
1390719_at	Lcor	1	0	1	0	0	0	6.74	6.76	7.48	7.12	6.87	0	0	0	0	0	0	0	9.38	9.45	9.47	9.53	9.57	9.75	9.44
1390722_at	---	1	0	1	1	1	0	7.92	7.85	8.79	6.85	7.98	0	0	0	0	0	0	0	7.86	7.64	7.91	8.11	8.00	7.39	7.03
1390733_at	---	1	0	1	0	0	1	6.44	6.51	5.91	6.33	5.52	0	0	0	0	0	0	0	6.15	6.24	6.41	6.34	6.31	6.44	6.46
1390735_at	---	1	0	1	0	0	1	5.30	5.34	5.93	5.59	5.94	0	0	0	0	0	0	0	6.69	6.67	6.81	6.58	6.66	6.74	5.74
1390739_at	Znf609	1	0	0	0	1	0	6.26	6.38	5.93	6.60	6.21	0	0	0	0	0	0	0	6.43	6.46	6.41	6.28	6.20	6.34	6.78
1390755_at	Sergef	1	0	0	0	1	1	7.38	7.37	6.88	7.68	6.86	0	0	0	0	0	0	0	9.24	9.26	9.24	9.16	9.17	9.25	9.46
1390757_at	Cdk11a	1	1	1	0	0	1	4.94	5.32	5.57	5.27	5.54	0	0	0	0	0	0	0	6.46	6.45	6.57	6.64	6.54	6.24	4.79
1390766_at	Tuba8	1	0	1	0	1	0	6.22	6.23	5.30	6.18	5.78	0	0	0	0	0	0	0	4.92	4.96	4.88	4.79	4.78	4.99	5.15
1390767_at	Ssr1	1	0	0	0	1	1	10.22	10.14	9.85	10.52	9.35	0	0	0	0	0	0	0	10.71	10.67	10.68	10.74	10.69	10.31	9.54
1390782_at	Clec14a	1	0	0	1	1	0	5.57	5.43	5.96	4.91	5.57	0	0	0	0	0	0	0	3.10	3.04	3.09	3.07	3.10	3.09	2.66
1390783_at	Abca8a	1	1	0	1	1	1	6.50	5.87	7.07	5.76	5.83	0	0	0	0	0	0	0	3.79	3.76	3.80	3.63	3.73	3.81	3.24
1390789_at	Acad11	1	0	0	0	0	1	6.15	6.02	6.46	6.21	6.93	0	0	0	0	0	0	0	5.64	5.72	5.78	5.59	5.63	5.72	5.42
1390795_at	LOC100909929 /	1	0	0	1	1	1	5.90	5.55	5.67	6.48	5.09	0	0	0	0	0	0	0	4.14	4.22	4.35	4.09	4.20	4.14	3.90
1390798_at	Ptprc	1	0	0	0	0	1	3.25	3.46	3.31	3.11	4.85	0	0	0	0	0	0	0	3.63	3.60	3.58	3.46	3.50	3.45	3.09
1390802_at	Plgrkt	1	0	0	0	1	0	7.19	6.97	7.68	6.95	7.51	0	0	0	0	0	0	0	10.55	10.61	10.69	10.82	10.79	10.83	10.53
1390805_at	Ccdc167	1	0	0	0	1	0	8.61	8.77	8.22	8.85	8.79	0	0	0	0	0	0	0	9.19	9.18	9.20	9.23	9.21	9.60	9.51
1390813_at	Msi2	1	0	0	0	0	1	5.69	5.71	6.02	5.66	6.59	0	0	0	0	0	0	0	8.68	8.55	8.48	8.35	8.34	8.22	8.30
1390820_at	Tut4	1	0	1	0	0	0	7.21	7.11	7.97	7.59	7.59	0	0	0	0	0	0	0	8.98	8.98	8.93	9.04	9.05	9.07	8.58
1390821_at	Pcdha4	1	0	0	0	0	1	7.09	7.26	7.59	7.05	7.66	0	0	0	0	0	0	0	9.69	9.75	9.82	9.81	9.80	9.47	9.17
1390839_at	Slc66a3	1	0	1	0	1	1	5.01	5.06	5.80	4.75	5.78	0	0	0	0	0	0	0	9.24	9.28	9.27	9.27	9.34	9.39	8.82
1390846_at	Col16a1	1	0	0	0	0	1	7.00	7.12	6.98	6.98	7.67	0	0	0	0	0	0	0	9.12	9.13	9.09	8.94	9.07	9.26	9.09
1390854_at	C2cd2l	1	0	0	0	0	1	7.04	7.25	7.32	7.07	7.67	0	0	0	0	0	0	0	8.20	8.20	8.27	8.11	8.14	8.15	7.94
1390856_at	Rfx3	1	0	1	0	0	1	5.83	5.92	6.78	6.32	6.50	0	0	0	0	0	0	0	7.62	7.72	7.78	7.94	7.90	7.95	7.87
1390864_at	---	1	0	1	0	0	0	6.70	6.72	7.35	6.73	6.96	0	0	0	0	0	0	0	8.87	8.79	8.93	9.04	9.00	8.92	8.71
1390874_at	Ireb2	1	0	0	0	1	0	7.15	7.00	7.70	6.98	7.33	0	0	0	0	0	0	0	8.63	8.63	8.75	8.81	8.76	8.67	8.69
1390880_at	Eif3l	1	0	0	1	1	0	8.17	7.79	8.62	7.60	7.83	0	0	0	0	0	0	0	11.44	11.45	11.51	11.57	11.50	11.54	10.69
1390883_at	Akap11	1	0	0	0	1	0	7.98	7.92	8.45	7.60	8.05	0	0	0	0	0	0	0	7.58	7.67	7.74	7.89	7.93	8.10	8.08
1390886_at	Ttc14	1	0	1	0	1	0	5.25	5.17	6.01	5.21	5.57	0	0	0	0	0	0	0	6.87	6.91	7.07	7.12	7.11	7.06	6.16
1390900_at	Arid3a	1	0	0	0	0	1	3.84	3.75	3.99	3.70	4.34	0	0	0	0	0	0	0	6.82	6.72	6.91	6.86	6.87	6.65	6.19
1390905_at	Mast4 /// NEWG	1	0	1	0	1	0	6.78	6.93	7.47	6.75	7.17	0	0	0	0	0	0	0	6.83	6.97	7.00	7.04	7.14	7.12	7.25
1390914_at	Fli1	1	0	0	1	0	0	4.28	4.01	4.29	3.78	4.15	0	0	0	0	0	0	0	3.63	3.70	3.78	3.76	3.80	4.05	3.72
1390916_at	---	1	0	0	1	1	0	7.06	7.21	6.99	7.68	7.31	0	0	0	0	0	0	0	6.52	6.67	6.84	6.95	6.98	6.78	6.74
1390918_at	Adprhl1 /// Grtp	1	0	0	0	1	0	6.00	5.63	5.50	6.13	5.58	0	0	0	0	0	0	0	8.32	8.46	8.52	8.64	8.52	8.60	8.63
1390923_a_at	Osbpl1a	1	1	0	1	0	1	7.58	7.03	7.37	6.87	7.07	0	0	0	0	0	0	0	7.35	7.29	7.52	7.59	7.46	7.02	6.57
1390939_at	---	1	0	0	0	0	1	5.51	5.32	5.49	5.37	5.00	0	0	0	0	0	0	0	5.90	5.73	5.98	5.95	5.82	5.42	4.86
1390943_at	Rsrp1	1	0	1	1	1	1	7.81	7.57	8.87	6.69	7.15	0	0	0	0	0	0	0	9.28	9.30	9.42	9.47	9.43	9.65	8.39
1390945_at	Znf292	1	0	1	0	1	0	5.65	5.91	6.45	5.73	6.08	0	0	0	0	0	0	0	8.19	8.29	8.31	8.27	8.23	8.06	8.04
1390954_at	Ppm1h	1	0	0	1	1	0	5.80	6.08	5.80	6.41	6.07	0	0	0	0	0	0	0	6.71	6.59	6.67	6.64	6.57	6.36	6.13
1390960_at	Azi2	1	0	0	0	0	1	6.83	6.66	6.89	6.62	6.25	0	0	0	0	0	0	0	7.11	7.05	7.12	7.20	7.10	6.89	6.79
1390965_at	Nr5a2	1	0	0	1	1	0	8.57	8.31	8.06	9.15	8.19	0	0	0	0	0	0	0	4.48	4.54	4.51	4.18	4.23	4.46	4.23
1390977_at	St7l	1	0	0	0	1	0	6.59	6.45	6.96	6.21	6.63	0	0	0	0	0	0	0	9.47	9.45	9.48	9.58	9.58	9.35	8.78
1390979_at	LOC102546354 /	1	0	0	0	0	1	3.99	4.07	4.17	4.04	5.54	0	0	0	0	0	0	0	7.10	7.08	7.23	7.22	7.14	7.10	6.80
1390983_at	---	1	0	0	0	1	0	4.98	4.69	4.52	5.11	4.84	0	0	0	0	0	0	0	4.04	4.06	4.06	4.04	3.92	4.04	3.76
1390988_at	H2al2a	1	0	1	0	1	0	4.92	4.84	4.34	5.00	4.61	0	0	0	0	0	0	0	4.36	4.43	4.42	4.39	4.31	4.12	4.04
1390989_at	Mospd2	1	0	1	0	1	0	7.09	7.07	7.73	6.93	7.36	0	0	0	0	0	0	0	8.58	8.57	8.68	8.80	8.73	9.01	8.60
1390997_at	Idua	1	0	0	0	0	1	7.58	7.42	7.70	7.59	7.07	0	0	0	0	0	0	0	7.72	7.70	7.89	7.82	7.79	7.43	7.04
1391009_a_at	---	1	0	1	0	0	0	4.88	4.84	4.29	4.70	4.74	0	0	0	0	0	0	0	5.10	5.13	4.92	4.90	4.99	5.03	4.66
1391018_at	Myo5c	1	0	0	1	0	1	5.85	5.82	5.95	6.44	3.90	0	0	0	0	0	0	0	3.67	3.61	3.59	3.58	3.53	3.63	3.21
1391023_at	---	1	0	0	1	1	0	7.38	6.96	7.60	6.70	7.23	0	0	0	0	0	0	0	10.40	10.57	10.46	10.41	10.64	10.85	10.64
1391031_at	---	1	0	0	1	1	0	5.97	6.01	5.66	6.50	5.87	0	0	0	0	0	0	0	6.69	6.71	6.78	6.63	6.64	6.66	6.79
1391039_at	---	1	0	1	0	1	1	6.35	6.39	7.03	6.04	6.98	0	0	0	0	0	0	0	9.81	9.92	9.91	9.96	9.97	9.79	9.24
1391079_at	Cux1	1	0	0	1	0	1	5.97	6.35	6.28	6.52	6.50	0	0	0	0	0	0	0	7.19	7.15	7.21	7.01	7.02	6.95	6.85
1391101_at	Arhgap12	1	0	0	0	1	0	5.67	5.51	6.19	5.41	5.53	0	0	0	0	0	0	0	5.93	6.01	6.19	6.26	6.16	6.11	6.02
1391117_at	Pnma8b	1	0	0	0	0	1	6.20	6.52	6.61	6.34	6.74	0	0	0	0	0	0	0	6.54	6.48	6.64	6.65	6.53	6.65	7.26
1391123_at	---	1	0	0	0	0	1	4.04	3.90	3.75	4.04	3.54	0	0	0	0	0	0	0	3.55	3.56	3.59	3.67	3.59	3.74	3.44
1391128_at	Bcl9l	1	0	0	0	0	1	7.92	7.75	7.52	7.98	7.33	0	0	0	0	0	0	0	7.39	7.40	7.38	7.35	7.29	7.41	7.17
1391136_at	Klk1	1	0	0	0	1	0	3.64	3.56	3.17	3.75	3.40	0	0	0	0	0	0	0	3.52	3.49	3.57	3.48	3.52	3.58	3.09
1391143_at	---	1	0	0	0	0	1	4.99	5.09	5.18	5.02	4.42	0	0	0	0	0	0	0	6.45	6.35	6.56	6.35	6.36	6.27	6.27
1391145_at	Usp9x	1	0	0	0	0	1	6.59	6.50	6.68	6.67	7.06	0	0	0	0	0	0	0	6.66	6.72	6.64	6.78	6.82	6.49	6.32
1391152_at	LOC302192	1	0	1	0	1	0	5.95	5.93	5.16	6.20	5.93	0	0	0	0	0	0	0	7.01	6.92	6.95	6.98	6.98	6.90	7.07
1391154_at	---	1	1	1	1	0	1	4.94	4.10	4.35	4.08	4.07	0	0	0	0	0	0	0	7.09	7.15	7.12	7.11	7.19	7.30	7.07
1391183_at	Gopc	1	0	0	0	1	0	6.26	6.02	6.73	5.91	6.45	0	0	0	0	0	0	0	9.13	9.15	9.19	9.14	9.21	9.38	8.96
1391190_at	Tnrc6a	1	0	1	0	1	0	6.10	5.91	6.72	6.00	6.27	0	0	0	0	0	0	0	8.45	8.51	8.66	8.69	8.78	8.58	8.09
1391210_at	---	1	0	0	0	0	1	5.61	5.95	5.99	5.96	6.51	0	0	0	0	0	0	0	6.57	6.43	6.51	6.57	6.52	6.62	6.63
1391212_at	Tceal1	1	0	1	0	1	1	6.44	6.39	7.41	6.09	7.30	0	0	0	0	0	0	0	8.75	8.84	8.94	9.03	9.08	8.88	8.45
1391221_at	Tmem67	1	0	0	0	1	0	6.38	6.34	6.95	6.07	6.44	0	0	0	0	0	0	0	7.90	7.96	8.08	8.08	8.11	7.95	7.66

Probeset ID	Gene Symbol	Islet Data Set (log2 ratio ≥0.263; FDR <20%) union n=5325 1=YES 0=NO	Islet DRlyp/lyp ND vs ND+Lp299v (log2 ratio ≥0.263; FDR<20%) n=429 1=YES 0=NO	Islet DRlyp/lyp ND vs HCD (log2 ratio ≥0.263; FDR<20%) n=2247 1=YES 0=NO	Islet DRlyp/lyp ND vs HCD+Lp299v (log2 ratio ≥0.263; FDR<20%) n=1595 1=YES 0=NO	Islet DRlyp/lyp HCD vs HCD+Lp299v (log2 ratio ≥0.263; FDR<20%) n=3026 1=YES 0=NO	Islet DRlyp/lyp ND vs Flyp/lyp ND (log2 ratio ≥ 0.263; FDR<20%) n=1950 1=YES 0=NO	Islet Mean Log2 Intensity DRlyp/lyp ND	Islet Mean Log2 Intensity DRlyp/lyp ND+Lp299v	Islet Mean Log2 Intensity DRlyp/lyp HCD	Islet Mean Log2 Intensity DRlyp/lyp HCD+Lp299v	Islet Mean Log2 Intensity Flyp40 ND	Rinm5f data union of all conditions n=3705 1=YES 0=NO	Rinm5f 30 µM propionate/ 6 µM butyrate vs Media (FDR<20%; 1.5 fold) n=3 1=YES 0=NO	Rinm5f 60 µM propionate/ 12 µM butyrate vs Media (FDR<20%; 1.5 fold) n=18 1=YES 0=NO	Rinm5f 120 µM propionate/ 24 µM butyrate vs Media (FDR<20%; 1.5 fold) n=164 1=YES 0=NO	Rinm5f 240 µM propionate /48 µM butyrate vs Media (FDR<20%; 1.5 fold) n=268 1=YES 0=NO	Rinm5f 1500 µM propionate/ 300 µM butyrate vs Media (FDR<20%; 1.5 fold) n=2491 1=YES 0=NO	Rinm5f 500 µM propionate/ 500 µM butyrate vs Media (FDR<20%; 1.5 fold) n=3001 1=YES 0=NO	Rinm5f Mean Log2 Intensity Media only	Rinm5f Mean Log2 Intensity 30 µM propionate/ 6 µM butyrate	Rinm5f Mean Log2 Intensity 60 µM propionate/ 12 µM butyrate	Rinm5f Mean Log2 Intensity 120 µM propionate/ 24 µM butyrate	Rinm5f Mean Log2 Intensity 240 µM propionate/ 48 µM butyrate	Rinm5f Mean Log2 Intensity 1500 µM propionate /300 µM butyrate	Rinm5f Mean Log2 Intensity 500 µM propionate/ 500 µM butyrate
1391222_at	N4bp1	1	0	0	1	0	0	6.97	7.30	7.43	7.47	7.35	0	0	0	0	0	0	0	7.19	7.07	7.34	7.39	7.31	7.42	7.61
1391236_at	Uba6	1	1	0	0	0	0	6.30	6.87	6.41	6.44	6.63	0	0	0	0	0	0	0	7.75	7.67	7.75	7.86	7.74	7.72	7.54
1391238_at	---	1	0	0	0	0	1	4.63	4.82	4.56	4.88	5.22	0	0	0	0	0	0	0	4.75	4.85	4.76	4.72	4.71	4.75	4.70
1391241_at	Etv1	1	0	0	1	1	0	6.00	6.07	6.36	5.47	6.11	0	0	0	0	0	0	0	11.12	11.28	11.20	11.15	11.41	11.48	10.78
1391256_at	---	1	0	1	0	1	0	5.16	5.40	6.21	5.20	5.30	0	0	0	0	0	0	0	8.19	7.91	8.25	8.37	8.29	8.66	7.67
1391262_at	Senp5	1	0	0	0	0	1	4.06	4.01	4.30	3.84	7.10	0	0	0	0	0	0	0	9.57	9.65	9.65	9.65	9.68	9.52	9.17
1391266_at	---	1	0	1	0	0	0	5.38	5.33	4.84	5.38	5.34	0	0	0	0	0	0	0	4.52	4.47	4.24	4.21	4.26	4.33	3.89
1391269_at	Pim2	1	0	0	1	0	0	7.35	7.27	7.36	6.73	7.77	0	0	0	0	0	0	0	7.79	7.78	7.75	7.60	7.69	7.60	7.61
1391280_at	Mcts2	1	0	0	0	1	0	4.97	4.86	5.55	4.75	5.34	0	0	0	0	0	0	0	9.06	9.08	9.12	9.33	9.29	9.45	9.42
1391287_at	---	1	0	1	0	0	0	6.48	6.31	5.95	6.24	6.20	0	0	0	0	0	0	0	6.67	6.65	6.48	6.37	6.39	6.45	5.72
1391315_at	Fnip2	1	0	0	1	1	0	5.34	5.55	5.30	5.91	5.63	0	0	0	0	0	0	0	5.58	5.45	5.59	5.41	5.38	5.51	5.78
1391327_at	LOC102557439 /	1	0	1	0	1	0	5.31	5.21	4.66	5.28	4.88	0	0	0	0	0	0	0	7.80	7.79	7.86	7.64	7.52	7.44	7.33
1391387_s_at	Slbp	1	0	1	1	0	1	4.92	5.05	5.64	5.43	5.44	0	0	0	0	0	0	0	9.56	9.64	9.37	9.55	9.76	9.92	8.85
1391399_at	LOC100911077 /	1	0	1	0	1	1	4.79	4.72	4.23	5.07	4.13	0	0	0	0	0	0	0	3.68	3.92	3.75	3.63	3.63	3.78	3.63
1391403_at	---	1	0	0	0	1	0	5.00	4.97	4.65	5.24	4.81	0	0	0	0	0	0	0	3.69	3.74	3.69	3.47	3.69	3.68	3.44
1391410_at	---	1	0	1	0	1	1	6.88	6.90	7.56	6.73	7.38	0	0	0	0	0	0	0	8.26	8.45	8.43	8.48	8.53	8.46	8.05
1391421_at	Acad8	1	0	0	0	0	1	7.81	7.57	7.31	7.86	7.29	0	0	0	0	0	0	0	7.57	7.67	7.81	7.87	7.87	7.53	6.86
1391431_at	---	1	0	0	1	0	0	6.06	5.94	6.63	6.54	6.47	0	0	0	0	0	0	0	7.72	7.77	7.81	7.79	7.82	7.91	8.05
1391432_at	Wdr75	1	0	1	0	1	1	6.89	6.85	7.60	6.89	7.55	0	0	0	0	0	0	0	8.53	8.59	8.66	8.70	8.67	8.38	8.07
1391435_at	Pltp	1	0	1	0	0	0	4.44	4.18	3.81	4.09	4.00	0	0	0	0	0	0	0	4.61	4.68	4.51	4.44	4.44	4.62	4.00
1391441_at	---	1	0	0	1	1	0	5.91	5.67	6.13	5.27	5.54	0	0	0	0	0	0	0	6.59	6.47	6.63	6.55	6.46	6.27	6.11
1391442_at	Ehd3	1	0	0	0	1	0	6.17	6.22	5.67	6.39	5.79	0	0	0	0	0	0	0	7.50	7.49	7.63	7.74	7.60	7.51	7.40
1391444_at	---	1	0	1	1	1	0	6.70	6.79	7.52	6.16	6.90	0	0	0	0	0	0	0	8.03	8.11	8.23	8.36	8.41	8.43	7.97
1391446_at	Ms4a1	1	0	0	0	0	1	4.15	4.26	3.90	4.17	5.77	0	0	0	0	0	0	0	4.53	4.40	4.49	4.39	4.31	4.39	3.63
1391447_at	Runx1t1	1	0	0	0	0	1	3.81	3.94	3.93	4.16	5.11	0	0	0	0	0	0	0	6.77	6.62	6.76	6.65	6.40	6.27	6.49
1391454_at	Crebzf	1	0	1	0	0	1	5.16	5.34	5.92	5.43	5.70	0	0	0	0	0	0	0	8.29	8.19	8.28	8.46	8.45	8.02	7.67
1391456_at	Jam3	1	0	0	1	1	0	7.11	7.03	7.38	6.59	6.86	0	0	0	0	0	0	0	7.34	7.28	7.44	7.46	7.42	7.68	7.59
1391457_a_at	Irx2	1	0	0	1	1	0	5.74	5.47	5.85	5.10	5.61	0	0	0	0	0	0	0	7.92	7.90	7.99	8.07	8.00	7.46	7.59
1391458_at	Ndrg1	1	0	0	1	1	0	8.38	8.19	8.76	7.63	8.06	0	0	0	0	0	0	0	10.47	10.61	10.44	10.14	10.19	10.47	9.26
1391459_at	Nudt10 /// Nudt	1	0	0	0	1	0	7.47	7.41	8.02	7.14	7.75	0	0	0	0	0	0	0	10.33	10.31	10.47	10.69	10.50	10.33	10.16
1391475_at	Hnrnpll	1	0	1	0	1	1	6.30	6.21	7.06	6.24	6.82	0	0	0	0	0	0	0	9.68	9.72	9.73	9.78	9.84	9.62	9.22
1391476_at	---	1	0	1	1	1	0	5.75	5.61	6.55	5.16	5.51	0	0	0	0	0	0	0	7.93	7.78	7.90	7.78	7.76	7.79	6.86
1391491_a_at	Rad23b	1	0	0	1	1	0	5.83	5.71	6.19	5.31	5.70	0	0	0	0	0	0	0	8.71	8.69	8.82	8.72	8.73	8.22	7.84
1391492_at	---	1	0	0	0	1	0	4.79	4.84	4.29	5.00	4.64	0	0	0	0	0	0	0	5.21	5.13	5.15	5.02	5.17	5.45	4.96
1391497_at	Tbc1d12	1	0	1	0	0	0	6.47	6.31	7.10	6.47	6.62	0	0	0	0	0	0	0	9.71	9.72	9.80	9.84	9.80	9.72	9.52
1391501_at	Ift80	1	0	0	0	0	1	5.25	5.38	5.74	5.08	5.75	0	0	0	0	0	0	0	7.88	8.04	8.06	8.09	8.15	8.24	8.44
1391505_x_at	---	1	0	0	0	0	1	4.91	4.94	4.97	5.07	5.53	0	0	0	0	0	0	0	5.79	5.69	5.87	5.80	5.82	5.68	5.54
1391532_at	Rundc3a	1	0	1	0	0	0	9.24	9.29	8.53	8.96	8.93	0	0	0	0	0	0	0	9.78	9.87	9.84	9.74	9.69	9.53	9.15
1391534_at	Elovl2	1	0	1	0	0	0	4.16	4.52	4.88	4.39	4.53	0	0	0	0	0	0	0	7.75	7.84	7.92	7.94	8.08	8.10	8.45
1391542_at	---	1	0	0	1	1	1	5.11	4.99	4.70	5.93	4.38	0	0	0	0	0	0	0	3.44	3.44	3.57	3.44	3.48	3.58	2.95
1391552_at	---	1	0	0	1	1	1	5.49	5.58	5.95	4.92	6.09	0	0	0	0	0	0	0	9.39	9.22	9.41	9.44	9.46	9.41	8.52
1391553_at	---	1	0	0	1	0	0	5.35	5.73	5.63	5.95	5.69	0	0	0	0	0	0	0	7.70	7.66	7.80	7.86	7.81	7.33	6.69
1391559_at	Tlcd1	1	0	1	0	0	0	5.52	5.86	6.08	5.73	5.91	0	0	0	0	0	0	0	7.75	7.71	7.70	7.71	7.63	7.70	7.41
1391560_at	Hivep1	1	0	0	0	0	1	5.19	5.19	5.37	5.43	5.70	0	0	0	0	0	0	0	7.69	7.71	7.70	7.63	7.64	7.24	7.74
1391566_at	Senp8	1	0	0	0	0	1	5.00	5.13	5.39	5.10	5.50	0	0	0	0	0	0	0	6.39	6.35	6.44	6.40	6.24	6.43	6.16
1391575_at	Hapln4	1	0	0	0	0	1	4.16	4.31	4.20	4.32	4.97	0	0	0	0	0	0	0	9.27	9.27	9.39	9.42	9.52	9.83	9.61
1391580_at	---	1	0	1	0	1	0	5.11	5.05	5.69	4.74	5.40	0	0	0	0	0	0	0	7.29	7.36	7.52	7.65	7.65	7.80	7.25
1391584_at	Tsn	1	0	0	1	1	0	7.19	7.05	7.69	6.67	7.06	0	0	0	0	0	0	0	10.59	10.54	10.67	10.67	10.64	10.55	9.78
1391585_at	Harbi1	1	0	0	0	0	1	5.73	5.71	5.85	5.71	6.43	0	0	0	0	0	0	0	6.57	6.53	6.68	6.75	6.59	6.70	6.62
1391587_at	---	1	0	0	0	0	1	3.44	3.55	3.74	3.48	4.55	0	0	0	0	0	0	0	4.20	4.33	4.32	4.33	4.28	4.33	4.10
1391589_at	Card6	1	0	0	1	0	0	4.75	4.87	5.10	5.27	4.59	0	0	0	0	0	0	0	4.96	5.04	5.16	5.20	5.36	5.47	5.31
1391591_at	Znf275	1	0	1	0	1	0	7.88	7.94	8.49	7.67	8.23	0	0	0	0	0	0	0	9.25	9.22	9.32	9.30	9.31	9.02	8.89
1391599_at	---	1	0	0	1	1	0	4.94	4.89	5.45	4.30	4.60	0	0	0	0	0	0	0	7.24	7.14	7.21	7.42	7.34	7.15	6.12
1391600_at	MGA	1	0	0	0	1	0	7.44	7.32	7.26	7.89	7.01	0	0	0	0	0	0	0	5.79	5.70	5.84	5.78	5.73	5.76	5.77
1391612_at	Il22ra2	1	0	0	0	0	1	3.13	3.14	3.04	3.16	3.78	0	0	0	0	0	0	0	3.64	3.71	3.69	3.69	3.73	3.69	3.07
1391618_at	Kifap3	1	0	0	0	0	1	7.47	7.86	7.88	7.58	7.95	0	0	0	0	0	0	0	8.21	8.18	8.23	8.13	8.05	7.85	7.20
1391625_at	Wasl	1	0	0	1	1	0	8.43	8.60	8.30	8.96	8.65	0	0	0	0	0	0	0	10.21	10.06	10.19	10.19	10.12	10.06	9.98
1391627_at	---	1	0	1	0	0	0	5.83	5.70	5.21	5.61	5.62	0	0	0	0	0	0	0	5.11	5.17	5.04	5.06	5.16	5.05	4.63
1391628_at	Ss18	1	0	1	1	1	0	6.60	6.55	7.53	5.84	6.65	0	0	0	0	0	0	0	9.54	9.56	9.65	9.65	9.60	9.48	8.59
1391635_at	Ctdspl	1	0	0	1	1	1	8.24	8.23	8.14	8.85	6.27	0	0	0	0	0	0	0	5.95	5.88	6.01	5.82	5.73	5.81	5.89
1391640_at	---	1	0	1	0	1	0	5.06	5.09	4.48	5.11	4.93	0	0	0	0	0	0	0	7.83	7.81	7.80	7.80	7.75	8.01	8.47
1391643_at	---	1	0	0	0	1	0	7.62	7.93	7.40	8.02	7.47	0	0	0	0	0	0	0	8.94	8.92	8.91	8.84	8.86	9.07	9.06
1391663_at	---	1	0	0	1	1	0	6.35	6.02	6.65	5.80	6.33	0	0	0	0	0	0	0	7.78	7.77	7.79	7.88	7.87	7.71	7.07
1391676_at	---	1	0	1	0	0	1	4.15	4.12	4.83	4.25	4.81	0	0	0	0	0	0	0	7.57	7.57	7.66	7.79	7.91	7.96	7.41
1391684_at	Tmem14a	1	0	0	0	1	0	5.55	5.46	5.98	5.15	5.84	0	0	0	0	0	0	0	9.78	9.75	9.89	9.93	9.90	10.01	9.76
1391693_at	Atp8b1	1	0	1	0	1	0	8.84	8.90	9.39	8.59	9.02	0	0	0	0	0	0	0	11.21	10.95	11.22	11.31	11.20	11.26	11.15
1391699_at	Polr2f	1	0	1	0	0	0	5.00	4.80	4.47	4.63	4.58	0	0	0	0	0	0	0	6.86	6.81	6.93	6.95	6.90	6.84	6.68
1391701_at	Kat6a	1	0	0	1	1	0	6.94	7.18	6.71	7.42	6.99	0	0	0	0	0	0	0	8.02	7.86	7.97	8.02	7.86	7.75	8.24
1391712_at	Il22ra1	1	0	1	0	1	0	6.37	6.22	5.57	6.44	6.06	0	0	0	0	0	0	0	4.52	4.61	4.55	4.42	4.58	4.45	4.57
1391717_at	LOC103691481	1	0	0	0	1	1	6.53	6.31	6.03	6.60	5.70	0	0	0	0	0	0	0	4.11	4.05	4.04	4.03	3.93	3.94	3.64
1391726_at	---	1	1	1	1	1	1	6.71	6.12	4.15	5.08	5.15	0	0	0	0	0	0	0	4.57	4.55	4.59	4.65	4.49	4.54	4.45
1391752_at	---	1	0	0	0	1	1	5.37	5.54	5.89	5.19	6.32	0	0	0	0	0	0	0	5.18	5.38	5.50	5.46	5.57	5.60	4.96
1391757_at	Ptpn4	1	0	0	0	0	1	4.85	4.66	4.87	5.17	5.39	0	0	0	0	0	0	0	5.03	4.92	5.21	5.15	5.06	4.94	5.12
1391759_at	Sh3glb1	1	0	1	1	1	0	4.87	4.54	5.53	4.09	4.76	0	0	0	0	0	0	0	7.20	7.15	7.31	7.42	7.33	7.10	6.23
1391774_at	---	1	0	1	1	1	0	5.18	5.19	5.96	4.51	5.60	0	0	0	0	0	0	0	9.46	9.54	9.56	9.57	9.72	9.76	9.40
1391776_at	Cmc1	1	0	0	0	0	1	7.56	7.36	7.54	7.83	6.19	0	0	0	0	0	0	0	8.97	9.01	8.90	8.88	8.94	9.02	8.85
1391805_at	Klhl32	1	1	1	0	1	1	7.03	7.58	7.66	6.96	7.79	0	0	0	0	0	0	0	7.35	7.39	7.47	7.33	7.34	7.41	7.12
1391822_at	---	1	0	0	0	0	1	4.66	4.65	4.54	4.63	5.74	0	0	0	0	0	0	0	5.95	5.92	6.04	5.61	5.64	5.64	5.50
1391828_at	Prdm10	1	0	1	0	0	0	4.45	4.72	5.06	4.75	4.69	0	0	0	0	0	0	0	7.14	7.13	7.19	7.25	7.19	6.83	6.22
1391838_at	Ankrd11	1	0	0	1	1	0	5.60	5.53	5.56	6.25	5.61	0	0	0	0	0	0	0	6.93	6.80	7.04	6.85	6.83	6.66	7.24
1391840_at	Rsph9	1	0	1	0	0	0	7.54	7.40	6.97	7.31	7.08	0	0	0	0	0	0	0	8.10	8.15	8.16	8.10	8.07	8.57	8.55
1391857_at	Mslnl	1	0	0	0	0	1	7.11	7.22	7.03	6.77	6.55	0	0	0	0	0	0	0	4.28	4.26	4.23	4.16	4.17	4.28	3.88
1391866_at	Npat	1	0	1	0	1	0	5.12	5.23	5.93	5.15	5.37	0	0	0	0	0	0	0	6.78	6.70	6.83	6.87	6.85	6.97	6.42
1391878_at	Myb	1	0	0	1	1	0	3.55	3.67	3.49	4.22	3.39	0	0	0	0	0	0	0	6.61	6.43	6.48	6.66	6.59	6.68	7.06
1391885_at	---	1	0	0	0	0	1	6.87	6.68	7.34	7.14	7.44	0	0	0	0	0	0	0	8.21	8.27	8.41	8.46	8.51	8.65	8.43
1391899_at	Purb	1	0	1	1	1	0	5.15	5.07	5.84	4.60	5.28	0	0	0	0	0	0	0	8.28	8.09	8.22	8.35	8.31	8.26	7.97
1391906_at	Kif1b	1	0	0	0	1	0	8.17	8.05	7.94	8.50	8.16	0	0	0	0	0	0	0	9.63	9.58	9.74	9.72	9.82	9.81	9.56
1391907_at	Nags	1	0	1	0	0	1	5.18	5.05	4.61	5.01	4.70	0	0	0	0	0	0	0	5.22	5.14	5.23	5.01	5.04	5.11	4.49
1391923_at	Loc100911216 /	1	0	1	0	1	1	10.35	10.45	11.27	9.96	10.91	0	0	0	0	0	0	0	9.40	9.52	9.64	9.91	9.90	9.81	8.46
1391925_at	Ccl19	1	0	0	0	0	1	5.52	5.71	5.13	5.30	6.15	0	0	0	0	0	0	0	5.19	5.05	5.10	4.81	4.97	5.07	4.82
1391928_at	Gopc	1	0	1	1	1	0	7.26	7.38	7.88	6.62	7.68	0	0	0	0	0	0	0	10.14	10.18	10.24	10.25	10.22	10.27	9.88
1391935_at	Eif4e3	1	0	0	1	0	0	5.56	5.48	5.45	5.08	5.27	0	0	0	0	0	0	0	8.28	8.20	8.19	7.98	7.97	8.01	8.16
1391944_at	Fam184a	1	0	0	0	1	0	8.34	8.34	8.72	8.00	8.55	0	0	0	0	0	0	0	8.41	8.41	8.60	8.56	8.53	8.88	8.86
1391952_at	---	1	0	1	0	1	1	3.75	3.92	4.40	3.68	4.28	0	0	0	0	0	0	0	5.68	5.83	5.90	6.07	6.13	6.07	5.25
1391981_at	---	1	0	1	0	1	1	6.38	6.58	7.26	6.05	7.08	0	0	0	0	0	0	0	7.54	7.42	7.30	7.26	7.13	7.12	7.29
1392012_at	Eppk1	1	0	1	1	0	0	5.13	4.93	4.13	4.55	4.90	0	0	0	0	0	0	0	3.86	3.91	3.87	3.79	3.80	3.81	3.71
1392013_at	Prex2	1	0	0	1	1	0	5.73	5.43	6.19	5.00	5.31	0	0	0	0	0	0	0	6.61	6.70	6.71	6.87	6.90	6.85	6.46
1392015_at	---	1	0	0	1	1	0	4.01	4.27	4.06	4.65	4.31	0	0	0	0	0	0	0	4.50	4.47	4.54	4.43	4.50	4.46	4.58
1392040_at	Sass6	1	0	0	0	0	1	4.89	4.95	5.42	4.89	5.84	0	0	0	0	0	0	0	6.79	6.71	6.78	6.81	6.59	6.23	6.15
1392044_at	ZBTB20	1	0	1	0	1	0	7.28	7.14	7.99	7.24	7.53	0	0	0	0	0	0	0	6.77	6.95	6.98	7.15	7.12	7.06	5.91
1392054_at	Pbrm1	1	0	1	0	0	0	6.22	6.50	6.88	6.39	6.60	0	0	0	0	0	0	0	9.14	9.04	9.19	9.20	9.10	8.96	8.78
1392085_at	Rfx7	1	0	1	0	1	0	4.27	4.40	4.89	4.00	4.69	0	0	0	0	0	0	0	4.96	4.76	4.84	4.78	4.68	4.60	4.25
1392119_at	---	1	0	0	0	0	1	7.48	7.56	7.67	7.35	7.97	0	0	0	0	0	0	0	9.58	9.66	9.68	9.71	9.86	9.99	9.57
1392166_at	---	1	1	1	1	1	0	7.31	8.08	8.77	6.70	7.68	0	0	0	0	0	0	0	3.16	3.14	3.35	3.17	3.17	3.29	2.86
1392170_at	Rwdd3	1	0	1	0	1	0	4.83	4.88	5.44	4.54	4.86	0	0	0	0	0	0	0	5.58	5.54	5.77	5.59	5.66	5.46	5.42
1392180_at	Sp1	1	0	0	1	0	0	7.36	7.39	7.35	7.83	7.76	0	0	0	0	0	0	0	8.46	8.34	8.44	8.40	8.36	8.25	8.35
1392186_at	Dgkd	1	0	0	0	1	0	7.16	7.18	6.89	7.48	6.76	0	0	0	0	0	0	0	6.80	6.83	6.87	6.79	6.75	6.73	6.31
1392192_at	---	1	0	1	0	1	1	4.66	4.60	4.12	4.69	4.13	0	0	0	0	0	0	0	5.69	5.68	5.82	5.99	5.79	6.12	4.87
1392211_at	Sec24b	1	0	0	0	0	1	3.76	3.90	4.31	3.91	4.33	0	0	0	0	0	0	0	7.46	7.58	7.69	7.80	7.83	7.74	6.66
1392221_at	Greb1l	1	0	1	1	1	0	8.18	8.32	7.59	8.70	7.86	0	0	0	0	0	0	0	5.41	5.36	5.51	5.76	5.71	5.63	4.90
1392257_at	Mis12	1	0	1	1	1	1	5.65	5.77	6.38	5.09	6.15	0	0	0	0	0	0	0	7.91	7.94	8.01	8.08	8.02	7.97	7.17
1392259_at	Hectd1	1	0	1	1	0	0	4.50	4.89	4.94	4.92	4.81	0	0	0	0	0	0	0	6.92	6.56	6.98	6.90	6.83	6.34	5.44
1392269_at	Sin3a	1	0	0	1	1	0	4.99	5.30	5.14	5.77	5.23	0	0	0	0	0	0	0	5.55	5.59	5.71	5.74	5.67	5.86	5.98
1392274_at	Pdgfc	1	0	0	1	0	0	4.79	4.82	4.99	5.49	4.71	0	0	0	0	0	0	0	4.65	4.67	5.02	5.20	5.22	5.19	5.29
1392289_at	Tmem87a	1	0	1	1	1	0	7.81	7.84	8.43	7.29	7.87	0	0	0	0	0	0	0	9.57	9.63	9.69	9.75	9.71	9.59	8.75
1392298_at	Acap3	1	0	0	0	1	0	6.78	6.79	6.46	7.04	6.54	0	0	0	0	0	0	0	6.63	6.65	6.71	6.57	6.61	6.59	6.38
1392317_at	---	1	0	1	0	0	0	4.18	4.33	4.90	4.46	4.58	0	0	0	0	0	0	0	5.35	5.40	5.51	5.68	5.67	5.51	5.73
1392332_at	Myt1	1	0	1	0	0	0	6.77	7.12	7.36	6.84	6.98	0	0	0	0	0	0	0	9.59	9.69	9.78	9.87	9.83	9.57	9.01
1392350_at	---	1	0	1	0	0	0	4.58	4.29	4.02	4.31	4.15	0	0	0	0	0	0	0	5.06	4.96	5.09	4.95	4.98	4.98	4.59
1392351_at	Fpgt	1	0	1	1	1	0	4.65	4.57	5.30	4.11	4.65	0	0	0	0	0	0	0	7.17	7.20	7.31	7.38	7.25	7.08	6.24
1392392_at	---	1	0	0	0	0	1	3.99	3.90	3.86	3.85	6.01	0	0	0	0	0	0	0	6.45	6.55	6.59	6.49	6.47	6.36	6.35
1392394_at	Vash2	1	0	1	0	1	0	7.06	7.23	6.48	7.05	6.96	0	0	0	0	0	0	0	6.75	6.73	6.52	6.43	6.52	6.47	5.89
1392411_at	Tinag	1	0	1	1	1	0	6.32	6.35	5.56	7.31	6.22	0	0	0	0	0	0	0	3.09	3.19	3.21	3.13	3.06	3.09	2.65
1392412_at	---	1	0	0	1	0	0	4.91	5.33	5.00	5.41	5.29	0	0	0	0	0	0	0	4.66	4.59	4.68	4.57	4.57	4.41	4.09
1392437_at	Alg2	1	0	1	1	1	0	5.95	5.95	6.57	4.97	5.89	0	0	0	0	0	0	0	7.75	7.87	7.96	8.13	8.00	7.87	6.22
1392446_at	Klhl2	1	0	1	0	0	1	5.08	4.91	5.77	5.31	5.78	0	0	0	0	0	0	0	9.87	9.91	9.83	9.90	9.99	9.98	9.21
1392453_at	Clcn3	1	0	0	1	1	0	8.38	8.52	8.50	9.10	8.26	0	0	0	0	0	0	0	9.72	9.69	9.79	9.76	9.75	9.86	9.73
1392462_at	Crk	1	0	0	0	1	0	7.99	7.79	8.46	7.73	8.00	0	0	0	0	0	0	0	10.58	10.49	10.59	10.68	10.63	10.73	10.49
1392463_at	Map7d2	1	0	1	0	1	1	4.78	4.95	5.63	4.57	5.35	0	0	0	0	0	0	0	6.67	6.68	6.81	6.92	6.87	6.98	6.80
1392476_at	H4c8	1	0	0	1	1	0	6.02	6.18	5.66	6.74	5.95	0	0	0	0	0	0	0	4.33	4.39	4.34	4.26	4.30	4.38	3.77
1392492_at	Igf1r	1	0	0	0	0	1	7.16	7.20	7.59	7.14	7.88	0	0	0	0	0	0	0	8.75	8.75	8.91	8.97	8.97	9.04	8.59
1392496_at	LOC312273	1	1	1	1	0	1	10.56	9.66	11.53	11.85	13.60	0	0	0	0	0	0	0	3.90	3.87	3.79	3.66	3.73	3.95	3.08
1392497_at	S100pbp	1	0	1	0	1	0	7.92	7.98	8.56	7.83	8.03	0	0	0	0	0	0	0	10.04	10.05	10.04	10.02	10.02	9.92	9.62
1392506_at	Cryzl1	1	0	0	0	0	1	5.91	6.29	6.22	6.33	6.42	0	0	0	0	0	0	0	8.01	8.01	8.02	8.11	8.06	8.04	7.82
1392519_at	Nktr	1	0	0	1	0	0	7.61	8.01	7.83	8.09	7.75	0	0	0	0	0	0	0	7.65	7.60	7.75	7.57	7.59	7.64	7.77

Probeset ID	Gene Symbol	Islet Data Set (log2 ratio ≥0.263; FDR <20%) union n=5325 1=YES 0=NO	Islet DRlyp/lyp ND vs ND+Lp299v (log2 ratio ≥0.263; FDR<20%) n=429 1=YES 0=NO	Islet DRlyp/lyp ND vs HCD (log2 ratio ≥0.263; FDR<20%) n=2247 1=YES 0=NO	Islet DRlyp/lyp ND vs HCD+Lp299v (log2 ratio ≥0.263; FDR<20%) n=1595 1=YES 0=NO	Islet DRlyp/lyp HCD vs HCD+Lp299v (log2 ratio ≥0.263; FDR<20%) n=3026 1=YES 0=NO	Islet DRlyp/lyp ND vs Flyp/lyp ND (log2 ratio ≥ 0.263; FDR<20%) n=1950 1=YES 0=NO	Islet Mean Log2 Intensity DRlyp/lyp ND	Islet Mean Log2 Intensity DRlyp/lyp ND+Lp299v	Islet Mean Log2 Intensity DRlyp/lyp HCD	Islet Mean Log2 Intensity DRlyp/lyp HCD+Lp299v	Islet Mean Log2 Intensity Flyp40 ND	Rinm5f data union of all conditions n=3705 1=YES 0=NO	Rinm5f 30 µM propionate/ 6 µM butyrate vs Media (FDR<20%; 1.5 fold) n=3 1=YES 0=NO	Rinm5f 60 µM propionate/ 12 µM butyrate vs Media (FDR<20%; 1.5 fold) n=18 1=YES 0=NO	Rinm5f 120 µM propionate/ 24 µM butyrate vs Media (FDR<20%; 1.5 fold) n=164 1=YES 0=NO	Rinm5f 240 µM propionate /48 µM butyrate vs Media (FDR<20%; 1.5 fold) n=268 1=YES 0=NO	Rinm5f 1500 µM propionate/ 300 µM butyrate vs Media (FDR<20%; 1.5 fold) n=2491 1=YES 0=NO	Rinm5f 500 µM propionate/ 500 µM butyrate vs Media (FDR<20%; 1.5 fold) n=3001 1=YES 0=NO	Rinm5f Mean Log2 Intensity Media only	Rinm5f Mean Log2 Intensity 30 µM propionate/ 6 µM butyrate	Rinm5f Mean Log2 Intensity 60 µM propionate/ 12 µM butyrate	Rinm5f Mean Log2 Intensity 120 µM propionate/ 24 µM butyrate	Rinm5f Mean Log2 Intensity 240 µM propionate/ 48 µM butyrate	Rinm5f Mean Log2 Intensity 1500 µM propionate /300 µM butyrate	Rinm5f Mean Log2 Intensity 500 µM propionate/ 500 µM butyrate
1392530_at	Ndufc1	1	0	0	1	0	0	9.69	9.68	9.78	10.17	9.56	0	0	0	0	0	0	0	11.61	11.62	11.55	11.47	11.51	11.50	11.14
1392531_at	Ttpal	1	0	0	0	0	1	5.79	5.74	6.26	5.73	6.41	0	0	0	0	0	0	0	7.56	7.55	7.59	7.50	7.52	7.12	7.36
1392534_at	Pmepa1	1	0	0	1	1	0	7.47	7.38	7.34	8.43	7.38	0	0	0	0	0	0	0	8.13	8.21	8.35	8.16	8.25	8.18	8.28
1392537_at	Arhgef10l	1	0	0	0	1	0	5.65	5.51	5.37	5.98	5.30	0	0	0	0	0	0	0	7.22	7.34	7.44	7.36	7.38	7.14	6.92
1392539_at	Blvrb	1	0	0	0	0	1	5.86	5.67	5.44	5.93	5.14	0	0	0	0	0	0	0	8.70	8.68	8.89	8.85	8.96	9.11	9.08
1392541_at	Ggct	1	0	0	1	1	0	7.36	7.31	6.89	7.84	7.43	0	0	0	0	0	0	0	8.30	8.36	8.30	8.25	8.30	8.53	8.76
1392542_at	Cdc42se2 /// LO	1	0	0	1	1	0	6.11	6.20	6.61	5.63	6.26	0	0	0	0	0	0	0	7.50	7.46	7.47	7.54	7.51	7.74	7.27
1392548_at	Cwc22	1	0	1	0	0	0	5.70	5.76	6.28	5.78	5.87	0	0	0	0	0	0	0	6.81	6.85	6.95	7.02	6.92	6.31	5.94
1392552_at	Gatad2b	1	0	0	1	1	0	6.34	6.65	6.13	6.82	6.34	0	0	0	0	0	0	0	6.86	6.76	6.78	6.76	6.83	6.70	6.85
1392557_at	Bicc1	1	0	1	0	0	0	7.36	7.33	7.94	7.35	7.68	0	0	0	0	0	0	0	8.25	8.35	8.52	8.53	8.54	8.50	8.77
1392561_at	Atp5c1 /// Taf3	1	0	0	0	1	0	6.06	6.29	5.90	6.49	6.09	0	0	0	0	0	0	0	7.80	7.79	7.85	7.68	7.67	7.56	7.91
1392569_at	Igf1	1	0	0	1	1	1	7.95	7.63	7.61	8.44	7.34	0	0	0	0	0	0	0	3.70	3.68	3.69	3.71	3.61	3.60	3.15
1392575_at	Znf654	1	0	1	0	0	1	5.52	5.58	6.31	5.97	5.99	0	0	0	0	0	0	0	8.07	8.26	8.24	8.27	8.27	8.17	7.72
1392586_at	Ap5m1	1	0	0	0	0	1	5.92	5.98	6.04	6.15	6.46	0	0	0	0	0	0	0	6.19	6.20	6.35	6.23	6.19	6.01	6.04
1392587_at	Pik3r1	1	1	0	0	0	0	8.23	7.62	8.05	8.03	8.12	0	0	0	0	0	0	0	8.66	8.64	8.67	8.68	8.68	8.80	8.72
1392590_at	Arhgap24	1	0	1	0	0	0	7.84	8.05	8.48	8.13	8.21	0	0	0	0	0	0	0	7.46	7.49	7.50	7.43	7.41	7.29	7.16
1392593_a_at	Prr12	1	1	0	0	0	0	4.96	5.44	5.16	5.37	5.34	0	0	0	0	0	0	0	5.93	5.86	6.10	6.00	6.08	5.53	5.53
1392633_at	Fbxo32	1	0	0	1	1	0	4.86	4.94	4.83	5.53	4.42	0	0	0	0	0	0	0	7.07	7.25	7.19	6.68	6.93	6.58	6.25
1392637_at	Mgc108823	1	1	0	0	0	0	3.40	5.76	3.06	3.11	3.18	0	0	0	0	0	0	0	3.58	3.58	3.56	3.62	3.66	3.58	3.16
1392639_at	Klhdc10	1	0	0	0	1	0	5.57	5.77	5.95	5.13	5.53	0	0	0	0	0	0	0	7.18	7.09	7.14	7.17	7.15	7.46	7.11
1392640_at	Cry1	1	1	1	1	1	1	5.44	6.35	6.15	7.29	6.79	0	0	0	0	0	0	0	8.34	8.25	8.32	8.16	8.20	7.86	7.88
1392645_at	Cbfa2t3	1	1	1	1	1	0	7.20	7.70	6.66	8.25	7.12	0	0	0	0	0	0	0	6.06	6.08	6.08	5.98	6.09	6.07	6.40
1392663_at	Fam204a	1	0	0	0	0	1	5.84	5.99	6.29	5.66	6.44	0	0	0	0	0	0	0	8.58	8.53	8.53	8.48	8.44	8.68	8.89
1392669_at	Rbm26	1	0	1	0	0	0	9.85	9.82	10.45	9.76	10.07	0	0	0	0	0	0	0	10.22	10.38	10.43	10.46	10.49	10.50	9.66
1392688_at	Rassf1	1	0	1	0	0	0	5.71	5.75	5.15	5.56	5.38	0	0	0	0	0	0	0	5.93	6.01	6.05	5.81	5.77	5.79	5.44
1392691_at	Lipt2	1	0	0	0	1	0	5.01	5.08	4.65	5.27	4.77	0	0	0	0	0	0	0	5.10	5.12	5.07	5.00	5.10	5.20	4.40
1392692_at	---	1	0	0	0	1	0	7.91	7.92	8.46	7.67	8.25	0	0	0	0	0	0	0	9.58	9.65	9.66	9.64	9.74	9.86	9.54
1392699_at	Znf346	1	0	0	0	0	1	4.51	4.89	4.75	4.72	4.99	0	0	0	0	0	0	0	5.23	4.99	5.36	5.20	5.32	5.49	5.50
1392711_at	---	1	0	0	1	1	0	7.04	6.79	7.11	6.06	7.37	0	0	0	0	0	0	0	4.66	4.47	4.67	4.59	4.64	4.73	4.60
1392719_at	Mthfs	1	0	0	0	1	0	7.64	7.66	8.12	7.41	7.62	0	0	0	0	0	0	0	8.54	8.51	8.50	8.49	8.49	8.33	7.91
1392727_at	Cep162	1	0	0	0	0	1	5.11	5.03	5.06	5.04	4.64	0	0	0	0	0	0	0	4.75	4.84	4.66	4.62	4.74	4.80	4.96
1392734_at	Nrcam	1	1	1	0	1	1	5.29	5.81	6.38	5.39	5.92	0	0	0	0	0	0	0	8.11	8.14	8.23	8.37	8.50	7.96	7.93
1392736_at	---	1	0	0	0	0	1	6.40	6.39	6.46	6.27	7.01	0	0	0	0	0	0	0	6.64	6.53	6.94	7.06	6.93	6.74	7.42
1392746_x_at	Larp1	1	0	1	0	1	0	8.52	8.40	7.93	8.71	8.27	0	0	0	0	0	0	0	8.19	8.19	8.34	8.41	8.37	8.38	7.47
1392761_at	---	1	0	1	0	1	0	6.17	6.34	6.91	5.98	6.52	0	0	0	0	0	0	0	7.70	7.84	7.93	7.83	7.88	7.90	7.92
1392789_at	Slc25a36	1	0	1	1	1	0	6.58	6.66	7.54	5.68	6.32	0	0	0	0	0	0	0	8.34	8.25	8.42	8.52	8.46	8.58	8.23
1392792_at	Taf1	1	0	1	0	1	1	5.82	5.98	6.59	5.75	6.43	0	0	0	0	0	0	0	8.18	8.20	8.32	8.35	8.38	7.97	7.62
1392798_at	Pdia2	1	0	1	0	1	1	12.11	11.92	11.48	12.04	11.55	0	0	0	0	0	0	0	3.81	3.94	3.89	3.78	3.90	3.73	3.65
1392813_at	Sox9	1	0	0	0	1	0	7.63	7.53	7.39	8.05	7.56	0	0	0	0	0	0	0	6.89	6.74	6.80	6.49	6.37	6.63	6.74
1392815_at	Arap2	1	0	1	0	1	0	6.42	6.37	7.17	6.29	6.80	0	0	0	0	0	0	0	7.72	7.54	7.73	7.78	7.71	7.41	7.01
1392825_at	RGD1559600	1	0	1	0	1	1	3.59	3.88	2.75	3.39	2.88	0	0	0	0	0	0	0	3.17	3.19	3.16	3.16	3.23	3.26	2.78
1392835_at	Zfp68	1	0	0	0	1	0	7.21	7.09	7.75	7.01	7.51	0	0	0	0	0	0	0	9.98	10.12	10.16	10.25	10.26	10.42	10.22
1392838_at	Washc4	1	0	0	1	0	0	6.94	7.29	7.29	7.46	7.13	0	0	0	0	0	0	0	7.63	7.54	7.73	7.78	7.74	7.61	7.48
1392858_at	Ncbp2	1	0	1	0	1	0	7.95	8.12	8.58	7.64	8.10	0	0	0	0	0	0	0	9.51	9.54	9.56	9.58	9.56	9.51	8.89
1392860_at	---	1	0	0	0	0	1	5.74	5.44	6.07	6.06	4.04	0	0	0	0	0	0	0	7.46	7.51	7.68	7.82	7.74	7.56	7.30
1392880_at	Rab37	1	0	0	0	0	1	4.40	4.01	4.56	3.98	3.69	0	0	0	0	0	0	0	9.42	9.43	9.45	9.45	9.41	9.02	8.87
1392883_at	Limch1	1	1	1	0	1	1	4.78	5.41	5.85	4.46	5.36	0	0	0	0	0	0	0	6.98	6.99	7.02	7.08	6.88	6.82	6.32
1392886_a_at	Fcrla	1	0	0	0	1	0	5.57	5.56	5.08	6.01	5.68	0	0	0	0	0	0	0	5.56	5.70	5.57	5.35	5.42	5.58	4.81
1392893_a_at	Nacc2	1	0	0	1	0	0	4.84	5.03	5.22	5.42	5.08	0	0	0	0	0	0	0	7.66	7.69	7.70	7.65	7.75	7.92	7.71
1392908_at	Akirin1	1	0	1	0	1	1	7.69	7.90	8.48	7.59	8.34	0	0	0	0	0	0	0	10.40	10.43	10.54	10.60	10.65	10.75	10.47
1392911_at	Bod1l1	1	0	1	0	0	0	6.60	6.63	7.22	6.58	6.88	0	0	0	0	0	0	0	9.20	9.32	9.34	9.42	9.46	9.52	9.14
1392920_at	---	1	0	0	0	0	1	5.79	6.17	5.94	5.85	6.37	0	0	0	0	0	0	0	7.47	7.51	7.55	7.70	7.79	7.78	7.28
1392929_at	C20orf194	1	0	1	0	1	0	7.60	7.52	8.24	7.43	7.98	0	0	0	0	0	0	0	8.41	8.36	8.42	8.49	8.42	8.20	7.98
1392932_at	Leng8	1	0	0	1	1	0	6.07	6.36	5.79	6.79	5.90	0	0	0	0	0	0	0	6.41	6.47	6.57	6.49	6.56	6.28	6.37
1392935_at	---	1	0	0	1	1	0	5.21	4.97	5.56	6.51	5.14	0	0	0	0	0	0	0	4.28	4.29	4.22	4.22	4.29	4.34	3.92
1392942_at	Tmem263	1	0	0	1	1	0	6.90	7.17	7.34	6.10	7.19	0	0	0	0	0	0	0	9.11	9.24	9.16	9.13	9.13	8.79	8.06
1392943_at	Mboat2	1	0	0	0	1	0	3.64	3.79	4.17	3.46	3.88	0	0	0	0	0	0	0	8.26	8.23	8.21	8.17	8.16	8.11	8.33
1392946_at	Il1r1	1	0	1	0	1	0	9.17	9.32	9.94	9.14	9.26	0	0	0	0	0	0	0	11.35	11.36	11.38	11.37	11.42	11.41	10.69
1392947_at	---	1	0	0	0	0	1	4.25	4.10	4.35	4.42	4.90	0	0	0	0	0	0	0	4.65	4.62	4.71	4.70	4.77	4.71	5.16
1392955_at	Sel1l	1	0	0	1	0	0	10.66	10.58	10.70	11.18	10.65	0	0	0	0	0	0	0	9.19	9.18	9.24	9.21	9.36	9.50	9.96
1392967_at	C18orf21	1	0	0	0	0	1	7.12	7.14	7.36	7.06	6.43	0	0	0	0	0	0	0	8.47	8.46	8.51	8.53	8.48	8.26	7.37
1392974_at	Wasf1	1	0	1	0	0	0	4.57	4.81	5.17	4.63	4.89	0	0	0	0	0	0	0	8.98	9.00	9.04	9.01	9.12	9.26	9.00
1392975_at	Pradc1	1	0	0	0	1	0	7.63	7.59	7.17	8.04	7.30	0	0	0	0	0	0	0	9.21	9.20	9.21	9.31	9.26	9.49	9.57
1392978_at	Slc25a28	1	0	0	0	0	1	8.36	8.27	8.03	8.39	7.73	0	0	0	0	0	0	0	9.11	9.04	9.14	9.12	9.03	9.05	8.94
1392980_at	Tiam1	1	0	0	0	0	1	5.67	5.66	6.13	5.47	6.20	0	0	0	0	0	0	0	7.16	7.07	7.07	7.09	7.15	7.27	7.24
1392986_at	Strn	1	0	1	0	1	0	5.26	5.51	6.15	5.45	5.53	0	0	0	0	0	0	0	9.00	8.99	9.12	9.23	9.19	8.98	8.53
1392990_at	Sox17	1	0	1	1	0	1	5.52	5.21	4.93	4.52	4.55	0	0	0	0	0	0	0	3.41	3.36	3.42	3.42	3.43	3.36	2.77
1392996_at	Cpeb1	1	1	1	0	0	1	5.85	6.53	6.43	6.21	6.89	0	0	0	0	0	0	0	9.48	9.52	9.50	9.50	9.48	9.25	9.24
1393000_at	Amph	1	0	1	0	1	1	6.80	7.02	7.71	6.47	7.42	0	0	0	0	0	0	0	8.02	7.96	8.01	8.23	8.13	8.26	8.09
1393003_at	Chst11	1	0	1	0	0	1	6.78	7.23	7.38	6.73	7.36	0	0	0	0	0	0	0	8.72	8.82	8.83	8.82	8.84	9.07	8.99
1393015_at	C1orf115	1	0	1	0	0	1	7.26	7.07	6.47	6.81	6.69	0	0	0	0	0	0	0	4.41	4.61	4.47	4.37	4.42	4.54	4.28
1393020_at	Tmem35b	1	0	0	0	0	1	5.83	5.86	6.19	5.69	5.16	0	0	0	0	0	0	0	6.06	6.15	6.12	6.04	6.02	6.04	6.69
1393021_at	Klhl15	1	0	1	0	1	1	5.62	5.53	6.39	5.62	6.13	0	0	0	0	0	0	0	6.52	6.80	6.79	6.76	6.89	6.94	6.18
1393030_at	---	1	0	0	0	0	1	3.65	3.71	3.57	3.64	4.32	0	0	0	0	0	0	0	5.69	5.68	5.82	5.65	5.62	5.73	5.32
1393037_at	Sh3bgrl2	1	0	0	0	0	1	7.45	7.54	7.56	7.42	8.15	0	0	0	0	0	0	0	7.67	7.85	7.74	7.72	7.72	7.96	8.05
1393043_at	Utp18	1	0	1	0	0	0	4.46	4.58	5.21	4.62	4.79	0	0	0	0	0	0	0	6.77	6.75	6.94	7.08	7.10	6.71	6.29
1393045_at	Rock2	1	0	0	0	1	0	5.10	5.14	5.43	4.70	5.02	0	0	0	0	0	0	0	7.77	7.63	7.92	8.01	7.86	8.00	7.82
1393049_at	Myo1g	1	0	1	0	0	0	5.17	4.89	4.56	4.91	4.84	0	0	0	0	0	0	0	5.18	5.25	5.18	5.02	5.04	5.17	4.93
1393051_at	Armcx1 /// LOC1	1	0	1	0	1	1	7.20	7.29	7.98	6.81	7.80	0	0	0	0	0	0	0	8.36	8.37	8.39	8.35	8.32	8.15	7.72
1393058_at	Eid2	1	0	0	1	1	0	6.98	6.89	7.54	6.50	7.27	0	0	0	0	0	0	0	9.07	9.09	9.16	9.18	9.13	8.86	8.60
1393067_at	Tek	1	0	0	0	0	1	7.11	6.98	6.86	6.76	6.61	0	0	0	0	0	0	0	3.67	3.69	3.56	3.49	3.60	3.68	3.68
1393070_at	Scrn2	1	0	0	0	0	1	6.29	6.05	5.86	6.41	5.72	0	0	0	0	0	0	0	6.14	6.19	6.18	6.21	6.28	6.20	6.10
1393072_at	Ube2q2	1	0	0	1	1	0	7.67	7.39	7.99	6.99	7.44	0	0	0	0	0	0	0	8.95	8.92	9.00	9.10	8.99	8.63	7.64
1393077_at	Abhd13	1	0	0	0	0	1	7.15	7.15	7.14	7.32	6.66	0	0	0	0	0	0	0	8.69	8.75	8.76	8.77	8.79	8.86	8.58
1393080_at	Zfr	1	0	1	0	1	0	6.80	7.23	7.58	6.76	7.15	0	0	0	0	0	0	0	8.88	8.89	9.01	9.16	9.16	9.00	8.50
1393085_at	Mitd1	1	0	1	0	0	0	4.85	5.07	5.46	4.94	5.09	0	0	0	0	0	0	0	7.09	7.00	7.15	7.18	7.12	7.09	6.72
1393110_at	Mpv17	1	0	0	0	0	1	7.02	7.21	7.49	7.18	7.49	0	0	0	0	0	0	0	8.98	9.01	9.04	8.88	8.93	8.94	8.79
1393113_at	Arhgap1	1	0	0	1	1	0	5.27	5.28	5.70	4.54	5.52	0	0	0	0	0	0	0	6.62	6.46	6.69	6.70	6.66	6.33	5.02
1393114_at	St6galnac4	1	0	0	0	0	1	7.11	7.26	7.05	7.39	8.32	0	0	0	0	0	0	0	8.44	8.43	8.46	8.43	8.45	8.41	8.49
1393119_at	---	1	0	1	0	1	0	5.05	5.03	5.74	4.90	5.49	0	0	0	0	0	0	0	10.46	10.51	10.52	10.59	10.69	10.62	10.51
1393120_at	---	1	0	1	0	0	1	5.33	5.59	5.96	5.58	6.12	0	0	0	0	0	0	0	9.14	9.12	9.13	9.23	9.06	8.66	8.42
1393135_at	---	1	0	1	0	1	0	4.49	4.18	5.11	4.37	4.63	0	0	0	0	0	0	0	4.80	4.67	4.84	4.75	4.88	4.69	4.35
1393138_at	Jund	1	0	0	0	1	0	10.87	11.16	10.55	11.21	10.54	0	0	0	0	0	0	0	11.83	11.78	11.89	11.83	11.77	11.79	11.32
1393147_at	Clca2	1	0	1	0	1	1	8.27	7.97	7.55	8.46	8.89	0	0	0	0	0	0	0	6.29	6.02	6.17	5.73	5.75	5.86	5.63
1393150_at	Klf3	1	0	0	1	1	0	6.71	6.62	6.61	7.27	6.68	0	0	0	0	0	0	0	6.44	6.39	6.46	6.33	6.30	6.32	7.04
1393159_at	---	1	0	1	0	0	0	5.57	5.66	6.18	5.54	5.77	0	0	0	0	0	0	0	7.94	7.97	8.02	8.12	8.12	8.38	8.06
1393161_at	Snapc3	1	0	1	0	1	0	6.30	6.37	7.04	6.26	6.61	0	0	0	0	0	0	0	8.46	8.57	8.57	8.63	8.57	8.28	7.91
1393162_at	Slc39a6	1	0	1	0	0	1	6.14	6.31	6.72	6.13	6.75	0	0	0	0	0	0	0	9.72	9.75	9.74	9.87	9.82	9.73	9.33
1393167_at	---	1	0	1	1	1	0	7.05	6.64	7.86	6.56	6.77	0	0	0	0	0	0	0	3.50	3.41	3.45	3.54	3.48	3.47	3.61
1393168_at	Mbtd1	1	0	1	0	0	0	4.58	4.41	5.10	4.91	4.81	0	0	0	0	0	0	0	6.91	7.03	7.10	7.12	7.14	7.15	6.48
1393173_at	Rasa1	1	0	1	0	0	0	5.97	5.81	6.61	6.07	6.43	0	0	0	0	0	0	0	8.70	8.69	8.74	8.83	8.80	8.41	8.14
1393179_at	Flt1	1	0	0	1	1	0	6.29	6.01	6.61	5.75	6.22	0	0	0	0	0	0	0	5.24	5.05	5.15	4.91	4.96	4.86	5.81
1393183_at	Stard5	1	0	1	0	1	0	7.67	7.81	8.43	7.25	8.03	0	0	0	0	0	0	0	9.18	9.20	9.21	9.26	9.23	9.39	9.38
1393185_at	LOC103691872 /	1	0	0	0	0	1	4.54	4.59	5.08	4.82	5.22	0	0	0	0	0	0	0	7.13	7.09	7.35	7.36	7.38	7.32	6.94
1393199_at	Kdm5c	1	0	1	0	1	0	5.14	5.32	5.73	4.95	5.37	0	0	0	0	0	0	0	6.88	6.89	7.10	7.09	7.00	6.45	6.01
1393201_at	Irag2	1	0	0	0	0	1	7.45	7.00	7.01	7.49	6.87	0	0	0	0	0	0	0	5.60	5.49	5.48	5.40	5.45	5.56	4.96
1393202_a_at	Igf2bp3	1	0	0	0	1	0	5.56	5.47	5.22	5.84	5.62	0	0	0	0	0	0	0	4.85	4.78	4.88	4.86	4.94	4.86	4.31
1393213_at	Aurkaip1 /// Ccr	1	0	0	1	1	0	6.80	6.92	7.21	6.03	6.70	0	0	0	0	0	0	0	7.66	7.62	7.70	7.71	7.69	7.45	5.91
1393220_at	---	1	0	0	0	1	0	4.20	4.43	4.73	3.89	4.63	0	0	0	0	0	0	0	6.23	6.37	6.42	6.56	6.70	6.56	6.41
1393237_at	Eif2s3y	1	1	0	0	0	0	6.50	4.88	6.71	6.37	6.41	0	0	0	0	0	0	0	9.98	10.19	10.09	10.18	10.34	10.34	9.69
1393247_at	Znf778	1	0	1	0	1	0	5.06	4.83	6.00	4.90	5.41	0	0	0	0	0	0	0	7.96	8.08	8.14	8.17	8.26	7.94	7.23
1393251_at	Arl10	1	0	0	0	0	1	6.47	6.90	6.88	6.59	6.96	0	0	0	0	0	0	0	8.06	8.10	8.29	8.40	8.42	8.50	8.19
1393265_at	Syncrip	1	0	1	0	1	0	3.74	3.69	4.50	3.45	3.82	0	0	0	0	0	0	0	4.98	4.92	5.14	5.37	5.11	4.69	3.54
1393267_at	Psip1	1	0	1	0	1	0	8.01	7.98	8.79	8.07	7.91	0	0	0	0	0	0	0	9.99	10.02	9.93	10.09	10.00	10.11	9.87
1393268_at	---	1	0	0	1	1	0	5.71	5.51	5.85	4.95	5.53	0	0	0	0	0	0	0	6.96	6.86	7.12	7.15	7.14	7.09	6.14
1393273_at	---	1	0	0	1	1	0	7.48	7.42	8.00	6.97	7.77	0	0	0	0	0	0	0	9.30	9.54	9.63	9.72	9.85	9.80	9.28
1393281_at	Cav1	1	0	0	1	1	1	7.01	6.67	6.92	5.77	6.22	0	0	0	0	0	0	0	3.88	3.89	3.91	3.74	3.73	4.04	3.88
1393291_at	Ube2ql1	1	0	0	0	0	1	4.76	4.83	5.18	4.81	5.36	0	0	0	0	0	0	0	8.33	8.31	8.36	8.25	8.34	8.40	8.19
1393297_at	Pou2af1	1	0	0	0	0	1	2.86	2.96	2.80	2.99	3.49	0	0	0	0	0	0	0	3.52	3.46	3.55	3.45	3.53	3.51	2.98
1393298_at	---	1	0	1	0	0	0	3.37	3.44	3.96	3.48	3.58	0	0	0	0	0	0	0	5.36	5.10	5.24	5.44	5.36	5.16	4.25
1393308_at	Ftx	1	0	1	0	1	0	5.89	6.01	6.58	5.85	6.36	0	0	0	0	0	0	0	5.92	5.97	6.30	6.35	6.39	6.14	5.66
1393309_at	Zfp599	1	0	1	0	0	0	6.19	6.22	6.77	6.28	6.37	0	0	0	0	0	0	0	9.24	9.29	9.27	9.22	9.26	9.11	8.79
1393325_at	Gtpbp6 /// LOC1	1	0	1	0	1	0	9.78	9.65	8.54	9.37	9.47	0	0	0	0	0	0	0	7.58	7.73	7.60	7.65	7.76	7.75	7.16
1393331_at	Heca	1	0	0	0	1	0	5.34	4.99	5.69	4.88	4.93	0	0	0	0	0	0	0	8.08	8.08	8.16	8.12	8.11	7.84	7.46
1393333_at	---	1	1	0	1	0	0	5.62	5.96	5.85	6.22	5.75	0	0	0	0	0	0	0	4.59	4.61	4.79	4.72	4.67	4.74	3.86
1393334_at	CDYL2	1	0	0	0	1	0	4.81	4.74	5.21	4.41	4.83	0	0	0	0	0	0	0	6.54	6.53	6.68	6.60	6.58	6.13	6.12
1393341_at	Avl9	1	0	0	0	1	0	9.26	9.15	9.82	9.10	9.41	0	0	0	0	0	0	0	10.70	10.74	10.75	10.74	10.77	10.76	10.47
1393343_at	Cul3	1	0	1	0	0	0	7.19	7.17	7.90	7.23	7.64	0	0	0	0	0	0	0	8.29	8.37	8.48	8.76	8.71	8.83	8.30
1393345_at	Asph	1	0	0	0	0	1	7.01	7.18	7.23	7.07	7.72	0	0	0	0	0	0	0	9.45	9.46	9.42	9.30	9.24	9.62	9.55
1393346_at	Ccdc125	1	0	1	0	0	0	7.48	7.63	8.27	7.90	7.80	0	0	0	0	0	0	0	3.82	3.76	4.08	4.20	4.23	4.34	4.23
1393351_at	Rdh10	1	0	1	0	0	0	5.94	6.05	6.63	6.14	6.41	0	0	0	0	0	0	0	8.16	8.28	8.36	8.41	8.45	8.48	7.73
1393359_at	Ap3b2	1	0	0	0	0	1	7.48	7.90	7.81	7.65	8.05	0	0	0	0	0	0	0	8.74	8.70	8.81	8.81	8.87	8.55	8.29
1393363_at	Zmym2	1	0	1	0	1	0	5.77	5.90	6.36	5.39	5.72	0	0	0	0	0	0	0	7.17	7.03	7.22	7.31	7.22	6.69	6.42
1393366_at	---	1	0	1	0	1	0	5.20	5.17	5.83	4.94	5.36	0	0	0	0	0	0	0	7.84	7.84	7.93	7.88	7.91	7.84	7.28
1393371_at	LOC102552284	1	0	1	0	1	0	4.67	4.97	5.36	4.65	5.11	0	0	0	0	0	0	0	5.29	5.37	5.42	5.49	5.55	5.37	4.82

Probeset ID	Gene Symbol	Islet Data Set (log2 ratio ≥0.263; FDR <20%) union n=5325 1=YES 0=NO	Islet DRlyp/lyp ND vs ND+Lp299v (log2 ratio ≥0.263; FDR<20%) n=429 1=YES 0=NO	Islet DRlyp/lyp ND vs HCD (log2 ratio ≥0.263; FDR<20%) n=2247 1=YES 0=NO	Islet DRlyp/lyp ND vs HCD+Lp299v (log2 ratio ≥0.263; FDR<20%) n=1595 1=YES 0=NO	Islet DRlyp/lyp HCD vs HCD+Lp299v (log2 ratio ≥0.263; FDR<20%) n=3026 1=YES 0=NO	Islet DRlyp/lyp ND vs Flyp/lyp ND (log2 ratio ≥ 0.263; FDR<20%) n=1950 1=YES 0=NO	Islet Mean Log2 Intensity DRlyp/lyp ND	Islet Mean Log2 Intensity DRlyp/lyp ND+Lp299v	Islet Mean Log2 Intensity DRlyp/lyp HCD	Islet Mean Log2 Intensity DRlyp/lyp HCD+Lp299v	Islet Mean Log2 Intensity Flyp40 ND	Rinm5f data union of all conditions n=3705 1=YES 0=NO	Rinm5f 30 µM propionate/6 µM butyrate vs Media (FDR<20%; 1.5 fold) n=3 1=YES 0=NO	Rinm5f 60 µM propionate/12 µM butyrate vs Media (FDR<20%; 1.5 fold) n=18 1=YES 0=NO	Rinm5f 120 µM propionate/24 µM butyrate vs Media (FDR<20%; 1.5 fold) n=164 1=YES 0=NO	Rinm5f 240 µM propionate/48 µM butyrate vs Media (FDR<20%; 1.5 fold) n=268 1=YES 0=NO	Rinm5f 1500 µM propionate/300 µM butyrate vs Media (FDR<20%; 1.5 fold) n=2491 1=YES 0=NO	Rinm5f 500 µM propionate/500 µM butyrate vs Media (FDR<20%; 1.5 fold) n=3001 1=YES 0=NO	Rinm5f Mean Log2 Intensity Media only	Rinm5f Mean Log2 Intensity 30 propionate/6 µM butyrate	Rinm5f Mean Log2 Intensity 60 µM propionate/12 µM butyrate	Rinm5f Mean Log2 Intensity 120 µM propionate/24 µM butyrate	Rinm5f Mean Log2 Intensity 240 µM propionate/48 µM butyrate	Rinm5f Mean Log2 Intensity 1500 µM propionate/300 µM butyrate	Rinm5f Mean Log2 Intensity 500 µM propionate/500 µM butyrate
1393382_at	RGD1566386	1	0	1	0	0	0	4.76	5.13	5.45	5.14	4.87	0	0	0	0	0	0	0	4.95	5.07	5.23	5.13	5.10	5.28	5.10
1393383_at	Arhgdig	1	0	1	1	1	1	11.00	11.02	10.20	11.50	10.30	0	0	0	0	0	0	0	8.50	8.62	8.58	8.51	8.55	8.24	7.98
1393391_at	Tgs1	1	0	1	0	0	0	6.28	6.17	6.87	6.36	6.36	0	0	0	0	0	0	0	8.51	8.51	8.62	8.72	8.74	8.64	8.13
1393399_at	Slc6a2	1	0	1	0	0	0	4.85	4.60	4.31	4.62	4.63	0	0	0	0	0	0	0	4.21	4.31	4.23	4.04	4.06	4.10	3.45
1393414_at	LOC686461 /// T	1	0	0	1	1	0	6.16	5.83	6.37	5.63	6.00	0	0	0	0	0	0	0	7.99	7.91	7.99	8.04	7.90	7.68	7.27
1393421_at	Pxmp4	1	0	0	0	0	1	7.03	6.86	7.05	6.94	5.64	0	0	0	0	0	0	0	8.67	8.66	8.77	8.69	8.72	8.95	8.68
1393423_at	Tnks2	1	0	0	1	1	0	6.60	6.54	7.01	5.90	6.38	0	0	0	0	0	0	0	9.16	9.00	9.12	9.16	9.19	9.15	8.94
1393425_at	Epb41l4b	1	0	0	0	0	1	4.57	4.98	4.92	4.60	5.29	0	0	0	0	0	0	0	5.20	5.20	5.21	5.16	5.25	5.48	5.66
1393427_s_at	Egfl7	1	0	0	1	0	0	6.49	6.25	6.30	5.98	6.05	0	0	0	0	0	0	0	8.51	8.65	8.71	8.84	8.86	9.08	8.45
1393436_at	Scgb1c1	1	0	0	0	1	0	6.49	6.28	5.98	6.94	6.32	0	0	0	0	0	0	0	3.83	3.89	3.81	3.89	3.80	3.94	4.08
1393438_at	Znf316	1	0	0	0	1	0	5.37	5.55	4.98	5.65	5.46	0	0	0	0	0	0	0	5.95	5.66	5.78	5.82	5.80	5.62	5.27
1393439_a_at	Ankh	1	0	0	0	0	1	8.26	8.52	8.36	8.27	8.94	0	0	0	0	0	0	0	8.76	8.86	8.92	8.93	8.98	9.25	9.54
1393450_at	---	1	0	1	0	0	0	6.30	6.26	7.03	6.57	6.76	0	0	0	0	0	0	0	8.59	8.61	8.68	8.78	8.74	8.76	7.87
1393459_at	Fmr1	1	0	1	0	1	0	6.87	6.85	7.70	6.89	7.26	0	0	0	0	0	0	0	9.13	9.26	9.15	9.28	9.33	9.12	8.47
1393465_at	Tarbp1	1	0	1	0	1	0	6.25	5.94	6.86	6.13	6.54	0	0	0	0	0	0	0	6.73	6.63	6.73	6.80	6.75	6.75	6.49
1393480_at	Ppp1r2	1	0	0	1	1	0	5.45	5.34	5.82	4.86	5.58	0	0	0	0	0	0	0	7.79	7.76	7.84	7.96	7.90	7.72	7.32
1393495_at	Ago4	1	0	0	0	0	1	5.30	5.41	5.53	5.30	5.83	0	0	0	0	0	0	0	7.10	7.10	7.21	7.07	7.03	6.68	6.32
1393499_at	Pik3ca	1	0	0	1	0	0	6.88	6.96	7.37	7.37	7.16	0	0	0	0	0	0	0	9.31	9.39	9.44	9.46	9.52	9.78	9.01
1393504_at	---	1	0	1	0	0	0	4.63	4.36	4.07	4.30	4.30	0	0	0	0	0	0	0	4.16	4.02	4.08	4.00	3.94	4.14	3.32
1393511_at	Gal3st4	1	0	1	0	1	0	5.07	5.07	4.51	5.08	5.02	0	0	0	0	0	0	0	4.22	4.25	4.08	4.02	4.08	4.06	3.69
1393516_at	Slc16a12	1	0	1	0	1	0	6.98	7.12	7.66	6.96	6.85	0	0	0	0	0	0	0	7.09	7.12	7.45	7.65	7.53	7.19	6.42
1393546_at	---	1	0	1	0	1	0	5.01	5.15	5.87	4.91	5.44	0	0	0	0	0	0	0	7.65	7.67	7.80	7.85	7.77	7.71	6.93
1393547_at	Rnf182	1	0	0	0	1	0	6.26	6.72	6.03	6.67	6.18	0	0	0	0	0	0	0	5.85	6.01	6.05	5.90	6.16	6.14	6.79
1393558_at	Itga6	1	0	0	1	1	0	5.98	6.12	5.85	6.52	6.07	0	0	0	0	0	0	0	4.33	4.25	4.30	4.18	4.12	4.59	4.68
1393559_at	Stc1	1	0	0	1	1	0	4.05	3.81	4.49	3.56	3.97	0	0	0	0	0	0	0	12.15	12.31	12.34	12.36	12.56	12.72	12.31
1393560_at	Bc024139	1	0	1	0	0	1	5.30	5.40	4.32	4.97	4.79	0	0	0	0	0	0	0	3.98	3.95	3.91	3.82	3.83	3.84	3.42
1393562_at	Ap3m1	1	0	0	0	0	1	6.23	6.47	6.42	6.20	6.86	0	0	0	0	0	0	0	8.44	8.53	8.44	8.54	8.55	8.32	8.00
1393563_at	Il1rap	1	0	0	1	1	0	5.51	5.38	5.54	6.28	5.21	0	0	0	0	0	0	0	6.48	6.39	6.40	6.34	6.33	6.48	6.62
1393566_at	Tmem120b	1	0	1	0	1	0	5.71	5.60	5.15	5.71	5.42	0	0	0	0	0	0	0	5.78	5.88	5.68	5.68	5.74	5.99	5.61
1393569_at	Rbm18	1	0	1	1	1	0	6.32	6.22	6.95	5.68	6.24	0	0	0	0	0	0	0	8.99	8.98	9.05	9.14	9.14	9.16	8.40
1393572_at	Znf592	1	0	1	0	1	1	5.96	6.18	6.81	6.11	6.46	0	0	0	0	0	0	0	8.04	8.10	8.12	8.26	8.30	8.27	7.90
1393576_at	---	1	0	0	0	1	0	4.99	4.82	4.59	5.22	4.59	0	0	0	0	0	0	0	4.43	4.39	4.41	4.21	4.31	4.49	4.81
1393595_at	Utp6	1	0	0	1	1	0	5.20	4.94	5.35	4.60	4.87	0	0	0	0	0	0	0	6.65	6.66	6.84	6.98	6.75	6.55	5.72
1393596_at	Atrx	1	0	0	1	0	1	5.12	5.11	5.50	5.59	5.40	0	0	0	0	0	0	0	6.20	6.10	6.40	6.60	6.44	5.96	5.11
1393613_at	Znf462	1	0	1	0	1	1	6.78	6.99	7.53	6.65	7.26	0	0	0	0	0	0	0	7.93	8.08	8.08	8.14	8.25	8.08	7.04
1393626_at	Sorl1	1	0	1	0	1	0	6.57	6.67	7.16	6.41	6.85	0	0	0	0	0	0	0	7.18	7.23	7.33	7.42	7.53	7.33	7.07
1393639_at	Myo10	1	0	0	0	1	0	6.57	6.80	6.24	6.83	6.64	0	0	0	0	0	0	0	6.60	6.58	6.62	6.51	6.38	6.45	6.42
1393652_at	Zbtb1	1	0	1	0	0	0	4.17	4.26	4.85	4.45	4.64	0	0	0	0	0	0	0	7.38	7.40	7.43	7.49	7.47	7.35	6.75
1393666_at	Arhgap5	1	0	1	0	0	0	6.87	6.75	7.48	6.84	7.01	0	0	0	0	0	0	0	7.19	7.37	7.32	7.54	7.57	7.57	7.25
1393669_at	Rab1b	1	1	0	1	1	0	5.90	5.21	6.42	5.15	5.58	0	0	0	0	0	0	0	9.28	9.34	9.34	9.25	9.32	9.19	8.26
1393677_at	Arhgap26	1	0	1	0	1	1	7.71	7.63	6.74	7.92	6.95	0	0	0	0	0	0	0	4.81	5.07	4.96	5.10	5.23	5.30	5.45
1393687_at	---	1	0	1	0	0	0	6.27	6.57	7.06	6.42	6.53	0	0	0	0	0	0	0	7.15	7.15	7.21	7.24	7.20	7.39	7.22
1393701_at	Dbr1	1	0	1	0	0	0	4.46	4.48	5.16	4.82	4.74	0	0	0	0	0	0	0	5.25	5.26	5.31	5.47	5.52	5.19	5.23
1393702_at	Xrn2	1	0	1	0	0	0	7.32	7.31	7.87	7.41	7.64	0	0	0	0	0	0	0	9.84	9.81	9.94	10.05	9.96	10.22	9.97
1393732_at	---	1	0	0	0	0	1	8.67	8.52	9.23	8.63	9.17	0	0	0	0	0	0	0	8.99	9.09	9.13	9.11	9.26	9.02	8.53
1393736_at	St18	1	0	1	0	1	1	9.09	9.29	10.11	8.87	9.78	0	0	0	0	0	0	0	10.34	10.47	10.40	10.44	10.58	10.49	9.09
1393752_at	Kmt2a	1	0	0	1	0	0	6.77	7.00	6.92	7.28	6.94	0	0	0	0	0	0	0	6.81	6.74	6.89	6.76	6.72	6.58	6.55
1393753_at	Nudcd1	1	0	0	0	0	1	5.64	5.67	6.09	5.59	6.24	0	0	0	0	0	0	0	8.07	8.18	8.11	8.18	8.25	8.21	7.30
1393758_at	Ttc14	1	0	1	0	0	0	5.60	5.84	6.26	5.77	5.84	0	0	0	0	0	0	0	6.86	6.85	6.88	6.92	6.84	6.62	5.89
1393772_at	---	1	0	1	0	1	0	6.59	6.34	7.24	6.33	6.86	0	0	0	0	0	0	0	8.03	8.18	8.11	8.19	8.17	8.00	7.66
1393776_at	Ly6e	1	0	1	0	0	0	4.45	4.38	3.90	4.35	4.22	0	0	0	0	0	0	0	5.96	5.78	5.87	5.82	5.76	5.43	4.95
1393777_at	---	1	0	1	0	1	0	6.50	6.62	7.23	6.25	6.68	0	0	0	0	0	0	0	9.21	9.29	9.28	9.27	9.31	9.70	9.20
1393779_x_at	P3h4	1	0	0	0	0	1	5.16	5.43	5.46	5.21	5.75	0	0	0	0	0	0	0	10.06	10.05	10.01	9.97	10.03	9.90	9.58
1393780_at	Znf131	1	0	1	0	0	0	4.73	4.41	5.31	4.81	4.73	0	0	0	0	0	0	0	6.68	6.84	6.82	6.90	6.95	6.53	5.15
1393794_at	---	1	0	0	0	1	0	6.87	6.54	7.33	6.60	7.14	0	0	0	0	0	0	0	7.82	7.88	7.90	7.94	7.96	8.00	8.20
1393798_at	Atrx /// LOC1030	1	0	1	0	0	0	7.09	6.83	7.52	7.21	7.25	0	0	0	0	0	0	0	8.28	8.26	8.38	8.49	8.31	8.06	7.63
1393800_at	Armcx5	1	0	1	0	1	0	5.82	5.72	6.70	5.60	6.27	0	0	0	0	0	0	0	8.44	8.50	8.41	8.45	8.39	8.08	7.50
1393806_at	---	1	0	1	0	1	1	7.58	7.26	6.91	7.71	7.11	0	0	0	0	0	0	0	5.15	5.15	5.17	5.21	5.34	5.66	5.72
1393809_at	Traf6	1	0	0	1	1	0	4.81	5.14	4.78	5.37	4.89	0	0	0	0	0	0	0	6.74	6.66	6.74	6.66	6.54	6.85	7.65
1393811_at	Ercc6l2	1	1	0	1	0	0	4.29	4.79	4.54	4.90	4.75	0	0	0	0	0	0	0	4.99	4.68	4.98	4.98	4.86	4.57	4.24
1393817_at	---	1	0	1	0	0	0	5.37	5.49	5.94	5.44	5.65	0	0	0	0	0	0	0	8.31	8.40	8.55	8.68	8.72	8.77	8.10
1393841_at	Lingo2	1	0	1	0	0	1	3.83	4.09	4.52	3.88	4.60	0	0	0	0	0	0	0	3.68	3.64	3.65	3.74	3.64	3.88	3.57
1393842_at	Ccdc77	1	0	0	0	0	1	6.63	6.66	7.03	6.70	5.10	0	0	0	0	0	0	0	7.30	7.24	7.40	7.40	7.32	7.06	6.85
1393860_at	Mical2	1	0	0	1	1	0	3.59	3.60	3.69	4.55	3.59	0	0	0	0	0	0	0	4.41	4.31	4.44	4.41	4.46	4.57	5.01
1393874_at	Aqp12a	1	0	1	0	1	0	11.90	11.94	11.20	12.28	11.64	0	0	0	0	0	0	0	6.16	6.19	6.14	5.96	5.94	6.07	5.71
1393881_at	Naa15	1	0	1	0	0	0	7.81	7.84	8.40	8.07	8.13	0	0	0	0	0	0	0	10.07	10.01	10.07	10.21	10.17	10.22	9.75
1393906_at	Fam149a	1	0	0	0	1	0	6.60	6.60	6.45	7.03	6.67	0	0	0	0	0	0	0	7.34	7.23	7.34	7.39	7.35	7.14	6.67
1393907_at	Pyy	1	0	1	0	0	0	10.83	11.06	11.56	10.87	10.55	0	0	0	0	0	0	0	4.60	4.68	4.44	4.35	4.33	4.50	4.68
1393920_at	---	1	0	0	0	0	1	4.66	4.81	4.60	4.81	5.19	0	0	0	0	0	0	0	5.67	5.60	5.60	5.52	5.39	5.60	5.57
1393922_at	Efcab7	1	0	1	0	1	0	4.35	4.38	5.12	4.24	4.57	0	0	0	0	0	0	0	7.97	8.07	8.14	8.21	8.21	8.05	7.65
1393929_at	Ccr6	1	0	0	0	0	1	4.51	4.56	4.27	4.52	5.10	0	0	0	0	0	0	0	4.54	4.53	4.38	4.41	4.36	4.41	3.95
1393930_at	Cul5	1	0	1	0	1	0	4.31	4.40	4.89	3.97	4.37	0	0	0	0	0	0	0	6.04	6.00	6.14	6.39	6.19	5.81	5.07
1393933_at	Sorl1	1	0	0	0	0	1	7.59	7.63	8.13	7.67	8.10	0	0	0	0	0	0	0	8.60	8.52	8.61	8.56	8.68	8.49	8.37
1393937_at	---	1	0	0	0	1	0	6.54	6.36	6.86	6.07	6.19	0	0	0	0	0	0	0	9.41	9.41	9.55	9.64	9.62	9.35	8.68
1393971_at	LOC102549203	1	0	0	0	1	0	5.98	5.93	5.62	6.38	5.80	0	0	0	0	0	0	0	4.93	4.83	4.91	4.74	4.82	4.83	4.25
1393974_at	Meox1	1	0	0	0	0	1	4.07	3.65	3.56	3.72	3.56	0	0	0	0	0	0	0	3.56	3.48	3.40	3.40	3.51	3.44	2.95
1393977_at	Mtmr9	1	0	1	0	1	0	6.90	6.87	7.49	6.64	7.36	0	0	0	0	0	0	0	7.67	7.77	7.79	7.77	7.67	7.67	7.25
1393980_at	---	1	0	1	0	0	0	6.57	6.09	5.54	5.92	5.98	0	0	0	0	0	0	0	6.94	6.97	6.96	6.77	6.88	6.93	6.63
1393995_at	---	1	0	1	0	0	0	8.29	8.33	8.96	8.37	8.58	0	0	0	0	0	0	0	5.82	5.83	6.15	5.90	5.88	6.25	6.06
1394026_at	---	1	0	1	0	0	0	6.57	6.53	7.14	6.90	6.82	0	0	0	0	0	0	0	6.72	6.58	6.76	6.97	6.71	6.64	6.21
1394029_at	Vma21	1	0	1	0	1	1	5.94	5.91	6.78	5.78	6.65	0	0	0	0	0	0	0	8.53	8.61	8.59	8.65	8.65	8.46	7.60
1394041_at	Ankhd1 /// Eif4e	1	0	1	0	0	0	7.01	7.13	7.59	7.03	7.43	0	0	0	0	0	0	0	9.29	9.24	9.26	9.31	9.22	9.13	8.80
1394058_at	Lgals12	1	0	1	0	1	0	7.84	7.52	7.13	8.16	7.48	0	0	0	0	0	0	0	3.48	3.41	3.51	3.39	3.41	3.50	3.09
1394059_s_at	Yme1l1	1	0	1	0	1	1	7.93	7.81	8.66	7.70	8.47	0	0	0	0	0	0	0	10.30	10.31	10.27	10.46	10.43	10.34	9.83
1394065_at	Ints2	1	0	1	0	1	1	5.74	5.95	6.72	5.45	6.72	0	0	0	0	0	0	0	7.92	8.00	8.02	8.17	8.15	7.80	7.16
1394066_at	Smarca2	1	0	0	0	0	1	5.45	5.69	5.98	5.74	6.13	0	0	0	0	0	0	0	7.16	6.94	6.97	6.93	6.98	6.76	6.12
1394082_at	Psmd14	1	0	0	0	1	0	7.15	7.14	7.47	6.72	7.45	0	0	0	0	0	0	0	9.82	9.82	9.90	9.95	9.90	9.72	9.02
1394086_at	Senp7	1	0	1	0	1	0	7.06	7.09	7.87	7.10	7.26	0	0	0	0	0	0	0	8.80	8.86	8.94	8.99	8.98	9.06	8.61
1394093_at	Smarca5	1	0	1	0	1	0	6.01	6.04	6.62	5.59	6.06	0	0	0	0	0	0	0	8.50	8.62	8.66	8.82	8.75	8.66	8.03
1394095_at	Gin1	1	0	1	0	1	0	5.26	5.42	6.25	4.96	5.87	0	0	0	0	0	0	0	7.21	7.13	7.34	7.40	7.41	7.03	6.42
1394129_at	---	1	0	0	0	1	0	4.02	4.24	4.57	3.82	4.18	0	0	0	0	0	0	0	7.11	7.08	7.13	7.14	6.96	6.65	6.35
1394135_at	---	1	0	0	0	1	0	8.80	9.00	9.26	8.53	8.91	0	0	0	0	0	0	0	11.48	11.49	11.55	11.63	11.61	11.61	11.25
1394155_at	Rbm7	1	0	0	1	1	0	6.83	6.74	7.03	6.24	6.75	0	0	0	0	0	0	0	7.89	8.01	7.90	7.93	7.84	7.71	6.68
1394156_at	Igsf3	1	0	1	0	0	0	5.04	5.11	4.47	4.93	4.83	0	0	0	0	0	0	0	4.47	4.39	4.61	4.54	4.55	4.52	4.69
1394160_at	Cemip2	1	0	0	0	0	1	3.79	4.03	3.83	3.87	4.39	0	0	0	0	0	0	0	8.23	8.11	8.22	8.29	8.32	8.56	8.96
1394169_at	Gpr150	1	1	0	0	0	0	4.37	4.86	4.89	4.69	4.69	0	0	0	0	0	0	0	6.32	6.30	6.37	6.33	6.38	6.35	6.01
1394238_x_at	---	1	0	1	0	1	0	8.67	8.67	8.08	8.92	8.32	0	0	0	0	0	0	0	6.97	7.04	6.91	6.72	6.84	6.99	6.55
1394264_at	Chst6	1	0	0	0	0	1	5.93	5.86	5.74	5.70	5.47	0	0	0	0	0	0	0	4.39	4.41	4.33	4.35	4.28	4.45	3.73
1394275_at	Stard5	1	0	0	0	1	0	7.49	7.52	8.02	7.02	7.63	0	0	0	0	0	0	0	8.63	8.62	8.65	8.78	8.74	8.89	8.79
1394290_at	---	1	0	0	0	0	1	5.11	4.71	4.71	5.47	4.77	0	0	0	0	0	0	0	5.64	5.53	5.70	5.81	5.76	5.55	5.67
1394309_at	Tekt5	1	0	1	0	0	0	4.09	3.87	3.52	3.94	3.67	0	0	0	0	0	0	0	3.89	3.76	3.75	3.74	3.69	3.81	3.15
1394318_at	Arpp19	1	0	1	1	1	0	5.21	5.30	5.88	4.62	5.51	0	0	0	0	0	0	0	7.38	7.32	7.45	7.63	7.58	7.77	7.31
1394347_at	Golga4	1	0	1	1	0	1	4.45	4.73	5.39	4.99	5.69	0	0	0	0	0	0	0	5.53	5.34	5.58	5.61	5.37	5.17	5.74
1394349_at	Lhx3	1	0	1	0	0	0	6.97	6.69	6.44	6.73	6.73	0	0	0	0	0	0	0	6.97	6.97	6.82	6.75	6.77	6.75	6.80
1394363_at	Riok3	1	0	0	0	0	1	5.96	5.74	6.02	5.61	5.31	0	0	0	0	0	0	0	8.90	8.94	9.04	9.14	9.09	8.96	8.39
1394388_at	Tug1	1	0	0	0	1	0	6.63	6.54	7.11	6.33	6.72	0	0	0	0	0	0	0	8.61	8.63	8.71	8.69	8.71	8.57	8.51
1394399_at	---	1	0	1	0	1	1	5.55	5.82	6.49	5.30	6.50	0	0	0	0	0	0	0	7.34	7.44	7.54	7.71	7.69	7.77	7.63
1394413_at	Pias2	1	0	0	1	0	0	7.06	7.25	7.52	7.53	7.15	0	0	0	0	0	0	0	9.31	9.30	9.44	9.53	9.47	9.51	9.29
1394424_at	Cfap36	1	0	0	0	0	1	6.12	6.25	6.32	5.92	6.66	0	0	0	0	0	0	0	8.90	8.79	8.84	8.95	8.88	9.07	8.33
1394427_at	---	1	0	1	1	1	0	8.05	8.06	8.69	6.82	7.84	0	0	0	0	0	0	0	10.16	10.15	10.22	10.22	10.15	9.91	8.91
1394430_at	Ttc33	1	0	1	0	0	1	5.14	5.30	6.07	5.36	5.43	0	0	0	0	0	0	0	6.71	6.68	6.89	6.84	6.86	6.89	6.72
1394435_at	Vangl1	1	0	0	1	0	0	6.23	6.27	6.46	6.97	6.38	0	0	0	0	0	0	0	7.18	7.07	7.25	7.12	7.02	7.08	7.01
1394436_at	Spag9	1	0	0	1	0	0	3.81	4.20	3.83	4.27	3.73	0	0	0	0	0	0	0	7.42	7.28	7.39	7.41	7.28	7.25	7.19
1394448_at	Osbpl6	1	0	0	0	0	1	6.42	6.38	6.38	6.62	5.94	0	0	0	0	0	0	0	6.76	6.78	6.91	6.83	6.76	6.89	7.18
1394451_at	---	1	0	0	1	0	1	6.62	6.73	6.13	6.00	6.09	0	0	0	0	0	0	0	5.17	5.03	4.99	4.75	4.98	5.16	4.44
1394467_at	---	1	0	0	1	0	1	6.12	6.56	6.35	6.65	6.73	0	0	0	0	0	0	0	5.71	5.56	5.60	5.71	5.65	5.66	5.25
1394477_at	Nom1	1	0	0	0	0	1	6.67	6.81	6.66	6.79	5.41	0	0	0	0	0	0	0	8.11	7.98	8.04	8.12	7.96	7.92	7.65
1394480_at	Kiaa0040	1	0	1	0	1	0	6.05	6.16	5.51	6.25	5.91	0	0	0	0	0	0	0	4.64	4.62	4.56	4.25	4.36	4.32	4.08
1394490_at	Abca1	1	1	1	0	0	0	4.91	5.40	5.52	4.77	4.90	0	0	0	0	0	0	0	5.82	5.60	5.94	5.81	5.70	5.91	5.48
1394493_at	---	1	0	1	0	0	1	6.28	6.35	6.80	6.64	6.58	0	0	0	0	0	0	0	8.47	8.40	8.61	8.51	8.48	8.60	8.64
1394497_at	Tcf7l2	1	0	0	0	1	0	3.57	3.58	3.38	4.00	3.38	0	0	0	0	0	0	0	3.91	4.06	4.07	3.98	4.01	4.16	4.51
1394524_at	Smim7	1	0	0	1	1	0	9.29	9.21	9.63	8.63	9.38	0	0	0	0	0	0	0	11.67	11.65	11.67	11.63	11.63	11.55	10.79
1394534_at	---	1	0	0	1	1	0	5.30	5.42	5.58	4.79	5.59	0	0	0	0	0	0	0	6.80	7.03	7.19	7.16	7.08	6.62	6.16
1394562_at	---	1	0	0	0	0	1	5.64	5.42	5.40	5.37	5.11	0	0	0	0	0	0	0	4.66	4.61	4.55	4.47	4.56	4.86	4.39
1394570_at	Xk	1	0	1	0	0	0	6.12	6.03	6.75	6.25	6.34	0	0	0	0	0	0	0	7.49	7.50	7.61	7.71	7.63	7.56	7.72
1394580_at	Nprl2	1	0	0	1	0	1	5.41	5.09	5.50	4.91	4.92	0	0	0	0	0	0	0	7.23	7.27	7.44	7.42	7.40	7.59	7.03
1394594_at	---	1	0	1	0	1	1	7.16	7.45	7.87	6.76	7.75	0	0	0	0	0	0	0	8.91	8.88	8.97	9.02	9.03	8.67	8.03
1394614_at	Gns /// LOC1009	1	0	0	0	1	0	7.62	7.72	8.17	7.19	7.61	0	0	0	0	0	0	0	9.45	9.51	9.51	9.58	9.57	9.86	8.89
1394616_at	Mesd	1	0	1	0	1	0	6.59	6.56	7.34	6.26	6.66	0	0	0	0	0	0	0	10.61	10.69	10.66	10.65	10.71	10.81	9.79
1394620_at	---	1	0	0	0	0	1	6.61	6.44	6.21	6.45	6.10	0	0	0	0	0	0	0	5.38	5.21	5.22	5.11	5.12	5.16	5.38
1394630_at	---	1	0	1	0	0	1	4.84	4.50	4.22	4.53	4.31	0	0	0	0	0	0	0	5.44	5.39	5.60	5.34	5.37	5.50	5.53
1394632_at	---	1	0	0	1	0	0	3.69	3.44	3.29	3.20	3.50	0	0	0	0	0	0	0	3.52	3.67	3.61	3.44	3.49	3.57	3.36
1394651_at	Lrrc71	1	0	1	0	0	0	5.79	5.51	5.14	5.49	5.47	0	0	0	0	0	0	0	4.25	4.34	4.19	4.23	4.25	4.56	4.22
1394653_at	---	1	0	0	1	1	0	5.80	5.89	5.41	6.30	6.00	0	0	0	0	0	0	0	5.97	5.84	5.93	5.96	5.83	6.07	5.59
1394657_at	---	1	0	0	0	0	1	4.33	4.38	4.24	4.56	3.78	0	0	0	0	0	0	0	5.22	5.38	5.23	4.97	5.18	5.29	4.75

Probeset ID	Gene Symbol	Islet Data Set (log2 ratio ≥0.263; FDR <20%) union n=5325 1=YES 0=NO	Islet DRlyp/lyp ND vs ND+Lp299v (log2 ratio ≥0.263; FDR<20%) n=429 1=YES 0=NO	Islet DRlyp/lyp ND vs HCD (log2 ratio ≥0.263; FDR<20%) n=2247 1=YES 0=NO	Islet DRlyp/lyp ND vs HCD+Lp299v (log2 ratio ≥0.263; FDR<20%) n=1595 1=YES 0=NO	Islet DRlyp/lyp HCD vs HCD+Lp299v (log2 ratio ≥0.263; FDR<20%) n=3026 1=YES 0=NO	Islet DRlyp/lyp ND vs Flyp/lyp ND (log2 ratio ≥ 0.263; FDR<20%) n=1950 1=YES 0=NO	Islet Mean Log2 Intensity DRlyp/lyp ND	Islet Mean Log2 Intensity DRlyp/lyp ND+Lp299v	Islet Mean Log2 Intensity DRlyp/lyp HCD	Islet Mean Log2 Intensity DRlyp/lyp HCD+Lp299v	Islet Mean Log2 Intensity Flyp40 ND	Rinm5f data union of all conditions n=3705 1=YES 0=NO	Rinm5f 30 µM propionate/6 µM butyrate vs Media (FDR<20%; 1.5 fold) n=3 1=YES 0=NO	Rinm5f 60 µM propionate/12 µM butyrate vs Media (FDR<20%; 1.5 fold) n=18 1=YES 0=NO	Rinm5f 120 µM propionate/24 µM butyrate vs Media (FDR<20%; 1.5 fold) n=164 1=YES 0=NO	Rinm5f 240 µM propionate/48 µM butyrate vs Media (FDR<20%; 1.5 fold) n=268 1=YES 0=NO	Rinm5f 1500 µM propionate/300 µM butyrate vs Media (FDR<20%; 1.5 fold) n=2491 1=YES 0=NO	Rinm5f 500 µM propionate/500 µM butyrate vs Media (FDR<20%; 1.5 fold) n=3001 1=YES 0=NO	Rinm5f Mean Log2 Intensity Media only	Rinm5f Mean Log2 Intensity 30 µM propionate/6 µM butyrate	Rinm5f Mean Log2 Intensity 60 µM propionate/12 µM butyrate	Rinm5f Mean Log2 Intensity 120 µM propionate/24 µM butyrate	Rinm5f Mean Log2 Intensity 240 µM propionate/48 µM butyrate	Rinm5f Mean Log2 Intensity 1500 µM propionate/300 µM butyrate	Rinm5f Mean Log2 Intensity 500 µM propionate/500 µM butyrate
1394671_at	---	1	0	0	1	0	0	4.22	4.35	4.37	4.78	4.10	0	0	0	0	0	0	0	4.20	4.22	4.26	4.18	4.10	4.14	3.87
1394682_at	Aff4	1	0	0	0	0	1	4.88	5.07	5.40	4.95	5.45	0	0	0	0	0	0	0	6.14	6.16	6.35	6.20	6.21	5.84	5.51
1394689_at	---	1	0	1	0	0	1	7.33	7.32	7.89	7.47	7.92	0	0	0	0	0	0	0	9.63	9.49	9.67	9.56	9.57	9.68	9.61
1394693_at	---	1	0	1	1	1	1	4.40	4.70	5.37	3.75	3.86	0	0	0	0	0	0	0	3.07	3.09	3.17	3.11	3.14	3.07	2.75
1394701_at	---	1	0	1	0	1	0	12.29	12.16	11.29	12.12	12.33	0	0	0	0	0	0	0	4.50	4.56	4.46	4.40	4.54	4.35	3.78
1394721_at	Baz2a	1	1	0	1	0	0	4.81	5.34	5.19	5.41	5.18	0	0	0	0	0	0	0	6.74	6.59	6.74	6.67	6.60	6.71	6.53
1394731_at	Csnk1g3	1	0	1	0	1	0	5.39	5.72	6.03	5.25	5.56	0	0	0	0	0	0	0	8.48	8.41	8.52	8.55	8.53	8.63	8.10
1394741_at	Hnrnpa3	1	0	1	1	1	0	6.45	6.51	7.22	5.89	6.30	0	0	0	0	0	0	0	8.27	8.33	8.46	8.63	8.68	8.74	8.10
1394756_at	Rbm26	1	0	1	0	0	0	7.03	7.06	7.61	7.35	7.48	0	0	0	0	0	0	0	6.84	6.88	6.88	7.03	7.06	6.92	6.55
1394764_at	Lmtk2	1	0	1	0	0	0	6.01	5.67	5.37	5.79	5.69	0	0	0	0	0	0	0	6.46	6.39	6.50	6.37	6.40	6.33	5.97
1394778_at	---	1	0	0	0	1	0	6.43	6.46	6.12	6.68	5.98	0	0	0	0	0	0	0	4.49	4.29	4.41	4.27	4.35	4.43	3.95
1394779_at	---	1	0	1	0	0	1	6.66	6.83	6.11	6.45	6.15	0	0	0	0	0	0	0	6.07	6.11	6.02	6.05	6.05	6.23	5.93
1394783_at	Dcun1d1	1	0	0	0	1	0	5.83	5.90	6.29	5.36	5.78	0	0	0	0	0	0	0	8.01	7.96	8.08	8.29	8.09	7.97	6.92
1394786_at	Sorl1	1	0	1	0	1	0	9.44	9.73	10.19	9.24	9.66	0	0	0	0	0	0	0	10.00	9.99	10.06	10.10	10.18	10.10	9.49
1394793_at	LOC102547322	1	0	1	0	1	1	6.22	6.57	7.16	5.94	7.07	0	0	0	0	0	0	0	7.30	7.52	7.76	7.80	7.85	7.06	6.72
1394808_at	Tm9sf4	1	0	0	0	1	0	8.64	8.85	8.37	8.95	8.50	0	0	0	0	0	0	0	10.00	9.89	9.91	9.77	9.80	9.57	8.78
1394818_at	---	1	0	0	1	1	1	8.05	7.94	8.57	7.36	7.52	0	0	0	0	0	0	0	10.59	10.63	10.72	10.78	10.76	10.84	9.94
1394820_at	---	1	0	0	0	0	1	6.54	6.69	6.98	6.62	7.14	0	0	0	0	0	0	0	7.46	7.43	7.64	7.73	7.80	7.84	7.83
1394837_at	---	1	0	1	1	1	1	5.75	6.09	6.74	5.23	6.75	0	0	0	0	0	0	0	3.61	3.65	3.69	3.57	3.52	3.74	3.29
1394839_at	---	1	0	0	0	0	1	3.26	3.38	3.23	3.33	4.64	0	0	0	0	0	0	0	3.91	3.93	3.92	3.87	3.84	3.94	3.40
1394849_at	Zbtb20	1	0	0	1	0	0	4.41	4.42	4.58	4.94	4.53	0	0	0	0	0	0	0	4.07	4.12	4.37	4.22	4.19	4.14	4.10
1394854_at	Terf1	1	0	1	0	0	0	6.37	6.47	6.96	6.27	6.70	0	0	0	0	0	0	0	8.77	8.83	8.83	8.92	8.86	8.67	8.06
1394855_at	Kcnj5	1	0	1	1	0	0	5.21	5.38	5.81	5.73	5.31	0	0	0	0	0	0	0	4.39	4.49	4.62	4.58	4.52	4.40	3.97
1394876_at	---	1	0	0	0	0	1	4.62	4.69	4.78	4.72	5.12	0	0	0	0	0	0	0	6.19	6.13	6.20	6.00	5.99	6.00	6.48
1394891_at	---	1	0	1	0	0	0	4.22	4.43	4.83	4.31	4.39	0	0	0	0	0	0	0	6.36	6.25	6.51	6.66	6.54	6.65	5.18
1394895_at	Ahi1 /// LOC103	1	0	0	0	0	1	5.54	5.79	6.01	5.46	6.27	0	0	0	0	0	0	0	6.19	6.09	6.27	6.21	6.22	6.47	7.04
1394931_at	Mbnl2	1	0	1	1	1	0	7.47	7.58	8.09	5.82	7.19	0	0	0	0	0	0	0	9.55	9.54	9.60	9.78	9.65	9.79	8.75
1394935_at	Wasf2	1	1	0	1	0	0	5.95	6.60	6.11	6.66	6.40	0	0	0	0	0	0	0	6.73	6.46	6.65	6.43	6.58	6.54	7.48
1394940_at	Tent5a	1	0	1	1	1	1	6.38	5.97	7.14	5.55	7.15	0	0	0	0	0	0	0	12.47	12.56	12.57	12.53	12.64	12.68	12.27
1394943_at	Crebzf	1	0	0	1	0	0	5.32	5.31	5.80	5.86	5.52	0	0	0	0	0	0	0	6.97	6.97	7.20	7.18	7.13	6.79	6.12
1394946_at	Kiaa0408	1	1	1	0	1	1	5.73	6.30	6.71	5.55	7.23	0	0	0	0	0	0	0	7.68	7.68	7.75	7.93	7.89	7.53	7.28
1394964_at	LOC691387 /// P	1	1	0	0	0	0	5.73	6.03	5.88	5.87	5.78	0	0	0	0	0	0	0	6.32	6.17	6.45	6.33	6.39	6.15	6.20
1394965_at	Clint1	1	0	0	0	1	0	6.36	6.76	6.76	5.99	6.39	0	0	0	0	0	0	0	7.78	7.80	7.87	7.89	7.88	8.00	7.45
1394968_at	LOC103690175 /	1	0	1	0	1	1	4.05	4.42	4.97	3.84	4.60	0	0	0	0	0	0	0	7.40	7.43	7.52	7.73	7.73	7.92	7.33
1394972_at	Ro60	1	0	1	1	1	1	4.26	4.42	5.04	3.75	4.77	0	0	0	0	0	0	0	6.29	6.10	6.37	6.47	6.38	6.54	5.71
1394975_at	Znf638	1	0	0	1	1	0	7.02	7.01	7.48	6.37	6.87	0	0	0	0	0	0	0	8.22	8.29	8.44	8.65	8.59	8.53	8.14
1394980_at	Tlcd4	1	0	0	0	1	0	4.27	4.32	3.85	4.66	4.06	0	0	0	0	0	0	0	4.26	4.31	4.36	4.15	4.19	4.12	3.85
1395009_at	---	1	0	1	1	1	0	5.03	5.43	5.94	4.34	5.46	0	0	0	0	0	0	0	6.10	6.09	6.38	6.50	6.37	5.97	4.53
1395030_at	---	1	0	0	0	0	1	7.24	7.57	7.11	7.13	6.78	0	0	0	0	0	0	0	6.44	6.43	6.44	6.30	6.42	6.60	6.20
1395035_at	LOC102547868	1	0	1	0	1	1	6.39	6.07	5.29	6.45	5.76	0	0	0	0	0	0	0	3.65	3.66	3.60	3.56	3.51	3.54	3.13
1395045_at	---	1	0	1	0	0	0	5.62	5.83	6.28	6.03	6.07	0	0	0	0	0	0	0	8.16	8.19	8.27	8.50	8.35	8.31	7.58
1395052_at	---	1	0	0	1	0	1	5.53	5.49	6.05	6.03	5.00	0	0	0	0	0	0	0	7.33	7.25	7.43	7.44	7.26	7.77	7.58
1395058_at	Wdr75	1	0	1	0	0	1	5.86	6.08	6.54	5.87	6.56	0	0	0	0	0	0	0	7.60	7.48	7.59	7.71	7.63	7.33	6.55
1395064_at	S100pbp	1	0	1	0	0	0	6.74	6.72	7.40	6.71	6.84	0	0	0	0	0	0	0	9.33	9.23	9.31	9.28	9.22	9.21	9.20
1395071_at	Zbtb6	1	0	1	1	1	0	4.30	4.28	4.94	3.48	4.15	0	0	0	0	0	0	0	6.50	6.48	6.56	6.80	6.69	6.55	5.34
1395076_at	Runx1t1	1	0	0	0	0	1	3.33	3.40	3.26	3.37	4.03	0	0	0	0	0	0	0	5.85	5.99	6.08	5.94	5.76	5.76	6.06
1395082_at	Cpsf6	1	0	1	0	1	0	3.97	4.24	4.94	3.93	4.30	0	0	0	0	0	0	0	7.26	7.31	7.51	7.53	7.54	7.35	6.38
1395083_at	---	1	0	1	0	1	1	4.00	4.13	4.84	3.85	4.84	0	0	0	0	0	0	0	6.62	6.94	7.09	6.94	7.10	6.82	5.93
1395100_at	Pbrm1	1	1	1	1	0	0	6.10	6.58	6.83	6.72	6.23	0	0	0	0	0	0	0	8.88	8.60	8.83	8.90	8.62	8.55	8.14
1395121_at	---	1	1	0	0	0	1	5.22	5.72	5.62	5.21	6.14	0	0	0	0	0	0	0	3.94	3.98	3.84	3.71	4.02	4.02	3.63
1395124_at	Wars1	1	0	0	0	0	1	6.54	6.84	6.82	6.58	7.04	0	0	0	0	0	0	0	9.60	9.63	9.71	9.71	9.69	9.80	8.99
1395147_at	---	1	0	1	0	1	0	5.61	5.70	6.46	5.23	5.89	0	0	0	0	0	0	0	8.48	8.48	8.54	8.51	8.49	8.34	7.93
1395155_at	Exoc5	1	0	1	0	1	0	5.15	5.17	5.72	4.91	5.30	0	0	0	0	0	0	0	7.53	7.56	7.57	7.78	7.71	7.55	6.76
1395157_at	Nedd4	1	0	1	0	1	0	7.66	7.65	8.17	7.18	7.30	0	0	0	0	0	0	0	9.10	9.00	9.10	9.13	9.03	8.98	7.93
1395161_at	Myt1	1	0	0	0	1	0	5.23	5.32	5.80	4.93	5.52	0	0	0	0	0	0	0	8.24	8.26	8.40	8.49	8.43	8.12	7.42
1395165_at	Psip1	1	0	1	0	1	0	5.01	5.42	6.08	4.85	5.46	0	0	0	0	0	0	0	8.01	8.04	8.15	8.23	8.22	8.16	7.30
1395211_s_at	Svil	1	0	0	1	0	0	5.21	5.55	5.38	5.81	5.06	0	0	0	0	0	0	0	5.11	5.04	5.04	4.98	4.90	4.90	5.35
1395214_at	Lyrm9	1	0	0	0	1	0	6.51	6.56	6.26	6.89	6.27	0	0	0	0	0	0	0	6.81	6.73	6.87	6.97	6.85	6.87	7.00
1395234_at	---	1	0	1	0	1	1	5.31	5.25	4.61	5.28	4.80	0	0	0	0	0	0	0	4.75	4.67	4.58	4.29	4.46	4.62	3.91
1395237_at	Eif5b	1	0	0	1	0	0	8.85	9.01	8.83	9.34	8.70	0	0	0	0	0	0	0	9.14	9.14	9.29	9.23	9.20	9.18	9.09
1395248_at	Edem1	1	0	1	0	1	1	8.47	8.32	7.71	8.72	7.88	0	0	0	0	0	0	0	7.67	7.70	7.75	7.72	7.70	7.73	7.25
1395263_at	---	1	0	1	0	1	0	4.78	5.10	5.53	4.61	5.05	0	0	0	0	0	0	0	5.11	5.16	5.42	5.35	5.22	5.26	4.91
1395264_at	Rif1	1	0	1	0	0	0	5.96	6.24	6.50	6.31	6.16	0	0	0	0	0	0	0	6.90	6.78	7.10	7.27	6.99	7.09	7.26
1395265_at	Gns /// LOC1009	1	0	0	0	1	0	7.32	7.31	7.82	7.01	7.24	0	0	0	0	0	0	0	9.77	9.73	9.79	9.74	9.82	10.08	9.82
1395273_at	Ahcyl1	1	0	0	1	1	0	5.26	5.47	5.82	4.75	5.07	0	0	0	0	0	0	0	9.40	9.30	9.29	9.34	9.32	9.29	8.59
1395294_at	Runx1t1	1	0	0	0	0	1	4.32	4.56	4.32	4.64	4.88	0	0	0	0	0	0	0	6.55	6.50	6.57	6.57	6.30	6.28	6.23
1395298_at	Ubp1	1	1	0	1	0	0	5.74	6.31	6.02	6.35	6.17	0	0	0	0	0	0	0	5.97	5.90	6.11	6.15	6.02	5.91	4.67
1395316_at	Mageh1	1	0	1	0	0	0	9.60	9.63	10.22	9.58	9.99	0	0	0	0	0	0	0	12.06	12.10	12.10	12.14	12.22	12.17	11.30
1395318_at	Chd9	1	1	0	1	1	0	6.92	7.60	6.81	7.45	6.95	0	0	0	0	0	0	0	5.15	5.11	5.18	5.25	5.10	5.01	4.99
1395328_at	Nsmce4a	1	0	0	0	1	0	5.74	5.60	6.20	5.33	5.61	0	0	0	0	0	0	0	9.74	9.72	9.80	9.75	9.71	9.50	9.02
1395331_at	Erlec1	1	0	0	1	0	0	9.09	8.84	9.14	8.52	8.84	0	0	0	0	0	0	0	10.92	11.01	10.93	10.86	10.88	10.65	10.00
1395335_at	---	1	0	0	0	1	0	8.74	8.94	9.21	8.31	8.89	0	0	0	0	0	0	0	4.84	4.74	4.64	4.66	4.61	4.74	4.83
1395338_at	Lrpprc	1	0	1	1	1	0	5.10	4.92	5.75	4.15	4.82	0	0	0	0	0	0	0	8.50	8.40	8.51	8.66	8.55	8.46	6.82
1395339_at	Impact	1	0	1	1	1	0	5.29	5.43	5.88	4.69	5.17	0	0	0	0	0	0	0	8.28	8.29	8.39	8.53	8.50	8.56	7.78
1395340_at	Slc30a9	1	0	1	1	1	0	7.19	7.28	8.11	5.91	7.04	0	0	0	0	0	0	0	9.83	9.84	9.86	9.79	9.78	9.76	8.54
1395346_at	Aamp	1	0	0	1	1	0	8.09	7.79	8.35	7.42	7.80	0	0	0	0	0	0	0	9.12	9.23	9.28	9.31	9.18	9.14	8.19
1395354_at	Lancl1	1	0	1	1	1	0	6.98	6.64	7.61	6.32	7.07	0	0	0	0	0	0	0	7.73	7.80	7.76	7.75	7.74	7.69	6.95
1395366_at	LOC102546433	1	0	0	0	0	1	5.50	5.79	5.77	5.65	4.50	0	0	0	0	0	0	0	5.02	4.86	5.18	5.10	5.05	4.78	4.47
1395368_at	Zfhx2	1	0	1	0	1	1	5.17	5.55	5.87	5.05	6.14	0	0	0	0	0	0	0	6.45	6.59	6.75	6.81	6.85	6.82	5.99
1395370_at	Gmcl1	1	0	1	1	1	0	5.03	4.95	5.88	4.44	4.92	0	0	0	0	0	0	0	8.18	8.23	8.16	8.20	8.22	8.30	7.16
1395371_at	Suds3	1	0	0	1	1	0	4.05	3.82	4.28	3.57	3.87	0	0	0	0	0	0	0	6.98	7.02	7.14	7.23	7.18	7.22	6.38
1395375_at	Ttc9c	1	0	1	1	1	0	6.93	6.82	7.53	5.92	7.22	0	0	0	0	0	0	0	10.41	10.34	10.37	10.42	10.33	10.24	9.74
1395381_at	---	1	0	1	0	1	0	6.26	6.42	5.62	6.24	6.13	0	0	0	0	0	0	0	6.56	6.35	6.52	6.55	6.48	6.67	6.74
1395385_at	Ralgapa1	1	0	0	1	1	0	4.95	4.88	5.47	4.37	5.00	0	0	0	0	0	0	0	5.97	5.89	5.76	5.88	5.81	5.70	4.10
1395394_at	Gga2	1	0	1	1	1	0	5.90	5.85	6.51	5.02	5.74	0	0	0	0	0	0	0	8.48	8.57	8.63	8.67	8.74	8.99	7.85
1395406_at	Sbno1	1	0	1	0	0	0	5.34	5.62	5.89	5.72	5.81	0	0	0	0	0	0	0	7.20	7.03	7.16	7.26	7.22	7.46	7.07
1395410_at	Ppp2r3a	1	0	0	1	1	0	6.53	6.44	6.20	7.07	6.41	0	0	0	0	0	0	0	5.80	5.63	5.85	5.72	5.72	5.94	6.16
1395411_at	Naprt	1	0	0	0	0	1	4.62	4.27	4.37	4.30	4.14	0	0	0	0	0	0	0	7.77	7.93	7.94	8.00	7.81	7.85	6.90
1395412_at	Caprin1	1	0	0	1	1	0	7.36	7.26	7.90	6.45	7.09	0	0	0	0	0	0	0	10.93	10.86	10.98	11.13	10.96	10.62	9.51
1395426_at	Ube2v1	1	0	0	1	1	0	8.60	8.32	8.85	8.11	8.37	0	0	0	0	0	0	0	10.54	10.58	10.58	10.60	10.46	10.51	9.59
1395436_at	---	1	0	0	1	0	0	6.57	6.31	6.71	7.11	6.46	0	0	0	0	0	0	0	7.21	7.43	7.54	7.49	7.51	7.64	7.54
1395465_at	Rae1	1	0	0	1	1	0	5.97	5.72	6.14	5.38	5.78	0	0	0	0	0	0	0	8.80	8.81	8.85	8.86	8.82	8.68	7.76
1395468_at	Dlg4	1	0	0	0	1	0	6.12	5.88	6.51	5.80	5.93	0	0	0	0	0	0	0	7.21	7.33	7.35	7.39	7.45	7.34	6.74
1395485_s_at	Myo1b	1	1	0	0	1	0	5.49	6.05	5.24	5.84	5.07	0	0	0	0	0	0	0	7.17	7.09	7.15	7.19	7.12	7.10	5.92
1395507_at	---	1	0	1	0	0	0	5.60	5.46	5.05	5.42	5.26	0	0	0	0	0	0	0	5.20	5.23	5.08	4.84	4.95	5.22	4.76
1395509_at	---	1	0	1	0	1	0	5.25	5.37	5.88	5.15	5.52	0	0	0	0	0	0	0	7.31	7.43	7.53	7.61	7.69	7.59	7.46
1395516_at	Arglu1	1	0	0	0	1	0	8.20	8.57	8.08	8.66	7.87	0	0	0	0	0	0	0	9.42	9.45	9.50	9.46	9.49	9.54	9.36
1395523_at	Rbmx	1	0	0	0	1	0	6.77	6.52	7.28	6.50	6.71	0	0	0	0	0	0	0	7.99	8.01	8.20	8.36	8.32	7.93	7.67
1395524_at	Naa25	1	0	0	0	0	1	3.61	3.98	4.11	4.06	4.22	0	0	0	0	0	0	0	6.78	6.67	6.86	6.93	6.92	6.77	6.30
1395533_at	Hars1	1	0	0	1	1	0	7.39	7.25	7.60	6.27	7.20	0	0	0	0	0	0	0	10.11	10.20	10.27	10.31	10.25	10.03	8.88
1395535_at	Fam98a	1	0	0	0	0	1	6.48	6.53	6.77	6.51	6.93	0	0	0	0	0	0	0	8.67	8.63	8.67	8.70	8.68	8.72	8.01
1395536_at	---	1	0	1	0	0	0	6.76	7.01	7.47	7.02	6.53	0	0	0	0	0	0	0	3.69	3.72	3.73	3.62	3.61	3.70	3.25
1395537_at	Slc35f6	1	0	0	0	1	0	5.43	5.56	5.19	5.81	5.40	0	0	0	0	0	0	0	5.47	5.54	5.63	5.52	5.55	5.74	5.60
1395540_at	Slc35e3	1	0	0	0	0	1	6.81	6.42	6.55	6.47	6.34	0	0	0	0	0	0	0	7.64	7.63	7.82	7.88	7.94	7.97	7.42
1395547_at	---	1	0	0	1	1	0	6.63	6.65	7.15	6.06	6.92	0	0	0	0	0	0	0	9.19	9.10	9.22	9.35	9.24	8.97	8.52
1395550_at	LOC103694231	1	0	1	0	0	0	5.43	5.21	4.90	5.32	5.13	0	0	0	0	0	0	0	4.59	4.63	4.66	4.55	4.36	4.65	3.90
1395552_s_at	Ankrd13c	1	0	1	0	1	0	4.32	4.25	5.03	4.00	4.55	0	0	0	0	0	0	0	8.00	8.09	8.02	8.12	8.18	8.19	7.06
1395553_at	Zfp68	1	0	1	1	1	0	4.26	4.32	5.29	3.35	4.12	0	0	0	0	0	0	0	8.29	8.28	8.42	8.57	8.52	8.56	7.66
1395559_at	---	1	0	0	1	0	0	6.69	6.28	6.80	6.13	6.27	0	0	0	0	0	0	0	6.93	6.94	6.93	6.94	6.88	6.85	6.86
1395565_at	Cops4	1	0	0	1	1	0	8.36	8.26	8.66	7.73	8.28	0	0	0	0	0	0	0	10.87	10.91	10.89	10.91	10.86	10.69	9.89
1395572_at	Dnaja4	1	0	0	1	1	0	5.67	5.82	6.14	4.86	5.50	0	0	0	0	0	0	0	7.39	7.43	7.53	7.62	7.50	7.42	6.51
1395574_at	Ptbp3	1	0	1	0	1	0	6.52	6.63	7.32	6.10	6.65	0	0	0	0	0	0	0	8.02	7.96	8.07	8.15	8.08	8.11	8.16
1395584_at	Zc3h11a	1	0	0	0	1	0	7.25	7.12	7.66	6.93	7.41	0	0	0	0	0	0	0	9.69	9.69	9.78	9.88	9.95	9.80	9.31
1395586_at	---	1	0	1	1	1	1	7.34	7.08	8.06	5.53	6.49	0	0	0	0	0	0	0	9.54	9.54	9.58	9.77	9.68	9.36	8.26
1395620_at	Fam133b	1	0	1	1	1	0	5.29	5.13	5.87	4.80	5.21	0	0	0	0	0	0	0	8.09	8.19	8.24	8.34	8.35	8.29	7.87
1395621_at	LOC100910864 /	1	0	0	1	1	0	6.81	6.77	7.37	6.16	6.92	0	0	0	0	0	0	0	9.24	9.18	9.22	9.25	9.21	9.11	8.57
1395640_at	Cdc23	1	0	0	1	1	0	5.41	5.63	5.86	4.85	5.21	0	0	0	0	0	0	0	8.18	8.14	8.14	8.19	8.03	7.95	6.47
1395655_at	---	1	0	0	1	0	0	8.16	7.85	8.23	8.69	7.99	0	0	0	0	0	0	0	9.13	9.33	9.26	9.28	9.37	9.36	9.16
1395663_at	Mall	1	0	1	0	0	1	4.24	4.08	3.50	3.93	3.71	0	0	0	0	0	0	0	4.44	4.34	4.39	4.27	4.33	4.40	4.31
1395666_at	---	1	0	0	1	1	0	4.26	4.47	4.20	4.81	4.34	0	0	0	0	0	0	0	4.44	4.29	4.35	4.21	4.19	4.11	3.63
1395678_at	Syt15	1	0	0	0	1	0	5.61	5.38	5.20	5.83	5.33	0	0	0	0	0	0	0	5.14	5.01	5.01	4.77	4.93	4.93	4.51
1395693_at	Erp44	1	0	0	1	0	0	6.94	6.69	6.92	6.38	6.86	0	0	0	0	0	0	0	8.88	8.99	8.93	9.00	9.09	8.82	7.76
1395694_at	Fbxo9	1	0	0	0	0	1	8.32	8.15	8.45	8.43	7.77	0	0	0	0	0	0	0	8.40	8.39	8.46	8.51	8.44	8.28	7.97
1395705_at	---	1	0	0	1	1	1	5.41	5.67	5.66	4.92	4.70	0	0	0	0	0	0	0	4.30	4.26	4.34	4.15	4.32	4.34	4.20
1395719_at	Tufm	1	0	0	1	1	0	6.58	6.24	6.65	5.94	6.23	0	0	0	0	0	0	0	9.46	9.51	9.55	9.62	9.53	9.72	9.22
1395720_at	---	1	0	0	1	1	0	6.11	5.97	6.65	5.25	6.10	0	0	0	0	0	0	0	9.20	9.20	9.34	9.48	9.49	9.43	8.92
1395721_at	Papss2	1	1	1	1	1	1	7.54	8.19	8.65	6.74	8.40	0	0	0	0	0	0	0	6.84	6.84	6.97	6.81	6.91	7.24	6.46
1395725_at	Pnpla8	1	0	0	0	1	0	7.21	7.18	7.53	6.75	7.15	0	0	0	0	0	0	0	9.55	9.56	9.61	9.67	9.66	9.59	9.27
1395726_at	Unc80	1	0	1	1	1	1	7.04	7.48	8.18	6.33	7.58	0	0	0	0	0	0	0	7.65	7.59	7.78	7.95	7.81	7.56	7.31
1395729_at	---	1	0	1	0	0	0	6.00	5.77	5.28	5.63	5.68	0	0	0	0	0	0	0	5.85	5.80	5.87	5.75	5.77	5.82	5.42
1395730_at	Gars1	1	0	0	1	1	0	6.95	6.58	6.93	5.91	6.52	0	0	0	0	0	0	0	10.45	10.43	10.48	10.48	10.50	10.45	9.38
1395736_at	Dstyk	1	0	0	0	1	0	6.29	6.16	6.67	5.94	6.33	0	0	0	0	0	0	0	7.92	7.76	7.92	7.94	7.83	7.79	7.58
1395739_at	Efr3a	1	0	0	1	1	0	6.39	6.20	6.85	5.51	6.47	0	0	0	0	0	0	0	9.39	9.35	9.49	9.46	9.50	9.80	9.33
1395741_at	Oga	1	0	1	0	1	0	7.43	7.36	8.01	7.24	7.46	0	0	0	0	0	0	0	9.61	9.56	9.70	9.79	9.84	10.07	9.69
1395745_at	---	1	0	1	1	1	0	5.82	6.13	6.68	5.28	5.96	0	0	0	0	0	0	0	8.45	8.33	8.37	8.21	8.18	8.56	8.10

Probeset ID	Gene Symbol	Islet Data Set (log2 ratio ≥0.263; FDR <20%) union n=5325 1=YES 0=NO	Islet DRlyp/lyp ND vs ND+Lp299v (log2 ratio ≥0.263; FDR<20%) n=429 1=YES 0=NO	Islet DRlyp/lyp ND vs HCD (log2 ratio ≥0.263; FDR<20%) n=2247 1=YES 0=NO	Islet DRlyp/lyp ND vs HCD+Lp299v (log2 ratio ≥0.263; FDR<20%) n=1595 1=YES 0=NO	Islet DRlyp/lyp HCD vs HCD+Lp299v (log2 ratio ≥0.263; FDR<20%) n=3026 1=YES 0=NO	Islet DRlyp/lyp ND vs Flyp/lyp ND (log2 ratio ≥ 0.263; FDR<20%) n=1950 1=YES 0=NO	Islet Mean Log2 Intensity DRlyp/lyp ND	Islet Mean Log2 Intensity DRlyp/lyp ND+Lp299v	Islet Mean Log2 Intensity DRlyp/lyp HCD	Islet Mean Log2 Intensity DRlyp/lyp HCD+Lp299v	Islet Mean Log2 Intensity Flyp40 ND	Rinm5f data union of all conditions n=3705 1=YES 0=NO	Rinm5f 30 µM propionate/ 6 µM butyrate vs Media (FDR<20%; 1.5 fold) n=3 1=YES 0=NO	Rinm5f 60 µM propionate/ 12 µM butyrate vs Media (FDR<20%; 1.5 fold) n=18 1=YES 0=NO	Rinm5f 120 µM propionate/ 24 µM butyrate vs Media (FDR<20%; 1.5 fold) n=164 1=YES 0=NO	Rinm5f 240 µM propionate /48 µM butyrate vs Media (FDR<20%; 1.5 fold) n=268 1=YES 0=NO	Rinm5f 1500 µM propionate/ 300 µM butyrate vs Media (FDR<20%; 1.5 fold) n=2491 1=YES 0=NO	Rinm5f 500 µM propionate/ 500 µM butyrate vs Media (FDR<20%; 1.5 fold) n=3001 1=YES 0=NO	Rinm5f Mean Log2 Intensity Media only	Rinm5f Mean Log2 Intensity 30 µM propionate/ 6 µM butyrate	Rinm5f Mean Log2 Intensity 60 µM propionate/ 12 µM butyrate	Rinm5f Mean Log2 Intensity 120 µM propionate/ 24 µM butyrate	Rinm5f Mean Log2 Intensity 240 µM propionate/ 48 µM butyrate	Rinm5f Mean Log2 Intensity 1500 µM propionate /300 µM butyrate	Rinm5f Mean Log2 Intensity 500 µM propionate/ 500 µM butyrate
1395748_at	---	1	0	1	0	0	0	6.44	6.25	5.81	6.19	6.30	0	0	0	0	0	0	0	6.68	6.62	6.61	6.54	6.51	6.68	6.47
1395758_at	Syncrip	1	0	0	1	0	0	4.54	4.23	4.63	3.98	4.19	0	0	0	0	0	0	0	5.69	5.61	5.73	5.89	5.63	5.24	4.33
1395772_at	---	1	1	0	0	0	1	3.32	3.80	3.75	3.19	3.88	0	0	0	0	0	0	0	3.16	3.19	3.24	3.16	3.26	3.19	2.69
1395779_at	Ccdc88a	1	0	1	0	1	0	4.65	4.92	5.36	4.51	5.10	0	0	0	0	0	0	0	7.99	8.09	8.27	8.48	8.46	8.56	7.95
1395799_at	Itsn2	1	0	1	1	1	0	5.62	5.39	6.03	4.77	5.44	0	0	0	0	0	0	0	8.42	8.32	8.45	8.46	8.33	8.21	6.70
1395806_at	LOC100909409 /	1	0	0	0	0	1	4.39	4.59	4.37	4.48	5.01	0	0	0	0	0	0	0	5.48	5.67	5.69	5.62	5.50	5.81	5.15
1395809_at	Sesn1	1	0	0	1	0	1	5.90	5.48	5.81	5.12	5.41	0	0	0	0	0	0	0	7.04	7.14	7.00	6.96	6.90	7.26	6.79
1395820_at	Rnf150	1	0	0	0	0	1	5.08	5.36	5.43	5.41	5.66	0	0	0	0	0	0	0	5.65	5.57	5.47	5.54	5.37	5.28	5.68
1395821_at	Cbfb	1	0	1	1	1	0	5.27	5.44	6.66	4.30	5.30	0	0	0	0	0	0	0	7.39	7.44	7.55	7.76	7.68	7.89	6.24
1395840_at	Eif1ax	1	0	1	1	1	0	5.48	5.41	6.33	4.41	5.60	0	0	0	0	0	0	0	8.53	8.66	8.64	8.91	8.86	8.58	7.19
1395850_at	Pus3	1	0	1	0	1	0	3.58	3.62	4.12	3.30	3.56	0	0	0	0	0	0	0	7.07	7.16	7.27	7.40	7.33	7.31	6.91
1395855_at	Arih2	1	0	0	1	1	0	5.12	4.85	5.50	4.37	4.75	0	0	0	0	0	0	0	7.52	7.56	7.70	7.67	7.59	7.55	6.41
1395886_at	Actr3	1	0	0	1	1	0	7.49	7.43	7.90	6.06	7.38	0	0	0	0	0	0	0	9.51	9.49	9.53	9.56	9.48	9.36	8.35
1395894_at	Api5	1	0	0	1	1	0	6.03	5.91	6.58	4.85	5.92	0	0	0	0	0	0	0	8.88	8.82	8.88	9.03	9.01	9.29	8.52
1395896_at	Zbtb21	1	0	0	0	0	1	3.87	3.78	4.30	4.01	4.44	0	0	0	0	0	0	0	7.46	7.56	7.60	7.69	7.72	7.29	6.83
1395900_at	Chtf8	1	0	1	0	1	0	4.88	5.06	5.53	4.59	4.73	0	0	0	0	0	0	0	7.72	7.73	7.81	7.70	7.57	7.50	6.38
1395907_at	Mamstr	1	0	0	1	1	0	4.28	4.33	4.00	5.88	4.21	0	0	0	0	0	0	0	5.38	5.22	5.20	4.99	5.10	5.20	5.17
1395914_at	Csnk1d	1	0	0	0	1	0	6.59	6.30	7.08	6.16	6.59	0	0	0	0	0	0	0	9.49	9.43	9.58	9.63	9.59	9.56	8.74
1395921_at	Usp35	1	0	0	0	0	1	5.91	6.26	6.24	5.89	6.45	0	0	0	0	0	0	0	8.22	8.33	8.34	8.32	8.42	8.32	8.11
1395923_at	Nipbl	1	0	1	0	0	0	6.11	6.05	6.64	6.03	6.38	0	0	0	0	0	0	0	8.05	7.97	8.11	8.15	8.00	8.08	7.97
1395965_at	---	1	0	1	0	1	1	5.93	6.04	6.79	5.94	6.58	0	0	0	0	0	0	0	7.79	7.83	7.90	8.01	8.11	8.19	7.58
1395985_at	Spg7	1	0	0	0	1	0	4.34	4.17	4.75	3.92	4.21	0	0	0	0	0	0	0	7.29	7.53	7.48	7.52	7.50	7.52	6.24
1395991_at	Rimbp2	1	0	1	0	1	0	9.16	9.22	9.87	8.68	9.30	0	0	0	0	0	0	0	10.32	10.36	10.41	10.45	10.50	9.93	9.01
1395998_at	Nop56	1	0	0	0	1	0	6.05	5.96	6.45	5.72	6.05	0	0	0	0	0	0	0	10.13	10.16	10.25	10.39	10.34	9.98	8.66
1396009_at	Prdm16	1	0	0	1	1	0	4.62	4.47	4.50	5.69	4.58	0	0	0	0	0	0	0	3.36	3.43	3.42	3.33	3.28	3.36	2.86
1396018_at	Cul5	1	0	0	0	1	0	4.10	4.13	4.55	3.75	4.10	0	0	0	0	0	0	0	5.35	5.23	5.55	5.66	5.40	5.02	4.36
1396024_at	Cyp3a23/3a1 ///	1	0	1	1	1	0	5.29	5.17	5.94	4.65	4.97	0	0	0	0	0	0	0	7.60	7.75	7.82	7.86	7.77	7.67	6.34
1396049_x_at	Rpl3	1	0	0	1	0	1	6.74	6.67	6.80	7.30	6.25	0	0	0	0	0	0	0	7.01	7.07	7.01	6.99	7.07	6.99	6.47
1396058_at	Igip	1	0	0	0	1	0	7.02	7.05	7.50	6.75	7.16	0	0	0	0	0	0	0	7.64	7.65	7.72	7.68	7.62	7.60	7.26
1396065_at	LOC103694852	1	0	0	0	1	1	10.89	10.63	10.45	11.23	10.17	0	0	0	0	0	0	0	3.46	3.43	3.48	3.44	3.41	3.51	2.98
1396071_at	---	1	0	0	1	0	0	3.93	3.70	3.94	3.31	3.68	0	0	0	0	0	0	0	4.04	3.96	4.20	4.05	4.08	4.19	3.82
1396072_at	Appbp2	1	0	0	0	1	0	3.78	3.72	4.25	3.48	3.94	0	0	0	0	0	0	0	5.42	5.17	5.36	5.52	5.38	5.29	5.24
1396077_at	LOC100909830 /	1	0	0	0	1	0	4.92	4.89	5.48	4.48	5.01	0	0	0	0	0	0	0	8.14	8.16	8.24	8.35	8.37	8.47	7.85
1396078_at	Krit1	1	1	0	0	0	0	3.82	4.34	4.29	4.09	4.13	0	0	0	0	0	0	0	6.61	6.59	6.61	6.75	6.70	6.43	5.65
1396081_at	Spire1	1	0	0	0	1	0	5.43	5.58	6.00	5.20	5.72	0	0	0	0	0	0	0	8.03	7.84	7.97	8.05	8.01	8.23	7.73
1396086_at	Tmem97	1	0	1	0	1	0	10.74	10.63	9.97	10.76	10.39	0	0	0	0	0	0	0	9.46	9.51	9.46	9.52	9.51	9.45	8.97
1396119_at	---	1	0	0	0	1	0	5.89	5.80	6.36	5.51	6.05	0	0	0	0	0	0	0	6.73	7.00	7.09	7.17	7.18	7.04	6.86
1396128_at	Gpbp1	1	0	0	1	1	0	6.29	6.23	6.54	5.74	6.06	0	0	0	0	0	0	0	9.02	9.07	9.06	9.09	9.06	9.06	8.70
1396140_at	Lrrc39	1	0	0	0	0	1	4.95	4.77	4.93	4.71	4.42	0	0	0	0	0	0	0	4.64	4.54	4.54	4.28	4.32	4.30	4.61
1396141_at	---	1	0	0	0	1	0	5.70	5.66	6.23	5.46	6.01	0	0	0	0	0	0	0	4.37	4.26	4.20	4.35	4.30	4.10	3.82
1396171_at	Ppil4	1	0	1	0	1	0	4.71	4.30	5.36	4.33	4.75	0	0	0	0	0	0	0	7.51	7.58	7.67	7.77	7.77	7.60	6.55
1396175_at	Zfp62	1	0	1	1	1	0	4.18	4.43	4.94	3.67	4.03	0	0	0	0	0	0	0	5.95	5.99	6.06	6.14	6.19	5.80	4.78
1396178_at	LOC102546688	1	0	0	1	1	0	5.54	5.46	5.45	6.24	5.17	0	0	0	0	0	0	0	5.03	5.14	5.01	4.88	5.14	5.44	5.33
1396203_at	Mios	1	0	0	0	0	1	4.91	4.73	4.59	4.83	4.44	0	0	0	0	0	0	0	6.24	6.23	6.21	6.39	6.19	5.94	5.85
1396207_at	Rdx	1	0	1	0	1	0	7.05	6.81	7.78	7.05	7.35	0	0	0	0	0	0	0	7.80	7.59	7.68	7.77	7.48	7.36	6.54
1396238_at	Galnt14	1	0	0	0	0	1	5.33	5.37	5.28	4.98	5.92	0	0	0	0	0	0	0	7.98	8.03	8.12	8.05	8.24	8.02	7.54
1396252_at	---	1	0	0	0	0	1	9.13	9.15	9.34	8.78	9.73	0	0	0	0	0	0	0	11.41	11.73	11.87	11.71	11.86	11.57	10.19
1396257_at	---	1	0	0	0	1	0	6.51	6.67	6.85	6.09	6.74	0	0	0	0	0	0	0	8.17	8.27	8.45	8.44	8.55	8.53	8.54
1396267_at	Pak2	1	0	1	0	1	0	5.29	5.41	6.10	4.82	5.60	0	0	0	0	0	0	0	7.76	7.74	7.82	8.00	7.85	7.55	6.72
1396274_at	Iqcc	1	0	0	0	0	1	6.22	6.11	6.46	6.14	5.48	0	0	0	0	0	0	0	6.55	6.58	6.67	6.61	6.63	6.44	5.99
1396279_at	Atp6ap2	1	0	1	1	1	0	7.80	7.95	8.89	6.47	8.04	0	0	0	0	0	0	0	9.88	9.93	9.88	9.90	9.92	9.94	9.19
1396317_at	Ccpg1	1	0	0	1	1	0	8.19	8.37	8.05	8.67	7.79	0	0	0	0	0	0	0	7.86	7.99	8.09	8.15	8.26	8.31	8.10
1396409_at	Dtnb	1	1	0	1	1	1	4.96	4.50	4.96	4.24	4.40	0	0	0	0	0	0	0	6.70	6.60	6.82	6.75	6.69	6.93	6.04
1396420_at	---	1	0	0	0	0	1	4.75	4.77	4.96	4.81	3.64	0	0	0	0	0	0	0	4.38	4.26	4.60	4.30	4.44	4.17	3.48
1396450_at	Enah	1	0	0	0	0	1	7.79	7.96	8.07	7.92	8.49	0	0	0	0	0	0	0	6.53	6.40	6.41	6.48	6.33	6.49	6.43
1396470_at	---	1	0	1	0	0	0	6.26	6.14	5.53	6.04	5.83	0	0	0	0	0	0	0	5.56	5.58	5.74	5.57	5.51	5.61	5.83
1396502_at	---	1	0	0	1	0	0	4.92	5.02	5.07	5.44	5.20	0	0	0	0	0	0	0	5.78	5.83	5.97	5.70	5.79	5.87	5.57
1396541_at	Jmjd1c	1	0	1	0	0	0	4.05	4.26	4.49	4.30	3.90	0	0	0	0	0	0	0	4.89	4.71	4.99	4.83	4.90	4.92	4.57
1396543_at	Ncstn	1	0	0	0	1	0	5.21	5.15	5.59	4.87	5.11	0	0	0	0	0	0	0	7.55	7.58	7.62	7.57	7.62	7.51	6.30
1396559_at	---	1	1	0	1	0	0	4.35	4.93	4.52	4.99	4.47	0	0	0	0	0	0	0	5.55	5.48	5.51	5.56	5.36	5.57	3.92
1396610_at	---	1	0	1	0	0	0	6.21	6.53	5.69	6.20	6.00	0	0	0	0	0	0	0	6.17	6.39	6.40	5.96	5.97	5.98	5.75
1396734_at	---	1	0	1	0	0	0	4.65	4.49	3.87	4.36	4.22	0	0	0	0	0	0	0	4.19	4.19	4.12	4.09	3.99	4.07	3.55
1396742_at	Ipo5	1	0	0	1	1	1	4.39	4.33	4.69	3.23	3.76	0	0	0	0	0	0	0	7.03	7.03	7.23	7.42	7.23	7.27	5.97
1396749_at	---	1	0	0	1	1	0	4.57	4.42	4.28	5.05	4.48	0	0	0	0	0	0	0	5.16	5.09	5.32	5.31	5.24	4.81	4.26
1396754_at	---	1	0	0	1	0	0	3.90	4.23	4.09	4.59	4.33	0	0	0	0	0	0	0	5.42	5.37	5.37	5.33	5.14	5.18	4.96
1396757_at	---	1	0	1	0	0	0	4.89	4.84	4.30	4.78	4.51	0	0	0	0	0	0	0	5.08	5.12	5.08	4.77	4.84	4.84	4.95
1396760_at	---	1	0	0	0	0	1	4.25	4.45	4.23	4.29	3.71	0	0	0	0	0	0	0	4.03	3.99	4.11	4.02	3.97	3.95	4.03
1396766_at	Ift81	1	0	0	0	1	0	3.77	3.75	4.26	3.41	3.87	0	0	0	0	0	0	0	6.76	6.81	6.87	6.97	6.92	6.78	6.33
1396773_at	---	1	0	0	1	0	0	6.30	6.48	6.39	6.88	6.22	0	0	0	0	0	0	0	7.25	7.25	7.50	7.39	7.30	6.91	6.93
1396775_at	---	1	0	0	0	0	1	4.75	5.16	5.22	4.60	6.01	0	0	0	0	0	0	0	6.39	6.61	6.67	6.36	6.50	5.91	5.75
1396791_at	---	1	0	1	0	0	0	4.36	4.21	3.79	3.90	3.96	0	0	0	0	0	0	0	4.00	4.16	4.16	4.01	4.06	4.16	3.65
1396828_at	---	1	0	1	0	0	0	5.10	4.96	4.54	4.80	4.97	0	0	0	0	0	0	0	5.32	5.46	5.47	5.45	5.35	5.41	4.79
1396831_at	---	1	0	1	0	1	1	5.78	6.17	6.48	5.43	5.03	0	0	0	0	0	0	0	8.54	8.53	8.70	8.48	8.63	8.50	7.45
1396892_at	---	1	0	0	0	1	0	5.17	5.20	4.88	5.52	4.85	0	0	0	0	0	0	0	5.71	5.68	5.59	5.56	5.55	5.75	5.59
1396917_at	---	1	0	0	0	0	1	5.88	6.01	5.98	5.76	5.27	0	0	0	0	0	0	0	5.90	5.73	5.76	5.57	5.60	5.68	4.71
1396938_at	---	1	0	0	0	1	0	5.06	5.10	4.59	5.19	4.93	0	0	0	0	0	0	0	4.78	4.90	4.83	4.66	4.64	4.74	4.78
1396957_at	---	1	0	1	0	0	0	5.88	6.06	6.59	5.93	5.62	0	0	0	0	0	0	0	6.40	6.45	6.76	6.74	6.67	6.76	6.28
1396963_at	---	1	0	1	0	1	1	4.64	4.96	5.25	4.53	5.44	0	0	0	0	0	0	0	6.01	6.11	6.34	6.41	6.39	6.22	5.90
1396983_at	---	1	0	0	1	1	0	6.80	6.78	7.02	6.21	6.99	0	0	0	0	0	0	0	6.03	6.05	6.30	6.33	6.27	6.18	5.70
1397184_at	Kiaa0408	1	0	0	0	0	1	6.26	6.54	6.61	6.13	7.45	0	0	0	0	0	0	0	7.63	7.69	7.76	7.87	7.82	7.75	7.78
1397190_at	---	1	0	1	0	0	0	3.91	3.68	3.39	3.66	3.74	0	0	0	0	0	0	0	3.65	3.73	3.74	3.61	3.68	3.68	3.26
1397200_at	Chd4	1	0	0	1	0	0	7.69	7.87	7.88	8.20	8.09	0	0	0	0	0	0	0	8.94	8.82	8.97	8.98	8.91	8.88	8.87
1397203_at	Prpf4b	1	0	0	1	0	0	8.46	8.56	8.75	8.97	8.63	0	0	0	0	0	0	0	10.13	9.96	10.11	10.06	10.03	10.04	9.72
1397204_s_at	---	1	0	1	0	1	0	5.37	5.26	4.79	5.43	5.13	0	0	0	0	0	0	0	6.47	6.53	6.76	6.77	6.87	6.91	6.40
1397211_at	Grb10	1	0	0	1	1	0	6.40	6.00	6.10	6.89	6.05	0	0	0	0	0	0	0	3.71	3.55	3.61	3.49	3.45	3.60	3.16
1397226_at	Dock4	1	0	0	0	0	1	3.66	3.50	3.96	3.48	4.22	0	0	0	0	0	0	0	6.59	6.44	6.83	6.83	6.78	6.95	7.12
1397227_at	Tmem35a	1	1	0	0	0	0	7.19	7.70	7.65	7.11	7.24	0	0	0	0	0	0	0	3.59	3.67	3.60	3.52	3.51	3.51	3.12
1397229_at	---	1	1	0	0	0	0	3.12	3.66	3.26	3.48	3.07	0	0	0	0	0	0	0	3.80	3.74	3.91	3.83	3.70	3.85	2.97
1397231_at	Sprn	1	0	1	0	0	1	7.05	6.86	6.52	6.86	6.59	0	0	0	0	0	0	0	6.07	6.25	6.16	6.30	6.27	6.15	5.21
1397245_at	Rad23a	1	0	0	1	1	0	6.21	5.95	6.35	5.57	5.94	0	0	0	0	0	0	0	7.76	7.81	7.92	7.86	7.87	7.91	7.24
1397255_at	Ccdc86	1	0	1	0	1	0	7.39	7.27	8.07	6.98	7.18	0	0	0	0	0	0	0	8.93	8.91	9.08	9.23	9.00	8.89	8.21
1397258_at	---	1	0	0	1	0	0	5.33	5.35	5.41	5.84	5.74	0	0	0	0	0	0	0	6.67	6.66	6.74	6.66	6.76	6.70	6.53
1397268_at	Slc17a4	1	0	0	0	0	1	4.60	4.76	4.45	4.49	5.50	0	0	0	0	0	0	0	6.24	6.43	6.63	6.71	6.75	6.49	5.78
1397274_at	---	1	0	0	1	0	0	3.94	3.60	3.64	3.44	3.66	0	0	0	0	0	0	0	6.71	6.61	6.77	6.88	6.73	6.67	6.16
1397288_at	Nudt21	1	0	1	1	1	0	4.48	4.34	5.20	3.89	4.29	0	0	0	0	0	0	0	6.23	6.30	6.44	6.73	6.74	6.79	5.79
1397300_at	Abcc4	1	0	0	0	0	1	5.92	6.04	6.42	6.06	6.95	0	0	0	0	0	0	0	8.55	8.50	8.65	8.69	8.78	8.98	9.10
1397304_at	Igtp	1	1	0	0	0	0	4.79	5.58	4.79	4.79	5.02	0	0	0	0	0	0	0	4.46	4.47	4.54	4.55	4.76	4.79	5.01
1397307_at	---	1	0	0	1	0	0	5.29	5.01	5.13	4.66	5.30	0	0	0	0	0	0	0	6.97	6.59	6.82	6.91	6.81	6.78	6.66
1397313_at	Pnrc2	1	0	0	1	1	0	7.60	7.48	7.97	6.77	7.38	0	0	0	0	0	0	0	9.34	9.30	9.28	9.33	9.31	8.91	7.85
1397343_at	Prdm16	1	0	0	1	1	0	5.39	5.29	5.14	5.98	5.19	0	0	0	0	0	0	0	4.05	3.93	4.05	3.97	4.00	4.07	4.97
1397350_at	Endov	1	0	1	0	0	0	5.78	5.63	5.20	5.52	5.37	0	0	0	0	0	0	0	5.95	5.90	5.91	5.76	5.87	5.76	5.32
1397361_x_at	---	1	0	1	1	1	0	8.68	8.22	6.78	7.54	8.44	0	0	0	0	0	0	0	4.02	4.06	4.14	3.96	4.04	4.26	3.87
1397362_at	---	1	0	0	0	0	1	5.70	5.83	5.98	6.06	6.22	0	0	0	0	0	0	0	8.28	8.40	8.46	8.52	8.50	8.27	7.39
1397380_at	---	1	0	0	0	0	1	5.61	5.44	6.16	5.69	6.12	0	0	0	0	0	0	0	6.42	6.44	6.61	6.38	6.33	6.60	6.31
1397384_at	---	1	0	0	1	0	0	5.78	5.97	5.82	6.26	6.05	0	0	0	0	0	0	0	6.60	6.62	6.70	6.57	6.69	6.93	6.13
1397386_at	Aff4	1	0	1	0	0	0	5.05	5.23	5.64	5.14	5.44	0	0	0	0	0	0	0	6.08	5.68	5.94	6.00	5.83	5.80	6.63
1397396_at	---	1	0	0	1	0	0	4.59	4.93	5.01	5.07	4.81	0	0	0	0	0	0	0	5.16	5.15	5.16	5.11	5.13	4.82	4.35
1397399_at	---	1	0	0	0	1	0	6.49	6.55	6.26	6.87	6.34	0	0	0	0	0	0	0	5.61	5.45	5.56	5.46	5.45	5.55	5.39
1397405_at	Ddx17	1	0	0	1	1	0	5.14	5.04	5.52	4.47	4.94	0	0	0	0	0	0	0	8.14	8.08	8.17	8.21	8.07	7.92	7.07
1397409_s_at	Wee1	1	0	1	0	0	0	6.59	6.14	7.17	6.88	6.92	0	0	0	0	0	0	0	8.90	8.86	8.96	9.07	8.90	8.67	8.40
1397415_at	Atl1	1	0	1	1	0	0	6.48	6.36	5.93	5.95	6.10	0	0	0	0	0	0	0	6.03	6.03	6.08	6.07	6.03	6.08	5.88
1397419_at	Mpp6	1	0	1	0	1	0	6.04	6.46	6.87	6.10	6.51	0	0	0	0	0	0	0	7.55	7.47	7.59	7.76	7.64	7.79	7.98
1397428_at	LOC103693658	1	1	0	0	0	0	3.45	3.96	3.68	3.86	3.79	0	0	0	0	0	0	0	4.20	4.17	4.20	4.22	4.05	4.35	4.00
1397439_at	Dgke	1	0	1	1	1	0	5.83	5.58	6.42	5.31	5.81	0	0	0	0	0	0	0	7.82	7.73	7.92	7.96	7.92	8.08	8.39
1397443_at	---	1	0	0	1	0	0	5.96	6.21	6.33	6.45	5.74	0	0	0	0	0	0	0	6.68	6.71	6.64	6.64	6.60	6.70	7.04
1397446_at	Rnf207	1	0	1	0	0	0	6.93	6.71	6.37	6.77	6.66	0	0	0	0	0	0	0	5.77	5.75	5.67	5.52	5.55	5.64	5.14
1397449_at	---	1	0	0	0	0	1	5.82	6.02	6.23	5.93	6.62	0	0	0	0	0	0	0	4.73	4.88	4.94	4.81	4.76	4.84	4.62
1397452_at	---	1	0	0	1	1	0	6.35	6.38	6.19	7.31	6.38	0	0	0	0	0	0	0	6.41	6.41	6.47	6.43	6.56	6.58	6.38
1397460_at	---	1	0	1	0	1	0	6.22	6.34	7.02	5.92	6.39	0	0	0	0	0	0	0	6.24	6.23	6.59	6.42	6.49	6.59	6.69
1397466_at	Eif3h	1	0	0	0	0	1	6.38	5.96	6.39	6.04	5.87	0	0	0	0	0	0	0	7.79	7.64	7.79	7.74	7.70	7.56	6.75
1397467_at	Znf329	1	0	1	0	1	0	5.74	5.91	6.37	5.60	6.12	0	0	0	0	0	0	0	6.87	6.93	7.08	7.15	7.06	7.08	6.53
1397503_at	---	1	0	0	0	1	0	4.08	4.46	3.88	4.48	3.87	0	0	0	0	0	0	0	5.55	5.67	5.86	5.69	5.65	5.44	5.01
1397512_at	Vps41	1	0	0	0	1	0	5.30	5.44	5.69	4.88	5.34	0	0	0	0	0	0	0	8.29	8.33	8.40	8.47	8.35	8.17	7.36
1397516_at	Alg2	1	0	1	1	1	0	6.47	6.82	7.21	5.47	6.54	0	0	0	0	0	0	0	8.65	8.58	8.81	8.93	8.82	8.69	7.29
1397519_at	Adipor2	1	0	0	1	1	0	7.41	7.33	7.82	6.78	7.33	0	0	0	0	0	0	0	8.93	8.91	8.97	8.92	8.86	9.07	8.61
1397523_at	Lrp11	1	0	1	0	1	0	8.04	8.33	8.82	7.66	8.32	0	0	0	0	0	0	0	10.39	10.38	10.37	10.33	10.31	10.54	10.06
1397525_at	Alkbh5	1	0	0	1	0	0	5.71	6.05	6.14	6.19	6.04	0	0	0	0	0	0	0	6.85	6.67	6.72	6.66	6.56	6.48	6.30
1397529_at	Ddx17	1	0	0	0	1	0	6.86	6.87	7.43	6.43	6.93	0	0	0	0	0	0	0	9.40	9.43	9.53	9.49	9.45	9.47	8.72
1397531_at	Dpy19l4	1	0	1	0	0	0	6.42	6.42	7.04	6.54	6.69	0	0	0	0	0	0	0	7.59	7.53	7.62	7.76	7.68	8.01	8.11
1397537_at	LOC102556259 /	1	0	1	0	0	0	6.57	6.17	5.88	6.30	6.43	0	0	0	0	0	0	0	11.10	11.16	11.14	11.06	11.08	10.70	10.52
1397540_at	---	1	0	0	1	1	0	6.28	6.02	6.16	7.09	5.92	0	0	0	0	0	0	0	4.90	5.08	5.07	5.15	5.22	5.15	5.04
1397552_at	Eml4	1	0	0	1	0	0	7.14	7.16	7.21	7.68	7.09	0	0	0	0	0	0	0	8.14	8.05	8.16	8.24	8.17	8.41	8.45
1397558_at	Tet3	1	1	0	0	0	0	5.87	5.30	5.80	6.31	5.69	0	0	0	0	0	0	0	6.70	6.77	6.92	7.04	6.97	6.57	6.35
1397564_at	---	1	1	1	0	1	1	5.15	4.59	4.37	5.22	4.62	0	0	0	0	0	0	0	4.55	4.52	4.61	4.57	4.50	4.56	4.00
1397569_at	---	1	1	1	1	1	0	5.66	6.27	6.39	5.14	5.23	0	0	0	0	0	0	0	5.35	5.22	5.24	5.24	5.36	5.40	4.49

Probeset ID	Gene Symbol	Islet Data Set (log2 ratio ≥0.263; FDR <20%) union n=5325 1=YES 0=NO	Islet DRlyp/lyp ND vs ND+Lp299v (log2 ratio ≥0.263; FDR<20%) n=429 1=YES 0=NO	Islet DRlyp/lyp ND vs HCD (log2 ratio ≥0.263; FDR<20%) n=2247 1=YES 0=NO	Islet DRlyp/lyp ND vs HCD+Lp299v (log2 ratio ≥0.263; FDR<20%) n=1595 1=YES 0=NO	Islet DRlyp/lyp HCD vs HCD+Lp299v (log2 ratio ≥0.263; FDR<20%) n=3026 1=YES 0=NO	Islet DRlyp/lyp ND vs Flyp/lyp ND (log2 ratio ≥ 0.263; FDR<20%) n=1950 1=YES 0=NO	Islet Mean Log2 Intensity DRlyp/lyp ND	Islet Mean Log2 Intensity DRlyp/lyp ND+Lp299v	Islet Mean Log2 Intensity DRlyp/lyp HCD	Islet Mean Log2 Intensity DRlyp/lyp HCD+Lp299v	Islet Mean Log2 Intensity Flyp40 ND	Rinm5f data union of all conditions n=3705 1=YES 0=NO	Rinm5f 30 µM propionate/ 6 µM butyrate vs Media (FDR<20%; 1.5 fold) n=3 1=YES 0=NO	Rinm5f 60 µM propionate/ 12 µM butyrate vs Media (FDR<20%; 1.5 fold) n=18 1=YES 0=NO	Rinm5f 120 µM propionate/ 24 µM butyrate vs Media (FDR<20%; 1.5 fold) n=164 1=YES 0=NO	Rinm5f 240 µM propionate /48 µM butyrate vs Media (FDR<20%; 1.5 fold) n=268 1=YES 0=NO	Rinm5f 1500 µM propionate/ 300 µM butyrate vs Media (FDR<20%; 1.5 fold) n=2491 1=YES 0=NO	Rinm5f 500 µM propionate/ 500 µM butyrate vs Media (FDR<20%; 1.5 fold) n=3001 1=YES 0=NO	Rinm5f Mean Log2 Intensity Media only	Rinm5f Mean Log2 Intensity 30 µM propionate/ 6 µM butyrate	Rinm5f Mean Log2 Intensity 60 µM propionate/ 12 µM butyrate	Rinm5f Mean Log2 Intensity 120 µM propionate/ 24 µM butyrate	Rinm5f Mean Log2 Intensity 240 µM propionate/ 48 µM butyrate	Rinm5f Mean Log2 Intensity 1500 µM propionate /300 µM butyrate	Rinm5f Mean Log2 Intensity 500 µM propionate/ 500 µM butyrate
1397581_at	Rhov	1	0	0	0	1	0	5.88	5.98	5.62	6.27	5.61	0	0	0	0	0	0	0	6.65	6.81	6.77	6.72	6.89	7.04	7.24
1397582_at	---	1	0	0	1	1	0	5.27	5.52	5.13	5.74	5.25	0	0	0	0	0	0	0	8.45	8.35	8.31	8.34	8.23	8.01	7.92
1397583_at	Os9	1	0	0	1	1	0	5.57	5.73	5.39	6.14	5.46	0	0	0	0	0	0	0	6.63	6.67	6.74	6.70	6.79	6.93	6.94
1397584_at	Rnf151	1	0	0	0	1	0	8.10	8.01	7.84	8.47	7.73	0	0	0	0	0	0	0	10.18	10.06	10.05	10.10	10.06	10.11	9.70
1397600_at	Vezt	1	0	1	0	0	0	3.67	3.89	4.25	3.64	3.71	0	0	0	0	0	0	0	7.57	7.66	7.77	7.78	7.75	7.64	7.43
1397611_at	LOC100909803	1	0	1	0	0	0	4.70	4.69	4.16	4.55	4.51	0	0	0	0	0	0	0	5.05	5.09	4.94	4.76	4.95	4.80	4.32
1397627_at	Diaph1	1	0	0	1	1	0	5.40	5.68	5.00	5.88	5.35	0	0	0	0	0	0	0	5.64	5.44	5.53	5.36	5.34	5.15	6.29
1397658_at	---	1	1	0	0	0	0	5.45	6.00	5.41	5.32	5.60	0	0	0	0	0	0	0	5.57	5.52	5.65	5.53	5.56	5.55	5.11
1397669_at	Cep43	1	0	0	0	1	0	3.68	3.88	4.25	3.48	3.61	0	0	0	0	0	0	0	6.60	6.51	6.62	6.55	6.61	6.02	5.13
1397675_at	Eif4h	1	0	0	0	1	1	5.69	5.50	5.95	5.25	5.09	0	0	0	0	0	0	0	9.53	9.66	9.67	9.78	9.68	9.77	8.64
1397693_at	Eif2s3	1	0	1	1	1	0	6.72	6.97	7.44	6.19	6.54	0	0	0	0	0	0	0	9.59	9.51	9.58	9.70	9.63	9.66	8.69
1397695_at	---	1	0	1	1	1	0	4.63	5.04	6.40	4.05	4.67	0	0	0	0	0	0	0	4.18	4.14	4.45	4.26	4.27	4.41	3.74
1397697_at	Eif4a2	1	0	0	1	1	0	7.32	7.14	7.85	6.44	6.92	0	0	0	0	0	0	0	9.45	9.55	9.65	9.83	9.90	9.48	8.49
1397698_at	---	1	0	0	0	1	0	7.09	6.93	7.54	6.76	7.32	0	0	0	0	0	0	0	6.85	6.78	7.01	7.17	7.07	7.20	7.35
1397700_x_at	---	1	1	1	1	1	0	7.21	7.70	8.69	6.59	7.56	0	0	0	0	0	0	0	3.01	3.06	3.09	2.99	3.10	3.13	2.74
1397706_at	Wrn	1	0	0	0	1	0	5.50	5.23	6.05	5.11	5.63	0	0	0	0	0	0	0	8.18	8.23	8.26	8.27	8.30	8.21	8.24
1397714_at	Relch	1	0	0	0	1	0	4.49	4.56	5.03	4.34	4.59	0	0	0	0	0	0	0	7.48	7.32	7.63	7.53	7.47	7.41	6.70
1397740_at	Sfxn1	1	0	0	0	0	1	7.92	7.53	7.96	7.91	7.19	0	0	0	0	0	0	0	8.87	8.86	9.03	9.11	9.08	8.88	8.37
1397741_at	Ankrd52	1	0	1	0	0	0	4.80	4.48	4.27	4.55	4.48	0	0	0	0	0	0	0	5.77	5.80	5.69	5.45	5.42	5.48	5.07
1397745_at	---	1	0	0	0	0	1	4.41	4.35	4.72	4.69	3.09	0	0	0	0	0	0	0	5.18	5.24	5.47	5.39	5.42	5.11	4.88
1397753_at	Pigq	1	0	1	0	0	1	6.64	6.48	6.07	6.42	6.18	0	0	0	0	0	0	0	7.04	7.06	7.04	6.87	7.06	6.83	6.11
1397766_at	---	1	0	1	0	0	0	8.14	8.30	8.74	8.20	8.58	0	0	0	0	0	0	0	8.97	8.97	8.75	8.70	8.70	8.52	8.51
1397779_at	Chd2	1	1	1	0	1	1	5.23	4.70	5.92	5.20	5.78	0	0	0	0	0	0	0	6.00	6.15	6.31	6.40	6.49	6.04	4.70
1397808_at	Ctsc	1	0	0	1	0	0	6.38	6.02	6.70	7.06	6.23	0	0	0	0	0	0	0	3.57	3.65	3.57	3.65	3.68	3.89	3.81
1397813_at	Gigyf2	1	0	1	0	1	0	5.20	5.15	5.72	4.85	4.97	0	0	0	0	0	0	0	6.93	6.75	6.98	6.89	6.68	6.68	6.49
1397815_at	Arl8b	1	0	0	1	1	0	7.39	7.50	7.79	6.30	7.36	0	0	0	0	0	0	0	9.82	9.79	9.81	9.95	9.91	10.00	9.36
1397824_at	Wac	1	0	1	1	1	0	7.11	7.10	7.73	6.41	6.97	0	0	0	0	0	0	0	8.17	8.09	8.15	8.32	8.22	8.03	7.76
1397828_at	Sphkap	1	0	1	0	0	0	7.27	7.71	7.85	7.36	7.55	0	0	0	0	0	0	0	7.52	7.59	7.87	7.75	7.89	7.55	7.04
1397834_at	Rdh13	1	0	1	0	0	0	6.70	6.69	6.11	6.40	6.46	0	0	0	0	0	0	0	6.87	6.99	7.04	6.95	6.92	6.95	6.13
1397843_at	Wdr44	1	0	1	1	1	0	3.53	3.65	4.23	3.05	3.64	0	0	0	0	0	0	0	4.57	4.64	4.81	5.12	4.93	4.93	4.59
1397848_at	Medag	1	1	0	0	0	0	3.65	4.39	3.99	4.01	3.59	0	0	0	0	0	0	0	3.70	3.89	4.10	3.99	3.99	3.82	3.26
1397852_at	Tada1	1	0	1	0	1	0	6.06	6.00	6.64	5.74	6.17	0	0	0	0	0	0	0	7.89	7.92	7.96	7.85	7.97	7.89	7.34
1397853_s_at	Arhgef9 /// LOC	1	0	1	0	1	0	4.98	5.17	5.63	4.80	5.09	0	0	0	0	0	0	0	6.70	6.65	6.75	6.65	6.62	6.90	6.84
1397866_at	Serpinb6b	1	0	0	1	0	0	5.54	5.58	5.62	6.19	5.69	0	0	0	0	0	0	0	3.96	3.80	3.96	3.75	3.71	3.82	3.61
1397871_at	---	1	0	1	0	1	1	5.63	5.76	6.40	5.46	6.40	0	0	0	0	0	0	0	7.09	7.18	7.18	7.13	7.19	6.94	6.72
1397879_at	---	1	0	0	0	0	1	4.97	5.30	5.20	4.99	5.45	0	0	0	0	0	0	0	5.53	5.43	5.47	5.45	5.51	5.45	4.89
1397884_at	Sel1l3	1	0	0	1	1	0	6.28	6.14	6.16	5.27	6.02	0	0	0	0	0	0	0	6.37	6.46	6.50	6.61	6.62	6.94	5.66
1397892_at	Eif1ax	1	0	1	1	1	0	7.38	7.42	8.30	6.77	7.85	0	0	0	0	0	0	0	9.67	9.72	9.82	9.95	9.91	9.73	8.45
1397897_x_at	LOC103690718	1	0	0	0	0	1	6.85	7.16	6.51	6.92	6.38	0	0	0	0	0	0	0	8.88	8.92	8.90	8.96	9.01	8.95	8.08
1397902_at	Cox15	1	0	1	1	1	0	5.87	5.59	6.50	5.38	5.60	0	0	0	0	0	0	0	8.50	8.55	8.59	8.66	8.64	8.57	7.75
1397906_at	---	1	0	1	0	0	0	7.45	7.36	6.91	7.35	7.23	0	0	0	0	0	0	0	3.71	3.66	3.68	3.52	3.52	3.57	3.56
1397907_at	Huwe1	1	0	0	0	0	1	4.85	4.99	5.26	5.26	5.47	0	0	0	0	0	0	0	6.82	7.05	7.23	7.27	7.25	7.07	6.65
1397917_at	Ctso	1	0	1	0	1	1	5.31	5.47	6.08	5.22	6.04	0	0	0	0	0	0	0	7.95	7.99	7.90	8.03	8.10	8.46	8.11
1397930_at	---	1	0	1	0	0	0	5.07	4.96	4.53	4.69	4.82	0	0	0	0	0	0	0	4.93	4.88	5.15	4.94	4.97	5.11	5.10
1397949_at	Mpp6	1	0	1	0	1	0	4.86	5.23	5.77	4.88	5.21	0	0	0	0	0	0	0	7.00	6.81	7.14	7.41	7.14	7.26	7.44
1397953_at	Stambpl1	1	0	0	1	0	0	6.52	6.22	6.75	7.19	6.46	0	0	0	0	0	0	0	9.50	9.54	9.62	9.72	9.72	9.46	8.89
1397964_at	Sec63	1	0	0	1	1	0	3.54	3.36	3.93	3.04	3.46	0	0	0	0	0	0	0	4.82	4.99	5.00	5.15	5.20	5.00	3.49
1398003_at	---	1	0	0	0	1	0	5.88	6.02	6.42	5.48	6.04	0	0	0	0	0	0	0	9.42	9.49	9.60	9.46	9.44	8.98	8.19
1398062_at	Smap2	1	1	0	0	0	0	6.18	6.65	6.16	6.48	6.27	0	0	0	0	0	0	0	6.79	6.67	6.96	7.03	7.01	7.07	6.50
1398089_at	---	1	0	0	0	0	1	7.33	7.40	6.99	7.35	6.69	0	0	0	0	0	0	0	6.72	6.75	6.78	6.60	6.63	6.85	6.96
1398118_at	---	1	0	1	0	0	0	5.42	5.42	4.89	5.25	5.28	0	0	0	0	0	0	0	6.13	6.16	6.01	5.97	5.98	5.96	5.52
1398191_at	---	1	0	0	0	1	0	4.10	4.24	4.54	3.77	4.18	0	0	0	0	0	0	0	5.99	5.88	6.35	6.32	6.06	5.55	5.12
1398196_at	---	1	0	1	0	0	0	5.31	5.29	4.74	5.12	5.06	0	0	0	0	0	0	0	4.61	4.83	4.58	4.57	4.54	4.62	4.31
1398216_at	---	1	0	1	0	0	0	5.49	5.65	6.18	5.54	5.70	0	0	0	0	0	0	0	5.36	5.41	5.73	5.56	5.32	5.63	4.85
1398217_at	Zbtb41	1	0	1	1	0	0	4.43	4.44	5.09	4.86	4.72	0	0	0	0	0	0	0	6.53	6.18	6.53	6.60	6.40	6.06	6.58
1398226_at	---	1	0	0	0	0	1	4.34	4.03	3.88	3.94	3.77	0	0	0	0	0	0	0	4.54	4.66	4.46	4.51	4.32	4.47	4.49
1398234_at	---	1	0	0	0	1	0	4.39	4.18	3.95	4.61	4.30	0	0	0	0	0	0	0	4.48	4.52	4.57	4.60	4.44	4.42	4.17
1398249_at	Slc25a20	1	0	0	0	1	0	7.75	7.49	8.26	7.28	7.57	0	0	0	0	0	0	0	8.52	8.53	8.70	8.91	8.89	9.10	8.70
1398262_at	Prps2	1	1	0	0	0	0	6.84	6.31	6.71	6.96	6.65	0	0	0	0	0	0	0	8.75	8.79	8.80	8.86	8.89	8.80	8.70
1398264_at	Slc30a2	1	0	1	1	1	1	7.01	6.58	5.36	6.22	5.87	0	0	0	0	0	0	0	4.53	4.63	4.59	4.51	4.46	4.48	4.63
1398272_at	B4galnt1	1	0	1	0	1	1	5.39	5.40	4.62	5.75	4.65	0	0	0	0	0	0	0	6.51	6.58	6.64	6.55	6.51	6.17	5.66
1398281_at	Ocln	1	0	0	1	1	1	7.44	7.44	7.32	6.63	6.94	0	0	0	0	0	0	0	7.03	7.12	7.20	7.30	7.30	7.47	7.08
1398305_at	Syt17	1	0	1	0	1	0	5.41	5.33	5.95	5.26	5.37	0	0	0	0	0	0	0	7.57	7.55	7.64	7.70	7.73	7.97	7.74
1398308_at	Rpa3	1	0	0	0	0	1	7.56	7.44	8.02	7.53	8.18	0	0	0	0	0	0	0	11.09	11.16	11.06	11.07	11.13	10.94	10.38
1398311_a_at	Kidins220	1	0	0	0	1	0	5.64	5.94	6.16	5.17	5.62	0	0	0	0	0	0	0	7.43	7.27	7.46	7.48	7.52	7.30	6.60
1398318_at	Muc1	1	0	1	0	1	0	9.89	9.96	8.74	10.27	9.48	0	0	0	0	0	0	0	5.60	5.80	5.66	5.47	5.66	5.90	5.24
1398325_at	Tspan3	1	0	1	0	1	0	9.86	9.81	10.58	9.46	10.23	0	0	0	0	0	0	0	11.63	11.67	11.61	11.57	11.60	11.70	11.37
1398329_at	---	1	0	1	0	0	0	5.47	5.37	4.86	5.31	5.17	0	0	0	0	0	0	0	5.65	5.70	5.45	5.39	5.51	5.62	4.87
1398339_at	Sf1	1	0	0	0	1	0	8.07	7.82	7.89	8.46	8.27	0	0	0	0	0	0	0	8.73	8.78	8.81	8.90	8.77	8.70	8.95
1398344_at	Med17	1	0	0	0	1	0	7.14	7.14	7.66	6.92	7.16	0	0	0	0	0	0	0	9.22	9.23	9.29	9.18	9.16	8.87	8.48
1398346_at	Mapk1	1	0	0	0	1	0	6.23	6.15	6.77	5.87	6.51	0	0	0	0	0	0	0	8.98	8.95	9.00	9.04	8.96	9.14	8.80
1398348_at	Ddah1	1	0	1	1	1	1	8.87	8.60	8.23	9.63	8.38	0	0	0	0	0	0	0	7.48	7.58	7.66	7.55	7.65	7.70	7.81
1398350_at	---	1	0	1	0	1	0	8.32	8.36	8.97	8.15	8.78	0	0	0	0	0	0	0	11.80	11.82	11.87	11.91	11.91	11.69	11.39
1398351_at	Usp7	1	0	0	1	0	0	8.13	8.15	8.39	8.64	8.44	0	0	0	0	0	0	0	8.76	8.64	8.75	8.70	8.63	8.47	9.31
1398354_at	Ctnnal1	1	0	1	0	0	0	6.31	6.32	6.92	6.36	6.28	0	0	0	0	0	0	0	7.01	7.00	7.04	7.13	7.07	6.88	6.71
1398355_at	Trpm7	1	0	1	0	1	0	7.99	7.79	8.73	8.01	8.34	0	0	0	0	0	0	0	9.05	9.20	9.13	9.12	9.24	9.33	8.66
1398364_at	Rsrp1	1	0	1	0	0	0	10.29	10.13	10.90	10.23	10.19	0	0	0	0	0	0	0	10.93	10.96	10.98	11.02	10.99	11.18	10.82
1398368_at	Unc80	1	0	1	0	1	1	8.48	8.70	9.25	8.02	9.07	0	0	0	0	0	0	0	8.13	8.16	8.25	8.37	8.34	8.15	8.17
1398370_at	Adarb1	1	0	0	0	1	0	5.59	5.23	5.99	5.23	6.00	0	0	0	0	0	0	0	8.68	8.72	8.74	8.72	8.77	8.87	8.98
1398379_at	Ess2	1	0	0	0	0	1	3.96	4.25	4.36	4.31	4.43	0	0	0	0	0	0	0	7.05	6.95	7.13	7.14	6.81	6.57	6.37
1398387_at	Gask1b	1	0	0	0	0	1	5.13	5.18	5.43	4.86	4.53	0	0	0	0	0	0	0	4.59	4.35	4.46	4.47	4.39	4.58	3.98
1398390_at	Cxcl13	1	0	1	0	0	1	4.35	4.54	3.74	4.13	7.57	0	0	0	0	0	0	0	3.62	3.68	3.77	3.60	3.63	3.72	3.07
1398396_at	RPS6KB1	1	1	0	0	0	1	7.84	7.25	7.77	7.62	7.38	0	0	0	0	0	0	0	8.47	8.45	8.56	8.60	8.62	8.44	8.06
1398406_at	Scrn1	1	0	1	0	0	1	6.18	6.48	6.77	6.19	6.95	0	0	0	0	0	0	0	6.69	6.73	6.97	7.11	7.13	6.77	6.90
1398409_at	St13	1	0	1	0	0	0	7.84	7.86	8.43	7.87	8.29	0	0	0	0	0	0	0	9.56	9.65	9.68	9.82	9.82	9.81	9.45
1398411_at	---	1	0	1	0	1	0	6.03	6.14	5.40	6.38	5.96	0	0	0	0	0	0	0	6.75	6.92	6.93	6.52	6.72	6.40	6.25
1398420_at	Smurf2	1	0	1	0	0	0	6.71	6.38	7.29	7.01	6.96	0	0	0	0	0	0	0	8.58	8.45	8.50	8.42	8.26	8.18	8.19
1398432_at	Ankrd28	1	0	0	0	1	0	7.04	6.97	7.56	6.77	7.40	0	0	0	0	0	0	0	8.88	8.91	8.99	9.00	9.02	9.05	9.31
1398433_at	Jph4	1	0	0	0	0	1	4.71	5.17	5.17	5.06	5.86	0	0	0	0	0	0	0	8.08	8.12	8.20	8.32	8.38	8.37	7.82
1398439_a_at	Orc6	1	0	1	0	1	1	3.95	4.04	4.73	3.80	4.52	0	0	0	0	0	0	0	7.40	7.38	7.56	7.69	7.61	7.19	6.72
1398440_at	Pnisr	1	0	1	0	1	0	6.75	6.84	7.58	6.58	7.03	0	0	0	0	0	0	0	8.63	8.78	8.84	9.05	9.14	8.99	8.17
1398445_at	---	1	0	1	0	1	0	4.81	5.06	5.49	4.56	4.74	0	0	0	0	0	0	0	8.81	8.79	8.88	8.68	8.78	8.38	7.26
1398448_at	---	1	0	0	0	0	1	7.55	7.73	8.01	7.79	8.08	0	0	0	0	0	0	0	6.86	6.74	6.97	7.09	6.98	7.10	7.40
1398455_at	Zdhhc13	1	0	1	0	0	0	6.44	6.47	7.09	6.45	6.75	0	0	0	0	0	0	0	9.11	9.06	9.11	9.16	9.16	9.03	8.98
1398473_at	Bloc1s2	1	0	0	0	0	1	5.79	5.73	5.99	5.74	6.27	0	0	0	0	0	0	0	10.35	10.35	10.33	10.35	10.31	10.09	9.81
1398486_at	Mia3	1	1	0	0	0	0	7.60	8.23	7.56	8.03	7.53	0	0	0	0	0	0	0	7.40	7.30	7.39	7.31	7.32	7.50	7.33
1398493_at	Pikfyve	1	0	1	0	0	0	6.66	6.71	7.34	6.67	6.83	0	0	0	0	0	0	0	8.39	8.43	8.37	8.40	8.44	8.38	8.17
1398500_at	Adgrf5	1	1	0	1	0	0	5.81	5.34	5.55	5.16	5.59	0	0	0	0	0	0	0	3.28	3.23	3.43	3.30	3.21	3.29	2.89
1398522_at	Ankle2	1	0	1	1	0	0	6.63	6.63	7.21	7.10	7.02	0	0	0	0	0	0	0	8.10	7.86	8.01	8.13	8.02	7.89	7.45
1398535_at	Hbs1l	1	0	0	0	0	1	6.83	6.58	6.38	6.83	5.98	0	0	0	0	0	0	0	7.97	8.03	7.95	7.87	7.80	7.69	7.31
1398536_at	Znf655	1	0	0	1	1	0	5.04	5.04	5.51	4.35	4.97	0	0	0	0	0	0	0	7.11	7.11	7.20	7.43	7.26	7.09	5.72
1398538_at	Abhd8	1	0	0	0	1	0	5.06	5.06	5.56	4.73	5.10	0	0	0	0	0	0	0	10.16	10.22	10.30	10.35	10.32	10.42	9.44
1398548_at	---	1	0	0	1	0	0	3.45	3.52	3.79	4.03	3.42	0	0	0	0	0	0	0	4.72	4.40	4.84	4.50	4.71	4.67	4.94
1398549_at	Ccnl2	1	0	0	1	1	0	7.11	7.11	7.66	6.26	6.90	0	0	0	0	0	0	0	7.89	7.86	7.89	7.84	7.89	7.82	6.81
1398552_a_at	Acrbp	1	0	1	0	1	0	7.21	7.36	6.68	7.28	7.00	0	0	0	0	0	0	0	6.07	6.13	6.10	5.99	5.99	6.06	5.67
1398557_at	Ikzf5	1	0	0	0	1	0	5.48	5.50	5.85	5.12	5.52	0	0	0	0	0	0	0	6.87	6.98	7.01	7.08	7.04	6.93	6.52
1398565_at	---	1	0	0	1	0	0	5.21	5.65	5.70	5.87	5.26	0	0	0	0	0	0	0	5.19	5.00	5.19	5.13	5.18	4.88	5.96
1398566_at	---	1	1	1	1	0	0	5.53	6.25	4.51	4.78	5.31	0	0	0	0	0	0	0	7.25	7.47	7.68	7.49	7.53	7.66	5.84
1398571_x_at	LOC102555503	1	0	1	0	0	0	8.27	8.13	7.70	8.17	8.23	0	0	0	0	0	0	0	5.96	6.00	6.02	5.75	5.81	5.82	5.78
1398575_at	Zbtb5	1	0	0	1	0	0	5.96	6.22	6.42	6.46	6.32	0	0	0	0	0	0	0	6.55	6.57	6.67	6.54	6.49	6.33	6.36
1398583_at	Snapc5	1	0	0	1	0	0	4.80	4.74	4.89	4.25	4.60	0	0	0	0	0	0	0	7.05	7.01	7.15	7.18	7.08	7.16	6.14
1398599_at	Chek1 /// Stt3a	1	0	0	0	0	1	6.45	6.76	7.01	6.61	7.19	0	0	0	0	0	0	0	9.26	9.30	9.29	9.34	9.34	9.28	8.39
1398600_at	---	1	0	1	0	1	1	6.12	6.24	6.92	6.02	6.97	0	0	0	0	0	0	0	9.06	9.07	9.04	9.08	9.08	9.19	9.38
1398601_at	Spire1	1	0	1	0	1	1	6.61	6.72	7.27	6.27	7.25	0	0	0	0	0	0	0	9.40	9.44	9.46	9.46	9.47	9.66	9.49
1398606_at	Golim4	1	0	0	0	1	0	8.20	8.11	8.63	7.92	8.35	0	0	0	0	0	0	0	9.75	9.67	9.74	9.86	9.87	10.10	9.83
1398611_at	Cul4b	1	0	1	0	1	0	4.44	4.58	5.34	4.24	4.63	0	0	0	0	0	0	0	7.65	7.69	7.81	8.06	7.96	8.07	7.41
1398612_at	Akr1c12	1	0	0	0	0	1	6.49	6.65	6.78	6.74	7.56	0	0	0	0	0	0	0	7.96	7.95	8.09	8.25	8.23	8.49	8.58
1398616_at	Ap1s2	1	0	1	1	1	0	9.83	9.85	10.60	9.25	10.16	0	0	0	0	0	0	0	13.33	13.41	13.44	13.50	13.56	13.84	13.45
1398617_at	Rps27a	1	0	0	1	0	0	5.66	5.29	5.92	6.25	5.69	0	0	0	0	0	0	0	8.42	8.44	8.50	8.61	8.54	8.40	7.67
1398621_at	Ak7	1	0	1	0	1	0	4.54	4.61	3.87	4.59	4.25	0	0	0	0	0	0	0	3.74	3.73	3.69	3.71	3.74	3.79	3.31
1398625_at	Zcchc18	1	0	0	0	1	0	11.36	11.64	11.76	10.99	11.66	0	0	0	0	0	0	0	11.61	11.64	11.82	12.06	12.01	12.10	11.53
1398633_at	---	1	0	0	0	0	1	6.53	6.65	6.89	6.70	7.03	0	0	0	0	0	0	0	7.68	7.52	7.67	7.75	7.69	7.25	7.07
1398640_at	Rab30	1	1	1	1	0	1	6.43	5.89	5.49	5.61	5.91	0	0	0	0	0	0	0	6.22	6.16	6.17	6.02	6.13	6.04	5.92
1398641_at	Plekha3	1	0	1	0	1	0	4.74	5.01	5.36	4.60	4.95	0	0	0	0	0	0	0	6.90	6.92	6.94	7.03	7.05	6.95	6.94
1398644_at	Plxnc1	1	0	0	0	0	1	4.71	4.98	4.98	4.67	5.60	0	0	0	0	0	0	0	6.04	6.11	6.27	6.33	6.33	6.50	6.43
1398652_at	Bpnt2	1	0	0	0	1	0	7.08	7.12	7.60	6.65	6.93	0	0	0	0	0	0	0	9.88	9.85	9.92	9.96	9.88	9.77	9.63
1398698_at	---	1	0	0	0	0	1	6.92	7.03	6.58	6.85	6.44	0	0	0	0	0	0	0	5.97	6.01	6.00	6.00	5.93	5.92	5.16
1398707_at	LOC102555246	1	0	0	0	0	1	3.49	3.74	3.73	3.82	4.09	0	0	0	0	0	0	0	10.08	10.13	10.12	10.04	10.12	10.26	10.23
1398716_at	---	1	1	1	1	1	1	7.09	7.65	8.58	6.41	7.62	0	0	0	0	0	0	0	3.12	3.01	3.08	3.00	3.04	3.18	2.74
1398727_at	---	1	0	0	1	1	0	6.11	5.76	6.10	7.26	5.69	0	0	0	0	0	0	0	4.30	4.12	4.32	4.29	4.39	4.50	5.07
1398729_s_at	Nom1	1	1	1	0	0	0	4.87	5.25	5.19	4.87	4.79	0	0	0	0	0	0	0	7.37	7.11	7.26	7.32	7.01	6.87	7.16
1398730_at	Srrt	1	0	0	0	0	1	5.54	5.50	5.46	5.34	4.89	0	0	0	0	0	0	0	7.99	8.06	8.20	8.21	8.18	7.85	7.06
1398734_at	---	1	0	0	0	1	0	5.30	5.37	5.07	5.64	5.27	0	0	0	0	0	0	0	5.30	5.20	5.27	5.21	5.14	5.13	4.53
1398750_at	Calr	1	0	0	0	0	1	9.72	10.01	10.11	10.18	10.43	0	0	0	0	0	0	0	11.54	11.56	11.59	11.57	11.63	11.67	10.85
1398754_at	Uba52	1	0	0	1	0	0	11.66	11.76	11.84	12.26	11.37	0	0	0	0	0	0	0	13.09	13.05	13.04	13.08	13.08	13.12	12.80

Probeset ID	Gene Symbol	Islet Data Set (log2 ratio ≥0.263; FDR <20%) union n=5325 1=YES 0=NO	Islet DRlyp/lyp ND vs ND+Lp299v (log2 ratio ≥0.263; FDR<20%) n=429 1=YES 0=NO	Islet DRlyp/lyp ND vs HCD (log2 ratio ≥0.263; FDR<20%) n=2247 1=YES 0=NO	Islet DRlyp/lyp ND vs HCD+Lp299v (log2 ratio ≥0.263; FDR<20%) n=1595 1=YES 0=NO	Islet DRlyp/lyp HCD vs HCD+Lp299v (log2 ratio ≥0.263; FDR<20%) n=3026 1=YES 0=NO	Islet DRlyp/lyp ND vs Flyp/lyp ND (log2 ratio ≥ 0.263; FDR<20%) n=1950 1=YES 0=NO	Islet Mean Log2 Intensity DRlyp/lyp ND	Islet Mean Log2 Intensity DRlyp/lyp ND+Lp299v	Islet Mean Log2 Intensity DRlyp/lyp HCD	Islet Mean Log2 Intensity DRlyp/lyp HCD+Lp299v	Islet Mean Log2 Intensity Flyp40 ND	Rinm5f data union of all conditions n=3705 1=YES 0=NO	Rinm5f 30 µM propionate/6 µM butyrate vs Media (FDR<20%; 1.5 fold) n=3 1=YES 0=NO	Rinm5f 60 µM propionate/12 µM butyrate vs Media (FDR<20%; 1.5 fold) n=18 1=YES 0=NO	Rinm5f 120 µM propionate/24 µM butyrate vs Media (FDR<20%; 1.5 fold) n=164 1=YES 0=NO	Rinm5f 240 µM propionate/48 µM butyrate vs Media (FDR<20%; 1.5 fold) n=268 1=YES 0=NO	Rinm5f 1500 µM propionate/300 µM butyrate vs Media (FDR<20%; 1.5 fold) n=2491 1=YES 0=NO	Rinm5f 500 µM propionate/500 µM butyrate vs Media (FDR<20%; 1.5 fold) n=3001 1=YES 0=NO	Rinm5f Mean Log2 Intensity Media only	Rinm5f Mean Log2 Intensity 30 µM propionate/6 µM butyrate	Rinm5f Mean Log2 Intensity 60 µM propionate/12 µM butyrate	Rinm5f Mean Log2 Intensity 120 µM propionate/24 µM butyrate	Rinm5f Mean Log2 Intensity 240 µM propionate/48 µM butyrate	Rinm5f Mean Log2 Intensity 1500 µM propionate/300 µM butyrate	Rinm5f Mean Log2 Intensity 500 µM propionate/500 µM butyrate
1398756_at	Npm1	1	0	0	1	1	0	7.53	7.37	7.84	8.41	7.21	0	0	0	0	0	0	0	10.10	10.13	10.14	10.31	10.22	9.87	9.24
1398764_at	Rpl21	1	0	0	1	0	0	11.82	11.73	11.98	12.32	11.58	0	0	0	0	0	0	0	12.31	12.34	12.27	12.27	12.28	12.22	12.05
1398788_at	Pdia3	1	0	0	0	0	1	10.92	11.21	11.36	11.08	11.51	0	0	0	0	0	0	0	12.83	12.85	12.87	12.80	12.87	12.68	12.06
1398797_at	Hnrnpk	1	0	0	0	1	0	11.18	11.05	11.67	10.94	11.24	0	0	0	0	0	0	0	12.10	12.12	12.15	12.22	12.19	12.08	11.66
1398800_at	Ywhab	1	0	0	0	0	1	9.86	10.04	10.25	9.94	10.40	0	0	0	0	0	0	0	12.00	11.94	12.00	12.02	11.99	11.99	12.08
1398817_at	Arf1	1	0	0	1	1	0	9.95	9.70	10.31	9.33	9.79	0	0	0	0	0	0	0	11.51	11.60	11.57	11.55	11.53	11.43	10.72
1398819_at	Dnaja1	1	0	0	0	1	0	8.93	9.02	9.49	8.60	9.13	0	0	0	0	0	0	0	11.27	11.29	11.32	11.38	11.30	10.92	10.39
1398821_s_at	Mylk2	1	0	1	0	0	0	4.79	4.46	4.21	4.72	4.48	0	0	0	0	0	0	0	4.03	4.08	4.10	3.93	3.91	4.11	3.83
1398822_at	Gdi2	1	0	0	1	1	0	8.66	8.48	9.04	7.92	8.64	0	0	0	0	0	0	0	10.82	10.80	10.88	10.93	10.88	10.78	10.24
1398823_at	Tsnax	1	0	0	1	1	0	7.56	7.52	8.07	6.61	7.54	0	0	0	0	0	0	0	9.14	9.16	9.28	9.34	9.30	9.29	8.61
1398824_at	Tmed2	1	0	0	1	1	0	9.82	9.66	10.19	9.06	9.69	0	0	0	0	0	0	0	12.22	12.27	12.28	12.27	12.29	12.19	11.13
1398826_s_at	Nr2f6	1	0	0	1	1	0	7.31	7.33	6.81	7.81	7.16	0	0	0	0	0	0	0	9.40	9.39	9.41	9.27	9.40	9.37	9.05
1398830_at	Rpl28	1	0	0	1	0	0	12.46	12.42	12.46	12.94	12.01	0	0	0	0	0	0	0	12.80	12.77	12.77	12.76	12.75	12.75	12.49
1398838_at	Rab7a	1	0	0	0	1	0	8.10	8.14	8.50	7.65	8.20	0	0	0	0	0	0	0	10.59	10.61	10.68	10.65	10.62	10.70	10.32
1398841_at	Rab1a	1	0	0	0	1	0	11.40	11.40	11.80	11.00	11.71	0	0	0	0	0	0	0	13.20	13.19	13.20	13.18	13.18	13.17	12.65
1398842_at	Cltc	1	0	0	0	0	1	10.42	10.40	10.82	10.29	10.91	0	0	0	0	0	0	0	12.14	12.16	12.17	12.15	12.20	12.17	12.08
1398847_at	Nudt4	1	1	0	0	0	1	10.14	9.63	9.90	9.92	9.41	0	0	0	0	0	0	0	10.02	10.00	9.99	9.92	9.98	10.07	9.90
1398854_at	Rpl24	1	0	0	0	0	1	12.13	11.79	12.34	12.40	11.64	0	0	0	0	0	0	0	12.67	12.68	12.67	12.67	12.68	12.64	12.23
1398856_at	Psma2	1	0	0	0	1	0	8.27	8.05	8.60	7.86	8.43	0	0	0	0	0	0	0	10.72	10.80	10.79	10.83	10.81	10.71	10.43
1398862_at	Atp2a2	1	0	0	0	1	0	8.06	7.78	7.54	8.14	8.28	0	0	0	0	0	0	0	7.56	7.50	7.66	7.48	7.44	7.15	6.99
1398866_at	Magi3	1	0	1	0	0	0	7.75	7.50	8.57	8.19	7.68	0	0	0	0	0	0	0	9.43	9.52	9.48	9.58	9.74	9.95	9.78
1398878_at	Cpsf4	1	0	0	0	1	1	7.80	7.49	7.48	8.09	7.10	0	0	0	0	0	0	0	8.94	8.97	9.01	9.07	9.06	9.26	9.18
1398880_at	Sub1	1	0	1	0	1	1	11.22	11.35	11.99	11.25	11.81	0	0	0	0	0	0	0	12.89	12.86	12.86	12.85	12.86	12.87	12.54
1398891_at	Mrpl15	1	0	0	0	0	1	8.89	8.55	8.52	8.91	8.38	0	0	0	0	0	0	0	9.98	10.05	10.02	9.96	9.93	9.68	9.30
1398893_at	Ndfip1	1	0	0	0	1	0	10.16	10.14	10.66	9.91	10.51	0	0	0	0	0	0	0	12.25	12.26	12.27	12.25	12.28	12.37	11.95
1398912_at	mrpl9	1	0	0	0	0	1	8.36	8.07	8.30	8.43	7.82	0	0	0	0	0	0	0	9.85	9.80	9.78	9.70	9.70	9.50	9.20
1398926_at	Pfdn1	1	0	0	1	0	0	7.19	7.37	7.45	7.81	7.21	0	0	0	0	0	0	0	10.74	10.72	10.67	10.62	10.58	10.46	10.29
1398928_at	Cuta	1	0	0	0	1	0	9.41	9.45	9.02	9.78	9.02	0	0	0	0	0	0	0	10.19	10.22	10.19	10.28	10.27	10.47	10.44
1398937_at	Dhx15	1	0	0	0	1	0	8.89	8.77	9.38	8.61	8.97	0	0	0	0	0	0	0	11.11	11.19	11.26	11.34	11.31	11.18	10.89
1398942_at	---	1	0	0	1	0	0	7.34	7.58	7.45	7.86	7.61	0	0	0	0	0	0	0	7.51	7.51	7.56	7.51	7.52	7.37	7.28
1398945_at	Ddx6	1	0	1	0	1	0	8.13	7.86	8.71	7.97	8.25	0	0	0	0	0	0	0	9.87	9.87	9.82	9.88	9.88	9.91	9.33
1398961_at	Rnf13	1	0	0	0	1	0	8.31	8.31	8.65	7.86	8.35	0	0	0	0	0	0	0	9.70	9.82	9.78	9.84	9.94	10.12	9.75
1398962_at	Abhd17a	1	0	0	0	1	0	7.01	6.95	6.76	7.36	6.93	0	0	0	0	0	0	0	9.34	9.31	9.30	9.17	9.18	9.33	9.65
1398964_at	Selenot	1	0	0	0	1	0	9.98	9.98	10.53	9.70	10.42	0	0	0	0	0	0	0	12.47	12.47	12.49	12.49	12.49	12.58	12.40
1398971_at	Kiaa0100	1	0	0	0	1	0	8.58	8.55	8.38	8.97	8.52	0	0	0	0	0	0	0	9.84	9.78	9.83	9.75	9.74	9.54	9.74
1398992_at	---	1	0	1	0	1	0	6.60	6.67	7.20	6.46	6.91	0	0	0	0	0	0	0	8.53	8.66	8.72	8.73	8.70	8.56	8.26
1398994_at	Tpst2	1	0	0	0	0	1	8.08	8.04	8.33	7.93	8.61	0	0	0	0	0	0	0	9.72	9.79	9.79	9.75	9.76	9.69	9.51
1399000_at	Atp9b	1	0	0	1	0	0	6.67	6.95	6.75	7.22	6.67	0	0	0	0	0	0	0	8.61	8.60	8.69	8.62	8.67	8.67	8.38
1399017_at	Tnpo1	1	0	0	0	1	0	9.01	8.84	9.58	8.87	9.15	0	0	0	0	0	0	0	10.46	10.51	10.54	10.60	10.62	10.51	10.35
1399018_at	Luc7l3	1	0	1	0	0	0	9.31	9.23	9.97	9.35	9.46	0	0	0	0	0	0	0	11.02	11.04	11.12	11.14	11.17	11.19	10.70
1399022_at	Clk1	1	0	1	0	1	0	9.23	9.05	9.94	8.80	8.95	0	0	0	0	0	0	0	9.37	9.47	9.55	9.62	9.60	9.39	8.86
1399025_at	Mier1	1	0	1	0	1	0	8.59	8.57	9.28	8.47	8.66	0	0	0	0	0	0	0	10.03	10.12	10.12	10.13	10.19	10.29	9.93
1399026_at	Paip2	1	0	0	0	1	0	8.71	8.58	9.13	8.30	8.68	0	0	0	0	0	0	0	11.50	11.53	11.48	11.43	11.45	11.45	10.89
1399027_at	Rhoa	1	0	1	0	0	1	8.24	8.24	8.83	8.30	8.75	0	0	0	0	0	0	0	10.69	10.64	10.52	10.44	10.46	10.43	10.19
1399032_at	Ercc1	1	0	0	0	1	0	6.28	6.25	5.95	6.52	6.26	0	0	0	0	0	0	0	8.19	8.19	8.23	8.28	8.16	8.01	7.80
1399033_at	Cbfb	1	0	1	0	1	0	8.49	8.49	9.23	8.20	8.75	0	0	0	0	0	0	0	9.94	9.89	10.02	10.12	10.05	10.17	9.86
1399045_at	Galnt1	1	0	1	0	0	0	7.94	8.01	8.56	7.87	8.00	0	0	0	0	0	0	0	10.56	10.58	10.64	10.74	10.67	10.63	10.14
1399049_at	Jkamp	1	0	1	0	1	0	7.65	7.71	8.25	7.29	7.98	0	0	0	0	0	0	0	10.34	10.33	10.34	10.34	10.36	10.31	10.05
1399062_at	Smim15	1	0	0	0	1	0	7.23	7.06	7.70	6.95	7.29	0	0	0	0	0	0	0	9.34	9.38	9.35	9.46	9.41	9.38	8.91
1399064_at	Ric8b	1	0	0	0	0	1	6.12	6.26	6.66	6.02	6.61	0	0	0	0	0	0	0	8.29	8.27	8.35	8.35	8.31	8.20	7.94
1399067_at	Gnl3l	1	0	0	0	1	0	8.35	8.28	8.91	8.13	8.73	0	0	0	0	0	0	0	10.04	10.04	10.11	10.18	10.15	10.09	10.28
1399069_at	Efcab14	1	0	0	0	1	1	8.38	8.49	8.92	8.16	8.90	0	0	0	0	0	0	0	8.97	8.90	8.92	8.86	8.97	9.20	9.20
1399074_at	Cdc16	1	0	0	0	0	1	8.40	8.22	8.29	8.45	7.86	0	0	0	0	0	0	0	10.88	10.89	10.89	10.84	10.86	10.73	10.48
1399083_at	Abhd11	1	0	0	0	0	1	5.96	5.93	5.72	5.85	5.38	0	0	0	0	0	0	0	7.62	7.62	7.68	7.73	7.65	7.68	7.79
1399099_at	Hnrnpul1	1	0	0	1	0	0	5.73	5.77	5.73	6.23	5.63	0	0	0	0	0	0	0	8.16	8.10	8.27	8.30	8.12	7.96	7.05
1399122_at	---	1	0	0	0	1	0	6.38	6.27	6.93	6.07	6.50	0	0	0	0	0	0	0	8.00	8.08	8.21	8.25	8.36	8.42	8.14
1399124_at	Emc1	1	0	0	0	0	1	7.07	7.23	7.40	7.09	7.55	0	0	0	0	0	0	0	10.29	10.29	10.36	10.21	10.33	10.22	9.98
1399156_at	Arid2	1	0	1	0	0	0	7.09	7.27	7.67	7.27	7.56	0	0	0	0	0	0	0	9.27	9.27	9.32	9.34	9.23	8.98	8.71
1399158_a_at	Npm1	1	0	0	0	0	1	9.59	9.31	10.03	9.74	9.02	0	0	0	0	0	0	0	11.18	11.23	11.23	11.34	11.31	11.09	10.48
1399160_a_at	Ube2d3	1	0	0	1	1	0	9.28	9.16	9.74	8.78	9.25	0	0	0	0	0	0	0	11.37	11.38	11.34	11.44	11.36	11.24	10.47
1367468_at	Scand1	0	0	0	0	0	0	8.65	8.63	8.34	8.86	8.30	1	0	0	0	0	0	1	10.27	10.28	10.28	10.26	10.15	9.84	9.59
1367486_at	Arfgap2	0	0	0	0	0	0	8.32	8.40	8.07	8.48	8.11	1	0	0	0	0	0	1	9.20	9.21	9.18	9.13	9.03	8.67	8.32
1367510_at	Txndc15	0	0	0	0	0	0	8.33	8.21	8.50	8.31	8.17	1	0	0	0	0	1	1	10.06	10.12	10.19	10.23	10.18	9.44	8.80
1367522_at	F8a1	0	0	0	0	0	0	8.34	8.36	8.32	8.45	8.38	1	0	0	0	0	0	1	9.21	9.18	9.19	9.18	9.04	8.71	8.40
1367551_a_at	Ccz1 /// Ccz1b	0	0	0	0	0	0	7.58	7.37	7.61	7.67	7.49	1	0	0	0	0	0	1	9.91	9.90	9.93	9.94	9.92	9.44	9.10
1367584_at	Anxa2	0	0	0	0	0	0	7.68	7.39	7.31	7.23	7.38	1	0	0	0	0	1	1	7.57	7.60	7.40	7.08	7.14	8.23	8.96
1367592_at	Tnnt2	0	0	0	0	0	0	2.93	2.88	2.75	2.83	2.89	1	0	0	0	0	1	1	3.89	3.95	4.12	4.21	4.35	5.93	7.04
1367593_at	Selenow	0	0	0	0	0	0	5.73	5.81	5.82	5.63	5.45	1	0	0	0	0	0	1	7.74	7.75	7.79	7.68	7.60	7.35	7.00
1367599_at	Atp5mc1	0	0	0	0	0	0	11.15	11.20	11.15	11.37	11.28	1	0	0	0	0	0	1	12.36	12.33	12.36	12.38	12.31	11.85	11.29
1367600_at	Des	0	0	0	0	0	0	5.30	5.27	5.14	5.21	5.23	1	0	0	0	0	0	1	6.05	6.07	6.02	5.91	6.02	6.46	6.87
1367602_at	Cited2	0	0	0	0	0	0	8.96	9.01	8.99	8.79	8.61	1	0	0	0	0	1	1	11.39	11.30	11.19	10.84	10.86	9.98	10.16
1367603_at	Tpi1	0	0	0	0	0	0	8.63	8.61	8.73	8.59	8.74	1	0	0	0	0	1	1	12.74	12.74	12.62	12.40	12.35	11.88	11.59
1367604_at	Crip2	0	0	0	0	0	0	7.70	7.46	7.42	7.48	7.40	1	0	0	0	0	1	1	7.45	7.49	7.61	7.70	7.91	9.48	9.84
1367605_at	Pfn1	0	0	0	0	0	0	9.75	9.58	9.65	9.74	9.82	1	0	0	0	0	1	1	11.00	10.96	11.07	11.06	10.96	10.39	9.50
1367612_at	Mgst1	0	0	0	0	0	0	5.46	5.48	5.49	5.92	5.12	1	0	0	0	0	1	1	4.14	4.11	4.15	4.15	4.37	6.29	7.08
1367636_at	Igf2r	0	0	0	0	0	0	8.07	8.02	8.40	8.39	8.21	1	0	0	0	0	1	1	9.85	9.85	9.96	9.86	10.03	10.44	10.64
1367650_at	Tinagl1	0	0	0	0	0	0	6.78	6.57	6.88	6.55	6.50	1	0	0	0	1	1	1	6.13	6.23	6.48	6.62	6.85	7.79	7.57
1367654_at	Fat1	0	0	0	0	0	0	8.10	8.10	8.64	8.16	8.18	1	0	0	0	0	1	1	10.88	10.78	11.09	11.29	11.29	12.11	12.67
1367660_at	Fabp3	0	0	0	0	0	0	5.18	5.25	5.18	5.30	5.42	1	0	0	1	0	1	1	12.22	12.22	12.01	11.50	11.69	10.49	10.14
1367675_at	Cib1	0	0	0	0	0	0	7.52	7.24	7.36	7.56	7.40	1	0	0	0	0	1	1	8.75	8.78	8.87	8.86	8.94	9.90	10.13
1367683_at	Kpna2	0	0	0	0	0	0	7.18	6.83	7.62	7.12	7.52	1	0	0	0	0	1	1	11.19	11.12	11.16	11.12	11.10	10.57	10.32
1367684_at	Crybb2	0	0	0	0	0	0	4.22	4.25	3.80	4.19	4.17	1	0	0	0	0	1	1	4.04	4.09	4.26	4.28	4.50	6.53	8.28
1367691_at	Cavin3	0	0	0	0	0	0	6.64	6.45	6.46	6.28	6.31	1	0	0	0	0	1	1	9.33	9.28	9.20	9.07	8.96	8.32	7.89
1367708_a_at	Fasn	0	0	0	0	0	0	8.10	8.50	8.08	8.49	8.46	1	0	0	0	0	1	1	10.57	10.50	10.56	10.55	10.45	9.94	9.72
1367715_at	Tnfrsf1a	0	0	0	0	0	0	7.03	6.84	6.94	6.97	7.08	1	0	0	0	0	0	1	9.29	9.20	9.29	9.20	9.23	8.80	8.18
1367727_at	Cyth2	0	0	0	0	0	0	7.82	7.73	7.91	7.64	7.85	1	0	0	0	0	0	1	10.46	10.48	10.54	10.53	10.45	9.96	9.70
1367740_at	Ckb	0	0	0	0	0	0	6.85	6.70	6.67	6.77	7.09	1	0	0	0	0	1	0	10.29	10.41	10.52	10.54	10.61	10.95	10.78
1367744_at	Maged2	0	0	0	0	0	0	8.46	8.59	8.63	8.29	8.65	1	0	0	0	0	1	1	10.35	10.34	10.35	10.21	10.14	9.58	9.36
1367748_at	Arf5	0	0	0	0	0	0	9.18	8.87	9.05	9.27	8.86	1	0	0	0	0	0	1	10.67	10.73	10.71	10.64	10.57	10.26	9.86
1367758_at	Afp	0	0	0	0	0	0	3.66	3.78	3.51	3.66	3.70	1	0	0	0	0	0	1	3.92	3.98	4.01	3.99	3.89	4.35	5.07
1367780_at	Pttg1	0	0	0	0	0	0	7.88	7.94	7.85	8.02	8.01	1	0	0	0	0	1	1	10.31	10.29	10.27	10.21	10.08	9.14	9.02
1367787_at	Ica1	0	0	0	0	0	0	9.22	9.24	9.41	9.32	9.45	1	0	0	0	0	0	1	10.20	10.28	10.32	10.35	10.31	9.78	9.52
1367799_at	Eef1a2	0	0	0	0	0	0	5.84	5.92	5.60	5.91	5.71	1	0	0	0	0	1	1	9.74	9.69	9.79	9.77	9.57	8.75	7.24
1367805_at	Gls	0	0	0	0	0	0	7.31	7.27	7.38	7.12	7.67	1	0	0	0	0	1	1	8.59	8.65	8.74	8.78	8.84	9.60	9.64
1367806_at	Gls	0	0	0	0	0	0	6.06	6.24	6.42	5.81	6.51	1	0	0	0	0	1	1	7.91	7.81	8.01	8.14	8.00	8.65	8.44
1367813_at	Ppp1r14a	0	0	0	0	0	0	4.62	4.74	4.34	4.87	4.17	1	0	0	0	0	1	1	8.46	8.56	8.73	8.86	8.97	9.50	9.10
1367817_at	Hdgf	0	0	0	0	0	0	7.39	7.24	7.40	7.68	7.38	1	0	0	0	0	1	1	10.27	10.24	10.28	10.18	10.02	9.52	8.76
1367831_at	Tp53	0	0	0	0	0	0	6.08	5.96	6.00	6.28	6.12	1	0	0	0	0	1	1	7.00	6.98	6.97	6.94	6.81	6.35	6.37
1367842_at	Apbb1	0	0	0	0	0	0	6.27	6.72	6.58	6.46	6.73	1	0	0	0	0	0	1	10.04	10.03	10.04	10.03	9.88	9.52	9.26
1367850_at	Fcgr2a	0	0	0	0	0	0	3.32	3.48	3.28	3.38	3.04	1	0	0	0	1	1	1	4.70	4.71	4.99	5.12	5.37	6.07	5.66
1367853_at	Slc12a2	0	0	0	0	0	0	9.50	9.27	9.41	9.66	9.12	1	0	0	0	0	0	1	9.87	9.79	9.89	9.88	9.86	10.25	10.54
1367867_at	Gfer	0	0	0	0	0	0	6.60	6.30	6.45	6.78	6.19	1	0	0	0	0	1	1	7.58	7.55	7.59	7.62	7.45	6.98	6.77
1367892_at	Pdk2	0	0	0	0	0	0	7.11	6.90	6.77	7.18	6.74	1	0	0	0	0	1	1	7.10	7.17	7.22	7.22	7.31	7.97	8.23
1367899_at	F2r	0	0	0	0	0	0	6.29	6.23	6.67	6.07	6.75	1	0	0	0	1	1	1	8.15	8.31	8.57	8.69	8.85	9.19	9.25
1367906_at	Acp2	0	0	0	0	0	0	7.73	7.85	7.82	8.11	7.71	1	0	0	0	0	0	1	9.02	9.00	9.12	9.18	9.16	8.57	7.82
1367918_at	Fez1	0	0	0	0	0	0	4.76	4.67	5.00	4.44	4.79	1	0	0	1	1	1	1	6.56	6.74	7.04	7.21	7.25	7.55	7.55
1367920_at	S1pr2	0	0	0	0	0	0	3.50	3.69	3.35	3.41	3.55	1	0	0	0	0	1	0	4.14	4.10	4.24	4.15	4.32	4.87	4.33
1367922_at	Adam17	0	0	0	0	0	0	6.72	6.78	7.23	6.89	7.03	1	0	0	0	0	1	1	7.92	8.02	8.19	8.33	8.43	8.82	9.06
1367932_at	Hmgcs1	0	0	0	0	0	0	7.31	7.15	7.36	7.12	7.67	1	0	0	0	0	0	1	9.49	9.54	9.59	9.63	9.63	9.07	8.82
1367936_at	Stk10	0	0	0	0	0	0	5.68	5.82	5.78	5.86	5.69	1	0	0	0	0	0	1	6.57	6.51	6.58	6.61	6.74	6.93	7.18
1367945_at	Atox1	0	0	0	0	0	0	8.83	8.90	8.69	9.19	8.79	1	0	0	0	0	1	0	9.92	9.94	9.96	10.09	10.11	10.65	10.45
1367949_at	Penk	0	0	0	0	0	0	6.77	6.69	6.44	6.71	6.74	1	0	0	0	0	1	1	6.22	6.17	6.17	6.06	6.26	6.99	8.01
1367979_s_at	Cyp51a1	0	0	0	0	0	0	8.07	8.13	7.82	8.05	8.37	1	0	0	0	0	0	1	10.08	9.99	10.15	10.27	10.18	9.61	9.44
1367990_at	Crybb3	0	0	0	0	0	0	4.80	4.99	4.90	4.96	4.86	1	0	0	0	0	1	0	5.60	5.71	5.74	5.80	5.90	6.34	6.43
1367994_at	Dpyd	0	0	0	0	0	0	4.08	3.77	3.93	4.01	3.99	1	0	0	0	0	0	1	3.78	3.76	3.72	3.72	3.67	4.32	5.42
1368009_at	Gne	0	0	0	0	0	0	6.12	6.10	6.03	6.08	6.16	1	0	0	0	0	0	1	7.83	7.83	7.82	7.73	7.66	7.30	6.89
1368014_at	Ptges	0	0	0	0	0	0	3.41	3.41	3.21	3.35	3.35	1	0	0	1	0	0	1	6.06	6.17	5.93	5.47	5.82	6.57	7.39
1368018_at	Mkln1	0	0	0	0	0	0	7.89	7.85	8.05	7.88	7.87	1	0	0	0	1	1	1	10.68	10.83	10.94	11.03	11.28	12.15	12.19
1368028_at	Prph	0	0	0	0	0	0	4.77	4.87	4.38	4.60	4.79	1	0	0	0	0	1	1	13.07	13.11	13.12	13.08	13.01	11.64	10.83
1368045_at	Slc31a1	0	0	0	0	0	0	5.45	5.22	5.36	5.28	5.41	1	0	0	0	0	1	0	6.18	6.29	6.32	6.55	6.50	6.87	6.79
1368047_at	Slc13a3	0	0	0	0	0	0	3.36	3.30	3.21	3.41	3.38	1	0	0	0	0	0	1	4.51	4.43	4.46	4.49	4.42	4.78	5.64
1368055_a_at	Lmna	0	0	0	0	0	0	8.21	8.09	7.88	8.44	7.98	1	0	0	0	0	1	1	10.40	10.36	10.41	10.37	10.27	9.82	9.56
1368059_at	Crym	0	0	0	0	0	0	4.38	4.31	4.27	4.38	4.40	1	0	0	0	0	1	1	6.31	6.23	6.26	6.30	6.33	8.38	9.78
1368060_at	Rida	0	0	0	0	0	0	8.19	8.08	8.11	8.12	8.17	1	0	0	0	0	1	1	8.55	8.74	8.76	8.91	9.05	9.68	9.57
1368061_at	Kcnh1	0	0	0	0	0	0	3.76	3.73	3.53	3.71	3.62	1	0	0	0	0	1	0	6.72	6.77	6.73	6.42	6.60	6.00	5.81
1368074_at	Gale	0	0	0	0	0	0	5.41	5.57	5.28	5.48	5.48	1	0	0	0	0	1	1	9.01	9.02	8.91	8.67	8.64	8.22	7.92
1368092_at	Fah	0	0	0	0	0	0	6.74	6.77	6.35	6.86	6.45	1	0	0	0	0	1	1	6.61	6.57	6.59	6.68	6.58	7.22	7.27
1368106_at	Plk2	0	0	0	0	0	0	7.38	7.60	7.10	6.95	7.15	1	0	0	0	0	0	1	7.13	7.06	7.13	7.20	7.18	7.65	8.33
1368109_at	St3gal5	0	0	0	0	0	0	6.04	6.06	6.04	5.85	6.20	1	0	0	0	0	1	0	8.17	8.26	8.38	8.46	8.50	8.80	8.70
1368113_at	Tff2	0	0	0	0	0	0	13.36	13.28	13.20	13.61	13.01	1	0	0	0	0	0	1	4.56	4.56	4.62	4.30	4.41	4.78	5.12
1368139_s_at	Alpl	0	0	0	0	0	0	4.80	4.56	4.48	4.60	4.65	1	0	0	0	0	1	1	6.15	6.24	6.37	6.44	6.65	7.42	7.32
1368148_at	Ngfr	0	0	0	0	0	0	3.91	3.89	3.65	3.65	3.77	1	0	0	0	0	1	1	6.78	6.93	7.07	7.05	7.29	8.13	8.41
1368156_at	Camkk1	0	0	0	0	0	0	5.33	5.52	5.37	5.32	4.93	1	0	0	0	0	0	1	7.97	7.89	8.00	8.03	8.04	8.49	8.67
1368158_at	Scfd1	0	0	0	0	0	0	8.64	8.66	8.86	8.42	9.09	1	0	0	0	0	0	1	10.09	10.12	10.10	10.21	10.13	9.70	9.18
1368160_at	Igfbp1	0	0	0	0	0	0	6.27	6.32	6.47	6.07	5.93	1	0	0	1	0	1	0	7.83	7.90	7.61	7.18	7.29	6.91	7.43

Probeset ID	Gene Symbol	Islet Data Set (log2 ratio ≥0.263; FDR <20%) union n=5325 1=YES 0=NO	Islet DRlyp/lyp ND vs ND+Lp299v (log2 ratio ≥0.263; FDR<20%) n=429 1=YES 0=NO	Islet DRlyp/lyp ND vs HCD (log2 ratio ≥0.263; FDR<20%) n=2247 1=YES 0=NO	Islet DRlyp/lyp ND vs HCD+Lp299v (log2 ratio ≥0.263; FDR<20%) n=1595 1=YES 0=NO	Islet DRlyp/lyp HCD vs HCD+Lp299v (log2 ratio ≥0.263; FDR<20%) n=3026 1=YES 0=NO	Islet DRlyp/lyp ND vs Flyp/lyp ND (log2 ratio ≥ 0.263; FDR<20%) n=1950 1=YES 0=NO	Islet Mean Log2 Intensity DRlyp/lyp ND	Islet Mean Log2 Intensity DRlyp/lyp ND+Lp299v	Islet Mean Log2 Intensity DRlyp/lyp HCD	Islet Mean Log2 Intensity DRlyp/lyp HCD+Lp299v	Islet Mean Log2 Intensity Flyp40 ND	Rinm5f data union of all conditions n=3705 1=YES 0=NO	Rinm5f 30 µM propionate/ 6 µM butyrate vs Media (FDR<20%; 1.5 fold) n=3 1=YES 0=NO	Rinm5f 60 µM propionate/ 12 µM butyrate vs Media (FDR<20%; 1.5 fold) n=18 1=YES 0=NO	Rinm5f 120 µM propionate/ 24 µM butyrate vs Media (FDR<20%; 1.5 fold) n=164 1=YES 0=NO	Rinm5f 240 µM propionate /48 µM butyrate vs Media (FDR<20%; 1.5 fold) n=268 1=YES 0=NO	Rinm5f 1500 µM propionate/ 300 µM butyrate vs Media (FDR<20%; 1.5 fold) n=2491 1=YES 0=NO	Rinm5f 500 µM propionate/ 500 µM butyrate vs Media (FDR<20%; 1.5 fold) n=3001 1=YES 0=NO	Rinm5f Mean Log2 Intensity Media only	Rinm5f Mean Log2 Intensity 30 µM propionate/ 6 µM butyrate	Rinm5f Mean Log2 Intensity 60 µM propionate/ 12 µM butyrate	Rinm5f Mean Log2 Intensity 120 µM propionate/ 24 µM butyrate	Rinm5f Mean Log2 Intensity 240 µM propionate/ 48 µM butyrate	Rinm5f Mean Log2 Intensity 1500 µM propionate /300 µM butyrate	Rinm5f Mean Log2 Intensity 500 µM propionate/ 500 µM butyrate
1368167_at	Ctse	0	0	0	0	0	0	3.74	3.87	3.75	3.67	4.06	1	0	0	0	0	1	1	5.66	5.75	5.71	5.83	5.93	6.44	5.72
1368170_at	Slc6a1	0	0	0	0	0	0	3.30	3.28	3.33	3.23	3.28	1	0	0	0	0	1	1	5.42	5.34	5.65	5.67	5.78	6.51	7.73
1368174_at	Egln3	0	0	0	0	0	0	3.14	3.34	3.26	3.04	3.15	1	0	0	1	0	0	0	8.87	8.94	8.70	7.87	8.35	8.60	8.38
1368188_at	Hpd	0	0	0	0	0	0	3.66	3.47	3.41	3.54	3.59	1	0	0	0	0	1	1	4.84	4.84	5.06	4.97	5.33	6.44	6.36
1368191_a_at	Slc22a1	0	0	0	0	0	0	3.56	3.52	3.26	3.57	3.38	1	0	0	0	0	0	1	4.32	4.40	4.16	4.21	4.24	4.82	5.98
1368207_at	Fxyd5	0	0	0	0	0	0	5.16	4.98	5.15	5.06	5.22	1	0	0	0	0	1	1	3.59	3.60	3.62	3.60	3.80	4.20	4.29
1368209_at	Pdzk1ip1	0	0	0	0	0	0	5.79	5.70	5.60	5.73	5.52	1	0	0	1	1	1	0	6.85	7.33	7.40	7.63	8.18	7.70	7.17
1368221_at	Nr3c1	0	0	0	0	0	0	7.75	7.69	7.96	7.96	7.74	1	0	0	0	0	1	0	9.66	9.70	9.63	9.70	9.80	10.30	9.71
1368250_at	Tekt1	0	0	0	0	0	0	3.56	3.87	3.64	3.66	3.69	1	0	0	0	0	0	1	4.40	4.55	4.60	4.48	4.55	4.65	6.45
1368252_at	Klhl41	0	0	0	0	0	0	2.93	3.02	2.91	2.95	3.00	1	0	0	0	0	1	1	5.18	5.20	5.14	4.86	4.69	4.39	4.20
1368254_a_at	Sphk1	0	0	0	0	0	0	5.17	5.24	5.00	5.36	5.07	1	0	0	0	0	1	1	5.69	5.73	5.63	5.46	5.64	6.41	5.93
1368279_at	Mllt3	0	0	0	0	0	0	4.81	4.85	4.88	5.26	4.92	1	0	0	0	0	1	1	6.72	6.69	6.98	7.02	6.96	7.50	7.95
1368320_at	Ncam1	0	0	0	0	0	0	5.90	6.30	5.94	6.10	6.05	1	0	0	0	0	1	1	8.64	8.52	8.71	8.72	8.76	9.31	9.45
1368328_at	Gys2	0	0	0	0	0	0	4.02	3.89	3.83	4.04	3.82	1	0	0	0	0	0	1	3.98	3.99	3.77	3.94	3.94	4.53	4.75
1368334_at	Grb7	0	0	0	0	0	0	4.80	4.86	4.82	4.81	4.72	1	0	0	0	0	0	1	8.09	8.13	8.07	7.93	7.90	7.60	7.04
1368356_a_at	Erap1	0	0	0	0	0	0	8.09	8.24	8.28	8.54	8.04	1	0	0	0	0	0	1	10.40	10.41	10.36	10.38	10.37	9.89	9.03
1368372_at	Sts	0	0	0	0	0	0	5.55	5.53	5.25	5.65	5.52	1	0	0	1	1	0	0	7.33	7.46	7.69	7.94	8.00	7.88	7.45
1368381_at	Crtac1	0	0	0	0	0	0	3.50	3.50	3.37	3.48	3.43	1	0	0	0	0	1	1	7.33	7.33	7.50	7.41	7.43	7.95	7.59
1368382_at	S100a3	0	0	0	0	0	0	5.14	5.28	5.10	5.19	5.35	1	0	0	0	0	0	1	9.67	9.71	9.74	9.87	9.82	9.21	8.70
1368390_at	Araf	0	0	0	0	0	0	7.54	7.61	7.55	7.70	7.79	1	0	0	0	0	0	1	7.94	7.89	7.94	7.99	7.87	7.49	6.88
1368395_at	Gpc3	0	0	0	0	0	0	4.89	4.76	4.79	4.79	4.62	1	0	0	0	0	1	1	7.60	7.71	7.77	7.95	8.17	9.66	9.40
1368417_at	Syt5	0	0	0	0	0	0	5.66	6.04	5.85	5.86	6.04	1	0	0	0	0	1	1	8.91	8.81	9.06	9.12	8.95	7.87	6.83
1368451_at	Hrh3	0	0	0	0	0	0	6.25	6.40	6.17	6.18	6.17	1	0	0	0	0	0	1	6.11	6.08	6.10	5.84	6.01	6.63	6.41
1368463_at	Vegfc	0	0	0	0	0	0	5.91	5.99	6.03	6.18	6.22	1	0	0	0	0	1	1	7.49	7.43	7.52	7.38	7.29	6.65	7.02
1368470_at	Ggh	0	0	0	0	0	0	13.44	13.27	13.59	13.59	13.28	1	0	0	0	0	1	1	11.77	11.71	11.80	11.89	11.96	12.58	12.40
1368495_at	Rln1	0	0	0	0	0	0	3.77	3.84	3.68	3.87	3.67	1	0	0	0	0	0	1	4.36	4.23	4.31	4.13	4.22	4.67	5.04
1368519_at	Serpine1	0	0	0	0	0	0	3.47	3.35	3.42	3.37	3.30	1	0	0	0	0	1	1	5.89	5.97	5.85	5.89	6.04	6.67	6.70
1368524_at	Kcnc1	0	0	0	0	0	0	3.42	3.27	3.09	3.30	3.29	1	0	0	0	0	1	1	4.85	5.01	5.12	5.22	5.19	5.47	5.87
1368525_at	Mrs2	0	0	0	0	0	0	5.48	5.12	5.47	5.60	5.41	1	0	0	0	0	0	1	7.59	7.59	7.67	7.63	7.58	7.11	6.63
1368538_at	Exoc7	0	0	0	0	0	0	6.47	6.56	6.82	6.59	6.53	1	0	0	0	0	0	1	9.99	10.07	10.13	10.16	10.14	9.64	8.72
1368540_at	Tpbg	0	0	0	0	0	0	3.01	2.99	3.14	3.15	3.02	1	0	0	0	0	0	1	6.34	6.40	6.40	6.50	6.36	6.00	5.47
1368544_a_at	Nol3	0	0	0	0	0	0	3.46	3.81	3.53	3.45	3.56	1	0	0	0	0	0	1	8.18	8.33	8.30	8.28	8.31	7.84	7.17
1368545_at	Cflar	0	0	0	0	0	0	5.40	5.49	5.25	5.22	5.42	1	0	0	0	0	1	1	6.09	6.08	6.02	5.86	5.74	5.47	4.92
1368546_at	Hivep2	0	0	0	0	0	0	5.89	5.86	6.06	5.86	6.06	1	0	0	0	0	1	1	8.96	8.89	8.86	8.72	8.52	7.69	7.69
1368561_at	Abcd2	0	0	0	0	0	0	3.44	3.58	3.39	3.59	3.37	1	0	0	0	0	1	1	5.33	5.31	5.50	5.72	5.72	6.74	7.01
1368562_at	Sult4a1	0	0	0	0	0	0	5.65	5.63	5.60	5.28	5.84	1	0	0	0	0	1	0	9.35	9.47	9.61	9.71	9.70	10.03	9.66
1368564_at	Slc17a6	0	0	0	0	0	0	5.09	5.10	4.87	5.13	5.00	1	0	0	0	0	0	1	5.38	5.15	5.45	5.55	5.42	5.68	5.65
1368567_at	Ndufv3	0	0	0	0	0	0	5.57	5.60	5.61	5.47	5.48	1	0	0	0	0	1	0	7.20	7.33	7.42	7.43	7.51	7.82	7.59
1368570_at	Lrat	0	0	0	0	0	0	2.92	2.86	2.66	2.92	2.79	1	0	0	0	0	0	1	3.76	3.69	3.62	3.40	3.60	3.89	4.20
1368588_at	Ddx52	0	0	0	0	0	0	4.39	4.60	4.89	4.49	4.55	1	0	0	0	0	0	1	7.51	7.64	7.62	7.78	7.74	7.23	6.28
1368607_at	Cyp4a22	0	0	0	0	0	0	3.66	3.66	3.54	3.79	3.70	1	0	0	1	1	0	0	6.58	6.66	6.81	7.29	7.55	6.61	6.16
1368620_at	Spag4	0	0	0	0	0	0	4.70	4.54	4.31	4.66	4.50	1	0	0	0	0	1	0	6.46	6.50	6.66	6.42	6.57	7.06	6.62
1368641_at	Wnt4	0	0	0	0	0	0	8.96	9.06	9.10	8.50	9.03	1	0	0	0	0	1	1	11.35	11.40	11.35	11.08	11.08	10.13	9.31
1368643_at	Spata6	0	0	0	0	0	0	4.17	4.10	4.42	4.12	4.36	1	0	0	0	0	1	1	7.25	7.29	7.43	7.57	7.64	8.23	8.37
1368644_at	Spata6	0	0	0	0	0	0	3.84	3.72	3.85	3.76	3.81	1	0	0	0	0	1	1	6.07	6.09	6.23	6.23	6.26	6.71	6.79
1368651_at	Pklr	0	0	0	0	0	0	6.10	6.47	6.66	6.06	6.27	1	0	0	0	0	0	1	8.82	8.71	8.84	8.89	8.70	8.47	7.89
1368662_at	Rnf39	0	0	0	0	0	0	5.15	5.42	5.07	5.45	5.27	1	0	0	0	0	0	1	7.36	7.43	7.48	7.59	7.51	7.12	6.29
1368686_at	Ambp	0	0	0	0	0	0	6.24	6.39	6.41	5.87	6.23	1	0	0	0	0	1	1	12.16	12.19	12.13	11.88	11.81	10.64	10.50
1368687_at	Tesk1	0	0	0	0	0	0	6.53	6.50	6.13	6.44	6.49	1	0	0	0	0	1	1	7.24	7.08	7.21	7.14	7.04	6.65	6.49
1368696_at	Fxyd7	0	0	0	0	0	0	4.28	4.21	3.99	4.44	3.99	1	0	0	0	0	1	1	4.57	4.58	4.49	4.43	4.57	5.52	5.98
1368700_at	Plcl1	0	0	0	0	0	0	5.34	5.18	5.48	4.99	5.26	1	0	0	0	0	1	1	8.73	8.63	8.92	9.07	9.00	9.57	9.70
1368702_at	Pawr	0	0	0	0	0	0	6.34	6.19	6.28	6.61	6.10	1	0	0	0	0	0	1	7.04	7.03	7.15	7.31	7.31	7.63	7.94
1368703_at	Pdlim5	0	0	0	0	0	0	5.18	5.39	5.03	5.56	5.17	1	0	0	0	0	0	1	4.82	4.75	4.70	4.83	4.79	5.10	5.39
1368717_at	Faah	0	0	0	0	0	0	6.37	6.35	6.39	6.29	6.27	1	0	0	0	0	0	1	8.73	8.80	8.83	8.82	8.72	8.28	7.70
1368721_at	Ascl2	0	0	0	0	0	0	4.84	4.78	4.79	4.76	4.83	1	0	0	0	0	0	1	6.31	6.44	6.51	6.46	6.33	5.92	5.63
1368732_at	Tap2	0	0	0	0	0	0	5.00	5.01	5.04	5.13	4.79	1	0	0	0	0	0	1	6.85	6.75	6.81	6.67	6.86	7.00	6.86
1368741_at	C9	0	0	0	0	0	0	4.57	4.54	4.28	4.42	4.52	1	0	0	0	0	1	1	7.54	7.53	7.47	7.53	7.53	6.36	5.50
1368748_at	Tesk2	0	0	0	0	0	0	3.91	3.71	3.95	4.00	3.85	1	0	0	0	0	1	1	5.33	5.32	5.47	5.60	5.75	6.03	6.32
1368750_a_at	Pde4d	0	0	0	0	0	0	4.06	4.16	3.81	4.05	4.05	1	0	0	0	0	0	1	5.49	5.41	5.53	5.48	5.45	5.08	4.71
1368751_at	Kcns3	0	0	0	0	0	0	3.46	3.52	3.35	3.44	3.42	1	0	0	0	0	1	1	5.21	5.05	5.49	5.47	5.58	6.94	6.77
1368756_at	Olah	0	0	0	0	0	0	4.60	4.39	4.14	4.41	4.20	1	0	0	0	0	1	1	5.60	5.46	5.55	5.32	5.12	4.91	4.66
1368758_a_at	Galr2	0	0	0	0	0	0	5.51	5.45	5.27	5.08	5.51	1	0	0	0	0	1	1	7.79	7.86	7.76	7.65	7.50	5.59	4.94
1368759_at	Cacng2	0	0	0	0	0	0	4.40	4.43	4.12	4.16	4.10	1	0	0	0	0	1	1	8.96	8.96	8.94	8.72	8.74	8.08	7.44
1368773_at	Cenpi	0	0	0	0	0	0	3.65	3.78	3.77	3.87	3.74	1	0	0	0	0	1	0	6.99	6.92	6.92	6.99	6.93	6.39	6.38
1368777_at	Bard1	0	0	0	0	0	0	4.16	4.20	3.95	4.12	4.18	1	0	0	0	0	1	1	5.88	5.72	5.77	5.68	5.51	5.02	4.91
1368799_at	Birc5	0	0	0	0	0	0	4.61	4.76	4.56	4.90	4.63	1	0	0	0	0	1	1	8.87	8.73	8.90	8.70	8.65	7.91	8.14
1368806_at	Selenop	0	0	0	0	0	0	13.04	12.92	12.82	13.04	12.89	1	0	1	1	1	1	1	4.88	5.09	5.53	6.17	6.30	8.53	9.39
1368811_at	Lmnb1	0	0	0	0	0	0	4.13	4.07	4.11	4.18	4.20	1	0	0	0	0	1	1	7.96	7.91	7.94	7.60	7.77	7.16	6.71
1368812_at	Lmnb1	0	0	0	0	0	0	3.54	3.66	3.35	3.36	3.40	1	0	0	0	0	1	1	6.67	6.65	6.66	6.34	6.44	5.76	4.51
1368813_at	Cebpd	0	0	0	0	0	0	4.04	3.88	4.15	3.87	4.00	1	0	0	0	0	1	1	10.62	10.69	10.60	10.26	10.34	9.61	7.85
1368816_at	Mpz	0	0	0	0	0	0	4.15	4.20	3.95	4.05	4.05	1	0	0	0	1	1	0	5.41	5.75	5.85	5.95	6.14	6.24	5.66
1368820_at	Nfyc	0	0	0	0	0	0	6.01	5.80	6.17	5.77	5.63	1	0	0	0	0	1	1	6.96	6.95	7.10	7.00	6.90	6.17	6.02
1368851_at	Ets1	0	0	0	0	0	0	6.97	6.67	7.07	6.73	6.95	1	0	0	0	0	0	1	6.09	6.02	6.19	6.21	6.13	6.34	7.40
1368860_at	Phlda1	0	0	0	0	0	0	3.70	3.62	3.61	3.32	3.38	1	0	0	0	0	0	1	6.70	6.67	6.99	6.83	6.83	7.22	6.35
1368861_a_at	Mag	0	0	0	0	0	0	4.10	4.09	3.77	4.20	4.02	1	0	0	0	0	1	1	4.91	5.00	5.05	4.94	5.03	5.74	6.04
1368863_at	Nme3	0	0	0	0	0	0	8.56	8.56	8.82	8.53	8.56	1	0	0	0	0	0	1	9.68	9.68	9.74	9.80	9.74	9.33	8.83
1368867_at	Ago2	0	0	0	0	0	0	6.18	6.15	6.26	6.31	6.32	1	0	0	0	0	1	0	9.62	9.36	9.48	9.39	9.31	9.00	9.31
1368882_at	St6galnac3	0	0	0	0	0	0	4.21	4.31	4.37	4.38	4.43	1	0	0	0	0	0	1	4.53	4.30	4.36	4.36	4.31	4.68	5.17
1368883_at	Ccn3	0	0	0	0	0	0	6.17	6.42	6.44	6.11	6.15	1	0	0	0	0	0	1	9.69	9.77	9.76	9.88	10.03	9.71	8.86
1368884_at	Entpd1	0	0	0	0	0	0	5.19	5.34	5.06	5.26	4.83	1	0	0	0	0	1	0	5.58	5.54	5.67	5.84	5.96	6.19	5.88
1368919_at	Pgf	0	0	0	0	0	0	5.08	5.25	4.92	5.10	5.08	1	0	0	0	0	0	1	5.97	6.15	6.08	5.94	6.08	6.33	6.15
1368939_a_at	Ntrk3	0	0	0	0	0	0	3.88	3.86	3.64	3.75	3.59	1	0	0	0	0	1	1	5.16	5.07	5.32	5.23	5.38	5.81	5.69
1368945_at	Bmp2	0	0	0	0	0	0	3.27	3.31	3.10	3.50	3.32	1	0	0	0	0	0	1	6.66	6.51	6.56	6.53	6.25	6.85	7.75
1368952_at	Gabbr2	0	0	0	0	0	0	4.99	5.08	4.62	4.74	4.73	1	0	0	0	0	0	1	5.29	5.20	5.31	5.26	5.18	4.95	4.13
1368962_at	Nxph3	0	0	0	0	0	0	4.39	4.63	4.32	4.19	4.31	1	0	0	0	0	1	0	4.85	5.14	5.22	5.31	5.38	5.95	5.17
1368965_at	Slc16a3	0	0	0	0	0	0	5.10	4.96	4.77	4.90	4.89	1	0	0	0	0	1	0	8.69	8.96	8.70	8.33	8.55	8.05	8.14
1368972_at	Ntrk2	0	0	0	0	0	0	4.37	4.36	4.54	4.31	4.21	1	0	0	0	0	0	1	6.95	7.09	7.12	6.95	7.08	6.52	5.64
1369006_at	Hk2	0	0	0	0	0	0	3.64	3.64	3.45	3.47	3.48	1	0	0	1	0	1	0	6.42	6.16	6.14	5.64	5.85	5.82	5.77
1369020_at	Slc5a5	0	0	0	0	0	0	3.29	3.47	3.23	3.38	3.43	1	0	0	0	0	0	1	4.59	4.52	4.52	4.30	4.47	4.99	5.76
1369025_at	Cd5	0	0	0	0	0	0	3.86	4.02	3.93	3.75	4.07	1	0	0	0	0	1	1	5.92	5.98	6.13	6.25	6.35	6.82	7.09
1369036_at	Grik2	0	0	0	0	0	0	3.50	3.52	3.28	3.37	3.32	1	0	0	0	0	1	0	5.01	5.15	5.20	5.22	5.25	5.73	5.64
1369041_at	Nlgn1	0	0	0	0	0	0	3.66	3.91	3.99	3.41	3.77	1	0	0	0	0	1	1	4.93	5.14	5.10	5.36	5.25	5.59	5.66
1369048_at	Gabrd	0	0	0	0	0	0	5.15	5.43	5.21	5.06	5.04	1	0	0	0	0	1	1	8.01	8.12	8.26	8.41	8.57	9.21	8.68
1369068_at	Cul5	0	0	0	0	0	0	4.16	4.56	4.66	4.46	4.21	1	0	0	0	0	0	1	5.35	5.36	5.41	5.53	5.31	4.89	4.41
1369098_at	Vldlr	0	0	0	0	0	0	6.32	6.30	6.72	6.62	6.56	1	0	0	0	0	1	1	8.81	8.72	8.66	8.25	8.29	7.48	6.84
1369102_at	Mapk10	0	0	0	0	0	0	3.90	3.91	4.02	3.84	4.09	1	0	0	0	0	1	1	5.96	5.92	5.92	5.72	5.59	5.16	5.12
1369103_at	Fyn	0	0	0	0	0	0	3.86	3.97	3.99	3.95	3.87	1	0	0	0	0	0	1	7.74	7.74	7.81	7.94	7.74	7.50	6.79
1369115_at	Adrb2	0	0	0	0	0	0	3.40	3.34	3.22	3.26	3.33	1	0	0	0	0	0	1	4.12	4.04	4.12	4.06	4.00	4.53	4.80
1369128_at	Grik5	0	0	0	0	0	0	5.81	5.56	5.46	5.87	5.61	1	0	0	0	0	0	1	8.29	8.31	8.43	8.36	8.24	7.73	7.35
1369155_at	Cntn4	0	0	0	0	0	0	4.32	4.30	4.07	4.32	4.26	1	0	0	0	0	1	1	5.43	5.58	5.69	5.83	5.96	6.89	6.12
1369162_at	Gucy2c	0	0	0	0	0	0	5.75	6.16	6.26	6.00	6.06	1	0	0	0	0	1	1	10.03	9.92	9.90	9.82	9.72	9.39	9.38
1369164_a_at	Trpc4	0	0	0	0	0	0	3.30	3.18	3.26	3.29	3.14	1	0	0	0	1	1	0	8.65	8.79	9.05	9.21	9.33	9.25	9.12
1369166_at	Mmp9	0	0	0	0	0	0	3.42	3.28	3.18	3.27	3.30	1	0	0	0	0	1	0	3.90	4.13	4.19	3.99	4.26	4.63	4.23
1369183_at	Mapk13	0	0	0	0	0	0	5.28	5.13	5.21	5.03	5.10	1	0	0	0	0	1	0	7.92	8.10	8.16	8.17	8.30	8.65	8.14
1369189_at	Npy4r	0	0	0	0	0	0	5.49	5.26	5.25	5.19	5.23	1	0	0	0	0	1	0	6.03	5.91	6.18	6.24	6.21	6.81	5.53
1369192_at	Cdkn1b	0	0	0	0	0	0	6.06	6.09	6.51	5.85	6.06	1	0	0	0	0	1	0	8.38	8.20	8.23	7.99	8.06	7.70	6.60
1369193_at	Cdkn2b	0	0	0	0	0	0	4.69	4.61	4.54	4.57	4.52	1	0	0	0	0	1	0	6.07	6.15	6.10	6.01	6.06	6.68	6.58
1369194_a_at	Cdkn2a	0	0	0	0	0	0	3.27	3.26	3.20	3.09	3.25	1	0	0	0	0	1	0	9.03	8.90	9.03	9.02	9.12	9.72	9.90
1369202_at	Mx1	0	0	0	0	0	0	2.89	2.98	2.88	2.91	2.78	1	0	0	0	0	1	1	4.53	4.46	4.54	4.65	4.88	5.22	5.63
1369204_at	Hck	0	0	0	0	0	0	4.13	4.35	3.90	4.09	4.28	1	0	0	0	0	1	1	4.87	5.00	5.27	5.24	5.39	6.15	6.85
1369210_at	Scn1a	0	0	0	0	0	0	3.13	3.24	3.08	3.11	2.99	1	0	0	0	0	0	1	5.67	5.76	5.87	5.98	6.02	6.24	6.42
1369213_at	L1cam	0	0	0	0	0	0	5.17	5.16	5.23	5.27	5.29	1	0	0	0	0	1	1	6.87	6.95	6.98	6.96	7.03	7.73	8.08
1369222_at	Kcnt1	0	0	0	0	0	0	4.84	4.72	4.53	4.77	4.66	1	0	0	0	0	1	0	5.14	5.30	5.33	5.26	5.31	5.74	5.45
1369224_at	Cdh17	0	0	0	0	0	0	5.20	5.04	4.82	4.90	4.89	1	0	0	0	0	1	1	6.00	5.86	5.99	6.24	6.53	7.59	7.07
1369255_at	Il1r1	0	0	0	0	0	0	4.28	4.58	4.75	4.19	4.29	1	0	0	0	0	0	1	7.53	7.41	7.53	7.61	7.65	7.59	5.84
1369304_at	Pts	0	0	0	0	0	0	6.96	6.80	7.43	7.34	7.21	1	0	0	0	0	1	1	6.98	7.05	7.18	7.41	7.41	7.73	7.45
1369305_at	Rab3il1	0	0	0	0	0	0	3.49	3.61	3.46	3.49	3.60	1	0	0	0	0	1	1	5.78	5.76	6.00	6.02	6.06	7.09	6.72
1369308_at	Stx3	0	0	0	0	0	0	4.56	4.52	4.58	4.28	4.49	1	0	0	0	0	0	1	7.37	7.37	7.37	7.21	7.13	6.82	5.78
1369309_a_at	Tac1	0	0	0	0	0	0	4.12	4.04	3.76	3.85	3.84	1	0	0	0	0	1	1	12.64	12.78	12.63	12.41	12.53	11.68	9.51
1369333_a_at	Rims2	0	0	0	0	0	0	3.42	3.76	3.74	3.50	3.58	1	0	0	0	0	1	1	7.16	7.12	7.28	7.21	7.09	6.53	6.15
1369358_a_at	Hap1	0	0	0	0	0	0	9.18	9.24	9.23	8.84	8.89	1	0	0	0	0	1	1	8.89	8.89	9.10	9.25	9.24	9.81	9.52
1369380_at	Kif2c	0	0	0	0	0	0	3.62	3.55	3.52	3.62	3.54	1	0	0	0	0	1	1	7.67	7.59	7.70	7.62	7.44	6.54	6.62
1369390_a_at	Dpp6	0	0	0	0	0	0	4.53	4.43	4.64	4.19	4.51	1	0	0	0	0	1	1	9.30	9.41	9.29	8.97	9.08	8.21	6.78
1369414_at	Stxbp3	0	0	0	0	0	0	7.03	6.98	7.44	6.89	7.04	1	0	0	0	0	1	1	7.50	7.61	7.68	7.73	7.73	8.19	8.70
1369415_at	Bhlhe40	0	0	0	0	0	0	4.89	5.02	4.72	4.67	5.04	1	0	0	1	0	1	1	9.66	9.54	9.37	8.97	9.11	8.67	8.20
1369445_at	Mre11	0	0	0	0	0	0	4.43	4.51	4.32	3.99	4.25	1	0	0	0	0	0	1	6.80	6.70	6.99	6.98	6.72	6.39	5.43
1369469_s_at	Spdya	0	0	0	0	0	0	3.37	3.50	3.34	3.31	3.19	1	0	0	0	0	1	1	5.11	5.13	5.28	5.21	5.33	6.49	6.90
1369473_at	Pgm1	0	0	0	0	0	0	5.82	5.87	6.23	5.66	6.03	1	0	0	0	0	1	1	9.53	9.52	9.40	9.19	9.12	8.71	8.24
1369482_a_at	Syn2	0	0	0	0	0	0	3.31	3.17	3.14	3.27	3.15	1	0	0	0	0	0	1	4.20	4.18	4.24	4.11	4.13	4.50	4.44
1369488_at	Fut4	0	0	0	0	0	0	3.69	3.63	3.64	3.51	3.46	1	0	0	0	0	1	1	7.60	7.64	7.61	7.66	7.49	6.92	5.05
1369493_at	Prlr	0	0	0	0	0	0	4.96	5.06	4.93	4.93	5.00	1	0	0	0	0	1	1	6.56	6.45	6.37	6.27	6.00	5.49	5.28
1369500_at	Kcnk1	0	0	0	0	0	0	5.65	5.31	5.99	5.50	5.43	1	0	0	0	0	0	1	9.11	9.19	9.08	8.87	8.96	8.72	7.60
1369509_a_at	A1bg	0	0	0	0	0	0	3.13	3.23	3.22	3.18	3.44	1	0	0	0	0	1	0	6.61	6.49	6.46	6.24	6.11	5.88	6.16
1369518_at	Pik3r3	0	0	0	0	0	0	5.23	5.41	5.62	5.21	5.33	1	0	0	0	0	1	1	6.47	6.19	6.52	6.66	6.57	7.60	7.61
1369525_at	Gata3	0	0	0	0	0	0	4.29	4.58	4.38	4.43	4.35	1	0	0	0	0	0	1	4.26	4.28	4.45	4.52	4.46	4.60	5.84

Probeset ID	Gene Symbol	Islet Data Set (log2 ratio ≥0.263; FDR <20%) union n=5325 1=YES 0=NO	Islet DRlyp/lyp ND vs ND+Lp299v (log2 ratio ≥0.263; FDR<20%) n=429 1=YES 0=NO	Islet DRlyp/lyp ND vs HCD (log2 ratio ≥0.263; FDR<20%) n=2247 1=YES 0=NO	Islet DRlyp/lyp ND vs HCD+Lp299v (log2 ratio ≥0.263; FDR<20%) n=1595 1=YES 0=NO	Islet DRlyp/lyp HCD vs HCD+Lp299v (log2 ratio ≥0.263; FDR<20%) n=3026 1=YES 0=NO	Islet DRlyp/lyp ND vs Flyp/lyp ND (log2 ratio ≥ 0.263; FDR<20%) n=1950 1=YES 0=NO	Islet Mean Log2 Intensity DRlyp/lyp ND	Islet Mean Log2 Intensity DRlyp/lyp ND+Lp299v	Islet Mean Log2 Intensity DRlyp/lyp HCD	Islet Mean Log2 Intensity DRlyp/lyp HCD+Lp299v	Islet Mean Log2 Intensity Flyp40 ND	Rinm5f data union of all conditions n=3705 1=YES 0=NO	Rinm5f 30 µM propionate/ 6 µM butyrate vs Media (FDR<20%; 1.5 fold) n=3 1=YES 0=NO	Rinm5f 60 µM propionate/ 12 µM butyrate vs Media (FDR<20%; 1.5 fold) n=18 1=YES 0=NO	Rinm5f 120 µM propionate/ 24 µM butyrate vs Media (FDR<20%; 1.5 fold) n=164 1=YES 0=NO	Rinm5f 240 µM propionate /48 µM butyrate vs Media (FDR<20%; 1.5 fold) n=268 1=YES 0=NO	Rinm5f 1500 µM propionate/ 300 µM butyrate vs Media (FDR<20%; 1.5 fold) n=2491 1=YES 0=NO	Rinm5f 500 µM propionate/ 500 µM butyrate vs Media (FDR<20%; 1.5 fold) n=3001 1=YES 0=NO	Rinm5f Mean Log2 Intensity Media only	Rinm5f Mean Log2 Intensity 30 µM propionate/ 6 µM butyrate	Rinm5f Mean Log2 Intensity 60 µM propionate/ 12 µM butyrate	Rinm5f Mean Log2 Intensity 120 µM propionate/ 24 µM butyrate	Rinm5f Mean Log2 Intensity 240 µM propionate/ 48 µM butyrate	Rinm5f Mean Log2 Intensity 1500 µM propionate /300 µM butyrate	Rinm5f Mean Log2 Intensity 500 µM propionate/ 500 µM butyrate
1369536_at	Edn2	0	0	0	0	0	0	4.30	4.44	4.28	4.40	4.39	1	0	0	0	0	0	1	4.68	4.65	4.76	4.70	4.70	4.90	5.74
1369560_at	Gpd1	0	0	0	0	0	0	3.95	4.07	3.85	3.94	4.07	1	0	0	0	0	0	1	7.69	7.78	7.84	8.00	7.93	7.75	7.25
1369567_at	Taar1	0	0	0	0	0	0	5.21	5.34	5.30	5.14	5.14	1	0	0	0	0	1	1	5.94	5.86	5.84	5.76	5.63	5.15	4.57
1369583_at	Jdp2	0	0	0	0	0	0	4.43	4.40	4.32	4.36	4.22	1	0	0	0	0	1	1	7.03	6.95	7.05	7.07	6.97	5.92	5.12
1369590_a_at	Ddit3	0	0	0	0	0	0	6.65	6.72	6.35	6.68	6.40	1	0	0	0	0	1	1	8.79	8.80	8.78	8.70	8.54	7.99	7.34
1369622_at	Prok2	0	0	0	0	0	0	3.56	3.58	3.64	3.55	3.62	1	0	0	1	1	1	0	5.98	5.81	5.61	5.10	5.10	4.98	5.75
1369630_at	Adk	0	0	0	0	0	0	9.49	9.34	9.53	9.38	9.47	1	0	0	0	0	0	1	9.72	9.75	9.75	9.82	9.80	10.08	10.03
1369633_at	Cxcl12	0	0	0	0	0	0	6.89	6.72	6.68	6.50	7.17	1	0	0	0	0	0	1	6.82	6.80	6.65	6.58	6.71	7.39	7.03
1369657_at	Cpa1	0	0	0	0	0	0	14.53	14.52	14.56	14.59	14.51	1	0	0	0	0	1	1	4.06	4.03	4.02	3.99	4.14	4.83	5.07
1369660_at	Defb1	0	0	0	0	0	0	5.62	5.83	6.12	5.78	5.76	1	0	0	0	1	1	0	6.77	6.86	6.89	7.08	7.36	7.93	7.25
1369662_at	Scn2a	0	0	0	0	0	0	3.58	3.51	3.79	3.39	3.62	1	0	0	0	0	1	0	4.51	4.40	4.57	4.87	4.97	5.14	4.77
1369663_at	Ephx2	0	0	0	0	0	0	6.46	6.47	6.75	6.54	6.28	1	0	0	0	0	0	1	7.56	7.61	7.68	7.56	7.49	8.11	8.02
1369665_a_at	Il18	0	0	0	0	0	0	3.80	4.01	3.94	3.62	4.12	1	0	0	0	0	1	0	6.39	6.51	6.66	6.83	6.82	6.98	6.63
1369666_at	Gpd2	0	0	0	0	0	0	6.94	6.89	7.37	7.04	7.31	1	0	0	0	0	1	1	7.10	7.03	7.26	7.21	7.26	7.78	8.36
1369667_at	Vps52	0	0	0	0	0	0	5.80	5.69	5.54	5.53	5.43	1	0	0	0	0	0	1	8.20	8.28	8.33	8.34	8.19	7.94	7.19
1369683_at	Bid	0	0	0	0	0	0	3.55	3.49	3.55	3.53	3.56	1	0	0	0	0	1	1	5.65	5.84	5.84	5.78	5.83	6.26	5.87
1369686_at	Dclk1	0	0	0	0	0	0	3.58	3.61	3.48	3.52	3.54	1	0	0	0	0	1	0	8.06	7.98	8.01	7.81	7.69	7.26	7.38
1369703_at	Epas1	0	0	0	0	0	0	5.20	5.27	5.16	4.99	5.12	1	0	0	0	0	1	0	7.22	7.09	7.03	6.88	6.87	6.50	5.59
1369723_at	Xylt2	0	0	0	0	0	0	4.79	4.50	4.56	4.66	4.58	1	0	0	0	0	1	0	6.79	6.85	7.01	6.94	7.02	7.58	7.03
1369737_at	Crem	0	0	0	0	0	0	3.33	3.57	3.53	3.41	3.54	1	0	0	0	0	1	1	7.74	7.81	7.84	7.96	7.97	7.16	6.19
1369738_s_at	Crem	0	0	0	0	0	0	5.81	5.99	5.88	5.98	5.83	1	0	0	0	0	0	1	8.77	8.79	8.84	8.80	8.78	8.26	7.63
1369755_at	B3gat2	0	0	0	0	0	0	3.53	3.66	3.50	3.42	3.52	1	0	0	0	0	0	1	4.43	4.25	4.11	4.02	4.03	4.95	5.66
1369775_at	Nucks1	0	0	0	0	0	0	7.18	7.36	7.13	7.41	7.11	1	0	0	0	0	1	1	9.98	9.87	9.95	9.90	9.81	9.15	9.20
1369787_at	Cckar	0	0	0	0	0	0	11.22	10.94	11.15	11.14	10.89	1	0	0	0	0	1	1	4.53	4.44	4.54	4.65	4.91	5.32	5.61
1369820_at	Mcf2l	0	0	0	0	0	0	5.60	5.81	5.87	5.54	6.02	1	0	0	0	0	1	1	6.75	6.65	6.75	6.70	6.72	5.99	5.13
1369822_at	Kit	0	0	0	0	0	0	4.94	4.90	4.91	4.49	4.56	1	0	0	0	0	1	0	9.67	9.64	9.84	10.00	10.02	10.59	9.67
1369837_at	Gulo	0	0	0	0	0	0	5.63	5.66	5.83	5.63	5.53	1	0	0	0	0	0	1	5.01	4.93	4.88	5.00	5.02	5.48	6.04
1369841_at	Hspa2	0	0	0	0	0	0	3.27	3.47	3.51	3.44	3.21	1	0	0	0	0	1	1	4.85	4.97	5.06	5.23	5.39	5.51	5.89
1369866_at	Cym	0	0	0	0	0	0	3.49	3.49	3.33	3.49	3.45	1	0	0	1	1	0	0	7.41	7.32	7.73	8.21	8.17	7.77	6.79
1369868_at	Magt1	0	0	0	0	0	0	9.14	9.12	9.30	8.84	9.11	1	0	0	0	0	1	0	9.32	9.37	9.43	9.61	9.66	9.99	9.65
1369871_at	Areg	0	0	0	0	0	0	2.90	2.97	3.12	2.90	3.25	1	0	0	0	0	0	1	4.15	4.01	4.08	4.13	4.07	4.60	5.16
1369891_at	Batf3	0	0	0	0	0	0	4.50	4.55	4.38	4.47	4.41	1	0	0	0	0	1	1	6.66	7.14	7.24	7.12	7.21	7.97	8.08
1369905_at	Gabra4	0	0	0	0	0	0	3.83	3.91	3.83	3.63	3.69	1	0	0	0	0	1	1	6.80	6.75	6.90	6.97	7.04	7.83	7.32
1369924_at	Sncb	0	0	0	0	0	0	5.35	5.43	5.10	5.09	5.13	1	0	0	0	0	1	1	9.02	9.06	8.92	8.56	8.53	8.36	7.61
1369928_at	Acta1	0	0	0	0	0	0	4.06	4.09	4.23	3.89	4.08	1	0	0	0	0	1	1	6.74	6.86	6.81	6.89	6.69	6.14	5.85
1369951_at	Ctrb2	0	0	0	0	0	0	14.59	14.59	14.54	14.52	14.62	1	0	0	0	0	1	1	4.66	4.64	4.61	4.71	4.77	5.40	5.73
1369956_at	Ifngr1	0	0	0	0	0	0	8.22	7.82	8.15	8.23	7.88	1	0	0	0	0	0	1	8.76	8.86	8.76	8.69	8.65	8.23	7.89
1369990_at	Ptpmt1	0	0	0	0	0	0	8.05	8.01	8.11	8.26	7.91	1	0	0	0	0	1	1	9.72	9.69	9.66	9.68	9.55	8.90	8.47
1370020_at	Slc25a10	0	0	0	0	0	0	6.63	6.58	6.43	6.91	6.33	1	0	0	0	0	1	1	9.81	9.69	9.81	9.65	9.58	9.13	8.86
1370032_at	Slc9a3r1	0	0	0	0	0	0	4.34	4.19	4.03	4.11	4.01	1	0	0	0	0	0	1	5.29	5.35	5.37	5.18	5.29	5.86	6.02
1370043_at	Alcam	0	0	0	0	0	0	8.21	8.17	8.49	8.44	8.35	1	0	0	0	0	1	1	8.61	8.46	8.46	8.38	8.36	9.45	9.84
1370054_at	Cdkn2c	0	0	0	0	0	0	7.43	7.40	7.58	7.48	7.79	1	0	0	0	0	1	1	10.14	10.08	10.07	9.93	9.81	9.38	9.24
1370058_at	Nefl	0	0	0	0	0	0	4.33	4.49	4.11	4.30	4.37	1	0	0	0	0	0	1	7.21	7.26	7.28	7.37	7.27	7.76	7.50
1370059_at	Nefl	0	0	0	0	0	0	3.18	3.25	2.97	3.22	3.18	1	0	0	0	0	1	1	5.95	6.01	6.16	6.39	6.11	6.71	6.96
1370071_at	Ada	0	0	0	0	0	0	5.04	4.93	4.96	5.31	4.99	1	0	0	0	0	1	1	6.73	6.89	7.03	7.16	7.22	7.95	8.82
1370075_at	Dhfr	0	0	0	0	0	0	6.53	6.61	6.69	6.63	6.58	1	0	0	0	0	1	1	10.81	10.73	10.54	10.27	10.24	9.98	9.74
1370079_at	Rhce /// Rhd	0	0	0	0	0	0	4.48	4.50	4.26	4.41	4.44	1	0	0	0	1	1	1	5.44	5.62	5.76	6.01	6.30	7.39	7.12
1370089_at	Ppargc1a	0	0	0	0	0	0	3.18	3.19	3.28	3.41	3.12	1	0	0	1	1	0	0	6.72	6.31	6.27	6.10	6.03	6.21	6.56
1370102_at	Kcnn1	0	0	0	0	0	0	5.78	5.86	5.51	5.60	5.70	1	0	0	0	0	1	0	7.05	7.23	7.22	7.15	7.19	7.66	7.28
1370108_a_at	Lin7a	0	0	0	0	0	0	3.62	3.67	3.49	3.50	3.66	1	0	0	0	0	1	1	7.63	7.44	7.48	7.47	7.22	6.34	5.58
1370111_at	Kcnn2	0	0	0	0	0	0	5.55	5.52	5.76	5.67	5.62	1	0	0	0	0	1	0	6.43	6.52	6.75	6.82	6.97	7.47	6.73
1370120_at	Fstl3	0	0	0	0	0	0	3.85	3.98	3.68	3.99	3.87	1	0	0	0	0	1	1	8.44	8.60	8.63	8.57	8.80	9.74	8.85
1370124_at	Mt3	0	0	0	0	0	0	6.25	6.29	6.16	6.31	6.39	1	0	0	0	0	1	1	7.90	8.01	8.02	8.16	8.42	9.50	9.64
1370146_at	Glrb	0	0	0	0	0	0	3.37	3.35	3.33	3.37	3.40	1	0	0	0	1	1	0	6.02	6.03	6.32	6.52	6.62	6.64	5.27
1370153_at	Gdf15	0	0	0	0	0	0	4.20	4.45	4.10	4.00	4.11	1	0	0	0	0	1	1	5.45	5.63	5.76	5.73	5.89	6.06	6.30
1370183_at	Dyrk1a	0	0	0	0	0	0	5.71	5.60	5.63	5.95	5.74	1	0	0	0	0	1	1	8.03	7.97	7.96	7.99	7.87	7.41	7.12
1370214_at	Pvalb	0	0	0	0	0	0	3.52	3.47	3.52	3.73	3.45	1	0	0	0	1	1	1	5.79	6.06	5.89	6.18	6.50	7.32	6.92
1370216_at	Ddr1	0	0	0	0	0	0	5.10	4.88	4.88	4.96	5.01	1	0	0	0	0	0	1	8.13	8.06	8.14	8.04	8.04	7.74	7.04
1370220_at	Scpep1	0	0	0	0	0	0	9.06	8.84	9.21	9.15	8.83	1	0	0	0	0	1	1	8.63	8.69	8.78	8.69	8.84	9.53	9.74
1370245_at	Ctsv	0	0	0	0	0	0	7.15	6.94	7.31	7.20	6.84	1	0	0	0	1	1	1	8.43	8.47	8.67	8.90	9.02	9.68	9.77
1370252_at	Avpi1	0	0	0	0	0	0	6.38	6.35	6.24	6.34	6.42	1	0	0	0	0	0	1	9.41	9.48	9.37	9.36	9.47	9.98	9.98
1370266_at	Parva	0	0	0	0	0	0	5.43	5.34	5.33	5.32	5.13	1	0	0	0	0	1	1	6.86	6.68	6.79	6.84	6.60	5.90	5.30
1370285_at	Calcoco1	0	0	0	0	0	0	7.12	6.94	7.05	7.42	6.83	1	0	0	0	0	1	1	8.47	8.51	8.57	8.53	8.61	9.19	8.90
1370286_at	Slc38a2	0	0	0	0	0	0	9.75	9.47	9.64	9.71	9.36	1	0	0	0	0	1	1	11.11	11.08	11.09	11.08	11.05	10.43	9.88
1370294_a_at	Cdc20	0	0	0	0	0	0	6.14	6.23	6.27	6.36	6.38	1	0	0	0	0	1	1	9.53	9.39	9.45	9.35	9.07	8.00	7.97
1370297_at	Plk1	0	0	0	0	0	0	6.27	6.41	6.29	6.23	6.38	1	0	0	0	0	1	1	9.47	9.41	9.44	9.36	9.25	8.23	8.00
1370302_at	Shroom2	0	0	0	0	0	0	7.03	6.91	7.05	6.90	7.04	1	0	0	0	0	1	0	6.43	6.55	6.72	6.80	6.89	7.25	7.21
1370309_a_at	Hnrnpab	0	0	0	0	0	0	8.69	8.43	9.04	8.55	8.75	1	0	0	0	0	0	1	11.33	11.30	11.37	11.42	11.33	10.79	10.37
1370329_at	Cyp2d22	0	0	0	0	0	0	6.23	6.43	6.04	6.24	6.16	1	0	0	0	0	1	0	6.73	6.81	6.82	6.72	6.79	7.35	7.42
1370345_at	Ccnb1	0	0	0	0	0	0	4.55	4.70	4.96	4.62	5.02	1	0	0	0	0	1	1	9.85	9.75	9.83	9.80	9.64	8.53	8.44
1370346_at	Ccnb1	0	0	0	0	0	0	5.59	5.80	5.76	5.44	5.99	1	0	0	0	0	1	1	10.39	10.36	10.40	10.39	10.30	9.28	9.06
1370361_at	Cgref1	0	0	0	0	0	0	4.53	4.50	4.24	4.63	4.31	1	0	0	0	0	0	1	7.53	7.69	7.61	7.53	7.51	6.99	6.65
1370365_at	Gss	0	0	0	0	0	0	6.55	6.62	6.61	6.64	6.63	1	0	0	0	0	1	0	8.06	8.11	8.20	8.15	8.17	8.69	8.57
1370366_at	Timm10	0	0	0	0	0	0	7.53	7.37	7.46	7.47	7.46	1	0	0	0	0	0	1	9.82	9.83	9.85	9.85	9.84	9.56	8.97
1370367_at	Slc1a1	0	0	0	0	0	0	4.81	4.85	4.69	4.79	4.85	1	0	0	0	0	1	1	10.72	10.83	10.76	10.52	10.77	9.50	8.60
1370372_at	Rasd2	0	0	0	0	0	0	4.21	4.15	3.92	4.18	4.08	1	0	0	0	0	1	1	6.48	6.57	6.61	6.55	6.88	8.28	8.49
1370381_at	Pnrc1	0	0	0	0	0	0	8.92	9.14	9.13	9.37	9.26	1	0	0	0	0	1	1	9.83	9.88	9.78	9.57	9.57	9.20	8.80
1370386_at	Ruvbl1	0	0	0	0	0	0	6.21	6.11	6.32	6.21	6.20	1	0	0	0	0	0	1	8.41	8.46	8.56	8.54	8.42	7.87	7.53
1370391_at	Crabp2 /// LOC1	0	0	0	0	0	0	3.58	3.83	3.54	3.73	3.57	1	0	0	0	0	1	1	5.94	6.01	6.07	6.07	6.14	7.54	8.09
1370409_at	Slc38a1	0	0	0	0	0	0	5.78	5.60	5.53	5.82	5.74	1	0	0	0	0	1	1	8.05	7.93	8.05	8.03	7.90	7.12	6.94
1370415_at	Rassf5	0	0	0	0	0	0	5.05	5.14	5.02	5.21	5.04	1	0	0	0	0	0	1	6.76	6.68	6.76	6.67	6.62	7.12	7.03
1370432_at	Pou3f1	0	0	0	0	0	0	3.50	3.58	3.27	3.40	3.44	1	0	0	0	0	1	1	3.67	3.62	3.78	3.88	3.85	4.43	5.32
1370433_at	Hsd3b7	0	0	0	0	0	0	7.01	7.10	7.06	7.16	7.08	1	0	0	0	0	0	1	9.16	9.17	9.09	8.97	8.91	8.58	7.99
1370438_at	Nos1ap	0	0	0	0	0	0	5.26	5.15	5.38	5.34	5.27	1	0	0	0	0	1	1	5.66	5.84	5.91	5.75	5.78	6.36	6.70
1370447_at	Plb1	0	0	0	0	0	0	3.36	3.34	3.29	3.34	3.34	1	0	0	0	0	0	1	4.70	4.68	4.47	4.60	4.58	4.68	5.20
1370448_at	Gpc2	0	0	0	0	0	0	4.10	4.00	4.19	4.14	4.13	1	0	0	0	0	1	1	6.10	6.20	6.41	6.48	6.62	7.23	6.83
1370450_at	Tpm3	0	0	0	0	0	0	3.77	3.73	3.62	3.78	3.72	1	0	0	0	0	1	1	8.21	8.16	8.10	8.06	8.05	7.24	6.51
1370455_a_at	Olfm3	0	0	0	0	0	0	3.01	3.08	2.98	3.05	2.92	1	0	0	0	0	0	1	3.62	3.47	3.63	3.60	3.55	3.93	4.56
1370458_at	Hdgfl3	0	0	0	0	0	0	6.09	6.07	6.46	6.05	6.25	1	0	0	0	0	1	1	8.58	8.56	8.67	8.76	8.64	7.91	7.46
1370461_at	Hmmr	0	0	0	0	0	0	4.71	4.59	4.79	4.75	4.70	1	0	0	0	0	1	1	7.93	7.81	7.91	7.95	7.69	6.58	6.54
1370462_at	Hmmr	0	0	0	0	0	0	5.20	5.18	5.54	5.25	5.58	1	0	0	0	0	1	1	8.09	8.04	8.09	8.10	7.86	6.82	6.87
1370469_at	Ptpn7	0	0	0	0	0	0	6.33	6.27	5.98	6.19	6.14	1	0	0	0	0	1	1	6.40	6.51	6.65	6.65	6.88	7.69	7.65
1370472_a_at	Kcnma1	0	0	0	0	0	0	5.27	5.51	5.63	5.33	5.58	1	0	0	0	0	1	1	7.40	7.29	7.36	7.09	7.09	6.15	5.55
1370476_at	Stambp	0	0	0	0	0	0	6.05	6.23	6.42	6.08	6.40	1	0	0	0	0	0	1	8.66	8.71	8.69	8.54	8.47	8.21	7.80
1370486_a_at	Ppp1r32	0	0	0	0	0	0	4.38	4.57	4.37	4.39	4.45	1	0	0	0	0	1	0	6.11	6.25	6.36	6.28	6.40	6.91	6.76
1370490_at	Pcdhb12	0	0	0	0	0	0	3.71	3.82	3.76	3.73	3.76	1	0	0	0	1	0	0	7.35	7.52	7.61	7.91	8.05	7.92	7.18
1370502_at	Pam	0	0	0	0	0	0	4.14	4.18	4.35	3.94	4.12	1	0	0	0	0	0	1	6.81	7.01	6.98	6.89	7.17	6.47	5.60
1370511_at	Fgb	0	0	0	0	0	0	4.02	3.71	3.95	3.61	3.87	1	0	0	0	0	0	1	7.62	7.65	7.66	7.74	7.69	7.12	6.69
1370517_at	Nptx1	0	0	0	0	0	0	3.60	3.71	3.37	3.66	3.81	1	0	0	0	0	1	1	5.46	5.62	5.83	5.48	5.70	7.30	8.31
1370537_at	Xrcc6	0	0	0	0	0	0	5.52	5.63	5.64	5.54	5.67	1	0	0	0	0	1	1	8.83	8.86	8.97	9.10	9.14	9.54	9.43
1370553_at	Stx2	0	0	0	0	0	0	4.09	4.16	4.07	3.96	4.05	1	0	0	0	0	1	0	5.76	5.98	6.20	6.21	6.17	6.42	5.80
1370580_a_at	Cyp2c19	0	0	0	0	0	0	3.04	3.17	2.90	3.01	2.95	1	0	0	0	0	1	1	8.53	8.69	8.82	9.10	8.97	6.36	4.54
1370584_a_at	Adora1	0	0	0	0	0	0	3.46	3.40	3.47	3.47	3.45	1	0	0	0	0	1	0	6.80	6.84	7.08	7.01	7.02	7.39	6.77
1370598_a_at	Kcnj6	0	0	0	0	0	0	3.78	3.82	3.72	3.84	3.80	1	0	0	0	0	0	1	5.85	5.68	5.70	5.68	5.66	5.29	4.74
1370623_at	Fgl2	0	0	0	0	0	0	4.66	4.71	4.49	4.34	4.60	1	0	0	0	0	0	1	11.43	11.50	11.39	11.28	11.39	11.03	9.32
1370627_at	Rhov	0	0	0	0	0	0	6.11	5.93	5.99	5.80	5.69	1	0	0	0	0	1	0	7.59	7.66	7.77	7.86	7.90	8.18	7.66
1370650_s_at	Bdkrb2	0	0	0	0	0	0	3.85	3.71	3.66	3.79	3.77	1	0	0	0	0	0	1	4.05	4.02	3.97	3.94	4.03	4.15	5.13
1370652_at	Ntrk2	0	0	0	0	0	0	3.92	3.99	4.07	3.94	4.05	1	0	0	0	0	0	1	5.62	5.64	5.72	5.37	5.44	5.05	4.74
1370658_a_at	St18	0	0	0	0	0	0	7.09	7.02	7.27	6.70	7.27	1	0	0	0	0	0	1	8.53	8.62	8.62	8.71	8.71	8.74	7.47
1370662_a_at	Ap2b1	0	0	0	0	0	0	5.63	5.77	5.73	5.57	5.64	1	0	0	0	0	0	1	7.97	7.71	7.91	7.88	7.76	7.50	6.70
1370678_s_at	Maoa	0	0	0	0	0	0	7.95	7.94	8.29	7.84	8.33	1	0	0	0	0	1	1	8.84	8.85	8.96	9.06	9.01	9.59	10.05
1370689_at	Pam	0	0	0	0	0	0	3.17	3.21	2.95	3.20	3.01	1	0	0	0	0	1	1	5.35	5.28	5.16	4.83	4.84	4.36	3.51
1370775_a_at	Calcb	0	0	0	0	0	0	3.22	3.31	3.21	3.30	3.31	1	0	0	0	0	1	1	5.96	5.94	6.01	6.21	6.44	7.76	7.08
1370776_a_at	Kcnk6	0	0	0	0	0	0	6.50	6.49	6.14	6.17	6.31	1	0	0	0	0	0	1	6.71	6.91	6.76	6.71	6.81	6.75	5.78
1370802_at	Itgb5	0	0	0	0	0	0	4.82	4.70	4.78	5.09	4.78	1	0	0	0	0	1	1	6.51	6.64	6.90	6.92	7.05	7.75	7.57
1370808_at	Cyb5r3	0	0	0	0	0	0	8.35	8.25	8.10	8.24	8.22	1	0	0	0	0	1	0	9.86	9.92	9.98	9.95	9.97	10.45	10.46
1370814_at	Dhrs4	0	0	0	0	0	0	8.03	7.96	8.18	8.39	8.10	1	0	0	0	0	1	1	7.58	7.48	7.66	7.76	7.59	6.99	6.42
1370820_at	Fbxo6	0	0	0	0	0	0	8.15	8.16	8.23	7.95	8.27	1	0	0	0	0	1	1	9.57	9.58	9.48	9.36	9.22	8.77	8.51
1370821_at	Tpmt	0	0	0	0	0	0	5.77	5.76	5.85	6.23	6.16	1	0	0	0	0	1	1	8.55	8.53	8.51	8.58	8.71	9.44	9.47
1370831_at	Mgll	0	0	0	0	0	0	7.62	7.18	7.16	7.54	7.17	1	0	0	0	0	1	1	5.80	5.77	5.99	5.85	5.79	6.71	7.95
1370834_at	Hs3st1	0	0	0	0	0	0	4.61	4.55	4.54	4.65	4.36	1	0	0	1	0	1	1	8.48	8.50	8.31	7.90	8.18	6.33	6.08
1370843_at	Gng8	0	0	0	0	0	0	3.77	4.03	3.86	4.06	3.86	1	0	0	0	0	1	1	4.54	4.61	4.59	4.60	4.79	6.28	6.96
1370845_at	Entpd2	0	0	0	0	0	0	4.74	4.54	4.32	4.81	4.39	1	0	0	0	0	1	1	7.24	7.32	7.34	7.16	7.44	8.51	8.77
1370848_at	Slc2a1	0	0	0	0	0	0	5.97	5.93	5.77	5.81	5.93	1	0	0	0	0	1	0	9.83	9.92	9.73	9.34	9.43	9.16	9.17
1370853_at	Camk2n1	0	0	0	0	0	0	7.41	7.21	7.78	7.12	7.43	1	0	0	0	0	1	1	10.30	10.26	10.34	10.05	10.00	9.49	9.19
1370882_at	LOC100909630 /	0	0	0	0	0	0	5.99	6.00	5.73	5.78	6.12	1	0	0	0	0	0	1	5.21	5.19	5.27	4.95	5.05	5.67	6.78
1370884_at	Spr	0	0	0	0	0	0	6.53	6.66	6.55	6.64	6.56	1	0	0	1	0	1	1	11.82	11.88	11.67	11.22	11.32	10.75	10.50
1370885_at	Ctsz	0	0	0	0	0	0	5.76	5.83	5.67	5.92	5.66	1	0	0	0	0	0	1	6.12	6.12	6.22	6.16	6.28	6.46	6.82
1370904_at	LOC100909593 /	0	0	0	0	0	0	5.23	5.45	5.20	5.32	5.40	1	0	0	0	0	1	1	7.19	7.23	7.17	7.25	7.35	8.43	8.66
1370916_at	Tec	0	0	0	0	0	0	7.27	7.19	7.49	7.38	7.31	1	0	0	0	0	0	1	6.59	6.67	6.58	6.55	6.58	6.95	7.15
1370922_at	Ctxn1	0	0	0	0	0	0	3.87	3.89	3.67	3.79	3.77	1	0	0	1	1	1	0	5.98	6.22	6.49	6.60	6.66	7.09	6.21
1370925_at	Atp9a	0	0	0	0	0	0	9.23	9.21	9.37	9.37	9.34	1	0	0	0	0	1	1	10.79	10.74	10.83	10.80	10.75	10.20	9.67
1370933_at	Myo1e	0	0	0	0	0	0	5.76	5.91	5.65	5.93	6.23	1	0	0	0	0	0	1	6.16	6.17	6.08	6.06	6.19	6.40	6.81
1370939_at	Acsl1	0	0	0	0	0	0	7.31	6.97	7.47	7.55	7.21	1	0	0	0	0	1	1	8.99	9.04	9.06	9.19	9.22	9.68	9.77
1370968_at	Nfkb1	0	0	0	0	0	0	7.59	7.63	7.91	7.76	8.04	1	0	0	0	0	1	1	8.80	8.68	8.72	8.66	8.57	8.11	7.68

Probeset ID	Gene Symbol	Islet Data Set (log2 ratio ≥0.263; FDR <20%) union n=5325 1=YES 0=NO	Islet DRlyp/lyp ND vs ND+Lp299v (log2 ratio ≥0.263; FDR<20%) n=429 1=YES 0=NO	Islet DRlyp/lyp ND vs HCD (log2 ratio ≥0.263; FDR<20%) n=2247 1=YES 0=NO	Islet DRlyp/lyp ND vs HCD+Lp299v (log2 ratio ≥0.263; FDR<20%) n=1595 1=YES 0=NO	Islet DRlyp/lyp HCD vs HCD+Lp299v (log2 ratio ≥0.263; FDR<20%) n=3026 1=YES 0=NO	Islet DRlyp/lyp ND vs Flyp/lyp ND (log2 ratio ≥ 0.263; FDR<20%) n=1950 1=YES 0=NO	Islet Mean Log2 Intensity DRlyp/lyp ND	Islet Mean Log2 Intensity DRlyp/lyp ND+Lp299v	Islet Mean Log2 Intensity DRlyp/lyp HCD	Islet Mean Log2 Intensity DRlyp/lyp HCD+Lp299v	Islet Mean Log2 Intensity Flyp40 ND	Rinm5f data union of all conditions n=3705 1=YES 0=NO	Rinm5f 30 µM propionate/ 6 µM butyrate vs Media (FDR<20%; 1.5 fold) n=3 1=YES 0=NO	Rinm5f 60 µM propionate/ 12 µM butyrate vs Media (FDR<20%; 1.5 fold) n=18 1=YES 0=NO	Rinm5f 120 µM propionate/ 24 µM butyrate vs Media (FDR<20%; 1.5 fold) n=164 1=YES 0=NO	Rinm5f 240 µM propionate /48 µM butyrate vs Media (FDR<20%; 1.5 fold) n=268 1=YES 0=NO	Rinm5f 1500 µM propionate/ 300 µM butyrate vs Media (FDR<20%; 1.5 fold) n=2491 1=YES 0=NO	Rinm5f 500 µM propionate/ 500 µM butyrate vs Media (FDR<20%; 1.5 fold) n=3001 1=YES 0=NO	Rinm5f Mean Log2 Intensity Media only	Rinm5f Mean Log2 Intensity 30 µM propionate/ 6 µM butyrate	Rinm5f Mean Log2 Intensity 60 µM propionate/ 12 µM butyrate	Rinm5f Mean Log2 Intensity 120 µM propionate/ 24 µM butyrate	Rinm5f Mean Log2 Intensity 240 µM propionate/ 48 µM butyrate	Rinm5f Mean Log2 Intensity 1500 µM propionate /300 µM butyrate	Rinm5f Mean Log2 Intensity 500 µM propionate/ 500 µM butyrate
1370970_at	Kcnj14	0	0	0	0	0	0	5.36	5.44	5.35	5.36	5.25	1	0	0	0	0	0	1	6.60	6.53	6.67	6.66	6.56	6.14	5.86
1370975_at	Kdm3a	0	0	0	0	0	0	6.29	6.35	6.77	6.34	6.42	1	0	0	0	0	1	0	9.13	9.22	9.11	8.90	8.91	8.37	8.52
1370987_at	Spn	0	0	0	0	0	0	3.99	3.87	3.77	3.84	3.98	1	0	0	0	0	1	1	3.67	3.80	3.61	3.61	3.75	4.85	6.43
1370999_at	Spag5	0	0	0	0	0	0	3.72	3.60	3.66	3.85	3.88	1	0	0	0	0	1	1	9.13	9.02	8.97	8.99	8.85	7.81	7.71
1371015_at	Mx2	0	0	0	0	0	0	3.23	3.43	3.00	3.08	3.26	1	0	0	0	0	1	1	3.92	3.91	4.07	4.08	4.21	4.68	4.67
1371094_at	Lhx2	0	0	0	0	0	0	2.76	2.79	2.69	2.78	2.70	1	0	0	0	0	1	1	8.50	8.60	8.73	8.76	8.93	9.50	9.76
1371106_at	Itgb7	0	0	0	0	0	0	4.71	4.78	4.59	4.80	4.78	1	0	0	0	0	1	1	5.60	5.61	5.51	5.59	5.61	6.40	6.49
1371115_at	Ptpre	0	0	0	0	0	0	3.77	3.68	3.70	3.83	3.78	1	0	0	1	0	1	1	6.68	6.61	6.51	6.04	6.11	5.77	6.06
1371121_at	Sert1	0	0	0	0	0	0	4.70	4.46	4.74	4.57	4.38	1	0	0	0	0	0	1	6.81	7.04	6.89	6.74	6.66	6.33	5.99
1371152_a_at	Oas1	0	0	0	0	0	0	3.25	3.69	3.19	3.17	3.30	1	0	0	0	0	1	1	5.69	5.89	5.97	5.89	6.11	7.30	7.43
1371161_at	Ppp1r3b	0	0	0	0	0	0	2.92	3.08	2.83	2.92	2.91	1	0	0	0	0	1	0	6.81	6.74	6.74	6.30	6.39	6.21	4.96
1371167_at	Mpp3	0	0	0	0	0	0	4.28	4.27	4.17	4.16	4.10	1	0	0	0	0	1	1	6.32	6.45	6.53	6.53	6.62	7.02	6.60
1371210_s_at	Rt1-ec2	0	0	0	0	0	0	3.73	3.57	3.70	3.70	3.57	1	0	0	0	0	1	0	4.62	4.85	4.89	4.78	4.91	5.21	4.87
1371248_at	Sprr1a	0	0	0	0	0	0	5.11	5.01	5.05	5.10	5.14	1	0	0	0	0	1	0	6.28	6.17	6.08	6.25	6.21	6.88	6.69
1371249_at	Xbp1	0	0	0	0	0	0	11.66	11.41	11.52	11.82	11.33	1	0	0	0	0	0	1	12.58	12.64	12.67	12.62	12.65	12.30	11.77
1371345_at	Mbd3	0	0	0	0	0	0	7.92	8.18	7.83	8.26	8.09	1	0	0	0	0	1	1	9.83	9.75	9.76	9.61	9.42	8.81	8.54
1371350_at	Mat2a	0	0	0	0	0	0	8.65	8.63	8.83	8.49	8.65	1	0	0	0	0	1	0	10.58	10.67	10.75	10.80	10.92	11.22	11.04
1371353_at	Sqstm1	0	0	0	0	0	0	9.96	9.91	10.36	9.75	10.02	1	0	0	0	0	0	1	11.37	11.44	11.49	11.63	11.48	11.11	10.29
1371436_at	Ddah2	0	0	0	0	0	0	5.50	5.50	5.47	5.37	5.32	1	0	0	0	0	1	1	7.39	7.46	7.59	7.46	7.65	8.36	8.81
1371444_at	Cers2	0	0	0	0	0	0	8.10	7.94	8.24	8.06	8.35	1	0	0	0	0	0	1	9.90	9.91	9.92	9.87	9.82	9.43	8.96
1371462_at	Igfbp4	0	0	0	0	0	0	7.87	7.68	7.50	7.92	7.43	1	0	0	0	0	1	0	8.17	8.23	8.17	8.05	8.06	7.55	7.04
1371464_at	Zfand6	0	0	0	0	0	0	9.78	9.71	10.17	9.79	9.89	1	0	0	0	0	1	1	10.83	10.87	10.91	10.96	11.01	11.50	11.59
1371470_at	Adh5	0	0	0	0	0	0	8.54	8.40	8.60	8.77	8.21	1	0	0	0	0	1	1	9.34	9.43	9.53	9.58	9.58	10.04	10.28
1371480_at	Cks1b	0	0	0	0	0	0	7.34	7.10	7.22	7.33	7.50	1	0	0	0	0	0	1	9.63	9.65	9.61	9.58	9.50	9.17	8.74
1371485_at	Sh3pxd2a	0	0	0	0	0	0	7.35	7.25	7.53	7.26	7.45	1	0	0	0	0	0	1	6.37	6.28	6.46	6.44	6.42	6.73	7.21
1371498_at	Aimp2	0	0	0	0	0	0	7.52	7.38	7.33	7.55	7.46	1	0	0	0	0	0	1	9.56	9.53	9.56	9.54	9.44	9.05	8.61
1371530_at	Krt8	0	0	0	0	0	0	10.18	10.11	10.18	10.19	10.10	1	0	0	0	0	0	1	12.37	12.34	12.39	12.35	12.21	11.83	11.46
1371535_at	Pmm2	0	0	0	0	0	0	7.18	7.33	7.16	7.37	7.37	1	0	0	0	0	0	1	9.12	9.12	9.18	9.04	8.93	8.58	7.80
1371541_at	Mylk	0	0	0	0	0	0	7.09	7.13	7.21	7.01	7.27	1	0	0	0	0	0	1	6.97	6.92	7.07	7.10	7.08	7.55	7.97
1371543_at	Mtmr2	0	0	0	0	0	0	8.18	7.95	8.33	8.09	8.13	1	0	0	0	0	1	1	10.16	10.15	10.15	10.12	10.05	9.50	9.15
1371560_at	Irf3	0	0	0	0	0	0	7.74	7.90	7.81	8.03	7.69	1	0	0	0	0	1	1	9.03	9.08	9.21	9.23	9.30	9.81	9.44
1371588_at	Parva	0	0	0	0	0	0	6.49	6.40	6.23	6.71	6.21	1	0	0	0	0	1	1	7.97	7.91	7.96	7.89	7.77	6.81	7.10
1371594_at	Fis1	0	0	0	0	0	0	8.65	8.63	8.86	8.75	8.58	1	0	0	0	0	0	1	10.14	10.18	10.17	10.12	10.05	9.73	9.37
1371613_at	Elmo2	0	0	0	0	0	0	7.25	7.14	7.26	6.97	7.23	1	0	0	0	0	0	1	9.43	9.47	9.54	9.51	9.55	9.97	9.99
1371615_at	Dgat2	0	0	0	0	0	0	6.11	5.97	5.80	6.34	5.97	1	0	0	1	1	1	1	8.91	9.09	9.29	9.64	9.82	10.22	10.17
1371621_at	---	0	0	0	0	0	0	4.16	4.34	4.53	4.20	4.52	1	0	0	0	0	1	0	6.75	6.63	6.67	6.34	6.33	5.92	5.17
1371624_at	Zc3h7b	0	0	0	0	0	0	6.88	6.98	7.23	7.06	7.08	1	0	0	0	0	1	1	9.06	9.01	9.05	9.06	8.96	8.30	8.03
1371653_at	Tpm4	0	0	0	0	0	0	8.48	8.28	8.72	8.54	8.62	1	0	0	0	0	1	1	12.15	12.15	12.14	12.03	11.99	11.54	11.26
1371664_at	Pxn	0	0	0	0	0	0	7.47	7.43	7.56	7.66	7.39	1	0	0	0	0	0	1	7.44	7.51	7.60	7.54	7.59	7.82	8.46
1371665_at	Smarcd3	0	0	0	0	0	0	4.66	4.54	4.37	4.64	4.51	1	0	0	0	0	1	1	7.32	7.46	7.65	7.80	7.83	8.31	8.51
1371669_at	Dhx38	0	0	0	0	0	0	6.26	6.21	6.22	6.22	6.20	1	0	0	0	0	1	1	7.56	7.49	7.44	7.42	7.32	6.92	6.69
1371683_at	Lsm4	0	0	0	0	0	0	8.21	8.52	8.17	8.64	8.33	1	0	0	0	0	1	1	10.85	10.74	10.73	10.66	10.49	10.08	9.89
1371709_at	Mrpl3	0	0	0	0	0	0	8.10	8.03	8.13	8.07	8.00	1	0	0	0	0	0	1	9.27	9.29	9.32	9.32	9.30	8.96	8.68
1371712_at	Lsm2	0	0	0	0	0	0	6.23	6.37	6.14	6.38	6.33	1	0	0	0	0	0	1	9.57	9.51	9.57	9.58	9.47	9.10	8.68
1371729_at	Ypel5	0	0	0	0	0	0	8.56	8.54	8.67	8.68	8.40	1	0	0	0	0	0	1	9.50	9.59	9.58	9.52	9.63	10.03	10.17
1371758_at	Ufc1	0	0	0	0	0	0	10.38	10.38	10.23	10.59	10.28	1	0	0	0	0	1	1	10.49	10.46	10.39	10.42	10.30	9.72	9.24
1371771_at	Hspg2	0	0	0	0	0	0	6.85	6.83	6.53	6.94	6.59	1	0	0	0	0	0	1	6.59	6.66	6.71	6.49	6.66	6.95	7.14
1371785_at	Tnfrsf12a	0	0	0	0	0	0	5.57	5.64	5.67	5.60	5.86	1	0	0	0	0	1	0	8.60	8.65	8.73	8.84	9.02	9.30	9.10
1371793_at	Ufsp1	0	0	0	0	0	0	5.04	5.07	5.43	5.17	5.19	1	0	0	0	0	1	0	8.64	8.68	8.76	8.81	8.92	9.32	9.31
1371804_at	Cdadc1	0	0	0	0	0	0	6.71	6.51	6.73	6.58	6.36	1	0	0	0	0	0	1	8.91	8.88	8.86	8.85	8.83	8.43	7.68
1371813_at	Hirip3	0	0	0	0	0	0	5.80	5.81	6.25	5.84	5.85	1	0	0	0	0	0	1	9.39	9.38	9.46	9.47	9.42	8.87	8.34
1371822_at	Polr3d	0	0	0	0	0	0	8.11	8.14	8.17	8.06	8.30	1	0	0	0	0	0	1	9.05	9.05	9.08	9.12	9.00	8.62	8.30
1371824_at	Ak4	0	0	0	0	0	0	4.19	4.08	3.88	4.01	4.09	1	0	0	1	0	1	0	8.67	8.62	8.46	8.00	8.10	7.94	8.33
1371883_at	Mmd	0	0	0	0	0	0	5.85	5.96	6.24	5.73	6.23	1	0	0	0	0	0	1	9.60	9.62	9.72	9.79	9.85	10.17	9.98
1371898_at	Kcnk5	0	0	0	0	0	0	4.10	4.22	4.04	3.99	4.06	1	0	0	0	0	0	1	5.67	5.60	5.67	5.51	5.53	6.02	6.59
1371919_at	Nudt19	0	0	0	0	0	0	7.45	7.43	7.31	7.44	7.43	1	0	0	0	0	0	1	8.72	8.72	8.81	8.87	8.74	8.35	7.91
1371924_at	Olfml3	0	0	0	0	0	0	4.39	4.28	4.36	4.12	4.15	1	0	0	0	0	0	1	10.16	10.32	10.40	10.45	10.35	9.73	9.11
1371928_at	Cdca8	0	0	0	0	0	0	4.95	5.09	5.04	5.32	5.29	1	0	0	0	0	1	1	8.87	8.80	8.87	8.77	8.58	7.43	7.21
1371936_at	Eif4a1	0	0	0	0	0	0	10.61	10.38	10.64	10.67	10.40	1	0	0	0	0	0	1	11.99	11.97	12.03	12.03	11.94	11.50	11.01
1371950_at	Pdzd11	0	0	0	0	0	0	6.66	6.86	6.84	6.87	6.93	1	0	0	0	0	0	1	8.65	8.60	8.70	8.73	8.62	8.09	7.98
1371955_at	Mrpl35	0	0	0	0	0	0	7.67	7.73	7.37	7.90	7.46	1	0	0	0	0	0	1	9.44	9.38	9.43	9.44	9.31	9.01	8.53
1371969_at	Cald1	0	0	0	0	0	0	6.42	6.24	6.69	6.16	6.42	1	0	0	0	0	1	1	4.19	3.95	4.36	4.48	4.59	5.08	5.12
1371993_at	Cpne3	0	0	0	0	0	0	7.99	7.85	8.23	7.89	7.89	1	0	0	0	0	1	0	8.45	8.56	8.66	8.65	8.74	9.12	8.86
1371997_at	Akr1e2	0	0	0	0	0	0	8.22	8.17	8.19	8.27	8.42	1	0	0	0	0	1	1	8.71	8.68	8.73	8.71	8.84	9.33	9.17
1372027_at	Spata13	0	0	0	0	0	0	6.17	5.93	6.63	6.10	6.27	1	0	0	0	0	1	1	5.31	5.30	5.25	5.11	5.30	6.11	6.89
1372029_at	Plin3	0	0	0	0	0	0	6.63	6.34	6.19	6.66	6.30	1	0	0	0	0	0	1	3.84	3.93	3.87	3.78	3.96	4.12	4.43
1372055_at	---	0	0	0	0	0	0	5.24	5.25	5.24	5.20	5.27	1	0	0	0	0	1	1	6.96	7.09	7.13	7.19	7.30	8.21	8.41
1372059_at	Cmss1	0	0	0	0	0	0	6.65	6.73	6.81	6.82	6.38	1	0	0	0	0	1	0	8.92	9.02	9.07	9.25	9.30	9.57	9.44
1372068_at	Zmynd11	0	0	0	0	0	0	9.50	9.64	9.94	9.53	9.67	1	0	0	0	0	0	1	9.76	9.68	9.69	9.71	9.60	9.18	8.75
1372086_at	Fhdc1	0	0	0	0	0	0	6.09	6.28	6.14	6.20	6.32	1	0	0	0	0	1	0	7.74	7.59	7.54	7.29	7.24	7.02	7.03
1372088_at	Ppfibp1	0	0	0	0	0	0	7.37	7.11	7.49	7.49	7.15	1	0	0	0	0	1	1	7.06	7.06	7.20	7.34	7.33	7.81	8.08
1372107_at	Fhl1	0	0	0	0	0	0	6.89	6.65	6.75	7.17	6.49	1	0	0	0	0	1	1	7.84	7.90	8.03	8.07	8.07	8.65	8.85
1372117_at	Mtmr10	0	0	0	0	0	0	6.59	6.47	6.81	6.73	6.65	1	0	0	0	0	1	1	7.17	7.11	7.25	7.22	7.31	7.80	8.49
1372169_at	Msl1	0	0	0	0	0	0	7.98	7.93	8.35	8.04	8.03	1	0	0	0	0	0	1	9.43	9.49	9.52	9.53	9.53	9.12	8.81
1372179_at	Hpcal1	0	0	0	0	0	0	8.85	8.55	8.73	8.85	8.48	1	0	0	0	0	1	1	9.52	9.54	9.61	9.62	9.69	10.25	10.29
1372181_at	Rpa1	0	0	0	0	0	0	6.42	6.32	6.65	6.66	6.74	1	0	0	0	0	1	1	9.99	10.01	10.07	10.17	10.20	10.76	10.68
1372182_at	Pfkp	0	0	0	0	0	0	5.12	5.31	5.38	5.14	5.44	1	0	0	0	0	1	0	11.64	11.66	11.49	11.12	11.20	10.95	10.76
1372186_a_at	Top2a	0	0	0	0	0	0	5.36	5.50	5.45	5.57	5.48	1	0	0	0	0	1	1	9.23	9.21	9.24	9.25	9.18	8.09	8.09
1372187_at	Prkd3	0	0	0	0	0	0	7.29	7.02	7.83	7.34	7.25	1	0	0	0	0	0	1	7.77	7.89	7.96	8.06	8.19	8.27	8.33
1372195_at	Tnnc2	0	0	0	0	0	0	4.18	4.01	3.71	3.92	3.80	1	0	0	0	1	1	1	4.69	4.75	4.95	5.02	5.38	5.82	6.41
1372207_at	Brd8	0	0	0	0	0	0	7.80	7.54	8.14	8.09	7.88	1	0	0	0	0	1	1	9.61	9.58	9.64	9.55	9.47	8.89	8.38
1372208_at	Ppp1r1b	0	0	0	0	0	0	4.47	4.48	4.27	4.51	4.31	1	0	0	0	0	1	0	5.59	5.75	5.69	5.69	5.75	6.18	5.91
1372239_at	LOC100909729	0	0	0	0	0	0	5.67	5.54	5.52	5.84	5.33	1	0	0	0	0	1	1	6.19	6.14	6.15	5.93	5.84	5.13	4.99
1372240_at	Sgca	0	0	0	0	0	0	4.81	4.76	4.62	4.50	4.57	1	0	0	0	0	0	1	4.45	4.41	4.47	4.16	4.29	4.58	5.25
1372262_at	Smim10l1	0	0	0	0	0	0	9.48	9.50	9.59	9.68	9.45	1	0	0	0	0	0	1	10.14	10.22	10.20	10.26	10.17	9.71	8.92
1372277_at	Aldh4a1 /// Iffo2	0	0	0	0	0	0	7.12	7.16	6.89	6.97	7.00	1	0	0	0	0	1	1	6.74	6.71	6.84	6.98	6.86	7.35	7.71
1372278_at	LOC100909490	0	0	0	0	0	0	5.27	5.24	5.04	5.08	5.19	1	0	0	0	0	1	1	7.45	7.47	7.46	7.22	7.17	6.72	6.24
1372306_at	Ethe1	0	0	0	0	0	0	6.15	6.21	6.14	6.26	6.25	1	0	0	0	0	1	1	7.53	7.67	7.79	7.83	7.90	8.24	8.08
1372310_at	LOC103694869	0	0	0	0	0	0	8.14	7.92	8.30	8.16	8.16	1	0	0	0	0	0	1	9.57	9.53	9.63	9.77	9.67	10.14	10.24
1372313_at	Gpr89a /// Gpr8	0	0	0	0	0	0	7.67	7.62	7.71	7.78	7.76	1	0	0	0	0	0	1	9.93	9.85	9.95	9.87	9.85	9.56	9.06
1372320_at	Msl3	0	0	0	0	0	0	7.51	7.31	7.62	7.50	7.46	1	0	0	0	0	1	1	9.37	9.32	9.31	9.25	9.17	8.65	8.39
1372323_at	Sardh	0	0	0	0	0	0	7.54	7.53	7.36	7.83	7.48	1	0	0	0	0	1	0	8.09	7.99	8.08	8.00	7.88	7.34	7.11
1372332_at	Mef2c	0	0	0	0	0	0	5.44	5.11	5.67	5.01	5.36	1	0	0	0	0	0	1	3.88	3.96	3.97	3.90	3.89	3.96	4.76
1372345_at	---	0	0	0	0	0	0	3.11	3.43	3.25	3.34	3.48	1	0	0	0	0	0	1	5.24	5.32	5.59	5.58	5.55	5.80	6.38
1372372_at	Cmbl	0	0	0	0	0	0	9.19	9.01	9.38	9.12	9.30	1	0	0	0	0	1	1	9.97	10.01	10.08	10.32	10.41	10.59	10.44
1372373_at	Wdr13	0	0	0	0	0	0	7.24	6.94	7.49	7.23	7.02	1	0	0	0	0	1	1	10.08	10.04	10.07	9.98	9.94	9.21	8.79
1372382_at	LOC102554253	0	0	0	0	0	0	3.60	3.55	3.51	3.53	3.60	1	0	0	0	0	0	1	5.89	5.93	5.92	6.03	6.07	5.43	4.59
1372387_at	Rec114	0	0	0	0	0	0	4.39	4.39	4.44	4.52	4.57	1	0	0	0	0	1	1	5.31	5.53	5.50	5.38	5.60	6.27	6.32
1372398_at	Kctd15	0	0	0	0	0	0	5.84	5.90	6.05	5.82	6.05	1	0	0	0	0	1	1	8.32	8.28	8.30	8.26	8.07	7.25	6.70
1372401_at	Nans	0	0	0	0	0	0	8.37	8.37	8.39	8.35	8.66	1	0	0	0	0	1	0	9.49	9.55	9.60	9.67	9.71	10.08	10.07
1372402_at	Nans	0	0	0	0	0	0	7.93	7.98	7.64	7.73	7.85	1	0	0	0	0	1	0	8.41	8.53	8.61	8.68	8.66	9.02	8.80
1372417_at	Sertad1	0	0	0	0	0	0	5.59	5.51	5.46	5.38	5.27	1	0	0	0	0	0	1	7.20	7.19	7.25	7.06	7.05	6.88	6.88
1372426_at	Adamtsl4	0	0	0	0	0	0	5.00	5.17	4.95	5.01	5.17	1	0	0	0	0	1	1	5.50	5.48	5.50	5.27	5.50	6.42	7.05
1372435_at	LOC102554233	0	0	0	0	0	0	4.90	4.90	4.59	4.83	4.71	1	0	0	0	0	1	1	4.71	4.58	4.74	4.63	4.65	5.67	5.50
1372450_at	Cmc2	0	0	0	0	0	0	6.80	6.84	6.50	6.89	6.68	1	0	0	0	0	1	0	7.76	7.75	7.72	7.79	7.80	8.40	8.51
1372452_at	Gpam	0	0	0	0	0	0	8.49	8.26	8.49	8.96	8.41	1	0	0	0	0	1	0	9.31	9.32	9.47	9.55	9.51	9.90	9.86
1372457_at	Mtus1	0	0	0	0	0	0	7.59	7.37	7.92	7.25	7.21	1	0	0	0	0	1	0	8.87	8.93	9.04	9.15	9.28	9.64	8.95
1372464_at	Copg1	0	0	0	0	0	0	8.41	8.61	8.67	8.62	8.79	1	0	0	0	0	0	1	10.07	10.07	10.09	10.04	9.95	9.54	9.37
1372467_at	Hs6st1	0	0	0	0	0	0	6.12	6.12	6.18	6.40	6.12	1	0	0	0	0	1	0	8.25	8.21	8.43	8.34	8.42	8.87	8.75
1372476_at	Fads3	0	0	0	0	0	0	5.45	5.43	5.63	5.55	5.63	1	0	0	0	0	1	1	8.61	8.61	8.75	8.81	8.89	9.34	9.43
1372490_at	Gpd1l	0	0	0	0	0	0	6.82	6.84	6.80	7.03	6.81	1	0	0	0	0	0	1	6.35	6.38	6.44	6.43	6.42	6.75	7.24
1372496_at	Fdx2	0	0	0	0	0	0	7.15	7.06	7.19	7.34	7.09	1	0	0	0	0	0	1	9.93	9.88	9.91	9.98	9.81	9.44	8.86
1372503_at	Tnfsf12	0	0	0	0	0	0	5.97	6.27	6.30	5.79	6.36	1	0	0	0	0	1	1	7.60	7.61	7.63	7.49	7.59	6.82	6.43
1372516_at	Kif22	0	0	0	0	0	0	5.36	5.47	5.59	5.55	5.67	1	0	0	0	0	1	1	10.18	10.13	10.10	10.05	9.87	8.80	8.94
1372521_at	Rnd2	0	0	0	0	0	0	7.09	7.24	6.97	6.96	7.27	1	0	0	0	0	1	1	7.58	7.71	7.65	7.64	7.83	8.59	8.68
1372524_at	Lpin1	0	0	0	0	0	0	5.66	5.70	5.64	5.64	5.93	1	0	0	0	0	0	1	7.88	7.91	7.93	7.74	7.85	8.22	8.73
1372525_at	Fkbp14	0	0	0	0	0	0	4.66	4.61	4.76	4.51	4.58	1	0	0	0	0	0	1	5.15	4.99	5.18	4.99	5.03	5.64	5.65
1372530_at	Hcfc1	0	0	0	0	0	0	7.62	7.73	7.96	7.89	7.92	1	0	0	0	0	0	1	9.54	9.54	9.59	9.56	9.52	8.97	8.70
1372532_at	Pitpnm2	0	0	0	0	0	0	6.89	6.92	6.65	6.95	6.86	1	0	0	0	0	0	1	7.97	8.05	7.93	7.74	7.74	7.43	7.03
1372539_at	Parm1	0	0	0	0	0	0	6.78	6.90	6.71	6.43	7.14	1	0	0	0	0	1	1	7.89	7.93	8.14	8.32	8.33	8.79	8.81
1372546_at	Mapkapk3	0	0	0	0	0	0	6.45	6.44	6.41	6.52	6.38	1	0	0	0	0	1	1	5.19	5.19	5.23	5.11	5.27	6.04	6.51
1372596_at	Get4	0	0	0	0	0	0	7.18	7.02	7.11	7.00	6.91	1	0	0	0	0	0	1	8.79	8.89	8.86	8.83	8.72	8.42	7.88
1372600_at	Fbxo31	0	0	0	0	0	0	6.89	6.69	6.64	7.03	6.67	1	0	0	0	0	1	0	7.26	7.39	7.44	7.48	7.47	7.85	7.75
1372614_at	Focad	0	0	0	0	0	0	7.43	7.36	7.39	7.47	7.55	1	0	0	0	0	0	1	7.22	7.15	7.29	7.28	7.22	7.59	7.90
1372616_at	Trim36	0	0	0	0	0	0	3.46	3.51	3.44	3.47	3.41	1	0	0	0	0	0	1	6.16	6.11	6.26	6.28	6.17	6.37	6.48
1372624_at	Ano6	0	0	0	0	0	0	9.24	9.26	9.61	9.41	9.50	1	0	0	0	0	1	1	9.76	9.72	9.89	9.93	9.93	10.52	10.67
1372628_at	Ap4s1	0	0	0	0	0	0	6.86	7.14	7.03	7.23	7.11	1	0	0	0	0	0	1	8.16	8.26	8.22	8.27	8.12	7.64	7.29
1372631_at	Tk1	0	0	0	0	0	0	6.36	6.38	6.24	6.66	6.57	1	0	0	0	0	1	1	9.64	9.58	9.66	9.62	9.47	8.98	8.91
1372644_at	C8orf33	0	0	0	0	0	0	7.02	6.77	7.04	7.19	6.85	1	0	0	0	0	0	1	9.26	9.34	9.33	9.26	9.19	8.75	8.28
1372651_at	Slc44a1	0	0	0	0	0	0	5.50	5.53	5.94	5.50	5.76	1	0	0	0	0	1	1	6.72	6.70	6.89	6.87	6.89	7.48	7.11
1372656_at	Snx15	0	0	0	0	0	0	6.88	6.95	7.05	6.71	7.26	1	0	0	0	0	1	1	8.39	8.36	8.38	8.42	8.28	7.80	7.82
1372665_at	Psat1	0	0	0	0	0	0	9.65	9.27	9.23	9.61	9.23	1	0	0	0	0	0	1	11.17	11.11	11.25	11.40	11.44	11.18	10.41
1372682_at	Pgap4	0	0	0	0	0	0	4.74	4.65	4.72	4.64	4.47	1	0	0	0	0	1	0	7.47	7.46	7.41	7.26	7.24	6.85	7.00
1372685_at	Cdkn3	0	0	0	0	0	0	5.78	5.98	6.11	6.11	6.08	1	0	0	0	0	1	1	10.47	10.39	10.44	10.32	10.21	9.33	9.64
1372689_at	Elp6	0	0	0	0	0	0	6.61	6.59	6.62	6.98	6.48	1	0	0	0	0	0	1	7.98	7.92	7.93	8.07	7.87	7.45	6.74

Probeset ID	Gene Symbol	Islet Data Set (log2 ratio ≥0.263; FDR <20%) union n=5325 1=YES 0=NO	Islet DRlyp/lyp ND vs ND+Lp299v (log2 ratio ≥0.263; FDR<20%) n=429 1=YES 0=NO	Islet DRlyp/lyp ND vs HCD (log2 ratio ≥0.263; FDR<20%) n=2247 1=YES 0=NO	Islet DRlyp/lyp ND vs HCD+Lp299v (log2 ratio ≥0.263; FDR<20%) n=1595 1=YES 0=NO	Islet DRlyp/lyp HCD vs HCD+Lp299v (log2 ratio ≥0.263; FDR<20%) n=3026 1=YES 0=NO	Islet DRlyp/lyp ND vs Flyp/lyp ND (log2 ratio ≥ 0.263; FDR<20%) n=1950 1=YES 0=NO	Islet Mean Log2 Intensity DRlyp/lyp ND	Islet Mean Log2 Intensity DRlyp/lyp ND+Lp299v	Islet Mean Log2 Intensity DRlyp/lyp HCD	Islet Mean Log2 Intensity DRlyp/lyp HCD+Lp299v	Islet Mean Log2 Intensity Flyp40 ND	Rinm5f data union of all conditions n=3705 1=YES 0=NO	Rinm5f 30 µM propionate/ 6 µM butyrate vs Media (FDR<20%; 1.5 fold) n=3 1=YES 0=NO	Rinm5f 60 µM propionate/ 12 µM butyrate vs Media (FDR<20%; 1.5 fold) n=18 1=YES 0=NO	Rinm5f 120 µM propionate/ 24 µM butyrate vs Media (FDR<20%; 1.5 fold) n=164 1=YES 0=NO	Rinm5f 240 µM propionate /48 µM butyrate vs Media (FDR<20%; 1.5 fold) n=268 1=YES 0=NO	Rinm5f 1500 µM propionate/ 300 µM butyrate vs Media (FDR<20%; 1.5 fold) n=2491 1=YES 0=NO	Rinm5f 500 µM propionate/ 500 µM butyrate vs Media (FDR<20%; 1.5 fold) n=3001 1=YES 0=NO	Rinm5f Mean Log2 Intensity Media only	Rinm5f Mean Log2 Intensity 30 µM propionate/ 6 µM butyrate	Rinm5f Mean Log2 Intensity 60 µM propionate/ 12 µM butyrate	Rinm5f Mean Log2 Intensity 120 µM propionate/ 24 µM butyrate	Rinm5f Mean Log2 Intensity 240 µM propionate/ 48 µM butyrate	Rinm5f Mean Log2 Intensity 1500 µM propionate /300 µM butyrate	Rinm5f Mean Log2 Intensity 500 µM propionate/ 500 µM butyrate
1372692_at	Tnk2	0	0	0	0	0	0	7.67	7.85	7.59	7.80	7.63	1	0	0	0	0	0	1	9.76	9.83	9.81	9.77	9.78	10.06	10.18
1372700_at	Tp53inp1	0	0	0	0	0	0	4.48	4.22	4.02	4.38	4.46	1	0	0	0	0	0	1	4.49	4.47	4.40	4.49	4.34	4.93	5.66
1372702_at	Gm884	0	0	0	0	0	0	5.35	5.43	5.67	5.42	5.61	1	0	0	1	1	1	1	6.48	6.85	6.90	7.19	7.36	7.95	8.41
1372709_at	Bcap29	0	0	0	0	0	0	5.42	5.36	5.88	5.39	5.62	1	0	0	0	0	1	1	9.65	9.77	9.81	10.04	10.08	10.54	10.54
1372728_at	Sort1	0	0	0	0	0	0	7.43	7.38	7.43	7.58	7.49	1	0	0	0	0	1	1	7.70	7.74	7.83	7.78	7.95	8.86	8.98
1372730_at	Mrpl39	0	0	0	0	0	0	6.38	6.23	6.36	6.48	6.26	1	0	0	0	0	0	1	8.54	8.58	8.56	8.64	8.59	8.06	7.61
1372746_at	Rex1bd	0	0	0	0	0	0	6.98	7.00	6.67	7.05	6.71	1	0	0	0	0	1	1	9.14	9.16	9.11	9.06	8.97	8.35	7.84
1372758_at	---	0	0	0	0	0	0	6.86	6.71	6.88	6.40	6.71	1	0	0	0	0	1	1	4.50	4.44	4.32	4.36	4.54	5.19	5.74
1372760_at	Zc3h12a	0	0	0	0	0	0	8.37	8.32	8.56	8.46	8.37	1	0	0	0	0	0	1	9.44	9.45	9.49	9.38	9.58	9.62	9.44
1372761_at	Ppp1r16b	0	0	0	0	0	0	4.96	4.86	4.75	4.91	4.91	1	0	0	0	0	0	1	5.45	5.47	5.47	5.30	5.47	5.95	5.74
1372765_a_at	Eci2	0	0	0	0	0	0	4.87	4.64	4.97	4.65	4.67	1	0	0	0	0	1	1	6.83	6.85	7.02	7.36	7.20	7.68	7.46
1372766_at	Cpne5	0	0	0	0	0	0	5.99	6.04	5.87	5.97	5.97	1	0	0	0	0	0	1	7.17	7.09	7.03	6.86	6.97	7.37	7.91
1372773_at	Npdc1	0	0	0	0	0	0	9.25	9.27	9.32	9.46	9.17	1	0	0	0	0	0	1	11.20	11.25	11.24	11.16	11.15	10.77	10.30
1372784_at	Lrrc41	0	0	0	0	0	0	7.71	7.69	7.60	8.00	7.77	1	0	0	0	0	1	1	9.53	9.53	9.58	9.57	9.51	8.92	8.59
1372803_at	Tmem254	0	0	0	0	0	0	8.24	8.15	8.04	8.52	8.12	1	0	0	0	0	1	1	9.70	9.58	9.58	9.45	9.36	8.47	8.16
1372838_at	Luc7l	0	0	0	0	0	0	7.11	7.08	7.20	7.01	6.91	1	0	0	0	0	0	1	7.95	8.00	8.08	8.10	8.17	8.30	8.37
1372841_at	Reep6	0	0	0	0	0	0	4.42	4.34	4.28	4.34	4.28	1	0	0	0	0	1	0	8.13	8.22	8.37	8.57	8.55	8.97	8.69
1372847_at	Sdhaf3	0	0	0	0	0	0	5.97	5.61	5.81	5.86	5.92	1	0	0	0	0	1	1	8.26	8.31	8.27	8.35	8.48	9.10	8.79
1372858_at	Mrln	0	0	0	0	0	0	4.69	4.71	4.31	4.55	4.43	1	0	0	0	0	1	1	6.48	6.37	6.58	6.92	6.86	7.50	7.17
1372860_at	Lhpp	0	0	0	0	0	0	5.91	6.17	6.01	6.34	6.13	1	0	0	0	0	1	1	7.80	7.76	7.99	8.06	8.09	8.66	8.52
1372879_at	Akt1s1	0	0	0	0	0	0	7.86	7.78	7.62	8.13	7.86	1	0	0	0	0	0	1	9.18	9.17	9.18	9.19	9.10	8.68	8.37
1372886_at	Tacc3	0	0	0	0	0	0	4.38	4.47	4.55	4.75	4.68	1	0	0	0	0	1	1	10.26	10.18	10.23	10.16	9.99	8.81	8.76
1372898_at	Heatr6	0	0	0	0	0	0	4.56	4.55	4.47	4.64	4.65	1	0	0	0	0	0	1	7.45	7.49	7.50	7.48	7.38	6.90	6.65
1372903_at	Kif18b	0	0	0	0	0	0	3.82	3.87	3.96	4.11	4.13	1	0	0	0	0	1	1	8.65	8.53	8.58	8.54	8.39	7.47	7.80
1372911_at	Mthfr	0	0	0	0	0	0	7.95	8.08	7.99	8.12	7.96	1	0	0	0	0	1	1	8.51	8.43	8.42	8.40	8.30	7.85	7.71
1372914_at	Ltbr	0	0	0	0	0	0	8.01	7.86	7.96	8.21	7.85	1	0	0	0	0	0	1	9.09	9.12	9.11	9.06	8.98	8.56	8.14
1372918_at	Smim11	0	0	0	0	0	0	7.19	7.16	7.43	7.34	7.33	1	0	0	0	0	0	1	9.86	9.85	9.82	9.75	9.76	9.34	8.88
1372946_at	Eola1 /// Eola2	0	0	0	0	0	0	6.56	6.35	6.22	6.68	6.37	1	0	0	0	0	1	0	6.83	6.97	6.92	6.92	7.03	7.46	7.53
1372950_at	Bet1l	0	0	0	0	0	0	7.19	7.10	7.04	7.06	7.06	1	0	0	0	0	0	1	8.55	8.55	8.49	8.43	8.36	8.03	7.59
1372952_at	Ctu2	0	0	0	0	0	0	5.38	5.21	5.23	5.46	4.99	1	0	0	0	0	0	1	5.66	5.61	5.75	5.80	5.66	5.20	4.91
1372955_at	Stk38l	0	0	0	0	0	0	6.47	6.24	6.72	6.23	6.48	1	0	0	0	0	1	0	5.92	6.01	6.00	6.10	6.20	6.55	6.34
1372957_at	Hjurp	0	0	0	0	0	0	5.92	5.93	5.68	6.05	6.02	1	0	0	0	0	1	1	7.75	7.62	7.69	7.51	7.33	6.54	6.31
1372960_a_at	Xylt2	0	0	0	0	0	0	6.90	7.11	7.00	7.07	6.84	1	0	0	0	0	1	1	7.51	7.51	7.59	7.56	7.62	8.16	8.30
1372980_at	Tspan33	0	0	0	0	0	0	7.35	7.55	7.51	7.50	7.40	1	0	0	0	0	1	1	6.40	6.42	6.58	6.66	6.77	7.28	7.41
1372986_at	Jdp2	0	0	0	0	0	0	5.39	5.21	5.20	5.12	5.22	1	0	0	0	0	1	1	8.24	8.19	8.20	8.17	8.18	7.31	6.44
1372990_at	Creb3	0	0	0	0	0	0	7.07	7.11	7.10	6.87	7.15	1	0	0	0	0	0	1	9.40	9.38	9.40	9.45	9.39	9.05	8.52
1372993_at	---	0	0	0	0	0	0	6.72	6.82	6.64	7.16	6.40	1	0	0	0	0	0	1	7.47	7.56	7.70	7.51	7.60	7.86	8.02
1372995_at	Prkd2	0	0	0	0	0	0	6.55	6.54	6.50	6.58	6.67	1	0	0	0	0	1	1	7.21	7.34	7.43	7.43	7.58	7.95	7.71
1373009_at	Czib	0	0	0	0	0	0	6.78	7.02	6.66	7.07	6.70	1	0	0	0	0	1	1	8.58	8.57	8.57	8.41	8.27	7.80	7.43
1373019_at	Eps15	0	0	0	0	0	0	7.11	6.83	7.58	6.91	7.39	1	0	0	0	0	0	1	8.95	8.98	9.06	9.10	9.21	9.52	9.82
1373023_at	Tmem30a	0	0	0	0	0	0	6.39	6.35	6.72	6.14	6.61	1	0	0	0	0	1	0	6.31	6.41	6.55	6.63	6.71	7.08	6.93
1373026_at	Spc24	0	0	0	0	0	0	5.32	5.20	5.30	5.57	4.95	1	0	0	0	0	1	1	9.43	9.31	9.31	9.18	9.11	8.52	8.32
1373031_at	Trim8	0	0	0	0	0	0	7.39	7.27	7.23	7.34	7.16	1	0	0	0	0	0	1	8.71	8.60	8.72	8.66	8.65	8.18	7.98
1373035_at	---	0	0	0	0	0	0	6.28	6.05	6.17	6.31	6.35	1	0	0	0	0	0	1	11.30	11.32	11.37	11.31	11.42	10.93	9.33
1373037_at	Ube2l6	0	0	0	0	0	0	6.19	6.61	6.36	6.11	6.57	1	0	0	0	0	0	1	9.93	9.96	9.93	10.03	9.96	9.36	8.64
1373047_at	Prkci	0	0	0	0	0	0	8.24	8.20	8.45	8.20	8.39	1	0	0	0	0	0	1	9.43	9.38	9.49	9.57	9.60	9.95	10.00
1373050_at	Tbc1d1	0	0	0	0	0	0	6.84	6.79	6.88	6.74	6.81	1	0	0	0	0	1	1	8.61	8.64	8.73	8.83	8.92	9.66	10.00
1373053_at	---	0	0	0	0	0	0	8.76	8.47	8.63	9.09	8.47	1	0	0	0	0	1	1	7.50	7.72	7.94	7.89	8.04	8.66	8.69
1373066_at	Cyria	0	0	0	0	0	0	3.01	2.95	3.19	2.94	3.08	1	0	0	0	0	1	1	5.76	5.74	5.78	5.65	5.67	6.64	7.06
1373072_at	Lims1	0	0	0	0	0	0	7.64	7.53	7.93	7.50	7.77	1	0	0	0	0	0	1	8.86	8.92	8.93	9.05	9.09	9.37	9.31
1373074_at	Timm21	0	0	0	0	0	0	5.93	5.94	6.39	6.19	6.18	1	0	0	0	0	1	1	8.81	8.80	8.79	8.87	8.78	8.21	7.64
1373079_at	Smim14	0	0	0	0	0	0	9.64	9.55	9.83	9.47	9.98	1	0	0	0	0	1	0	8.65	8.78	8.80	8.90	9.04	9.24	8.93
1373085_at	Cbr3	0	0	0	0	0	0	3.98	3.99	4.10	3.88	3.89	1	0	0	0	0	1	0	7.61	7.65	7.72	7.93	8.00	8.29	7.94
1373092_at	Tgfbr3	0	0	0	0	0	0	6.07	5.80	6.02	5.75	6.15	1	0	0	0	0	0	1	5.48	5.41	5.52	5.05	5.20	5.80	6.68
1373101_at	Pigk	0	0	0	0	0	0	8.96	8.96	9.26	9.35	8.87	1	0	0	0	0	0	1	10.75	10.78	10.75	10.80	10.79	10.40	9.76
1373108_at	Ppp1r3c	0	0	0	0	0	0	3.68	3.91	3.55	3.71	3.63	1	0	0	1	1	0	1	8.16	8.04	7.80	7.47	7.49	8.09	8.95
1373121_at	Mrps28	0	0	0	0	0	0	7.10	7.15	7.04	7.36	7.06	1	0	0	0	0	1	0	9.16	9.11	9.11	9.04	8.96	8.54	8.44
1373132_at	---	0	0	0	0	0	0	6.79	6.85	6.89	6.79	6.87	1	0	0	0	0	0	1	9.11	9.10	9.23	9.17	9.16	8.77	8.39
1373153_at	---	0	0	0	0	0	0	4.35	4.44	4.53	4.31	4.75	1	0	0	0	0	1	0	5.98	6.04	6.14	6.08	5.91	5.30	5.13
1373161_at	Tmem98	0	0	0	0	0	0	8.88	9.08	9.23	8.91	9.02	1	0	0	0	0	1	1	9.98	10.14	10.23	10.42	10.51	11.12	10.61
1373167_at	Lrrc20	0	0	0	0	0	0	6.28	6.35	6.03	6.55	6.16	1	0	0	0	0	1	1	6.97	7.08	7.09	7.06	7.15	7.60	7.81
1373177_x_at	LOC100909409	0	0	0	0	0	0	3.40	3.16	3.54	3.16	3.67	1	0	0	0	1	1	0	7.42	7.60	7.62	7.80	8.08	8.36	7.52
1373179_at	Btbd8	0	0	0	0	0	0	6.21	5.99	6.05	6.04	5.99	1	0	0	0	0	1	1	8.71	8.72	8.57	8.13	8.23	7.82	7.77
1373202_at	Gng3	0	0	0	0	0	0	4.28	4.40	4.04	4.31	4.19	1	0	0	1	0	0	0	10.97	11.05	11.27	11.56	11.48	11.01	9.76
1373205_at	U2af1l4	0	0	0	0	0	0	6.80	6.87	7.11	7.00	7.05	1	0	0	0	0	1	1	9.45	9.36	9.31	9.27	9.08	8.72	8.29
1373207_at	Cers6	0	0	0	0	0	0	7.91	7.79	8.22	8.11	8.15	1	0	0	0	0	1	1	8.09	8.04	8.11	8.21	8.21	8.68	9.22
1373211_at	Fbxw4	0	0	0	0	0	0	7.57	7.42	7.38	7.67	7.32	1	0	0	0	0	1	1	8.70	8.69	8.78	8.65	8.60	8.04	7.70
1373216_at	C15orf65	0	0	0	0	0	0	5.81	5.82	6.13	5.88	5.86	1	0	0	0	0	1	0	7.05	7.12	7.17	7.21	7.19	7.65	7.46
1373218_at	Fam81a	0	0	0	0	0	0	3.47	3.35	3.09	3.31	3.27	1	0	0	0	0	1	1	5.97	6.03	6.07	6.01	6.13	6.64	7.08
1373220_at	---	0	0	0	0	0	0	6.85	6.98	6.76	7.16	7.01	1	0	0	0	0	1	0	6.64	6.59	6.87	7.01	7.17	7.31	6.46
1373233_at	Lhfpl2	0	0	0	0	0	0	5.39	5.34	5.25	5.71	5.12	1	0	0	0	0	0	1	6.31	6.37	6.46	6.40	6.34	6.71	7.37
1373250_at	Anln /// Anlnl1	0	0	0	0	0	0	5.74	5.44	5.99	5.67	5.95	1	0	0	0	0	1	1	10.60	10.55	10.52	10.32	10.32	9.47	9.81
1373252_at	Fmnl3	0	0	0	0	0	0	6.10	6.16	5.96	5.96	6.16	1	0	0	0	0	0	1	7.37	7.28	7.35	7.22	7.38	7.81	8.30
1373254_at	Krt10	0	0	0	0	0	0	5.12	5.12	5.25	5.29	5.02	1	0	0	0	0	1	1	7.89	7.80	7.78	7.87	7.71	7.01	6.93
1373256_at	Chd3	0	0	0	0	0	0	5.91	6.02	5.98	6.10	6.02	1	0	0	0	0	1	1	6.73	6.72	6.74	6.64	6.50	5.84	5.34
1373260_at	Caprin2	0	0	0	0	0	0	6.09	5.92	6.60	6.04	6.15	1	0	0	0	0	1	1	5.88	5.98	6.15	6.18	6.20	6.62	6.99
1373267_at	Sh3yl1	0	0	0	0	0	0	5.35	5.20	5.56	5.80	5.23	1	0	0	0	0	0	1	4.92	4.76	4.93	4.89	4.93	5.20	5.10
1373270_at	Wipi1	0	0	0	0	0	0	6.95	6.89	7.13	7.35	6.58	1	0	0	0	0	1	1	8.06	8.15	8.07	7.95	8.13	8.84	8.83
1373274_at	Txnrd3	0	0	0	0	0	0	5.85	5.76	5.81	5.86	5.72	1	0	0	0	0	1	1	7.04	7.13	7.14	6.99	7.06	7.74	7.90
1373283_at	Tmpo	0	0	0	0	0	0	6.49	6.49	6.92	6.58	6.63	1	0	0	0	0	0	1	10.30	10.25	10.29	10.29	10.22	9.80	9.53
1373298_at	Ubash3b	0	0	0	0	0	0	5.25	5.24	5.56	5.27	5.27	1	0	0	0	0	1	1	6.35	6.39	6.51	6.65	6.67	7.01	7.32
1373302_at	Acer2	0	0	0	0	0	0	4.66	4.38	4.67	4.32	4.38	1	0	0	0	0	0	1	4.22	4.14	4.28	4.29	4.31	4.76	5.04
1373309_at	Tmem86a	0	0	0	0	0	0	5.82	5.90	6.11	6.02	5.66	1	0	0	0	0	1	1	6.90	7.05	7.06	7.14	7.23	7.77	7.84
1373321_at	Myrf	0	0	0	0	0	0	8.15	7.93	7.86	8.35	7.78	1	0	0	0	0	1	1	7.28	7.34	7.52	7.64	7.70	8.17	8.59
1373324_at	Dusp14	0	0	0	0	0	0	4.96	5.13	5.00	4.90	5.13	1	0	0	0	0	1	1	7.58	7.60	7.67	7.73	7.79	9.18	9.59
1373330_at	Myo15b	0	0	0	0	0	0	7.25	7.49	7.32	7.22	7.34	1	0	0	0	0	1	0	7.58	7.47	7.61	7.61	7.50	6.94	7.07
1373338_at	Vmac	0	0	0	0	0	0	8.08	8.07	8.09	8.33	7.86	1	0	0	0	0	0	1	8.02	8.03	8.02	8.04	8.14	8.57	8.79
1373343_at	---	0	0	0	0	0	0	6.17	5.96	6.11	6.27	6.21	1	0	0	0	0	1	1	6.92	6.85	6.97	6.68	6.69	6.04	5.66
1373348_at	Dtx1	0	0	0	0	0	0	5.38	5.23	5.09	5.37	5.29	1	0	0	0	0	1	1	6.60	6.58	6.58	6.49	6.67	7.38	7.98
1373360_at	---	0	0	0	0	0	0	4.70	4.67	4.43	4.92	4.60	1	0	0	0	0	1	0	6.23	6.06	6.00	5.72	5.74	5.62	4.96
1373371_a_at	Fmc1	0	0	0	0	0	0	8.01	7.92	7.85	8.03	7.94	1	0	0	0	0	1	1	9.98	9.91	9.86	9.76	9.68	9.29	9.05
1373379_at	Irak1	0	0	0	0	0	0	10.19	10.14	10.07	10.56	10.21	1	0	0	0	0	1	1	10.74	10.71	10.80	10.80	10.75	9.84	9.24
1373410_at	Mef2c	0	0	0	0	0	0	5.50	5.22	5.37	5.35	5.59	1	0	0	0	0	0	1	4.11	4.07	4.27	4.35	4.33	4.38	5.18
1373414_at	Tmem59l	0	0	0	0	0	0	6.65	6.55	6.55	6.23	6.83	1	0	0	1	1	1	1	6.41	6.90	6.93	7.07	7.26	8.13	8.36
1373415_at	Heg1	0	0	0	0	0	0	7.61	7.49	7.79	7.19	7.50	1	0	0	0	0	1	0	8.37	8.42	8.43	8.43	8.53	9.01	9.40
1373430_at	Baz2b	0	0	0	0	0	0	7.40	7.37	7.87	7.24	7.56	1	0	0	0	0	0	1	8.28	8.40	8.35	8.31	8.42	7.88	7.20
1373440_at	Lyrm2	0	0	0	0	0	0	7.03	7.11	7.19	7.48	6.97	1	0	0	0	0	0	1	9.08	9.10	9.12	9.13	9.05	8.58	7.99
1373451_at	Rnaseh2b	0	0	0	0	0	0	5.21	5.16	5.29	5.52	5.28	1	0	0	0	0	1	1	7.00	6.90	6.96	6.86	6.60	6.20	5.63
1373465_at	Slc66a2	0	0	0	0	0	0	7.86	7.77	8.04	7.96	7.91	1	0	0	0	0	0	1	9.33	9.42	9.39	9.33	9.31	8.85	8.27
1373475_at	Ccdc58	0	0	0	0	0	0	6.90	6.75	6.98	7.02	6.79	1	0	0	0	0	1	0	9.77	9.77	9.69	9.62	9.49	9.12	8.76
1373478_at	---	0	0	0	0	0	0	4.85	4.92	4.57	4.98	4.71	1	0	0	0	0	1	1	6.45	6.44	6.58	6.74	6.91	7.26	7.84
1373484_at	Klhl13	0	0	0	0	0	0	6.42	6.52	6.75	6.42	6.69	1	0	0	0	0	1	1	7.11	7.10	7.21	7.26	7.28	7.85	7.77
1373485_at	Rnf122	0	0	0	0	0	0	3.51	3.65	3.48	3.81	3.60	1	0	0	0	0	0	1	4.00	3.84	3.87	3.75	3.83	4.17	4.75
1373501_at	Nek7	0	0	0	0	0	0	7.44	7.37	7.82	7.49	7.34	1	0	0	0	0	1	1	7.32	7.29	7.58	7.84	7.90	8.58	8.84
1373543_at	Znf729	0	0	0	0	0	0	6.14	6.39	6.40	6.24	6.48	1	0	0	0	0	0	1	7.53	7.71	7.65	7.55	7.61	7.22	6.91
1373556_at	Ptrhd1	0	0	0	0	0	0	6.01	5.96	5.80	6.17	5.85	1	0	0	0	0	1	1	8.86	8.78	8.88	8.78	8.61	7.82	7.44
1373578_at	Trim2	0	0	0	0	0	0	6.45	6.18	6.28	6.10	6.21	1	0	0	0	0	1	1	8.16	8.25	8.42	8.56	8.66	9.03	8.94
1373588_at	Frmd8	0	0	0	0	0	0	5.95	5.93	5.89	5.87	5.95	1	0	0	0	0	1	1	9.24	9.30	9.35	9.45	9.49	10.04	10.34
1373590_at	Stom	0	0	0	0	0	0	5.57	5.61	5.36	5.51	5.43	1	0	0	0	0	1	1	6.51	6.39	6.50	6.41	6.43	7.43	8.09
1373596_at	Vstm4	0	0	0	0	0	0	4.11	3.99	3.98	3.88	3.93	1	0	0	1	1	1	1	5.97	6.05	6.36	6.58	6.61	7.24	7.47
1373608_at	---	0	0	0	0	0	0	5.35	5.16	5.43	5.39	5.36	1	0	0	0	0	1	1	6.38	6.39	6.47	6.31	6.32	5.67	5.15
1373617_at	Emp2	0	0	0	0	0	0	4.80	4.96	4.93	4.79	5.10	1	0	0	0	0	1	1	4.81	4.93	5.10	5.05	5.12	6.64	6.38
1373623_at	Itpkc	0	0	0	0	0	0	6.75	6.86	6.50	7.01	6.70	1	0	0	0	0	0	1	7.90	7.91	7.81	7.70	7.65	7.46	6.81
1373627_at	---	0	0	0	0	0	0	6.15	6.15	6.09	6.56	6.07	1	0	0	0	0	0	1	7.43	7.45	7.50	7.43	7.50	7.95	7.88
1373661_a_at	Cxcr4	0	0	0	0	0	0	5.40	5.24	5.73	5.21	5.70	1	0	0	0	0	1	1	6.34	6.42	6.50	6.56	6.53	7.36	7.39
1373670_at	Stat2	0	0	0	0	0	0	6.22	6.53	6.01	6.13	6.35	1	0	0	0	0	1	0	7.30	7.36	7.39	7.43	7.57	7.90	7.64
1373672_at	Serpinb9	0	0	0	0	0	0	6.44	6.35	6.64	6.42	6.44	1	0	0	0	0	1	0	6.00	6.17	6.24	6.54	6.44	6.68	6.54
1373699_at	---	0	0	0	0	0	0	4.03	3.91	4.08	4.49	4.00	1	0	0	0	0	0	1	4.69	4.46	4.50	4.62	4.82	5.12	4.70
1373705_at	Mrpl28	0	0	0	0	0	0	8.49	8.51	8.28	8.77	8.17	1	0	0	0	0	1	1	9.32	9.36	9.38	9.51	9.53	9.93	9.95
1373722_at	Kif20a	0	0	0	0	0	0	5.94	5.79	6.45	6.18	6.29	1	0	0	0	0	1	1	9.95	9.90	9.88	9.82	9.58	8.54	8.50
1373741_at	Pus1	0	0	0	0	0	0	7.07	7.04	6.89	7.05	6.88	1	0	0	0	0	0	1	8.68	8.63	8.60	8.59	8.54	8.13	7.73
1373745_at	Gtf2h4	0	0	0	0	0	0	5.73	5.82	5.74	5.98	5.71	1	0	0	0	0	0	1	9.18	9.21	9.27	9.27	9.20	8.83	8.35
1373750_at	P3h3	0	0	0	0	0	0	6.10	6.17	6.34	6.15	6.04	1	0	0	0	0	1	0	6.91	6.88	6.92	6.89	6.81	6.07	5.40
1373751_at	Kif21b	0	0	0	0	0	0	4.43	4.46	4.26	4.44	4.54	1	0	0	0	1	1	1	7.52	7.86	7.88	7.93	8.16	8.42	8.32
1373761_at	Sinhcaf	0	0	0	0	0	0	5.31	5.30	5.77	5.42	5.52	1	0	0	0	0	0	1	6.40	6.33	6.41	6.45	6.38	6.96	7.09
1373773_at	Gpm6a	0	0	0	0	0	0	3.78	3.52	4.10	3.63	3.73	1	0	0	0	0	0	1	3.41	3.23	3.51	3.42	3.44	3.87	4.57
1373797_at	Naa80	0	0	0	0	0	0	7.01	6.99	6.74	7.02	6.80	1	0	0	0	0	0	1	6.96	7.03	7.04	7.12	7.19	7.47	7.61
1373808_at	Tead1	0	0	0	0	0	0	7.50	7.17	7.73	7.75	7.60	1	0	0	0	0	1	1	8.00	7.99	8.14	8.16	8.14	8.65	9.48
1373829_at	Fgfr2	0	0	0	0	0	0	5.12	4.94	5.11	5.34	5.13	1	0	0	0	0	1	1	7.05	7.11	7.22	7.12	7.24	7.88	8.28
1373843_at	Pakap	0	0	0	0	0	0	5.56	5.61	5.68	5.31	5.63	1	0	0	0	0	0	1	3.77	3.78	4.08	3.90	3.86	4.29	4.68
1373891_at	Zcchc12	0	0	0	0	0	0	7.63	7.81	7.99	7.66	7.86	1	0	0	0	0	1	1	8.71	8.75	8.88	9.14	9.19	10.14	10.15
1373896_at	Syt1	0	0	0	0	0	0	5.40	5.60	5.58	4.92	5.60	1	0	0	0	0	1	1	6.68	6.51	6.55	6.43	6.46	7.58	7.94
1373931_at	Pianp	0	0	0	0	0	0	4.64	4.82	4.60	4.66	4.54	1	0	0	0	0	1	1	7.93	7.93	8.03	7.93	7.79	7.27	6.46
1373934_at	Rassf3	0	0	0	0	0	0	8.06	8.05	8.41	8.31	8.26	1	0	0	0	0	1	1	8.58	8.59	8.73	8.76	8.88	9.59	9.51
1373937_at	Fyco1	0	0	0	0	0	0	7.81	7.88	8.01	8.00	7.57	1	0	0	0	0	1	1	5.45	5.54	5.75	5.81	5.88	6.69	7.37

Probeset ID	Gene Symbol	Islet Data Set (log2 ratio ≥0.263; FDR <20%) union n=5325 1=YES 0=NO	Islet DRlyp/lyp ND vs ND+Lp299v (log2 ratio ≥0.263; FDR<20%) n=429 1=YES 0=NO	Islet DRlyp/lyp ND vs HCD (log2 ratio ≥0.263; FDR<20%) n=2247 1=YES 0=NO	Islet DRlyp/lyp ND vs HCD+Lp299v (log2 ratio ≥0.263; FDR<20%) n=1595 1=YES 0=NO	Islet DRlyp/lyp HCD vs HCD+Lp299v (log2 ratio ≥0.263; FDR<20%) n=3026 1=YES 0=NO	Islet DRlyp/lyp ND vs Flyp/lyp ND (log2 ratio ≥ 0.263; FDR<20%) n=1950 1=YES 0=NO	Islet Mean Log2 Intensity DRlyp/lyp ND	Islet Mean Log2 Intensity DRlyp/lyp ND+Lp299v	Islet Mean Log2 Intensity DRlyp/lyp HCD	Islet Mean Log2 Intensity DRlyp/lyp HCD+Lp299v	Islet Mean Log2 Intensity Flyp40 ND	Rinm5f data union of all conditions n=3705 1=YES 0=NO	Rinm5f 30 µM propionate/ 6 µM butyrate vs Media (FDR<20%; 1.5 fold) n=3 1=YES 0=NO	Rinm5f 60 µM propionate/ 12 µM butyrate vs Media (FDR<20%; 1.5 fold) n=18 1=YES 0=NO	Rinm5f 120 µM propionate/ 24 µM butyrate vs Media (FDR<20%; 1.5 fold) n=164 1=YES 0=NO	Rinm5f 240 µM propionate /48 µM butyrate vs Media (FDR<20%; 1.5 fold) n=268 1=YES 0=NO	Rinm5f 1500 µM propionate/ 300 µM butyrate vs Media (FDR<20%; 1.5 fold) n=2491 1=YES 0=NO	Rinm5f 500 µM propionate/ 500 µM butyrate vs Media (FDR<20%; 1.5 fold) n=3001 1=YES 0=NO	Rinm5f Mean Log2 Intensity Media only	Rinm5f Mean Log2 Intensity 30 µM propionate/ 6 µM butyrate	Rinm5f Mean Log2 Intensity 60 µM propionate/ 12 µM butyrate	Rinm5f Mean Log2 Intensity 120 µM propionate/ 24 µM butyrate	Rinm5f Mean Log2 Intensity 240 µM propionate/ 48 µM butyrate	Rinm5f Mean Log2 Intensity 1500 µM propionate /300 µM butyrate	Rinm5f Mean Log2 Intensity 500 µM propionate/ 500 µM butyrate
1373945_at	Celf5	0	0	0	0	0	0	4.88	5.19	4.93	4.87	5.01	1	0	0	0	0	1	0	8.44	8.60	8.74	8.83	8.90	9.07	8.94
1373973_at	Sap30bp	0	0	0	0	0	0	4.91	5.01	4.59	4.91	4.71	1	0	0	0	0	0	1	6.58	6.54	6.50	6.38	6.36	6.07	5.86
1373975_at	Inmt	0	0	0	0	0	0	5.57	5.42	5.34	5.22	5.81	1	0	0	1	1	0	0	7.65	7.60	8.00	8.51	8.25	7.99	7.54
1373985_at	---	0	0	0	0	0	0	8.48	8.66	8.60	8.47	8.77	1	0	0	0	0	1	0	8.68	8.74	8.81	8.96	8.97	9.28	9.35
1373997_at	---	0	0	0	0	0	0	6.81	6.68	6.56	6.69	6.41	1	0	0	0	0	1	1	8.10	8.13	8.13	8.09	8.23	8.85	9.33
1374001_at	Dcaf17	0	0	0	0	0	0	6.86	6.63	7.02	6.66	6.86	1	0	0	0	0	1	1	8.80	9.00	8.83	8.57	8.69	8.11	7.90
1374014_at	Nsmaf	0	0	0	0	0	0	7.51	7.47	7.87	7.44	7.88	1	0	0	0	0	1	1	8.58	8.57	8.62	8.61	8.45	7.91	7.42
1374034_at	Cars1	0	0	0	0	0	0	7.99	7.87	7.83	8.06	7.71	1	0	0	0	0	0	1	9.26	9.20	9.35	9.41	9.36	8.96	8.44
1374046_at	Hs3st2	0	0	0	0	0	0	3.54	3.62	3.59	3.93	3.69	1	0	0	0	0	1	1	7.82	7.92	7.97	7.94	8.04	8.84	8.95
1374048_at	Nrtn	0	0	0	0	0	0	7.90	8.23	8.12	8.07	7.64	1	0	0	0	0	1	1	7.92	7.98	8.02	8.09	8.27	9.00	8.81
1374060_at	---	0	0	0	0	0	0	5.87	5.51	5.79	5.88	5.73	1	0	0	0	0	0	1	6.82	6.85	6.88	6.91	6.83	6.26	6.15
1374084_at	---	0	0	0	0	0	0	3.32	3.32	3.25	3.41	3.25	1	0	0	0	0	0	1	4.35	4.25	4.20	4.26	4.44	4.72	4.84
1374087_at	---	0	0	0	0	0	0	5.65	5.25	5.33	5.45	5.26	1	0	0	0	0	1	0	7.57	7.65	7.79	7.88	7.94	8.27	8.43
1374143_at	Epha2	0	0	0	0	0	0	4.71	4.67	4.68	4.73	4.55	1	0	0	0	0	0	1	5.76	5.72	5.79	5.61	5.64	6.23	6.44
1374144_at	---	0	0	0	0	0	0	7.79	7.81	8.13	7.65	7.92	1	0	0	0	0	1	1	8.78	8.79	8.95	9.06	9.08	9.66	9.82
1374149_at	Kremen1	0	0	0	0	0	0	5.34	5.54	5.80	5.36	5.60	1	0	0	0	0	0	1	6.44	6.48	6.54	6.36	6.51	6.87	6.95
1374155_at	LOC688452	0	0	0	0	0	0	3.96	3.90	4.31	3.88	4.28	1	0	0	0	0	0	1	6.28	6.22	6.28	6.32	6.30	5.84	4.95
1374156_at	Mpp5	0	0	0	0	0	0	6.70	6.74	7.12	6.72	6.91	1	0	0	0	0	1	1	7.43	7.57	7.70	7.91	7.99	8.63	8.64
1374172_at	Col8a2	0	0	0	0	0	0	3.50	3.43	3.50	3.41	3.37	1	0	0	0	0	0	1	3.64	3.70	3.64	3.69	3.76	4.10	5.70
1374176_at	Lurap1l	0	0	0	0	0	0	8.45	8.19	8.66	8.70	8.27	1	0	0	0	0	1	1	8.04	7.98	8.00	7.92	7.87	6.93	6.36
1374177_at	Taf13	0	0	0	0	0	0	8.05	7.98	8.05	8.07	8.20	1	0	0	0	0	1	1	9.87	9.93	9.99	10.27	10.33	10.80	10.81
1374181_at	Rbmx	0	0	0	0	0	0	6.46	6.31	6.64	6.58	6.56	1	0	0	0	0	0	1	8.72	8.75	8.86	8.86	8.78	8.32	8.02
1374190_at	Clybl	0	0	0	0	0	0	4.19	4.30	4.43	4.45	4.33	1	0	0	0	0	0	1	5.35	5.28	5.38	5.33	5.44	5.86	6.22
1374213_at	Arfgef2	0	0	0	0	0	0	5.15	5.35	5.23	5.39	5.29	1	0	0	0	0	1	0	7.55	7.40	7.54	7.44	7.36	6.92	7.17
1374216_at	Gas2l3	0	0	0	0	0	0	5.72	5.46	5.43	5.53	5.68	1	0	0	0	0	1	1	8.32	8.30	8.31	8.17	8.07	7.29	7.53
1374220_at	Ccny	0	0	0	0	0	0	6.95	6.97	6.87	7.14	6.71	1	0	0	0	0	1	1	9.01	8.95	8.84	8.46	8.70	7.99	7.86
1374222_at	Slc22a18	0	0	0	0	0	0	5.34	5.23	4.87	5.26	5.28	1	0	0	0	0	0	1	5.26	5.00	4.84	4.98	5.11	5.60	6.03
1374225_at	Cep128	0	0	0	0	0	0	5.54	5.41	5.46	5.42	5.62	1	0	0	0	0	1	1	7.93	7.79	7.96	7.83	7.78	7.06	7.36
1374241_at	---	0	0	0	0	0	0	6.85	6.75	7.43	6.84	6.85	1	0	0	0	0	0	1	6.72	6.76	6.71	6.54	6.62	7.26	6.97
1374249_at	Syne4	0	0	0	0	0	0	7.82	7.61	7.41	7.82	7.58	1	0	0	0	0	0	1	10.00	10.04	10.08	10.04	9.97	9.81	9.01
1374275_at	Ca11	0	0	0	0	0	0	6.11	6.25	5.98	6.05	6.01	1	0	0	0	0	1	1	8.61	8.71	8.72	8.75	8.71	8.00	6.70
1374284_at	Rassf4	0	0	0	0	0	0	4.05	4.14	3.78	4.01	3.93	1	0	0	0	1	1	1	6.88	7.10	7.14	7.32	7.48	8.23	8.12
1374294_at	Tmem265	0	0	0	0	0	0	6.57	6.53	6.41	6.82	6.49	1	0	0	0	0	1	1	7.67	7.70	7.73	7.72	7.66	7.08	6.63
1374298_at	Strip2	0	0	0	0	0	0	3.82	3.75	4.11	3.78	3.90	1	0	0	0	0	1	1	6.34	6.26	6.44	6.44	6.47	7.12	7.15
1374310_at	Ppm1j	0	0	0	0	0	0	5.12	5.32	4.83	4.89	5.02	1	0	0	0	0	1	1	7.68	7.81	7.77	7.42	7.70	8.28	8.23
1374340_at	Glrx2	0	0	0	0	0	0	7.19	6.96	7.53	7.06	7.09	1	0	0	0	0	1	0	8.58	8.78	8.84	8.96	8.99	9.20	8.84
1374344_at	Plppr3	0	0	0	0	0	0	6.14	6.16	5.73	6.18	5.90	1	0	0	0	0	1	1	7.77	7.98	8.20	8.15	8.28	8.84	9.10
1374350_at	---	0	0	0	0	0	0	6.89	6.64	7.15	6.80	6.98	1	0	0	0	0	1	1	8.46	8.53	8.61	8.72	8.65	9.27	9.44
1374354_at	Phf19	0	0	0	0	0	0	5.01	5.07	5.08	5.26	5.02	1	0	0	0	0	1	0	7.89	7.85	7.84	7.70	7.67	7.26	7.04
1374360_at	Cdc20	0	0	0	0	0	0	3.24	3.27	3.12	3.32	3.28	1	0	0	0	0	1	1	7.62	7.52	7.60	7.52	7.44	5.84	5.58
1374420_at	Apmap	0	0	0	0	0	0	7.29	7.15	7.52	7.16	7.18	1	0	0	0	0	0	1	8.90	8.96	9.03	9.11	9.04	8.75	8.11
1374432_at	---	0	0	0	0	0	0	8.33	8.15	8.55	8.59	8.45	1	0	0	0	0	1	1	8.79	8.71	8.74	8.73	8.81	9.93	10.22
1374449_at	Cdca3	0	0	0	0	0	0	6.07	5.95	6.42	6.50	6.11	1	0	0	0	0	1	1	9.37	9.34	9.34	9.33	9.16	8.36	8.29
1374458_at	Sgk3 /// C8orf44	0	0	0	0	0	0	6.33	6.11	6.69	6.38	6.18	1	0	0	0	0	0	1	6.81	6.85	7.05	7.27	7.03	7.22	7.53
1374465_at	Uxt	0	0	0	0	0	0	6.94	6.92	7.09	7.16	7.19	1	0	0	0	0	1	1	9.37	9.38	9.32	9.25	9.13	8.63	8.49
1374484_at	Tmem39a	0	0	0	0	0	0	7.47	7.50	7.73	7.34	7.53	1	0	0	0	0	1	1	9.10	9.11	9.08	8.96	9.00	8.37	7.99
1374509_at	---	0	0	0	0	0	0	6.58	6.56	6.48	6.83	6.29	1	0	0	0	0	1	1	8.20	8.14	8.34	8.44	8.54	8.81	8.83
1374530_at	---	0	0	0	0	0	0	3.29	3.33	3.19	3.22	3.30	1	0	0	0	0	0	1	4.76	4.51	4.54	4.39	4.48	4.78	5.59
1374539_at	Atp10d	0	0	0	0	0	0	5.74	5.76	5.78	5.67	5.82	1	0	0	0	0	1	1	6.13	5.92	6.09	6.28	6.28	6.87	7.91
1374546_at	Pcdh17	0	0	0	0	0	0	4.70	4.60	4.78	4.70	4.77	1	0	0	0	0	1	1	6.00	6.06	6.18	6.31	6.42	7.69	7.39
1374552_at	Mapk1ip1l	0	0	0	0	0	0	6.50	6.46	6.69	6.57	6.55	1	0	0	0	0	1	0	7.83	7.90	7.98	7.99	8.14	8.54	8.25
1374557_at	Wls	0	0	0	0	0	0	7.20	7.24	7.74	7.16	7.38	1	0	0	0	0	1	0	9.59	9.66	9.64	9.80	9.91	10.39	10.10
1374558_at	Icoslg /// LOC10	0	0	0	0	0	0	3.79	3.83	3.79	3.76	4.00	1	0	0	0	0	1	1	5.71	5.57	5.63	5.80	5.88	6.43	6.29
1374564_at	Dtx2	0	0	0	0	0	0	5.73	5.93	5.82	5.89	5.94	1	0	0	0	0	1	1	7.31	7.32	7.44	7.46	7.50	8.13	8.69
1374566_at	Nisch	0	0	0	0	0	0	5.46	5.44	5.56	5.75	5.50	1	0	0	0	0	0	1	6.67	6.67	6.77	6.70	6.62	6.11	5.65
1374569_at	Grwd1	0	0	0	0	0	0	6.28	6.26	6.29	6.49	6.22	1	0	0	0	0	1	1	8.13	8.06	8.08	8.12	7.96	7.39	6.92
1374576_at	Haus5	0	0	0	0	0	0	4.69	4.70	4.57	4.75	4.64	1	0	0	0	0	1	1	7.55	7.56	7.64	7.63	7.50	6.75	6.56
1374588_at	Sumf1	0	0	0	0	0	0	5.92	5.95	6.42	6.14	6.12	1	0	0	0	0	0	1	8.84	8.83	8.83	8.73	8.75	8.35	7.43
1374604_at	Mfsd9	0	0	0	0	0	0	5.10	5.35	5.19	5.45	5.06	1	0	0	0	0	1	1	8.39	8.39	8.56	8.60	8.65	9.43	9.24
1374613_at	---	0	0	0	0	0	0	7.54	7.34	7.54	7.61	7.29	1	0	0	0	0	1	1	9.01	9.12	9.26	9.33	9.41	9.97	9.95
1374618_at	Aff1	0	0	0	0	0	0	7.57	7.56	7.73	7.90	7.70	1	0	0	0	0	1	1	7.20	7.03	7.14	7.09	7.16	7.86	8.05
1374635_at	Adam23	0	0	0	0	0	0	3.95	4.02	3.90	3.97	4.11	1	0	0	0	0	1	1	9.64	9.75	9.87	9.99	10.15	10.50	10.46
1374643_at	Fat4	0	0	0	0	0	0	4.42	4.29	4.33	4.21	4.33	1	0	0	0	0	0	1	5.83	5.68	5.82	5.70	5.80	6.34	7.01
1374648_at	Gpr155	0	0	0	0	0	0	3.79	4.04	3.82	4.16	4.03	1	0	0	0	0	0	1	4.59	4.58	4.61	4.54	4.57	4.95	5.27
1374649_at	Rasgrp2	0	0	0	0	0	0	3.52	3.58	3.39	3.52	3.58	1	0	0	0	1	1	1	5.20	5.28	5.65	5.69	6.12	6.97	7.02
1374655_at	---	0	0	0	0	0	0	5.03	4.92	4.85	4.83	4.91	1	0	0	0	0	0	1	6.41	6.47	6.38	6.43	6.65	6.80	6.51
1374661_at	Aldh16a1	0	0	0	0	0	0	7.27	7.26	7.15	7.70	6.93	1	0	0	0	0	0	1	8.29	8.27	8.34	8.39	8.15	7.90	7.17
1374666_at	---	0	0	0	0	0	0	7.06	7.11	7.37	7.06	7.26	1	0	0	0	0	1	1	8.64	8.70	8.67	8.67	8.62	7.99	7.27
1374674_at	Atxn3	0	0	0	0	0	0	6.28	5.94	6.40	6.30	6.06	1	0	0	0	0	1	1	7.18	7.21	7.42	7.39	7.40	7.82	7.97
1374677_at	Adss1	0	0	0	0	0	0	3.95	4.07	4.12	3.92	3.81	1	0	0	0	0	0	1	7.69	7.77	7.86	7.77	7.87	8.23	8.30
1374681_at	Adcyap1r1	0	0	0	0	0	0	6.67	6.62	6.54	6.44	6.51	1	0	0	0	1	0	0	7.79	7.90	8.11	8.23	8.39	8.34	8.13
1374690_at	Gle1	0	0	0	0	0	0	6.64	6.53	6.72	6.94	6.51	1	0	0	0	0	1	1	8.38	8.33	8.37	8.32	8.14	7.42	7.19
1374704_at	Poglut3	0	0	0	0	0	0	5.60	5.67	5.72	5.55	5.67	1	0	0	0	0	1	1	7.71	7.63	7.65	7.53	7.42	6.05	5.43
1374708_at	Arhgef10	0	0	0	0	0	0	6.02	5.73	5.82	5.78	5.78	1	0	0	0	0	0	1	7.35	7.23	7.33	7.30	7.40	7.60	7.69
1374714_at	Znf516	0	0	0	0	0	0	7.24	7.52	7.68	7.29	7.65	1	0	0	0	0	0	1	7.54	7.56	7.63	7.53	7.67	8.09	8.41
1374716_at	Rrp15	0	0	0	0	0	0	6.18	6.12	6.06	6.09	6.08	1	0	0	0	0	1	1	7.46	7.44	7.51	7.58	7.35	6.83	6.64
1374724_at	Mapk4	0	0	0	0	0	0	6.27	6.30	6.18	5.97	6.02	1	0	0	0	0	1	1	8.33	8.30	8.32	8.01	8.01	7.58	7.37
1374728_at	Arhgap28	0	0	0	0	0	0	4.30	4.24	4.26	4.31	4.32	1	0	0	0	0	0	1	4.88	4.81	4.84	4.70	4.82	5.20	5.94
1374731_at	---	0	0	0	0	0	0	9.05	8.71	8.97	8.98	8.60	1	0	0	0	0	0	1	10.13	10.18	10.24	10.25	10.31	10.65	10.70
1374746_at	---	0	0	0	0	0	0	7.94	7.92	8.13	8.21	8.15	1	0	0	0	0	0	1	8.79	8.88	8.81	8.90	9.02	9.35	9.77
1374768_at	Ccdc65	0	0	0	0	0	0	4.60	4.59	4.68	4.74	4.71	1	0	0	0	1	1	1	4.62	4.83	5.02	5.14	5.36	5.84	6.32
1374770_at	Asah1	0	0	0	0	0	0	7.87	8.12	8.42	8.06	8.16	1	0	0	0	0	1	0	10.39	10.47	10.45	10.53	10.63	10.99	10.42
1374787_at	---	0	0	0	0	0	0	3.63	3.39	3.45	3.64	3.42	1	0	0	0	0	1	1	5.77	5.80	5.85	5.54	5.73	4.77	3.79
1374799_at	Ncapd2	0	0	0	0	0	0	4.12	3.92	4.06	4.26	4.15	1	0	0	0	0	1	1	7.70	7.62	7.71	7.73	7.62	6.91	6.90
1374812_at	Ptpn13	0	0	0	0	0	0	5.76	5.72	6.18	5.76	5.94	1	0	0	0	0	1	1	3.83	3.76	3.88	3.86	3.99	4.52	5.17
1374817_at	Wnt5a	0	0	0	0	0	0	2.91	2.89	2.86	2.85	2.92	1	0	0	0	0	0	1	3.59	3.52	3.54	3.50	3.59	3.73	3.89
1374818_at	Nxph3	0	0	0	0	0	0	5.64	5.65	5.41	5.57	5.64	1	0	0	0	0	1	1	7.43	7.43	7.46	7.51	7.72	8.48	8.30
1374831_at	Pgghg	0	0	0	0	0	0	5.36	5.49	5.38	5.50	5.33	1	0	0	0	0	1	1	7.62	7.50	7.66	7.60	7.46	6.84	6.59
1374833_at	---	0	0	0	0	0	0	5.84	6.02	6.12	5.91	6.26	1	0	0	1	1	1	1	5.37	5.54	5.91	6.15	6.26	7.10	6.73
1374840_at	Ppih	0	0	0	0	0	0	6.30	6.12	6.52	6.73	6.35	1	0	0	0	0	1	1	9.22	9.13	9.14	9.12	8.96	8.25	7.92
1374848_at	Gtf3c6	0	0	0	0	0	0	6.58	6.66	6.83	6.75	6.80	1	0	0	0	0	1	1	7.83	7.79	7.82	7.80	7.71	7.12	6.81
1374852_at	C1orf122	0	0	0	0	0	0	8.88	8.74	8.67	9.07	8.73	1	0	0	0	0	0	1	9.99	9.93	9.96	9.97	9.92	9.56	9.11
1374860_at	LOC100910581 /	0	0	0	0	0	0	5.95	6.11	6.00	6.16	5.96	1	0	0	0	0	1	1	8.70	8.66	8.58	8.41	8.28	7.82	7.43
1374861_at	Tle2	0	0	0	0	0	0	7.24	7.27	7.17	7.27	7.21	1	0	0	0	0	0	1	6.42	6.56	6.64	6.67	6.82	6.89	6.41
1374869_at	---	0	0	0	0	0	0	5.86	5.88	5.98	5.89	6.22	1	0	0	0	0	1	1	9.06	9.20	9.28	9.51	9.36	8.12	6.69
1374871_at	Asrgl1	0	0	0	0	0	0	6.16	6.19	6.37	6.00	6.42	1	0	0	0	0	1	1	8.44	8.57	8.67	8.75	8.82	9.64	9.83
1374872_at	Rasgrp2	0	0	0	0	0	0	3.36	3.38	3.29	3.30	3.27	1	0	0	0	0	1	1	5.17	5.25	5.13	5.18	5.42	5.76	5.43
1374896_at	Srek1	0	0	0	0	0	0	5.95	5.97	6.19	6.00	6.16	1	0	0	0	0	0	1	7.72	7.80	7.83	7.90	7.91	7.33	6.90
1374897_at	Alyref	0	0	0	0	0	0	7.46	7.33	7.52	7.61	7.33	1	0	0	0	0	0	1	11.30	11.26	11.27	11.29	11.18	10.85	10.36
1374902_at	Iqgap3	0	0	0	0	0	0	4.54	4.53	4.47	4.47	4.60	1	0	0	0	0	1	1	7.45	7.26	7.39	7.31	7.12	6.42	6.38
1374907_at	ARHGEF17	0	0	0	0	0	0	4.77	4.76	4.56	4.73	4.91	1	0	0	0	0	1	1	6.41	6.68	6.55	6.31	6.60	7.04	7.19
1374912_at	Kif2c	0	0	0	0	0	0	4.69	4.70	4.68	4.83	4.82	1	0	0	0	0	1	1	8.51	8.51	8.59	8.51	8.42	7.59	7.87
1374916_at	Pwp2	0	0	0	0	0	0	4.68	4.75	4.54	4.55	5.02	1	0	0	0	0	0	1	7.47	7.43	7.65	7.72	7.62	7.09	6.66
1374924_at	LOC100910237	0	0	0	0	0	0	6.08	5.92	5.84	6.39	5.70	1	0	0	0	0	1	0	7.86	7.89	7.93	8.05	8.12	8.53	8.37
1374934_at	---	0	0	0	0	0	0	3.90	3.96	3.72	3.87	3.97	1	0	0	0	0	0	1	4.04	4.11	4.18	4.08	4.23	4.56	4.78
1374941_at	N4bp2	0	0	0	0	0	0	7.51	7.37	7.84	7.56	7.68	1	0	0	0	0	1	1	7.45	7.39	7.43	7.40	7.20	6.54	6.20
1374945_at	Trmt61a	0	0	0	0	0	0	6.79	6.55	6.55	6.79	6.73	1	0	0	0	0	0	1	8.29	8.27	8.34	8.38	8.30	7.79	7.41
1374961_at	Pmf1 /// Pmf1-b	0	0	0	0	0	0	5.05	5.18	4.95	5.35	5.14	1	0	0	0	0	1	1	8.35	8.25	8.31	8.22	8.05	7.39	6.98
1374971_at	Plekhh2	0	0	0	0	0	0	4.85	4.56	4.54	4.60	4.76	1	0	0	0	0	1	1	5.15	4.86	4.99	4.91	5.08	6.15	6.22
1374984_at	Epb41l5	0	0	0	0	0	0	6.07	5.77	5.97	6.12	6.04	1	0	0	0	0	1	1	4.72	5.02	4.85	5.17	5.17	5.87	6.50
1374995_at	Elmo3	0	0	0	0	0	0	7.14	7.13	7.26	7.15	7.24	1	0	0	0	0	0	1	8.01	8.11	8.15	8.14	8.16	7.52	6.55
1375016_at	---	0	0	0	0	0	0	4.30	4.24	4.38	4.42	4.30	1	0	0	0	0	0	1	6.34	6.29	6.52	6.46	6.46	6.81	7.78
1375026_at	Calml4	0	0	0	0	0	0	5.13	5.37	5.41	4.95	5.20	1	0	0	0	0	1	1	10.54	10.56	10.61	10.59	10.50	9.56	8.44
1375030_at	B3galt5	0	0	0	0	0	0	2.93	2.88	2.85	2.91	2.97	1	0	1	1	1	1	1	6.14	6.30	6.77	7.12	7.34	8.38	8.29
1375032_at	Kndc1	0	0	0	0	0	0	3.41	3.40	3.37	3.45	3.34	1	0	0	0	1	1	1	6.14	6.28	6.48	6.42	6.74	6.95	6.94
1375048_at	Mms19	0	0	0	0	0	0	6.78	6.62	6.54	6.81	6.53	1	0	0	0	0	0	1	8.52	8.59	8.73	8.75	8.75	9.09	9.18
1375053_at	Pknox2	0	0	0	0	0	0	3.76	3.82	3.62	3.81	3.76	1	0	0	0	0	1	1	6.75	6.82	7.11	7.06	7.12	7.55	7.47
1375057_at	Tspan18	0	0	0	0	0	0	5.44	5.35	5.04	5.36	5.25	1	0	0	0	0	1	1	5.53	5.78	5.70	5.80	5.93	6.70	6.85
1375062_at	Ap1m2	0	0	0	0	0	0	6.98	7.05	6.88	7.26	6.78	1	0	0	0	0	1	1	8.63	8.56	8.61	8.54	8.50	7.66	7.35
1375066_at	Ifitm10	0	0	0	0	0	0	4.54	4.56	4.23	4.37	4.39	1	0	0	0	0	0	1	6.24	6.39	6.35	6.14	6.25	5.91	5.71
1375071_at	Nup133	0	0	0	0	0	0	4.30	4.24	4.31	4.64	4.43	1	0	0	0	0	1	1	6.80	6.61	6.67	6.76	6.82	6.05	6.06
1375105_at	Aven	0	0	0	0	0	0	4.54	4.49	4.55	4.43	4.55	1	0	0	0	0	0	1	5.45	5.37	5.46	5.42	5.25	5.03	3.79
1375117_at	Akt1s1	0	0	0	0	0	0	6.13	5.87	6.05	5.85	5.89	1	0	0	0	0	0	1	7.93	7.97	8.06	8.12	7.96	7.53	6.71
1375118_at	---	0	0	0	0	0	0	5.10	4.67	5.08	4.85	4.98	1	0	0	0	0	1	1	6.77	6.70	6.86	6.84	6.79	6.14	5.17
1375123_at	Sox4	0	0	0	0	0	0	6.14	5.99	6.59	6.21	6.22	1	0	0	0	0	1	1	10.18	10.15	10.31	10.39	10.40	11.04	10.49
1375163_at	Rab11b	0	0	0	0	0	0	8.92	8.88	9.08	8.95	8.93	1	0	0	0	0	0	1	10.14	10.12	10.14	10.04	9.98	9.75	9.05
1375183_at	Id4	0	0	0	0	0	0	4.41	4.41	4.29	4.59	4.33	1	0	0	0	0	1	0	7.01	6.94	6.99	7.04	6.74	6.33	6.65
1375186_at	Dph3	0	0	0	0	0	0	8.87	8.90	8.78	9.01	8.94	1	0	0	0	0	1	1	10.74	10.71	10.66	10.61	10.51	10.07	9.98
1375205_at	Kat2b	0	0	0	0	0	0	8.22	8.01	8.46	8.00	8.38	1	0	0	0	0	1	1	9.19	9.15	9.23	9.19	9.05	8.31	7.78
1375206_at	Wscd1	0	0	0	0	0	0	5.85	5.80	5.75	5.76	5.78	1	0	0	0	0	1	1	7.97	8.09	8.21	8.23	8.31	8.74	8.65
1375215_x_at	Pgpep1	0	0	0	0	0	0	8.80	8.71	8.66	9.19	8.59	1	0	0	0	0	1	1	10.32	10.44	10.41	10.43	10.58	10.95	10.57
1375216_at	Nectin2	0	0	0	0	0	0	5.83	5.66	5.86	5.93	5.97	1	0	0	0	0	1	1	8.09	8.21	8.33	8.37	8.54	9.05	8.87
1375243_at	BTBD2	0	0	0	0	0	0	7.79	8.09	7.79	7.89	8.10	1	0	0	1	0	0	0	6.66	6.58	6.47	6.06	6.22	6.33	6.37
1375264_at	---	0	0	0	0	0	0	6.40	6.19	6.65	6.41	6.11	1	0	0	0	0	1	0	7.32	7.30	7.48	7.21	7.33	6.73	6.87
1375267_at	Ppic	0	0	0	0	0	0	4.28	4.03	4.36	4.16	4.13	1	0	0	0	1	1	0	5.65	5.86	5.90	5.97	6.26	6.29	4.58

Probeset ID	Gene Symbol	Islet Data Set (log2 ratio ≥0.263; FDR <20%) union n=5325 1=YES 0=NO	Islet DRlyp/lyp ND vs ND+Lp299v (log2 ratio ≥0.263; FDR<20%) n=429 1=YES 0=NO	Islet DRlyp/lyp ND vs HCD (log2 ratio ≥0.263; FDR<20%) n=2247 1=YES 0=NO	Islet DRlyp/lyp ND vs HCD+Lp299v (log2 ratio ≥0.263; FDR<20%) n=1595 1=YES 0=NO	Islet DRlyp/lyp HCD vs HCD+Lp299v (log2 ratio ≥0.263; FDR<20%) n=3026 1=YES 0=NO	Islet DRlyp/lyp ND vs Flyp/lyp ND (log2 ratio ≥ 0.263; FDR<20%) n=1950 1=YES 0=NO	Islet Mean Log2 Intensity DRlyp/lyp ND	Islet Mean Log2 Intensity DRlyp/lyp ND+Lp299v	Islet Mean Log2 Intensity DRlyp/lyp HCD	Islet Mean Log2 Intensity DRlyp/lyp HCD+Lp299v	Islet Mean Log2 Intensity Flyp40 ND	Rinm5f data union of all conditions n=3705 1=YES 0=NO	Rinm5f 30 µM propionate/ 6 µM butyrate vs Media (FDR<20%; 1.5 fold) n=3 1=YES 0=NO	Rinm5f 60 µM propionate/ 12 µM butyrate vs Media (FDR<20%; 1.5 fold) n=18 1=YES 0=NO	Rinm5f 120 µM propionate/ 24 µM butyrate vs Media (FDR<20%; 1.5 fold) n=164 1=YES 0=NO	Rinm5f 240 µM propionate /48 µM butyrate vs Media (FDR<20%; 1.5 fold) n=268 1=YES 0=NO	Rinm5f 1500 µM propionate/ 300 µM butyrate vs Media (FDR<20%; 1.5 fold) n=2491 1=YES 0=NO	Rinm5f 500 µM propionate/ 500 µM butyrate vs Media (FDR<20%; 1.5 fold) n=3001 1=YES 0=NO	Rinm5f Mean Log2 Intensity Media only	Rinm5f Mean Log2 Intensity 30 µM propionate/ 6 µM butyrate	Rinm5f Mean Log2 Intensity 60 µM propionate/ 12 µM butyrate	Rinm5f Mean Log2 Intensity 120 µM propionate/ 24 µM butyrate	Rinm5f Mean Log2 Intensity 240 µM propionate/ 48 µM butyrate	Rinm5f Mean Log2 Intensity 1500 µM propionate /300 µM butyrate	Rinm5f Mean Log2 Intensity 500 µM propionate/ 500 µM butyrate
1375294_at	---	0	0	0	0	0	0	5.73	5.96	6.02	5.55	6.06	1	0	0	0	0	1	1	5.76	5.92	6.21	6.03	6.27	6.40	6.11
1375308_at	Znf646	0	0	0	0	0	0	4.29	4.33	4.36	4.21	4.42	1	0	0	0	0	0	1	8.24	8.27	8.33	8.23	8.15	7.72	6.86
1375351_at	Ssbp4	0	0	0	0	0	0	6.21	6.06	6.22	5.91	6.21	1	0	0	0	0	1	0	8.58	8.64	8.81	8.73	8.82	9.17	8.48
1375353_at	Cables1	0	0	0	0	0	0	6.60	6.76	6.41	6.71	6.63	1	0	0	0	0	0	1	8.26	8.25	8.26	8.10	8.09	7.81	7.20
1375374_at	LOC102555400	0	0	0	0	0	0	7.45	7.77	7.47	7.46	7.28	1	0	0	0	0	0	1	8.67	8.73	8.91	8.94	8.91	8.45	7.73
1375435_at	Arpin /// Arpin-a	0	0	0	0	0	0	7.30	7.26	7.01	7.32	7.10	1	0	0	0	0	1	1	8.56	8.45	8.46	8.44	8.26	7.53	7.04
1375443_at	Tpgs1	0	0	0	0	0	0	5.66	5.50	5.55	5.54	5.50	1	0	0	0	0	0	1	9.08	9.05	9.13	9.15	9.05	8.63	8.04
1375455_at	Guca1a	0	0	0	0	0	0	3.12	3.17	3.04	3.13	3.16	1	0	0	0	0	0	1	3.99	3.96	4.11	3.96	3.91	4.40	5.14
1375464_at	Vsig10l	0	0	0	0	0	0	5.52	5.99	5.67	5.78	5.71	1	0	0	0	0	1	1	5.83	5.71	5.66	5.77	5.62	5.21	4.45
1375526_at	Tmem256	0	0	0	0	0	0	8.30	8.26	8.13	8.58	8.12	1	0	0	0	0	0	1	8.70	8.80	8.80	8.79	8.83	9.28	9.40
1375558_at	Rab34	0	0	0	0	0	0	6.66	6.78	6.55	6.65	6.67	1	0	0	0	0	0	1	8.20	8.28	8.32	8.14	8.06	7.81	7.10
1375579_at	Ndufaf8	0	0	0	0	0	0	7.34	7.34	6.99	7.51	6.99	1	0	0	0	0	1	1	9.40	9.36	9.32	9.28	9.19	8.68	8.45
1375580_at	Dpp9	0	0	0	0	0	0	7.60	7.68	7.29	7.79	7.52	1	0	0	0	0	1	1	9.28	9.25	9.29	9.24	9.12	8.66	8.70
1375633_at	Clic1	0	0	0	0	0	0	9.11	9.09	9.34	8.85	9.35	1	0	0	0	0	1	1	11.28	11.28	11.28	11.22	11.13	10.57	10.19
1375640_at	Fkbp9	0	0	0	0	0	0	8.13	8.14	8.10	8.30	7.69	1	0	0	0	0	1	1	6.66	6.77	6.74	6.70	6.85	7.67	7.30
1375649_at	POU2F2	0	0	0	0	0	0	5.10	5.16	4.74	5.25	5.12	1	0	0	0	0	1	0	6.42	6.58	6.66	6.49	6.70	7.08	6.93
1375658_at	Sash1	0	0	0	0	0	0	7.14	7.21	7.20	7.20	7.18	1	0	0	0	0	0	1	7.28	7.30	7.34	7.33	7.40	7.79	8.37
1375660_at	Septin8	0	0	0	0	0	0	6.40	6.44	6.43	6.54	6.45	1	0	0	0	0	0	1	7.96	7.96	7.98	7.96	7.84	7.58	7.26
1375681_at	Dcaf15	0	0	0	0	0	0	4.66	4.48	4.51	4.53	4.49	1	0	0	0	0	0	1	6.50	6.46	6.60	6.54	6.38	6.03	5.10
1375691_at	B4galnt4	0	0	0	0	0	0	4.28	4.09	4.02	4.09	4.16	1	0	0	0	0	1	0	6.06	6.20	6.37	6.43	6.44	6.66	5.67
1375693_at	---	0	0	0	0	0	0	4.07	4.19	3.80	3.95	3.98	1	0	0	0	0	1	1	6.65	6.65	6.68	6.52	6.39	5.76	4.80
1375729_at	Epha4	0	0	0	0	0	0	4.85	4.71	4.79	4.72	4.76	1	0	0	0	0	1	1	5.03	4.97	4.93	4.63	4.83	5.64	6.93
1375763_at	Heatr6	0	0	0	0	0	0	5.94	5.80	5.86	6.02	6.02	1	0	0	0	0	1	0	8.54	8.32	8.48	8.33	8.22	7.96	7.99
1375764_at	Chmp2b	0	0	0	0	0	0	3.57	3.39	3.58	3.25	3.53	1	0	0	0	0	0	1	4.82	4.73	4.86	4.99	4.84	4.47	3.35
1375771_at	Sidt1	0	0	0	0	0	0	4.61	4.51	4.54	4.50	4.37	1	0	0	0	0	0	1	8.41	8.36	8.32	8.22	8.18	7.88	7.47
1375795_at	---	0	0	0	0	0	0	4.18	4.23	4.05	4.16	4.05	1	0	0	0	0	0	1	5.05	5.22	5.30	5.20	5.09	5.57	5.66
1375820_at	---	0	0	0	0	0	0	3.19	3.19	3.12	3.14	3.16	1	0	0	0	0	1	1	6.84	7.02	7.29	7.42	7.25	6.09	5.17
1375836_at	---	0	0	0	0	0	0	2.73	2.70	2.71	2.81	2.91	1	0	0	0	0	1	0	4.80	4.61	4.89	4.57	4.51	4.08	3.94
1375849_at	Rgma	0	0	0	0	0	0	4.56	4.40	4.21	4.33	4.32	1	0	0	0	0	1	1	6.71	6.90	6.87	6.99	7.00	7.68	8.11
1375851_at	Klhl8	0	0	0	0	0	0	6.50	6.37	6.67	6.40	6.20	1	0	0	0	0	1	1	6.71	6.62	6.83	6.78	6.82	7.46	7.54
1375890_at	Dact3	0	0	0	0	0	0	4.71	4.74	4.61	4.83	4.78	1	0	0	0	0	1	1	7.84	7.87	7.96	7.96	7.97	8.66	8.49
1375893_a_at	C6orf89	0	0	0	0	0	0	5.25	5.35	4.99	5.39	5.39	1	0	0	0	0	1	0	6.52	6.50	6.69	6.70	6.68	7.21	7.24
1375894_at	Lztfl1	0	0	0	0	0	0	6.44	6.10	6.93	6.40	6.38	1	0	0	0	0	0	1	6.68	6.84	6.82	6.80	6.86	6.44	5.80
1375905_at	Ttyh1	0	0	0	0	0	0	3.44	3.49	3.62	3.48	3.37	1	0	0	0	1	1	1	3.64	3.62	4.02	4.18	4.47	5.43	5.05
1375906_at	Fam104b	0	0	0	0	0	0	6.25	5.97	6.04	6.10	6.13	1	0	0	0	0	1	1	9.27	9.27	9.31	9.29	9.22	8.67	8.33
1375910_at	Cdc42ep3	0	0	0	0	0	0	8.14	8.23	8.54	8.26	8.24	1	0	0	0	0	0	1	11.17	11.28	11.27	11.23	11.34	11.24	9.71
1375911_at	Gpcpd1	0	0	0	0	0	0	4.76	4.69	4.89	5.16	4.89	1	0	0	0	0	1	1	7.02	7.16	7.23	7.43	7.51	7.73	7.75
1375934_at	Rnf128	0	0	0	0	0	0	7.50	7.30	7.31	7.83	7.47	1	0	0	0	0	1	1	7.45	7.45	7.42	7.36	7.38	8.28	8.64
1375937_a_at	Cenpt	0	0	0	0	0	0	3.74	3.66	3.47	3.70	3.57	1	0	0	0	0	1	1	6.87	6.79	6.88	6.90	6.66	5.60	5.94
1375938_at	Ccdc12	0	0	0	0	0	0	6.99	6.94	7.04	7.04	7.06	1	0	0	0	0	1	1	7.52	7.55	7.56	7.50	7.36	6.93	6.34
1375954_at	S100a13	0	0	0	0	0	0	7.27	7.26	7.13	7.26	7.23	1	0	0	0	1	1	1	7.33	7.54	7.61	7.77	8.06	9.17	9.31
1375957_at	Nkain1	0	0	0	0	0	0	4.73	4.79	4.58	4.77	4.76	1	0	0	0	0	1	1	7.70	7.78	7.90	7.82	7.96	8.33	8.40
1375965_at	Ube2k	0	0	0	0	0	0	7.22	6.99	7.55	7.14	7.42	1	0	0	0	1	1	0	8.54	8.63	8.88	9.07	9.13	9.52	9.24
1375971_at	Cln5	0	0	0	0	0	0	6.31	6.34	6.55	6.43	6.49	1	0	0	0	0	1	1	6.74	6.65	6.87	7.01	6.92	7.37	7.64
1375978_at	Fcho1	0	0	0	0	0	0	3.61	3.56	3.40	3.55	3.48	1	0	1	1	1	1	0	5.59	5.92	6.29	6.26	6.38	6.90	5.49
1375981_a_at	Suv39h1	0	0	0	0	0	0	6.96	7.12	6.98	7.13	7.16	1	0	0	0	0	1	1	9.16	9.06	9.06	9.03	8.90	8.43	8.52
1375988_at	Hyls1	0	0	0	0	0	0	6.87	6.84	7.22	6.98	6.98	1	0	0	0	0	0	1	9.11	9.06	9.06	9.10	9.01	8.54	8.28
1375992_at	Cfap77	0	0	0	0	0	0	4.81	4.76	4.57	4.85	4.85	1	0	0	0	0	1	1	5.50	5.57	5.64	5.85	6.00	7.49	7.33
1376004_at	Nudt21	0	0	0	0	0	0	6.76	6.41	7.32	6.67	6.82	1	0	0	0	0	1	0	8.44	8.58	8.72	8.82	8.93	9.07	8.75
1376008_at	Cbx8	0	0	0	0	0	0	4.98	5.00	4.83	4.97	4.90	1	0	0	0	0	1	1	6.48	6.48	6.51	6.34	6.20	5.76	5.23
1376011_at	Tmem44	0	0	0	0	0	0	3.62	3.55	3.50	3.53	3.58	1	0	0	0	0	1	0	6.56	6.67	6.63	6.46	6.46	5.90	6.00
1376017_at	Wdr17	0	0	0	0	0	0	3.06	3.02	2.95	3.07	2.94	1	0	0	0	0	0	1	4.05	4.06	4.10	3.98	4.00	4.59	5.00
1376028_at	Msl1	0	0	0	0	0	0	7.26	7.04	7.14	7.60	7.06	1	0	0	0	0	0	1	8.44	8.44	8.53	8.47	8.37	7.87	7.78
1376035_at	Ttc7b	0	0	0	0	0	0	6.60	6.50	6.87	6.31	6.60	1	0	0	0	0	1	1	9.47	9.36	9.39	9.37	9.31	8.84	8.64
1376037_at	Aaas	0	0	0	0	0	0	4.85	4.96	4.74	5.05	4.99	1	0	0	0	0	1	1	8.00	8.01	8.15	8.14	7.94	7.39	7.12
1376038_at	Tex2	0	0	0	0	0	0	7.57	7.49	7.67	7.72	7.65	1	0	0	0	0	1	1	9.56	9.61	9.66	9.60	9.77	10.32	10.46
1376039_at	Aurka	0	0	0	0	0	0	4.17	4.05	4.35	4.31	4.42	1	0	0	0	0	1	1	9.24	9.14	9.19	9.11	8.99	7.73	7.65
1376040_at	Sipa1l2	0	0	0	0	0	0	6.41	6.19	6.56	6.16	6.34	1	0	0	0	0	0	1	6.85	7.01	7.03	6.93	7.08	7.40	7.75
1376048_at	Guca1b	0	0	0	0	0	0	3.99	4.14	3.76	3.88	3.88	1	0	0	0	1	1	1	6.78	6.96	7.11	7.28	7.40	7.95	7.31
1376051_at	Cryl1	0	0	0	0	0	0	6.32	6.27	6.44	6.54	6.31	1	0	0	0	0	0	1	6.43	6.43	6.52	6.55	6.43	7.01	7.32
1376066_at	Rnd3	0	0	0	0	0	0	6.52	6.65	6.86	6.90	6.78	1	0	0	0	0	0	1	5.99	6.06	6.20	6.16	6.09	6.36	6.96
1376072_at	Fam174a	0	0	0	0	0	0	5.73	5.74	6.29	5.71	6.06	1	0	0	0	0	0	1	8.20	8.17	8.11	8.08	8.12	7.71	6.97
1376082_at	Mecom	0	0	0	0	0	0	7.68	7.58	7.43	7.96	7.40	1	0	0	0	0	0	1	7.30	7.34	7.46	7.58	7.61	7.87	7.85
1376084_a_at	Espl1	0	0	0	0	0	0	5.23	5.28	5.14	5.30	5.34	1	0	0	0	0	1	1	8.65	8.59	8.65	8.62	8.48	7.36	7.77
1376085_at	Tmem185a	0	0	0	0	0	0	8.06	7.93	8.30	8.02	8.10	1	0	0	0	0	0	1	10.16	10.14	10.10	10.06	10.05	9.61	9.23
1376089_at	Ldlr	0	0	0	0	0	0	8.25	8.58	8.35	8.17	8.57	1	0	0	0	0	1	1	9.71	9.63	9.64	9.54	9.53	8.51	8.30
1376106_at	Tmem178a	0	0	0	0	0	0	4.39	4.35	4.18	4.08	4.24	1	0	0	0	0	0	1	9.16	9.23	9.26	9.19	9.23	8.71	8.04
1376110_at	Rpp25	0	0	0	0	0	0	3.47	3.56	3.33	3.51	3.51	1	0	0	0	1	1	1	4.98	4.95	5.13	5.42	5.66	6.96	7.34
1376112_a_at	Nutf2	0	0	0	0	0	0	7.59	7.68	7.79	7.69	7.77	1	0	0	0	0	1	0	8.00	8.03	8.13	8.26	8.35	8.64	8.71
1376123_at	Arfgap1	0	0	0	0	0	0	7.07	7.14	7.10	7.22	7.06	1	0	0	0	0	0	1	9.64	9.66	9.68	9.60	9.52	9.19	8.75
1376148_at	LOC103694491	0	0	0	0	0	0	6.47	6.73	6.76	6.62	6.78	1	0	0	0	0	1	1	7.68	7.57	7.66	7.54	7.44	6.93	6.64
1376183_at	Dpp6	0	0	0	0	0	0	6.47	6.55	6.49	6.32	6.83	1	0	0	0	0	1	1	9.64	9.70	9.58	9.27	9.44	8.68	8.28
1376184_at	Lynx1	0	0	0	0	0	0	4.69	4.54	4.24	4.77	4.45	1	0	0	0	0	1	1	6.78	6.97	6.98	7.03	7.15	7.86	7.99
1376185_at	Kifc1	0	0	0	0	0	0	5.96	5.74	5.71	6.03	5.94	1	0	0	0	0	1	1	9.74	9.65	9.71	9.63	9.50	8.72	8.85
1376217_at	Casc1	0	0	0	0	0	0	5.92	5.77	5.58	5.74	5.89	1	0	0	0	0	1	0	5.67	5.93	5.89	5.90	5.95	6.29	6.77
1376228_at	Kcnn1	0	0	0	0	0	0	4.08	4.13	3.89	3.85	4.18	1	0	0	0	0	1	0	6.52	6.61	6.70	6.59	6.64	7.12	7.15
1376239_at	Atp6v1c2	0	0	0	0	0	0	4.94	4.89	5.11	4.87	4.71	1	0	0	0	0	1	1	6.37	6.45	6.57	6.53	6.71	7.22	7.22
1376252_at	Srsf3	0	0	0	0	0	0	6.47	6.56	6.66	6.55	6.86	1	0	0	0	0	1	0	8.30	8.39	8.53	8.78	8.74	9.01	8.88
1376255_at	Map4k1	0	0	0	0	0	0	4.85	4.97	4.88	4.83	4.86	1	0	0	0	0	1	1	7.23	7.31	7.43	7.37	7.60	7.88	8.09
1376259_at	Prkcq	0	0	0	0	0	0	4.95	4.93	4.77	5.00	4.98	1	0	0	0	0	0	1	4.65	4.72	4.54	4.43	4.53	4.72	5.59
1376282_at	Nuak1	0	0	0	0	0	0	5.99	6.20	5.86	6.11	6.20	1	0	0	0	0	1	0	7.79	7.66	7.73	7.33	7.42	7.10	7.86
1376284_at	---	0	0	0	0	0	0	4.34	4.26	4.26	4.21	4.30	1	0	0	1	1	1	1	7.09	7.19	7.48	7.74	7.83	8.45	8.19
1376295_at	Glt1d1	0	0	0	0	0	0	5.95	5.90	5.99	5.61	5.73	1	0	0	0	0	0	1	7.34	7.35	7.46	7.52	7.33	6.80	6.07
1376345_at	Caly	0	0	0	0	0	0	7.04	7.36	7.15	6.84	7.23	1	0	0	0	1	0	0	8.63	8.89	9.07	9.15	9.33	9.16	8.27
1376349_a_at	LOC100911721 /	0	0	0	0	0	0	5.28	5.32	5.24	5.42	5.25	1	0	0	0	0	1	1	7.89	7.89	7.99	7.99	7.87	7.27	7.13
1376358_at	---	0	0	0	0	0	0	3.33	3.68	3.59	3.47	3.59	1	0	0	0	0	0	1	5.70	5.68	5.91	6.03	6.11	6.21	6.96
1376369_at	Troap	0	0	0	0	0	0	6.51	6.79	6.68	6.70	6.81	1	0	0	0	0	1	1	9.81	9.80	9.85	9.72	9.65	9.04	9.26
1376381_at	Xkr5	0	0	0	0	0	0	4.63	4.72	4.46	4.65	4.53	1	0	0	0	0	1	1	7.61	7.38	7.52	7.34	7.11	6.21	5.99
1376387_at	---	0	0	0	0	0	0	3.63	3.76	3.57	3.65	3.70	1	0	0	0	0	1	1	4.18	4.27	4.38	4.25	4.34	4.92	5.05
1376410_at	Mmp17	0	0	0	0	0	0	5.98	6.24	5.61	6.03	5.72	1	0	0	0	0	1	0	7.71	7.87	7.77	7.63	7.94	8.68	9.01
1376417_at	Syt6	0	0	0	0	0	0	4.94	4.99	4.49	4.75	4.90	1	0	0	0	0	1	0	6.91	6.99	7.21	7.06	7.05	6.29	5.90
1376418_a_at	Iars1	0	0	0	0	0	0	8.63	8.43	8.95	8.58	8.89	1	0	0	0	0	0	1	9.68	9.70	9.72	9.95	9.93	9.34	8.50
1376425_at	Tgfb2	0	0	0	0	0	0	5.91	6.00	6.45	5.80	6.05	1	0	0	0	0	1	0	6.27	6.29	6.31	5.96	6.21	5.62	5.61
1376457_at	Crispld2	0	0	0	0	0	0	4.69	4.31	4.37	4.25	4.31	1	0	0	0	0	0	1	3.99	4.03	3.93	3.77	3.94	4.46	4.79
1376462_at	---	0	0	0	0	0	0	3.42	3.52	3.51	3.43	3.22	1	0	0	0	0	0	1	7.57	7.63	7.81	7.80	7.78	7.20	6.45
1376485_at	Traip	0	0	0	0	0	0	3.82	3.90	3.68	3.99	3.91	1	0	0	0	0	1	1	6.97	6.98	7.04	6.90	6.94	6.32	6.12
1376496_at	Apol9a /// Apol9	0	0	0	0	0	0	3.24	3.36	3.16	3.25	3.19	1	0	0	0	1	0	0	5.02	5.05	5.18	5.30	5.63	5.54	4.87
1376522_at	---	0	0	0	0	0	0	4.45	4.60	4.12	4.56	4.32	1	0	0	0	0	1	1	6.51	6.49	6.50	6.03	6.07	5.37	5.31
1376532_at	Fndc3b	0	0	0	0	0	0	3.98	4.06	4.00	3.67	4.10	1	0	0	0	0	0	1	9.00	8.77	8.96	8.86	9.04	8.96	7.41
1376554_at	Tatdn1	0	0	0	0	0	0	5.71	5.62	5.86	5.79	5.73	1	0	0	0	0	1	0	6.86	6.96	6.92	7.03	7.12	7.46	6.85
1376573_at	Rab34	0	0	0	0	0	0	7.22	7.20	7.43	7.12	7.25	1	0	0	0	0	0	1	10.51	10.52	10.52	10.45	10.41	9.93	9.31
1376592_at	Mcee	0	0	0	0	0	0	6.68	6.58	6.86	6.74	6.65	1	0	0	0	0	1	1	8.63	8.65	8.72	8.85	8.94	9.36	9.34
1376610_a_at	Ttc28	0	0	0	0	0	0	6.60	6.59	6.83	6.48	7.03	1	0	0	0	0	1	1	5.80	5.89	6.00	5.95	6.17	6.41	6.93
1376611_at	Pola1	0	0	0	0	0	0	4.94	5.06	5.34	5.17	5.33	1	0	0	0	0	1	0	9.08	9.17	9.16	9.31	9.44	9.68	9.55
1376625_at	Oip5-as1	0	0	0	0	0	0	5.29	5.35	5.12	5.57	5.06	1	0	0	0	0	1	0	6.87	6.72	6.81	6.65	6.49	6.13	6.07
1376627_at	C1galt1	0	0	0	0	0	0	7.72	7.83	8.03	7.86	7.93	1	0	0	0	0	1	0	9.29	9.33	9.32	9.34	9.52	9.94	10.02
1376632_at	Lmcd1	0	0	0	0	0	0	4.67	4.54	4.56	4.72	4.70	1	0	0	0	0	1	1	3.98	4.02	4.18	4.17	4.34	5.44	5.80
1376655_at	Arpp19	0	0	0	0	0	0	7.92	7.80	8.25	7.75	8.13	1	0	0	0	0	1	1	8.54	8.62	8.74	8.82	8.94	9.47	9.16
1376661_at	Kazn	0	0	0	0	0	0	5.06	5.20	4.80	5.32	5.02	1	0	0	0	0	1	1	6.53	6.89	6.95	6.91	7.03	7.49	7.60
1376663_at	Dcaf15	0	0	0	0	0	0	5.30	5.43	5.14	5.27	5.30	1	0	0	0	0	0	1	7.60	7.57	7.61	7.58	7.46	7.16	6.98
1376677_a_at	Zbtb11os1	0	0	0	0	0	0	4.65	4.71	5.13	4.75	4.82	1	0	0	0	0	0	1	8.09	8.08	8.15	8.17	8.08	7.62	6.79
1376679_at	Mtx3	0	0	0	0	0	0	5.60	5.56	5.88	5.65	5.68	1	0	0	0	0	0	1	7.82	7.92	7.97	8.01	7.93	7.49	7.08
1376684_at	Dlgap5	0	0	0	0	0	0	5.64	5.56	5.87	5.80	5.81	1	0	0	0	0	1	1	8.40	8.41	8.46	8.36	8.29	7.67	7.97
1376700_at	Lima1	0	0	0	0	0	0	7.34	7.45	7.62	7.60	7.29	1	0	0	0	0	1	1	7.30	7.32	7.47	7.50	7.64	8.49	9.19
1376711_at	Cldn11	0	0	0	0	0	0	3.32	3.25	3.22	3.15	3.20	1	0	0	0	0	1	1	4.38	4.28	4.61	4.58	4.64	5.47	7.07
1376719_at	Tmem38b	0	0	0	0	0	0	3.94	3.92	4.14	3.93	3.81	1	0	0	0	0	1	0	6.97	7.10	7.02	7.16	7.25	7.58	7.18
1376751_at	Stk19	0	0	0	0	0	0	5.92	5.98	6.05	5.99	5.76	1	0	0	0	0	0	1	8.07	8.14	8.15	8.11	8.00	7.81	7.18
1376755_at	Rarb	0	0	0	0	0	0	3.82	3.85	3.88	3.86	3.86	1	0	0	0	0	0	1	5.58	5.51	5.60	5.88	5.58	6.05	6.65
1376765_at	Mro	0	0	0	0	0	0	3.24	3.23	3.11	3.20	3.22	1	0	0	0	0	0	1	4.25	4.20	4.34	4.27	4.25	4.78	5.39
1376770_at	Efhd1	0	0	0	0	0	0	6.50	6.42	6.54	6.87	6.47	1	0	0	0	0	1	0	6.38	6.48	6.61	6.61	6.89	7.19	7.56
1376779_at	Foxo1	0	0	0	0	0	0	6.57	6.56	6.58	6.51	6.56	1	0	0	0	0	0	1	6.53	6.52	6.59	6.64	6.65	7.11	8.12
1376783_at	Arrb1	0	0	0	0	0	0	4.59	4.50	4.41	4.58	4.38	1	0	0	0	0	1	1	6.39	6.42	6.58	6.48	6.53	7.07	7.14
1376786_a_at	Neo1	0	0	0	0	0	0	9.24	9.33	9.53	9.18	9.32	1	0	0	0	0	1	1	7.79	7.84	8.02	7.96	7.98	8.49	9.09
1376795_at	Pik3ap1	0	0	0	0	0	0	4.84	4.93	5.06	5.08	5.11	1	0	0	0	0	1	1	5.30	5.28	5.36	5.43	5.58	5.93	5.86
1376799_a_at	Crlf1	0	0	0	0	0	0	3.80	3.99	3.68	4.09	3.68	1	0	0	0	0	1	1	7.03	7.08	7.36	7.37	7.56	8.06	8.54
1376808_at	Mturn	0	0	0	0	0	0	6.38	6.35	6.58	6.72	5.98	1	0	0	0	0	1	1	6.66	6.82	6.76	6.76	6.80	7.75	8.28
1376809_at	Fbrsl1	0	0	0	0	0	0	6.37	6.21	6.52	6.38	6.46	1	0	0	0	0	1	0	8.42	8.51	8.60	8.58	8.65	9.01	8.81
1376831_at	Pimreg	0	0	0	0	0	0	3.46	3.45	3.43	3.35	3.31	1	0	0	0	0	1	1	8.02	7.99	7.99	7.99	7.75	6.63	7.03
1376836_at	LOC102546798 /	0	0	0	0	0	0	3.32	3.15	2.94	3.08	3.10	1	0	0	1	1	1	0	5.45	5.41	5.82	6.32	6.04	6.12	5.91
1376844_at	Ap3b2	0	0	0	0	0	0	4.37	4.37	4.54	4.29	4.45	1	0	0	0	0	0	1	7.08	7.04	7.30	7.34	7.26	6.99	5.68
1376860_at	Slc22a23	0	0	0	0	0	0	5.02	4.74	5.30	5.46	5.10	1	0	0	0	0	1	1	6.72	6.64	6.90	7.07	6.94	7.60	7.94
1376865_at	Onecut2	0	0	0	0	0	0	3.54	3.92	3.55	3.72	3.64	1	0	0	0	0	1	1	7.41	7.44	7.72	7.85	7.84	8.73	9.08
1376868_at	Cobll1	0	0	0	0	0	0	8.14	7.95	8.29	8.16	8.12	1	0	0	0	0	1	1	7.64	7.62	7.75	7.92	7.93	8.65	8.73
1376873_at	Cbln1	0	0	0	0	0	0	5.02	5.15	4.81	4.96	5.05	1	0	0	0	0	1	1	8.98	9.11	9.19	9.27	9.38	10.20	9.89
1376899_at	Ccdc106	0	0	0	0	0	0	4.23	3.93	3.90	4.12	4.06	1	0	0	0	0	0	1	5.49	5.45	5.39	5.34	5.11	4.92	4.69

Probeset ID	Gene Symbol	Islet Data Set (log2 ratio ≥0.263; FDR <20%) union n=5325 1=YES 0=NO	Islet DRlyp/lyp ND vs ND+Lp299v (log2 ratio ≥0.263; FDR<20%) n=429 1=YES 0=NO	Islet DRlyp/lyp ND vs HCD (log2 ratio ≥0.263; FDR<20%) n=2247 1=YES 0=NO	Islet DRlyp/lyp ND vs HCD+Lp299v (log2 ratio ≥0.263; FDR<20%) n=1595 1=YES 0=NO	Islet DRlyp/lyp HCD vs HCD+Lp299v (log2 ratio ≥0.263; FDR<20%) n=3026 1=YES 0=NO	Islet DRlyp/lyp ND vs Flyp/lyp ND (log2 ratio ≥ 0.263; FDR<20%) n=1950 1=YES 0=NO	Islet Mean Log2 Intensity DRlyp/lyp ND	Islet Mean Log2 Intensity DRlyp/lyp ND+Lp299v	Islet Mean Log2 Intensity DRlyp/lyp HCD	Islet Mean Log2 Intensity DRlyp/lyp HCD+Lp299v	Islet Mean Log2 Intensity Flyp40 ND	Rinm5f data union of all conditions n=3705 1=YES 0=NO	Rinm5f 30 µM propionate/ 6 µM butyrate vs Media (FDR<20%; 1.5 fold) n=3 1=YES 0=NO	Rinm5f 60 µM propionate/ 12 µM butyrate vs Media (FDR<20%; 1.5 fold) n=18 1=YES 0=NO	Rinm5f 120 µM propionate/ 24 µM butyrate vs Media (FDR<20%; 1.5 fold) n=164 1=YES 0=NO	Rinm5f 240 µM propionate /48 µM butyrate vs Media (FDR<20%; 1.5 fold) n=268 1=YES 0=NO	Rinm5f 1500 µM propionate/ 300 µM butyrate vs Media (FDR<20%; 1.5 fold) n=2491 1=YES 0=NO	Rinm5f 500 µM propionate/ 500 µM butyrate vs Media (FDR<20%; 1.5 fold) n=3001 1=YES 0=NO	Rinm5f Mean Log2 Intensity Media only	Rinm5f Mean Log2 Intensity 30 µM propionate/ 6 µM butyrate	Rinm5f Mean Log2 Intensity 60 µM propionate/ 12 µM butyrate	Rinm5f Mean Log2 Intensity 120 µM propionate/ 24 µM butyrate	Rinm5f Mean Log2 Intensity 240 µM propionate/ 48 µM butyrate	Rinm5f Mean Log2 Intensity 1500 µM propionate /300 µM butyrate	Rinm5f Mean Log2 Intensity 500 µM propionate/ 500 µM butyrate
1376901_a_at	Elfn2	0	0	0	0	0	0	3.03	3.10	3.10	3.05	3.09	1	0	0	0	0	1	1	7.94	8.08	8.14	8.18	8.38	8.71	8.43
1376909_at	Rasl10a	0	0	0	0	0	0	3.59	3.47	3.59	3.56	3.57	1	0	0	0	0	1	1	7.26	7.26	7.27	7.29	7.24	6.65	6.24
1376911_at	Atp2b4	0	0	0	0	0	0	6.26	6.57	6.72	6.39	6.55	1	0	0	0	0	0	1	8.10	8.13	8.24	8.15	8.27	8.55	9.02
1376912_at	Zswim7	0	0	0	0	0	0	3.64	3.87	3.83	3.89	3.48	1	0	0	0	0	1	1	4.95	4.99	5.16	5.20	5.27	6.22	6.77
1376914_at	Depdc1	0	0	0	0	0	0	3.59	3.56	4.08	3.82	4.02	1	0	0	0	0	1	1	8.98	9.04	8.94	8.91	8.78	7.30	7.21
1376916_at	Zbtb2	0	0	0	0	0	0	5.85	5.80	5.53	5.86	5.67	1	0	0	0	0	1	1	7.15	7.20	7.20	7.21	7.09	6.24	6.13
1376933_at	Pgm2l1	0	0	0	0	0	0	4.70	4.45	5.04	4.88	4.73	1	0	0	1	1	1	1	7.26	7.33	7.62	7.89	7.95	8.40	8.73
1376941_at	Rcc1l	0	0	0	0	0	0	5.21	5.29	5.12	5.49	5.05	1	0	0	0	0	0	1	7.44	7.48	7.52	7.40	7.36	6.94	6.49
1376942_at	---	0	0	0	0	0	0	4.04	4.03	4.24	3.92	3.97	1	0	0	0	0	1	1	5.81	5.59	5.65	5.81	5.56	5.00	4.60
1376951_at	Mad2l1	0	0	0	0	0	0	4.31	4.38	4.36	4.48	4.66	1	0	0	0	0	1	1	8.45	8.35	8.41	8.40	8.24	7.51	7.33
1376969_at	Slc23a2	0	0	0	0	0	0	6.46	6.30	6.76	6.65	6.71	1	0	0	0	0	1	0	8.16	8.22	8.36	8.51	8.55	8.75	9.01
1376973_at	Sdcbp2	0	0	0	0	0	0	4.94	4.95	4.78	4.96	4.84	1	0	0	0	0	1	1	7.84	7.86	7.92	8.14	7.90	7.20	6.30
1376989_at	Pik3r3	0	0	0	0	0	0	6.10	5.94	6.07	5.97	6.29	1	0	0	0	0	1	1	5.92	5.81	6.13	6.14	6.14	7.04	8.03
1376997_at	Fzd4	0	0	0	0	0	0	5.09	4.86	4.77	5.06	4.69	1	0	0	0	0	0	1	4.30	4.33	4.27	4.30	4.30	4.62	5.20
1377013_at	---	0	0	0	0	0	0	8.39	8.33	8.81	8.18	8.48	1	0	0	1	1	1	0	9.07	9.13	9.47	9.75	9.85	9.79	9.30
1377022_at	Abhd10	0	0	0	0	0	0	5.67	5.68	6.13	5.73	5.83	1	0	0	0	0	1	1	8.58	8.53	8.58	8.53	8.44	7.93	7.55
1377037_at	Acot4	0	0	0	0	0	0	3.90	3.73	3.70	3.85	3.58	1	0	0	0	0	0	1	4.15	4.04	4.22	4.23	4.30	4.72	4.97
1377043_at	Cyrib	0	0	0	0	0	0	4.86	4.87	5.05	4.87	4.85	1	0	0	0	0	1	1	8.80	8.90	8.95	9.08	9.14	9.45	9.28
1377049_at	Pnpla7	0	0	0	0	0	0	6.12	6.04	5.97	6.53	5.81	1	0	0	0	0	0	1	5.24	5.25	5.33	5.27	5.34	5.81	5.86
1377051_at	LOC100362572 /	0	0	0	0	0	0	6.82	6.85	7.28	6.88	7.09	1	0	0	0	0	1	1	6.43	6.51	6.63	6.69	6.69	7.21	7.49
1377058_at	Slc37a2	0	0	0	0	0	0	3.88	3.77	3.61	3.79	3.78	1	0	0	0	0	0	1	4.12	4.14	3.88	3.91	4.00	4.36	4.83
1377059_at	Mapk10	0	0	0	0	0	0	4.33	4.57	4.47	4.25	4.80	1	0	0	0	0	0	1	6.97	7.01	6.98	6.77	6.80	6.42	5.62
1377067_at	Fam178b	0	0	0	0	0	0	7.03	6.92	6.85	6.82	6.85	1	0	0	0	0	1	0	7.09	7.14	7.20	7.17	7.28	7.73	7.52
1377088_at	Slc52a3	0	0	0	0	0	0	3.46	3.55	3.51	3.64	3.37	1	0	0	0	1	1	0	7.48	7.58	7.87	8.00	8.10	8.35	8.06
1377090_at	Dock6	0	0	0	0	0	0	5.47	5.31	5.49	5.16	5.14	1	0	0	0	1	1	1	7.40	7.60	7.72	7.82	8.04	8.41	8.41
1377093_at	Brpf3	0	0	0	0	0	0	6.25	6.02	6.42	6.22	6.17	1	0	0	0	0	0	1	7.31	7.21	7.27	7.22	7.06	6.77	6.34
1377103_at	Midn	0	0	0	0	0	0	8.52	8.67	8.47	8.78	8.54	1	0	0	0	0	0	1	10.74	10.77	10.69	10.61	10.60	10.26	9.78
1377110_at	Arl5c	0	0	0	0	0	0	2.98	3.04	2.89	2.95	3.12	1	0	0	0	0	1	1	5.57	5.73	5.93	5.87	6.10	6.85	6.96
1377112_at	Cda	0	0	0	0	0	0	5.18	5.19	5.01	5.13	5.12	1	0	0	0	0	1	1	6.66	6.78	6.69	6.71	6.82	7.42	7.71
1377116_at	Rnasel	0	0	0	0	0	0	6.00	6.12	6.27	6.14	6.18	1	0	0	0	0	0	1	6.28	6.31	6.39	6.40	6.35	6.69	7.14
1377142_at	Trpm3	0	0	0	0	0	0	3.55	3.52	3.83	3.67	3.70	1	0	0	1	0	0	1	5.18	5.23	5.50	5.79	5.64	5.75	6.26
1377145_at	Adgrv1	0	0	0	0	0	0	5.48	5.40	5.15	5.45	5.38	1	0	0	0	0	1	1	4.51	4.43	4.52	4.52	4.48	5.09	5.48
1377163_at	Inhbb	0	0	0	0	0	0	3.89	3.91	3.85	3.90	4.06	1	0	0	0	0	0	1	6.82	6.87	7.02	7.03	6.98	6.51	6.02
1377171_at	Lzts1	0	0	0	0	0	0	5.62	5.65	5.63	5.62	5.56	1	0	0	0	0	1	1	6.95	6.96	7.08	7.13	7.11	7.59	7.94
1377173_at	---	0	0	0	0	0	0	7.02	7.09	6.61	6.85	6.76	1	0	0	0	0	1	1	7.64	7.57	7.71	7.53	7.77	8.30	8.98
1377175_at	---	0	0	0	0	0	0	4.32	4.28	4.38	4.42	4.38	1	0	0	0	0	1	1	6.87	6.81	7.00	6.93	6.73	5.78	5.18
1377180_at	Znf827	0	0	0	0	0	0	5.05	4.88	5.54	5.03	5.22	1	0	0	0	0	1	1	6.40	6.46	6.73	6.63	6.63	5.66	5.20
1377190_at	Ripor2	0	0	0	0	0	0	3.04	3.06	3.18	3.01	3.44	1	0	0	0	0	1	1	3.65	3.71	3.89	3.89	3.97	5.84	5.58
1377197_at	---	0	0	0	0	0	0	4.33	4.42	4.67	4.31	4.49	1	0	0	0	0	1	1	5.29	5.14	5.33	5.50	5.62	6.05	6.02
1377234_at	---	0	0	0	0	0	0	4.40	4.50	4.33	4.51	4.47	1	0	0	0	0	0	1	6.38	6.38	6.49	6.47	6.33	5.93	5.43
1377240_at	---	0	0	0	0	0	0	4.70	4.70	4.86	4.83	4.40	1	0	0	1	1	1	1	6.75	7.00	7.21	7.35	7.48	8.04	8.18
1377245_a_at	Cep295	0	0	0	0	0	0	3.54	3.53	3.64	3.56	3.61	1	0	0	0	0	1	0	7.32	7.29	7.34	7.33	7.38	6.71	6.29
1377299_at	Nasp	0	0	0	0	0	0	6.54	6.34	6.75	6.25	6.55	1	0	0	0	0	1	1	10.50	10.45	10.48	10.52	10.39	9.86	9.49
1377301_at	---	0	0	0	0	0	0	6.66	6.54	6.59	6.62	6.86	1	0	0	0	0	1	0	7.54	7.69	7.78	7.74	7.94	8.17	8.09
1377302_a_at	Mmaa	0	0	0	0	0	0	5.06	4.95	5.06	5.06	4.85	1	0	0	0	0	0	1	5.15	5.38	5.37	5.29	5.35	5.53	6.00
1377308_a_at	Ntrk3	0	0	0	0	0	0	3.60	3.87	3.70	3.77	3.54	1	0	0	0	0	1	1	4.65	4.65	4.91	4.85	4.95	5.59	5.68
1377325_a_at	Pgm2l1	0	0	0	0	0	0	5.87	5.84	6.26	6.31	6.00	1	0	0	0	1	1	1	8.65	8.81	8.97	9.23	9.28	9.74	9.82
1377328_at	Mitf	0	0	0	0	0	0	4.70	4.94	4.82	4.80	5.07	1	0	0	0	0	1	1	7.00	7.07	7.14	7.10	7.35	7.95	7.95
1377336_at	Sema3b	0	0	0	0	0	0	3.62	3.72	3.53	3.64	3.75	1	0	0	0	0	0	1	3.97	3.99	3.97	4.02	4.08	4.38	4.69
1377341_at	Map11	0	0	0	0	0	0	6.12	6.05	5.95	6.10	6.03	1	0	0	0	0	0	1	8.77	8.79	8.80	8.73	8.67	8.29	7.85
1377346_at	Ccdc149	0	0	0	0	0	0	7.37	7.38	7.25	7.33	7.24	1	0	0	0	0	1	1	7.64	7.62	7.63	7.65	7.48	6.57	6.53
1377350_at	Nsl1	0	0	0	0	0	0	5.35	5.42	5.32	5.70	5.32	1	0	0	0	0	0	1	7.38	7.22	7.43	7.32	7.19	6.80	6.83
1377361_at	---	0	0	0	0	0	0	3.68	3.97	3.78	3.72	3.61	1	0	0	0	0	1	0	4.86	5.03	5.08	5.04	5.28	5.77	3.55
1377368_at	NCAM1	0	0	0	0	0	0	4.20	4.32	4.12	4.37	4.49	1	0	0	0	0	0	1	5.59	5.65	5.55	5.46	5.55	5.82	6.11
1377387_a_at	Eef1akmt4	0	0	0	0	0	0	4.82	4.89	4.68	5.10	4.75	1	0	0	0	0	1	1	5.30	5.38	5.37	5.44	5.48	6.07	6.33
1377394_at	---	0	0	0	0	0	0	6.21	6.26	6.47	6.08	6.38	1	0	0	1	1	1	1	6.21	6.33	6.62	6.84	6.96	7.35	7.20
1377404_at	Stc1	0	0	0	0	0	0	6.24	6.13	6.02	6.05	6.00	1	0	0	0	0	1	0	12.05	12.13	12.23	12.27	12.51	12.74	12.61
1377405_at	---	0	0	0	0	0	0	3.66	3.57	3.76	3.79	3.71	1	0	0	0	0	0	1	6.47	6.29	6.67	6.65	6.51	6.84	7.30
1377410_at	E2f8	0	0	0	0	0	0	4.06	4.11	4.00	4.39	4.14	1	0	0	0	0	1	0	7.99	7.85	7.99	8.00	7.83	7.40	7.52
1377456_at	---	0	0	0	0	0	0	6.44	6.57	6.76	6.39	6.83	1	0	0	0	1	1	1	7.92	8.07	8.22	8.44	8.64	9.22	9.16
1377462_at	---	0	0	0	0	0	0	3.07	3.08	3.01	3.04	3.13	1	0	0	0	0	1	1	5.44	5.56	5.70	5.63	5.77	6.04	5.81
1377469_at	---	0	0	0	0	0	0	7.01	6.91	7.54	6.89	6.89	1	0	0	0	0	1	1	8.20	8.14	8.37	8.43	8.43	9.13	9.04
1377472_at	Extl1	0	0	0	0	0	0	4.79	4.88	4.54	5.05	4.65	1	0	0	0	0	1	1	6.13	6.12	6.06	6.03	6.24	6.84	6.72
1377473_at	---	0	0	0	0	0	0	5.83	5.99	5.95	5.86	6.04	1	0	0	0	0	0	1	8.27	8.34	8.25	8.30	8.28	8.00	7.39
1377493_at	Mto1	0	0	0	0	0	0	6.39	6.63	6.40	6.61	6.44	1	0	0	0	0	1	1	6.95	6.79	7.10	7.03	6.83	6.36	6.32
1377556_at	---	0	0	0	0	0	0	6.87	7.13	7.32	6.98	6.89	1	0	0	0	0	1	1	7.97	7.89	8.04	8.17	8.14	8.88	9.20
1377569_at	Sav1	0	0	0	0	0	0	4.14	4.28	3.83	4.29	3.88	1	0	0	0	0	1	0	5.01	4.88	4.82	4.57	4.57	4.41	4.13
1377596_a_at	Med30	0	0	0	0	0	0	5.98	5.63	6.24	5.95	5.93	1	0	0	0	0	1	1	9.41	9.47	9.43	9.44	9.39	8.71	7.99
1377598_at	Coq2	0	0	0	0	0	0	5.99	6.06	6.25	6.18	6.13	1	0	0	0	0	1	0	7.43	7.60	7.70	7.84	7.83	8.13	7.67
1377627_at	Utp20	0	0	0	0	0	0	5.47	5.30	5.39	5.40	5.53	1	0	0	0	0	1	1	8.28	8.25	8.45	8.43	8.30	7.53	7.11
1377642_at	Cav2	0	0	0	0	0	0	5.38	5.15	5.15	5.05	4.96	1	0	0	0	0	1	1	3.67	3.66	3.87	3.82	3.96	4.48	4.93
1377654_at	Fam3a	0	0	0	0	0	0	8.23	8.28	8.52	8.37	8.37	1	0	0	0	0	0	1	9.53	9.52	9.57	9.51	9.54	9.12	8.75
1377676_at	Nucks1	0	0	0	0	0	0	7.98	7.71	8.10	8.11	7.81	1	0	0	0	0	1	1	11.15	11.12	11.18	11.16	11.04	10.49	10.26
1377688_at	---	0	0	0	0	0	0	7.49	7.23	7.19	7.44	7.32	1	0	0	0	0	1	1	7.75	7.81	7.93	8.07	8.08	8.54	8.29
1377698_at	Cd40	0	0	0	0	0	0	5.42	5.49	5.13	5.25	5.44	1	0	0	0	0	1	1	7.60	7.69	7.75	7.71	7.81	8.83	9.07
1377702_at	Lpar6	0	0	0	0	0	0	7.03	7.18	7.41	7.14	7.10	1	0	0	0	0	0	1	6.58	6.55	6.73	6.72	6.88	6.14	5.04
1377714_at	Nectin3	0	0	0	0	0	0	4.75	4.74	4.89	4.54	4.71	1	0	0	0	0	1	0	6.25	5.93	6.09	5.80	5.71	5.56	5.07
1377718_at	Fancb	0	0	0	0	0	0	3.89	3.87	3.98	3.88	4.05	1	0	0	0	0	1	1	6.30	6.11	6.44	6.56	6.25	5.67	5.39
1377729_at	Elovl4	0	0	0	0	0	0	3.90	3.83	4.34	3.96	4.10	1	0	0	0	0	1	1	8.28	8.41	8.57	8.72	8.69	9.10	9.14
1377736_at	LOC100912084	0	0	0	0	0	0	4.50	4.44	4.52	4.42	4.42	1	0	0	0	0	1	1	5.81	5.72	6.01	6.06	6.23	6.74	6.74
1377744_at	Pstpip2	0	0	0	0	0	0	3.95	3.98	4.38	3.87	4.10	1	0	0	0	0	1	1	6.18	6.21	6.35	6.32	6.42	6.82	6.98
1377751_at	Reps2	0	0	0	0	0	0	7.08	7.08	7.60	6.92	7.23	1	0	0	0	0	1	0	8.01	8.18	8.23	8.41	8.45	8.66	8.03
1377752_at	Emp2	0	0	0	0	0	0	3.59	3.91	3.62	3.67	3.64	1	0	0	0	0	1	1	4.63	4.35	4.53	4.43	4.38	5.29	5.02
1377768_at	Kctd1	0	0	0	0	0	0	5.98	5.89	5.73	5.86	5.86	1	0	0	0	0	0	1	7.90	7.88	7.92	7.92	7.86	7.33	7.02
1377772_at	Tmeff1	0	0	0	0	0	0	2.80	2.85	2.77	2.85	2.79	1	0	0	0	1	1	0	6.69	7.00	7.07	7.10	7.33	7.33	7.09
1377795_at	Pcdh7	0	0	0	0	0	0	3.00	3.10	2.94	3.06	3.11	1	0	0	0	1	0	0	4.79	4.53	4.72	4.63	4.15	4.80	5.18
1377797_at	Emg1	0	0	0	0	0	0	7.12	7.08	7.25	7.46	7.02	1	0	0	0	0	1	1	8.73	8.49	8.57	8.60	8.47	8.01	7.46
1377802_at	Kat6b	0	0	0	0	0	0	7.26	7.15	6.95	7.48	7.19	1	0	0	0	0	1	1	6.55	6.39	6.62	6.42	6.18	5.65	6.37
1377813_at	Col22a1	0	0	0	0	0	0	3.91	3.76	3.68	3.72	3.73	1	0	0	0	0	1	1	5.67	5.62	5.70	5.84	5.91	6.58	6.67
1377821_at	Erbb3	0	0	0	0	0	0	5.22	5.08	5.21	5.57	4.99	1	0	0	0	0	1	1	4.04	4.12	4.31	4.15	4.34	5.95	6.70
1377832_at	Plk4	0	0	0	0	0	0	5.07	4.85	5.50	5.07	5.35	1	0	0	0	0	1	1	8.82	8.81	8.85	8.89	8.82	8.23	8.10
1377834_at	Epc1	0	0	0	0	0	0	6.56	6.37	6.77	6.61	6.37	1	0	0	0	0	1	1	7.12	7.06	7.05	7.03	6.91	6.25	6.08
1377848_at	Abraxas1	0	0	0	0	0	0	5.01	4.96	5.46	4.98	4.95	1	0	0	0	0	1	1	7.81	7.84	7.91	7.96	8.03	8.60	8.46
1377850_at	Znrf2	0	0	0	0	0	0	5.53	5.52	6.06	5.83	5.32	1	0	0	0	0	1	1	7.81	7.80	7.91	7.85	7.93	8.45	9.11
1377854_at	Tmem62	0	0	0	0	0	0	6.97	7.02	7.40	7.03	7.33	1	0	0	0	0	1	1	9.14	9.12	9.21	9.28	9.14	8.39	7.99
1377877_at	Ninl	0	0	0	0	0	0	3.82	3.75	3.81	3.96	3.74	1	0	0	0	0	1	1	9.11	9.29	9.13	8.81	9.09	8.51	7.67
1377890_at	LOC689574	0	0	0	0	0	0	5.41	5.42	5.95	5.47	5.46	1	0	0	0	1	1	1	7.07	7.09	7.36	7.61	7.66	8.57	8.43
1377921_at	Etnk2	0	0	0	0	0	0	4.73	4.63	4.49	4.70	4.53	1	0	0	0	0	1	1	6.41	6.39	6.47	6.33	6.48	7.05	7.37
1377929_at	Cacna1e	0	0	0	0	0	0	3.36	3.49	3.27	3.33	3.36	1	0	0	0	0	0	1	5.43	5.48	5.61	5.62	5.59	5.91	6.09
1377939_at	---	0	0	0	0	0	0	6.67	6.59	7.22	6.76	6.91	1	0	0	0	0	0	1	8.66	8.66	8.76	8.78	8.91	9.16	8.78
1377959_at	Fam32a	0	0	0	0	0	0	6.63	6.50	6.74	6.48	6.45	1	0	0	0	0	0	1	9.29	9.32	9.27	9.23	9.12	8.84	8.34
1377994_at	Pmaip1	0	0	0	0	0	0	3.04	2.99	2.89	2.97	2.94	1	0	0	0	0	1	1	5.02	5.12	5.31	5.46	5.39	6.31	7.02
1378010_at	---	0	0	0	0	0	0	7.38	7.30	7.77	7.26	7.60	1	0	0	0	0	1	0	7.72	7.83	8.00	8.24	8.24	8.32	8.23
1378028_at	Mad2l1	0	0	0	0	0	0	4.51	4.53	4.56	4.40	4.41	1	0	0	0	0	1	1	8.41	8.34	8.41	8.34	8.11	7.41	6.99
1378042_at	Tmem212	0	0	0	0	0	0	2.96	2.94	2.80	2.81	2.84	1	0	0	0	1	1	0	6.68	6.92	7.01	7.07	7.53	7.83	6.73
1378045_at	C1ql1	0	0	0	0	0	0	4.18	4.23	4.15	4.23	4.22	1	0	0	0	0	0	1	4.69	4.52	4.51	4.23	4.46	5.24	5.45
1378046_at	Fcsk	0	0	0	0	0	0	5.97	5.77	5.84	5.82	5.94	1	0	0	0	0	1	0	6.32	6.30	6.57	6.56	6.64	6.97	6.87
1378070_at	---	0	0	0	0	0	0	6.33	6.25	6.59	6.19	6.44	1	0	0	0	0	1	1	7.70	7.64	7.66	7.63	7.47	6.94	6.50
1378075_at	Tm9sf4	0	0	0	0	0	0	8.19	8.07	8.04	8.23	7.86	1	0	0	0	0	0	1	10.22	10.23	10.17	10.11	10.07	9.80	9.10
1378076_at	---	0	0	0	0	0	0	5.14	4.90	5.00	4.98	4.75	1	0	0	0	0	1	1	8.06	7.93	8.10	8.06	7.88	6.93	6.58
1378079_at	Golga3	0	0	0	0	0	0	6.55	6.52	6.43	6.45	6.49	1	0	0	0	0	1	1	7.46	7.43	7.48	7.37	7.28	6.59	6.51
1378081_at	Tsc22d1	0	0	0	0	0	0	5.36	5.40	5.78	5.62	5.66	1	0	0	0	0	1	1	6.62	6.61	6.69	6.62	6.56	5.96	5.59
1378083_at	---	0	0	0	0	0	0	4.06	4.27	4.06	4.15	4.05	1	0	0	0	0	1	1	6.37	6.37	6.35	6.33	6.41	7.08	6.83
1378084_at	---	0	0	0	0	0	0	5.16	5.26	5.47	4.95	5.18	1	0	0	0	0	1	0	6.20	6.38	6.46	6.39	6.57	6.84	6.65
1378101_at	Ppp1r17	0	0	0	0	0	0	4.21	4.20	3.96	4.02	4.08	1	0	0	0	1	1	1	5.66	5.97	6.06	6.17	6.37	7.34	8.04
1378105_at	---	0	0	0	0	0	0	3.51	3.48	3.62	3.52	3.58	1	0	0	0	0	0	1	6.87	6.86	7.02	7.09	7.14	7.38	7.48
1378106_at	Phlda2	0	0	0	0	0	0	3.50	3.37	3.32	3.47	3.32	1	0	0	0	0	1	1	4.52	4.48	4.48	4.69	4.67	5.53	5.66
1378116_at	Zbtb45	0	0	0	0	0	0	4.85	4.90	4.78	5.18	4.94	1	0	0	0	0	1	1	6.80	6.56	6.82	6.73	6.61	5.92	5.95
1378118_at	---	0	0	0	0	0	0	4.76	4.54	4.87	4.95	4.74	1	0	0	0	1	0	0	5.41	5.63	5.88	5.93	6.03	5.86	5.64
1378120_at	Mrto4	0	0	0	0	0	0	3.71	3.60	3.73	3.75	3.74	1	0	0	0	0	1	1	6.97	6.95	7.07	7.11	6.98	6.32	6.04
1378128_at	GRHL1	0	0	0	0	0	0	5.72	5.72	5.67	5.57	5.58	1	0	0	0	0	1	1	7.36	7.38	7.56	7.61	7.63	8.49	9.33
1378131_at	Slc9a9	0	0	0	0	0	0	5.72	5.54	5.86	5.66	5.85	1	0	0	0	0	1	1	4.49	4.38	4.49	4.55	4.70	5.35	5.36
1378143_at	LOC690155	0	0	0	0	0	0	4.53	4.34	4.72	4.47	4.63	1	0	0	0	0	1	0	6.72	6.88	6.94	7.06	7.14	7.34	6.83
1378146_at	---	0	0	0	0	0	0	4.56	4.59	4.62	4.82	4.64	1	0	0	0	0	0	1	6.03	6.21	6.19	6.22	6.38	6.56	6.75
1378152_at	Cdkl5	0	0	0	0	0	0	5.42	5.44	5.40	5.55	5.48	1	0	0	0	0	1	1	5.54	5.49	5.55	5.85	5.63	6.17	6.60
1378154_at	---	0	0	0	0	0	0	4.47	4.58	4.34	4.39	4.41	1	0	0	0	0	1	1	6.32	6.39	6.40	6.63	6.74	7.67	7.78
1378155_at	Prrc2c	0	0	0	0	0	0	7.41	7.47	7.77	7.38	7.58	1	0	0	0	0	1	0	9.71	9.62	9.80	9.72	9.67	9.09	8.89
1378156_at	Cys1	0	0	0	0	0	0	4.33	4.49	4.49	4.49	4.41	1	0	0	0	0	1	1	4.73	4.62	4.68	4.59	4.73	5.87	6.37
1378168_at	Rflnb	0	0	0	0	0	0	6.68	6.43	6.47	6.68	6.44	1	0	0	0	0	1	1	7.34	7.43	7.45	7.40	7.45	8.05	7.91
1378173_at	Onecut2	0	0	0	0	0	0	3.04	3.36	3.07	3.32	3.23	1	0	0	0	0	1	1	7.27	7.39	7.57	7.73	7.81	8.64	8.94
1378197_at	Kifc2	0	0	0	0	0	0	6.13	6.23	5.93	6.29	6.23	1	0	0	0	0	1	1	7.94	8.11	8.17	8.30	8.50	8.84	8.08
1378240_at	Fancd2	0	0	0	0	0	0	4.89	4.98	4.86	4.89	4.89	1	0	0	0	0	1	1	7.16	7.13	7.19	7.13	7.08	6.30	6.37
1378243_at	Tmem17	0	0	0	0	0	0	5.87	5.81	6.07	6.10	6.28	1	0	0	0	0	0	1	9.27	9.33	9.34	9.32	9.25	8.78	8.16
1378245_at	Fam81a	0	0	0	0	0	0	4.05	4.01	3.72	3.89	3.92	1	0	0	0	0	1	1	6.16	6.19	6.32	6.24	6.21	6.83	7.39
1378247_at	Eaf2	0	0	0	0	0	0	2.89	2.78	2.71	2.92	2.77	1	0	0	0	0	1	1	3.69	3.64	3.69	3.73	3.66	4.32	5.21

Probeset ID	Gene Symbol	Islet Data Set (log2 ratio ≥0.263; FDR <20%) union n=5325 1=YES 0=NO	Islet DRlyp/lyp ND vs ND+Lp299v (log2 ratio ≥0.263; FDR<20%) n=429 1=YES 0=NO	Islet DRlyp/lyp ND vs HCD (log2 ratio ≥0.263; FDR<20%) n=2247 1=YES 0=NO	Islet DRlyp/lyp ND vs HCD+Lp299v (log2 ratio ≥0.263; FDR<20%) n=1595 1=YES 0=NO	Islet DRlyp/lyp HCD vs HCD+Lp299v (log2 ratio ≥0.263; FDR<20%) n=3026 1=YES 0=NO	Islet DRlyp/lyp ND vs Flyp/lyp ND (log2 ratio ≥ 0.263; FDR<20%) n=1950 1=YES 0=NO	Islet Mean Log2 Intensity DRlyp/lyp ND	Islet Mean Log2 Intensity DRlyp/lyp ND+Lp299v	Islet Mean Log2 Intensity DRlyp/lyp HCD	Islet Mean Log2 Intensity DRlyp/lyp HCD+Lp299v	Islet Mean Log2 Intensity Flyp40 ND	Rinm5f data union of all conditions n=3705 1=YES 0=NO	Rinm5f 30 µM propionate/6 µM butyrate vs Media (FDR<20%; 1.5 fold) n=3 1=YES 0=NO	Rinm5f 60 µM propionate/12 µM butyrate vs Media (FDR<20%; 1.5 fold) n=18 1=YES 0=NO	Rinm5f 120 µM propionate/24 µM butyrate vs Media (FDR<20%; 1.5 fold) n=164 1=YES 0=NO	Rinm5f 240 µM propionate /48 µM butyrate vs Media (FDR<20%; 1.5 fold) n=268 1=YES 0=NO	Rinm5f 1500 µM propionate/300 µM butyrate vs Media (FDR<20%; 1.5 fold) n=2491 1=YES 0=NO	Rinm5f 500 µM propionate/500 µM butyrate vs Media (FDR<20%; 1.5 fold) n=3001 1=YES 0=NO	Rinm5f Mean Log2 Intensity Media only	Rinm5f Mean Log2 Intensity 30 µM propionate/6 µM butyrate	Rinm5f Mean Log2 Intensity 60 µM propionate/12 µM butyrate	Rinm5f Mean Log2 Intensity 120 µM propionate/24 µM butyrate	Rinm5f Mean Log2 Intensity 240 µM propionate/48 µM butyrate	Rinm5f Mean Log2 Intensity 1500 µM propionate /300 µM butyrate	Rinm5f Mean Log2 Intensity 500 µM propionate/500 µM butyrate
1378257_at	Trex1	0	0	0	0	0	0	5.84	6.19	6.06	5.97	6.17	1	0	0	0	0	0	1	7.38	7.41	7.38	7.35	7.20	7.07	6.61
1378275_at	Upf3a	0	0	0	0	0	0	7.38	7.45	7.36	7.54	7.29	1	0	0	0	0	1	1	10.21	10.11	10.20	10.10	10.01	9.58	9.38
1378284_at	Agpat5	0	0	0	0	0	0	6.13	6.00	5.93	6.12	5.84	1	0	0	0	0	1	1	6.98	7.01	7.15	7.41	7.39	7.63	7.94
1378291_at	---	0	0	0	0	0	0	6.35	6.19	6.81	6.28	6.29	1	0	0	0	1	1	0	7.45	7.68	7.87	8.02	8.10	8.19	7.55
1378295_at	Steap2	0	0	0	0	0	0	4.57	4.64	4.37	4.52	4.51	1	0	0	0	0	0	1	6.68	6.74	6.72	6.72	6.80	6.58	5.66
1378296_at	Ncaph	0	0	0	0	0	0	6.24	6.16	6.03	6.23	6.22	1	0	0	0	0	1	1	9.66	9.67	9.66	9.57	9.46	8.58	8.43
1378304_at	---	0	0	0	0	0	0	5.20	5.28	5.35	5.29	5.33	1	0	0	0	0	1	0	5.94	6.09	6.19	6.43	6.46	6.71	6.34
1378310_at	Nmb	0	0	0	0	0	0	4.45	4.55	4.13	4.21	4.31	1	0	0	0	0	1	1	5.31	5.27	5.36	5.24	5.39	6.41	7.16
1378311_at	Tln1	0	0	0	0	0	0	4.97	5.13	4.77	5.04	5.03	1	0	0	0	0	0	1	7.21	7.19	7.31	7.30	7.17	6.81	6.03
1378312_at	Mfsd9	0	0	0	0	0	0	4.77	4.76	4.53	4.55	4.61	1	0	0	0	0	1	0	6.18	6.38	6.32	6.40	6.33	6.99	6.06
1378314_at	---	0	0	0	0	0	0	5.05	4.94	4.91	4.63	4.78	1	0	0	0	0	1	0	6.63	6.38	6.41	6.08	6.17	5.97	6.30
1378321_at	Rassf4	0	0	0	0	0	0	4.93	4.79	4.68	4.72	4.67	1	0	0	0	0	1	1	7.25	7.31	7.44	7.63	7.73	8.52	8.29
1378334_a_at	---	0	0	0	0	0	0	5.24	5.13	5.26	5.44	5.16	1	0	0	0	0	1	1	3.61	3.66	3.64	3.69	3.80	4.57	5.04
1378339_at	Fam124a	0	0	0	0	0	0	3.72	3.71	3.54	3.63	3.65	1	0	0	0	0	0	1	4.96	5.05	5.20	5.05	5.01	5.33	5.94
1378340_at	LOC685158	0	0	0	0	0	0	4.57	4.56	4.28	4.49	4.41	1	0	0	0	0	1	1	5.67	5.72	5.88	5.90	6.00	6.35	6.35
1378350_at	LOC102547703	0	0	0	0	0	0	4.08	4.23	4.40	3.78	4.42	1	0	0	0	0	1	1	7.03	7.30	7.33	7.14	7.45	7.74	7.65
1378368_at	L3mbtl3	0	0	0	0	0	0	5.30	5.57	5.38	5.70	5.54	1	0	0	0	0	0	1	5.61	5.43	5.57	5.46	5.36	6.16	6.64
1378369_at	Dact1	0	0	0	0	0	0	5.82	6.07	5.89	6.03	5.98	1	0	0	0	0	0	1	7.85	7.75	7.90	7.99	8.00	8.43	8.43
1378389_at	Nfatc1	0	0	0	0	0	0	6.47	6.23	6.45	6.51	6.24	1	0	0	0	0	0	1	7.03	7.07	7.12	7.04	7.06	7.32	7.23
1378414_at	Ccdc9b	0	0	0	0	0	0	5.16	5.11	4.74	5.06	4.99	1	0	0	0	0	0	1	7.56	7.56	7.68	7.67	7.62	7.14	6.59
1378420_at	---	0	0	0	0	0	0	6.58	6.51	6.42	6.93	6.51	1	0	0	0	0	0	1	7.35	7.28	7.40	7.60	7.51	7.93	8.12
1378453_at	Itga1	0	0	0	0	0	0	6.99	6.70	6.97	6.87	6.82	1	0	0	0	0	1	1	5.77	5.86	5.84	5.66	5.92	6.61	7.07
1378462_at	Mtap	0	0	0	0	0	0	4.47	4.48	4.47	4.64	4.62	1	0	0	0	0	1	1	4.88	4.83	5.00	5.03	5.09	6.01	6.43
1378470_at	Paqr5	0	0	0	0	0	0	4.20	4.17	4.06	4.15	4.19	1	0	0	0	0	0	1	5.07	5.11	5.07	4.99	5.04	5.52	6.15
1378471_at	Raver2	0	0	0	0	0	0	3.15	3.09	3.06	3.15	3.09	1	0	0	0	0	0	1	3.76	3.91	3.98	3.98	4.01	4.17	5.03
1378477_a_at	Fhod1	0	0	0	0	0	0	5.41	5.23	5.35	5.30	5.24	1	0	0	0	0	0	1	5.64	5.62	5.56	5.68	5.76	6.09	6.33
1378484_at	Rasl12	0	0	0	0	0	0	5.11	4.89	4.97	4.77	4.96	1	0	0	0	0	1	1	4.27	4.28	4.55	4.37	4.54	5.15	5.57
1378487_at	---	0	0	0	0	0	0	3.60	3.63	3.72	3.68	3.87	1	0	0	0	0	1	1	6.77	6.73	6.91	7.06	6.82	6.07	5.19
1378496_at	C2cd4a	0	0	0	0	0	0	4.68	4.84	4.77	4.65	4.73	1	0	0	0	0	0	1	7.51	7.82	7.70	7.31	7.71	6.93	5.57
1378531_at	---	0	0	0	0	0	0	5.62	5.71	5.95	5.38	5.75	1	0	0	0	0	1	1	4.27	4.36	4.48	4.61	4.72	6.38	6.79
1378543_at	Hnrnpa2b1	0	0	0	0	0	0	6.58	6.46	7.02	6.40	6.98	1	0	0	1	1	1	0	7.99	8.26	8.43	8.64	8.82	8.69	7.83
1378556_at	Enpp1	0	0	0	0	0	0	5.85	5.73	5.80	6.15	5.94	1	0	0	0	0	1	1	8.75	8.67	8.93	9.15	9.30	9.76	9.41
1378557_at	Tafa4	0	0	0	0	0	0	3.12	3.13	3.09	3.12	2.96	1	0	0	0	0	0	1	4.09	3.95	3.98	3.99	3.95	4.59	4.73
1378563_at	Ybey	0	0	0	0	0	0	4.18	4.09	4.20	4.12	4.24	1	0	0	0	0	0	1	6.35	6.30	6.26	6.36	6.18	6.16	5.34
1378569_at	Pcdh12	0	0	0	0	0	0	4.41	4.38	4.32	4.24	4.35	1	0	0	0	0	0	1	3.87	3.91	3.82	3.67	3.93	4.38	4.76
1378616_at	Ccsap	0	0	0	0	0	0	5.21	5.16	5.23	5.04	5.10	1	0	0	0	0	1	1	7.28	7.25	7.33	7.27	7.16	6.59	6.38
1378617_at	Trerf1	0	0	0	0	0	0	3.80	3.74	3.65	3.82	3.82	1	0	0	0	0	1	0	5.47	5.30	5.57	5.32	5.17	4.75	5.15
1378624_at	---	0	0	0	0	0	0	3.38	3.41	3.30	3.28	3.37	1	0	0	0	0	1	1	3.68	3.79	3.75	3.77	3.79	4.58	4.41
1378642_at	Kiz	0	0	0	0	0	0	5.26	5.22	5.35	5.22	5.07	1	0	0	0	0	0	1	8.79	8.85	8.95	9.00	9.08	9.37	9.02
1378669_at	Incenp	0	0	0	0	0	0	3.62	3.55	3.31	3.55	3.46	1	0	0	0	0	1	1	7.71	7.53	7.58	7.46	7.23	6.83	6.71
1378673_at	---	0	0	0	0	0	0	5.68	5.56	5.53	5.76	5.79	1	0	0	0	0	1	1	5.26	5.47	5.47	5.47	5.70	6.21	6.45
1378675_at	Tgm3	0	0	0	0	0	0	6.86	7.05	6.67	6.85	6.75	1	0	0	0	0	0	1	8.26	8.45	8.42	8.42	8.54	8.10	7.27
1378681_at	---	0	0	0	0	0	0	3.47	3.60	3.50	3.50	3.52	1	0	0	0	0	1	0	7.12	7.21	7.37	7.22	7.31	7.75	7.52
1378682_at	---	0	0	0	0	0	0	5.65	5.66	5.65	5.97	5.76	1	0	0	0	0	0	1	6.91	7.04	7.04	6.78	6.76	6.38	5.91
1378687_at	---	0	0	0	0	0	0	3.68	3.88	3.84	3.71	3.86	1	0	0	0	0	1	1	5.87	5.92	5.86	5.83	5.66	5.19	4.02
1378691_at	---	0	0	0	0	0	0	6.08	5.98	6.62	6.18	6.34	1	0	0	0	0	1	1	7.76	7.74	7.75	7.83	7.59	6.90	6.64
1378717_at	---	0	0	0	0	0	0	4.49	4.54	4.64	4.40	4.64	1	0	0	0	0	1	1	6.84	6.94	6.94	7.23	7.32	7.89	7.69
1378732_at	---	0	0	0	0	0	0	3.41	3.14	3.21	3.33	3.12	1	0	0	0	0	1	0	4.39	4.48	4.72	4.95	4.87	5.00	5.44
1378733_at	Strip2	0	0	0	0	0	0	3.05	3.11	3.12	3.11	3.12	1	0	0	0	0	1	1	5.04	4.99	5.09	5.32	5.19	5.70	6.16
1378739_at	Cidec	0	0	0	0	0	0	3.94	4.05	3.75	4.04	4.09	1	0	0	0	0	1	1	6.33	6.46	6.32	6.03	5.95	4.87	4.63
1378761_at	Slc38a9	0	0	0	0	0	0	4.25	4.05	4.34	4.37	4.19	1	0	0	0	0	1	0	5.93	6.09	6.21	6.33	6.46	6.61	6.34
1378763_at	Vstm5	0	0	0	0	0	0	4.43	4.39	4.23	4.46	4.50	1	0	0	0	0	1	1	6.30	6.33	6.29	6.27	6.20	5.65	5.46
1378768_at	---	0	0	0	0	0	0	3.62	3.65	3.52	3.52	3.56	1	0	0	0	0	0	1	4.03	4.05	4.15	4.34	4.09	4.44	4.88
1378783_at	---	0	0	0	0	0	0	6.23	6.45	6.26	6.29	6.33	1	0	0	0	0	0	1	6.78	6.83	7.07	6.94	7.06	7.25	7.23
1378787_a_at	Ess2	0	0	0	0	0	0	3.90	3.80	3.59	3.71	3.59	1	0	0	0	0	1	1	6.25	6.34	6.34	6.28	6.16	5.67	5.50
1378801_at	Gata3	0	0	0	0	0	0	3.46	3.44	3.26	3.58	3.36	1	0	0	1	1	1	1	5.71	5.78	6.07	6.42	6.37	7.21	8.09
1378816_a_at	Osbp	0	0	0	0	0	0	4.09	4.15	4.14	3.91	4.01	1	0	0	0	0	0	1	6.34	6.30	6.43	6.62	6.43	6.30	4.72
1378830_at	Irs4	0	0	0	0	0	0	2.98	2.89	2.91	3.01	2.93	1	0	0	0	0	1	1	4.30	4.39	4.34	4.27	4.20	5.22	4.94
1378833_at	LOC102553043	0	0	0	0	0	0	3.61	3.51	3.50	3.62	3.46	1	0	0	1	1	1	1	7.40	7.43	7.20	6.59	6.63	5.90	5.97
1378859_at	Alx3	0	0	0	0	0	0	3.68	3.75	3.55	3.71	3.67	1	0	0	0	0	1	1	7.60	7.59	7.51	7.57	7.44	6.78	6.36
1378860_at	Dpy19l3	0	0	0	0	0	0	7.62	7.63	7.92	7.64	7.90	1	0	0	0	1	1	1	7.26	7.49	7.54	7.72	7.85	8.33	8.22
1378871_at	Adgrb2	0	0	0	0	0	0	4.23	4.34	4.02	4.27	4.42	1	0	0	0	0	1	1	7.97	8.05	8.10	8.01	8.23	8.65	8.95
1378874_at	Cdk2	0	0	0	0	0	0	4.20	4.07	4.30	4.28	4.07	1	0	0	0	0	1	1	7.47	7.42	7.56	7.68	7.72	8.10	8.02
1378906_at	Ddias	0	0	0	0	0	0	3.07	2.98	3.03	3.22	3.08	1	0	0	0	0	1	1	6.47	6.36	6.56	6.60	6.47	5.65	5.61
1378909_at	---	0	0	0	0	0	0	3.96	4.07	4.19	3.87	4.25	1	0	0	0	0	1	1	4.67	4.63	4.89	5.02	5.07	5.95	7.26
1378924_at	Ints10	0	0	0	0	0	0	3.12	3.06	3.01	2.95	3.01	1	0	0	0	0	1	0	4.77	4.94	5.16	5.27	5.16	5.41	5.11
1378925_at	Crem	0	0	0	0	0	0	5.91	5.95	6.33	6.04	6.20	1	0	0	0	0	0	1	9.40	9.43	9.46	9.48	9.51	8.98	8.15
1378940_at	Shq1	0	0	0	0	0	0	3.31	3.33	3.69	3.40	3.41	1	0	0	0	0	1	1	7.83	8.01	8.10	8.28	8.41	9.17	8.82
1378945_at	---	0	0	0	0	0	0	4.47	4.33	4.51	4.56	4.47	1	0	0	0	0	0	1	7.20	7.34	7.43	7.24	7.23	6.97	6.21
1378965_at	---	0	0	0	0	0	0	5.75	5.72	6.09	6.05	5.69	1	0	0	0	0	1	1	6.81	6.62	6.93	6.91	6.89	7.66	8.17
1378967_at	Hnrnpdl	0	0	0	0	0	0	5.15	5.19	5.05	4.91	4.96	1	0	0	0	0	0	1	7.36	7.40	7.44	7.44	7.39	6.93	6.05
1378986_at	HINT3	0	0	0	0	0	0	4.45	4.70	4.60	4.48	4.59	1	0	0	0	0	1	1	5.23	5.44	5.61	5.79	5.76	6.25	6.16
1379013_at	Chpf2	0	0	0	0	0	0	5.76	5.76	5.63	6.10	5.66	1	0	0	0	0	1	1	8.56	8.53	8.62	8.50	8.42	7.95	7.52
1379028_at	Spag7	0	0	0	0	0	0	8.29	8.32	8.33	8.16	8.19	1	0	0	0	0	1	1	8.57	8.53	8.52	8.37	8.21	7.72	7.22
1379048_at	---	0	0	0	0	0	0	6.56	6.56	6.25	6.53	6.57	1	0	0	0	0	0	1	7.01	7.14	6.99	6.95	6.92	6.78	6.21
1379059_at	Micall2	0	0	0	0	0	0	3.58	3.68	3.50	3.73	3.54	1	0	0	0	0	0	1	7.07	7.14	7.19	7.15	7.32	7.53	8.09
1379064_at	---	0	0	0	0	0	0	5.58	5.47	5.59	5.63	5.55	1	0	0	0	0	0	1	6.88	6.87	6.88	6.67	6.66	6.60	6.26
1379080_at	---	0	0	0	0	0	0	3.35	3.49	3.34	3.39	3.38	1	0	0	0	0	0	1	4.29	4.32	4.44	4.25	4.14	4.66	4.90
1379084_at	Dcaf5	0	0	0	0	0	0	5.48	5.60	5.91	5.84	5.52	1	0	0	0	0	1	0	6.79	6.72	6.78	6.71	6.59	6.19	6.08
1379091_at	Hgsnat	0	0	0	0	0	0	5.83	5.96	5.96	5.98	5.97	1	0	0	0	0	1	0	6.95	6.86	6.92	6.92	7.18	7.55	7.06
1379114_at	---	0	0	0	0	0	0	4.68	4.97	4.75	4.69	4.97	1	0	0	0	0	0	1	4.91	4.97	5.18	5.16	5.34	5.35	5.60
1379118_at	LOC102550246	0	0	0	0	0	0	4.04	4.11	3.88	4.26	4.05	1	0	0	0	0	0	1	4.32	4.39	4.33	4.26	4.30	4.81	4.79
1379130_at	Slc44a1	0	0	0	0	0	0	6.27	6.09	6.55	6.26	6.37	1	0	0	0	0	1	1	6.83	6.95	7.07	7.12	7.19	7.71	7.58
1379162_at	---	0	0	0	0	0	0	5.46	5.58	5.36	5.67	5.41	1	0	0	0	0	1	0	7.13	7.04	7.10	6.94	7.05	6.53	6.11
1379167_at	---	0	0	0	0	0	0	3.67	3.64	3.54	3.84	3.65	1	0	0	0	0	0	1	4.63	4.65	4.68	4.64	4.61	4.99	5.17
1379222_at	Flt3lg	0	0	0	0	0	0	3.24	3.50	3.27	3.26	3.19	1	0	0	0	0	1	0	5.97	5.99	6.05	5.88	5.84	5.37	5.14
1379228_at	Flt3lg	0	0	0	0	0	0	3.73	3.78	3.87	3.87	3.93	1	0	0	0	0	1	1	7.61	7.66	7.65	7.53	7.46	7.00	6.46
1379234_a_at	Cdc45	0	0	0	0	0	0	6.12	6.37	6.12	6.25	6.35	1	0	0	0	0	1	1	8.57	8.47	8.46	8.34	8.25	7.51	7.20
1379235_x_at	Cdc45	0	0	0	0	0	0	6.86	6.86	6.60	6.91	7.06	1	0	0	0	0	1	1	9.31	9.21	9.23	9.14	9.03	8.38	7.86
1379252_at	LOC102556753	0	0	0	0	0	0	7.89	8.22	8.35	8.04	8.17	1	0	0	0	0	1	0	9.92	10.02	10.16	10.30	10.39	10.51	10.12
1379257_at	Epb41l4a	0	0	0	0	0	0	6.87	6.58	6.77	6.68	6.77	1	0	0	0	0	0	1	9.60	9.62	9.69	9.67	9.70	9.26	8.93
1379258_at	Klhl5	0	0	0	0	0	0	7.06	6.84	6.97	6.98	6.94	1	0	0	0	0	1	1	7.76	7.82	7.87	7.83	7.92	8.40	8.40
1379264_at	Znrf2	0	0	0	0	0	0	9.32	9.19	9.32	9.45	8.99	1	0	0	0	0	1	1	10.32	10.41	10.46	10.44	10.58	11.09	11.49
1379269_at	Cd2ap	0	0	0	0	0	0	9.17	8.89	9.17	9.45	8.86	1	0	0	0	0	0	1	10.03	10.07	10.12	10.11	10.10	10.61	11.26
1379270_at	---	0	0	0	0	0	0	5.98	5.92	5.78	5.90	5.83	1	0	0	0	0	1	0	7.31	7.53	7.59	7.53	7.68	7.92	7.04
1379275_at	Snx10	0	0	0	0	0	0	4.27	4.47	4.38	4.33	4.20	1	0	0	0	0	1	0	7.61	7.67	7.86	8.12	8.14	8.20	7.21
1379288_at	Czib	0	0	0	0	0	0	6.24	6.22	6.25	6.12	6.15	1	0	0	0	0	0	1	7.75	7.70	7.78	7.87	7.71	7.49	6.86
1379292_at	Txndc16	0	0	0	0	0	0	6.25	6.30	6.34	6.35	5.97	1	0	0	0	0	1	1	6.61	6.66	6.68	6.79	6.79	7.26	7.11
1379301_at	---	0	0	0	0	0	0	3.95	3.87	4.11	3.93	4.13	1	0	0	0	0	1	1	8.05	8.12	8.10	8.02	7.94	7.03	6.72
1379312_at	Pprc1	0	0	0	0	0	0	6.27	6.12	6.62	6.52	6.27	1	0	0	0	0	0	1	9.49	9.46	9.53	9.55	9.59	9.08	8.39
1379319_at	Faap24	0	0	0	0	0	0	5.19	5.06	5.15	5.06	5.08	1	0	0	0	0	1	1	7.75	7.76	7.70	7.62	7.51	7.10	6.64
1379337_at	Fastkd2	0	0	0	0	0	0	4.40	4.22	4.39	4.30	4.65	1	0	0	0	0	0	1	6.77	6.79	6.91	6.89	6.88	6.35	5.56
1379338_at	Pgm2l1	0	0	0	0	0	0	5.77	5.82	6.14	6.12	5.78	1	0	0	0	0	1	1	8.42	8.47	8.70	8.96	8.96	9.39	9.44
1379339_at	Isoc1	0	0	0	0	0	0	7.16	7.10	7.41	6.88	6.89	1	0	0	0	0	0	1	8.38	8.30	8.43	8.51	8.46	8.85	8.34
1379350_at	Tfcp2	0	0	0	0	0	0	5.95	5.94	6.25	6.10	5.66	1	0	0	0	0	1	0	7.18	7.18	7.41	7.44	7.54	7.80	7.64
1379361_at	Pex11a	0	0	0	0	0	0	6.15	6.06	6.12	6.20	5.83	1	0	0	0	0	1	1	7.49	7.55	7.64	7.68	7.82	8.33	8.07
1379373_at	---	0	0	0	0	0	0	6.40	6.54	6.65	6.38	6.86	1	0	0	0	0	0	1	7.93	7.88	8.02	7.99	8.10	8.34	8.49
1379376_at	Mreg	0	0	0	0	0	0	5.55	5.51	5.07	5.27	5.57	1	0	0	0	0	1	1	6.29	6.12	6.33	6.51	6.44	7.03	7.55
1379385_at	Eipr1	0	0	0	0	0	0	6.21	6.51	6.34	6.49	6.60	1	0	0	0	0	0	1	8.62	8.71	8.68	8.54	8.45	8.16	7.71
1379386_at	Gtse1	0	0	0	0	0	0	6.69	6.71	6.54	6.68	6.79	1	0	0	0	0	1	1	9.29	9.17	9.27	9.13	8.92	8.18	8.06
1379393_at	Vil1	0	0	0	0	0	0	8.03	8.21	8.35	8.19	8.31	1	0	0	0	0	1	0	5.48	5.45	5.50	5.55	5.65	6.07	6.37
1379394_at	---	0	0	0	0	0	0	3.63	3.75	3.53	3.49	3.60	1	0	0	0	0	1	1	5.80	5.63	5.88	5.92	5.60	4.86	4.82
1379411_at	LOC100911353	0	0	0	0	0	0	3.38	3.33	3.36	3.39	3.29	1	0	0	0	0	1	1	7.24	7.22	7.36	7.76	7.70	8.44	8.78
1379435_at	Dguok	0	0	0	0	0	0	3.77	3.64	3.73	3.64	3.66	1	0	0	0	0	1	0	6.75	6.70	6.73	6.58	6.44	6.09	6.10
1379437_at	---	0	0	0	0	0	0	3.32	3.10	2.95	3.05	3.04	1	0	0	0	0	0	1	4.13	4.03	4.29	4.20	4.09	4.34	4.66
1379448_at	Ttk	0	0	0	0	0	0	4.14	4.11	4.38	4.50	4.39	1	0	0	0	0	1	1	7.44	7.38	7.47	7.54	7.31	6.39	6.93
1379451_at	Ssbp2	0	0	0	0	0	0	3.65	3.39	3.89	3.48	3.49	1	0	0	0	0	1	1	4.62	4.75	4.91	4.82	5.17	6.00	5.75
1379458_at	Klhl14	0	0	0	0	0	0	3.08	2.96	2.89	3.01	2.98	1	0	0	1	1	1	1	4.15	4.30	4.55	4.79	4.89	5.83	5.24
1379476_at	Nup35	0	0	0	0	0	0	6.91	6.66	7.16	6.86	6.97	1	0	0	0	0	1	1	8.07	8.12	8.12	8.15	8.00	7.18	6.65
1379479_at	Kif4a	0	0	0	0	0	0	4.93	4.70	4.91	5.08	4.98	1	0	0	0	0	1	1	8.60	8.52	8.62	8.54	8.40	7.47	7.56
1379483_at	---	0	0	0	0	0	0	5.25	5.41	5.16	5.07	5.33	1	0	0	0	0	1	0	10.38	10.25	10.25	9.86	10.05	9.76	9.13
1379487_at	Sptlc2	0	0	0	0	0	0	6.15	6.05	6.31	5.88	6.38	1	0	0	0	0	1	0	7.28	7.18	7.45	7.57	7.59	7.93	8.22
1379509_at	---	0	0	0	0	0	0	4.35	4.56	4.43	4.29	4.48	1	0	0	0	0	1	1	5.34	5.45	5.66	5.69	5.85	6.28	5.88
1379513_at	Tmem30b	0	0	0	0	0	0	7.26	7.01	7.20	7.56	6.88	1	0	0	1	1	1	1	7.01	7.28	7.58	7.74	7.97	8.16	8.07
1379527_at	Epc1	0	0	0	0	0	0	7.08	6.99	7.20	7.04	6.76	1	0	0	0	0	1	1	7.32	7.29	7.28	7.28	7.03	6.38	5.86
1379531_at	---	0	0	0	0	0	0	3.28	3.31	3.27	3.23	3.09	1	0	0	0	0	1	1	6.86	6.78	6.82	6.73	6.44	5.91	5.55
1379536_at	Plekha6	0	0	0	0	0	0	5.62	5.84	5.89	5.96	5.93	1	0	0	0	0	1	1	9.06	8.98	9.00	8.84	8.84	8.46	8.12
1379540_at	Nsd3	0	0	0	0	0	0	6.69	6.62	6.63	6.86	6.79	1	0	0	0	0	0	1	8.15	8.09	8.13	8.05	8.11	8.41	8.78
1379542_at	Snrpf	0	0	0	0	0	0	9.00	8.76	9.14	9.20	8.96	1	0	0	0	0	0	1	11.45	11.44	11.43	11.42	11.41	11.12	10.55
1379575_at	---	0	0	0	0	0	0	6.51	6.61	6.58	6.46	6.42	1	0	0	0	0	1	1	7.97	8.15	8.32	8.25	8.35	8.80	8.86
1379594_at	---	0	0	0	0	0	0	6.03	5.68	6.45	5.76	5.93	1	0	0	0	0	1	1	6.40	6.56	6.68	6.66	6.66	7.05	7.35
1379605_at	---	0	0	0	0	0	0	3.26	3.26	3.09	3.14	3.18	1	0	0	1	1	1	0	5.36	5.51	5.72	6.04	6.12	6.33	5.18
1379610_at	LOC102554796	0	0	0	0	0	0	3.91	3.89	4.13	4.17	4.17	1	0	0	0	0	1	1	5.90	5.83	6.19	6.28	6.40	6.75	6.74
1379623_at	Fra10ac1	0	0	0	0	0	0	6.34	6.20	6.12	6.44	6.43	1	0	0	0	0	1	1	8.94	9.00	9.13	9.26	9.36	9.94	10.06
1379636_at	Rmdn2	0	0	0	0	0	0	4.55	4.51	4.64	4.71	4.95	1	0	0	0	0	1	1	5.69	5.75	5.77	5.69	5.49	4.68	4.23

Probeset ID	Gene Symbol	Islet Data Set (log2 ratio ≥0.263; FDR <20%) union n=5325 1=YES 0=NO	Islet DRlyp/lyp ND vs ND+Lp299v (log2 ratio ≥0.263; FDR<20%) n=429 1=YES 0=NO	Islet DRlyp/lyp ND vs HCD (log2 ratio ≥0.263; FDR<20%) n=2247 1=YES 0=NO	Islet DRlyp/lyp ND vs HCD+Lp299v (log2 ratio ≥0.263; FDR<20%) n=1595 1=YES 0=NO	Islet DRlyp/lyp HCD vs HCD+Lp299v (log2 ratio ≥0.263; FDR<20%) n=3026 1=YES 0=NO	Islet DRlyp/lyp ND vs Flyp/lyp ND (log2 ratio ≥ 0.263; FDR<20%) n=1950 1=YES 0=NO	Islet Mean Log2 Intensity DRlyp/lyp ND	Islet Mean Log2 Intensity DRlyp/lyp ND+Lp299v	Islet Mean Log2 Intensity DRlyp/lyp HCD	Islet Mean Log2 Intensity DRlyp/lyp HCD+Lp299v	Islet Mean Log2 Intensity Flyp40 ND	Rinm5f data union of all conditions n=3705 1=YES 0=NO	Rinm5f 30 µM propionate/ 6 µM butyrate vs Media (FDR<20%; 1.5 fold) n=3 1=YES 0=NO	Rinm5f 60 µM propionate/ 12 µM butyrate vs Media (FDR<20%; 1.5 fold) n=18 1=YES 0=NO	Rinm5f 120 µM propionate/ 24 µM butyrate vs Media (FDR<20%; 1.5 fold) n=164 1=YES 0=NO	Rinm5f 240 µM propionate /48 µM butyrate vs Media (FDR<20%; 1.5 fold) n=268 1=YES 0=NO	Rinm5f 1500 µM propionate/ 300 µM butyrate vs Media (FDR<20%; 1.5 fold) n=2491 1=YES 0=NO	Rinm5f 500 µM propionate/ 500 µM butyrate vs Media (FDR<20%; 1.5 fold) n=3001 1=YES 0=NO	Rinm5f Mean Log2 Intensity Media only	Rinm5f Mean Log2 Intensity 30 µM propionate/ 6 µM butyrate	Rinm5f Mean Log2 Intensity 60 µM propionate/ 12 µM butyrate	Rinm5f Mean Log2 Intensity 120 µM propionate/ 24 µM butyrate	Rinm5f Mean Log2 Intensity 240 µM propionate/ 48 µM butyrate	Rinm5f Mean Log2 Intensity 1500 µM propionate /300 µM butyrate	Rinm5f Mean Log2 Intensity 500 µM propionate/ 500 µM butyrate
1379649_at	Gxylt1	0	0	0	0	0	0	4.69	4.65	5.14	4.48	4.83	1	0	0	0	0	0	1	7.98	7.88	8.06	8.14	8.05	8.44	8.14
1379659_at	Bmp2k	0	0	0	0	0	0	3.33	3.28	3.49	3.42	3.77	1	0	0	0	0	0	1	5.85	5.81	6.02	5.97	6.03	6.25	6.91
1379673_at	Uap1l1	0	0	0	0	0	0	5.20	5.19	5.16	5.10	5.23	1	0	0	0	0	1	0	7.02	6.74	7.02	6.67	6.78	6.43	6.26
1379684_at	---	0	0	0	0	0	0	4.23	4.21	4.05	3.97	4.02	1	0	0	0	0	1	0	7.19	7.34	7.47	7.54	7.54	7.80	7.65
1379694_at	Epc2	0	0	0	0	0	0	4.70	4.71	4.93	4.79	4.69	1	0	0	0	0	1	1	6.00	5.90	6.06	6.15	6.01	5.26	4.89
1379696_at	---	0	0	0	0	0	0	3.22	3.41	3.26	3.29	3.61	1	0	0	0	0	1	0	4.35	4.41	4.53	4.59	4.70	5.19	4.93
1379710_at	Il17rd	0	0	0	0	0	0	4.44	4.34	4.30	4.73	4.31	1	0	0	0	0	1	1	3.95	3.96	4.06	4.00	3.94	4.68	5.32
1379721_at	Swsap1	0	0	0	0	0	0	5.09	5.37	5.18	4.94	5.05	1	0	0	0	0	0	1	7.19	7.18	7.24	7.27	7.10	6.76	6.50
1379725_at	Ptpn9	0	0	0	0	0	0	7.14	6.95	7.24	7.19	7.21	1	0	0	0	0	0	1	7.68	7.71	7.81	7.73	7.78	7.37	7.30
1379792_at	---	0	0	0	0	0	0	3.72	3.76	3.46	3.63	3.71	1	0	0	0	0	1	1	6.02	6.06	6.15	6.15	6.41	6.89	7.47
1379858_at	Mettl2a	0	0	0	0	0	0	6.60	6.52	6.64	6.70	6.66	1	0	0	0	0	0	1	8.35	8.34	8.46	8.40	8.31	7.84	7.30
1379866_at	---	0	0	0	0	0	0	6.09	6.01	6.19	6.04	6.17	1	0	0	0	0	0	1	7.45	7.54	7.67	7.55	7.71	7.84	7.93
1379868_at	Pbxip1	0	0	0	0	0	0	6.27	6.34	6.20	6.19	6.03	1	0	0	0	0	1	1	5.40	5.35	5.45	5.47	5.57	6.73	7.14
1379881_at	---	0	0	0	0	0	0	5.95	5.86	6.27	6.09	6.22	1	0	0	0	1	1	0	6.07	6.36	6.54	6.65	6.78	6.94	6.36
1379894_at	Armh4	0	0	0	0	0	0	3.66	3.62	3.65	3.61	3.66	1	0	0	0	0	1	1	8.26	8.28	8.32	8.26	8.34	9.10	9.39
1379895_at	LOC102554860	0	0	0	0	0	0	3.39	3.55	3.72	3.50	3.67	1	0	0	0	0	0	1	3.86	3.76	3.84	3.91	3.85	4.23	4.48
1379907_at	Hpcal4	0	0	0	0	0	0	3.52	3.38	3.29	3.40	3.34	1	0	0	0	0	0	1	4.48	4.45	4.29	4.33	4.48	4.59	5.73
1379908_at	Asb4	0	0	0	0	0	0	3.11	3.09	2.89	3.07	2.99	1	0	0	0	0	1	0	9.22	9.30	9.36	9.34	9.59	10.18	10.36
1379925_at	---	0	0	0	0	0	0	4.19	4.14	4.18	4.20	4.20	1	0	0	1	1	1	0	7.42	7.51	7.72	8.04	8.16	8.42	7.70
1379939_a_at	C3orf14	0	0	0	0	0	0	6.73	6.70	6.78	6.97	6.75	1	0	0	0	0	0	1	7.88	7.87	7.87	7.79	7.72	7.32	6.89
1379941_at	Vps13c	0	0	0	0	0	0	6.22	6.23	5.95	6.39	6.18	1	0	0	0	0	1	1	7.31	7.47	7.50	7.40	7.50	8.09	8.33
1379972_at	Msl3l2	0	0	0	0	0	0	5.97	6.15	6.26	5.81	6.28	1	0	0	0	0	1	1	7.97	7.97	8.05	8.07	7.90	7.11	6.60
1379990_at	Fam162a	0	0	0	0	0	0	4.46	4.66	4.66	4.59	4.85	1	0	0	0	0	1	0	7.48	7.47	7.50	6.98	7.02	6.68	6.84
1380003_at	Rgs17	0	0	0	0	0	0	4.88	4.91	4.83	4.72	5.04	1	0	0	0	0	0	1	8.03	8.21	8.12	8.16	8.29	7.93	6.61
1380017_at	RBMS3	0	0	0	0	0	0	6.68	6.61	6.68	6.43	6.63	1	0	0	0	0	0	1	7.43	7.65	7.73	7.73	7.77	7.27	6.43
1380022_at	Rrn3	0	0	0	0	0	0	6.52	6.15	6.74	6.10	6.33	1	0	0	0	0	0	1	8.68	8.63	8.77	8.80	8.71	8.38	7.83
1380027_at	Crebbp	0	0	0	0	0	0	6.32	6.60	6.49	6.62	6.51	1	0	0	0	0	1	1	8.24	8.32	8.30	8.20	8.06	7.59	6.90
1380065_at	---	0	0	0	0	0	0	4.56	4.55	4.36	4.47	4.42	1	0	0	0	0	1	1	7.32	7.17	7.18	6.93	6.89	6.15	5.12
1380097_at	---	0	0	0	0	0	0	3.87	3.79	3.86	4.02	3.97	1	0	0	0	0	0	1	5.26	5.17	5.43	5.23	4.90	5.34	4.78
1380137_at	Uros	0	0	0	0	0	0	4.46	4.52	4.83	4.58	4.65	1	0	0	0	0	1	1	4.44	4.45	4.64	4.65	4.75	5.24	5.76
1380217_at	Ndc80	0	0	0	0	0	0	4.73	4.56	4.54	4.78	4.61	1	0	0	0	0	1	1	6.72	6.75	6.99	6.96	6.81	5.79	5.93
1380225_at	---	0	0	0	0	0	0	5.57	5.67	5.53	5.60	5.70	1	0	0	0	0	1	1	6.75	6.73	6.72	6.69	6.66	5.94	5.77
1380226_at	Samd10	0	0	0	0	0	0	4.85	4.93	4.97	5.01	4.82	1	0	0	0	0	1	1	8.24	8.16	8.23	8.15	8.00	7.44	7.02
1380277_at	Rad51ap1	0	0	0	0	0	0	4.07	3.92	3.97	4.10	4.07	1	0	0	0	0	1	1	7.15	7.08	7.15	7.10	7.11	6.33	5.75
1380286_x_at	Cep295	0	0	0	0	0	0	4.04	4.16	4.19	4.09	4.00	1	0	0	0	0	1	0	7.81	7.80	7.79	7.87	7.77	7.18	7.41
1380293_at	LOC361346	0	0	0	0	0	0	3.90	3.76	4.14	3.57	3.77	1	0	0	0	0	0	1	8.80	8.79	8.87	9.08	8.94	8.36	7.75
1380303_at	Haspin	0	0	0	0	0	0	3.99	4.09	3.81	4.14	3.98	1	0	0	0	0	1	1	7.23	7.16	7.21	7.10	7.05	6.41	6.59
1380305_at	Lrrc73	0	0	0	0	0	0	5.70	6.05	5.45	5.75	5.86	1	0	0	0	0	1	1	8.47	8.37	8.35	8.18	8.14	7.28	7.04
1380320_at	Lin54	0	0	0	0	0	0	5.52	5.19	5.87	5.69	5.66	1	0	0	0	0	1	0	8.23	8.12	8.18	8.24	8.10	7.57	7.57
1380338_at	Sh2d5	0	0	0	0	0	0	3.14	3.23	3.04	3.22	3.12	1	0	0	0	0	1	1	5.77	5.86	5.96	5.95	6.12	6.82	7.09
1380355_s_at	Slc6a17	0	0	0	0	0	0	5.01	5.03	5.06	5.00	5.02	1	0	0	0	0	1	0	6.80	6.69	6.72	6.60	6.46	6.13	6.01
1380358_at	---	0	0	0	0	0	0	5.24	5.19	5.62	5.53	5.38	1	0	0	0	0	0	1	8.37	8.41	8.51	8.49	8.46	7.96	7.17
1380365_at	---	0	0	0	0	0	0	3.47	3.75	3.49	3.51	3.66	1	0	0	0	0	1	0	6.72	6.88	6.92	6.88	7.13	7.42	7.77
1380380_at	Ttl	0	0	0	0	0	0	5.53	5.59	5.41	5.59	5.59	1	0	0	0	0	0	1	6.79	6.74	6.90	6.79	6.63	6.29	5.82
1380383_at	Arl4d	0	0	0	0	0	0	6.20	6.33	6.24	5.77	5.93	1	0	0	0	0	1	1	6.75	6.69	6.83	6.74	6.83	7.82	7.77
1380391_at	Srsf7	0	0	0	0	0	0	4.40	4.63	4.60	4.10	4.41	1	0	0	0	0	0	1	7.04	6.96	7.11	7.09	6.96	6.51	5.46
1380393_at	Cryz	0	0	0	0	0	0	4.95	4.86	5.00	5.23	4.62	1	0	0	0	1	1	1	4.81	4.87	5.12	5.06	5.49	6.13	6.40
1380403_at	Rwdd2a	0	0	0	0	0	0	3.08	3.15	3.18	3.25	3.09	1	0	0	0	0	0	1	3.57	3.52	3.72	3.47	3.56	3.80	4.13
1380424_at	Ubxn11	0	0	0	0	0	0	4.77	4.63	4.56	4.78	4.50	1	0	0	0	0	1	1	6.25	6.37	6.61	6.54	6.74	7.13	6.95
1380430_at	---	0	0	0	0	0	0	6.41	6.21	6.60	6.44	6.53	1	0	0	0	0	1	0	7.18	7.12	7.15	7.07	6.96	6.60	6.39
1380443_at	Pwp1	0	0	0	0	0	0	3.65	3.63	3.89	3.74	3.83	1	0	0	0	0	0	1	7.08	7.17	7.34	7.47	7.36	6.77	6.25
1380446_at	Mllt10	0	0	0	0	0	0	6.66	6.64	7.15	7.02	6.97	1	0	0	0	0	1	0	8.52	8.28	8.44	8.32	8.19	7.82	8.18
1380449_at	---	0	0	0	0	0	0	6.84	6.86	7.30	6.93	7.13	1	0	0	0	0	1	1	7.76	7.74	7.80	7.84	7.82	7.16	6.98
1380452_at	Cfdp1	0	0	0	0	0	0	5.83	6.01	5.85	6.09	6.18	1	0	0	1	1	1	0	6.37	6.55	6.83	7.01	7.01	7.41	6.98
1380474_at	Loxl2	0	0	0	0	0	0	5.55	5.62	5.81	5.49	5.90	1	0	0	0	0	1	1	6.12	6.06	6.16	6.12	6.29	5.50	4.70
1380479_at	Wdr35	0	0	0	0	0	0	3.92	3.83	3.87	3.91	3.90	1	0	0	0	1	1	1	5.26	5.42	5.55	5.75	5.87	6.16	6.08
1380517_at	Zmym3	0	0	0	0	0	0	3.56	3.53	3.59	3.45	3.43	1	0	0	0	0	0	1	6.80	6.90	6.91	6.81	6.87	6.21	5.63
1380531_at	Slc9a9	0	0	0	0	0	0	3.77	3.99	3.60	3.86	3.75	1	0	0	0	0	1	1	4.35	4.40	4.49	4.65	4.55	4.99	5.18
1380539_at	Ermp1	0	0	0	0	0	0	4.45	4.32	4.62	4.53	4.43	1	0	0	0	0	0	1	6.87	6.83	6.93	7.02	7.10	7.46	7.77
1380568_a_at	Ddb2	0	0	0	0	0	0	3.85	3.79	3.95	4.08	3.83	1	0	0	0	0	0	1	5.04	5.06	4.95	4.86	4.80	5.16	5.66
1380577_at	Abcg2	0	0	0	0	0	0	4.86	4.84	4.36	4.75	4.54	1	0	0	0	0	0	1	4.20	4.27	4.18	4.10	4.19	4.74	4.87
1380602_at	---	0	0	0	0	0	0	4.63	4.91	4.54	4.72	4.71	1	0	0	0	0	1	1	5.75	5.84	5.91	5.67	5.85	7.09	7.43
1380610_at	---	0	0	0	0	0	0	4.61	4.73	4.76	4.61	4.58	1	0	0	0	0	1	1	5.53	5.56	5.89	5.94	5.92	6.33	6.38
1380627_at	LOC102547503	0	0	0	0	0	0	4.01	4.03	3.99	4.26	3.92	1	0	0	0	0	0	1	5.13	5.10	5.12	4.98	5.02	5.50	5.69
1380662_at	---	0	0	0	0	0	0	5.44	5.45	5.18	5.53	5.26	1	0	0	0	0	1	1	6.74	7.18	7.03	7.06	7.24	7.66	7.91
1380674_at	---	0	0	0	0	0	0	4.56	4.57	4.21	4.48	4.72	1	0	0	0	0	1	1	5.82	5.95	6.01	6.01	6.07	6.47	6.09
1380675_s_at	Ca11	0	0	0	0	0	0	6.05	5.95	5.96	6.03	6.01	1	0	0	0	0	0	1	9.69	9.80	9.83	9.83	9.79	9.19	8.33
1380679_at	---	0	0	0	0	0	0	3.92	3.69	3.66	3.92	3.74	1	0	0	0	0	1	1	5.01	5.11	5.09	5.15	5.16	5.71	6.31
1380688_at	Galnt18	0	0	0	0	0	0	5.20	5.34	5.72	5.37	4.98	1	0	0	0	0	1	1	6.27	6.39	6.48	6.51	6.48	7.71	7.55
1380705_at	Ube3c	0	0	0	0	0	0	6.31	6.36	6.75	6.11	6.36	1	0	0	0	0	1	1	8.44	8.50	8.60	8.72	8.86	9.04	8.83
1380768_at	Raet1d /// Raet1	0	0	0	0	0	0	3.85	3.84	4.14	4.01	3.82	1	0	0	1	1	1	0	6.53	6.75	7.02	7.47	7.70	8.51	8.67
1380773_at	---	0	0	0	0	0	0	4.80	4.85	4.87	4.81	4.76	1	0	0	0	0	1	1	7.17	7.10	7.11	7.10	6.95	6.55	6.11
1380775_at	Kif20b	0	0	0	0	0	0	4.13	4.22	4.62	4.26	4.47	1	0	0	0	0	1	1	9.02	9.01	9.06	9.15	9.03	8.02	8.04
1380777_at	---	0	0	0	0	0	0	7.07	6.65	6.89	7.10	6.77	1	0	0	0	0	0	1	5.99	6.09	6.19	6.14	6.12	6.39	6.62
1380781_at	---	0	0	0	0	0	0	3.95	3.77	3.81	4.03	3.87	1	0	0	0	0	0	1	5.61	5.56	5.70	5.61	5.58	5.21	4.91
1380784_at	Klhl5	0	0	0	0	0	0	3.70	3.61	3.41	3.48	3.43	1	0	0	0	0	1	1	4.20	4.41	4.39	4.36	4.49	4.81	5.07
1380789_at	Pcdhb17	0	0	0	0	0	0	3.60	3.31	3.47	3.33	3.15	1	0	0	0	0	0	1	8.04	8.03	8.15	8.17	8.17	7.61	6.76
1380807_at	---	0	0	0	0	0	0	5.91	6.26	6.01	6.08	5.76	1	0	1	0	1	1	1	6.02	6.21	6.62	6.61	6.70	6.72	6.71
1380816_at	Ccsap	0	0	0	0	0	0	5.62	5.62	5.54	5.51	5.59	1	0	0	0	0	0	1	7.13	7.09	7.17	7.04	6.91	6.72	5.82
1380828_at	Gabra1	0	0	0	0	0	0	3.20	3.12	3.23	3.16	3.10	1	0	0	0	0	0	1	3.99	4.17	4.26	4.07	4.35	4.46	4.84
1380852_at	Ralyl	0	0	0	0	0	0	3.93	4.22	3.98	4.05	3.99	1	0	0	0	0	0	1	5.37	5.41	5.30	5.22	5.10	5.67	5.74
1380875_at	Sfswap	0	0	0	0	0	0	6.52	6.41	6.73	6.43	6.49	1	0	0	0	0	0	1	8.56	8.53	8.63	8.60	8.52	8.18	7.64
1380884_at	Clcn4	0	0	0	0	0	0	2.80	2.78	2.83	2.95	2.87	1	0	0	0	0	1	1	5.14	5.03	5.45	5.48	5.65	6.16	6.21
1380887_at	---	0	0	0	0	0	0	3.44	3.39	3.44	3.33	3.49	1	0	0	0	0	0	1	6.13	6.13	6.30	6.15	6.15	6.59	7.12
1380895_at	Aplp2	0	0	0	0	0	0	6.93	7.11	6.87	7.23	6.70	1	0	0	0	0	0	1	8.74	8.71	8.96	8.87	8.91	9.06	8.91
1380908_at	---	0	0	0	0	0	0	4.88	4.91	4.81	4.96	4.97	1	0	0	0	0	0	1	6.42	6.49	6.75	6.67	6.87	6.04	5.40
1380919_at	---	0	0	0	0	0	0	3.19	3.28	3.21	3.30	3.28	1	0	0	0	0	0	1	4.64	4.41	4.61	4.31	4.33	4.16	3.60
1380924_at	---	0	0	0	0	0	0	3.37	3.15	3.11	3.18	3.21	1	0	0	0	0	0	1	4.49	4.58	4.52	4.54	4.47	4.99	5.59
1380941_at	LOC102549836	0	0	0	0	0	0	4.18	4.12	3.88	4.18	4.02	1	0	0	0	0	0	1	4.94	5.11	5.04	4.88	5.00	5.41	6.22
1380966_at	---	0	0	0	0	0	0	5.08	5.03	4.86	5.33	4.99	1	0	0	0	0	1	0	5.73	5.74	5.67	5.55	5.85	6.36	6.33
1380967_at	Nts	0	0	0	0	0	0	3.77	3.78	3.58	3.90	3.73	1	0	0	0	0	0	1	4.40	4.61	4.59	4.65	4.52	4.60	5.26
1380969_at	---	0	0	0	0	0	0	4.00	4.27	4.00	4.08	3.96	1	0	0	0	0	0	1	5.82	5.95	6.17	6.01	6.06	6.23	6.74
1380989_at	Numa1	0	0	0	0	0	0	4.89	4.87	4.62	5.13	4.55	1	0	0	0	0	1	1	7.75	7.52	7.67	7.47	7.30	6.92	6.41
1381022_at	Cilp2	0	0	0	0	0	0	3.45	3.49	3.24	3.25	3.39	1	0	0	0	0	1	1	5.85	5.89	5.68	5.65	5.78	6.45	6.74
1381027_at	Micu1	0	0	0	0	0	0	3.95	3.94	3.98	3.80	3.94	1	0	1	0	0	1	1	4.53	4.60	5.19	5.06	5.05	5.38	6.00
1381054_at	---	0	0	0	0	0	0	5.08	5.23	4.96	5.03	5.04	1	0	0	0	0	1	1	6.94	7.08	6.92	6.64	6.76	6.32	6.04
1381080_at	Dgkg	0	0	0	0	0	0	3.55	3.68	3.42	3.58	3.37	1	0	0	0	0	0	1	4.31	4.29	4.14	4.04	4.17	4.50	5.06
1381108_at	Kansl2	0	0	0	0	0	0	5.12	5.14	5.42	5.40	5.26	1	0	0	0	0	0	1	7.31	7.39	7.46	7.33	7.27	6.80	6.51
1381122_at	---	0	0	0	0	0	0	4.12	4.16	4.08	4.06	4.05	1	0	0	0	0	1	1	5.79	5.82	5.92	6.02	6.06	6.67	7.05
1381130_at	Mcm8	0	0	0	0	0	0	3.29	3.10	3.22	3.19	3.38	1	0	0	0	0	1	0	6.95	6.97	7.13	7.05	7.01	6.36	5.29
1381158_at	Spdya	0	0	0	0	0	0	2.85	2.83	2.78	2.81	2.86	1	0	0	0	0	1	1	4.33	4.07	4.28	4.36	4.45	5.40	6.01
1381189_at	Fzr1	0	0	0	0	0	0	5.04	5.05	5.12	4.75	5.05	1	0	0	0	0	0	1	8.17	8.20	8.28	8.31	8.18	8.03	7.21
1381207_at	---	0	0	0	0	0	0	5.21	5.46	5.35	5.37	5.26	1	0	0	0	0	0	1	7.35	7.56	7.59	7.50	7.58	7.83	7.67
1381217_at	Trmt6	0	0	0	0	0	0	4.92	5.25	5.03	5.25	5.06	1	0	0	0	0	0	1	7.44	7.48	7.55	7.62	7.56	7.18	6.39
1381244_at	Cux1	0	0	0	0	0	0	5.48	5.45	5.60	5.72	5.55	1	0	0	0	0	1	0	6.67	6.48	6.55	6.38	6.34	6.08	6.51
1381262_at	---	0	0	0	0	0	0	5.29	5.49	5.27	5.48	5.32	1	0	0	0	0	0	1	4.25	4.17	4.23	4.11	4.21	4.63	4.93
1381263_at	---	0	0	0	0	0	0	3.19	3.13	3.14	3.19	3.09	1	0	0	0	0	0	1	5.83	5.88	5.95	5.77	5.85	5.62	4.89
1381284_at	---	0	0	0	0	0	0	6.52	6.90	6.80	6.29	6.80	1	0	0	0	0	1	0	8.45	8.53	8.60	8.41	8.48	7.67	6.88
1381289_at	Haus8	0	0	0	0	0	0	3.59	3.63	3.64	3.54	3.52	1	0	0	0	0	1	0	6.31	6.38	6.37	6.39	6.18	5.71	5.61
1381293_at	---	0	0	0	0	0	0	3.10	3.16	3.18	3.08	3.11	1	0	0	0	0	1	1	5.15	5.13	5.12	4.92	4.95	4.35	3.72
1381298_at	Mcm10	0	0	0	0	0	0	4.71	4.85	4.45	4.77	4.69	1	0	0	0	0	1	1	7.27	7.24	7.23	7.19	7.08	6.47	6.32
1381335_at	---	0	0	0	0	0	0	4.62	4.51	4.37	4.68	4.57	1	0	0	0	0	1	0	5.86	6.05	6.26	6.15	6.34	7.03	6.37
1381349_a_at	Ubr2	0	0	0	0	0	0	6.14	6.15	6.27	6.39	5.97	1	0	0	0	0	1	0	7.83	7.78	7.75	7.54	7.63	7.20	6.98
1381448_at	Nars2	0	0	0	0	0	0	5.63	5.39	5.32	5.47	5.45	1	0	0	0	0	1	1	6.95	7.01	7.27	7.17	7.30	7.58	7.82
1381449_s_at	Tgfa	0	0	0	0	0	0	5.99	6.08	6.30	6.22	5.97	1	0	0	0	0	1	1	9.33	9.29	9.35	9.34	9.54	10.01	9.83
1381463_at	LOC103690511 /	0	0	0	0	0	0	5.80	5.79	5.53	5.89	5.61	1	0	0	0	0	0	1	6.33	6.50	6.65	6.57	6.58	6.01	5.31
1381476_at	Rnf32	0	0	0	0	0	0	3.76	3.78	4.13	3.60	3.92	1	0	0	0	0	1	1	6.36	6.44	6.56	6.64	6.63	7.05	7.14
1381486_at	---	0	0	0	0	0	0	3.56	3.57	3.31	3.36	3.47	1	0	1	1	0	0	0	5.87	6.10	6.46	6.45	6.41	5.63	5.36
1381537_at	Klc3	0	0	0	0	0	0	5.16	5.31	4.80	4.82	4.95	1	0	0	0	0	0	1	7.35	7.31	7.37	7.32	7.44	7.64	7.81
1381542_at	Ubxn4	0	0	0	0	0	0	8.14	8.12	8.33	8.34	8.36	1	0	0	0	0	0	1	9.98	9.93	10.04	10.04	9.96	9.56	8.86
1381583_at	Nlgn1	0	0	0	0	0	0	3.24	3.39	3.40	3.06	3.25	1	0	0	0	0	1	1	4.88	4.80	5.09	5.26	5.24	5.59	5.95
1381610_at	---	0	0	0	0	0	0	3.67	3.63	3.54	3.75	3.71	1	0	0	0	0	0	1	4.27	4.14	4.32	4.22	4.27	4.45	4.81
1381611_at	Ahctf1	0	0	0	0	0	0	5.37	5.32	5.25	5.19	5.27	1	0	0	0	0	1	0	7.02	6.79	6.98	6.91	6.74	6.40	6.36
1381619_at	Zbtb1	0	0	0	0	0	0	3.69	3.74	3.68	3.88	3.76	1	0	0	0	0	0	1	6.94	7.01	7.09	6.89	6.94	6.38	5.76
1381620_at	---	0	0	0	0	0	0	3.06	3.05	3.01	3.24	2.99	1	0	0	1	0	1	1	5.20	5.11	5.65	5.80	5.67	6.64	6.93
1381632_at	---	0	0	0	0	0	0	4.14	4.30	4.23	3.93	4.05	1	0	0	0	0	1	1	4.51	4.69	4.59	4.54	4.58	5.17	5.76
1381637_at	Camk2a	0	0	0	0	0	0	3.59	3.54	3.35	3.64	3.51	1	0	0	0	0	1	1	6.55	6.66	6.85	6.80	6.91	7.41	6.79
1381677_at	Elavl2	0	0	0	0	0	0	2.91	3.07	3.08	3.07	3.01	1	0	0	0	0	1	0	6.57	6.57	6.73	7.03	6.91	7.22	6.16
1381687_at	Mrm3	0	0	0	0	0	0	3.80	3.81	3.67	3.79	3.77	1	0	0	0	0	0	1	6.52	6.46	6.59	6.54	6.46	5.98	5.28
1381719_at	---	0	0	0	0	0	0	4.29	4.28	4.02	4.02	4.27	1	0	0	0	0	0	1	5.27	5.21	5.41	5.38	5.39	5.42	4.96
1381722_at	Plin5	0	0	0	0	0	0	3.51	3.62	3.47	3.63	3.65	1	0	0	0	0	0	1	4.29	4.21	4.21	4.18	4.07	4.69	4.49
1381741_at	Asphd1	0	0	0	0	0	0	4.69	4.80	4.57	4.66	4.53	1	0	0	0	0	1	1	7.35	7.14	7.41	7.18	7.13	6.47	6.50
1381757_at	Wdpcp	0	0	0	0	0	0	7.18	7.34	7.29	6.99	7.58	1	0	0	0	0	1	1	8.41	8.39	8.30	8.13	8.04	7.71	7.64
1381759_at	Crnde	0	0	0	0	0	0	3.21	3.16	3.05	3.21	3.12	1	0	0	0	0	1	0	7.63	7.59	7.35	7.32	7.25	6.90	6.81
1381768_at	Mthfs	0	0	0	0	0	0	5.05	5.06	5.27	4.63	4.86	1	0	0	0	0	0	1	6.89	6.85	7.02	7.01	6.94	6.60	5.52

Probeset ID	Gene Symbol	Islet Data Set (log2 ratio ≥0.263; FDR <20%) union n=5325 1=YES 0=NO	Islet DRlyp/lyp ND vs ND+Lp299v (log2 ratio ≥0.263; FDR<20%) n=429 1=YES 0=NO	Islet DRlyp/lyp ND vs HCD (log2 ratio ≥0.263; FDR<20%) n=2247 1=YES 0=NO	Islet DRlyp/lyp ND vs HCD+Lp299v (log2 ratio ≥0.263; FDR<20%) n=1595 1=YES 0=NO	Islet DRlyp/lyp HCD vs HCD+Lp299v (log2 ratio ≥0.263; FDR<20%) n=3026 1=YES 0=NO	Islet DRlyp/lyp ND vs Flyp/lyp ND (log2 ratio ≥ 0.263; FDR<20%) n=1950 1=YES 0=NO	Islet Mean Log2 Intensity DRlyp/lyp ND	Islet Mean Log2 Intensity DRlyp/lyp ND+Lp299v	Islet Mean Log2 Intensity DRlyp/lyp HCD	Islet Mean Log2 Intensity DRlyp/lyp HCD+Lp299v	Islet Mean Log2 Intensity Flyp40 ND	Rinm5f data union of all conditions n=3705 1=YES 0=NO	Rinm5f 30 µM propionate/ 6 µM butyrate vs Media (FDR<20%; 1.5 fold) n=3 1=YES 0=NO	Rinm5f 60 µM propionate/ 12 µM butyrate vs Media (FDR<20%; 1.5 fold) n=18 1=YES 0=NO	Rinm5f 120 µM propionate/ 24 µM butyrate vs Media (FDR<20%; 1.5 fold) n=164 1=YES 0=NO	Rinm5f 240 µM propionate /48 µM butyrate vs Media (FDR<20%; 1.5 fold) n=268 1=YES 0=NO	Rinm5f 1500 µM propionate/ 300 µM butyrate vs Media (FDR<20%; 1.5 fold) n=2491 1=YES 0=NO	Rinm5f 500 µM propionate/ 500 µM butyrate vs Media (FDR<20%; 1.5 fold) n=3001 1=YES 0=NO	Rinm5f Mean Log2 Intensity Media only	Rinm5f Mean Log2 Intensity 30 µM propionate/ 6 µM butyrate	Rinm5f Mean Log2 Intensity 60 µM propionate/ 12 µM butyrate	Rinm5f Mean Log2 Intensity 120 µM propionate/ 24 µM butyrate	Rinm5f Mean Log2 Intensity 240 µM propionate/ 48 µM butyrate	Rinm5f Mean Log2 Intensity 1500 µM propionate /300 µM butyrate	Rinm5f Mean Log2 Intensity 500 µM propionate/ 500 µM butyrate
1381769_at	---	0	0	0	0	0	0	5.62	5.91	5.95	5.70	5.97	1	0	0	0	0	0	1	7.36	7.39	7.36	7.43	7.29	6.87	6.50
1381773_at	Abhd3	0	0	0	0	0	0	3.73	3.48	3.32	3.47	3.73	1	0	0	0	0	1	0	4.63	4.78	4.67	4.87	4.91	5.24	4.77
1381795_at	---	0	0	0	0	0	0	4.63	4.91	4.93	4.82	4.93	1	0	0	0	0	1	1	7.28	7.20	7.47	7.73	7.84	8.09	7.10
1381806_at	Ptgs1	0	0	0	0	0	0	6.14	6.15	5.96	6.08	6.18	1	0	0	0	0	1	1	6.94	7.02	7.13	7.14	7.26	7.61	7.42
1381823_at	---	0	0	0	0	0	0	3.67	3.68	3.75	3.71	3.58	1	0	0	0	0	1	1	6.33	6.17	6.35	6.15	5.95	5.18	5.07
1381833_at	Cdca2	0	0	0	0	0	0	4.54	4.48	4.45	4.71	4.61	1	0	0	0	0	1	1	7.27	7.21	7.20	7.24	7.08	6.21	6.62
1381834_at	---	0	0	0	0	0	0	3.51	3.60	3.61	3.62	3.69	1	0	0	0	0	1	0	5.70	5.71	5.88	5.94	5.94	6.41	6.36
1381901_at	Tmem212	0	0	0	0	0	0	5.26	5.26	5.09	5.19	5.13	1	0	0	0	0	1	0	7.67	7.77	7.81	7.76	8.13	8.53	7.43
1381904_at	Vwa7	0	0	0	0	0	0	3.57	3.62	3.46	3.53	3.63	1	0	0	0	0	1	1	7.23	7.23	7.38	7.24	7.19	6.39	6.23
1381932_at	Tent5a	0	0	0	0	0	0	6.85	6.83	6.76	6.69	6.73	1	0	0	0	0	1	0	10.01	10.18	10.18	10.14	10.23	10.59	10.13
1381958_at	Topbp1	0	0	0	0	0	0	3.33	3.27	3.11	3.48	3.38	1	0	0	0	0	0	1	7.18	6.88	7.18	7.27	7.15	6.75	6.14
1381974_at	Btbd3	0	0	0	0	0	0	7.10	6.94	7.28	6.93	7.30	1	0	0	0	0	0	1	8.64	8.61	8.66	8.87	8.81	9.13	9.73
1381975_at	Prune2	0	0	0	0	0	0	8.71	8.85	8.50	9.03	8.35	1	0	0	0	0	1	0	7.95	7.95	8.09	8.21	8.22	8.58	8.81
1382017_at	LOC102550366	0	0	0	0	0	0	3.19	3.18	3.10	3.11	3.17	1	0	0	0	0	1	1	7.83	7.89	8.00	8.12	7.97	6.84	6.42
1382027_at	---	0	0	0	0	0	0	4.32	4.16	4.11	4.20	4.27	1	0	0	0	0	0	1	5.69	5.45	5.47	5.40	5.50	6.00	7.12
1382028_at	Pex19	0	0	0	0	0	0	6.29	6.43	6.42	6.39	6.62	1	0	0	0	0	1	0	6.69	6.80	6.86	6.87	7.01	7.30	7.30
1382037_at	Crim1	0	0	0	0	0	0	5.49	5.45	5.18	5.40	5.13	1	0	0	0	0	1	0	5.83	5.85	5.85	5.84	5.75	6.49	6.50
1382040_at	Eprs1	0	0	0	0	0	0	7.59	7.70	8.07	7.41	7.47	1	0	0	0	0	0	1	8.78	8.65	8.75	8.83	8.72	8.36	7.11
1382050_at	Col4a4 /// LOC1(	0	0	0	0	0	0	5.25	5.22	5.63	5.17	5.18	1	0	0	0	0	0	1	4.62	4.61	4.69	4.70	4.73	4.94	4.84
1382051_at	Hscb	0	0	0	0	0	0	5.84	5.60	5.95	6.23	5.50	1	0	0	0	0	1	1	7.81	7.81	7.82	7.91	7.97	8.44	8.38
1382069_at	Atp11a	0	0	0	0	0	0	4.31	4.43	4.27	4.31	4.76	1	0	0	0	0	1	0	7.36	7.22	7.42	7.47	7.57	8.02	7.69
1382072_at	Olfml2a	0	0	0	0	0	0	3.36	3.48	3.22	3.34	3.14	1	0	0	0	0	1	1	4.18	4.13	4.08	3.99	4.17	5.17	5.93
1382086_at	---	0	0	0	0	0	0	4.13	4.18	4.25	4.03	4.13	1	0	0	0	1	1	1	5.87	5.88	6.32	6.42	6.50	6.91	7.05
1382111_at	Glod5	0	0	0	0	0	0	3.53	3.62	3.35	3.60	3.51	1	0	0	0	0	0	1	4.31	4.31	4.26	4.26	4.41	4.59	5.46
1382162_at	---	0	0	0	0	0	0	5.87	6.01	6.08	6.01	6.11	1	0	0	0	0	1	1	7.01	7.05	7.29	7.23	7.44	7.86	8.01
1382163_at	Gata3	0	0	0	0	0	0	3.12	3.20	3.06	3.09	3.14	1	0	0	0	0	1	1	4.17	4.16	4.51	4.64	4.51	4.83	6.84
1382174_at	Pik3cb	0	0	0	0	0	0	6.22	6.31	6.49	6.24	6.34	1	0	0	0	0	1	1	8.08	8.02	8.13	8.28	8.18	8.66	9.08
1382194_at	---	0	0	0	0	0	0	5.50	5.36	5.65	5.35	5.77	1	0	0	0	0	1	0	7.06	6.97	7.07	7.09	6.91	6.35	6.53
1382197_at	Rhod	0	0	0	0	0	0	5.47	5.52	5.42	5.67	5.50	1	0	0	0	0	1	1	7.52	7.54	7.59	7.74	7.79	8.20	8.17
1382201_at	Unc5c	0	0	0	0	0	0	5.95	5.98	6.16	5.65	6.00	1	0	0	0	0	0	1	7.58	7.67	7.72	7.60	7.55	7.27	7.24
1382202_at	Cux2	0	0	0	0	0	0	3.54	3.52	3.70	3.60	3.58	1	0	0	0	0	0	1	6.15	6.27	6.29	6.39	6.51	6.52	6.71
1382205_at	Zic1	0	0	0	0	0	0	2.91	2.87	2.72	3.05	2.90	1	0	0	0	0	1	1	6.21	6.41	6.34	6.62	6.72	6.81	6.74
1382215_at	Taf4b	0	0	0	0	0	0	3.92	3.64	3.97	4.18	3.62	1	0	0	0	0	0	1	6.08	5.97	6.28	6.34	6.22	6.52	7.01
1382216_at	Gemin6	0	0	0	0	0	0	5.20	5.09	5.11	5.17	5.10	1	0	0	0	0	1	1	7.80	7.71	7.75	7.66	7.49	7.14	6.76
1382218_at	Stra6l	0	0	0	0	0	0	5.04	4.89	5.21	4.81	4.77	1	0	0	0	0	1	1	5.94	5.91	6.16	6.04	6.08	4.69	4.68
1382221_at	---	0	0	0	0	0	0	3.66	3.63	3.42	3.56	3.51	1	0	0	0	0	0	1	4.31	4.29	4.23	4.23	4.33	4.41	4.96
1382244_at	QPRT	0	0	0	0	0	0	5.54	5.91	5.50	5.87	5.92	1	0	0	1	0	0	0	6.43	6.25	6.28	5.83	6.11	6.09	5.67
1382258_at	---	0	0	0	0	0	0	4.24	4.24	4.24	4.69	4.17	1	0	0	0	0	1	0	5.33	5.17	5.44	5.52	5.71	6.08	6.49
1382264_at	Crebbp	0	0	0	0	0	0	7.44	7.12	7.51	7.35	7.51	1	0	0	0	0	1	1	9.12	9.03	9.14	9.11	9.03	8.52	8.18
1382269_at	Cnnm2	0	0	0	0	0	0	5.76	5.94	5.85	6.15	6.15	1	0	0	0	0	0	1	6.68	6.63	6.74	6.74	6.89	7.24	7.29
1382283_at	Wipf1	0	0	0	0	0	0	3.52	3.50	3.65	3.20	3.57	1	0	0	0	0	1	0	7.30	7.35	7.47	7.65	7.72	7.98	7.75
1382284_at	Nek3	0	0	0	0	0	0	5.30	5.44	5.37	5.45	5.44	1	0	0	0	0	1	1	7.38	7.50	7.60	7.69	7.73	7.98	8.09
1382329_at	---	0	0	0	0	0	0	2.70	2.80	2.69	2.77	2.71	1	0	0	0	0	0	1	9.22	9.39	9.46	9.56	9.61	9.77	9.41
1382330_at	LOC102555735 /	0	0	0	0	0	0	7.33	7.19	7.57	7.65	7.32	1	0	0	0	0	0	1	8.77	8.92	8.85	8.84	9.01	9.29	8.93
1382333_at	Thumpd3	0	0	0	0	0	0	5.13	4.98	5.17	5.03	5.32	1	0	0	0	0	1	0	6.39	6.46	6.62	6.74	6.83	7.05	6.77
1382355_at	Galnt4	0	0	0	0	0	0	6.48	6.47	6.45	6.42	6.52	1	0	0	0	0	0	1	5.11	5.10	4.96	5.00	5.02	5.41	5.91
1382363_at	Mpp5	0	0	0	0	0	0	6.06	6.16	6.50	6.09	6.35	1	0	0	0	0	1	1	6.97	7.04	7.17	7.29	7.36	8.14	7.76
1382370_at	Ccnf	0	0	0	0	0	0	4.97	5.14	4.82	5.08	5.06	1	0	0	0	0	0	1	8.10	8.07	8.14	8.05	7.96	7.67	7.26
1382374_at	LOC102550324 /	0	0	0	0	0	0	7.26	7.44	7.55	7.14	7.41	1	0	0	0	0	0	1	9.27	9.40	9.44	9.33	9.33	9.04	8.30
1382375_at	Wnt5a	0	0	0	0	0	0	4.24	4.17	4.04	4.07	4.13	1	0	0	0	0	0	1	4.51	4.38	4.55	4.18	4.31	4.70	5.46
1382413_at	---	0	0	0	0	0	0	3.50	3.43	3.44	3.34	3.64	1	0	0	0	0	0	1	5.34	5.09	5.30	5.39	5.34	5.04	4.60
1382421_at	Dst	0	0	0	0	0	0	3.68	3.66	3.69	3.64	3.60	1	0	0	0	0	0	1	4.91	4.83	4.93	4.88	4.89	5.23	5.71
1382433_at	Sorcs1	0	0	0	0	0	0	3.29	3.46	3.08	3.14	3.16	1	0	0	0	0	0	1	5.74	5.67	5.70	5.64	5.48	6.29	5.61
1382455_at	Xpr1	0	0	0	0	0	0	7.09	7.04	7.58	7.05	7.21	1	0	0	0	0	1	1	8.05	8.04	8.19	8.27	8.34	8.76	9.18
1382457_at	LOC103692931	0	0	0	0	0	0	4.21	4.22	4.25	3.90	4.29	1	0	0	0	0	1	1	7.38	7.45	7.53	7.53	7.59	7.99	8.37
1382459_at	Ska3	0	0	0	0	0	0	4.14	4.18	4.19	4.15	4.16	1	0	0	0	0	1	1	6.74	6.74	6.81	6.82	6.80	6.12	5.81
1382469_at	---	0	0	0	0	0	0	6.00	6.34	6.55	6.31	6.37	1	0	0	0	0	0	1	5.60	5.54	5.73	5.62	5.71	6.04	6.76
1382490_at	---	0	0	0	0	0	0	4.54	4.63	4.65	4.96	4.64	1	0	0	0	0	1	0	5.90	5.76	5.88	5.45	5.60	5.29	5.11
1382493_at	Cenpf	0	0	0	0	0	0	5.77	5.85	6.17	5.91	6.14	1	0	0	0	0	1	1	8.58	8.66	8.71	8.75	8.61	7.81	7.89
1382514_at	---	0	0	0	0	0	0	3.20	3.36	3.34	3.41	3.32	1	0	0	0	0	0	1	5.72	5.80	5.96	5.98	5.64	5.40	5.17
1382525_at	Lypd6b	0	0	0	0	0	0	3.55	3.50	3.86	3.56	3.59	1	0	0	0	0	1	0	5.37	5.29	5.25	5.23	5.04	4.44	4.54
1382541_at	Alk	0	0	0	0	0	0	5.07	5.19	4.88	4.93	5.14	1	0	0	0	0	1	1	6.13	6.26	6.27	6.20	6.38	7.25	7.08
1382552_at	Dip2c	0	0	0	0	0	0	4.56	4.61	4.57	4.32	4.72	1	0	0	0	0	1	0	6.03	5.76	5.91	5.89	5.79	5.31	5.63
1382557_at	Lsm11	0	0	0	0	0	0	5.31	5.13	5.09	5.54	5.14	1	0	0	0	0	1	1	6.77	6.85	6.69	6.53	6.53	5.66	5.23
1382568_at	Rhbdd1	0	0	0	0	0	0	6.63	6.62	6.88	6.79	6.75	1	0	0	0	0	0	1	7.49	7.29	7.40	7.49	7.39	6.98	6.38
1382571_at	Podxl	0	0	0	0	0	0	6.49	6.46	6.39	6.22	6.37	1	0	0	0	1	1	0	5.96	6.18	6.24	6.25	6.59	7.09	6.20
1382572_at	RGD1310951	0	0	0	0	0	0	6.41	6.29	6.37	6.41	6.42	1	0	0	0	0	0	1	5.49	5.52	5.50	5.50	5.51	5.70	6.20
1382575_at	---	0	0	0	0	0	0	3.50	3.27	3.76	3.29	3.48	1	0	0	0	0	1	0	6.29	6.42	6.54	6.61	6.76	6.98	6.33
1382581_at	Ehf	0	0	0	0	0	0	4.28	4.34	4.09	4.64	4.22	1	0	0	0	0	0	1	5.13	5.06	4.93	5.08	4.81	4.58	4.45
1382591_at	---	0	0	0	0	0	0	3.60	3.60	3.68	3.64	3.63	1	0	0	0	0	0	1	3.84	3.91	3.97	3.96	3.91	3.93	4.41
1382592_at	Spdl1	0	0	0	0	0	0	4.55	4.66	5.00	4.79	4.79	1	0	0	0	0	0	1	7.68	7.61	7.67	7.80	7.71	7.17	6.76
1382617_at	Cuedc1	0	0	0	0	0	0	5.30	5.35	5.09	5.47	5.23	1	0	0	0	0	0	1	6.68	6.62	6.86	6.74	6.61	6.10	5.81
1382619_at	---	0	0	0	0	0	0	3.09	2.96	2.93	3.13	2.91	1	0	0	0	0	1	1	3.70	3.66	3.82	3.63	3.74	5.10	5.50
1382667_at	Mfsd10	0	0	0	0	0	0	4.90	4.86	5.10	4.68	4.95	1	0	0	0	0	0	1	8.23	8.23	8.36	8.44	8.35	7.94	7.09
1382685_at	Slit2	0	0	0	0	0	0	3.30	3.32	3.30	3.52	3.23	1	0	0	0	0	1	0	4.41	4.41	4.36	4.27	4.29	5.00	5.63
1382686_at	---	0	0	0	0	0	0	5.07	5.55	5.04	4.76	5.24	1	0	0	0	0	0	1	6.07	6.11	6.16	5.97	6.05	5.65	5.24
1382748_at	Mmut	0	0	0	0	0	0	7.92	7.82	7.90	8.03	7.95	1	0	0	0	0	1	0	6.84	6.88	7.01	7.23	7.33	7.59	6.97
1382768_at	---	0	0	0	0	0	0	4.27	4.44	4.41	4.43	4.57	1	0	0	0	0	1	1	6.78	6.75	6.88	6.76	6.69	6.11	4.99
1382776_at	Mboat1	0	0	0	0	0	0	3.95	4.00	3.71	4.00	3.89	1	0	0	0	0	1	1	7.30	7.36	7.52	7.73	7.70	8.31	8.52
1382783_at	Blm	0	0	0	0	0	0	4.40	4.47	4.68	4.67	4.52	1	0	0	0	0	1	1	8.04	8.01	8.10	8.21	8.05	7.31	7.30
1382791_at	Zfp961	0	0	0	0	0	0	3.00	2.83	3.17	3.05	2.97	1	0	0	0	0	1	0	3.65	3.73	3.75	3.76	3.93	4.25	3.83
1382793_at	---	0	0	0	0	0	0	4.51	4.52	4.17	4.27	4.37	1	0	0	0	0	1	0	6.35	6.58	6.66	6.72	6.66	6.96	6.89
1382803_at	Map4k1	0	0	0	0	0	0	4.65	4.68	4.44	4.63	4.62	1	0	0	0	0	1	0	4.93	5.12	5.26	5.11	5.30	5.81	5.45
1382830_at	Suv39h2	0	0	0	0	0	0	3.65	3.65	3.79	3.66	3.87	1	0	0	0	0	1	1	6.03	5.97	5.95	5.86	5.77	4.94	5.03
1382841_at	Tssc4	0	0	0	0	0	0	7.13	6.95	7.15	6.84	6.82	1	0	0	0	0	0	1	9.08	9.17	9.14	9.12	8.97	8.77	7.97
1382844_at	RGD1310951	0	0	0	0	0	0	5.15	5.46	5.27	5.31	5.37	1	0	0	0	0	0	1	4.26	4.23	4.37	4.15	4.15	4.39	4.96
1382848_at	---	0	0	0	0	0	0	3.01	3.15	2.96	3.05	3.14	1	0	0	0	0	1	0	7.61	7.61	7.52	7.51	7.51	6.96	6.98
1382849_at	C7orf57	0	0	0	0	0	0	2.78	2.70	2.80	2.77	2.74	1	0	1	1	1	1	1	5.61	5.86	6.23	6.54	6.85	8.61	9.44
1382857_at	---	0	0	0	0	0	0	8.06	7.98	7.99	8.51	8.23	1	0	0	1	0	1	0	8.57	8.77	9.02	9.15	9.13	9.21	8.97
1382886_at	---	0	0	0	0	0	0	5.19	5.09	4.90	5.09	5.08	1	0	0	0	0	1	0	7.24	7.20	7.45	7.57	7.57	7.84	7.40
1382905_at	Mrc2	0	0	0	0	0	0	5.36	5.17	5.06	5.16	5.09	1	0	0	0	0	1	1	6.58	6.65	6.64	6.69	6.83	7.42	7.45
1382909_at	Borcs7	0	0	0	0	0	0	5.59	5.36	5.66	5.51	5.47	1	0	0	0	0	1	1	6.35	6.07	6.01	6.02	5.92	5.40	5.49
1382923_at	Syncrip	0	0	0	0	0	0	6.28	6.29	6.46	6.13	6.31	1	0	0	0	0	0	1	7.70	7.62	7.76	7.79	7.64	7.21	6.82
1382929_at	Leng9	0	0	0	0	0	0	3.86	3.93	3.97	3.90	3.98	1	0	0	0	0	1	1	6.44	6.38	6.56	6.44	6.35	5.79	5.28
1382931_at	Arl4a	0	0	0	0	0	0	5.07	5.07	5.39	5.06	5.14	1	0	0	0	0	1	1	5.27	5.50	5.63	5.73	5.81	6.31	6.89
1382943_at	Pcdhga1 /// Pcd	0	0	0	0	0	0	4.73	4.90	4.79	4.51	4.52	1	0	0	0	0	0	1	7.55	7.53	7.73	7.79	7.58	7.05	5.80
1382944_at	Ero1b	0	0	0	0	0	0	11.48	11.44	11.61	11.24	11.95	1	0	0	0	0	1	1	9.60	9.68	9.69	9.77	9.96	10.64	10.59
1382967_at	Adgrg2	0	0	0	0	0	0	4.14	4.21	4.07	4.13	4.13	1	0	0	0	0	1	1	8.63	8.67	8.54	8.45	8.61	7.96	6.92
1382995_at	Nrp2	0	0	0	0	0	0	5.50	5.76	5.30	5.52	5.59	1	0	0	0	0	0	1	4.59	4.64	4.55	4.33	4.53	4.93	5.39
1383008_at	Smc4	0	0	0	0	0	0	4.75	4.71	4.96	4.85	4.98	1	0	0	0	0	0	1	8.07	7.95	7.99	8.07	7.95	7.54	7.38
1383010_at	Bcl11a	0	0	0	0	0	0	2.93	2.97	2.78	2.95	3.20	1	0	0	0	0	1	1	3.51	3.56	3.61	3.62	3.64	4.40	6.34
1383035_at	Ffar2	0	0	0	0	0	0	5.58	5.68	5.74	5.50	5.70	1	0	0	0	0	0	1	7.68	7.74	7.66	7.42	7.40	7.19	6.39
1383066_at	---	0	0	0	0	0	0	3.67	3.75	3.69	3.64	3.83	1	0	0	0	0	1	1	5.55	5.85	5.88	6.13	5.96	6.25	6.43
1383069_at	Rbms3	0	0	0	0	0	0	6.16	6.17	6.13	6.25	6.31	1	0	0	0	0	0	1	6.98	7.06	7.26	7.27	7.30	6.67	5.93
1383081_at	LOC100365289	0	0	0	0	0	0	7.26	7.33	7.59	7.39	7.59	1	0	0	0	0	1	1	8.57	8.54	8.57	8.50	8.40	7.88	7.42
1383108_at	Taf13	0	0	0	0	0	0	7.62	7.35	7.80	7.18	7.72	1	0	0	0	0	1	1	9.14	9.24	9.33	9.62	9.62	10.13	9.95
1383110_at	Klhl24	0	0	0	0	0	0	6.32	6.24	6.84	6.51	6.05	1	0	0	0	0	0	1	8.24	8.27	8.12	8.15	8.02	7.68	6.88
1383111_at	---	0	0	0	0	0	0	3.85	3.94	3.56	3.80	3.79	1	0	0	0	0	0	1	7.31	7.42	7.39	7.37	7.63	6.88	6.34
1383120_at	Arsk	0	0	0	0	0	0	5.39	5.25	5.59	5.58	5.69	1	0	0	1	1	1	1	6.32	6.73	6.78	6.96	7.22	7.55	6.65
1383131_at	Itgb2	0	0	0	0	0	0	4.67	4.63	4.68	4.66	4.89	1	0	0	0	0	0	1	4.99	4.75	4.90	4.69	4.96	4.92	5.42
1383135_at	Ntrk2	0	0	0	0	0	0	8.30	8.52	8.68	8.30	8.42	1	0	0	0	0	1	1	9.64	9.67	9.55	9.35	9.45	8.90	8.59
1383148_at	---	0	0	0	0	0	0	5.48	5.25	5.23	5.54	5.14	1	0	0	0	0	1	1	5.58	5.59	5.79	5.78	5.75	6.62	6.63
1383150_at	---	0	0	0	0	0	0	6.37	6.17	6.50	6.59	6.43	1	0	0	0	0	1	1	6.78	6.87	7.04	7.29	7.31	7.54	7.50
1383174_at	---	0	0	0	0	0	0	5.73	5.74	5.92	5.62	5.84	1	0	0	0	0	1	1	7.10	7.24	7.35	7.29	7.51	8.02	7.95
1383184_at	Znrf2	0	0	0	0	0	0	5.58	5.48	5.62	5.35	5.19	1	0	0	0	0	1	0	7.27	7.26	7.31	7.36	7.40	7.90	7.72
1383208_at	Pole3	0	0	0	0	0	0	4.94	4.96	4.82	4.97	4.81	1	0	0	0	0	1	1	7.47	7.38	7.47	7.41	7.16	6.79	6.03
1383221_at	Smim22	0	0	0	0	0	0	8.51	8.64	8.26	8.59	8.36	1	0	0	0	0	0	1	10.22	10.25	10.23	10.16	10.12	9.82	9.65
1383228_at	LOC103691212	0	0	0	0	0	0	6.20	6.25	6.36	6.46	6.38	1	0	0	0	0	1	1	6.19	6.08	6.31	6.36	6.32	7.14	7.13
1383247_a_at	Spns2	0	0	0	0	0	0	6.05	5.95	6.01	6.03	6.03	1	0	0	0	0	1	0	9.11	9.15	9.32	9.32	9.44	9.84	9.21
1383248_at	Fmo5	0	0	0	0	0	0	5.27	5.02	5.00	4.97	5.28	1	0	0	0	0	0	1	4.32	4.24	4.46	4.25	4.38	4.48	5.02
1383253_at	---	0	0	0	0	0	0	8.50	8.36	8.74	8.84	8.38	1	0	0	0	0	1	0	8.54	8.62	8.75	8.90	8.89	9.25	9.34
1383258_at	Mark1	0	0	0	0	0	0	4.93	4.81	5.40	4.87	5.22	1	0	0	0	0	1	1	8.11	8.15	8.12	8.01	7.98	7.41	6.97
1383270_at	Tpgs2	0	0	0	0	0	0	5.64	5.68	5.56	5.91	5.55	1	0	0	0	0	1	0	7.64	7.62	7.74	7.74	7.89	8.23	7.92
1383279_at	Rcbtb2	0	0	0	0	0	0	6.83	6.69	6.86	6.74	6.76	1	0	0	0	0	0	1	5.92	5.81	5.97	5.86	5.76	5.37	4.08
1383284_at	Ncam1	0	0	0	0	0	0	8.56	8.78	8.81	8.58	8.82	1	0	0	0	0	1	1	10.04	9.98	10.04	10.05	10.18	10.69	11.03
1383287_at	Fubp1	0	0	0	0	0	0	6.97	6.86	7.32	6.79	6.99	1	0	0	0	0	1	0	9.09	9.09	9.10	9.09	8.97	8.44	7.96
1383292_at	Incenp	0	0	0	0	0	0	5.44	5.51	5.32	5.64	5.63	1	0	0	0	0	1	1	8.05	7.90	7.94	7.92	7.83	7.33	6.82
1383322_at	Rasl11b	0	0	0	0	0	0	3.84	3.67	3.60	3.90	3.70	1	0	0	0	0	1	1	5.71	5.71	5.82	5.92	5.94	7.10	7.53
1383323_at	Dsn1	0	0	0	0	0	0	4.65	4.55	4.61	4.45	4.71	1	0	0	0	0	1	1	8.59	8.53	8.61	8.62	8.49	7.89	7.52
1383326_a_at	Pdcd4	0	0	0	0	0	0	10.12	10.02	9.94	10.31	9.92	1	0	0	0	0	1	0	8.68	8.84	8.76	8.87	9.06	9.28	8.36
1383353_at	Efnb2	0	0	0	0	0	0	6.49	6.47	6.58	6.79	6.51	1	0	0	0	0	0	1	7.16	7.14	7.16	7.19	7.06	7.34	8.50
1383370_at	Brinp2	0	0	0	0	0	0	6.36	6.36	6.12	6.35	6.23	1	0	0	0	0	1	0	7.21	7.20	7.31	7.29	7.33	7.87	7.77
1383375_at	Pip4p2	0	0	0	0	0	0	7.72	7.45	8.23	7.65	8.16	1	0	0	0	0	1	1	9.00	8.99	9.00	9.04	8.98	8.40	8.19
1383385_at	Wdr78	0	0	0	0	0	0	4.57	4.44	4.51	4.51	4.65	1	0	0	0	0	0	1	5.51	5.31	5.46	5.32	5.27	6.06	6.55
1383390_at	Amer2	0	0	0	0	0	0	4.41	4.84	4.93	4.35	4.67	1	0	0	0	0	1	1	6.35	6.39	6.45	6.34	6.17	5.61	5.40

Probeset ID	Gene Symbol	Islet Data Set (log2 ratio ≥0.263; FDR <20%) union n=5325 1=YES 0=NO	Islet DRlyp/lyp ND vs ND+Lp299v (log2 ratio ≥0.263; FDR<20%) n=429 1=YES 0=NO	Islet DRlyp/lyp ND vs HCD (log2 ratio ≥0.263; FDR<20%) n=2247 1=YES 0=NO	Islet DRlyp/lyp ND vs HCD+Lp299v (log2 ratio ≥0.263; FDR<20%) n=1595 1=YES 0=NO	Islet DRlyp/lyp HCD vs HCD+Lp299v (log2 ratio ≥0.263; FDR<20%) n=3026 1=YES 0=NO	Islet DRlyp/lyp ND vs Flyp/lyp ND (log2 ratio ≥ 0.263; FDR<20%) n=1950 1=YES 0=NO	Islet Mean Log2 Intensity DRlyp/lyp ND	Islet Mean Log2 Intensity DRlyp/lyp ND+Lp299v	Islet Mean Log2 Intensity DRlyp/lyp HCD	Islet Mean Log2 Intensity DRlyp/lyp HCD+Lp299v	Islet Mean Log2 Intensity Flyp40 ND	Rinm5f data union of all conditions n=3705 1=YES 0=NO	Rinm5f 30 µM propionate/ 6 µM butyrate vs Media (FDR<20%; 1.5 fold) n=3 1=YES 0=NO	Rinm5f 60 µM propionate/ 12 µM butyrate vs Media (FDR<20%; 1.5 fold) n=18 1=YES 0=NO	Rinm5f 120 µM propionate/ 24 µM butyrate vs Media (FDR<20%; 1.5 fold) n=164 1=YES 0=NO	Rinm5f 240 µM propionate /48 µM butyrate vs Media (FDR<20%; 1.5 fold) n=268 1=YES 0=NO	Rinm5f 1500 µM propionate/ 300 µM butyrate vs Media (FDR<20%; 1.5 fold) n=2491 1=YES 0=NO	Rinm5f 500 µM propionate/ 500 µM butyrate vs Media (FDR<20%; 1.5 fold) n=3001 1=YES 0=NO	Rinm5f Mean Log2 Intensity Media only	Rinm5f Mean Log2 Intensity 30 µM propionate/ 6 µM butyrate	Rinm5f Mean Log2 Intensity 60 µM propionate/ 12 µM butyrate	Rinm5f Mean Log2 Intensity 120 µM propionate/ 24 µM butyrate	Rinm5f Mean Log2 Intensity 240 µM propionate/ 48 µM butyrate	Rinm5f Mean Log2 Intensity 1500 µM propionate /300 µM butyrate	Rinm5f Mean Log2 Intensity 500 µM propionate/ 500 µM butyrate
1383402_at	Aff3	0	0	0	0	0	0	3.30	3.42	3.26	3.36	3.46	1	0	0	0	0	1	0	8.20	8.24	8.18	7.88	8.00	7.46	7.46
1383403_at	Casd1	0	0	0	0	0	0	7.77	7.69	8.33	7.80	7.87	1	0	0	0	0	0	1	8.75	8.80	8.81	9.02	8.97	8.68	7.91
1383404_at	Ncam1	0	0	0	0	0	0	8.41	8.50	8.76	8.29	8.52	1	0	0	0	0	1	1	9.68	9.69	9.77	9.81	9.90	10.45	10.76
1383409_at	LOC100911221	0	0	0	0	0	0	7.28	7.27	7.18	7.70	7.21	1	0	0	0	0	0	1	7.63	7.69	7.67	7.50	7.63	7.80	8.15
1383422_at	Bend5	0	0	0	0	0	0	4.33	4.26	4.71	4.15	4.36	1	0	0	0	0	0	1	6.88	6.96	7.09	6.94	6.95	6.31	6.24
1383432_at	Dapl1	0	0	0	0	0	0	4.91	5.12	5.19	5.24	5.14	1	0	0	1	1	1	1	5.98	6.07	6.41	6.69	6.89	7.47	7.23
1383457_at	Mfsd4a	0	0	0	0	0	0	3.84	3.91	3.43	3.76	3.53	1	0	0	0	0	1	1	8.49	8.47	8.55	8.53	8.64	9.09	8.92
1383481_at	Auts2	0	0	0	0	0	0	4.85	4.94	4.68	4.87	4.62	1	0	0	0	0	1	1	7.55	7.35	7.53	7.32	7.34	6.89	6.95
1383503_at	Plek2	0	0	0	0	0	0	4.95	5.10	5.09	5.21	5.24	1	0	0	0	0	1	0	6.63	6.63	6.99	7.05	7.05	7.31	6.80
1383505_at	---	0	0	0	0	0	0	4.43	4.63	4.53	4.33	4.45	1	0	0	0	0	0	1	6.73	6.63	6.87	6.86	6.79	7.19	6.99
1383506_at	Bcl11a	0	0	0	0	0	0	3.80	3.89	3.68	3.88	3.76	1	0	0	0	0	0	1	4.54	4.38	4.30	4.36	4.32	4.69	5.06
1383515_at	Ube2k	0	0	0	0	0	0	6.13	6.06	6.39	6.03	6.30	1	0	0	0	0	1	0	7.35	7.40	7.52	7.71	7.81	7.97	7.79
1383519_at	Hk2	0	0	0	0	0	0	5.00	5.16	4.70	4.74	4.93	1	0	0	1	1	0	0	8.25	8.28	8.01	7.34	7.66	7.81	8.26
1383536_at	Dhfr	0	0	0	0	0	0	4.31	4.39	4.76	4.77	4.60	1	0	0	0	0	1	0	10.62	10.57	10.38	10.15	10.14	9.86	9.63
1383541_at	Nagpa	0	0	0	0	0	0	5.36	5.27	5.29	5.46	5.23	1	0	0	0	0	0	1	7.03	6.96	7.05	6.93	6.90	6.57	6.33
1383542_at	---	0	0	0	0	0	0	3.59	3.42	3.27	3.52	3.58	1	0	0	0	0	1	1	6.51	6.62	6.74	6.91	6.99	7.74	7.95
1383549_at	Rbm48	0	0	0	0	0	0	5.14	5.01	5.33	5.15	5.20	1	0	0	0	0	1	1	7.39	7.42	7.43	7.39	7.35	6.51	5.80
1383550_at	Btd	0	0	0	0	0	0	3.40	3.44	3.56	3.41	3.41	1	0	0	0	0	0	1	4.66	4.60	4.70	4.72	4.39	4.12	3.93
1383557_at	---	0	0	0	0	0	0	3.60	3.79	4.12	3.48	3.94	1	0	0	0	0	1	1	5.67	5.70	5.85	5.99	6.23	6.84	7.24
1383568_at	Tube1	0	0	0	0	0	0	4.63	4.49	4.66	4.61	4.68	1	0	0	0	0	0	1	7.89	7.85	8.05	8.06	7.84	7.32	6.88
1383574_at	Man1a1	0	0	0	0	0	0	7.91	7.77	7.70	7.90	7.46	1	0	0	0	0	0	1	7.42	7.18	7.13	7.14	7.06	7.75	7.95
1383580_at	Rnf157	0	0	0	0	0	0	4.42	4.28	4.57	4.34	4.57	1	0	0	0	0	1	1	7.01	7.07	7.14	7.07	7.12	7.77	8.28
1383581_at	Sapcd2	0	0	0	0	0	0	4.36	4.15	4.34	4.38	4.28	1	0	0	0	0	1	1	8.61	8.59	8.57	8.38	8.27	7.56	7.55
1383585_s_at	Snx10	0	0	0	0	0	0	3.36	3.24	3.49	3.00	3.75	1	0	0	0	1	1	0	8.57	8.73	8.91	9.15	9.23	9.24	9.04
1383590_at	Csgalnact1	0	0	0	0	0	0	4.34	4.12	4.11	3.96	4.03	1	0	0	0	0	0	1	6.78	6.72	6.89	6.92	6.98	7.30	7.60
1383601_at	Mtfr2	0	0	0	0	0	0	5.40	5.10	5.86	5.68	5.64	1	0	0	0	0	1	1	8.50	8.47	8.51	8.55	8.50	7.85	7.73
1383610_at	Eya2	0	0	0	0	0	0	4.01	3.88	3.79	3.88	3.84	1	0	0	0	0	0	1	4.91	5.04	5.12	4.99	4.97	5.33	5.99
1383612_at	Dnm3	0	0	0	0	0	0	4.73	4.78	4.59	4.67	4.60	1	0	0	0	0	1	1	6.22	6.32	6.54	6.47	6.68	7.46	7.37
1383632_at	---	0	0	0	0	0	0	8.67	8.48	8.95	9.08	8.73	1	0	0	0	0	1	1	8.70	8.74	8.86	9.04	9.04	9.46	9.67
1383634_at	Znf653	0	0	0	0	0	0	5.45	5.40	5.14	5.15	5.15	1	0	0	0	0	1	0	7.18	7.14	7.24	7.15	6.96	6.48	6.68
1383645_at	---	0	0	0	0	0	0	6.29	6.17	6.10	6.54	5.97	1	0	0	0	0	1	1	8.08	8.22	8.32	8.30	8.41	8.96	8.94
1383647_a_at	Crb3	0	0	0	0	0	0	6.85	6.91	7.00	7.00	6.83	1	0	0	0	0	1	0	8.68	8.71	8.58	8.53	8.54	8.08	7.84
1383650_at	Ints6l	0	0	0	0	0	0	6.40	6.25	6.69	6.04	6.62	1	0	0	0	0	1	0	9.53	9.64	9.66	9.83	9.96	10.54	8.74
1383654_a_at	Fn3k	0	0	0	0	0	0	6.54	6.57	6.50	6.43	6.24	1	0	0	0	0	1	1	6.01	6.12	6.20	6.09	6.19	6.72	6.98
1383661_at	Hs3st3b1	0	0	0	0	0	0	7.63	7.52	7.29	7.56	7.92	1	0	0	0	0	1	1	4.93	4.91	4.96	4.90	4.99	6.03	6.43
1383663_at	Gm13420	0	0	0	0	0	0	5.38	5.30	5.40	5.43	5.06	1	0	0	0	0	1	1	8.58	8.52	8.43	8.21	8.12	7.05	6.73
1383664_a_at	Tm7sf2	0	0	0	0	0	0	7.15	7.07	6.85	7.07	6.74	1	0	0	0	0	1	1	8.82	8.81	8.83	8.72	8.56	8.11	7.66
1383684_at	Asf1b	0	0	0	0	0	0	3.63	3.79	3.55	3.77	3.74	1	0	0	0	0	1	1	7.93	7.79	7.87	7.81	7.64	7.05	7.26
1383688_at	LOC100911864	0	0	0	0	0	0	4.25	4.25	3.84	4.26	4.12	1	0	0	0	0	1	1	7.01	7.07	7.13	6.90	6.86	6.04	4.93
1383695_at	Vipr1	0	0	0	0	0	0	6.92	6.84	6.98	6.98	6.64	1	0	0	0	0	1	1	5.58	5.57	5.66	5.54	5.90	6.43	7.06
1383696_at	Hacd1	0	0	0	0	0	0	6.27	5.96	6.11	6.08	5.89	1	0	0	0	0	1	1	6.37	6.28	6.32	6.11	5.89	5.46	5.16
1383699_at	Rit2	0	0	0	0	0	0	5.63	5.74	5.69	5.31	5.99	1	0	0	0	0	1	1	8.87	8.90	8.90	8.91	8.83	8.21	7.01
1383702_at	---	0	0	0	0	0	0	4.12	4.07	4.00	4.08	3.97	1	0	0	0	0	1	1	5.10	5.10	5.28	5.26	5.27	5.98	6.21
1383712_at	LOC102556004	0	0	0	0	0	0	7.58	7.50	7.99	7.67	7.63	1	0	0	0	0	0	1	10.86	10.83	10.82	10.86	10.79	10.31	9.60
1383736_at	Elavl2	0	0	0	0	0	0	3.80	3.81	3.82	3.88	3.78	1	0	0	1	1	1	0	9.23	9.33	9.52	9.86	9.82	10.04	9.97
1383741_at	Stil	0	0	0	0	0	0	4.96	5.15	4.87	4.97	4.97	1	0	0	0	0	1	0	8.41	8.37	8.35	8.30	8.24	7.70	7.98
1383753_at	LOC102551211	0	0	0	0	0	0	5.24	5.39	5.50	5.47	5.42	1	0	0	0	0	1	0	7.96	7.97	7.89	7.82	7.82	7.32	7.06
1383757_at	C3orf80	0	0	0	0	0	0	3.39	3.39	3.69	3.52	3.58	1	0	0	0	0	0	1	9.18	9.22	9.28	9.36	9.30	8.62	7.87
1383768_at	Elavl2	0	0	0	0	0	0	3.77	3.97	3.80	3.71	3.56	1	0	0	1	0	1	0	9.44	9.39	9.69	10.06	9.92	10.19	9.64
1383770_at	Lpp	0	0	0	0	0	0	6.22	5.96	6.10	6.27	6.05	1	0	0	0	0	1	0	8.16	8.42	8.45	8.56	8.53	8.80	8.94
1383780_at	Hectd1	0	0	0	0	0	0	3.57	3.60	3.63	3.37	3.57	1	0	0	0	0	1	0	5.46	5.16	5.42	5.29	5.22	4.78	4.14
1383784_at	Fut10	0	0	0	0	0	0	5.47	5.65	5.84	5.42	5.49	1	0	0	0	0	0	1	6.76	6.67	6.84	6.82	6.75	7.04	7.40
1383787_at	Popdc3	0	0	0	0	0	0	3.65	3.60	3.64	3.77	3.72	1	0	0	0	0	1	0	5.39	5.22	5.41	5.39	5.21	4.69	5.56
1383799_at	Pik3cb	0	0	0	0	0	0	4.66	4.68	4.83	4.54	4.78	1	0	0	0	0	0	1	6.76	6.71	6.90	6.94	6.88	7.32	7.46
1383812_at	Bfsp2	0	0	0	0	0	0	3.99	4.03	3.83	4.11	3.98	1	0	0	0	0	1	0	5.62	5.79	5.87	6.00	6.13	6.27	5.26
1383818_at	Thnsl1	0	0	0	0	0	0	5.70	5.68	5.84	5.49	5.48	1	0	0	0	0	0	1	7.19	7.34	7.42	7.46	7.40	7.78	7.82
1383821_at	Ilf3	0	0	0	0	0	0	6.05	5.99	6.10	5.87	6.15	1	0	0	0	0	1	1	8.54	8.49	8.59	8.45	8.43	7.91	7.48
1383840_at	Csgalnact1	0	0	0	0	0	0	4.12	4.05	3.99	4.34	3.99	1	0	0	0	0	1	1	6.50	6.72	6.75	6.80	6.91	7.19	7.54
1383860_at	Fosl2	0	0	0	0	0	0	4.59	4.39	4.18	4.59	4.40	1	0	0	0	0	1	1	8.47	8.38	8.41	8.13	8.08	7.25	5.77
1383866_at	---	0	0	0	0	0	0	6.20	5.98	6.55	5.90	6.37	1	0	0	0	1	1	1	6.55	6.70	6.96	7.07	7.22	7.60	7.95
1383881_at	LOC102547290	0	0	0	0	0	0	5.06	5.12	5.03	5.16	4.93	1	0	0	0	0	0	1	6.21	6.21	6.41	6.16	6.04	5.76	5.53
1383897_at	Macroh2a2	0	0	0	0	0	0	4.74	4.65	4.46	4.69	4.64	1	0	0	0	0	1	1	8.37	8.38	8.33	8.25	8.17	7.13	5.97
1383901_at	---	0	0	0	0	0	0	5.87	6.18	5.97	6.04	6.21	1	0	0	0	0	1	1	6.73	6.79	6.81	6.70	6.63	5.87	5.55
1383903_at	St8sia5	0	0	0	0	0	0	3.99	3.95	3.80	4.06	3.73	1	0	0	0	0	1	1	6.41	6.60	6.63	6.59	6.98	7.47	7.04
1383921_at	---	0	0	0	0	0	0	4.37	4.24	4.53	4.58	4.32	1	0	0	0	0	0	1	8.49	8.53	8.63	8.90	8.81	8.20	7.91
1383925_at	Miip	0	0	0	0	0	0	5.32	5.32	5.01	5.38	5.27	1	0	0	0	0	1	0	7.10	7.08	7.03	6.88	6.86	6.50	6.42
1383926_at	Bub1b	0	0	0	0	0	0	4.65	4.72	4.67	4.66	4.70	1	0	0	0	0	1	1	9.05	8.96	8.93	8.85	8.72	7.89	7.91
1383931_at	Ptprk	0	0	0	0	0	0	6.13	6.20	6.30	6.31	6.23	1	0	0	0	0	1	1	5.10	4.91	4.85	4.84	5.02	5.81	5.96
1383935_at	Ccdc88c	0	0	0	0	0	0	5.48	5.34	5.27	5.21	5.17	1	0	0	0	0	1	1	7.47	7.46	7.56	7.59	7.60	8.18	8.49
1383940_at	Nuf2	0	0	0	0	0	0	5.04	5.01	5.41	5.27	5.48	1	0	0	0	0	1	1	9.55	9.50	9.50	9.44	9.32	8.69	9.07
1383942_at	Snx9	0	0	0	0	0	0	8.28	8.27	8.68	8.13	8.41	1	0	0	0	0	1	1	9.62	9.50	9.51	9.38	9.29	8.91	8.48
1383952_at	Mical1	0	0	0	0	0	0	3.57	3.67	3.51	3.65	3.39	1	0	0	0	0	1	1	4.43	4.71	4.74	4.71	4.68	5.06	5.36
1383958_at	Cdca2	0	0	0	0	0	0	4.05	4.18	4.00	4.10	4.10	1	0	0	0	0	1	1	6.72	6.61	6.69	6.54	6.49	6.03	5.94
1383966_at	Epb41l4a	0	0	0	0	0	0	4.15	4.12	3.95	4.03	3.91	1	0	0	0	0	0	1	6.96	6.89	7.02	6.99	7.14	6.40	6.03
1383976_at	---	0	0	0	0	0	0	4.61	4.57	4.69	4.42	4.97	1	0	0	0	0	1	1	3.91	3.86	3.90	3.79	4.05	4.83	5.77
1383981_at	Tp53bp2	0	0	0	0	0	0	7.08	7.07	7.24	7.30	7.21	1	0	0	0	0	0	1	5.25	5.22	5.40	5.41	5.48	5.71	6.08
1383984_at	Nsmce2	0	0	0	0	0	0	3.73	3.83	3.65	3.82	3.79	1	0	0	0	0	1	1	4.66	4.65	4.67	4.91	4.89	5.41	4.79
1383987_at	---	0	0	0	0	0	0	4.31	4.36	4.65	4.22	4.28	1	0	0	0	0	1	1	7.79	7.63	7.79	7.74	7.67	6.96	6.22
1383988_at	LOC102554727	0	0	0	0	0	0	5.59	5.62	5.86	5.56	5.39	1	0	0	0	0	1	1	7.92	7.88	7.87	7.79	7.55	7.12	7.05
1383989_at	Sox4	0	0	0	0	0	0	5.47	5.27	5.16	5.29	5.17	1	0	0	0	0	0	1	7.70	7.48	7.74	7.57	7.61	7.87	8.62
1383992_at	Ermp1	0	0	0	0	0	0	4.56	4.45	4.56	4.54	4.43	1	0	0	0	0	1	1	7.13	7.11	7.29	7.35	7.35	7.90	7.67
1384013_at	Chl1	0	0	0	0	0	0	4.73	4.57	4.37	4.90	4.37	1	0	0	0	0	0	1	6.97	7.18	7.13	6.59	6.86	6.49	6.02
1384032_at	---	0	0	0	0	0	0	6.90	6.74	7.05	6.85	7.21	1	0	0	0	0	0	1	8.00	7.92	7.94	7.95	7.84	7.51	7.39
1384034_at	Vamp4	0	0	0	0	0	0	4.43	4.44	4.54	4.45	4.49	1	0	0	0	0	1	1	6.85	6.96	6.80	6.69	6.62	6.23	5.85
1384042_at	---	0	0	0	0	0	0	4.84	4.94	5.05	4.94	4.85	1	0	0	1	0	0	0	6.29	6.34	6.73	6.91	6.77	6.69	6.20
1384056_at	Fgfr3	0	0	0	0	0	0	6.98	6.96	7.05	7.19	6.83	1	0	0	0	0	1	1	6.96	6.95	7.00	6.83	7.02	7.77	7.82
1384068_at	Ckap2	0	0	0	0	0	0	5.17	5.50	5.58	5.38	5.34	1	0	0	0	0	1	1	10.26	10.20	10.21	10.14	9.96	9.16	9.17
1384070_at	Gmip	0	0	0	0	0	0	5.21	5.24	5.17	5.33	5.19	1	0	0	0	0	1	1	7.54	7.45	7.55	7.58	7.44	6.89	6.32
1384081_at	Tent5b	0	0	0	0	0	0	3.69	3.56	3.38	3.40	3.46	1	0	0	0	0	0	1	4.59	4.59	4.63	4.82	4.74	5.00	5.87
1384096_at	---	0	0	0	0	0	0	3.48	3.53	3.49	3.36	3.72	1	0	0	0	0	1	0	8.03	8.11	8.28	8.40	8.58	8.80	8.62
1384101_at	Wasl	0	0	0	0	0	0	5.83	5.84	6.17	5.84	5.99	1	0	0	0	0	0	1	8.34	8.30	8.34	8.33	8.27	7.77	7.31
1384109_at	---	0	0	0	0	0	0	5.04	4.98	5.29	5.04	5.09	1	0	0	0	0	1	0	6.11	6.17	6.31	6.35	6.49	6.70	5.80
1384120_at	---	0	0	0	0	0	0	4.39	4.45	4.89	4.29	4.40	1	0	0	1	0	0	0	4.69	4.73	4.97	5.34	4.84	4.78	4.20
1384136_at	Osbpl3	0	0	0	0	0	0	6.39	6.34	6.73	6.43	6.45	1	0	0	0	0	0	1	5.68	5.67	5.59	5.59	5.65	6.09	6.45
1384138_at	Kcnt1	0	0	0	0	0	0	4.12	4.08	4.06	4.14	4.28	1	0	0	0	0	1	1	5.91	6.02	6.19	6.33	6.31	6.92	6.79
1384173_at	Skap1	0	0	0	0	0	0	4.36	4.53	4.38	4.37	4.57	1	0	0	0	1	0	0	6.35	6.44	6.58	6.91	7.05	6.90	6.73
1384175_at	Efna4	0	0	0	0	0	0	4.66	4.67	4.50	4.87	4.52	1	0	0	0	0	0	1	4.23	4.31	4.31	4.18	4.20	4.51	5.30
1384178_at	Lrrc4b	0	0	0	0	0	0	6.02	5.91	5.69	5.90	5.85	1	0	0	0	0	0	1	7.90	7.93	8.00	7.97	7.91	7.39	6.78
1384192_at	Chst1	0	0	0	0	0	0	4.02	3.93	3.94	3.94	4.01	1	0	0	0	0	1	0	8.14	8.27	8.41	8.42	8.56	8.96	8.56
1384196_at	Wdr41	0	0	0	0	0	0	6.13	5.98	5.99	6.02	5.87	1	0	0	0	0	1	1	7.35	7.32	7.34	7.32	7.14	6.39	6.17
1384203_at	Pde1b	0	0	0	0	0	0	3.74	3.77	3.62	3.72	3.71	1	0	0	0	0	1	1	5.66	5.57	5.68	5.76	5.85	6.51	7.01
1384211_at	Col11a1	0	0	0	0	0	0	2.89	2.76	3.00	3.25	2.86	1	0	0	0	0	1	1	3.19	3.25	3.21	3.35	3.43	4.94	3.68
1384227_at	Ptprk	0	0	0	0	0	0	6.28	5.96	6.19	6.71	6.26	1	0	0	0	0	0	1	3.95	3.91	3.85	3.72	3.89	4.43	5.94
1384233_at	---	0	0	0	0	0	0	3.59	3.66	3.69	3.77	3.68	1	0	0	0	0	1	0	7.53	7.41	7.45	7.46	7.27	6.84	7.27
1384244_at	Hsdl2	0	0	0	0	0	0	8.10	7.77	8.66	8.04	8.03	1	0	0	0	0	1	1	7.95	8.05	8.10	8.30	8.29	9.03	8.86
1384256_at	Larp7	0	0	0	0	0	0	7.63	7.70	7.75	7.61	7.65	1	0	0	0	0	0	1	9.96	9.83	9.87	9.89	9.76	9.41	9.10
1384259_at	Racgap1	0	0	0	0	0	0	5.00	4.92	5.00	4.85	5.18	1	0	0	0	0	1	1	9.21	9.19	9.33	9.31	9.17	8.41	8.40
1384262_at	Ppp1r3b	0	0	0	0	0	0	3.53	3.53	3.65	3.33	3.33	1	0	0	0	0	1	0	9.03	8.98	8.91	8.56	8.66	8.25	8.82
1384264_at	Myh14	0	0	0	0	0	0	6.66	6.52	6.21	6.74	6.39	1	0	0	0	0	1	1	7.97	8.06	8.25	8.19	8.25	8.66	9.14
1384269_at	---	0	0	0	0	0	0	2.69	2.89	2.69	2.90	2.71	1	0	1	1	1	1	0	6.42	6.80	7.01	7.43	7.62	7.83	6.21
1384283_at	Asb9	0	0	0	0	0	0	5.95	6.16	6.28	6.05	6.17	1	0	0	0	1	0	0	7.38	7.50	7.57	7.80	8.03	7.54	6.33
1384291_at	LOC100909559	0	0	0	0	0	0	3.37	3.53	3.66	3.35	3.43	1	0	0	0	0	0	1	7.83	7.88	7.85	7.85	7.95	8.41	8.50
1384304_at	Flrt1	0	0	0	0	0	0	4.16	4.36	4.49	4.24	4.40	1	0	0	0	1	1	1	7.44	7.62	7.80	7.96	8.10	8.63	8.69
1384310_at	Pde5a	0	0	0	0	0	0	4.99	5.23	5.22	5.23	5.09	1	0	0	0	0	1	1	6.55	6.57	6.77	6.76	6.72	7.33	7.13
1384319_at	Tlk2	0	0	0	0	0	0	5.98	6.20	6.35	6.31	6.24	1	0	0	0	0	0	1	8.02	8.00	8.12	8.16	8.08	7.60	7.02
1384320_at	Adgrb3	0	0	0	0	0	0	3.02	2.97	3.18	2.96	2.83	1	0	0	0	0	1	0	4.55	4.59	4.65	4.76	4.68	5.15	5.47
1384330_at	Thg1l	0	0	0	0	0	0	6.93	7.15	7.01	7.15	6.96	1	0	0	0	0	1	1	8.65	8.60	8.59	8.43	8.36	7.80	7.42
1384338_at	Cdc45	0	0	0	0	0	0	6.08	6.08	5.95	6.03	6.09	1	0	0	0	0	1	1	7.87	7.77	7.82	7.74	7.56	6.67	6.20
1384342_at	Nisch	0	0	0	0	0	0	4.74	4.63	4.86	4.67	4.85	1	0	0	0	0	0	1	6.27	6.25	6.30	6.29	6.15	5.92	5.44
1384361_at	Mal2	0	0	0	0	0	0	3.02	3.05	3.32	2.69	3.07	1	0	0	0	0	1	0	7.73	7.90	8.02	8.15	8.27	8.33	6.17
1384364_at	Fbxo4	0	0	0	0	0	0	5.00	4.94	5.13	5.28	4.98	1	0	0	0	0	1	1	7.97	7.99	7.93	7.86	7.53	6.78	5.61
1384388_at	Tmed9	0	0	0	0	0	0	7.61	7.66	7.69	8.02	7.75	1	0	0	0	0	1	0	10.18	10.15	10.17	9.82	10.02	9.59	9.27
1384392_at	Cyp26b1	0	0	0	0	0	0	3.91	3.80	3.72	3.74	3.78	1	0	0	0	0	1	1	6.16	6.09	6.12	5.82	6.08	6.99	6.95
1384393_at	---	0	0	0	0	0	0	3.03	3.33	3.02	3.05	2.98	1	0	0	1	1	1	1	5.24	5.44	5.59	5.87	6.05	6.66	6.81
1384408_at	Map3k5	0	0	0	0	0	0	6.71	6.92	6.57	7.06	6.55	1	0	0	0	0	1	0	8.00	7.90	7.89	7.83	7.70	7.27	7.20
1384422_at	---	0	0	0	0	0	0	4.57	4.60	4.83	4.74	4.23	1	0	0	1	0	1	1	5.63	5.59	5.95	6.28	6.10	6.51	6.32
1384444_at	Tmem91	0	0	0	0	0	0	4.52	4.54	4.30	4.43	4.41	1	0	0	0	0	0	1	3.88	3.97	4.03	3.75	3.91	4.20	5.11
1384445_at	Ccdc163	0	0	0	0	0	0	4.73	4.83	4.60	4.62	4.56	1	0	0	0	0	0	1	5.93	5.96	6.13	5.91	5.82	5.44	5.44
1384448_at	Rnf138rt1	0	0	0	0	0	0	4.54	4.85	4.80	4.43	4.79	1	0	0	0	0	1	1	6.90	7.05	7.04	7.06	7.00	8.01	7.91
1384453_at	---	0	0	0	0	0	0	6.62	6.59	6.98	6.43	6.56	1	0	0	0	0	0	1	8.13	8.24	8.21	8.13	8.15	7.75	7.20
1384456_at	Usp24	0	0	0	0	0	0	5.27	5.36	5.53	5.55	5.45	1	0	0	0	0	0	1	7.53	7.50	7.63	7.70	7.57	7.16	6.66
1384466_at	Crppa	0	0	0	0	0	0	4.22	4.30	4.55	4.53	4.38	1	0	0	0	0	1	1	6.69	6.70	6.75	6.75	6.67	5.76	4.50
1384478_at	Mllt3	0	0	0	0	0	0	5.94	6.08	6.13	6.23	6.19	1	0	0	0	0	1	1	6.84	6.83	7.02	7.03	7.12	7.56	7.61
1384481_at	---	0	0	0	0	0	0	5.09	5.32	5.25	5.31	5.05	1	0	0	0	0	1	1	7.19	7.13	7.10	6.97	6.90	6.47	5.92
1384485_at	Ptpru	0	0	0	0	0	0	5.60	5.34	5.11	5.54	5.23	1	0	0	0	0	1	1	8.02	8.01	8.05	7.93	8.11	8.79	8.77

Probeset ID	Gene Symbol	Islet Data Set (log2 ratio ≥0.263; FDR <20%) union n=5325 1=YES 0=NO	Islet DRlyp/lyp ND vs ND+Lp299v (log2 ratio ≥0.263; FDR<20%) n=429 1=YES 0=NO	Islet DRlyp/lyp ND vs HCD (log2 ratio ≥0.263; FDR<20%) n=2247 1=YES 0=NO	Islet DRlyp/lyp ND vs HCD+Lp299v (log2 ratio ≥0.263; FDR<20%) n=1595 1=YES 0=NO	Islet DRlyp/lyp HCD vs HCD+Lp299v (log2 ratio ≥0.263; FDR<20%) n=3026 1=YES 0=NO	Islet DRlyp/lyp ND vs Flyp/lyp ND (log2 ratio ≥ 0.263; FDR<20%) n=1950 1=YES 0=NO	Islet Mean Log2 Intensity DRlyp/lyp ND	Islet Mean Log2 Intensity DRlyp/lyp ND+Lp299v	Islet Mean Log2 Intensity DRlyp/lyp HCD	Islet Mean Log2 Intensity DRlyp/lyp HCD+Lp299v	Islet Mean Log2 Intensity Flyp40 ND	Rinm5f data union of all conditions n=3705 1=YES 0=NO	Rinm5f 30 µM propionate/ 6 µM butyrate vs Media (FDR<20%; 1.5 fold) n=3 1=YES 0=NO	Rinm5f 60 µM propionate/ 12 µM butyrate vs Media (FDR<20%; 1.5 fold) n=18 1=YES 0=NO	Rinm5f 120 µM propionate/ 24 µM butyrate vs Media (FDR<20%; 1.5 fold) n=164 1=YES 0=NO	Rinm5f 240 µM propionate /48 µM butyrate vs Media (FDR<20%; 1.5 fold) n=268 1=YES 0=NO	Rinm5f 1500 µM propionate/ 300 µM butyrate vs Media (FDR<20%; 1.5 fold) n=2491 1=YES 0=NO	Rinm5f 500 µM propionate/ 500 µM butyrate vs Media (FDR<20%; 1.5 fold) n=3001 1=YES 0=NO	Rinm5f Mean Log2 Intensity Media only	Rinm5f Mean Log2 Intensity 30 µM propionate/ 6 µM butyrate	Rinm5f Mean Log2 Intensity 60 µM propionate/ 12 µM butyrate	Rinm5f Mean Log2 Intensity 120 µM propionate/ 24 µM butyrate	Rinm5f Mean Log2 Intensity 240 µM propionate/ 48 µM butyrate	Rinm5f Mean Log2 Intensity 1500 µM propionate /300 µM butyrate	Rinm5f Mean Log2 Intensity 500 µM propionate/ 500 µM butyrate
1384488_at	Cdkn2d	0	0	0	0	0	0	6.79	6.83	6.89	6.60	7.06	1	0	0	0	0	1	1	10.35	10.33	10.32	10.22	10.16	9.68	9.56
1384500_at	Ccdc30	0	0	0	0	0	0	6.09	5.85	6.38	6.02	5.73	1	0	0	1	1	1	1	6.12	6.37	6.52	6.72	6.84	7.66	7.35
1384503_at	Klk8	0	0	0	0	0	0	3.36	3.48	3.24	3.58	3.29	1	0	0	0	0	1	0	4.40	4.48	4.79	4.75	4.79	5.29	4.96
1384507_at	Otulinl	0	0	0	0	0	0	3.28	3.25	3.22	3.09	3.18	1	0	0	0	0	1	1	4.80	5.06	5.06	5.14	5.17	5.90	5.97
1384509_s_at	Pcdh17	0	0	0	0	0	0	4.04	3.92	4.16	4.03	3.96	1	0	0	0	1	1	1	5.54	5.46	5.91	5.93	6.16	7.57	7.69
1384513_at	---	0	0	0	0	0	0	2.67	2.64	2.63	2.74	2.68	1	0	0	0	0	1	1	4.32	4.23	4.33	4.39	4.59	4.92	4.29
1384529_at	Greb1	0	0	0	0	0	0	3.41	3.31	3.41	3.39	3.38	1	0	0	0	0	1	1	4.19	4.19	4.46	4.45	4.42	4.92	5.50
1384538_at	Msto1	0	0	0	0	0	0	4.32	4.35	4.04	4.43	4.18	1	0	0	0	0	1	0	6.52	6.41	6.62	6.41	6.27	5.93	5.79
1384562_at	---	0	0	0	0	0	0	5.18	5.45	5.62	5.03	5.50	1	0	0	0	0	1	1	6.15	6.26	6.38	6.16	6.08	5.42	4.43
1384582_at	Traf6	0	0	0	0	0	0	5.65	5.74	5.88	6.06	6.06	1	0	0	0	0	0	1	7.01	6.89	7.02	7.06	6.91	7.44	8.13
1384603_at	Abca4	0	0	0	0	0	0	3.47	3.51	3.16	3.32	3.25	1	0	0	0	0	1	0	5.51	5.26	5.33	4.99	4.98	4.84	4.35
1384606_at	Tedc2	0	0	0	0	0	0	3.76	3.69	3.74	3.72	3.61	1	0	0	0	0	0	1	6.04	6.05	6.01	6.06	6.12	5.71	4.65
1384639_at	Reep6	0	0	0	0	0	0	4.23	4.16	4.45	4.10	4.17	1	0	0	0	0	1	0	8.08	8.13	8.35	8.58	8.63	9.12	7.95
1384643_at	Slc52a3	0	0	0	0	0	0	3.30	3.23	3.19	3.31	3.24	1	0	0	0	1	1	0	5.99	6.35	6.53	6.57	6.78	6.92	6.49
1384667_x_at	Galr2	0	0	0	0	0	0	3.04	3.08	3.01	2.91	3.16	1	0	0	0	0	1	1	8.73	8.78	8.73	8.57	8.42	5.98	4.62
1384671_at	LOC103693493	0	0	0	0	0	0	4.02	4.24	4.03	4.08	4.10	1	0	0	0	0	1	1	6.16	6.16	6.08	5.99	5.68	5.29	5.01
1384695_at	Upk3a	0	0	0	0	0	0	5.14	5.00	4.85	4.84	4.93	1	0	0	0	0	1	1	5.65	5.65	5.58	5.41	5.69	6.74	8.13
1384717_at	---	0	0	0	0	0	0	3.50	3.34	3.40	3.29	3.36	1	0	0	0	0	1	1	10.37	10.52	10.63	10.58	10.69	10.98	10.66
1384723_at	Fut11	0	0	0	0	0	0	4.69	4.80	4.92	4.51	4.61	1	0	0	0	0	1	1	6.75	6.71	6.64	6.79	6.47	6.04	5.14
1384760_at	Snx32	0	0	0	0	0	0	4.26	4.08	4.25	4.20	4.31	1	0	0	0	0	1	0	6.42	6.57	6.62	6.70	6.63	7.06	6.80
1384762_at	Sgo1	0	0	0	0	0	0	3.56	3.68	3.73	3.88	3.72	1	0	0	0	0	1	1	8.07	8.05	8.05	8.15	8.01	6.93	7.12
1384769_a_at	LOC683508 /// Z	0	0	0	0	0	0	5.40	5.42	5.61	5.47	5.50	1	0	0	0	0	0	1	6.79	6.80	6.97	6.89	6.68	6.30	5.68
1384770_at	Slc26a7	0	0	0	0	0	0	3.28	3.11	3.01	3.00	2.99	1	0	0	0	0	0	1	3.83	3.89	3.97	3.91	3.77	4.12	4.61
1384814_at	Rorb	0	0	0	0	0	0	2.65	2.68	2.69	2.68	2.63	1	0	0	0	0	1	1	4.69	4.71	4.98	5.11	4.96	5.88	6.05
1384834_at	Cobl	0	0	0	0	0	0	4.83	4.83	4.72	5.03	4.77	1	0	0	0	0	1	1	8.03	7.94	8.16	8.13	8.20	8.63	8.84
1384842_s_at	Fas	0	0	0	0	0	0	3.70	3.71	3.45	3.78	3.61	1	0	0	0	0	0	1	6.76	6.80	6.70	6.79	6.70	7.20	7.29
1384851_at	Smarcad1	0	0	0	0	0	0	4.99	4.89	4.79	4.88	4.86	1	0	0	0	0	0	1	6.68	6.50	6.68	6.68	6.44	6.24	5.68
1384861_at	Efna3	0	0	0	0	0	0	4.95	4.96	4.81	5.06	4.89	1	0	0	0	0	1	1	6.85	6.87	6.95	6.90	7.02	7.57	7.84
1384862_at	---	0	0	0	0	0	0	2.69	2.80	2.72	2.74	2.73	1	0	0	1	0	1	1	4.35	4.34	4.57	4.94	4.72	5.38	5.06
1384863_at	Cpne7	0	0	0	0	0	0	5.63	5.91	5.50	6.04	5.69	1	0	0	0	0	0	1	5.87	6.01	5.95	5.84	5.92	6.33	6.53
1384864_at	Dhh	0	0	0	0	0	0	5.26	5.46	5.00	5.34	5.19	1	0	0	0	0	1	1	4.90	5.02	5.00	4.85	4.94	5.96	6.33
1384877_at	Aqp11	0	0	0	0	0	0	4.89	4.89	4.54	4.64	4.65	1	0	0	0	0	1	1	5.74	5.69	5.76	5.73	5.83	6.44	6.59
1384882_at	Hectd2	0	0	0	0	0	0	5.34	5.18	5.64	5.57	5.56	1	0	0	0	0	1	1	8.21	8.22	8.34	8.48	8.57	9.10	8.98
1384885_at	Tekt2	0	0	0	0	0	0	4.51	4.39	4.35	4.45	4.37	1	0	0	0	0	1	0	6.34	6.57	6.63	6.71	6.78	7.06	6.74
1384889_at	Nin	0	0	0	0	0	0	4.94	4.76	5.12	4.75	4.91	1	0	0	0	0	0	1	7.27	7.35	7.46	7.43	7.33	7.70	8.11
1384895_at	Pacs2	0	0	0	0	0	0	4.85	4.88	4.83	5.03	4.88	1	0	0	0	0	1	0	6.14	5.90	6.07	5.87	5.80	5.56	5.52
1384939_at	Il18r1	0	0	0	0	0	0	3.27	3.15	3.14	3.12	3.15	1	0	0	0	0	1	1	5.75	5.93	6.00	5.93	6.11	6.99	7.00
1384940_at	Znf518a	0	0	0	0	0	0	4.96	4.89	5.49	5.25	5.22	1	0	0	1	1	1	0	6.69	6.87	7.04	7.42	7.37	7.52	7.10
1384985_at	Parm1	0	0	0	0	0	0	4.61	4.76	4.29	4.47	4.60	1	0	0	0	0	1	0	5.51	5.57	5.78	5.76	5.76	6.13	4.96
1384988_at	Fbxo5	0	0	0	0	0	0	3.98	4.08	3.94	4.05	3.99	1	0	0	0	0	1	1	8.53	8.38	8.38	8.36	8.27	7.59	7.31
1384993_at	Rassf10	0	0	0	0	0	0	6.08	6.15	6.02	6.08	5.96	1	0	0	0	0	0	1	5.98	5.98	6.13	5.93	5.87	6.27	6.74
1385001_at	Gsdmd	0	0	0	0	0	0	4.71	4.62	4.56	4.56	4.49	1	0	0	0	0	1	1	4.40	4.41	4.59	4.54	4.59	5.14	5.45
1385023_at	RASGRF1	0	0	0	0	0	0	3.79	3.93	4.06	4.05	3.96	1	0	0	0	0	0	1	7.74	7.77	7.83	7.81	7.92	7.31	6.51
1385027_at	Ypel1	0	0	0	0	0	0	3.61	3.40	3.46	3.41	3.39	1	0	0	0	0	0	1	4.28	4.16	4.15	4.13	4.14	4.72	5.31
1385030_at	Plpp5	0	0	0	0	0	0	4.61	4.69	4.95	4.73	4.81	1	0	0	0	0	1	1	7.83	7.84	7.91	7.95	8.08	8.42	8.50
1385046_at	Nxpe3	0	0	0	0	0	0	4.46	4.40	4.25	4.20	4.46	1	0	0	0	0	1	0	7.46	7.50	7.60	7.69	7.70	8.11	8.05
1385052_at	---	0	0	0	0	0	0	6.27	6.26	5.94	6.23	6.18	1	0	0	0	0	1	1	6.10	6.19	6.24	6.22	6.37	7.18	7.12
1385068_at	G2e3	0	0	0	0	0	0	5.46	5.43	6.03	5.64	5.57	1	0	0	0	0	1	1	7.64	7.70	7.60	7.64	7.56	6.84	6.87
1385072_at	Galm	0	0	0	0	0	0	4.50	4.23	4.35	4.88	4.51	1	0	0	0	0	1	1	4.20	4.35	4.32	4.23	4.46	4.99	5.68
1385074_at	Smarca2	0	0	0	0	0	0	6.77	6.94	6.99	6.80	7.13	1	0	0	0	0	1	0	9.49	9.38	9.43	9.09	9.11	8.82	8.68
1385089_at	Grhl1	0	0	0	0	0	0	4.19	4.00	4.40	4.05	3.91	1	0	0	0	0	1	1	7.26	7.35	7.42	7.61	7.71	8.30	8.45
1385118_at	---	0	0	0	0	0	0	6.49	6.57	6.91	6.29	6.96	1	0	0	0	0	1	0	8.39	8.47	8.51	8.59	8.71	9.00	9.06
1385124_at	Trib2	0	0	0	0	0	0	5.18	5.07	5.11	4.96	5.15	1	0	0	0	0	1	1	6.30	6.38	6.51	6.59	6.60	7.08	7.09
1385133_at	Spata5l1	0	0	0	0	0	0	4.51	4.41	4.64	4.89	4.79	1	0	0	0	0	1	1	6.45	6.37	6.56	6.46	6.33	5.78	5.90
1385134_at	T2	0	0	0	0	0	0	3.49	3.54	3.45	3.58	3.43	1	0	0	0	0	1	0	6.45	6.38	6.48	6.55	6.62	7.03	6.64
1385151_at	---	0	0	0	0	0	0	5.02	5.18	4.66	4.92	4.94	1	0	0	0	0	0	1	6.35	6.23	6.38	6.26	6.12	5.82	5.70
1385152_at	Eif4g1 /// LOC10	0	0	0	0	0	0	5.89	5.99	6.07	6.01	5.97	1	0	0	0	0	0	1	8.18	7.84	8.13	8.19	7.94	7.69	7.03
1385166_at	---	0	0	0	0	0	0	3.47	3.57	3.44	3.47	3.25	1	0	0	0	0	0	1	4.45	4.25	4.77	4.69	4.64	5.02	5.21
1385173_at	Ebf3	0	0	0	0	0	0	3.47	3.54	3.43	3.52	3.36	1	0	0	0	0	1	1	7.65	7.59	7.94	8.13	8.05	8.50	7.80
1385176_at	---	0	0	0	0	0	0	3.65	3.82	3.63	3.77	3.74	1	0	0	0	1	1	0	6.75	7.04	7.06	7.22	7.35	7.49	6.33
1385183_at	---	0	0	0	0	0	0	5.47	5.41	5.40	5.33	5.40	1	0	0	0	0	1	0	6.16	6.20	6.44	6.49	6.53	6.75	6.08
1385211_at	RGD1310951	0	0	0	0	0	0	7.20	7.19	7.47	7.08	7.45	1	0	0	0	0	1	1	6.50	6.57	6.47	6.38	6.48	7.12	7.75
1385218_at	Noc3l	0	0	0	0	0	0	2.96	2.88	2.99	2.92	2.95	1	0	0	0	0	1	0	4.80	4.84	4.79	5.02	4.83	4.18	4.00
1385229_at	Pcdh20	0	0	0	0	0	0	3.13	3.15	3.15	3.04	3.11	1	0	0	0	0	1	1	5.84	5.83	6.01	6.20	6.14	7.12	7.32
1385244_at	Adam4 /// Gm4	0	0	0	0	0	0	5.59	5.61	5.80	5.34	5.59	1	0	0	0	1	0	0	7.20	7.32	7.60	7.63	7.79	7.73	7.08
1385275_at	Wnt16	0	0	0	0	0	0	3.38	3.41	3.13	3.21	3.34	1	0	0	0	0	1	1	4.18	4.06	4.08	3.93	4.09	5.12	5.79
1385297_at	Recql4	0	0	0	0	0	0	4.19	4.19	4.08	4.31	4.15	1	0	0	0	0	1	1	7.00	6.88	6.91	6.81	6.61	6.04	5.84
1385307_at	Ubtf	0	0	0	0	0	0	5.21	5.47	5.17	5.59	5.15	1	0	0	0	0	1	1	9.10	9.12	9.04	8.86	8.88	8.18	7.79
1385316_at	Nectin3	0	0	0	0	0	0	4.77	4.52	4.49	4.71	4.60	1	0	0	0	0	1	1	5.91	5.81	5.80	5.62	5.53	5.30	5.21
1385323_at	Pgm2l1	0	0	0	0	0	0	4.53	4.72	4.58	4.56	4.52	1	0	1	1	1	1	0	6.08	6.36	6.75	6.79	6.85	7.08	6.95
1385365_at	Extl1	0	0	0	0	0	0	3.76	3.64	3.44	3.95	3.45	1	0	0	0	0	1	1	5.82	5.85	5.99	6.11	6.27	7.21	7.57
1385393_at	Ppargc1a	0	0	0	0	0	0	4.07	3.78	4.00	3.75	3.83	1	0	0	0	1	0	0	7.06	6.83	6.96	6.63	6.38	6.76	6.98
1385411_at	Usp43	0	0	0	0	0	0	5.30	5.36	4.96	5.39	4.98	1	0	0	0	0	0	1	5.23	5.25	5.14	5.25	5.21	5.80	6.51
1385428_at	Xpo4	0	0	0	0	0	0	5.27	5.10	5.23	5.09	5.33	1	0	0	0	0	1	0	7.16	7.10	7.20	7.24	7.03	6.45	6.36
1385432_at	Gal3st1	0	0	0	0	0	0	5.91	5.93	5.52	5.97	5.64	1	0	0	0	0	1	1	7.85	7.85	7.76	7.68	7.53	7.06	6.85
1385463_at	LOC102550863	0	0	0	0	0	0	3.72	4.04	3.84	3.92	3.82	1	0	0	0	0	1	1	8.69	8.71	8.69	8.76	8.85	9.36	9.38
1385464_at	Foxq1	0	0	0	0	0	0	4.16	4.24	4.37	4.35	4.05	1	0	0	0	0	1	0	7.02	6.96	6.99	6.89	6.89	6.42	5.88
1385472_at	Zbtb8a	0	0	0	0	0	0	6.39	6.31	6.52	6.53	6.45	1	0	0	0	0	1	1	7.59	7.62	7.63	7.65	7.55	6.77	6.20
1385517_at	Gtf2i	0	0	0	0	0	0	5.90	5.99	6.03	6.01	5.92	1	0	0	0	0	0	1	7.70	7.71	7.87	7.75	7.75	7.28	6.58
1385521_at	---	0	0	0	0	0	0	4.72	5.11	4.88	4.99	4.91	1	0	0	0	0	1	1	7.60	7.09	7.76	7.30	7.36	6.72	6.70
1385558_at	Esyt3	0	0	0	0	0	0	6.38	6.69	6.89	6.47	6.56	1	0	0	0	0	1	0	4.76	5.12	5.20	5.09	5.11	5.44	5.79
1385559_at	Rnf227	0	0	0	0	0	0	4.85	4.69	4.90	4.71	4.97	1	0	0	0	0	1	1	5.72	5.71	5.86	5.81	6.00	6.73	6.45
1385573_at	Homer3	0	0	0	0	0	0	5.49	5.39	5.46	5.86	5.31	1	0	0	0	0	1	0	7.27	7.30	7.45	7.52	7.65	7.94	7.47
1385589_at	Slc9b2	0	0	0	0	0	0	3.34	3.51	3.48	3.66	3.52	1	0	0	0	0	1	0	5.77	5.83	5.91	5.73	6.00	6.36	7.17
1385619_at	Kif11	0	0	0	0	0	0	4.58	4.67	4.45	4.65	4.56	1	0	0	0	0	1	1	8.78	8.74	8.84	8.84	8.76	8.09	7.59
1385622_at	COL25A1	0	0	0	0	0	0	3.07	2.97	3.23	3.03	3.02	1	0	0	1	1	1	1	4.49	4.77	4.99	5.23	5.19	6.52	6.39
1385626_at	Mturn	0	0	0	0	0	0	3.60	3.77	3.85	3.68	3.65	1	0	0	0	0	0	1	5.48	5.48	5.60	5.38	5.40	5.93	6.33
1385630_at	---	0	0	0	0	0	0	3.96	3.87	4.01	3.75	3.93	1	0	0	0	0	1	0	5.71	5.62	5.86	6.02	5.92	6.64	5.05
1385695_at	Loxl3	0	0	0	0	0	0	4.22	4.17	3.82	4.15	4.05	1	0	0	0	0	1	1	5.17	5.37	5.49	5.59	5.65	6.64	6.73
1385733_at	Exo1	0	0	0	0	0	0	3.38	3.40	3.23	3.50	3.40	1	0	0	0	0	1	1	6.10	5.80	6.13	6.14	6.07	5.22	5.12
1385767_at	Igsf5	0	0	0	0	0	0	8.25	8.39	8.49	8.45	8.42	1	0	0	0	1	1	1	7.66	7.79	7.89	8.15	8.30	8.84	8.92
1385781_at	Ercc6l	0	0	0	0	0	0	3.42	3.26	3.24	3.43	3.39	1	0	0	0	0	1	1	6.13	6.07	6.05	6.18	6.04	5.51	5.59
1385808_at	Phf7	0	0	0	0	0	0	3.80	3.63	3.88	3.98	3.76	1	0	0	0	0	1	0	5.86	5.95	6.17	6.05	6.10	6.51	6.14
1385816_at	Fam72a	0	0	0	0	0	0	3.06	3.17	3.10	3.12	3.11	1	0	0	0	0	1	1	6.20	5.98	6.26	6.15	6.03	4.99	5.36
1385835_at	Plxdc1	0	0	0	0	0	0	5.66	5.78	5.42	5.64	5.38	1	0	0	0	0	1	1	5.45	5.51	5.59	5.74	5.89	6.78	7.29
1385851_at	Capn5	0	0	0	0	0	0	4.87	4.82	4.70	5.25	4.51	1	0	0	0	0	0	1	7.50	7.50	7.59	7.58	7.58	7.96	8.26
1385853_at	Baz1a	0	0	0	0	0	0	2.92	2.83	3.08	2.90	3.00	1	0	0	0	0	1	0	6.31	6.01	6.31	6.23	6.10	5.52	5.15
1385865_at	Poc1a	0	0	0	0	0	0	3.21	3.03	3.16	3.38	3.21	1	0	0	0	0	1	1	4.68	4.79	4.90	4.70	4.56	4.06	3.71
1385874_at	Usp42	0	0	0	0	0	0	3.71	3.84	3.69	3.74	3.67	1	0	0	0	0	1	0	5.40	5.20	5.46	5.36	5.25	4.81	4.50
1385910_at	---	0	0	0	0	0	0	4.46	4.57	4.54	4.52	4.47	1	0	0	0	0	1	0	7.21	7.23	7.29	7.08	7.09	6.41	5.84
1385912_at	Dusp15	0	0	0	0	0	0	5.53	5.73	5.51	5.58	5.60	1	0	0	0	0	1	1	7.73	7.79	7.93	8.13	8.25	8.81	8.16
1385950_at	Fam131b	0	0	0	0	0	0	3.49	3.60	3.39	3.29	3.33	1	0	0	0	0	0	1	7.83	7.84	7.97	8.08	7.96	7.54	6.81
1385953_at	Mllt3	0	0	0	0	0	0	7.49	7.64	7.51	7.82	7.54	1	0	0	0	0	1	1	8.01	8.11	8.20	8.25	8.31	8.88	9.28
1386006_at	Larp7	0	0	0	0	0	0	5.54	5.60	5.76	5.70	5.81	1	0	0	0	0	1	1	7.18	6.93	7.10	7.16	6.91	6.59	6.43
1386039_x_at	---	0	0	0	0	0	0	3.62	3.61	3.42	3.49	3.50	1	0	0	0	0	0	1	7.85	7.73	7.89	7.77	7.87	7.56	6.01
1386080_at	Hey1	0	0	0	0	0	0	6.11	5.73	6.12	5.72	5.91	1	0	0	0	0	1	1	7.27	7.35	7.34	7.37	7.52	8.14	8.28
1386106_at	Zcchc24	0	0	0	0	0	0	3.21	3.17	3.14	3.31	3.12	1	0	0	0	1	1	1	5.11	5.19	5.42	5.57	5.71	6.47	5.23
1386129_at	Ing2	0	0	0	0	0	0	7.59	8.06	7.85	7.89	7.50	1	0	0	0	0	1	0	10.25	10.21	10.20	10.07	10.01	9.66	9.27
1386132_at	Ube2u	0	0	0	0	0	0	3.59	3.73	3.45	3.59	3.51	1	0	0	0	0	0	1	4.85	4.80	4.73	4.78	4.82	5.25	5.39
1386160_at	Tchh	0	0	0	0	0	0	3.06	3.07	2.94	2.82	3.05	1	0	0	0	0	0	1	3.53	3.70	3.72	3.58	3.50	4.10	4.58
1386162_at	Txk	0	0	0	0	0	0	3.56	3.54	3.23	3.48	3.53	1	0	0	0	0	1	1	4.29	4.53	4.51	4.54	4.70	5.33	5.66
1386174_at	Zfp101	0	0	0	0	0	0	3.25	3.32	3.57	3.33	3.55	1	0	0	0	0	1	1	6.11	6.09	6.23	6.35	5.99	5.17	4.72
1386186_s_at	---	0	0	0	0	0	0	7.20	7.35	6.87	7.01	7.25	1	0	0	0	0	0	1	5.94	5.96	5.88	5.81	5.81	6.52	6.52
1386252_at	---	0	0	0	0	0	0	4.18	4.10	4.57	4.05	4.23	1	0	0	0	0	0	1	6.85	6.74	6.99	6.91	6.92	6.47	5.84
1386348_at	Fgf11	0	0	0	0	0	0	5.40	5.27	5.15	5.14	5.40	1	0	0	0	0	0	1	5.53	5.43	5.56	5.48	5.47	5.74	6.29
1386379_at	Atp8a1	0	0	0	0	0	0	4.37	4.50	4.59	4.27	4.43	1	0	0	0	0	0	1	6.42	6.39	6.52	6.55	6.50	6.79	7.84
1386472_at	---	0	0	0	0	0	0	6.26	6.33	6.12	6.20	6.24	1	0	0	0	0	1	0	9.31	9.28	9.19	8.72	9.02	8.42	7.84
1386530_at	Stc1	0	0	0	0	0	0	3.73	3.56	3.46	3.65	3.62	1	0	0	0	1	1	0	9.08	9.28	9.36	9.51	9.84	10.06	9.23
1386540_at	Kit	0	0	0	0	0	0	6.85	6.60	6.95	6.75	6.42	1	0	0	0	0	1	0	11.30	11.24	11.40	11.51	11.65	12.08	11.70
1386571_at	Tom1l1	0	0	0	0	0	0	4.98	4.81	5.37	5.27	4.92	1	0	0	0	1	1	1	7.61	7.69	7.93	8.12	8.20	8.48	8.22
1386573_a_at	C6orf89	0	0	0	0	0	0	5.48	5.43	5.33	5.64	5.56	1	0	0	0	0	1	0	6.93	6.97	6.96	7.03	7.00	7.56	7.63
1386574_at	C6orf89	0	0	0	0	0	0	5.90	5.89	5.75	5.85	6.02	1	0	0	0	0	1	0	6.88	6.92	7.10	7.03	7.11	7.57	7.32
1386597_s_at	Spdya	0	0	0	0	0	0	3.26	3.21	3.17	3.25	3.13	1	0	0	0	0	1	1	4.42	4.31	4.46	4.48	4.43	5.06	4.97
1386637_at	Fgl2	0	0	0	0	0	0	3.88	3.73	3.83	3.54	3.94	1	0	0	0	0	0	1	10.94	10.92	10.88	10.77	10.93	10.69	9.16
1386695_at	Ly75	0	0	0	0	0	0	3.13	3.27	3.05	3.12	3.24	1	0	0	0	0	1	1	5.17	5.21	5.49	5.60	5.67	6.26	6.09
1386773_at	Btd	0	0	0	0	0	0	6.43	6.26	6.25	6.28	6.38	1	0	0	0	0	1	1	7.35	7.27	7.29	7.29	7.14	6.71	6.04
1386774_at	Brinp2	0	0	0	0	0	0	3.27	3.37	3.27	3.27	3.17	1	0	0	0	0	1	0	5.65	5.67	5.68	5.90	5.96	6.50	6.17
1386829_at	Map3k10	0	0	0	0	0	0	3.80	3.71	3.73	3.83	3.64	1	0	0	0	0	0	1	5.59	5.73	5.79	5.56	5.46	5.00	5.29
1386853_s_at	Stx5	0	0	0	0	0	0	6.07	5.82	5.92	5.88	5.91	1	0	0	0	0	0	1	8.72	8.74	8.68	8.67	8.60	8.29	7.37
1386860_at	Mfge8	0	0	0	0	0	0	7.45	7.40	7.19	7.35	7.44	1	0	0	0	0	1	1	8.37	8.41	8.43	8.41	8.65	9.90	9.93
1386872_at	Igf2r	0	0	0	0	0	0	8.54	8.40	8.64	8.97	8.64	1	0	0	0	0	0	1	10.39	10.32	10.40	10.22	10.39	10.87	11.16
1386880_at	Acaa2	0	0	0	0	0	0	8.53	8.09	8.68	8.20	8.10	1	0	0	0	0	1	1	9.25	9.23	9.37	9.47	9.45	9.96	9.86
1386884_at	Htra1	0	0	0	0	0	0	7.24	7.01	7.17	7.25	7.16	1	0	0	0	0	0	1	3.85	3.88	3.78	3.69	3.75	4.16	4.96
1386895_at	Maged1	0	0	0	0	0	0	10.55	10.53	11.00	10.55	10.97	1	0	0	0	0	1	1	12.72	12.75	12.74	12.68	12.62	12.07	11.64
1386906_a_at	Septin9	0	0	0	0	0	0	6.38	6.28	6.16	6.35	6.50	1	0	0	0	0	1	1	10.54	10.51	10.56	10.33	10.33	9.84	8.92

Probeset ID	Gene Symbol	Islet Data Set (log2 ratio ≥0.263; FDR <20%) union n=5325 1=YES 0=NO	Islet DRlyp/lyp ND vs ND+Lp299v (log2 ratio ≥0.263; FDR<20%) n=429 1=YES 0=NO	Islet DRlyp/lyp ND vs HCD (log2 ratio ≥0.263; FDR<20%) n=2247 1=YES 0=NO	Islet DRlyp/lyp ND vs HCD+Lp299v (log2 ratio ≥0.263; FDR<20%) n=1595 1=YES 0=NO	Islet DRlyp/lyp HCD vs HCD+Lp299v (log2 ratio ≥0.263; FDR<20%) n=3026 1=YES 0=NO	Islet DRlyp/lyp ND vs Flyp/lyp ND (log2 ratio ≥ 0.263; FDR<20%) n=1950 1=YES 0=NO	Islet Mean Log2 Intensity DRlyp/lyp ND	Islet Mean Log2 Intensity DRlyp/lyp ND+Lp299v	Islet Mean Log2 Intensity DRlyp/lyp HCD	Islet Mean Log2 Intensity DRlyp/lyp HCD+Lp299v	Islet Mean Log2 Intensity Flyp40 ND	Rinm5f data union of all conditions n=3705 1=YES 0=NO	Rinm5f 30 µM propionate/ 6 µM butyrate vs Media (FDR<20%; 1.5 fold) n=3 1=YES 0=NO	Rinm5f 60 µM propionate/ 12 µM butyrate vs Media (FDR<20%; 1.5 fold) n=18 1=YES 0=NO	Rinm5f 120 µM propionate/ 24 µM butyrate vs Media (FDR<20%; 1.5 fold) n=164 1=YES 0=NO	Rinm5f 240 µM propionate /48 µM butyrate vs Media (FDR<20%; 1.5 fold) n=268 1=YES 0=NO	Rinm5f 1500 µM propionate/ 300 µM butyrate vs Media (FDR<20%; 1.5 fold) n=2491 1=YES 0=NO	Rinm5f 500 µM propionate/ 500 µM butyrate vs Media (FDR<20%; 1.5 fold) n=3001 1=YES 0=NO	Rinm5f Mean Log2 Intensity Media only	Rinm5f Mean Log2 Intensity 30 µM propionate/ 6 µM butyrate	Rinm5f Mean Log2 Intensity 60 µM propionate/ 12 µM butyrate	Rinm5f Mean Log2 Intensity 120 µM propionate/ 24 µM butyrate	Rinm5f Mean Log2 Intensity 240 µM propionate/ 48 µM butyrate	Rinm5f Mean Log2 Intensity 1500 µM propionate /300 µM butyrate	Rinm5f Mean Log2 Intensity 500 µM propionate/ 500 µM butyrate
1386912_at	Pcolce	0	0	0	0	0	0	6.62	6.64	6.45	6.48	6.99	1	0	0	0	0	1	1	7.15	7.20	7.31	7.32	7.60	8.48	8.66
1386917_at	Pc	0	0	0	0	0	0	6.20	6.28	6.22	6.22	6.33	1	0	0	0	0	0	1	6.53	6.48	6.61	6.41	6.45	6.67	7.22
1386926_at	Acsl5	0	0	0	0	0	0	8.15	8.23	8.29	8.14	8.32	1	0	0	0	0	1	1	10.50	10.31	10.34	10.35	10.10	9.03	8.64
1386943_at	Pllp	0	0	0	0	0	0	4.79	4.87	5.06	5.03	4.81	1	0	0	0	0	1	0	4.79	4.69	4.91	4.94	4.85	5.46	5.23
1386947_at	Cdh1	0	0	0	0	0	0	8.95	8.85	9.32	9.16	9.33	1	0	0	0	1	1	1	7.58	7.67	7.95	8.10	8.45	9.49	9.97
1386953_at	Hsd11b1	0	0	0	0	0	0	6.27	6.03	6.17	5.87	5.89	1	0	0	0	0	0	1	4.99	4.89	4.95	4.73	4.68	5.09	5.59
1386969_at	Nrn1	0	0	0	0	0	0	3.57	3.64	3.43	3.58	3.59	1	0	0	0	0	0	1	5.22	5.13	5.01	4.95	5.07	5.24	6.50
1386975_at	Pdk2	0	0	0	0	0	0	5.70	5.50	5.35	5.49	5.49	1	0	0	0	0	1	0	6.19	6.24	6.22	6.13	6.26	6.81	6.20
1386989_at	S1pr2	0	0	0	0	0	0	5.53	5.39	5.06	5.43	5.35	1	0	0	0	0	0	1	5.85	6.09	6.02	5.81	5.96	6.33	6.84
1386994_at	Btg2	0	0	0	0	0	0	9.71	9.93	9.52	9.52	9.59	1	0	0	0	0	0	1	10.13	10.19	10.21	10.11	10.21	10.67	10.61
1386995_at	Btg2	0	0	0	0	0	0	10.45	10.64	10.39	10.19	10.24	1	0	0	0	0	0	1	11.45	11.41	11.51	11.43	11.48	12.01	11.77
1387011_at	Lcn2	0	0	0	0	0	0	3.36	3.39	3.22	3.36	3.40	1	0	0	0	0	0	1	5.29	5.45	5.30	5.26	5.53	5.75	5.79
1387023_at	Gstm2	0	0	0	0	0	0	6.91	6.90	6.71	7.03	7.35	1	0	0	0	0	1	1	8.94	8.91	9.00	8.94	9.04	10.08	10.49
1387048_at	Ddx39a	0	0	0	0	0	0	7.34	7.12	7.47	7.25	7.44	1	0	0	0	0	0	1	9.46	9.47	9.51	9.60	9.47	9.09	8.51
1387068_at	Arc	0	0	0	0	0	0	5.59	5.88	5.67	5.46	5.51	1	0	0	0	0	1	1	7.11	7.07	7.15	7.24	7.46	8.62	7.83
1387070_at	Tmpo	0	0	0	0	0	0	4.67	4.53	4.54	4.24	4.40	1	0	0	0	0	0	1	7.20	7.09	7.24	7.35	7.19	6.87	5.58
1387086_at	Camlg	0	0	0	0	0	0	6.85	6.82	7.25	6.77	7.16	1	0	0	0	0	1	1	9.40	9.41	9.38	9.48	9.42	8.63	7.65
1387091_at	Padi2	0	0	0	0	0	0	5.82	5.69	5.47	5.69	5.70	1	0	0	0	0	0	1	7.02	6.90	7.07	6.82	6.83	7.34	6.92
1387120_at	Psmc3	0	0	0	0	0	0	8.74	8.51	8.55	8.80	8.56	1	0	0	0	0	0	1	11.17	11.21	11.23	11.26	11.17	10.77	10.30
1387129_at	Xrcc1	0	0	0	0	0	0	7.15	7.10	7.14	7.22	6.90	1	0	0	0	0	1	0	8.15	8.04	8.06	7.98	7.93	7.50	7.57
1387130_at	Slc40a1	0	0	0	0	0	0	6.90	6.93	6.92	7.13	6.88	1	0	0	0	0	1	1	5.94	5.90	5.92	5.89	6.03	6.88	7.50
1387131_at	Serpini1	0	0	0	0	0	0	9.73	9.53	9.86	10.18	9.79	1	0	0	0	0	1	1	9.93	9.92	9.99	10.17	10.22	10.84	10.45
1387144_at	Itga1	0	0	0	0	0	0	7.18	6.75	7.03	7.15	7.09	1	0	0	0	0	1	1	5.01	4.84	5.07	5.17	5.09	5.66	6.33
1387151_at	Nup107	0	0	0	0	0	0	5.88	5.92	5.96	5.86	5.92	1	0	0	0	0	1	0	8.47	8.45	8.52	8.47	8.37	7.87	7.75
1387164_at	Cnmd	0	0	0	0	0	0	3.50	3.43	3.48	3.44	3.37	1	0	0	1	1	1	1	4.07	4.25	4.34	4.73	4.95	5.36	5.02
1387172_a_at	Tgfb2	0	0	0	0	0	0	6.07	6.06	6.22	6.03	5.98	1	0	0	0	0	1	0	6.80	6.89	6.84	6.30	6.54	6.05	6.05
1387176_at	Rph3al	0	0	0	0	0	0	8.39	8.56	8.42	8.33	8.55	1	0	0	0	0	0	1	8.69	8.78	8.72	8.65	8.65	8.38	7.59
1387179_at	Adcy8	0	0	0	0	0	0	3.51	3.48	3.65	3.73	3.76	1	0	0	0	0	1	1	4.69	4.53	4.59	4.69	4.58	5.52	7.40
1387180_at	Il1r2	0	0	0	0	0	0	3.11	3.26	3.04	2.94	3.05	1	0	0	0	0	0	1	9.22	9.27	9.27	9.35	9.34	9.15	8.41
1387182_at	Gpr37	0	0	0	0	0	0	3.30	3.30	3.21	3.26	3.17	1	0	0	0	0	1	1	4.21	4.17	4.28	4.21	4.25	4.85	5.37
1387199_a_at	Arhgef9	0	0	0	0	0	0	6.63	6.81	6.77	6.58	6.82	1	0	0	0	0	1	0	6.80	6.83	7.05	7.08	7.07	7.51	7.46
1387200_at	Olig1	0	0	0	0	0	0	3.58	3.61	3.34	3.61	3.55	1	0	0	1	1	0	0	5.70	5.36	5.39	5.11	5.11	5.39	6.40
1387209_at	Sec16b	0	0	0	0	0	0	3.58	3.69	3.77	3.64	3.78	1	0	0	0	1	1	1	4.10	4.21	4.35	4.50	4.71	5.97	5.58
1387211_at	Barhl1	0	0	0	0	0	0	5.70	5.55	5.47	5.63	5.56	1	0	0	0	0	0	1	6.77	6.93	6.93	6.77	6.73	6.49	5.96
1387219_at	Adm	0	0	0	0	0	0	4.07	4.29	3.59	3.88	3.84	1	0	0	1	1	1	1	11.27	11.22	11.09	10.46	10.68	10.07	9.47
1387224_at	Dgkb	0	0	0	0	0	0	4.60	4.70	4.94	4.45	4.90	1	0	0	0	0	0	1	9.99	10.08	10.17	10.26	10.17	9.42	8.76
1387241_at	Gpr88	0	0	0	0	0	0	3.43	3.57	3.33	3.38	3.60	1	0	0	0	0	1	1	9.35	9.34	9.25	9.00	9.00	7.25	5.71
1387253_at	Guca2b	0	0	0	0	0	0	4.31	4.20	4.19	4.25	4.21	1	0	0	0	0	1	0	7.99	8.04	8.03	7.99	8.06	8.69	9.00
1387257_at	Sct	0	0	0	0	0	0	4.21	4.04	3.91	4.12	4.11	1	0	0	0	0	1	1	8.96	9.06	9.02	8.83	8.76	7.67	8.24
1387264_at	Kcnk6	0	0	0	0	0	0	6.95	6.88	6.62	7.02	6.83	1	0	0	0	0	0	1	7.68	7.69	7.74	7.65	7.77	7.23	6.73
1387269_s_at	Plaur	0	0	0	0	0	0	4.81	4.64	4.57	4.71	4.68	1	0	0	0	0	1	1	8.03	8.10	8.03	7.88	7.87	7.44	6.69
1387274_at	Dlx5	0	0	0	0	0	0	4.47	4.54	4.34	4.42	4.58	1	0	0	0	0	1	0	4.38	4.51	4.66	4.43	4.48	4.99	4.00
1387279_at	F11r	0	0	0	0	0	0	8.15	8.15	8.25	8.17	8.27	1	0	0	0	0	1	0	8.21	8.24	8.38	8.31	8.41	8.80	8.81
1387281_a_at	Pnck	0	0	0	0	0	0	5.27	5.32	5.24	5.18	5.10	1	0	0	0	0	1	0	5.84	5.90	5.89	5.89	5.87	6.51	6.06
1387282_at	Hspb8	0	0	0	0	0	0	4.28	4.22	4.10	4.25	4.32	1	0	0	0	0	1	1	6.39	6.48	6.69	6.78	6.88	7.53	7.22
1387283_at	Mx1	0	0	0	0	0	0	3.50	3.62	3.13	3.43	3.51	1	0	0	0	0	1	1	5.05	5.08	5.29	5.25	5.39	5.77	5.28
1387285_at	Atp2b2	0	0	0	0	0	0	3.81	3.62	3.50	3.46	3.80	1	0	0	0	0	0	1	5.36	5.19	5.57	5.33	5.20	4.89	4.80
1387286_at	Grm1	0	0	0	0	0	0	3.22	3.33	3.17	3.20	3.23	1	0	0	0	0	1	0	5.14	5.22	5.29	5.49	5.47	5.81	5.06
1387301_at	Fgf1	0	0	0	0	0	0	5.10	5.16	4.81	4.91	4.81	1	0	0	0	0	1	1	7.06	7.12	7.04	6.73	6.78	6.18	5.52
1387310_at	Atp2c2	0	0	0	0	0	0	4.34	4.36	4.07	4.50	4.21	1	0	0	0	0	1	1	4.85	5.04	5.04	4.96	5.07	5.57	5.58
1387312_a_at	Gck	0	0	0	0	0	0	6.56	6.71	6.95	6.31	6.74	1	0	0	0	0	0	1	8.50	8.65	8.78	8.80	8.79	8.05	7.10
1387356_at	Wfs1	0	0	0	0	0	0	6.29	6.31	6.27	6.13	6.59	1	0	0	0	0	1	1	6.61	6.61	6.82	6.78	6.71	7.32	7.22
1387359_at	Stx1a	0	0	0	0	0	0	4.74	4.84	5.07	4.94	4.81	1	0	0	0	0	1	1	7.47	7.53	7.67	7.71	7.80	8.51	7.80
1387360_at	Stx1a	0	0	0	0	0	0	5.58	5.62	5.72	6.00	5.71	1	0	0	0	0	1	1	7.83	7.86	8.03	8.17	8.18	8.89	8.54
1387377_a_at	Pak1	0	0	0	0	0	0	3.50	3.40	3.41	3.37	3.37	1	0	0	0	0	1	0	6.24	6.35	6.42	6.50	6.47	6.86	6.78
1387383_at	Gabbr2	0	0	0	0	0	0	6.17	6.37	6.63	6.14	6.56	1	0	0	0	0	0	1	8.06	8.03	8.18	8.14	8.20	7.59	6.80
1387395_at	Adora2b	0	0	0	0	0	0	5.44	5.57	5.66	5.25	5.29	1	0	0	0	0	1	0	6.85	6.81	6.89	6.97	6.93	7.53	7.67
1387404_at	Slc8a3	0	0	0	0	0	0	4.51	4.40	4.36	4.53	4.34	1	0	0	0	0	0	1	5.63	5.53	5.66	5.51	5.54	5.25	4.94
1387414_at	Duox2	0	0	0	0	0	0	3.53	3.73	3.42	3.37	3.43	1	0	1	1	1	1	0	6.60	6.32	6.00	5.34	5.42	5.23	6.00
1387435_at	St8sia3	0	0	0	0	0	0	6.07	6.30	6.18	6.04	6.52	1	0	0	0	0	1	1	8.69	8.56	8.77	8.93	8.66	7.22	6.41
1387450_at	Tgfa	0	0	0	0	0	0	4.79	4.76	4.91	4.62	4.72	1	0	0	0	0	1	1	8.23	8.19	8.28	8.35	8.42	8.98	8.85
1387483_at	Plcg2	0	0	0	0	0	0	3.91	4.01	3.70	3.93	3.90	1	0	0	0	0	0	1	4.56	4.45	4.62	4.52	4.52	5.00	5.20
1387488_a_at	Calcr	0	0	0	0	0	0	3.06	3.03	3.02	3.06	2.98	1	0	0	0	0	0	1	4.49	4.54	4.67	4.50	4.41	4.80	5.57
1387493_at	Akap5	0	0	0	0	0	0	3.36	3.41	3.19	3.55	3.29	1	0	0	0	0	0	1	3.76	3.83	3.80	3.66	3.72	4.22	4.47
1387498_a_at	Fgfr1	0	0	0	0	0	0	6.02	5.73	5.55	6.05	5.75	1	0	0	0	0	0	1	8.25	8.25	8.27	7.83	8.06	7.73	6.73
1387503_at	Cpn1	0	0	0	0	0	0	8.42	8.60	8.60	8.72	8.42	1	0	0	0	0	1	1	8.75	8.65	8.61	8.54	8.41	7.80	7.53
1387526_at	Gpr27	0	0	0	0	0	0	5.64	5.59	5.45	5.57	5.55	1	0	0	0	0	0	1	8.27	8.24	8.30	8.23	8.17	7.91	7.21
1387530_a_at	Fosl2	0	0	0	0	0	0	4.64	4.55	4.41	4.73	4.57	1	0	0	0	0	1	1	7.98	8.02	7.98	7.77	7.74	6.99	5.37
1387531_at	Msra	0	0	0	0	0	0	6.55	6.25	6.57	6.76	6.30	1	0	0	0	0	1	1	5.35	5.47	5.57	5.37	5.09	4.70	4.24
1387540_at	Brca1	0	0	0	0	0	0	3.24	3.22	3.16	3.34	3.19	1	0	0	0	0	0	1	5.14	5.07	5.35	5.08	5.12	4.62	4.60
1387557_s_at	Vps33a	0	0	0	0	0	0	6.38	6.58	6.56	6.76	6.50	1	0	0	0	0	0	1	7.63	7.69	7.73	7.64	7.60	7.12	6.72
1387562_at	Padi3	0	0	0	0	0	0	3.85	4.09	3.83	3.81	3.82	1	0	0	0	0	1	1	6.59	6.55	6.77	6.73	7.00	7.95	8.43
1387585_at	LOC100911721 /	0	0	0	0	0	0	4.42	4.58	4.39	4.55	4.48	1	0	0	0	0	0	1	7.30	7.33	7.35	7.45	7.29	6.96	6.00
1387622_at	Rfng	0	0	0	0	0	0	5.99	5.68	5.87	5.66	5.69	1	0	0	0	0	0	1	8.83	8.98	8.96	9.05	8.97	8.77	7.73
1387623_at	Stc1	0	0	0	0	0	0	4.20	4.12	4.13	4.14	4.05	1	0	0	0	1	1	0	10.52	10.57	10.75	10.88	11.18	11.47	10.64
1387663_at	Gmfb	0	0	0	0	0	0	5.16	5.22	5.08	5.05	5.05	1	0	0	0	0	1	1	6.65	6.52	6.49	6.51	6.43	5.74	5.53
1387669_a_at	Ephx1	0	0	0	0	0	0	7.19	6.85	7.17	7.46	6.95	1	0	0	0	0	1	0	6.89	7.01	7.00	7.12	7.15	7.68	7.38
1387675_at	Plau	0	0	0	0	0	0	5.27	4.98	5.10	4.87	5.20	1	0	0	0	0	0	1	4.51	4.57	4.41	4.46	4.66	4.82	5.13
1387676_at	Mark1	0	0	0	0	0	0	4.78	4.71	5.04	4.56	5.01	1	0	0	0	0	1	1	7.36	7.33	7.33	7.33	7.20	6.43	6.01
1387698_at	Kcnj11	0	0	0	0	0	0	7.92	8.03	8.35	7.83	7.96	1	0	0	0	0	1	1	10.45	10.40	10.36	10.17	10.03	8.80	7.86
1387702_at	Galr1	0	0	0	0	0	0	4.53	4.49	4.99	4.44	4.98	1	0	0	0	0	1	1	9.47	9.43	9.50	9.48	9.34	8.78	7.94
1387708_at	Adra2a	0	0	0	0	0	0	5.21	5.44	5.56	5.16	5.56	1	0	0	0	0	0	1	5.74	5.79	5.97	6.10	5.90	5.63	4.24
1387726_at	Cdx2	0	0	0	0	0	0	4.83	4.74	4.69	4.77	4.78	1	0	0	0	0	1	1	9.16	9.20	9.18	9.23	9.34	10.34	10.19
1387740_at	Pex11a	0	0	0	0	0	0	3.69	3.79	3.75	3.75	3.54	1	0	0	0	0	1	1	5.16	5.31	5.19	5.43	5.61	6.17	5.69
1387750_at	Twist1	0	0	0	0	0	0	3.63	3.43	3.26	3.19	3.29	1	0	0	0	0	0	1	7.37	7.26	7.31	7.36	7.22	6.84	6.21
1387762_s_at	Jund	0	0	0	0	0	0	4.60	4.95	4.38	4.85	4.47	1	0	0	0	0	0	1	6.93	6.69	6.98	7.11	6.89	6.48	6.50
1387766_a_at	Rbp2	0	0	0	0	0	0	3.04	3.17	3.14	3.12	3.10	1	0	0	0	0	1	1	6.41	6.50	6.39	6.10	6.10	5.70	5.62
1387776_at	Tgm2	0	0	0	0	0	0	6.36	6.20	6.10	6.20	6.31	1	0	0	0	0	1	0	6.63	6.59	6.80	6.81	6.93	7.27	6.74
1387794_at	Fcna	0	0	0	0	0	0	4.17	4.25	3.75	4.25	4.21	1	0	0	0	0	0	1	4.23	4.40	4.40	4.43	4.32	4.60	4.75
1387811_at	Agt	0	0	0	0	0	0	4.40	4.66	4.40	4.39	4.20	1	0	0	0	0	0	1	10.51	10.60	10.58	10.58	10.65	10.11	8.92
1387819_at	Cela1	0	0	0	0	0	0	14.44	14.43	14.30	14.35	14.39	1	0	0	0	0	1	1	4.31	4.21	4.27	4.15	4.35	5.53	6.14
1387824_at	Srek1	0	0	0	0	0	0	6.44	6.47	6.72	6.17	6.37	1	0	0	0	0	1	1	7.61	7.55	7.72	7.74	7.61	6.55	5.89
1387836_at	Ykt6	0	0	0	0	0	0	6.32	6.31	6.49	6.17	6.23	1	0	0	0	0	0	1	9.51	9.55	9.56	9.60	9.51	9.30	8.32
1387844_at	Lasp1	0	0	0	0	0	0	3.78	3.86	3.85	3.71	3.67	1	0	0	0	0	0	1	7.97	8.04	7.99	8.05	7.85	7.67	6.34
1387876_at	Stat5b	0	0	0	0	0	0	5.71	5.88	5.88	5.78	5.76	1	0	0	0	0	0	1	6.98	6.91	6.89	6.78	6.76	6.60	6.19
1387883_a_at	Tmsb10 /// Tms	0	0	0	0	0	0	11.36	11.13	11.33	11.28	11.57	1	0	0	0	0	1	0	11.05	11.22	11.15	11.17	11.30	11.77	11.48
1387889_at	Folr1	0	0	0	0	0	0	4.98	4.83	4.64	5.00	4.81	1	0	0	0	0	0	1	4.85	4.72	4.80	4.59	4.64	5.13	5.11
1387892_at	Tubb	0	0	0	0	0	0	8.45	8.42	8.83	8.87	8.60	1	0	0	0	0	0	1	12.18	12.16	12.21	12.16	12.07	11.80	11.38
1387895_s_at	Cdc20	0	0	0	0	0	0	5.09	5.03	5.01	5.24	5.17	1	0	0	0	0	1	1	9.90	9.82	9.86	9.77	9.56	8.46	8.27
1387898_at	Hspb6	0	0	0	0	0	0	6.70	6.79	6.82	6.83	6.78	1	0	0	0	0	1	1	8.73	8.56	8.56	8.45	8.24	7.22	7.08
1387900_at	Cdipt	0	0	0	0	0	0	8.38	8.27	8.50	8.43	8.45	1	0	0	0	0	0	1	10.19	10.21	10.23	10.23	10.16	9.86	9.50
1387907_at	Itpr1	0	0	0	0	0	0	7.56	7.54	7.96	7.41	7.47	1	0	0	0	0	1	1	8.09	7.99	8.26	8.32	8.38	8.89	9.25
1387917_at	Tor1aip1	0	0	0	0	0	0	7.72	7.92	7.89	7.94	7.71	1	0	0	0	0	1	0	8.49	8.32	8.34	8.26	8.08	7.81	7.87
1387922_at	Crispld2	0	0	0	0	0	0	5.04	4.81	4.72	4.74	4.66	1	0	0	0	0	0	1	4.58	4.53	4.58	4.41	4.49	4.88	4.84
1387932_at	Slc1a1	0	0	0	0	0	0	3.89	4.12	4.07	4.02	3.98	1	0	0	0	0	1	1	10.28	10.40	10.24	10.04	10.24	8.97	7.96
1387956_s_at	Cklf	0	0	0	0	0	0	3.74	3.63	3.65	3.57	3.85	1	0	0	0	0	1	1	6.16	6.22	6.33	6.43	6.57	7.60	7.47
1387959_at	Aspg	0	0	0	0	0	0	3.55	3.59	3.30	3.52	3.56	1	0	0	0	0	1	1	6.69	6.70	6.64	6.69	6.76	7.46	6.76
1387966_at	Asrgl1	0	0	0	0	0	0	5.15	5.24	5.28	5.01	5.25	1	0	0	0	0	1	1	8.29	8.42	8.58	8.62	8.69	9.42	9.76
1387972_at	Cdhr5	0	0	0	0	0	0	4.45	4.55	4.30	4.65	4.39	1	0	0	0	0	1	1	5.67	5.79	5.83	5.98	6.05	6.65	6.62
1387995_a_at	Ifitm3	0	0	0	0	0	0	8.62	8.52	8.45	8.30	8.46	1	0	0	0	1	1	0	4.79	5.00	4.93	5.20	5.41	5.44	4.93
1387999_at	Slc18a1	0	0	0	0	0	0	4.72	5.00	4.93	4.89	5.14	1	0	0	1	1	0	0	6.43	6.53	6.74	7.04	7.17	6.50	6.11
1388006_at	Muc13	0	0	0	0	0	0	4.32	4.41	4.22	4.27	4.00	1	0	0	0	0	1	1	9.87	9.83	9.86	9.88	9.80	8.27	7.94
1388010_at	Parpbp	0	0	0	0	0	0	4.11	4.16	4.29	4.14	4.24	1	0	0	0	0	1	1	8.27	8.17	8.28	8.20	8.02	6.99	6.82
1388020_a_at	Pde1c	0	0	0	0	0	0	3.96	4.05	3.91	3.95	4.16	1	0	0	0	0	0	1	6.20	6.36	6.42	6.32	6.29	5.86	5.49
1388028_at	Ttl	0	0	0	0	0	0	4.64	4.74	4.84	4.72	4.75	1	0	0	0	0	1	1	6.59	6.47	6.60	6.62	6.36	5.79	5.60
1388088_a_at	Usf2	0	0	0	0	0	0	4.71	4.52	4.88	4.91	4.67	1	0	0	0	0	1	1	7.05	7.08	7.24	7.16	6.99	6.12	5.61
1388099_a_at	Tfpt	0	0	0	0	0	0	4.40	4.59	4.58	4.61	4.61	1	0	0	0	0	0	1	7.14	7.21	7.30	7.34	7.16	6.84	6.17
1388101_at	Dpysl3	0	0	0	0	0	0	3.63	3.66	3.20	3.44	3.46	1	0	0	0	0	1	1	4.65	4.69	4.77	4.85	4.90	5.40	6.04
1388120_at	Pdcd6ip	0	0	0	0	0	0	7.05	6.97	6.90	7.02	7.16	1	0	0	0	0	0	1	8.68	8.60	8.73	8.62	8.50	8.11	7.78
1388157_at	Marcks	0	0	0	0	0	0	6.81	6.97	6.75	6.93	6.99	1	0	0	0	0	0	1	7.93	7.82	7.93	8.03	7.91	7.65	6.45
1388160_a_at	Idh3B	0	0	0	0	0	0	8.36	8.16	8.57	8.31	8.23	1	0	0	0	0	0	1	10.89	10.90	10.89	10.97	10.83	10.48	9.95
1388170_at	Kctd1	0	0	0	0	0	0	5.14	4.89	5.18	5.14	5.11	1	0	0	0	0	1	1	8.38	8.32	8.40	8.38	8.26	7.48	7.11
1388175_at	Clcnka	0	0	0	0	0	0	4.11	4.06	3.66	4.03	4.07	1	0	0	0	0	1	1	4.70	4.75	4.70	4.89	5.02	6.05	6.69
1388187_at	Camk2a	0	0	0	0	0	0	4.07	4.12	4.02	4.07	4.08	1	0	0	0	0	1	0	5.01	5.18	5.09	5.15	5.20	5.77	4.92
1388201_at	Bmp6	0	0	0	0	0	0	5.16	5.24	5.14	5.18	5.07	1	0	0	0	0	1	0	5.30	5.35	5.41	5.37	5.58	6.02	5.75
1388204_at	Mmp13	0	0	0	0	0	0	2.89	2.99	2.87	2.97	2.98	1	0	0	0	0	0	1	11.04	11.21	11.14	11.12	11.42	11.45	11.54
1388219_at	Htr5b	0	0	0	0	0	0	3.57	3.67	3.63	3.71	3.83	1	0	0	0	0	1	1	8.47	8.50	8.54	8.37	8.27	7.36	6.79
1388221_at	Slc24a3	0	0	0	0	0	0	4.00	3.96	3.80	3.85	3.90	1	0	0	0	0	1	0	6.83	6.84	7.05	7.40	7.41	7.56	6.07
1388224_at	Sec14l3	0	0	0	0	0	0	3.19	3.06	3.07	3.08	3.12	1	0	0	1	1	1	1	5.53	5.79	5.98	6.17	6.45	6.72	6.92
1388255_x_at	Hla-a	0	0	0	0	0	0	4.00	4.03	4.14	4.35	4.11	1	0	0	0	0	1	1	4.81	4.82	4.90	5.00	5.06	6.36	6.32
1388300_at	Mgst3	0	0	0	0	0	0	7.30	7.14	7.01	7.21	7.06	1	0	0	0	0	1	1	8.77	8.80	8.80	8.79	8.96	9.80	9.77
1388333_at	Rbx1	0	0	0	0	0	0	9.03	8.99	9.33	9.08	9.21	1	0	0	0	0	0	1	12.22	12.21	12.19	12.12	12.10	11.79	11.24
1388335_at	Tagln2	0	0	0	0	0	0	10.16	10.16	10.41	10.16	10.42	1	0	0	0	0	1	1	11.15	11.17	11.12	11.00	10.93	10.20	9.67
1388338_at	Ptpa	0	0	0	0	0	0	8.21	8.12	7.97	8.24	8.05	1	0	0	0	0	1	1	9.25	9.23	9.20	9.10	9.02	8.62	8.18
1388364_at	Ndufs3	0	0	0	0	0	0	9.00	8.94	8.87	9.33	8.74	1	0	0	0	0	0	1	10.45	10.44	10.42	10.46	10.36	9.94	9.33

Probeset ID	Gene Symbol	Islet Data Set (log2 ratio ≥0.263; FDR <20%) union n=5325 1=YES 0=NO	Islet DRlyp/lyp ND vs ND+Lp299v (log2 ratio ≥0.263; FDR<20%) n=429 1=YES 0=NO	Islet DRlyp/lyp ND vs HCD (log2 ratio ≥0.263; FDR<20%) n=2247 1=YES 0=NO	Islet DRlyp/lyp ND vs HCD+Lp299v (log2 ratio ≥0.263; FDR<20%) n=1595 1=YES 0=NO	Islet DRlyp/lyp HCD vs HCD+Lp299v (log2 ratio ≥0.263; FDR<20%) n=3026 1=YES 0=NO	Islet DRlyp/lyp ND vs Flyp/lyp ND (log2 ratio ≥ 0.263; FDR<20%) n=1950 1=YES 0=NO	Islet Mean Log2 Intensity DRlyp/lyp ND	Islet Mean Log2 Intensity DRlyp/lyp ND+Lp299v	Islet Mean Log2 Intensity DRlyp/lyp HCD	Islet Mean Log2 Intensity DRlyp/lyp HCD+Lp299v	Islet Mean Log2 Intensity Flyp40 ND	Rinm5f data union of all conditions n=3705 1=YES 0=NO	Rinm5f 30 µM propionate/ 6 µM butyrate vs Media (FDR<20%; 1.5 fold) n=3 1=YES 0=NO	Rinm5f 60 µM propionate/ 12 µM butyrate vs Media (FDR<20%; 1.5 fold) n=18 1=YES 0=NO	Rinm5f 120 µM propionate/ 24 µM butyrate vs Media (FDR<20%; 1.5 fold) n=164 1=YES 0=NO	Rinm5f 240 µM propionate /48 µM butyrate vs Media (FDR<20%; 1.5 fold) n=268 1=YES 0=NO	Rinm5f 1500 µM propionate/ 300 µM butyrate vs Media (FDR<20%; 1.5 fold) n=2491 1=YES 0=NO	Rinm5f 500 µM propionate/ 500 µM butyrate vs Media (FDR<20%; 1.5 fold) n=3001 1=YES 0=NO	Rinm5f Mean Log2 Intensity Media only	Rinm5f Mean Log2 Intensity 30 µM propionate/ 6 µM butyrate	Rinm5f Mean Log2 Intensity 60 µM propionate/ 12 µM butyrate	Rinm5f Mean Log2 Intensity 120 µM propionate/ 24 µM butyrate	Rinm5f Mean Log2 Intensity 240 µM propionate/ 48 µM butyrate	Rinm5f Mean Log2 Intensity 1500 µM propionate /300 µM butyrate	Rinm5f Mean Log2 Intensity 500 µM propionate/ 500 µM butyrate
1388385_at	Cryba2	0	0	0	0	0	0	7.41	7.74	7.66	7.24	7.79	1	0	0	0	0	1	1	7.07	7.02	7.04	7.02	6.93	5.69	5.01
1388394_at	Aars1	0	0	0	0	0	0	9.65	9.45	9.45	9.56	9.38	1	0	0	0	0	0	1	10.29	10.30	10.39	10.40	10.31	9.73	9.06
1388395_at	G0s2	0	0	0	0	0	0	6.38	5.94	5.90	6.22	5.92	1	0	0	0	0	1	1	8.99	9.00	8.84	8.49	8.69	7.82	7.98
1388407_at	Mcrip1	0	0	0	0	0	0	7.65	7.98	7.80	7.93	7.89	1	0	0	0	0	1	1	8.77	8.85	8.83	8.83	8.88	9.38	9.76
1388409_at	Zc3h7b	0	0	0	0	0	0	6.83	6.98	6.90	6.91	7.13	1	0	0	0	0	1	1	9.30	9.13	9.27	9.23	9.08	8.64	8.04
1388416_at	Lrp1	0	0	0	0	0	0	6.65	6.27	6.36	6.42	6.41	1	0	0	0	0	1	0	8.25	8.39	8.42	8.34	8.48	8.89	8.91
1388419_at	Cdk5rap2	0	0	0	0	0	0	6.23	6.09	6.01	5.96	6.20	1	0	0	0	0	1	1	7.63	7.58	7.56	7.55	7.40	6.60	6.29
1388423_at	C6orf136	0	0	0	0	0	0	5.56	5.53	5.47	5.85	5.60	1	0	0	0	0	1	0	8.48	8.36	8.51	8.41	8.33	7.89	7.71
1388432_at	Optn	0	0	0	0	0	0	6.91	6.69	6.90	6.68	6.73	1	0	0	0	0	0	1	7.71	7.78	7.82	7.86	7.89	8.18	8.53
1388436_at	Snrpa	0	0	0	0	0	0	6.73	7.07	6.63	7.05	7.06	1	0	0	0	0	0	1	9.21	9.21	9.17	9.10	9.00	8.65	8.29
1388441_at	LOC689574	0	0	0	0	0	0	7.87	7.85	8.24	7.99	8.00	1	0	0	0	0	1	1	9.28	9.34	9.42	9.44	9.50	10.21	10.36
1388443_at	Cdk2ap1	0	0	0	0	0	0	8.73	8.71	9.11	8.77	9.14	1	0	0	0	0	0	1	11.60	11.58	11.60	11.59	11.54	11.04	10.55
1388452_at	Spock2	0	0	0	0	0	0	6.85	7.08	7.05	6.96	7.18	1	0	0	0	0	1	1	7.54	7.64	7.63	7.58	7.62	6.86	6.50
1388456_at	S100a1	0	0	0	0	0	0	6.88	6.91	6.64	6.65	6.71	1	0	0	0	1	1	1	8.09	8.26	8.37	8.56	8.94	9.51	9.28
1388474_at	Ube2i	0	0	0	0	0	0	8.30	8.25	8.69	8.24	8.44	1	0	0	0	0	1	0	8.79	8.79	8.96	9.08	9.09	9.40	9.27
1388479_at	---	0	0	0	0	0	0	5.14	5.06	4.95	5.00	4.96	1	0	0	0	0	1	1	6.82	7.03	7.06	7.36	7.31	8.17	8.24
1388484_at	Ube2c	0	0	0	0	0	0	6.01	5.87	6.19	6.09	6.39	1	0	0	0	0	1	1	10.97	10.89	10.88	10.86	10.73	9.57	9.50
1388488_at	Lsm3	0	0	0	0	0	0	8.09	8.09	8.19	8.13	8.19	1	0	0	0	0	1	1	9.95	9.82	9.84	9.75	9.63	8.94	8.91
1388497_at	Acot13	0	0	0	0	0	0	6.34	6.15	5.83	6.14	6.10	1	0	0	0	0	1	1	7.20	7.25	7.40	7.48	7.47	8.48	8.38
1388513_at	Mvb12b	0	0	0	0	0	0	6.43	6.45	6.11	6.46	6.38	1	0	0	0	0	0	1	8.13	8.09	8.15	8.06	8.00	7.58	7.38
1388526_at	Gstz1	0	0	0	0	0	0	9.46	9.40	9.68	9.13	9.39	1	0	0	0	0	1	1	10.31	10.28	10.27	10.26	10.08	9.13	8.72
1388534_at	Slc31a1	0	0	0	0	0	0	6.82	6.54	6.82	7.03	6.91	1	0	0	0	0	1	1	7.28	7.39	7.40	7.49	7.50	8.11	8.50
1388543_at	Plpp4	0	0	0	0	0	0	4.74	4.78	4.58	4.70	4.49	1	0	0	0	0	1	0	6.17	6.18	6.23	6.41	6.50	7.13	6.83
1388556_at	Stx6	0	0	0	0	0	0	6.91	6.86	7.25	6.97	7.08	1	0	0	0	0	1	1	7.49	7.63	7.70	7.73	7.65	8.08	8.43
1388598_at	LOC728392	0	0	0	0	0	0	3.79	3.81	3.83	3.77	3.59	1	0	0	0	0	1	1	5.85	5.96	6.18	6.32	6.21	6.82	7.02
1388618_at	Nid2	0	0	0	0	0	0	6.05	5.96	6.41	6.12	6.40	1	0	0	0	0	0	1	5.19	5.09	5.01	4.86	4.88	5.30	6.50
1388620_at	Tada3	0	0	0	0	0	0	6.56	6.51	6.56	6.59	6.62	1	0	0	0	0	0	1	7.90	7.90	7.99	7.90	7.82	7.36	7.04
1388624_at	C9orf16	0	0	0	0	0	0	7.40	7.49	7.02	7.24	7.45	1	0	0	0	0	1	0	9.31	9.32	9.37	9.50	9.58	10.01	9.82
1388634_at	Pgm1	0	0	0	0	0	0	8.01	8.16	8.14	8.07	8.20	1	0	0	0	0	1	1	10.33	10.32	10.19	9.96	9.88	9.46	9.28
1388661_at	Mtfp1	0	0	0	0	0	0	5.10	5.20	5.05	5.05	5.04	1	0	0	0	0	0	1	6.36	6.47	6.52	6.53	6.58	6.94	6.93
1388666_at	Enc1	0	0	0	0	0	0	7.57	7.36	7.99	7.59	7.46	1	0	0	0	0	1	0	9.79	9.97	10.03	10.02	10.17	10.52	10.27
1388668_at	---	0	0	0	0	0	0	6.67	6.56	6.71	6.88	6.74	1	0	0	0	0	0	1	8.77	8.70	8.84	8.79	8.75	8.19	7.68
1388675_at	Spns1	0	0	0	0	0	0	7.12	7.11	6.91	7.29	6.86	1	0	0	0	0	0	1	8.91	8.91	8.93	8.80	8.81	8.53	8.07
1388686_at	Rcan1	0	0	0	0	0	0	6.27	6.03	6.38	5.96	6.28	1	0	0	0	0	1	0	8.38	8.46	8.60	8.68	8.62	9.03	8.78
1388698_at	Ecm1	0	0	0	0	0	0	5.04	4.88	4.65	4.66	4.78	1	0	0	0	0	0	1	7.30	7.33	7.37	7.38	7.42	7.77	7.67
1388703_at	Esam	0	0	0	0	0	0	7.34	7.14	7.27	7.05	7.31	1	0	0	0	0	1	1	10.51	10.53	10.55	10.47	10.41	9.75	9.19
1388705_at	Selenom	0	0	0	0	0	0	9.00	9.46	9.17	8.99	9.25	1	0	0	0	0	1	1	10.20	10.34	10.28	10.31	10.34	10.86	10.81
1388721_at	Hspb8	0	0	0	0	0	0	3.36	3.47	3.10	3.35	3.34	1	0	0	0	0	1	1	7.18	7.32	7.36	7.48	7.63	8.21	8.18
1388729_at	Rras	0	0	0	0	0	0	7.10	6.89	6.78	7.01	6.92	1	0	0	0	0	1	1	6.84	6.99	7.00	6.81	6.96	7.67	8.14
1388735_at	---	0	0	0	0	0	0	4.17	4.33	4.11	4.22	4.18	1	0	0	0	0	1	1	6.05	5.96	6.09	6.07	5.91	5.18	4.97
1388753_at	Sulf2	0	0	0	0	0	0	6.28	6.19	6.23	6.01	6.53	1	0	0	0	0	1	1	8.71	8.82	8.95	9.00	9.01	9.62	9.61
1388761_at	Hdac1	0	0	0	0	0	0	7.86	7.76	8.14	7.72	8.04	1	0	0	0	0	1	1	9.54	9.58	9.71	9.79	9.86	10.20	10.34
1388763_at	Cnn2	0	0	0	0	0	0	7.05	7.21	6.99	6.99	7.27	1	0	0	0	0	1	1	5.35	5.39	5.53	5.30	5.31	6.22	6.87
1388778_at	Ppm1k	0	0	0	0	0	0	6.66	6.75	6.53	6.60	6.35	1	0	0	0	0	1	1	6.93	6.92	7.07	6.99	7.15	7.60	7.68
1388786_at	SYNPO	0	0	0	0	0	0	6.38	6.28	6.19	6.54	6.07	1	0	0	0	0	1	1	7.34	7.39	7.62	7.65	7.72	8.75	8.68
1388811_at	---	0	0	0	0	0	0	4.20	4.21	4.06	4.21	4.18	1	0	0	0	0	0	1	6.53	6.58	6.68	6.59	6.53	6.96	7.31
1388812_at	Tp53i13	0	0	0	0	0	0	7.82	7.89	7.66	7.80	7.76	1	0	0	0	0	1	0	8.19	8.26	8.29	8.37	8.44	8.85	8.73
1388816_at	Dlg1	0	0	0	0	0	0	8.23	8.08	8.24	8.10	8.15	1	0	0	0	0	1	0	8.05	8.14	8.33	8.49	8.50	8.68	8.86
1388833_at	Pole3	0	0	0	0	0	0	6.55	6.67	6.51	6.79	6.65	1	0	0	0	0	0	1	7.23	7.24	7.26	7.04	7.12	6.67	6.71
1388837_at	Slc44a2	0	0	0	0	0	0	6.37	6.25	6.27	6.43	6.34	1	0	0	0	0	1	1	7.43	7.53	7.66	7.63	7.72	8.33	8.34
1388840_at	Plekhg2	0	0	0	0	0	0	3.98	4.14	3.84	4.05	3.87	1	0	0	0	0	0	1	4.80	4.85	4.88	4.60	4.71	5.21	5.70
1388884_at	Eci2 /// Eci3	0	0	0	0	0	0	5.47	5.50	5.40	5.47	5.43	1	0	0	0	0	1	1	6.36	6.43	6.58	6.80	6.85	7.87	7.68
1388889_at	Ermp1	0	0	0	0	0	0	6.89	6.73	6.90	7.02	7.10	1	0	0	0	0	1	1	9.66	9.69	9.76	9.73	9.83	10.30	10.52
1388891_at	Parvb	0	0	0	0	0	0	5.08	5.14	5.33	5.21	5.44	1	0	0	0	0	1	1	7.83	7.88	7.95	8.03	8.00	8.42	8.51
1388909_at	Oxnad1	0	0	0	0	0	0	7.45	7.13	7.36	7.34	7.46	1	0	0	0	0	0	1	9.84	9.80	9.89	9.89	9.77	9.35	8.63
1388917_at	Myo1d	0	0	0	0	0	0	8.37	8.33	8.66	8.24	8.73	1	0	0	0	0	1	1	9.28	9.29	9.39	9.41	9.54	9.94	10.03
1388920_at	Bmp6	0	0	0	0	0	0	4.06	4.30	3.98	4.15	4.24	1	0	0	0	0	1	1	5.37	5.37	5.63	5.54	5.64	6.49	6.86
1388922_at	Aip	0	0	0	0	0	0	7.68	7.75	7.75	7.69	7.71	1	0	0	0	0	0	1	9.22	9.16	9.14	9.08	8.97	8.69	8.24
1388923_at	Haus8	0	0	0	0	0	0	5.32	5.49	5.37	5.63	5.47	1	0	0	0	0	1	0	7.71	7.62	7.73	7.65	7.49	7.06	6.88
1388927_at	Ift27	0	0	0	0	0	0	6.64	6.91	6.49	7.03	6.62	1	0	0	0	0	1	1	9.32	9.32	9.30	9.32	9.41	10.00	10.03
1388930_at	Tmem123	0	0	0	0	0	0	9.60	9.52	10.09	9.40	9.94	1	0	0	0	0	1	1	8.33	8.35	8.32	8.50	8.57	9.23	9.03
1388932_at	Lama5	0	0	0	0	0	0	6.77	6.69	6.71	7.02	6.56	1	0	0	0	0	1	1	7.82	7.91	8.04	8.13	8.25	8.65	8.43
1388953_at	Gnl3	0	0	0	0	0	0	8.19	7.87	8.49	8.30	7.76	1	0	0	0	0	0	1	10.34	10.36	10.39	10.50	10.42	9.92	9.37
1388963_at	Astn1	0	0	0	0	0	0	4.76	4.93	5.31	4.82	5.20	1	0	0	0	0	0	1	8.58	8.69	8.77	8.82	8.80	8.30	7.71
1389007_at	Fam189a2	0	0	0	0	0	0	4.10	4.20	3.95	4.04	4.29	1	0	0	0	0	0	1	4.63	4.76	4.81	4.60	5.03	5.19	5.93
1389010_at	Lta4h	0	0	0	0	0	0	7.93	7.79	8.12	8.15	7.96	1	0	0	0	0	0	1	10.40	10.43	10.47	10.44	10.43	10.83	10.93
1389015_at	Fundc2	0	0	0	0	0	0	7.72	7.87	7.95	7.72	8.05	1	0	0	0	0	0	1	10.60	10.63	10.71	10.81	10.75	10.33	9.74
1389018_at	Ndufa4l2	0	0	0	0	0	0	5.68	5.80	5.69	5.73	5.82	1	0	0	0	0	1	1	7.15	7.14	7.16	6.99	7.04	6.26	5.91
1389039_at	Nrp2	0	0	0	0	0	0	4.65	4.82	4.92	4.59	4.91	1	0	0	0	0	1	1	5.24	5.27	5.38	5.39	5.34	6.61	7.04
1389050_at	Dusp3	0	0	0	0	0	0	6.98	7.09	7.07	7.23	7.20	1	0	0	0	0	1	1	7.80	7.91	7.99	8.07	8.17	8.48	9.05
1389085_at	---	0	0	0	0	0	0	7.97	7.78	7.77	8.25	7.65	1	0	0	0	0	1	1	8.00	7.99	8.09	8.06	8.12	8.66	9.04
1389093_at	Pak1	0	0	0	0	0	0	5.48	5.79	5.77	5.63	5.88	1	0	0	0	0	1	1	8.41	8.46	8.51	8.63	8.70	9.02	9.05
1389102_at	Dock1	0	0	0	0	0	0	7.48	7.24	7.28	7.59	7.27	1	0	0	0	0	1	1	7.36	7.37	7.57	7.71	7.88	8.07	7.92
1389103_at	Gins4	0	0	0	0	0	0	4.96	5.01	4.90	4.99	4.96	1	0	0	0	0	0	1	9.01	8.98	9.06	9.01	8.96	8.43	8.34
1389118_s_at	S100a14	0	0	0	0	0	0	3.31	3.34	3.20	3.23	3.24	1	0	0	0	0	0	1	3.95	4.03	4.02	3.92	4.03	4.34	4.57
1389164_at	Herc3	0	0	0	0	0	0	6.82	7.05	7.33	6.80	7.27	1	0	0	0	0	1	1	8.81	8.80	8.95	9.07	9.14	9.44	9.48
1389169_at	Pgrmc2	0	0	0	0	0	0	7.96	8.03	8.32	7.89	8.17	1	0	0	0	0	1	0	9.88	9.96	9.97	9.91	10.05	10.48	10.24
1389172_at	Pdlim5	0	0	0	0	0	0	6.60	6.53	6.69	6.79	6.73	1	0	0	0	0	1	1	6.37	6.46	6.44	6.44	6.52	6.98	7.72
1389184_at	Rpp30	0	0	0	0	0	0	5.40	5.37	5.44	5.40	5.35	1	0	0	0	0	1	1	8.87	8.84	8.85	8.91	8.81	8.22	7.76
1389215_at	Sephs1	0	0	0	0	0	0	5.91	5.62	6.32	5.91	5.90	1	0	0	0	0	1	0	8.66	8.57	8.61	8.66	8.50	8.05	7.96
1389244_x_at	Cxcr4	0	0	0	0	0	0	6.01	5.79	6.01	5.54	6.09	1	0	0	0	0	0	1	6.56	6.60	6.60	6.62	6.56	7.12	7.49
1389248_at	Galk1	0	0	0	0	0	0	6.60	6.51	6.18	6.65	6.36	1	0	0	0	0	0	1	6.71	6.83	6.81	6.75	6.65	7.01	7.54
1389250_at	---	0	0	0	0	0	0	4.58	4.50	4.55	4.85	4.45	1	0	0	0	0	1	1	5.76	5.81	5.90	5.70	5.83	6.38	6.15
1389251_at	Nudt7	0	0	0	0	0	0	9.22	9.07	9.56	9.17	8.99	1	0	0	0	0	1	1	7.78	7.82	7.91	7.89	7.98	8.37	8.38
1389257_at	Lancl2	0	0	0	0	0	0	7.58	7.50	7.74	7.35	7.62	1	0	0	0	0	1	1	8.66	8.59	8.64	8.63	8.42	7.97	7.78
1389260_at	Ubxn6	0	0	0	0	0	0	7.36	7.42	7.35	7.80	7.03	1	0	0	0	0	0	1	8.56	8.58	8.61	8.59	8.50	8.21	7.82
1389263_at	Rai14	0	0	0	0	0	0	4.98	5.05	4.96	5.18	4.97	1	0	0	0	0	0	1	6.55	6.56	6.62	6.44	6.50	7.03	6.94
1389265_at	Gbe1	0	0	0	0	0	0	8.25	8.19	8.46	8.28	8.32	1	0	0	1	0	1	1	10.72	10.71	10.51	10.11	10.20	9.74	9.82
1389266_at	---	0	0	0	0	0	0	3.88	3.90	3.81	3.95	3.87	1	0	0	0	0	0	1	5.18	5.22	5.46	5.48	5.54	5.61	5.75
1389267_at	---	0	0	0	0	0	0	3.52	3.50	3.71	3.62	3.64	1	0	0	0	0	1	0	5.28	5.45	5.63	5.75	5.64	5.87	5.46
1389276_at	L3mbtl3	0	0	0	0	0	0	4.84	4.64	4.80	5.12	4.88	1	0	0	0	0	1	1	6.84	6.89	6.82	6.73	6.77	7.66	7.64
1389277_at	Ecpas	0	0	0	0	0	0	8.39	8.35	8.64	8.19	8.43	1	0	0	0	0	1	0	9.19	9.11	9.19	9.19	9.10	8.60	8.39
1389285_at	Mpp3	0	0	0	0	0	0	6.20	6.20	6.29	6.05	6.35	1	0	0	0	0	1	1	7.87	7.99	8.06	8.13	8.20	8.65	8.78
1389295_at	Olfml2b	0	0	0	0	0	0	6.13	6.23	6.10	5.93	6.18	1	0	0	0	0	0	1	5.98	5.90	5.84	5.69	5.70	6.21	6.49
1389302_at	Ino80c	0	0	0	0	0	0	5.58	5.59	6.00	5.59	5.50	1	0	0	0	0	0	1	8.59	8.55	8.61	8.68	8.57	8.19	7.40
1389311_at	Ppm1a	0	0	0	0	0	0	5.57	5.43	5.98	5.55	5.25	1	0	0	0	0	1	1	6.27	6.36	6.60	6.65	6.71	6.97	6.94
1389356_at	Xrcc3	0	0	0	0	0	0	4.20	4.30	3.97	4.33	4.34	1	0	0	0	0	1	1	6.52	6.59	6.63	6.16	6.20	5.46	5.10
1389359_at	Smc4	0	0	0	0	0	0	3.38	3.17	3.67	3.43	3.46	1	0	0	0	0	0	1	6.40	6.49	6.50	6.55	6.60	6.01	5.43
1389368_at	Cnksr3	0	0	0	0	0	0	5.91	5.88	6.04	6.08	5.67	1	0	0	0	0	1	0	8.78	8.80	8.75	8.63	8.73	9.41	9.41
1389379_at	---	0	0	0	0	0	0	4.12	3.95	4.06	4.05	3.99	1	0	0	0	0	0	1	5.18	5.13	5.27	5.16	5.21	5.46	5.66
1389381_at	Sqstm1	0	0	0	0	0	0	8.50	8.43	8.76	8.20	8.08	1	0	0	0	0	0	1	9.94	9.98	10.10	10.18	10.01	9.64	9.40
1389394_at	Itga1	0	0	0	0	0	0	8.30	8.20	8.31	8.25	8.01	1	0	0	0	0	1	1	7.09	7.16	7.17	7.11	7.29	7.84	7.99
1389409_at	Tes	0	0	0	0	0	0	8.17	8.19	8.26	7.98	8.49	1	0	0	0	0	0	1	9.33	9.33	9.29	9.16	9.19	9.77	10.43
1389420_at	Stap2	0	0	0	0	0	0	5.47	5.26	5.24	5.33	5.34	1	0	0	0	0	1	1	5.64	5.69	5.71	5.78	6.09	6.76	7.09
1389422_at	Magt1	0	0	0	0	0	0	10.10	10.03	10.47	10.21	10.43	1	0	0	0	0	1	0	9.80	9.85	9.96	10.04	10.19	10.51	10.42
1389425_at	Nt5c	0	0	0	0	0	0	4.48	4.44	4.32	4.47	4.37	1	0	0	0	0	1	1	8.12	8.07	8.04	7.98	7.88	7.31	6.82
1389430_at	Hsd17b7	0	0	0	0	0	0	4.20	4.20	4.29	4.37	4.53	1	0	0	0	0	0	1	5.28	5.18	5.64	5.53	5.57	5.83	5.99
1389434_at	Bbs9	0	0	0	0	0	0	5.81	5.67	5.71	5.79	5.81	1	0	0	0	0	0	1	7.21	7.25	7.34	7.37	7.43	7.70	7.83
1389440_at	Cdca5	0	0	0	0	0	0	4.24	4.11	4.21	4.25	4.22	1	0	0	0	0	1	1	8.78	8.62	8.68	8.58	8.49	7.31	7.21
1389448_at	Septin4	0	0	0	0	0	0	6.56	6.55	6.75	6.47	6.49	1	0	0	0	0	1	1	7.68	7.81	7.96	8.05	8.15	8.59	8.39
1389463_at	Prkar1b	0	0	0	0	0	0	8.57	8.47	8.69	8.57	8.61	1	0	0	0	0	1	1	10.12	10.07	10.18	10.12	9.97	9.18	8.64
1389484_at	Coq4	0	0	0	0	0	0	5.55	5.59	5.34	5.68	5.55	1	0	0	0	0	0	1	7.88	7.88	7.81	7.70	7.70	7.36	6.94
1389487_at	Dnajb12	0	0	0	0	0	0	6.31	6.02	6.16	6.51	5.92	1	0	0	0	0	1	1	7.29	7.29	7.37	7.34	7.14	6.68	5.99
1389490_at	Cd248	0	0	0	0	0	0	5.72	5.53	5.25	5.59	5.61	1	0	0	0	0	1	1	5.63	5.64	5.90	5.70	5.89	6.86	7.97
1389497_at	Smyd5	0	0	0	0	0	0	6.00	6.07	5.81	6.14	5.88	1	0	0	0	0	1	1	8.18	8.16	8.25	8.01	7.94	7.53	7.27
1389501_at	Pde6g	0	0	0	0	0	0	6.15	6.19	5.80	6.25	6.07	1	0	0	1	0	0	0	6.71	6.63	6.38	6.09	6.16	6.35	6.18
1389506_x_at	Cdc20	0	0	0	0	0	0	3.45	3.35	3.42	3.53	3.55	1	0	0	0	0	1	1	7.95	7.91	8.00	7.90	7.71	6.33	6.13
1389538_at	Nfkbia	0	0	0	0	0	0	8.11	8.24	7.96	7.79	7.68	1	0	0	0	0	1	1	9.28	9.27	9.26	9.20	9.13	8.69	8.21
1389542_at	Cenpe	0	0	0	0	0	0	4.39	4.30	4.10	4.37	4.23	1	0	0	0	0	1	1	7.65	7.70	7.82	7.83	7.61	6.74	7.13
1389546_at	Amotl2	0	0	0	0	0	0	6.01	6.10	5.72	6.24	6.11	1	0	0	0	1	1	0	5.05	4.67	4.72	4.48	4.31	4.39	4.66
1389547_at	Pphln1	0	0	0	0	0	0	7.00	6.71	7.01	6.91	7.04	1	0	0	0	0	1	0	9.27	9.29	9.39	9.53	9.55	9.90	9.75
1389560_at	Chmp3	0	0	0	0	0	0	8.57	8.51	9.11	8.47	8.69	1	0	0	0	0	0	1	9.49	9.44	9.47	9.46	9.32	9.00	8.60
1389561_at	Tmem218	0	0	0	0	0	0	5.93	5.89	6.00	5.92	5.95	1	0	0	0	0	1	1	7.68	7.77	7.82	7.87	8.00	8.38	8.35
1389566_at	Ccnb2	0	0	0	0	0	0	6.34	6.20	6.60	6.44	6.58	1	0	0	0	0	1	1	9.74	9.72	9.73	9.67	9.48	8.39	8.41
1389572_at	Me3	0	0	0	0	0	0	5.82	5.85	5.94	5.83	5.60	1	0	0	0	0	1	1	5.52	5.50	5.45	5.14	5.09	4.92	4.58
1389578_at	Yrdc	0	0	0	0	0	0	6.32	6.29	6.83	6.17	6.56	1	0	0	0	0	0	1	9.25	9.28	9.29	9.30	9.18	8.89	8.43
1389605_at	Jakmip1	0	0	0	0	0	0	3.49	3.61	3.24	3.37	3.46	1	0	0	1	1	1	1	5.68	5.86	6.01	6.33	6.58	7.71	8.65
1389611_at	Vldlr	0	0	0	0	0	0	8.66	8.44	8.92	8.94	8.94	1	0	0	0	0	1	1	10.78	10.74	10.55	10.27	10.29	9.53	9.24
1389613_at	Atp8a1	0	0	0	0	0	0	7.14	7.15	7.44	7.00	7.21	1	0	0	0	0	0	1	9.07	9.17	9.14	9.21	9.19	9.59	9.99
1389625_at	Chchd4	0	0	0	0	0	0	4.63	4.54	4.60	4.18	4.47	1	0	0	0	0	0	1	8.54	8.55	8.64	8.71	8.58	8.26	7.50
1389626_at	Vps33a	0	0	0	0	0	0	4.78	4.72	5.04	5.03	4.87	1	0	0	0	0	1	1	6.09	6.00	6.22	6.13	6.03	5.36	5.08
1389628_at	Plcd3	0	0	0	0	0	0	6.06	6.19	5.81	5.90	5.99	1	0	0	0	0	1	0	7.02	7.14	7.03	7.04	7.18	7.63	7.12
1389637_at	---	0	0	0	0	0	0	4.78	4.84	4.65	4.66	4.45	1	0	0	0	0	1	1	8.12	8.27	8.45	8.53	8.56	8.80	8.85
1389648_at	Ripk4	0	0	0	0	0	0	7.67	7.64	7.81	7.75	7.62	1	0	0	0	0	1	1	9.27	9.29	9.40	9.42	9.44	9.90	9.92
1389653_at	Pcdhb9	0	0	0	0	0	0	2.82	3.05	3.13	2.83	2.93	1	0	0	1	1	1	0	6.36	6.75	6.91	7.15	7.19	7.14	4.93

Probeset ID	Gene Symbol	Islet Data Set (log2 ratio ≥0.263; FDR <20%) union n=5325 1=YES 0=NO	Islet DRlyp/lyp ND vs ND+Lp299v (log2 ratio ≥0.263; FDR<20%) n=429 1=YES 0=NO	Islet DRlyp/lyp ND vs HCD (log2 ratio ≥0.263; FDR<20%) n=2247 1=YES 0=NO	Islet DRlyp/lyp ND vs HCD+Lp299v (log2 ratio ≥0.263; FDR<20%) n=1595 1=YES 0=NO	Islet DRlyp/lyp HCD vs HCD+Lp299v (log2 ratio ≥0.263; FDR<20%) n=3026 1=YES 0=NO	Islet DRlyp/lyp ND vs Flyp/lyp ND (log2 ratio ≥ 0.263; FDR<20%) n=1950 1=YES 0=NO	Islet Mean Log2 Intensity DRlyp/lyp ND	Islet Mean Log2 Intensity DRlyp/lyp ND+Lp299v	Islet Mean Log2 Intensity DRlyp/lyp HCD	Islet Mean Log2 Intensity DRlyp/lyp HCD+Lp299v	Islet Mean Log2 Intensity Flyp40 ND	Rinm5f data union of all conditions n=3705 1=YES 0=NO	Rinm5f 30 µM propionate/ 6 µM butyrate vs Media (FDR<20%; 1.5 fold) n=3 1=YES 0=NO	Rinm5f 60 µM propionate/ 12 µM butyrate vs Media (FDR<20%; 1.5 fold) n=18 1=YES 0=NO	Rinm5f 120 µM propionate/ 24 µM butyrate vs Media (FDR<20%; 1.5 fold) n=164 1=YES 0=NO	Rinm5f 240 µM propionate /48 µM butyrate vs Media (FDR<20%; 1.5 fold) n=268 1=YES 0=NO	Rinm5f 1500 µM propionate/ 300 µM butyrate vs Media (FDR<20%; 1.5 fold) n=2491 1=YES 0=NO	Rinm5f 500 µM propionate/ 500 µM butyrate vs Media (FDR<20%; 1.5 fold) n=3001 1=YES 0=NO	Rinm5f Mean Log2 Intensity Media only	Rinm5f Mean Log2 Intensity 30 µM propionate/ 6 µM butyrate	Rinm5f Mean Log2 Intensity 60 µM propionate/ 12 µM butyrate	Rinm5f Mean Log2 Intensity 120 µM propionate/ 24 µM butyrate	Rinm5f Mean Log2 Intensity 240 µM propionate/ 48 µM butyrate	Rinm5f Mean Log2 Intensity 1500 µM propionate /300 µM butyrate	Rinm5f Mean Log2 Intensity 500 µM propionate/ 500 µM butyrate
1389656_at	Tspan15	0	0	0	0	0	0	6.71	6.71	6.59	6.76	6.39	1	0	0	0	0	1	0	7.69	7.69	7.63	7.55	7.64	8.28	8.27
1389668_at	Spc25	0	0	0	0	0	0	5.47	5.36	5.76	5.82	5.94	1	0	0	0	0	0	1	9.17	9.11	9.28	9.39	9.32	8.67	8.47
1389680_at	Ell2	0	0	0	0	0	0	8.13	8.11	8.13	8.09	8.16	1	0	0	1	0	1	0	8.21	8.24	8.52	8.83	8.75	8.80	8.41
1389687_at	LOC103690149	0	0	0	0	0	0	7.47	7.53	7.91	7.51	7.50	1	0	0	0	0	0	1	8.38	8.33	8.48	8.45	8.53	8.89	9.23
1389695_at	Fam20c	0	0	0	0	0	0	7.79	7.94	8.03	7.86	7.86	1	0	0	0	0	1	1	9.98	9.96	10.11	10.21	10.11	10.65	10.84
1389701_at	---	0	0	0	0	0	0	3.48	3.54	3.49	3.77	3.70	1	0	0	0	0	0	1	6.32	6.34	6.50	6.59	6.60	6.25	5.83
1389707_at	Hcn4	0	0	0	0	0	0	6.00	5.98	6.26	6.07	6.34	1	0	0	0	0	0	1	7.86	7.92	7.89	7.60	7.67	7.27	7.08
1389712_at	Ebf3	0	0	0	0	0	0	4.12	3.82	3.72	3.81	3.88	1	0	0	0	0	1	1	8.57	8.52	8.80	8.99	8.95	9.40	9.03
1389715_at	Ypel4	0	0	0	0	0	0	4.41	4.71	4.75	4.28	4.61	1	0	0	0	0	0	1	8.70	8.87	8.84	8.79	8.75	8.25	7.52
1389718_at	Asb4	0	0	0	0	0	0	3.33	3.52	3.32	3.44	3.42	1	0	0	0	1	1	0	9.99	10.28	10.26	10.24	10.58	11.10	11.19
1389720_at	---	0	0	0	0	0	0	3.28	3.31	3.12	3.17	3.22	1	0	0	0	0	1	1	5.33	5.34	5.45	5.39	5.49	5.93	6.07
1389726_at	Lsm11	0	0	0	0	0	0	6.40	6.20	6.30	6.41	6.45	1	0	0	0	0	1	1	6.81	6.86	6.83	6.60	6.56	6.10	5.81
1389728_at	Meis2	0	0	0	0	0	0	6.92	6.85	7.35	7.15	7.13	1	0	0	0	0	1	1	6.43	6.29	6.45	6.48	6.58	7.61	7.69
1389734_x_at	H2-t24	0	0	0	0	0	0	8.71	8.85	8.77	8.76	8.72	1	0	0	0	0	0	1	4.74	4.75	4.60	4.52	4.55	5.02	5.03
1389737_at	Dtna	0	0	0	0	0	0	4.67	4.62	4.41	4.73	4.34	1	0	0	0	0	0	1	7.34	7.31	7.42	7.32	7.24	6.89	6.13
1389741_at	Cers4	0	0	0	0	0	0	4.65	4.70	4.90	5.01	4.73	1	0	0	0	0	1	0	6.63	6.54	6.43	6.40	6.42	5.97	5.31
1389742_at	Znf217	0	0	0	0	0	0	5.81	5.82	5.83	5.91	5.81	1	0	0	0	0	1	1	7.10	7.14	7.34	7.39	7.49	8.32	8.67
1389745_at	---	0	0	0	0	0	0	2.95	2.88	2.85	2.85	2.98	1	0	0	0	1	1	1	3.67	3.86	3.88	3.95	4.36	4.41	4.14
1389752_at	Sorcs3	0	0	0	0	0	0	3.24	3.28	3.28	3.43	3.42	1	0	0	0	0	1	1	4.89	4.89	4.89	4.90	4.97	5.87	6.08
1389754_at	---	0	0	0	0	0	0	3.70	3.54	3.50	3.51	3.51	1	0	0	0	0	0	1	4.38	4.13	4.33	4.37	4.31	4.72	5.17
1389759_at	Celsr1	0	0	0	0	0	0	3.98	3.83	3.91	4.04	4.11	1	0	0	0	0	1	1	5.10	5.23	5.26	5.27	5.55	6.99	7.42
1389786_at	Wwp1	0	0	0	0	0	0	3.95	3.87	3.98	3.97	3.98	1	0	0	1	1	1	0	5.09	5.30	5.55	5.70	5.69	6.11	5.45
1389787_at	Ptk7	0	0	0	0	0	0	5.84	5.91	5.84	6.20	5.85	1	0	0	0	0	0	1	4.92	4.98	4.92	4.76	4.83	5.28	5.81
1389793_at	Gatad2a	0	0	0	0	0	0	5.40	5.32	5.46	5.20	5.05	1	0	0	0	0	1	1	7.76	7.89	7.95	8.15	8.27	8.64	8.69
1389796_at	---	0	0	0	0	0	0	4.60	4.48	4.52	4.72	4.35	1	0	0	0	1	1	0	5.01	5.18	5.15	5.55	5.67	5.88	6.13
1389811_at	Rasgef1c	0	0	0	0	0	0	4.05	4.11	4.02	4.02	3.78	1	0	0	0	1	1	1	5.34	5.53	5.73	5.90	6.04	6.70	6.43
1389852_at	Cenpm	0	0	0	0	0	0	4.93	5.08	4.78	5.11	5.06	1	0	0	0	0	1	1	7.71	7.62	7.61	7.54	7.36	7.10	7.16
1389858_at	Tk1	0	0	0	0	0	0	4.79	4.91	5.05	5.20	5.07	1	0	0	0	0	0	1	9.33	9.34	9.39	9.36	9.18	8.78	8.68
1389881_at	---	0	0	0	0	0	0	4.95	5.14	4.97	5.01	4.94	1	0	0	0	0	0	1	5.70	5.93	5.90	5.74	5.66	5.34	4.65
1389885_at	Limd2	0	0	0	0	0	0	8.39	8.48	8.50	8.29	8.48	1	0	0	0	0	0	1	10.07	10.14	10.14	10.18	10.10	9.64	9.13
1389889_at	Chpf2	0	0	0	0	0	0	4.72	4.61	4.54	4.62	4.62	1	0	0	0	0	0	1	7.61	7.72	7.75	7.83	7.76	7.26	6.06
1389893_at	RGD1560784	0	0	0	0	0	0	4.26	4.04	3.90	4.10	3.93	1	0	0	0	0	1	1	6.45	6.44	6.37	6.20	6.03	5.68	5.16
1389973_a_at	Surf4	0	0	0	0	0	0	10.36	10.51	10.39	10.75	10.62	1	0	0	0	0	0	1	11.45	11.47	11.43	11.44	11.42	11.12	10.63
1389996_at	Nek1	0	0	0	0	0	0	3.82	3.69	4.18	3.50	3.65	1	0	0	0	0	1	1	7.47	7.37	7.52	7.68	7.66	8.10	7.47
1390003_at	Zfp57	0	0	0	0	0	0	5.77	6.00	6.21	5.82	6.24	1	0	0	0	0	0	1	8.17	8.19	8.32	8.27	8.22	7.68	7.39
1390034_at	---	0	0	0	0	0	0	3.59	3.74	3.98	3.46	4.05	1	0	0	0	0	1	1	7.02	7.02	7.26	7.38	7.41	7.94	8.30
1390042_at	Tmem140	0	0	0	0	0	0	6.13	6.59	6.03	6.37	6.14	1	0	0	0	0	1	1	4.53	4.43	4.47	4.65	4.68	5.25	5.28
1390049_at	Fhl1	0	0	0	0	0	0	4.27	4.03	4.02	4.14	3.81	1	0	0	0	0	1	1	5.38	5.46	5.64	5.74	5.57	6.19	5.61
1390051_at	Mtus2	0	0	0	0	0	0	3.70	3.74	3.71	4.01	3.56	1	0	0	0	0	1	1	6.03	6.12	6.23	6.28	6.34	6.65	6.68
1390053_at	Mplkip	0	0	0	0	0	0	4.70	4.60	4.97	4.32	4.33	1	0	0	0	0	0	1	6.53	6.53	6.64	6.57	6.40	6.06	5.38
1390098_at	Eef1akmt1	0	0	0	0	0	0	6.03	5.99	6.13	6.30	5.86	1	0	0	0	0	0	1	8.11	8.03	8.14	8.09	7.98	7.59	7.09
1390103_at	Phf2	0	0	0	0	0	0	6.42	6.40	6.63	6.63	6.76	1	0	0	0	0	0	1	8.21	8.19	8.28	8.29	8.28	7.88	7.74
1390107_at	Sytl2	0	0	0	0	0	0	5.43	5.35	5.61	5.38	5.35	1	0	0	0	0	0	1	4.02	3.97	3.97	3.99	4.01	4.44	4.93
1390110_at	Uckl1	0	0	0	0	0	0	7.15	7.05	7.45	7.17	7.29	1	0	0	0	0	1	1	9.52	9.50	9.52	9.42	9.37	8.80	8.30
1390124_at	RGD1560464	0	0	0	0	0	0	6.17	6.12	6.48	6.20	6.39	1	0	0	0	0	0	1	9.93	9.94	9.95	9.94	9.97	9.59	8.84
1390137_at	Knstrn	0	0	0	0	0	0	5.03	4.91	5.37	5.14	5.15	1	0	0	0	0	1	1	9.67	9.62	9.61	9.51	9.32	7.99	8.26
1390141_at	Mthfd1l	0	0	0	0	0	0	3.78	3.48	3.94	4.03	3.63	1	0	0	0	0	1	1	7.49	7.31	7.24	7.15	7.03	6.11	6.08
1390167_at	Ttc9b	0	0	0	0	0	0	3.90	3.87	3.53	3.73	3.68	1	0	0	0	0	0	1	6.12	6.16	6.35	6.35	6.20	6.66	6.41
1390170_at	LOC103690803	0	0	0	0	0	0	3.93	4.08	3.71	3.84	3.94	1	0	0	0	0	1	0	7.47	7.49	7.38	7.13	7.21	6.80	6.74
1390190_at	Plac1	0	0	0	0	0	0	3.04	3.14	3.09	3.08	3.02	1	0	0	1	1	1	1	6.56	6.72	6.95	7.34	7.45	7.93	7.79
1390198_at	Kmt5b	0	0	0	0	0	0	6.86	6.77	7.26	6.98	6.95	1	0	0	0	0	1	1	7.34	7.48	7.46	7.43	7.36	6.70	6.93
1390209_at	Syngr3	0	0	0	0	0	0	4.33	4.45	4.27	4.46	4.38	1	0	0	0	0	1	1	6.78	6.90	6.99	7.09	7.26	7.64	6.82
1390215_at	---	0	0	0	0	0	0	5.91	5.93	6.22	5.79	6.03	1	0	0	0	0	1	1	4.67	4.63	4.76	4.75	4.92	5.88	6.63
1390218_at	Meaf6	0	0	0	0	0	0	7.47	7.53	7.80	7.50	7.66	1	0	0	0	0	0	1	10.66	10.68	10.67	10.65	10.56	10.19	9.80
1390227_at	Pcp4l1	0	0	0	0	0	0	3.89	3.86	3.84	3.93	3.83	1	0	0	0	0	1	1	6.20	6.28	6.43	6.44	6.44	7.39	7.01
1390243_at	---	0	0	0	0	0	0	4.09	4.25	4.23	4.14	4.34	1	0	0	1	1	1	1	6.17	6.32	6.61	6.80	6.84	7.24	7.22
1390246_at	Stxbp4	0	0	0	0	0	0	4.65	4.76	5.16	4.83	4.84	1	0	0	1	1	1	1	4.65	4.90	4.96	5.26	5.34	6.06	6.54
1390258_at	---	0	0	0	0	0	0	4.69	4.83	4.66	4.87	4.85	1	0	0	0	0	0	1	5.78	5.79	6.11	5.93	5.88	5.33	4.87
1390272_at	Dph5	0	0	0	0	0	0	5.21	4.99	5.37	5.19	4.96	1	0	0	0	0	0	1	8.13	8.07	8.14	8.23	8.10	7.66	6.86
1390273_at	---	0	0	0	0	0	0	5.31	5.26	5.16	5.36	5.35	1	0	0	0	0	1	1	6.48	6.41	6.50	6.42	6.29	5.78	5.70
1390298_at	Snx18	0	0	0	0	0	0	8.43	8.46	8.90	8.32	8.45	1	0	0	0	0	1	1	9.61	9.63	9.78	9.87	9.84	10.31	10.28
1390300_at	---	0	0	0	0	0	0	6.85	6.89	7.24	6.93	6.47	1	0	0	0	1	1	1	8.62	8.87	9.04	9.04	9.22	9.65	9.65
1390339_at	Usp13	0	0	0	0	0	0	6.11	6.25	6.41	5.94	6.43	1	0	0	0	0	0	1	6.81	6.83	6.89	6.80	6.77	6.38	5.85
1390352_at	Pcgf1	0	0	0	0	0	0	6.29	6.33	6.48	6.33	6.47	1	0	0	0	0	1	0	8.48	8.35	8.47	8.33	8.25	7.82	7.58
1390365_at	Leng1	0	0	0	0	0	0	3.98	4.21	4.07	4.13	4.03	1	0	0	0	0	1	1	5.78	5.80	5.89	5.73	5.59	5.04	5.02
1390383_at	Plin2	0	0	0	0	0	0	7.33	7.38	7.54	7.26	7.46	1	0	0	0	0	1	1	7.83	7.79	7.93	8.09	8.27	9.00	9.00
1390384_at	H2ax	0	0	0	0	0	0	6.48	6.57	6.67	6.90	6.45	1	0	0	0	0	0	1	6.64	6.60	6.81	6.78	6.59	6.09	6.67
1390387_at	Sh3d19	0	0	0	0	0	0	7.48	7.36	7.58	7.74	7.41	1	0	0	0	0	1	1	7.03	7.13	7.24	7.38	7.44	7.76	7.68
1390415_at	Trip13	0	0	0	0	0	0	4.15	4.12	4.04	4.21	4.25	1	0	0	0	0	1	1	8.30	8.28	8.35	8.34	8.23	7.33	7.08
1390426_at	Notch1	0	0	0	0	0	0	6.28	6.10	6.25	6.15	6.12	1	0	0	0	0	0	1	4.40	4.09	4.09	4.04	4.11	4.55	5.36
1390437_at	Sema5a	0	0	0	0	0	0	5.70	5.82	5.94	5.42	5.60	1	0	0	0	0	1	1	3.98	4.00	4.04	3.96	4.04	5.22	5.51
1390441_at	Asap1	0	0	0	0	0	0	6.19	6.08	6.37	5.88	6.44	1	0	0	0	0	1	1	9.85	9.92	9.91	10.02	10.14	10.51	10.47
1390443_at	C21orf91	0	0	0	0	0	0	6.71	6.39	6.95	6.44	6.34	1	0	0	0	0	1	0	7.31	7.28	7.55	7.66	7.64	8.19	7.95
1390447_at	Stx3	0	0	0	0	0	0	5.97	5.98	6.14	5.98	6.25	1	0	0	0	0	1	1	8.02	8.03	8.00	7.83	7.77	7.16	6.71
1390449_at	---	0	0	0	0	0	0	4.39	4.39	4.40	4.11	4.46	1	0	0	0	0	1	1	6.18	6.25	6.33	6.53	6.47	7.09	7.22
1390451_at	Wdr41	0	0	0	0	0	0	5.85	5.71	5.97	5.81	5.79	1	0	0	0	0	1	1	7.39	7.32	7.30	7.29	7.07	6.54	6.27
1390468_at	Neo1	0	0	0	0	0	0	7.78	7.69	8.27	7.65	7.76	1	0	0	0	0	1	1	6.98	7.08	7.14	7.07	7.11	7.65	8.41
1390472_at	Naaa	0	0	0	0	0	0	5.12	5.09	5.14	4.94	5.17	1	0	0	0	1	1	1	8.47	8.74	8.78	8.85	9.13	9.77	9.20
1390474_at	Nipal2	0	0	0	0	0	0	7.82	7.68	7.68	7.95	7.77	1	0	0	0	0	1	1	8.63	8.71	8.75	8.69	8.59	7.82	7.49
1390481_a_at	Ube2t	0	0	0	0	0	0	5.00	5.04	5.20	5.17	5.42	1	0	0	0	0	1	1	9.19	9.13	9.14	9.09	8.99	8.22	8.06
1390498_at	Ca5b	0	0	0	0	0	0	5.24	5.12	5.36	4.98	5.26	1	0	0	0	0	1	1	3.45	3.48	3.51	3.67	3.85	4.63	5.48
1390505_a_at	Lrfn4	0	0	0	0	0	0	5.55	5.68	5.38	5.49	5.56	1	0	0	0	0	1	1	7.76	7.65	7.67	7.61	7.60	7.14	6.84
1390521_at	---	0	0	0	0	0	0	3.82	3.83	3.84	3.94	3.73	1	0	0	0	0	1	0	6.38	6.27	6.48	6.48	6.24	5.73	5.86
1390527_at	Fam174c	0	0	0	0	0	0	7.16	7.41	7.10	7.38	7.28	1	0	0	0	0	0	1	9.95	9.88	9.86	9.85	9.70	9.42	9.02
1390529_at	Cd83	0	0	0	0	0	0	6.13	6.20	6.17	5.80	6.55	1	0	0	0	0	1	1	8.17	8.13	8.17	8.33	8.29	8.93	8.94
1390539_at	---	0	0	0	0	0	0	2.71	2.76	2.70	2.74	2.71	1	0	0	0	0	0	1	5.70	5.69	6.06	6.27	6.17	6.23	6.26
1390541_at	Ush1c	0	0	0	0	0	0	5.24	5.21	5.17	5.13	5.20	1	0	0	0	0	1	0	6.56	6.73	6.81	6.82	6.94	7.46	7.41
1390545_at	Dhx40	0	0	0	0	0	0	8.12	8.03	8.30	8.24	8.29	1	0	0	0	0	1	1	9.00	9.11	9.15	9.28	9.32	9.73	9.69
1390557_at	Gca	0	0	0	0	0	0	6.64	6.70	7.03	6.68	7.05	1	0	0	0	0	1	1	5.69	5.76	5.84	5.89	6.11	6.39	6.94
1390559_at	Slc38a1	0	0	0	0	0	0	3.83	3.76	3.64	4.02	3.87	1	0	0	0	0	0	1	5.79	5.77	5.93	5.94	5.75	5.51	4.44
1390569_at	Cndp1	0	0	0	0	0	0	3.43	3.58	3.37	3.39	3.41	1	0	0	0	0	1	1	3.87	3.97	4.08	4.08	4.14	4.96	5.62
1390579_at	Tma16	0	0	0	0	0	0	4.97	4.92	4.94	5.09	5.06	1	0	0	0	0	1	1	7.48	7.56	7.62	7.69	7.53	6.74	6.25
1390583_at	---	0	0	0	0	0	0	3.25	3.16	3.32	3.35	3.15	1	0	0	0	0	1	1	5.65	5.64	5.65	5.88	5.59	4.94	4.40
1390586_at	Kdm5c	0	0	0	0	0	0	7.15	7.43	7.60	7.26	7.55	1	0	0	0	0	1	1	8.74	8.62	8.70	8.64	8.55	8.07	7.80
1390590_at	Pofut2	0	0	0	0	0	0	6.69	6.55	6.76	6.63	6.40	1	0	0	0	0	0	1	9.31	9.39	9.28	9.19	9.11	8.75	8.12
1390626_at	Onecut2	0	0	0	0	0	0	3.08	3.13	3.08	3.12	3.28	1	0	0	0	0	1	1	7.46	7.54	7.78	7.89	7.91	8.59	9.08
1390628_at	Cpeb2	0	0	0	0	0	0	8.25	7.81	8.22	7.97	8.00	1	0	0	0	0	1	1	8.64	8.76	8.84	8.96	9.05	9.55	9.91
1390638_at	Epha4	0	0	0	0	0	0	3.65	3.56	3.86	3.51	3.57	1	0	0	0	0	1	1	4.78	4.60	4.94	4.80	4.83	6.12	6.33
1390672_at	Rprm	0	0	0	0	0	0	4.70	4.71	4.56	4.68	4.46	1	0	0	0	0	0	1	5.10	5.11	5.28	4.81	5.01	5.22	6.42
1390675_at	---	0	0	0	0	0	0	4.25	4.29	4.07	4.25	4.29	1	0	0	0	0	1	1	5.20	5.35	5.36	5.27	5.41	5.93	5.78
1390707_at	Rgs10	0	0	0	0	0	0	4.39	4.23	4.06	4.05	4.20	1	0	0	0	0	1	1	8.54	8.63	8.68	8.75	8.88	9.45	9.69
1390731_at	Rasgef1b	0	0	0	0	0	0	5.15	5.59	5.38	5.43	5.15	1	0	0	0	0	1	1	4.96	5.09	5.05	4.92	4.99	5.56	6.25
1390756_at	Fam155a	0	0	0	0	0	0	4.13	4.14	4.47	3.81	4.10	1	0	0	0	0	1	1	8.88	8.77	8.88	8.86	8.69	8.11	7.08
1390763_at	Efna3	0	0	0	0	0	0	4.47	4.45	4.31	4.64	4.55	1	0	0	0	0	1	0	8.32	8.44	8.49	8.42	8.49	9.04	9.26
1390781_at	Abcb10	0	0	0	0	0	0	6.44	6.57	6.39	6.77	6.26	1	0	0	0	0	0	1	6.65	6.55	6.74	6.72	6.68	6.84	7.52
1390800_a_at	Cav2	0	0	0	0	0	0	5.15	5.13	5.05	4.95	4.78	1	0	0	0	0	1	1	3.39	3.37	3.54	3.47	3.68	4.23	3.54
1390801_at	Tmem252	0	0	0	0	0	0	5.81	5.90	5.84	5.36	5.98	1	0	0	0	0	1	1	10.66	10.73	10.61	10.48	10.60	9.99	9.06
1390807_at	Ca5b	0	0	0	0	0	0	7.80	7.80	7.63	7.70	7.56	1	0	0	1	1	1	1	5.43	5.69	5.71	6.10	6.40	8.05	8.93
1390818_at	Tfdp2	0	0	0	0	0	0	6.18	6.03	6.55	6.16	6.20	1	0	0	0	0	0	1	6.19	6.06	6.23	6.24	6.23	6.64	6.98
1390830_at	Abhd18	0	0	0	0	0	0	3.88	3.88	3.74	3.77	3.84	1	0	0	0	0	1	1	5.75	5.73	5.52	5.17	5.18	4.72	4.36
1390837_at	---	0	0	0	0	0	0	6.25	6.30	6.49	6.14	5.94	1	0	0	0	0	1	0	6.41	6.47	6.60	6.76	6.65	7.11	6.96
1390847_at	Tmem86a	0	0	0	0	0	0	6.44	6.51	6.68	6.30	6.48	1	0	0	0	0	1	0	7.64	7.69	7.74	7.81	7.94	8.40	7.70
1390851_at	---	0	0	0	0	0	0	7.19	6.93	7.34	7.25	7.31	1	0	0	0	0	1	0	6.82	6.93	7.09	7.28	7.36	7.50	7.54
1390853_at	---	0	0	0	0	0	0	6.36	6.43	6.26	6.50	6.41	1	0	0	0	0	1	0	6.33	6.32	6.38	6.43	6.39	6.99	6.64
1390857_at	Xylb	0	0	0	0	0	0	6.63	6.56	6.49	6.90	6.21	1	0	0	0	0	0	1	4.93	4.86	4.99	5.02	5.10	5.11	5.75
1390869_at	Cbln1	0	0	0	0	0	0	3.23	3.36	3.39	3.40	3.31	1	0	0	0	0	1	1	6.70	6.69	6.86	6.93	7.10	8.10	7.72
1390878_at	Thbs3	0	0	0	0	0	0	4.87	4.85	4.63	4.83	4.66	1	0	0	0	1	1	1	7.45	7.59	7.77	7.90	8.13	8.65	7.97
1390891_at	Kif11	0	0	0	0	0	0	4.75	4.46	4.93	5.00	4.80	1	0	0	0	0	0	1	9.73	9.70	9.77	9.80	9.74	9.19	9.13
1390892_at	Depdc1b	0	0	0	0	0	0	4.54	4.68	4.58	4.76	4.69	1	0	0	0	0	1	1	7.21	7.15	7.17	7.09	7.00	6.33	6.27
1390903_at	LOC102550354	0	0	0	0	0	0	3.41	3.52	3.34	3.45	3.39	1	0	0	0	0	0	1	4.33	4.09	4.20	4.05	4.11	4.71	4.77
1390912_at	Pcp4l1	0	0	0	0	0	0	3.76	3.81	3.52	3.64	3.52	1	0	0	0	0	1	1	5.57	5.76	5.81	5.75	5.77	6.39	6.43
1390917_at	---	0	0	0	0	0	0	3.43	3.48	3.44	3.52	3.39	1	0	0	0	0	1	0	3.89	4.01	4.12	4.12	4.22	4.54	4.13
1390925_a_at	Igsf3	0	0	0	0	0	0	4.63	4.79	4.68	4.91	4.65	1	0	0	0	0	0	1	6.96	6.94	6.99	6.91	6.99	7.43	7.65
1390937_at	Ppp1r36	0	0	0	0	0	0	6.22	6.29	6.38	6.08	6.05	1	0	0	0	0	1	1	8.83	8.94	9.12	9.29	9.35	9.73	9.70
1390957_at	Tmem260	0	0	0	0	0	0	6.59	6.77	6.76	6.91	6.71	1	0	0	0	0	0	1	6.44	6.56	6.65	6.64	6.74	6.97	7.01
1390959_at	Ttc32	0	0	0	0	0	0	4.54	4.32	4.62	4.68	4.56	1	0	0	0	0	1	1	5.10	5.00	5.34	5.62	5.12	4.42	4.39
1390966_at	---	0	0	0	0	0	0	6.21	6.13	6.53	6.18	6.22	1	0	0	0	0	1	1	7.39	7.46	7.57	7.76	7.85	8.37	8.67
1390976_at	Lynx1	0	0	0	0	0	0	6.23	6.25	6.04	6.18	6.14	1	0	0	0	0	1	1	7.05	7.08	7.12	7.14	7.27	7.89	8.06
1390987_at	---	0	0	0	0	0	0	5.13	5.23	5.31	5.04	5.02	1	0	0	0	0	1	1	6.60	6.69	6.73	6.74	6.89	7.24	7.70
1390990_at	Ppp1cb	0	0	0	0	0	0	7.17	6.86	7.34	6.81	6.99	1	0	0	0	0	1	1	7.40	7.40	7.64	7.79	7.84	8.30	8.20
1390999_at	---	0	0	0	0	0	0	3.67	3.80	3.64	3.75	3.68	1	0	0	0	1	0	0	5.02	4.80	5.40	5.47	5.69	5.16	4.99
1391013_at	Pcdh8	0	0	0	0	0	0	3.55	3.60	3.76	3.42	3.38	1	0	0	1	1	1	1	6.10	6.25	6.57	6.83	6.90	7.51	7.71
1391016_at	Wars2	0	0	0	0	0	0	5.00	4.97	4.92	4.95	5.17	1	0	0	0	0	0	1	6.64	6.66	6.68	6.57	6.47	6.13	5.56
1391022_at	Lamb3	0	0	0	0	0	0	3.79	3.57	3.42	3.73	3.75	1	0	0	0	0	1	1	7.69	7.68	7.75	7.69	7.98	8.35	8.54
1391027_at	Trim65	0	0	0	0	0	0	6.55	6.51	6.72	6.54	6.60	1	0	0	0	0	1	1	8.82	8.88	8.82	8.80	8.72	8.10	7.40

Probeset ID	Gene Symbol	Islet Data Set (log2 ratio ≥0.263; FDR <20%) union n=5325 1=YES 0=NO	Islet DRlyp/lyp ND vs ND+Lp299v (log2 ratio ≥0.263; FDR<20%) n=429 1=YES 0=NO	Islet DRlyp/lyp ND vs HCD (log2 ratio ≥0.263; FDR<20%) n=2247 1=YES 0=NO	Islet DRlyp/lyp ND vs HCD+Lp299v (log2 ratio ≥0.263; FDR<20%) n=1595 1=YES 0=NO	Islet DRlyp/lyp HCD vs HCD+Lp299v (log2 ratio ≥0.263; FDR<20%) n=3026 1=YES 0=NO	Islet DRlyp/lyp ND vs Flyp/lyp ND (log2 ratio ≥ 0.263; FDR<20%) n=1950 1=YES 0=NO	Islet Mean Log2 Intensity DRlyp/lyp ND	Islet Mean Log2 Intensity DRlyp/lyp ND+Lp299v	Islet Mean Log2 Intensity DRlyp/lyp HCD	Islet Mean Log2 Intensity DRlyp/lyp HCD+Lp299v	Islet Mean Log2 Intensity Flyp40 ND	Rinm5f data union of all conditions n=3705 1=YES 0=NO	Rinm5f 30 µM propionate/ 6 µM butyrate vs Media (FDR<20%; 1.5 fold) n=3 1=YES 0=NO	Rinm5f 60 µM propionate/ 12 µM butyrate vs Media (FDR<20%; 1.5 fold) n=18 1=YES 0=NO	Rinm5f 120 µM propionate/ 24 µM butyrate vs Media (FDR<20%; 1.5 fold) n=164 1=YES 0=NO	Rinm5f 240 µM propionate /48 µM butyrate vs Media (FDR<20%; 1.5 fold) n=268 1=YES 0=NO	Rinm5f 1500 µM propionate/ 300 µM butyrate vs Media (FDR<20%; 1.5 fold) n=2491 1=YES 0=NO	Rinm5f 500 µM propionate/ 500 µM butyrate vs Media (FDR<20%; 1.5 fold) n=3001 1=YES 0=NO	Rinm5f Mean Log2 Intensity Media only	Rinm5f Mean Log2 Intensity 30 µM propionate/ 6 µM butyrate	Rinm5f Mean Log2 Intensity 60 µM propionate/ 12 µM butyrate	Rinm5f Mean Log2 Intensity 120 µM propionate/ 24 µM butyrate	Rinm5f Mean Log2 Intensity 240 µM propionate/ 48 µM butyrate	Rinm5f Mean Log2 Intensity 1500 µM propionate /300 µM butyrate	Rinm5f Mean Log2 Intensity 500 µM propionate/ 500 µM butyrate
1391037_at	Vwa5b2	0	0	0	0	0	0	7.65	7.87	7.86	7.52	7.85	1	0	0	0	0	1	1	9.28	9.20	9.37	9.39	9.25	8.48	8.12
1391041_at	Vwa3b	0	0	0	0	0	0	4.56	4.47	4.29	4.65	4.39	1	0	0	0	0	0	1	3.81	3.90	3.98	3.82	3.86	4.25	5.01
1391045_at	Dgat2	0	0	0	0	0	0	4.45	4.40	4.02	4.55	4.16	1	0	0	1	1	1	1	8.25	8.38	8.63	8.92	9.00	9.57	9.05
1391059_at	Npw	0	0	0	0	0	0	3.87	3.94	3.88	3.92	4.08	1	0	0	0	0	1	1	4.39	4.50	4.51	4.45	4.64	5.77	6.56
1391063_at	Kif23	0	0	0	0	0	0	4.62	4.61	5.02	4.84	4.91	1	0	0	0	0	1	1	8.79	8.75	8.78	8.74	8.59	7.70	7.70
1391070_at	Heatr4	0	0	0	0	0	0	5.74	5.85	5.54	5.83	5.82	1	0	0	0	0	1	0	6.85	6.99	7.04	7.14	7.32	7.73	7.60
1391071_at	---	0	0	0	0	0	0	4.59	4.57	4.52	4.50	4.62	1	0	0	1	0	1	1	9.06	8.96	8.80	8.46	8.51	7.50	7.60
1391074_at	Crabp1	0	0	0	0	0	0	4.58	4.55	4.44	4.51	4.66	1	0	0	0	0	1	1	8.03	8.17	8.20	8.30	8.35	9.16	9.33
1391077_at	Clspn	0	0	0	0	0	0	4.45	4.63	4.40	4.60	4.66	1	0	0	0	0	0	1	7.18	7.03	7.18	7.25	7.04	6.61	6.54
1391078_at	Rfc1	0	0	0	0	0	0	6.11	6.16	6.34	6.31	6.25	1	0	0	0	0	0	1	8.75	8.58	8.72	8.75	8.80	9.25	9.31
1391081_at	---	0	0	0	0	0	0	2.91	2.97	2.88	2.94	2.82	1	0	0	0	0	0	1	8.43	8.67	8.81	8.75	8.82	8.64	7.72
1391084_at	---	0	0	0	0	0	0	2.87	2.98	3.02	3.01	2.94	1	0	0	0	1	1	1	4.43	4.44	4.87	4.97	5.04	5.15	4.25
1391092_at	Glrb	0	0	0	0	0	0	2.83	2.83	2.95	2.85	2.83	1	0	0	1	1	1	0	6.60	6.80	7.04	7.35	7.28	7.35	6.85
1391114_at	---	0	0	0	0	0	0	5.46	5.35	5.04	5.33	5.26	1	0	0	0	0	1	0	8.01	7.95	8.12	8.04	7.79	7.40	7.32
1391134_at	LOC103693999 /	0	0	0	0	0	0	4.97	5.22	5.23	4.97	4.80	1	0	0	0	0	0	1	7.51	7.53	7.65	7.59	7.37	7.13	6.66
1391150_at	---	0	0	0	0	0	0	2.87	2.84	2.83	2.98	2.89	1	0	0	0	0	0	1	3.43	3.32	3.55	3.29	3.39	3.87	4.32
1391153_at	---	0	0	0	0	0	0	3.63	3.65	3.45	3.50	3.56	1	0	0	0	0	1	0	7.31	7.20	7.10	7.11	7.07	6.70	5.71
1391155_at	Fip1l1	0	0	0	0	0	0	4.11	4.11	4.58	4.02	4.20	1	0	0	0	1	1	0	5.76	5.86	6.08	6.27	6.36	6.45	5.46
1391169_at	Tmem117	0	0	0	0	0	0	4.94	4.87	5.17	4.89	4.83	1	0	0	0	0	1	1	8.50	8.54	8.50	8.41	8.30	7.32	7.05
1391172_at	LOC103690947	0	0	0	0	0	0	4.65	4.73	4.73	4.64	4.73	1	0	0	0	0	1	0	8.41	8.35	8.34	8.26	8.14	7.61	7.81
1391184_at	Rfx2	0	0	0	0	0	0	5.24	5.48	5.30	5.34	5.52	1	0	0	0	0	0	1	6.53	6.62	6.70	6.80	6.81	7.05	7.23
1391187_at	Ppl	0	0	0	0	0	0	6.26	6.34	6.66	6.22	6.28	1	0	0	0	0	0	1	7.64	7.62	7.76	7.79	7.83	7.97	8.30
1391208_at	Pcdh20	0	0	0	0	0	0	2.90	2.74	2.74	2.78	2.72	1	0	0	0	0	1	1	4.13	4.12	4.42	4.38	4.50	5.36	5.39
1391228_at	Rsph1	0	0	0	0	0	0	3.78	3.60	3.86	3.76	3.91	1	0	0	0	0	1	1	5.92	5.96	6.06	6.01	5.98	6.62	7.20
1391229_at	Camk1g	0	0	0	0	0	0	7.38	7.48	7.77	7.28	7.54	1	0	0	0	0	1	1	6.99	7.13	7.19	7.21	7.40	7.83	7.74
1391240_at	Hs3st6	0	0	0	0	0	0	4.75	4.71	4.87	4.47	4.65	1	0	0	0	0	1	1	6.55	6.65	6.79	6.90	6.92	7.25	7.19
1391246_at	---	0	0	0	0	0	0	3.30	3.23	3.05	3.13	3.17	1	0	0	0	0	1	0	6.68	6.70	6.79	6.71	6.60	6.07	5.69
1391277_at	S100a5	0	0	0	0	0	0	4.23	4.25	4.00	4.25	4.33	1	0	0	1	1	1	1	7.05	7.33	7.59	8.11	8.43	9.42	9.07
1391285_at	Zfyve9	0	0	0	0	0	0	4.49	4.59	4.44	4.67	4.39	1	0	0	0	0	1	0	5.67	5.58	5.85	5.49	5.60	5.04	4.95
1391292_at	---	0	0	0	0	0	0	7.75	7.98	7.73	8.21	8.08	1	0	0	0	0	0	1	7.31	7.30	7.36	7.45	7.29	6.78	6.46
1391317_at	Ska1	0	0	0	0	0	0	3.23	3.25	3.37	3.34	3.32	1	0	0	0	0	1	1	8.99	8.93	8.98	8.99	8.78	7.55	7.63
1391341_at	Plxdc1	0	0	0	0	0	0	6.35	6.46	6.64	6.27	6.27	1	0	0	0	1	1	1	6.57	6.74	6.73	6.88	7.35	8.94	8.76
1391342_at	Cdk5	0	0	0	0	0	0	4.93	5.09	5.20	5.09	5.20	1	0	0	0	0	1	0	7.58	7.69	7.87	8.07	8.07	8.32	8.15
1391345_at	Bmper	0	0	0	0	0	0	2.75	2.77	2.76	2.65	2.71	1	0	0	0	0	1	0	4.25	4.26	4.44	4.57	4.38	5.07	4.35
1391374_at	Tacc3	0	0	0	0	0	0	4.94	5.22	4.98	5.08	5.19	1	0	0	0	0	1	1	6.85	6.86	6.92	6.84	6.75	6.17	5.65
1391383_at	Crtac1	0	0	0	0	0	0	3.78	3.63	3.65	3.66	3.64	1	0	0	0	0	1	1	7.02	7.10	7.23	7.17	7.10	7.68	8.04
1391451_at	---	0	0	0	0	0	0	5.26	4.97	4.95	5.42	5.08	1	0	0	0	0	0	1	5.83	5.79	6.02	6.07	6.00	5.47	4.76
1391460_at	---	0	0	0	0	0	0	7.88	7.87	8.23	7.93	8.02	1	0	0	0	0	0	1	9.93	9.94	9.99	9.98	9.97	9.41	9.10
1391478_at	Znf532	0	0	0	0	0	0	6.44	6.33	6.47	6.46	6.64	1	0	0	0	0	1	1	7.87	7.80	7.92	7.95	7.90	7.19	6.97
1391494_at	Vamp4	0	0	0	0	0	0	7.37	7.52	7.53	7.64	7.76	1	0	0	0	0	1	1	8.44	8.50	8.40	8.34	8.23	7.80	7.37
1391495_at	Borcs7	0	0	0	0	0	0	7.83	7.72	8.19	7.89	7.89	1	0	0	0	0	1	1	9.55	9.40	9.40	9.33	9.05	8.59	8.57
1391499_at	---	0	0	0	0	0	0	3.77	3.84	3.92	3.64	3.81	1	0	0	1	1	1	1	6.64	6.93	7.09	7.30	7.58	8.26	8.06
1391507_at	Znf467	0	0	0	0	0	0	5.72	5.76	5.84	5.75	5.68	1	0	0	0	0	1	1	7.88	7.93	8.01	8.03	8.00	7.00	6.40
1391518_at	Cenpw	0	0	0	0	0	0	4.20	4.18	4.32	4.22	4.32	1	0	0	0	0	1	1	8.81	8.79	8.79	8.82	8.66	7.52	7.65
1391521_at	LOC100910774	0	0	0	0	0	0	3.23	3.42	3.16	3.30	3.33	1	0	0	0	0	1	1	4.59	4.50	4.63	4.61	4.48	6.04	7.16
1391527_at	Stat6	0	0	0	0	0	0	5.22	5.39	5.35	5.29	5.30	1	0	0	0	0	0	1	7.16	7.22	7.23	7.19	7.01	6.60	5.64
1391547_at	---	0	0	0	0	0	0	3.22	3.33	3.07	3.21	3.17	1	0	0	1	1	1	1	5.44	5.13	5.60	6.06	6.17	6.18	6.23
1391556_at	Sema5a	0	0	0	0	0	0	4.20	4.19	4.16	4.16	4.12	1	0	0	0	0	0	1	3.99	3.97	3.96	3.95	3.93	4.49	4.20
1391558_at	Znf608	0	0	0	0	0	0	5.09	5.26	5.08	5.19	5.15	1	0	0	0	0	0	1	6.72	6.81	6.96	6.93	6.91	7.18	7.37
1391563_at	Pwwp3b	0	0	0	0	0	0	5.44	5.38	5.95	5.37	5.57	1	0	0	0	0	1	1	4.05	4.12	4.27	4.36	4.56	6.19	7.11
1391574_at	Exoc6	0	0	0	0	0	0	4.46	4.24	4.63	4.61	4.46	1	0	0	0	1	1	0	8.82	8.92	9.07	9.37	9.40	9.65	9.46
1391593_at	Rassf4	0	0	0	0	0	0	3.37	3.47	3.31	3.35	3.43	1	0	0	0	0	1	1	6.75	6.77	7.00	7.17	7.31	8.17	7.76
1391597_at	Gpr75	0	0	0	0	0	0	4.93	4.79	4.66	4.82	4.72	1	0	0	0	0	0	1	6.36	6.31	6.50	6.44	6.32	5.88	5.40
1391601_at	Lrrn2	0	0	0	0	0	0	8.23	8.23	8.08	8.23	8.18	1	0	0	0	0	1	1	9.73	9.67	9.67	9.54	9.47	8.95	8.56
1391611_at	Kif12	0	0	0	0	0	0	7.85	8.05	8.16	7.75	8.11	1	0	0	0	0	1	1	8.57	8.58	8.55	8.52	8.29	7.44	7.02
1391636_at	---	0	0	0	0	0	0	3.82	3.71	4.20	3.86	4.05	1	0	0	1	1	1	0	5.77	5.89	6.23	6.51	6.54	6.76	6.00
1391637_at	Arhgap15	0	0	0	0	0	0	3.82	3.74	3.76	3.73	4.07	1	0	0	0	0	1	1	5.73	5.76	5.74	5.47	5.27	4.80	3.53
1391649_a_at	Prmt2	0	0	0	0	0	0	5.29	5.30	5.31	5.24	5.33	1	0	0	0	0	1	1	8.66	8.62	8.62	8.60	8.49	7.87	8.01
1391654_at	---	0	0	0	0	0	0	4.65	5.08	5.11	4.94	5.03	1	0	0	0	0	1	1	5.81	5.78	5.99	5.92	5.71	5.01	4.31
1391662_at	C1orf159	0	0	0	0	0	0	4.31	4.30	4.16	4.30	4.35	1	0	0	0	0	1	1	6.06	6.01	5.93	6.03	5.89	5.12	4.66
1391670_at	ELOC	0	0	0	0	0	0	4.61	4.65	4.79	4.61	4.79	1	0	0	0	0	1	0	7.70	7.82	7.94	8.14	8.21	8.36	7.99
1391672_at	Castor2	0	0	0	0	0	0	7.62	7.41	7.55	7.54	7.66	1	0	0	0	0	1	0	8.59	8.59	8.83	8.89	8.91	9.21	9.45
1391680_at	---	0	0	0	0	0	0	3.35	3.31	3.27	3.33	3.49	1	0	0	0	0	1	1	5.89	5.85	6.09	6.14	6.00	6.52	6.38
1391700_at	---	0	0	0	0	0	0	4.90	5.21	5.19	5.11	5.19	1	0	0	0	0	1	1	6.25	6.33	6.59	6.51	6.40	6.84	6.86
1391710_at	---	0	0	0	0	0	0	6.48	6.83	6.57	6.62	6.55	1	0	0	0	0	1	1	7.61	7.56	7.59	7.56	7.48	6.89	5.82
1391730_at	LOC102550606	0	0	0	0	0	0	6.58	6.63	6.30	6.63	6.51	1	0	0	0	0	1	1	7.03	6.89	6.86	6.80	6.51	5.62	5.14
1391731_at	---	0	0	0	0	0	0	3.62	3.75	3.80	3.69	3.91	1	0	0	0	0	1	0	5.13	5.16	5.44	5.38	5.63	5.73	5.05
1391749_a_at	Nxnl2	0	0	0	0	0	0	4.21	3.99	3.87	4.14	3.84	1	0	0	0	0	0	1	4.97	4.97	4.95	4.83	4.86	5.23	5.32
1391780_at	Tent5b	0	0	0	0	0	0	4.98	5.05	4.62	4.95	4.92	1	0	0	0	0	0	1	5.51	5.60	5.53	5.48	5.55	5.95	6.16
1391791_at	Acer2	0	0	0	0	0	0	4.93	4.91	4.86	4.74	4.81	1	0	0	0	0	1	1	4.83	4.71	4.87	4.83	4.79	5.49	5.49
1391826_at	Tp53inp1	0	0	0	0	0	0	3.95	3.90	3.63	3.83	3.85	1	0	0	0	0	0	1	5.18	5.10	5.06	4.94	4.91	5.62	5.74
1391863_at	---	0	0	0	0	0	0	7.67	7.70	7.47	8.01	7.54	1	0	0	0	0	0	1	7.60	7.73	7.84	7.56	7.55	7.07	7.04
1391870_at	---	0	0	0	0	0	0	4.10	4.17	3.92	3.95	4.08	1	0	0	0	0	0	1	4.85	4.83	4.86	4.82	4.63	5.12	6.76
1391871_at	Stard13	0	0	0	0	0	0	7.07	6.97	7.28	7.29	7.18	1	0	0	0	0	1	1	4.73	4.58	4.80	4.98	5.06	5.52	6.33
1391889_at	Ddx52	0	0	0	0	0	0	4.61	4.57	4.76	4.67	4.71	1	0	0	0	0	0	1	7.57	7.53	7.65	7.68	7.64	7.06	6.76
1391898_at	---	0	0	0	0	0	0	3.80	3.80	3.73	3.83	3.99	1	0	0	1	1	1	0	5.34	5.47	5.76	6.20	6.15	6.52	6.37
1391905_at	Pwp2	0	0	0	0	0	0	6.24	6.04	6.22	6.24	6.14	1	0	0	0	0	1	1	8.18	8.13	8.19	8.10	7.98	7.60	6.96
1391916_at	Arhgap26	0	0	0	0	0	0	7.79	7.37	7.42	7.62	7.44	1	0	0	0	1	1	1	6.85	6.94	7.08	7.31	7.45	8.34	8.23
1391919_at	Tcerg1l	0	0	0	0	0	0	4.86	4.92	4.66	4.79	4.78	1	0	0	0	0	0	1	6.60	6.58	6.76	6.60	6.61	6.90	7.29
1391932_at	Mab21l3	0	0	0	0	0	0	5.76	5.80	5.65	5.72	5.63	1	0	0	0	0	1	1	7.17	7.22	7.19	6.98	7.37	7.77	7.63
1391934_at	---	0	0	0	0	0	0	3.54	3.57	3.48	3.53	3.46	1	0	0	0	0	1	0	6.50	6.43	6.39	6.37	6.34	5.86	5.98
1391941_at	LOC100912988 /	0	0	0	0	0	0	4.65	4.46	4.59	4.56	4.64	1	0	0	0	0	0	1	6.03	6.07	6.14	5.95	5.97	5.64	5.21
1391970_at	Ca11	0	0	0	0	0	0	7.51	7.57	7.36	7.62	7.39	1	0	0	0	0	1	1	9.45	9.44	9.48	9.40	9.39	8.84	8.36
1391975_at	---	0	0	0	0	0	0	4.50	4.59	4.72	4.28	4.60	1	0	0	1	1	0	0	6.24	6.45	6.77	6.84	6.84	6.57	5.87
1392026_at	---	0	0	0	0	0	0	7.34	7.73	7.35	7.14	7.16	1	0	0	0	0	1	0	7.82	7.74	8.10	7.76	7.94	8.42	6.69
1392061_at	Mcm10	0	0	0	0	0	0	3.67	3.83	3.56	3.85	3.83	1	0	0	0	0	1	1	6.96	6.73	6.85	6.91	6.70	6.09	5.78
1392065_at	Kif18a	0	0	0	0	0	0	3.29	3.42	3.40	3.44	3.51	1	0	0	0	0	1	1	7.39	7.53	7.45	7.45	7.40	6.41	5.72
1392072_at	---	0	0	0	0	0	0	4.13	4.10	4.18	4.15	4.13	1	0	0	0	0	1	0	5.26	5.21	5.56	5.49	5.54	5.90	5.59
1392082_a_at	Cd7	0	0	0	0	0	0	4.99	5.21	4.79	4.99	5.21	1	0	0	0	0	1	1	5.36	5.33	5.37	5.33	5.63	7.53	8.30
1392107_at	Sbno2	0	0	0	0	0	0	4.25	4.33	4.14	4.50	4.13	1	0	0	0	0	0	1	7.67	7.78	7.79	7.64	7.67	7.16	5.99
1392111_at	Exosc3	0	0	0	0	0	0	6.93	6.80	7.29	7.25	6.95	1	0	0	0	0	0	1	9.53	9.53	9.58	9.63	9.55	9.15	8.43
1392120_at	---	0	0	0	0	0	0	5.03	4.78	5.46	4.88	4.98	1	0	0	0	0	1	1	6.15	6.11	6.21	6.31	6.29	6.82	6.72
1392144_at	Nt5dc1	0	0	0	0	0	0	5.43	5.43	5.79	5.53	5.54	1	0	0	0	0	1	0	6.58	6.67	6.81	6.99	6.96	7.32	6.80
1392149_at	LOC102557354	0	0	0	0	0	0	6.18	6.09	6.17	5.99	5.91	1	0	0	0	0	0	1	8.07	8.05	8.08	8.06	8.15	8.58	8.69
1392184_at	---	0	0	0	0	0	0	6.52	6.49	6.07	6.22	6.16	1	0	0	0	0	1	1	6.60	6.53	6.47	6.46	6.38	5.81	5.26
1392188_at	---	0	0	0	0	0	0	4.86	4.98	4.92	5.21	4.92	1	0	0	0	0	0	1	7.21	7.05	7.17	6.98	7.01	6.80	6.25
1392232_at	Gm15483	0	0	0	0	0	0	4.89	4.95	4.80	4.94	4.78	1	0	0	0	0	1	1	7.98	7.98	8.09	8.18	8.05	7.18	6.75
1392249_at	EPB41L2	0	0	0	0	0	0	5.44	5.38	5.40	5.57	5.70	1	0	0	0	0	0	1	9.43	9.62	9.58	9.50	9.63	9.90	9.39
1392264_s_at	Serpine1	0	0	0	0	0	0	3.74	3.67	3.47	3.57	3.61	1	0	0	0	0	1	0	4.89	5.06	5.14	5.09	5.26	5.74	5.50
1392272_at	LOC102546594	0	0	0	0	0	0	5.27	5.19	5.03	5.17	5.03	1	0	0	0	0	1	1	7.04	7.02	6.92	6.74	6.65	6.20	5.99
1392279_at	Dnai1	0	0	0	0	0	0	5.07	5.35	5.24	5.17	5.32	1	0	0	0	0	1	1	6.41	6.46	6.62	6.61	6.84	7.68	8.18
1392335_at	---	0	0	0	0	0	0	4.34	4.52	4.33	4.45	4.39	1	0	0	0	0	0	1	6.17	5.98	6.13	6.05	5.87	5.97	5.45
1392343_at	---	0	0	0	0	0	0	3.56	3.51	3.50	3.53	3.38	1	0	0	0	0	0	1	6.11	5.95	6.02	6.15	5.96	5.60	5.39
1392382_at	Tgfb2	0	0	0	0	0	0	5.21	5.44	5.66	5.13	5.24	1	0	0	0	0	1	0	5.27	5.29	5.17	4.87	4.92	4.61	4.34
1392386_at	---	0	0	0	0	0	0	6.60	6.76	7.09	6.47	6.77	1	0	0	0	0	1	1	7.29	7.25	7.29	7.26	7.22	6.16	5.16
1392397_at	---	0	0	0	0	0	0	3.27	3.38	3.34	3.36	3.24	1	0	0	0	0	1	1	4.73	4.77	4.90	5.03	5.05	5.52	5.61
1392428_at	Spata20	0	0	0	0	0	0	4.36	4.23	4.10	4.24	4.24	1	0	0	0	0	1	0	5.76	6.08	6.18	6.21	6.22	6.69	6.35
1392440_at	Bard1	0	0	0	0	0	0	5.06	5.01	5.15	5.21	5.26	1	0	0	0	0	1	1	7.91	7.84	7.92	7.74	7.61	6.58	6.44
1392451_at	C1orf127	0	0	0	0	0	0	6.82	6.96	7.07	6.72	6.82	1	0	0	0	0	1	1	7.69	7.63	7.91	8.16	7.79	6.86	6.23
1392471_at	Gng12	0	0	0	0	0	0	8.66	8.71	9.17	8.51	8.90	1	0	0	0	0	1	1	8.21	8.15	8.23	8.50	8.47	9.17	9.75
1392508_at	Eri1	0	0	0	0	0	0	4.18	4.47	4.67	4.42	4.42	1	0	0	0	0	0	1	8.55	8.55	8.50	8.60	8.62	8.96	9.09
1392512_at	H2bc5	0	0	0	0	0	0	4.96	5.05	5.18	5.19	5.37	1	0	0	0	0	1	1	6.92	6.93	6.94	6.84	6.76	6.26	5.73
1392527_at	Dnajc19	0	0	0	0	0	0	9.04	8.90	9.04	9.20	8.98	1	0	0	0	0	0	1	11.16	11.11	11.07	11.09	11.04	10.61	9.97
1392533_at	---	0	0	0	0	0	0	5.19	5.12	4.98	5.05	4.99	1	0	0	0	0	1	1	5.71	5.78	5.94	6.07	5.99	6.41	6.26
1392547_at	C15orf48	0	0	0	0	0	0	3.27	3.34	3.16	3.26	3.24	1	0	0	0	0	1	1	4.83	5.01	4.78	5.06	5.02	5.51	5.60
1392550_at	Dact1	0	0	0	0	0	0	5.57	5.44	5.88	5.32	5.74	1	0	0	0	0	1	0	7.63	7.76	7.80	7.84	7.99	8.30	8.11
1392553_at	Adamtsl5	0	0	0	0	0	0	4.31	4.36	4.26	4.30	4.37	1	0	0	0	0	1	1	6.22	6.46	6.32	6.44	6.69	7.51	7.25
1392562_at	Mzt2a	0	0	0	0	0	0	7.51	7.44	7.40	7.47	7.28	1	0	0	0	0	1	1	8.49	8.48	8.51	8.52	8.47	7.90	7.48
1392568_at	RGD1562272	0	0	0	0	0	0	4.33	4.45	4.47	4.69	4.66	1	0	0	0	0	1	1	6.65	6.69	6.82	6.92	6.95	7.35	7.25
1392572_at	Msi2	0	0	0	0	0	0	3.97	4.02	4.07	3.96	4.00	1	0	0	0	0	1	0	6.26	6.15	6.17	5.92	5.74	5.55	5.11
1392592_at	Tcf7l2	0	0	0	0	0	0	3.45	3.28	3.31	3.61	3.29	1	0	0	0	0	1	1	5.65	5.68	5.80	5.62	5.84	6.24	5.97
1392597_at	Aif1l	0	0	0	0	0	0	6.21	6.13	5.86	6.10	6.05	1	0	0	0	0	0	1	6.81	6.97	6.92	6.87	6.92	6.90	7.20
1392600_a_at	Maml2	0	0	0	0	0	0	5.62	5.75	5.75	5.69	5.49	1	0	0	0	0	1	1	5.22	5.17	5.44	5.19	5.17	5.85	5.94
1392616_at	Magt1	0	0	0	0	0	0	9.36	9.43	9.61	9.57	9.80	1	0	0	0	0	1	1	9.28	9.31	9.29	9.49	9.63	9.97	9.86
1392621_at	Tcf25	0	0	0	0	0	0	9.66	9.37	9.55	9.26	9.27	1	0	0	0	0	0	1	9.44	9.51	9.51	9.49	9.46	9.26	8.33
1392626_at	Pkmyt1	0	0	0	0	0	0	5.43	5.35	5.33	5.58	5.40	1	0	0	0	0	1	1	7.94	7.81	7.86	7.78	7.64	7.18	6.93
1392660_at	Traf6	0	0	0	0	0	0	6.46	6.52	6.72	6.71	6.84	1	0	0	0	0	0	1	8.46	8.32	8.41	8.34	8.31	8.76	9.23
1392666_at	---	0	0	0	0	0	0	4.33	4.63	4.50	4.39	4.47	1	0	0	0	0	0	1	3.45	3.44	3.57	3.57	3.44	3.77	4.02
1392701_at	Gmppb	0	0	0	0	0	0	6.80	7.04	6.57	6.88	7.00	1	0	0	0	0	1	1	7.75	7.83	7.81	7.70	7.60	7.09	6.90
1392713_a_at	LOC100362572 /	0	0	0	0	0	0	5.55	5.40	5.71	5.67	5.77	1	0	0	0	0	0	1	4.49	4.59	4.51	4.63	4.67	4.97	5.28
1392715_at	Ppargc1b	0	0	0	0	0	0	5.79	5.81	6.09	6.12	5.94	1	0	0	0	0	0	1	6.73	6.68	6.71	6.73	6.77	7.15	7.45
1392720_at	Cyp4f17 /// Cyp4	0	0	0	0	0	0	4.54	4.92	4.86	4.51	4.79	1	0	0	0	0	1	0	4.66	4.85	4.96	5.01	5.08	5.57	5.60
1392725_at	Ggact	0	0	0	0	0	0	4.53	4.33	4.28	4.61	4.14	1	0	0	0	0	0	1	5.96	6.11	6.02	6.07	5.84	5.51	4.99
1392732_at	Nusap1	0	0	0	0	0	0	6.27	6.17	6.32	6.23	6.43	1	0	0	0	0	1	1	10.36	10.29	10.32	10.29	10.18	9.02	8.66
1392733_at	Mipol1	0	0	0	0	0	0	3.76	3.82	4.22	3.85	3.82	1	0	0	0	0	1	1	7.21	7.13	7.26	7.14	6.96	6.21	6.22

Probeset ID	Gene Symbol	Islet Data Set (log2 ratio ≥0.263; FDR <20%) union n=5325 1=YES 0=NO	Islet DRlyp/lyp ND vs ND+Lp299v (log2 ratio ≥0.263; FDR<20%) n=429 1=YES 0=NO	Islet DRlyp/lyp ND vs HCD (log2 ratio ≥0.263; FDR<20%) n=2247 1=YES 0=NO	Islet DRlyp/lyp ND vs HCD+Lp299v (log2 ratio ≥0.263; FDR<20%) n=1595 1=YES 0=NO	Islet DRlyp/lyp HCD vs HCD+Lp299v (log2 ratio ≥0.263; FDR<20%) n=3026 1=YES 0=NO	Islet DRlyp/lyp ND vs Flyp/lyp ND (log2 ratio ≥ 0.263; FDR<20%) n=1950 1=YES 0=NO	Islet Mean Log2 Intensity DRlyp/lyp ND	Islet Mean Log2 Intensity DRlyp/lyp ND+Lp299v	Islet Mean Log2 Intensity DRlyp/lyp HCD	Islet Mean Log2 Intensity DRlyp/lyp HCD+Lp299v	Islet Mean Log2 Intensity Flyp40 ND	Rinm5f data union of all conditions n=3705 1=YES 0=NO	Rinm5f 30 µM propionate/6 µM butyrate vs Media (FDR<20%; 1.5 fold) n=3 1=YES 0=NO	Rinm5f 60 µM propionate/12 µM butyrate vs Media (FDR<20%; 1.5 fold) n=18 1=YES 0=NO	Rinm5f 120 µM propionate/24 µM butyrate vs Media (FDR<20%; 1.5 fold) n=164 1=YES 0=NO	Rinm5f 240 µM propionate /48 µM butyrate vs Media (FDR<20%; 1.5 fold) n=268 1=YES 0=NO	Rinm5f 1500 µM propionate/300 µM butyrate vs Media (FDR<20%; 1.5 fold) n=2491 1=YES 0=NO	Rinm5f 500 µM propionate/500 µM butyrate vs Media (FDR<20%; 1.5 fold) n=3001 1=YES 0=NO	Rinm5f Mean Log2 Intensity Media only	Rinm5f Mean Log2 Intensity 30 µM propionate/6 µM butyrate	Rinm5f Mean Log2 Intensity 60 µM propionate/12 µM butyrate	Rinm5f Mean Log2 Intensity 120 µM propionate/24 µM butyrate	Rinm5f Mean Log2 Intensity 240 µM propionate/48 µM butyrate	Rinm5f Mean Log2 Intensity 1500 µM propionate /300 µM butyrate	Rinm5f Mean Log2 Intensity 500 µM propionate/500 µM butyrate
1392739_a_at	Eepd1	0	0	0	0	0	0	4.99	5.13	4.85	5.01	4.89	1	0	0	0	0	1	0	6.57	6.73	6.83	6.76	6.87	7.28	7.66
1392754_at	Adam8	0	0	0	0	0	0	4.04	4.25	3.95	3.94	4.02	1	0	0	0	0	1	1	4.93	4.89	5.03	5.05	5.10	5.62	6.18
1392769_at	Arxes1 /// Arxes	0	0	0	0	0	0	4.94	5.00	5.49	4.98	5.04	1	0	0	0	1	1	1	5.74	6.00	6.19	6.30	6.42	6.96	6.78
1392773_at	Pcsk5	0	0	0	0	0	0	5.83	5.95	5.76	5.95	5.81	1	0	0	0	0	1	0	5.90	5.84	5.73	5.38	5.44	5.31	5.24
1392784_at	Gas6	0	0	0	0	0	0	6.63	6.54	6.32	6.73	6.60	1	0	0	1	0	1	1	8.90	8.91	8.77	8.25	8.53	7.85	7.44
1392796_at	Cnot6l	0	0	0	0	0	0	4.45	4.46	4.85	4.52	4.59	1	0	0	0	0	1	0	5.91	5.63	5.77	5.87	5.79	5.29	4.76
1392800_at	Msantd3	0	0	0	0	0	0	4.82	4.70	4.64	4.97	4.71	1	0	0	0	0	1	0	6.32	6.23	6.32	6.11	5.88	5.66	5.65
1392818_at	Gas5	0	0	0	0	0	0	7.38	7.31	7.75	7.28	7.71	1	0	0	0	0	1	1	10.19	10.24	10.36	10.57	10.56	9.51	8.09
1392820_at	Fgf1	0	0	0	0	0	0	3.25	3.31	3.08	3.18	3.13	1	0	0	0	0	1	1	7.24	7.21	7.20	6.96	7.00	6.10	4.85
1392834_at	C17orf64	0	0	0	0	0	0	3.56	3.58	3.45	3.44	3.46	1	0	0	0	0	1	1	4.94	4.86	4.96	5.05	5.38	5.93	6.60
1392844_at	Znf236	0	0	0	0	0	0	4.46	4.59	4.70	4.48	4.82	1	0	0	0	0	1	1	6.68	6.56	6.67	6.79	6.64	6.04	5.49
1392887_at	---	0	0	0	0	0	0	6.28	6.32	6.33	6.00	6.40	1	0	0	0	0	1	0	8.14	8.16	8.37	8.49	8.54	8.93	8.66
1392940_at	Ptpdc1	0	0	0	0	0	0	6.77	6.84	6.92	7.18	6.62	1	0	0	0	0	1	1	7.45	7.48	7.63	7.66	7.79	8.28	8.42
1392953_at	Hacd1	0	0	0	0	0	0	6.20	5.97	6.54	6.28	6.08	1	0	0	0	0	1	1	7.68	7.63	7.53	7.42	7.23	6.68	6.19
1392994_at	Nmrk1	0	0	0	0	0	0	5.48	5.08	5.73	5.54	5.37	1	0	0	0	0	1	1	7.81	7.79	7.98	7.99	8.02	8.63	8.73
1393017_at	Rhpn1	0	0	0	0	0	0	6.04	6.03	5.97	5.99	6.05	1	0	0	0	0	1	1	7.97	7.95	8.12	7.89	7.95	8.75	8.26
1393018_at	LOC100912649	0	0	0	0	0	0	4.02	4.01	3.93	3.98	4.00	1	0	0	0	0	1	1	5.41	5.28	5.51	5.74	5.91	6.03	7.00
1393036_at	Psrc1	0	0	0	0	0	0	4.54	4.42	4.28	4.61	4.45	1	0	0	0	0	1	1	7.60	7.47	7.52	7.48	7.30	6.75	6.75
1393059_at	Znf799	0	0	0	0	0	0	4.81	4.98	5.16	4.98	5.21	1	0	0	0	0	1	0	5.27	5.52	5.54	5.67	5.78	5.91	5.79
1393062_at	Fam219b	0	0	0	0	0	0	6.62	6.67	6.71	6.63	6.64	1	0	0	0	0	0	1	7.39	7.32	7.33	7.29	7.27	6.85	6.28
1393103_at	Mettl22	0	0	0	0	0	0	5.97	5.90	5.98	6.16	5.74	1	0	0	0	0	1	1	8.66	8.65	8.71	8.61	8.54	8.07	7.68
1393129_at	P4ha3	0	0	0	0	0	0	4.16	4.28	3.98	4.10	4.36	1	0	0	0	0	1	1	5.59	5.87	5.95	5.84	5.99	6.85	7.01
1393148_at	Gpr137b	0	0	0	0	0	0	7.06	7.18	7.37	6.86	7.53	1	0	0	0	0	0	1	7.86	7.82	7.92	7.72	7.78	8.37	8.67
1393157_at	Doc2b	0	0	0	0	0	0	3.47	3.62	3.43	3.35	3.59	1	0	0	0	0	1	0	10.65	10.61	10.49	10.07	10.39	9.83	9.26
1393158_at	Cenpq	0	0	0	0	0	0	3.76	3.59	3.68	3.74	3.71	1	0	0	0	0	1	1	7.66	7.58	7.59	7.72	7.55	6.70	6.68
1393160_at	Tbx3	0	0	0	0	0	0	3.15	3.13	2.99	3.27	3.10	1	0	0	1	1	0	0	6.01	6.11	6.44	6.66	6.68	6.48	6.61
1393181_at	Nfasc	0	0	0	0	0	0	4.63	4.60	4.86	4.49	4.83	1	0	0	0	0	1	0	7.76	7.84	7.97	8.06	8.10	8.39	8.12
1393189_at	---	0	0	0	0	0	0	4.70	4.79	5.06	4.55	5.10	1	0	0	0	0	0	1	5.52	5.23	5.47	5.57	5.56	5.94	6.55
1393191_at	Nkain1	0	0	0	0	0	0	5.89	5.73	5.64	5.76	5.77	1	0	0	0	0	0	1	7.45	7.51	7.48	7.56	7.63	7.82	7.91
1393212_at	LOC102554019	0	0	0	0	0	0	5.20	5.40	5.30	5.51	5.13	1	0	0	0	0	0	1	7.42	7.42	7.41	7.41	7.30	6.89	6.75
1393223_a_at	Rita1	0	0	0	0	0	0	5.79	5.72	5.47	5.67	5.63	1	0	0	0	0	1	0	7.44	7.61	7.57	7.61	7.56	8.09	8.02
1393226_at	Itgav	0	0	0	0	0	0	5.91	6.10	5.94	6.32	6.11	1	0	0	0	0	1	0	7.39	7.54	7.58	7.75	7.81	8.44	7.73
1393231_at	Ppp4r2	0	0	0	0	0	0	6.42	6.40	6.98	6.45	6.43	1	0	0	0	1	1	1	9.11	9.28	9.35	9.59	9.71	10.04	9.94
1393233_at	Armc6	0	0	0	0	0	0	6.85	7.02	6.57	7.06	6.79	1	0	0	0	0	1	1	8.29	8.26	8.39	8.43	8.24	7.64	7.36
1393261_at	LOC102547242	0	0	0	0	0	0	5.86	5.82	6.20	5.79	5.73	1	0	0	0	0	1	1	7.84	7.79	7.93	7.66	7.62	6.65	6.08
1393274_at	Ndufaf7	0	0	0	0	0	0	3.92	4.02	4.00	3.74	4.38	1	0	0	0	0	1	0	6.12	6.20	6.35	6.44	6.47	6.75	6.77
1393282_at	Spns2	0	0	0	0	0	0	5.32	5.23	5.11	5.16	5.35	1	0	0	0	0	1	0	7.69	7.73	7.93	7.86	8.00	8.42	7.97
1393302_at	---	0	0	0	0	0	0	4.08	4.42	4.16	4.24	4.28	1	0	0	0	0	1	0	6.49	6.61	6.79	6.96	6.97	7.16	6.82
1393307_at	Phactr3	0	0	0	0	0	0	3.26	3.33	3.28	3.37	3.35	1	0	0	0	0	1	1	5.35	5.34	5.43	5.46	5.49	6.01	6.21
1393314_at	Fgf1	0	0	0	0	0	0	5.81	6.02	5.78	5.90	5.58	1	0	0	0	0	1	1	9.62	9.57	9.49	9.20	9.31	8.58	8.23
1393336_at	Swap70	0	0	0	0	0	0	5.84	5.67	5.79	6.01	5.97	1	0	0	0	0	1	1	6.45	6.59	6.47	6.51	6.62	7.10	7.62
1393344_at	---	0	0	0	0	0	0	6.30	6.31	6.44	6.47	6.54	1	0	0	0	0	1	0	8.72	8.67	8.93	8.98	8.97	9.33	9.34
1393350_at	LOC103694321	0	0	0	0	0	0	3.09	3.13	3.29	3.29	3.27	1	0	0	0	0	0	1	4.41	4.50	4.38	4.25	4.19	3.83	3.41
1393356_at	Pde5a	0	0	0	0	0	0	4.44	4.57	4.89	4.61	4.58	1	0	0	0	0	1	0	5.04	4.88	5.13	5.23	5.26	5.69	5.83
1393357_at	Gle1	0	0	0	0	0	0	6.13	6.11	6.12	6.13	5.92	1	0	0	0	0	1	1	7.59	7.60	7.60	7.45	7.45	6.76	6.20
1393369_at	Cdkn2aip	0	0	0	0	0	0	4.57	4.48	5.06	4.50	4.33	1	0	0	0	0	1	0	6.88	6.98	7.13	7.39	7.41	7.49	7.27
1393373_at	Opn3	0	0	0	0	0	0	4.68	4.54	4.58	4.63	5.12	1	0	0	0	0	1	0	8.32	8.42	8.57	8.56	8.44	7.65	7.87
1393387_at	Endou	0	0	0	0	0	0	3.29	3.27	3.04	3.08	3.07	1	0	0	0	0	1	1	3.63	3.83	3.82	3.81	3.74	4.28	5.36
1393397_at	Cpa2	0	0	0	0	0	0	14.62	14.62	14.60	14.62	14.59	1	0	0	0	0	1	0	7.25	7.21	7.27	7.14	6.96	6.60	5.95
1393401_at	Ecscr	0	0	0	0	0	0	5.07	5.04	4.98	4.65	4.85	1	0	0	0	1	1	1	6.81	6.84	7.05	7.18	7.41	8.04	8.30
1393405_at	Xrcc2	0	0	0	0	0	0	4.50	4.56	4.38	4.51	4.58	1	0	0	0	0	1	1	6.47	6.45	6.62	6.57	6.45	5.68	5.81
1393428_at	Atxn3	0	0	0	0	0	0	5.25	5.28	5.64	5.22	5.31	1	0	0	0	0	1	1	6.24	6.25	6.57	6.56	6.50	6.93	7.30
1393441_at	Cdkn2d	0	0	0	0	0	0	6.14	6.35	6.39	5.98	6.52	1	0	0	0	0	1	1	10.38	10.34	10.34	10.31	10.20	9.78	9.34
1393443_a_at	Emc9	0	0	0	0	0	0	5.90	6.38	5.97	6.26	6.03	1	0	0	0	0	1	1	5.75	5.77	6.17	6.01	6.06	6.57	6.24
1393451_at	Cenpn	0	0	0	0	0	0	6.03	6.34	6.25	6.46	6.49	1	0	0	0	0	1	1	8.41	8.38	8.31	8.37	8.26	7.70	7.63
1393454_at	Pcdh17	0	0	0	0	0	0	3.47	3.29	3.38	3.26	3.19	1	0	0	0	0	1	1	6.08	6.13	6.43	6.47	6.55	8.03	7.73
1393477_at	---	0	0	0	0	0	0	5.11	5.47	5.19	5.18	5.31	1	0	0	0	0	0	1	5.60	5.45	5.53	5.47	5.52	5.94	6.20
1393478_at	Pde4d	0	0	0	0	0	0	8.03	8.03	8.24	8.26	8.14	1	0	0	0	0	0	1	9.84	9.92	9.85	9.76	9.82	9.34	8.79
1393487_at	Cenpa	0	0	0	0	0	0	3.22	3.33	3.26	3.31	3.32	1	0	0	0	0	1	0	6.60	6.52	6.63	6.57	6.40	5.97	5.89
1393489_at	Spaca9	0	0	0	0	0	0	3.61	3.71	3.58	3.62	3.43	1	0	0	0	0	1	0	4.70	4.51	4.69	4.76	4.79	5.50	5.05
1393550_at	Crem	0	0	0	0	0	0	3.97	3.84	3.89	4.00	3.85	1	0	0	0	0	0	1	6.50	6.54	6.75	6.70	6.71	6.05	5.14
1393553_at	Cdkl4	0	0	0	0	0	0	4.35	4.74	4.87	4.76	4.49	1	0	0	0	0	1	1	4.26	4.26	4.24	4.39	4.50	5.40	5.46
1393575_at	LOC102553298	0	0	0	0	0	0	3.36	3.38	3.20	3.34	3.31	1	0	0	0	0	1	0	5.32	5.19	5.25	5.24	4.99	4.69	4.31
1393581_at	Aspm	0	0	0	0	0	0	4.13	4.14	4.49	4.28	4.47	1	0	0	0	0	1	1	9.27	9.18	9.26	9.21	8.99	7.95	8.21
1393582_at	---	0	0	0	0	0	0	7.50	7.59	7.84	7.54	7.77	1	0	0	0	0	1	1	5.88	6.02	5.93	6.15	6.12	6.80	7.05
1393598_at	Ccna1	0	0	0	0	0	0	4.30	4.26	4.12	4.44	4.26	1	0	0	0	0	1	0	6.91	6.95	6.79	6.57	6.49	5.94	5.95
1393619_at	Cnot6l	0	0	0	0	0	0	7.04	7.00	7.53	7.21	7.19	1	0	0	0	0	0	1	8.76	8.72	8.78	8.85	8.71	8.42	7.78
1393620_at	Sesn3	0	0	0	0	0	0	5.81	5.57	5.50	5.72	5.58	1	0	0	0	0	0	1	6.39	6.41	6.49	6.34	6.35	6.66	7.35
1393627_at	Mgarp	0	0	0	0	0	0	2.90	3.00	2.93	2.92	2.97	1	0	0	0	0	0	1	7.02	7.19	7.03	6.80	6.87	7.34	8.27
1393638_at	Ptger4	0	0	0	0	0	0	3.35	3.39	3.18	3.38	3.44	1	0	0	0	0	0	1	4.27	4.21	4.23	4.48	4.48	4.78	5.47
1393655_at	Rad54l	0	0	0	0	0	0	3.89	4.12	3.92	4.07	4.01	1	0	0	0	0	1	1	7.54	7.44	7.60	7.43	7.35	6.81	6.50
1393683_at	Ecpas	0	0	0	0	0	0	6.68	6.38	6.78	6.29	6.50	1	0	0	0	0	1	1	7.08	6.96	7.05	7.11	6.93	6.32	5.51
1393684_at	Hells /// LOC100	0	0	0	0	0	0	3.32	3.30	3.48	3.21	3.44	1	0	0	0	0	1	1	8.61	8.65	8.69	8.97	8.93	9.27	9.12
1393691_at	Kif26a	0	0	0	0	0	0	3.49	3.57	3.80	3.47	3.45	1	0	0	0	0	0	1	6.51	6.51	6.83	6.47	6.65	7.09	7.32
1393698_at	---	0	0	0	0	0	0	6.70	6.69	6.48	6.60	6.62	1	0	0	0	0	1	0	7.75	7.67	7.73	7.48	7.41	7.11	7.20
1393706_at	Steap1	0	0	0	0	0	0	4.28	4.68	4.11	4.36	4.21	1	0	0	0	0	0	1	6.34	6.18	6.17	5.90	6.03	6.46	6.96
1393715_s_at	T2	0	0	0	0	0	0	3.26	3.40	3.30	3.52	3.24	1	0	0	0	0	1	1	4.99	4.97	5.22	5.29	5.15	5.64	5.69
1393723_at	Plk5	0	0	0	0	0	0	4.95	5.04	4.68	5.00	4.81	1	0	0	0	0	1	1	8.62	8.64	8.66	8.66	8.74	9.23	9.08
1393728_at	---	0	0	0	0	0	0	5.69	5.60	5.45	5.58	5.70	1	0	0	1	0	1	1	8.91	8.94	8.86	8.31	8.63	7.03	6.81
1393730_at	Adamts4	0	0	0	0	0	0	4.11	4.07	3.94	4.24	3.99	1	0	0	0	0	1	0	3.77	3.96	4.01	3.99	4.21	4.44	4.88
1393750_at	G2e3	0	0	0	0	0	0	3.09	3.18	3.52	3.19	3.22	1	0	0	0	0	1	1	5.91	5.81	5.83	5.86	5.73	5.04	4.72
1393754_at	Tbc1d1	0	0	0	0	0	0	3.87	3.99	3.74	3.68	3.76	1	0	0	0	0	1	1	6.36	6.31	6.49	6.46	6.59	7.38	7.45
1393760_at	Wars2	0	0	0	0	0	0	5.63	5.68	5.85	5.73	5.70	1	0	0	0	0	1	1	6.66	6.64	6.69	6.51	6.44	6.05	5.50
1393762_at	Kat2b	0	0	0	0	0	0	3.87	3.80	3.86	3.62	3.79	1	0	0	0	0	0	1	5.38	5.32	5.51	5.50	5.47	4.92	4.17
1393766_at	---	0	0	0	0	0	0	5.78	5.94	6.00	6.07	5.54	1	0	0	0	0	1	0	5.46	5.62	5.67	5.69	5.62	6.26	5.18
1393793_at	---	0	0	0	0	0	0	3.84	3.93	3.65	3.87	3.87	1	0	0	0	0	1	1	9.53	9.64	9.47	9.10	9.17	8.50	8.11
1393799_at	Unc5b	0	0	0	0	0	0	5.86	5.82	5.97	5.88	5.93	1	0	0	0	0	1	1	6.80	6.88	6.86	6.79	6.71	7.61	8.02
1393803_at	Casp8ap2	0	0	0	0	0	0	5.01	4.93	5.23	4.95	4.97	1	0	0	0	0	1	0	8.17	8.25	8.43	8.65	8.71	8.78	8.69
1393816_at	Cenpw	0	0	0	0	0	0	3.36	3.23	3.69	3.51	3.65	1	0	0	0	0	1	1	8.50	8.55	8.48	8.39	8.29	6.74	6.38
1393824_at	Tmem170a	0	0	0	0	0	0	6.01	5.82	6.55	6.09	5.99	1	0	0	0	0	1	1	6.30	6.26	6.47	6.32	6.56	7.01	7.14
1393836_at	Mitf	0	0	0	0	0	0	5.01	5.19	4.77	5.22	5.15	1	0	0	0	0	1	1	6.40	6.29	6.39	6.38	6.44	7.14	7.62
1393844_at	Cenpu	0	0	0	0	0	0	4.01	3.99	3.92	4.32	4.05	1	0	0	0	0	1	1	7.32	7.29	7.25	7.29	7.12	6.26	6.22
1393848_at	Rrm2	0	0	0	0	0	0	3.67	3.68	3.90	3.52	3.78	1	0	0	0	0	1	1	10.50	10.38	10.51	10.56	10.33	9.62	8.83
1393858_at	Aunip	0	0	0	0	0	0	3.00	2.79	2.78	3.00	2.85	1	0	0	0	0	1	1	6.78	6.73	6.83	6.82	6.77	6.07	5.49
1393872_at	Sel1l3	0	0	0	0	0	0	3.86	3.93	4.06	4.22	4.09	1	0	0	0	0	1	0	4.59	4.61	4.85	4.84	5.02	5.42	5.19
1393883_at	Flt4	0	0	0	0	0	0	4.97	5.18	5.00	5.13	5.11	1	0	0	0	0	0	1	3.65	3.55	3.61	3.58	3.57	4.17	4.73
1393887_at	Chpt1	0	0	0	0	0	0	6.42	6.34	6.43	6.66	6.37	1	0	0	0	0	1	1	6.39	6.19	6.07	6.00	5.96	5.77	5.37
1393893_at	Morn5	0	0	0	0	0	0	3.46	3.52	3.29	3.30	3.32	1	0	0	0	0	1	1	4.52	4.40	4.66	4.49	4.56	5.44	6.23
1393894_at	Cyp4a22	0	0	0	0	0	0	5.14	5.07	4.81	5.19	5.13	1	0	0	1	1	0	0	6.79	6.98	7.27	7.38	7.66	7.13	7.06
1393898_at	---	0	0	0	0	0	0	3.91	4.05	3.80	3.99	3.84	1	0	0	0	0	1	1	7.11	7.08	6.98	6.89	6.85	6.33	5.95
1393904_at	Lin28a	0	0	0	0	0	0	4.37	4.39	4.06	4.53	4.44	1	0	0	0	0	1	1	5.27	5.21	5.23	5.11	5.37	5.93	5.86
1393905_at	Exoc1	0	0	0	0	0	0	3.92	3.82	3.83	3.66	3.82	1	0	0	0	0	0	1	7.01	6.92	6.98	6.85	6.78	6.63	6.04
1393912_at	---	0	0	0	0	0	0	6.33	6.26	6.41	6.36	6.39	1	0	0	0	0	1	1	7.07	7.17	7.25	7.15	7.36	7.67	7.71
1393928_at	---	0	0	0	0	0	0	3.70	3.54	4.11	3.84	3.68	1	0	0	0	0	1	1	4.78	4.95	4.97	4.99	5.02	5.54	5.42
1393938_at	---	0	0	0	0	0	0	3.84	3.97	4.17	3.81	4.11	1	0	0	0	0	1	0	5.83	5.99	6.13	6.04	6.26	6.45	5.01
1393945_at	---	0	0	0	0	0	0	2.70	2.80	2.62	2.70	2.67	1	0	0	0	0	1	1	6.64	6.70	6.75	7.03	6.82	5.61	5.19
1393959_at	Sbno1	0	0	0	0	0	0	7.44	7.48	7.76	7.71	7.69	1	0	0	0	0	1	0	9.33	9.43	9.50	9.52	9.62	9.93	9.79
1393963_at	Nthl1	0	0	0	0	0	0	4.17	4.10	3.74	4.12	3.89	1	0	0	0	0	0	1	5.28	5.30	5.30	5.41	5.54	5.70	5.95
1393987_s_at	Grk6	0	0	0	0	0	0	8.69	8.75	8.41	8.76	8.36	1	0	0	0	0	0	1	9.21	9.20	9.20	9.24	9.22	9.54	9.78
1393994_at	Flt3lg	0	0	0	0	0	0	5.78	5.72	5.60	5.85	5.84	1	0	0	0	0	1	0	6.93	6.79	6.79	6.67	6.64	6.32	6.10
1394031_at	Fam83d	0	0	0	0	0	0	3.65	3.73	3.66	3.67	4.00	1	0	0	0	0	1	1	6.77	6.67	6.69	6.70	6.51	5.76	5.96
1394077_at	Rnd3	0	0	0	0	0	0	4.35	4.55	4.59	4.14	4.58	1	0	0	0	0	0	1	4.33	4.24	4.24	4.21	4.12	4.47	4.75
1394100_at	Ffar2	0	0	0	0	0	0	5.32	5.23	5.22	5.20	4.90	1	0	0	0	0	0	1	6.02	6.29	5.95	5.90	5.83	5.77	5.33
1394200_at	Hspa2	0	0	0	0	0	0	2.93	3.01	3.08	2.81	3.00	1	0	0	0	0	1	1	4.58	4.73	4.93	5.00	5.09	5.33	5.71
1394209_at	Galnt4	0	0	0	0	0	0	6.12	6.19	6.09	6.35	6.17	1	0	0	0	0	1	1	4.68	4.68	4.87	4.77	4.80	5.29	5.92
1394252_at	Spock3	0	0	0	0	0	0	2.90	2.88	2.74	2.82	2.74	1	0	0	0	0	0	1	4.26	4.32	4.27	4.25	4.18	4.41	4.18
1394258_at	---	0	0	0	0	0	0	2.91	3.05	2.90	2.94	2.92	1	0	0	0	0	1	1	7.18	7.21	7.30	7.41	7.45	6.42	5.11
1394270_at	Mks1	0	0	0	0	0	0	5.77	5.78	5.91	5.64	5.90	1	0	0	0	0	0	1	7.44	7.40	7.47	7.45	7.43	7.14	6.65
1394280_at	Rgs17	0	0	0	0	0	0	6.56	6.61	6.78	6.20	6.52	1	0	0	0	0	0	1	9.80	9.82	9.81	9.79	9.86	9.62	8.13
1394292_at	Mrpl37	0	0	0	0	0	0	6.19	6.09	6.09	5.94	5.95	1	0	0	0	0	0	1	8.61	8.61	8.61	8.64	8.48	8.34	7.59
1394295_at	Tmem255a	0	0	0	0	0	0	2.70	2.68	2.68	2.67	2.64	1	0	0	0	0	1	1	5.70	5.85	5.97	6.06	6.25	6.89	5.81
1394320_at	Cdk2	0	0	0	0	0	0	5.49	5.19	5.19	5.26	5.17	1	0	0	0	0	1	0	8.47	8.46	8.52	8.56	8.65	9.15	8.85
1394340_at	Inpp1	0	0	0	0	0	0	4.71	4.43	4.97	4.40	4.75	1	0	0	0	0	0	1	6.84	6.91	6.83	6.77	6.75	6.42	5.79
1394359_at	Mtfr2	0	0	0	0	0	0	3.84	3.80	3.95	3.93	4.03	1	0	0	0	0	1	1	7.14	7.11	7.19	7.15	7.06	6.44	6.46
1394403_at	Spata20	0	0	0	0	0	0	3.80	3.82	3.60	3.88	3.63	1	0	0	0	0	1	1	5.99	6.00	6.34	6.33	6.29	6.86	6.98
1394407_at	Kiaa0930	0	0	0	0	0	0	5.91	6.02	5.90	5.96	6.09	1	0	0	0	0	1	1	7.39	7.51	7.65	7.78	7.79	8.42	8.62
1394446_at	Dnm3	0	0	0	0	0	0	3.70	3.57	3.52	3.68	3.58	1	0	0	0	0	1	1	4.35	4.40	4.48	4.49	4.55	4.95	6.00
1394455_at	FGF12	0	0	0	0	0	0	3.66	3.78	3.50	3.67	3.70	1	0	0	0	0	1	1	5.84	5.81	6.04	6.32	6.20	6.87	6.94
1394465_at	---	0	0	0	0	0	0	4.84	4.91	4.82	4.62	4.83	1	0	0	0	0	1	0	7.16	7.12	7.10	6.96	6.86	6.40	6.08
1394472_at	Rgl1	0	0	0	0	0	0	5.32	5.38	5.34	5.31	5.37	1	0	0	0	0	0	1	5.11	5.07	5.18	5.13	5.17	5.66	5.79
1394475_at	Wnt4	0	0	0	0	0	0	8.01	8.35	8.21	7.71	8.15	1	0	0	0	0	1	1	9.45	9.55	9.51	9.26	9.28	8.46	8.05
1394479_at	Afmid	0	0	0	0	0	0	5.22	5.06	5.15	5.28	5.28	1	0	0	0	0	0	1	6.72	6.63	6.72	6.68	6.78	7.01	6.89
1394489_at	Rnf32	0	0	0	0	0	0	3.55	3.51	3.65	3.51	3.61	1	0	0	0	0	1	0	4.09	4.27	4.39	4.36	4.30	4.68	4.59
1394509_at	---	0	0	0	0	0	0	4.65	4.80	4.67	4.69	4.84	1	0	0	0	0	0	1	5.91	5.91	6.12	6.24	6.00	5.62	4.98
1394530_at	---	0	0	0	0	0	0	6.23	6.33	6.77	6.15	6.65	1	0	0	0	0	0	1	7.27	7.31	7.51	7.36	7.42	7.14	6.08

Probeset ID	Gene Symbol	Islet Data Set (log2 ratio ≥0.263; FDR <20%) union n=5325 1=YES 0=NO	Islet DRlyp/lyp ND vs ND+Lp299v (log2 ratio ≥0.263; FDR<20%) n=429 1=YES 0=NO	Islet DRlyp/lyp ND vs HCD (log2 ratio ≥0.263; FDR<20%) n=2247 1=YES 0=NO	Islet DRlyp/lyp ND vs HCD+Lp299v (log2 ratio ≥0.263; FDR<20%) n=1595 1=YES 0=NO	Islet DRlyp/lyp HCD vs HCD+Lp299v (log2 ratio ≥0.263; FDR<20%) n=3026 1=YES 0=NO	Islet DRlyp/lyp ND vs Flyp/lyp ND (log2 ratio ≥ 0.263; FDR<20%) n=1950 1=YES 0=NO	Islet Mean Log2 Intensity DRlyp/lyp ND	Islet Mean Log2 Intensity DRlyp/lyp ND+Lp299v	Islet Mean Log2 Intensity DRlyp/lyp HCD	Islet Mean Log2 Intensity DRlyp/lyp HCD+Lp299v	Islet Mean Log2 Intensity Flyp40 ND	Rinm5f data union of all conditions n=3705 1=YES 0=NO	Rinm5f 30 µM propionate/6 µM butyrate vs Media (FDR<20%; 1.5 fold) n=3 1=YES 0=NO	Rinm5f 60 µM propionate/12 µM butyrate vs Media (FDR<20%; 1.5 fold) n=18 1=YES 0=NO	Rinm5f 120 µM propionate/24 µM butyrate vs Media (FDR<20%; 1.5 fold) n=164 1=YES 0=NO	Rinm5f 240 µM propionate/48 µM butyrate vs Media (FDR<20%; 1.5 fold) n=268 1=YES 0=NO	Rinm5f 1500 µM propionate/300 µM butyrate vs Media (FDR<20%; 1.5 fold) n=2491 1=YES 0=NO	Rinm5f 500 µM propionate/500 µM butyrate vs Media (FDR<20%; 1.5 fold) n=3001 1=YES 0=NO	Rinm5f Mean Log2 Intensity Media only	Rinm5f Mean Log2 Intensity 30 µM propionate/6 µM butyrate	Rinm5f Mean Log2 Intensity 60 µM propionate/12 µM butyrate	Rinm5f Mean Log2 Intensity 120 µM propionate/24 µM butyrate	Rinm5f Mean Log2 Intensity 240 µM propionate/48 µM butyrate	Rinm5f Mean Log2 Intensity 1500 µM propionate/300 µM butyrate	Rinm5f Mean Log2 Intensity 500 µM propionate/500 µM butyrate
1394533_at	---	0	0	0	0	0	0	3.98	3.92	3.69	3.92	3.89	1	0	0	0	0	0	1	4.33	4.17	4.21	4.18	4.03	4.47	4.65
1394539_at	---	0	0	0	0	0	0	4.64	4.65	4.31	4.56	4.51	1	0	0	0	0	0	1	6.68	6.82	6.85	6.64	6.78	7.08	6.94
1394548_at	Cpm	0	0	0	0	0	0	5.59	5.91	5.79	5.67	5.60	1	0	0	0	0	0	1	6.89	6.94	6.77	6.67	6.81	7.14	7.61
1394554_at	Ppih	0	0	0	0	0	0	5.92	5.79	5.58	5.70	5.76	1	0	0	0	0	1	1	7.36	7.29	7.38	7.26	7.16	6.57	6.38
1394561_at	Hapln3	0	0	0	0	0	0	4.83	4.90	4.72	4.86	4.77	1	0	0	0	0	1	0	6.55	6.77	6.64	6.80	6.84	7.42	7.13
1394576_at	---	0	0	0	0	0	0	5.76	5.91	5.89	6.22	6.03	1	0	0	0	0	1	1	8.52	8.67	8.64	8.47	8.76	9.11	9.42
1394577_at	Esrrg	0	0	0	0	0	0	6.39	6.20	6.75	6.47	6.27	1	0	0	0	0	1	1	5.25	5.33	5.52	5.60	5.56	5.92	6.37
1394590_at	Nkiras1	0	0	0	0	0	0	5.98	5.89	6.15	5.74	6.22	1	0	0	0	0	1	1	7.14	7.12	7.19	7.25	7.24	8.02	8.31
1394609_at	Ablim2	0	0	0	0	0	0	3.77	3.90	3.91	3.85	4.04	1	0	0	0	0	1	1	5.29	5.40	5.55	5.61	5.56	6.31	6.75
1394656_at	LOC102547990	0	0	0	0	0	0	4.68	4.77	4.36	4.55	4.63	1	0	0	0	0	0	1	7.12	7.09	7.11	7.06	7.32	7.58	7.78
1394668_at	Cadm3	0	0	0	0	0	0	4.24	4.34	4.03	4.09	4.16	1	0	0	0	0	1	0	4.46	4.58	4.71	4.89	4.82	5.43	4.89
1394678_at	Fgd2	0	0	0	0	0	0	4.40	4.31	4.45	4.32	4.45	1	0	0	0	0	1	1	5.38	5.49	5.48	5.82	5.75	6.40	7.12
1394698_at	---	0	0	0	0	0	0	5.80	5.59	5.80	5.80	5.87	1	0	0	0	0	0	1	6.86	6.86	6.96	6.78	6.82	6.46	6.07
1394699_at	Onecut2	0	0	0	0	0	0	3.78	3.86	3.63	3.88	3.88	1	0	0	0	0	1	1	7.44	7.44	7.57	7.71	7.83	8.61	9.37
1394710_at	Azi2	0	0	0	0	0	0	4.52	4.42	4.75	4.51	4.56	1	0	0	0	0	0	1	6.28	6.31	6.30	6.27	6.10	5.80	5.40
1394807_at	---	0	0	0	0	0	0	4.47	4.44	4.44	4.32	4.41	1	0	0	0	0	1	1	7.09	6.94	6.99	6.95	6.81	6.43	5.31
1394815_at	Tmem170a	0	0	0	0	0	0	6.71	6.84	6.73	7.07	6.44	1	0	0	0	0	1	0	6.47	6.50	6.56	6.52	6.57	7.09	7.37
1394869_at	Cep135	0	0	0	0	0	0	3.94	3.97	3.85	3.73	4.03	1	0	0	0	0	1	1	7.10	7.00	7.11	7.13	7.05	6.47	6.41
1394870_at	---	0	0	0	0	0	0	3.12	3.01	3.16	3.29	3.12	1	0	0	0	0	1	1	4.58	4.52	4.54	4.41	4.38	3.95	3.72
1394880_at	---	0	0	0	0	0	0	3.51	3.61	3.49	3.48	3.44	1	0	0	0	0	0	1	4.77	4.70	5.02	4.82	4.78	5.24	5.94
1394881_at	Fam81a	0	0	0	0	0	0	3.83	3.78	3.68	3.70	3.68	1	0	0	0	0	1	1	5.61	5.68	5.83	5.64	5.71	6.33	6.86
1394883_at	---	0	0	0	0	0	0	3.32	3.14	3.15	3.18	3.08	1	0	0	0	0	1	1	4.36	4.48	4.62	4.73	4.71	6.10	6.82
1394916_at	---	0	0	0	0	0	0	2.95	3.05	3.06	3.06	2.88	1	0	0	0	0	1	1	4.39	4.57	4.61	4.72	4.92	5.92	6.93
1394933_at	---	0	0	0	0	0	0	4.03	4.12	4.38	4.16	4.41	1	0	0	0	0	0	1	7.25	7.39	7.45	7.43	7.33	6.99	6.37
1394960_at	Sdr42e1	0	0	0	0	0	0	4.01	4.13	3.88	3.89	3.99	1	0	0	0	0	1	0	6.74	6.82	6.98	6.90	6.92	7.39	6.76
1394963_at	---	0	0	0	0	0	0	3.45	3.53	3.51	3.18	3.29	1	0	0	0	0	1	1	5.74	5.67	5.72	5.57	5.31	5.01	4.24
1394973_at	Pde1c	0	0	0	0	0	0	3.80	4.13	3.79	3.85	4.01	1	0	0	0	0	1	1	6.66	6.66	6.67	6.69	6.67	6.00	5.82
1394991_at	Irak1	0	0	0	0	0	0	6.71	6.71	6.66	6.46	6.57	1	0	0	0	0	1	1	8.34	8.23	8.39	8.39	8.30	7.58	6.63
1395032_at	---	0	0	0	0	0	0	5.57	5.74	5.87	5.73	5.78	1	0	0	0	0	0	1	4.46	4.37	4.44	4.35	4.40	4.75	5.13
1395036_at	Bpnt2	0	0	0	0	0	0	5.26	5.42	5.68	5.18	5.41	1	0	0	0	0	0	1	7.95	7.83	7.99	8.08	8.00	7.55	6.99
1395065_at	Wdr41	0	0	0	0	0	0	6.51	6.57	6.47	6.51	6.59	1	0	0	0	0	1	1	7.81	7.67	7.73	7.73	7.57	6.96	6.69
1395092_at	Rbm39	0	0	0	0	0	0	8.21	8.31	8.36	8.06	8.24	1	0	0	0	0	1	1	10.07	10.05	10.11	10.10	10.07	9.39	9.10
1395102_at	Kcnt1	0	0	0	0	0	0	3.36	3.34	3.31	3.32	3.20	1	0	0	0	0	1	1	5.17	5.26	5.42	5.64	5.52	5.98	5.76
1395120_at	---	0	0	0	0	0	0	3.70	3.48	3.46	3.59	3.63	1	0	0	0	0	0	1	4.23	4.26	4.38	4.34	4.39	4.58	5.87
1395142_at	Nexmif	0	0	0	0	0	0	5.60	5.46	5.48	5.82	5.29	1	0	0	0	0	1	1	7.28	7.14	7.34	7.41	7.10	6.10	5.27
1395152_at	Mak16	0	0	0	0	0	0	5.03	5.15	5.34	4.91	5.19	1	0	0	0	0	0	1	8.37	8.33	8.46	8.54	8.45	8.16	7.12
1395183_at	---	0	0	0	0	0	0	3.15	3.11	2.96	3.17	3.01	1	0	0	0	0	1	1	8.75	8.76	8.59	8.41	8.45	7.93	7.51
1395241_at	---	0	0	0	0	0	0	5.77	5.78	5.77	6.13	5.86	1	0	0	0	0	0	1	6.36	6.37	6.44	6.29	6.37	6.03	5.80
1395269_s_at	Gabrd	0	0	0	0	0	0	8.43	8.30	8.15	8.12	8.27	1	0	0	0	0	1	0	8.82	8.88	8.93	8.94	9.13	9.76	9.01
1395287_at	---	0	0	0	0	0	0	3.86	3.94	4.01	3.73	3.99	1	0	0	0	0	0	1	6.61	6.58	6.73	6.57	6.50	7.03	6.94
1395329_at	Gmppb	0	0	0	0	0	0	5.79	5.92	5.89	5.63	6.16	1	0	0	0	0	1	1	7.56	7.65	7.65	7.57	7.43	6.97	6.12
1395342_at	Ckap4	0	0	0	0	0	0	5.36	5.40	5.74	5.25	5.72	1	0	0	0	0	0	1	9.59	9.46	9.58	9.47	9.34	9.04	8.03
1395376_at	Ddx11	0	0	0	0	0	0	5.38	5.55	5.39	5.63	5.48	1	0	0	0	0	1	1	6.38	6.37	6.44	6.37	6.38	5.43	5.87
1395382_at	Unkl	0	0	0	0	0	0	5.02	5.36	5.42	5.14	5.31	1	0	0	1	1	1	0	6.16	6.37	6.58	6.82	6.83	6.79	6.37
1395393_at	C12orf65	0	0	0	0	0	0	4.82	4.67	4.69	4.80	4.63	1	0	0	0	0	0	1	6.34	6.29	6.31	6.47	6.32	6.65	6.87
1395420_at	C2orf68	0	0	0	0	0	0	5.25	5.08	5.12	5.44	5.08	1	0	0	0	0	0	1	6.64	6.58	6.67	6.69	6.57	6.28	6.11
1395433_at	---	0	0	0	0	0	0	4.52	4.54	4.71	4.54	4.51	1	0	0	0	0	1	1	6.92	6.87	6.89	6.88	6.63	5.92	5.38
1395440_at	Idnk	0	0	0	0	0	0	4.70	4.58	4.96	4.62	4.44	1	0	0	0	0	1	1	5.90	5.85	5.91	5.84	5.86	6.53	6.26
1395474_at	---	0	0	0	0	0	0	4.26	4.11	4.05	4.47	4.19	1	0	0	0	0	1	1	5.44	5.65	5.58	5.46	5.61	6.17	6.63
1395487_at	---	0	0	0	0	0	0	2.80	2.94	2.86	2.92	2.84	1	0	0	0	0	0	1	3.64	3.64	3.87	3.68	3.72	4.14	4.06
1395497_at	Cdca5	0	0	0	0	0	0	4.31	4.20	4.09	4.24	4.24	1	0	0	0	0	0	1	4.71	4.71	4.75	4.71	4.66	4.50	4.44
1395512_at	Crlf1	0	0	0	0	0	0	4.28	4.50	4.11	4.26	4.54	1	0	0	0	0	1	0	6.58	6.70	6.82	6.92	7.08	7.30	6.61
1395522_at	LOC100911357	0	0	0	0	0	0	4.47	4.47	4.45	4.43	4.38	1	0	0	0	0	0	1	5.02	4.92	4.90	4.66	4.74	4.71	4.55
1395544_at	Tmem214	0	0	0	0	0	0	6.26	6.24	6.02	6.35	6.35	1	0	0	0	0	0	1	6.57	6.64	6.74	6.72	6.52	6.15	5.52
1395555_at	Gosr1	0	0	0	0	0	0	6.03	5.78	6.30	5.66	5.86	1	0	0	0	0	0	1	8.28	8.26	8.27	8.27	8.18	7.78	7.29
1395594_at	Wnt11	0	0	0	0	0	0	4.45	4.36	4.09	4.41	4.28	1	0	0	0	0	1	1	4.00	3.99	4.12	4.07	4.04	4.63	4.49
1395601_at	Nudt12	0	0	0	0	0	0	4.10	4.05	4.44	4.53	4.38	1	0	0	0	0	1	0	4.75	4.70	5.13	5.09	5.11	5.47	5.55
1395604_at	---	0	0	0	0	0	0	4.97	4.83	4.77	5.20	4.96	1	0	0	0	0	0	1	5.74	5.77	5.82	5.66	5.74	6.22	6.40
1395610_at	Tspan33	0	0	0	0	0	0	6.04	5.89	6.35	5.77	6.00	1	0	0	0	0	1	0	5.88	5.98	6.19	6.08	6.08	6.55	5.98
1395625_at	LOC102554630	0	0	0	0	0	0	7.45	7.67	7.87	7.85	7.82	1	0	0	0	0	1	1	9.42	9.38	9.34	9.28	9.22	8.79	8.74
1395632_at	Mthfsd	0	0	0	0	0	0	5.49	5.40	5.25	5.65	5.50	1	0	0	0	0	0	1	6.79	6.83	6.86	6.87	6.75	6.49	5.81
1395634_at	Sptbn2	0	0	0	0	0	0	3.39	3.51	3.42	3.41	3.28	1	0	0	0	1	1	1	6.03	6.19	6.33	6.37	6.67	6.86	7.08
1395636_at	LOC100912622	0	0	0	0	0	0	6.06	6.38	6.03	6.48	5.80	1	0	0	0	0	1	0	8.30	8.25	8.28	8.24	8.17	7.71	7.76
1395644_at	Abca8	0	0	0	0	0	0	3.16	3.12	3.10	3.20	3.10	1	0	0	0	1	1	1	4.52	4.57	4.73	4.92	5.15	5.93	5.71
1395651_at	---	0	0	0	0	0	0	4.66	4.74	4.76	4.86	4.44	1	0	0	0	0	1	0	6.38	6.49	6.73	6.81	6.79	6.99	7.35
1395660_at	---	0	0	0	0	0	0	3.87	4.11	4.21	3.79	4.06	1	0	0	1	1	1	0	7.78	7.85	8.19	8.49	8.51	8.62	7.89
1395662_at	---	0	0	0	0	0	0	5.33	5.35	5.66	5.51	5.34	1	0	0	0	0	1	0	7.77	7.76	7.72	7.54	7.51	6.94	6.99
1395689_at	Fam169a	0	0	0	0	0	0	2.96	3.06	2.99	3.01	3.00	1	0	0	0	0	1	0	5.72	5.66	5.76	6.00	5.76	5.06	4.30
1395704_at	---	0	0	0	0	0	0	5.32	5.30	5.88	5.68	5.70	1	0	0	0	0	1	1	6.90	6.93	7.09	7.21	7.22	7.72	7.55
1395728_at	---	0	0	0	0	0	0	6.74	6.75	6.95	6.39	6.63	1	0	0	0	0	1	1	9.32	9.32	9.36	9.37	9.28	8.34	7.26
1395733_at	---	0	0	0	0	0	0	4.47	4.57	4.80	4.87	4.76	1	0	0	0	0	0	1	7.50	7.39	7.50	7.33	7.34	6.96	6.62
1395770_at	---	0	0	0	0	0	0	3.31	3.24	2.80	3.33	3.07	1	0	0	0	0	1	1	4.14	4.36	4.44	4.58	4.51	4.79	5.72
1395775_at	Hs3st1	0	0	0	0	0	0	4.09	4.14	4.25	4.28	4.18	1	0	0	0	0	1	1	6.35	6.29	6.25	6.05	6.17	4.81	4.53
1395791_at	Chpf2	0	0	0	0	0	0	5.26	5.36	5.12	5.11	5.22	1	0	0	0	0	0	1	7.62	7.70	7.73	7.72	7.62	7.26	6.25
1395816_at	Fam13c	0	0	0	0	0	0	3.15	3.29	3.43	3.27	3.31	1	0	0	1	1	1	0	5.16	5.52	5.64	5.75	5.76	5.85	5.80
1395828_at	Kif23	0	0	0	0	0	0	5.16	5.19	5.04	5.08	5.15	1	0	0	0	0	1	1	7.37	7.25	7.44	7.34	7.07	6.32	5.80
1395856_at	---	0	0	0	0	0	0	5.85	5.70	6.15	6.23	6.11	1	0	0	0	0	1	1	7.97	7.99	8.01	8.02	7.95	7.25	6.14
1395870_at	Trmt2a	0	0	0	0	0	0	3.81	3.58	3.64	3.55	3.69	1	0	0	0	0	0	1	6.37	6.52	6.63	6.55	6.55	6.26	5.58
1395927_at	---	0	0	0	0	0	0	4.74	4.97	4.59	4.66	4.47	1	0	0	1	0	1	1	6.06	5.56	6.18	5.46	5.67	5.26	6.13
1395947_at	---	0	0	0	0	0	0	4.25	4.57	4.22	4.36	4.55	1	0	0	0	0	1	1	5.26	5.38	5.34	5.47	5.60	6.04	5.76
1395968_at	Myo15b	0	0	0	0	0	0	6.43	6.57	6.60	6.54	6.75	1	0	0	0	0	1	1	7.80	7.61	7.65	7.65	7.43	6.72	6.81
1395986_at	Slit2	0	0	0	0	0	0	3.20	3.14	3.12	3.14	3.09	1	0	0	0	0	1	1	4.64	4.74	4.94	4.80	4.74	5.99	7.54
1396016_at	---	0	0	0	0	0	0	3.42	3.23	3.25	3.38	3.31	1	0	0	0	0	0	1	4.52	4.38	4.44	4.48	4.55	4.72	4.90
1396025_at	Gpr61	0	0	0	0	0	0	3.07	3.16	3.09	3.09	3.06	1	0	0	0	0	0	1	3.70	3.71	3.74	3.70	3.59	3.84	3.81
1396036_at	Ralgps2	0	0	0	0	0	0	4.51	4.65	4.54	4.55	4.61	1	0	0	0	0	1	1	5.72	5.75	5.93	5.99	5.98	6.34	6.40
1396038_at	Rtbdn	0	0	0	0	0	0	3.42	3.37	3.08	3.10	3.25	1	0	0	0	1	1	1	6.38	6.65	6.79	6.85	7.25	9.00	8.83
1396059_at	Ubxn4	0	0	0	0	0	0	8.22	8.12	8.24	7.78	8.16	1	0	0	0	0	0	1	9.66	9.65	9.69	9.71	9.64	9.28	8.80
1396092_at	Nkiras1	0	0	0	0	0	0	3.91	3.97	4.29	3.77	4.06	1	0	0	0	0	1	1	5.56	5.71	5.77	5.83	5.78	6.48	6.37
1396095_at	LOC102550950	0	0	0	0	0	0	3.06	3.07	3.01	3.11	3.05	1	0	0	0	0	1	1	4.69	4.66	4.80	4.83	4.82	5.53	5.73
1396098_at	Acin1	0	0	0	0	0	0	5.48	5.47	5.50	5.40	5.55	1	0	0	0	0	0	1	6.70	6.66	6.77	6.58	6.69	6.51	5.58
1396099_at	Tspan33	0	0	0	0	0	0	6.52	6.32	6.78	6.37	6.50	1	0	0	0	0	0	1	5.72	5.65	5.95	5.73	5.89	6.28	6.57
1396112_at	Mtmr10	0	0	0	0	0	0	5.62	5.67	5.95	5.46	5.77	1	0	0	0	0	0	1	6.52	6.52	6.70	6.52	6.62	6.97	7.44
1396135_at	---	0	0	0	0	0	0	5.10	4.92	4.76	4.89	4.81	1	0	0	0	0	1	1	5.73	5.88	6.17	6.00	6.17	6.53	6.09
1396139_at	---	0	0	0	0	0	0	4.64	4.65	4.41	4.58	4.65	1	0	0	0	0	0	1	5.58	5.67	5.64	5.64	5.71	6.15	5.83
1396148_at	---	0	0	0	0	0	0	6.62	6.65	6.76	6.37	6.68	1	0	0	0	1	0	0	7.56	7.76	7.93	8.05	8.18	7.73	7.01
1396164_at	Znf668	0	0	0	0	0	0	3.50	3.42	3.48	3.47	3.43	1	0	0	0	0	0	1	5.76	5.81	5.84	6.12	5.76	5.43	4.60
1396167_at	Acp6	0	0	0	0	0	0	6.05	5.71	5.72	5.70	5.72	1	0	0	0	0	1	1	6.65	6.75	6.88	7.10	7.19	7.79	7.09
1396208_at	Ggt5	0	0	0	0	0	0	5.44	5.62	5.82	5.50	5.52	1	0	0	0	0	0	1	4.91	4.90	4.76	4.74	4.82	5.18	5.30
1396211_at	---	0	0	0	0	0	0	3.73	3.72	3.82	3.81	3.76	1	0	0	0	0	0	1	4.20	4.20	4.29	4.25	4.40	4.51	4.79
1396233_at	Arsk	0	0	0	0	0	0	2.90	2.78	3.02	2.88	3.15	1	0	0	0	1	1	1	5.25	5.38	5.46	5.70	5.92	6.38	5.00
1396236_at	---	0	0	0	0	0	0	3.33	3.28	3.36	3.17	3.23	1	0	0	0	0	1	0	4.36	4.45	4.59	4.58	4.65	4.97	5.13
1396278_at	Snx11	0	0	0	0	0	0	5.87	5.69	6.24	5.57	5.91	1	0	0	0	0	1	0	7.44	7.52	7.66	7.72	7.74	8.04	7.34
1396281_at	LOC103690403	0	0	0	0	0	0	3.52	3.47	3.52	3.49	3.44	1	0	0	0	0	1	0	6.33	6.49	6.55	6.44	6.70	6.95	7.22
1396295_at	Gpr173	0	0	0	0	0	0	4.42	4.63	4.66	4.58	4.48	1	0	0	0	0	1	1	7.99	7.94	7.87	7.80	7.73	7.32	6.86
1396332_at	---	0	0	0	0	0	0	5.73	5.51	5.42	5.48	5.62	1	0	0	0	0	1	1	7.62	7.59	7.50	7.25	7.20	6.96	6.41
1396342_at	Pou3f2	0	0	0	0	0	0	4.93	4.90	4.78	5.12	4.91	1	0	0	0	0	1	0	8.29	8.44	8.54	8.64	8.71	9.03	7.85
1396347_at	LOC100911864	0	0	0	0	0	0	3.09	3.12	3.05	3.18	3.14	1	0	0	0	0	1	1	6.08	6.04	6.18	5.83	5.69	4.87	3.94
1396352_at	Myrfl	0	0	0	0	0	0	4.68	4.64	4.40	4.53	4.65	1	0	0	0	0	1	1	6.93	7.06	6.94	6.87	6.91	6.28	5.27
1396353_at	Ppp2r3d	0	0	0	0	0	0	5.18	5.41	5.19	5.44	5.14	1	0	0	0	0	1	0	6.86	6.81	6.83	6.56	6.61	6.13	5.88
1396372_at	---	0	0	0	0	0	0	3.99	4.08	3.82	3.99	3.91	1	0	0	0	0	1	0	5.67	5.50	5.54	5.35	5.31	4.81	4.89
1396385_at	---	0	0	0	0	0	0	3.34	3.56	3.73	3.44	3.49	1	0	0	0	0	1	0	4.25	4.26	4.41	4.55	4.64	4.87	4.88
1396389_at	Pip5kl1	0	0	0	0	0	0	4.43	4.40	4.28	4.42	4.42	1	0	0	0	1	1	1	6.34	6.51	6.77	6.82	6.93	8.39	8.67
1396390_at	Riiad1	0	0	0	0	0	0	3.39	3.36	3.37	3.11	3.40	1	0	0	0	0	1	1	5.63	5.76	5.97	5.97	6.10	7.04	7.11
1396392_at	Dctn6	0	0	0	0	0	0	3.29	3.45	3.54	3.20	3.34	1	0	0	0	0	1	1	6.35	6.28	6.36	6.36	6.38	5.66	5.18
1396401_at	Rbm17	0	0	0	0	0	0	3.21	3.44	3.34	3.29	3.33	1	0	0	0	0	1	0	5.89	6.03	6.26	6.39	6.47	6.62	6.47
1396404_at	FUNDC2	0	0	0	0	0	0	4.99	4.82	5.36	4.79	4.72	1	0	0	0	0	1	1	8.39	8.43	8.40	8.50	8.36	7.73	6.76
1396417_at	---	0	0	0	0	0	0	5.14	5.04	5.05	5.00	4.90	1	0	0	1	1	1	0	4.83	4.90	5.29	5.45	5.49	5.62	5.62
1396487_at	---	0	0	0	0	0	0	5.69	5.51	5.35	5.65	5.44	1	0	0	0	0	0	1	6.58	6.53	6.68	6.47	6.33	6.23	5.44
1396492_at	Nfam1	0	0	0	0	0	0	6.43	6.49	6.36	6.35	6.40	1	0	0	0	0	0	1	7.07	7.01	7.07	6.85	6.93	6.71	6.22
1396525_at	---	0	0	0	0	0	0	4.87	4.73	4.77	4.81	4.71	1	0	0	0	0	0	1	5.82	5.76	5.91	5.81	5.89	5.39	4.65
1396542_at	---	0	0	0	0	0	0	3.19	3.50	3.38	3.35	3.26	1	0	0	1	1	0	0	4.35	4.49	4.79	4.97	4.95	4.68	4.39
1396545_at	Crem	0	0	0	0	0	0	5.34	5.36	5.15	5.05	4.88	1	0	0	0	0	0	1	6.19	6.09	6.38	6.36	6.34	5.81	5.27
1396620_at	Gpc3	0	0	0	0	0	0	3.05	3.02	2.96	3.10	2.98	1	0	0	0	0	0	1	4.09	4.00	3.82	4.05	4.00	4.37	4.35
1396621_at	---	0	0	0	0	0	0	6.21	6.26	6.55	6.17	6.37	1	0	0	0	0	1	1	7.16	7.22	7.18	7.22	7.09	6.47	6.08
1396810_at	---	0	0	0	0	0	0	2.99	2.91	3.11	2.98	3.10	1	0	0	0	0	0	1	3.26	3.40	3.46	3.52	3.39	3.81	3.80
1396847_at	LOC102553109	0	0	0	0	0	0	5.67	5.53	5.60	5.44	5.27	1	0	0	0	0	0	1	6.99	6.88	7.02	6.68	6.70	6.44	5.65
1396851_at	---	0	0	0	0	0	0	3.11	3.28	3.11	3.34	3.21	1	0	0	0	0	1	1	4.37	4.32	4.36	4.43	4.46	5.32	4.89
1396884_at	---	0	0	0	0	0	0	2.98	3.21	3.17	3.24	3.38	1	0	0	0	0	0	1	4.48	4.67	4.62	4.63	4.38	4.42	4.44
1396893_at	---	0	0	0	0	0	0	5.86	5.91	6.08	5.61	5.95	1	0	0	0	0	0	1	6.77	6.70	6.88	6.87	6.88	7.22	7.35
1396895_at	Isl1	0	0	0	0	0	0	3.84	3.88	3.87	3.72	3.99	1	0	0	0	0	1	0	6.17	6.06	6.34	6.54	6.68	6.87	5.53
1396936_at	---	0	0	0	0	0	0	3.99	3.95	3.89	4.03	3.90	1	0	0	0	0	1	1	6.31	6.34	6.30	6.29	6.28	5.47	5.38
1396965_at	---	0	0	0	0	0	0	8.17	8.19	8.05	8.25	8.23	1	0	0	0	0	1	1	7.62	7.65	7.72	7.67	7.74	8.22	8.17
1396997_at	---	0	0	0	0	0	0	5.29	5.41	5.15	5.13	5.23	1	0	0	0	0	1	0	6.44	6.51	6.66	6.39	6.59	7.05	6.14
1397004_at	---	0	0	0	0	0	0	6.94	6.99	7.12	6.79	6.90	1	0	0	0	0	1	1	8.21	8.48	8.61	8.49	8.66	9.24	8.61
1397025_at	---	0	0	0	0	0	0	6.19	6.20	5.87	6.23	6.12	1	0	0	0	0	1	0	7.28	7.45	7.60	7.76	7.83	7.88	7.79

Probeset ID	Gene Symbol	Islet Data Set (log2 ratio ≥0.263; FDR <20%) union n=5325 1=YES 0=NO	Islet DRlyp/lyp ND vs ND+Lp299v (log2 ratio ≥0.263; FDR<20%) n=429 1=YES 0=NO	Islet DRlyp/lyp ND vs HCD (log2 ratio ≥0.263; FDR<20%) n=2247 1=YES 0=NO	Islet DRlyp/lyp ND vs HCD+Lp299v (log2 ratio ≥0.263; FDR<20%) n=1595 1=YES 0=NO	Islet DRlyp/lyp HCD vs HCD+Lp299v (log2 ratio ≥0.263; FDR<20%) n=3026 1=YES 0=NO	Islet DRlyp/lyp ND vs Flyp/lyp ND (log2 ratio ≥ 0.263; FDR<20%) n=1950 1=YES 0=NO	Islet Mean Log2 Intensity DRlyp/lyp ND	Islet Mean Log2 Intensity DRlyp/lyp ND+Lp299v	Islet Mean Log2 Intensity DRlyp/lyp HCD	Islet Mean Log2 Intensity DRlyp/lyp HCD+Lp299v	Islet Mean Log2 Intensity Flyp40 ND	Rinm5f data union of all conditions n=3705 1=YES 0=NO	Rinm5f 30 µM propionate/ 6 µM butyrate vs Media (FDR<20%; 1.5 fold) n=3 1=YES 0=NO	Rinm5f 60 µM propionate/ 12 µM butyrate vs Media (FDR<20%; 1.5 fold) n=18 1=YES 0=NO	Rinm5f 120 µM propionate/ 24 µM butyrate vs Media (FDR<20%; 1.5 fold) n=164 1=YES 0=NO	Rinm5f 240 µM propionate /48 µM butyrate vs Media (FDR<20%; 1.5 fold) n=268 1=YES 0=NO	Rinm5f 1500 µM propionate/ 300 µM butyrate vs Media (FDR<20%; 1.5 fold) n=2491 1=YES 0=NO	Rinm5f 500 µM propionate/ 500 µM butyrate vs Media (FDR<20%; 1.5 fold) n=3001 1=YES 0=NO	Rinm5f Mean Log2 Intensity Media only	Rinm5f Mean Log2 Intensity 30 µM propionate/ 6 µM butyrate	Rinm5f Mean Log2 Intensity 60 µM propionate/ 12 µM butyrate	Rinm5f Mean Log2 Intensity 120 µM propionate/ 24 µM butyrate	Rinm5f Mean Log2 Intensity 240 µM propionate/ 48 µM butyrate	Rinm5f Mean Log2 Intensity 1500 µM propionate /300 µM butyrate	Rinm5f Mean Log2 Intensity 500 µM propionate/ 500 µM butyrate
1397045_at	---	0	0	0	0	0	0	4.80	4.59	4.64	4.54	4.52	1	0	0	0	0	0	1	5.15	5.27	5.36	5.22	5.32	5.43	5.41
1397107_at	---	0	0	0	0	0	0	3.95	4.04	3.85	3.99	3.77	1	0	0	0	0	0	1	4.53	4.65	4.72	4.65	4.65	4.94	5.17
1397115_at	---	0	0	0	0	0	0	3.83	3.74	3.59	3.70	3.79	1	0	0	0	0	1	1	5.49	5.62	5.74	5.65	5.60	6.19	6.26
1397120_at	---	0	0	0	0	0	0	6.58	6.53	6.59	6.30	6.66	1	0	0	0	0	1	1	6.41	6.63	6.74	6.60	6.75	7.35	7.54
1397130_at	---	0	0	0	0	0	0	4.24	4.17	4.00	4.29	3.85	1	0	0	0	0	0	1	5.24	5.15	5.21	5.21	5.09	4.90	3.96
1397173_at	Wnk3	0	0	0	0	0	0	6.42	6.47	6.59	6.08	6.88	1	0	0	1	1	1	1	6.09	5.66	6.48	6.87	6.79	7.55	7.47
1397180_at	Styxl1	0	0	0	0	0	0	5.85	5.70	5.37	5.78	5.45	1	0	0	0	0	0	1	5.93	5.86	6.06	5.84	5.86	6.18	6.38
1397201_at	Camsap2	0	0	0	0	0	0	4.34	4.52	4.36	4.44	4.42	1	0	0	0	0	1	0	6.36	6.16	6.20	6.17	6.03	5.74	5.77
1397214_at	Fyb1	0	0	0	0	0	0	3.49	3.57	3.37	3.57	3.72	1	0	0	0	0	1	1	7.72	7.67	7.72	7.69	7.62	6.88	6.00
1397215_at	---	0	0	0	0	0	0	4.55	4.64	4.74	4.54	4.61	1	0	0	0	0	1	0	6.35	6.26	6.43	6.37	6.33	5.68	5.49
1397238_at	---	0	0	0	0	0	0	3.08	3.06	3.56	3.15	3.52	1	0	0	0	1	1	0	4.16	4.36	4.52	4.56	4.81	4.99	4.28
1397243_at	Ing2	0	0	0	0	0	0	4.00	4.07	3.72	3.91	3.89	1	0	0	0	0	1	1	5.85	5.89	5.96	5.70	5.76	5.24	5.15
1397246_at	Ntrk2	0	0	0	0	0	0	7.72	7.90	8.07	7.86	7.79	1	0	0	0	0	1	1	8.88	8.83	8.83	8.57	8.69	8.09	7.70
1397266_at	Klf11	0	0	0	0	0	0	4.01	4.38	3.91	4.00	3.80	1	0	0	0	0	0	1	7.70	7.77	7.87	7.89	7.75	7.71	6.42
1397267_at	Rimbp2	0	0	0	0	0	0	6.71	6.98	7.09	6.76	7.05	1	0	0	0	0	1	1	8.26	8.22	8.35	8.30	8.20	7.57	6.99
1397277_at	---	0	0	0	0	0	0	3.65	3.68	3.77	3.69	3.83	1	0	0	1	1	1	0	5.53	5.88	5.87	6.21	6.23	6.26	5.70
1397280_at	Pou2f2	0	0	0	0	0	0	4.03	4.11	3.91	3.95	4.08	1	0	0	0	0	0	1	5.39	5.37	5.47	5.65	5.56	5.81	5.92
1397317_at	Itgb3	0	0	0	0	0	0	3.60	3.66	3.46	3.62	3.62	1	0	0	0	0	1	1	4.63	4.71	4.73	4.65	4.85	5.29	6.23
1397341_at	Pbk	0	0	0	0	0	0	5.56	5.52	5.53	5.56	5.72	1	0	0	0	0	1	1	8.77	8.76	8.72	8.73	8.60	7.97	7.74
1397346_at	Casz1	0	0	0	0	0	0	4.47	4.73	4.42	4.60	4.40	1	0	0	0	0	0	1	6.08	6.01	6.18	6.21	6.04	6.33	6.95
1397349_at	Ubxn8	0	0	0	0	0	0	3.65	3.53	3.94	3.94	4.02	1	0	0	0	0	1	1	6.86	6.85	6.91	7.02	6.87	5.54	4.79
1397367_at	Adam23	0	0	0	0	0	0	3.69	3.60	3.36	3.62	3.62	1	0	0	0	0	1	0	6.36	6.30	6.44	6.75	6.63	7.05	6.44
1397372_at	Mob1a	0	0	0	0	0	0	4.71	4.89	4.81	4.41	4.64	1	0	0	0	0	0	1	7.06	6.88	7.00	7.05	6.86	6.82	5.56
1397375_at	Acsl5	0	0	0	0	0	0	7.60	7.71	7.28	7.63	7.56	1	0	0	0	0	1	1	9.31	9.18	9.24	9.14	8.94	7.64	6.90
1397400_at	Draxin	0	0	0	0	0	0	4.33	4.35	4.12	4.36	4.31	1	0	0	0	0	1	1	5.50	5.47	5.66	5.61	5.75	7.10	7.20
1397458_at	Pmm2	0	0	0	0	0	0	4.41	4.38	4.55	4.02	4.53	1	0	0	0	0	0	1	7.79	7.74	7.79	7.75	7.67	7.27	5.85
1397476_at	Ttk	0	0	0	0	0	0	3.77	3.57	3.54	3.68	3.61	1	0	0	0	0	1	1	5.96	5.95	5.98	5.86	5.65	4.84	4.79
1397478_at	Phb2	0	0	0	0	0	0	5.38	5.50	5.47	5.66	5.00	1	0	0	0	0	0	1	6.18	6.13	6.50	6.43	6.24	5.91	5.00
1397495_at	---	0	0	0	0	0	0	4.75	4.99	4.94	4.75	5.17	1	0	0	0	0	1	1	7.85	7.88	7.89	7.99	8.10	8.51	8.68
1397535_at	Mpp5	0	0	0	0	0	0	7.09	7.09	7.18	7.26	7.16	1	0	0	0	0	1	1	6.63	6.68	6.92	7.10	7.11	7.94	8.59
1397539_at	Incenp	0	0	0	0	0	0	3.70	3.57	3.51	3.56	3.48	1	0	0	0	0	1	1	7.83	7.54	7.71	7.51	7.37	6.72	6.88
1397561_at	Kiaa0319	0	0	0	0	0	0	3.53	3.55	3.50	3.39	3.40	1	0	0	0	0	0	1	4.70	4.54	4.65	4.89	4.69	4.92	4.74
1397566_at	LOC103692946 /	0	0	0	0	0	0	5.12	4.94	4.84	5.01	5.08	1	0	0	0	0	1	1	6.90	6.99	6.96	6.88	6.78	6.27	5.66
1397567_at	Kif14	0	0	0	0	0	0	3.45	3.57	3.43	3.64	3.65	1	0	0	0	0	1	1	6.78	6.77	6.78	6.65	6.46	5.89	5.99
1397570_at	---	0	0	0	0	0	0	4.40	4.34	4.51	4.18	4.12	1	0	0	0	0	0	1	4.07	4.22	4.30	4.38	4.24	4.40	4.31
1397588_at	C16orf90	0	0	0	0	0	0	5.53	5.63	5.50	5.57	5.45	1	0	0	0	0	1	1	8.29	8.19	8.23	8.12	7.96	7.52	7.12
1397593_at	---	0	0	0	0	0	0	4.43	4.43	4.30	4.67	4.20	1	0	0	0	0	1	1	4.58	4.41	4.68	4.54	4.59	5.28	5.67
1397596_at	Trim2	0	0	0	0	0	0	3.59	3.55	3.63	3.26	3.48	1	0	0	0	0	1	1	6.08	6.03	6.36	6.48	6.60	6.73	6.48
1397599_at	---	0	0	0	0	0	0	3.32	3.30	3.12	3.25	3.25	1	0	0	0	0	1	1	3.85	3.83	4.07	3.90	4.02	4.51	4.25
1397604_at	Vbp1	0	0	0	0	0	0	4.14	3.94	4.18	4.41	3.84	1	0	0	0	0	1	1	5.04	5.03	5.12	5.10	4.64	4.02	3.60
1397609_at	Knstrn	0	0	0	0	0	0	3.77	3.71	3.93	3.66	3.84	1	0	0	0	0	1	1	8.18	8.15	8.12	8.11	7.79	6.42	5.99
1397614_at	Gabra2	0	0	0	0	0	0	2.97	3.03	2.93	3.07	2.99	1	0	0	0	0	1	0	5.30	5.16	5.16	5.13	4.96	4.58	4.14
1397653_at	Ccnb1	0	0	0	0	0	0	6.79	6.97	6.58	6.86	6.78	1	0	0	0	0	1	0	7.10	7.04	6.91	6.94	6.91	6.51	6.43
1397654_at	---	0	0	0	0	0	0	3.69	3.93	3.75	3.54	3.76	1	0	0	0	0	1	0	6.19	6.24	6.31	6.63	6.72	6.86	6.53
1397704_at	Esco2	0	0	0	0	0	0	4.31	4.41	4.42	4.56	4.47	1	0	0	0	0	0	1	7.70	7.69	7.80	7.89	7.79	7.12	6.93
1397746_at	Klhdc8a	0	0	0	0	0	0	4.72	4.82	4.45	4.56	4.36	1	0	0	0	0	1	1	6.32	6.36	6.41	6.21	6.16	5.52	5.07
1397765_at	---	0	0	0	0	0	0	6.35	6.20	6.20	6.52	6.49	1	0	0	0	0	1	0	7.20	7.38	7.49	7.62	7.68	7.87	7.32
1397798_at	---	0	0	0	0	0	0	3.75	3.75	3.69	3.57	3.72	1	0	0	0	0	1	1	5.10	5.21	5.43	5.30	5.50	5.69	5.92
1397810_at	---	0	0	0	0	0	0	3.88	3.91	3.99	3.72	3.86	1	0	0	0	0	1	0	5.30	5.26	5.14	5.39	5.59	6.26	5.57
1397838_at	---	0	0	0	0	0	0	5.54	5.44	5.88	5.76	5.66	1	0	0	0	0	1	1	8.85	8.82	8.78	8.64	8.64	7.92	7.45
1397854_at	Dpf3	0	0	0	0	0	0	3.18	3.13	3.66	3.17	3.44	1	0	0	0	0	1	1	3.76	3.91	3.88	3.94	4.01	4.62	4.66
1397874_at	Thg1l	0	0	0	0	0	0	6.56	6.59	6.72	6.62	6.73	1	0	0	0	0	1	0	7.54	7.46	7.47	7.20	7.25	6.92	6.60
1397881_at	Ccdc88a	0	0	0	0	0	0	3.82	3.90	3.73	3.71	3.83	1	0	0	0	0	1	0	7.36	7.45	7.76	7.93	7.93	7.98	7.75
1397882_at	Zbtb8a	0	0	0	0	0	0	5.03	5.12	5.07	5.14	4.84	1	0	0	0	0	1	1	6.59	6.71	6.85	6.63	6.70	5.89	5.25
1397890_at	C8orf34	0	0	0	0	0	0	2.79	2.89	2.84	2.87	2.68	1	0	0	0	0	0	1	4.64	4.67	4.55	4.57	4.38	4.19	3.44
1397904_at	LOC100909928	0	0	0	0	0	0	5.59	5.64	5.66	5.50	5.56	1	0	0	0	0	0	1	5.90	5.83	5.81	5.85	6.00	5.78	4.84
1397924_at	LOC100912473	0	0	0	0	0	0	8.16	8.08	8.64	8.39	8.29	1	0	0	0	0	1	0	7.76	7.78	7.80	7.66	7.67	7.14	6.97
1397951_at	---	0	0	0	0	0	0	3.93	4.11	3.90	3.90	3.93	1	0	0	0	0	1	0	6.46	6.50	6.66	6.74	6.97	7.05	7.06
1398015_at	---	0	0	0	0	0	0	3.94	3.84	3.61	3.93	3.91	1	0	0	0	0	0	1	4.18	4.27	4.25	4.09	4.20	4.41	4.89
1398022_at	---	0	0	0	0	0	0	4.94	5.12	5.31	4.73	5.17	1	0	0	0	0	0	1	4.08	4.00	4.16	4.22	4.15	4.52	4.69
1398061_at	---	0	0	0	0	0	0	3.34	3.40	3.39	3.31	3.32	1	0	0	0	0	0	1	5.44	5.28	5.48	5.33	5.45	5.07	4.16
1398115_at	---	0	0	0	0	0	0	5.74	5.86	5.81	5.77	5.64	1	0	0	0	0	0	1	6.40	6.46	6.59	6.40	6.63	6.86	6.66
1398124_at	Jazf1	0	0	0	0	0	0	3.78	3.93	3.70	3.53	3.89	1	0	0	0	0	1	1	5.61	5.66	5.77	5.55	5.54	4.77	4.59
1398131_at	---	0	0	0	0	0	0	5.80	6.02	5.83	5.73	5.96	1	0	0	0	0	1	0	5.52	5.54	5.76	5.78	5.85	6.18	6.05
1398133_at	---	0	0	0	0	0	0	3.45	3.44	3.16	3.36	3.39	1	0	0	0	0	1	1	4.57	4.55	4.79	4.64	4.63	5.17	6.15
1398221_at	---	0	0	0	0	0	0	3.51	3.47	3.31	3.44	3.43	1	0	0	0	0	1	1	6.04	6.14	6.16	5.71	5.72	5.34	5.16
1398229_at	---	0	0	0	0	0	0	2.74	2.88	2.83	2.92	2.77	1	0	0	0	0	1	1	3.92	4.06	4.15	3.95	3.98	4.52	4.11
1398246_s_at	Fcgr2a	0	0	0	0	0	0	4.17	4.19	4.14	4.24	4.03	1	0	0	0	0	1	1	5.01	5.09	5.18	5.32	5.54	5.88	5.98
1398255_at	Slc15a2	0	0	0	0	0	0	3.86	3.69	3.79	3.75	3.82	1	0	0	0	0	1	1	7.24	7.14	7.19	7.13	7.01	5.83	5.29
1398268_at	Nfyc	0	0	0	0	0	0	8.13	7.99	8.05	8.38	7.96	1	0	0	0	0	1	0	8.94	8.85	8.93	8.92	8.73	8.34	8.30
1398270_at	Bmp2	0	0	0	0	0	0	4.29	4.33	4.38	4.35	4.47	1	0	0	0	0	0	1	7.17	7.03	6.95	6.82	6.69	7.21	8.22
1398273_at	Efna1	0	0	0	0	0	0	4.91	4.87	5.01	4.58	4.73	1	0	0	0	0	0	1	8.32	8.41	8.40	8.33	8.23	7.91	7.28
1398275_at	Mmp9	0	0	0	0	0	0	3.40	3.42	3.22	3.30	3.38	1	0	0	0	0	1	1	3.67	3.81	3.76	3.83	3.89	4.86	5.22
1398287_at	Plau	0	0	0	0	0	0	4.33	4.35	4.45	4.03	4.35	1	0	0	0	0	1	0	3.95	4.02	3.88	4.06	3.99	4.54	4.13
1398319_at	Bmp2k	0	0	0	0	0	0	4.70	4.72	5.06	4.89	4.88	1	0	0	0	0	1	1	8.00	8.00	8.07	8.16	8.15	8.66	9.18
1398365_at	Tppp3	0	0	0	0	0	0	6.70	6.60	6.26	6.63	6.45	1	0	0	0	0	1	0	6.35	6.48	6.49	6.60	6.72	7.09	6.97
1398375_at	Mta3	0	0	0	0	0	0	6.45	6.45	6.97	6.81	6.50	1	0	0	0	0	0	1	8.45	8.44	8.51	8.56	8.40	7.93	7.05
1398407_at	Tnfaip8	0	0	0	0	0	0	4.72	4.58	4.68	4.93	4.45	1	0	0	0	0	1	1	6.50	6.44	6.45	6.30	6.44	7.48	7.31
1398430_at	Myo6	0	0	0	0	0	0	7.41	7.54	7.59	7.20	7.57	1	0	0	0	0	0	1	6.70	6.69	6.76	6.69	6.73	7.22	7.49
1398435_at	Slc22a15	0	0	0	0	0	0	4.98	5.22	5.18	5.26	5.25	1	0	0	0	0	1	1	8.15	8.10	8.05	7.89	7.86	7.09	7.17
1398438_at	Gtpbp3	0	0	0	0	0	0	5.64	5.88	6.00	5.92	5.86	1	0	0	0	0	1	0	7.42	7.42	7.60	7.81	7.76	8.13	7.48
1398442_at	Tedc1	0	0	0	0	0	0	4.95	5.19	5.03	5.25	5.04	1	0	0	0	0	0	1	8.07	8.05	8.15	8.08	7.90	7.56	7.31
1398449_at	Ptpn7	0	0	0	0	0	0	5.45	5.58	5.09	5.33	5.52	1	0	0	0	0	1	1	5.61	5.47	5.64	5.69	5.77	6.74	6.99
1398453_at	Klhl36	0	0	0	0	0	0	5.59	5.65	5.21	5.63	5.45	1	0	0	0	0	0	1	5.75	5.68	5.77	5.67	5.72	6.10	6.31
1398460_at	Cep295	0	0	0	0	0	0	4.03	3.91	3.96	3.97	4.05	1	0	0	0	0	1	0	6.43	6.40	6.52	6.53	6.41	5.85	5.75
1398470_at	Foxm1	0	0	0	0	0	0	3.31	3.41	3.42	3.38	3.43	1	0	0	0	0	1	1	5.26	4.99	5.17	5.19	5.09	4.44	4.26
1398480_at	Lrrc24	0	0	0	0	0	0	3.52	3.51	3.51	3.52	3.52	1	0	0	0	1	1	1	7.29	7.52	7.60	7.85	8.10	8.49	7.99
1398482_at	Bcl3	0	0	0	0	0	0	3.33	3.45	3.27	3.50	3.42	1	0	0	0	0	1	1	7.95	8.02	8.01	7.82	7.89	7.23	5.83
1398495_at	---	0	0	0	0	0	0	3.41	3.51	3.67	3.44	3.58	1	0	0	1	1	1	0	5.98	6.12	6.39	6.59	6.61	6.68	6.51
1398553_at	Tmed5	0	0	0	0	0	0	6.21	6.42	6.12	6.61	6.06	1	0	0	0	0	1	0	5.43	5.10	5.18	5.19	5.18	4.76	4.18
1398578_at	Ttyh1	0	0	0	0	0	0	2.90	2.97	3.12	2.99	2.87	1	0	0	0	1	1	1	3.42	3.44	3.54	3.68	4.01	4.71	4.24
1398597_at	Rnf144a	0	0	0	0	0	0	4.76	4.69	4.81	4.47	4.76	1	0	0	0	0	0	1	6.91	6.91	7.01	7.08	7.01	7.24	7.37
1398608_at	Cenpl	0	0	0	0	0	0	6.40	6.19	6.74	6.50	6.45	1	0	0	0	0	1	1	8.39	8.34	8.35	8.39	8.33	7.77	7.60
1398623_at	---	0	0	0	0	0	0	3.12	2.95	2.86	3.03	2.94	1	0	0	0	0	1	1	5.45	5.58	5.81	5.76	5.82	6.33	6.43
1398629_at	Dgkk	0	0	0	0	0	0	3.33	3.37	3.14	3.29	3.33	1	0	0	0	0	0	1	4.24	4.15	4.19	4.12	4.19	4.59	4.51
1398662_at	Fam167a	0	0	0	0	0	0	4.70	4.61	4.36	4.42	5.15	1	0	0	0	0	1	0	6.63	6.61	6.72	6.74	6.94	7.30	7.52
1398690_at	H3-3a /// H3-3b	0	0	0	0	0	0	5.41	5.53	5.79	5.34	5.66	1	0	0	0	0	1	0	6.39	6.39	6.70	6.68	6.84	7.27	6.94
1398693_at	Man1c1	0	0	0	0	0	0	7.19	7.17	7.57	7.21	7.33	1	0	0	0	0	1	1	6.27	6.32	6.49	6.58	6.66	7.05	6.89
1398704_at	---	0	0	0	0	0	0	6.33	6.42	6.70	6.20	6.55	1	0	0	0	0	1	0	5.79	5.92	6.02	6.02	6.10	6.52	6.48
1398721_at	Fsd1	0	0	0	0	0	0	3.72	3.61	3.62	3.67	3.64	1	0	0	0	0	1	0	7.61	7.83	7.88	7.89	7.94	8.24	8.25
1398732_at	---	0	0	0	0	0	0	6.24	6.35	6.74	6.53	6.14	1	0	0	0	0	0	1	8.79	8.96	8.83	8.67	8.86	9.05	9.10
1398733_at	LOC103692984	0	0	0	0	0	0	2.97	2.99	2.91	2.87	2.96	1	0	0	0	1	1	1	6.26	6.42	6.63	6.71	7.07	7.85	7.65
1398739_at	---	0	0	0	0	0	0	4.22	4.39	4.48	3.99	4.12	1	0	1	1	1	1	1	4.58	4.79	5.17	5.43	5.40	5.87	5.12
1398809_at	Nde1	0	0	0	0	0	0	6.76	6.51	6.52	7.01	6.44	1	0	0	0	0	0	1	5.82	5.88	5.86	5.90	5.81	6.11	6.85
1398818_at	Gng5	0	0	0	0	0	0	4.20	4.08	4.37	4.24	4.19	1	0	0	0	0	1	0	5.71	5.74	5.85	6.18	6.11	6.47	5.97
1398840_at	Vamp5	0	0	0	0	0	0	6.98	7.05	6.82	6.65	6.89	1	0	0	0	0	1	1	5.99	6.02	6.13	6.14	6.29	7.10	7.42
1398857_at	Surf1	0	0	0	0	0	0	7.93	7.64	7.94	7.86	7.72	1	0	0	0	0	0	1	10.31	10.31	10.32	10.25	10.23	9.84	9.21
1398886_at	Selenoh	0	0	0	0	0	0	9.34	9.21	9.15	9.02	9.22	1	0	0	0	0	1	1	10.57	10.54	10.53	10.42	10.27	9.64	9.59
1398913_at	Numa1	0	0	0	0	0	0	7.32	7.28	7.41	7.53	7.41	1	0	0	0	0	0	1	8.92	8.91	8.95	8.89	8.81	8.34	8.24
1398915_at	Lamtor4	0	0	0	0	0	0	7.67	7.72	7.57	7.79	7.61	1	0	0	0	0	0	1	9.97	10.03	9.98	9.95	9.87	9.59	9.29
1398916_at	Aurkaip1 /// Ccr	0	0	0	0	0	0	7.76	7.61	7.55	7.68	7.64	1	0	0	0	0	0	1	8.62	8.67	8.65	8.64	8.57	8.22	7.92
1398943_at	Lage3	0	0	0	0	0	0	9.36	9.30	9.26	9.49	9.49	1	0	0	0	0	1	1	10.35	10.35	10.33	10.42	10.27	9.56	8.94
1398946_at	Mrps16	0	0	0	0	0	0	8.50	8.57	8.31	8.85	8.45	1	0	0	0	0	1	1	9.96	9.95	9.86	9.83	9.70	9.22	9.11
1398956_at	Nsd1	0	0	0	0	0	0	8.08	7.90	8.24	7.93	8.10	1	0	0	0	0	0	1	8.53	8.54	8.69	8.67	8.61	8.10	7.96
1399037_at	Ttc19	0	0	0	0	0	0	6.25	6.35	6.46	6.34	6.59	1	0	0	0	0	1	1	7.61	7.69	7.73	7.91	7.86	8.37	8.39
1399088_at	Tlk2	0	0	0	0	0	0	7.64	7.60	7.80	7.60	7.68	1	0	0	0	0	0	1	8.92	8.98	9.11	9.14	9.02	8.65	8.26
1399096_at	Add3	0	0	0	0	0	0	7.30	7.24	7.87	7.57	7.50	1	0	0	0	0	0	1	8.97	8.95	9.02	9.07	9.12	9.54	10.06
1399108_at	Dis3l	0	0	0	0	0	0	7.33	7.42	7.21	7.60	7.26	1	0	0	0	0	1	1	7.90	7.88	7.89	7.87	7.81	7.17	6.67
1399118_at	Msl1	0	0	0	0	0	0	6.25	5.95	6.32	6.21	6.08	1	0	0	0	0	0	1	8.15	8.07	8.31	8.19	8.19	7.73	7.39
1399161_a_at	Erap1	0	0	0	0	0	0	8.07	8.14	8.18	8.18	7.89	1	0	0	0	0	1	1	10.16	10.20	10.13	10.16	10.08	9.57	8.72
1399166_a_at	Bcl7b	0	0	0	0	0	0	6.97	7.07	7.27	6.93	7.23	1	0	0	0	0	1	1	8.50	8.55	8.56	8.51	8.40	7.83	7.96
